# Hypoxia-induced stabilization of HIF2A promotes cardiomyocyte proliferation by attenuating DNA damage

**DOI:** 10.20517/jca.2023.43

**Published:** 2024-01-29

**Authors:** Shah R. Ali, Ngoc Uyen Nhi Nguyen, Ivan Menendez-Montes, Ching-Cheng Hsu, Waleed Elhelaly, Nicholas T. Lam, Shujuan Li, Abdallah Elnwasany, Yuji Nakada, Suwannee Thet, Roger S. Y. Foo, Hesham A. Sadek

**Affiliations:** 1 Department of Medicine, Division of Cardiology, Columbia University Irving Medical Center, New York, NY 10032, USA.; 2 Department of Internal Medicine, Division of Cardiology, The University of Texas Southwestern Medical Center, Dallas, TX 75390, USA.; 3 Department of Pediatric Cardiology, The First Affiliated Hospital, Sun Yat-sen University, Guangzhou 510080, Guangdong, China.; 4 Department of Biomedical Engineering, University of Alabama at Birmingham, Birmingham, AL 35249, USA.; 5 Cardiovascular Research Institute, National University of Singapore, and Genome Institute of Singapore, Singapore 119228, Singapore.; 6 Department of Biophysics, The University of Texas Southwestern Medical Center, Dallas, TX 75390, USA.; 7 Department of Molecular Biology, The University of Texas Southwestern Medical Center, Dallas, TX 75390, USA.; 8 Center for Regenerative Science and Medicine, The University of Texas Southwestern Medical Center, Dallas, TX 75390, USA.; 9 Centro Nacional de Investigaciones Cardiovasculares (CNIC), Madrid 28029, Spain.

**Keywords:** Hypoxia, regeneration, myocardial infarction, DNA damage, cell division, HIF2A

## Abstract

**Introduction:**

Gradual exposure to a chronic hypoxic environment leads to cardiomyocyte proliferation and improved cardiac function in mouse models through a reduction in oxidative DNA damage. However, the upstream transcriptional events that link chronic hypoxia to DNA damage have remained obscure.

**Aim:**

We sought to determine whether hypoxia signaling mediated by the hypoxia-inducible factor 1 or 2 (HIF1A or HIF2A) underlies the proliferation phenotype that is induced by chronic hypoxia.

**Methods and Results:**

We used genetic loss-of-function models using cardiomyocyte-specific HIF1A and HIF2A gene deletions in chronic hypoxia. We additionally characterized a cardiomyocyte-specific HIF2A overexpression mouse model in normoxia during aging and upon injury. We performed transcriptional profiling with RNA-sequencing on cardiac tissue, from which we verified candidates at the protein level. We find that HIF2A - rather than HIF1A - mediates hypoxia-induced cardiomyocyte proliferation. Ectopic, oxygen-insensitive HIF2A expression in cardiomyocytes reveals the cell-autonomous role of HIF2A in cardiomyocyte proliferation. HIF2A overexpression in cardiomyocytes elicits cardiac regeneration and improvement in systolic function after myocardial infarction in adult mice. RNA-sequencing reveals that ectopic HIF2A expression attenuates DNA damage pathways, which was confirmed with immunoblot and immunofluorescence.

**Conclusion:**

Our study provides mechanistic insights about a new approach to induce cardiomyocyte renewal and mitigate cardiac injury in the adult mammalian heart. In light of evidence that DNA damage accrues in cardiomyocytes with aging, these findings may help to usher in a new therapeutic approach to overcome such age-related changes and achieve regeneration.

## Graphical Abstract

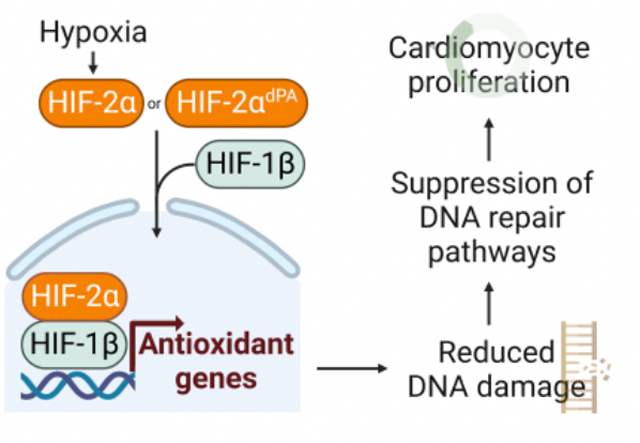

## INTRODUCTION

We previously showed that systemic exposure to a graduated hypoxic environment can promote cardiomyocyte proliferation and cardiac regeneration in mouse models^[1]^. Activation of DNA damage repair pathways is a potent anti-proliferative signal in cardiomyocytes^[2]^. We observed that lower oxygen tension leads to a concomitant reduction in oxidative stress that attenuates the DNA damage response. As a result, a fraction of cardiomyocytes enters the cell cycle during systemic hypoxia. However, the upstream transcriptional mechanism for this response remained uncharacterized. Given the prominent role of the HIF1 and HIF2 transcription factors in effecting the body’s response to acute and chronic hypoxia, we hypothesized that they could be central to mediating the transcriptional changes that result from chronic hypoxia.

## MATERIALS AND METHODS

### Mice

All mouse experiments were performed as per protocols approved by the Institutional Animal Care and Use Committee (IACUC) at the University of Texas Southwestern Medical Center (UTSW). All animal experiments complied with relevant ethical regulations on animal research. Mice had *ad libitum* access to water and food and were housed in 12:12 h light:dark cycles in a temperature-controlled room in the Animal Research Center at UTSW. The age of the animal is indicated in the text and/or in the figure legend for each experiment. Littermate controls were used whenever possible for experiments with multiple genotypes. Statistical tests were not used to predetermine sample size. All surgeries and echocardiographic studies were carried out blinded to the genotype of the mice during the experiments and outcome assessments. Myh6-MCM (#005657), HIF1A-floxed (#007561), HIF2A-floxed (#008407), and HIF2A-OE (#009674) mice were obtained from Jackson Laboratory. Hypoxia chamber experiments were performed as previously described^[1]^.

### Drug administration

Tamoxifen (Sigma) was dissolved in sesame oil (Sigma) at 20 mg/mL and administered by intraperitoneal injection. For MADM experiments, adult MADM mice (2–3 months old) were administered 2 weeks of tamoxifen (1mg every day for 14 days), and mice were sacrificed 2 weeks after the last tamoxifen dose.

### Mouse model of adult MI

Adult anterior wall myocardial infarction (MI) was performed as previously described^[1]^. In brief, 8-week-old mice were subjected to MI by ligation of the proximal aspect of the left anterior descending (LAD) coronary artery. Mice were anesthetized using 4% isoflurane, then endotracheally intubated and ventilated using a volume control ventilator with 100% O_2_ supplemented with 2% vaporized isoflurane (Harvard Apparatus). After lateral thoracotomy and pericardiectomy, the LAD coronary artery was identified. Prolene sutures (6–0 non-absorbable) were used to ligate the LAD. Vicryl sutures (6–0 absorbable) were used to close the thoracic cavity. Tamoxifen was administrated 1 week after MI at 0.5 mg every other day for 3 doses.

### Transthoracic echocardiography

Assessment of cardiac function was performed on conscious, non-sedated mice using a Vevo2100 micro-ultrasound system, MS400C probe (VisualSonics). Echocardiographic M-mode images were obtained from a parasternal short-axis perspective at the level of the papillary muscles. LV internal diameters at end-diastole (LVIDd) and end-systole (LVIDs) were determined by M-mode images. Six representative contraction cycles were selected for analysis, and average indices (LVIDs, LVIDs, EF, and FS) were determined for each mouse. All echocardiography measurements were performed by a blinded operator.

### Histology

The hearts were collected and treated in 4% paraformaldehyde fixative (in PBS) overnight at 4 0C and then processed for either paraffin or cryo embedding. Masson Trichrome staining was performed according to standard procedures at UTSW Histology core facility on paraffin sections.

### Immunofluorescence staining

Immunostaining was performed according to prior reports^[1]^. Briefly, heart cryosections were equilibrated with antigen retrieval buffer in epitope retrieval buffer (IHC World) or 1× citrate buffer (Antigen Retrieval Citra Plus, Biogenex). Samples were permeabilized and blocked with 0.3% Triton X-100 and 10% serum from the host animal of secondary antibodies in PBS for 1 h at room temperature. Then, the samples were incubated overnight at 4 0C with primary antibodies. After three washes in PBS, samples were incubated at room temperature for 1h with the corresponding fluorescence secondary antibodies conjugated to Alexa Fluor 488 or 555 (Invitrogen) at 1:400. The slides were mounted in Vectashield Antifade Mounting Medium (Vector Laboratories). Slides were viewed under Nikon fluorescence or Zeiss LSM 510 confocal microscopes. Primary antibodies: pH3 Ser10 (EMD Millipore, 06–570; 1:100); troponin T (Thermo Scientific, MS-295-P1; 1:200); 8-oxoG (Abcam ab64548, 1:25). DAPI was used for nuclear staining. Images were obtained on a Nikon Eclipse Ni or Nikon A1 laser scanning confocal microscopes.

### TUNEL staining

Cryo-sections underwent immunofluorescent staining for cardiac troponin T (as above.) After incubation with the corresponding secondary antibody (Alexa Fluor 555, Invitrogen), TUNEL staining was performed according to the manufacturer’s protocol (In situ Cell Death Detection Kit, Fluorescein, Roche). All staining was performed on three hearts per group, and three sections per heart.

### WGA staining and cardiomyocyte size quantification

WGA staining and quantification was performed as per prior report^[3]^. Briefly, the slides were incubated with WGA conjugated to Alexa Fluor 488 (50 mg/mL, Life Technologies) for 1 h at room temperature following PBS washes. To quantify the cross-sectional cardiomyocyte cell size, three to four independent hearts per genotype/group were captured at 40× magnification from three different views and positions (e.g., right ventricle, left ventricle, septum). Cellprofiler was used to quantify the size of cardiomyocytes, which were round and had a nucleus^[4]^. Quantification of at least 500 cells per sample was performed. For cell size after MI, WGA-Alexa Fluor 647 (Thermo Fisher W32466) was used with DAPI counterstain; 50 cells per sample were counted.

### Cardiomyocyte isolation

Adult hearts were freshly collected and fixed in 4% PFA at 4 0C overnight after removal of atria. The heart was cut from the apex towards the base twice (in perpendicular cuts) while preserving the basal connections to expose the inside surfaces of the heart. The hearts were subsequently incubated with collagenase type 2 (Worthington-Biochem, Cat# LS004176) supplemented with 1% penicillin-streptomycin (Thermo Fisher) overnight at 37 0C with constant rotation. For the next 7 days, the supernatant was collected twice a day and stored at 4 0C [supplemented with fetal bovine serum (Hyclone)] while additional collagenase 2 was added to the remnant cardiac tissue for further digestion. At the end of the 7-day period, the cardiac tissue was mostly connective tissue, and the pooled cells were allowed to settle by gravity before aspiration of the supernatant, followed by resuspension in 1 mL of PBS and passage through a 160 μm nylon mesh filter. The isolated cardiomyocytes were then counted after serial dilution using a bright field microscope.

### Western blotting

Ventricles were collected and lysed in RIPA buffer (Millipore Sigma) with the addition of a complete protease inhibitor cocktail (Roche). Protein concentration was determined using Pierce BCA protein assay kit (Pierce Biotechnology), with three biological replicates. Following separation via SDS-PAGE gels, proteins were transferred to nitrocellulose membranes (Bio-Rad), blocked in 5% skim milk/TBS, and incubated with primary antibodies: Gamma H2AX (Cell signaling 9718, 1:1,000); p-ATM (Santa Cruz Biotechnology sc-47739, 1:1,000); ATM (Genetex GTX70103, 1:1,000); p-CHK2 (Abcam ab59408, 1:1,000); CHK2 (Cell signaling 2662T, 1:1,000); p-CHK1 (Cell signaling 2348S, 1:1,000); CHK1 (Cell signaling 2360S, 1:1,000); GAPDH (Millipore AB2302, 1:6,000). Horseradish peroxidase-conjugated peroxidase anti-mouse, anti-rabbit, or anti-goat antibodies were used as secondary antibodies (ImmunoResearch: 115–035-166, 111–035-144, 703–035-155, 705–035-147; 1:25,000–1:50,000). The membranes were exposed using Licor Odyssey Fc system and quantified by Image Studio Lite v.5.2 software.

### qPCR and RNA sequencing

Snap-frozen heart tissues from Myh6-MCM control (*n* = 3) and experimental Myh6-MCM;HIF2A-OE (*n* = 3) were crushed with mortar and pestle under liquid nitrogen. The crushed samples were resuspended in TRIzol^™^ reagent (Thermo Fisher Scientific, #15596018) and total RNA was prepared using Direct-zol^™^ RNA miniprep (Zymo Research, #R2061) according to the manufacturer’s protocol. RNA integrity was assessed using the Agilent bioanalyzer and only samples with RIN > 6 were used for the library prep. Paired-end libraries were constructed using Tru-seq kits (Illumina) and the libraries were sequenced on the Hiseq4000, generating 2 × 151-bp paired-end reads. Reads were mapped against the mouse genome using Tophat version 2.0.11 with default parameters. Gene count was computed using htseq-count. Differential gene expression analysis was performed using Edge R.A gene and was considered to be differentially expressed between control and experimental samples if the FDR-adjusted *P*-value was less than 0.05.

## RESULTS

**Figure 1.**
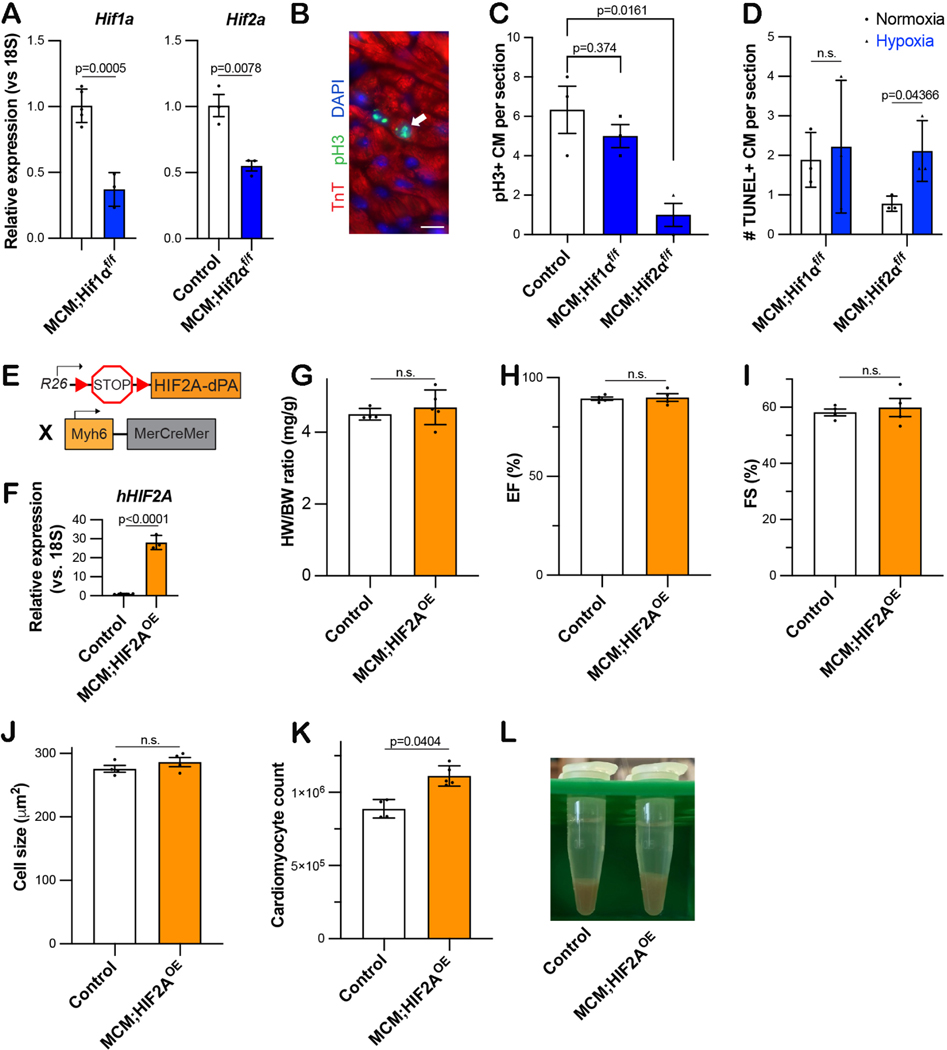
HIF2A is necessary for hypoxia-mediated cardiomyocyte proliferation. (A) qPCR for HIF1A and HIF2A in control and MCM;HIF1A^f/f^ or HIF2A^f/f^ mice, respectively, in normoxia. (B) Quantification of pH3+ cardiomyocytes in hypoxia-exposed control, Myh6-MCM;HIF1A^f/f^, and Myh6-MCM;HIF2A^f/f^ mice. (C) Representative image of pH3-labeled cardiomyocyte nucleus (white arrow) in Myh6-MCM; HIF2A^f/f^. (Scale bar 10 μm). (D) TUNEL staining quantification in the hearts of control, Myh6-MCM;HIF1A^f/f^, and Myh6-MCM;HIF2A^f/f^ mice placed in chronic hypoxia. (E) Genetic model used for HIF2A-OE experiments. (F) qPCR for human HIF2A in control and MCM;HIF2A-OE hearts, relative to 18 S. (G) Heart weight-body weight ratio (mg/g) of control and MCM;HIF2A-OE mice. (H-I) Cardiac function assessed by EF and FS of control and MCM;HIF2A-OE mice. (J) Average cardiomyocyte cell size determined by WGA quantification of control and MCM;HIF2A-OE mice. (K) Mean number of isolated cardiomyocytes digested from control and MCM;HIF2A-OE hearts. (L) Representative cell pellets derived upon digestion of control and MCM;HIF2A-OE hearts. pH3: Phosphorylated histone H3; EF: ejection fraction; FS: fractional shortening; TUNEL: terminal deoxynucleotidyl transferase dUTP nick end labeling; OE: overexpression; WGA: wheat germ agglutinin. Each dot represents one biological replicate.

We generated cardiomyocyte-specific (Myh6-MCM, aka MCM) homozygous HIF1A- or HIF2A-floxed mice (cKO), and we validated efficient gene deletion using qPCR [Figure 1A] We exposed these mice to the graduated hypoxia protocol along with control mice. Following the 4-week hypoxia protocol, the number of cardiomyocytes in mitosis - as indicated by the presence of phosphorylated Histone H3 (pH3) - was similar in control and MCM;HIF1A^f/f^ mice, whereas the MCM;HIF2A^f/f^ mice showed a ~70% reduction in pH3+ CMs [Figure 1B and C]. These data indicate that HIF2A is necessary for cardiomyocyte proliferation upon systemic hypoxia, suggesting that it is the key mediator of this phenomenon. In addition, there were more apoptotic cardiomyocytes - based on TUNEL expression - in HIF2A cKO hearts compared to HIF1A cKO or control hearts [Figure 1D], indicating a protective role for HIF2A during chronic hypoxia.

**Figure 2.**
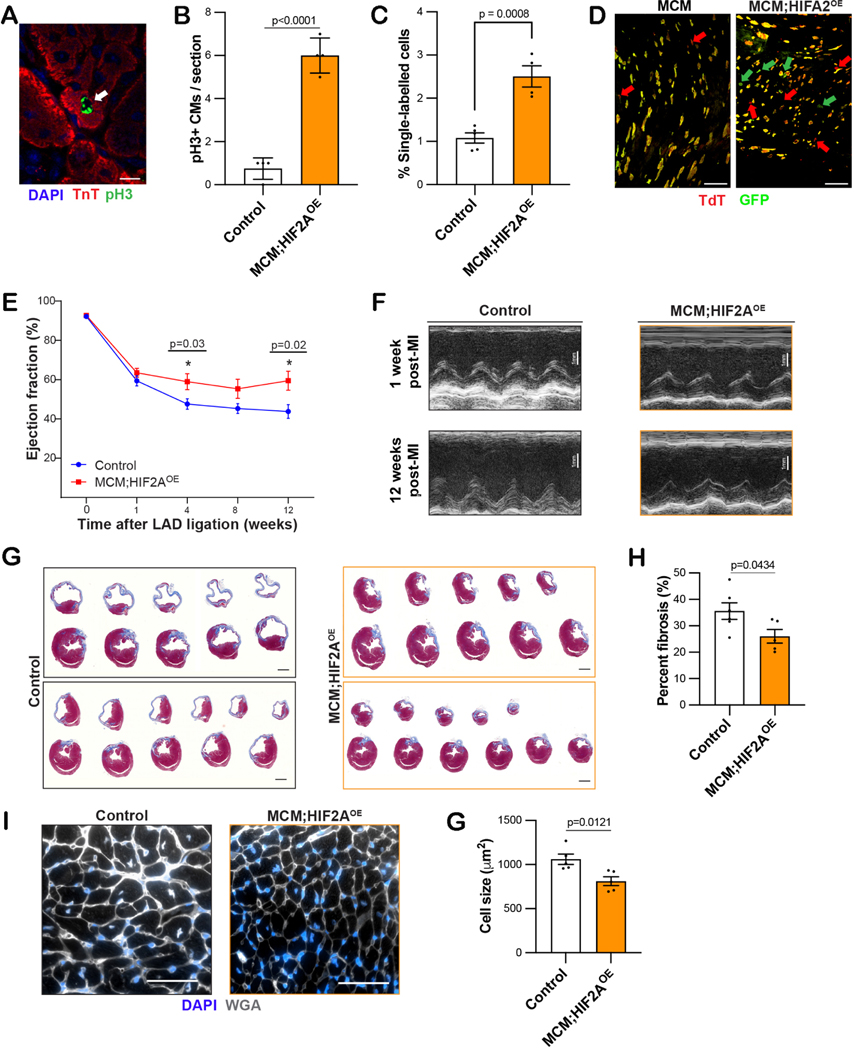
Ectopic HIF2A promotes cardiomyocyte proliferation and improves cardiac function after injury. (A and B) Representative pH3+ cardiomyocyte nucleus (white arrow) from a MCM;HIF2A-OE heart and quantification (scale bar 10 μm). (C and D) MADM quantification and tissue section images of single-labeled and double-labeled cells from control and MCM;HIF2A-OE tissue sections. Red and green arrows point to single-labeled CMs (scale bar 80 μm). (E and F) Baseline and post-injury ejection fraction of control and MCM;HIF2A-OE mice following adult LAD ligation-induced MI, along with representative M-mode echocardiographic images. (G and H) Mean percent fibrosis in control and MCM;HIF2A-OE hearts 12 weeks after injury using Masson trichrome staining and representative cross-sectional images. (I and J) Cell size in control and MCM;HIF2A-OE hearts 12 weeks after MI using WGA staining, along with representative images. MADM: Mosaic analysis with double markers; LAD: left anterior descending artery; OE: overexpression. Each dot represents one biological replicate. *P < 0.05 by unpaired t test in (E).

To test this hypothesis, we used Rosa26^HIF2A-dPA/+^ knock-in mice to overexpress hydroxylation-resistant human HIF2A in cardiomyocytes by crossing it with Myh6-MCM mice (“HIF2A-OE”) [Figure 1E], which we confirmed with qPCR [Figure 1F]^[5]^. At baseline, these mice have normal cardiac structure and heart weight-body weight ratio (mg/g) [Figure 1G], and the systolic function is also preserved [Figure 1H and I]. While the cardiomyocytes had a similar size [Figure I and J, there were more CMs present in adult HIF2A-OE hearts compared to the control hearts four weeks after tamoxifen induction (8.8 × 10^5^ *vs.* 11 × 10^5^ CM/heart, *P* = 0.0404) [Figure 1K and L]. In addition, there was a significant increase in cardiomyocyte proliferation in the HIF2A-OE mice relative to controls (5.8 *vs.* 1.1 pH3+ CMs per section, *P* < 0.0001) [Figure 2A and B]. To determine whether this mitotic activity led to completed cell division, we generated Myh6-MCM;MADM;HIF2A^OE^ mice, which had a two-fold increase in the percent of single-labeled cardiomyocytes compared to control Myh6-MCM;MADM control mice. Since single-labeled cells in the MADM model can only arise through the completion of cytokinesis, this finding indicates that twice as many cardiomyocytes were born upon stable HIF2A overexpression compared to baseline [Figure 2C and D]^[6]^.

To determine if this degree of cardiomyogenesis can ameliorate cardiac function after injury, we performed myocardial infarction (MI) in adult mice by ligation of the left anterior descending artery (LAD). One week later, we checked the systolic function by echocardiography and continued the experiment on mice whose ejection fraction (EF) was in the 45%−75% range to ensure a similar degree of injury (all mice had a normal EF at baseline). These mice were treated with tamoxifen at 1 week post-MI to induce Cre recombination. Serial echocardiography demonstrated that control mice experienced progressive systolic dysfunction, with a mean 12-week EF of 44% [Figure 2E and F]. In contrast, the HIF2A-OE mice initially maintained and then slightly improved their cardiac function, with a mean 12-week post-MI EF of 60% [Figure 2E and F]. After the 12-week time point, the hearts were explanted and the fibrotic scar size was quantified: HIF2A-OE mice had a smaller scar area relative to the control mice (26.1% *vs.* 35.6% using Masson Trichrome staining, [Figure 2G and H]. Moreover, the cardiomyocyte cell size was smaller in HIF2A-OE relative to control hearts [Figure 2I and J]. These data collectively indicate that overexpression of oxygen-stable HIF2A can renew functional myocardial tissue after injury, as characterized by improved ventricular function and diminished scar formation.

**Figure 3.**
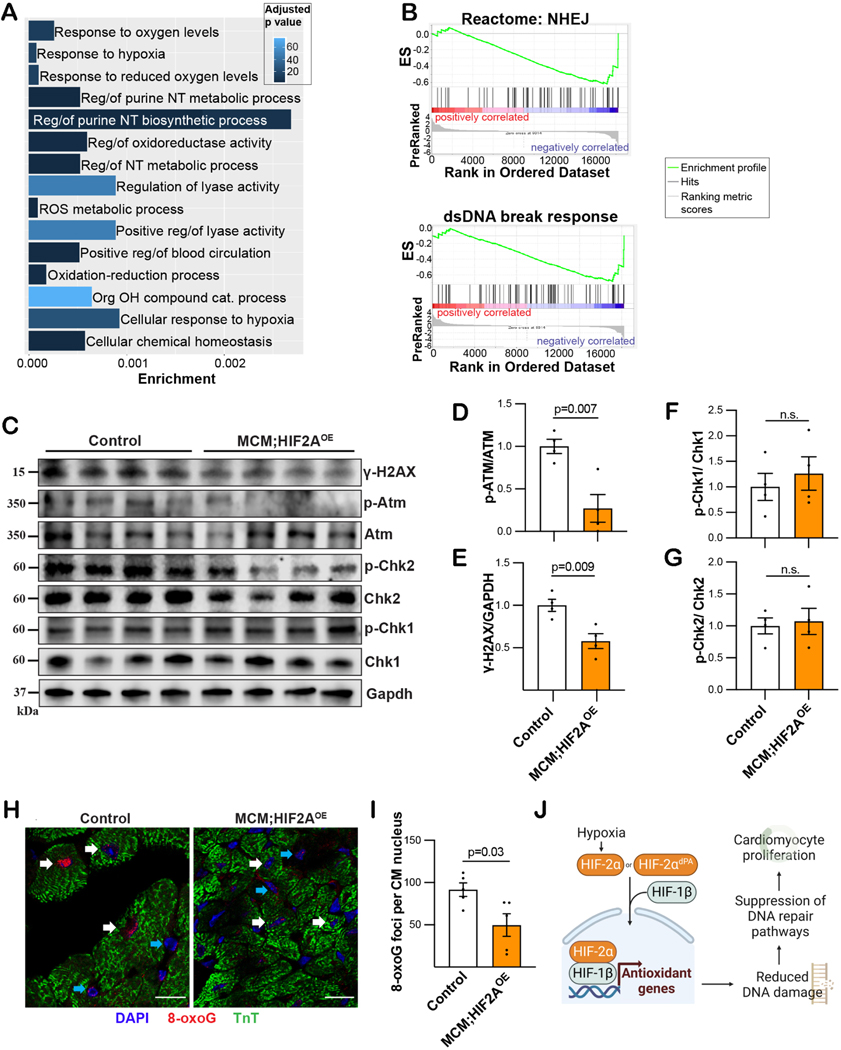
Ectopic HIF2A is associated with less DNA damage. (A) Gene ontology analysis by Gorilla algorithm shows pathways that are significantly upregulated in MCM;HIF2A-OE relative to control hearts. Reg/of: Regulation of; NT: nucleotide; ROS: reactive oxygen species; Org: organic; OH: hydroxy; cat: catabolic. (B) Gene Set Enrichment Analysis (GSEA) shows that pathways associated with DNA damage and repair are upregulated in the control hearts. Zero cross is at 9014. Top: non-homologous end-joining (NHEJ) and bottom: DNA double-strand break response. EF: Enrichment score. (C-G) Quantification and immunoblots of DNA damage-associated proteins in control and MCM;HIF2A-OE hearts. (H and I) Representative immunofluorescent stained cardiac tissue sections and quantification of 8-oxoG nuclei from control and MCM;HIF2A-OE hearts. (J) Proposed schematic for HIF2A mechanism in CMs (created with BioRender.com.) OE: Overexpression; NHEJ: non-homologous end-joining; dsDNA: double stranded DNA; ES: enrichment score. Each dot represents one biological replicate.

To delineate the mechanism by which HIF2A can promote cardiomyocyte proliferation and cardiac regeneration, we evaluated the transcriptional output after HIF2A overexpression in normoxia. We performed RNA-seq on cardiac lysate from Myh6-MCM control and Myh6-MCM;HIF2A^OE^ models four weeks after tamoxifen treatment. Bioinformatic analysis using Gene Ontology and GSEA showed that the most upregulated pathways in the HIF2A-OE hearts were related to angiogenesis, as well as several pathways involved in oxidation-reduction reactions [Supplementary Table 1, Figure 3A and B]. HIF2A activates antioxidant genes, and HIF2A^−/−^ mice exhibit lower levels of antioxidant gene expression and more oxidative damage in multiple organs (including the heart)^[7]^. Our prior data indicate that suppression of DNA damage - which can be caused by oxidative stress - can promote adult murine cardiomyocyte proliferation. Therefore, we hypothesized that HIF2A upregulates genes that can mitigate oxidative damage and elicit cardiomyocyte renewal. Signaling caused by DNA damage activates a series of sensors (Mre11-Rad50-Nbs1 complex), mediators (ATM, ATR/DNA-PKc), and effectors (Chk1, Chk2, *etc.*) of the damage response^[8,9]^. To investigate the DNA damage response in cardiomyocytes following HIF2A overexpression, we examined the activity of both the ATM mediator and the effectors Chk1 and Chk2 using immunoblot [Figure 3C–G]. Upon HIF2A-OE, there is a significant decrease in ATM activity [Figure 3C] without Chk1/2 activation [Figure 3D and E], using phosphorylation as an indicator of activity. This finding suggests that the overexpression of HIF2A reduces DNA damage and does not alter DNA damage response or repair (through effector activity). Concordantly, we also found that HIF2A-OE hearts had decreased expression of y-H2AX [Figure 3F], a marker of double-strand DNA breaks^[10]^. We next analyzed tissue sections for evidence of DNA damage directly in cardiomyocytes, which showed fewer oxidatively damaged guanine residues as determined by quantification of 8-oxoG puncta in cardiomyocyte nuclei [Figure 3H and I]. Therefore, ectopic expression of an oxygen-stable HIF2A in cardiomyocytes during normoxia lessens DNA damage and, thereby, suppresses DNA damage-activated pathways that inhibit cell proliferation [Figure 3J].

## DISCUSSION

We previously showed that cardiomyocytes experience an increase in oxygen tension upon birth (compared to the relative intrauterine hypoxia), which is accompanied by a shift from anerobic glycolysis to mitochondrial metabolism^[2]^. One important consequence of this postnatal reliance on mitochondrial oxidation is increased ROS production and DNA damage, which activates the DNA damage response and dampens the cell cycle to render the heart a non-regenerative organ. Here, we expand on our previous work and show that hypoxia amelioration of the DNA damage response pathway - and the attendant increase in cardiomyocyte proliferation - is achieved in a HIF2A-dependent manner; HIF1A is dispensable for this phenomenon. Consistent with our findings, a recent study showed that human aging is associated with the acquisition of DNA mutations in cardiomyocytes due to oxidative DNA damage and inefficient repair pathway activation^[11]^. Therefore, hypoxia and HIF2A may help overcome some of the deleterious effects of aging in the heart.

However, it is likely that other factors also contribute to the hypoxia phenotype, as a comparison of the degree of cardiomyogenesis using the MADM model suggests that hypoxia induces a greater degree of cardiomyocyte proliferation than cardiomyocyte-specific HIF2A overexpression. These factors could include - but are not limited to - endothelial-specific Hif expression and epigenetic changes that occur in an oxygen-dependent manner (e.g., histone lysine demethylase suppression)^[12–14]^.

It was reported that loss of HIF2A in cardiomyocytes (but not HIF1A) worsens infarct sizes after ischemia-reperfusion (IR) injury, which was shown to be upstream of the epidermal growth factor amphiregulin (AREG); recombinant AREG protein could rescue the loss of HIF2A^[15]^. However, we did not detect significant AREG expression in our RNA-seq experiment. Similarly, IL-6 was shown to act downstream of HIF2A in IR injury, but there was no significant difference in IL-6 expression in the heart by RNA-seq^[16]^. These differences could be due to a different pattern of HIF2A-activated genes in hypoxia relative to normoxia or relative to IR injury.

This work also helps to reconcile prior reports that stabilized Hif transcription factors at the protein level either through deletion of VHL or PHD2 yet revealed distinct outcomes. For example, cardiomyocyte-specific PHD2 deletion led to a cardiomyopathy phenotype and worsening of cardiac function in one study^[5]^, whereas other studies demonstrated that the decline in cardiac function after injury is mitigated in cardiomyocyte-specific PHD2 knockout mice^[17,18]^. First, the majority of the literature supports the notion that excess HIF1A signaling is detrimental to cardiac function, and our data concordantly shows that the benefit of hypoxia is not due to HIF1A^[19]^. Second, our report suggests that the age at which Hif signaling is activated and its duration are important. For example, the use of Myh6-Cre to activate HIF2A-dPA leads to a decline in systolic function by 11 weeks of life: in this model, Cre recombinase activity starts in late embryonic development and persists^[5]^. Here, we predominantly induced ectopic HIF2A in the adult heart, and we did not observe a deleterious effect in uninjured mice at 4 weeks following tamoxifen treatment. It is worth noting that hypoxia leads to a steady improvement in EF after MI, whereas with HIF2A-OE the degree of EF improvement after MI is less than with hypoxia^[1]^. This difference between hypoxia exposure and HIF2A overexpression could be a result of non-Hif2-mediated effects of hypoxia, or a result of excess HIF2A signaling in the OE model. Therefore, strict control of HIF2A activity via a temporal burst of HIF2A downstream signaling may be more salutary to cardiac function, which could allow us to harness the therapeutic potential of chronic hypoxia.

## Supplementary Material

Supplementary Material

## Data Availability

The data that support the findings of this study are available within the paper and its Supplementary Materials. Source data or other materials are available from the corresponding authors upon reasonable request.
