# Supplementary material for: Hypoxia-induced stabilization of HIF2A promotes cardiomyocyte proliferation by attenuating DNA damage
Source: J Cardiovasc Aging. Author manuscript; Available in PMC 2024 Mar 7. (PMC10919901; doi:10.20517/jca.2023.43)
Supplement: Supplementary Material [file NIHMS1963614-supplement-Supplementary_Material.pdf]

Hypoxia-induced stabilization of Hif2a promotes cardiomyocyte proliferation by attenuating DNA damage

Shah R. Ali[1,#], Ngoc Uyen Nhi Nguyen[2,#], Ivan Menendez-Montes[2], Ching-Cheng Hsu[2], Waleed Elhelaly[2], Nicholas T. Lam[2], Shujuan Li[3], Abdallah Elnwasany[2], Yuji Nakada[4], Suwannee Thet[2], Roger S. Y. Foo[5], Hesham A. Sadek[2,6,7,8,9]

[1]Department of Medicine, Division of Cardiology, Columbia University Irving Medical Center, New York, NY 10032, USA.
[2]Department of Internal Medicine, Division of Cardiology, University of Texas Southwestern, Dallas, TX 75390, USA.
[3]Department of Pediatric Cardiology, the First Affiliated Hospital, Sun Yat-sen University, Guangzhou 510080, Guangdong, China.
[4]Department of Biomedical Engineering, University of Alabama at Birmingham, AL 35249, USA.
[5]Cardiovascular Research Institute, National University of Singapore, and Genome Institute of Singapore, Singapore 119228, Singapore.
[6]Department of Biophysics, University of Texas Southwestern, Dallas, TX 75390, USA.
[7]Department of Molecular Biology, University of Texas Southwestern, Dallas, TX 75390, USA.
[8]Center for Regenerative Science and Medicine, University of Texas Southwestern, Dallas, TX 75390, USA.
[9]Centro Nacional de Investigaciones Cardiovasculares (CNIC), Madrid 28029, Spain.
[#]Authors contributed equally.

Correspondence to: Prof. Shah R. Ali, Department of Medicine, Division of Cardiology, Columbia University Irving Medical Center, Black Building, 650 W. 168th St. New York, NY 10032, USA. E-mail: sra2180@cumc.columbia.edu; Prof. Hesham A. Sadek, Department of Internal Medicine, Division of Cardiology, University of Texas Southwestern, Simmons Biomedical Research Building, 6000 Harry Hines Blvd. Dallas, TX 75390, USA. E-mail: Hesham.sadek@utsouthwestern.edu

ORCID: Shah R. Ali(0000-0002-1241-6826)

Supplementary Table 1. **RNA-seq data from control (Myh6-MCM) and Hif2a-OE (Myh6-MCM;Hif2a-OE) adult mice (*n* = 3 each) administered tamoxifen at P60 and heart explanted and RNA obtained 4 weeks later**

FEATURE_NAME	WT314	WT361	WT366	TG363	TG364	TG365
Egln3	4009	2499	2791	23748	20624	27849
Dpp6	5	1	5	96	126	91
Ddit4	1165	650	569	1697	1507	2156
Adh1	959	625	531	1639	1065	3058
C1qtnf9	2529	1695	1439	3345	2627	4476
Ccdc85a	1282	822	672	1679	1111	2490
Stc1	164	87	102	246	309	449
Scarb1	1161	739	698	1464	1147	2299
Rgcc	970	626	491	1125	1107	2114
Hecw2	1233	791	759	1707	1119	2768
Rsad2	1483	1180	812	2816	1350	2915
Nos2	461	263	239	638	406	844
Sema7a	1376	949	911	1885	1409	2633
Aqp1	18451	11619	9566	25451	16595	34239
Papln	420	196	179	528	367	775
Akap5	154	134	125	277	266	509
Adm	406	218	169	677	442	563
Stab2	24	20	9	64	78	70
Car4	1254	717	696	1368	1357	2259
Pfkp	1614	1197	1093	2753	2838	2739
Arrdc2	457	285	260	540	518	1134
Kcna5	1027	630	705	1398	958	2311
Klra9	66	64	21	152	98	195
Gpr56	2460	1367	1160	2873	1743	4149
Hsph1	3557	2839	4566	2217	1356	2218
Sgk1	3259	1726	1926	3503	3545	7070
Prg4	848	583	591	1015	1167	2025
Slc10a6	164	97	107	221	207	417
1810011O10Rik	3297	2086	1844	3751	2981	5489
Ptp4a3	9937	5875	4915	11383	9659	14714
Sh2d3c	986	586	481	1019	895	1769
Sprr1a	2	2	0	8	8	57
Cd200	3641	2486	2312	4837	3197	6265
Ces2e	57	32	39	108	69	152
Cilp	402	306	311	774	372	1094
Gm7120	747	397	339	270	166	477
Itpripl1	531	341	268	631	479	880
Pcdh17	596	369	361	676	643	1145
Rnf144a	962	537	536	1158	764	1581
Hbb-b2	2833	3811	1859	1840	1245	2012
Hbb-bt	2833	3811	1859	1840	1245	2012
Ssu2	50	21	27	90	58	107
Ednrb	915	671	685	1190	1093	1893
Cdc42ep4	678	376	400	827	545	1390

Fam102b	1479	927	863	1653	1281	2852
Timp4	1790	1001	1059	2834	1200	3857
Prkch	1088	691	653	1304	883	1861
Nfkbia	1465	903	794	1471	1425	2382
St8sia4	1575	903	730	1695	1156	2203
Robo4	1880	1102	843	2035	1351	2884
Tie1	3894	2211	2015	4121	3008	6358
Alox5	534	302	280	244	192	328
Trp53i11	647	333	315	714	503	971
Myo1d	778	549	482	1002	659	1279
Chst15	453	261	252	498	432	787
Cd109	172	111	83	226	150	375
Efnb2	1838	1044	888	2024	1585	2341
Elk3	2490	1426	1165	2631	1871	3410
Ablim3	3932	2505	1978	4124	3199	6566
Sult1a1	1858	1336	1126	2153	1649	4195
Cacna1h	250	145	150	416	262	353
Bnip3	5718	3466	3329	6050	7176	9219
H2-Q4	1558	905	648	1520	1190	2419
Spry1	1263	666	518	1415	877	1666
Selenbp1	2872	1549	1223	2842	2139	3793
Esam	2197	1223	1264	2348	1720	3978
Mcf2l	3079	1819	2009	3525	2673	6455
S1pr1	3992	2984	2538	4756	3908	7218
Snca	231	316	176	151	87	186
Snrk	5112	2895	2684	5242	4412	7743
Gata2	828	536	460	900	677	1368
Arhgap27	799	542	362	870	618	1452
Bche	4102	1887	1738	2154	1199	2055
Exoc3l	143	76	70	176	132	261
Fscn1	758	394	429	857	556	1541
Fam46c	114	144	60	48	45	47
Sez6l2	208	121	88	278	143	488
Gpihbp1	2011	1132	1104	2007	1602	3748
Abcb1a	1686	1035	1109	1799	1669	3055
Aplnr	1687	1112	776	2136	1170	2460
Fmnl3	1618	1004	737	1833	1114	2237
Gypa	49	50	28	14	20	14
Aoc3	867	530	357	1182	566	1237
B4galt4	714	508	459	894	614	1190
Car7	186	102	93	211	173	325
Grap	393	218	228	454	326	674
Srms	11	13	12	33	26	54
Cd38	831	535	614	932	946	1750
Dll4	945	545	496	1119	663	1305

Mmrn2	2127	1407	1233	2305	1747	3263
Xdh	10182	6443	5591	11115	8098	16428
Cxcr4	278	183	140	393	221	434
Galnt15	626	402	315	749	509	855
Gatsl3	129	101	80	178	134	332
Aqp7	1125	611	597	1007	994	2203
Tshz2	1373	854	741	1373	1127	2050
Cdh5	12088	7908	6699	13032	10077	20606
Fzd4	2703	1810	1645	3014	2176	4120
Hbb-b1	17237	23808	12285	11863	8841	13462
Hbb-bs	17237	23808	12285	11863	8841	13462
Myh7b	6439	5084	3153	3621	2764	4793
E330017L17Rik	109	73	74	179	134	184
Nxpe4	912	540	470	911	684	1522
Kctd12b	1485	875	926	1656	1200	2199
Mllt4	6074	4130	3146	6329	5688	8719
Esm1	26	25	24	72	73	59
Ly6c1	5075	3126	2470	5190	3891	7494
Rps2	1926	1243	1019	3627	1078	4130
Sox17	841	449	368	758	738	1170
Alas2	1224	1778	931	975	647	963
Bai2	160	110	61	239	134	254
Cd247	47	49	29	69	83	168
Fxyd6	649	478	328	817	489	1072
Gt(ROSA)26Sor	421	174	252	138	94	289
Tspan15	344	238	288	465	380	837
Sybu	350	214	132	415	299	466
Tubb1	84	51	48	31	30	38
Ptgs1	482	282	211	501	364	741
Aldh1l1	155	104	134	203	196	466
Clec1a	1002	664	652	1083	886	1812
Lrrn1	42	15	14	57	34	86
Chek2	208	125	126	235	282	334
Entpd1	1252	1015	625	1565	1142	1774
Tspan13	3899	2478	2302	4178	3005	6730
Gcnt1	276	172	191	404	355	389
Cd93	5018	3354	3442	5931	4534	8838
Fmo1	4214	2845	2063	4365	3348	6191
Mir8114	217	165	124	121	91	123
F2rl3	12	3	4	16	19	30
H2-Q6	385	120	119	323	322	562
H2-Q8	385	120	119	323	322	562
Tbx2	386	228	240	446	335	627
Timeless	736	471	302	780	572	1025
Kctd14	6	5	4	11	41	20

Slc4a1	111	141	67	45	41	89
Scarna2	4	4	1	0	0	0
Mylk4	18999	11355	9361	9128	8162	12710
Ppargc1a	17830	12810	9938	12120	7716	11229
Adam12	14	12	11	26	22	71
Erg	944	676	609	1178	738	1573
Hist2h4	7	4	2	0	0	1
Ldha	21830	12720	11708	26686	17773	27993
Akr1c14	780	377	360	651	614	1436
Hsp90aa1	2121	1454	2750	1572	912	1685
Lama3	213	120	76	229	197	291
Podxl	11686	7954	6217	13338	9529	16430
Flt1	9993	6547	5265	10597	8462	13870
Cd300lg	4282	2594	2399	3880	3850	7988
Btla	43	14	29	54	50	120
Fmo2	5573	3142	2869	5340	4861	7607
Mylip	1820	1192	980	1704	1605	2935
Mxd4	997	504	443	1034	614	1382
Apol11b	42	62	20	19	12	23
Sema6a	1498	1013	884	1601	1341	2059
Adarb1	1731	1124	996	1817	1474	2413
Zfp366	580	391	299	684	516	751
Gpr116	22850	15370	12597	24416	19607	36970
Kank3	2693	1490	1215	2396	1830	4199
Mdfic	1041	641	508	1065	742	1419
Pcdh12	1049	629	436	1338	658	1334
Sncaip	563	425	316	701	466	854
Itgb6	654	500	537	459	337	496
Spock2	583	372	274	697	486	713
Cyb5r3	2897	1968	1441	3067	1989	4351
Prom2	17	9	12	7	1	4
Lhx6	302	173	137	294	259	470
Atp2b4	1880	1349	1110	2059	1467	3073
Capn11	5	0	1	145	3	0
Hist2h2bb	24	0	0	0	0	0
2810025M15Rik	842	529	403	968	531	1271
Abcg2	1929	1262	1403	2208	1705	3504
Dapk2	170	120	97	221	164	270
Gm960	0	0	0	2	3	4
Rad51ap1	34	13	11	38	29	81
Slc18a1	9	3	6	21	16	20
Snora74a	8	4	5	1	0	2
Ttc28	1661	1083	739	1775	1205	2074
4632428N05Rik	1259	844	719	1309	999	1839
Il19	5	2	1	0	0	0

Cubn	479	265	187	217	176	257
Rrp12	912	584	624	578	421	748
Bmp10	16	0	20	3	0	0
Col28a1	70	40	70	142	69	228
Dennd3	541	289	344	581	440	916
Paqr9	1655	1163	1009	1186	780	1340
Dok4	709	408	366	824	452	1054
Myh11	2237	1588	1820	3742	2264	3232
Apod	450	286	226	476	382	626
Mir25	0	0	0	2	4	3
Pfkfb2	11812	8280	7131	8014	5723	8577
H2-T22	844	431	334	969	466	1193
H2-T9	839	429	331	962	464	1182
Cebpd	564	330	264	611	482	669
Egfl7	2445	1226	1187	2115	1755	3627
Fam124b	19	12	12	40	19	47
Gipr	9	17	8	25	35	41
Narf	6002	3626	3388	3998	2645	3972
Polq	667	528	568	524	244	617
Tmem88	448	261	308	507	383	824
Tspan14	611	338	288	702	381	872
Gpx3	8877	6297	5541	10741	7984	12294
Mgme1	1261	775	605	791	542	789
Pald1	820	526	480	899	574	1381
Mmp28	157	96	68	221	108	248
Dusp6	940	595	567	902	851	1537
Gm10069	39	28	33	24	9	19
Nrp2	2321	1499	1303	2295	1738	3631
Rin3	793	507	333	923	490	1171
Spp1	11	12	8	14	13	160
AU021092	199	140	104	204	198	364
Ccdc28b	263	154	154	284	219	439
Serpinf2	2	3	2	0	0	0
Draxin	12	11	7	3	4	2
Fam46b	45	27	20	101	39	67
Gja4	304	204	139	378	233	424
Ptprb	14177	8974	7526	14474	10660	21813
Slc9a2	584	294	248	324	188	311
Calcrl	2669	1731	1218	2834	1787	3418
Olfr558	425	233	210	502	294	552
Kel	1	5	2	0	0	0
Gm16793	617	396	351	361	297	371
Tnfrsf21	723	461	415	870	479	1137
Fjx1	21	10	14	25	31	59
Ptprg	4790	2990	2584	4687	3633	7054

Dpysl2	1161	758	673	1394	785	1687
Gbp9	1648	1081	860	1455	1488	2582
Nav3	236	171	197	230	358	700
Tmem204	1286	834	703	1559	845	1767
Pde2a	4057	2565	2114	4171	2891	5547
Apol10b	211	194	130	265	258	393
Gm7609	18	5	5	20	21	34
Lfng	205	114	67	267	128	293
Lipe	2173	1116	882	1836	1432	3127
Smco4	286	201	183	319	259	541
Pcdh1	1352	729	547	1270	859	1752
Cirbp	1308	1005	737	1207	1284	2418
Msrb1	2186	1557	1461	2830	1486	4200
Cxcl9	244	168	140	267	189	588
Tbxa2r	31	27	22	59	35	77
Tmem181b-ps	962	608	463	367	464	602
Utp14a	1236	846	598	820	521	925
Lao1	5	2	1	8	10	14
Gabra1	35	8	23	41	43	80
Hpcal1	571	361	255	692	340	909
Hr	895	795	555	758	338	702
Phactr1	569	398	267	592	510	770
Rpph1	28	20	0	0	4	2
Mrvi1	811	533	449	947	637	1006
Serpini1	660	458	429	843	495	1041
Sh3tc2	246	148	135	276	171	454
4931406P16Rik	3208	1921	1679	3054	2294	4230
Tap1	624	295	202	591	373	850
Tspan17	262	141	147	259	218	434
Nlrp4f	0	0	0	6	2	1
Masp1	231	203	153	405	342	300
Bmp6	1204	776	748	1145	1018	2057
Pik3r6	356	184	153	330	271	492
Cyp4f18	15	8	4	18	14	47
Hba-a1	9464	14197	4830	6510	4756	7122
Hba-a2	9464	14197	4830	6510	4756	7122
Ptprr	129	79	73	135	119	250
F13a1	1885	1256	1060	1203	771	1739
Dnm3	333	231	168	323	292	582
Klhl4	425	290	293	430	432	835
Atp6v0c	329	208	212	650	213	527
Atp6v0c-ps2	329	208	212	650	213	527
Kcnj15	60	35	47	84	74	119
Pdgfrb	3948	2472	1765	4429	2571	4862
Akt1	8537	5382	4464	5683	3620	6605

Lmo3	0	0	0	5	1	2
Selenbp2	32	0	0	0	0	1
Tagln	818	532	722	1467	743	1336
Cntn1	3	2	1	0	0	0
Spata6	610	363	295	604	414	925
Utp20	1172	868	639	761	578	959
Marcks	3782	2222	1996	4031	2329	5723
Hspa8	20596	16611	14425	15518	10286	17553
Rnf152	370	194	223	417	289	522
Sla2	7	4	2	0	0	2
Trib2	2329	1358	1153	2745	1735	2471
Ets2	6161	3513	2829	6428	4598	6910
Zfp36	1348	872	822	1283	1073	2272
C130074G19Rik	1461	751	732	1123	1191	2331
A630019I02Rik	19	22	20	10	10	7
Id1	1126	621	647	1021	854	1798
Sox18	1033	562	446	1096	641	1210
Rarg	929	557	528	957	705	1204
Rgs12	1018	653	551	1098	718	1331
Btbd3	2028	1284	788	2079	1360	2539
Notch4	2025	934	891	2002	1144	2657
Cd177	0	0	0	2	1	4
Oas1g	20	9	6	23	16	53
Fam117b	2138	1121	935	2221	1181	2934
Tspan9	2802	1960	1781	3405	2256	3606
Gprc5b	962	692	549	1100	649	1747
Bcl2l1	1861	937	958	1765	1147	2937
8430427H17Rik	257	173	137	269	214	416
Myo1b	1938	1183	1124	1948	1383	2785
Ramp2	1249	826	573	1411	744	1895
Ankrd63	188	112	79	321	135	218
Thsd1	580	313	253	569	392	734
Apobec3	555	392	286	534	447	959
Kcnip2	11028	6235	5238	7160	3887	7910
Pdgfb	1622	750	746	1417	1026	2139
Tmem144	2730	1599	1459	1617	1342	1764
Cmpk2	607	324	255	720	325	870
Col13a1	44	30	19	45	69	80
Il1b	23	10	16	9	3	10
Met	125	86	105	67	59	91
Sgms1	2525	1637	1727	2452	2386	4085
Maml2	649	514	446	746	617	1066
Emp2	2970	1664	1308	2799	2073	3306
Hcn1	220	181	138	128	107	173
Prkd2	922	543	459	938	577	1300

Sema6b	394	243	214	396	283	690
Id3	1086	513	537	1086	650	1450
Ltbp2	73	99	97	215	99	300
Fli1	1445	947	921	1327	1345	2351
Gm15471	0	0	1	3	2	7
Ccm2l	749	449	440	659	697	1159
H2-T23	702	505	325	826	449	1121
Sdc3	3137	1942	1572	3332	1878	4419
Slc38a1	2775	1988	1764	2382	821	1986
Wdr27	1	0	1	3	4	8
Zbp1	50	35	29	76	37	128
Gabrb2	34	13	21	44	27	88
Slco2a1	183	78	70	181	149	218
Tsc22d3	6221	3827	3245	7472	4051	8029
Epha2	339	216	200	367	277	512
Klf2	1626	929	986	1424	1416	2589
Myrfl	106	93	70	57	57	52
A530016L24Rik	860	517	456	809	998	1011
Btaf1	1917	1127	1114	1279	818	1617
Map3k6	751	476	352	931	411	1208
Ushbp1	2132	1125	1026	1635	1699	3198
Vmn2r3	5	1	9	1	0	1
Mir3061	4	1	2	7	9	11
Nos1	20	8	20	12	0	4
Srgn	1143	921	871	1312	1078	2176
Gimap4	1403	903	950	1507	1090	2192
Fstl5	3	3	0	0	0	0
Hoxd10	3	3	0	0	0	0
Ift140	879	558	368	375	425	464
Sparcl1	17415	12041	9646	19949	12948	24600
Spry4	592	471	401	854	459	943
Wsb1	897	662	573	933	737	1527
Xkr4	0	0	1	5	2	4
H2-L	4460	2273	2128	4669	2528	5977
Tbx3	836	475	456	861	576	1091
Fam196a	18	14	49	12	1	19
Psmb8	532	277	225	504	322	788
Adamts9	1079	673	649	1001	824	1894
Arhgef15	2952	1805	1337	2655	1993	3854
Pck2	554	331	218	566	359	736
Slc22a13b-ps	21	12	10	4	3	12
Steap4	1861	1276	1266	2329	1408	2678
Cyp2d22	1087	716	640	992	910	1744
Gm14420	3760	2656	2740	2462	1900	3686
Rhob	6991	4027	3218	7150	4384	8598

Car8	1590	1075	1156	1513	1761	2816
Ddah2	473	288	249	514	325	663
Mir7080	3	0	3	0	0	0
Usp43	147	93	89	182	108	280
Hspa1a	115	63	138	89	41	45
Impdh1	873	529	406	828	610	1112
Colec11	517	324	261	595	320	779
Prex2	3231	1761	1684	2978	2074	4417
Abi3	306	204	114	313	240	416
Rapgef3	1094	617	601	1005	774	1587
She	1577	847	748	1489	986	1955
Smad1	757	535	448	800	582	1142
Tuba8	6182	4765	3893	5017	2919	4866
Xlr3a	5	2	0	6	6	17
Eltd1	5945	3426	3389	5873	4147	8876
Ier3	1237	768	465	1313	904	1333
Ypel3	1643	964	749	1439	1132	2118
Chordc1	2572	1639	1967	1985	1267	2017
Smagp	311	175	136	305	220	419
St3gal5	2614	1615	1703	2047	1110	2014
Tnfaip2	1811	1063	957	1344	643	1302
Cish	3175	2557	2583	2235	2059	2039
Ppp1r18	642	424	342	721	401	1052
Rbl1	270	164	118	258	192	427
Slc25a37	1533	1069	808	972	717	1337
Igfbp3	1254	1256	745	2003	1263	1750
Mir365-1	2	2	1	0	0	0
Col4a1	16863	10464	10011	21789	10761	25222
Cysltr2	1	1	0	7	2	5
Cyyr1	1228	808	608	1230	855	1603
Kcng4	1	0	1	7	1	7
Mir6900	6	4	2	0	2	0
Pcbp3	90	60	43	100	79	156
Vtn	726	415	354	813	425	997
Lnx2	744	526	425	839	586	974
BC068281	387	250	219	252	179	253
Mir682	0	0	0	1	2	3
Prss38	2	1	2	0	0	0
Sulf2	2868	2505	1694	4440	2583	3671
Papss2	854	578	579	1074	621	1262
Kcnj14	7	5	3	12	18	14
Mustn1	269	176	153	333	211	362
Ttyh3	504	288	209	513	305	707
Fmo4	22	15	14	18	38	74
Kcng1	3	1	1	0	0	0

Sparc	13246	8414	8956	15818	8896	27350
Fbxl7	249	156	130	281	181	351
Ppp1r3g	13	13	14	1	3	12
Irx3	1007	857	565	1201	888	1420
Fancb	106	84	64	122	102	225
2610206C17Rik	0	0	0	2	0	5
Rprd1a	1589	1198	1006	1309	791	1180
1700113H08Rik	3	6	6	13	18	16
Arhgap25	146	132	101	168	155	364
Kbtbd13	180	100	94	99	71	53
Lox	190	142	185	330	147	612
Prepl	6340	4482	3250	4127	3225	4043
Rbbp7	6361	4355	3806	6875	5754	7917
Sema3f	596	310	273	552	385	806
Snx16	559	362	368	383	256	458
Ebf1	1017	878	862	1121	1174	2291
Dcbld1	295	170	201	350	227	485
Ankrd33b	2493	1671	1584	1813	1319	1745
Mir697	1	2	2	0	0	0
2810408A11Rik	10	14	4	14	25	37
A630033H20Rik	76	27	34	103	54	96
Fam167b	5	4	5	10	10	22
H2-K1	8024	3617	3180	7915	4213	10027
Nrn1	513	488	335	665	576	801
Rasgrf2	700	462	361	577	640	1121
Fbln2	4395	2717	2378	4970	2765	5843
Ahr	826	498	315	914	560	894
1600014C23Rik	1	1	2	2	10	11
Kcnj8	1636	1036	822	1781	1041	2011
Ptrf	10366	6685	5507	10920	6952	14428
Svopl	21	9	6	33	12	40
AW112010	597	381	399	681	431	992
Rnf125	178	127	125	203	184	307
Tspo	429	218	197	454	262	549
Syne2	3155	2183	1781	3149	2715	4051
Hook1	1085	816	635	871	520	774
Piezo1	1530	922	687	1349	1120	1854
Lrrc29	7	10	4	15	16	24
Tmem200c	2	7	6	1	1	1
Kif6	83	58	37	110	88	108
Kctd16	17	2	2	21	19	17
Abca5	865	631	503	608	436	693
Rassf7	9	4	5	10	14	26
Fam171a1	1463	939	730	1473	938	1938
Usp40	1727	1213	1022	1226	852	1553

Ccdc88c	570	377	231	514	509	724
Zfp568	1297	744	787	924	606	869
Hspe1	1577	977	1208	1067	726	1522
Irf7	560	312	244	547	304	1029
Jade2	1059	787	685	1191	855	1541
Bag2	1742	1292	1249	1368	911	1631
Fgl2	5026	3593	3026	4143	1963	4138
Cdh23	692	401	226	619	516	816
Ano6	2693	1688	1278	2685	1696	3460
Gm15328	66	86	78	65	36	46
Serpine1	1385	591	543	1358	809	1439
Tnfrsf12a	853	671	847	785	460	686
Arfgef1	8831	5954	5545	6354	4599	6693
Kalrn	1074	747	695	1113	1136	1414
Lrrc8c	953	569	446	1009	570	1208
Ttc22	0	0	0	4	1	1
Shroom4	1126	696	483	1149	751	1320
Cd300lf	2	0	1	8	3	5
Cbs	40	8	25	12	11	3
Gm10863	3	0	2	0	0	0
Prkd1	417	295	214	491	283	605
Thap2	777	533	430	583	354	508
Hif3a	86	69	63	100	88	230
Plin2	1645	973	956	1434	1323	2422
Plekhg1	814	596	570	859	702	1373
Shisa3	123	111	79	64	68	88
Atp1b2	1332	1001	678	1495	960	1741
C4bp-ps1	121	79	88	33	59	90
Stmn1-rs1	0	0	0	1	4	1
Tnfsf10	1717	1199	625	1618	1290	2196
Cav2	5132	2773	2667	4543	3373	7593
8430408G22Rik	1516	500	419	1277	763	1802
H2-D1	7567	3851	3464	7683	4096	10083
Sigirr	212	97	110	232	132	317
Pogk	1433	998	789	1043	667	1204
Cacna1s	780	524	343	520	283	580
Postn	2097	1794	1383	2676	1395	5180
Zfp52	628	578	444	789	535	1279
Ccl28	27	15	19	7	6	20
Slc26a2	318	208	179	228	129	217
Hist1h1b	9	9	7	4	1	5
Snord42b	4	8	2	1	0	2
Ano1	415	296	278	417	416	677
Rhoh	22	12	11	4	5	13
Ptbp3	3078	1887	1619	2965	1969	4099

Tmem33	2426	1709	1719	1983	1218	2065
Zfp688	56	29	31	59	52	99
Gpr27	65	33	22	25	20	27
Tmem79	2	3	2	9	5	10
Asz1	0	0	0	3	3	0
Heph	144	79	90	159	112	248
Ccdc150	10	5	5	7	14	41
Serinc3	24547	16356	13768	26383	18459	33803
Litaf	594	346	325	649	361	884
Lims2	3389	1940	1497	3170	1894	4711
A930011G23Rik	0	0	0	0	2	4
Ephb1	244	201	121	260	201	446
Itga6	3220	1964	2381	3420	2577	5655
Kdr	10666	5624	5533	10484	6990	13082
Lect2	0	0	0	2	1	2
Mir126	20	10	5	29	18	28
Prcp	538	267	288	470	381	805
Slc10a7	306	215	151	341	220	447
Srrm4	1088	609	561	663	522	695
Whrn	1768	1029	690	1137	675	996
Fat4	1462	983	821	1487	1139	1797
Gm16386	117	87	75	133	102	244
Iqsec3	50	27	25	49	59	79
Mcam	1612	953	875	1724	941	2249
Tgtp1	133	103	66	145	118	228
Tmem255b	115	83	49	161	81	173
Tpt1	241	194	172	186	102	214
Art4	671	536	357	718	809	821
Pafah1b3	32	26	8	42	29	62
Rab27a	214	144	120	258	125	477
Wasf2	2407	1754	1113	2406	1738	3196
Bsn	5	9	2	1	1	2
1700030C10Rik	6	21	8	22	44	34
Mir1195	2	0	2	3	7	9
Ly6e	5943	3684	2693	5522	3670	9055
Bpgm	1452	1393	950	1081	816	1484
Snord34	1	1	1	5	2	8
Star	62	32	21	54	52	106
Adamts18	0	0	0	1	2	2
Mapk3	2075	1408	1010	2013	1383	2870
9230112J17Rik	608	379	470	417	310	504
Gpld1	226	156	111	150	80	159
Calr	12716	9135	8405	9950	6146	11108
Gimap6	1645	1096	1367	1788	1569	3111
Map4k3	2501	1667	1314	2231	1951	3396

Gm14305	77	66	53	46	36	59
Klra10	3	0	3	11	5	7
N4bp3	419	232	133	424	264	492
Gm2897	5	4	3	7	10	19
Nr5a2	32	26	34	62	40	79
Adipoq	37	53	22	31	13	20
Hist2h2ab	0	0	0	2	2	1
Jdp2	269	169	150	257	197	484
Mir92-1	0	0	0	2	2	1
Emcn	1585	1374	1413	1811	1897	3312
Tmem44	545	342	277	455	423	890
Tnc	80	48	31	64	38	364
Dclre1b	352	248	235	365	298	622
Net1	675	603	403	813	596	1028
Dock9	4804	2906	2378	4046	3644	6448
Ece1	4136	2910	2445	4279	3093	5864
Npr1	1425	849	637	1220	937	1903
Sez6	11	9	5	9	16	46
Tcf4	9222	5758	5370	8429	6950	14092
Cdc14b	2224	978	843	1125	796	1379
Tgtp2	266	154	131	230	215	389
G6b	20	24	16	12	10	11
Mfsd6l	7	3	3	9	8	20
Gstt4	1	0	4	0	0	0
Nudt4	18056	11011	10025	20358	14092	20577
Dusp1	1943	1123	999	1638	1331	2854
Mdn1	1094	824	652	734	568	1032
AI414108	359	238	211	278	150	214
4921511C10Rik	0	0	0	3	1	1
Gckr	0	0	0	3	1	1
Gzmk	5	4	0	1	0	0
Ly6g6c	0	0	0	3	1	1
Mir6975	0	0	0	3	1	1
Cdc20	41	22	21	52	26	90
Cited2	2395	1604	1066	2293	1744	2844
Mrgprx2	3	1	4	0	0	1
Mpzl3	25	23	11	12	4	17
Eml3	481	281	239	428	343	695
Celsr1	468	306	226	304	217	254
Myo6	1539	975	985	1366	1304	2483
Ecscr	386	310	205	397	311	661
Sema3c	944	652	529	703	452	741
Tle3	640	398	326	715	422	783
Gbp7	2621	1529	1078	2215	1748	3209
Ggta1	1579	987	832	1549	1076	1928

Spdef	1	0	0	2	2	5
Ccdc19	9	4	3	7	10	31
Hspa4l	3305	2332	2529	2715	1848	2671
D430041D05Rik	10	12	3	5	1	3
Fhit	22	24	26	52	30	67
Igfbp7	4550	3577	2697	5425	3057	7811
Mboat2	759	495	474	374	247	820
Slc16a6	371	286	365	247	220	362
Ajuba	374	198	180	379	232	498
Col11a2	176	97	95	84	81	105
Clec2e	14	9	11	17	16	50
Cxcl5	47	32	14	22	15	14
D8Ertd82e	886	509	399	870	521	1128
Ucp1	0	0	0	5	0	1
Mertk	516	286	314	466	417	769
Fam13c	613	463	400	572	634	1013
3010001F23Rik	0	1	0	4	1	4
Ccdc125	52	40	28	49	73	100
Cml3	4	3	5	2	0	1
Fxyd3	0	0	0	3	0	2
Oxgr1	0	0	1	4	1	4
Pfkfb1	697	448	325	546	527	1191
Sp9	0	6	0	0	0	0
Hspd1	12370	8702	8513	8731	6860	10566
Pabpc1l	8	2	4	10	11	16
Apobec1	228	190	133	256	183	437
Cngb1	61	24	26	54	50	95
Dnajc27	958	584	538	715	434	613
Efnb1	1356	794	669	1274	921	1587
Fgl1	21	14	15	7	6	15
Gas2	128	129	124	195	140	348
Gnb4	601	368	305	668	346	871
Lonrf3	412	259	235	279	170	330
Nceh1	16654	11114	10849	12575	8204	13505
Plekhh2	507	397	245	500	394	812
Rasip1	1567	875	736	1413	935	2032
Serp2	1	4	1	6	7	9
Tes	129	78	86	134	96	314
Tsix	90	153	57	26	31	121
Fcgrt	2516	1228	1067	2159	1706	2621
Pcdh15	3	2	3	6	10	10
A830018L16Rik	7	1	1	1	0	0
Plekhg5	1064	623	437	1100	588	1315
Trim10	24	23	12	14	7	13
Cort	0	0	0	0	2	3

Msh4	0	0	0	0	2	3
Tesc	2587	1670	1475	2482	1977	3184
Ahsa1	3968	2655	3011	3106	1992	3661
Hcls1	245	155	153	272	162	468
Myo7a	540	322	264	462	377	826
P2ry14	243	164	101	278	145	374
Cox4i2	233	181	133	240	188	423
Klf6	4729	3393	2826	4689	3688	7035
Egfbp2	6	5	7	13	9	25
Klk1b26	6	5	7	13	9	25
4931440J10Rik	6	2	2	0	0	2
Stip1	4957	3891	3653	4260	2687	4263
Gm13889	118	69	71	135	84	208
Aldh16a1	200	104	90	183	285	185
Tpm3	717	757	614	1016	697	1691
Fas	843	571	592	821	687	1509
Hmgcs2	435	282	374	386	604	893
Scn3a	212	139	98	247	147	279
Slc6a17	451	293	187	333	139	227
Trmt61a	106	70	67	66	45	73
Ppbp	252	165	161	76	154	128
3110057O12Rik	3165	1919	1473	1898	1567	2162
Aim1	725	501	337	690	460	1203
Arrdc4	636	346	437	594	475	1217
BC033916	0	2	2	0	0	0
C030046E11Rik	2077	1324	1045	1996	1466	2466
D330050I16Rik	27	18	14	34	36	41
Dach1	505	294	633	539	687	1988
Dapp1	508	399	327	450	209	387
Eepd1	774	505	437	782	592	968
Gm10432	3	2	1	4	5	13
Hrsp12	383	296	245	269	174	365
Ifngr1	2506	1781	1632	2756	1724	3885
Il17rd	651	428	341	755	419	830
Lyn	1071	647	552	1004	667	1495
Mrc1	1512	1006	805	1020	601	1433
Mroh6	20	9	5	6	4	5
Necap2	501	282	278	460	340	784
Peo1	1885	1185	1109	1392	950	1437
Pik3ip1	2778	1586	1357	2221	1977	3592
Plcg1	2943	2001	1623	2811	2144	3829
Plxnd1	4887	2863	2505	4657	3282	5915
Rrn3	3171	2230	1952	2553	1601	2693
Serinc2	4	6	5	19	5	19
Slk	15121	9258	8014	9804	6932	12275

Tcf19	126	60	53	115	84	195
Tpm2	837	591	661	1092	781	1156
Upk2	3	2	2	1	0	0
Vegfa	16481	10837	9522	15632	18372	19823
Xpo1	3810	2528	2388	2874	1891	3317
Zfp493	90	61	47	47	34	66
Aifm3	0	1	0	1	6	3
Myl9	1006	678	821	1383	857	1466
Slc6a1	3	2	2	0	1	0
Fastkd2	1801	1161	1071	1274	854	1656
I830012O16Rik	223	110	77	208	123	324
Apba3	2076	1128	1029	1449	861	1467
Efr3b	860	536	558	970	597	1173
Ptges3	1720	1085	1181	1241	921	1452
Stk10	584	411	262	659	389	740
Tyw3	276	169	159	187	125	178
Igfbpl1	65	69	22	42	11	42
Pfkfb3	542	417	330	497	510	919
Arr3	1	3	0	0	0	0
Ctdsp2	5597	3060	2651	5321	3488	6520
Cyp4b1	1314	644	635	1237	696	1859
F730043M19Rik	7	5	7	12	11	24
Gm15133	1	1	1	4	2	8
Gm4907	1	3	0	0	0	0
Grrp1	192	86	78	203	119	225
Ipw	1	3	0	0	0	0
Prss16	1	3	0	0	0	0
Tmem229b	204	123	117	220	155	287
Usp29	1	3	0	0	0	0
Wnt5b	82	45	42	82	82	113
Gja5	278	211	176	358	249	369
Rell1	652	521	475	710	548	1238
1700030M09Rik	0	0	0	1	0	4
9430020K01Rik	8131	5214	3792	8470	5332	9434
Mylk	2030	1319	1236	2361	1384	2542
Otc	2	0	2	0	0	0
Slc22a7	2	0	2	0	0	0
Tnfsf12Tnfsf13	611	406	327	564	473	852
Prickle2	776	467	367	688	553	970
Ptp4a2	13350	8850	8120	14741	10133	17223
Rbp7	342	175	187	300	235	550
Abcc12	4	2	3	1	0	1
Abra	1711	1333	1603	1491	1018	1572
Atp2a3	717	457	354	700	477	933
Ccdc169	5	4	2	1	0	2

Col4a2	11514	7341	6920	14107	7271	15780
Emilin1	814	505	370	856	450	1127
Gucy2d	4	2	1	1	0	0
Gyk	2774	1769	1711	1883	1421	2487
Jmjd6	1322	878	960	1068	665	1148
Klhdc8a	621	366	434	498	211	507
Mrps36	169	141	125	102	92	157
Ncbp2	1475	990	1007	1190	688	1354
Rbm19	638	343	329	402	254	483
Rfc5	76	71	58	108	78	163
Rpl17	373	250	260	251	164	355
Slpi	1	0	1	4	6	2
Trim58	2	1	1	0	0	0
Klrc2	2	2	0	0	0	0
P2ry4	2	2	0	0	0	0
Plcxd1	2	2	0	0	0	0
Ublcp1	1047	771	667	852	541	880
Zfhx2	824	703	395	642	397	480
E130215H24Rik	13	9	5	18	13	27
Flt4	1146	739	630	1148	782	1463
Mapkapk3	1214	797	613	900	571	828
Prrg3	887	480	409	916	470	1157
LOC102633035	144	76	63	75	41	98
Notch3	2160	1347	984	2214	1359	2451
Ntf3	144	101	103	151	142	269
Wif1	120	102	74	164	209	133
Kcnn3	151	79	44	63	49	73
Slc6a8	9966	6476	5884	7923	4608	7448
Reep3	3218	2274	1749	3360	2254	4136
Col8a1	1617	1215	949	2063	955	2669
Ctnnbip1	1143	667	640	1076	765	1507
Dusp13	10	4	1	2	0	2
Mospd2	1123	688	624	1005	796	1551
Lrrd1	10	5	5	1	2	5
St8sia5	7	6	13	4	1	6
Limd2	96	65	50	111	74	152
Elmo1	583	393	287	624	354	852
Gm10390	3	1	0	0	0	0
Lonrf2	441	297	256	334	203	310
Olfr183	3	1	0	0	0	0
Cables2	815	511	410	783	563	998
Dusp18	4886	3228	2361	3691	2171	3222
Hspb1	1528	813	1303	1169	527	1424
Nid2	1415	797	724	1317	822	1979
Arrdc1	126	95	57	134	107	194

Casp1	165	81	68	158	101	230
Clcn1	576	305	222	243	216	427
Dcdc2b	96	67	60	59	35	79
Endou	107	66	63	124	78	179
Haghl	266	169	166	260	212	431
Kcnmb1	98	61	51	101	77	160
Map3k7cl	82	37	37	86	56	115
Olr1	1	1	1	5	2	6
Plxna3	69	48	37	40	28	43
Popdc3	968	715	708	762	540	892
Serpinb6c	4	1	2	0	1	0
Sncg	200	136	141	229	170	325
Spata33	204	151	132	140	88	182
Tek	4340	2939	2509	4228	3182	5950
Trim30a	913	551	464	776	598	1377
Timm9	776	459	543	523	355	716
Adam10	2996	1976	1690	2909	2071	3867
Ano2	4	0	0	0	0	0
Cd101	16	13	11	10	4	8
Efcab4a	468	232	186	435	278	544
Gdf10	108	69	88	175	96	167
Hist1h2bj	4	0	0	0	0	0
Irs1	2266	1584	1625	1499	1337	2083
Manf	964	701	695	786	448	965
Mir1896	4	0	0	0	0	0
Ndc1	463	319	281	359	228	299
Nr2f2	1161	722	566	1218	760	1302
Pls1	18	8	8	5	2	10
Slc28a2	1749	957	888	1319	1249	2515
Baiap3	19	7	6	30	10	29
Fbxw9	87	58	42	89	70	147
Prnp	6608	4232	4489	7206	5981	8155
Rasd1	128	85	69	117	115	218
Capg	310	146	146	294	166	488
Rimklb	1	0	1	2	3	6
Sptbn2	39	20	15	38	39	54
Heatr3	1610	1198	978	1278	871	1333
Wipf3	3977	3081	2221	3079	2055	3343
Hsp90b1	19899	14500	15235	15007	11297	17638
Leprot	1168	723	722	1244	752	1664
Arhgap31	5005	3429	2482	5522	3401	5875
Dll1	228	126	119	216	156	337
Mfap2	35	25	18	49	32	56
Mir223	2	4	1	0	0	1
Haao	1	0	1	6	0	7

Fktn	1430	1144	931	1248	783	1019
P4ha1	3400	2169	2876	3083	1624	2713
Qsox1	1748	975	868	1587	1083	2102
H2-Q7	500	171	147	383	278	569
H2-Q9	500	171	147	383	278	569
Pvr	1361	1049	967	991	855	1142
Slc2a12	737	378	394	481	317	502
Clec2d	885	577	599	875	747	1247
Fgd5	2834	1700	1199	2924	1760	2984
Kcnc1	100	21	48	18	38	20
Kcne4	96	57	80	122	93	175
Klhl35	0	0	1	1	5	3
Mir700	23	22	18	19	9	8
Pear1	1045	545	460	925	619	1227
Prnd	97	77	100	149	70	406
Rit1	2515	1582	1340	2059	1904	3541
Sdf2l1	145	70	98	95	41	103
Trim55	2848	2211	1754	2472	1494	2223
Cntn2	417	414	247	578	397	620
Tm4sf1	2116	1346	1210	1994	1432	2952
Prrg4	50	39	25	54	47	98
Kitl	8481	5138	4840	7768	6195	11278
Rgl3	490	289	253	326	202	374
Trmt1l	2064	1631	1293	1734	1063	1850
C330018D20Rik	1218	854	846	898	676	1119
Gimap8	692	460	560	629	707	1387
Apaf1	564	302	244	524	337	697
Abcb11	0	0	0	0	4	0
Helz2	1967	1122	972	1727	1241	2511
Irf1	1069	637	501	1007	623	1400
Clca2	0	1	0	4	2	2
Ccdc112	39	30	21	56	38	61
Fastkd1	1653	944	920	1152	803	1206
Pparg	600	386	385	622	398	1035
Ccdc93	1461	1056	859	1194	745	1108
5430417L22Rik	1679	1232	1043	2256	1166	2127
Atp6v0a2	3907	2395	2308	3055	1772	3126
Ddit4l	50	27	39	58	67	79
Ephb4	3259	1885	1355	2952	1918	3757
H2-Bl	4	0	4	0	1	0
Hspa5	25257	15745	17298	19150	12296	20247
Ip6k1	3296	1851	1505	3007	1947	3818
Nat10	1035	750	561	740	523	902
Zfp457	108	22	39	14	20	57
2610307P16Rik	14	15	8	15	19	51

Bsg	45794	30480	26872	45583	36016	65929
Gabrq	7	11	5	4	2	4
Galnt18	364	224	194	370	244	502
Idua	1321	822	639	933	565	1052
Mest	355	197	130	349	162	643
Plk2	1746	1159	873	1711	1233	2053
Tmeff1	378	217	206	198	183	289
Lipa	1890	1300	1132	1852	1294	2893
Mir6953	1	0	0	2	4	2
Agfg2	539	326	292	557	370	668
Flrt2	562	375	267	530	423	700
Mecom	1126	648	476	1100	727	1165
Mettl11b	14	7	12	2	5	9
Stk39	7997	5839	4362	6241	3615	7041
Tacstd2	0	0	0	1	3	0
Ulbp1	46	21	19	45	35	70
Aldh1a2	200	131	171	99	51	239
Tbc1d10a	272	167	131	264	187	364
Anxa3	1087	769	622	1056	716	1829
Tmem178	23	16	10	28	23	40
Dennd2d	53	42	24	21	19	42
Adam17	2800	1743	1656	2145	1242	2445
Hs3st5	111	56	76	98	98	228
Unc5b	1152	678	461	1215	752	1124
Mir7009	1	2	4	1	0	0
Tnfrsf1a	1567	1038	843	1588	1032	1947
Mthfd2	196	72	164	100	86	119
Tnfaip8l1	67	36	34	65	53	111
Aqp4	218	226	127	332	387	231
App	11380	7269	6134	11711	7272	15099
Slc39a2	47	28	25	20	12	37
Cntln	419	265	247	387	311	653
Dbndd2	645	417	353	675	419	864
Fuca1	907	542	486	872	567	1195
Ifi47	200	99	82	205	110	270
Gm12657	0	0	0	2	2	0
Tmem74b	53	32	30	67	45	78
Akap1	6258	3998	4179	4894	3188	4959
Stmn2	382	272	197	378	262	604
Pde3b	544	298	286	370	239	347
Ccdc103	10	2	1	11	8	14
Nps	1	1	0	2	2	7
Cdh26	1	0	1	4	2	4
Lmo7	24695	18735	13442	25948	23939	29968
Gm16532	0	0	0	1	2	1

Ndufaf4	4582	3037	2932	3588	2404	3684
Clcf1	19	14	7	23	15	43
Micall2	107	53	55	94	80	176
Rftn1	1718	1060	776	1588	1118	1948
Exoc4	1955	1486	1240	1654	1044	1584
Clec14a	2130	1190	781	1963	1216	2385
Fam76b	995	639	522	846	734	1359
Pecam1	9457	6298	5463	9038	6549	14044
St6galnac1	0	0	0	3	1	0
Tuft1	59	55	42	24	19	68
Ints10	1355	879	702	973	654	1065
A230056P14Rik	35	31	26	26	11	27
Plekhg2	1125	656	501	1114	623	1381
Gucy1a3	1694	1159	822	1846	1100	1938
Crabp1	0	0	0	0	2	2
Sptbn4	72	49	18	32	23	33
Cnn1	82	50	88	223	77	130
Rgs3	1660	1094	755	1626	1051	1974
5430416O09Rik	506	273	219	332	382	827
Aldoa	95334	56331	49124	99412	66580	117768
Dbil5	10	8	6	1	3	7
Dok1	81	40	27	101	39	117
Mybph	0	0	0	2	1	1
Zcchc13	0	0	0	2	1	1
C1rl	35	30	24	46	44	65
Serac1	1197	804	622	824	589	1017
4930455C13Rik	4	3	0	1	0	0
Agpat9	1030	587	570	689	471	845
Arap3	3398	1884	1563	2991	2000	4031
D330041H03Rik	248	131	122	37	138	74
Dnajb9	3206	2300	1790	2422	1599	2948
Gcnt2	614	370	402	586	483	896
Grasp	296	191	183	297	226	455
Kif4	49	25	24	40	39	102
Klf17	0	0	0	1	1	2
Klhl5	994	696	708	990	854	1542
Mirlet7c-1	1	0	0	0	2	8
Nod2	99	68	54	98	102	146
Oit3	0	0	0	1	1	2
Pde8b	134	103	82	151	120	212
Slc46a3	924	462	402	781	633	971
Sp100	1861	1355	857	1621	1338	2743
Stmn4	11	4	10	6	2	3
Syt2	0	0	0	1	1	2
Ttc27	556	351	259	375	239	404

Tubd1	495	298	234	298	215	382
Ept1	1521	1001	906	1233	739	1172
Fam219a	513	283	240	449	327	678
Tbck	465	316	237	337	219	303
Dazl	6	7	5	8	16	20
Cd40lg	0	0	0	3	0	1
Epas1	21999	13557	12791	26575	14754	26458
Mir486	3	0	2	6	6	5
Retn	10	17	5	10	1	1
Tiam2	247	163	167	223	86	147
Wisp2	87	65	170	134	220	382
Zfp820	149	97	79	146	117	230
Gabra2	0	0	0	0	1	3
Mir101a	0	0	0	0	1	3
Mir7020	0	0	0	0	1	3
Snora20	0	0	0	0	1	3
Tnfrsf14	86	33	37	96	52	107
Sirpb1a	1	1	0	3	0	11
Bcl2l15	10	6	4	3	1	5
2610318N02Rik	0	0	0	2	0	2
Slc43a3	5130	3719	3170	6032	3369	7590
6430562O15Rik	54	30	29	53	47	89
Fv1	556	374	327	467	245	337
Spib	6	4	5	2	1	3
G630055G22Rik	2	1	0	1	5	8
Nxn	479	248	205	443	275	591
Aasdh	1319	854	630	699	696	1040
Gpr157	3636	2243	1840	2768	1699	2431
Pcdhb10	17	4	6	1	4	6
Gdpd1	923	735	621	838	480	752
4933408J17Rik	0	0	0	1	0	3
Aldh3b1	52	28	19	34	44	110
Ccdc108	0	0	0	1	0	3
Cldn19	0	0	0	1	0	3
Crlf1	11	12	17	22	18	51
Sh2b3	904	522	441	843	583	1045
Sox15	0	0	0	1	0	3
Trabd	1421	832	741	1258	1173	1531
Fryl	3138	2269	1899	2989	2772	4114
Gm2694	1	1	1	5	2	5
Il20rb	84	44	38	81	55	136
Wnk1	38018	30362	24226	31150	20472	29044
Zim1	83	39	55	47	18	65
Amdhd2	172	115	84	177	123	252
Mapk8ip3	4545	2998	2505	3370	2353	3385

1700029I15Rik	6	1	3	11	4	12
1700037C18Rik	34	32	19	51	32	66
A630076J17Rik	0	0	2	2	1	12
B3gat2	0	0	0	0	0	4
BC027231	320	245	189	230	151	288
Creb3l4	8	6	5	17	9	16
Ky	1093	746	646	876	565	797
Lmod1	198	164	174	404	161	320
Obp2a	0	0	0	0	0	4
Rcan1	2741	1894	2374	2311	1470	2738
Sh3tc1	561	313	210	502	357	633
Tmtc2	299	245	338	343	396	834
Wscd1	317	214	128	349	244	343
Pvrl4	14	13	6	9	3	5
H2-Q5	153	57	35	117	94	180
Mir3092	0	0	0	0	0	5
Rgl1	1850	1371	1024	1920	1395	2311
Slc38a3	4253	2493	1772	3089	1535	2924
Usp50	0	0	0	0	0	5
Agrn	3479	1945	1570	2700	2178	4550
Zfp933	1022	683	594	788	478	900
Pde1b	75	49	45	81	53	160
Abca9	1969	1260	1055	1593	774	1620
Aga	857	584	521	663	410	753
C030013G03Rik	4	2	4	10	10	7
Csrp1	1612	1070	942	1664	1041	2141
Gbp5	1141	854	518	1102	752	1649
Ptprf	383	231	158	362	256	457
Reep1	1651	910	794	1137	720	1169
Tnni2	27	11	11	27	43	24
Aldh1a3	54	41	30	65	45	97
Gnl3	954	718	679	741	521	960
Hyou1	2065	1239	1215	1666	932	1494
Myo3b	1	0	2	4	3	5
Iqub	11	4	4	2	1	5
Nfe2	15	9	7	5	6	5
Procr	33	32	26	47	34	86
B2m	14977	8517	7391	16083	8299	18740
Bbc3	56	22	23	62	32	79
Gm20748	102	70	52	98	80	181
Mtif2	3207	2107	2119	2397	1709	2986
P2ry1	2812	1593	1348	2105	1166	1776
Rgs6	1132	931	631	1027	486	915
Samd12	21	18	10	27	23	40
Syt12	188	92	117	109	57	157

Tap2	1430	747	584	1293	843	1505
Cers6	190	122	108	135	73	146
Tcf15	358	256	272	386	350	552
Bcl11b	167	130	112	130	89	105
Plscr2	1406	1013	1016	1186	1304	2827
Slc5a11	3	1	2	0	0	1
Mir1981	1	0	0	2	2	3
Rbm46	40	4	2	5	2	8
Mir6993	1	0	0	2	2	3
Stx2	927	541	400	880	539	1086
Ltv1	863	730	603	665	481	920
Adam2	0	0	3	0	0	0
Acan	14	6	12	38	11	22
Kirrel3	20	19	8	36	23	29
Kremen2	3	4	1	8	5	9
Nnt	40618	26025	20070	18697	23853	19605
Patl1	2300	1531	1415	1804	1219	1835
Acer2	1895	936	931	1446	1304	2349
Fam110c	10	4	5	4	3	0
Mctp2	11	5	4	13	10	20
BC027072	5	2	3	0	1	2
Eml2	1090	695	481	1041	694	1261
Gm13286	0	1	2	0	0	0
Myod1	3	5	1	2	0	0
Pros1	1557	966	855	1512	963	2033
Tcirg1	575	360	230	525	360	736
Abca7	1179	623	470	1096	707	1193
Gm10639	1	0	0	3	0	5
Gm8909	3	2	1	0	1	0
Mettl3	639	458	407	469	330	590
Chdh	6	7	5	2	2	4
Myh2	2	0	0	4	1	5
Plxnb1	4545	3005	2268	3663	1542	3666
Rps4l	419	464	331	582	402	905
Slc25a34	6026	3444	3131	3951	2582	5205
Ankrd13a	2225	1442	1223	2048	1533	2886
Rps6ka2	1602	1101	927	1178	831	1440
Trim34a	330	230	176	316	229	535
2810405F15Rik	0	2	1	0	0	0
Oaz3	0	2	1	0	0	0
Ppid	1929	1543	1388	1615	1086	1917
Dzip3	487	344	341	391	262	390
1300002K09Rik	1	0	2	0	0	0
1700019B03Rik	1	2	0	0	0	0
1700092K14Rik	1	1	0	3	5	2

1810014B01Rik	462	262	296	318	211	378
4931440L10Rik	0	3	0	0	0	0
4933431G14Rik	1	1	1	0	0	0
5730480H06Rik	69	49	48	77	57	151
9530027J09Rik	1	1	1	0	0	0
Amhr2	4	3	5	12	9	9
Ankrd37	210	133	69	127	48	140
Anxa4	793	461	406	639	764	877
Ascl1	1	2	0	0	0	0
B4galt1	5157	3589	3434	4153	2649	4741
Bcl2a1a	1	0	0	1	1	6
Cacnb4	146	94	54	123	107	226
Ccng2	1192	550	532	969	660	1496
Ccr6	1	1	1	0	0	0
Cd1d2	0	3	0	0	0	0
Cd209d	1	2	0	0	0	0
Cd24a	201	209	153	172	119	186
Chil3	3	2	2	4	15	4
Col18a1	591	335	285	635	317	756
Ctdspl	475	294	207	478	302	553
Cttnbp2nl	1430	900	854	1306	953	2130
D630033O11Rik	2	4	7	3	0	1
Dcun1d2	4002	2571	2307	3125	1934	3247
Ddah1	343	225	205	432	250	410
Defb1	2	3	3	1	0	1
Dnaja4	7760	4850	5212	6057	3808	6886
Entpd3	1	2	0	0	0	0
Erich3	8	5	5	1	2	5
Fam69c	1	2	0	0	0	0
Fam98a	2414	1767	1496	1930	1294	2167
G0s2	1094	561	476	1274	618	1035
Gm14634	20	8	9	17	21	33
Hagh	1816	1207	1051	2004	1071	2602
Ifi44	276	175	163	381	128	526
Il22ra2	0	3	0	3	4	6
Iqgap2	365	298	186	276	135	329
Jup	9139	5802	4190	8961	5633	10911
Kcnj16	1	0	2	0	0	0
Kirrel2	1	1	1	0	0	0
L2hgdh	2677	1850	1561	2084	1433	2113
Lad1	99	51	45	86	128	98
Laptm4b	3027	1878	1796	2390	1234	2740
Lurap1l	123	59	57	99	93	181
Masp2	8	10	8	7	1	5
Mdp1	2202	1466	1216	2365	1341	2868

Mfng	234	135	99	230	145	298
Mir3102	1	2	0	0	0	0
Mir5133	1	2	0	0	0	0
Mira	5	5	1	5	8	17
Mtmr10	1311	999	922	1267	1166	2089
Nek10	1	2	0	0	0	0
Nfkbie	47	27	38	66	33	106
Nol8	1230	925	899	805	783	1161
Notum	15	2	2	1	3	1
Pdcd1	4	6	2	2	0	2
Pik3c2a	1734	1020	797	1479	992	2399
Ppap2b	4273	2891	2655	4386	3081	5743
Ppp1r1c	1	2	0	0	0	0
Pxn	2589	1525	1223	2047	1802	3218
Rapgef5	1408	935	759	1270	1047	1750
Rgs13	1	1	1	0	0	0
Sardh	216	154	104	262	150	272
9-Sep	1854	1200	865	1847	1111	2264
Slc22a23	674	467	452	515	370	554
Slc27a2	1	2	0	0	0	0
Sostdc1	15	14	10	10	4	9
Tmsb4x	8887	5728	6126	9258	6004	15476
Tor3a	709	473	411	720	465	1019
Ttc7	851	519	418	637	348	620
Ubiad1	830	517	484	707	329	579
Vsig2	341	195	162	239	294	500
Wdr76	148	94	89	152	122	218
Tmx1	3111	2057	1826	2902	2791	3617
Myocd	2197	2237	1724	1790	1651	1674
Psmb9	223	96	88	187	125	301
Kcnab1	70	44	38	82	51	108
Lman2l	1147	809	666	944	579	895
Pdia6	3172	2126	2121	2562	1702	2799
Spata5	519	364	382	419	271	457
Susd2	95	66	56	136	64	143
Slc37a1	662	388	288	650	383	796
Tmem173	197	142	112	221	136	309
4933417E11Rik	2	0	1	0	0	0
A530065N20Rik	2	1	0	0	0	0
Adra2b	61	21	28	66	41	74
Ano4	539	405	249	391	238	423
BC028528	176	116	125	175	135	366
BC029722	903	754	579	1145	600	1497
C030023E24Rik	2	1	0	0	0	0
Cacybp	3457	2604	2714	2664	2098	3421

Ccin	2	0	1	0	0	0
D930048N14Rik	176	95	71	167	113	221
Dnaja1	1323	803	1098	1119	511	1318
Ehd2	6818	4069	2601	6443	3926	7975
Fam213a	1015	851	739	888	574	1008
Fig4	3021	1926	1455	2732	2125	3358
Gm14326	312	285	243	260	191	284
Gm3264	2	0	1	0	0	0
Golgb1	6053	4987	3899	4502	3583	5697
Lcn8	2	0	1	0	0	0
Marveld3	2	1	0	0	0	0
Mir4660	2	1	0	0	0	0
Mir7093	2	1	0	0	0	0
Mir7237	2	0	1	0	0	0
Mir7672	2	0	1	0	0	0
Myo10	4235	3031	2881	4096	3614	6336
Pak7	4	1	1	0	0	1
Phlpp1	1489	1043	732	1475	1027	1755
Pof1b	2	1	0	0	0	0
Ppargc1b	1132	836	539	822	592	774
Ppp1r16b	860	466	346	1030	535	748
Prn	141	89	135	184	95	447
Ptpla	1410	1062	916	1221	723	1272
Rnu12	2	0	1	0	0	0
Serinc5	1170	726	552	1078	823	1284
Snora69	2	0	1	0	0	0
Tarm1	2	1	0	0	0	0
Tbr1	2	0	1	0	0	0
Tekt3	2	0	1	0	0	0
Tomt	2	1	0	0	0	0
Usp17le	2	1	0	0	0	0
Utp18	543	398	393	429	267	540
Vash1	1286	761	808	1344	781	1880
Foxn3	1286	992	681	951	734	886
Mirlet7f-1	3	0	1	3	4	7
0610040F04Rik	2	2	0	1	7	8
Extl1	1462	850	751	1100	615	1114
Jak3	996	534	417	776	651	1154
4933432I09Rik	32	23	18	18	10	23
Cldn23	1	0	0	1	2	4
Mpp7	614	445	362	457	326	514
Nxph1	1	0	0	1	2	4
Rrm2	98	56	41	99	54	169
Zfp251	975	711	579	823	488	788
Cdc14a	198	141	115	195	160	307

Ddit3	777	598	435	632	364	680
Pgm5	10797	7659	6711	9536	5035	8559
Csrnp1	446	255	273	408	304	712
Gypc	1519	1146	892	1535	1228	1909
Ager	12	8	4	9	11	36
Asns	270	147	90	83	133	109
Dpy19l3	62	53	44	92	64	97
Epha1	11	10	5	15	12	26
Klhl34	302	220	265	274	160	243
Klrd1	14	8	3	5	1	6
Notch1	2864	1634	1303	2605	1733	3172
Tusc5	139	103	113	176	102	322
Pdgfra	3115	2262	1720	2809	1124	2580
2310008N11Rik	3	0	0	0	0	0
Cbln1	8	5	5	15	6	19
Cyp8b1	3	0	0	0	0	0
Ext1	1179	911	880	1210	1125	1792
Igf2bp1	3	0	0	0	0	0
LOC101055769	3	0	0	0	0	0
Mmp21	3	0	0	0	0	0
Nrip2	74	47	44	82	67	106
Odf3	3	0	0	0	0	0
Prg2	3	0	0	0	0	0
Prtg	14	6	10	18	14	27
Rep15	23	16	17	11	10	16
Serpine3	3	0	0	0	0	0
Sertad1	259	142	130	254	155	354
Slc5a2	3	0	0	0	0	0
Col12a1	103	69	61	129	74	153
Etl4	3337	2422	2090	2856	3028	5038
Cdk15	4	1	3	1	1	0
Fam171b	72	51	36	46	25	52
Nppb	3836	3041	3926	3271	2210	4508
Rasl12	394	255	207	452	279	442
Slc5a1	25	12	16	12	11	9
Smad3	1100	736	595	827	558	874
Ctxn3	23	10	15	27	18	44
Lmcd1	964	572	602	827	856	1228
Snord123	4	4	5	1	2	2
E130311K13Rik	476	344	348	336	265	466
Ddx26b	1436	914	701	1266	924	1827
Ly6a	3819	1933	1949	3572	2013	5177
Smad9	362	207	164	260	142	199
Hhatl	2586	1453	1111	2431	1831	2401
Spryd4	1092	615	667	853	475	871

Tnfaip3	187	120	100	210	140	230
Abracl	682	359	365	573	586	747
Mir3057	193	137	120	142	91	153
Mtmr14	828	593	434	819	584	1054
Oacyl	0	1	3	2	8	7
Vsig8	0	1	0	1	2	4
Dixdc1	563	320	301	468	481	662
Greb1	33	23	27	49	37	55
Hk3	49	34	22	27	16	34
Igdcc4	1094	618	521	802	457	732
Mir6901	1	0	1	1	5	4
Nrp1	12076	7822	8105	11938	9419	17429
Parm1	2714	2699	1832	2865	1444	2087
Trpm6	13	8	5	17	12	22
Acot9	1028	563	559	969	638	1241
Hrasls	1916	1423	1393	1749	943	1812
Prosc	3484	2337	2124	2896	1730	2998
Sphk1	28	11	11	29	19	41
Sptlc2	852	553	498	834	576	1130
Hoxd4	55	20	11	13	16	20
Il23a	17	6	9	3	5	9
Hsd17b1	20	17	20	16	6	16
Ppapdc2	704	486	430	672	565	954
Rdh9	6	4	6	7	11	20
Vwa3a	368	223	268	247	357	887
Zfp84	1182	818	634	967	510	1000
Ccs	571	330	287	508	371	731
Cldn5	437	229	244	360	291	671
Plcd1	1132	703	698	1250	827	1281
Scai	875	571	433	818	559	1117
4931408D14Rik	2	1	0	4	1	7
Abce1	4272	3057	2684	3497	2324	3577
5830418P13Rik	32	6	19	11	9	12
Naa15	5330	3868	3525	4132	3049	4742
Tmem186	1435	930	852	1155	730	1081
Acp1	353	260	258	586	249	478
Emid1	57	33	30	63	35	105
Ppox	587	356	268	536	351	774
Pced1b	112	46	55	115	65	154
Slc35e2	1107	710	493	762	483	877
Dysf	4030	2361	1964	3725	2546	4542
Enpp3	265	190	168	287	209	383
Mov10l1	1590	1223	953	1672	1462	1882
Mreg	1562	898	838	1050	804	1140
Neurl2	799	412	351	787	439	880

D19Bwg1357e	1063	791	633	798	590	962
Lrrc18	17	12	21	23	22	60
Strbp	1170	823	575	782	594	1009
Adig	13	8	4	3	6	2
Heatr1	732	525	408	584	344	613
Nutf2-ps1	65	46	35	34	34	39
Etohi1	1159	989	710	906	727	913
Gm10416	2	2	1	3	4	9
Odc1	1076	768	708	881	601	903
Pacs2	3145	1892	1680	2369	1387	2634
Polr3d	830	627	590	692	460	780
Prkar2b	149	127	86	131	65	88
Rhbdf2	483	294	217	454	329	556
Slc16a10	1695	1076	955	1354	836	1256
Prdm16	941	625	645	904	857	1241
Adamts15	430	242	287	358	189	237
Gm12530	5	5	8	0	4	3
Gm19705	12	6	5	3	5	3
Gm5741	1	0	0	0	2	6
Mir6973b	12	2	3	13	9	15
Tmem8	394	245	187	266	146	324
Larp4b	4796	3814	2931	4125	2682	3604
Vipr2	63	41	26	28	17	51
Cd226	40	25	13	20	13	19
Ramp3	145	74	61	162	78	184
2610203C20Rik	556	437	343	379	296	557
Kif1a	63	65	29	108	68	82
Sstr1	11	4	4	4	3	1
Apbb2	2880	1999	1662	2451	2352	3874
Dusp23	452	396	221	534	277	835
Gfpt1	1615	1206	936	1277	887	1393
Gucy1b3	1266	945	639	1459	757	1690
Mettl7a3	2	1	1	2	4	8
Abcg1	362	218	196	366	261	430
Asxl3	39	16	22	27	27	105
AU019990	5	3	9	5	17	24
Casp7	506	334	302	482	361	743
Coro2a	47	27	24	52	25	102
Loxl3	218	152	142	250	157	333
S1pr4	20	16	9	38	14	32
Sall1	61	60	20	33	15	51
Sdc1	139	102	96	196	99	220
Duox2	20	13	6	7	5	11
Gata3	98	55	23	58	17	39
2900079G21Rik	0	0	0	0	3	0

Abcb1b	470	357	268	387	454	729
Tcf3	850	510	416	818	507	1031
Stard8	2765	1739	1458	2654	1660	3475
Lrp2	1	0	1	3	6	1
Nfia	4640	3466	2537	4359	3516	6358
Fam81a	592	528	431	611	246	546
Inpp5j	1173	743	629	970	521	820
Polr1b	723	430	360	504	335	520
Pyroxd2	114	125	43	173	190	108
Fsd1l	6119	4065	3200	4838	2648	5069
Ceacam1	655	541	499	817	569	966
Etv4	13	4	14	22	9	40
Hspa1b	84	45	108	75	31	70
Krt222	1732	1107	1030	1200	940	1533
Lpgat1	7546	5238	5084	6378	4245	5312
Mlxipl	1884	1191	835	1083	942	1511
Nudt16	170	112	120	184	151	253
Socs2	2100	1479	1411	1471	1268	1913
Tmem140	1166	641	685	1103	676	1727
Tprn	93	50	51	79	89	131
Dus4l	339	241	204	278	138	292
Aqp9	4	5	2	2	0	2
Gal3st2	2	3	2	6	4	9
Ift122	445	290	226	492	265	573
Pcolce2	1149	946	695	968	508	1181
Rnf141	216	154	129	165	109	145
Crim1	3518	2294	1711	3267	2427	3985
Adra2c	10	4	3	8	11	18
Gpr152	3	6	1	1	1	1
Mir770	0	0	0	1	2	0
Prkca	1937	1435	1071	1600	975	1591
Ncoa4	321	243	222	275	163	266
Gdf6	28	19	24	42	22	65
Dlgap1	32	19	25	17	18	15
Fbn1	1783	1267	1198	1838	1155	3232
Serpinb10	1	2	0	3	3	5
Cav1	21550	12357	10451	18748	12904	29547
Exosc4	421	216	210	427	233	525
Ndrg1	2085	1304	1065	2019	1256	2551
Zfp185	66	37	28	84	39	86
2810047C21Rik1	1	2	0	4	2	5
Abcg5	0	0	0	0	2	1
Akr1b7	0	0	0	0	2	1
Cd274	710	401	235	580	456	784
Cdkn1a	726	343	474	732	457	1034

Foxh1	1	0	0	1	5	1
Gm5122	0	0	0	0	2	1
Gm5434	0	0	0	0	2	1
Impa2	360	173	178	270	298	428
Mir709	0	0	0	0	2	1
Nrep	1223	766	722	1228	750	1688
Pom121l2	0	0	0	0	2	1
Tifa	143	87	79	167	97	187
Tspan7	5670	3661	3101	5796	3448	7540
4930405A21Rik	18	4	16	2	8	8
Angptl7	309	239	240	486	253	419
Arhgap28	224	103	114	166	172	310
Atf5	1986	954	1147	1471	718	1563
Bak1	657	640	359	837	509	1003
BC049715	6	7	8	4	4	2
Ccdc78	2	0	0	2	4	3
Ccnb1	4	4	5	5	4	33
Cwh43	0	0	0	2	1	0
Folr1	0	0	0	2	1	0
Gm609	0	0	0	2	1	0
Gtf2h1	1346	938	829	1015	715	1266
H60c	0	0	0	2	1	0
Hoxa3	46	27	23	44	39	73
Hspa4	10258	7740	7107	8546	5876	9130
Lman1	4843	3276	2764	3887	2472	3805
Mir6939	3	1	4	2	0	0
Nhp2l1	427	309	267	332	184	414
Noc3l	555	383	349	404	296	488
Pnkd	1939	1386	1030	1914	1320	2456
Pnp	389	258	227	410	234	623
Scg2	0	0	0	2	1	0
Six4	443	329	302	325	275	335
C1qtnf5	129	59	48	152	65	147
Mfrp	129	59	48	152	65	147
Doc2g	6563	4105	3679	4729	3129	5768
Igf2bp2	350	183	234	247	150	280
Itga8	323	235	190	400	221	430
Per1	2481	1886	1328	2271	1889	3393
Arhgap27os3	8	3	1	1	0	3
B3gnt3	412	212	185	389	214	550
BC065397	163	90	69	76	69	112
Dip2a	907	473	401	796	524	1023
E030044B06Rik	0	0	0	1	1	1
Evl	974	604	476	949	558	1225
Git2	1961	1372	989	1838	1363	2414

Gm8817	0	0	0	1	1	1
Gprc5c	341	174	183	307	218	458
Hao1	0	0	0	1	1	1
Hipk1	6365	3998	3477	6009	4025	8610
Mir6418	502	345	271	555	320	655
Mvp	1915	1213	1042	1665	1491	2261
Nr4a2	59	47	52	39	24	63
Scube1	85	68	48	86	88	134
Slc9a3r2	4897	3009	2078	5091	2720	5504
St13	4393	3439	3097	3849	2577	3790
Taf4b	254	159	167	185	126	197
Tank	1066	835	635	884	562	974
Cabp1	110	52	55	91	89	147
Rnf215	539	342	302	549	348	703
Prss23	544	423	394	705	382	859
Myct1	2102	1376	894	1961	1229	2623
Rab3d	966	575	453	914	652	1000
Tex30	224	139	123	154	99	168
3930402G23Rik	0	0	0	2	0	1
9630033F20Rik	4134	3060	2730	3660	2219	3493
A530032D15Rik	2	4	0	1	0	0
A530046M15Rik	0	0	0	0	1	2
Abcc2	0	0	0	0	1	2
Ankmy1	7	1	2	1	0	2
Arhgap18	1217	837	1007	1049	1279	2268
Asb17os	0	0	0	0	1	2
Ccdc3	74	40	72	133	59	130
Cryba4	120	59	56	143	82	124
Dbndd1	0	0	0	2	0	1
Duxbl1	0	0	0	0	1	2
Duxbl2	0	0	0	0	1	2
Duxbl3	0	0	0	0	1	2
Dyrk2	758	466	353	528	367	459
E2f6	4266	2830	2622	3349	2259	3634
Edn3	99	55	152	80	41	113
Elovl5	1939	1377	1206	1978	1369	2638
Fam188b	183	107	93	190	113	251
Gm10872	0	0	0	2	0	1
Gm11651	0	0	0	2	0	1
Gm2027	5	0	2	5	6	8
Hip1	3915	2553	1673	4149	2453	4078
Leo1	519	411	411	460	280	508
Lgals9	977	710	485	1008	544	1547
Lin7a	257	182	162	332	160	389
Lpcat1	198	123	112	200	149	261

Mir32	0	0	0	2	0	1
Mir5616	0	0	0	0	1	2
Mtrf1l	507	370	380	411	282	467
Ncoa7	276	218	184	342	199	439
Nobox	0	0	0	2	0	1
Nup62cl	6	3	3	2	1	2
Oaz2	3244	2136	1787	3216	2062	3978
Pde6a	0	0	0	2	0	1
Phf11b	37	24	15	37	23	75
Prokr1	18	18	17	12	4	20
Ptms	3412	2279	1594	3212	2063	4338
Snord15a	0	0	0	3	0	0
Syt8	0	0	0	0	1	2
Taf1a	870	615	573	669	488	788
Tle6	225	123	113	183	161	318
Trpm5	0	0	0	0	1	2
Wdr65	0	0	0	2	0	1
Zpld1	0	0	0	0	1	2
Hccs	4461	2812	2492	3453	1976	3964
Tnfrsf26	40	19	17	48	28	48
Ung	785	508	438	561	413	616
Zfp30	1189	1008	733	1232	289	952
Fam43a	1042	581	469	965	614	1130
Gimap1	192	132	130	199	160	296
Ltb4r2	0	1	0	4	0	3
Sit1	0	1	0	4	0	3
Slc38a2	13901	9382	7420	15833	9582	15352
Tsga10	147	130	78	95	83	109
Zfp773	30	11	17	19	7	10
4930469G21Rik	0	0	0	1	0	2
Ankrd66	0	0	0	1	0	2
Art2b	0	0	0	1	0	2
B4galt2	157	103	94	162	127	220
Cyp2j9	111	79	77	111	105	191
Dnase2a	216	134	115	193	154	319
F2	0	0	0	1	0	2
Hrct1	118	92	62	100	96	246
Mirlet7bhg	41	31	23	49	35	70
Oprl1	0	0	0	1	0	2
Rab31	734	477	377	794	430	921
Slc15a3	65	61	44	107	43	142
Tprg	0	0	0	1	0	2
Zfp397	2658	1794	1573	2092	1396	2289
Jam3	826	503	499	780	566	1096
Atp13a3	3673	2375	2055	2972	1824	2910

Gnal	37	15	18	41	29	45
Mlec	5812	3786	3353	4601	2893	4696
Ppap2a	1945	1097	1202	1824	1301	2540
Erlin1	547	410	334	443	269	495
Tgm1	13	10	5	8	0	7
D10Bwg1379e	3	2	2	1	0	1
4930428G15Rik	0	0	0	0	0	3
4930583K01Rik	0	0	0	0	0	3
AI463170	0	0	0	0	0	3
Chrnb3	0	0	0	0	0	3
Frmd5	5568	3920	3619	4946	2943	3879
Gm10778	18	22	13	14	9	12
Gsap	153	126	88	162	128	250
Ntm	46	37	17	46	46	70
Olfm2	0	0	0	0	0	3
Podn	1570	1108	678	1244	614	1181
S100a16	808	578	520	871	530	1216
Tdrd1	0	0	0	0	0	3
Tnfrsf22	73	24	31	73	42	89
Trip11	2083	1607	1370	1643	1327	1576
Trnp1	30	23	19	67	17	50
Ftsj3	1547	1164	906	1153	831	1522
Slc15a2	284	15	102	267	56	747
Fkbp4	14818	9092	8582	10872	7047	12812
Gsg1l	337	243	156	455	283	305
Gtf2e1	508	320	250	402	193	366
4833412C05Rik	5	5	0	9	4	12
4930443O20Rik	9	0	1	7	8	10
Cnih4	2861	1984	1750	2432	1418	2519
Eef1e1	450	295	330	352	241	389
Gpr137b-ps	287	142	135	258	159	386
Homer2	6067	4243	3858	4730	3553	4524
Iffo2	371	222	214	389	232	500
Krt80	89	44	68	52	47	50
Mpp2	292	145	136	291	169	332
Myot	2761	1326	2046	1635	1644	1702
Snta1	1777	891	997	1585	1538	1727
Srd5a1	48	21	23	47	33	69
Tpst2	897	556	458	864	543	1100
Trim25	2888	1511	1247	2286	1699	3343
Wtip	239	146	126	258	150	307
Itga1	2604	1691	1731	2667	1727	3720
1500017E21Rik	558	381	335	432	265	497
4933417O13Rik	0	0	2	2	3	4
Ccl8	19	8	11	23	12	36

Hhipl1	83	44	28	88	41	120
Snx14	1723	1268	1071	1461	936	1505
Gpt	1350	731	631	1151	811	1544
Tbc1d22a	1267	719	596	1099	926	1277
Chpf2	363	206	155	344	195	484
Tex38	17	20	8	23	30	29
Ppm1h	565	411	325	595	439	695
Pfkl	5191	3184	2443	5264	3521	5142
Atp6ap1l	6	3	7	13	5	19
Aldoc	1	1	3	3	7	6
Il34	162	94	60	175	87	205
Fgfr4	2	3	6	1	1	2
G2e3	465	250	208	363	280	648
Grap2	58	41	30	31	30	35
Grip1	9	12	5	18	16	17
Mpg	182	103	116	173	130	292
Msl3l2	61	29	29	61	44	79
Ppara	7004	4792	3644	5976	3239	3963
Cenpi	16	12	9	20	11	41
Dennd4b	3260	1896	1526	2284	1483	2482
Etohd2	145	119	84	97	81	112
Folr2	276	173	126	180	84	238
Ntsr2	266	177	158	158	123	258
Pak4	223	144	136	161	100	186
Rragd	6429	4265	4260	5190	3564	5025
Acta2	3837	1828	3097	3973	3689	5038
Msn	9851	7231	5692	10811	6671	12903
Mtrr	921	517	465	614	443	678
B930025P03Rik	5	12	4	3	0	7
Ccdc68	159	166	146	139	74	205
Cyp2b10	44	41	35	10	23	50
Mir7050	3	2	2	0	0	2
Mzb1	46	27	25	24	20	29
Vav3	336	208	174	312	199	527
Arhgef5	1575	983	864	1443	1086	1843
Igsf9b	89	70	37	63	39	23
Hip1r	1137	725	473	1034	661	1385
Hoga1	17	14	10	17	18	42
Lrrcc1	1059	685	687	696	515	1125
Angptl3	101	77	64	78	46	77
Cdkn2d	419	266	234	455	250	565
Mir1907	0	1	0	2	1	3
Serpinb7	2	3	4	0	1	2
Tspan11	44	27	21	51	33	62
4930473A02Rik	2	2	1	0	0	1

Ankef1	2	2	1	0	0	1
Gng3	2	2	1	0	0	1
Lnx1	816	501	395	679	468	1278
Nrarp	579	314	495	571	483	1089
Tmem50a	2098	1328	1251	2027	1350	2799
Larp4	3116	2102	1783	2603	1573	2524
Dyrk4	0	0	1	2	1	3
Pnpla6	564	359	267	576	371	625
Scn2b	40	25	15	49	35	44
Ly6d	1	1	1	6	3	2
Tnfsf13	341	244	200	302	294	496
Mrgprh	206	148	113	153	107	123
P2rx3	33	25	18	17	12	27
Best3	289	232	154	214	162	198
Gabrd	2	4	1	3	6	11
A330041J22Rik	24	14	10	16	24	48
Cdc6	34	29	15	30	30	80
Liph	1	0	0	1	2	3
Slc38a11	38	24	30	70	42	43
Gaa	6706	4047	3206	4957	3029	4811
Gsdmd	315	243	181	302	263	462
Tdrp	166	136	120	178	138	328
Camk2n2	17	13	10	24	15	32
H3f3b	3760	2654	2240	3536	2643	5338
Ipo7	6317	4828	4346	5708	3608	4712
Ldlrad3	333	251	273	392	261	621
Dcst1	1	0	1	2	1	6
Gtpbp4	2125	1562	1360	1741	1079	2147
Hmox1	347	207	154	284	224	479
Lpar4	227	137	102	190	143	342
Slc12a8	23	12	15	28	23	34
A630007B06Rik	933	849	661	763	652	778
Csdc2	2313	1471	1173	2637	1559	2250
9330175E14Rik	9	5	1	16	4	14
AI317395	278	180	172	198	124	254
Aqp8	51	33	95	31	40	52
Cd34	6300	4112	3569	6590	3643	9075
Cplx1	5	0	0	4	10	2
Fam13a	1568	1388	1234	1408	1009	1616
Fbp1	5	0	1	0	0	1
Lhx9	0	1	0	3	1	2
Naip2	120	84	63	125	95	166
Psma3	305	222	207	247	145	286
Ranbp1	936	768	646	712	558	992
Wwox	168	110	72	167	124	202

Ahsa2	1959	1282	1565	1669	1044	1867
Adcy1	244	228	277	267	89	308
Peak1	3256	2544	1861	3419	2692	3862
Rap1gap	483	297	219	426	316	587
Edem2	417	265	221	368	274	607
Ell2	1242	946	773	1118	626	1088
Plxnc1	296	222	116	165	101	278
Slc23a1	1	2	2	0	0	1
Dnm3os	241	168	101	250	164	292
Htra2	833	462	371	747	520	888
Rasa4	480	213	140	379	259	507
Tsr1	908	610	581	747	477	764
Chst11	404	230	141	410	224	433
Gfpt2	676	429	510	611	282	607
Gm3258	2	1	2	0	1	0
Smarcd2	1016	617	460	948	575	1217
Cdca3	25	11	2	17	13	47
Erdr1	367	82	32	134	25	90
Fgf5	2	2	5	1	1	1
Fhl1	5853	4470	3679	5652	5067	7790
Olfr1393	16	9	13	11	7	4
Pprc1	785	530	343	625	312	526
Fam227b	6	2	3	3	1	0
Slc9a3	24	9	4	3	8	7
Zfp770	1891	1348	1196	1747	954	1372
Fam49b	1541	979	949	1172	802	1386
Nmnat2	13	8	5	23	6	23
Rel	244	190	123	185	125	158
Btg2	1484	782	738	1247	903	1724
Ccdc58	535	431	403	433	307	555
Zfp300	43	16	17	41	26	57
Slc47a1	3379	1822	1550	2206	1562	2465
Gm5577	12	5	8	3	4	7
Sat2	107	72	76	95	101	204
Clec1b	31	32	33	16	26	24
Il6ra	254	142	120	151	94	208
Spryd3	529	309	270	513	316	658
6030408B16Rik	17	6	18	25	13	46
2010002M12Rik	305	218	187	254	142	253
Sox6	1276	821	540	839	661	734
Tspan4	1047	557	507	942	555	1325
C330013E15Rik	20	16	14	25	22	39
Cdc42ep1	1923	1153	844	1862	1075	2151
Fam101b	2659	1748	1565	2554	1649	3694
Lrrk1	944	631	425	1058	583	988

Neurl1b	362	198	160	355	194	449
S1pr3	922	511	574	1064	518	1191
Mavs	3298	2284	2126	3086	1588	2643
Psme3	3447	2523	1962	2679	1909	3105
Ankle1	11	3	0	10	4	20
Gng4	9	8	6	3	4	6
P2rx1	19	7	16	8	8	10
Cthrc1	8	7	6	5	7	46
Exoc3l4	109	65	45	113	80	123
Gper1	59	29	38	76	37	89
Crip1	512	336	353	634	310	780
Mgat4a	1312	810	802	1182	794	2170
Mpzl1	494	313	276	453	323	707
Tor4a	258	162	121	266	152	334
AI506816	16	18	18	15	11	6
Kcnc3	194	119	90	107	94	137
Nsun7	2	3	1	6	3	7
Orc1	0	5	1	9	0	19
Sntb1	65	41	39	74	66	79
Armcx4	634	412	371	472	328	522
Brix1	1126	770	664	875	566	1053
Tcam1	6	2	1	1	1	1
Rwdd4a	2105	1507	1409	1813	1137	1924
Tmem110	427	246	262	409	268	639
Map1lc3a	10369	4949	5233	9897	5636	11882
0610039K10Rik	1	1	0	2	1	5
1110015O18Rik	2	0	0	0	0	0
1600016N20Rik	1	0	0	1	1	4
1700003G18Rik	0	1	1	0	0	0
1700009J07Rik	1	1	0	0	0	0
1700067K01Rik	0	2	2	2	5	6
1700112E06Rik	11	13	10	21	18	22
1700123O21Rik	0	2	0	0	0	0
2010001E11Rik	2	0	0	0	0	0
2010106E10Rik	0	0	2	0	0	0
2210409D07Rik	1	0	0	0	2	4
2310015D24Rik	46	35	26	48	46	70
2310047M10Rik	549	267	218	481	327	554
2610028H24Rik	65	30	20	22	25	35
3300002I08Rik	1	1	2	4	1	8
4921508D12Rik	0	1	1	0	0	0
4930452G13Rik	1	0	1	0	0	0
4930502E18Rik	1	1	0	0	0	0
4930503L19Rik	334	269	160	334	222	519
4930542D17Rik	0	1	1	0	0	0

4930578C19Rik	381	260	161	285	318	503
4933402C06Rik	2	0	0	0	0	0
4933413J09Rik	2	0	0	0	0	0
4933425L06Rik	2	0	0	0	0	0
5730507C01Rik	5	1	6	13	5	10
5830432E09Rik	10	1	4	11	7	13
6330403A02Rik	183	91	114	146	148	270
6430531B16Rik	1	0	1	0	0	0
8030423F21Rik	1	1	0	0	0	0
9830166K06Rik	2	0	0	0	0	0
A230077H06Rik	2	0	0	0	0	0
A330050F15Rik	0	1	1	1	2	6
A430093F15Rik	7	10	6	4	4	5
A530013C23Rik	42	31	17	33	44	71
A730043L09Rik	1	1	0	0	0	0
A830009L08Rik	2	0	0	0	0	0
Aaed1	1174	847	676	941	653	964
Aanat	1	1	0	0	0	0
Abcb7	5287	3863	3372	4880	2458	4675
Abcc9	28695	22968	16758	26908	12713	24088
Abhd1	156	70	60	76	91	329
Abhd3	264	145	171	187	118	212
Acad12	2121	1097	951	1491	901	1439
Acta1	8519	2735	6846	5889	12069	11331
Actl10	0	1	1	0	0	0
Actn4	5309	3710	2696	5437	3397	6450
Adam8	54	20	19	29	12	24
Adap1	107	74	81	77	56	91
Adck3	13245	7832	6745	10658	11967	13767
Adcy2	20	16	8	10	9	9
Adora2b	25	16	14	40	18	34
Adora3	33	23	20	15	15	27
Agmo	279	159	121	281	147	346
Agpat4	155	74	61	145	92	175
AK129341	319	248	173	208	168	297
Akr1c19	64	42	38	63	59	95
Alkbh8	335	232	197	258	180	249
Ampd3	2725	1698	1566	1997	1499	2188
Ankk1	0	3	2	0	0	1
Ankrd44	734	458	369	606	481	1010
Ankrd49	435	333	299	363	210	440
Aox4	0	1	1	0	0	0
Arfgef2	4382	2964	2511	3547	2355	3352
Arhgap17	850	555	450	742	555	1167
Arl15	470	337	317	458	339	781

Arl16	278	159	145	257	197	320
Atic	1335	956	698	1032	691	1132
Atp11c	1398	823	651	1201	811	1719
B4galt6	2063	1458	1260	1700	1039	1959
Baat	3	2	3	1	0	2
Barx1	2	0	0	0	0	0
BC048507	2	0	0	0	0	0
Bcam	10214	6393	4500	9607	6361	11029
Bcar3	285	136	126	233	181	333
Bcl6b	832	519	622	760	645	1381
Bco2	17	13	6	8	3	12
Bdh1	4335	3257	3506	3126	3062	3782
Bhlha15	0	1	1	2	3	3
Bst2	450	248	213	451	201	698
C1galt1c1	534	353	367	410	269	512
Camk1g	6	8	4	14	8	14
Canx	18578	12393	11464	13840	10465	15308
Cblb	1288	755	558	1060	861	1385
Cbx6	2109	1103	956	1928	1195	2205
Ccdc121	0	2	0	0	0	0
Ccdc82	1047	689	595	941	778	1286
Cck	0	0	2	0	0	0
Ccl7	29	17	13	16	8	18
Ccl9	381	339	271	315	164	462
Ccnd2	6928	4623	3821	5232	3788	5155
Ccr10	2	1	1	5	2	5
Cct3	4612	2934	3128	3925	2182	4206
Cct6a	5018	3436	3380	4254	2544	4783
Cd200r3	1	0	1	0	0	0
Cdh4	206	118	224	112	146	172
Cdk5rap1	742	389	371	537	285	557
Cdk6	217	147	111	134	114	164
Cdkal1	905	664	506	759	439	795
Chgb	6	6	7	14	4	25
Chid1	840	543	553	667	438	746
Cldn12	1974	1232	1070	1405	994	1765
Cldn13	0	0	2	0	0	0
Clhc1	4	1	0	4	3	7
Clrn1	1	1	1	3	3	4
Col5a3	497	326	230	479	270	723
Colec10	0	1	1	0	0	0
Commd7	466	311	265	448	328	606
Crhbp	2	0	0	0	0	0
Crispld1	176	92	87	165	105	236
Csf1	2075	1534	1533	1786	1310	1701

Csgalnact1	297	193	186	263	213	496
Csrp2	590	477	379	878	354	845
Ctnnal1	2630	1715	1483	2156	1245	2255
Ctnnd1	5113	3126	2462	4969	2988	5914
Ctso	1098	664	620	1011	702	1389
Cx3cl1	260	190	153	295	179	352
Cygb	2716	2075	1363	3154	1597	3481
Cyp26a1	2	2	0	4	5	3
Cyp2s1	8	3	3	8	15	7
D030018L15Rik	1	0	1	0	0	0
D030025P21Rik	0	0	2	0	0	0
D3Ertd751e	1673	1462	1140	1274	1150	1585
D5Ertd579e	8485	6210	4872	7234	4547	6013
Daam2	550	341	283	575	333	657
Dbt	5755	4069	3237	4576	3041	4805
Defb20	2	0	0	0	0	0
Dffa	1080	631	634	854	476	932
Dgat2l6	1	1	0	0	0	0
Dis3	787	532	473	572	434	695
Disc1	46	26	20	51	33	59
Dnajb2	3379	2098	2025	2670	1847	2609
Dpep2	33	16	13	33	18	55
Dpp4	192	131	78	93	76	169
DQ267102	0	0	2	0	0	0
Drd3	1	0	1	0	0	0
Dscaml1	0	1	1	0	0	0
Dynlrb2	0	2	0	1	3	5
E030018B13Rik	1	0	1	0	0	0
E230019M04Rik	2	0	0	0	0	0
Edaradd	11	3	7	5	1	5
Egfl8	28	17	7	28	18	41
Enc1	490	279	423	552	421	747
Enpp2	806	473	440	570	328	750
Epha6	1	0	1	4	1	3
Epha8	0	2	0	1	3	5
Ephb6	36	22	11	39	24	48
Esf1	1457	1157	938	1100	850	1539
Esrrb	1633	1075	794	1306	775	1153
Eva1b	162	127	92	192	123	225
F11r	2297	1244	1170	2061	1441	2487
F12	0	1	1	0	0	0
Faah	14	8	6	9	1	6
Fam107a	807	518	354	781	397	1214
Fam115c	142	97	78	130	98	241
Fam154a	1	2	0	1	4	6

Fam163a	3	4	3	0	1	3
Fcer1a	1	0	1	3	0	6
Fcrl1	4	2	1	4	4	10
Fhod1	834	450	333	672	469	1010
Flot1	5591	3972	2817	5137	3772	7275
Foxd4	2	0	0	0	0	0
Fpr2	2	3	5	2	0	2
Frmd8	366	180	141	377	169	429
Fut7	1	3	3	0	0	2
Gab3	94	68	67	85	94	171
Gbp3	1233	804	513	999	697	1818
Gbp4	1938	1254	762	1445	1386	2358
Gdpd4	0	2	0	0	0	0
Gfra2	125	74	48	52	42	106
Gfra4	162	85	61	155	106	169
Gja10	1	1	0	0	0	0
Gje1	0	2	0	0	0	0
Gk2	1	1	0	0	0	0
Gm101	2	0	0	0	0	0
Gm10640	3	0	2	1	0	0
Gm10941	0	0	2	0	0	0
Gm12338	48	67	11	22	20	39
Gm13271	2	0	0	0	0	0
Gm14085	2	0	0	0	0	0
Gm16432	1	1	0	0	0	0
Gm19299	1	0	1	0	0	0
Gm19395	1	1	0	0	0	0
Gm20300	2391	1551	1423	1920	1202	2079
Gm20594	1	0	1	3	1	4
Gm3696	4	1	1	3	3	11
Gm5091	2	3	2	2	0	0
Gm5177	2	1	2	8	1	6
Gm5878	1	0	0	2	0	4
Gm6416	402	262	221	330	134	341
Gm6654	2	0	0	0	0	0
Gm805	1	0	1	0	0	0
Gnmt	29	20	19	30	54	31
Gnpda1	568	403	349	452	295	503
Gpr1	16	10	2	19	9	25
Gpr176	11	11	5	13	12	26
Gpr25	0	0	2	0	0	0
Gpr98	4	2	1	4	4	10
Gprasp1	1693	1223	984	1384	923	1448
Guca2b	0	1	0	1	6	0
Hamp2	0	2	0	0	0	0

Hand1	0	1	1	0	0	0
Haus7	161	101	94	168	105	236
Hfm1	0	2	0	0	0	0
Hist1h3a	0	2	0	0	0	0
Hormad2	1	1	0	0	0	0
Hoxc4	1	0	1	0	0	0
Hsf1	1339	901	596	956	634	1046
Hsh2d	2	3	2	0	2	0
Hsp90ab1	64397	47702	46915	53451	35523	58082
Hspb8	20704	13448	12394	17180	9205	18187
Htt	1761	1236	904	1333	869	1592
I730030J21Rik	1	1	0	0	0	0
Icam2	595	399	321	578	373	802
Il1f9	0	3	2	1	0	0
Intu	2948	2013	1596	1977	1636	2632
Itgbl1	292	247	250	428	228	501
Itsn1	3754	2602	2023	2977	2061	2780
Jpx	112	84	59	69	57	87
Kcnip3	108	54	41	104	53	155
Kcnt1	51	27	17	34	44	72
Kif20a	68	28	25	57	32	109
Kif21a	2172	1512	1241	1686	1225	1701
Klf10	884	493	514	886	485	1194
Klf13	3081	2061	1727	3622	1677	4007
Klhl12	1565	858	880	1143	711	1308
Klhl7	3758	2447	2052	3097	1838	3046
Klk1b16	0	2	0	0	0	0
Klk9	0	1	1	0	0	0
Ldb2	377	276	267	360	307	633
Leap2	2	0	0	0	0	0
Lgals12	3	3	7	3	2	0
Lgals3	128	49	47	108	58	178
Lig1	159	129	86	153	120	280
Lmod3	6195	4664	4020	4770	3491	6150
LOC100504608	0	0	2	0	0	0
LOC101243624	0	2	0	0	0	0
Lsmem1	1146	739	668	805	612	1053
Lyrm7	1019	714	614	762	603	829
Lysmd1	25	17	12	31	18	42
Lyve1	1196	914	656	1022	525	1125
Manba	998	891	718	839	487	1250
Map3k11	1452	875	675	1331	840	1676
Map6d1	13	9	12	2	4	15
Mapt	4237	2544	2582	2970	2333	3614
3-Mar	68	28	28	61	43	86

Mat1a	1	2	2	0	1	0
Mc1r	0	0	2	0	0	0
Mcoln3	2	0	0	0	0	0
Megf8	2068	1161	876	1711	1492	1957
Mfap3l	1312	956	887	1226	730	962
Mgl2	258	182	190	201	75	294
Mia2	1	1	0	0	0	0
Mical1	473	321	310	465	344	701
Mina	1035	813	764	900	625	976
Mir1897	2	0	0	0	0	0
Mir192	0	2	0	0	0	0
Mir208b	1	0	1	0	0	0
Mir26a-2	2	0	0	0	0	0
Mir3094	0	1	1	0	0	0
Mir339	1	0	1	0	0	0
Mir344g	1	0	1	0	0	0
Mir3569	2	0	0	0	0	0
Mir5121	660	377	349	644	378	822
Mir678	43	38	20	56	36	67
Mir6905	1	6	6	0	0	5
Mir6960	0	2	0	0	0	0
Mir6976	5	3	2	0	2	2
Mir7006	0	1	1	0	0	0
Mir7090	0	2	0	0	0	0
Mir7648	1	1	0	0	0	0
Mir8104	3	7	0	0	1	2
Mpzl2	13	7	5	11	14	21
Mrpl3	4085	2511	2519	2908	2056	3901
Mrrf	1642	1178	994	1318	898	1521
Mrs2	1281	934	880	1093	779	1030
Mrto4	492	328	290	355	226	474
Msln	11	4	5	15	11	13
Mtmr11	192	113	104	175	127	277
Mvk	191	105	97	161	133	258
Myf6	2	0	0	0	0	0
Mylk2	3	0	0	4	3	3
Myo1h	11	3	2	11	5	18
Naa50	5084	3368	3286	4172	2644	4508
Ndufa4l2	487	287	223	476	245	657
Nkx6-3	1	0	1	0	0	0
Nme7	154	112	85	165	120	206
Nmral1	56	51	27	72	35	118
Npnt	30	31	21	61	34	42
Nptx2	2	0	0	0	0	0
Npy5r	1	0	1	0	0	0

Nr1d1	2317	1727	832	1789	969	1361
Nr4a3	195	249	131	201	128	122
Nrg4	40	48	27	23	23	42
Nrgn	137	129	116	73	116	91
Nrip1	776	583	410	546	476	504
Nrsn1	1	1	0	0	0	0
Nrsn2	2	0	0	0	0	0
Nudt14	70	41	35	70	50	97
Nudt15	56	39	38	43	23	40
Olfr1396	50	41	32	44	65	90
Olfr433	2	0	0	0	0	0
Orc2	913	624	608	732	446	912
Os9	1936	1380	916	1735	1314	2424
Pabpc4	5922	4042	3687	4621	3320	4975
Parp14	2741	1690	1420	2518	1612	3471
Pdxk	1293	719	586	1027	475	862
Peg13	3457	2293	1605	3335	2219	3796
Phc2	1239	699	626	1181	748	1345
Phlda2	1	1	0	0	0	0
Phospho2	1847	1352	1293	1685	988	1640
Phox2b	2	0	0	0	0	0
Pik3r3	1026	588	586	910	753	1151
Pip5k1b	1559	1108	936	1412	741	1316
Pisd-ps1	1401	593	547	976	791	1563
Piwil2	90	63	74	52	59	69
Pkhd1	0	0	2	0	0	0
Plbd1	2423	1498	1622	1712	1253	2430
Plekha5	1668	1175	745	1131	840	1360
Plekhg3	837	482	381	780	441	1048
Pm20d2	6722	4503	4131	5395	3622	5527
Pml	667	381	253	600	340	816
Pnma5	0	2	0	0	0	0
Pomt2	958	627	432	724	410	721
Ppic	1057	613	654	937	636	1750
Ppm1f	1989	1130	1085	1830	1173	2513
Prkag3	252	141	153	199	87	195
Prss55	22	12	2	8	6	4
Psme2	857	513	429	810	485	1065
Psmg1	607	384	320	425	282	523
Ptplad2	151	133	108	148	140	310
Ptpn2	1474	915	841	1040	798	1237
Pxdn	3710	2316	2158	3249	2344	5429
Rab4b	368	245	193	331	288	464
Rab5c	1882	1195	973	1748	1156	2332
Rab9b	12	9	11	6	8	5

Rasal2		4165	2610	1871	3972	2647	4303
Rasgrp3		2545	1663	1523	2386	1745	3313
Rassf9		984	565	338	910	614	907
Rbm44		1	1	0	1	1	7
Rdh19		1	0	1	0	0	0
Reg4		0	1	1	0	0	0
Rhoc		1521	872	808	1411	841	1997
Rhog		479	292	225	486	264	607
Rmrp		17	10	0	1	5	4
Rnmtl1		700	537	524	623	391	673
Rock2		12000	9049	7541	9367	7044	10475
Ror1		270	168	106	212	90	119
Rrm2b		2562	1805	1411	2023	1380	2128
Runx3		20	10	9	28	11	29
Sash1		2404	1611	1167	2305	1421	3039
Sbk3		452	359	346	485	598	554
Scarna10		2	0	0	0	0	0
Scarna8		1	1	0	0	0	0
Scel		5	2	3	0	2	2
Sema3g		926	471	467	810	532	1113
	4-Sep	1245	975	918	1391	860	2169
Serpinh1		7723	5141	5611	7145	3363	7225
Sesn3		270	197	144	217	135	180
Sgk2		0	0	2	0	0	0
Sh2d2a		6	3	4	13	6	9
Sh2d4b		15	11	6	3	7	9
Shank3		3030	1684	1388	2149	1952	3917
Shd		15	12	7	6	4	12
Sik3		4408	3263	2127	3584	2168	3313
Sim2		2	0	0	0	0	0
Sipa1		1127	618	495	972	646	1250
Slc22a13		1	0	1	0	0	0
Slc22a4		379	228	182	231	184	295
Slc24a3		106	76	75	126	85	169
Slc24a4		7	0	1	9	9	3
Slc25a28		845	559	446	729	590	1114
Slc26a4		7	12	7	7	2	6
Slc2a9		58	41	33	34	64	133
Slc44a2		4428	2962	2040	4354	2836	4834
Slc7a14		0	2	0	0	0	0
Slfn5		7908	5295	4755	7564	5087	11685
Snap25		1	0	1	0	0	0
Snord23		1	0	1	0	0	0
Snrpn		2796	1931	1748	2837	2022	3390
Snurf		2796	1931	1748	2837	2022	3390

Snx31	1	0	1	0	0	0
Sorbs2os	1	2	2	0	1	0
Sort1	4215	3063	2158	3618	1967	3138
Spaca1	1	0	0	0	2	4
Spatc1l	0	0	2	0	0	0
Spats2	235	171	147	250	196	312
Spink2	1	1	0	0	0	0
Sptlc3	1	0	1	0	0	0
Srgap2	1675	1152	920	1556	1088	2295
Srm	286	219	147	232	119	242
Sstr2	1	1	0	0	0	0
St7	685	447	368	571	298	518
Stx3	646	424	349	557	412	981
Susd5	49	30	52	68	68	78
Syce1	0	0	2	0	0	0
Syde2	2439	1821	1016	2656	1744	2491
Syngap1	148	127	90	120	68	133
Syt4	0	0	1	1	1	4
Taco1	1675	1232	1103	1553	917	1305
Tanc1	3060	1709	1398	2660	1852	3162
Tbc1d22b	1037	721	593	740	604	877
Tbkbp1	599	303	284	437	409	738
Tcaim	3566	2320	2337	2855	1992	3081
Tfam	2284	1469	1375	1822	1204	1934
Thbs4	198	235	209	387	187	454
Timp3	29452	20267	16040	30178	19781	37335
Tmem252	6	2	11	11	8	28
Tmem26	3	2	1	8	3	5
Tmem52	53	12	20	18	15	23
Tmem91	6	4	4	1	2	4
Tnfrsf9	8	9	6	2	5	6
Trat1	1	1	0	0	0	0
Tril	503	306	223	488	313	561
Trp73	1	1	0	0	0	0
Trpc7	0	0	2	0	0	0
Ttc34	5	5	3	1	1	4
Ttll7	592	394	448	661	428	915
Tubb4b	4992	3254	2849	4027	2434	4202
Txlna	1902	1322	889	1418	907	1579
Uchl1os	2	0	0	0	0	0
Uggt1	3274	2331	1888	2858	1674	2686
Uhmk1	302	213	169	237	146	242
Unc13b	1050	686	523	901	774	1202
Uros	1207	711	639	845	547	1089
Ust	183	116	84	141	50	135

Vdr	24	23	11	12	11	17
Vegfc	378	219	195	351	253	446
Vsig10	406	270	254	387	286	619
Wdhd1	105	42	52	92	63	148
Wdr12	802	529	454	619	351	755
Zc3h6	356	265	239	272	332	705
Zc3h8	434	295	302	316	239	411
Zdhhc11	0	2	0	0	0	0
Zdhhc18	973	556	480	902	602	1044
Zdhhc20	1378	885	765	1109	937	2062
Zfp46	2005	1344	1006	1582	1536	2567
Zfp541	0	2	0	0	0	0
Zfp9	499	368	318	401	252	470
Zfp948	487	352	387	479	365	1069
Zfp961	612	430	352	515	307	465
Zkscan6	288	160	140	230	195	380
Txndc2	4	6	9	4	3	3
Cir1	226	139	129	220	122	405
P4ha2	2261	1305	1356	1831	1001	1928
Sema3d	330	249	127	277	115	193
Eda	123	84	84	153	91	176
Akt3	2367	1529	1299	2196	1547	2861
Cnpy4	207	131	134	228	139	296
Neto2	134	117	74	126	53	92
Adam15	2569	1462	1294	2256	1428	3265
Ide	7722	3599	4687	4612	3265	7023
Chst10	35	28	21	30	9	25
Trp53cor1	7	4	3	6	7	17
Ret	288	151	201	175	95	308
Fhl2	58912	38378	37078	67307	42164	72636
Ralgps2	827	568	418	607	442	621
Fam98c	266	125	118	219	162	311
Akap17b	298	232	205	205	174	301
Card10	1301	718	585	1133	788	1360
Arpc5	1807	1084	1007	1662	1069	2379
Cyp2e1	104	154	86	101	87	75
Zfp750	15	9	6	6	4	9
Fbln5	1152	690	617	1303	610	1371
Filip1	6105	5213	3906	5236	3897	4638
Mtf1	1278	833	761	1062	651	1047
Nkain4	5	4	4	12	10	6
Pah	4	4	3	3	0	2
Pak3	32	28	18	20	20	14
Rbm11	1	1	1	3	1	6
Tmem200b	129	64	62	158	77	134

Tmem50b	3289	2154	1949	2841	1582	2815
Trim69	11	9	7	5	3	9
Tshz1	1985	1355	1148	1898	1412	2397
Ube3c	3271	2143	1856	2723	1679	2608
4930599N23Rik	17	8	11	9	4	11
Coro1b	1453	867	675	1234	837	1796
Dcakd	1452	890	673	1124	619	1092
Epb4.1l1	637	473	341	698	398	840
Fdx1l	610	420	389	457	322	588
Gngt2	221	134	134	217	131	368
Oas1e	0	1	0	0	3	3
Pitx3	7	6	4	2	4	3
Rab30	266	167	197	202	151	209
Sycp3	5	1	1	4	6	7
Uchl1	543	307	298	672	271	673
Homer3	331	176	176	303	201	427
Rnf6	2574	1754	1550	2132	1341	2291
Mir546	443	263	255	448	296	523
Gnb2	4400	2198	1964	3961	2289	4784
Grwd1	352	199	190	258	160	249
Iars	3618	2545	1993	2972	1956	2891
Plek	446	358	185	270	211	400
Dusp4	110	64	48	136	53	147
Dock6	3504	2156	1314	2864	2176	3792
Trmt10c	1159	930	863	989	741	1114
Hpn	46	12	12	28	32	53
Nat8l	131	62	85	91	45	100
Rsl1d1	3196	2294	2145	2593	1799	3225
Tgfb1	946	646	493	887	582	1251
Rom1	559	276	314	459	395	675
Enpep	638	320	379	725	466	594
Rfwd2	1627	1232	1018	1308	954	1535
Vamp8	963	691	564	899	671	1328
Fbln7	23	29	19	28	7	16
Prickle1	2022	1221	845	1852	1200	2120
Col6a6	304	194	199	199	157	292
Cdv3	4341	2697	2912	3372	2355	3918
Kcnj3	1685	1069	715	1258	810	867
Lrp4	1045	631	481	700	520	776
Rab28	3593	2520	2244	2881	1858	3581
Blcap	2166	1414	1158	1615	1076	1942
Dlk2	8	3	3	2	2	3
Gp5	14	13	8	3	11	5
Foxs1	54	28	20	60	28	71
Anapc2	1758	1155	881	1811	1062	1942

Arl4a	1151	679	672	1032	776	1410
AW549542	98	80	51	63	121	176
Csl	18	6	7	5	7	6
Gm1141	10	1	2	2	1	2
Gm11837	101	60	71	74	40	86
Lnp	1792	1120	971	1402	913	1410
Mir6362	8	2	6	3	0	5
Parp8	777	472	395	629	555	942
Pdp2	1138	894	770	1023	695	869
Pkd2l2	351	336	306	361	241	266
Slc35d2	110	52	55	98	65	159
Snx10	629	449	428	619	514	869
Stap2	647	389	306	506	410	882
Stxbp6	558	395	309	490	395	814
Ulk4	19	18	13	9	6	21
Uqcc1	7707	3728	4839	5122	3902	5749
Abhd17a	758	418	335	641	419	926
Ddx21	2523	1815	1569	2161	1428	2072
Gdf7	3	3	2	2	1	0
Nudt18	661	426	462	512	306	684
Usp24	7471	5005	4453	5730	4360	5661
Mb	448394	329273	283581	485502	372478	626708
Mvb12a	508	258	266	471	290	622
Ago2	1734	1335	948	1463	1008	895
Nkrf	249	170	129	189	113	192
Zfp759	465	268	293	300	252	390
F5	66	73	40	39	46	54
Chn1os3	3	5	3	7	7	10
Gm15417	277	165	209	311	182	463
Uba7	1025	517	433	708	616	1272
9330102E08Rik	199	159	136	162	113	170
Acss2	2197	1234	1231	1523	1131	1794
Cyp26b1	181	199	808	298	1199	1476
Myh10	2317	1760	1400	2046	1249	2030
Nos3	1547	840	634	1353	815	1705
Pde3a	1945	1577	975	1668	1083	1006
Zhx3	707	623	454	724	578	1113
Aen	726	457	447	539	373	654
Cyb5d2	1038	701	651	860	544	932
Eef1a1	17098	10830	9907	17239	9958	24451
Rec8	8	2	2	12	10	4
0610009B22Rik	789	492	471	599	412	742
1100001G20Rik	1	0	0	0	0	0
1110002L01Rik	424	312	264	365	250	360
1110017D15Rik	3	1	1	2	3	6

1110065P20Rik	188	101	85	186	97	208
1600014K23Rik	1	0	0	0	0	0
1600015I10Rik	4	1	3	5	2	9
1700001K19Rik	0	1	0	0	0	0
1700007K09Rik	0	0	0	1	0	1
1700011A15Rik	0	0	0	0	0	1
1700011I03Rik	2	0	3	1	0	1
1700012A03Rik	0	0	0	1	0	0
1700013G24Rik	0	0	0	0	1	0
1700013H16Rik	0	0	0	1	0	0
1700016G22Rik	0	0	1	0	0	0
1700017G19Rik	0	0	0	0	1	0
1700017J07Rik	0	0	0	1	0	0
1700019B21Rik	0	0	1	0	0	0
1700019G17Rik	71	42	53	43	36	71
1700019L03Rik	4	1	1	3	5	4
1700020A23Rik	0	0	0	1	0	0
1700020G17Rik	0	1	0	0	0	0
1700020M21Rik	0	0	0	1	0	0
1700020N18Rik	0	0	0	0	0	1
1700021N21Rik	0	0	0	1	0	0
1700022I11Rik	0	0	1	0	0	0
1700023F02Rik	0	0	1	0	0	0
1700029F12Rik	0	0	1	0	0	0
1700029H14Rik	0	0	0	0	1	1
1700031F05Rik	0	0	0	0	1	0
1700037H04Rik	185	115	86	135	77	146
1700041M19Rik	0	0	0	1	0	0
1700042G15Rik	0	1	0	0	0	0
1700045H11Rik	0	0	0	0	0	1
1700047G03Rik	0	1	0	0	0	0
1700055N04Rik	4	1	2	0	0	3
1700061I17Rik	1	0	0	0	0	0
1700064J06Rik	1	0	0	0	0	0
1700088E04Rik	59	30	34	52	49	71
1700094J05Rik	3	4	3	1	3	2
1700102H20Rik	9	8	1	8	9	13
1700102P08Rik	30	15	12	17	11	19
1700105P06Rik	3	1	1	1	0	1
1700106J16Rik	0	0	0	0	0	1
1700112H15Rik	0	0	0	0	1	0
1700120K04Rik	0	3	1	0	1	0
1810010H24Rik	70	54	36	39	39	54
1810034E14Rik	342	231	206	280	189	279
1810046K07Rik	0	0	0	1	0	0

1810055G02Rik	251	217	120	192	99	244
1810065E05Rik	0	0	0	0	0	1
2010109A12Rik	3	2	0	2	5	4
2010308F09Rik	0	0	0	0	0	1
2210011C24Rik	55	21	24	24	17	41
2210013O21Rik	423	266	209	398	235	522
2210416O15Rik	6	1	3	10	1	11
2310001H17Rik	56	24	23	27	52	75
2310005G13Rik	1	2	0	3	2	4
2310009B15Rik	77	46	35	81	48	92
2310015A10Rik	1063	697	619	790	867	1302
2310030A07Rik	0	0	0	0	0	1
2310034G01Rik	29	11	11	12	7	17
2310034O05Rik	0	0	0	1	0	1
2310036O22Rik	1500	992	854	1286	1059	1922
2310057J18Rik	0	0	1	0	0	0
2310061I04Rik	3564	2547	1973	2791	1900	3570
2310069G16Rik	104	88	55	87	23	100
2410002F23Rik	380	255	212	312	262	515
2410004N09Rik	65	57	44	51	38	64
2410004P03Rik	5	3	1	5	4	8
2410006H16Rik	135	100	70	139	91	189
2610005L07Rik	105	88	49	57	57	95
2610020H08Rik	41	25	39	50	36	74
2610305D13Rik	719	576	576	584	666	1308
2610524H06Rik	17	21	12	27	15	40
2700046A07Rik	2	0	3	0	1	1
2700049A03Rik	398	301	222	279	213	380
2700069I18Rik	0	0	0	0	0	1
2810004N23Rik	1779	1325	1189	1360	1176	1578
2810006K23Rik	623	528	435	566	357	621
2810417H13Rik	29	13	9	15	11	80
2810429I04Rik	1	2	2	1	0	1
2810442N19Rik	0	0	0	0	0	1
2810454H06Rik	33	26	25	35	34	56
2900026A02Rik	2640	1536	1098	2772	1365	2544
2900057B20Rik	0	0	0	0	0	2
2900092D14Rik	0	0	0	1	0	1
3110015C05Rik	1	0	0	0	0	0
3632454L22Rik	1	0	0	0	0	0
3830417A13Rik	0	0	1	0	0	0
4833418N02Rik	50	28	29	36	46	73
4833424O15Rik	22	14	15	19	12	9
4833427F10Rik	9	15	3	10	8	43
4921504E06Rik	1	0	0	0	0	0

4921509O07Rik	0	0	0	1	0	0
4921524L21Rik	0	0	0	0	0	2
4921525O09Rik	0	0	0	0	1	0
4921529L05Rik	1	0	0	0	0	0
4930404A10Rik	0	1	0	0	0	0
4930412O13Rik	11	4	3	3	2	7
4930413F20Rik	1	0	0	0	0	0
4930415O20Rik	0	1	0	1	5	0
4930417O13Rik	0	0	0	0	0	1
4930417O22Rik	0	0	1	0	0	0
4930419G24Rik	0	0	0	0	1	0
4930426D05Rik	1	0	0	0	1	3
4930429B21Rik	8	4	9	2	2	8
4930430A15Rik	0	0	1	0	0	0
4930431F12Rik	0	0	0	0	1	0
4930432J09Rik	0	0	1	1	1	2
4930448K20Rik	0	0	0	0	0	1
4930452B06Rik	133	115	115	108	77	148
4930453H23Rik	1	0	0	0	0	0
4930455B14Rik	0	1	0	0	0	0
4930461G14Rik	0	0	0	0	1	1
4930467E23Rik	0	0	0	0	0	1
4930467K11Rik	0	0	0	0	0	1
4930471C04Rik	0	2	1	3	1	6
4930471G03Rik	0	0	0	0	0	1
4930471M09Rik	0	0	0	1	0	0
4930478L05Rik	0	0	0	0	0	1
4930483J18Rik	0	2	0	2	4	1
4930483O08Rik	0	0	0	0	0	1
4930500J02Rik	0	0	0	0	2	0
4930503E24Rik	1	0	0	3	2	0
4930509E16Rik	3	0	1	0	0	1
4930512B01Rik	0	0	0	0	1	0
4930513D17Rik	1	0	0	0	0	0
4930519G04Rik	0	1	0	0	0	0
4930522O17Rik	7	6	3	4	12	15
4930523C07Rik	1156	738	786	1042	708	1877
4930527F14Rik	0	0	1	0	0	0
4930529C04Rik	0	0	0	0	0	1
4930540M03Rik	0	0	0	0	1	1
4930542C21Rik	0	1	0	0	0	0
4930557A04Rik	0	1	0	0	0	0
4930558K02Rik	0	0	0	0	0	2
4930562C15Rik	0	0	0	0	0	1
4930565N06Rik	0	0	0	1	0	1

4930572O03Rik	0	0	0	0	0	1
4930579K19Rik	8	2	6	2	2	5
4930590J08Rik	12	2	3	5	9	15
4930593C16Rik	0	0	0	0	0	1
4930596I21Rik	4	2	3	0	3	1
4931406B18Rik	0	0	1	0	0	0
4931406C07Rik	406	376	296	433	369	694
4931412M21	0	1	0	0	0	0
4931428L18Rik	0	0	0	1	0	0
4931429I11Rik	0	0	0	0	1	1
4931431B13Rik	2	1	1	5	3	2
4932411E22Rik	0	0	1	0	0	0
4932412D23Rik	0	0	0	1	0	0
4932416H05Rik	97	64	66	100	81	133
4933402E13Rik	0	0	0	1	0	0
4933406G16Rik	0	0	0	0	0	1
4933407E24Rik	5	1	5	4	7	12
4933411G06Rik	0	0	0	0	1	0
4933412O06Rik	0	0	0	0	0	1
4933416E03Rik	0	0	0	0	0	1
4933417A18Rik	0	0	0	1	0	0
4933422H20Rik	2	2	1	0	1	1
4933428G20Rik	0	0	0	1	0	0
4933431E20Rik	239	175	131	171	118	243
4933433C11Rik	0	0	0	1	0	0
4933433H22Rik	1	0	0	1	1	3
4933434E20Rik	2468	1454	1366	1699	1228	2271
4933436I01Rik	0	0	1	0	0	0
4933439C10Rik	92	57	48	69	80	143
4933440J02Rik	1	2	1	1	4	5
5031410I06Rik	0	0	0	0	1	0
5031414D18Rik	97	53	78	57	61	80
5031425E22Rik	558	345	231	425	372	675
5031439G07Rik	1690	1067	914	1328	906	1254
5330411J11Rik	0	0	0	0	0	1
5330417C22Rik	3	3	1	5	2	7
5430416N02Rik	30	21	24	14	16	31
5430421F17Rik	2	1	1	0	0	1
5430421N21Rik	0	0	0	0	1	0
5430437J10Rik	1	0	0	0	0	0
5430440P10Rik	0	0	0	1	1	0
5730409E04Rik	735	449	395	689	444	841
5730422E09Rik	3	0	1	4	1	5
5830417I10Rik	1196	801	679	940	615	1089
5930438M14Rik	0	0	0	1	0	0

6030469F06Rik	0	0	0	0	1	0
6030498E09Rik	0	1	0	0	0	0
6330403K07Rik	1	6	2	11	2	9
6330409D20Rik	1	0	0	0	0	0
6330416G13Rik	591	442	359	485	333	531
6330549D23Rik	10	7	10	4	4	10
6430411K18Rik	1	0	0	2	0	2
7420461P10Rik	0	0	0	0	0	1
8030462N17Rik	1184	862	773	1115	725	861
8430419L09Rik	459	232	216	323	204	336
8430436N08Rik	0	0	0	1	0	1
9030404E10Rik	0	0	1	0	0	0
9030612E09Rik	131	69	58	112	88	139
9030624G23Rik	13	15	9	23	17	23
9130015A21Rik	0	1	0	0	1	3
9130024F11Rik	0	0	0	0	1	1
9130227L01Rik	0	0	0	0	0	1
9130409I23Rik	1	0	0	0	0	0
9230102K24Rik	0	0	0	0	0	1
9230104L09Rik	0	0	0	0	0	1
9230105E05Rik	0	0	0	0	0	1
9330111N05Rik	4	3	4	0	2	4
9330162B11Rik	0	1	0	0	0	0
9330179D12Rik	25	14	17	24	19	41
9430015G10Rik	202	145	85	118	83	190
9430037G07Rik	88	48	42	75	68	105
9430038I01Rik	155	99	96	138	108	224
9430069I07Rik	0	0	0	1	0	1
9530002B09Rik	0	0	0	0	0	1
9530036O11Rik	0	0	0	0	0	1
9530052E02Rik	4	1	2	0	2	1
9930012K11Rik	18	11	10	22	17	24
9930014A18Rik	95	58	45	81	74	127
9930111J21Rik2	0	1	0	2	0	2
A1cf	14	38	27	39	55	42
A230046K03Rik	1595	1090	879	1326	813	1407
A230065H16Rik	1	0	0	0	0	0
A230070E04Rik	3	1	0	0	1	0
A230072C01Rik	215	120	102	140	103	166
A230103J11Rik	10	4	5	5	3	4
A330035P11Rik	23	21	10	14	11	18
A330040F15Rik	0	0	1	0	0	0
A330102I10Rik	22	22	18	21	13	8
A630020A06	1	0	0	0	0	0
A630066F11Rik	33	18	28	27	28	66

A630075F10Rik	0	0	0	0	0	1
A630095N17Rik	0	0	0	1	1	0
A730020M07Rik	115	76	76	123	89	149
A930001A20Rik	0	0	1	0	0	0
A930001C03Rik	6	3	5	3	2	3
A930019D19Rik	1	0	0	0	0	0
AA536875	0	0	0	1	0	0
Aadac	0	0	0	1	0	0
Aamdc	1323	783	807	1211	868	1641
Aars	3051	2009	1919	2588	1705	2838
Abat	1131	962	942	1124	699	1119
Abca16	0	0	0	0	0	2
Abca2	3835	2492	1869	2990	2039	2848
Abca4	336	125	70	271	239	187
Abcb10	2362	1524	1351	1946	1208	2147
Abcb9	532	324	290	407	242	478
Abcg4	17	4	4	14	7	19
Abcg8	0	1	0	0	0	0
Abhd17c	1273	675	726	989	568	1097
Abhd4	2540	1440	1160	2168	1522	2502
Abtb1	761	451	355	610	439	943
Abtb2	1407	901	843	1301	667	948
Acacb	21056	13468	10956	15531	10710	17233
Accs	219	146	126	181	173	325
Ace2	64	69	64	97	57	160
Ace3	0	0	0	0	0	1
Acot10	0	0	2	2	1	3
Acot11	1963	1323	1042	1783	870	1682
Acot3	23	11	29	17	9	24
Acp6	1212	669	577	800	579	1026
Acr	23	26	14	16	32	55
Acsl3	438	291	226	313	215	380
Acsm3	2	3	4	2	1	1
Acsm5	111	43	85	59	152	148
Actb	11275	7326	6747	11017	6353	15316
Actbl2	0	0	0	0	0	1
Actg2	22	15	31	53	18	49
Actn1	1761	1059	822	1782	998	1777
Actr3b	1819	1084	1019	1651	1211	1911
Acvrl1	1682	953	701	1588	858	1783
Adad1	0	0	1	0	0	0
Adad2	0	0	1	0	0	0
Adal	648	412	346	486	330	559
Adam11	736	438	390	492	380	627
Adamts6	138	137	94	146	138	233

Adamtsl1	119	79	73	147	105	131
Adamtsl5	4238	2704	2096	3195	2092	3706
Adap2	472	307	263	444	261	669
Adar	1219	758	575	1032	710	1463
Adck1	1020	638	659	800	540	938
Adcy10	0	3	1	0	1	0
Adh6a	0	0	0	1	0	0
Adora1	850	711	432	988	534	1148
Adra2a	0	0	0	0	0	1
Adrb1	878	555	497	819	363	638
AF357426	0	1	0	0	0	0
AF529169	0	0	0	0	2	0
Afmid	423	325	204	451	299	463
Afp	0	0	0	0	0	1
Agap2	518	313	195	445	317	585
Agbl2	142	156	107	154	53	189
Ago4	1159	693	637	866	591	942
Agpat1	2903	1821	1458	2257	1453	2521
Agr2	0	1	0	0	0	0
Agtr1a	2420	1769	1325	1989	1084	2507
Agtr1b	0	0	0	0	0	1
Agtr2	0	0	0	1	0	0
Agxt	0	0	0	0	1	0
Agxt2	0	0	0	0	0	2
Ahcy	115	40	79	68	53	65
Ahrr	133	75	57	103	142	110
AI314278	1	0	0	1	1	2
AI593442	28	12	36	32	42	54
AI597479	1782	1143	1124	1467	989	1626
AI837181	984	619	439	924	555	1010
AI848285	0	0	0	2	0	0
Aif1	40	29	23	35	32	69
Aifm1	4948	3106	2837	4540	3515	5463
Aig1	505	365	336	429	293	444
Aipl1	0	0	0	0	0	1
Aire	0	0	1	0	0	0
AK010878	216	102	131	145	94	184
Ak4	1667	909	1001	1728	1096	1580
Akap14	0	0	0	0	0	1
Akap6	11632	8924	6696	9619	7349	8197
Akip1	297	171	190	305	183	406
Akr1c12	1	0	1	4	2	0
Akr1c13	24	26	18	9	6	36
Akr1cl	0	0	2	4	0	5
Akt2	4793	3177	2185	3569	2375	3663

Alad	1207	615	541	962	751	1251
Aldh1b1	1531	981	740	1332	508	1271
Aldh5a1	3111	1973	1874	2580	1623	2760
Aldh8a1	0	1	0	3	0	1
Alk	0	0	0	0	0	2
Alkbh2	46	34	30	37	20	40
Allc	0	1	0	0	0	0
Alms1-ps2	4	1	0	1	0	0
Alox12	265	187	128	216	234	277
Alox15	1	0	1	1	5	2
Alox8	14	19	11	47	15	14
Alpk1	520	204	167	453	397	328
Alpk2	8714	7083	5569	8303	5350	6031
Als2cl	1681	934	684	1223	1009	1820
Als2cr11	0	0	0	0	0	1
Als2cr12	0	0	0	0	0	1
Alx1	0	0	1	0	0	0
Ambp	0	0	0	0	0	1
Amelx	0	0	0	0	1	0
Amer2	0	1	0	0	0	0
Amigo1	1557	1076	957	1488	738	1282
Amigo2	324	208	231	277	275	494
Ammecr1	444	421	285	462	256	333
Amot	604	434	519	548	611	911
Angpt2	195	115	100	176	159	186
Angptl2	4979	3432	2051	3626	2167	4496
Angptl4	232	54	133	67	109	688
Ank1	14975	10309	9127	12841	7428	14548
Ank2	7574	5673	4830	6973	4254	6942
Ankrd13b	676	439	308	471	363	485
Ankrd16	541	296	241	380	327	656
Ankrd2	2	5	6	5	8	15
Ankrd22	0	0	0	2	0	0
Ankrd29	281	183	206	283	206	389
Ankrd32	2153	1434	1269	1650	1141	2057
Ankrd33	0	0	0	1	0	0
Ankrd42	28	28	20	19	12	32
Ankrd45	198	163	234	230	96	222
Ankrd52	3236	2321	1768	2685	1842	2115
Ankrd54	224	120	93	183	127	265
Ankrd55	33	11	7	9	11	15
Ankrd60	0	0	1	0	0	0
Ankrd61	2	2	1	2	2	9
Ankrd9	1295	714	580	1197	793	1198
Anks4b	0	0	0	0	1	0

Ankub1	1	0	0	0	0	0
Ano5	48	18	73	22	28	67
Antxr1	819	658	441	816	311	785
Antxr2	9634	6989	5406	8334	5068	8022
Anxa5	3368	2469	2181	3437	2319	4747
Aox1	2223	1300	1083	1488	1603	2938
Ap1g2	247	149	98	206	143	280
Ap1m2	0	0	0	0	2	0
Ap2s1	1202	626	571	1094	589	1331
Ap3m1	1546	1073	941	1205	874	1452
Ap3m2	319	215	185	294	226	453
Apba1	475	327	242	397	194	385
Apcdd1	131	84	74	146	73	189
Apitd1	17	15	11	27	18	26
Apol7a	0	0	0	0	1	0
Apol7b	0	0	0	1	0	0
Appl1	2327	1780	1448	1887	1343	2324
Appl2	4520	2985	2048	2766	2316	4058
Ar	56	39	28	52	19	35
Arap2	860	604	631	895	650	1196
Arc	115	67	37	80	39	66
Arcn1	5557	3950	3559	4744	3234	4874
Arf3	1215	772	561	1229	657	1360
Arf5	1635	905	784	1405	988	1706
Arg1	0	1	0	0	0	6
Arhgap20os	0	0	0	0	0	1
Arhgap29	6644	4658	3918	5654	4521	9523
Arhgap33	52	24	10	31	33	59
Arhgap42	664	491	465	605	379	642
Arhgap44	1685	967	791	1260	874	1074
Arhgap5	3748	2737	2126	3304	2167	2865
Arhgap8	28	14	15	26	19	48
Arhgef10	1212	796	650	1062	804	1523
Arhgef19	1645	851	759	1326	580	1099
Arhgef2	2479	1689	1287	2430	1457	2849
Arhgef25	685	447	369	625	426	831
Arhgef3	2067	1541	1119	2096	1519	2367
Arhgef6	5106	3684	2772	4137	2842	4392
Arid3b	174	130	87	122	94	120
Arl1	1917	1388	1254	1626	1151	1659
Arl4d	586	363	284	670	337	615
Arl5a	2807	1960	1634	2608	1379	2361
Arl8a	1063	732	615	1033	670	1301
Armc1	2618	1740	1718	2388	1402	2401
Armc12	1	0	0	0	0	0

Armc4	0	0	0	1	0	0
Arpc1b	1340	713	696	1245	700	1679
Arpc4	1092	628	639	1020	643	1388
Arsa	511	349	302	584	276	690
Arsj	41	25	15	20	13	31
Art5	762	329	276	558	405	715
Arx	0	0	0	0	1	0
Asb16	10	9	5	15	7	19
Ascl4	0	1	0	0	0	0
Asic4	1	0	0	0	0	0
Asl	208	105	95	206	99	238
Asmt	0	0	0	0	1	0
Asphd1	1	0	0	1	0	3
Aspn	2251	1602	1431	2348	1339	3213
Aspscr1	993	581	437	843	545	1082
Astn1	92	64	39	83	56	144
Atad1	6387	4344	3706	5329	3511	5523
Atad3a	1863	1293	970	1352	1074	1575
Atad5	135	87	72	114	90	211
Atat1	315	187	150	284	211	315
Atf6	9618	7127	5894	8693	5339	7023
Atf7	452	341	218	352	252	361
Atg101	503	304	268	487	274	647
Atg2b	1906	1199	953	1570	970	1428
Athl1	214	117	85	176	121	253
Atl1	220	130	136	193	176	289
Atl2	3293	2122	2066	2758	1803	2937
Atox1	426	224	227	367	222	571
Atp10d	634	363	322	580	392	728
Atp11a	8919	6775	4915	7996	4943	6706
Atp1a1	44536	30112	25044	39197	22323	32010
Atp1a3	13	5	14	10	5	9
Atp5g2	44	14	8	13	13	20
Atp6v0a1	4344	3114	2497	3588	2431	3823
Atp6v1e2	0	0	0	0	0	1
Atp8b2	1183	729	529	1115	602	1344
Atxn1l	1500	972	783	1554	873	1559
Atxn2	3793	3161	2351	3533	2279	3002
Atxn7	2157	1497	1184	1643	1208	1853
AU018091	0	0	1	0	0	0
AU023762	0	0	0	1	0	1
AU040320	2349	1639	1281	1860	1261	1991
AV039307	9	2	8	5	1	5
AW146154	289	192	164	229	192	442
AW209491	626	429	439	518	330	620

AW495222	0	0	0	0	0	1
AW554918	384	257	204	317	268	509
Awat2	1	6	3	2	1	2
Axin2	313	210	174	250	181	223
B130006D01Rik	9	3	2	6	12	10
B230206H07Rik	0	0	0	0	1	0
B230209E15Rik	0	0	0	0	1	0
B330016D10Rik	232	117	108	166	158	275
B3galnt2	1932	1132	1169	1615	1013	1513
B4galnt2	0	0	0	1	0	0
B830017H08Rik	0	0	0	0	1	0
Bach1	2616	1626	1256	1869	1359	2164
Bahd1	874	493	416	828	510	842
Bai3	0	1	0	1	0	4
Banp	664	424	298	589	266	334
Baz1b	6130	4403	3232	4910	3235	5205
Baz2b	2089	1549	1289	1701	1674	2968
BB014433	3	2	1	4	3	5
BB031773	1	0	0	0	0	0
Bbs4	245	190	134	210	178	389
BC005624	820	706	471	619	510	860
BC016579	1	0	0	0	0	0
BC017643	461	242	241	457	238	541
BC021767	0	6	3	0	1	3
BC025920	160	113	107	108	97	135
BC031361	105	74	67	82	53	82
BC035044	15	10	9	9	8	6
BC048546	2	1	2	1	0	1
BC051019	0	0	0	0	1	1
BC055402	0	0	0	0	0	2
BC061194	1	1	1	4	5	1
BC064078	9	4	9	14	11	13
Bcap29	2771	1820	1629	2084	1516	2709
Bcas3os1	0	1	1	1	2	3
Bcat1	37	24	21	40	18	67
Bccip	2376	1609	1405	1855	1243	2461
Bcl2a1b	1	6	3	8	5	7
Bcl2a1d	0	0	1	0	0	0
Bcl9	1629	1202	820	1439	1158	1825
Bdkrb2	1	2	0	3	6	1
Becn2	0	0	0	1	0	0
Bend4	249	129	164	159	141	186
Bet1l	420	214	240	399	232	492
Bex6	0	0	0	1	0	0
Bglap	0	0	0	1	0	1

Bhlhe40	1946	1377	873	2207	1563	1553
Bicd1	379	326	221	313	216	391
Bin2	37	21	13	24	13	20
Bloc1s1	1483	1003	891	1428	967	1820
Bloc1s3	136	84	72	145	94	159
Blzf1	1554	1223	1055	1359	927	1656
Bmf	350	198	189	276	220	460
Bmp7	54	22	31	52	55	53
Bmp8a	0	0	0	0	0	1
Bmpr1b	5	12	4	3	4	6
Bms1	1883	1507	1315	1641	1221	1943
Bnc2	50	48	27	51	12	41
Boc	320	177	148	353	164	359
Bok	74	45	30	81	36	97
Bop1	689	397	307	507	274	533
Bpifa1	0	0	0	0	1	0
Bpifa6	0	0	0	0	0	1
Bpifb5	0	0	0	0	0	1
Bpifc	0	0	0	0	1	0
Bpnt1	1100	694	789	978	583	1070
Bri3bp	1943	1152	1046	1408	1003	1708
Brms1l	1167	717	653	876	592	1033
Brpf3	866	614	423	721	594	1060
Brs3	0	0	0	1	0	0
Bsnd	1	0	0	0	0	0
Bsx	1	0	0	0	0	0
Btbd17	0	0	0	0	0	2
Btk	57	30	25	36	14	49
Btn1a1	0	0	1	0	0	0
Btnl1	0	0	0	1	0	0
Btnl9	1246	603	644	826	913	1440
Bub1	18	11	9	15	4	61
Bub1b	71	39	24	59	30	119
Bves	2677	1734	1482	2193	1478	2168
Bysl	1030	679	579	846	540	872
C130026I21Rik	1	1	1	4	2	3
C130026L21Rik	0	1	0	2	2	0
C130036L24Rik	17	9	5	5	2	13
C130071C03Rik	0	0	0	0	1	0
C130080G10Rik	6118	4421	4740	6096	6610	7228
C130083M11Rik	0	0	0	0	1	1
C1qtnf2	104	70	72	123	56	176
C1qtnf7	312	272	175	287	143	290
C230004F18Rik	0	0	0	1	0	0
C230024C17Rik	2	1	1	0	1	0

C2cd4b	13	15	9	5	10	11
C2cd4d	0	1	1	1	2	4
C330022C24Rik	0	0	0	0	0	1
C330024C12Rik	0	1	0	0	0	0
C330046G13Rik	0	0	0	0	1	0
C430002N11Rik	0	0	0	1	1	0
C430049B03Rik	29	20	24	24	32	64
C530005A16Rik	145	104	87	105	84	88
C5ar1	156	104	86	120	69	130
C5ar2	29	42	34	32	27	20
C630028M04Rik	0	0	0	0	0	1
C77080	238	141	111	252	135	239
C8a	0	1	0	0	0	0
Cab39l	1503	1002	815	1133	792	1318
Cabp4	0	0	0	0	0	1
Cabp5	0	0	1	0	0	0
Cacna1f	1	2	1	1	0	0
Cacna1i	4	3	0	0	1	2
Cacng8	1	0	0	0	0	0
Cacul1	2011	1335	1235	1699	1104	1773
Cadm1	153	121	84	151	112	223
Cadm2	68	50	45	69	55	112
Cadm3	144	68	64	85	45	110
Cadps	1	0	0	0	0	0
Calcb	0	0	0	0	1	0
Cald1	3294	2383	2313	3623	2224	4541
Calml4	8	8	0	10	3	21
Caly	1	2	4	0	1	2
Camk1	1924	1236	1021	1717	1151	2316
Cap1	2117	1359	1210	2157	1170	2713
Capn13	0	1	0	0	0	0
Capn8	0	0	1	0	0	0
Capn9	4	4	2	1	2	3
Capns2	0	0	0	0	0	1
Caprin1	9099	6679	5267	7841	4973	7927
Capsl	3	0	1	0	0	1
Car12	0	0	0	1	0	0
Car2	307	230	217	241	186	316
Car3	181	297	107	233	100	103
Car5a	0	0	0	0	0	1
Carhsp1	1095	608	630	1118	571	1279
Casc5	21	10	4	15	5	48
Casd1	2430	1635	1239	1933	1284	2087
Caskin1	21	19	5	13	8	13
Caskin2	3704	2209	1483	3539	1949	3626

Casp12	563	344	367	557	373	723
Casp3	275	157	146	267	151	329
Casp8	376	227	292	412	247	616
Cass4	18	16	15	15	6	19
Catsper4	7	3	2	7	4	12
Cbfa2t3	1816	1118	835	1794	1079	1762
Cbln2	0	1	0	0	0	0
Cbln3	11	8	4	6	4	6
Cbx2	300	159	164	234	132	232
Cc2d2a	893	689	526	788	702	1137
Ccdc106	2	1	4	8	2	6
Ccdc113	1	0	0	0	0	0
Ccdc117	1757	1035	915	1379	859	1498
Ccdc122	66	43	49	62	53	110
Ccdc132	1348	915	768	1075	720	1300
Ccdc152	3	1	3	1	0	2
Ccdc155	0	0	1	0	0	0
Ccdc158	2	6	1	6	2	11
Ccdc160	23	10	14	21	13	43
Ccdc162	2	1	3	1	0	1
Ccdc167	692	484	395	472	421	587
Ccdc17	87	59	54	77	71	127
Ccdc176	175	136	118	137	93	190
Ccdc18	5	6	10	12	8	17
Ccdc183	0	0	1	0	0	0
Ccdc184	43	22	22	63	23	40
Ccdc25	1195	916	837	1056	646	1251
Ccdc30	2	4	2	5	5	7
Ccdc34os	0	0	0	0	0	1
Ccdc36	1	0	0	0	0	0
Ccdc43	1095	670	691	878	600	974
Ccdc60	0	0	0	0	0	1
Ccdc64b	0	0	1	0	0	0
Ccdc67	6	14	20	9	28	40
Ccdc69	458	269	206	398	158	327
Ccdc73	5	3	5	7	10	9
Ccdc85b	893	452	372	720	488	997
Ccdc87	2	0	0	1	2	3
Ccdc9	792	481	426	649	544	912
Ccdc92	46	40	16	31	15	37
Ccl12	28	14	9	34	13	29
Ccl2	41	20	16	28	7	28
Ccna1	2	1	0	4	2	2
Ccne1	33	25	12	26	32	48
Ccnh	1484	1176	922	1270	863	1502

Ccnyl1	552	333	302	479	346	660
Ccp110	390	232	164	324	248	446
Ccr5	258	175	122	227	68	213
Cct2	6134	4267	3896	4994	3531	5625
Cct8l1	11	7	3	6	4	4
Cd164l2	1	1	4	1	0	2
Cd200r2	0	0	0	1	1	0
Cd209f	10	7	2	2	4	5
Cd2ap	2059	1503	1423	2093	1453	3035
Cd300e	1	0	1	2	2	3
Cd300ld	146	96	121	113	50	182
Cd3d	6	1	0	7	5	3
Cd46	68	26	24	40	47	98
Cd47	2087	1283	1211	2061	1281	2328
Cd5l	2	0	1	2	4	3
Cd72	37	30	17	40	22	71
Cd83	161	92	98	102	64	157
Cd8b1	4	7	1	13	4	9
Cd9	1442	1072	866	1449	1020	1781
Cda	9	11	7	12	11	21
Cdadc1	2782	1875	1626	2165	1373	2884
Cdc37l1	5634	3261	3414	4494	2958	4850
Cdc42bpa	3444	2321	2032	3431	2267	4070
Cdc42ep5	67	41	23	72	34	91
Cdc42se2	1836	951	959	1636	1010	2028
Cdc5l	1929	1647	1335	1807	1232	1849
Cdc73	1171	871	710	1044	633	1010
Cdca8	24	20	13	19	13	64
Cdh1	1	2	2	0	1	1
Cdh11	227	240	159	177	136	290
Cdhr3	13	18	8	26	8	31
Cdk2	546	274	234	413	324	627
Cdk20	94	63	46	103	78	107
Cdk7	1415	921	823	1069	746	1400
Cdk8	900	696	560	689	541	938
Cdk9	831	509	379	688	479	1001
Cdkl1	533	366	375	350	349	491
Cdkl5	124	112	76	92	100	58
Cdkn1c	975	858	592	902	763	1689
Cdkn2aipnl	533	404	333	547	370	692
Cdkn2c	413	305	214	425	261	550
Cds1	253	133	164	175	211	353
Ceacam10	0	0	0	0	1	0
Cebpe	1	0	0	0	0	0
Cel	0	0	0	0	1	0

Cela2a	0	0	1	0	0	0
Cenpk	5	10	7	11	5	26
Cenpq	446	290	302	350	211	446
Cenpt	93	65	47	91	56	138
Cenpu	33	20	19	24	8	28
Cep104	1669	1154	969	1446	982	1273
Cep131	386	248	156	316	264	450
Cep350	3623	2353	1904	2858	2041	2764
Cep63	2138	1534	1330	1643	1299	2160
Cep68	3325	2218	2117	2962	1841	2585
Cep83	1211	1018	761	934	855	1144
Cep85	2390	1425	1303	1757	1368	1817
Cerk	2123	1504	1281	2197	1569	2290
Cers4	1859	1394	1015	1828	1282	2128
Ces2b	6	3	1	2	1	2
Cfl1	2651	1830	1628	2605	1619	3752
Cfp	357	207	133	219	120	305
Chadl	25	12	14	33	13	34
Chaf1a	128	84	71	109	91	215
Chaf1b	24	15	17	17	20	56
Chat	0	0	0	0	0	1
Chchd4	1102	673	758	854	620	1087
Chd3os	28	18	15	15	15	20
Chd5	51	20	18	19	17	29
Chd6	7695	5357	3582	5354	4440	5835
Chd9	2481	1772	1387	1925	1379	2405
Chia1	3	0	1	1	4	7
Chil6	1	0	0	0	0	0
Chit1	0	0	0	1	0	0
Chrd	130	64	70	135	65	176
Chrm1	1	0	0	1	1	3
Chrna1	6	0	7	0	3	2
Chrna4	0	0	0	0	1	0
Chrna5	0	1	0	0	0	0
Chrna7	1	0	3	0	0	1
Chrna9	1	0	0	0	0	0
Chrng	0	0	1	0	0	0
Ciapin1	1773	1158	1144	1594	954	1587
Cib3	10	4	8	4	7	3
Cidea	866	694	597	842	642	1292
Cisd3	1522	1006	917	1546	932	1915
Ckap4	1386	1092	1036	1448	666	1491
Ckmt1	6	2	4	7	9	9
Cks2	1	2	0	6	0	4
Clasp1	15832	13426	10790	15837	9586	13762

Clca4	0	0	0	0	0	1
Clca6	0	0	0	0	2	0
Clcn5	70	50	23	44	14	54
Cldn10	0	0	0	1	0	1
Cldn15	114	33	75	80	82	161
Cldn22	1	0	0	0	0	0
Cldn3	0	0	0	0	0	1
Cldn4	0	0	0	0	1	0
Cldn7	2	1	2	0	0	2
Cldn8	1	0	0	0	0	0
Cldn9	1	0	0	0	0	0
Clec10a	51	38	31	48	37	102
Clec12b	81	42	44	47	62	130
Clec16a	3260	2423	1895	2855	1805	2581
Clec3a	2	12	8	25	16	5
Clec3b	1545	1114	782	1401	575	1360
Clec4b2	0	0	0	0	0	1
Clec7a	96	94	82	131	76	194
Clic1	1303	878	636	1227	676	1702
Clic4	25612	17310	14811	22322	12332	22523
Clint1	3115	2281	1889	2495	1899	2902
Clip2	457	355	292	429	361	658
Clmn	337	180	117	215	118	249
Clmp	121	99	89	88	68	141
Clspn	36	21	11	29	19	57
Cma2	0	0	0	0	0	1
Cmbl	1139	733	674	963	746	1473
Cmip	1382	937	632	1328	795	1517
Cml5	0	0	0	0	1	0
Cmtm3	437	281	239	428	243	661
Cmtm6	1456	886	877	1406	867	1744
Cmtm8	385	233	180	339	239	413
Cnbd2	27	34	24	41	37	53
Cndp1	0	0	0	1	0	0
Cnga1	0	0	0	0	1	0
Cnih3	0	0	0	1	0	0
Cnn3	2116	1424	1251	2065	1200	3089
Cnot4	1773	1371	1073	1530	1061	1644
Cnot6	2238	1412	1131	2095	1283	2688
Cnp	360	247	200	351	223	463
Cnppd1	1104	705	488	1003	644	1262
Cnpy3	567	366	271	478	387	635
Cnr2	32	16	14	34	13	54
Cntd1	33	17	11	10	9	27
Cntfr	223	227	135	211	110	218

Cntnap2	0	0	0	0	0	2
Coa4	313	183	183	197	136	306
Cog5	1507	1010	776	1381	731	1096
Cog8	616	420	341	485	312	591
Col14a1	1341	885	378	1163	305	792
Col17a1	4	1	3	3	4	10
Col20a1	122	66	81	64	61	108
Col27a1	427	341	203	312	209	421
Col3a1	6260	3727	3520	5516	2327	13146
Col6a3	1895	1149	854	1438	459	1750
Col6a4	0	0	0	1	0	1
Col8a2	22	21	10	40	15	30
Col9a1	0	0	0	1	0	0
Comtd1	97	62	54	54	46	89
Coq10b	2445	1661	1586	2184	1219	2423
Coro2b	708	363	263	561	370	779
Cox11	1075	652	625	864	541	975
Cox6a2	18223	11278	9993	16566	16027	17945
Cox7b2	4	13	14	6	2	14
Cpa4	1	0	0	0	0	0
Cpeb3	12106	6541	5800	6838	5991	10259
Cplx2	215	161	131	198	159	316
Cpm	659	419	367	632	443	786
Cpn2	4	1	0	4	3	5
Cpne1	1491	861	659	1225	813	1625
Cpne6	3	1	1	3	1	7
Cpne7	40	18	15	44	21	50
Cpox	2386	1365	1263	2092	1668	2234
Cpsf2	1780	1223	1049	1569	929	1658
Cpsf3	2307	1554	1360	1843	1125	2262
Cpsf4l	0	1	0	0	3	1
Cpxm2	634	430	990	241	690	733
Creb5	202	134	89	125	91	167
Crisp3	0	0	0	0	1	0
Crnde	134	115	71	144	91	208
Crx	0	0	0	0	0	1
Crybb2	0	0	0	1	0	0
Crybb3	51	19	7	35	33	45
Cse1l	3397	2338	2003	3042	1840	2758
Csf2	0	1	0	0	0	0
Csf3	1	0	0	0	0	0
Csmd3	1	0	0	0	0	0
Csnka2ip	1	0	0	0	0	0
Cspg4	1333	868	675	1577	739	1467
Csrp2bp	1685	1205	886	1399	966	1365

Cst8	0	0	0	1	0	0
Cstb	251	203	146	236	163	405
Ctcfl	14	2	9	5	3	8
Ctcflos	4	8	2	4	1	2
Ctdp1	516	347	256	520	328	556
Ctf1	151	58	58	113	127	122
Ctgf	5039	2966	2091	4408	2527	6617
Ctnna2	0	0	0	1	0	0
Ctr9	1311	974	861	1067	789	1359
Ctrcos	0	0	0	0	1	0
Ctrl	17	16	6	4	11	11
Ctsg	0	0	0	0	0	1
Cttnbp2	83	61	20	55	77	99
Cul1	5120	3599	2880	4045	2959	4658
Cul2	2977	2009	1902	2534	1663	2721
Cul5	4352	2846	2627	3525	2389	3756
Cul7	1131	741	514	1048	750	1171
Cuta	837	542	446	742	485	1097
Cux2	560	350	259	439	256	402
Cxcl11	0	0	1	0	0	0
Cxxc4	145	112	134	155	145	281
Cyb5rl	311	168	165	195	156	218
Cyba	488	265	258	419	296	564
Cyct	13	3	4	0	2	8
Cyp11a1	1	1	3	1	0	1
Cyp11b1	0	0	0	0	0	1
Cyp2d10	0	0	0	0	1	1
Cyp2d26	0	1	0	0	0	0
Cyp2j13	0	0	0	0	0	1
Cyp2j6	436	294	316	414	328	655
Cyp2t4	0	0	0	0	1	1
Cyp3a13	1	1	4	4	4	5
Cyp3a57	0	0	0	0	1	0
Cyp4f15	4	2	2	0	4	16
Cyp4f16	178	96	105	135	117	272
Cysltr1	152	119	81	156	99	256
Cytip	54	37	27	37	11	51
D030025E07Rik	0	0	0	0	0	2
D030047H15Rik	2	0	1	3	2	3
D130020L05Rik	77	35	30	52	67	87
D16Ertd519e	0	0	0	0	0	2
D230030E09Rik	0	1	0	0	0	0
D3Bwg0562e	13	4	8	9	4	3
D430019H16Rik	25	26	18	17	10	28
D630024D03Rik	9	10	11	4	9	9

D730001G18Rik	0	1	0	0	0	0
D730005E14Rik	37	19	10	27	35	38
D7Ertd715e	763	416	347	410	472	1236
D830005E20Rik	925	804	521	692	586	907
D930020B18Rik	0	0	0	0	0	1
Daam1	5907	4938	4678	4980	4477	5183
Dab2ip	3963	2392	1756	3880	2225	4120
Dact3	415	292	224	477	342	393
Dagla	185	110	87	135	81	131
Dand5	481	330	287	376	276	369
Dao	0	1	0	0	0	0
Dapl1	0	0	0	0	1	0
Daw1	0	0	0	0	0	2
Dazap2	9329	5807	5017	8306	6059	10778
Dbf4	101	61	56	90	59	156
Dcaf12l1	245	144	105	175	87	181
Dcaf12l2	0	0	0	0	0	1
Dcdc2a	1	0	0	0	0	0
Dcdc2c	1	0	0	0	0	0
Dchs1	838	488	454	906	454	985
Dclk1	217	147	104	146	76	228
Dclk3	22	20	20	47	16	43
Dclre1a	627	473	402	483	387	603
Dcp1b	151	81	74	111	102	200
Dcpp2	1	0	0	0	0	0
Dctn6	2256	1574	1368	2071	1525	3123
Dctpp1	343	261	199	244	155	378
Dcun1d1	1460	919	967	1234	784	1402
Ddhd2	1221	814	721	909	644	1198
Ddo	1999	1495	1441	1973	1193	1807
Ddr2	2368	1529	1448	2017	1054	2284
Ddx1	4989	3553	3009	3941	2856	4918
Ddx25	0	0	0	1	0	0
Ddx60	457	219	180	276	248	663
Dear1	1	0	0	0	0	0
Dennd2a	587	365	274	466	236	477
Dennd2c	260	168	118	242	153	304
Desi1	1240	907	820	1128	629	1238
Desi2	1581	993	941	1421	736	1356
Det1	514	292	299	339	276	407
Dfna5	10	10	4	11	11	19
Dgkh	153	75	58	115	85	175
Dhrs9	0	1	0	2	0	2
Dhx29	1276	863	747	1002	716	1095
Dhx58	87	79	37	92	48	170

Diap2	1339	1009	742	1240	962	1615
Dido1	3622	2601	1818	2909	1954	3241
Dio3	1	2	1	4	2	4
Dip2c	3870	2667	2013	3044	2166	2902
Dis3l	2288	1528	1233	1726	1226	2055
Dkkl1	1	0	0	0	0	0
Dlg2	89	88	91	129	95	173
Dlg3	544	411	293	406	267	530
Dlgap2	1	0	0	0	0	0
Dll3	0	0	0	0	1	0
Dlx2	0	0	0	1	0	0
Dlx3	0	0	0	0	1	0
Dlx4	0	0	0	0	0	1
Dmbt1	0	0	0	0	0	1
Dmkn	0	0	0	1	0	1
Dmrta1	0	0	0	0	1	1
Dmrta2	1	1	1	4	4	0
Dmrtc1a	0	5	3	3	0	1
Dmrtc1b	0	0	1	0	0	0
Dmxl2	2732	1578	1443	1817	1523	2092
Dnah10	14	6	6	3	7	5
Dnah8	135	10	86	28	53	45
Dnaja2	15240	11474	9870	12678	8905	15238
Dnajb1	832	422	436	622	315	679
Dnajb4	6410	4188	4447	5387	3610	5186
Dnajb5	4191	2754	2124	3595	1929	3242
Dnajb7	0	0	0	1	0	0
Dnajc11	2704	1750	1792	2162	1545	2329
Dnajc13	3341	2270	1971	2776	1968	2881
Dnajc18	2078	1473	1220	1908	1038	1875
Dnajc28	2916	2208	1933	2389	1833	2599
Dnase2b	0	0	0	1	0	0
Dner	1	0	0	2	0	2
Dnmt3a	3134	2068	1634	2445	1745	2669
Dnmt3aos	0	0	0	0	0	1
Dnph1	48	22	14	31	11	24
Dnttip2	1811	1671	1302	1524	1077	2135
Dock3	14	10	15	8	10	10
Dock4	973	578	547	681	692	1359
Doxl2	0	0	0	0	0	1
Dpagt1	861	522	425	843	510	902
Dph5	169	156	139	153	105	176
Dpm2	297	198	179	309	195	380
Dppa2	1	0	0	0	0	0
Dpt	3331	2458	2199	3195	1232	3598

DQ267100	0	1	0	0	0	0
Drosha	4012	2995	2250	3338	2243	3442
Dsc2	3788	2509	1787	2706	1854	3412
Dsc3	0	0	1	0	0	0
Dscam	0	0	0	1	0	0
Dsg2	8256	6314	4201	6083	4978	6744
Dsg4	0	0	0	0	1	0
Dstn	5988	4483	4217	6533	4791	7465
Dstyk	1789	1160	1003	1649	1186	2107
Dtx2	308	174	127	259	177	324
Duox1	27	8	12	15	1	19
Duoxa2	3	0	0	1	2	5
Dupd1	0	1	1	3	0	3
Dusp2	15	3	3	6	1	6
Dusp26	11	6	7	12	14	14
Dusp9	0	0	0	1	1	0
Dvl3	1322	1019	712	1057	798	959
Dxo	239	159	109	232	150	305
Dydc1	0	1	0	0	0	0
Dym	3963	2651	2304	3108	2249	3626
Dync1li1	2442	1712	1486	2149	1403	2082
Dynlt1a	19	10	12	18	12	33
E030019B06Rik	1	0	0	0	0	0
E130006D01Rik	8	4	3	11	1	21
E130018N17Rik	1	0	0	0	0	0
E130112N10Rik	12	11	7	13	13	22
E130309F12Rik	0	0	0	1	1	0
E130317F20Rik	65	46	30	50	62	91
E230025N22Rik	0	0	0	0	0	2
E2f1	31	18	11	28	12	54
E2f2	30	34	21	20	17	36
E2f4	860	564	442	656	401	779
E330013P04Rik	0	0	0	1	0	0
E430018J23Rik	125	64	55	114	73	148
Ebag9	894	694	573	750	546	880
Ebf2	730	632	416	690	361	721
Ebf3	1026	625	422	984	596	1042
Ebf4	38	25	8	31	26	47
Ecm2	2039	1544	1130	1953	1426	2500
Edc4	1734	1054	805	1588	1011	1726
Edil3	46	36	29	21	26	46
Edn2	0	0	0	1	0	0
Ednra	3605	2508	1880	3435	1652	2296
Eef1g	3002	2061	1787	2519	1563	2962
Eefsec	209	140	100	212	138	236

Efcab1	0	0	1	0	0	0
Efcab2	5743	4039	3357	4878	3394	4501
Efcab6	1	0	0	0	0	0
Efcab7	61	41	28	43	47	103
Efemp2	313	153	148	283	143	386
Efhc2	7	2	5	8	9	8
Efna4	7	3	1	3	8	9
Efr3a	4768	3145	2683	3902	2540	4072
Efs	80	54	42	75	26	57
Egln1	24241	14572	14498	23756	18688	25009
Egr2	12	6	13	22	7	26
Ehbp1l1	15152	10410	8831	12421	8399	13237
Ehd3	187	149	123	227	132	264
Ehmt2	2099	1296	1035	1755	1333	2369
Eif2b4	1668	1080	1007	1317	905	1635
Eif2s1	2157	1532	1359	1636	1222	2154
Eif3j1	858	711	699	699	573	981
Eif3j2	858	711	699	699	573	981
Eif4a2	15646	10457	9469	12418	8564	14993
Eif4e	5616	3913	3560	4717	3169	5239
Eif4e2	1894	1204	1209	1621	1004	1651
Eif4ebp2	1103	782	567	924	614	864
Eif4enif1	2358	1773	1482	2085	1412	2089
Eif5	9088	6244	5633	6912	5221	8523
Elf1	1381	922	712	1309	863	1540
Elf3	1	0	0	0	0	0
Eln	956	607	374	916	495	1076
Elp3	1818	1140	934	1430	901	1542
Emc9	811	426	446	710	436	1018
Eml5	531	306	300	302	253	547
Emr1	396	227	159	272	96	325
Endod1	674	362	332	620	358	782
Enho	87	39	60	90	67	120
Enkd1	55	39	31	61	44	82
Eno4	2	3	0	1	0	1
Enpp1	367	245	137	297	158	220
Enthd1	0	0	0	0	0	1
Entpd2	428	260	220	488	192	529
Entpd8	0	0	0	1	1	0
Epb4.2	3	7	10	4	3	6
Epdr1	1134	672	750	919	603	1018
Epha4	2279	1481	1100	1723	1301	1513
Epha5	0	0	0	0	0	1
Ephx3	54	39	39	59	60	73
Epn2	2202	1494	1102	1998	1449	2531

Eppk1	7	13	8	23	13	14
Eprs	8033	5937	4934	6333	4954	7461
Eps8l1	30	258	282	88	183	53
Eps8l2	198	117	88	181	115	222
Epsti1	112	63	40	58	29	92
Erbb2	493	254	183	448	242	473
Erbb4	126	78	63	86	62	66
Ercc1	824	428	367	657	451	950
Ercc5	1461	1151	915	1202	790	1578
Ercc6l2	1171	741	624	899	647	970
Erf	753	417	363	730	398	838
Eri1	835	618	465	737	417	776
Erlec1	2872	2276	1880	2402	1843	2936
Ermn	0	0	0	1	0	0
Ero1l	936	691	571	800	493	918
Erp44	2224	1671	1426	1975	1156	2196
Espnl	3	1	2	4	4	6
Esr2	3	2	0	1	0	1
Esrrg	3412	2363	1941	2917	1929	2652
Esyt1	1537	968	775	1505	801	1825
Etf1	7663	5273	5193	5915	4545	7026
Ets1	4875	2811	2590	4392	3018	5335
EU599041	0	0	1	0	0	0
Eva1a	133	75	79	92	81	248
Evc2	448	344	266	374	212	463
Evi2a-evi2b	123	151	98	149	63	131
Evi2b	110	131	84	126	51	112
Evpl	23	8	5	25	11	20
Evx1	0	1	0	0	0	0
Exd2	1008	615	561	825	498	866
Exo5	139	99	96	141	112	195
Exoc3	4316	3146	2720	3717	2472	4004
Exph5	1	1	1	1	5	2
F11	1	0	0	0	0	0
F2r	1985	1414	1285	2064	1290	2782
F2rl2	8	8	3	4	4	5
F630042J09Rik	1	0	0	0	0	0
F7	20	8	3	3	3	12
F730035M05Rik	1	1	0	1	2	3
F9	1	0	0	0	4	0
F930015N05Rik	17	19	7	9	3	21
Fabp12	3	1	2	0	1	1
Fabp4	25097	15197	16499	20440	18501	40944
Fads1	941	608	593	929	587	1195
Faf1	4532	2956	2707	3635	2450	4333

Faf2	1202	875	671	1069	634	1077
Fah	487	294	381	272	473	961
Faim2	4	9	4	5	3	2
Fam110b	214	181	172	187	164	143
Fam120a	8702	6361	5353	7429	5001	8110
Fam120aos	442	329	255	395	317	595
Fam129b	1455	868	537	1422	798	1334
Fam131a	951	484	388	833	485	953
Fam132a	217	122	110	210	133	232
Fam135b	1	0	0	0	0	0
Fam136a	1196	713	719	876	639	1102
Fam151b	86	56	40	86	57	118
Fam166b	2	1	1	0	0	1
Fam167a	21	13	12	12	3	18
Fam169a	55	33	41	42	19	52
Fam169b	3	1	2	3	3	7
Fam170b	0	1	0	0	0	0
Fam173a	533	267	243	427	280	673
Fam175a	720	553	446	565	408	720
Fam185a	946	655	609	857	534	763
Fam210b	554	350	307	501	393	623
Fam212a	113	73	64	121	68	172
Fam213b	1818	953	1122	1645	1108	2189
Fam214a	3050	1703	1399	2071	1880	4050
Fam216b	0	0	0	1	0	0
Fam219aos	1	0	0	0	0	0
Fam220a	2000	1264	1218	1654	1111	1739
Fam26d	0	0	1	0	0	0
Fam3b	1	0	0	0	0	0
Fam46a	1806	1502	935	2075	1235	2258
Fam47e	4	0	1	1	0	0
Fam53b	1244	817	617	1180	913	1263
Fam63b	4809	3443	3079	4281	2744	4248
Fam65b	298	320	212	272	176	351
Fam69b	644	380	319	501	240	596
Fam71e1	17	19	9	10	11	14
Fam71f1	0	0	1	0	0	0
Fam71f2	6	3	1	11	3	6
Fam72a	45	20	20	41	26	53
Fam73a	1418	1006	780	1190	749	1247
Fam73b	2816	1552	1427	2245	1245	2302
Fam78b	225	122	140	171	106	177
Fam83b	0	0	0	1	0	0
Fam83c	0	0	0	0	0	1
Fam83e	5	3	3	2	1	3

Fam84a	54	42	30	90	43	57
Fam84b	1031	635	567	982	628	1203
Fam92b	0	0	0	0	0	1
Fan1	361	228	205	267	206	261
Fancd2os	0	0	0	1	0	0
Fance	223	124	118	220	140	245
Fancg	141	72	45	126	70	180
Farsb	2161	1432	1174	1769	1087	1791
Fbxl16	164	110	86	122	73	140
Fbxl2	34	22	17	21	15	23
Fbxl20	2503	1675	1299	2030	1783	2981
Fbxl22	1527	753	935	1281	568	1158
Fbxo15	0	0	0	0	0	1
Fbxo17	55	40	36	29	21	60
Fbxo21	1876	1019	849	1512	1196	1858
Fbxo30	2021	1407	1254	1789	1067	1971
Fbxo40	4396	3516	3002	4059	2797	2537
Fbxo43	1	0	0	0	0	0
Fbxo47	12	4	7	15	15	12
Fbxo9	1591	1101	847	1438	1045	2030
Fbxw10	9	10	4	13	10	16
Fbxw15	0	0	0	0	0	1
Fcho2	5605	3664	2429	4531	3721	6192
Fcrl5	0	1	0	0	0	0
Fcrlb	1	0	0	0	0	0
Fdft1	1453	1054	851	1228	1108	1825
Fem1b	1837	1090	1087	1547	930	1578
Fen1	207	103	117	202	106	303
Fez2	3777	2363	2451	3754	2273	5057
Fezf1	0	0	0	1	0	0
Fgd4	698	489	350	534	379	442
Fgf11	294	203	172	268	132	257
Fgf14	64	47	35	82	54	68
Fgf23	0	0	0	0	0	2
Fgf4	0	1	0	0	0	0
Fgg	0	0	0	0	1	0
Fhl3	183	158	104	218	123	248
Fhl4	27	20	26	18	14	26
Fhl5	1	0	0	2	1	1
Fibcd1	0	0	0	1	0	0
Figla	1	0	0	0	0	0
Fis1	2373	1449	1279	2221	1282	3189
Fitm2	3832	2483	2128	3403	1960	3021
Fkbp10	918	657	542	817	568	1323
Fkbp1b	13	12	10	23	9	24

Fkbp5	1930	901	883	1791	1000	1889
Fkbp7	661	524	436	579	314	773
Fkbp8	3091	1736	1507	2709	1743	3305
Flna	7597	4795	3715	7936	4169	8311
Flnc	17253	11463	11016	15849	8757	12706
Flt3l	290	167	120	192	203	351
Flywch1	950	601	507	769	486	789
Fmnl1	1064	678	425	1126	550	1167
Fmod	49	95	46	182	73	65
Fmr1nb	4	9	0	3	10	18
Fndc1	1148	746	424	766	408	1088
Fndc5	22361	14317	13389	17371	10991	20065
Fndc9	0	0	1	0	0	0
Fnip1	5806	3502	3351	4377	3163	4688
Focad	1088	641	530	869	454	894
Fosb	19	16	12	18	22	30
Foxa3	1	0	0	0	0	0
Foxd2	11	11	5	22	14	9
Foxf1	18	17	9	19	18	36
Foxg1	0	1	0	0	0	0
Foxj1	5	0	0	5	2	4
Foxk2	1671	1201	1030	1493	912	1324
Foxl2os	0	0	1	0	0	0
Foxr2	0	0	0	0	1	1
Fpgs	215	157	102	93	124	202
Fpgt	1026	649	531	814	512	883
Frem2	538	272	276	300	316	315
Frmd3	11	3	1	5	12	10
Frmpd4	1	0	0	0	0	0
Frrs1	88	75	68	76	53	89
Frs3os	0	1	0	0	0	0
Fscn2	2	0	0	3	2	1
Fscn3	0	1	0	0	0	0
Fshr	0	1	0	0	0	0
Fsip1	0	0	0	0	1	1
Fstl1	4647	2982	2616	4653	2294	6768
Ftx	336	202	108	229	121	213
Fubp1	3747	2504	2331	2964	2031	3579
Fut4-ps1	0	0	0	1	0	1
Fxr1	11420	8141	7060	9677	6318	10609
Fzd8	280	163	148	326	164	312
G6pc2	0	0	0	0	0	1
Gabbr1	835	580	401	587	638	1082
Gabbr2	3	2	3	1	1	2
Gabrp	0	1	0	0	0	0

Gabrr1	0	1	0	0	0	0
Gabrr3	1	0	0	0	0	0
Gal	0	0	0	0	0	2
Gal3st1	4	3	2	2	1	1
Gal3st4	5	0	2	6	2	6
Galc	675	450	385	531	319	618
Galnt1	5030	3374	3519	4422	2872	4876
Galnt10	647	342	310	611	347	693
Galnt2	2771	1646	1412	2181	1325	2217
Galnt9	1	0	0	0	0	0
Galr1	0	0	0	1	0	0
Gapdhs	0	0	0	0	0	1
Garem	1311	837	898	951	833	1084
Gars	3583	2303	2077	2817	1807	3478
Gart	2070	1294	1114	1658	1001	1857
Gas7	596	405	326	457	239	597
Gast	0	0	1	0	0	0
Gatc	1019	780	677	965	546	1003
Gba2	1082	655	553	899	644	1290
Gbp11	9	6	2	8	15	10
Gca	1152	798	689	887	612	1126
Gcgr	6	6	8	19	6	11
Gclc	1000	735	527	786	529	976
Gcm1	0	1	0	0	0	0
Gcnt4	11	10	4	15	10	16
Gdap1	4	9	1	2	2	3
Gdap10	305	108	100	173	221	286
Gdf15	23	8	10	10	6	12
Gem	182	105	101	195	113	230
Gemin4	229	165	110	192	97	183
Ggt6	1	0	0	1	2	2
Gh	0	0	0	1	0	0
Ghdc	456	253	218	444	286	419
Ghsr	0	1	0	0	0	0
Gid4	3847	2469	2115	3084	2170	2950
Gif	0	1	0	0	0	0
Gimap5	110	69	93	101	104	206
Gipc1	798	532	418	787	510	926
Gipc3	85	44	35	73	48	110
Gja3	1055	645	515	776	520	885
Gja6	6	7	4	5	0	6
Gjc1	945	638	519	911	604	1161
Gjd4	0	0	0	1	0	1
Glb1l	172	104	84	122	119	289
Glce	1043	677	607	962	698	1275

Glis3	13	6	4	23	8	12
Glo1	3626	1422	2218	2078	1933	2637
Glod5	0	1	0	0	0	0
Glrp1	26	15	24	14	15	26
Gltpd2	0	0	0	0	0	1
Gm10336	3914	2014	2379	3152	5565	2886
Gm10439	0	0	0	0	0	1
Gm10440	0	0	0	0	1	0
Gm10474	0	0	0	0	0	1
Gm10494	0	0	1	0	0	0
Gm10509	150	133	110	109	90	164
Gm10556	1	0	0	0	0	0
Gm10638	43	22	17	31	33	51
Gm10684	0	1	0	0	0	0
Gm10745	0	0	0	0	1	0
Gm10768	0	0	0	0	0	1
Gm10825	0	0	0	1	0	1
Gm10857	1	0	0	0	0	0
Gm10865	0	1	0	0	0	0
Gm11128	0	0	0	1	1	0
Gm11201	0	1	0	0	0	0
Gm11549	0	0	0	0	0	2
Gm11747	0	0	0	0	0	1
Gm11974	36	23	13	12	8	34
Gm11978	0	0	0	0	0	1
Gm12060	13	2	4	8	7	17
Gm12070	41	20	17	52	16	57
Gm12159	0	0	0	0	0	1
Gm12169	0	1	0	0	0	0
Gm12298	0	0	0	0	0	2
Gm12409	23	7	15	15	28	27
Gm12429	0	1	0	0	0	0
Gm12504	7	0	1	8	4	5
Gm12522	1	1	1	3	0	5
Gm12603	0	0	1	0	0	0
Gm12718	1	0	0	0	0	0
Gm13032	0	0	0	0	1	1
Gm13051	0	0	0	0	0	1
Gm13102	1	0	0	0	0	0
Gm13154	186	93	99	116	151	269
Gm13238	1	0	0	1	0	5
Gm13272	1	0	0	0	0	0
Gm13277	0	0	0	0	0	1
Gm13546	1	0	0	0	0	0
Gm13547	0	0	0	1	0	0

Gm13582	1	0	0	0	0	0
Gm13749	7	0	3	1	1	3
Gm13807	0	0	0	0	0	1
Gm14005	69	53	40	84	42	125
Gm14057	107	88	55	89	46	95
Gm14169	1	7	1	5	4	11
Gm14403	1691	1438	1266	1251	1198	1849
Gm14431	1	0	0	0	0	0
Gm14461	0	0	0	0	1	0
Gm14827	48	29	33	25	20	47
Gm14858	0	0	0	0	0	1
Gm15104	2	2	2	0	0	3
Gm15179	30	18	15	11	14	23
Gm15319	0	0	0	0	0	1
Gm15421	19	8	8	4	7	13
Gm15612	9	9	2	10	9	15
Gm1564	0	0	0	0	0	1
Gm15713	0	0	0	1	0	0
Gm15787	24	24	21	14	20	15
Gm16023	26	10	15	14	8	18
Gm16287	0	1	0	0	1	4
Gm16294	0	0	0	1	0	0
Gm16596	4	0	1	1	0	1
Gm16617	1	0	0	0	0	0
Gm16675	25	14	12	15	20	42
Gm16702	0	0	0	1	0	1
Gm16845	5	13	4	9	11	21
Gm16853	0	0	0	1	1	0
Gm16863	1	0	1	2	2	2
Gm16897	19	13	9	9	6	14
Gm17019	1	0	0	0	0	0
Gm17296	357	195	144	256	142	248
Gm17660	0	1	0	0	2	3
Gm17745	21	12	14	13	23	36
Gm17751	0	0	0	0	1	0
Gm1821	4038	2439	1830	3392	2339	4554
Gm19276	0	0	0	0	0	1
Gm19303	0	0	1	0	0	0
Gm19345	0	0	0	1	0	0
Gm19434	0	0	0	0	0	1
Gm1979	0	1	0	0	0	0
Gm20187	0	0	0	0	1	0
Gm20319	0	0	1	0	0	0
Gm20324	7	7	2	4	3	3
Gm20337	7	2	6	3	4	2

Gm20356	0	0	0	0	0	1
Gm2061	0	0	0	0	0	1
Gm20756	1	0	0	0	0	0
Gm20939	18	9	10	15	14	34
Gm2115	12	8	12	4	6	12
Gm21221	0	0	1	0	0	0
Gm21269	0	0	0	1	0	0
Gm2447	0	0	0	0	0	1
Gm3086	24	15	13	24	14	41
Gm3317	1	0	0	3	1	1
Gm3336	0	1	0	0	0	0
Gm3414	79	44	31	87	52	82
Gm3458	1	0	0	0	0	0
Gm3488	1	0	0	3	1	1
Gm4371	0	0	0	0	0	1
Gm4598	1	0	0	0	0	0
Gm4710	0	0	0	1	0	0
Gm4787	26	20	13	14	12	22
Gm4788	8	1	5	14	3	9
Gm4827	0	1	0	0	0	0
Gm4832	0	0	1	0	0	0
Gm4861	0	0	0	0	1	0
Gm4872	0	0	0	0	0	1
Gm4890	16	12	11	8	9	11
Gm4937	1	0	0	0	0	0
Gm4951	63	74	62	123	65	112
Gm4956	156	92	79	164	72	210
Gm4961	0	0	0	1	0	0
Gm4984	0	0	1	0	0	0
Gm5	1	0	0	0	0	0
Gm5105	50	41	40	52	19	46
Gm5108	0	0	0	0	0	1
Gm5113	447	332	251	395	235	310
Gm5126	1	1	1	1	0	9
Gm5129	6	2	2	1	2	2
Gm525	0	0	1	0	0	0
Gm53	0	0	0	0	1	0
Gm5415	0	0	0	0	1	0
Gm5458	0	0	0	0	1	1
Gm5460	1	0	0	0	0	0
Gm5464	5	2	2	2	1	1
Gm5523	24	10	16	27	14	37
Gm5607	1	0	0	0	3	1
Gm5627	0	0	1	0	0	0
Gm5766	0	0	1	0	0	0

Gm5803	2	0	0	2	2	2
Gm5868	1	0	0	0	0	0
Gm5936	0	1	0	0	0	0
Gm6116	0	0	1	0	0	0
Gm6150	0	0	0	0	1	0
Gm6194	1	0	0	1	0	3
Gm6213	1	0	0	0	0	0
Gm6377	20	17	12	34	19	28
Gm6559	1	0	0	0	0	0
Gm6578	1	0	0	0	0	0
Gm6614	0	0	0	0	1	1
Gm6634	13	8	17	8	9	10
Gm6642	3	1	0	0	0	1
Gm6682	1	0	0	1	1	3
Gm6760	0	0	0	0	0	1
Gm6787	4	4	4	1	1	5
Gm6938	0	0	0	0	1	0
Gm6994	0	0	0	0	1	0
Gm7008	4	1	1	2	0	0
Gm7244	1	0	0	0	0	0
Gm766	7	0	1	0	0	2
Gm7904	0	0	1	0	2	4
Gm806	1	0	0	0	0	0
Gm8898	1	0	0	0	0	0
Gm9047	1	0	0	0	0	0
Gm9199	0	0	0	1	0	0
Gm9696	0	0	0	0	1	0
Gm9733	0	1	0	0	0	0
Gm996	22	6	11	8	6	12
Gmnn	1640	1207	1157	1464	1223	2505
Gnai2	10804	7455	5630	10566	6198	13732
Gnat2	3	0	5	2	0	2
Gnaz	20	14	9	13	8	10
Gnpnat1	285	154	170	206	120	261
Golga4	32101	23675	21491	24007	21771	24983
Golga7b	29	16	17	16	8	23
Golim4	2535	1764	1382	2100	1268	2256
Golt1b	884	526	470	662	392	762
Gon4l	1521	1040	720	1052	734	1397
Gp1ba	12	13	6	4	11	4
Gp6	3	6	3	1	3	3
Gpam	6607	3918	3967	4039	4391	4571
Gpatch4	504	441	375	335	351	594
Gpd1	2922	2238	1897	2841	1424	3113
Gpd2	1140	771	529	846	554	979

Gpha2	2	1	1	0	1	0
Gpm6b	1855	1287	933	1553	639	1785
Gpr115	23	9	12	21	20	32
Gpr119	0	0	1	0	0	0
Gpr12	1	0	0	0	0	0
Gpr126	150	86	52	73	50	130
Gpr141	6	1	6	13	4	12
Gpr143	0	0	0	0	1	1
Gpr146	3633	2612	2134	3107	2029	3376
Gpr151	0	0	0	0	0	1
Gpr162	29	15	11	27	22	33
Gpr165	1	2	1	1	0	0
Gpr171	12	4	3	7	2	3
Gpr31b	0	0	0	1	0	0
Gpr34	125	107	50	104	39	112
Gpr37l1	36	19	29	20	11	40
Gpr39	2	7	5	5	9	21
Gpr4	579	381	290	515	342	743
Gpr45	0	0	1	0	0	0
Gpr50	1	0	0	0	0	0
Gpr85	10	9	5	21	7	14
Gpr97	65	41	27	60	34	108
Gprc5d	0	1	0	0	0	0
Gpsm3	119	64	58	119	63	154
Gpx7	227	156	130	226	130	330
Gramd1c	52	26	25	40	41	76
Grb14	6957	4770	4328	6349	6140	7828
Grid1	0	1	0	0	0	0
Grid2	0	0	0	1	0	1
Grin2c	132	68	70	106	46	63
Grip2	1400	711	564	873	637	946
Grk5	1465	1078	790	1565	1050	1452
Grpr	0	0	0	0	0	1
Grxcr1	0	0	0	0	0	1
Gsdma	3	1	3	3	7	5
Gsdmcl1	0	0	0	0	0	1
Gspt1	5693	3919	3602	4633	3042	5422
Gsta4	2222	1521	1412	2048	1740	2795
Gstm7	1366	866	594	1140	940	1309
Gstt1	517	326	253	497	353	562
Gstt2	371	168	144	234	206	457
Gstt3	179	116	111	172	130	248
Gtdc1	600	438	320	517	507	684
Gtf2a1l	0	4	0	0	1	0
Gtf3c4	1618	993	894	1345	819	1160

Gtf3c5	599	434	290	476	274	549
Gtpbp10	588	382	371	424	307	560
Gtse1	22	10	10	18	13	36
Gtsf1l	0	0	1	0	0	0
Guca1b	27	12	11	14	9	14
Guca2a	0	0	1	0	0	0
Gucy1b2	0	1	0	2	0	2
Gulo	0	0	1	0	0	0
Gulp1	394	288	276	347	304	612
Gzmb	10	8	4	5	0	9
Gzmc	0	0	0	0	0	1
H19	612	824	625	521	376	1084
H1f0	4777	3430	2888	4738	3078	6095
H1foo	0	0	0	0	0	1
H1fx	40	33	15	42	30	68
H2-Aa	1740	978	715	2133	651	2205
H2-Eb1	1519	892	661	1836	537	1856
H2-M2	0	0	1	0	0	0
H2-M3	167	68	94	162	91	217
H2-Q10	1	5	4	2	0	4
Hapln1	17	24	11	11	9	18
Hapln2	0	0	0	0	1	1
Has3	224	107	78	122	87	158
Hbb-bh1	0	0	1	0	0	0
Hbq1b	3	2	1	1	0	2
Hbs1l	3169	2168	2008	2668	1770	3133
Hcar2	0	0	1	5	0	0
Hcst	2	0	1	5	0	3
Hdac1	381	170	185	288	213	444
Hdac5	3562	2133	1436	3014	2066	3439
Hdac9	1125	894	741	1023	1042	1509
Hdc	83	37	36	77	36	116
Hddc2	61	52	38	60	51	106
Hdhd2	2081	1441	1208	1612	1086	2000
Hdx	9	4	3	11	6	11
Heg1	5325	4062	2553	3831	2885	4538
Helt	40	16	21	17	16	31
Herc4	2551	1816	1473	2181	1350	2430
Herc6	594	366	236	459	302	836
Herpud1	18279	9871	7021	16930	8983	18300
Hes1	797	595	506	832	503	1190
Hes2	0	0	0	0	1	0
Hes3	0	0	0	0	1	0
Hexa	2540	1792	1616	2073	1410	2607
Hey1	549	286	301	570	292	630

Hgf	31	26	15	19	11	31
Hgs	1144	753	505	1029	743	1126
Hhipl2	14	6	4	6	2	6
Hiatl1	1404	709	645	1291	659	1558
Higd1a	1976	1311	1436	1671	1001	2212
Higd1c	1	0	0	0	0	0
Hils1	0	0	0	0	1	0
Hint1	4092	2451	2355	3914	2505	4835
Hipk2	1440	1604	1067	1394	1206	746
Hist1h1a	3	0	1	2	2	6
Hist1h1c	1004	798	728	1038	836	1318
Hist1h1d	3	1	3	6	3	6
Hist1h2ab	0	0	0	1	0	0
Hist1h2ad	8	0	6	2	2	3
Hist1h2ai	0	0	0	0	0	1
Hist1h2ak	1	0	0	0	0	0
Hist1h2bf	1	0	0	0	0	0
Hist1h2bm	1	0	0	0	0	0
Hist1h2bn	4	4	1	0	2	3
Hist1h3c	1	2	1	0	0	1
Hist1h3d	14	5	2	5	3	3
Hist1h3i	0	3	3	1	0	1
Hist1h4a	0	0	0	0	0	1
Hist1h4d	4	3	1	2	1	1
Hist1h4i	25	9	14	13	11	11
Hist3h2a	241	214	158	230	134	238
Hist4h4	1	0	0	0	0	0
Hkdc1	0	0	1	0	0	0
Hltf	1536	1064	935	1153	906	1512
Hlx	280	137	179	213	227	405
Hmbs	983	674	649	801	521	973
Hmga2-ps1	75	17	42	19	34	37
Hmgb1-rs17	0	0	0	1	0	0
Hmgcll1	0	4	2	3	4	7
Hmgn3	256	199	187	293	161	478
Hmha1	402	263	181	251	165	390
Hnf1a	0	0	0	0	0	1
Hnrnpc	4754	3302	3055	4036	2565	4612
Hook2	155	94	96	113	128	257
Hoxa10	0	0	0	1	0	1
Hoxa13	0	0	1	0	0	0
Hoxb2	47	12	16	37	31	46
Hoxb9	1	0	0	0	0	0
Hoxd13	1	0	0	0	0	0
Hrh2	13	10	11	15	13	26

Hrh3	3	0	4	4	1	14
Hrk	37	24	25	42	29	57
Hs1bp3	672	443	335	531	318	538
Hs6st1	1435	871	827	1129	739	1214
Hs6st2	74	44	25	45	26	39
Hscb	396	235	211	332	272	448
Hsd17b11	1255	871	802	1122	858	1872
Hsd17b6	0	0	0	0	0	1
Hsdl1	796	496	426	764	500	879
Hspa12a	1006	682	501	878	972	842
Hspa2	290	204	116	306	174	327
Hspg2	22937	13769	9528	21278	12048	24776
Htr4	0	0	0	0	1	0
Htr5b	0	1	0	0	0	0
Htr7	35	35	38	63	37	64
Hus1	835	548	488	637	480	694
Hyal4	0	0	0	0	1	0
Hyal5	0	0	0	1	0	0
Hyi	56	32	30	54	39	81
Iah1	431	239	224	403	248	491
Ica1	376	269	292	408	264	585
Icam5	2	1	2	1	0	1
Ier2	362	199	212	334	218	521
Ifitm1	157	86	69	162	90	162
Ifitm3	2115	1348	1001	1909	1001	2992
Ifitm6	3	3	3	8	3	9
Ifna13	0	0	0	0	1	0
Ifngr2	9549	6665	5764	7771	5403	8257
Ifnz	0	0	0	1	0	0
Ifrd1	1880	1153	1110	1316	1022	1872
Ift22	176	100	75	150	111	189
Ift74	275	209	157	285	191	388
Igf1	1024	705	791	732	521	1261
Igfals	102	60	34	39	35	84
Igfbp1	0	0	1	0	0	0
Igfn1	0	1	0	0	0	0
Igsf10	249	157	113	191	91	178
Igsf21	6	2	3	4	4	16
Igsf9	34	17	9	19	8	20
Ikbkap	1595	1027	890	1362	781	1378
Ikzf1	147	75	62	105	46	108
Ikzf2	428	235	245	268	209	381
Ikzf3	18	13	11	17	6	9
Il10	0	0	1	3	0	2
Il12rb1	1	0	1	2	0	6

Il13ra1	3980	2841	2521	3811	1924	3645
Il17ra	154	115	86	182	102	219
Il17rb	3	3	2	5	4	6
Il17rc	245	108	86	169	139	251
Il18bp	431	352	233	342	208	454
Il1r2	6	0	2	2	0	1
Il1rapl1	0	0	0	1	1	0
Il20	0	0	0	0	0	1
Il20ra	0	0	1	0	0	0
Il21r	24	9	13	15	4	17
Il22	0	0	1	0	0	0
Il25	0	0	0	0	1	0
Il3ra	96	63	50	102	63	135
Il4	1	0	0	0	0	0
Il6st	11243	8260	6689	9676	6602	9541
Il7r	16	21	10	39	11	27
Il9r	0	1	2	0	0	1
Ina	0	0	0	1	0	0
Ino80	1276	831	612	952	647	1075
Ino80dos	34	18	30	38	29	64
Inpp5d	395	258	175	415	192	568
Inpp5k	1931	1219	924	1576	1303	2132
Insc	21	6	6	8	5	11
Insm2	0	0	1	0	0	0
Ints6	884	580	418	674	457	645
Ints7	630	512	344	530	350	566
Ip6k2	430	305	265	384	307	621
Ipo11	1724	1234	996	1457	1021	1482
Ipo5	10613	7291	6181	8498	6228	8658
Ipo8	2076	1373	1148	1685	1113	1805
Ippk	435	278	248	316	234	398
Iqca	0	0	0	1	0	0
Iqcc	534	392	254	401	288	429
Iqcf5	1	0	0	0	0	0
Iqch	0	0	0	1	0	1
Irak1bp1	233	155	132	172	114	222
Irak2	336	251	258	291	320	602
Irf2bp2	2874	1710	1391	2545	1772	3101
Irgc1	1	0	0	0	0	0
Irgm1	1817	894	712	1493	857	1834
Irgm2	2244	1081	855	2376	871	2222
Irs2	201	142	120	141	113	174
Irs4	0	0	1	0	0	0
Irx5	363	353	191	426	271	496
Isl1	2	2	0	1	0	0

Isl2	1	1	3	0	1	1
Islr	1137	746	582	1039	350	960
Isx	1	0	0	0	0	0
Isyna1	512	332	177	427	384	468
Itfg1	3731	2638	2231	3161	2125	3423
Itga11	113	114	75	128	45	99
Itga2b	134	90	49	65	77	89
Itga9	3767	2315	2383	3121	2136	2888
Itgal	119	63	39	70	46	59
Itgam	262	161	128	164	87	277
Itgb1bp1	1132	707	688	795	515	1216
Itgb3	151	95	100	117	83	117
Itgb5	3640	2474	1946	3583	2356	4058
Itih1	0	0	0	1	0	0
Itln1	1	0	0	0	0	0
Itm2a	533	303	228	381	137	510
Itpk1	316	187	158	328	195	353
Itpr2	2315	1285	1055	1962	1276	2413
Ivl	0	0	1	0	0	0
Ivns1abp	113569	71903	57371	77148	57239	97512
Izumo4	113	73	52	79	89	169
Jade1	2489	1683	1552	2029	1389	2424
Jag1	1280	732	580	1156	661	1362
Jag2	1483	792	578	1467	766	1440
Jmy	4578	3323	2798	3811	2805	3877
Josd1	1245	834	757	1186	588	1052
Jph4	8	8	5	4	3	8
Kank4os	0	0	0	1	1	0
Kansl1l	2322	1547	1275	1790	1328	2040
Kansl2	644	390	353	438	315	575
Kat2a	1036	669	517	742	516	976
Katnal2	0	1	0	2	0	2
Kbtbd12	797	615	566	643	513	726
Kcna1	339	216	170	178	146	363
Kcna10	0	0	1	0	0	0
Kcna6	157	73	70	83	57	123
Kcnb2	0	2	2	1	0	0
Kcnc2	66	43	33	44	32	50
Kcnc4	33	5	4	19	12	39
Kcnd2	2238	1367	1052	1800	1092	1502
Kcne1	100	68	59	133	105	86
Kcnh1	15	10	10	18	17	20
Kcnh4	0	0	0	1	0	0
Kcnh6	2	2	0	1	0	0
Kcnj4	28	15	8	9	13	13

Kcnj6	0	0	0	0	0	1
Kcnk10	0	0	0	1	0	0
Kcnk12	1	0	0	0	0	0
Kcnk13	22	16	16	17	8	20
Kcnk2	159	86	102	111	52	167
Kcnk6	215	157	152	243	149	333
Kcnma1	20	5	15	21	19	23
Kcnmb2	0	0	0	0	0	1
Kcnmb3	0	1	0	0	0	0
Kcnq2	0	0	0	0	0	1
Kcnq3	0	0	1	0	0	0
Kcnrg	4	0	4	2	6	10
Kctd1	500	389	304	388	286	472
Kctd10	2757	1565	1275	2562	1341	3049
Kctd17	463	281	227	391	284	602
Kctd4	0	0	1	0	0	0
Kctd7	206	158	105	184	84	177
Kdelc1	1016	668	580	823	486	987
Kdm5c	3995	2683	1368	3643	1305	2192
Kdm6a	2380	1529	664	1872	825	1329
Kdm8	188	108	98	138	93	120
Kdsr	647	408	360	502	425	911
Khdc1a	0	0	1	0	0	0
Khdrbs1	2714	1892	1597	2293	1497	2549
Khnyn	989	515	481	802	564	1065
Kif12	0	0	0	1	0	0
Kif16b	3434	1973	2206	2289	2116	2506
Kif26a	1000	517	358	830	514	1002
Kif27	12	12	7	6	6	9
Kif3c	531	363	281	472	215	422
Kiss1r	16	13	15	9	11	15
Kit	224	115	178	230	151	415
Klf4	1691	1054	1224	1555	1293	2515
Klhdc8b	522	280	180	426	239	593
Klhdc9	3	2	4	1	1	3
Klhl6	374	281	264	419	255	566
Klk1	0	0	0	0	0	2
Klk11	0	0	2	4	3	0
Klk13	1	1	1	4	1	4
Klk14	14	1	3	5	12	17
Klk1b8	0	1	0	0	0	0
Klra19	0	0	0	0	0	1
Klra21	2	1	1	3	2	4
Klra3	0	1	0	1	0	3
Klra6	0	0	0	0	0	1

Klrg2	16	5	17	11	5	9
Klri1	0	1	0	3	0	2
Kmt2a	5849	4273	3085	4127	3308	5060
Kncn	0	1	0	0	0	0
Kntc1	29	7	3	12	7	58
Kpna1	6321	4319	4073	5866	3487	4646
Kpna6	5114	3359	2836	4316	2610	4150
Kras	2846	1918	1637	2325	1402	2814
Krr1	1681	1178	1055	1349	989	1541
Krt1	0	0	0	0	0	1
Krt18	18	23	14	31	21	37
Krt28	0	0	0	0	0	1
Krt78	3	0	0	3	4	2
Krtap5-3	0	0	0	0	0	1
L1cam	231	170	122	182	114	211
Lamb3	577	368	166	484	404	498
Lamc2	572	372	401	586	250	463
Lamtor3	1615	1132	1140	1378	750	1853
Larp1b	3638	2732	2452	2995	2141	3949
Lasp1	1885	1061	814	1827	898	2111
Lat2	79	45	24	57	43	110
Layn	183	110	93	200	98	249
Lbh	15933	10227	8501	11789	8635	12907
Lbr	1507	802	722	1228	880	1702
Lca5l	38	28	16	37	32	47
Lcn11	0	1	0	0	0	0
Lcn2	143	63	113	139	119	202
Lctl	0	1	0	2	0	3
Ldhd	1502	927	758	1013	848	1244
Lefty2	0	0	0	1	0	0
Lekr1	137	114	99	103	70	151
Leng1	1089	810	671	848	572	1198
Leprel2	471	287	211	418	263	538
Lgals4	1801	729	552	705	1588	1879
Lgals6	1550	604	485	617	1400	1616
Lgr5	8	3	4	16	4	10
Lgsn	0	2	2	0	1	0
Lhcgr	3	3	1	2	1	0
Lhfpl1	0	1	0	0	0	0
Lhfpl4	31	23	10	14	7	27
Lhx3	0	1	0	0	0	0
Lifr	5316	3314	2959	3906	2654	5150
Lin28a	0	0	0	0	0	1
Lin9	229	146	114	230	146	244
Lingo3	296	181	207	240	129	290

Lman1l	33	26	28	32	29	70
Lman2	4779	3005	2522	3862	2439	4031
Lmf2	1362	877	612	1252	793	1411
Lmo2	1164	916	836	968	630	1311
LOC100038947	1	1	0	1	1	4
LOC100503496	133	83	98	109	66	121
LOC100503676	80	58	36	56	33	57
LOC100861615	0	0	0	0	0	2
LOC100862268	1	0	0	0	0	0
LOC101056149	0	0	0	1	0	0
LOC102632430	0	0	0	0	1	0
LOC102634101	1	0	0	0	0	0
Loxhd1	0	0	1	0	1	3
Loxl2	584	314	333	533	246	989
Lpar1	615	447	358	541	223	669
Lpar6	1015	663	436	919	571	1180
Lphn2	3898	2510	2080	3589	2715	4114
Lrba	2680	2138	1553	2354	1568	2042
Lrch1	1226	881	668	1150	827	1424
Lrch2	122	81	52	103	80	153
Lrguk	1	0	2	5	2	1
Lrit2	1	0	0	0	0	0
Lrp10	2572	1381	1264	2335	1359	2694
Lrp8	35	30	34	46	28	82
Lrpprc	12474	8103	6951	9884	6883	10081
Lrrc1	1053	765	670	884	601	1034
Lrrc10	7736	6170	3485	7952	4505	9724
Lrrc26	0	0	0	0	1	0
Lrrc3	33	14	10	30	20	33
Lrrc36	53	20	19	23	43	75
Lrrc7	0	0	1	0	0	0
Lrriq3	6	4	9	13	8	15
Lrriq4	0	0	0	0	0	1
Lrrk2	1170	791	708	1086	805	1552
Lrrtm4	0	0	0	1	0	1
Lrtm1	33422	21725	20008	24153	20797	26260
Lsm3	1514	1390	1281	1474	529	2096
Ltb4r1	9	3	5	7	2	2
Ltbr	844	496	453	752	517	989
Lxn	144	78	75	149	69	194
Ly6g6f	1	0	0	3	1	1
Ly6i	9	2	7	1	5	5
Lyl1	129	62	54	118	71	154
Lypd1	12	11	3	7	5	6
Lyrm1	247	179	154	173	142	252

Lyrm9	1873	1288	1099	1445	1082	1686
Lztfl1	1406	824	859	892	753	1365
Maats1	1	3	0	0	0	1
Madcam1	0	0	0	1	0	1
Maf1	2899	1640	1372	2482	1655	3070
Magea4	0	0	0	0	0	1
Maged2	1064	637	400	963	571	1111
Magi1	1097	750	582	983	749	1241
Magt1	3424	1961	2031	2537	1739	2979
Mak	1	0	0	0	0	0
Man2a2	4636	3331	2520	4276	2187	4084
Mansc4	0	0	0	0	1	0
Map1b	952	682	549	1012	685	1057
Map2	316	193	197	327	201	389
Map2k2	2237	1177	1015	2045	1198	2397
Map2k6	226	171	202	241	194	399
Map3k12	389	228	168	299	224	491
Map3k13	119	91	54	62	62	97
Map3k15	77	64	78	44	56	91
Map3k19	2	0	0	4	2	0
Map4k4	7420	5143	4281	6129	3874	7250
Map6	243	209	135	307	156	323
Map7d1	15725	10395	7715	12848	7509	13054
Mapk12	1291	808	692	1166	742	1538
Mapk1ip1l	1004	722	617	917	542	915
Mapk6	1533	1096	953	1288	873	1395
Mapk9	2982	2111	1809	2567	1584	2841
1-Mar	0	0	0	0	1	0
2-Mar	1343	753	781	1157	816	1714
Marco	2	1	2	0	1	1
Mark1	697	375	334	498	340	499
Marveld1	1625	930	773	1673	922	1627
Mast1	16	3	4	4	4	5
Matn1	0	1	0	0	0	0
Matn3	0	0	0	0	0	1
Max	2635	1855	1352	2464	1715	3000
Mbd1	1599	1102	1110	1209	941	1695
Mbip	376	274	197	329	261	511
Mblac1	36	13	16	29	32	36
Mboat4	1	0	4	1	1	0
Mcc	1407	872	662	1105	893	1560
Mcf2	0	0	0	1	0	0
Mcm2	312	224	156	223	133	295
Mcm5	142	80	65	127	62	225
Mcm8	324	269	184	286	121	339

Mcoln1	1128	706	550	810	538	1011
Mcrs1	512	271	212	427	291	559
Mdga1	1097	817	528	908	311	1072
Med1	3157	2103	1711	2469	1839	2504
Med18	116	57	70	65	45	113
Med21	944	614	590	720	508	977
Med26	401	276	184	403	277	449
Med27	377	213	179	253	163	333
Mef2b	8	3	1	5	5	12
Mesdc2	2036	1306	1138	1737	947	1788
Mesp2	0	0	0	1	0	0
Metap1d	1868	1167	1096	1218	1005	1980
Metrnl	505	268	277	410	212	381
Mettl13	311	196	126	214	141	236
Mettl14	1043	871	702	926	673	1049
Mettl16	820	588	534	712	441	730
Mettl2	471	317	291	354	249	474
Mettl7a2	0	0	2	2	4	1
Mfi2	3	1	0	0	1	0
Mfsd7c	247	198	108	277	159	280
Mfsd8	1109	620	613	834	577	878
Mgll	9076	4499	4438	7091	4658	11515
Mib1	725	459	385	702	483	790
Mical2	5015	3493	3004	4256	2740	4409
Micall1	5624	3994	3089	5013	2936	4207
Micu1	603	362	320	536	382	686
Mid1ip1	5623	3149	2719	5321	4172	4605
Midn	3926	2907	2077	4407	2604	4463
Mief2	1312	816	663	1159	803	1453
Mier1	2784	1697	1582	2417	1746	3331
Miip	451	260	196	333	289	597
Mir100	0	0	0	0	1	1
Mir107	0	0	0	1	0	1
Mir1190	0	0	0	1	0	0
Mir1192	2	2	0	3	2	4
Mir1231	0	0	1	0	0	0
Mir1247	1	0	0	0	0	0
Mir1249	4	0	1	1	4	7
Mir1251	0	0	0	0	1	0
Mir130c	0	0	0	1	0	0
Mir133a-2	13	4	5	6	3	6
Mir134	1	0	0	0	0	0
Mir138-2	0	0	0	0	1	0
Mir143hg	230	162	104	181	161	298
Mir15a	0	0	0	1	0	0

Mir17	0	0	0	0	0	1
Mir181a-1	1	0	0	0	0	0
Mir181a-2	0	0	0	1	0	1
Mir1839	6	5	2	3	3	2
Mir186	9	5	2	15	3	13
Mir1893	12	9	12	32	8	16
Mir1894	3	3	5	0	1	5
Mir190	0	1	0	0	0	0
Mir1902	382	235	204	284	177	303
Mir1905	0	0	1	0	0	0
Mir191	0	0	0	0	1	1
Mir1931	0	0	0	0	0	1
Mir1932	0	0	0	0	0	2
Mir1934	0	0	0	0	1	0
Mir1960	0	0	1	0	0	0
Mir1964	2	0	4	1	0	1
Mir1969	0	0	0	0	0	2
Mir199a-2	0	0	0	2	0	0
Mir19a	0	0	0	0	0	1
Mir19b-1	0	0	0	0	0	1
Mir1a-2	0	0	0	1	0	1
Mir1b	0	0	0	0	0	1
Mir207	0	0	0	0	1	0
Mir20a	0	0	0	0	0	1
Mir22	4	3	2	7	1	10
Mir2861	3	1	1	5	2	4
Mir29a	0	0	0	0	0	1
Mir300	1	0	0	0	0	0
Mir301	0	0	0	0	0	2
Mir3098	1	0	0	0	0	0
Mir30c-1	1	0	0	0	0	0
Mir30e	1	0	0	0	0	0
Mir3101	6	6	3	7	6	13
Mir330	0	1	0	0	0	0
Mir3473c	1	0	0	0	0	0
Mir3473d	1	0	0	0	0	0
Mir3473e	0	0	1	0	0	0
Mir3473f	0	0	0	0	1	0
Mir350	0	0	0	1	0	0
Mir351	1	1	0	3	0	3
Mir365-2	0	0	0	0	0	1
Mir374	1	2	5	0	1	3
Mir377	0	1	0	0	0	0
Mir3963	0	0	0	0	0	1
Mir423	0	0	0	0	1	0

Mir433	1	0	0	0	0	0
Mir451	0	0	0	0	0	1
Mir485	1	0	0	0	0	0
Mir490	1	0	0	0	0	0
Mir504	0	0	0	2	0	0
Mir5046	49	37	24	29	28	28
Mir5100	0	0	0	1	0	0
Mir5103	0	0	0	0	1	0
Mir5119	5	5	2	1	3	2
Mir5123	0	0	1	0	0	0
Mir5131	0	0	1	3	1	0
Mir5132	4	7	5	1	4	5
Mir5136	0	0	0	0	0	1
Mir544	1	0	0	0	0	0
Mir5618	1	0	0	0	0	0
Mir5619	0	0	1	0	0	0
Mir5621	1	0	0	0	0	0
Mir5623	0	1	0	0	0	0
Mir5709	10	4	6	5	4	5
Mir6352	1	0	0	0	0	0
Mir6353	0	0	0	0	0	1
Mir6360	0	0	0	0	0	1
Mir6391	0	0	0	0	1	0
Mir6392	0	0	0	0	1	0
Mir6403	1	1	3	5	5	2
Mir6412	0	0	0	1	0	0
Mir671	19	5	6	17	6	26
Mir675	52	79	63	50	33	99
Mir6896	0	0	0	0	1	0
Mir6899	0	0	0	0	0	1
Mir6902	0	0	0	0	1	0
Mir6908	0	0	1	0	0	0
Mir6913	2	0	0	6	1	0
Mir6914	41	39	23	29	27	18
Mir6915	5	2	2	2	1	1
Mir692-1	0	0	0	0	1	0
Mir6924	1	0	0	0	0	0
Mir6926	0	0	0	0	0	1
Mir6930	1	0	0	0	0	0
Mir6934	0	0	0	1	0	0
Mir6946	0	0	1	0	2	2
Mir6948	0	1	1	1	3	3
Mir6959	0	0	0	0	0	1
Mir6962	0	0	0	0	0	2
Mir6964	0	0	0	0	0	2

Mir6968	0	0	0	0	1	0
Mir6978	0	0	0	0	1	0
Mir6980	0	0	1	0	0	0
Mir6981	9	1	2	4	10	9
Mir6984	2	1	2	0	0	2
Mir6987	0	0	0	1	0	0
Mir6996	0	0	0	1	0	1
Mir7022	0	0	1	0	0	0
Mir7032	0	1	0	0	0	0
Mir7039	0	0	1	0	0	0
Mir7042	0	0	0	0	0	1
Mir7054	1	0	0	0	0	0
Mir7057	0	0	1	0	0	0
Mir7062	0	0	0	0	0	1
Mir7065	0	0	0	0	0	1
Mir7074	0	0	0	1	0	0
Mir7075	0	0	0	0	1	0
Mir7083	1	0	0	0	0	0
Mir7085	0	0	0	0	0	1
Mir7089	0	0	0	0	0	1
Mir711	1	0	0	0	0	0
Mir7118	0	1	0	0	0	0
Mir7215	0	0	0	0	0	1
Mir7232	0	0	0	0	0	1
Mir7662	0	0	1	0	0	0
Mir7663	0	0	0	0	0	1
Mir7668	1	0	0	1	2	1
Mir7674	3	1	2	0	0	2
Mir7678	0	0	0	2	0	0
Mir7679	0	0	0	0	0	1
Mir804	0	0	0	0	1	0
Mir8093	9	14	11	5	7	13
Mir8105	0	0	0	0	0	1
Mir8112	5	2	2	1	2	2
Mir8113	0	0	0	1	0	0
Mir874	1	0	0	0	0	0
Mir99b	0	0	0	0	0	1
Mirlet7a-2	0	0	0	1	0	0
Mirlet7b	7	6	4	10	5	17
Mirlet7g	0	0	0	0	2	0
Mirlet7i	0	0	1	0	0	0
Mis18a	133	89	79	143	83	207
Mis18bp1	24	12	10	18	9	67
Mkks	2587	1795	1706	2238	1340	2579
Mkrn1	1370	958	799	1054	720	1419

Mkx	27	16	10	23	7	11
Mlh1	536	381	351	402	304	565
Mlkl	187	120	90	167	112	263
Mlxip	3166	1985	1723	2556	2062	3854
Mmachc	2932	1958	1762	2483	1573	2538
Mmgt1	3865	2708	2442	3332	2223	3257
Mnd1-ps	0	0	0	0	0	1
Mndal	1721	1217	931	1522	1190	2267
Mnt	891	546	395	594	439	679
Mocos	230	141	132	219	150	280
Mocs1	443	263	255	444	260	583
Morc1	0	0	0	0	0	1
Morf4l2	2229	1614	1465	1980	1198	2206
Morn1	30	24	14	25	24	55
Morn5	1	0	0	0	2	3
Mospd3	262	168	121	225	155	328
Mpdu1	308	168	151	249	191	389
Mpdz	5284	3769	3197	4491	2923	4329
Mphosph10	819	693	574	692	537	820
Mphosph9	749	504	385	529	405	677
Mpo	0	0	0	1	0	1
Mpp1	612	395	310	570	346	831
Mrap	282	222	210	272	152	275
Mrap2	0	0	0	0	0	1
Mrgpra4	0	0	0	0	0	1
Mrgprb8	0	1	0	0	0	0
Mrpl19	1638	1123	1139	1375	904	1616
Mrpl34	1444	914	834	1088	780	1305
Mrpl35	2013	1349	1319	1671	1025	1961
Mrpl45	2970	1824	1892	2352	1539	3028
Mrpl57	669	410	356	501	314	627
Mrps2	1646	1171	1078	1406	937	1507
Mrps6	870	582	642	739	458	951
Ms4a18	0	0	0	0	2	0
Ms4a3	0	0	0	0	0	1
Ms4a8a	6	3	1	3	5	12
Msantd1	27	10	13	11	7	22
Msantd4	3540	2571	2408	3115	1975	3317
Msi1	10	2	4	3	3	2
Msl3	3464	2338	1987	2815	1864	2979
Mss51	50	18	23	26	36	71
Mt3	24	17	15	22	10	11
Mtag2	0	0	1	1	0	3
Mtap	1299	1022	886	1180	770	1261
Mtfp1	4566	2782	2595	3523	2106	4379

Mtfr1	2494	1642	1401	2024	1360	2055
Mtfr2	13	7	9	12	5	51
Mthfd1	1530	1044	897	1365	745	1305
Mtmr7	31	16	18	28	24	49
Mtnr1a	0	0	0	1	0	0
Mto1	1323	844	746	1125	653	1168
Mtrf1	631	446	376	466	341	633
Mtx1	650	442	416	492	379	639
Mtx3	1590	1101	917	1143	845	1606
Muc4	74	31	24	13	35	44
Mug1	0	0	0	0	0	1
Mup4	0	0	1	0	0	0
Mut	8618	5939	5003	7407	4737	7073
Mxd3	2	3	1	6	1	9
Mybbp1a	2955	1927	1423	2302	1431	2547
Myh15	0	0	1	0	0	0
Myh7	19746	17797	13544	27079	12483	33244
Myh8	0	0	1	1	3	0
Myh9	7334	4852	3267	7119	4122	8359
Myl7	1668	1316	1622	1649	1319	1210
Myo16	2	1	3	0	0	2
Myo5b	2167	1563	1490	1661	1405	1861
Myo7b	1	0	0	0	0	0
Myog	0	0	0	1	0	0
Mypn	9948	7816	6222	9434	5416	7789
N4bp2	426	303	259	367	232	355
N4bp2l1	155	99	93	116	68	144
Naa11	0	0	0	0	1	0
Naa20	1538	1017	1044	1315	767	1561
Naa25	904	709	554	775	564	790
Naaladl1	0	0	1	0	0	0
Nadsyn1	210	122	82	186	122	230
Nags	4	4	3	3	2	2
Naip1	0	0	0	0	1	0
Naip7	44	34	23	46	34	66
Nanos2	1	1	0	0	1	7
Napepld	340	267	284	318	185	341
Narfl	1784	1068	957	1438	832	1597
Nat14	107	55	44	62	36	76
Nat3	0	1	0	0	0	0
Nbl1	459	233	215	501	217	445
Ncam1	315	276	199	478	230	398
Ncam2	5	1	0	2	5	7
Ncan	0	0	1	0	0	0
Ncaph	71	27	36	63	38	107

Ncbp1	1637	1079	934	1343	820	1565
Nccrp1	0	0	0	1	0	0
Nck2	539	389	308	529	383	688
Nckap5l	299	199	138	279	169	384
Ncln	2005	1291	875	1565	927	1598
Ncoa1	2231	1700	1434	2032	1284	2017
Ndc80	24	18	13	18	18	69
Ndst1	3308	2059	1544	2458	1711	2221
Ndufb4	1064	592	485	830	478	687
Necab2	0	0	0	1	0	0
Negr1	285	185	101	222	82	176
Nek1	851	621	559	723	476	778
Neo1	4230	3013	2341	3508	2546	3258
Nespas	0	0	0	0	1	0
Neto1	0	0	0	1	0	1
Neu1	992	557	486	756	400	878
Neu2	5	2	0	1	0	2
Neu3	285	148	166	234	185	360
Neurl3	189	98	112	152	117	281
Neurl4	2959	1777	1280	2072	1418	2321
Nf1	2159	1578	1354	1823	1265	2126
Nfe2l3	0	0	0	0	0	1
Nhlrc1	501	312	247	414	255	310
Nhlrc2	1198	849	755	1011	715	871
Nhsl2	517	380	273	448	388	698
Nifk	744	576	491	609	410	779
Nipa2	1890	1195	1087	1584	934	1737
Nipal1	2	1	3	2	0	1
Nipsnap1	36	13	18	26	21	57
Nipsnap3b	175	133	111	176	132	265
Nkain1	570	311	420	383	312	578
Nkiras1	1464	1059	1057	1237	853	1631
Nkpd1	0	0	0	0	0	1
Nkx2-2	0	0	1	0	0	0
Nkx2-9	0	1	0	0	0	0
Nkx3-2	0	0	0	1	0	0
Nkx6-1	0	1	0	4	0	1
Nle1	230	95	107	118	95	180
Nlgn2	584	303	243	453	330	619
Nlgn3	54	20	24	24	20	35
Nlrc5	586	248	195	504	278	594
Nlrp10	860	535	478	779	993	721
Nlrp4c	0	0	0	1	0	0
Nlrp4g	1	0	0	0	0	0
Nmbr	2	1	2	3	5	4

Nme8	4	2	3	6	7	5
Nmi	445	298	232	425	248	633
Nmur1	0	0	1	0	0	0
Noa1	891	559	488	702	470	737
Nob1	922	426	379	550	379	664
Nog	16	6	6	8	3	9
Nol10	489	349	262	423	226	421
Nol11	1433	1015	837	1226	784	1265
Nol4	1	0	0	0	0	0
Nop14	1122	917	757	943	676	1131
Nop16	480	331	262	350	237	440
Nop2	881	617	470	704	454	791
Nop58	900	699	617	727	509	959
Nosip	1159	675	562	1013	698	1229
Nostrin	265	179	236	271	221	448
Nov	156	129	84	178	96	231
Npas1	0	0	1	0	0	0
Npm2	9	5	1	3	1	5
Npm3	258	121	147	157	101	234
Npw	1	0	0	0	0	0
Npy	1	1	2	0	0	1
Npy6r	0	0	0	0	0	1
Nr1i2	0	0	0	0	1	0
Nr2f1	162	111	68	156	101	195
Nrbp2	1671	1138	762	1379	1159	1915
Nrcam	20	10	9	12	5	12
Nrde2	586	361	318	499	377	693
Nrxn1	90	90	55	174	43	167
Nsf	1126	864	643	952	603	1066
Nsmce1	1175	675	594	953	707	1465
Nsun3	105	74	73	58	63	97
Nt5dc1	671	476	424	478	379	671
Nt5e	746	644	536	821	682	1037
Ntng1	12	8	5	3	4	9
Ntrk2	30	22	17	41	18	43
Ntsr1	0	0	0	1	0	0
Nucb2	271	260	214	322	214	504
Nudc	2086	1490	1134	1617	1138	1970
Nudcd1	304	230	194	239	180	280
Nudt12	651	360	478	617	301	486
Nudt21	129	96	45	74	50	120
Nuggc	0	0	1	0	0	0
Nup37	223	176	138	183	100	222
Nup62	882	622	456	821	422	651
Nupl2	368	254	208	275	182	337

Nutf2	577	378	315	430	270	553
Nvl	868	612	443	710	422	796
Nxph2	0	0	0	0	0	1
Nxph3	9	2	3	11	7	8
Oas1c	34	28	21	29	27	71
Oas1f	1	0	0	0	0	0
Oasl1	94	43	25	67	44	129
Oasl2	666	496	300	605	342	1213
Obfc1	834	464	393	490	412	692
Oca2	1	0	0	0	0	0
Ocstamp	0	0	0	0	0	2
Odf3l2	0	0	0	1	0	0
Ogdhl	4074	2693	3186	3871	4175	5108
Ogfod1	1911	1251	1111	1591	1085	1497
Ogn	8721	6753	5213	7445	5085	8099
Olfm3	1	0	0	2	1	2
Olfm4	1	0	0	0	0	0
Olfml2b	1052	484	605	698	578	452
Olfr1030	0	0	0	0	1	0
Olfr1031	0	0	0	0	1	1
Olfr1349	0	0	0	0	0	1
Olfr1372-ps1	66	32	44	29	35	60
Olfr1444	0	0	0	0	0	1
Olfr153	1	0	0	0	0	0
Olfr18	1	0	0	2	1	1
Olfr221	0	1	0	0	0	0
Olfr222	0	0	0	0	1	0
Olfr304	0	0	1	0	0	0
Olfr315	1	0	0	0	0	0
Olfr33	0	0	0	0	1	0
Olfr372	0	0	0	1	0	1
Olfr449	0	1	0	0	0	0
Olfr523	0	0	0	1	0	0
Olfr550	7	1	3	3	1	2
Olfr555	0	0	0	1	0	0
Olfr561	0	0	0	0	0	1
Olfr629	0	0	0	1	0	0
Olfr658	1	0	0	0	0	0
Olfr691	0	0	0	1	0	0
Olfr71	1	0	0	0	0	0
Olfr749	0	0	0	1	0	0
Olfr78	172	108	113	118	165	305
Olfr873	1	0	0	0	0	0
Olig1	0	0	0	0	0	2
Omg	0	0	0	0	0	1

Onecut3	3	11	2	4	0	6
Ooep	0	1	0	0	0	0
Oosp3	0	0	1	0	0	0
Opa1	13560	9734	8753	11246	8531	11629
Opn5	0	1	0	0	0	0
Oprm1	0	0	0	0	0	1
Orc3	1987	1396	1186	1565	1131	1916
Orm1	1	3	0	0	0	1
Osbpl10	85	31	35	81	46	86
Osbpl3	1999	1328	1000	1687	950	1591
Osbpl6	1150	833	646	933	698	931
Osgin2	521	319	298	421	239	481
Osmr	1693	1168	771	1624	1036	1869
Ostf1	1439	883	884	1280	910	1868
Otoa	3	1	0	1	2	9
Otof	1	0	0	0	2	2
Otop1	0	0	0	1	0	1
Otos	0	0	1	0	0	0
Otud6b	3659	2447	2375	3035	2141	3297
Ovgp1	122	83	70	41	77	101
Oxsm	1499	944	918	1338	781	1236
Oxt	0	0	1	0	0	0
P2rx2	1	2	3	0	0	2
P2rx5	873	627	489	685	475	774
Pabpc2	0	0	1	0	0	0
Pabpc4l	21	12	6	23	9	35
Pabpc5	1	1	2	0	0	1
Pabpc6	0	0	0	0	0	1
Pabpn1l	6	5	2	1	3	3
Pacsin1	18	20	36	22	9	34
Pacsin2	7078	5229	4414	6119	3988	6339
Palld	6292	5211	3349	5666	3762	4410
Palm	2340	1518	882	2258	1279	2281
Palmd	2805	1961	1800	2470	2744	3188
Pam16	24	14	13	40	10	34
Pamr1	119	87	48	184	60	137
Pank4	2613	1417	1227	1783	1164	2075
Papola	6419	4405	4047	5488	3445	5935
Papss1	512	320	275	485	293	698
Paqr8	158	77	89	146	124	175
Pard3	1875	1311	1106	1601	1087	1472
Parg	1218	876	699	1083	657	1081
Parn	613	409	337	577	428	751
Parp10	759	363	300	684	361	816
Parp4	3029	2518	1809	2646	1836	3033

Pask	21	13	15	29	9	50
Pawr	80	65	43	96	50	122
Pax1	0	0	0	0	1	0
Pax6	0	0	1	0	0	0
Pax8	0	0	0	0	0	1
Pbdc1	431	328	203	357	178	399
Pbld1	28	33	26	26	46	72
Pbp2	1	0	0	0	0	0
Pbx2	863	505	458	760	559	957
Pcdh19	1089	676	647	1060	667	1358
Pcdh7	7332	5472	4177	5756	4519	5855
Pcdh9	103	74	38	80	39	70
Pcdha1	34	14	22	22	14	23
Pcdha10	34	14	22	22	14	23
Pcdha11	34	14	23	22	14	23
Pcdha12	34	15	22	22	14	23
Pcdha2	34	14	22	22	14	23
Pcdha4	35	14	23	22	14	23
Pcdha6	34	14	22	22	14	23
Pcdha7	34	14	22	22	14	23
Pcdhac1	34	14	22	22	14	23
Pcdhb15	18	9	14	14	17	34
Pcdhb16	117	49	53	96	75	142
Pcp2	1	0	0	0	0	0
Pcp4l1	6573	6708	3893	7943	5420	9414
Pcsk2os2	0	1	0	0	0	0
Pcsk6	4464	2860	2156	3693	1529	3765
Pcsk7	964	549	489	840	590	1032
Pcsk9	0	0	0	1	0	0
Pde4c	418	202	183	305	242	463
Pde4d	3481	2475	1876	3036	1766	2718
Pde8a	1214	891	635	889	726	1016
Pdgfd	713	412	376	681	392	840
Pdia3	6768	4803	4721	6101	3539	7208
Pdilt	0	0	0	1	0	1
Pdk3	127	110	71	171	86	179
Pdlim3	645	525	358	768	466	723
Pdlim4	323	180	146	341	150	366
Pdlim7	1108	764	619	1180	653	1367
Pdpr	2124	1720	1375	1925	1473	1182
Pds5a	1970	1445	1196	1736	1101	1514
Peg10	128	84	75	105	59	79
Peg12	29	14	14	35	18	34
Peli2	1132	696	607	1025	672	1409
Pex1	645	410	365	479	341	582

Pfn1	4465	3056	2354	4027	2864	5432
Pfn2	2238	1412	1227	1715	1236	1919
Pga5	0	0	0	0	1	1
Pgap2	2977	1859	1831	2260	1173	3305
Pglyrp1	5	2	4	10	1	15
Pglyrp2	9	4	3	5	1	4
Pgpep1l	5	4	2	7	7	7
Pgrmc1	1187	685	631	1099	667	1432
Phf10	1520	1075	926	1134	925	1381
Phf11c	62	33	21	34	54	89
Phf11d	408	344	211	402	269	615
Phf13	317	241	173	299	205	458
Phf20	1434	1086	808	1434	880	1853
Phf21b	111	84	62	77	120	155
Phgr1	0	0	0	1	0	0
Phkb	3698	2675	2246	3087	2175	3443
Phkg2	594	388	278	513	367	739
Phlda3	307	130	116	286	132	300
Phox2a	19	16	11	9	7	21
Pianp	32	21	20	38	27	42
Piezo2	35	37	21	53	26	59
Pif1	7	0	2	3	1	19
Piga	252	129	122	165	102	208
Pih1d1	577	318	285	491	367	612
Pilrb2	16	7	11	10	2	13
Pim3	2632	1486	1400	1974	1795	3134
Pin1rt1	0	0	0	1	0	0
Pip5k1c	1740	1144	851	1463	1168	1870
Pip5kl1	3	1	0	0	1	0
Pira2	7	2	0	5	1	13
Pitpnc1	4712	3506	2741	4359	3402	5772
Pitrm1	2974	2279	1741	2502	1832	2637
Pkd1l3	1	2	2	5	2	4
Pkdcc	1241	772	616	1174	491	869
Pkdrej	549	386	307	457	310	336
Pkmyt1	45	41	29	51	38	96
Pkn3	555	318	227	440	331	641
Pkp2	6698	4714	3981	5943	5159	8030
Pla2g10	0	1	0	0	0	0
Pla2g2d	55	38	32	48	55	70
Pla2g4b	365	213	138	247	139	278
Pla2g4c	12	3	5	11	6	17
Pla2g4f	0	0	0	0	0	1
Pla2g5	1732	1063	951	1881	1051	1748
Plag1	293	170	163	241	170	420

Plagl1	381	337	185	264	213	353
Plat	787	476	401	718	418	1034
Plcb1	422	338	179	437	276	562
Plcb2	143	90	40	175	70	133
Plch1	0	0	0	0	0	1
Plcxd3	3	1	3	3	0	0
Pld6	8	9	6	12	5	24
Pldi	0	0	0	0	0	1
Plekhh1	38	32	18	29	73	34
Plekhh3	686	341	224	632	353	579
Plet1os	677	572	553	521	432	871
Plgrkt	793	483	498	685	519	1125
Pllp	10	5	4	9	9	15
Plp2	329	217	190	353	199	410
Pls3	3522	2472	2253	3678	2207	4839
Plscr3	955	504	431	702	521	1146
Plscr4	1688	1033	810	1243	1130	2140
Plscr5	0	1	0	0	0	0
Pltp	1350	762	514	1150	647	1603
Plxdc1	221	134	143	225	139	324
Pmaip1	17	19	17	14	6	24
Pmp2	2	2	2	1	0	2
Pms1	255	172	156	174	150	236
Pnck	236	124	90	189	147	253
Pnkp	661	387	294	542	426	701
Pnldc1	11	8	7	11	9	27
Pnlip	0	0	0	1	0	0
Pnmal1	0	0	0	2	0	0
Pnmt	18	5	5	11	13	21
Pnn	3188	2600	2089	2539	1985	3613
Pno1	1718	1159	1142	1337	972	1735
Pnp2	4	4	1	6	6	6
Podnl1	3	2	1	1	1	1
Podxl2	347	241	166	265	150	316
Polb	1434	1006	855	1102	744	1506
Pold4	246	171	120	232	166	294
Pole	209	140	93	181	128	274
Polg	3341	2038	1587	2666	1502	2744
Polk	467	298	231	379	321	555
Polr2m	13261	8987	7700	10848	7083	11581
Por	2221	1689	1196	1759	1196	2218
Pot1b	223	200	159	222	187	373
Pou3f3	0	0	0	1	0	0
Pou5f2	6	4	2	4	5	15
Pou6f1	722	487	364	617	457	965

Ppapdc1a	0	0	0	0	0	1
Ppef1	1	0	0	4	1	0
Ppfia1	3049	2157	1692	2280	1853	2221
Ppfia3	8	6	6	6	4	4
Pphln1	1260	914	768	1071	685	1175
Ppip5k2	15337	9514	6897	12791	9153	18971
Ppm1e	1415	1294	1041	1477	807	1371
Ppm1m	378	200	202	323	216	459
Ppme1	2123	1478	1160	1815	1119	1853
Ppp1r1b	47	33	34	49	63	48
Ppp1r2	2942	1644	1606	2611	1764	3347
Ppp1r21	624	405	334	561	426	753
Ppp1r3c	6620	4759	4224	6322	3540	4496
Ppp2r5e	2859	2000	1688	2386	1597	2615
Ppp3cb	5123	3134	2724	4054	2504	4202
Ppp3cc	1563	1114	973	1207	915	1497
Ppp4r1l-ps	227	124	123	145	178	360
Ppp4r4	44	21	20	44	30	51
Pptc7	10525	6639	6882	8942	5535	8342
Pramel4	0	0	0	0	0	1
Prdm1	167	88	123	184	130	217
Prdm12	0	0	0	0	1	0
Prdm14	0	0	0	2	0	0
Prdm6	41	15	9	17	9	21
Prdm9	199	168	108	102	122	197
Prep	2284	1656	1276	1895	1211	1990
Prex1	391	275	191	393	238	464
Prg3	0	0	1	0	0	0
Prkar2a	5232	3696	3040	4497	2871	3556
Prkcdbp	524	371	295	591	302	683
Prkg2	26	11	20	31	20	40
Prkrir	6318	4246	4052	5353	3594	5707
Prl5a1	0	0	1	0	0	0
Prl6a1	0	0	1	0	0	0
Prl7a2	0	1	0	0	0	0
Prl7c1	0	1	0	0	0	0
Prlhr	0	0	0	1	0	0
Prm1	0	1	0	0	0	0
Prmt3	761	433	405	575	373	570
Prmt5	1814	1280	1020	1435	998	1581
Prok2	0	0	0	0	0	1
Prph	14	3	5	5	1	8
Prpsap1	844	592	435	809	529	1012
Prr13	975	687	556	1052	597	1146
Prr19	0	0	0	1	0	1

Prr30	0	0	0	1	1	0
Prr32	0	0	0	1	1	0
Prrt1	36	30	15	23	15	29
Prrt3	0	0	0	0	0	2
Prrt4	143	74	38	130	82	123
Prss22	0	0	0	0	0	2
Prss39	0	0	0	0	1	0
Prss41	0	0	0	0	1	0
Prss44	0	0	0	1	0	0
Prss8	0	0	0	0	0	1
Prx	74	42	26	85	51	72
Psenen	48	29	45	30	29	41
Psg22	1	0	0	0	0	0
Psmb11	1	0	0	0	0	0
Psmb3	1229	703	674	1104	692	1454
Psmb4	3729	1918	1771	3219	1859	4432
Pspc1	767	615	441	641	396	762
Ptcd3	6303	4787	4059	5387	3704	5887
Ptchd1	20	7	12	16	17	28
Ptchd2	0	0	0	1	0	1
Ptchd4	0	0	1	0	3	2
Ptcra	1	0	0	0	0	0
Ptger4	385	216	228	288	282	555
Ptgs2os	1	0	3	0	0	1
Pth2r	1	0	0	0	0	0
Ptma	886	520	489	886	475	1093
Ptplad1	1361	840	810	1126	705	1119
Ptpn12	1861	1207	1062	1821	1210	2097
Ptpn18	78	54	31	38	22	78
Ptpn20	0	0	1	2	2	0
Ptpn4	554	363	330	446	274	537
Ptpn5	1	0	0	0	0	0
Ptpre	185	104	123	159	126	310
Ptprj	471	345	241	404	174	415
Ptprk	1578	1069	754	1405	1030	1656
Pvalb	15	17	6	17	1	9
Pwp2	733	439	401	505	375	630
Pxdc1	443	222	226	357	212	616
Pycard	292	158	154	231	120	208
Pydc3	78	74	44	106	45	144
Pydc4	17	12	5	22	5	35
Pygo1	375	291	233	302	265	206
Qpct	236	134	102	155	75	213
Qprt	0	1	2	3	1	5
Qrich2	0	1	0	0	0	0

Qrsl1	1393	811	829	1148	654	1205
Qser1	4453	3158	2777	3597	2665	3740
Qsox2	746	421	426	509	375	630
Qtrt1	197	167	127	138	105	230
Qtrtd1	647	387	310	426	300	591
Rab10	9580	6914	5857	8094	5324	8458
Rab11fip4	3	2	2	2	0	1
Rab37	39	32	24	43	23	82
Rab3a	1593	1076	783	1589	1058	1686
Rab3b	25	16	22	27	29	39
Rab8a	955	736	562	985	686	1142
Rabl6	4439	3063	2440	3480	2412	3747
Rac3	31	13	15	24	22	44
Racgap1	95	36	44	80	40	141
Rad18	67	50	32	54	48	112
Rad51	20	15	14	21	14	49
Rag1	0	0	0	0	0	1
Rag2	0	0	0	0	1	0
Rai1	1663	1254	886	1430	960	1309
Rala	2352	1580	1637	1941	1349	2447
Ralgapb	3569	2440	2238	3255	2045	2893
Ralgds	628	464	326	560	403	841
Ranbp3l	0	0	0	0	1	0
Rap1gap2	3952	2225	2109	3071	2254	2315
Rapgef2	2558	1839	1396	2753	1592	2968
Rars	2031	1454	1282	1651	1115	2054
Rasal3	46	26	17	37	12	23
Raver2	153	126	116	141	86	148
Rbak	407	295	214	339	278	605
Rbbp5	1302	874	760	1086	713	1159
Rbfox3	0	0	1	0	3	1
Rbm15	640	376	365	499	345	468
Rbm26	1256	979	822	1066	750	1313
Rbm28	2100	1143	1112	1494	1033	1695
Rbm43	291	152	155	255	164	349
Rbm46os	0	0	1	0	0	0
Rbp2	0	1	0	3	1	0
Rcsd1	5310	4222	3185	5519	3903	6510
Rdh12	8	5	7	12	7	15
Rdh16	0	1	0	0	2	3
Reep5	20930	13915	11794	18114	9638	18151
Reg3b	0	0	0	1	0	0
Ren1	0	1	0	0	0	0
Ren2	0	1	0	0	0	0
Renbp	107	73	49	98	72	163

Rerg	211	130	122	236	120	288
Resp18	1	0	0	0	0	0
Rex2	0	0	0	0	0	1
Rffl	446	197	179	334	237	517
Rfpl3s	0	0	0	0	0	1
Rftn2	365	280	234	448	243	445
Rfx3	271	168	116	243	161	328
Rfx6	0	0	1	0	0	0
Rgl2	1768	926	692	1401	933	1907
Rgs1	1	1	0	4	0	4
Rgs19	187	101	73	101	66	147
Rgs7	215	158	98	142	82	199
Rgsl1	9	6	5	8	12	13
Rhbg	1	0	0	0	0	0
Rhd	61	17	27	21	124	28
Rhobtb2	1628	1093	857	1366	813	1348
Rhov	0	0	0	0	0	1
Rhox2b	0	0	0	1	0	0
Rhox3f	0	0	0	1	0	0
Rhox5	44	41	32	67	34	81
Rhox8	0	1	0	0	0	0
Rian	233	172	146	188	91	261
Ric8	929	507	454	761	516	1049
Rictor	1803	1012	822	1386	1041	2009
Rilpl1	10453	7371	5935	8319	5714	8985
Rimkla	81	45	45	38	42	71
Rims2	7	7	3	8	7	17
Ripk3	74	38	33	59	36	124
Ripply3	260	146	123	217	157	336
Rlbp1	0	0	0	1	0	0
Rln1	0	0	0	0	1	0
Rln3	1	0	0	0	0	0
Rmdn2	663	447	432	539	368	595
Rnaseh2c	251	138	124	215	141	315
Rnaset2a	59	34	26	63	29	92
Rnaset2b	93	49	47	88	46	143
Rnf112	0	0	0	1	0	0
Rnf122	268	168	137	209	218	335
Rnf148	1	0	0	0	0	0
Rnf165	27	14	7	21	25	26
Rnf167	2226	1286	1084	1874	1287	2593
Rnf183	0	0	0	0	1	0
Rnf20	2567	1905	1510	2122	1477	2451
Rnf219	237	158	117	175	109	177
Rnf26	147	89	51	136	84	154

Rnf34	1765	1279	1069	1531	1025	1607
Rnf43	2	2	3	6	4	5
Rnh1	1495	864	717	1361	809	1597
Rnps1	528	453	362	700	350	724
Rnu11	0	4	0	1	0	0
Rnu73b	6	5	2	3	5	19
Robo2	69	40	24	69	44	74
Robo3	3	0	0	5	1	3
Ror2	83	62	31	68	22	66
Rp1l1	0	0	0	0	1	1
Rpf2	542	416	370	425	344	552
Rpgrip1	5	11	4	7	10	21
Rpl10a	54	16	30	18	19	40
Rpl27	15	16	13	22	14	36
Rpl35a	1452	1444	1434	1515	1075	1812
Rpl36	93	57	64	69	42	77
Rpl37	1153	651	657	1048	621	1536
Rpl9	262	175	166	235	96	266
Rpp38	158	106	90	106	82	130
Rprd1b	1843	1201	978	1519	966	1540
Rps14	5099	3003	2644	4508	2772	6881
Rps27	7	4	10	9	14	16
Rps27rt	4	1	3	6	4	5
Rps6ka1	292	160	121	245	152	380
Rps6ka4	846	501	373	732	479	891
Rptor	2100	1413	1141	1722	1185	1653
Rragb	116	98	74	92	65	105
Rras	1220	809	632	1165	723	1541
Rreb1	3614	2296	2076	2627	2289	2725
Rrh	0	0	0	1	0	1
Rrp15	212	189	150	154	123	231
Rsad1	955	638	530	719	568	781
Rsf1	1903	1175	934	1548	1253	2024
Rsph6a	0	0	0	0	0	1
Rsu1	1554	1115	809	1505	976	1866
Rtn2	2597	1556	1193	2459	1444	2708
Rtn4ip1	4354	2796	2546	3544	2284	3613
Rtn4r	174	93	54	111	62	76
Rtp3	272	143	119	187	191	307
Rttn	390	271	239	308	226	347
Rubie	0	0	0	1	0	1
Rufy4	112	71	47	68	46	96
Rwdd3	75	34	23	53	40	93
Rxfp4	0	0	0	0	0	1
Rybp	292	193	171	250	135	236

S100a13	582	398	384	573	400	792
S100a7a	8	6	5	4	1	7
Sall2	134	98	85	159	82	193
Samd9l	2181	1367	990	2044	1106	2604
Sat1	918	694	552	919	565	1305
Sbk2	125	62	62	120	112	110
Sbno1	4827	3524	3092	3619	3024	4668
Sc5d	839	631	504	669	495	794
Scarb2	5912	4202	3840	5139	3184	5547
Scarna17	31	16	18	14	13	28
Scarna6	0	0	0	0	1	0
Scarna9	0	0	0	2	0	0
Scd2	1668	1435	1011	2088	1285	1900
Scgb1a1	0	0	0	0	0	1
Scml2	53	47	17	25	27	43
Scn3b	75	42	38	54	27	56
Scnn1g	1	0	0	0	0	0
Sco2	387	230	249	292	178	379
Scrn3	2057	1467	1277	1608	1295	1867
Scrt1	0	0	0	1	0	0
Scube2	431	326	405	487	258	376
Scube3	4	5	2	14	3	6
Sdad1	764	600	486	612	492	645
Sdk2	13	11	12	20	10	34
Sdpr	11675	7732	6386	10552	7782	14604
Sdr9c7	0	0	0	0	0	1
Sec14l1	4711	2959	2343	4203	2897	5553
Sec14l2	29	32	24	42	24	63
Sec14l3	0	0	1	0	0	0
Sec24a	1241	921	627	949	707	1118
Sel1l3	28	23	22	49	24	34
Sema4c	601	328	340	559	346	723
Senp1	897	563	412	821	542	945
Senp2	1702	1128	1050	1296	935	1686
Senp6	4020	3089	2486	3242	2552	3673
Senp8	429	347	293	402	254	383
12-Sep	0	0	1	0	0	0
14-Sep	0	0	0	0	1	0
6-Sep	281	183	144	250	274	279
Serf2	5678	3862	3574	4709	3063	5438
Serhl	917	610	551	813	582	1286
Serpina1b	2	4	1	4	6	4
Serpina1d	2	2	0	4	1	4
Serpina3m	0	0	3	0	0	1
Serpina5	0	0	0	1	0	0

Serpina9	8	3	5	1	5	2
Serpinb1a	160	114	68	168	98	208
Serpinb1c	17	10	9	13	15	32
Serpinb6b	2003	1367	1165	1684	1751	2228
Sertad3	149	96	72	149	83	203
Sertm1	136	68	44	106	12	71
Sestd1	2177	1589	1174	1715	1197	2062
Setd8	9465	6954	6899	8441	5874	8242
Sfrp2	99	105	63	142	72	196
Sfrp5	57	45	26	47	63	68
Sftpc	0	0	0	0	0	1
Sgcb	10699	7613	5792	8847	5703	9066
Sgcd	758	554	563	733	468	461
Sgol1	16	7	8	12	12	34
Sgol2	872	616	471	681	681	1100
Sgpp2	5	2	3	4	7	10
Sgsm1	34	11	14	32	25	29
Sh3bgrl	2983	1917	1855	2726	1862	3926
Sh3bgrl2	276	157	131	231	193	306
Sh3bp5	1411	1140	981	1249	1379	1965
Sh3gl1	556	293	260	521	297	569
Sh3rf2	1476	712	624	809	1113	1605
Shank1	104	77	64	75	67	78
Shisa5	1476	1038	757	1507	821	1913
Shisa6	10	8	8	12	25	8
Shisa7	4	1	2	1	0	2
Shkbp1	301	152	137	230	186	352
Shpk	98	49	50	81	66	132
Sidt1	54	22	21	37	35	77
Siglech	7	3	10	4	3	6
Sim1	1	0	0	0	0	0
Skap2	392	218	200	309	244	469
Skint4	0	0	0	1	0	0
Skint8	0	0	0	0	0	1
Skiv2l2	2295	1705	1417	2010	1257	2271
Slamf1	33	22	15	14	14	28
Slamf9	82	43	39	75	40	142
Slc10a1	0	0	0	2	0	0
Slc12a2	2744	1527	1532	2345	1795	3165
Slc12a5	53	35	45	50	49	100
Slc13a2	0	0	0	0	1	1
Slc13a3	22	14	2	11	3	13
Slc13a5	0	1	0	1	1	2
Slc15a5	0	0	0	0	0	1
Slc16a4	54	11	23	34	24	87

Slc16a7	317	245	172	259	175	246
Slc19a1	140	78	55	82	54	105
Slc22a14	0	0	0	0	1	0
Slc22a29	1	0	0	0	0	0
Slc22a3	290	224	218	242	167	316
Slc22a30	0	0	0	1	0	1
Slc25a1	183	143	126	174	139	301
Slc25a19	1374	872	777	1107	686	1182
Slc25a23	3131	2575	1690	2770	1515	2994
Slc25a29	403	204	192	335	130	269
Slc25a30	260	167	170	279	181	358
Slc25a38	764	459	402	619	356	625
Slc25a46	4427	3129	2728	3854	2530	3995
Slc25a47	163	71	94	102	66	126
Slc26a6	341	192	149	272	119	221
Slc2a10	47	36	37	48	35	103
Slc2a5	8	6	6	5	5	2
Slc2a8	449	247	258	435	267	559
Slc30a7	1022	640	501	897	627	1155
Slc32a1	0	0	0	0	1	0
Slc35a4	1393	863	744	1151	662	1104
Slc35b3	243	137	101	212	132	324
Slc35f3	28	12	1	12	6	7
Slc35g3	0	0	0	1	1	0
Slc38a8	0	0	0	1	1	0
Slc39a4	3	3	3	2	0	3
Slc41a2	64	40	33	71	31	106
Slc44a4	0	0	0	0	0	1
Slc44a5	19	6	12	18	17	28
Slc45a3	175	114	107	211	98	272
Slc4a4	2007	1299	1246	1970	1757	2084
Slc4a8	348	236	156	313	216	456
Slc50a1	715	413	354	610	430	825
Slc51b	0	0	0	0	0	1
Slc5a4a	1	0	0	0	0	0
Slc5a8	1	0	0	0	0	0
Slc5a9	0	0	0	0	1	0
Slc6a11	0	0	1	0	0	0
Slc6a12	1	0	0	1	1	2
Slc6a15	9	6	4	4	1	6
Slc6a19os	0	0	0	0	1	0
Slc6a3	0	0	0	0	1	0
Slc6a4	21	7	5	4	10	7
Slc9a5	124	78	50	91	76	178
Slc9a9	168	130	104	127	79	170

Slc9b1	2	2	1	3	3	7
Slco1b2	2	1	0	4	3	1
Slco5a1	1547	981	807	1010	945	1088
Slfn14	3	4	1	1	0	2
Slfn2	360	222	153	365	163	533
Slfn5os	5	3	9	5	1	4
Slitrk1	0	0	0	1	0	0
Slmo1	13	4	5	6	4	2
Smap2	2673	1862	1649	2223	1393	2616
Smarcc1	1620	1171	929	1359	898	1337
Smarce1	1489	1192	878	1188	893	1502
Smc1b	5	1	4	0	1	4
Smc6	3471	2659	2199	2898	2136	3468
Smcr8	1810	1218	1028	1582	928	1378
Smg7	3791	2740	2047	2973	2216	3131
Smim1	118	81	80	127	80	185
Smim13	2612	1528	1565	2204	1344	2086
Smim18	0	0	0	0	0	2
Smim4	133	77	84	123	93	200
Smim9	0	0	1	1	1	3
Smoc1	231	196	110	152	96	272
Smurf2	3096	2184	1919	2741	1754	2662
Smyd3	288	180	122	266	171	305
Smyd4	283	179	143	237	215	314
Smyd5	343	256	230	282	199	327
Snai2	203	152	114	244	120	264
Snap91	595	461	453	595	281	659
Sncb	0	0	0	1	0	0
Snhg11	29	11	16	11	39	36
Snhg18	229	115	133	241	136	278
Snora26	7	1	2	2	2	1
Snora43	13	3	7	1	3	10
Snora52	18	17	9	6	10	17
Snora61	12	5	5	9	7	23
Snord11	0	0	0	0	0	1
Snord15b	5	2	2	0	1	3
Snord19	7	9	0	3	1	3
Snord1c	0	0	0	1	0	0
Snord42a	1	0	0	1	3	1
Snord43	1	0	0	2	1	1
Snord49a	3	1	2	1	1	1
Snord49b	0	0	0	0	1	1
Snord52	3	1	2	0	0	2
Snord58b	0	1	0	1	1	3
Snord61	0	0	0	0	0	1

Snord70	0	0	0	0	0	1
Snord90	1	0	0	0	0	0
Snord96a	20	9	14	24	11	36
Snrnp35	193	130	117	164	159	254
Sntg1	8	1	0	0	1	2
Sntg2	5	2	1	7	3	7
Sntn	0	1	0	0	0	0
Snupn	404	274	270	328	176	413
Snx21	596	375	296	544	419	585
Snx25	731	433	346	660	432	760
Snx27	2222	1340	1184	1748	1117	1711
Snx5	3478	2318	1951	2744	1871	3361
Soga3	0	0	0	1	0	1
Sorbs3	1913	1225	961	1757	1106	2384
Sord	8010	4601	4628	5533	3647	7749
Sos2	3759	2582	2139	2946	2218	3213
Sox10	89	63	41	61	42	52
Sox21	0	0	0	1	0	0
Sox2ot	6	1	2	1	3	0
Sox5	134	91	80	108	51	130
Sp5	0	0	0	0	0	1
Spaca5	0	1	0	0	0	0
Spaca6	104	80	55	92	85	169
Spag4	8	6	6	8	12	15
Spag5	26	21	19	32	14	74
Spag6	0	0	0	0	0	2
Spata22	0	0	0	0	0	1
Spata31d1a	0	0	0	0	1	1
Spata9	0	0	0	0	1	1
Spats1	6	4	10	7	2	5
Spc25	30	18	10	20	15	56
Spcs3	3914	2478	2437	3218	1990	3561
Speer4a	0	0	0	1	0	0
Speer8-ps1	0	0	0	0	0	1
Speg	11742	7257	5362	8769	5533	8882
Spesp1	0	0	0	1	0	0
Spg21	503	313	285	458	333	602
Spice1	571	355	295	432	282	476
Spin4	172	74	46	79	47	118
Spink14	0	0	0	0	1	0
Spon1	1564	867	889	1052	651	1583
Spred1	1957	1370	1162	2038	1164	2447
Sprr2f	0	0	0	1	0	1
Spryd7	2800	1916	1450	2343	1418	2245
Spsb4	377	242	281	388	149	302

Spta1	658	715	328	743	547	1016
Sptssb	0	0	1	0	0	0
Src	172	117	81	200	98	213
Srf	3695	2341	2087	3210	1942	2929
Srl	55457	37380	35792	49667	29697	47010
Srp72	3475	2610	1927	2715	1984	3365
Srpk2	3955	3035	2503	3408	2328	3989
Srpx2	222	158	150	252	134	357
Srsf3	3339	2014	1868	2428	1543	3219
Srsf5	4792	3081	2680	3672	3390	6476
Srxn1	1219	736	754	1012	579	1085
Ssbp2	1795	1415	1164	1629	1056	1766
Ssfa2	2543	1534	1095	2419	1315	2612
Sspn	14255	9640	8613	11996	8473	11000
Sstr3	1	0	1	1	3	2
Ssxb9	0	0	0	0	0	1
St3gal6	1671	1121	1022	1487	877	1502
St5	1858	1122	958	1664	1150	1968
St7l	688	481	421	591	352	568
Stac	12	12	9	24	15	15
Stard13	2347	1554	1187	2021	1565	2592
Stard3	627	379	287	502	392	700
Stard4	297	172	159	267	173	390
Stat5a	1077	782	564	907	555	978
Stau2	4006	2956	2667	3224	2518	3623
Stfa2	0	0	0	0	0	1
Stfa2l1	0	0	0	1	0	0
Stk11ip	594	370	282	579	371	659
Stk19	163	106	92	150	113	219
Stk24	2106	1538	1252	1799	1249	1704
Stk4	1211	952	723	1093	705	1011
Stmnd1	13	9	4	6	1	11
Stox1	55	31	23	57	45	53
Stpg2	0	0	0	0	1	0
Stradb	2985	1646	1941	2192	1518	2877
Strc	4	2	0	6	3	3
Strn3	7922	5404	4935	6852	4515	7058
Stt3b	3652	2506	2389	3298	1938	3532
Styk1	10	1	3	2	2	3
Styxl1	0	0	1	1	3	1
Sugct	5	5	5	11	3	16
Sulf1	1538	1242	833	1505	591	1340
Sult1c1	0	1	0	0	0	0
Sult2b1	0	1	0	0	0	0
Sult4a1	5	2	2	0	1	3

Sult5a1	16	9	24	29	16	41
Sult6b1	9	6	7	4	4	8
Supv3l1	1955	1299	1203	1568	1147	1793
Suv39h1	334	182	136	296	167	367
Syce1l	23	10	7	9	7	14
Syce3	0	1	0	0	0	0
Sycp1	0	0	0	0	0	1
Syna	1	0	0	0	0	0
Syne4	7	6	2	9	7	10
Synj2bp	4409	3027	2702	3566	2553	4217
Synpo2	6194	4980	3711	5618	3609	3692
Synpr	0	0	0	1	1	0
Syt14	0	0	0	0	1	0
Syt15	44	27	17	45	33	53
Syt6	2	0	2	0	0	1
Syt7	2810	1817	1380	2080	1387	2314
Sytl5	0	5	1	3	3	9
Tac2	0	0	0	0	0	1
Tacc1	4322	2969	2421	3930	2922	5015
Taf6	1417	1005	762	1198	746	1246
Tal1	299	194	194	270	204	425
Taok2	3149	2068	1448	2675	1999	3639
Tapbp	3184	1750	1599	2881	1700	3624
Tapbpl	307	175	120	313	168	307
Tars2	1978	1208	1052	1526	932	1704
Tas1r1	2	0	0	0	3	5
Tas1r2	0	0	0	1	0	0
Tas2r137	0	0	0	1	0	0
Tas2r143	1	0	0	2	2	1
Tat	0	0	3	1	0	0
Tbc1d1	987	638	563	919	663	1094
Tbc1d17	1011	577	405	815	577	1031
Tbc1d2b	1101	681	596	1086	683	1274
Tbc1d30	90	55	52	96	71	95
Tbc1d4	6001	3548	3196	3837	3398	5175
Tbx1	56	34	16	24	23	33
Tbx10	0	0	0	0	1	0
Tbx6	73	35	22	33	25	50
Tcap	37922	20909	15460	29631	23056	41850
Tcea3	4445	3192	2337	3782	3291	5186
Tcf23	214	101	99	155	115	314
Tcf7l2	637	453	354	521	478	804
Tcof1	1036	807	590	869	575	983
Tcp10a	0	1	0	0	0	0
Tctn1	315	210	165	267	197	426

Tdgf1	1	1	2	4	2	3
Tdh	0	0	0	0	0	1
Tdrd5	1	0	0	0	0	0
Tdrd7	896	590	456	817	540	1135
Tead1	11889	8367	7460	9051	7610	8420
Tead2	270	175	109	267	152	316
Tecpr1	1596	1060	793	1483	971	1764
Tecrl	4112	2835	2273	3756	2954	4685
Tekt4	3	1	1	3	3	6
Tenc1	8963	6155	4075	7737	5701	10495
Tenm1	6	1	1	2	0	2
Tenm4	36	27	15	28	13	20
Tescl	0	0	0	0	0	1
Tex12	0	0	0	0	0	1
Tex14	6	9	6	10	14	15
Tex19.1	0	0	0	0	0	1
Tex2	2841	1898	1720	2434	1655	2176
Tex21	0	0	0	0	1	0
Tex26	4	2	3	3	0	2
Tex29	0	0	2	4	4	0
Tex33	0	0	0	0	1	0
Tex9	139	85	111	115	121	237
Tfap2c	1	0	0	0	0	0
Tfb1m	431	291	219	319	212	386
Tfb2m	851	677	584	660	583	832
Tfeb	1085	653	481	1147	584	1047
Tfpi	5699	3830	3100	4573	2798	5334
Tgfb3	849	618	505	1046	542	974
Tgm4	4	3	0	0	0	3
Thap1	422	259	221	320	188	370
Thap3	357	158	172	261	222	392
Thap6	432	296	244	316	248	369
Thbs2	1449	862	800	1469	822	1659
Them4	682	506	497	633	373	673
Themis3	0	0	1	0	0	0
Thsd4	554	363	213	430	208	377
Tiam1	312	230	155	248	138	271
Timm10	1355	913	943	1074	674	1550
Timm8a2	0	0	0	0	0	1
Timmdc1	2095	1404	1296	1644	1139	2059
Tjp1	8370	5844	4131	7610	5545	9218
Tjp2	1558	1130	869	1439	1115	1925
Tjp3	3	7	0	2	1	1
Tll2	33	11	10	7	3	29
Tlr3	835	539	508	754	570	1105

Tlr7	155	113	108	135	56	174
Tlr8	95	68	30	70	21	67
Tlx2	0	0	0	0	1	0
Tm4sf19	0	0	1	0	2	3
Tm9sf1	1739	1069	856	1418	746	1527
Tmbim1	2307	1672	1255	2337	1538	2783
Tmbim4	1526	896	840	1377	910	1710
Tmc6	453	219	165	387	219	522
Tmc8	127	74	58	103	75	179
Tmcc3	4062	2387	2097	3326	2494	4648
Tmed6	0	0	0	0	0	1
Tmem107	127	67	44	83	32	80
Tmem120b	315	203	176	231	142	319
Tmem129	1180	760	651	1047	543	1023
Tmem132e	10	17	4	12	0	10
Tmem135	2741	1837	1527	2280	1418	2486
Tmem150a	1171	666	466	742	525	947
Tmem159	765	401	439	682	503	883
Tmem160	609	272	287	522	317	679
Tmem170b	1800	1048	961	1345	968	1472
Tmem179	2	2	2	0	2	0
Tmem181c-ps	3	1	3	0	2	1
Tmem182	3408	2365	2086	3320	1730	2749
Tmem184a	2	3	1	2	0	0
Tmem184c	1288	1071	835	1071	752	1359
Tmem19	763	426	377	657	491	802
Tmem198b	353	195	136	298	215	388
Tmem2	969	680	598	843	701	1436
Tmem203	126	66	46	119	71	144
Tmem207	0	0	0	0	0	1
Tmem236	9	3	1	5	1	1
Tmem237	174	96	64	178	73	209
Tmem246	2154	1263	1196	1888	944	1748
Tmem25	498	348	299	407	246	444
Tmem251	403	266	227	311	196	383
Tmem253	40	44	27	33	30	23
Tmem27	0	0	1	0	0	0
Tmem28	1	1	0	2	3	1
Tmem30b	14	10	10	13	5	7
Tmem35	510	356	291	431	148	528
Tmem55a	1539	987	888	1269	790	1440
Tmem59l	0	0	0	1	0	1
Tmem62	339	202	178	262	158	280
Tmem64	3047	1867	1426	2342	1380	2431
Tmem69	1317	923	857	1385	807	1734

Tmem70	5035	2940	2898	3773	2516	4669
Tmem71	829	588	441	768	494	1211
Tmem81	99	52	45	84	68	127
Tmem86a	442	251	212	377	287	501
Tmigd1	20	12	11	14	19	40
Tmod2	661	438	299	517	260	559
Tmprss11a	1	0	0	0	0	0
Tmprss2	1	0	0	0	0	0
Tmprss3	0	1	0	0	0	0
Tmprss4	0	0	0	0	0	1
Tmprss6	0	0	0	0	0	2
Tmprss7	0	0	0	1	1	0
Tmsb15b2	6	6	4	9	6	12
Tnfaip1	3238	1929	1907	3046	1921	4024
Tnfrsf13b	2	1	1	1	3	6
Tnfrsf1b	436	263	193	453	260	439
Tnfrsf8	0	1	0	1	0	5
Tnfsf12	703	442	346	636	417	863
Tnk2	789	454	368	687	530	811
Tnn	8	1	0	1	1	1
Tomm6	1033	638	570	930	599	1251
Topaz1	0	0	0	0	0	2
Tor2a	257	122	99	219	143	268
Tpbpb	1	0	0	0	0	0
Tpi1	21578	14429	12511	21921	13828	25506
Tpk1	102	67	52	114	63	135
Tpo	0	0	0	0	1	0
Tpsg1	0	0	0	0	0	1
Tra2a	2122	1321	1145	1490	1022	2119
Traf3	1217	772	694	984	659	996
Trak1	12724	8975	6919	10749	6845	9909
Trak2	16543	11656	11414	14253	10060	13630
Trank1	3	4	1	1	1	2
Trap1a	1	0	0	0	0	0
Trappc13	1896	1297	1120	1572	1042	1798
Trappc6b	1690	1156	1127	1284	1010	1775
Trem3	1	0	0	0	0	0
Treml1	14	8	9	9	7	5
Trh	1	0	0	0	0	0
Trim12c	900	554	475	797	512	1225
Trim14	184	100	89	166	104	231
Trim16	182	118	120	143	228	230
Trim21	439	253	227	392	264	530
Trim24	2179	1578	1443	1973	1635	3083
Trim30d	206	107	92	162	107	299

Trim30e-ps1	0	0	1	0	0	0
Trim34b	4	5	3	3	3	1
Trim38	5	6	7	4	2	7
Trim47	781	455	322	826	368	808
Trim62	91	64	46	82	64	137
Trim66	17	20	8	26	18	24
Triqk	155	104	82	160	93	218
Trmt10b	230	164	129	167	122	214
Trmt2b	3698	2485	2090	3138	1964	3207
Tro	69	74	29	56	33	55
Trove2	2150	1438	1224	2284	1359	2404
Trp53inp1	1397	748	955	1209	870	1965
Trp53inp2	35456	20839	21383	28591	18329	27033
Trp53rk	68	31	30	47	19	38
Trpc3	503	271	241	459	288	507
Trpv3	2	0	2	5	0	6
Trub1	534	399	318	344	289	567
Trub2	1143	630	519	703	500	1026
Tsc1	2489	1685	1395	1973	1447	2065
Tsc22d1	10227	5369	4832	8799	6532	9154
Tsen15	358	250	241	286	176	348
Tsks	0	1	0	0	0	0
Tspan1	0	0	0	0	0	2
Tspan2os	0	0	1	0	0	0
Tspan32	13	11	7	7	4	12
Tspan5	330	190	122	286	203	351
Tspan8	8	5	4	8	13	11
Tspo2	4	2	3	0	0	5
Tspyl4	971	739	677	949	535	861
Ttc12	1251	827	560	985	1197	1136
Ttc19	1979	1348	1508	1715	1241	1909
Ttc21a	0	0	0	1	0	1
Ttc21b	535	405	315	502	289	421
Ttc23	366	265	222	379	275	437
Ttc39a	94	87	95	72	56	133
Ttc39b	2009	1324	1098	1420	1110	1746
Ttl	1599	1073	1028	1297	980	1446
Ttll1	2121	1240	1065	1636	938	1747
Ttll11	79	56	56	67	29	73
Ttll2	0	0	0	0	0	1
Ttll3	413	234	203	359	224	528
Ttpa	36	22	23	29	26	76
Ttyh2	173	104	110	164	117	277
Tuba3a	0	0	0	0	1	0
Tuba3b	5	2	0	5	3	6

Tuba4a	24655	16869	13523	22604	9304	22270
Tubb3	20	24	18	28	18	51
Twsg1	6261	4347	3613	5421	3268	5394
Txlng	698	464	378	477	367	676
Txndc12	1308	852	857	1085	595	1459
Txnrd1	2557	1737	1598	2153	1418	2257
Tyk2	891	558	358	773	552	926
Tyr	0	0	0	1	0	0
Uba5	1498	994	917	1064	819	1596
Ubb	4069	2444	1840	3437	2344	4574
Ube2d3	14120	10993	9552	11416	8971	14456
Ube2g1	5734	3738	3334	4481	3201	5138
Ube2j2	765	503	448	762	485	911
Ube2l6	532	478	288	436	282	572
Ube2q2	1476	1008	955	1222	855	1423
Ube2ql1	501	352	331	431	269	455
Ube2t	173	69	77	94	58	113
Ube2v2	269	181	150	194	123	252
Ubxn2a	3904	2920	2799	3501	2559	3062
Ubxn4	3947	2888	2462	3148	2391	3874
Ubxn7	2133	1436	1373	1835	1498	2904
Uchl4	5	4	5	4	3	2
Uck2	699	503	564	560	386	786
Ucn	3	4	0	1	0	2
Ucn3	0	0	0	0	2	0
Ufc1	1697	1011	880	1477	964	2216
Ufsp1	172	142	137	155	98	191
Ugt8a	0	0	0	1	0	1
Uhrf1bp1l	2756	1970	1581	2131	1615	2563
Unc45b	10114	6596	6992	9400	4946	9384
Unc79	4	2	6	2	2	2
Upb1	6	13	5	2	7	3
Urb1	604	380	271	422	318	456
Ush2a	5	1	3	2	1	1
Usmg5	1016	706	514	753	422	1034
Usp10	1828	1294	1084	1544	1023	1546
Usp15	4499	3364	2883	3601	2848	3945
Usp17la	0	0	0	0	1	0
Usp17lc	0	0	1	0	0	0
Usp21	842	517	392	659	360	685
Usp28	8202	6533	5287	7157	4738	7964
Usp31	1203	797	623	973	626	958
Usp35	81	30	35	70	45	83
Usp37	751	478	400	556	401	659
Usp44	0	0	0	0	1	0

Usp8	2693	2144	1692	2345	1595	2670
Usp9y	0	0	1	0	0	0
Utp6	2060	1543	1233	1754	1182	1885
Uts2b	0	0	1	0	0	0
Vangl2	47	20	15	39	23	66
Vat1	1172	781	557	1216	621	1377
Vat1l	2	5	4	4	1	1
Vaultrc5	0	0	1	0	0	0
Vax2	0	1	0	0	0	0
Vcam1	1373	962	685	1124	620	1312
Vcpkmt	96	55	45	71	77	120
Veph1	0	0	0	2	0	0
Vgll3	163	86	69	148	79	198
Vill	21	9	10	14	17	31
Vipr1	60	30	53	67	46	127
Vldlr	21463	12195	11934	12788	12178	15980
Vma21	1093	806	663	928	564	1086
Vmn1r235	1	0	0	0	0	0
Vmn1r3	0	0	0	0	0	1
Vmn1r53	0	0	0	0	0	1
Vmn2r1	0	2	0	4	0	4
Vmn2r29	46	34	34	29	28	33
Vmn2r30	1	0	0	0	0	0
Vmn2r49	1	0	0	0	0	0
Vmn2r87	0	0	1	0	0	0
Vmn2r-ps54	0	0	0	1	0	0
Vnn1	6	6	6	4	2	6
Vprbp	1588	1168	1070	1508	955	1294
Vps13c	3413	2374	2036	2674	2065	2776
Vps28	1341	942	742	1234	849	1704
Vps8	1644	1061	866	1280	850	1305
Vsig10l	98	71	58	77	82	171
Vsig4	57	22	35	38	20	40
Vstm2b	13	8	6	7	2	10
Vwa5b2	0	1	0	1	1	2
Vwa8	12465	9200	7846	9791	8013	11064
Vwa9	832	645	502	749	437	799
Vwc2l	0	1	0	0	0	0
Vwce	1	0	0	0	0	0
Wars	2263	1434	1309	2225	1386	2707
Wars2	447	311	260	376	220	356
Wbscr17	235	128	116	176	101	153
Wdfy1	1355	390	338	1184	364	1472
Wdfy4	204	112	59	141	63	98
Wdpcp	151	97	94	163	101	187

Wdr17	3	0	2	1	1	0
Wdr19	134	96	81	133	94	196
Wdr3	713	458	390	588	320	652
Wdr43	1390	1079	908	1107	809	1513
Wdr5	1640	1036	871	1228	805	1454
Wdr52	0	0	0	1	0	1
Wdr63	0	0	0	0	0	1
Wdr64	0	0	1	1	3	0
Wdr70	219	159	105	215	148	267
Wdr75	780	502	532	579	430	745
Wdr78	45	35	30	42	17	37
Wdr86	13	4	11	2	5	13
Wdr92	609	418	356	481	332	570
Wfdc1	521	316	242	448	155	408
Wfdc2	0	0	0	0	2	0
Wfikkn2	354	148	207	319	276	352
Whamm	570	390	306	509	365	721
Wipf2	935	575	492	823	631	1033
Wnk3	9	11	6	9	2	7
Wnt1	0	0	0	2	0	0
Wnt4	13	12	2	4	7	7
Wnt5a	569	339	272	434	300	349
Wrn	502	339	289	523	315	620
Wscd2	5	3	3	12	3	6
Wwtr1	5585	3786	3330	5515	3760	6965
Xbp1	2475	1660	1358	2055	1168	2389
Xcl1	6	3	0	3	0	1
Xirp2	46737	36827	36309	37987	32967	36855
Xist	25876	16429	6	15890	10	18
Xk	718	561	509	739	356	605
Xkr9	0	0	1	0	0	0
Xlr3b	3	2	0	0	2	16
Xpo4	335	229	220	249	209	216
Xrcc3	33	23	19	25	19	18
Xrcc6	1386	903	783	1063	771	1278
Xrra1	0	0	0	0	1	1
Xylt1	45	21	19	46	32	48
Yae1d1	1342	836	716	1103	679	1021
Yars	1383	831	747	1047	646	1293
Yes1	1859	1316	1307	1514	1477	3047
Ypel2	1511	1056	825	1059	898	1322
Ypel5	1913	1373	1305	1819	1426	2570
Ythdc2	850	602	513	661	475	856
Ywhag	10056	6122	5430	8345	4823	7551
Zak	15328	10700	9176	12015	9736	12085

Zbed4	151	82	90	114	70	112
Zbtb22	710	414	356	586	417	860
Zbtb38	1275	1065	852	1275	784	1076
Zbtb4	3386	2266	1797	3135	2169	3707
Zbtb43	1632	1057	811	1321	810	1326
Zbtb8b	1	1	2	4	1	4
Zbtbd6	9	4	3	3	1	5
Zc3h12b	8	10	4	12	10	17
Zc3h12c	597	367	297	516	379	672
Zc3hav1l	209	142	88	179	72	152
Zdhhc13	592	414	401	487	351	522
Zdhhc19	0	0	0	0	1	0
Zdhhc21	933	581	520	747	475	794
Zdhhc24	193	116	94	159	124	261
Zdhhc5	6462	4658	4175	5812	3621	5784
Zeb2	2844	2019	1530	2234	1596	2522
Zer1	1660	1094	811	1581	1079	1779
Zfand2b	309	164	139	273	179	346
Zfhx3	923	666	516	858	654	1129
Zfp106	50214	38032	33242	41565	33343	36788
Zfp109	79	67	62	68	51	72
Zfp128	333	213	171	265	153	273
Zfp131	1732	1201	1046	1399	1014	1617
Zfp133-ps	93	31	63	35	60	43
Zfp142	1028	673	463	841	508	725
Zfp146	1159	897	706	1023	701	1034
Zfp160	873	573	489	817	527	1162
Zfp27	884	669	500	763	436	863
Zfp287	226	167	121	215	160	285
Zfp346	907	546	538	666	492	798
Zfp354b	81	82	70	72	56	88
Zfp362	647	425	368	616	418	777
Zfp367	2363	1503	1265	1839	1051	2393
Zfp37	160	114	103	153	115	242
Zfp383	788	486	402	544	360	780
Zfp414	220	166	118	201	166	306
Zfp428	239	145	125	218	153	301
Zfp438	338	271	176	254	194	281
Zfp456	59	50	40	71	44	96
Zfp467	377	194	178	290	247	438
Zfp583	64	51	43	54	22	58
Zfp60	833	542	495	646	462	676
Zfp62	2461	1871	1577	2031	1481	2287
Zfp644	1883	1366	1072	1551	1030	1744
Zfp651	5645	3148	2970	4301	2902	4023

Zfp663	0	0	1	0	0	0
Zfp672	1692	1171	1015	1545	825	1622
Zfp677	115	94	99	112	104	238
Zfp69	48	40	32	53	44	77
Zfp711	53	34	19	53	33	76
Zfp72	151	125	87	120	90	126
Zfp738	372	307	206	364	244	577
Zfp74	572	364	346	435	283	538
Zfp764	143	89	59	136	81	184
Zfp771	303	163	99	236	184	307
Zfp787	586	404	314	582	372	679
Zfp799	647	448	424	458	362	675
Zfp800	587	368	310	410	312	474
Zfp809	927	683	703	698	639	978
Zfp862-ps	348	243	172	287	143	266
Zfp932	256	225	192	213	139	321
Zfp937	130	78	85	126	81	204
Zfp939	152	79	78	101	61	120
Zfp943	1261	953	777	1242	861	1654
Zfp944	565	363	342	542	342	817
Zfp946	418	312	315	386	355	691
Zfp955b	633	529	503	573	419	618
Zfp964	52	44	27	28	31	39
Zfr2	102	62	53	56	50	85
Zfyve16	1395	949	819	1179	763	1274
Zfyve9	1467	926	836	1187	813	1218
Zic3	1	0	0	0	0	0
Zic4	0	0	0	0	0	2
Zkscan1	1504	1136	1055	1318	888	1512
Zkscan17	1060	637	529	961	572	1217
Zkscan4	73	41	39	28	41	58
Zmpste24	2770	1893	1784	2380	1600	2545
Zmym6	1570	1077	953	1118	894	1673
Zmynd15	96	50	35	84	50	126
Znfx1	2699	1814	1511	2673	1657	3100
Zranb3	234	177	104	239	185	256
Zscan10	0	1	0	0	0	0
Zscan2	159	94	91	140	138	177
Zscan4a	0	0	0	0	1	0
Zscan4b	1	0	0	0	0	0
Zswim5	108	85	65	173	76	119
Zwint	915	690	602	785	579	833
Zyg11b	5986	4169	3622	5186	3457	4807
Foxo3	1759	1223	766	1446	974	907
Usp14	4394	2700	2438	3144	2472	3923

Rela	1225	814	597	1184	732	1293
1700052N19Rik	533	365	291	417	276	495
Apol7d	0	2	1	0	0	1
Arhgef16	15	9	11	11	6	11
Cbx7	1016	660	476	828	733	1022
Cd209b	0	2	0	4	1	1
Fam20a	180	144	108	164	157	240
Gm12992	61	42	48	43	38	56
Gstm2	2391	1556	1165	1802	1018	2409
Hspa9	43620	30990	27762	35513	25613	40466
Mfhas1	3381	2046	2077	2930	1821	2800
Nat1	6	12	9	20	18	8
P2ry13	61	58	35	55	25	62
Tmem126a	3006	2166	2024	2493	1651	3418
Vil1	3	9	6	11	6	16
Vps26a	5040	3620	3034	3983	3115	4600
1300017J02Rik	7	4	4	1	1	8
Ap1b1	2812	1668	1521	2265	1380	2432
Cd53	246	194	187	217	105	314
Drap1	4518	2993	2799	4213	2850	5897
Fsd2	12917	9491	8134	11360	8237	9765
Larp1	5305	3667	3153	4557	2944	4769
Ltbp1	4460	3364	2087	4142	1827	3855
Med14	2298	1695	1157	1947	1242	1930
Mmp11	252	141	127	207	147	315
Nrbf2	158	106	100	160	116	198
Rrp1b	529	376	290	391	297	498
Slc9a6	1898	1254	991	1550	1028	1568
Zfp521	799	633	465	758	621	961
Fgfr1op2	4528	3197	2735	3794	2550	4359
4930430F08Rik	396	248	219	303	201	346
AA986860	50	38	29	44	39	82
Abcf2	2695	1814	1514	2298	1471	2304
Ccdc89	8	5	3	5	6	17
Fam222a	108	55	52	75	50	65
Frmd6	2084	1558	1184	1628	1299	1886
Gspt2	44	32	22	27	23	36
Hap1	72	43	33	59	45	98
Hdhd1a	2	2	0	7	0	3
Jakmip3	11	5	6	7	4	5
Myom2	72317	56675	36280	57135	39961	62208
Cyp2j8	2	3	1	0	0	3
3110052M02Rik	288	183	198	198	184	261
Adprhl2	260	141	117	198	169	283
B630019K06Rik	46	28	15	43	31	48

BC030307	150	101	79	133	106	189
Mir8094	370	263	175	248	160	388
Phka2	1280	837	609	1154	742	1432
Prrx1	3568	2740	2279	3111	2160	3512
Trim13	305	230	186	259	158	303
Ascl2	1	1	4	2	0	1
Baiap2l1	890	778	582	873	543	863
Ccdc64	33	26	12	31	21	51
Cetn4	24	23	14	18	13	21
Cnrip1	134	91	85	122	97	194
Ctsk	174	120	78	176	78	264
Esr1	106070	96221	89211	109724	71509	105659
Gm10046	4	0	1	0	0	2
Gm11992	74	37	35	26	36	63
Lix1l	1516	936	738	1284	961	1576
Magee1	261	166	134	271	149	296
Mir6903	26	15	10	18	21	35
Mpp6	2927	2026	1801	2250	1796	2837
Ptafr	44	35	34	39	17	48
Slc29a4	17	14	5	9	2	17
Slx4ip	72	72	38	49	41	76
Eif4ebp3	319	218	142	247	203	422
Hoxa5	43	25	13	29	26	61
Mafg	849	630	459	617	530	748
Zfp385b	607	394	350	473	330	545
Mir3535	4	3	2	3	4	10
Wdr89	163	113	73	94	101	111
B4galnt4	6	4	0	0	0	5
Bbox1	3	2	1	2	1	0
Dnaja3	8101	5615	5151	6791	4769	7576
Mir6990	49	23	23	25	21	36
Mgat5b	12	12	5	11	15	19
Mast3	367	201	168	266	231	428
Six2	13	9	2	7	3	7
Pld2	643	376	272	548	377	648
Idi1	10	9	10	6	4	13
Rabif	592	375	314	543	367	674
Ralbp1	3160	2172	1742	2966	2012	3591
Bfar	3667	2273	2402	2716	1951	3948
Ghrl	2	1	4	2	4	9
H2-Ab1	1173	710	489	1317	465	1337
Pan2	1361	768	670	967	828	1672
Sf3a1	2191	1525	1135	1927	1064	1907
Usp18	187	102	90	195	64	292
Exoc6	2152	1459	1285	1634	1270	2075

Clec11a	40	27	15	26	11	34
Uba3	3703	2690	2567	3310	2150	3811
Ttc32	653	419	418	431	400	641
Ezr	3227	2830	1924	3143	1706	3403
Mpnd	1478	835	578	1295	768	1439
Zfp322a	1400	941	871	1171	803	1298
Il2rg	428	255	219	371	230	557
Mmel1	6	2	5	4	3	1
Mgarp	6	3	8	5	0	7
Pias3	743	417	345	631	391	849
Slc26a11	182	122	92	164	126	217
1700021F05Rik	2374	1979	1858	1933	1612	2865
Anapc16	1582	1076	1009	1515	1054	1961
Dsg1a	3	1	0	5	4	0
Rtp4	379	257	192	336	175	646
Cd5	2	1	4	9	2	4
Gpr183	39	49	17	45	8	36
Gria1	11	10	6	18	11	13
Man1a	2116	1374	1065	1810	1017	1724
Mef2d	3486	2635	1748	2887	2080	2653
Toe1	336	226	165	310	210	395
Zfp191	1975	1317	1054	1717	1015	1614
Alpk3	9988	7255	5527	7896	6274	7780
Dkk2	8	6	5	5	10	17
Ermap	7	2	2	7	6	6
Fam160a1	3936	2249	2340	3473	2009	2861
Fam184a	56	26	26	25	31	33
Hn1l	281	148	148	212	120	224
Kif1b	15686	10763	9971	13388	9766	12067
Ralb	1248	870	684	1235	757	1457
Tm6sf2	43	29	20	21	16	44
Unc93b1	525	346	284	478	287	733
Rassf3	9334	5797	5454	7551	4843	8524
Themis2	123	69	69	121	58	187
Tnks	2559	1810	1504	2174	1566	2050
Obscn	67979	44158	34444	51850	33828	57769
Pcdha9	35	14	22	23	14	23
Cdh8	0	2	1	1	0	0
Ift46	525	377	305	482	339	715
Slco2b1	1066	673	778	998	730	1520
Sssca1	263	151	142	276	161	266
Zdhhc12	73	73	26	82	54	104
Tpp2	3847	2735	2533	3344	2298	3665
Apoa1bp	1182	878	758	1185	796	1541
Fam63a	1152	724	538	1057	606	1318

Pnpla8	8575	5768	5276	7116	4947	7764
1190005I06Rik	27	12	25	30	24	41
1810064F22Rik	1	0	2	0	0	1
Aqp6	1	2	1	4	4	1
Atp5g3	42478	22239	23456	40318	22206	50895
Bicc1	677	542	345	582	344	636
Bin3	343	212	204	281	227	472
Dnajc25	920	726	596	813	586	862
Dnpep	1922	1275	893	1681	1152	2129
Gimap9	120	61	99	118	95	174
Gm17455	1	0	2	0	0	1
Lrrn2	168	85	77	113	54	143
Mrps10	550	338	364	480	380	740
Plcl1	617	371	278	560	205	441
Pomp	2630	1638	1583	2393	1661	3137
Rnf11	5967	3741	3468	5005	3115	5162
Rprd2	2413	1631	1331	2055	1326	2006
Slc24a1	10	6	8	9	14	15
Stk36	28	11	14	29	15	33
Ttc3	7431	5559	4511	6257	4809	6041
Zfp954	207	138	114	150	101	196
Gbp6	1166	386	484	774	411	633
Gm7694	524	408	274	580	309	653
Casz1	3271	2364	1835	2727	1961	2797
2500004C02Rik	226	159	131	149	124	230
3830406C13Rik	1032	698	639	846	554	1047
Adgb	4	2	6	7	4	11
Arxes2	40	45	25	40	12	48
Camta2	2635	1741	1251	2042	1323	2287
Creld1	1578	951	817	1174	773	1461
Dmrt2	1	0	0	4	0	0
Dnajc16	1226	845	612	1027	701	812
Fam171a2	139	69	41	145	51	145
Gm13315	2	2	2	1	2	0
Gm9899	629	408	419	627	547	630
Irf3	1045	622	458	837	609	1123
Lrp12	350	191	178	257	157	285
Ly6g5b	3	3	2	4	3	8
Maml1	841	537	492	812	558	916
Megf11	10	9	22	4	3	27
Mir7005	58	11	9	38	28	41
Pip4k2a	781	532	463	737	503	963
Primpol	235	161	126	192	163	318
Prl	1	0	0	4	0	0
Psmb6	3118	2123	1801	2895	1919	3836

Rhobtb1	9914	6567	4947	7843	5524	7332
Smoc2	3728	2229	1361	3048	1208	2760
Thy1	315	225	174	352	147	480
Tmem202	9	9	10	18	5	25
Trpc4	13	10	8	19	13	15
Txnl4b	223	172	161	177	148	215
Zc4h2	75	54	47	72	47	130
Zfp422	984	644	573	911	671	1094
Pcdhb18	102	44	45	83	66	100
1700029J07Rik	237	170	134	140	136	245
6720483E21Rik	4	0	4	0	2	2
Abhd17b	714	414	362	616	421	792
Cebpz	1713	1242	1015	1337	1043	1666
Fip1l1	1482	1186	906	1294	921	1420
Gm15545	21	15	21	10	14	24
Immp1l	1457	890	860	1090	738	1476
Paqr4	1014	730	725	962	517	1095
Srr	1570	1172	940	1398	934	1387
Tbccd1	515	339	292	403	269	479
Urb2	353	234	210	325	163	295
Ush1g	2	6	3	1	1	5
Zfp958	514	378	297	387	283	528
Zyx	2085	1368	1123	2034	1198	2388
AU022793	0	3	0	1	0	0
Klhdc3	2710	1541	1177	2334	1554	2577
Ltk	0	3	0	1	0	0
Slc1a7	7	12	3	11	8	18
Coro1c	1389	988	654	1298	772	1709
Alg10b	1354	818	679	1095	716	937
Ctsz	809	560	515	778	491	1148
Fibp	758	516	482	666	512	1045
Prps1l3	1133	811	691	1106	561	1015
Acsl5	1032	664	555	1017	538	1282
Adam23	247	158	133	110	118	283
Celf3	1	1	1	0	0	1
Chm	1567	995	905	1186	925	1362
Cramp1l	1501	993	729	1296	859	1805
Diras2	17	6	10	9	4	13
Fam19a2	7	6	6	4	4	6
Fech	7414	5145	4535	6270	4300	6766
Gm16861	6	2	0	2	6	7
Hal	1	1	1	0	0	1
Kif5a	76	38	41	57	56	96
Klhdc4	558	366	340	523	377	674
Mylpf	33	19	21	27	28	49

Naip6	65	49	39	61	45	108
Pan3	1750	1127	849	1346	1091	2112
Pclo	48	58	33	42	35	47
Phpt1	1061	639	564	976	584	1233
Sumf1	1001	655	568	918	622	1189
Vamp4	677	438	417	522	349	692
Wdr62	84	61	37	76	58	109
Rabep1	3306	2444	2056	2880	2082	2895
Eif5a2	275	152	145	246	157	324
Atp2a2	739646	456956	383250	598089	358489	514080
1110008F13Rik	1930	1211	1165	1540	959	1964
2900005J15Rik	21	17	19	14	28	47
A930015D03Rik	31	4	13	2	16	9
Ankrd1	56058	46441	52538	59417	49458	109630
Csrnp3	24	10	18	10	11	22
Ddhd1	970	690	600	734	585	986
Fchsd1	86	55	42	75	63	103
Fhad1	17	18	28	14	22	12
Itga3	477	328	201	434	329	468
Mark4	1072	746	547	821	622	894
Polr2h	228	130	144	190	86	217
Rsph9	17	13	7	22	12	21
Treml2	18	23	19	16	20	10
Tubg2	98	46	55	87	53	133
Wnt6	7	2	4	9	7	6
Zfp658	131	84	71	75	51	147
Zxda	238	193	129	232	171	315
Aldoart2	0	1	2	0	1	0
Ap4s1	1982	1184	1150	2174	1013	2303
Atg3	1386	1086	901	1141	863	1474
Btbd6	731	393	378	616	448	768
Cyth3	2220	1515	1199	2275	1246	2597
Dlk1	6	4	2	3	1	4
Dusp16	1936	1342	1107	1670	1086	1664
Dyrk1a	3905	2849	2285	3492	2219	3413
Galnt5	5	4	2	1	3	3
Helq	275	163	152	190	144	239
Manea	1892	1342	1115	1630	1023	1825
Mir7019	13	3	2	2	0	9
Mprip	8870	5587	4679	8045	5402	9858
Nbeal2	931	516	392	783	542	879
Parvg	44	35	15	26	12	44
Scrn1	299	215	144	274	124	235
Slc31a1	1840	1181	1182	1655	931	1739
Snord69	4	0	0	1	1	8

Tmsb10	300	259	206	372	162	546
Tomm5	649	368	422	467	320	670
Xpot	2138	1349	1087	1664	1128	1786
Tmem39b	174	97	81	144	112	191
Cyp2r1	16	9	10	16	15	21
3425401B19Rik	7468	6381	4742	6993	4921	6421
Cdh3	2	2	2	4	6	2
Spsb3	737	378	396	785	360	778
Zfp831	8	7	5	5	2	8
0610031J06Rik	1243	694	613	1104	667	1342
Bhlhe41	1694	1269	1003	1530	938	1611
Msl2	2570	1790	1354	2086	1421	2259
Sele	42	27	27	47	24	67
Uhrf1bp1	1100	649	638	987	571	750
Mir6947	1	0	2	1	0	0
5330439B14Rik	1	2	0	0	0	1
Cntf	46	18	26	33	37	55
Elovl7	12	10	10	9	5	12
Il5ra	1	2	0	0	0	1
Inpp4b	433	297	276	308	300	340
Mrpl37	3061	2044	1724	2518	1569	3007
Myt1l	1	2	0	0	0	1
Olfr690	1	2	0	0	0	1
Ppm1n	1	2	0	0	0	1
Scarf1	969	492	380	667	557	1003
Soat1	301	179	125	212	115	256
Tmem176b	866	656	534	949	471	1275
Tnpo3	3718	2600	2261	3194	2127	3435
Pafah1b1	13836	9091	8071	11886	7426	11738
Mybl2	11	10	5	14	5	24
Adh7	4	4	4	2	3	3
Asgr2	5	9	7	6	2	8
Ctps	1984	1163	1165	1588	1031	1760
Rnf38	2091	1414	1039	1797	1336	2356
Sfn	67	52	25	53	50	94
Gpt2	3079	1729	1435	2557	1284	2321
Pkm	37771	25277	21223	39909	23002	43170
Aloxe3	2	3	2	1	1	2
BC024978	430	272	205	263	224	394
Cyb561d1	486	302	261	471	270	584
Lmln	155	125	79	118	90	128
Rab21	3652	2292	2311	3155	2041	3152
Crat	23618	15896	13193	18559	13379	20169
Gm15446	9	18	14	13	16	43
Bri3	1346	1049	825	1357	904	1739

Gtf2f2	465	379	353	452	260	517
Scn1b	490	356	321	469	340	681
4931403E22Rik	2	4	8	2	4	3
Catsperg2	1	1	0	1	0	5
Dusp12	298	194	160	205	156	281
Epb4.1l4b	97	62	62	65	47	98
Fam210a	12417	8417	8193	10324	8045	9841
Mfsd1	1140	683	719	1003	711	1479
Mmp14	397	248	214	374	210	526
Ndnf	217	174	176	234	139	162
Rab40b	183	127	127	141	81	213
Uhrf1	92	46	30	76	34	129
Tonsl	44	16	9	30	18	51
Adi1	2101	1451	1293	1627	1311	1949
Rps6ka3	3610	2377	1850	3439	2172	3883
Arhgap15	51	38	27	58	31	72
Ccdc149	380	293	201	380	245	482
Crybb1	38	18	23	47	20	46
Gm10549	3	3	5	0	1	6
1810026B05Rik	1125	651	688	761	575	1185
6030468B19Rik	1	1	1	1	0	0
Dyx1c1	58	43	42	45	55	103
Foxp3	1	1	1	1	0	0
Pgpep1	1402	779	808	1193	714	1025
Ppp1r16a	689	399	294	622	355	730
Sez6l	5	8	4	4	4	4
Sp7	1	1	1	1	0	0
Trpc6	13	5	6	10	11	16
P2ry12	104	95	74	66	65	129
Armc9	156	91	87	117	63	142
Suclg1	13031	8277	7572	10990	9466	14838
Ccr7	9	3	1	2	1	5
Psmd9	619	458	354	606	411	765
Noxred1	45	36	24	37	28	22
Sipa1l1	1139	796	604	1066	715	1318
1700092M07Rik	3	3	0	2	0	1
1810006J02Rik	11	8	6	3	2	14
Ddx51	561	362	280	425	285	480
Fndc3b	3945	2484	1664	3110	1796	3083
Gm1653	13	6	9	8	4	10
Noc2l	1427	1018	878	1141	877	1366
Nqo1	1988	1048	992	1301	1459	2114
Rbks	71	43	38	69	40	99
Sapcd2	3	2	2	1	2	13
Zfp286	46	24	31	26	19	46

Apoc1	3	5	4	1	2	5
Apopt1	898	615	571	815	622	1148
Arih1	5447	3990	3487	4862	3275	4923
Hcfc1r1	2719	2095	1600	2665	1853	3335
Klhl36	108	46	60	103	50	144
Pcdh11x	6	3	3	2	3	3
Slc25a43	6	3	1	8	2	8
Trpm3	110	85	59	110	84	138
Zfp455	91	50	59	48	50	85
D830031N03Rik	981	681	533	809	892	948
Gm684	78	45	32	47	32	61
Setbp1	763	599	512	635	506	741
Sgms2	106	54	60	93	34	74
Tcf24	46	19	21	31	16	26
Zscan20	439	215	273	285	258	323
Kctd19	16	20	16	13	32	36
Lrwd1	269	154	112	245	125	324
Phf21a	1204	862	662	1037	872	1407
Prpf3	1005	842	657	989	576	1014
Rpl7l1	2067	1263	1214	1839	861	1975
Scx	84	37	48	50	39	63
Tpr	10323	8319	6434	8440	6863	9670
Zfp511	248	168	134	185	117	242
Rabepk	314	209	173	306	190	385
Ggnbp1	894	518	454	381	476	916
Gprasp2	143	111	55	119	54	111
Vps16	885	577	507	801	564	1048
Gm12216	38	20	21	36	25	49
Prokr2	4	4	2	3	6	9
Arpc3	2230	1546	1390	2031	1433	3000
Camk2n1	1514	1118	1143	1552	1137	2113
Dgke	1494	1150	1142	1385	1070	2611
Dhrs7b	409	292	231	393	288	476
Nrm	63	39	21	70	36	60
Pold2	649	483	345	540	328	627
Rhot1	4903	2911	2702	3708	2596	4319
Scara5	1458	845	934	1173	599	1589
Timp1	40	51	40	57	18	169
Tmsb15l	8	6	7	10	7	17
Vmo1	2	0	1	2	1	4
Zfp382	48	45	34	64	41	71
Ccdc63	2	1	0	1	2	4
Cenpe	59	33	25	42	19	129
Dnah7b	91	81	60	80	45	98
Erich2	0	0	3	3	1	4

Greb1l	60	57	48	63	26	68
Hmgb3	968	763	695	859	608	1044
Mlh3	873	670	578	744	568	831
Nkain3	1	2	0	1	0	0
Smim5	419	263	196	432	201	497
Spink4	1	2	0	3	1	3
Swap70	915	605	551	765	637	1156
Tmem51	188	131	104	245	113	196
Tmem67	297	173	150	247	174	361
1110019D14Rik	205	130	147	158	118	194
Anks1b	33	16	14	17	14	22
Atxn1	2561	1650	1295	1890	1410	2224
C77370	47	16	9	23	6	27
Cbl	560	385	292	433	323	446
Drd2	135	129	54	94	64	137
Fam198a	54	31	16	32	20	34
Fam3a	798	494	391	737	469	843
Glb1l3	4	3	0	3	2	9
Ido2	2	0	4	2	0	1
Ifit2	1708	1065	866	1501	794	2463
Kpna3	5399	3742	3234	4552	3219	4605
Ptk2	2100	1470	1185	2016	1278	2561
Slain2	12031	8506	7931	10223	7596	10561
Ccr2	241	170	104	312	126	237
Clec4f	7	0	5	1	2	4
Nox4	92	109	93	203	84	141
Rrs1	1127	711	615	948	532	1032
Mir705	21	11	15	13	6	21
Apela	2	0	1	0	0	1
Dpysl4	327	192	171	270	186	428
Kif14	24	12	8	17	2	16
Cmss1	2814	1966	1710	2580	1379	2827
Trafd1	2971	1895	1458	2519	1839	3185
Mir6236	16989	18848	3125	2742	14824	3147
Gprin3	2	2	4	9	2	5
Hmcn1	2651	1595	1039	1683	1057	2515
Ppp1r14a	43	32	54	58	47	88
Stag3	8	11	8	10	11	23
Ctse	6	8	6	8	1	6
E2f3	527	391	378	489	328	476
Mir1291	40	20	13	43	14	49
Dpep1	2009	1380	1642	2475	1207	3116
Fxr2	5735	3648	3079	4519	2979	5145
Psmb5	1317	764	707	1181	722	1528
Cd55	502	427	334	484	224	596

Fbln1	3337	2587	1493	3669	1285	1845
Rpl24	15	19	14	10	10	21
Col5a2	1441	975	824	1378	692	2254
Lpcat3	1398	893	810	1172	1029	1538
Smc2	290	191	144	273	153	383
Orc4	1608	1095	1070	1354	905	1675
Polr2k	22	12	15	18	9	14
Spred3	43	38	28	30	43	87
Smim11	1179	934	774	1004	738	1231
Setd4	93	51	57	42	51	89
Cyp39a1	632	439	351	626	440	673
Gm1673	4	7	3	10	8	6
Arhgap6	317	267	192	331	242	413
C3	7172	5474	5526	6513	3518	9235
Cchcr1	126	109	64	116	83	211
Coq3	2445	1515	1673	1950	1390	2466
Ehd1	6130	3636	2727	5888	3190	6181
Ermp1	921	686	537	805	493	910
Jph3	13	31	10	17	4	25
Mmp12	14	10	10	14	5	41
Ppp1r12b	3401	2942	2298	3026	2730	1644
Rgs5	11345	8212	6695	12811	6319	14551
Rhbdf1	1072	660	502	976	580	1165
Snap23	2668	1939	1590	2208	1529	2674
Usp30	520	303	244	432	338	526
Zfp612	1976	1575	1050	1364	1078	2259
Aplf	144	97	70	127	96	178
Prkcg	238	135	128	150	163	352
Sfxn3	494	303	238	450	278	553
Ifi202b	190	0	114	0	77	0
Zbtb34	529	258	227	408	278	566
Slc30a9	8538	5849	5102	6965	4859	8084
AA414768	34	23	23	40	17	62
Akr1e1	934	883	716	1024	621	929
Ddx58	1091	752	593	994	611	1481
Fam175b	1419	911	828	1192	762	1268
Hpse	74	36	30	43	32	48
Pik3r2	1088	682	487	1001	627	1088
Snord8	1	0	3	2	4	3
Socs6	851	466	446	684	406	644
Ubxn11	14	12	7	14	16	19
Zfp120	720	535	415	615	474	1039
Zscan22	329	236	146	266	163	250
BC021785	48	24	21	27	21	33
Mir6925	666	517	341	573	382	528

Poc1a	150	98	68	136	76	212
Csde1	37293	26742	24538	30806	22565	35377
Lars2	21582	10054	4203	4344	12595	5039
Mob3a	216	178	118	233	151	272
Olfr1033	795	484	531	670	340	862
Rnft2	129	64	54	103	74	142
S100a6	406	311	388	437	313	809
Slc35g1	163	88	95	119	74	139
Strap	5242	3636	3394	4194	2954	5591
Abl2	1735	1079	989	1449	974	1367
D830030K20Rik	4	1	2	2	0	2
Ldb1	2013	1423	1043	1811	1281	2347
Prob1	4935	3312	2566	4031	2706	4038
Rcan2	28483	20112	18714	23042	17730	26322
Tefm	837	530	519	615	478	820
Tmem41b	1010	625	534	813	451	961
Tubgcp4	1299	887	802	1079	757	1221
Paqr3	61	34	14	30	27	30
Raet1e	2	1	3	3	0	0
Asun	1488	1080	801	1181	803	1486
BC049762	9	10	2	7	3	5
Lpin2	521	333	295	477	284	722
Mars2	539	351	346	468	290	486
Nyx	31	17	14	22	25	40
Pld3	912	611	439	850	589	920
Rmdn3	887	548	485	754	402	793
S100pbp	315	257	189	247	188	324
Wdr93	2	10	3	4	4	1
Syf2	2546	1841	1507	2241	1826	3125
Zfp830	698	482	407	573	379	661
Kctd12	2125	1259	1024	1836	1099	2499
Psme1	4716	3730	2614	4666	3142	5888
1700008I05Rik	1	2	0	2	4	1
Arnt2	17	9	13	16	4	12
Ston1	662	374	269	592	329	691
Arhgdia	4966	3172	2459	4537	2709	5797
Myo19	550	353	193	427	215	389
Pcdhga7	2696	1758	1411	2497	1452	3327
Tmem238	41	22	18	23	19	28
Slc35a3	1028	578	512	826	577	1184
Iqcd	3	7	2	3	1	4
Limd1	3579	1772	2129	2993	2288	3870
Lrrc3b	1075	701	488	1070	626	1041
Pcdha3	34	14	22	22	15	23
Urgcp	3140	2014	1651	2844	1543	2412

Armc3	5	0	2	5	4	4
Cby1	689	504	351	575	347	631
Coa7	772	517	403	618	429	624
Slc4a11	9	5	5	12	7	11
Dennd4c	1873	1354	950	1490	1168	1338
Azin1	4858	3760	3011	4686	2677	4546
4631405J19Rik	193	135	91	153	78	175
Ate1	2578	1734	1548	2138	1499	2341
Dopey2	981	728	491	947	658	1060
Kcnq5	52	31	19	30	77	28
Mir1306	24	14	6	24	20	18
Ncmap	7	5	2	9	5	9
Ncoa6	2396	1983	1305	2176	1448	2002
Pgm3	351	229	233	294	190	330
Rbms1	2167	1648	1261	1952	1504	2799
Tec	787	525	566	598	531	731
Zdhhc6	1204	886	808	1051	709	1248
Zfp641	508	451	291	430	289	545
2510003E04Rik	2382	1600	1428	2091	1316	2122
Acbd3	1704	1211	1103	1523	995	1634
Agps	2427	1425	1262	1860	1244	2060
Ankrd17	7189	5629	4121	6342	4539	5703
Bmi1	3885	2659	2129	3028	2280	3408
C7	173	81	555	134	204	373
Cdc20b	1	2	0	0	1	0
Cmc2	1117	774	766	978	605	1185
Col9a2	19	6	2	20	8	13
Cox7c	127	79	77	77	69	123
E030030I06Rik	10	13	8	5	6	14
Gng12	2270	1525	1272	2079	1402	2696
Hand2	1619	1220	951	1613	1172	1797
Lama5	8869	4611	3219	7417	5265	6947
Ldb3	50211	34582	29286	41757	26400	46557
Mllt1	1293	867	727	1119	725	1036
Pdxp	36	16	9	19	8	23
Prkce	2981	2072	1837	2491	1652	3067
Psmd10	382	268	212	345	286	434
Rasgrp1	28	13	16	37	12	35
Rtn4	4920	3495	3113	5253	3222	5829
S100a10	1235	827	639	1242	614	1575
Scara3	123	89	68	151	68	156
Siah1a	789	446	440	655	470	951
Snap29	1601	1090	975	1321	916	1548
Tarsl2	2714	1880	1537	2208	1622	2332
Trim2	543	341	404	546	344	804

Trmt10a	166	81	75	134	115	154
Triobp	827	525	397	836	444	878
Nbn	1566	1072	897	1287	897	1424
Reep2	23	14	17	26	13	42
Zfp617	750	586	479	652	443	768
Sdhaf1	752	538	454	536	411	845
Trip6	418	226	195	368	251	406
Cuedc2	1588	1010	882	1384	954	1909
Ptx3	23	14	10	15	10	14
Taf1b	417	306	255	396	298	530
Atp5sl	1833	1224	1051	1459	1003	1792
1700119H24Rik	2	1	0	1	0	0
5830411N06Rik	2	1	0	1	0	0
Cxcl12	13553	7536	6392	10295	7853	15320
Ifi204	703	274	429	307	392	578
Ly6c2	5	7	1	9	2	13
Nmrk2	76	37	93	63	34	98
Pigm	944	546	524	794	525	1196
Trappc3l	2	1	0	1	0	0
H3f3a	1595	991	1024	1458	961	2118
2810029C07Rik	23	15	19	22	22	39
Bdp1	2084	1335	1015	1700	1113	1514
Ccsap	39	25	21	37	21	64
En2	4	2	0	0	1	2
Gm3435	4	2	3	3	2	13
Ifi203	2589	1509	1366	2125	1623	2799
Lsm7	29	13	7	12	12	14
Scand1	608	365	215	508	315	647
Tnrc18	2453	1627	1096	2308	1579	2274
6330408A02Rik	675	425	408	520	383	613
Pmm1	485	291	234	402	281	568
Tmem88b	137	96	109	100	85	147
Cdca2	51	16	16	16	6	46
Gm867	8	6	9	4	3	11
Uba6	515	419	332	469	329	457
Xpr1	3728	2835	2490	3644	2317	3065
Dctd	22	9	8	19	22	14
Jakmip1	1	2	1	6	2	1
2810428I15Rik	796	441	423	711	478	827
4921507P07Rik	22	13	18	18	20	41
5530601H04Rik	152	105	62	99	69	132
Aldh3a1	34	24	23	37	29	46
Cav3	1608	916	938	1445	982	1774
Ccdc174	527	399	362	447	274	615
Cecr5	919	457	411	746	476	953

Cpped1	3284	2610	2051	2874	2093	3174
Cys1	342	250	121	399	177	345
Ect2	38	21	24	28	14	98
Eef2k	1794	1219	957	1531	1348	1865
Etv6	876	594	521	823	566	1053
Fam214b	1254	756	696	1109	798	1354
Gm7367	175	83	67	137	79	211
Has2os	4	1	1	0	2	1
Hoxa9	3	2	2	0	0	4
Hoxb6	8	0	0	0	1	2
Igf2bp3	3	0	2	3	2	5
Nasp	1248	818	640	968	625	1170
Nln	853	607	502	753	466	782
Nmd3	1048	830	742	959	653	1095
Ntn1	5223	3924	2639	4814	2528	4515
Ostm1	1937	1315	1195	1579	1082	1952
Pigw	259	174	173	211	155	229
Ppp1r13l	1700	1031	716	1374	945	1823
Rpl38	48	15	30	38	28	68
Ruvbl2	871	507	408	670	379	739
Smug1	529	329	314	424	257	509
Snora16a	13	2	3	14	5	10
Tgoln2	2797	1849	1737	2426	1670	2303
Tiparp	1088	706	688	840	624	1091
Tmem191c	81	33	28	28	24	71
1700017B05Rik	415	250	200	408	207	481
1700025N23Rik	2	0	2	1	2	6
2900097C17Rik	4635	2811	2928	3432	2958	3759
6030419C18Rik	817	416	459	579	427	657
9130221H12Rik	343	224	216	345	218	430
9330162012Rik	42	36	30	38	23	38
Adamts7	1456	969	587	1268	905	1425
Agpat6	2540	1745	1455	2266	1343	2275
Bag3	12447	9726	7660	11333	7257	11803
Ccdc62	166	108	87	114	123	238
Cdh24	24	13	4	4	11	14
Cpa2	3	0	0	0	0	1
Ddx19b	909	660	547	813	499	862
Dhrs11	2893	1944	1362	2714	1770	2904
Fam206a	261	195	117	176	136	242
Gpr18	7	2	3	4	1	3
Hadhb	14778	8979	9021	11481	8182	13459
Haus3	381	264	220	309	265	527
Impad1	2443	1686	1441	2229	1384	1967
Ktn1	23236	20680	15759	20812	14975	24965

Mdm2	2627	1885	1798	2482	1492	2583
Metap1	2047	1470	1224	1692	1173	2039
Mfn1	22801	15134	13819	18831	12678	20729
Mir1966	60	32	20	72	25	58
Mns1	12	8	9	12	8	26
Mrgprf	92	52	54	100	48	121
Ms4a4c	17	6	6	18	7	19
Nnmt	92	33	47	46	26	84
Nova1	236	115	152	179	150	341
Nts	118	86	24	49	53	85
Pcdha8	35	14	22	23	15	23
Pcdhb2	10	5	2	4	1	7
Pdcl3	2440	1703	1522	2148	1713	3103
Pla2g3	12	11	6	11	10	24
Pnma2	2	1	4	2	1	1
Slc37a2	226	143	145	211	109	179
Sned1	458	279	177	269	231	362
Socs5	1738	1042	969	1492	835	1505
Tnrc6a	5083	3514	2962	4518	3417	6244
Ubald2	175	83	68	138	79	211
Zfand6	3249	1928	1824	2955	1831	3757
Arhgef40	1314	806	653	1382	708	1301
Gmfg	49	29	20	28	13	47
Mir7029	8	12	7	24	6	15
St3gal4	1071	750	618	976	551	973
Sacs	41	24	18	81	16	29
Hnrnpm	4656	3330	2605	3933	2556	4363
Lynx1	11541	7975	6521	10351	6345	9201
Rps6ka6	12	10	4	13	19	7
Pou3f1	22	7	19	34	19	19
Cep192	610	371	299	506	357	711
Mamdc4	129	47	47	63	42	93
Aldh3a2	1948	1240	1041	1819	1142	2166
Morf4l1	1459	760	709	1278	719	1599
Ppm1j	647	335	315	556	344	694
Psmg2	1051	576	562	772	464	1004
2310002L09Rik	2864	2220	1765	2975	2004	3374
Ralgps1	535	326	249	427	398	510
Sepsecs	232	170	122	190	114	212
Uqcrq	9743	5196	5330	8652	5236	11239
Medag	1138	734	612	1022	433	1095
Asap3	797	503	419	597	402	748
Cers1	23	8	12	16	6	13
Dpysl3	2492	2148	1500	3300	1549	2970
Fundc2	9223	6346	5555	7858	5046	8838

Gpr160	163	94	111	129	97	293
Mir6340	2	0	1	0	1	0
Nlrp6	2	0	1	0	1	0
Pir	174	96	119	152	133	220
Rnf8	641	424	346	582	404	746
Rtn1	129	80	61	105	78	171
Satb2	26	16	16	28	23	30
Tmem178b	6	7	3	7	2	2
Bora	50	31	26	34	31	90
Spns2	730	384	292	550	408	739
Fam115a	403	256	229	456	242	415
Gadd45g	293	164	132	267	197	254
Sh3glb2	1398	799	772	1216	766	1687
Als2	1008	659	583	779	573	945
3110021N24Rik	8	14	8	7	9	7
Mir3474	3	0	1	5	0	4
Azi2	4182	2827	2551	3428	2490	3811
Bend5	226	153	151	171	190	330
Cd209g	3	2	2	6	5	2
Cox19	1039	562	707	852	486	1039
Fgf10	37	16	30	16	20	36
Gng11	1076	722	789	1000	704	1672
Mrm1	860	566	478	816	383	715
R3hdm2	9298	7225	5719	8041	6139	7720
Sap30	329	211	205	271	187	275
Xirp1	12012	9393	8312	12789	6889	10089
Capn1	1551	920	622	1414	826	1471
Srgap1	291	184	142	252	166	360
Snapc4	640	454	286	416	353	578
Ranbp6	1654	1105	1060	1560	897	1452
Unc119	134	76	89	120	94	179
9930111J21Rik1	122	79	53	108	67	160
AI118078	106	66	80	76	59	107
BC018507	3988	2653	2293	3087	2348	3627
E430025E21Rik	2409	1807	1398	2152	1402	2164
Emd	1074	754	709	943	622	1060
Fam161b	835	518	409	610	475	617
Fam193b	1813	1086	768	1171	1139	2163
Gm10653	1	1	0	2	1	2
Gm13152	177	123	99	166	134	204
Kctd18	335	207	154	282	196	388
Mzt2	278	201	143	252	188	339
Naip5	128	81	50	73	86	192
Pqlc2	157	93	87	150	102	177
Slc35d1	360	278	222	255	241	345

Slc43a1	145	83	83	136	43	116
Stk40	2765	1809	1615	2445	1519	2297
Tm4sf5	1	1	0	2	1	2
Tmem154	131	75	82	119	82	180
Unc80	4	15	11	38	9	10
Wdr95	1	1	0	2	1	2
Wfdc18	1	1	0	2	1	2
Wnt11	182	89	84	153	96	205
Zfp174	214	135	124	187	167	238
BC022687	73	45	73	80	72	116
Dgkz	4194	2632	1788	3160	2347	5064
Dse	401	271	180	340	139	361
Gpnmb	76	55	77	111	57	130
Mmab	1722	1208	892	1403	969	1488
Nkain2	17	16	12	13	7	19
Nmnat1	532	349	330	453	288	481
Oxct1	76980	51137	51632	61663	44516	74059
Pdss2	996	646	612	882	528	900
Strn4	2691	1605	1361	2489	1587	2672
Tbcb	999	637	553	874	601	1203
Ccser2	13565	10008	8297	11596	8365	11991
Ctdspl2	1209	740	500	948	704	1284
Dhrs3	3055	1736	1605	2301	1990	3349
Palm2	248	236	205	252	182	245
Pln	389869	259825	217108	299008	209892	332497
Slc35f4	2	1	0	0	1	0
Supt16	1881	1338	1086	1527	1050	1881
Tex10	595	361	314	465	272	548
Trem2	42	19	10	27	19	55
1700120C14Rik	237	131	124	223	136	261
Abcb4	2350	1817	1853	2117	1476	2741
Calca	3	0	0	1	0	0
Ccdc47	7630	5242	4588	6333	4448	7033
Cep97	722	434	380	506	367	677
F830045P16Rik	2	0	0	0	4	1
Foxl2	3	0	0	1	0	0
Frg1	813	576	519	727	529	1158
Galm	1257	893	758	961	772	1237
Gm20604	706	406	384	579	295	644
Kpna4	6170	4188	3821	5038	3551	5945
Lrrc4	35	27	21	31	17	26
Mir3091	3	0	0	1	0	0
Ninj1	1168	835	660	1193	771	1266
Pcdhgb1	2625	1742	1389	2449	1404	3313
Pecr	374	260	202	279	179	387

Rsbn1	1452	912	747	1212	882	1657
Scnn1b	3	0	0	1	0	0
Snrpb2	889	535	559	603	493	928
Suz12	2258	1451	1209	1803	1206	2009
Tvp23a	19	17	14	11	6	27
Gm17801	2	0	0	2	2	1
Nuf2	22	12	10	19	4	54
Smarca4	4907	3392	2640	3995	2643	4552
Dnaic1	30	21	36	43	34	50
Gatad2a	1451	961	732	1348	885	1537
Lgi1	65	29	60	61	85	67
6-Mar	11443	7184	6425	9437	6315	9221
Mif4gd	1073	669	545	813	568	928
Mob4	2687	1840	1720	2208	1495	2846
Pde7b	1491	1076	825	1227	904	1270
Pet117	62	71	42	56	45	60
Psd	5	5	4	8	5	10
Stxbp3a	1014	567	537	850	603	1103
Zfp790	977	719	681	866	595	1004
Cyp1a1	6	10	6	13	10	12
Lrrc8a	3397	2274	1840	3403	2048	3639
Mrpl51	2836	2141	1892	2445	1669	3133
Ocel1	187	102	89	159	114	203
Sema4d	1046	741	554	917	579	838
Trap1	7241	4913	3897	5649	4052	6480
Tspan2	987	545	528	828	517	1224
4933407K13Rik	62	42	44	52	42	119
Pmpca	8515	5639	4855	6735	4627	8175
Fam60a	20	8	11	17	16	23
Gjd3	1	0	2	4	2	1
Gm15915	10	8	11	3	7	13
Gm5801	726	470	409	700	449	814
Has2	32	15	16	26	12	14
Hbegf	936	659	652	919	509	892
Hdac3	2272	1547	1249	2050	1437	2643
Hps1	68	26	30	36	45	89
Kcnh3	1	1	0	1	2	2
Klhl14	1	1	0	2	2	1
Mir7060	2	0	0	1	2	2
Mir7213	1	0	2	4	2	1
Nolc1	1313	964	805	1207	786	1114
Pcm1	4861	3619	2965	4147	3126	4123
Pgk1	578	231	231	548	218	565
Pmpcb	9115	6530	5761	7411	5465	9139
Rnf7	2476	1588	1490	1923	1328	2533

Rsl24d1	966	542	571	681	493	950
Thbs3	418	309	200	369	185	364
Tlk2	2408	1799	1512	2063	1493	2319
Umps	452	245	240	363	194	371
Zbtb46	334	185	156	292	167	395
Znrf3	359	235	199	309	193	268
2310022B05Rik	2761	1694	1449	2676	1623	2782
Snx19	2742	1806	1661	2460	1480	2430
Birc5	46	14	13	29	14	66
Gfm1	9748	6661	5934	8167	5531	9210
Tbc1d2	85	61	57	79	62	134
1110032F04Rik	10	13	4	12	7	25
Gm15408	24	32	19	33	27	49
Gng10	524	313	331	419	197	584
Ndufb2	3233	1945	2043	2896	1962	4090
Grb10	5614	3819	2997	5363	4063	5431
Mme	823	316	360	431	393	550
Thnsl2	248	158	140	233	155	300
Anxa9	24	14	11	12	21	39
Cyth1	2931	1865	1767	2606	1783	3676
Gpr37	1	3	4	2	1	2
Ms4a2	4	2	1	3	0	1
A730056A06Rik	62	53	39	45	42	54
Mroh1	1599	898	686	1183	656	1355
Tmbim6	9796	5476	4803	8691	5327	10256
Art1	6550	3942	3183	5088	3702	8098
Lmbr1l	408	203	165	323	195	455
Tlr9	25	23	12	25	7	20
Mtr	2044	1146	1102	1743	2057	1334
1010001N08Rik	456	295	226	313	275	340
Akr1b8	151	86	85	158	75	194
Apex2	266	163	85	197	175	261
Kif5c	94	89	80	93	55	111
Pcca	4223	2899	2452	3549	2367	3915
Acsl1	52924	37562	31633	41477	32686	45761
LOC101669761	5	6	4	2	1	8
Ruvbl1	276	216	179	226	159	295
Slc5a6	600	345	322	483	298	472
Ube2e3	2048	1261	1197	1859	1233	2407
Fcamr	6	3	1	8	7	2
2610002J02Rik	228	155	102	244	120	257
4921531C22Rik	186	139	140	152	125	176
Agfg1	1633	1048	885	1391	860	1367
Bcl2l13	5137	3231	3058	4472	2705	4477
Cpne3	2215	1404	1070	2114	1190	2389

Fndc7	9	3	2	16	3	5
Frmd7	38	22	35	32	15	41
Ints2	454	293	232	381	219	369
Ipp	481	322	280	392	268	427
Kcnab2	87	44	30	51	30	64
Ptpn1	1011	669	513	958	596	1097
Rpl12	64	57	46	48	32	81
Ticam2	41	37	22	26	18	48
Zfp941	19	18	26	14	12	31
Klhdc1	1452	910	773	979	931	1095
Nras	2008	1252	1149	1734	1155	2587
Pcdhgb5	2642	1737	1391	2466	1401	3301
Ppp1r27	6	2	2	4	5	8
Klhl9	6082	4393	3351	5372	3276	5390
Prox1	1436	1161	896	1178	1118	840
Tcea1	543	476	350	498	344	547
Pnrc1	4087	2194	2185	2851	2542	5051
Ubd	0	0	1	4	0	0
Akirin1	1091	729	706	982	574	1071
Coq7	2724	1714	1557	2140	1967	3080
Huwe1	16132	12550	8888	13609	9933	13440
Pcdhga9	2595	1721	1370	2422	1385	3257
Polr1e	984	736	530	971	571	1259
Rab22a	2166	1560	1394	1826	1323	2141
Tars	1555	1149	939	1287	948	1518
Zfp260	2435	1805	1424	2131	1421	2259
Fam227a	84	49	42	78	50	102
Mfap4	458	405	221	694	236	525
Prmt7	1198	711	646	945	583	1095
Wbp1l	3438	2293	1843	3079	2465	3443
Acbd7	7	7	5	6	8	17
Trim17	7	5	4	4	2	6
Alpl	2299	2110	1610	2347	1578	2167
Kank4	105	58	40	71	30	87
Lrp5	3098	2004	1438	2878	1842	3038
Mta1	2639	1885	1298	2417	1666	2914
Nfatc4	219	131	98	216	123	223
Nr1h2	1636	937	647	1251	996	1537
Nup210	230	108	78	148	120	261
Slc37a3	958	583	460	718	458	850
Capn10	231	113	107	215	137	207
Chuk	2161	1186	1114	1500	1078	1948
Cluap1	637	463	335	487	352	611
Ddx31	196	128	124	194	81	162
Gm10094	104	84	61	70	75	80

Lzic	1075	716	693	949	586	1040
Nrros	219	137	110	246	107	252
Nynrin	497	321	209	415	303	533
Rangap1	1316	815	656	1092	620	1113
Sppl2a	5265	3511	2786	4846	3210	5963
Wnt9a	278	134	132	184	128	206
Zcwpw1	41	33	26	31	40	69
H2-T10	89	34	47	59	59	109
Nup205	987	675	468	820	474	853
1110059G10Rik	545	414	360	445	293	632
Cgrrf1	1017	668	675	829	533	1113
Gm21119	1	1	0	1	1	3
Zfp871	3864	2155	1988	3337	2114	4247
Hmgb2	110	84	83	86	69	265
Mkl2	3099	2328	1693	2568	2345	3663
Samhd1	2165	1333	1069	1807	1328	2304
Cops2	5774	4394	3558	4986	3439	5691
Spdya	2	7	5	3	8	16
Comt	4383	2685	2702	3095	2537	4357
Tnfrsf19	391	301	288	303	200	516
1110028F11Rik	3	0	0	0	1	0
1600023N17Rik	1	3	1	5	2	3
4833420G17Rik	1158	693	579	872	672	1438
4930526I15Rik	69	26	51	58	51	93
4931440F15Rik	5	1	1	2	0	2
6030458C11Rik	1212	804	713	938	717	1128
A330023F24Rik	1450	757	695	674	713	1447
AB124611	37	19	15	32	22	43
Ado	1470	975	832	1339	757	1240
Astn2	99	85	46	92	31	95
BC049635	2	2	3	3	3	7
Bloc1s2	170	100	100	123	75	167
Btbd1	6401	4617	4293	5412	3946	6362
Ccdc120	29	14	15	19	22	41
Ccl22	10	2	7	6	1	7
Ccno	14	11	4	7	9	5
Cdt1	73	25	25	60	25	86
Cep72	42	29	14	29	35	51
Cep76	556	389	361	498	297	546
Chkb	1844	1048	945	1294	1075	2384
Cks1b	105	68	56	82	37	102
Clcn4-2	5694	3838	3531	4855	3260	5271
Cln8	1061	648	578	895	515	928
Cmya5	57833	46786	37085	53393	39572	39723
Cnnm3	950	599	446	879	522	1015

Cox20	2020	1721	1469	1724	1328	2412
Crtam	2	3	3	3	2	11
Cyp2u1	390	261	291	414	233	232
Derl2	1525	940	877	1380	950	1695
Dhps	564	291	273	443	322	614
Diexf	480	321	259	326	301	402
Dlx6	0	1	0	0	0	4
Dlx6os1	6	2	3	3	5	11
Dnm1l	9764	7111	6292	8517	5897	9328
E130201H02Rik	3	0	0	0	1	0
Eed	1141	666	655	918	578	1030
Fam120c	1909	1371	1046	1619	1122	1605
Fam154b	20	11	14	8	11	19
Fam228a	20	16	13	11	13	18
Fbxl13	15	14	3	7	6	12
Fbxl5	2536	1556	1329	2145	1596	2714
Fgf7	150	140	91	164	78	123
Fnbp1	3159	2171	1808	2537	1991	2586
Gale	28	23	26	31	7	33
Gga3	951	610	481	782	450	849
Gm10052	453	320	259	317	254	473
Gm15816	103	70	72	70	42	129
Gna11	3339	2128	1945	3109	2024	3833
Grhpr	1329	806	677	964	595	1347
Gstcd	88	43	40	53	35	70
Gucy1a2	763	404	338	673	335	891
Hirip3	356	296	206	266	224	363
Hist1h4k	6	3	1	8	3	6
Hnrnph1	7482	5379	4401	6095	4217	7591
Il16	426	287	183	420	223	480
Il22ra1	15	5	20	15	29	18
Itprip	763	383	331	648	472	629
Kcnj11	7101	4534	3540	5895	3623	5555
Lgalsl	2183	1491	1297	2131	1412	2476
Mir5106	0	1	0	0	0	4
Mir6537	2	0	0	3	1	1
Mir6999	45	30	28	25	25	44
Mkln1os	32	24	32	28	16	38
Mmp8	4	0	4	8	3	4
Mphosph8	2343	1822	1667	1856	1493	2756
Msantd2	587	342	295	400	242	600
Mtcl1	230	135	114	159	125	170
Mtf2	897	629	495	724	576	1215
Myoz1	13	5	2	6	7	19
Narg2	494	326	284	361	288	456

Nckap5	400	277	182	312	228	259
Ncoa5	1205	786	660	1005	652	1026
Ncs1	434	225	220	353	200	284
Nfam1	126	91	57	114	50	92
Nop56	702	530	408	555	411	709
Nr0b2	279	178	104	321	122	284
Otud7a	5	0	0	1	0	1
Pak6	646	392	359	637	449	602
Pcdhga8	2584	1718	1374	2414	1374	3272
Pcdhgb2	2695	1824	1462	2546	1460	3432
Pcdhgb4	2635	1742	1394	2460	1397	3317
Pde1c	3608	3033	2231	2927	2582	3265
Pfas	976	506	377	624	625	958
Phka1	3924	2524	2008	3140	2203	3027
Pla2g2f	4	0	3	1	1	2
Prrc2a	9734	6581	4628	8365	4916	7364
Pyurf	2399	1744	1592	2035	1492	2413
Raver1-fdx1l	1489	955	786	1209	776	1314
Rbm14	772	417	310	580	319	549
Rbp1	268	258	175	347	180	400
Rims4	17	4	10	8	7	9
Rras2	2095	1222	1420	1637	1244	1926
Sik2	676	584	365	639	408	540
Sike1	1212	850	738	1143	622	1129
Slc22a1	44	20	23	41	35	40
Slc35f2	5	2	3	8	4	5
Slc44a3	7	12	1	4	4	6
Slfn4	15	8	9	16	7	26
Sox13	888	518	318	725	519	807
Srgap3	75	54	43	51	49	56
Sri	1937	1291	1061	1778	1073	2475
T	3	0	0	0	1	0
Tada2a	409	290	229	306	223	408
Taf9	914	727	609	735	598	988
Tigd3	17	7	8	10	9	31
Tle4	743	449	349	635	480	723
Tmed4	3989	2631	2400	3293	2198	3851
Tprkb	568	407	368	470	311	609
Tpx2	94	45	28	62	26	166
Ubap2l	5527	4456	3092	4950	3359	4729
Usp26	3	0	0	1	1	5
Zan	3	1	4	1	0	4
Zdhhc9	1844	1250	1093	1716	964	1628
Zfp180	1147	650	654	932	596	946
Zfp26	974	552	485	784	531	1147

Zfp429	53	78	69	90	60	156
Zfp459	29	26	37	28	40	73
Zfp551	94	51	38	66	44	50
Zfp704	2351	1422	1238	1700	1335	1984
Zfp850	66	57	56	72	61	113
1500011B03Rik	72	48	43	63	60	91
Alkbh6	343	240	172	278	255	398
Epb4.1l4a	730	377	301	407	499	817
Adrb2	155	93	68	131	88	185
Mir3097	0	3	1	1	4	4
Fitm1	1998	1066	965	1735	1101	1969
Nap1l1	3089	2034	1778	2532	1687	2960
Sqstm1	18314	12147	10215	16234	12639	20434
Tep1	976	593	427	644	481	867
Trim37	928	632	494	770	517	777
Rab11fip3	2289	1544	1185	1948	1268	1776
Ccdc88b	88	47	44	71	62	98
0610010F05Rik	877	556	461	688	460	769
2010107G23Rik	58	38	43	53	46	91
2410004B18Rik	592	374	437	477	327	644
4930525G20Rik	14	15	8	27	7	23
4933404O12Rik	53	30	24	37	19	42
4933424G05Rik	4	3	5	6	1	1
6430571L13Rik	190	123	175	170	124	184
9230116N13Rik	22	9	11	15	20	24
A430105I19Rik	2537	1706	1559	2082	2081	2827
A730098P11Rik	2	5	1	6	2	7
Abca8a	6951	5103	3768	5969	3314	7159
Abcf3	2393	1745	1373	2070	1351	2275
Ackr3	4260	2681	2262	2959	2585	3544
AF251705	53	18	18	42	17	68
AI846148	472	256	225	399	256	515
Aldh18a1	394	215	256	335	172	365
Anks1	4149	2302	1813	3444	1837	2752
Arhgap24	1012	763	696	1034	457	1115
Asic1	16	9	5	6	3	15
Atg9b	55	20	17	24	15	40
Atl3	2114	1292	1076	1881	1095	2546
Atoh8	529	291	248	415	290	613
Atp6v1h	1193	855	780	1070	644	1256
Bst1	37	18	18	30	20	52
Btbd8	22	16	14	20	18	36
Btd	543	328	252	521	300	549
Cabyr	10	3	7	6	4	5
Cacnb3	187	111	61	176	83	206

Casc4	624	490	534	675	349	699
Cd200r1	26	21	14	23	10	21
Cd99l2	4993	3260	2969	4366	2633	4564
Cdca4	255	158	134	248	143	297
Ceacam16	0	0	1	0	0	4
Chic1	169	86	110	150	101	231
Cib1	279	158	146	272	138	335
Cited4	502	297	269	505	212	289
Ckap2	33	20	16	16	8	103
Clec4a2	59	36	25	57	27	81
Clock	2496	1654	1634	2163	1446	2390
Cmah	138	92	85	97	73	138
Cmklr1	802	581	370	836	268	601
Coa6	518	345	285	403	246	527
Ctnnbl1	483	346	243	422	315	584
Cxcr5	2	3	1	4	3	4
D030028A08Rik	25	11	14	14	10	19
D830026I12Rik	156	92	95	66	154	254
Decr2	805	530	528	668	400	881
Deptor	2038	1326	1219	1473	1151	2125
Dock10	405	294	210	435	228	481
Ear2	2	2	2	4	3	4
Ebpl	117	52	49	101	53	132
Egfr	287	216	209	285	131	317
Eif6	716	399	328	717	323	770
Elavl2	2	5	2	2	0	4
Ergic1	1543	990	862	1270	816	1415
Fbxo27	10	8	4	11	2	3
Fetub	47	23	32	32	20	42
Fkbp6	2	1	1	0	1	9
Flywch2	6	7	4	7	9	11
Foxl1	2	1	1	1	0	1
Fzd6	1592	995	901	1296	1011	1920
Gadl1	3	2	2	5	1	7
Ggps1	1038	755	607	832	657	936
Gigyf2	2404	1878	1485	2080	1543	2274
Gltscr2	3646	2370	1965	3207	2236	4179
Gm5086	7	3	6	10	2	16
Gm9958	43	36	20	42	33	59
Gpc2	26	15	13	11	14	21
Gpc4	1045	689	553	1114	547	1171
Gpcpd1	18882	13783	10102	13295	11363	18095
Gpr52	2	1	1	3	3	2
Gpsm2	92	64	54	76	31	97
Grm7	8	6	7	8	8	17

Gtf2h3	574	397	309	439	315	541
Gzf1	2213	1485	1396	1914	1221	2182
Heatr9	0	1	1	1	1	3
Hist1h2ae	2	1	1	1	0	1
Hoxc6	0	1	3	1	0	1
Hspa12b	1853	1000	741	1496	856	2028
Hspbp1	672	366	308	505	262	578
Icam1	631	372	276	467	249	565
Ifi205	319	256	420	439	246	865
Inpp5f	367	415	226	351	268	806
Irx2	289	274	172	378	228	332
Jun	3014	1865	1288	2353	1409	2372
Katnal1	886	651	496	753	537	745
Kcnu1	1	5	0	3	2	8
Kif2a	1196	856	747	966	710	1228
Klhl40	1039	574	566	812	466	961
Lgals1	3074	2282	2195	3033	2057	4565
Lhx2	1	2	1	1	0	1
Lmnb1	215	139	128	196	128	300
Lrrc4b	993	713	601	1063	347	951
Mab21l2	1	2	1	3	2	3
Mal	752	511	747	506	549	969
Maoa	302	204	207	287	226	386
Map3k1	1789	1176	914	1536	1202	1843
Mbnl2	18533	13103	9995	15206	10210	16619
Mfge8	4586	2700	2499	4541	2316	5319
Misp	30	22	14	34	6	15
Morc2b	16	15	12	19	12	33
Mrpl44	2117	1230	1271	1997	1282	2331
Mthfd2l	232	157	145	195	134	195
Mtmr2	1350	918	809	1224	847	1702
Nbas	1703	1078	846	1401	918	1271
Ndn	232	135	125	205	128	295
Ndufa11	5850	3681	3269	5735	3202	6924
Nhs	25	22	13	19	25	41
Nod1	1371	783	580	1090	811	1320
Npr2	3129	2001	1553	2724	1852	3381
Nr3c1	6631	4629	3975	5457	3935	6094
Nrd1	14170	10147	8601	11071	8991	12957
Obp2b	1	1	0	0	4	1
Optc	7	6	5	4	3	7
Panx2	14	10	3	5	26	8
Pcdhga2	2726	1811	1442	2545	1462	3393
Pcdhga6	2664	1749	1399	2482	1400	3327
Perp	6342	4396	4204	5803	5040	7328

Pex10	486	314	284	362	282	444
Pex2	1809	1108	1049	1538	854	1751
Pgbd5	15	17	12	13	10	15
Phlda1	624	734	480	1016	518	901
Pik3c2g	10	3	1	3	4	1
Pitpnm3	188	143	122	159	172	247
Ppp1cc	251	131	104	236	107	282
Ppp1r14c	8272	5288	4540	6949	4029	7704
Ppp2ca	10217	6889	6742	8968	5831	9820
Ppp2r3a	27650	20496	16515	23487	16924	24023
Prkaa2	11829	8299	6910	9890	7545	8886
Prkab1	1380	982	599	1118	1008	1389
Psmd4	3924	2591	2137	3334	2522	4582
Ptrh2	605	334	317	518	350	663
Pxk	1660	1143	843	1475	1130	1718
Rasef	88	63	30	80	29	50
Rbm4	456	288	249	439	268	518
Rbmx2	133	118	83	122	96	236
Rcn2	2398	1542	1428	2015	1267	2300
Rergl	1	2	0	1	1	5
Ric3	159	122	118	117	148	273
Ripk4	16	14	10	26	3	39
Rnf138	425	276	253	386	253	566
Rnf214	1297	1061	878	1190	853	1284
Rpl36a	14	12	11	10	5	17
Rpn1	3176	1977	1864	2771	1645	2845
Rrp36	376	219	177	252	180	332
Sec31a	5524	4065	3113	5000	3093	4795
Sf3b6	1018	727	608	783	571	1099
Sfi1	830	371	310	478	331	639
Sgcg	9105	5486	4388	8095	5656	8716
Sgsh	343	258	197	321	234	445
Shroom2	273	164	166	229	168	376
Siah1b	96	82	61	98	85	128
Skiv2l	1492	853	631	1223	830	1475
Slc22a17	156	71	55	125	77	155
Slc25a16	1716	1112	963	1385	973	1514
Slc27a6	20	7	6	9	6	11
Slc4a5	0	1	3	1	0	1
Snrpd1	515	437	368	425	308	642
Socs7	1875	1242	1034	1675	1010	1506
Spata18	10	10	9	4	4	16
Spem1	0	1	1	1	1	3
St6galnac4	886	546	426	887	464	916
Stat2	1425	885	645	1131	713	1862

Svip	2573	1645	1469	2065	1449	2308
Tagln2	2273	1354	997	2001	1110	2533
Tasp1	387	268	247	332	227	344
Tceb2	1778	1067	925	1488	961	2163
Tfap2b	3	1	2	3	3	5
Tgif2	17	8	6	26	6	15
Tial1	3915	2281	2064	2825	2042	3577
Ticrr	8	10	2	4	2	9
Tldc1	538	367	294	452	283	472
Tmem212	1	1	0	2	0	3
Tmem40	7	3	5	6	1	4
Tmtc1	12624	8170	6839	9833	7244	10478
Tprgl	4713	2862	2423	4175	2683	5191
Tpsab1	0	2	2	1	0	1
Trf	1075	943	814	1074	505	1455
Trim7	759	336	363	398	354	659
Ttc25	25	16	8	32	15	22
Upk3b	20	6	15	23	32	5
Upp1	158	112	102	109	79	183
Ush1c	388	248	188	231	168	425
Usp6nl	438	275	238	393	265	517
Uvssa	1019	823	625	716	897	1523
Vapa	3812	2676	2603	3432	2206	3850
Wbscr27	31	23	30	27	38	55
Wdtc1	3480	2084	1672	2771	1851	2486
Xlr4c	0	2	2	1	0	1
Xrcc6bp1	132	78	70	76	66	126
Xylt2	538	353	255	427	334	651
Yipf1	518	252	239	444	249	559
Ylpm1	2292	1859	1313	2101	1366	2108
Zcchc4	241	151	149	171	122	250
Zfp395	936	564	401	800	533	939
Zfp433	21	21	26	11	13	38
Zfp446	345	215	163	243	166	307
Zfp623	378	238	226	365	264	406
Zfp706	4910	2845	3150	4110	2395	4842
Zfp707	201	102	83	133	128	222
Zfp708	217	216	176	197	166	244
Zswim7	288	161	165	206	213	347
Eif3a	11477	9340	7741	10127	7498	11707
Eif4ebp1	1852	1211	964	1529	1149	2181
Poc1b	432	330	238	447	294	491
Sigmar1	514	340	297	430	240	512
Krt8	3	1	2	5	2	4
A530054K11Rik	501	379	303	380	320	487

Akna	358	239	156	301	220	407
Ift172	2374	1627	1192	1997	1285	1936
Zfp955a	684	438	439	660	296	650
Fblim1	8313	5415	4465	6549	4885	6598
4833403I15Rik	82	35	87	58	81	170
Il4i1	9	5	5	12	4	14
Zfp423	122	96	45	137	75	129
Mtmr6	2732	1750	1548	2740	1764	2681
Npy4r	7	6	6	6	1	8
Fbxw2	1937	1196	1122	1723	1118	2390
Atf1	1854	1377	1111	1627	1088	1770
Ecel1	1	1	0	0	2	3
Lhfp	1646	1306	1115	1850	1005	2346
Nanp	1	0	0	0	3	0
Prss42	1	0	0	0	3	0
Tubb2a	457	266	268	488	215	584
Tnfrsf4	28	11	8	13	6	20
4930556M19Rik	23	22	20	21	27	46
Mir147	7	1	4	1	1	6
Cdca7l	13	5	3	7	5	22
Crabp2	0	1	1	1	3	1
Crp	4	0	2	2	8	1
Dnajc5b	0	1	2	2	0	6
Gpatch8	2936	2205	1370	2050	1603	2951
Gtf2b	875	487	478	620	450	794
Hgd	0	1	1	1	3	1
Hus1b	1	2	1	0	0	2
Ltbp3	1942	1287	852	1859	1052	2019
Mitf	4047	2900	2300	3186	2526	3616
Psma8	50	23	37	43	25	97
Tchh	19	21	12	18	9	19
Tm9sf2	9830	6480	5884	8403	5141	9482
Tmem65	14351	9324	8052	11710	7996	12303
Tnks1bp1	2708	1677	1245	2442	1506	2763
Trim26	1010	681	590	872	665	1240
Ythdf3	6681	4408	3495	5437	3580	5719
Mageh1	239	125	142	225	123	310
Bre	1015	829	606	1043	704	1271
Ifih1	891	596	468	815	474	1151
Mir702	106	95	31	92	41	72
Far2	1	0	1	2	0	3
Mterfd1	1347	958	885	1140	774	1438
Pla2g2e	1	0	1	2	0	3
Ran	2156	1478	1418	1889	1233	2160
Sorl1	503	368	280	498	270	344

A630089N07Rik	27	25	23	32	13	23
Aamp	3337	1839	1706	2855	1855	3484
Angel2	2163	1320	1131	1843	1309	2354
Angptl6	93	38	44	64	33	63
Ano10	1279	704	631	991	929	1141
Asna1	1598	978	845	1404	952	1727
Atrx	5852	3970	3139	5019	3851	6769
BC003331	2328	1715	1441	1871	1452	2351
Calm3	5932	3904	3324	5688	3525	6866
Ces5a	5	3	1	2	1	3
Chil1	2	1	1	4	1	3
Clstn3	3	4	1	10	4	1
Cltb	2591	1786	1402	2234	1641	3105
Cnot7	3121	1966	1818	2486	1776	2856
Col4a5	2412	1597	1354	2143	1815	2388
Cpne9	1	1	2	0	1	1
Creld2	413	293	261	335	205	461
Dab1	2	1	1	0	0	2
Dars	4365	2956	2413	3623	2348	4083
Dmd	6434	4344	4162	7189	4529	6494
Dnajb12	1479	1079	921	1277	918	1381
Doc2b	38	14	13	34	14	43
Elf4	713	432	363	559	319	661
Fam20b	6459	4495	4061	5769	3884	5488
Fam89a	43	26	17	44	18	58
Fbxo4	426	243	235	311	197	407
Gfra3	84	37	34	46	44	124
Gm13446	1	2	1	2	2	4
Gm3500	1	0	1	3	0	2
Gna12	3457	2170	2053	2533	2324	2550
Gpatch2	663	470	412	542	396	640
Gpr63	17	20	19	17	10	24
Gpr89	585	431	312	498	319	522
Gsg1	2	0	0	2	0	3
Hnrnpll	1417	869	702	1078	697	1286
Hoxb3	42	15	15	26	26	45
Ibtk	2648	1765	1477	2301	1389	2388
Inppl1	9936	6282	4914	8571	4644	8103
Map1s	372	232	130	339	215	340
Med22	722	355	360	662	373	720
Moap1	680	396	368	566	283	626
Mrpl42	9774	7835	6203	8029	6021	10693
Nup54	523	353	319	448	293	468
Pcdhgb8	2667	1739	1384	2437	1398	3325
Pxmp2	2065	1338	1307	1681	1484	2604

Rnf114	3300	2261	1732	3038	2004	3687
Rnls	260	291	179	423	261	269
Rpa2	497	302	271	464	313	525
Rpp25l	315	154	159	295	153	341
Rps15a-ps6	12	8	6	7	0	14
Slc52a3	47	26	28	46	33	56
Ssr4	881	548	486	722	558	1046
Syde1	926	566	467	791	501	1107
Tctex1d2	151	81	100	150	87	200
Tfrc	3308	1462	1789	2868	1241	2336
Tinagl1	3028	1658	1224	2547	1382	3281
Tmem130	6	0	1	1	9	2
Tusc2	1132	837	710	997	628	1178
Tyro3	88	45	27	105	39	68
Ube2f	1230	953	938	1141	740	1426
Ugdh	740	535	498	671	414	760
Usp12	1246	885	703	1084	668	1182
Ybx2	1495	874	554	862	566	1482
Hk2	18804	12917	11001	15861	11074	15820
D030056L22Rik	539	355	313	448	252	545
Necab1	79	63	13	52	21	58
Pex11b	1019	632	593	838	478	1029
Tsr2	696	502	371	574	364	668
Zfp597	943	570	531	745	527	787
Cyp1b1	440	385	412	431	278	593
Dcaf15	376	212	173	284	210	454
Rgs10	190	115	101	132	83	188
Hivep3	108	100	83	114	110	158
1600002K03Rik	97	51	68	69	52	84
Ap1g1	3197	2250	1885	2892	1795	2898
Arfgap1	1200	795	591	1082	731	1277
Asb5	827	485	519	616	457	800
Ddx54	1288	804	647	1035	636	1119
Mir7117	4	0	2	5	4	2
Pgm2l1	1168	687	601	1042	628	1320
Slmap	12816	9394	7744	10473	8118	11587
Ube2c	40	27	24	23	10	52
Carkd	1025	733	531	890	634	1267
Kdm3a	1931	1280	1036	1594	1308	2171
Ubap2	4254	3347	2274	3680	2576	3641
Vasp	1060	581	590	1087	511	1191
Col9a3	19	9	6	19	9	21
Dennd1a	1285	936	687	1078	739	1138
Elk1	794	601	456	753	381	781
Gnpat	13509	8366	7476	10779	7351	11508

Impact	1746	1130	936	1257	993	1657
Mcoln2	28	6	12	16	7	14
Mir1843b	2	1	1	1	4	3
Mir5114	36	25	13	33	9	22
Mllt11	485	298	297	347	280	462
Myc	137	77	60	95	94	158
Pdia4	1393	951	928	1268	701	1496
Scarna3a	2	1	1	1	4	3
Slc45a2	1	1	2	2	3	3
Ccng1	68128	46242	45033	57695	42271	57448
Lgi3	176	122	120	172	92	145
Prpf39	2249	1577	1428	1854	1596	3056
Tsta3	584	370	323	478	373	718
Twist2	28	9	14	18	9	16
Zfp91Cntf	2848	2113	2037	2395	1940	2895
Spns3	2	1	1	2	0	7
Abcg3	26	19	10	28	13	37
AI450353	342	96	92	102	156	148
Ak7	1	1	2	1	1	0
Aqp11	38	23	17	43	28	33
Atp8b5	4	0	0	2	2	4
Ckb	1419	875	713	1632	572	1706
Cst7	3	2	2	4	0	0
Dhx37	745	511	373	612	381	656
Dhx57	842	541	397	648	513	1005
Eif3f	4955	2805	2505	4209	2666	5556
Enkur	70	49	28	71	28	108
Entpd7	114	65	56	103	64	136
Gm11545	2	2	0	1	0	1
Gm5150	1	4	0	4	2	4
Hint2	1173	749	745	1054	719	1546
Hmga1	189	233	133	286	134	349
Klhl30	1681	1074	893	1450	807	1515
Lrrc20	1548	1052	1048	1365	978	1380
Mir3076	19	7	11	19	7	30
Mmd	2017	1122	1059	1893	1042	2176
Mtss1	1568	1159	1006	1357	1234	1944
Paip2b	2473	1496	1298	2077	1226	2064
Pomt1	616	426	304	505	322	518
Ptpn21	1941	1419	1248	1510	1347	1756
Rabep2	276	190	122	223	181	323
Tmem128	411	269	213	350	241	524
Tom1l2	8151	5311	4177	6892	4312	6481
Wash	814	490	395	676	505	848
Npm3-ps1	1	10	0	9	12	1

Tmem82	721	291	311	438	241	615
Rbm20	15257	11133	8715	11249	10131	13089
Zfp947	55	27	34	52	30	77
Nipal3	2122	1282	1111	1715	1101	1767
Steap3	1593	1059	839	1341	822	1433
Armcx1	234	169	141	210	162	317
Fam162a	2685	1682	1709	2272	1814	3315
Top1	4674	3453	2955	4261	2674	4614
Cldn1	25	6	6	13	7	7
Apmap	1606	1016	1001	1286	811	1730
Arl6ip1	1570	832	740	1286	776	1756
Eif2s2	2772	2124	1942	2190	1736	3305
Habp4	3900	2972	2416	3548	2184	4014
Lig4	599	412	400	503	341	615
Ndfip1	3117	1863	1838	3071	1764	3453
Surf1	1332	762	684	1073	753	1533
Ndufaf5	825	578	474	809	472	1051
Tbc1d13	743	452	340	677	406	772
Usf1	814	510	410	715	468	920
Vwa5a	2645	1659	1462	2430	1606	2862
Anapc11	1188	699	505	994	619	1258
Opa3	2274	1699	1560	2191	1432	2040
Prdm8	5	4	6	6	3	18
A330009N23Rik	85	56	39	75	53	106
Ggct	479	375	316	353	303	540
Mical3	4505	3709	2833	3828	2994	4478
Pcdhga3	2658	1772	1398	2468	1404	3339
Tecpr2	767	525	377	623	388	680
Unc119b	974	484	422	893	449	944
1700086O06Rik	41	31	25	39	25	73
C030037D09Rik	150	192	143	190	106	195
Creg1	6508	4436	3659	4646	4134	5724
Mrpl16	2312	1624	1547	2178	1593	2975
Pabpc1	7106	6659	4583	6111	4570	8454
Pcdha5	35	14	22	24	14	25
Qrich1	2518	1801	1546	2217	1484	2372
Cenpl	94	42	34	64	44	118
Chrna2	56	21	55	13	79	120
Clnk	1	0	0	1	2	0
Dclre1c	421	272	228	242	216	467
E330011O21Rik	256	208	162	264	193	330
Grb7	1	0	0	1	2	0
Hpx	13	3	6	4	8	2
Isca1	7300	4950	4483	5954	4445	6699
Klra12	1	2	1	3	1	4

Lgals3bp	1992	1081	726	1743	760	2296
Pnpla2	14251	8741	6862	10727	9130	15956
Rrp9	550	306	278	404	244	500
Tmem214	1535	1032	750	1246	762	1396
Traf7	1770	1075	865	1514	990	1926
Ankle2	2090	1458	1238	1791	1171	2020
Arl5c	19	14	12	15	19	30
Farp2	902	527	504	642	519	778
Gdf1	23	8	12	16	7	13
Inf2	1570	821	494	1112	786	1543
Lipt1	344	246	223	231	234	335
Map2k3os	157	146	118	119	119	173
Nacc2	1752	1060	1065	1451	1058	1346
Perm1	17468	11476	10689	15633	10152	13553
Rltpr	15	7	9	23	8	15
Rpl15	1367	778	763	1067	598	1365
Sacm1l	3264	2104	2065	2773	1919	2987
Sec16a	3808	2712	2124	3519	2107	3110
Xrcc5	1169	883	680	1033	712	1029
Zfp629	2084	1503	1297	1868	1228	1963
Aifm2	1023	667	583	862	757	1089
Alkbh3	1226	948	805	1130	882	1680
Edc3	454	320	225	412	267	559
Gbx2	10	2	4	7	6	12
Ifnar2	1300	950	716	1243	786	1596
Lrrc75b	96	51	36	75	66	89
Ntpcr	367	198	199	341	209	394
Rps29	4	4	2	7	2	8
Zkscan16	3	3	3	2	5	9
Zswim1	230	149	119	250	157	208
2310016D03Rik	13	5	10	7	5	11
Enox1	31	21	23	21	16	32
Zbed3	559	367	322	415	305	556
2010015L04Rik	68	42	36	27	48	50
Anp32e	4944	3193	2969	4101	2775	4573
Fam150b	23	9	17	11	7	25
Bub3	1307	814	708	1246	729	1454
Ubxn2b	389	300	256	319	238	409
Cutal	43	28	31	34	25	35
Naprt1	471	212	175	384	199	483
Ttc17	2174	1584	1260	1929	1336	1769
Kdelc2	964	597	518	786	444	924
Tmem41a	480	328	251	503	258	565
Tut1	469	343	263	365	252	486
Zfp934	117	103	105	131	113	189

Arhgef39	21	9	9	14	11	32
Mmgt2	195	129	85	167	83	140
Arrdc3	2764	1947	1936	2334	1728	2916
Bhlhe22	8	15	9	18	5	32
Cdc25a	309	186	168	223	152	293
Ergic2	2578	1831	1777	2197	1612	2650
Lhfpl3	6	3	1	4	3	10
Tcp1	9095	6472	5676	7780	5351	8858
Tmem119	122	120	83	151	44	125
Ulk3	463	261	220	362	257	543
Vmn2r57	15	21	12	9	14	18
Atm	1149	718	613	826	622	1101
Dpf2	2273	1619	1296	2002	1273	2078
Gm13826	46	41	18	25	20	51
Dhx16	1295	722	637	988	615	1095
Ephx1	2213	1395	1028	1910	1384	2152
Mir214	10	7	2	3	5	6
Pa2g4	3161	2472	1887	2730	1834	3357
Pomc	2	0	3	1	5	4
Ptprc	489	333	255	453	169	474
Lrrc30	14	8	8	6	6	13
Tmie	58	53	34	68	51	72
Cxcl10	10	7	7	17	2	21
Tomm70a	4288	2996	2940	3817	2701	3977
Zfp91	10163	6858	6986	8398	6394	9826
Pcdhgc5	2613	1733	1374	2418	1376	3278
Ankrd13d	41	23	17	25	19	28
Runx1t1	345	292	219	357	254	467
Usp25	5246	3718	3112	4430	3158	4737
A830082K12Rik	35	23	19	30	31	42
Foxj3	1730	1279	1001	1513	1084	1443
Snora5c	10	2	3	6	2	2
Tlr4	822	618	586	761	513	828
Wbscr22	472	269	250	352	216	437
Zfp12	741	439	327	535	350	619
Pdcd11	1343	920	724	1109	769	1155
Rps10	973	280	339	734	332	956
Sox8	16	12	9	29	8	18
Dcaf4	490	306	247	342	259	446
Igsf23	53	28	29	31	33	32
Magix	1225	712	585	961	792	1219
Riok3	5652	2832	2824	3739	3609	6248
Smad7	932	489	407	898	484	805
Tk2	437	272	257	380	278	536
Lrat	12	12	10	10	12	3

Pcyt1a	7563	4613	4467	6533	3961	6576
Armcx5	469	328	292	424	345	570
Gabpa	3750	2683	2097	3028	2242	3450
Vstm5	8	7	5	9	6	16
1110020A21Rik	22	21	14	14	31	36
Ackr2	53	36	38	53	27	37
Dpep3	2	0	2	0	0	2
Grm8	2	0	2	0	0	2
Mir22hg	949	579	498	617	537	1484
Ppm1b	4122	2608	2417	3320	2339	3732
Zcchc9	435	323	327	407	242	483
Cldn25	1752	1178	1096	1545	975	1694
E330020D12Rik	9	4	2	3	2	6
Kdm5d	0	0	626	0	763	1119
Krit1	2312	1664	1376	1858	1371	2345
Lsm8	591	394	333	565	363	684
Nppa	20253	14462	14786	27102	8825	34249
Nr5a1	1	0	0	0	2	1
Rabac1	2387	1147	976	2035	1103	2265
Sh3d19	1332	1090	893	1426	651	1424
Slc9a7	77	49	40	92	52	71
Taf8	664	415	329	549	425	716
Tas2r108	1	0	0	0	2	1
Ccl24	22	31	16	25	29	45
Cdkn1b	2620	1783	1353	2337	1740	2729
Cyp4a14	0	1	1	2	0	3
Gnao1	1346	932	710	1340	1031	1163
Myrip	346	193	179	375	176	349
Tgfb1i1	651	461	332	666	377	728
Atp8b4	54	33	25	41	9	54
Dctn2	4523	2808	2425	4053	2530	5273
Gp1bb	60	40	29	72	34	65
Kcng2	2985	1979	1492	2667	1508	2306
Knop1	1268	911	836	1198	819	1737
Mycbp	759	575	513	677	464	775
Simc1	180	143	99	139	94	188
Slit3	504	469	351	512	328	514
Ubtf	3895	2728	2186	3401	2134	3553
Vim	12034	7705	5569	10833	5645	15549
2310057M21Rik	457	297	259	366	252	404
Aatf	445	362	289	382	287	447
Arhgap4	107	52	39	70	40	71
Fam208b	1464	982	850	1305	777	1331
Mrpl38	1954	1329	1001	1556	996	1889
Nr4a1	256	243	258	264	296	452

Synj2	2992	1834	1569	2044	2268	3287
Tmem102	13	2	0	5	11	6
Usp47	8985	6939	6291	8212	5759	9055
Cdc23	1947	1272	1015	1615	990	1759
Cep57l1	157	104	67	136	105	167
Fam126a	3536	2120	1726	2440	1930	3107
Fut4	62	41	33	45	22	63
Gldc	22	10	14	17	12	39
Hapln4	1	2	0	0	2	5
Mab21l1	5	3	2	3	2	2
Lcor	121	81	52	140	66	121
1700001L05Rik	26	12	11	26	8	38
Rho	7	4	1	6	6	7
1700123I01Rik	4	9	2	5	2	4
1810012K16Rik	6	4	6	1	4	7
2900041M22Rik	15	8	8	7	6	13
A630077J23Rik	30	19	13	27	21	36
Ccdc11	5	11	8	15	10	13
Cinp	642	442	404	532	361	647
Hmga1-rs1	185	231	130	284	129	345
Lpxn	47	22	24	33	6	46
Med29	147	88	95	126	67	133
Mir6992	4	2	2	2	4	8
Mrpl1	1989	1368	1337	1642	1173	2119
Ogfod2	380	205	216	373	224	390
Pcdhga4	2657	1780	1406	2468	1411	3334
Rps28	617	413	324	476	314	597
Sesn2	169	68	79	119	60	117
Slc26a8	13	6	7	9	9	21
Trpv1	17	3	4	2	3	12
Ydjc	52	14	19	25	17	30
Zbtb5	377	240	181	301	166	333
Zfp282	396	211	216	367	236	403
Zkscan2	6	16	10	7	23	22
Shmt1	326	220	169	186	194	319
Sh3gl3	17	12	6	16	16	17
Gm10767	22	19	29	32	30	39
Cmpk1	1702	1046	1094	1543	1083	2060
Pgls	732	418	384	662	408	790
Lin7b	10	4	7	7	7	19
Pcdhga11	2664	1752	1398	2447	1406	3310
Ccdc94	198	121	104	149	95	173
Commd6	450	295	285	429	318	514
Cyp26c1	1	0	0	2	1	0
Fndc8	1	0	0	2	1	0

Pde4a	3506	2574	2051	2900	2124	3372
Asxl2	2225	1578	1202	1640	1319	2178
Tmem161a	2048	1178	924	1767	1157	1962
Kcnq1	1908	1248	938	1649	783	1818
Bpifb4	2	0	0	4	0	1
Amh	4	3	3	2	3	2
Lsg1	796	567	513	580	459	917
Atrnl1	2200	1310	1046	1843	1025	1714
Bzw2	7294	4756	4436	5885	4128	7105
Gapvd1	2415	1595	1322	2100	1280	2097
Mterfd3	1364	912	857	1088	766	1435
Pcdhgc4	2614	1734	1386	2420	1378	3287
Phf7	285	176	155	228	205	320
Psca	1	2	1	1	3	4
Wfdc6a	2	2	2	5	1	5
Xlr4b	6	20	1	13	1	47
Gbp10	182	13	14	38	185	20
Gmppa	1110	663	569	820	559	1031
Gm4070	199	120	98	181	103	248
Gvin1	199	120	98	181	103	248
Mir6516	15	12	7	8	6	15
Mpv17l2	293	212	177	273	196	386
0610040B10Rik	67	53	44	56	63	98
1190002N15Rik	3291	2301	2179	3146	1907	3035
Aff4	5751	3627	3145	4898	3115	4603
Gdap1l1	4	0	1	2	1	7
Ggt5	393	210	198	339	205	450
Men1	756	516	367	743	447	789
Plxna2	4302	3031	2413	3488	2164	4626
Smco2	15	6	6	16	9	14
Agt	212	138	95	170	84	183
Edar	20	12	17	16	17	39
Parp9	1313	783	696	1165	693	1555
Arhgap26	5745	3374	2295	4457	3620	5207
Dusp14	17	10	10	14	3	15
Mpst	928	530	373	825	468	901
Pi4ka	4097	2872	2196	3541	2296	3472
Gm16701	12	6	8	4	9	7
Cables1	293	146	116	228	134	335
Cep135	556	384	290	395	305	549
Eqtn	26	6	7	10	5	16
Atp8b3	3	0	1	1	0	1
Dgat1	693	416	353	612	396	761
Hectd2	203	132	122	147	112	196
Ifit1	626	344	215	477	228	862

Zfp114	3	0	1	1	0	1
Sipa1l2	3499	2469	2028	3066	2087	3029
Hhex	210	123	120	150	137	297
Fra10ac1	730	444	470	521	356	841
Gm19589	3	1	0	1	0	1
Jak1	8930	6150	4656	7822	6891	8534
Lrsam1	722	489	402	520	444	656
Naaa	714	448	296	545	324	577
Npm1	4110	2881	2494	3401	2185	4449
Pank1	4013	2410	2381	3120	2222	3740
Ryr2	108083	75481	63383	95705	63310	81337
Rab35	1794	1109	982	1583	1095	1940
Mir1199	5	1	1	4	4	4
Skor1	2	1	2	1	2	7
Ssna1	403	221	193	340	214	452
Kctd3	701	478	441	614	372	700
Ubqln4	3722	2633	2109	3094	2196	3393
Dgat2	16100	9016	6970	10717	7909	13068
l7Rn6	1099	652	681	886	571	1079
Myl12b	1817	1219	1085	1586	1112	2379
1700023F06Rik	1	0	0	1	1	1
4930451C15Rik	1	0	0	1	1	1
4930593A02Rik	1	0	0	1	1	1
Ackr1	22	5	9	9	7	13
Anapc4	2419	1597	1330	2212	1468	2728
Ankrd10	1926	1111	881	1484	1075	2088
Ankrd50	1449	951	809	1413	856	1591
Asb11	6587	4042	3725	5413	3179	6345
Capns1	9592	6712	5061	8595	5955	11316
Cd19	16	9	7	8	6	13
Cttn	2065	1535	1141	1880	1335	2526
Cyp2f2	2	2	0	1	1	0
D930016D06Rik	795	466	400	585	524	882
Ern1	499	335	251	355	320	357
Fbxw7	3878	2737	2042	3169	2291	3260
Fkrp	2123	1292	1029	1733	1015	1779
Glp1r	1	0	0	1	1	1
Gm10789	3	0	0	2	4	0
Gm12238	1	0	0	1	1	1
Gm14137	0	1	0	1	2	0
Gm16712	2	2	0	1	1	0
Gm20139	0	1	0	1	2	0
Gm4894	1	0	0	1	1	1
Gmeb2	666	443	315	539	390	816
Haus4	152	75	75	102	80	218

Hcfc1	3579	2582	1688	3088	1926	2858
Hist1h2bk	1	0	0	1	1	1
Iqcg	21	13	8	14	8	14
Kctd11	194	121	116	220	96	247
Kirrel	782	519	387	787	427	844
Lrch3	2228	1391	1024	1988	1270	2235
Lurap1	57	24	28	39	46	56
Mettl1	209	149	102	133	120	194
Mpz	0	4	7	11	2	11
Muc2	4	0	3	3	1	10
Myoc	243	237	251	290	161	310
Nop10	1231	748	783	914	641	1345
Ntrk1	10	4	5	6	2	7
Olfml1	237	199	137	232	115	232
Orm3	0	4	3	6	2	6
Pax9	1	0	0	1	1	1
Pcdhac2	38	17	25	27	18	27
Pcdhgb7	2629	1758	1398	2449	1398	3284
Pea15a	3653	2274	2111	3342	2060	4454
Phtf2	6663	4633	3725	5779	3863	5454
Pkd2l1	0	1	0	1	2	0
Pon2	2144	1542	1221	2136	1469	2251
Prdx1	10545	6382	6182	7960	5653	10299
Prss43	0	2	0	0	0	7
Rnf157	242	255	182	371	184	342
Slc16a3	54	31	26	34	20	50
Slc46a2	6	2	2	3	1	3
Snhg7	114	70	84	79	62	123
Sox11	5	3	3	4	1	3
Sv2c	6	3	4	4	4	1
Tpd52l1	295	232	199	234	178	333
Trip4	1516	1052	892	1213	889	1498
Trmt12	258	186	174	201	136	300
Txndc9	2155	1587	1435	1802	1271	2413
Vav1	104	64	48	74	37	98
Zfp445	7138	4845	5101	5173	3951	8977
Zfp618	11	7	2	9	7	14
5-Sep	157	126	108	204	88	232
Bend3	480	329	335	382	302	483
Pla2g4e	406	315	196	382	280	457
Slc25a40	711	471	412	515	404	719
Yme1l1	5799	4074	3554	4730	3453	5720
Cd69	2	6	1	7	3	6
Slbp	478	283	260	469	269	512
Ccdc170	16	8	7	5	9	11

Chst14	311	176	134	294	144	337
Cpd	6877	4507	3532	6147	4294	7374
Dst	36642	27314	22462	28701	24609	31994
F830002L21Rik	3	5	2	3	2	2
Gen1	23	15	17	24	17	36
Gzma	8	6	3	8	6	12
Mag	1	2	3	3	3	5
Mcu	1039	684	610	842	591	981
Slc7a6	723	381	450	481	330	793
Trim3	1190	736	545	997	662	1290
Trmt1	1033	645	511	783	513	956
Il5	6	1	2	2	1	3
Pcdhgb6	2683	1798	1423	2530	1412	3336
1700001O22Rik	2	2	0	1	2	5
Gm19466	1	1	0	0	1	4
Tnfrsf17	5	0	5	2	3	1
Nktr	3936	2631	2159	2800	2211	4025
Rgs9bp	10	3	4	4	2	7
Srp19	1463	1095	1046	1130	896	1796
4932441J04Rik	33	13	12	24	9	16
Eif2ak1	1826	1425	1189	1732	1119	1764
Gli2	137	113	56	145	41	100
Gm10516	239	174	158	185	144	249
Ifitm2	1597	1071	758	1421	840	1919
Jph2	20703	12610	10860	18094	10070	16047
Mir7677	2	0	1	6	0	1
Msi2	9444	7671	6205	8107	6558	8989
Pbx4	1	1	0	1	0	4
Prss57	7	7	4	4	5	5
Rab8b	920	611	516	834	538	1124
Rnf4	1642	1127	967	1368	823	1776
2700038G22Rik	28	33	26	21	17	45
Ercc6	728	480	358	499	370	731
Fbxo41	0	0	1	1	2	0
Fem1c	851	596	418	744	449	667
Galns	485	340	288	445	130	575
Gm14207	29	14	21	23	26	39
Gm9895	13	7	5	13	10	13
Gpr174	22	8	9	12	6	15
Nxph4	17	9	9	19	14	16
P2rx7	298	215	162	276	186	380
Pck1	8	19	13	12	11	11
Ppp1r12c	2800	1764	1436	2388	1694	3031
Prune	1581	1120	892	1300	926	1493
Snord88a	0	0	1	1	2	0

Tatdn3	478	361	259	345	255	529
Atf3	264	206	164	355	83	161
Osbpl2	2525	1824	1583	2208	1548	2395
Mir7231	8	2	5	6	2	3
Rcc1	359	187	152	274	128	285
Tmco3	1327	824	709	1164	667	1079
Rbck1	1606	867	705	1272	841	1657
Ccdc146	167	140	107	112	103	198
Rnf180	5	8	3	9	4	13
Nt5c1a	320	264	154	309	201	114
Padi4	9	6	10	8	13	17
Brd7	4247	3178	3163	3636	2887	4471
Mapk14	3841	2309	2138	3128	1800	3762
Muc15	0	3	1	2	0	0
Rpl35	618	811	662	660	596	897
Arl9	0	2	1	0	3	4
Mapkbp1	694	460	386	670	444	740
Tbce	1163	857	720	883	726	1237
Adssl1	5411	3543	3033	4838	3557	5924
Ccpg1	6481	4846	3954	5629	3897	6239
Gpank1	232	158	129	220	154	268
Grik2	59	28	16	26	19	46
Lrfn1	3	5	5	10	1	13
Sh3bgrl3	609	363	264	505	310	701
Arhgef9	1248	980	836	1174	734	1328
Mrgbp	283	156	153	238	191	290
Ing2	252	169	145	176	190	351
Trnt1	1383	1037	856	1194	834	1398
Immp2l	478	290	301	366	219	528
Ubald1	607	381	269	533	376	587
1500012F01Rik	224	125	117	187	113	300
2310014L17Rik	6	2	3	5	3	10
4933412E12Rik	93	58	50	83	68	104
5730403I07Rik	1	0	0	0	1	2
8430426J06Rik	8	6	6	4	12	15
A330069E16Rik	36	21	26	23	19	35
A730020E08Rik	7	0	2	6	3	6
Abca17	1	0	0	0	1	2
Abcb6	1154	679	644	849	656	1016
Abhd15	109	53	52	68	57	71
Acbd4	585	385	307	505	348	707
Adck2	487	272	233	369	282	556
Ambra1	1490	1066	804	1356	966	1690
Atp2c1	3215	2090	2044	2951	2168	3712
Babam1	1739	1191	884	1513	1040	2011

Bex1	45	16	16	21	24	15
Btnl2	4	2	5	3	2	3
C1ql2	3	0	1	0	1	1
Cacna1b	21	6	11	16	12	26
Cadps2	95	50	34	81	48	100
Cdkn2b	5	8	6	13	6	11
Cflar	2805	1870	1536	2291	1572	2545
Chrm2	1753	1799	1130	2015	1249	1202
Col4a3	222	158	174	191	149	418
Cym	4	0	0	2	1	5
Ddx3y	0	0	3440	0	3377	6250
Dzip1	154	129	106	125	93	175
Efhd2	1196	711	674	1213	686	1212
Fam89b	518	301	246	462	277	558
Fastkd5	581	434	351	562	318	514
Fbxl6	400	198	186	286	159	335
Fbxo16	10	13	8	15	16	14
Fhod3	20305	16201	12346	18582	12242	19111
G630025P09Rik	9	8	4	11	10	10
Gigyf1	2247	1284	988	1580	1351	2412
Gm6525	1	0	0	0	1	2
Gsto1	6181	3706	3815	4232	2729	7498
Hdac7	2532	1442	941	2118	1302	2376
Ifit3	669	324	256	459	225	1012
Isg20	129	142	84	96	88	167
Itgb1bp2	8228	5828	4610	7456	5229	9950
Itih4	73	49	39	114	45	56
Itih5	1308	781	585	1168	416	1119
Kcnt2	197	144	100	185	121	250
Ldlr	227	142	139	194	165	272
Lrrc17	218	166	87	162	93	207
Maz	1820	978	892	1558	1010	1780
Mir6374	0	1	0	2	1	0
Mir6911	3	1	0	0	1	1
Mmp9	11	16	7	14	7	7
Naca	22919	20228	10580	20202	14581	15540
Nlrc4	19	10	18	18	17	33
Nxpe3	99	56	51	91	49	132
Patl2	0	1	0	2	1	0
Pcdhga1	2716	1801	1420	2496	1424	3388
Per2	2593	1652	1199	2125	1538	2773
Pf4	222	157	142	191	109	233
Phc1	613	422	323	554	424	648
Pisd-ps2	158	72	60	100	83	186
Plb1	82	54	28	54	30	69

Plcl2	780	574	454	677	577	928
Polr2l	211	108	103	151	84	180
Ppcdc	267	163	172	238	165	361
Ptplb	236	152	132	228	168	240
Rnf181	1736	1018	975	1564	984	1934
Skil	1842	1135	965	1541	934	1579
Slc25a14	114	68	66	113	71	132
Slc27a4	1017	655	516	917	452	872
Snord111	1	0	0	0	1	2
Snph	0	1	0	2	1	0
Srd5a3	305	155	141	228	171	338
Taf1c	211	101	80	169	101	218
Tbc1d16	3963	2330	1960	3268	2091	2755
Tfec	16	15	9	18	8	7
Tmem143	3728	2179	1928	2989	1981	2963
Tmem161b	813	523	439	615	429	786
Ttll4	530	372	279	417	276	517
Tyw1	380	217	192	302	242	413
Ube2z	2535	1571	1285	2348	1463	2583
Ulk2	5849	3861	3119	4918	3140	5038
Upk1a	29	22	8	15	6	31
Yipf5	1109	757	621	917	560	1137
Zfp526	294	229	156	278	156	239
Zfp654	1478	986	794	1157	791	1442
Mir686	548	298	293	488	295	614
Dnase1l3	27	5	2	24	5	21
Srpk1	1125	774	624	902	657	1035
Csk	874	633	502	886	496	1101
Dsel	520	302	252	476	266	577
Fut11	516	317	266	464	266	640
Grin3a	8	13	4	28	4	10
Itpkb	4345	2376	1919	3592	2409	4115
Ppp1r32	10	5	12	2	6	14
Snx11	585	318	264	487	347	551
1110058L19Rik	1079	582	683	803	487	1170
2900060B14Rik	1	0	0	2	0	1
A330048O09Rik	0	1	2	1	1	5
Aak1	5584	4109	3400	4595	3582	5170
Alx4	22	10	15	15	11	15
Anxa7	5747	4208	3731	5286	2727	6605
Cyp2c44	1	2	2	1	0	2
Dcp2	1467	950	810	1268	743	1343
Dsc1	1	0	0	2	0	1
Eng	9987	5727	4427	8050	5320	10671
Hnrnpa0	3281	1825	1654	2826	1771	3426

Nek4	241	170	159	195	157	212
Osbpl1a	4774	2839	2759	3719	2431	4682
Otx2	1	0	0	2	0	1
Rngtt	805	616	517	734	452	854
Slc1a6	1	0	0	2	0	1
Tmem232	18	10	5	12	6	9
Zbtb45	258	142	103	211	143	259
Mboat1	58	48	36	57	31	46
Stx8	607	459	431	581	448	842
Fam178a	2188	1437	1211	1676	1299	1966
Hn1	1781	933	960	1513	852	2207
Peli3	47	39	27	28	30	47
Rmdn1	2037	1319	1311	1570	1158	2180
Rrp8	509	364	320	442	290	498
Slc25a36	1558	890	872	1174	870	1333
Sp1	3315	2166	1768	3008	2068	3473
Vps13a	3460	2277	1939	2919	1956	3046
Zfp503	95	61	40	92	62	93
Lipg	1	1	3	0	0	3
1700066B17Rik	11	6	7	7	0	13
2410127L17Rik	472	349	320	392	294	490
3830403N18Rik	63	46	37	50	33	57
AA543186	7	4	2	2	7	12
Adamts12	45	32	38	63	19	90
Adc	28	20	22	37	17	44
Arhgap39	332	194	138	237	160	248
Arhgdig	12	5	9	2	5	14
Asph	12368	8258	7780	10259	7465	11400
Atp5d	15238	9259	7974	13959	8229	17413
Aurkb	29	11	11	16	5	64
Bbs10	190	129	112	134	118	170
BC005764	16	8	5	12	5	6
Brinp1	8	5	3	1	0	11
Bspry	2	3	3	2	3	0
Btc	83	90	103	97	72	105
Camsap1	1741	1190	986	1553	1051	1294
Ccnk	1909	1384	1094	1677	1092	1778
Ccr3	0	1	0	1	1	1
Cd180	57	40	21	48	15	46
Cd1d1	108	87	74	102	75	183
Ces1a	6	1	2	1	1	4
Cirh1a	1012	668	595	841	580	931
Clcnkb	91	57	44	65	72	107
Cndp2	1206	730	719	1085	670	1523
Coa5	9396	6329	6241	8153	5891	8383

Coro7	560	394	230	496	306	653
Cst3	10933	7718	7071	11313	6740	13954
Cyb5r2	5	3	0	3	0	2
Cyp4v3	304	202	223	302	194	447
Dab2	2289	1786	1189	1781	1289	2403
Dhfr	207	153	140	145	122	241
Dnajc2	1519	1291	1018	1200	1016	1807
Dph7	211	128	114	182	121	268
Dusp8	1702	1076	1027	1728	789	1383
Efna1	960	646	503	920	569	1037
Enpp6	77	79	48	99	45	141
Ercc8	173	103	87	130	78	150
Exosc3	464	320	272	365	252	474
Fam174b	14160	8456	7709	10779	7461	12795
Fdxacb1	296	179	156	218	158	253
Fgf12	135	108	121	112	125	89
Fmo6	4	1	2	6	4	2
G3bp1	3904	2427	2152	3354	1948	3488
Gabra4	3	1	0	0	0	2
Gdf9	21	17	12	12	14	18
Gdpd2	1	0	0	1	0	2
Gjb4	0	0	1	2	1	0
Glul	28806	17255	14276	24155	17576	30444
Gm14446	277	195	142	213	159	235
Gm2381	0	1	0	1	1	1
Gm6249	56	16	23	27	28	77
Id2	580	464	281	432	320	594
Ift20	771	542	465	638	419	804
Iigp1	5466	2585	2166	3591	2890	5596
Inpp5b	806	475	384	653	460	882
Klra8	2	1	2	3	2	4
Ksr2	0	1	0	1	1	1
Lamtor5	1036	682	658	970	654	1277
Map3k4	2784	1869	1690	2321	1727	2423
Mif	597	288	267	387	266	489
Mir1968	1	0	0	1	0	2
Mir6945	2	2	1	3	2	4
Morc4	259	207	131	221	147	221
Mrgprb2	0	2	2	3	3	2
Mtmr12	682	452	390	646	384	854
Ndufb7	7006	4313	3919	6315	3842	8519
Nucks1	3802	2420	2401	3066	2107	3895
Oprd1	38	34	27	39	24	28
Pacrg	49	33	42	30	35	51
Papolg	832	567	489	665	489	788

Pcdhga10	2687	1782	1435	2487	1419	3352
Phldb2	1882	1521	1351	1803	1419	2715
Pim1	524	323	216	370	265	403
Pla2g2c	29	13	8	23	11	36
Plce1	2071	1424	1369	1781	1316	1914
Pmf1	217	186	147	267	140	303
Polr1a	2960	2180	1542	2575	1758	2395
Polr3b	838	577	486	670	516	740
Ppard	1264	898	611	1075	677	1050
Pqlc3	177	140	110	218	97	245
Prelid2	207	110	76	106	161	215
Psma4	3158	2256	2171	2476	2000	3531
Rai14	509	334	225	482	302	501
Rapgef4	1667	1212	1159	1517	1375	2010
Rasgrp2	1299	725	512	1066	692	1228
Rasl11a	28	20	24	34	17	52
Rassf1	697	370	318	551	368	750
Relb	168	130	117	162	135	231
Rexo4	617	397	309	463	316	568
Rnf128	5275	3481	3362	4408	3035	5211
Rnf25	681	415	373	597	389	807
Rps19	1318	852	608	1039	712	1643
Scg3	16	20	9	15	8	17
Serbp1	14124	10856	9112	12067	8647	14858
Sgip1	62	35	46	49	52	90
Slamf6	9	5	4	3	5	6
Slc25a22	1669	836	685	1103	616	1513
Smtnl1	5	5	2	3	2	4
Sp3os	155	104	106	156	102	212
Srbd1	470	334	300	405	270	465
Stmn3	6	2	0	6	1	7
Surf2	690	569	391	543	378	804
Tet3	1788	1026	868	1410	1114	1772
Thsd7a	690	496	275	586	491	655
Tkt	1162	643	525	897	613	1277
Tmem248	1908	1287	1133	1701	990	1856
Tmem8c	1	0	0	1	0	2
Tmprss9	3	5	2	2	3	2
Tnfrsf10b	138	102	81	118	98	196
Tnr	2	6	1	2	4	11
Trim9	0	1	0	1	1	1
Ube2w	1187	725	649	963	624	1054
Utrn	5991	4525	3463	5665	4349	6788
Vrk3	793	445	375	634	435	871
Wbp11	2642	1906	1601	2371	1478	2619

Wnt2	18	8	3	7	4	12
Xkrx	124	76	55	71	53	123
Xpo5	1199	853	669	1018	739	970
Yars2	1160	694	683	931	594	1125
Ythdc1	3149	2256	1905	2511	1861	3336
Zbtb37	141	81	91	141	101	152
Zc3h7b	5603	3635	3050	4793	3081	4580
Zfp697	502	635	488	693	519	961
Zfp746	587	381	268	545	340	602
Zfp866	1207	791	643	991	667	1036
Zfp951	43	38	35	34	41	89
Zgpat	1131	687	579	842	589	1036
Cyp20a1	380	281	228	333	199	386
Anapc1	4261	2663	2123	3455	2148	3667
Arl11	37	18	15	16	13	34
Galnt12	12	8	7	6	3	14
Gm3230	41	26	24	38	25	60
Gpc3	174	131	89	224	95	188
Ly6k	3	1	2	3	1	7
Mir5625	2	2	1	2	0	1
Rassf4	883	481	493	687	521	1052
Rfng	564	355	290	421	282	534
Sntb2	774	453	333	567	438	871
Naa40	1277	735	740	957	703	1171
Pqbp1	671	465	417	662	437	795
Fam124a	339	248	296	367	267	510
4732491K20Rik	208	159	137	168	139	193
4930579G24Rik	95	42	48	67	54	120
Bmp3	13	3	7	6	7	4
C030016D13Rik	23	16	14	19	10	19
C130060C02Rik	3	1	1	1	1	1
Ccdc66	1011	779	661	837	660	1055
Dock5	823	552	494	671	533	648
Fcna	84	43	47	40	37	89
Fgf1	10696	9473	7195	10979	6624	10911
Hif1an	3816	2587	2145	3245	2425	2642
Hpca	3	2	2	2	5	5
Iffo1	632	398	279	512	350	726
Lyar	1241	912	856	866	821	1438
Notch2	1578	903	614	1151	683	1252
Otx1	0	3	2	2	0	1
Pcdhga12	2724	1790	1416	2477	1425	3366
Pih1d2	8	13	9	11	5	10
Rab39	11	5	8	6	4	10
Rhpn1	3	1	1	1	1	1

Rnf208	17	9	6	11	5	11
Snord87	3	1	1	1	1	1
Snx29	131	88	69	122	105	129
St8sia1	18	6	11	12	8	9
Sun3	37	18	22	12	18	39
Ttf2	224	177	117	212	177	246
Zfp523	597	390	255	439	360	713
Fgd3	44	20	20	28	15	34
Sema3a	125	93	53	101	109	125
Zc3hav1	862	529	378	769	438	933
Zfp760	500	328	242	289	282	493
C1rb	0	1	0	3	0	0
Gm1965	0	1	0	3	0	0
Rps26	2641	2241	1499	2032	1683	2893
Samd15	13	6	3	3	5	9
Tomm20l	22	10	16	2	15	21
1810013A23Rik	0	0	1	1	1	1
6720489N17Rik	50	74	53	75	65	108
Arid5b	1165	938	747	1079	954	1410
D1Ertd622e	161	95	99	153	104	189
Dlg1	5201	3435	2932	4159	3164	4336
E230008N13Rik	0	0	1	1	1	1
Ehf	1	3	1	0	0	3
Exoc2	1147	754	658	986	598	1091
Galnt11	1267	787	692	1014	658	1180
Gata5	9	8	5	21	6	7
Gm14378	96	50	29	65	52	106
Heatr5a	2311	1430	1126	1943	1144	1850
Kctd2	1059	523	588	747	473	1019
Olfr520	0	0	1	1	1	1
Rad17	1130	856	814	1026	686	1262
Rexo2	1639	1225	1128	1300	1030	1901
Sbsn	55	46	30	50	38	88
Tigit	3	1	1	1	0	2
Trip12	10588	7526	6735	9365	6309	10108
Acad11	9166	6045	5573	7020	5813	8237
Ago1	1403	900	769	1256	913	1460
Arntl	139	84	151	131	81	167
Arpc1a	2053	1219	1138	1897	1125	2306
Ccl21a	311	204	160	234	164	283
Cln5	1639	1140	1052	1378	1003	1629
Coprs	267	152	161	274	143	314
Crebrf	3898	2425	2075	2929	2628	4426
Fam209	3	1	2	0	6	5
Gm11627	48	39	47	38	35	56

Gm1987	311	204	160	234	164	283
Hamp	28	27	15	12	27	17
Lum	5171	3643	2024	4392	1703	4876
Nfkbiz	270	158	126	193	154	347
Spata24	103	56	58	74	62	151
Tns4	22	8	7	8	6	17
Tsnax	3560	2122	1925	2834	2215	3999
Zbtb21	541	353	273	428	273	486
Zfp575	3	0	2	3	3	3
Brsk1	63	45	33	69	38	77
Camk2b	413	336	301	482	360	455
Hdgfrp2	1828	1210	937	1533	1120	2088
Nedd8	1524	1065	834	1391	878	1894
Rfx5	479	318	231	384	307	551
Nomo1	6777	4211	3637	5428	3875	5303
Atr	1071	655	664	871	589	1033
Ccni	6683	4333	3830	6473	4148	7232
Crlf2	116	58	58	98	46	172
Csmd2	0	2	3	1	1	1
Fnbp1l	2315	1568	1157	2019	1341	2685
Lin28b	14	8	2	11	7	17
Mir7063	0	1	0	0	1	2
Nppc	1	1	3	0	1	2
Prmt10	751	490	494	785	487	850
Sohlh2	0	1	0	0	1	2
Trp53bp2	827	640	455	767	591	950
Txlnb	42942	36834	28647	35376	32003	39771
Zfp647	72	43	42	59	54	89
Cep89	630	387	333	446	357	557
Rsl1	102	139	100	164	100	209
Zfp873	230	91	90	133	83	174
Ercc6l	38	22	26	31	25	63
Cul3	8703	6644	5854	7545	5820	8475
Ccnt2	2562	1759	1409	1926	1429	2642
Bard1	66	41	30	23	38	62
C630043F03Rik	73	50	51	71	48	113
Capn15	411	201	148	262	174	290
Ccdc39	20	23	15	21	9	24
Cyp17a1	1	0	0	0	0	3
Dppa3	1	0	0	0	0	3
Nfkbil1	184	121	95	158	108	240
Nfyc	1003	583	477	898	578	952
Pias1	1617	1064	833	1541	975	1638
Smim14	2680	1811	1412	2552	1576	2914
Fam57b	407	252	178	315	188	576

Mmp17	113	57	55	97	55	139
Acss1	17298	10303	9164	13935	9175	13866
Bcr	899	574	415	852	507	888
Ccdc115	441	371	290	395	262	495
Cd86	140	89	83	86	65	161
Chchd3	16373	11850	10861	13284	10399	16743
Cldn11	12	6	6	7	4	9
Fgfrl1	580	371	284	502	391	571
Gm15441	30	20	21	21	19	25
Gm16740	23	18	20	29	18	38
Immt	30377	22595	18653	27075	17577	29148
Itpripl2	2624	1578	1390	2465	1244	3243
Myh4	5	4	2	1	0	7
Ndst2	650	383	272	548	322	715
Npdc1	570	310	272	463	281	678
Pcbp4	1866	1072	936	1448	828	1765
Pofut2	1789	1155	975	1495	943	1647
Ptgr1	131	98	73	135	76	179
Purb	5414	3405	3232	5077	3372	6077
Slain1	4	6	5	8	2	1
Smim3	430	291	272	304	245	477
Zcchc14	2557	1543	1286	2217	1480	2658
Zfp369	287	178	151	193	181	217
1700003F12Rik	0	3	2	0	1	2
2310011J03Rik	571	314	296	530	317	578
2310030G06Rik	177	136	110	139	148	250
4930447K03Rik	5	1	3	2	5	8
4933411K16Rik	3	1	1	2	0	1
9130011E15Rik	886	539	436	702	465	703
Acaca	307	283	197	298	179	335
Agbl4	2	1	0	2	1	3
AI314180	11928	7842	7341	9853	7099	10915
Aimp2	769	507	416	627	378	758
Airn	572	401	291	440	410	674
Aldob	162	131	128	127	189	223
Arhgap23	1175	755	454	1099	606	1142
Asic3	14	12	6	15	12	18
AU021063	0	0	1	0	1	2
Bambi-ps1	88	50	40	80	62	80
Card11	9	4	12	6	5	10
Catsper2	276	205	146	179	162	288
Ccdc102a	193	140	100	196	116	230
Ccdc42	0	1	0	2	0	1
Cd79a	5	6	4	7	7	9
Cdipt	1534	849	776	1450	764	1590

Chac2	897	615	554	798	505	848
Col22a1	33	11	15	33	13	35
Crnkl1	1528	1204	900	1361	945	1461
Csnk1e	2880	2018	1520	2374	1834	3460
Ctla2a	766	570	764	669	796	1304
Dhrs7c	6063	3050	2256	4069	2682	4274
Edrf1	957	655	503	761	533	886
Eif1ax	1922	1329	1213	1640	1124	1927
Foxp2	268	184	157	245	211	282
Fyco1	18886	15507	13806	22396	11134	14868
Ggt7	61	23	25	27	17	56
Glipr2	196	88	99	131	62	186
Gm12359	26	12	12	18	10	16
Gm6329	0	0	1	0	1	2
Gpr21	23	10	10	30	10	20
Gtpbp6	304	144	127	240	163	290
Jrk	151	108	58	89	86	120
Ly9	20	24	19	25	7	28
Mchr1	0	1	0	2	0	1
Mir7004	0	0	1	0	1	2
Mir7675	0	0	1	0	1	2
Ncor1	8007	5779	4506	6957	4765	6927
Npy1r	88	64	78	106	57	154
Pcdhb6	6	2	0	3	5	5
Pcmtd1	5509	4164	3497	4710	3604	5206
Plekho2	561	366	334	539	328	685
Proca1	27	17	29	31	25	47
Psme2b	91	69	57	93	77	105
Ptgr2	14771	9909	9323	12377	8946	13546
Rhbdd3	475	226	242	366	263	518
Rmi2	44	25	10	21	8	41
Serinc4	9	2	8	2	2	11
Slc36a1	710	430	347	567	315	644
Snhg1	230	140	137	196	132	320
Snord104	13	1	6	8	3	3
Snord73a	1	1	1	1	0	6
Snrnp25	174	109	81	145	95	221
Soga1	2027	1331	867	1817	1095	2125
St6galnac3	105	82	58	98	64	160
Suds3	3805	2567	2356	3032	2329	3697
Syngr4	0	0	1	0	1	2
Tab2	5235	3913	3069	4519	3357	4463
Tcerg1l	0	0	1	0	1	2
Tmem126b	882	534	634	791	476	879
Tpst1	786	412	436	600	411	1041

Trappc2	298	204	180	239	162	300
Unkl	171	126	116	184	116	225
Uso1	3393	2293	1969	2821	1983	3171
Usp51	0	1	0	2	0	1
Zfp600	0	1	0	2	0	1
Zfp827	1458	1097	910	1040	977	1555
Cep78	236	179	141	167	136	263
Gm16894	7	3	2	3	2	4
Rab3gap1	3190	2271	1831	2592	1954	3014
Snx3	4797	3406	2856	3998	2809	4743
Tcea2	169	85	102	134	119	191
1700028E10Rik	3	3	5	4	8	6
Ccdc23	320	184	158	261	191	352
Klra13-ps	2	2	1	4	1	4
Zc3h7a	2335	1570	1220	1839	1510	2773
Zfp953	136	102	101	138	109	186
Morn4	44	22	23	41	24	53
Slc38a4	505	272	205	394	368	376
Zfp420	262	171	180	205	166	245
Zfp90	250	191	152	221	184	333
Hgfac	7	2	0	3	3	9
Shox2	23	13	16	19	16	7
Ccl19	4	6	9	8	1	7
Mir7010	2	7	2	12	2	6
Nedd9	1123	744	900	1100	819	1572
Recql	542	347	312	422	291	525
Pink1	19568	10616	9535	15902	11699	19210
Ece2	167	108	113	132	74	192
Wdr83os	589	446	376	552	291	648
Xlr	41	30	41	42	18	50
Phyhd1	2314	1303	1440	1712	1581	2871
Slc19a3	20	15	20	23	20	34
Msrb3	7263	5224	5032	7232	4409	6424
Cic	2606	1549	1063	2140	1424	2548
Klrb1a	6	1	0	1	0	3
1810043G02Rik	386	218	208	316	243	425
6030443J06Rik	24	10	21	12	14	23
Cad	673	346	309	505	293	529
Cct4	6339	4182	3782	5377	3202	6523
Gdf3	1	1	1	1	2	3
Nr6a1	152	89	78	91	78	143
Tmed8	968	590	550	820	623	1066
Tanc2	544	432	445	569	410	844
Acsl6	728	501	478	583	335	905
Lcat	69	49	46	47	33	82

Slc16a12	205	155	142	184	134	343
1700020L24Rik	0	0	1	2	0	1
Abhd12	2120	1362	1233	1644	1177	2120
Accsl	0	0	1	2	0	1
Bid	47	33	28	28	20	56
Casc1	38	21	37	36	38	58
Chd7	1995	1388	1055	1404	1228	1884
Cox15	2064	1356	1266	1987	1108	1726
Dnah6	11	6	16	7	2	21
Gm13212	148	149	99	186	144	171
Gpbar1	0	0	1	2	0	1
Gtf3c3	619	414	365	474	374	579
Inpp1	532	355	285	466	317	650
Kremen1	6495	3825	3355	5440	3265	5130
Lgr4	2081	1426	1261	1869	1250	1758
Mgat3	228	164	168	203	141	226
Mov10	372	257	177	302	262	406
Pkig	5151	3193	2615	4641	2846	5630
Sh3bp1	127	82	58	90	55	117
Thoc1	1117	760	707	858	590	1306
Zfp846	235	170	159	220	168	321
Psd3	2954	2095	1567	2576	1664	2578
Arhgap19	242	68	124	118	64	233
Bag5	1030	649	589	991	577	1178
Irg1	1	2	3	1	0	3
Kpna2	160	115	70	126	76	135
Kti12	654	427	358	497	373	599
Psen2	470	299	226	422	278	495
Syn2	144	67	114	124	83	243
Tmem255a	87	49	38	62	42	129
2210015D19Rik	697	525	448	598	397	767
1700031A10Rik	3	0	0	0	3	3
Armc10	410	351	290	420	309	596
C3ar1	401	258	148	317	120	365
Gak	1477	953	797	1376	851	1627
Hnrnph3	2168	1661	1542	1818	1357	2555
Rtp1	0	1	0	1	0	2
Spata19	0	1	0	1	0	2
Tor1a	341	226	182	293	202	433
Brpf1	1281	921	606	1203	779	1357
C920025E04Rik	11	2	0	3	0	5
Ccdc86	348	254	193	261	213	325
Cnot1	6333	4535	3296	5526	3699	4883
Cul9	1328	880	592	1044	686	1096
D1Pas1	0	1	1	1	0	4

Eif4g2	63774	41914	36414	54926	34978	53035
Etnk1	3798	2420	2099	2944	2061	3680
Fmr1	1365	893	763	1077	830	1784
Foxd2os	33	40	17	31	30	65
Ftcd	2	3	4	3	3	10
Gla	166	116	98	167	85	248
Golm1	631	481	375	611	491	681
Hexdc	288	148	117	195	159	322
Hmgxb3	1280	843	642	1054	668	1121
Hsd17b14	1	1	1	1	3	2
Kcnh8	0	1	1	1	0	4
Llgl1	831	459	320	645	431	834
Mmaa	1622	1026	1017	1331	930	1581
Ncoa3	3348	2373	1838	3086	2096	3856
Nrip3	6	2	8	7	9	9
Psmc4	2753	1746	1560	2430	1613	3245
Rspo1	15	11	7	22	8	17
Scgb3a1	3	7	1	5	6	7
Snord45b	10	1	4	3	1	7
Stxbp1	841	463	341	724	440	778
Tdrd3	1436	1074	881	1311	830	1430
Thumpd3	835	611	529	693	536	811
Zfp157	513	367	303	387	289	552
Zfp58	362	205	216	243	190	365
Cops3	3716	2676	2312	3066	2247	3831
Mtus2	6964	4404	3345	4925	4467	4358
Nxf2	4	0	3	2	2	9
Tmem221	7	2	3	4	1	4
Trim59	132	90	55	73	34	166
Xylb	22	15	11	17	10	16
Zfp664	3000	1924	1652	2566	1643	2592
Cutc	645	329	338	471	370	758
Enah	9732	6922	6849	8404	6678	8831
A930017M01Rik	8	5	4	8	4	14
Ankib1	1246	809	628	997	654	1107
Atp2b1	4624	3081	2774	3758	2749	4356
BC051142	5	6	3	2	2	7
Fcrls	202	159	100	178	73	217
Ngp	8	1	0	5	5	4
Rad23b	5482	4134	3510	4935	3425	5263
Rint1	738	504	413	635	359	739
9430008C03Rik	115	98	79	121	83	176
Fn3krp	427	245	215	356	243	477
Ltc4s	41	28	20	26	28	68
Dgkb	82	40	33	77	43	76

Tmem47	1723	1060	1035	1826	1019	1744
BC052688	1	2	0	2	1	3
C78339	535	366	271	487	282	388
Cacna1g	758	466	325	788	516	517
Cdk19	2180	1255	1030	1883	1257	2075
Eda2r	287	166	131	205	167	347
Fxyd1	7380	5125	4005	6480	4703	8566
H2-K2	153	115	52	98	112	194
Irgq	1362	950	835	1428	697	1097
Ly86	90	70	55	90	50	150
Mir1940	3	1	0	2	0	0
Mpi	4052	2720	2511	4344	2219	4957
Pag1	429	218	159	301	171	310
Ppia	19	17	14	13	14	18
Prpf40b	1030	762	517	710	612	1040
Psme4	21739	14696	13688	19652	12631	18956
Ctnna3	1456	1098	831	1339	915	1144
Homer1	3545	2523	2223	3237	2201	3069
Odf3b	24	20	14	16	22	43
Prkab2	2635	1844	1630	2360	1625	2295
Cstf2	972	668	598	760	502	1123
Grina	3159	1897	1424	2610	1729	3264
Slitrk6	127	84	50	83	51	120
Snord35a	9	1	1	3	6	8
Gm5069	190	99	103	166	119	197
Uchl3	993	752	706	775	669	1125
Pdpk1	3040	1998	1749	2775	1695	2504
Xpnpep3	334	285	214	334	226	256
Cisd2	5200	3637	2865	4338	2688	5300
Farp1	650	417	288	634	328	685
Gm20199	8	5	2	13	4	6
Frmpd1	6	4	3	3	9	8
Glb1l2	10	7	8	6	7	8
Gm16523	8	0	1	3	1	1
Gpr55	0	0	1	1	0	2
Nfkb2	824	568	400	798	550	764
Rnf223	7	4	3	3	3	5
Tas1r3	21	6	4	16	12	14
Tnxb	3944	2460	1890	3263	1530	3882
Wipi2	2108	1349	1175	1835	1335	2316
Ireb2	3084	1974	1730	2578	1620	2950
Eif2s3x	582	325	200	513	186	346
Pde12	534	375	347	471	324	494
Pus3	324	253	208	274	202	328
Pknox2	484	286	214	395	219	359

Ptpn6	164	124	67	160	97	187
Arv1	118	81	57	84	59	108
Catsper3	6	3	6	5	2	5
Ctns	720	420	373	543	366	638
Fnip2	1151	775	866	819	942	914
Gm11710	0	2	2	4	3	1
Gm11711	0	2	2	4	3	1
Gm13375	933	767	524	824	627	1287
H2-DMb1	97	49	40	111	24	127
Igfbp5	5891	4373	3495	6409	4584	5404
Med13	4691	3207	2518	3824	2660	4257
Mir6935	301	127	152	149	124	298
Mir7024	6	0	1	2	1	1
Mtmr9	668	492	402	614	391	599
Rpl11	76	43	26	64	34	91
Snx13	5991	4127	3557	5301	3451	5364
Tyms	81	42	45	57	52	110
Vav2	483	324	260	385	286	397
Zfand1	604	425	369	478	357	616
Zfp566	90	56	31	64	53	109
Gtf2e2	591	404	362	577	372	707
Golga3	3615	2811	2264	3273	2318	3404
Smtnl2	830	452	313	773	403	719
2410131K14Rik	607	401	354	616	307	434
Trmt44	95	74	52	104	52	134
Lgi2	74	42	51	46	35	83
Ccdc12	882	514	480	764	431	1148
Cenph	6	2	3	5	2	11
Zfp41	125	53	66	99	75	132
Srprb	1473	846	718	1231	658	1227
Rgs20	1	4	0	1	0	2
6430710C18Rik	40	24	38	26	53	61
Asrgl1	2299	1640	1262	1956	1258	2240
Hcfc2	859	664	547	753	545	861
Plcxd2	29	6	7	19	10	29
Poldip3	1175	669	541	996	616	1213
Rac1	9114	6037	5069	8475	5534	10231
Rps6ka5	709	410	284	614	412	617
Ankrd23	4638	2102	4544	3615	6539	4150
Mgst2	0	1	2	0	2	5
Prpf8	9315	6641	4866	8486	5091	7511
Uckl1os	274	146	85	237	104	297
1700012D14Rik	15	6	11	8	5	15
1810010D01Rik	1	2	2	2	4	3
2310009A05Rik	266	201	189	201	175	297

9430091E24Rik	104	44	48	62	44	79
A930005H10Rik	401	118	106	171	138	240
Ap1s2	2440	1668	1481	2346	1533	2837
Asb18	147	92	107	124	96	115
Bai1	5	3	7	3	3	6
Cx3cr1	63	39	36	80	22	91
Ddx59	427	308	291	330	279	446
Dhx15	3926	2756	2280	3315	2239	3840
Fes	502	262	217	402	238	557
Fignl1	33	18	10	22	13	51
Fzd3	204	122	99	162	95	162
Gm12295	541	262	166	313	305	529
Gm608	2527	1854	1369	2095	1549	2233
Gmps	2309	1653	1405	2141	1274	2243
Kcnq1ot1	793	595	422	525	508	795
Med16	1498	822	635	1246	820	1372
Mir1949	2	0	3	0	1	2
Mir707	5	5	3	9	5	6
Rps13	23	15	12	9	11	25
Smpd3	111	78	55	54	58	124
Snx4	1696	1329	1031	1473	1079	1686
Tmem127	2312	1472	1390	2099	1456	2634
Zc3h12a	237	162	149	225	116	218
Zmat5	424	298	244	464	233	505
Tmem95	2	3	0	2	0	1
Mki67	403	260	164	320	98	824
Pdgfrl	113	95	56	128	34	94
Pnma1	2	2	1	1	3	5
Abcc8	2351	1354	934	1880	1020	1692
Cmtr2	200	110	109	146	107	157
Hnmt	653	548	419	632	527	811
Fancf	73	43	42	45	43	62
Gpkow	1625	1115	796	1361	813	1501
AI987944	679	553	430	578	428	731
B3gnt1	2210	1352	1095	2016	1127	2461
C130046K22Rik	356	201	161	251	177	282
Cd164	10784	7362	6402	9352	6069	10138
Dnajb6	667	366	349	536	384	742
Larp7	1546	1105	1042	1234	968	1707
Lmtk2	2549	1760	1313	2045	1560	2018
Nup50	974	722	603	898	537	998
Psors1c2	3	2	1	1	1	2
Rdh1	9	3	1	1	3	5
Samd4	4682	3552	3241	3940	3385	4321
Ube3b	7065	4676	3764	6001	3990	5637

Zfp111	299	179	165	240	199	343
Zfp472	161	109	115	173	99	226
Zfp593	74	54	38	70	39	112
1700020I14Rik	4354	3256	2913	4063	2931	3683
2410076I21Rik	10	3	9	8	4	6
3010026O09Rik	50	45	28	50	28	93
Calr4	1	1	3	2	1	7
Cd84	108	77	61	87	63	178
Dok5	19	20	16	28	4	19
Dpys	0	0	1	0	0	3
Fam120b	1886	1344	951	1543	1020	1791
Il12rb2	3	0	2	2	0	1
Mir1898	13	21	13	12	24	33
Mybl1	82	51	44	67	49	114
Nt5m	578	353	368	494	279	588
Nup160	714	457	354	525	380	653
Pipox	48	33	34	41	25	45
R3hcc1	654	464	342	591	412	754
Rbm10	872	587	429	684	473	770
Rxrg	2415	1539	1420	2144	1190	2347
Slc25a32	683	490	403	500	422	703
Slc47a2	2	1	3	2	0	2
Slc5a7	0	0	1	0	0	3
Smc3	2739	2240	1640	2383	1753	2796
Sms	1903	1269	1045	1544	1087	1732
Uspl1	1190	787	683	924	674	1178
2010310C07Rik	2	3	1	2	0	2
9330175M20Rik	3	1	2	1	1	2
Adamts19	86	45	24	50	27	66
Aff3	190	139	92	174	118	231
Akap9	4015	3029	2431	3338	2685	3649
Arhgdib	757	673	474	659	395	987
B230217C12Rik	28	19	18	28	7	26
B3gnt7	2	0	1	0	3	3
Bcas2	1589	1142	947	1320	924	1645
Cacna2d1	16544	11923	9345	14301	9753	14536
Cltc	10658	7349	6407	9624	5881	9976
Csnk1d	2927	1851	1534	2714	1741	2951
Cyb561	628	333	293	572	249	770
Cyp7b1	42	29	26	38	29	62
Dpy19l2	1	1	2	3	0	5
Ehd4	28429	20815	14692	24288	14185	27763
Fam122b	399	269	198	401	237	409
Fars2	2052	1260	1155	1801	1223	2302
Fcgr2b	332	263	242	290	145	460

Gemin8	68	46	31	68	51	63
Gm15760	31	29	23	12	24	40
Gm17066	1112	768	553	834	696	1414
Gm6548	374	212	211	294	196	527
Gng5	413	257	247	363	207	371
H60b	1	2	2	1	2	0
Hist1h3e	3	1	1	2	1	0
Htra3	4606	3202	2161	4678	1632	3844
Ido1	24	12	14	10	19	43
Irf2bp1	782	475	321	740	409	728
Itgb3bp	251	193	158	201	149	269
Iyd	3	2	1	2	0	2
Lims1	11027	7785	6044	9547	6014	10227
Lzts1	47	28	26	43	40	46
Mirlet7d	12	9	11	11	7	32
Mmp19	199	107	100	157	70	180
Mrgpre	83	55	54	114	46	93
Mtap7d3	52	57	30	48	29	58
Mum1	699	448	411	592	460	825
Npepl1	2434	1422	1229	1989	1458	2506
Paqr6	5	6	8	11	8	10
Park7	4440	3027	2737	3989	2861	5526
Pcgf2	251	207	118	215	176	322
Pkhd1l1	213	199	125	127	160	236
Ppil1	1142	713	597	939	808	1115
Ppp2r2c	12	1	5	8	1	4
Prdx4	526	395	353	590	316	695
Rabl2	185	93	75	160	84	196
Rbm47	52	26	14	34	12	37
Rcn3	730	501	376	651	287	742
Sag	13	7	9	10	13	18
Slc7a7	125	72	69	114	81	136
Slc9a3r1	195	134	96	181	122	220
Srcin1	21	25	14	13	18	23
Thap11	808	475	457	767	485	827
Tulp4	1532	1016	818	1251	952	1131
Ube2e2	299	249	165	299	217	360
Ubtd2	335	242	168	281	152	333
Uty	0	0	936	0	963	1467
Zfp13	104	64	54	90	71	116
Zfp213	287	158	148	234	136	218
Grpel2	946	600	681	768	609	931
Klhl22	879	527	434	607	473	794
Nptx1	12	2	4	3	9	15
Phtf1	574	386	307	542	323	680

Slc25a39	3961	2425	2091	2856	2097	3875
Hbp1	4552	2969	2267	3940	2606	5116
Csad	553	342	242	371	335	678
Col1a1	3114	1870	1605	2928	1105	4814
Pcdhga5	2714	1798	1429	2509	1419	3314
Slc11a2	1204	756	621	1070	600	968
Snord98	0	2	1	2	2	2
Dpp3	780	469	423	687	446	878
Gtpbp8	272	197	191	286	197	336
Acbd6	958	569	504	865	513	1061
Atg5	861	581	566	791	477	844
Atg7	660	411	336	591	387	702
Ccdc171	116	93	61	85	69	111
Cdk18	1087	736	543	771	571	1124
Csf2rb	69	60	41	57	29	84
Ech1	31346	15731	16906	21931	20936	33586
Fzd5	431	279	205	368	216	324
Lactb2	1112	1005	805	1020	767	1311
Mycl	14	10	3	6	7	9
Orai3	652	370	297	517	388	650
Oxnad1	2862	2046	2058	2711	1705	3029
Slc39a10	773	615	543	828	556	984
Ttc9	65	36	23	47	29	89
Vps4b	2012	1326	1187	1762	1262	2408
Zfp202	239	133	152	171	131	226
Bcs1l	1275	855	690	1043	695	1203
Klhl24	15933	9884	8929	11440	9572	14113
Gramd1a	1352	761	646	1007	769	1493
Mmd2	1	2	3	2	1	1
Coch	14	9	12	17	6	7
1110007C09Rik	2293	1550	1156	1759	1267	2150
Bbs9	447	327	252	435	328	465
Dnajc12	379	208	171	327	206	377
Slc16a11	13	1	8	3	4	10
Stx5a	1280	788	616	1092	730	1343
Adamts2	900	586	506	893	405	1253
Ifi27	889	394	373	812	335	902
Rnf139	2812	1791	1741	2531	1517	2616
Slc30a4	1327	752	762	1141	765	1504
Zfp184	33	21	19	29	14	25
Fmn1	102	59	79	73	62	101
Wdr83	949	639	569	863	613	1109
Rhot2	7962	5039	4409	6503	4394	7089
Zfp28	295	259	200	270	288	371
Junb	486	265	227	356	213	422

C2cd5	1077	692	563	837	670	1280
Cluh	13988	8775	6898	12037	7137	10596
Ndufaf2	618	450	461	475	364	787
4933430I17Rik	6	6	6	3	7	4
Arhgap10	2907	2071	1615	2483	1694	2700
Asb1	1191	700	639	1071	585	919
Capza1	453	430	369	554	353	696
Ccna2	88	54	44	57	21	222
Dlgap5	22	6	11	10	3	55
Dsn1	104	48	44	63	57	132
Gm7538	2	2	2	1	2	1
Golph3	4608	3046	2656	4061	2560	4066
Lrrc42	312	168	137	270	157	321
Mga	3746	2694	2276	3137	2398	3446
Mir7091	16	10	5	12	11	20
Mtch1	3938	2346	2106	3844	2086	4138
Nars	4255	2960	2906	3745	2630	4270
Phf19	86	51	40	65	55	104
Prkar1b	67	59	58	73	49	132
Rasl11b	1458	1005	613	1869	810	1056
Rem1	218	128	102	217	110	227
Rwdd2a	21	19	4	21	15	24
Sass6	239	132	88	166	101	175
Slc10a3-ubl4	945	670	588	837	595	832
Slc26a7	11	9	9	13	5	7
Smpd1	8885	5386	5380	7669	4566	8367
Tg	54	34	27	43	60	40
Timm21	1643	1068	1005	1293	974	1631
Wdr36	1537	1079	896	1374	826	1514
Zfp182	601	436	321	441	396	852
Mrpl10	2629	1646	1461	2138	1372	2519
Zfp87	213	143	132	198	133	281
9530077C05Rik	17	11	20	15	8	22
Ada	26	18	19	25	19	42
Cd74	4100	2574	1788	4525	1463	4944
Chrm4	1	1	7	2	0	5
Depdc1b	6	1	2	3	1	12
Dnal4	566	345	316	510	347	616
E130114P18Rik	4	5	2	2	4	2
Eif4g1	24717	17498	14813	20618	15620	21420
Fam181b	135	80	69	100	85	173
Fbl	36	25	25	31	15	37
Fibin	919	670	326	1221	428	746
Insr	10730	6920	5879	7839	6137	10311
Kptn	663	361	274	535	365	625

Lama1	1	1	4	3	0	1
Lmo1	78	26	42	52	44	95
Nrg2	70	73	31	79	23	65
Nt5dc2	777	421	395	534	380	719
Pmel	6	3	7	5	3	5
Pum1	3704	2544	2142	3288	2090	3355
Rab44	3	7	0	3	1	3
Rdh13	1437	851	910	1290	795	1210
Rft1	372	203	232	302	256	427
Rnf170	669	517	473	588	411	760
Scfd1	1323	1002	889	1117	826	1472
Scn10a	212	156	133	228	110	315
Sec61a2	708	473	345	539	364	667
Slc22a15	198	172	127	204	156	260
Sp140	99	85	65	103	66	158
Sp6	14	15	6	18	11	20
Thap4	1345	862	737	1054	753	1251
Gm16576	317	198	188	290	180	218
Oxsr1	3930	2492	2305	3108	2377	3469
Tlr13	115	71	57	104	36	99
Zfp266	1987	1215	1073	1671	1193	2201
Dusp5	114	73	32	101	36	58
Rbm38	4943	3202	2388	4162	2683	3677
Abcc4	889	617	584	791	681	1031
Gmppb	214	122	117	202	124	231
Ppwd1	388	277	252	282	237	430
Bcl9l	1705	1066	764	1458	863	1174
Edn1	346	198	160	240	150	325
Etv5	587	489	376	702	331	820
Gemin7	578	316	354	564	327	635
Gm13031	5	2	4	7	5	5
Gm9961	3	2	1	3	5	2
Hint3	906	509	532	839	489	1044
Mgrn1	9794	6005	4811	8255	4835	7901
Nbea	1633	1046	912	1677	1108	1427
Pfdn4	328	237	220	285	188	343
Rgs2	2066	1208	1509	1827	1348	2812
Spin2c	3	1	5	5	3	7
Tmed7	5272	3303	3130	4618	2665	5041
Tmem53	355	207	170	284	187	431
Tti2	653	366	309	474	307	571
Txndc5	2039	1292	1036	1684	885	2037
Rps12	63	19	28	29	23	48
Mrps15	1943	1605	1258	1569	1242	2293
Erbb3	448	246	191	311	253	257

Ube2d2a	1322	826	824	1065	673	1430
Aspa	163	101	89	112	67	180
Commd5	262	177	140	234	146	343
Btbd9	973	522	463	718	523	684
Card9	50	34	21	33	41	61
Ctdsp1	3337	1971	1572	3020	1856	3137
Dgkg	38	19	35	42	30	53
Dimt1	183	158	140	149	130	212
Nap1l5	60	51	64	66	23	92
Nufip1	318	272	244	265	211	398
Ptbp2	1274	894	740	1114	856	1498
Sephs2	1267	881	845	1139	760	1254
Sla	95	71	72	125	47	155
Slc5a3	312	189	213	327	150	241
Sqle	26	11	17	27	12	36
Treml4	0	2	1	2	1	3
Utp15	553	399	291	445	284	564
Vps13d	9208	6543	4936	7508	5613	7845
0610030E20Rik	1324	896	720	976	753	1341
Gm5124	7	3	7	7	1	6
Pram1	25	20	10	11	15	23
Ryr1	8	2	3	3	2	5
Spata20	9	4	3	6	10	7
Xpa	258	196	203	249	206	378
BC003965	1448	820	879	1143	749	1413
Il4ra	626	374	416	531	359	915
Man2b2	1077	763	493	897	659	1215
Rbfox2	5789	3807	3199	4953	3249	4924
Tac1	60	77	85	112	39	205
Tdp2	957	623	618	718	541	1070
Aspg	5	4	5	7	3	12
Capn12	13	6	5	13	13	7
Fam131b	37	37	26	39	16	43
Slfn3	153	101	98	140	107	193
Stam	1068	740	609	941	569	1034
Xiap	4543	2643	2470	3610	2584	5439
Axin1	945	580	469	837	576	922
Eme2	227	166	132	166	142	220
Gjb5	2	0	3	4	0	6
Palb2	67	33	23	44	37	76
Snx9	1163	848	680	1029	684	1093
Clpb	3126	2081	1721	2556	1757	2946
Lancl2	951	620	461	759	487	847
2700054A10Rik	35	30	15	24	20	30
Gm6251	2	2	2	0	2	2

Trip13	24	12	9	23	11	27
Zbtb49	98	70	47	89	72	106
Ccnf	13	9	10	15	4	31
1110008L16Rik	457	318	244	366	254	425
Hspb2	1719	919	859	1574	830	1787
Plekhb2	5691	3707	3303	4930	3192	5014
Lnpep	3008	1931	1727	2999	1511	2312
Ap1ar	971	774	674	974	596	986
Ttc39d	3	2	1	4	2	4
Hmgcl	1879	1108	961	1645	990	2066
Arl2	1014	557	505	803	574	1074
Ska2	344	244	200	299	210	278
Snapc2	396	201	145	308	190	394
Ephx4	9	2	4	5	2	18
Pus7	704	496	424	591	415	684
Syce2	11	8	8	14	10	14
Camk4	18	10	3	9	4	13
Cebpa	176	101	110	169	84	256
Gfod1	2832	1811	1456	2383	1680	1921
Trim6	52	39	30	35	38	91
Srrm1	6030	4775	3274	4376	3669	6551
Cd320	520	271	252	381	201	489
Hist3h2bb-ps	0	3	2	0	2	1
Kctd20	1340	839	774	1124	741	1206
Lgr6	1240	980	612	1079	728	1069
Adprm	423	287	200	338	268	490
Abcf1	5155	3695	3030	4144	3085	5211
Asb13	668	375	407	633	393	731
Colgalt2	605	409	366	549	356	801
Cradd	376	202	177	293	229	376
Hoxd8	50	19	20	39	20	59
Hspa13	1137	749	667	1009	607	1064
Kif3b	908	619	514	763	496	879
Krtcap3	31	13	15	22	20	36
Laptm5	706	449	303	566	251	684
Mief1	1371	771	836	1191	711	1183
Ncstn	2640	1850	1244	2524	1393	2994
Nuak1	1872	1220	1027	1778	986	1432
Nubp1	437	165	215	346	230	409
Polr3g	320	193	193	225	215	245
Pts	1766	1106	1047	1424	944	1787
Rapsn	599	255	251	430	225	430
Rb1cc1	5766	4197	3556	4888	3554	5665
Setd7	5086	3377	3188	4350	3121	4551
Sirt7	441	251	224	364	231	527

Snhg10	12	3	4	7	9	11
Top2a	200	85	67	121	40	362
Tti1	1413	981	787	1157	881	1202
Zfp462	512	384	267	447	385	553
Tacc3	115	83	52	91	65	168
Camkmt	52	32	36	51	31	78
Dlat	26452	17184	16908	22706	14531	26099
Gins3	84	56	55	81	48	129
Ino80b	333	212	168	272	204	388
Prkcsh	2176	1339	1183	1913	998	2028
2310007L24Rik	30	20	25	24	21	62
Efcc1	60	32	23	46	17	45
Hectd1	9142	6816	5374	8344	5761	7503
Nmnat3	826	526	459	627	478	758
Polr2c	1183	746	551	947	669	1323
Pvt1	28	27	27	28	13	40
Tmem45b	1	4	1	6	1	4
Wwp2	1179	781	537	1020	733	1167
Fbxl15	78	46	44	65	51	98
Rab13	159	98	108	138	104	225
Rgn	22	12	19	24	19	29
1700066B19Rik	5	2	2	5	4	5
Araf	3830	2451	2027	2879	2014	3835
Ascc2	787	552	369	612	465	620
Atp5e	6835	3178	3923	6332	3097	8096
B3galt2	406	234	280	274	305	289
BC029214	311	164	178	240	186	379
Bola1	272	179	134	221	179	306
Cuzd1	40	26	14	33	30	39
D830032E09Rik	335	266	270	282	292	544
E030013I19Rik	6	8	7	12	8	11
Ear12	2	2	2	3	3	4
Ear3	2	2	2	3	3	4
Ecd	1824	1213	1018	1561	986	1718
Emr4	28	25	14	20	14	29
Fam78a	1093	700	609	1378	771	737
Gm1045	0	4	2	2	1	1
Leprel4	958	575	522	791	559	1106
Lrrc59	1820	1218	970	1509	961	1748
Mir7026	6	6	5	1	3	10
Nlrp5-ps	20	25	5	23	21	24
Nt5c	288	177	116	245	155	302
Pou2f2	46	43	25	54	34	61
Rad1	309	181	170	247	149	279
Rasal1	4	0	0	1	0	1

Reck	560	397	267	494	231	541
Taf3	884	741	524	841	528	881
Tceanc	159	101	68	101	128	169
Tma16	169	123	113	116	112	178
Zfp772	141	70	106	98	72	148
Ccl26	4	0	0	1	0	1
Cd160	4	0	1	1	0	2
Zbtb10	1188	770	760	941	715	1194
Meox2	1147	674	577	906	649	1294
Mrps25	1370	1015	916	1122	821	1593
Tenm3	364	280	153	353	170	252
2700089E24Rik	6160	4593	3987	5087	3888	6509
AI182371	17	11	16	15	14	34
Fcgr1	78	51	44	95	24	127
Gin1	503	337	278	363	284	508
Isg20l2	760	469	410	621	367	723
Kif13a	9150	6454	4793	8212	5266	7018
Mpv17	3327	2265	1960	2930	1715	3439
Nthl1	94	63	37	81	63	96
Set	1588	925	916	1219	788	1624
Slc25a2	9	1	2	5	5	8
Thoc7	1959	1451	1260	1601	1181	2196
Tnfaip6	69	35	23	51	22	44
Upp2	7	8	5	6	4	7
1500015O10Rik	8	4	5	15	3	8
1700023L04Rik	7	5	2	5	3	14
2310003H01Rik	560	344	190	419	245	394
Ablim1	29799	21336	17591	23824	19092	26638
Akap8	3850	2711	1989	2820	2218	3839
Amd2	2281	1469	1084	2117	1058	1718
Aste1	266	151	145	222	121	226
Calm1	10570	7037	6600	9789	6478	13335
Cant1	1101	639	534	889	628	1155
Car10	6	0	2	5	2	6
Ccdc83	9	3	7	6	1	9
Cct5	5687	3797	3298	4766	3042	5685
Cdc42bpb	4615	3279	2139	4059	2841	4924
Clip1	20456	14553	10979	15699	12974	17328
Corin	23989	17432	11557	20646	12936	19810
Crybg3	4222	3074	2716	3628	2712	4116
Cyp4f41-ps	4	1	1	2	0	2
Dbh	205	157	84	249	98	220
Dguok	729	468	405	648	445	823
E030024N20Rik	13	5	5	17	5	11
Eif2a	3058	2192	1733	2546	1790	3023

Elmod1	9	4	3	5	2	6
Erh	75	37	49	79	31	110
Etv1	393	329	215	417	259	472
Fundc1	1503	962	807	1250	777	1402
Gbx1	1	1	1	2	0	4
Gnl3l	2588	1628	1362	2211	1398	2106
Gpbp1	4789	3310	2780	4090	2774	4489
Ikbkg	2450	1461	1416	2059	1303	2195
Klk10	6	6	3	4	6	13
Lmbrd2	323	222	180	258	208	246
Mre11a	359	270	233	316	233	329
Mrpl50	3459	2457	2322	3014	2154	3533
Mt1	4311	2808	1894	2964	2272	3950
Mybphl	338	268	234	388	189	272
Ndst3	20	7	5	9	12	23
Npr3	1239	993	857	757	810	1674
Oaz1	1589	838	788	1429	758	1668
Rbm42	1176	672	554	1023	606	1214
Rbm7	1001	673	545	857	561	1287
Rmi1	280	181	215	234	163	307
Rph3al	265	151	136	169	151	225
Rsbn1l	2263	1441	1164	1786	1520	2368
Sergef	133	61	68	80	63	111
Sh3bp2	116	67	59	87	40	115
Sin3b	1685	983	852	1406	949	1783
Slc20a1	1435	972	877	1141	866	1444
Slc25a26	925	551	565	766	486	889
Smdt1	2609	1649	1566	2411	1437	3274
Stard7	9137	5146	5021	7740	4453	7748
Stk35	1527	942	649	1339	792	1548
Tmem199	390	276	252	307	210	450
Ube2l3	4532	3126	2599	3897	2533	4338
Usp38	1634	1087	990	1431	885	1625
Zfp341	136	122	79	149	104	169
Zfp354c	284	218	136	268	179	332
Zkscan7	195	127	136	142	128	189
Amd1	2281	1469	1084	2118	1058	1718
Nabp1	1978	1339	1283	1436	1554	2608
Txnl1	3826	2546	2428	2949	2264	4098
Snord82	2	0	1	4	0	2
Fbxo5	64	50	23	60	33	87
Ankrd6	178	149	101	177	92	163
Cd7	14	6	2	10	1	6
Slc1a1	164	94	158	151	122	296
Sik1	921	585	764	607	863	1447

Def8	2830	1853	1762	2613	1503	2732
1200014J11Rik	1105	781	648	905	645	1119
1700034P13Rik	1	1	1	4	0	2
Acadsb	9557	6698	5297	7436	6135	8197
Actl9	4	1	1	2	1	1
Afap1l1	5804	3654	3078	4989	3171	7025
Amigo3	75	41	39	78	48	68
Ap2a2	3730	2194	1763	2972	1845	3083
Arhgap35	4565	3283	2474	4244	2629	3600
Bbip1	1027	726	603	847	742	1215
Ccdc141	7258	5383	3983	6105	4628	6083
Ccl4	3	3	0	2	0	2
Cxcl1	55	44	37	51	29	54
Dars2	1030	686	616	890	582	975
Dnajc10	2849	1917	1566	2678	1680	3144
Dus2	383	217	207	238	214	360
Elmsan1	1514	970	812	1469	958	1427
Elp6	96	55	31	66	26	82
Erc2	32	19	17	20	22	18
Gm10791	8	9	4	3	2	13
Gm13242	7	1	0	3	6	3
Gm4841	593	26	26	220	10	174
Gm9159	1	2	0	3	0	3
Gpr3	1	1	1	4	0	2
Hibch	4779	3301	3166	3991	2868	5070
Hnrnpf	753	518	452	643	411	761
Hyls1	104	59	63	92	62	134
Ist1	2512	1471	1340	2234	1388	2687
Kbtbd2	1598	963	897	1414	950	1722
Kmt2d	4653	3029	2054	3283	2457	4181
Mettl6	395	305	262	377	274	541
Mir145	4	2	1	2	1	2
Mrpl28	2708	1460	1583	2395	1453	3114
Necap1	1011	666	616	927	532	958
Nsmce2	1220	926	692	1036	689	1277
Ogg1	197	105	61	151	97	193
Ovol1	2	3	2	1	2	2
Pdlim1	1616	992	973	1342	759	1738
Pkd1l2	2	0	1	2	0	4
Ptn	114	110	32	93	45	104
Pxmp4	816	512	456	713	472	966
Rbm22	1195	887	659	1117	754	1408
Rccd1	194	118	100	135	82	203
Rhoj	1336	969	795	1296	756	1761
Rnf115	1239	792	795	1144	820	1420

Rnf144b	1968	1050	956	1361	1081	1544
Scin	152	113	117	118	121	266
Seh1l	1904	1184	1066	1486	1029	1864
Senp5	1355	910	873	1126	811	1375
Slc26a9	3	1	2	0	2	2
Spag17	0	2	3	1	2	0
Stim2	1198	825	614	1079	739	1295
Supt6	3269	2525	1890	3010	1923	3157
Tnip2	456	280	205	347	286	483
Trnau1ap	595	373	321	506	345	706
Ube2o	1685	1161	835	1361	959	1456
Zap70	9	3	1	12	2	6
Zcchc24	3140	2036	1478	2909	1643	3341
Zfp513	299	189	193	264	196	381
Zfp952	606	432	354	513	347	589
Zmat3	703	412	361	612	325	570
Zscan21	707	462	425	620	443	867
Mir208a	49	38	15	17	27	47
Adcy3	174	86	89	163	75	207
1700019D03Rik	14	5	7	7	8	23
Cnot10	1645	1052	777	1283	830	1490
Ggh	485	349	277	459	299	596
Gm2a	1232	948	690	1247	717	1534
Mettl22	913	615	486	695	521	874
Mir6998	3	2	0	2	1	0
Ryr3	85	67	47	79	74	95
Slc8a3	8	5	2	6	1	5
Stard3nl	485	283	259	415	277	545
Trmt6	520	364	336	387	319	566
Vps52	2507	1503	1199	1959	1261	2142
Zfyve27	1072	616	511	818	632	1125
2610015P09Rik	367	277	205	232	221	415
Clec4b1	10	2	4	8	2	15
Mgam	9	4	4	4	0	10
Vapb	5610	3636	3392	4745	3086	5485
Arhgap12	2111	1390	1259	1728	1246	2008
Gsta2	3	0	1	0	5	2
Mcat	1038	677	537	932	607	1126
Snora33	7	4	9	13	6	11
Trim44	3775	2886	2298	3181	2285	4029
Ttc8	154	114	93	150	98	207
Zdhhc4	547	387	278	449	319	723
Lyz2	3209	2467	1883	2808	1495	3906
1700040L02Rik	742	588	473	640	461	800
Acat3	9	3	6	5	3	7

Cideb	123	99	63	138	67	161
Fendrr	1	4	2	2	5	5
Figf	150	130	88	125	105	249
Gbas	39632	24381	21901	33953	19482	35171
Impa1	1064	692	606	917	562	1004
Ncl	14348	11053	9406	12004	9162	15430
Rplp2-ps1	105	74	34	51	68	67
Tmem256	659	363	347	601	330	732
Cnnm2	271	175	121	261	157	264
Elmod3	1016	533	425	731	529	1068
B430010I23Rik	6	4	1	2	6	9
Taf2	1381	898	794	1167	786	1247
9430083A17Rik	20	15	21	11	21	17
Actr10	2658	1588	1496	2398	1445	3053
AI462493	871	560	546	744	548	1111
AI662270	69	51	30	45	35	65
Anp32b	2624	2003	1608	2083	1552	3034
Anxa13	36	37	16	39	29	49
Aqr	1800	1309	985	1689	956	1647
Bmp4	535	347	219	495	190	417
Cap2	9284	7632	6160	9497	5809	8784
Casp6	433	340	301	340	242	565
Ccdc90b	1551	1062	939	1221	984	1493
Cfb	97	53	32	64	38	141
Clec2l	23	14	16	19	8	23
Cml1	49	16	27	32	25	67
Cxadr	2467	1681	1659	2299	1439	2412
Derl3	4	0	0	3	2	2
Dgka	1149	823	544	984	700	1307
Dhtkd1	485	297	247	258	225	576
Elmo3	65	38	28	52	50	60
Emc6	1817	1178	1142	1536	1034	1804
Fgfr3	648	300	224	535	238	665
Hlcs	356	284	203	303	266	461
Kank1	2552	1926	1628	2473	1518	2449
Kcns1	5	2	2	4	3	7
Kif18a	69	51	37	70	46	83
Lats2	2536	1694	1300	2318	1500	2694
Ldlrap1	630	421	303	581	336	721
Lin54	634	416	352	495	346	618
Lrrc4c	47	57	44	37	32	81
Mat2b	4688	2835	2675	4196	2713	5278
Mir1982	10	19	11	11	9	17
Mkrn3	26	15	18	16	15	24
Nlrp1a	5	10	2	2	2	10

Nodal	1	0	2	1	1	4
Ogt	10217	6476	5141	6992	5349	10375
Pcdh8	3	3	1	1	0	4
Phf11a	13	13	4	2	1	23
Phf3	3313	2412	1924	2672	2116	3171
Pla2g4a	216	179	113	223	74	225
Ppp3ca	2450	1934	1663	2330	1791	3200
Repin1	1052	588	518	891	488	822
Selt	9369	6850	5803	8140	5709	9245
Slc16a2	737	583	402	710	440	965
Slc27a3	34	28	17	37	28	37
Slc35e3	288	201	151	248	156	243
Slc39a1	1933	1086	968	1588	998	2180
Slc6a19	3	2	2	3	1	1
Sptssa	1726	942	830	1436	842	1899
Srsf1	8300	5292	4470	6544	4193	8318
Syncrip	5395	3839	3395	4652	3239	5412
Synm	975	623	520	820	582	1115
Tatdn2	1690	1082	1073	1503	959	1590
Tfdp2	6877	4048	3509	5883	4635	6167
Tm2d1	837	458	491	676	363	843
Tst	459	234	174	353	253	408
Vdac3	4015	1836	2389	3605	1924	4720
Wapal	2511	1829	1408	2069	1612	2194
Zfp108	109	88	67	92	69	101
Zfp292	2208	1530	1254	1753	1360	2063
Map1lc3b	5498	3206	2656	4552	3145	5582
Nup153	2384	1607	1226	2129	1277	1946
Pja1	1040	645	597	958	688	1030
Vpreb1	2	0	0	0	3	1
Vwf	3523	2541	1373	2101	1694	3800
Ankrd13c	1178	840	700	1135	661	1030
Ccnjl	104	75	72	122	37	93
D630039A03Rik	25	12	14	19	32	14
Pik3c2b	1970	1262	862	1707	1247	1761
Slc2a13	57	33	36	46	32	42
4931428F04Rik	52	36	38	54	43	65
A930016O22Rik	120	45	54	74	39	98
Cpeb4	5909	4058	2958	4636	3577	4738
Dnajc17	177	132	104	157	141	207
Prom1	131	49	42	117	58	96
4930528A17Rik	15	9	9	9	11	27
Stx1b	22	23	14	21	14	20
B430306N03Rik	9	7	3	6	3	7
Phtf1os	56	21	24	32	35	62

Rab14	8112	5420	4760	6984	4354	8013
Tor1aip2	3375	2346	2028	3174	2229	3798
Wdr33	2786	1999	1697	2394	1630	2888
1110051M20Rik	463	296	228	381	233	391
B3glct	578	391	289	420	310	568
Gnaq	2257	1502	1394	1982	1441	2771
Mark3	2883	1988	1595	2441	1601	2787
Mterf1b	43	20	16	23	17	33
Tia1	2182	1391	1121	1524	1202	2161
Vsnl1	14	11	3	7	10	4
Gbp2b	679	4	1	608	378	3
Med10	440	311	378	462	315	676
Dync2h1	275	177	115	267	151	265
Elavl1	2219	1379	1238	1858	1127	2122
15-Sep	4284	2855	2595	3751	2541	5611
Clec4g	10	8	8	11	5	23
Fads2	158	114	73	153	89	187
0610012G03Rik	1615	1030	1108	1415	897	1705
1190002F15Rik	4	3	1	3	2	8
2010109I03Rik	2	3	1	5	2	3
9030025P20Rik	33	17	14	16	15	28
Abcc1	1832	1273	941	1572	1318	1803
Abcd3	8259	5778	5490	6293	4775	9857
Abhd14b	556	362	266	407	356	655
Abhd5	3083	1798	1916	2721	1686	2690
Ap3b1	2215	1825	1195	1874	1446	2036
Asnsd1	4820	3021	3096	4062	2694	4823
Bnip2	6330	4529	4061	5329	3692	6850
C1ql3	8	4	6	10	6	10
Camk2a	2541	1995	1478	2336	1586	2318
Casp2	612	359	290	492	354	647
Cdk17	1383	900	728	1075	855	1655
Chst7	129	92	63	127	73	156
Clp1	231	150	119	244	127	242
Clptm1l	2789	1747	1561	2411	1412	2613
Coro6	10470	6341	5378	5878	6046	10561
Cpq	1757	1263	1082	1664	904	1839
Crip3	55	13	8	27	11	23
Cryl1	141	103	79	127	80	208
Eif2s3y	0	0	2198	0	1802	3650
Eml6	478	363	293	428	292	452
Epm2a	362	241	229	324	231	270
Ezh1	2552	1742	1288	2276	1613	2627
Fahd2a	773	570	496	745	446	1114
Fam160b1	1491	950	779	1355	884	1538

Fam193a	1386	1079	679	1123	800	1328
Fam221b	14	12	4	14	5	6
Gkn3	9	12	10	8	13	25
Gmds	120	75	84	107	81	169
Gprc5a	24	18	23	19	22	51
Grsf1	12504	8880	7976	10718	7910	11880
Gtl3	762	543	385	614	498	907
Htra1	4131	3063	2134	3591	2136	4106
Hunk	48	18	44	30	18	60
Il15ra	1353	859	641	1064	742	1087
Il1r1	931	691	593	946	568	1192
Il27ra	124	68	47	69	50	113
Itfg2	506	329	268	436	245	462
Klf11	739	385	420	538	388	1009
Klhdc2	4813	3242	3320	4403	2802	4882
Matn4	15	6	7	11	5	8
Meiob	17	13	6	11	8	13
Mir3473g	4	0	0	0	0	2
Mir5129	8	10	6	19	8	8
Mir6941	6	0	0	1	3	6
Ms4a7	46	36	33	50	24	85
N4bp2l2	1922	1287	1165	1584	1129	1896
Nfic	8426	6148	4104	7822	5604	8426
Npc1l1	11	11	9	11	6	11
Pafah2	612	414	356	563	291	601
Pds5b	1956	1429	1054	1622	1181	1786
Pias2	1696	1133	950	1321	942	1738
Pigf	80	58	51	90	56	93
Pigo	974	556	439	812	448	723
Ppan	266	167	126	165	150	241
Prr5	47	41	33	51	29	84
Rabgef1	1009	685	657	936	558	1002
Ralgapa1	6102	4011	3393	5173	3403	5430
Rgag4	127	87	59	126	77	134
Rheb	4167	2728	2535	3524	2329	4129
Selk	2029	1209	1132	1772	1130	2324
Setd3	4162	3113	2735	3678	2584	4365
Slc7a10	2	3	3	4	0	2
Slfn1	28	22	7	21	4	27
Snord72	11	7	10	7	6	12
Sucla2	33574	23666	21437	28128	20241	33620
Taf5	300	161	151	199	124	305
Tmem177	725	406	393	575	390	580
Tnks2	6495	4083	3907	5768	3592	5663
Tnnc1	83265	59230	52542	80283	52581	110479

Tns1	59623	40520	32105	59040	40174	57941
Trappc8	2931	1864	1609	2431	1636	2592
Vdac2	16330	10210	10068	15100	9842	19616
Zbed6	2078	1001	960	1463	1063	2270
Zfp280d	1203	813	683	948	789	1500
Zfp687	703	388	350	562	399	748
Zmiz2	2512	1683	1108	2052	1290	2043
Epgn	8	8	9	10	7	4
Mstn	11	18	14	14	29	17
Sbspon	1	3	3	2	2	1
3110039I08Rik	2	3	3	1	2	3
D630032N06Rik	2	3	3	1	2	3
D630045M09Rik	118	100	75	76	64	160
Epc1	1680	1213	912	1394	1006	1559
Kctd21	268	172	125	207	140	217
Lrrc51	81	50	61	58	52	79
Ube2q1	1452	965	847	1313	870	1721
Cerkl	12	18	13	20	12	30
Fst	26	24	23	15	16	40
4930412C18Rik	46	61	35	35	32	75
Slc6a7	4	2	3	4	3	7
Fcer1g	183	172	126	224	97	329
Cd2	15	18	8	14	6	18
1810032O08Rik	117	59	58	65	52	113
Akr1a1	6194	3808	3334	5685	3235	7242
Ccdc114	77	44	33	55	36	56
Cd200r4	8	1	3	2	0	7
Hoxa4	27	7	9	19	19	17
Igsf8	403	218	177	412	168	404
Pld1	846	492	403	780	399	927
Plxna1	1701	1101	846	1513	893	1307
4930453N24Rik	1132	781	839	1019	668	1269
Lemd3	1137	714	641	981	600	1028
Ppp1r3a	14666	9741	8296	11891	9274	11598
Reps2	143	67	99	119	43	154
Tceanc2	643	423	348	540	341	585
Casp8ap2	753	478	419	620	478	868
Lrrc9	27	15	20	8	13	37
Eci1	9257	5014	5106	6047	5166	8639
Pax3	4	1	1	0	1	3
Prdm11	55	35	26	41	30	38
Pex16	532	252	227	377	272	554
Kifap3	2207	1703	1321	2025	1337	2142
Rbm5	4017	2892	2138	3230	2304	3934
Sdhaf2	2624	1798	1657	2127	1602	2706

Zfp637	575	321	267	483	336	544
E130008D07Rik	0	0	2	1	0	4
Gm1661	3	0	1	2	2	3
Mrpl47	746	561	559	657	480	853
Rtfdc1	1327	841	756	1172	807	1487
Ccdc13	8	4	1	2	1	7
Ptpra	2277	1665	1327	2222	1391	2710
Sec23ip	1737	1232	966	1496	972	1688
Nrf1	561	394	310	495	291	533
Glis1	8	2	1	4	1	3
Tcp11l1	79	63	43	73	41	68
Sugp1	1090	777	582	878	694	1373
Rab23	551	358	284	417	248	588
Ddb2	286	165	130	183	171	358
Sppl3	1390	769	668	1111	809	1360
Suv420h2	358	219	150	274	210	382
4930567H12Rik	2	2	0	2	2	3
5830403L16Rik	3	1	4	1	1	4
6230400D17Rik	14	9	4	11	15	10
Abca12	232	142	75	148	242	119
Alkbh7	880	453	449	796	393	995
Ankrd39	243	139	130	191	102	233
Asah2	1425	1102	857	1355	963	1728
Atp6v1b2	4156	2521	2492	4054	2565	4241
BC100451	5	4	5	7	4	10
Col7a1	61	25	21	29	29	38
Creb3l3	4	2	0	1	0	3
Gjc3	36	23	23	34	14	31
Glrx5	1998	1106	1130	1671	1101	2308
Gm13483	14	16	12	17	8	14
Hrc	53505	40638	33198	47084	33457	50416
Itpr3	723	430	317	615	391	727
Kbtbd3	633	420	411	569	416	796
Map3k7	4670	3238	2825	3725	2721	4944
Mfap5	868	641	529	1082	366	1222
Plet1	13	4	5	6	6	6
Ptpro	179	139	77	155	66	179
Ptrhd1	183	108	80	151	93	215
Selm	589	377	321	581	310	674
Sh3bgr	7466	5925	5181	7947	4977	10023
Smg5	3230	1994	1638	2724	1801	2409
Sorcs2	925	632	367	1038	511	736
Tead4	128	80	59	96	94	134
Usp33	2429	1629	1383	2006	1366	2372
Wac	2510	1960	1482	2238	1562	2446

Wrap53	303	169	151	277	173	294
Zfp945	1311	1061	909	893	1001	1479
Amotl1	5330	3397	2992	5882	3113	4990
Slc30a2	20	7	17	13	10	17
Trim45	155	112	85	145	110	175
Pigx	402	285	256	357	200	443
Birc3	425	268	268	359	271	549
Glg1	4372	3364	2424	4312	2611	3339
Wwp1	4997	3907	3402	4286	3656	4641
BC020402	120	71	72	115	80	130
Pik3cb	1137	729	714	1041	789	1221
Plk1	30	26	12	16	9	85
Tk1	39	16	19	26	19	54
Ptpn22	39	26	18	35	13	31
Uaca	4690	3409	3158	4170	3609	5743
Spint1	3	2	1	2	2	0
Exoc8	1662	1035	892	1353	920	1456
Gas6	7645	4991	3742	6951	4030	8620
Rmnd5b	538	305	253	442	301	555
Zfp930	707	554	497	602	470	786
Hoxb7	7	0	3	1	2	4
Spata25	2	4	2	1	2	3
Bcar1	826	551	399	781	457	876
Gle1	1434	857	786	1244	865	1502
Mettl8	262	149	158	175	147	257
Muc1	23	25	7	21	22	30
Olfr1034	47	24	29	32	21	44
Rpl27a	669	425	413	514	342	745
Serpinc1	3	4	2	6	3	5
Cdc25c	8	2	3	5	0	18
Dhx32	1706	1163	1122	1455	1044	1740
Ptprs	3591	2311	1681	3022	1713	3162
Pyroxd1	663	452	394	555	482	757
Tstd2	931	567	514	754	495	850
Vwc2	23	25	10	24	12	17
Zfp536	107	42	39	74	67	84
Klkb1	6	1	4	2	4	2
Atpaf2	1413	873	817	1234	771	1225
Il15	1749	1063	821	1356	1014	1870
Sapcd1	4	3	5	1	0	9
BC023829	1045	671	661	1004	534	974
Urod	1969	1206	915	1843	916	2178
Ywhaz	3362	2181	1733	2957	1852	3817
2810474O19Rik	1726	1626	3019	1680	1596	3691
Il17re	13	5	4	5	13	12

Mir704	31	10	13	25	11	37
A330021E22Rik	66	58	27	59	43	93
Cpsf4	359	183	190	310	186	395
Srsf2	5415	3313	2771	4041	2617	5476
2510049J12Rik	38	21	23	21	35	51
6430550D23Rik	14	16	6	10	5	18
Bcas3	1402	1019	734	1483	826	1442
Crb1	51	36	29	32	21	62
Ddx39b	3413	2182	1631	2451	1631	3558
Fabp7	3	3	5	2	1	6
Fosl2	1375	714	843	1196	765	1590
Gm6498	11	3	3	9	6	9
Gramd3	770	456	459	652	409	693
Haus6	325	271	149	245	178	337
Klf9	7217	5332	4787	6873	5016	9092
Mesdc1	402	231	245	371	202	532
Mir5116	118	75	61	97	64	86
N28178	243	181	147	225	124	245
Pter	536	390	438	530	344	567
Rsph3b	503	348	317	413	316	477
Sdcbp	4996	3138	2902	4659	2802	5847
Socs1	12	6	6	11	4	20
Ssbp4	647	390	288	508	359	705
Syngr2	4940	2915	2262	4100	2035	4387
Trmt5	1836	1296	1056	1442	1040	1971
Zfp40	359	260	271	294	270	570
Dnah17	37	21	13	17	29	46
Ifitm5	7	4	6	5	5	16
Olfm1	276	211	169	259	143	285
Pogz	1649	1008	726	1206	832	1434
Sufu	519	337	251	467	325	512
2210016F16Rik	1041	658	570	864	612	1229
Cers3	8	4	4	7	3	3
Cps1	2	1	1	2	1	4
Cstf3	611	399	384	498	340	632
Diap1	1349	912	708	1179	744	1143
Eral1	1010	657	591	824	557	1008
Gm8801	6	0	7	1	6	0
Mtm1	1257	731	615	948	694	996
Plekhd1	11	6	6	25	3	7
Pnpt1	1647	1244	1072	1419	1093	1662
Psmc3ip	103	65	50	71	45	102
Purg	190	102	114	174	130	188
Ypel1	29	16	14	20	7	29
Sptan1	20656	14318	10273	19255	11812	23191

Wt1os	2	2	0	3	1	3
Arhgef1	2380	1309	963	1732	1249	2437
2810410L24Rik	104	56	48	85	64	106
Ace	3474	2162	2173	3174	1968	4403
Adh5	3516	2096	1971	3080	1906	4082
Adpgk	504	319	270	428	227	490
Ak3	7019	4607	4695	6401	4076	6786
Ano8	964	543	407	644	463	837
Apol9a	6	2	3	10	2	5
Cdr2	380	296	233	318	184	468
Cenpc1	422	270	273	357	220	440
Ces1f	5	6	0	5	2	0
Cxcl14	509	429	692	401	247	1180
Ddx27	668	505	382	539	381	720
Fam217b	450	272	180	412	242	412
Gm572	128	90	73	111	67	115
Gpatch2l	819	568	496	722	477	772
Gstm4	391	222	217	351	225	423
Itga4	232	142	118	225	91	177
Lysmd2	459	291	260	313	233	513
Mier2	434	226	175	347	236	396
Msantd3	577	395	394	476	397	522
Odf2l	630	475	358	441	404	665
Oplah	2587	1342	1094	1952	1373	2472
Pin4	3	1	0	1	2	4
Rhbdd1	778	504	458	642	443	732
Rnf5	884	561	530	711	497	871
Samd5	164	89	73	145	99	147
Sdk1	54	47	30	56	25	47
Spata13	1104	711	576	928	660	1232
Tcf7l1	724	522	314	653	405	810
Thumpd1	1277	889	808	1053	759	1354
Tipin	346	248	211	280	218	515
Trim12a	627	484	381	526	417	903
Ttc5	630	392	381	486	420	794
Wdr61	908	555	550	710	470	960
Zfp410	1050	673	612	973	591	1247
Zfp783	26	20	22	31	20	40
Terf1	316	245	238	286	232	498
Dmc1	10	5	4	3	4	9
Gnl2	2105	1572	1206	1803	1219	2160
Prdx2	8361	5426	4786	7759	4872	9609
1700101E01Rik	0	0	2	0	0	1
BC018473	0	0	2	0	0	1
Car15	89	42	29	46	44	55

Cdk5r1	85	78	65	65	78	72
Commd10	379	289	264	422	233	512
Diablo	2772	1731	1655	2295	1486	2773
Dkc1	1135	834	717	939	677	1253
Dnajb11	2279	1537	1359	1899	1238	2402
Endov	319	219	132	249	202	349
Gucy2f	0	0	2	0	0	1
H2afz	84	38	46	67	45	102
Hcar1	8	15	4	17	4	21
Kcna4	72	50	30	77	49	62
Kcne2	0	0	2	0	0	1
Mir7038	0	0	2	0	0	1
Scamp4	683	412	297	534	316	567
Sebox	0	0	2	0	0	1
Stk17b	825	478	461	681	470	968
Zfp786	45	28	22	32	19	41
Slc37a4	704	309	308	584	361	595
Timd4	76	51	35	59	19	84
Prr18	13	6	2	8	5	3
2810002D19Rik	222	171	145	215	128	212
Acrbp	154	97	71	84	83	150
Agap1	1905	1334	1042	1767	1223	2039
Ildr2	54	43	29	67	33	62
Pkia	12986	8228	8313	10389	8338	11515
Pole3	318	245	216	285	177	363
Rab7l1	293	213	177	280	183	377
Retnla	80	50	55	68	42	75
Uap1l1	221	84	68	147	93	211
Utp14b	49	37	26	40	22	48
Nmb	121	46	74	114	58	138
Pygl	250	202	177	214	182	245
Plxna4os1	11	3	5	10	3	15
Prpf4b	3596	2461	1995	2894	2025	3592
Champ1	657	394	327	522	331	564
Snx22	30	9	14	26	9	10
A230050P20Rik	206	171	103	137	101	257
Hey2	402	270	253	317	297	509
Milr1	27	14	12	31	9	32
Nipa1	496	328	284	491	206	463
Nnat	2	2	6	1	2	5
Pinx1	148	138	84	124	92	160
Rasl10a	104	47	43	102	58	76
Rtkn2	27	5	7	4	9	18
Mycbp2	5002	3278	2662	3911	2874	4560
Rragc	2850	1915	1668	2432	1517	2953

Spred2	973	720	585	1017	682	994
Tmem138	69	47	44	59	47	100
Fabp5	822	586	561	754	515	1176
Rapgef6	1781	1229	929	1580	1183	1809
A430005L14Rik	360	240	226	266	222	364
Cecr6	1	1	0	0	3	1
Cobl	285	137	148	187	271	198
Fam168a	2967	1818	1547	2922	1550	3029
Gm5595	250	180	176	155	152	324
Lyrm4	483	356	382	346	342	589
Mepce	1474	840	665	1207	787	1478
Pola2	491	326	291	387	314	658
Snord68	3	1	4	2	2	2
Srebf1	1177	640	485	1087	570	1034
Supt7l	609	408	366	555	322	573
Trim27	576	341	277	491	330	588
2310020H05Rik	47	43	25	28	34	47
Apba2	49	18	22	27	22	33
Atp6v0a4	21	11	4	20	17	9
B930041F14Rik	52	36	22	45	30	66
Cblc	11	11	5	14	11	12
Cxcl2	1	1	0	2	2	0
Elmod2	961	626	530	794	540	874
Gm13034	1	1	0	2	2	0
Hdac2	3183	2400	1953	2729	1992	3287
Lrrc23	17	11	10	12	16	23
Oaz1-ps	1576	822	779	1420	746	1619
Pigs	891	604	408	725	466	753
Plvap	221	189	108	162	153	342
Plxna4	1248	909	784	1054	792	1252
Sap18	1053	702	611	801	611	1086
Sclt1	472	354	331	358	334	504
Slc5a5	8	1	3	8	0	12
Spire2	1	1	0	1	2	1
Tln1	8616	5738	4229	8210	5079	8475
Zfp2	290	212	164	228	203	385
Zfp207	4644	3161	2662	3784	2604	4704
Gm14393	3	2	2	3	5	3
1700064M15Rik	0	1	1	0	0	1
Acy1	468	256	216	368	253	496
Asic2	0	1	1	0	0	1
Atf7ip	2629	2065	1458	2290	1748	2209
Bex2	0	1	1	0	0	1
Ccdc104	726	537	444	651	539	841
Ccdc175	0	1	1	0	0	1

Ccdc33	0	1	1	0	0	1
Cyc1	24133	12581	12019	20729	11895	25918
Fam129a	803	615	453	818	353	814
Fxyd5	453	419	278	513	291	637
Gfap	3	2	0	1	0	9
Gm1943	20	10	13	16	7	17
Gm5084	0	1	2	1	1	4
Gm6623	0	1	1	0	0	1
Hes6	375	206	174	328	196	378
Myl4	2089	1375	1891	2164	1647	1425
Nr1d2	10284	7000	5304	7954	5820	9664
Pcdhb12	41	20	24	21	28	28
Pdcd10	592	417	374	631	329	752
Prrxl1	0	1	1	0	0	1
Pum2	9138	5887	5453	7821	5177	8458
Rasgrf1	4	0	3	1	0	4
Tceal6	0	1	1	0	0	1
Tpsb2	13	12	2	14	5	20
Trim39	695	477	410	620	434	851
Tuba1b	633	397	233	476	250	552
Upk1b	3	2	4	2	9	3
Zfand5	7227	4726	4133	5718	4143	7045
C330007P06Rik	875	514	465	642	489	776
1700123M08Rik	120	92	59	90	66	115
4930503B20Rik	24	11	12	10	18	11
A530064D06Rik	2	0	0	1	1	2
AF357399	0	2	1	3	0	3
Arhgap21	6213	4870	3498	5026	4093	5853
Arhgap9	103	76	76	81	57	125
Atraid	1295	863	735	1132	813	1476
Bcor	1682	1162	919	1537	989	1315
Bricd5	1	0	1	1	1	2
Ctnnb1	20321	12816	10425	18674	11974	20724
Edem3	2342	1670	1288	2119	1366	1997
Emilin3	2	0	0	1	1	2
Fam172a	805	546	427	840	416	902
Farsa	721	456	314	515	330	697
Fnbp4	1615	1204	890	1187	1139	2135
Gata5os	1	5	0	2	1	1
Gpr75	25	9	12	6	10	25
Gramd1b	675	550	443	527	630	851
Grik4	3	0	1	2	1	4
Hck	67	40	29	47	26	60
Hpdl	147	69	86	121	59	122
Hps5	770	451	339	624	406	812

Ifi35	707	396	340	613	325	819
Klra18	2	0	0	1	1	2
Lamb1	5326	3379	2800	4689	2418	5059
Lrp1b	22	24	12	20	8	28
9-Mar	141	74	65	129	73	141
Mir6904	81	59	72	83	56	71
Mir7068	3	1	0	0	3	4
Nyap1	64	40	40	74	22	104
Obsl1	5331	2701	2022	4399	1931	3641
Pex14	645	460	404	638	449	713
Pign	745	459	442	588	422	709
Pira4	12	3	2	10	1	15
Pira7	12	3	2	10	1	15
Ppa2	4353	3177	3127	3815	2419	5404
Rapgefl1	48	20	19	35	26	49
Rnd1	58	38	39	61	33	82
Slco3a1	6276	3975	3215	5084	3431	5338
Tmem165	1151	769	689	1065	680	1393
Ttc38	1744	1127	964	1531	1001	2021
Vcan	739	524	450	686	289	876
Rpp30	485	360	276	404	275	497
C330027C09Rik	111	69	84	97	64	194
Casq1	253	101	175	211	222	202
Nrp	19	12	15	17	16	29
Prr22	18	11	8	10	6	18
Rimbp3	11	6	3	6	0	11
Pcyt2	1492	922	860	1121	753	1629
Rpusd2	240	165	152	209	137	229
Snrnp40	539	379	270	536	313	572
Ccnj	342	213	208	285	184	323
Dio3os	5	3	9	3	6	20
Ephb3	429	219	145	319	175	265
Gm11744	25	19	25	28	11	29
Kcnn2	527	511	330	658	362	680
Tax1bp3	94	46	47	84	53	94
2210404O09Rik	60	45	37	45	48	90
Aldh1l2	432	284	271	357	309	286
Cops7b	482	280	236	393	219	414
Dgkeos	20	8	8	9	11	11
Fam155a	0	0	2	1	0	0
Fkbp3	10035	8024	7154	8528	6765	11581
Htr1b	0	0	2	1	0	0
Mir7023	0	0	2	1	0	0
Plekha7	202	668	455	694	82	665
Spg11	1186	759	688	992	704	1024

Shprh	1126	726	557	949	670	1200
Cklf	33	12	16	26	16	38
Fam163b	16	15	7	14	6	15
Iscu	2040	1481	1325	1775	1434	2623
Itgb2	265	180	120	219	106	256
Itpa	598	367	367	470	321	612
Myo1e	735	468	394	731	409	764
Nek9	9262	5807	5454	7992	5329	7904
Olfr560	5	0	3	2	3	8
Phf1	1004	553	434	778	554	976
Rap2c	2208	1421	1357	1945	1236	2096
Rinl	72	53	43	53	48	120
Rtel1	683	399	318	452	292	718
S100a8	11	5	11	19	12	7
Slc6a13	4	0	2	0	0	4
Tmprss13	105	68	89	119	66	68
Zbtb8a	185	90	84	119	78	161
Zfp81	214	166	184	187	159	237
5330434G04Rik	0	2	0	0	0	1
a	0	2	0	0	0	1
Aadat	0	2	0	0	0	1
Adrm1	308	216	169	241	173	302
Atxn7l3	1317	763	675	1182	740	1298
C4b	600	345	413	482	393	805
Fkbp1a	6036	4030	3407	5565	3513	7083
Foxa2	0	2	0	0	0	1
Gpi1	20324	13984	10784	20012	12085	21639
Igip	962	552	460	704	440	920
Klc4	1320	821	634	1132	786	1300
Lsm14a	3868	2543	2300	3406	2101	3739
Nudcd2	647	411	384	527	363	618
Pcif1	832	583	415	707	508	953
Rmnd5a	8246	5556	4495	6953	4801	7142
Rps23	19	10	9	9	12	32
Shc2	127	90	93	127	44	155
Snrpb	1938	1006	910	1879	800	1988
Sync	1150	969	751	1248	732	990
Tmem38b	1489	916	923	1273	1023	1617
Zbtb18	2536	1539	1343	1843	1510	2196
Znrf2	924	641	550	839	726	897
Bdh2	17	19	5	22	11	23
Eid1	2576	1939	1610	2349	1466	2783
Kxd1	2748	1717	1445	2219	1712	2958
Rap1b	3584	2339	1964	3206	1998	4230
Rnf207	3618	2369	1433	2060	1830	3663

Spata7	260	213	179	221	200	398
Trpc1	314	214	182	270	177	284
Klrk1	23	13	6	14	6	18
Gm11346	6	9	2	7	4	15
Nelfe	379	267	220	340	242	468
Slc25a5	7193	4429	5295	6858	3709	7663
Stra6	14	7	3	3	5	12
Tmem171	104	34	61	54	53	80
Pstpip1	62	43	29	45	28	59
Lysmd3	990	743	564	926	550	949
Agk	323	207	183	271	174	289
Clstn2	37	30	19	41	24	46
Efcab9	2	5	2	4	1	2
Mefv	3	2	2	0	3	2
Mir3060	8	7	3	5	7	14
Nhsl1	1105	777	581	926	821	1110
Pdzk1ip1	0	2	3	2	5	2
Ssbp3	1994	1235	930	1791	1175	1835
Tmc7	626	370	306	515	320	482
Vstm2a	4	0	3	3	0	2
Zfp39	239	202	189	320	172	302
Atp6v1g2	186	142	115	142	130	171
Foxd3	28	24	8	19	10	27
Hist1h2be	100	88	66	91	76	79
A930004D18Rik	237	143	145	256	163	213
Cpt2	10121	6026	5318	7288	5799	8915
1700016L04Rik	0	1	1	1	0	0
2900055J20Rik	6	2	3	3	8	5
4930502E09Rik	0	1	1	1	0	0
6720468P15Rik	1	1	0	0	2	2
A230020J21Rik	0	1	1	1	0	0
Abhd8	681	305	278	592	300	603
Adamts20	40	16	25	19	15	44
Agl	18285	12567	10484	17572	10261	14340
Cgref1	20	8	20	16	13	41
Clec4e	4	1	4	2	2	3
Cryab	55776	34607	38081	45423	29204	62143
Fbxo45	1288	764	767	1176	648	1136
Fgfr1op	1146	741	706	973	653	1113
Galk1	257	146	102	208	141	251
Hipk4	13	2	5	3	7	5
Hmgcs1	2286	1645	1501	2053	1360	2413
Igdcc3	5	2	3	3	1	13
Lrrc2	21376	14736	13744	16167	14706	19943
Ntmt1	337	201	198	339	183	378

Rad52	308	182	154	209	193	379
Shc1	1037	603	525	866	604	1053
Slc25a44	1123	742	617	910	667	996
Slc4a7	1166	754	697	945	690	1107
Snord92	0	1	1	1	0	0
Synrg	2427	1528	1261	2077	1310	1996
Ufl1	2091	1492	1200	1721	1270	2038
Zkscan3	2230	1260	1134	1780	1037	2038
Rufy1	2300	1605	1398	1777	1488	2282
Pet112	1781	1187	1031	1410	1063	1743
Ppp1r10	924	688	687	661	659	1102
Usp32	2385	1633	1426	2055	1479	2131
Vps35	6355	4463	3705	5544	3816	5733
Churc1	993	471	564	833	482	1162
Esco1	894	610	540	772	518	866
Hebp1	222	153	158	194	98	277
Mrps11	664	415	360	600	351	792
Plekhs1	2	2	4	0	6	8
Stambp	723	574	457	697	521	890
Uggt2	466	368	280	436	274	456
Mlf1	7600	4993	4047	5230	4216	8060
Ccdc79	1	2	1	1	2	4
1700001G11Rik	8	3	2	1	4	5
Deaf1	593	376	254	422	378	635
Myb	5	3	1	2	2	3
Sar1a	9812	5812	5273	7910	5209	8568
Atp2b3	4	5	8	7	2	6
Hook3	5149	3249	2619	4355	2518	4686
Sec22c	550	365	293	490	288	466
Ctsl	6956	4067	4063	5916	4324	7541
1500009L16Rik	108	52	76	103	74	118
1500015A07Rik	104	63	49	74	67	126
1700049G17Rik	201	117	134	158	150	246
1700084E18Rik	9	10	5	17	6	11
2010107E04Rik	6894	4492	5397	6968	4335	9932
4930482G09Rik	0	2	0	1	0	0
4930519F09Rik	1	0	1	0	0	1
4933416M06Rik	0	0	2	0	1	0
4933422A05Rik	1	1	0	3	1	0
5730408K05Rik	5	6	4	6	5	11
6430573F11Rik	73	46	45	54	54	100
A930018P22Rik	2	0	0	2	0	2
Acot13	9487	7647	6590	7965	5904	11792
Adsl	2835	1673	1622	2358	1491	2617
Ahsg	142	136	103	157	102	233

Aim1l	1	0	1	2	0	2
Aldh6a1	15417	9629	8169	11442	9396	12691
Ankrd24	332	176	135	171	182	270
Ap5s1	779	490	403	656	378	718
Apobec2	7856	5366	4590	7749	4778	8672
Apom	1	0	1	0	0	1
Arap1	1180	726	516	916	529	1077
Arl10	83	53	40	67	40	66
Arrdc5	1	0	1	0	0	1
Asb6	277	172	131	257	163	269
AV051173	5	4	4	3	3	15
B3gnt6	4	4	1	4	0	3
Bach2os	0	1	1	2	1	1
Bcat2	3914	2431	1892	2980	1824	3918
Brk1	2165	1301	1323	2026	1227	2497
Cdnf	1590	1147	993	1442	1028	1406
Cep128	478	425	332	510	437	535
Cep290	553	404	265	453	330	443
Cfh	8856	7611	5381	9697	4683	8834
Coq2	3786	2225	2007	3039	1860	3604
Crebl2	922	500	435	653	492	728
Ctsj	1	0	1	0	0	1
Cyp2d9	1	0	1	0	0	1
Dnah7a	0	0	2	0	1	0
Dnajc3	3766	2339	2215	3227	2025	3548
Dnm1	532	351	255	391	220	590
Dock8	626	393	371	456	374	614
Dpp9	2343	1522	1217	2004	1217	2111
E130308A19Rik	234	137	108	169	119	191
Eif3c	6737	4895	3827	5795	3978	6403
Esco2	27	11	13	16	5	58
Eya2	82	45	65	93	56	97
Fam25c	0	0	2	0	1	0
Fam83a	1	1	0	3	1	0
Folh1	2	1	0	6	0	0
Foxf2	5	4	4	3	2	6
Frem3	0	2	0	1	0	0
G6pc3	1310	754	681	1018	654	1206
Gcg	1	0	1	0	0	1
Gm13710	1	1	2	1	2	4
Gm4980	115	60	68	95	45	106
Golga1	1437	1059	830	1215	930	1302
Gpr22	2728	2215	1460	3301	1806	2503
Higd1b	239	124	110	188	109	288
Htr3a	0	1	1	1	2	1

Hyal3	13	8	7	11	10	17
Ibsp	1	0	1	0	0	1
Ints9	706	555	408	691	385	681
Itgav	1863	1337	895	1522	994	1777
Kcnj10	17	20	7	12	12	16
Klf16	133	77	107	127	87	196
Klhl29	40	37	38	47	18	53
Klhl8	635	392	384	509	335	647
Krt20	2	0	0	2	0	2
Lipo1	521	372	323	432	277	590
Megf9	598	402	278	479	288	559
Mettl5	581	347	339	458	307	562
Mfsd11	1349	780	766	1182	673	1179
Mill2	238	120	86	147	77	219
Mocs2	1209	868	887	1077	726	1407
Myo15	5	1	1	1	0	4
Nefl	3	0	3	4	1	5
Nfkb1	2416	1504	1241	2143	1374	2522
Nova2	705	366	190	489	354	615
Nsfl1c	1632	1094	901	1392	1053	1796
Nxnl2	1	1	2	1	2	4
Oscp1	95	46	53	85	54	102
Osm	1	1	4	3	1	7
Pbx1	4011	2854	2330	3573	2711	4552
Pdpn	199	134	143	163	88	256
Pilrb1	6	11	1	6	4	19
Plac8l1	0	1	1	2	1	1
Plcb4	4150	2710	2248	3316	2797	4503
Pnma3	0	2	0	1	0	0
Ppp1r26	302	153	130	196	143	244
Ppp2r1a	7582	4794	3794	7342	3913	7986
Ppp2r3d	486	414	240	542	322	507
Prdx5	9991	5846	5932	8592	5521	12363
Prps1	1376	865	752	1035	757	1365
Prr15l	0	0	2	0	1	0
Psg16	0	2	0	1	0	0
Ptger1	13	10	12	12	5	16
Pvrl2	1272	705	592	1063	582	1451
R3hdm1	3895	2730	2023	3034	2356	3542
Rbm12	818	626	512	762	481	838
Rbp4	15	31	13	42	18	24
Rnft1	1597	1049	865	1345	925	1373
Rp1	94	65	38	86	44	56
Rps11	4829	3467	3146	4477	3064	6435
Sash3	71	27	23	42	17	55

Sdr42e1	226	168	135	182	98	281
Serpinb9b	7	1	3	2	4	11
Sf1	4697	3332	2326	3845	2685	4159
Sfxn1	491	348	369	474	318	732
Shroom3	1755	1212	1093	1922	1178	1703
Slc41a3	549	341	352	524	418	522
Smc2os	3	3	2	2	3	1
Snapc3	445	331	325	406	276	488
Snord100	11	6	1	10	3	13
Snord65	4	0	3	4	3	4
Sod2	26921	18751	16519	22657	16350	25794
Srek1ip1	1155	747	739	958	632	1240
Stac2	4	3	5	4	2	4
Stat6	3185	2203	1670	2803	1654	3022
Stc2	343	219	181	307	260	301
Tdrd12	2	3	2	3	3	5
Tex11	5	3	4	4	4	10
Tfe3	787	455	421	704	408	882
Tgoln1	6643	4173	3826	5634	3697	5934
Tmem132a	415	237	190	354	160	346
Tmem56	111	96	90	134	50	127
Tmevpg1	0	2	0	1	0	0
Tmub1	287	174	140	222	119	282
Tnfsf8	1	6	0	3	0	2
Txk	2	3	2	6	0	6
Vps39	1965	1394	979	1622	1078	1831
Yipf3	1649	961	794	1488	878	1601
Zbtb9	303	196	189	289	191	355
Zfp874b	897	528	505	617	506	890
Mbtd1	953	607	597	733	644	1220
Frs2	2319	1608	1244	1836	1387	2170
Steap2	60	45	38	60	34	93
1700084C01Rik	5	3	7	5	2	6
4930474N09Rik	0	1	1	0	1	0
5430402O13Rik	10	4	10	5	7	9
6330407A03Rik	3	1	0	1	1	5
9230114K14Rik	196	142	125	185	168	202
A3galt2	12	9	4	15	8	11
Aagab	911	588	557	866	516	1099
Actn3	6	3	2	5	4	7
Adat2	79	42	47	103	33	83
Apol11a	0	1	1	0	1	0
Apol8	1	1	0	0	0	1
Aptx	744	512	475	578	466	779
Asap2	1274	901	675	1275	794	1271

Asgr1	1	1	0	0	0	1
Atp7b	9	2	1	3	4	1
Cacng6	14	18	19	14	18	15
Cbx5	5293	3357	2848	4377	2947	4620
Ccdc8	225	126	100	260	103	189
Ccdc97	1930	1333	955	1663	988	1771
Cdk13	2243	1630	1229	1949	1307	2104
Chac1	139	73	102	71	129	203
Cited1	23	15	7	17	9	15
Cnih2	14	3	6	9	10	12
Cnot8	1651	1103	946	1306	897	1765
Coil	258	160	157	198	142	256
Cyld	2750	1973	1625	2293	1692	2724
Ddx3x	14302	9877	5732	13222	6270	10458
Defb8	1	1	0	0	0	1
Dek	3226	2110	1962	2792	1961	3971
Dnah2	50	30	23	40	40	47
Dnali1	0	2	0	1	2	1
Edem1	878	575	488	750	515	1054
Erbb2ip	4794	3351	2741	4267	3360	5088
F830016B08Rik	1181	163	179	747	151	266
Fam134a	924	545	441	853	485	905
Fam217a	18	12	8	13	15	22
Fam98b	1257	916	744	1085	753	1252
Gdnf	12	15	12	21	15	17
Gls	4531	2887	2422	3761	2414	4161
Gm14306	1	7	5	4	3	4
Gm4349	1	1	0	0	0	1
Gm9833	13	5	6	8	5	8
Gmfb	2640	1755	1658	2181	1428	2940
Gmnc	1	0	1	3	1	0
Gpn3	837	577	437	659	489	770
Gpr137c	244	173	139	226	138	201
Gpx4	2895	2093	1731	2243	1669	3333
Gpx8	1057	733	691	911	606	1153
Gstm3	0	2	1	2	0	4
Gstm6	37	21	22	35	33	32
Gtf2h2	591	429	380	466	374	635
Gtf2i	11718	7689	6117	10098	6490	9617
Gtpbp3	441	255	203	374	239	450
Ipo13	3173	1890	1322	2632	1755	2920
Kcne3	12	4	4	13	4	11
Kif22	32	19	22	27	10	72
Kif2c	14	10	7	11	1	40
Klf8	39	21	23	27	20	33

Klhl17	750	438	346	581	357	641
Large	2833	2044	1943	2826	1699	2758
Las1l	2519	1822	1494	2003	1573	2593
Lclat1	9333	6614	4977	7674	5641	8257
LOC381967	7	9	1	7	2	5
Maff	298	168	141	240	161	326
Matr3	7904	5726	4592	6758	4685	7810
Mea1	683	455	414	510	409	713
Mff	5361	3495	3170	4674	2922	5147
Mir5107	270	176	115	299	99	317
Mir6921	0	1	1	0	1	0
Mir703	77	25	32	69	18	95
Mir7653	10	2	4	2	3	8
Morc2a	1702	1169	904	1435	966	1556
Mospd1	1884	1193	1172	1604	1235	2205
Mtmr4	1497	976	773	1395	711	1292
Ngfrap1	134	105	59	124	74	176
Npc1	3611	2085	1537	2721	2077	3458
Nrap	39525	28702	25654	35440	26411	33675
Nrbp1	3884	2456	1946	3253	2332	4040
Nsmce4a	1906	1300	1007	1736	1083	2180
Pde10a	185	112	109	179	97	230
Pja2	9241	6677	5533	8439	5673	8180
Pkp1	0	1	1	0	1	0
Polr2j	863	583	555	745	442	976
Ppp1r1a	34	20	22	32	21	46
Prickle4	52	35	21	43	34	58
Psmf1	900	553	511	787	540	996
Puf60	2266	1390	1183	2052	1195	2492
Rassf8	601	521	341	528	481	363
Rbm6	2461	1775	1577	2056	1565	2555
Rbpms2	1619	1127	1023	1497	816	1781
Rcbtb1	732	440	428	595	464	843
Rfk	4787	3172	2520	3763	2702	4499
Rgp1	2427	1506	1405	1938	1621	2692
Rhbdd2	733	454	377	563	389	675
Rhof	22	14	8	23	11	24
Rnd2	59	31	36	40	37	87
Rtf1	3026	2187	1769	2719	1881	3721
Rxrb	1457	889	760	1159	717	1415
Selp	19	24	15	8	20	25
Sfxn2	450	258	226	296	276	553
Skp2	67	50	43	60	22	83
Slc25a42	2943	1796	1309	1910	1555	2761
Slfn10-ps	19	13	14	22	14	25

Snord38a	0	1	1	0	1	0
Sp2	718	478	370	622	451	766
Ssbp1	1166	909	788	924	761	1381
Tbrg1	1299	943	747	1240	762	1588
Tgfbi	1009	945	559	1081	471	1141
Thoc5	789	519	472	698	385	822
Tmem136	699	466	457	505	429	779
Top1mt	201	150	121	179	122	180
Trpt1	459	305	229	415	263	505
Uap1	2197	1443	1259	1915	1087	2262
Ube2v1	316	204	212	336	208	345
Vezt	1748	1131	1012	1371	1051	1666
Wtap	1609	1096	937	1398	1010	1904
Zc3hc1	600	349	283	524	322	608
Zfp386	1677	1268	1085	1203	998	2152
Zmym4	1695	1275	1064	1518	1051	1736
Zswim4	456	255	202	340	250	491
Zyg11a	0	1	1	0	1	0
Tmc3	5	3	1	1	1	5
Ggcx	1284	808	685	1076	611	1270
Metrn	524	263	238	392	192	474
Kynu	5	0	1	2	0	2
C1qtnf3	4	1	2	5	1	5
Ppfibp1	4534	3367	2776	4311	3334	4901
Slc16a13	131	75	80	134	76	144
Aplp1	91	45	44	86	52	82
Blvrb	495	399	331	482	162	697
Fbrs	1271	788	514	1115	653	1238
Ispd	326	184	174	264	154	289
Mgea5	7160	4547	4176	5879	4091	6661
Rcc2	903	541	431	731	382	857
Vti1b	3233	2130	1941	2487	1846	3503
Cog6	4264	2836	2522	3516	2519	4118
Dpy19l1	757	445	357	671	384	792
Gpr114	10	2	0	6	4	7
Gpr133	198	131	168	147	118	261
Grk1	2	4	3	0	3	4
Pbx3	893	667	522	880	445	901
Secisbp2l	3053	2149	1773	2546	1785	3113
Armcx2	427	357	265	456	265	577
0610040J01Rik	114	98	87	84	76	150
1110008P14Rik	431	262	263	392	239	539
1700012L04Rik	1	1	0	0	1	3
2700099C18Rik	12	9	8	8	9	24
2900011O08Rik	1	1	0	1	0	0

5830444B04Rik	67	38	39	29	62	93
AI467606	52	41	23	42	20	53
Anapc15	189	116	96	149	121	213
Ankrd35	85	86	78	94	79	153
Ap3b2	1	0	1	1	0	0
Arpc5l	3327	2260	2341	2779	1965	3848
Arvcf	824	461	469	677	432	715
Atp5j2	15318	9672	9750	14109	8998	19337
Avpi1	224	141	131	201	127	283
B4galnt3	1	3	0	4	3	0
Bahcc1	816	578	402	727	608	729
Brcc3	1459	964	859	1132	827	1546
Brf1	1227	816	691	996	808	1411
Bzw1	4202	2809	2421	3776	2301	3900
Cd3eap	514	353	356	481	323	708
Cd8a	13	24	4	22	5	9
Cd96	2	8	2	12	2	6
Cdh13	5039	3934	2785	5337	3467	5019
Cebpg	2285	1295	1462	1981	1319	2804
Ces1b	1	1	0	1	0	0
Col15a1	3466	2328	1546	3913	1203	4268
Copg1	2628	1735	1404	2443	1523	2800
Copz2	1001	708	518	874	611	1141
Csf2ra	231	153	123	171	99	262
Ctla2b	185	94	117	116	133	244
Cyb5r4	1356	856	940	1288	704	1407
Dak	248	144	120	254	130	231
Dctn4	3270	2278	1916	2813	1958	3135
Diras1	1	1	0	0	1	3
Dnah1	2	2	0	3	0	4
Dynlrb1	3773	2913	2398	3027	2427	4249
Enoph1	349	248	226	304	201	362
Exosc1	393	352	263	342	248	486
Extl3	3089	2122	1767	3080	1901	3204
Eya3	2082	1525	1180	1931	1209	1887
Fam26e	131	81	64	140	60	146
Fam83d	10	5	2	6	3	16
Flad1	2355	1442	1277	1977	1163	2254
Foxd1	5	2	1	4	0	2
Fry	9439	6065	4142	8114	4874	6605
Gcn1l1	2373	1537	996	2092	1575	1893
Ghitm	31436	19452	18549	25881	17450	29079
Gls2	24	15	13	21	13	14
Gm19510	0	2	0	2	1	1
Gm4814	1	5	4	3	3	2

Gm7168	0	2	0	2	1	1
Grk6	888	539	414	744	459	974
Hepacam2	25	9	2	16	5	7
Hist1h4c	1	1	0	3	0	1
Hist1h4f	1	0	1	1	0	0
Htr1d	1	1	0	1	0	0
Ighmbp2	204	105	104	153	126	216
Insl3	83	27	26	35	49	82
Kcnj2	4117	2909	2550	3788	2657	3406
Kdelr3	208	122	122	175	76	223
Kdm3b	2664	1824	1403	2303	1698	2857
Kdm7a	2697	1901	1700	2237	1701	2744
Knstrn	44	19	22	42	16	54
Ksr1	331	218	156	330	171	349
Lactb	1126	784	768	899	705	1262
Lars	3318	1971	1732	2473	1882	2936
Luc7l3	5494	4018	3564	4278	3362	6381
Mafb	563	322	320	532	217	517
Magee2	1	0	1	1	0	0
Marf1	4068	2762	2228	3144	2388	3989
Mir6359	0	2	0	0	1	0
Mir6943	1	1	0	1	0	0
Mir744	1	0	1	1	0	0
Mlf2	5409	3394	2854	4589	3118	6035
Morn3	1	1	0	1	0	0
Muc19	1	4	8	2	4	5
Nelfb	1351	835	659	1207	727	1399
Neu4	0	2	0	0	1	0
Nicn1	1133	726	613	955	620	1014
Oas1b	141	56	47	89	58	155
Ociad2	170	96	100	157	103	186
Pard3b	1040	793	578	954	615	958
Parp6	1157	737	698	937	669	1132
Pdcd6	1333	789	856	1236	769	1588
Pde11a	9	4	7	5	16	6
Pdzd2	7503	5007	3305	6974	4632	6543
Phf5a	617	396	358	547	345	771
Pigg	1011	592	522	836	570	737
Pmvk	388	272	274	307	216	478
Pou2f3	18	4	2	8	3	8
Ppil6	63	33	25	51	32	68
Prr16	5	4	4	2	3	6
Pskh1	1012	609	486	777	549	835
Psrc1	9	11	7	11	5	24
Ralgapa2	3809	2708	3161	2981	3260	3462

Rasa1	2715	1886	1450	2540	1625	2941
Rasgrp4	78	43	36	68	34	48
Retnlg	1	3	2	6	2	2
Rhobtb3	1040	661	585	879	584	932
Rnaseh1	253	150	136	217	150	281
Rpap2	512	397	275	444	360	607
Rpl10	88	56	52	68	35	98
Rpl13	305	159	136	234	129	382
Scml4	57	56	28	63	13	66
Shcbp1l	1	1	0	1	0	0
Shisa4	107	105	88	129	76	190
Sil1	648	420	328	483	323	663
Sirt4	555	295	259	395	311	604
Slc46a1	51	31	31	54	26	66
Slc7a13	1	1	0	3	0	1
Smg1	7349	4105	3607	5729	4322	7392
Snora3	4	2	2	3	5	4
Sptb	8819	6494	4639	7787	5865	5826
Stx11	100	70	61	71	65	94
Tcfl5	6	4	4	2	2	8
Tinf2	532	348	271	443	329	589
Tm9sf4	2180	1462	1126	1887	1206	1858
Tmc1	1	1	0	1	0	0
Tmem115	900	521	470	838	468	934
Tmem241	143	119	90	164	94	180
Tob1	3791	2419	1906	3141	2138	3029
Trappc2l	637	447	388	641	370	774
Tyw5	191	158	127	156	128	209
Ube2i	308	184	165	258	190	335
Ube4b	2950	2099	1575	2395	1726	2867
Ubl4	3899	2560	2372	3478	2199	3680
Usp5	4230	2684	2113	3603	1909	4066
Vps37d	14	7	10	14	7	22
Wwc2	2742	1921	1491	2381	1637	2365
Zfp619	120	81	79	93	104	147
5930430L01Rik	169	125	105	152	144	184
A830080D01Rik	353	231	179	259	177	360
Acvr1c	4	7	6	5	4	6
Kdm4a	2326	1397	1100	2111	1302	2155
Klhl31	7183	5178	4530	6419	4870	8928
Lonp1	7455	4862	3992	5850	4308	6842
Magi2	519	416	294	445	353	450
Mpeg1	528	318	272	550	218	628
Nkd1	77	44	40	60	32	69
Sgca	4280	2972	2166	3905	2600	4490

Sh3yl1	48	52	32	60	35	78
Tiprl	1592	1190	1003	1459	892	1752
Ubr5	12254	8657	7257	9516	8105	11414
1700007K13Rik	2	0	0	1	0	3
Abhd10	680	438	442	633	444	790
Arl8b	5440	3666	3441	4647	3380	5151
Ccdc85c	265	199	160	267	171	176
Ect2l	2	0	5	6	2	4
Efcab12	5	5	4	5	8	7
Gm20125	3	2	1	1	3	0
Gm4013	5	3	5	4	6	9
Kcnmb4	6	2	1	2	1	4
Kctd8	31	25	18	31	14	27
Safb	3442	2414	1782	2723	1991	3326
Slc16a14	2	4	4	9	2	5
Tmc5	47	19	6	19	37	28
Tsen34	1121	594	507	983	552	1055
Wbscr16	1092	564	533	785	500	1000
Zranb2	3297	2048	2029	2519	1864	3523
9330182L06Rik	2	7	2	4	2	3
A330076H08Rik	3	5	5	5	3	3
Csf1r	1248	808	598	1053	506	1262
Dlec1	6	3	1	3	2	3
Dok2	86	46	22	46	32	67
Fchsd2	1379	968	812	1223	834	1257
Fgf9	362	243	254	255	252	377
Fkbp2	379	272	249	289	214	457
Ints8	1112	757	617	944	613	1079
Isca2	1975	1402	1237	1525	1147	2305
Jmjd1c	4129	2898	2474	3509	2542	3958
Mirlet7c-2	0	1	1	2	0	2
Mras	373	246	194	331	192	311
Ms4a6d	143	86	72	107	42	163
Mtg2	1152	611	522	809	532	1029
Parp11	365	190	204	320	200	392
Pbld2	84	35	40	84	42	70
Piwil4	1	0	1	3	0	1
Ptch2	37	21	18	28	23	46
Rad54l2	1321	930	734	1013	854	1220
Rasgef1c	20	15	16	16	15	39
Rps4x	3903	2053	2248	3696	1713	4782
Siglec1	210	148	80	161	54	235
Slc52a2	156	85	93	135	87	192
St3gal3	2663	1775	1668	2351	1556	2596
St8sia6	212	126	102	170	115	250

Tbc1d10b	902	585	403	855	482	879
Tnnt2	295675	255847	194038	276451	245632	395504
Use1	2328	1509	1363	2020	1280	3010
Wdr35	982	656	529	782	568	921
Zfp335	912	575	339	789	508	810
Zfp558	73	57	46	55	51	68
Znrd1as	441	377	278	358	292	506
Zxdc	928	571	504	772	480	857
Rnasek	1838	1409	1109	1674	1186	2381
BB123696	19	13	21	11	23	11
Cacna1a	247	155	130	181	101	277
Cd28	254	170	124	208	189	245
Cdk5rap3	769	482	383	571	492	850
Hax1	220	151	123	173	117	223
Lats1	1662	1043	880	1242	845	1742
Mthfsd	318	219	192	235	164	375
Zfp507	922	639	526	735	580	858
Creb3l1	154	135	112	173	103	235
Mbd3	2294	1265	1156	1877	1223	2415
Ank	12866	8178	6760	11023	6884	10911
Clec4a1	169	132	94	154	78	180
Def6	109	40	49	78	49	120
Dnajc7	4116	2963	2734	3624	2909	5252
Mrpl18	4216	2931	2747	3444	2475	4765
Ncapg	27	12	14	11	2	79
Phip	2623	1646	1276	2047	1558	2838
Ski	4717	3336	2128	4292	2350	3796
Slc1a2	44	20	25	23	24	38
Traf3ip1	501	306	269	382	268	471
Mon2	3924	2470	1839	2996	2035	3544
Bmyc	221	151	96	140	116	217
Gabra3	158	134	74	184	41	147
Hif1a	4361	2899	2164	3473	2421	3908
Lonp2	4553	3035	2557	3806	2601	4285
Otub2	261	143	121	204	108	224
Pira1	23	1	2	16	1	21
Pira11	23	1	2	16	1	21
Rab11b	2659	1717	1480	2215	1682	2945
Rsg1	14	7	8	8	2	17
Rufy3	1333	937	723	1116	794	1217
Sema4f	36	4	10	10	11	20
Tgm2	13949	9144	8263	13553	8776	14751
Zfp119a	153	120	129	147	121	247
Arl6ip4	941	624	506	780	529	1175
Cend1	55	44	24	48	33	76

Ppp1r15b	2132	1277	1145	1851	1187	2315
Slc38a5	3	0	2	3	3	2
Prima1	7	6	2	8	6	7
Dbnl	1261	810	639	1096	709	1378
Ctps2	567	380	302	502	338	645
Kdm1b	1087	710	627	821	716	946
Shisa2	44	32	21	50	17	24
Efcab8	1	0	1	1	0	3
Get4	790	473	456	684	474	877
Tppp2	1	0	1	1	0	3
4930447N08Rik	2	0	0	0	0	1
Bad	403	230	215	360	215	442
Cdk5r2	2	0	0	0	0	1
Clca1	94	95	39	58	48	123
D6Ertd474e	2	0	0	0	0	1
G630071F17Rik	2	0	0	0	0	1
Gja8	2	0	0	0	0	1
Gm11413	2	0	0	0	0	1
Gm16336	2	0	0	0	0	1
Hoxd3os1	2	0	0	0	0	1
Il17f	2	0	0	0	0	1
Lrrc6	0	2	0	0	3	1
Mbp	167	87	91	105	70	180
Mir3068	2	0	0	0	0	1
Mir499	2	0	0	0	0	1
Mir688	2	0	0	0	0	1
Mtpap	1520	990	853	1177	853	1547
Orm2	2	0	0	0	0	1
Sepw1	2940	2251	1793	2792	1918	3606
Slc16a5	2	0	0	0	0	1
Slc8b1	375	202	176	294	206	391
Sub1	2250	1524	1295	1726	1227	2499
Tchp	336	247	198	283	253	397
Thada	529	330	300	448	292	461
Tmed9	2356	1324	1331	2029	1034	2293
Zfp398	272	177	149	252	152	194
AW046200	21	10	14	12	8	23
Echdc1	1565	1037	883	1296	995	1278
Ndufb8	19952	12680	12698	19193	11696	24296
Prpf38b	2127	1696	1291	1778	1348	2339
Pgm2	9870	6032	5118	8353	6008	10020
Bbs1	281	211	156	244	147	290
Nek7	6134	3902	3469	5209	3580	5175
Ash1l	6095	4572	3160	5276	3836	4891
Ces1g	1	0	1	0	1	0

Efna3	1	0	1	0	1	0
Ilk	3480	2290	1857	3322	1889	3829
Lrrtm1	1	0	1	0	1	0
Oas1a	112	55	37	94	24	161
Pcsk1n	1	0	1	0	1	0
Rpl29	390	197	174	271	193	454
Sspo	40	20	18	27	24	48
Tbc1d12	614	470	316	591	408	651
Zfp36l3	1	0	1	0	1	0
Fgfr2	9	16	13	12	20	21
Nanos3	2	3	1	0	4	6
Nubp2	403	219	226	324	251	430
Paics	8573	5795	5125	7525	4713	8590
Gorasp1	1062	612	546	839	554	919
BC006965	0	0	2	2	1	1
Btg1	1354	968	644	1185	824	1453
Dusp11	5182	3381	2535	3758	2805	5057
Jmjd7-pla2g4b	599	355	227	441	247	515
Ppp5c	3404	2398	1795	2862	1958	3183
Rxfp1	37	43	31	40	38	24
Sprtn	416	321	249	367	239	438
Acaa2	28384	16538	15468	21687	17217	32885
Cyb561a3	668	363	316	562	369	636
Gm20743	3	0	2	1	4	3
Iqck	4	4	3	4	5	7
Nif3l1	581	328	329	503	262	537
Pid1	552	303	301	529	248	644
Ptgfr	613	440	269	566	302	469
Slc25a45	150	125	98	137	115	211
Taf12	942	639	561	791	543	945
Usp16	2251	1688	1603	1904	1472	2607
2510009E07Rik	1712	1360	1170	1636	1163	1688
2900009J06Rik	20	8	8	20	7	21
5730455P16Rik	2554	1938	1577	2260	1686	2429
9230112D13Rik	2	0	0	1	0	0
9530068E07Rik	6102	3990	3297	5243	3133	5812
Aass	29	14	10	19	12	18
Abcc5	2797	1693	1423	1836	1465	2942
Alg9	439	290	219	399	218	356
Ap2b1	2996	1975	1504	2608	1478	2758
Arglu1	2785	2008	1678	2099	1911	3772
As3mt	2700	1684	1446	2281	1740	2693
AU022252	323	221	198	351	223	311
Barx2	2	0	0	1	0	0
Best2	2	0	0	1	0	0

Brinp3	2	0	0	1	0	0
C1qtnf6	321	182	200	300	173	390
C2cd2	1801	1158	978	1829	968	1858
Cacng4	2	0	0	1	0	0
Cbll1	801	549	416	694	441	703
Cbr1	992	606	536	868	564	1074
Ccdc147	5	3	8	8	2	4
Cd248	337	322	259	418	212	555
Cdc42se1	979	630	623	977	548	1174
Chmp5	1795	1191	1028	1535	1068	2174
Chtf18	57	37	20	41	27	79
Cml2	2	0	0	1	0	0
Cnga3	144	102	85	127	148	114
Cntn3	7	6	5	11	7	7
Cntn5	119	100	39	106	64	70
Cspg5	10	11	8	6	7	14
Ctsh	1516	1023	822	1217	727	1689
Cystm1	292	204	209	345	201	314
Dnm2	3725	2663	1959	3500	2253	4044
Doc2a	2	0	0	1	0	0
Dpm3	128	96	77	132	84	154
Eif1a	795	550	515	676	461	857
Elfn1	5	5	9	11	2	4
Exosc7	547	344	283	387	310	517
Fbxo22	1876	1354	1236	1676	1096	2063
Galnt6	24	16	18	11	15	30
Gbe1	3793	2720	2352	3425	2335	3615
Gfra1	828	514	360	724	335	704
Glis2	513	322	207	449	179	443
Glrx	706	436	427	619	374	665
Gm10220	2	2	1	3	1	4
Gm19897	2	0	0	1	0	0
Gm20605	1667	973	697	1226	892	1748
Gm826	127	81	97	112	95	176
Gm9731	2	0	0	1	0	0
Gpd1l	3951	2573	2320	3462	2325	3463
Hacl1	493	338	308	431	254	534
Hapln3	8	5	4	6	3	6
Hpd	1	1	0	0	1	0
Ifnar1	1860	1204	936	1599	1126	1909
Kcnj9	2	0	0	1	0	0
Kif1c	37217	24949	19777	31835	21093	31017
Kif26b	115	91	63	106	74	83
Kif3a	1201	876	709	1001	855	1428
Kifc1	20	4	9	14	7	24

Klrb1f	4	8	2	5	2	5
Leng9	112	75	50	98	61	133
Lif	10	5	6	13	5	11
Lig3	1398	907	786	1245	791	1190
Lilra6	21	6	5	25	2	18
Lsm5	71	40	36	51	41	93
Mfap1a	892	679	591	890	544	866
Mgat5	301	245	226	269	251	246
Mia3	4595	3512	2761	3949	3080	4281
Mir3962	90	28	67	42	33	111
Mir7072	2	0	0	1	0	0
Mmp15	4783	2969	1912	3910	2287	3531
Mob1a	755	434	358	649	295	673
Mroh7	19	21	8	8	19	14
Murc	4417	3104	3494	4250	3808	5160
Mzt1	3116	2219	1672	2516	1927	2870
Nin	1395	1034	749	1241	927	1546
Nit1	1007	680	549	865	593	1191
Olfr267	1	1	0	0	1	0
Otogl	5	4	4	3	6	10
P4ha3	5	2	1	4	2	6
Palm3	13	13	10	5	19	26
Pappa	56	59	57	46	34	103
Pard6a	165	79	61	126	85	151
Pdzd9	5	1	2	0	2	4
Pitx2	18	17	20	25	14	13
Plk4	115	76	56	89	61	162
Pnrc2	3778	2493	2283	3385	2241	4624
Prkag2	1684	1292	1001	1551	991	1718
Prkd3	2012	1304	1066	1833	1126	2198
Prmt2	425	310	231	424	293	420
Prss35	7	6	5	17	2	8
Prss45	2	2	1	3	1	4
Ranbp2	6195	4154	3734	5729	3489	5616
Rasgef1a	36	29	26	30	21	40
Rassf6	0	0	2	1	1	2
Rnf151	2	0	0	1	0	0
Rpain	313	236	167	253	227	374
Rspry1	875	569	513	682	504	888
Runx2	74	76	34	67	33	87
Scnn1a	25	15	9	22	18	22
Sel1l	4431	3135	2663	3830	2653	4385
Sf3b3	2802	1731	1495	2534	1361	2462
Smg8	582	374	287	520	345	592
Snora62	1	1	0	0	1	0

Snx32	389	236	191	329	213	430
Stap1	11	4	10	6	4	13
Tbpl1	884	528	511	826	498	956
Tbx15	31	8	21	14	28	36
Tekt1	9	2	1	3	5	9
Tesk2	175	96	95	118	80	179
Tespa1	7	6	2	5	1	7
Tex35	0	4	1	1	3	5
Tmem164	2248	1426	864	2467	1107	1744
Tmem258	890	527	521	713	577	988
Tmem39a	436	250	212	356	183	395
Trmt112	155	76	63	115	92	140
Ubash3b	109	70	59	105	63	125
Ugt1a6a	76	38	32	57	30	101
Unc5c	25	23	8	42	14	18
Vps11	1329	743	677	1007	768	1431
Vta1	825	497	432	715	485	849
Xrn2	1929	1480	1090	1615	1288	2458
Ybx3	7091	5036	4210	6102	4268	6926
Zfp316	376	257	172	335	254	348
Zfp691	770	523	463	657	492	663
Zmynd8	3342	2384	1819	2572	1989	3449
Zp3	1	1	0	0	1	0
Alyref2	133	96	83	107	82	130
Kif23	113	62	50	61	30	139
Card14	13	5	7	10	7	17
Cytl1	243	254	190	227	163	352
Nup43	161	119	80	124	79	169
A430078G23Rik	5	0	2	7	1	3
Abhd6	388	269	255	306	249	400
Ahcyl2	1546	824	835	1259	813	1233
B9d1	93	47	42	80	47	96
Clcn7	1644	984	842	1467	925	1631
Cyp4f14	5	4	9	6	5	17
Eif2ak3	593	377	359	533	348	719
Esyt3	2	0	2	1	1	5
Gm4285	115	55	85	97	71	158
Gsta1	8	1	4	2	6	11
Hsf5	0	2	0	0	2	2
Lcorl	478	240	223	325	232	400
Lrrc61	413	267	223	308	242	382
7-Mar	1889	1361	996	1560	1348	2002
8-Mar	2800	1683	1560	2360	1540	2428
Meox1	471	305	241	479	203	582
Napb	141	107	64	126	71	197

Nus1	3835	2263	2445	3181	2182	3702
P2rx6	114	54	41	52	88	106
Ptov1	3321	1996	1457	2714	1742	3376
Ramp1	274	174	185	366	121	327
Ripk1	676	472	384	598	399	845
Scarna13	2	1	2	1	4	3
Sh3rf1	636	312	295	549	337	556
Slc26a10	1356	696	485	761	816	1306
Spidr	178	119	75	160	108	175
Sypl	6017	3617	3329	5269	3443	6564
Thumpd2	139	104	103	101	91	168
Vstm4	347	219	191	318	184	418
Wdr82	3255	1993	1797	3030	1570	2863
Xkr6	5	1	4	9	3	3
Zfp444	255	129	128	229	130	254
Zranb1	2297	1582	1287	1879	1287	2356
Sh2b2	19	17	10	29	11	21
Acsbg1	12	9	6	14	9	13
Akap10	1065	666	569	927	541	958
Lrrfip2	6271	5010	3994	4730	4005	7633
Rab1b	5050	3073	2509	4294	2924	5082
BC030336	1658	1033	949	1456	998	1806
Celf2	6123	3839	2990	4673	3165	5735
D11Wsu47e	233	154	128	182	149	293
Fam110a	134	69	55	128	69	112
Fgr	11	10	5	14	8	13
Klhl42	608	375	333	561	367	617
Lamb2	18100	11598	7897	15320	8757	14207
Nars2	811	556	490	691	454	839
Nfil3	580	341	257	489	310	344
Pex5l	47	29	16	31	29	55
Polr3k	932	553	560	690	520	959
Psmc6	5471	4051	3671	4401	3601	6074
Rnf150	1857	1433	1327	1810	1303	1731
Scn4b	1314	625	1172	602	893	1698
Slc35e4	224	111	113	192	123	219
Snhg5	188	166	104	176	117	283
Snord88c	2	1	2	3	1	4
Hibadh	13896	9025	8317	11189	7802	14277
Ube2a	1935	1308	1267	1707	1008	2212
Thnsl1	576	368	320	450	338	523
2310033P09Rik	329	285	234	315	250	484
Atxn7l1	642	479	355	497	411	625
Bace1	1605	1056	1011	1392	992	1491
Cct7	5408	3513	3142	4763	2702	5510

Egf	573	505	363	346	336	865
Harbi1	441	262	245	368	229	388
Ivd	11229	6612	6194	9692	5943	9334
Ngfr	20	7	8	12	6	14
Slc35b2	789	438	318	683	382	735
Slc9a8	1476	870	686	1110	772	1249
Ube3a	3965	2684	2388	3290	2376	3934
Ccdc96	39	34	17	36	20	31
Dld	28387	19876	18101	23935	18221	27058
Lhpp	544	361	313	463	305	510
Wdr53	262	153	134	217	145	297
Syne1	6231	4485	3614	5296	4745	6617
A230057D06Rik	2	1	2	4	1	3
Ankfy1	2055	1345	1015	1746	1041	1857
Ascc1	914	574	479	772	501	1061
Cdc42bpg	1188	664	536	844	605	1011
Elp5	1216	779	682	1005	639	1211
Espl1	57	20	20	32	18	79
Itga2	23	12	12	14	13	17
Mecr	654	435	395	535	367	677
Penk	229	116	225	189	182	380
Taf7	715	451	419	490	426	747
Zbtb48	216	145	94	180	118	252
Zfp740	2536	1437	1287	1966	1437	2713
2310039L15Rik	80	48	50	73	38	121
Dync2li1	194	99	100	172	99	205
Hvcn1	57	29	22	45	24	68
Nupr1l	6	2	7	2	7	14
Tra2b	1449	951	906	1154	767	1674
Yy2	304	183	145	207	149	298
Apold1	534	268	464	302	470	869
Pcdha4-g	517	334	245	481	276	542
Zfp956	99	79	64	98	56	159
2700081O15Rik	1284	746	612	1095	680	1298
Mn1	813	456	393	785	344	551
2610027K06Rik	5	8	9	4	7	9
4930506M07Rik	259	224	146	226	146	292
Fbxo31	4489	2444	2037	3429	2687	4040
Lamp2	12751	8378	6859	11088	6806	11491
Trim30b	12	4	3	9	1	6
Wfikkn1	10	3	6	8	7	11
Zfp652	700	494	429	489	545	566
Traf6	1053	625	593	916	613	1135
Mpc1	237	156	139	169	131	256
Wfdc3	7	0	2	2	5	6

Hadha	73069	46815	41621	55719	43662	66639
Hat1	1173	794	807	1073	644	1300
Homez	227	148	121	171	120	223
Itpr1	2698	1816	1530	2424	1629	3053
Map4k1	47	37	18	38	34	55
Pm20d1	771	653	526	745	609	981
Slx1b	509	339	229	396	336	519
Ubr2	10148	6657	4982	8435	5517	8562
Anapc7	1607	1051	897	1378	1034	1714
Meg3	676	515	395	438	356	914
Zfp580	100	69	42	91	51	120
Lck	17	23	4	30	9	22
Prelid1	779	506	420	662	353	812
Srpr	3871	2663	2089	3457	2059	3631
AA388235	39	23	32	43	34	42
Apbb1ip	345	277	188	354	136	373
Bbx	1208	801	609	967	788	1270
Car13	37	32	18	27	11	50
Cbr4	1551	1022	995	1350	990	1916
Dhx35	847	576	402	683	416	824
F630028O10Rik	94	77	46	105	39	72
Fmo3	0	1	2	1	0	1
Gtf2a1	1395	827	760	1096	867	1505
Havcr2	17	18	16	22	6	23
Ints5	451	250	208	357	218	347
Lyrm2	1106	771	675	881	659	1196
Megf6	123	113	55	101	65	130
Mrps22	925	731	642	839	592	1054
Nup35	104	82	53	89	47	110
Pcdhb21	23	12	9	18	12	27
Pdia5	187	104	82	97	101	171
Pex12	474	258	264	363	339	452
Psmc5	3893	2463	2432	3625	2206	4767
Rab24	1379	861	729	1068	868	1518
Sec22a	524	387	284	459	275	532
Snora28	26	30	34	33	25	32
Srpk3	1429	917	692	1177	697	1335
Ttll5	801	681	462	801	643	829
Vegfb	10681	7123	5353	8561	5543	10341
Vpreb3	0	1	2	1	0	1
Ywhab	6693	4235	3463	6106	3549	7433
Zfp622	1003	709	588	886	627	1219
Cnksr3	1332	709	760	906	805	1156
Cnnm4	347	228	206	309	201	295
Wdr72	43	43	24	59	9	42

Fam96a	2094	1246	1248	1632	1160	2094
4930414L22Rik	82	41	51	67	54	94
9930021J03Rik	1921	1442	1101	1706	1208	1762
Cep164	565	380	272	439	372	610
Ddx39	547	270	294	476	279	569
Bcas3os2	3	0	0	2	2	1
Cdkl3	301	223	222	271	206	304
Ifrd2	1339	781	648	1003	710	1159
Lbx2	15	5	3	13	8	9
Lsm14b	2696	1824	1479	2383	1429	2587
Nup98	2067	1459	1185	1892	1198	1882
Osbpl11	1691	1051	917	1535	845	1487
Osbpl5	763	489	343	627	324	725
Tmod4	678	452	512	632	482	888
Ubash3a	4	2	4	4	3	1
Mitd1	167	95	143	171	103	251
Atg13	3419	2240	1769	2776	1881	3194
Cstl1	0	1	1	0	1	3
Dhx33	855	578	470	706	497	795
Erlin2	1370	932	775	1211	881	1500
Zhx2	1913	1372	960	1755	1077	1540
2310067B10Rik	5892	3901	2459	4493	3093	4898
5430419D17Rik	6	6	5	6	6	12
Creb3l2	909	624	456	810	520	700
Gns	5714	3497	3101	4638	3239	4916
Krt7	6	3	1	4	2	2
Lztr1	1638	985	743	1287	775	1463
Med31	287	193	147	197	160	293
Rpgr	346	254	189	274	195	359
Six1	107	75	53	97	70	118
Snord99	8	3	3	9	2	9
Tacc2	33256	22279	16325	26176	19684	26643
Tbc1d7	812	508	431	703	427	967
Tmem200a	4	3	0	3	1	7
Tmod3	4370	2639	1917	3786	2106	4605
Tpra1	957	583	476	762	451	923
Aven	692	481	446	634	375	734
Cers2	1913	1250	1072	1747	1117	2116
Dtx1	32	37	25	40	24	28
Gm5860	137	107	85	114	76	154
Optn	4162	2985	3063	3074	3160	4394
BC048609	5	1	0	3	0	1
Vcpip1	1176	743	615	990	618	1062
Tmem14c	2394	1505	1500	2199	1485	2696
Tceal3	8	3	3	3	3	6

9030624J02Rik	1405	1043	785	1382	839	1607
Abca1	3334	2248	1845	2548	1825	3626
Agtpbp1	10226	6942	5025	8018	6214	8300
AI847159	2	0	0	0	1	0
Akap3	2	0	0	0	1	0
Alg13	348	220	179	251	181	348
Atp6v1f	898	509	514	786	497	992
BC005561	549	396	310	458	358	682
C730036E19Rik	51	30	18	28	52	36
Catip	31	36	26	24	32	71
Ccdc137	397	251	204	302	222	354
Cdc40	1698	1218	950	1428	972	1739
D130040H23Rik	44	16	20	31	26	42
D6Wsu163e	535	334	282	431	286	482
Ddr1	1348	669	622	1175	760	1063
Dlx1	11	14	6	11	6	28
Dnajc15	2241	1852	1593	1951	1439	2834
Dnajc24	187	115	116	179	111	220
Exog	185	97	92	150	67	168
Folr4	0	2	1	1	0	1
Galnt16	205	128	165	181	128	336
Gm19522	369	167	169	220	296	276
Gm5294	0	2	0	3	1	0
Ifi44l	0	2	1	1	0	1
Insig2	3088	1996	1914	2769	1703	3060
Ipo9	2199	1477	1135	1799	1172	2138
Ism1	30	27	28	29	27	57
Macc1	2	0	0	0	1	0
Med13l	2234	1536	1118	1980	1226	1874
Mir7081	2	0	0	0	1	0
Mir762	0	2	1	1	0	1
Mir8116	6	1	2	2	5	6
Nav1	2894	2191	1506	2645	1728	3411
Nupr1	120	132	83	149	59	147
Pip4k2b	1647	945	834	1354	855	1347
Pkd1	4314	2499	1760	4192	1940	3923
Ppfia4	138	75	62	118	81	128
Ppp1ca	8585	5407	4637	8018	4606	9466
Prss30	0	2	1	1	0	1
Pyhin1	48	47	25	77	10	89
Rsph3a	271	138	126	176	150	202
Rtca	791	565	483	706	486	1023
Sh3gl2	48	38	42	54	21	58
Slc22a5	1248	678	651	991	643	1426
Smchd1	2067	1373	1069	1766	1138	1818

Sorcs1	31	6	17	12	19	41
Spdl1	17	13	15	8	6	31
Stx7	2734	1875	1820	2483	1588	2905
Tbx21	5	5	2	7	0	3
Tmeff2	19	10	4	11	14	17
Tmem229a	2	0	0	0	1	0
Zbtb26	455	271	228	360	249	363
Zfa-ps	2	0	0	0	1	0
Zfp324	214	116	110	174	111	154
Hesx1	0	2	2	4	1	2
Etfdh	31721	20517	20257	24464	19491	32284
Gpr83	2	1	0	1	3	1
Fam131c	14	3	13	12	3	13
Mgat1	2391	1298	1229	2170	1187	2423
Slc28a1	8	3	3	6	1	5
Tirap	610	414	280	581	319	646
Tmem185b	618	391	360	545	307	606
2610018G03Rik	28	30	21	32	19	27
2810001G20Rik	334	168	182	273	145	293
Acad8	1945	1214	1057	1615	969	1931
Ap3d1	3858	2677	1996	3332	2101	3568
Cd14	201	132	88	143	75	220
Cdc42ep3	1782	1160	1032	1659	1100	1885
Chfr	926	583	455	765	511	1030
Crem	512	303	258	477	256	548
Dcaf13	803	599	484	683	462	885
Emc2	5395	4269	3792	4731	3430	6478
Epn3	843	523	411	650	619	747
Eva1c	69	47	21	45	23	67
Fdxr	299	186	122	253	168	290
Gm715	7	1	5	2	2	7
Hbq1a	0	3	0	0	0	2
Ift81	1595	1047	890	1272	1439	1265
Ift88	396	320	247	363	236	426
Jrkl	629	433	377	527	376	616
Kcna2	406	281	199	265	215	446
Kdelr2	1442	928	785	1177	743	1460
Lilrb4	179	126	93	105	79	234
Lrp1	7476	5195	3644	7938	2956	6144
Map2k1	2074	1305	1075	1761	1052	1902
Mapre2	6211	4129	3562	5529	3384	5833
Mir719	70	53	42	56	48	59
Myom3	234	172	203	221	155	422
Nek2	22	13	4	14	5	36
Ogfod3	533	347	284	418	281	528

Pepd	865	531	428	738	398	785
Poli	112	104	64	75	73	139
Pon3	816	623	541	746	593	693
Ptpn14	2047	1708	965	1865	1195	1814
Rfc1	2448	1850	1478	2049	1612	2443
Rpe	720	467	446	651	366	737
Scarletltr	1	0	2	0	0	2
Sec11c	613	418	423	516	352	702
Shank2	11	7	6	14	8	10
Slc22a21	39	19	21	23	15	40
Socs4	378	226	179	291	216	274
Sumo2	11	14	11	11	15	23
Syne3	301	197	170	237	191	252
Tcf20	1280	945	702	1055	854	1094
Tet1	477	344	298	396	309	465
Thbs1	401	279	195	439	220	384
Timm23	134	116	91	123	85	148
Tm6sf1	403	240	236	338	191	578
Tmem215	4	2	5	4	5	7
Ttc30a1	265	187	155	212	152	273
Usp46	1478	1039	909	1369	882	1391
Zfp599	10	19	14	3	15	22
Zhx1	2343	1750	1408	2052	1478	2309
Slc7a4	75	38	23	57	41	65
Gnb1	6549	4193	3789	6248	3705	7340
Hnrnpl	5827	3454	3060	4475	3245	6912
Mir24-2	1	1	1	0	0	2
Mrpl14	1167	637	631	941	641	1278
Rab43	800	479	441	743	424	877
Zfyve28	40	22	30	35	16	42
Ppie	519	317	296	402	254	550
Sarnp	428	350	364	392	295	551
Slfn9	116	65	44	72	46	174
Btf3l4	405	302	224	379	260	469
Spic	12	8	2	5	6	8
1810019D21Rik	14	11	12	9	11	15
Hmmr	29	17	14	25	4	61
0610009L18Rik	110	48	66	100	55	124
1700110C19Rik	12	10	9	7	4	19
1810013L24Rik	3359	2120	1906	2656	1880	3363
2610203C22Rik	46	12	21	32	22	45
2700029M09Rik	1062	904	603	875	661	1215
4931406H21Rik	7	6	7	7	13	7
5031426D15Rik	307	240	188	189	179	413
A430035B10Rik	55	24	24	36	28	65

A630001G21Rik	72	51	43	44	52	118
A830052D11Rik	1	1	1	0	1	1
Acmsd	0	1	2	0	1	1
Adprh	1396	805	732	1273	709	1476
Agpat5	1196	875	786	1178	685	1218
Ak8	5	0	0	0	0	3
Aknad1	1	1	1	0	1	1
Ap4b1	1042	745	677	925	714	1298
Apoo	1164	684	769	987	685	1504
Atp2b2	27	22	21	29	31	28
Atp6v1g1	2211	1360	1410	1966	1410	2500
AW549877	3831	2638	2367	2908	2501	3920
B3galnt1	141	99	66	105	60	152
Bmpr2	9182	6308	4480	8372	5567	9171
Cd37	54	37	31	33	18	75
Cdk5rap2	616	457	315	490	365	593
Celf1	5993	4222	3529	5153	3666	5661
Chrdl2	1	1	1	0	1	1
Cobll1	7184	5006	3657	5582	4796	5349
Cog3	1429	973	775	1193	780	1417
Cstf2t	1949	1245	1072	1839	967	1706
Ddx41	622	361	286	494	345	640
Dmtn	192	148	95	161	99	191
Dram2	4881	3169	3058	3845	2844	5246
Elavl3	8	6	3	6	4	5
Elovl4	3	7	10	11	2	6
Eps15	3353	2208	1846	2780	1865	3261
Ereg	3	6	2	6	4	6
Ezh2	327	201	209	292	155	339
Fat3	46	24	25	43	19	30
Flcn	3015	1566	1252	2033	1628	3010
Gabrr2	75	31	23	32	72	40
Gatsl2	1089	656	497	1027	657	869
Glrx2	1431	997	877	1252	835	1465
Gps2	909	734	478	679	575	977
Grm3	4	2	1	2	3	0
Gtpbp2	2816	1618	1342	2315	1563	2754
Gucy2e	7	8	6	11	3	5
Hras	1266	767	752	1167	733	1384
Hsf2	1077	753	702	1015	633	1067
Inpp5a	16806	11217	10031	14136	10185	15523
L3mbtl3	438	263	212	387	208	340
Lsr	36	21	20	24	25	49
Mcfd2	2982	1643	1715	2378	1370	3088
Mir6936	0	1	2	0	1	1

Mir7078	2	0	1	1	3	1
Mmp23	221	146	133	233	96	310
Mri1	323	182	154	200	180	422
Mthfd1l	207	100	82	137	71	187
Myd88	560	316	326	466	248	568
Nckipsd	452	298	225	420	244	495
Nphp4	16	13	6	14	8	25
Nphs2	1	0	2	1	0	1
Nt5c3	3233	2123	2114	2719	2069	4173
Olfml2a	113	83	39	127	48	119
Pdik1l	410	282	224	384	259	437
Pdzd3	84	35	50	60	69	73
Plin4	14161	8192	6509	11299	8648	12840
Pmis2	2	2	1	0	4	4
Ppat	1660	1313	884	1425	1040	1585
Ppp1r9a	2068	1498	1113	1351	1268	2310
Prorsd1	666	451	384	537	338	740
Psmd12	5340	4046	3609	4671	3427	5890
Rab10os	203	134	95	150	87	213
Rnf19b	966	637	555	743	576	968
Rnf44	2495	1590	1278	2255	1430	2531
Rpl22	4964	3676	3143	4271	2907	5601
Rsrc2	2916	2268	1929	2393	1990	3248
Scaper	1570	1176	868	1362	1002	1402
Serpinb6a	4769	3293	3127	4710	2824	6038
Slc25a53	11	13	15	16	12	26
Snord16a	5	0	1	2	3	4
Stx12	2955	1916	1697	2552	1776	3371
Syk	2227	1674	1352	2007	1174	2566
Synj1	1887	1243	1008	1599	1160	2053
Taf15	1987	1447	1332	1565	1280	2291
Tbc1d32	553	398	326	460	345	533
Tlr12	5	0	3	7	0	6
Tmcc1	2442	1680	1194	2027	1432	2006
Tmco6	145	85	51	115	77	143
Tmem176a	257	163	133	229	114	350
Tmem86b	93	63	35	64	43	83
Tnmd	0	1	2	0	1	1
Tnpo2	1434	856	725	1235	826	1416
Trex1	420	290	187	336	272	438
Tsc22d2	2061	1280	1185	1717	1119	1992
Ube2u	1	1	3	2	3	3
Wdr8	270	137	121	225	146	247
Yipf4	2969	1880	1638	2367	1637	2924
Zcchc11	1798	1395	994	1429	1205	1720

Zdhhc2	193	108	114	149	92	191
Zfp189	472	263	245	429	279	432
Zfp51	629	503	382	503	426	662
Zfp788	1100	673	693	952	609	1447
Zfr	4724	3573	2895	4219	3056	4532
Nme6	363	236	202	303	188	352
Ache	144	90	62	124	77	156
Cbr3	68	58	36	82	45	74
Gabarapl2	481	275	341	459	312	554
Itm2b	28407	18689	15490	25946	15582	33664
Nprl2	616	400	327	521	316	581
Slc8a1	20803	15291	14125	19047	14610	18108
Rpl13a	7385	4961	4199	6359	4143	9409
Dpysl5	18	12	5	7	14	8
Itgax	45	22	9	38	3	28
Fam53c	1220	613	567	1070	622	1068
Mlx	887	590	499	777	480	843
Srsf7	2836	1700	1605	2322	1384	2920
Ss18l1	281	188	159	236	160	257
Rbm12b1	383	227	234	320	184	394
Taf1d	475	372	406	341	324	696
Afg3l2	8554	5591	5055	7100	5015	8237
0610011F06Rik	272	203	161	257	140	281
1110004F10Rik	1736	1180	1028	1572	1003	2101
1110006O24Rik	5	7	7	5	8	4
1190007I07Rik	272	186	212	271	182	379
1600002D24Rik	1	0	0	0	0	2
1700001J03Rik	10	12	1	9	7	15
1700007B14Rik	1	2	0	1	1	0
1700007L15Rik	26	15	24	30	20	34
1700012D01Rik	40	30	32	40	33	54
1700012I11Rik	0	1	0	0	1	1
1700030J22Rik	11	10	9	11	7	11
1700047M11Rik	17	8	3	4	10	9
1700052K11Rik	142	113	111	144	108	198
1810008I18Rik	0	2	1	1	2	2
1810021B22Rik	53	31	24	32	47	49
1810037I17Rik	1091	976	756	1004	736	1348
1810058I24Rik	2265	1622	1352	1951	1501	2689
2010005H15Rik	1	0	2	0	2	0
2010107G12Rik	7	0	4	2	5	9
2010300C02Rik	41	31	24	42	36	38
2010320M18Rik	297	157	148	213	182	309
2200002D01Rik	20	13	13	19	16	23
2210018M11Rik	1341	1086	644	1152	827	1186

2510039O18Rik	1363	700	675	1118	732	1270
2610034B18Rik	574	404	315	548	371	582
2610306M01Rik	187	114	109	118	86	235
2610528A11Rik	1	2	0	0	0	2
2700060E02Rik	2866	2167	1872	2457	1786	3331
2700062C07Rik	169	133	128	152	100	210
2810408M09Rik	260	149	146	252	149	257
2810433D01Rik	0	5	1	5	1	4
3010033K07Rik	1	1	3	2	0	2
3110007F17Rik	7	3	7	10	4	9
3110062M04Rik	201	142	116	165	127	260
4632415L05Rik	832	550	405	724	443	690
4833411C07Rik	15	8	12	10	16	19
4921524J17Rik	973	695	540	810	571	977
4930413G21Rik	3	2	3	2	3	7
4930447C04Rik	7	13	9	3	7	18
4930505A04Rik	2	1	2	0	1	3
4930506C21Rik	4	0	5	1	1	6
4930525D18Rik	1	0	2	3	0	2
4930550C14Rik	16	7	9	11	7	12
4930562F07Rik	1	0	0	0	0	2
4930563D23Rik	2	0	2	2	0	1
4930563E18Rik	1	1	3	0	1	3
4930570G19Rik	13	11	8	18	3	24
4931423N10Rik	0	1	2	1	1	3
4933413G19Rik	0	3	2	1	2	1
4933427G17Rik	2	0	1	1	1	3
4933433G15Rik	3	0	6	2	2	4
5031434C07Rik	4	4	3	3	6	6
5430435G22Rik	257	198	138	234	116	286
5730522E02Rik	1	1	1	2	0	3
5830428M24Rik	1	2	0	3	0	2
5930412G12Rik	2	1	0	1	2	2
6430584L05Rik	32	13	7	16	13	18
8430423G03Rik	1	0	0	0	0	2
8430431K14Rik	93	65	59	41	73	172
9330117O12Rik	2	1	1	0	1	2
9330159M07Rik	108	74	73	97	69	148
9530082P21Rik	434	273	247	256	351	518
A230009B12Rik	4	4	2	4	6	4
A230072E10Rik	9	6	2	4	4	7
A230073K19Rik	0	0	1	1	1	0
A330074K22Rik	12	7	6	4	12	5
A330093E20Rik	0	0	1	0	1	1
A430033K04Rik	341	239	203	304	191	342

A830082N09Rik	141	84	83	113	78	127
Aaas	535	343	316	436	287	557
Aacs	135	83	73	121	67	109
Aard	4	0	2	6	1	2
AB041803	17	13	8	10	15	24
Abcb8	3076	1660	1362	2379	1670	2842
Abi1	1962	1186	984	1647	1021	2171
Abi2	1130	705	613	968	641	1235
Abi3bp	717	540	325	609	368	695
Abr	2058	1392	1025	2007	1173	1980
Abt1	427	311	235	386	207	456
Acaa1a	1481	781	716	1145	753	1569
Acaa1b	0	1	0	0	1	1
Acadm	52540	33561	32236	41297	32184	51640
Acap2	2462	1498	1096	1897	1335	2616
Acin1	4817	3633	2637	3996	2999	4716
Acot2	2238	1092	1258	1463	1511	2340
Acot6	72	43	29	53	37	51
Acsf2	1006	665	683	726	772	1319
Actr1a	4978	3043	2632	4524	2801	5010
Adam1a	162	92	77	100	79	160
Adam4	13	11	9	13	9	22
Adamts5	2230	1257	1194	1697	1072	2909
Adcy4	1625	940	671	1387	837	1465
Adprhl1	16792	10905	10726	13192	10950	16538
Adra1a	1078	588	504	796	659	645
Adrbk1	3178	1942	1606	2589	1685	2848
Adrbk2	1275	1022	849	1190	1013	1496
Aes	22237	14434	11578	19773	12456	24343
AF357359	5	6	4	4	5	12
Afap1	1343	892	703	1104	780	1208
Aff2	90	124	54	121	70	77
Aggf1	1663	1146	1043	1513	955	1701
AI197445	2	0	1	1	1	0
AI429214	78	59	34	68	47	93
AI464131	613	455	383	594	385	561
AI507597	1	1	2	0	0	3
AI854703	74	49	24	57	34	52
Aimp1	2317	1496	1235	1785	1258	2361
Ak6	635	402	389	504	378	641
Akap11	3268	2323	1992	3006	1968	3169
Akap13	6307	4596	3160	5436	4616	5997
Akirin2	2079	1219	1176	1797	1127	2325
Akt1s1	2044	1256	972	1750	1143	1996
Aktip	3530	2525	2204	3009	2120	3814

Aldh2	12055	7164	5891	10201	6861	11721
Alg11	606	357	295	474	354	625
Alkbh5	5913	3466	3007	5028	3136	4638
Alms1	232	181	124	198	142	222
Ammecr1l	1839	1079	848	1489	1002	1794
Amotl2	3352	1928	2079	2264	2135	4487
Amph	68	48	43	66	40	91
Angel1	275	182	157	216	141	293
Ankhd1	3764	2875	2171	3333	2444	3444
Ankrd11	6616	5164	3822	5487	4497	6400
Ankrd27	800	493	395	682	442	850
Ankrd34b	1	0	2	1	1	0
Anxa1	1242	723	681	1161	544	1527
Aoah	68	40	34	65	37	71
Ap4m1	284	162	144	198	168	337
Ap5b1	63	58	26	71	42	65
Ap5m1	703	492	383	606	395	679
Apex1	513	324	344	497	314	597
Api5	2884	1894	1701	2600	1566	2819
Apoa2	11	11	1	9	4	19
Apobr	47	39	31	43	25	53
Apoh	11	0	5	7	5	10
Apol6	18	8	12	23	8	18
Apol7c	2	1	0	2	1	2
Apol7e	2	1	0	1	0	1
Appbp2	870	553	506	734	491	831
Aprt	261	170	116	220	103	247
Arf2	1234	768	725	1263	666	1303
Arf6	1909	1285	1148	1864	1072	2237
Arhgef12	14169	10505	8715	12680	9489	12546
Arhgef26	141	83	61	102	70	174
Arhgef28	554	372	276	444	352	606
Arhgef4	15	7	6	3	5	18
Arid1b	2319	1560	1224	2084	1519	2203
Arid2	2466	1557	1118	2088	1462	2246
Arl14ep	756	448	385	606	419	838
Arl3	1704	1220	984	1512	1117	1925
Arsg	81	36	42	51	53	87
Aspdh	19	11	14	17	6	21
Asphd2	19	3	14	14	8	11
Ass1	103	50	43	76	51	107
Atad3aos	11	17	8	8	13	13
Atf4	3025	1965	1867	2752	1649	3066
Atg4a	235	148	143	179	158	291
Atmin	1438	837	733	1190	746	1215

Atn1	2241	1462	993	1913	1400	1955
Atp13a4	1	1	1	1	2	2
Atp1b1	15834	10861	10812	13893	9903	16209
Atp1b3	2732	2038	1711	2472	1546	3101
Atp5s	1273	888	760	957	796	1387
Atp6ap2	1258	706	689	1061	529	1293
Atp6v0d2	4	8	4	9	4	10
Atp6v0e	1070	688	629	851	576	1172
Atp8b1	657	407	351	608	388	636
Atxn7l3b	2119	1350	1274	2061	1120	2517
AU041133	133	106	95	121	83	150
Auh	3853	2517	1868	2957	2080	3639
Auts2	1228	805	803	930	891	1123
Avp	1	0	0	0	0	2
Avpr1a	66	45	35	77	32	74
AW551984	15	9	3	22	5	9
AW822252	0	1	2	0	2	0
B230312C02Rik	27	24	14	22	14	30
B3galt6	158	60	77	115	65	115
B3gnt2	1011	646	620	913	612	1159
B3gnt8	83	64	25	67	32	73
B3gnt9	123	102	68	149	64	150
Bach2	343	218	136	313	149	373
Barhl1	1	0	2	1	1	0
Batf	20	7	3	8	8	22
Batf2	35	18	15	28	24	31
Bax	446	246	225	363	243	472
BB557941	0	0	1	1	1	0
BC004004	7401	4254	4571	5787	4446	6982
BC017158	277	156	141	213	172	288
BC018242	239	111	73	122	143	219
BC021891	131	74	74	86	100	147
BC048679	1	0	0	0	0	2
BC049352	1	0	2	0	4	1
Bckdhb	2768	1893	1567	2310	1490	2977
Bcl3	97	65	71	91	56	96
Bcl7c	191	106	96	172	100	194
Bcmo1	1	3	2	2	1	2
Bean1	53	43	38	55	21	65
Bex4	3	4	2	2	2	4
Bfsp2	22	9	12	10	11	20
Bicd2	7598	5215	4562	6752	4487	7361
Blnk	71	37	53	37	27	109
Bloc1s5	628	433	408	595	417	736
Bmp1	1967	1350	1019	1659	1090	2394

Bmp2k	507	300	240	417	242	441
Bnip1	664	373	413	507	349	694
Bpifb3	1	0	0	2	0	0
Brat1	319	182	143	260	158	343
Brd4	2531	1912	1294	2404	1611	2613
Brsk2	5	2	7	6	2	5
Brwd1	3381	2171	1757	2610	1955	3125
Btrc	1098	819	679	1056	699	1303
Bud13	338	241	214	261	214	370
C030034I22Rik	108	57	54	68	72	121
C1galt1	597	374	314	534	355	599
C1qb	1094	755	586	899	497	1268
C1qbp	5614	3605	3752	4520	3227	6401
C1ql1	2	0	1	0	0	2
C1ra	631	490	375	630	313	687
C230035I16Rik	62	50	40	57	36	104
C4bp	1	0	0	0	1	1
C530044C16Rik	1	0	3	1	1	1
C920009B18Rik	4	3	2	5	2	6
Cacna1d	153	86	70	144	102	114
Cacnb2	4095	3304	2375	3963	2499	3954
Calu	16499	11594	10482	15445	9369	16505
Caml	480	322	284	423	320	532
Camp	1	0	0	0	1	1
Cand1	2189	1506	1239	2094	1141	2073
Capn2	5573	3692	3182	5155	3266	6182
Car5b	48	28	26	32	22	50
Car6	0	3	0	1	0	1
Cars2	1470	859	737	1201	720	1344
Casc3	1772	1187	947	1479	977	1712
Casp4	173	90	103	135	97	216
Cbwd1	527	374	281	408	329	495
Cbx1	700	496	446	578	456	716
Cc2d1a	609	413	269	471	301	592
Ccdc151	3	0	0	0	0	2
Ccdc37	24	16	10	17	11	21
Ccdc59	602	488	436	517	388	753
Ccdc74a	8	1	1	5	2	7
Ccl6	355	321	354	410	154	610
Ccnb2	34	16	13	25	4	64
Ccnd3	1683	950	800	1544	786	1699
Ccr9	0	2	2	1	1	1
Cct8	6720	4601	4300	5951	3827	7079
Cd209a	11	7	3	8	5	15
Cd300a	60	28	41	36	33	103

Cd300lb	31	20	10	16	12	32
Cd300lh	1	2	1	2	1	0
Cd40	80	53	48	70	42	74
Cd52	74	55	42	93	25	108
Cd79b	21	9	11	10	14	14
Cd80	22	19	20	23	12	28
Cd81	12006	8008	6467	10969	6714	13532
Cdc42	7709	5066	4488	7081	4393	8898
Cdc42ep2	575	356	340	554	283	700
Cdc45	116	77	53	97	65	133
Cdk12	1370	989	771	1243	808	1284
Cds2	11097	7548	6021	11942	6464	10389
Cebpzos	1080	886	755	910	726	1316
Celsr2	541	367	206	445	273	413
Cemip	0	4	1	1	0	3
Cenpn	36	29	21	38	22	47
Cep250	849	633	462	643	616	993
Ces3b	1	2	0	2	0	0
Cfdp1	1231	1050	854	1057	837	1506
Cfhr2	15	3	3	9	3	6
Cftr	1	0	0	1	1	0
Chad	6	13	3	18	6	7
Chchd1	1680	1158	1152	1398	927	2087
Chd8	3151	2220	1631	2700	2024	3318
Chek1	31	15	16	19	18	43
Chmp3	3064	2050	1914	2503	1763	3413
Chn1	1360	948	839	1288	864	1544
Chpt1	17484	12000	10928	14328	11634	16346
Chrnb2	33	24	24	23	21	37
Clca5	2	13	5	4	4	11
Clec12a	67	63	29	64	31	112
Clec2i	19	17	8	18	12	27
Clec4a4	0	0	1	1	1	0
Clec4n	41	27	42	50	12	55
Clip3	346	263	165	393	191	345
Clip4	14004	9606	8133	11944	8199	13590
Clk1	7084	4470	3555	5458	3867	8520
Clk3	1674	1097	845	1307	1004	1896
Clns1a	1136	689	608	857	606	1136
Clpx	4365	2957	2854	3617	3175	5123
Clstn1	4652	2816	2235	4009	2611	4493
Clu	24330	14769	14502	21365	14458	27460
Clvs1	49	51	41	54	59	62
Cnga2	10	1	2	8	2	8
Col23a1	156	80	65	95	75	133

Col24a1	5	4	1	7	1	6
Col25a1	38	18	18	26	18	49
Col2a1	2	1	1	2	4	0
Col4a6	103	79	51	147	46	96
Commd1	1832	1217	1156	1637	1109	2270
Copb1	2638	1844	1586	2469	1471	2648
Cope	2339	1377	1127	1960	1150	2544
Coro1a	208	128	85	219	84	210
Cox5a	25305	14158	15900	23256	12922	31102
Cox5b	6737	5051	5043	6421	4208	7981
Cox7a2l	4789	3340	3140	4160	2746	5477
Cp	5206	3344	3518	3689	3479	5605
Cpa1	2	1	0	1	0	4
Cpa3	25	14	7	14	4	27
Cpeb1	1577	1036	785	998	908	1684
Cpeb2	3420	2114	1477	2924	2067	2847
Cpne8	495	331	261	414	282	601
Cpt1c	109	100	75	120	75	158
Creb3	816	510	413	703	418	934
Creg2	0	2	1	1	2	2
Crip2	41152	25538	19103	36485	21165	43644
Crispld2	3918	2765	2201	3997	1616	4050
Crlf3	587	362	342	516	320	705
Crot	2330	1679	1427	2034	1417	2389
Cry1	1094	685	550	1038	587	1068
Cryzl1	1043	670	637	845	585	1118
Csmd1	50	39	27	30	31	55
Csnk1g2	3707	2376	1953	3185	2164	3950
Csnk1g3	939	664	537	803	585	1125
Csnk2b	2722	1875	1413	2446	1603	2898
Ctbs	322	209	173	280	184	379
Ctc1	744	521	383	685	462	754
Ctif	667	467	324	561	442	679
Ctla4	159	84	100	78	116	235
Ctsd	41552	26618	23963	34514	24460	37621
Ctsf	1665	1019	845	1374	942	1756
Cuedc1	3595	1846	1572	2408	2320	3051
Cul4a	5467	3694	3162	4550	3213	5324
Cul4b	1479	1036	878	1245	953	1808
Cyb561d2	75	51	31	76	49	63
Cybrd1	9	9	9	7	15	13
Cyp51	650	588	433	647	440	711
Cyr61	1728	1087	1030	1293	1243	1874
D16Ertd472e	319	207	174	279	166	291
D5Ertd605e	3	3	2	1	3	8

D630041G03Rik	6	8	6	10	6	11
D730045A05Rik	1	1	1	1	2	2
D7Ertd443e	71	42	58	42	55	74
Dach2	1	2	1	0	2	1
Dact2	37	23	18	28	20	29
Daxx	676	462	378	610	393	799
Dazap1	1978	1262	951	1505	1161	2198
Dcaf7	3482	2295	1950	2962	2014	3285
Dck	287	193	132	221	115	320
Dclk2	84	44	30	59	42	88
Dcn	28966	20909	17094	27464	13476	32350
Dcun1d4	829	550	543	676	545	801
Ddx18	404	283	250	339	224	440
Ddx43	2	0	2	0	1	2
Ddx52	1155	703	656	1000	655	1308
Deb1	1614	917	960	1342	742	1735
Dedd	1117	685	624	911	603	1083
Dedd2	495	232	224	342	205	442
Defb25	3	0	0	0	0	2
Depdc7	63	45	41	37	29	89
Dffb	365	191	138	202	160	342
Dfnb59	1	0	0	1	1	0
Dhcr24	120	59	63	100	64	128
Dhx30	2012	1252	1029	1663	1066	1837
Dhx36	2576	1860	1441	2199	1673	3015
Dhx38	974	698	479	891	582	1008
Dhx9	3834	2792	2201	3396	2289	3730
Dio2	66	61	61	103	51	88
Dip2b	2467	1718	1380	2178	1454	2286
Dis3l2	570	400	330	531	360	635
Disp1	472	380	284	459	302	437
Dlst	32941	22566	19841	30141	18587	31943
Dmwd	2225	1470	1177	1896	1354	2393
Dna2	93	52	48	53	36	112
Dnaaf1	1	0	0	1	0	1
Dnajb3	1	3	3	2	2	7
Dnajc5	4022	2746	2488	3743	2286	3956
Dnmt3l	2	2	4	2	3	7
Dock1	2490	1738	1515	2140	1713	2836
Dok7	728	357	308	674	270	460
Dolpp1	285	161	169	219	138	292
Dph2	159	108	96	131	98	145
Dpm1	2212	1477	1464	1765	1481	2909
Dr1	1868	1132	1030	1508	1031	1776
Drg2	1004	657	522	822	516	1000

Dtd2	190	141	120	194	131	216
Dtna	3343	2222	1840	2866	2016	2931
Dtnbp1	3011	1833	1758	2410	1947	3305
Dtwd2	118	79	75	93	73	116
Dtx3	1862	1093	805	1291	1032	2009
Dusp10	405	249	189	320	242	270
Dusp22	903	544	588	812	516	1105
Dusp27	2239	1717	1422	2186	1383	2175
Dusp3	6943	4176	4599	6104	4350	8215
Dync1h1	18381	12680	8957	15945	10150	15149
Dync1li2	5305	3337	3006	4303	3016	5016
Dynll1	482	268	280	529	214	523
Dynll2	20147	13264	13401	18674	10904	21323
E230029C05Rik	6	5	6	5	3	8
E2f7	113	81	38	69	42	125
E330033B04Rik	252	139	122	120	138	268
Ebna1bp2	1205	843	730	1010	725	1266
Eci3	1	1	2	1	0	2
Ecm1	1172	751	667	1016	613	1478
Eef1a2	59464	39579	31064	51732	30799	53850
Efcab3	0	1	0	2	0	0
Efhd1	61	66	76	70	37	120
Eftud1	520	422	322	499	356	647
Ehmt1	1274	1025	731	1159	800	1283
Eid2b	590	384	341	513	389	637
Eif1b	1581	937	977	1387	924	1828
Eif2ak2	948	672	639	774	594	1393
Eif2b2	926	583	457	802	511	962
Eif3e	4185	2790	2532	3622	2317	4404
Eif4g3	3745	2801	2235	3600	2349	4427
Eif5b	11189	9091	7915	9417	7272	14184
Elmo2	1002	606	530	905	547	1032
Elovl6	50	52	30	90	23	64
Emc1	3021	1942	1431	2416	1570	2730
Emg1	1541	833	783	1272	676	1410
Eno2	70	47	28	61	46	68
Ensa	5610	3371	3122	4756	3004	5049
Entpd6	701	425	321	476	386	630
Eny2	1480	989	924	1254	923	1826
Epb4.1l2	3067	2270	1576	2814	1760	3558
Epc2	1923	1297	996	1514	1161	1712
Erich1	202	174	128	177	162	273
Erich4	5	1	7	8	3	8
Ero1lb	227	131	113	191	93	205
Erp29	1452	877	946	1240	746	1586

Esx1	0	1	0	0	1	1
Evi5l	279	172	171	268	166	202
Exosc10	2433	1713	1188	1920	1416	2299
F2rl1	66	28	29	47	19	59
Fabp9	1	3	1	0	2	2
Faim	131	109	104	140	91	199
Fam109a	486	230	188	409	205	436
Fam111a	466	310	282	385	253	498
Fam134c	2071	1087	1045	1672	1030	2212
Fam149b	1309	879	791	1064	813	1305
Fam151a	1	0	0	1	1	0
Fam160b2	1234	751	628	1062	729	1197
Fam166a	3	2	1	2	4	3
Fam179b	1462	931	816	1230	783	1446
Fam181a	0	0	1	0	1	1
Fam186b	0	3	2	3	3	2
Fam187b	1	0	0	0	1	1
Fam189a1	3	1	0	2	1	3
Fam189a2	2065	1327	1165	1741	1194	2406
Fam35a	309	206	202	209	218	311
Fam49a	661	397	428	565	283	776
Fam76a	2014	1377	1183	1850	1241	2240
Fam83h	34	23	22	14	14	54
Fam92a	904	738	636	860	627	975
Fancc	193	153	88	191	114	217
Fancm	252	187	157	210	156	267
Fank1	0	3	2	0	2	7
Fastk	8603	5233	4120	6386	4553	8001
Fat1	1915	1342	977	1820	1073	1451
Fate1	0	0	1	0	1	1
Fbxl3	4832	2792	2190	3692	2675	4791
Fbxo24	9	8	3	7	8	12
Fbxo34	524	346	255	465	271	441
Fbxo6	1017	600	575	827	609	1126
Fbxo8	2712	1617	1564	2116	1451	2765
Fbxw17	125	75	63	96	66	162
Fbxw8	2244	1300	1049	2067	1103	2146
Fcf1	465	290	254	370	259	578
Fcho1	18	13	9	17	7	15
Fcrla	11	1	3	7	1	4
Fer1l5	0	0	2	2	0	2
Fert2	1000	797	607	957	635	988
Fez1	54	60	32	48	45	91
Ffar4	3	0	1	2	0	1
Fgd6	724	471	349	642	470	652

Fgfbp1	2	0	0	0	3	0
Fgfbp3	164	65	96	80	67	192
Fgfr1	1564	987	803	1411	710	1495
Fkbpl	198	143	136	167	125	212
Fn3k	783	480	411	589	401	790
Foxo1	2070	1367	1161	1675	1188	2030
Foxp1	2312	1604	927	2105	1374	2037
Foxp4	587	327	238	530	323	457
Frrs1l	1	4	2	3	1	2
Fubp3	782	542	391	610	497	900
Fyb	275	209	157	232	138	324
G6pc	1	3	0	0	1	2
Gabre	34	23	24	24	34	41
Gadd45b	231	194	107	239	171	213
Galr2	2	1	0	1	0	4
Gapt	13	6	8	12	2	12
Garnl3	67	75	38	66	46	121
Gbf1	2864	1969	1461	2653	1553	2428
Gbgt1	28	21	10	18	14	25
Gbp2	1355	1116	810	1262	795	1977
Gcdh	3494	2344	2000	2841	1956	3683
Gcfc2	246	173	155	176	132	309
Gde1	1608	1310	1094	1547	944	1948
Gdpd3	49	769	24	34	33	1835
Gemin5	731	532	440	672	447	675
Gfm2	3393	2258	2002	2966	1987	3221
Gga1	2060	1213	887	1622	990	1747
Gga2	2280	1525	1241	2056	1295	2579
Gins1	76	52	39	58	48	98
Gins4	592	471	342	495	340	675
Gipc2	149	93	108	111	89	168
Gltp	704	518	365	662	386	852
Gltscr1l	1340	889	704	1061	801	1235
Gm10190	0	2	7	2	3	3
Gm10433	2	1	2	1	2	1
Gm10451	12	20	6	14	7	16
Gm10548	15	10	11	7	7	21
Gm10658	21	17	14	16	11	25
Gm10731	2	0	1	2	0	0
Gm11127	1	0	0	2	0	0
Gm12669	1165	739	655	993	672	1027
Gm12942	82	36	40	41	47	65
Gm13251	82	45	61	62	47	84
Gm13283	2	2	0	2	0	1
Gm14124	0	1	0	1	1	0

Gm14391	6	0	1	1	1	3
Gm14436	1	7	5	5	3	4
Gm15698	2	0	0	0	3	0
Gm15772	1787	1167	988	1502	746	2097
Gm15941	2	2	0	1	0	2
Gm16796	1	0	2	0	1	1
Gm19557	37	14	20	17	15	39
Gm1968	1	1	1	1	1	0
Gm20257	26	24	14	20	16	48
Gm20597	9	4	4	6	8	8
Gm20753	2	6	2	4	2	9
Gm20757	10	7	7	4	9	9
Gm2382	66	41	49	67	44	83
Gm2848	0	0	1	0	1	1
Gm3002	1	0	0	1	1	0
Gm3716	5	2	6	4	1	7
Gm4262	9	2	5	7	5	10
Gm4461	1	0	2	2	1	2
Gm4532	13	13	12	8	12	32
Gm4759	1	2	0	1	0	1
Gm527	114	55	69	69	81	140
Gm5424	103	49	43	76	51	107
Gm5640	3	1	0	2	0	1
Gm5796	3	0	0	1	0	1
Gm5820	10	11	9	9	6	14
Gm6297	38	43	27	34	39	66
Gm6402	6	1	6	3	4	12
Gm648	3	1	2	1	0	4
Gm6567	1	0	0	1	1	0
Gm6710	6	0	1	1	1	3
Gm6904	1	2	2	1	2	5
Gm711	2	0	4	0	4	6
Gm8615	31	1	3	1	15	27
Gm9855	55	87	57	94	54	116
Gmpr2	457	364	260	462	281	550
Gna14	30	12	8	21	17	23
Gna15	14	9	14	10	8	18
Gnas	46841	30886	25316	42643	27104	51975
Gng2	382	269	192	336	223	433
Gnptab	2130	1717	1132	1863	1393	1908
Gnrhr	1	0	0	2	0	0
Gpa33	1	0	0	0	0	2
Gpbp1l1	3002	2354	1688	2442	1909	3212
Gpr65	56	58	47	72	26	76
Gpr82	4	0	0	3	1	2

Gpx2-ps1	1	0	0	1	0	1
Gramd2	1	0	0	1	1	0
Grb2	3596	2221	2028	3432	2003	3756
Grcc10	1902	1253	1072	1700	1160	2077
Grhl2	280	235	144	307	160	334
Grhl3	1	2	1	2	0	1
Gria4	9	6	2	13	2	9
Grm4	0	1	0	0	1	1
Gsg2	16	17	10	11	15	30
Gsn	48416	33534	26377	52694	19175	42789
Gss	497	330	298	450	295	588
Gstk1	2564	1635	1426	2200	1427	3002
Gtf2f1	1059	829	621	900	615	1203
Gtf3c1	3755	2590	1724	3187	2122	2974
Gtf3c2	3123	1923	1513	2206	1632	3138
Guf1	1894	1233	1196	1601	1062	2020
Gusb	1258	824	723	1176	490	1412
Gys2	2	0	1	2	0	0
H2-DMa	164	103	65	126	58	161
H2-Ob	43	24	22	32	25	53
Has1	1	1	2	1	1	1
Hdac10	379	227	189	288	178	376
Heatr5b	2349	1504	1076	2113	1727	1671
Heatr6	700	504	364	620	391	644
Hephl1	2	2	1	1	1	2
Herc1	5810	3803	3069	5049	3675	5835
Hes5	1	0	0	0	0	2
Hes7	1	3	0	1	1	1
Heyl	1299	838	632	1458	618	1189
Hilpda	119	53	64	103	36	106
Hipk3	19921	13003	11039	16708	12129	16594
Hist1h2bc	1641	1344	1002	1376	1132	1749
Hist1h2bp	1	0	0	0	0	2
Hist2h2ac	0	1	0	0	1	1
Hist3h2ba	3	4	3	2	3	4
Hmgcr	444	304	290	415	246	449
Hmgxb4	790	505	433	623	511	887
Hnrnpa3	1312	759	723	1065	736	1465
Hnrnph2	2138	1503	1340	1868	1349	2141
Hnrnpul2	6806	4742	3907	5616	4207	6553
Hoxa1	10	9	5	4	4	15
Hoxa6	0	3	0	0	0	7
Hs3st3a1	55	43	40	58	21	66
Hsd17b13	2	1	0	5	0	0
Hspb7	61303	44650	51240	79681	45100	67970

Htra4	225	192	111	233	106	220
Hydin	9	10	5	9	8	15
Hykk	134	98	76	112	81	186
Iba57	868	427	463	681	487	883
Icam4	23	9	12	17	7	18
Idh1	3450	2382	1816	2697	2304	3846
Idh3b	29477	20474	17156	23809	17763	29192
Ids	2606	1621	1348	2357	1462	2607
Ier3ip1	1605	953	1051	1387	786	1771
Ifi27l2b	4	7	3	1	2	10
Ifi30	222	150	127	219	114	281
Ift27	248	137	120	199	124	285
Ift57	537	417	283	492	346	626
Igsf1	921	538	571	906	447	812
Igsf5	1	0	0	1	0	1
Igsf6	85	67	49	81	48	125
Igtp	1884	489	391	1446	488	1197
Ihh	5	3	3	4	2	4
Ik	4299	3593	2829	4045	2877	4641
Ikzf4	21	23	18	19	13	31
Il12b	1	0	0	1	0	1
Il18r1	16	14	8	18	15	15
Il1rap	354	240	216	307	177	389
Il1rl2	80	49	36	56	36	116
Il2	1	0	0	0	0	2
Il31	1	0	0	2	0	0
Il31ra	1	0	0	2	0	0
Ildr1	3	2	0	5	0	3
Inadl	1305	972	846	1165	869	1312
Inmt	429	408	490	537	511	597
Insl5	1	0	0	1	1	0
Ints4	2394	1407	1298	1956	1754	2017
Ip6k3	430	294	316	357	288	461
Ipmk	1419	899	760	1220	749	1325
Ipo4	1634	1011	703	1308	788	1388
Iqcb1	529	351	303	451	297	511
Iqsec1	4916	3325	2772	3975	3173	4329
Irak4	226	189	163	236	171	299
Irf2bpl	1629	1020	795	1400	968	1538
Irf4	76	34	26	54	50	53
Irf5	145	80	75	113	62	137
Isoc1	3444	2433	1844	3258	1989	2789
Isoc2a	1592	799	704	1030	763	1427
Itga7	7113	3894	3042	6267	3592	6161
Itgad	3	1	2	3	3	3

Itgb8	33	19	12	33	17	28
Jmjd8	1148	816	622	1021	599	1180
Jsrp1	31	15	15	25	13	22
Jund	2531	1472	991	2055	1076	1990
Kansl1	2423	1631	1113	2087	1326	1946
Kat5	1054	736	576	759	679	1045
Kat8	599	323	297	491	287	661
Katna1	792	517	462	670	379	872
Kazn	219	143	110	164	138	254
Kbtbd7	945	562	430	768	473	757
Kbtbd8	93	44	67	75	47	86
Kcna7	628	379	335	530	314	584
Kcnb1	4308	3061	2185	4033	2555	4433
Kcnn1	620	318	328	448	328	531
Kdf1	0	0	1	0	1	1
Kdm2a	3666	2558	2022	2981	2261	3473
Kdm4d	2	1	0	1	2	2
Khk	317	216	157	218	202	397
Kif15	34	15	11	16	6	66
Kif17	1	4	0	1	1	2
Kif20b	78	48	40	44	43	126
Kif7	103	60	50	79	47	92
Kin	602	448	407	506	382	683
Kiz	3341	2563	2059	2706	2289	3420
Kl	3	4	1	3	0	4
Klc1	4983	3346	2527	3850	2780	4915
Klc3	3	3	1	2	2	2
Klhdc10	3012	1992	1852	2625	1900	2738
Klhl13	1766	1277	840	1638	814	1675
Klhl18	858	590	439	763	439	792
Klhl20	1071	781	621	907	637	1123
Klra23	3	2	1	4	1	4
Klrc3	0	1	0	0	1	1
Klrg1	1	2	0	1	2	2
L3mbtl1	2	2	0	1	0	2
L3mbtl4	0	0	1	0	2	0
Lace1	2529	1561	1665	2063	1487	2640
Lamc3	7	8	17	7	5	22
Lamtor4	694	400	352	564	353	807
Lat	11	8	5	12	7	12
Lbp	224	139	179	187	151	237
Lcmt1	750	480	427	666	423	868
Lcp1	897	601	475	722	367	1070
Lct	1	2	1	1	1	1
Ldlrad4	386	268	174	356	241	357

Ldoc1l	370	280	219	333	212	375
Leng8	7851	5393	3630	4612	4543	8488
Letm2	449	342	223	354	276	420
Lias	2274	1487	1386	1942	1442	2657
Limk2	1225	719	661	1002	725	1266
Lin37	260	164	139	249	125	314
Lin7c	3671	2291	2041	2957	2055	3565
Lins	312	178	131	218	165	245
Llgl2	568	297	232	432	327	484
LOC106740	567	335	324	443	293	572
Lonrf1	992	622	496	786	614	743
Lphn1	1765	1083	759	1353	860	1565
Lrch4	1309	798	540	1010	699	1340
Lrrc15	4	2	4	5	2	7
Lrrc32	589	409	349	567	347	696
Lrrc55	79	42	27	60	20	68
Lrrc56	137	69	45	87	78	126
Lrrc63	1	0	0	1	1	0
Lsm10	333	215	134	261	199	331
Lsm11	335	163	183	263	182	355
Lsm6	632	652	604	813	501	1104
Lss	100	56	57	71	71	113
Lst1	10	6	11	12	4	23
Lta4h	1550	939	837	1401	866	1609
Ltbp4	16629	10627	6449	15738	7354	17013
Ltf	6	0	2	3	2	1
Luzp1	3859	2679	2204	3507	2260	3496
Luzp2	22	10	6	6	10	19
Ly6g6e	0	1	0	2	0	0
Lypla1	6174	3858	3439	5047	3463	5762
Lysmd4	886	548	420	756	456	697
Lyzl4	2	0	1	0	0	6
Lzts3	341	189	132	291	183	283
Mad2l1	196	117	105	151	111	247
Mad2l2	206	182	130	225	128	284
Mafa	9	5	4	9	5	10
Mafk	1845	1098	980	1604	1008	1952
Mak16	769	504	459	585	479	763
Mal2	1	0	2	3	2	0
Mamstr	269	210	139	298	158	277
Mansc1	186	129	105	165	119	215
Map2k4	3205	1943	1802	2649	1796	2965
Map2k5	929	694	548	791	580	947
Map3k5	3400	1989	1662	2772	2044	3170
Map7d2	67	35	29	66	38	53

Mapk1	7182	4767	4036	6384	3936	6674
Mapk8	1543	1108	1011	1517	962	1452
Mapkapk2	13553	8172	8306	11971	7645	12125
Mapre1	4409	2969	2357	4213	2298	4994
1-Mar	99	52	65	92	50	131
Mat2a	9726	6084	5365	7457	5031	10358
Matk	10	10	16	4	13	18
Matn2	1361	889	620	1228	493	1344
Mboat7	850	558	451	696	516	720
Mccc1	6467	4460	3482	5535	3713	5879
Mcur1	4126	2849	2659	3495	2575	4240
Mdk	99	102	60	110	56	110
Med24	2205	1319	1027	1719	1144	1899
Med9	1113	676	629	1015	610	1226
Mettl15	410	266	216	361	289	377
Mettl21e	2	2	0	1	0	2
Mettl4	262	192	126	204	149	247
Mettl9	3256	1798	2082	2649	2024	3752
Mfsd10	193	110	87	160	92	216
Mfsd9	54	34	29	47	27	73
Mgmt	147	76	100	115	64	166
Miat	4	0	4	4	1	7
Mib2	1223	709	483	1070	639	1015
Micalcl	24	21	13	18	10	30
Mid2	1706	1316	992	1762	1031	1983
Mir103-2	2	1	1	0	1	2
Mir142	2	1	0	1	0	1
Mir148b	0	1	0	0	1	1
Mir181b-2	1	0	0	2	0	0
Mir1928	1	0	0	0	1	1
Mir1929	2	2	0	0	1	2
Mir1938	1	0	0	0	0	2
Mir1943	3	0	4	2	1	3
Mir23a	3	0	0	1	1	3
Mir3100	32	18	28	33	15	31
Mir335	1	0	0	1	0	1
Mir3572	12	7	1	8	10	7
Mir541	2	0	0	0	3	0
Mir684-1	12	5	4	6	5	8
Mir6898	0	0	1	1	1	0
Mir6910	0	0	1	0	1	1
Mir6920	2	0	1	2	0	0
Mir6928	1	0	0	2	0	0
Mir6929	10	7	2	7	4	15
Mir6942	30	15	9	20	14	33

Mir6954	4	1	1	4	0	5
Mir6970	9	0	2	3	4	8
Mir698	5	6	5	4	3	8
Mir6988	2	1	1	1	0	2
Mir7028	0	1	3	1	1	1
Mir7036b	1	0	0	2	0	0
Mir7041	1	0	0	1	0	1
Mir7045	10	9	2	4	8	6
Mir7049	3	0	0	1	0	1
Mir7051	25	12	15	13	13	25
Mir7061	7	2	5	3	8	8
Mir7079	8	6	5	8	2	18
Mir7115	11	5	5	8	5	6
Mir7238	1	0	0	1	0	1
Mir7682	1	2	0	0	0	2
Mir8098	1	0	0	2	0	0
Mir877	1	2	1	2	0	1
Mks1	117	95	70	93	82	171
Mlana	5	13	9	11	6	9
Mlph	36	26	11	26	17	50
Mmp16	42	34	15	51	22	40
Mmp24	0	1	3	3	1	3
Mob3b	100	57	46	71	54	79
Mob3c	277	208	194	253	181	402
Mocs3	148	97	80	123	87	181
Mok	29	30	22	32	30	39
Morc3	1344	896	828	1069	812	1401
Mospd4	4	1	2	2	1	3
Mrfap1	5456	3360	3103	4553	2973	5201
Mrpl15	2664	1698	1859	2271	1421	3142
Mrpl22	638	439	480	601	373	734
Mrpl27	584	407	331	546	350	674
Mrpl36	985	596	521	744	507	986
Mrpl41	1516	1043	1042	1328	829	1846
Mrps12	672	444	343	502	465	711
Mrps18c	737	437	504	690	406	962
Mrps5	1090	854	779	925	754	1279
Ms4a4b	22	34	4	26	11	18
Msl1	4086	2755	2449	3626	2364	4004
Mst1	0	1	0	0	2	0
Msx1	131	63	71	118	52	160
Mta2	2032	1374	1144	1859	1238	2190
Mta3	565	438	366	619	366	658
Mterfd2	501	304	311	407	260	533
Mtl5	253	89	99	150	66	234

Mtss1l	1870	1206	972	1429	1084	1760
Mum1l1	127	90	117	116	103	120
Mxi1	2667	1703	1316	2453	1596	2445
Mycbpap	27	28	14	22	19	28
Myef2	454	340	263	393	265	637
Myh1	1	4	1	1	0	4
Mynn	1176	724	606	916	649	1072
Myoz3	1	0	0	1	1	0
Naa60	2795	1955	1673	2541	1621	2748
Nab2	334	212	176	337	163	372
Naf1	484	378	265	391	281	533
Naglu	448	245	184	357	221	443
Nap1l4	7409	4881	4139	6460	4107	6970
Nat9	608	394	312	497	358	687
Ncaph2	2328	1507	1150	1985	1336	2422
Ncdn	795	453	348	699	451	661
Ncor2	4415	3478	2291	4507	2519	3501
Ndor1	659	384	324	472	387	738
Ndufa8	10983	7194	6767	9305	7087	13136
Ndufaf1	2832	1861	2014	2550	1693	3077
Ndufaf6	593	404	417	493	373	659
Ndufb3	5066	3521	4006	4769	3702	6775
Ndufs1	41493	26865	23974	35709	23712	37504
Ndufs3	7207	3592	4151	6178	3625	8038
Ndufv1	28011	15757	13970	23885	14972	28228
Nedd4l	1752	1246	1007	1539	1206	1911
Neil3	27	9	12	11	3	60
Nenf	1319	930	774	1223	750	1622
Nepn	125	63	69	110	47	172
Nfasc	30	18	20	32	19	10
Nfatc1	1425	952	605	1288	760	1404
Nfya	990	673	500	1006	549	959
Nhlrc4	3	2	1	0	1	4
Nkx2-5	1538	899	736	1262	958	1357
Noc4l	459	306	195	356	208	441
Nol3	1720	1265	958	1547	1184	1787
Nol7	2777	2051	1701	2320	1780	2911
Npas2	121	102	92	78	94	158
Npas4	6	8	6	12	6	9
Npat	861	643	525	755	490	950
Npff	77	43	34	49	55	81
Nrn1l	14	4	6	7	5	10
Nsg2	9	4	3	5	5	4
Nsl1	24	12	19	20	17	35
Nsmf	646	435	307	517	364	757

Nsun2	2536	1820	1494	2196	1510	2599
Nsun5	334	233	201	285	179	361
Nuak2	219	107	101	158	104	247
Nudt19	2417	1552	1524	2137	1260	2616
Numbl	258	175	86	249	129	231
Nup210l	34	31	18	31	25	48
Nupl1	740	537	405	706	420	626
Nxpe2	138	110	93	90	99	166
Nxt2	542	356	266	444	265	518
Oat	10263	6212	5601	8538	5409	9444
Opcml	96	67	59	105	64	102
Ormdl1	733	473	448	652	410	706
Oser1	140	105	88	119	84	147
Osgep	1410	879	764	1069	760	1446
Otog	2	6	3	3	0	7
Otud5	1827	1278	985	1602	1113	2055
P2rx4	840	583	468	643	487	887
P2ry10	19	9	5	13	2	16
Pacsin3	5543	3289	2955	4561	3368	5648
Padi3	0	1	0	2	0	0
Pagr1a	1090	683	543	824	585	1040
Pak2	3161	2197	1882	2987	1765	3039
Paox	53	28	25	21	31	51
Papd5	1884	1330	1027	1550	1168	1719
Pard6b	106	66	72	102	80	113
Pard6g	216	168	112	205	129	267
Parl	1641	1090	1021	1442	995	2013
Parp12	1489	887	679	1163	757	1628
Parp3	2229	1490	1466	2062	1411	2666
Patz1	902	521	404	751	502	845
Pbk	16	10	11	4	4	52
Pcbd2	287	173	170	243	140	289
Pcbp2	11597	7198	5892	10067	6388	12375
Pcdhb19	60	29	27	39	51	47
Pced1a	383	241	200	284	199	382
Pcf11	2144	1327	1138	1587	1350	2397
Pcgf1	156	113	97	140	86	235
Pcgf3	1612	1238	985	1525	1085	1399
Pcnxl2	13	8	12	11	11	21
Pcnxl3	999	575	393	799	494	979
Pcnxl4	372	226	197	332	152	356
Pcolce	1417	751	733	1241	584	1710
Pcsk4	78	48	33	39	40	81
Pde6g	11	11	5	14	10	11
Pdha1	67964	47814	42858	60562	42949	61026

Pdk1	12382	8355	6847	10175	7933	10130
Pdss1	792	472	509	681	429	806
Pdxdc1	5723	4141	3355	4843	3550	5680
Pdxk-ps	1	2	1	1	1	1
Pdzd7	11	4	2	6	3	14
Peli1	1104	764	569	1033	606	1214
Pex5	1069	705	531	961	603	1092
Pex6	1684	808	670	1201	765	1248
Pfdn5	2749	2174	1848	2417	2033	3563
Pgam5	1315	744	720	1080	657	1215
Pgap1	448	270	238	419	244	462
Pgap3	396	238	184	280	199	369
Pgbd1	99	69	58	80	59	93
Pgm1	318	229	202	271	221	411
Pgs1	725	448	405	676	432	720
Phb2	5813	3565	3192	4735	3416	6457
Phldb1	6293	4011	2970	5236	3692	6224
Pigl	130	101	86	95	87	152
Pik3cg	134	81	57	124	66	139
Pik3r5	60	26	30	30	24	62
Pikfyve	1063	674	578	746	636	1073
Pirb	184	153	97	150	86	231
Pisd	1287	781	782	1037	736	1284
Pitpna	4387	3363	2479	3858	2537	4653
Pkn2	3431	2454	2197	3049	2183	3424
Plaa	3622	2442	2280	3088	2182	3734
Plac1	1	2	1	1	0	2
Plekha2	2303	1494	1262	2136	1295	2494
Plekhf2	636	380	333	539	312	755
Plekhj1	531	278	238	443	258	523
Plekhm1	959	663	514	872	608	986
Pnpla3	90	74	55	88	55	83
Pofut1	1207	714	603	956	580	1143
Pold1	409	241	153	314	249	352
Polg2	126	59	64	98	59	149
Polh	297	193	166	291	194	286
Polr2a	3640	2707	1620	2939	2050	3357
Pomgnt2	620	349	258	492	278	493
Pomk	596	411	326	518	342	549
Pon1	1	0	4	2	1	1
Porcn	126	92	65	127	70	149
Pou2af1	11	5	12	13	7	18
Ppap2c	41	17	27	39	21	47
Ppdpf	289	168	167	248	130	283
Ppif	8139	5341	4296	7007	4441	7460

Ppih	108	79	62	58	67	130
Ppil4	1709	1194	990	1488	985	1724
Ppip5k1	4244	2554	2712	3006	2968	3819
Ppm1l	860	689	596	830	682	650
Ppp1r11	1632	1122	1036	1218	1030	1822
Ppp1r3f	200	115	87	145	94	174
Ppp1r9b	2553	1696	1349	2342	1591	2496
Ppp2r2a	2714	2060	1709	2319	1677	3058
Ppp2r4	2156	1179	1128	1776	998	2004
Ppp6c	1621	1165	984	1470	988	1597
Ppt2	1445	901	731	1268	699	1295
Prc1	98	44	42	56	21	183
Prdm10	209	144	104	147	136	175
Prickle3	1084	670	406	695	583	918
Prkag1	4208	2473	2130	3481	2469	4094
Prmt1	1635	1100	826	1416	846	1982
Proser2	338	224	153	274	170	296
Proz	1	4	6	7	2	1
Prpf38a	622	429	324	547	350	734
Prpsap2	174	123	127	170	106	254
Prrx2	2	1	2	6	0	2
Prss34	1	0	0	1	0	1
Prss51	1	0	0	0	0	2
Prune2	10471	9579	8129	9351	9968	14889
Psg17	1	2	3	2	1	2
Psip1	1594	1115	956	1406	939	1609
Psmb7	4411	2530	2771	4094	2465	5005
Psmc1	4472	3301	3051	4143	2993	5801
Ptbp1	1850	1338	926	1649	1067	2072
Ptch1	568	387	235	457	425	451
Ptgdr	1	0	0	0	2	0
Ptgds	12989	6549	8864	9345	12021	10896
Ptger2	13	3	5	14	5	9
Ptger3	1	3	3	4	1	1
Ptges	57	24	33	51	15	86
Ptges3l	1304	866	812	1263	711	1590
Pthlh	32	24	22	34	21	22
Ptpn23	920	716	480	809	559	843
Ptpn9	692	428	386	732	309	789
Ptprn	209	191	152	196	152	339
Ptprt	7	6	5	11	5	8
Ptx4	1	1	1	3	0	2
Pycrl	253	150	117	231	146	231
Pygb	38390	24034	18261	34401	24377	32284
Pygo2	708	356	323	639	326	643

Pzp	29	29	13	27	35	25
Qk	22715	15985	13660	19662	14981	19978
Qpctl	246	153	121	214	130	277
Qrfpr	3	1	0	2	3	1
Rab11fip1	39	40	16	26	23	47
Rab25	1	0	0	0	0	2
Rab27b	128	101	88	107	84	143
Rab2b	325	265	207	301	238	413
Rab34	351	189	140	262	175	363
Rab39b	15	8	6	14	3	10
Rab5b	2565	1652	1402	2246	1444	2276
Rab6b	1306	567	464	1039	598	976
Rabggtb	3203	2096	1973	2659	1846	3384
Rac2	105	63	39	106	39	114
Rad51ap2	1	0	0	1	1	0
Rad9a	324	184	152	262	132	297
Raet1c	2	1	2	3	0	1
Ranbp17	18	16	11	17	12	30
Rapgef1	7300	4358	4017	6412	4582	7039
Rarres1	22	12	13	15	8	24
Rasa2	868	479	432	605	432	1090
Rasa3	1371	954	770	1295	763	1607
Rasgef1b	468	370	262	392	301	454
Rasl2-9	127	84	80	107	94	152
Rax	1	0	0	2	0	0
Rbm14-rbm4	525	333	285	498	298	555
Rbm17	1304	1043	910	1151	872	1521
Rbm18	2348	1581	1409	2018	1312	2432
Rbm48	351	235	222	265	190	421
Rcan3	720	442	433	631	358	722
Rchy1	1416	877	793	1283	828	1489
Rcn1	1561	966	903	1242	762	1732
Rcor1	620	463	336	577	381	500
Rdh11	149	73	80	142	68	155
Recql4	60	31	26	55	25	64
Rfxank	848	598	503	858	486	971
Rgs11	26	7	4	8	7	18
Rgs16	45	8	20	21	22	50
Rgs17	107	59	96	97	53	211
Rhbdl3	615	339	292	500	318	460
Rhebl1	106	49	48	79	47	126
Rif1	1835	1198	1053	1584	1054	1734
Rimbp2	0	2	0	1	0	3
Rnasel	464	271	236	246	248	524
Rnd3	1139	877	608	1248	548	973

Rnf123	4545	2959	2095	4122	2033	3917
Rnf145	1234	721	608	1009	592	1128
Rnf17	29	30	30	33	28	55
Rorc	2282	1395	1417	1869	1451	2670
Rp9	1352	949	875	1149	894	1756
Rpap3	959	645	590	785	590	963
Rpl18	1007	668	658	956	551	1336
Rpl26	1787	1167	988	1502	746	2097
Rpl3	574	584	404	583	463	874
Rpl37a	1099	764	740	1032	662	1449
Rpl39	723	368	429	647	350	850
Rpl4	18145	13176	10730	15337	10278	19933
Rplp0	12195	7660	6443	10848	4491	13169
Rpp14	1267	791	809	1097	728	1270
Rps18	1131	657	715	1055	587	1379
Rps21	1068	778	670	957	644	1439
Rps25	877	503	585	816	440	1177
Rps27a	26	21	9	22	11	21
Rps6kb1	2616	1998	1741	2215	1728	2923
Rpusd1	152	101	70	142	76	174
Rpusd3	173	106	84	160	96	177
Rqcd1	919	532	471	821	425	786
Rrad	5575	3084	2627	3938	3498	3557
Rslcan18	92	53	46	74	49	69
Rtl1	14	5	5	9	3	10
Rundc1	779	525	428	692	382	798
Rwdd2b	240	183	159	247	161	290
Rxra	3391	1926	1663	2922	1993	2958
Ryk	2646	1888	1690	2493	1491	2780
S100a14	3	0	1	0	1	2
S100a9	21	9	20	35	13	19
Saa3	2	0	1	0	1	1
Samd4b	1404	859	691	1328	733	1363
Sap25	287	149	131	175	161	331
Sap30l	940	631	528	820	597	1013
Sart3	923	625	458	767	501	851
Sbds	2175	1722	1484	1951	1654	2780
Sbf1	2744	1712	1253	2133	1407	2425
Scaf11	4505	3241	2625	3828	2884	4148
Scd3	2	7	4	4	10	5
Scgb1c1	136	66	70	113	101	97
Scmh1	2054	1469	1075	1835	1148	1914
Scn1a	2	0	1	1	0	1
Scn2a1	42	25	20	26	25	35
Scnm1	606	564	439	620	440	921

Scoc	768	420	452	611	403	716
Scyl2	982	769	597	935	630	935
Sde2	892	566	429	714	444	839
Sdf4	4236	2983	2456	3830	2750	4662
Sdha	75535	48233	41817	65054	42438	65584
Sdsl	171	114	88	153	96	204
Sec1	8	2	7	10	6	7
Sec13	2050	1287	1152	1797	1140	2336
7-Sep	9312	6950	5731	8432	5477	9871
Serpina3g	23	17	17	21	13	23
Serpina3i	6	2	1	1	2	11
Serpinb6e	0	1	0	2	0	0
Setd2	3438	2582	1835	2986	2062	3315
Sf3b4	169	140	114	178	81	195
Sfr1	4607	3108	3127	3725	2720	5474
Sfrp1	737	584	513	839	377	800
Sgpp1	2015	1290	1123	1865	1020	1834
Sgsm2	595	340	254	453	310	438
Sh2d1a	1	0	2	1	2	2
Siglecg	7	7	6	7	5	7
Sirt6	210	118	102	159	129	213
Slamf8	21	13	7	17	3	20
Slc10a3	241	139	140	221	152	236
Slc10a4	1	1	1	1	1	3
Slc12a1	1	0	0	1	0	1
Slc12a3	28	8	3	16	5	13
Slc12a7	10749	6717	5596	9074	6199	8633
Slc12a9	214	115	96	136	140	223
Slc13a4	12	26	4	20	7	12
Slc14a2	1	0	0	1	0	1
Slc17a7	1200	460	592	1048	561	999
Slc17a8	4	1	1	1	3	5
Slc18b1	338	216	216	309	185	322
Slc19a2	793	482	398	721	356	682
Slc1a5	1572	901	824	1314	744	1474
Slc20a2	9317	6130	4632	7501	5255	8165
Slc25a13	8386	5902	5215	7277	5418	7893
Slc25a15	838	439	456	651	431	729
Slc25a21	8	0	0	3	1	1
Slc26a5	0	1	3	1	1	1
Slc29a1	2853	1665	1363	2440	1255	2575
Slc2a1	807	479	344	650	521	645
Slc2a4rg-ps	339	173	145	209	168	287
Slc30a10	3	0	0	0	0	2
Slc35a5	605	349	327	506	351	635

Slc35c1	349	225	154	260	169	325
Slc36a2	847	495	439	771	513	744
Slc36a4	217	169	142	173	132	259
Slc39a14	3256	1961	1988	2920	2059	2460
Slc48a1	2230	1450	1295	1926	1375	2490
Slc4a2	1436	904	681	1403	670	1501
Slc4a3	10474	5617	4596	7827	5970	9504
Slc7a11	0	2	1	2	1	2
Slc8a2	47	27	28	44	26	30
Slc9a1	1125	701	533	1017	630	1062
Slc9a4	17	14	2	8	14	20
Slc9b2	3	1	2	2	3	4
Slmo2	2797	1962	1942	2478	1692	3188
Slx4	323	242	175	272	199	300
Smad4	2615	1695	1442	2236	1483	2381
Smad5	3210	1971	1574	2772	1851	3042
Smarcad1	956	614	534	843	503	911
Smco3	1	0	0	2	0	0
Smg6	1618	1131	772	1341	1077	1596
Smim7	3625	1850	1802	3093	1714	3673
Smn1	408	337	270	347	242	509
Smpx	14668	9798	8933	13562	8453	17947
Smtn	5938	3731	2575	4170	3461	6443
Smyd1	24285	17973	15528	22412	15889	22700
Snora30	1	0	3	4	0	3
Snora47	2	1	1	0	0	3
Snora64	5	5	3	7	4	7
Snora7a	3	4	1	4	1	7
Snord14a	1	2	3	4	2	3
Snord37	15	5	4	5	8	8
Snrnp48	648	489	380	459	429	720
Snrpc	455	245	273	343	216	484
Snrpg	167	99	94	131	74	175
Socs3	163	66	84	117	67	210
Sod1	5994	4538	4131	5442	4227	8009
Sod3	943	685	581	817	601	1228
Sorbs1	46958	33980	28246	40748	31729	38421
Sowahb	1	1	1	2	0	0
Sox2	3	8	3	4	2	7
Sox7	1554	916	663	1439	658	1581
Sox9	183	130	72	145	70	186
Spaca3	1	0	2	1	2	2
Spag1	157	94	75	111	76	150
Spata17	13	29	17	22	14	25
Spata2	623	361	343	550	334	480

Specc1	167	145	86	160	95	155
Sphk2	856	501	415	743	420	683
Spint2	786	440	405	689	410	811
Spire1	431	324	234	378	248	421
Spn	54	38	17	51	24	62
Spon2	4	17	15	13	5	20
Spsb2	73	35	33	52	35	86
Spt1	34	23	25	8	15	61
Srcrb4d	15	10	6	11	10	19
Srd5a2	5	0	3	1	4	7
Srebf2	977	598	439	830	443	836
Srek1	2239	1620	1299	1670	1327	2559
Srp14	1898	1212	1145	1698	1038	2343
Srp54a	35	17	17	33	20	32
Srrd	202	145	122	163	133	277
Srrm4os	5	6	6	4	2	10
Srrt	2708	1924	1402	2229	1497	2721
Srsf6	6007	3614	3144	4434	2985	6218
Srsf9	556	355	295	453	303	691
Ss18	1553	1085	841	1324	854	1565
Ssc5d	368	243	204	345	157	371
Ssmem1	3	1	1	0	1	3
Ssrp1	2445	1617	1397	2034	1416	2389
St14	11	13	5	14	15	8
St18	1	0	0	2	0	0
St6galnac6	3054	1791	1744	2863	1683	3115
Stard5	318	193	182	289	177	356
Steap1	1	3	0	1	0	2
Stoml1	358	224	206	296	205	448
Ston2	474	354	210	478	186	416
Stox2	1610	1071	879	1375	1055	1665
Stpg1	12	16	11	13	12	13
Strn	1599	1320	913	1579	1100	1232
Stt3a	1551	1096	896	1452	825	1546
Stx16	1837	1255	1011	1323	1054	2043
Stx4a	2583	1742	1301	1971	1421	2606
Stx6	640	430	396	623	402	697
Suclg2	5350	3384	3223	4284	3406	4813
Sucnr1	11	14	4	10	4	14
Sumo1	1367	906	888	1114	815	1515
Supt3	517	371	301	410	321	532
Surf6	332	209	218	326	209	368
Susd4	9	5	4	6	6	4
Suv39h2	84	49	47	66	47	68
Svil	18101	14340	10882	17075	11793	16234

Sympk	4416	2921	2261	3853	2562	3433
Syndig1	5	5	7	7	1	8
Synpo2l	4725	3097	2868	4164	3004	3867
Sypl2	121	68	89	152	53	157
Sytl1	7	1	2	6	2	6
Sytl4	105	66	82	86	52	130
Szt2	1496	914	655	1062	745	1411
Tab3	913	666	537	833	573	829
Tada3	769	578	423	660	470	739
Taf11	799	551	505	765	466	985
Taf9b	898	773	539	874	615	1140
Tango2	5213	3076	2773	3902	2814	5029
Taok3	571	440	325	501	341	581
Tax1bp1	7459	5808	4886	6625	5053	7683
Taz	2116	1126	1008	1595	1188	2057
Tbc1d31	343	205	184	216	206	330
Tbc1d5	1324	1055	810	1208	849	1355
Tbc1d8b	630	435	344	569	419	654
Tbk1	1289	855	682	1044	750	1512
Tbl1x	1271	962	686	1180	747	1158
Tbl1xr1	2352	1479	1350	1788	1460	2203
Tbrg3	8	3	3	7	5	7
Tbrg4	2877	1721	1483	2283	1498	2697
Tceal7	16	8	12	9	13	11
Tceal8	845	715	543	898	523	1124
Tcf12	2240	1426	1150	1968	1236	2394
Tcf25	7805	5355	4279	6698	4908	8716
Tcp11	1	2	0	1	0	1
Tctn2	177	118	86	151	75	174
Tdg	56	87	57	94	54	117
Tecr	15029	10311	7839	12984	8201	14066
Tef	12591	7187	5450	9464	6139	10501
Tfcp2l1	59	16	23	15	31	40
Tfg	2909	2141	1702	2776	1621	2916
Tfip11	1170	765	568	1083	670	1126
Tgm3	2	2	1	2	0	2
Themis	5	6	1	3	4	3
Thpo	14	5	7	7	6	11
Timm8a1	92	33	53	64	28	89
Tldc2	1	0	0	0	0	2
Tle1	574	347	245	507	278	576
Tll1	29	14	15	20	20	33
Tlr2	202	148	91	216	96	224
Tm2d2	1248	752	719	1149	648	1430
Tm9sf3	7021	4680	4050	6066	3971	6899

Tma7	343	187	258	314	184	504
Tmco1	3509	2497	2299	2964	2165	3891
Tmco4	505	299	273	418	274	431
Tmco5	1	0	0	1	0	1
Tmem100	761	1028	747	1657	415	1470
Tmem106b	12795	8151	6389	10121	7363	10899
Tmem106c	413	286	227	332	257	511
Tmem108	123	113	71	92	82	148
Tmem117	567	317	317	472	321	431
Tmem121	0	1	0	1	1	0
Tmem125	0	0	1	0	1	1
Tmem139	2	2	0	2	1	0
Tmem145	1	0	0	0	0	2
Tmem150b	1	2	4	2	0	4
Tmem158	46	35	21	40	38	44
Tmem179b	232	137	128	196	130	265
Tmem18	890	508	438	703	502	903
Tmem189	1006	688	630	913	550	1052
Tmem194b	210	109	85	127	109	252
Tmem198	21	8	7	16	9	21
Tmem201	4625	2691	2343	3821	2387	3959
Tmem231	162	104	69	149	72	116
Tmem261	853	562	553	755	458	931
Tmem263	1119	665	613	1007	609	1175
Tmem30a	7493	5133	4390	6185	4413	7588
Tmem55b	694	380	295	535	316	754
Tmem87b	1970	1200	1086	1554	1043	1993
Tmtc4	380	278	173	335	247	377
Tmub2	1069	683	564	975	601	1128
Tmx2	2141	1176	1337	1966	912	2204
Tmx3	2870	1811	1617	2521	1650	3199
Tnfrsf13c	1	2	2	2	0	2
Tnfsf13b	99	59	43	63	44	152
Tnfsf15	1	1	1	1	1	3
Tnip1	1343	912	833	1186	930	1491
Tnnc2	8	2	4	1	7	11
Tnpo1	1943	1262	1088	1860	1272	1798
Tns3	1276	900	711	1257	680	1145
Tom1	82	41	43	47	46	71
Tom1l1	876	474	404	537	570	589
Toporsos	103	56	58	104	55	104
Tpcn1	2179	1544	1265	1949	1271	2154
Tph2	1	0	0	0	1	1
Tpmt	266	177	160	238	161	328
Tppp	1962	1272	928	1708	993	1655

Trabd2b	7838	5449	4378	6938	4441	7496
Traf3ip2	228	173	135	203	143	307
Tram1	2538	1756	1484	2357	1529	2861
Tram2	110	80	62	108	82	112
Trappc10	2710	1884	1590	2426	1686	2392
Trim23	1019	747	615	866	655	1023
Trim36	86	47	22	51	28	73
Trim43a	9	2	4	8	1	4
Trmt11	398	308	266	369	231	453
Trmu	468	305	271	391	302	531
Troap	13	6	5	3	5	26
Trp53i13	235	122	96	155	97	209
Trpa1	1	2	1	0	0	8
Trpv2	147	62	60	112	65	145
Tsacc	81	54	41	51	57	64
Tshz3	302	197	163	313	150	330
Tsku	91	69	84	109	64	142
Tspyl5	366	230	241	315	286	380
Tssk1	18	15	14	15	20	24
Tssk6	2	0	1	1	1	0
Ttbk1	10	4	4	5	6	5
Ttc13	894	618	539	712	556	907
Ttc14	3359	2125	1691	2642	1810	4007
Ttn	354356	272402	211646	304594	257495	266643
Ttr	2	5	8	10	8	4
Tuba1a	1031	662	599	1080	401	1020
Tufm	1337	760	750	1274	701	1382
Tulp1	2	0	1	1	0	1
Tvp23b	935	633	508	794	530	888
Twf1	1086	730	636	943	624	1359
Txn1	1920	1082	1114	1716	1053	2033
Txnl4a	2863	1820	1501	2400	1529	2680
U2surp	3776	2723	2099	3079	2207	3977
Uba2	1903	1247	1017	1705	1045	2127
Uba52	330	182	152	272	133	412
Ube2d1	2641	1963	1639	2253	1567	3012
Ube2h	2972	1609	1465	2465	1545	2990
Ubl3	4226	2590	2341	3685	2518	4378
Ubqln1	3471	2482	2167	3237	2133	3366
Ubqln2	1727	1081	808	1526	985	1630
Uchl5	1122	881	801	976	751	1324
Ucn2	1	0	0	0	2	0
Ucp3	2732	1073	1150	1107	1180	3905
Uevld	423	270	218	352	261	444
Ugt1a1	68	38	31	53	28	94

Ugt1a10	68	38	31	53	28	94
Ugt1a2	68	38	31	53	28	94
Ugt1a5	68	38	31	53	28	94
Ugt1a6b	67	36	31	51	27	94
Ugt1a9	68	38	31	53	28	94
Ulk1	3772	2531	1770	2797	2285	3198
Unc13d	29	10	6	24	11	21
Unc5cl	2	1	0	4	0	1
Upf3a	532	390	280	456	322	659
Uqcc2	1567	953	965	1464	804	1962
Uqcrfs1	36249	20032	20204	31371	19216	40380
Urah	9	5	16	4	3	24
Uri1	1238	950	827	1027	839	1391
Usp13	13116	9652	8508	11390	8963	12588
Usp42	639	437	322	565	417	623
Usp9x	5900	3721	3245	5336	3521	4163
Utp3	1325	1108	936	1145	915	1622
Uts2r	10	11	6	10	9	6
Vamp2	3626	2694	1960	3039	2059	3794
Vamp5	835	405	457	633	463	895
Vars2	1057	776	472	908	524	997
Vash2	22	13	5	9	7	22
Vcp	7716	4670	4161	6649	4460	8086
Vgll2	3	0	0	1	0	1
Vimp	1024	731	598	787	555	1222
Vit	142	95	120	139	70	177
Vmac	263	181	173	216	184	340
Vmp1	2242	1561	1529	2074	1288	2517
Vopp1	1006	743	721	1001	617	1083
Vps33b	233	135	152	265	121	255
Vps53	1362	996	830	1254	799	1399
Vrk2	183	141	127	193	111	259
Vstm2l	2	0	1	1	0	1
Was	73	50	37	53	34	79
Wbp2	3745	2460	1901	3174	2277	3863
Wbp5	585	439	434	607	368	813
Wdfy2	226	126	105	168	124	161
Wdr18	1209	701	590	928	587	1133
Wdr34	170	75	67	130	92	140
Wdr60	196	130	123	176	125	237
Wdr96	7	51	10	42	41	9
Wfs1	5328	3549	2709	4790	2942	4271
Whsc1	1310	858	763	1090	761	1275
Wipf1	1374	756	696	1203	654	1481
Wls	3583	2415	2068	3495	1813	3415

Wnk2	5150	3614	2226	4299	3043	3712
Wnt7b	0	0	1	1	1	0
Wsb2	3374	2128	1920	2913	1736	3388
Xndc1	522	323	264	384	283	493
Xntrpc	528	301	212	340	266	449
Xpnpep2	16	5	0	10	9	7
Xrn1	863	637	451	737	533	780
Xxylt1	702	395	373	578	369	587
Yaf2	1013	718	613	914	674	1159
Yap1	5157	3442	2962	4886	2760	4644
Ybey	263	182	141	242	140	229
Yipf2	266	156	127	213	151	281
Yipf7	5177	3047	2571	4220	2588	4516
Yrdc	367	259	276	301	229	441
Ythdf2	1210	821	699	1215	708	1293
Ywhae	20292	14108	11219	17373	11152	19910
Zadh2	4791	3318	2921	4332	2853	4551
Zbtb40	620	372	272	507	309	480
Zbtb41	1731	1067	980	1598	901	1546
Zbtb42	278	138	132	257	123	276
Zc2hc1c	212	114	93	156	109	231
Zc3h14	4153	3041	2583	3548	2617	4294
Zc3h15	1985	1346	1251	1674	1200	2069
Zcchc2	1190	858	573	1018	676	1051
Zcchc3	563	290	246	441	252	439
Zcrb1	2709	2198	2118	2091	1963	3627
Zdhhc14	377	211	184	324	219	357
Zdhhc17	2391	1547	1267	1940	1338	2279
Zeb2os	106	66	58	104	65	109
Zfat	206	119	86	157	92	168
Zfhx2os	2	2	0	0	1	2
Zfhx4	46	58	21	42	46	67
Zfp217	912	558	486	822	519	929
Zfp235	238	165	110	204	128	279
Zfp239	10	6	4	7	3	18
Zfp273	144	117	78	156	101	147
Zfp318	1514	1131	936	1144	1001	1674
Zfp326	899	634	524	713	529	966
Zfp36l1	4084	2656	1912	3630	2382	3848
Zfp384	1109	676	555	913	658	1099
Zfp385c	16	9	13	18	4	16
Zfp442	124	98	76	103	84	180
Zfp473	8	4	3	3	10	7
Zfp534	0	0	1	1	1	0
Zfp54	119	66	83	87	62	133

Zfp563	512	304	333	426	280	526
Zfp574	444	238	177	389	217	392
Zfp598	1162	730	619	870	698	1076
Zfp608	936	728	487	964	663	834
Zfp65	387	307	267	351	249	439
Zfp668	145	87	52	128	58	158
Zfp7	78	57	50	63	40	93
Zfp712	142	112	88	146	98	168
Zfp781	25	30	26	21	23	40
Zfp804a	0	0	1	1	1	0
Zfp872	2	0	1	0	1	1
Zfp935	507	363	274	404	322	632
Zfyve1	934	566	482	836	519	931
Zfyve19	340	290	199	315	251	424
Zgrf1	120	84	67	57	52	177
Zmat4	8	4	3	4	6	10
Zmynd19	285	155	160	240	142	244
Znhit2	198	107	96	180	95	205
Zpbp2	1	0	0	1	1	0
Zscan26	3788	2744	1972	2730	2253	4046
Zxdb	209	104	100	178	107	207
Zzz3	2117	1431	1176	1796	1275	1872
Pom121	1093	665	496	872	565	920
Cenpw	22	11	6	12	4	22
Mir7046	4	2	1	1	0	5
Polrmt	1117	781	519	903	599	1062
1110038F14Rik	423	262	183	384	176	344
2010012O05Rik	666	442	376	545	420	767
4930486L24Rik	94	51	42	78	50	94
4930539E08Rik	5	1	2	2	4	5
4930579G18Rik	4	5	1	1	5	2
Actc1	506460	347025	287391	498385	285422	591766
Agbl3	141	96	91	106	94	202
Alg3	305	210	146	257	153	288
Bnip3l	2601	1799	1520	2225	1556	3190
Cpsf7	2349	1448	1234	1892	1408	2464
D10Jhu81e	10817	6708	6409	9056	5896	10894
Fgf13	3953	3069	1932	3588	2231	3661
Fzd10	1	2	1	2	3	1
Gnb3	190	115	116	81	167	146
Klf3	1216	921	568	1013	691	1153
Mir27b	8	13	5	6	10	8
Mir3062	10	18	9	10	16	23
Mtfmt	329	233	191	234	214	345
Phxr4	10	14	5	7	8	13

Rnmt	752	415	389	506	398	741
Saal1	239	137	102	160	119	213
Snrpa	750	489	471	686	392	784
Spcs1	2015	1178	1173	1846	973	2419
Xkr8	125	78	73	107	72	155
Zdbf2	19	7	6	9	8	13
Zfp553	462	254	233	389	259	446
4933406C10Rik	21	15	17	11	22	35
A130077B15Rik	5	2	4	1	2	7
Adcy6	14983	9578	7209	13063	7961	11994
Ahcyl1	6447	4599	3685	5436	3906	6408
Amt	172	111	72	126	93	142
Anks3	632	377	263	486	355	608
Aph1b	584	380	365	514	310	620
Armc2	1706	807	716	897	1162	894
Atad2b	1079	719	592	967	616	1201
Atp8a2	309	238	173	344	222	269
Brd9	1697	1212	989	1380	1029	1809
Btbd11	207	119	78	168	113	184
Camsap3	47	33	25	32	21	56
Cd6	8	3	0	9	4	1
Cdkn2a	3	0	0	0	1	1
Clk4	2406	1645	1240	1963	1395	2804
Cln3	263	123	107	179	151	230
Dennd6a	1411	875	773	1209	801	1538
Fam184b	0	0	1	2	0	0
Fam3c	1213	839	691	1086	710	1421
Fanca	52	19	18	23	39	40
Gcsh	2299	1508	1459	1896	1343	2506
Gfi1b	0	0	1	2	0	0
Gm5111	0	0	1	2	0	0
Gnpda2	752	528	434	597	483	940
Lca5	204	152	77	160	100	185
Maf	694	503	362	580	436	801
Mir3960	3	0	0	0	1	1
Mir7031	1	2	0	0	2	0
Mir7036	14	13	7	8	10	27
Mrpl55	2072	1231	1261	1598	1061	2342
Mtx2	4381	2878	2895	3734	2565	4802
Ndufc2	6862	4150	3890	5899	3799	7821
Nol12	291	190	165	229	145	322
Nrg1	25	29	6	12	22	20
Phf2	2689	1737	1416	2348	1489	2354
Plk5	52	30	18	34	28	35
Prkcq	449	191	174	310	178	337

Pten	5855	3962	3807	5428	3508	5761
Qdpr	2555	1459	1429	2216	1180	3162
Rhoq	8855	6232	5378	8550	5379	7777
Rnf39	31	8	13	17	18	28
Samd1	742	472	403	624	432	643
Sco1	1235	785	773	1094	670	1278
Serpine2	1101	666	977	1138	689	1626
Slc16a8	5	4	5	6	3	4
Snora15	4	2	1	3	1	2
Snord89	4	3	2	1	2	5
Sox4	557	356	319	494	425	477
Strip1	1281	846	718	1221	751	1323
Tmem37	70	33	31	32	34	68
Tmf1	1946	1410	1249	1605	1338	1970
Tomm40	2380	1428	1196	1930	1171	2265
Tox	41	47	21	52	28	57
Trpc2	200	85	63	98	86	157
Tssk2	3	0	0	0	1	1
Uqcr10	6168	3516	4105	5796	3294	7619
Uqcr11	6666	3858	4167	5768	3798	7890
Wdr24	599	411	306	567	351	607
Zbtb20	1766	1321	922	1566	1122	1946
Zfp296	19	12	9	9	14	15
Znrd1	378	213	217	298	230	419
Mlc1	3	0	0	0	1	1
Rbbp8	294	210	160	260	159	288
Dgcr6	722	333	361	643	316	705
Polr1c	610	329	326	443	287	625
Tnni1	12	9	7	16	7	13
1110054M08Rik	451	302	288	435	266	539
2210417A02Rik	0	1	0	1	0	1
9530091C08Rik	48	30	23	30	45	43
Adipor2	4734	3238	3114	4295	3098	5682
Apool	2249	1420	1427	1899	1167	2556
Arid4a	2093	1542	1355	1534	1492	2294
Cacna1e	9	11	2	4	9	6
Casq2	42887	33081	31553	43919	28006	44147
Ccdc134	242	129	126	186	95	249
Ccdc181	565	409	361	509	418	627
Cep120	1979	1449	1132	1856	1106	1975
Cldn20	0	1	0	1	0	1
D230025D16Rik	1050	629	578	876	544	1006
Dvl2	270	140	110	210	133	259
Gm5134	0	1	0	1	0	1
Gm6086	106	75	40	89	85	77

Gyltl1b	0	1	0	1	0	1
Htatsf1	5907	4456	3670	4988	3684	6566
Insm1	0	1	0	1	0	1
Invs	329	224	189	284	185	321
Keap1	2533	1564	1373	2065	1519	2701
Lgi4	194	109	145	174	129	240
Marcksl1-ps4	12	11	10	13	13	16
Mcph1	318	192	178	264	218	310
Mgst1	1450	905	1201	1026	1231	2054
Mir1946b	0	1	0	1	0	1
Mir7067	2	0	4	1	2	2
Mkl1	387	259	194	393	206	387
Mpped2	1108	886	534	1021	575	1120
Msto1	469	331	255	409	287	540
Nox1	0	1	0	1	0	1
Nptxr	191	117	76	159	88	210
Rab7	1603	1108	1062	1516	1041	1470
Rad51d	591	404	268	475	369	603
Rbm3	503	249	323	317	330	641
Recql5	483	275	187	375	250	455
Rnf138rt1	1	3	0	3	0	0
Rsph1	17	13	14	14	12	18
Sec61a1	1583	997	921	1438	898	1747
Spen	2895	2194	1452	2478	1787	2615
Tmem134	1769	981	867	1451	837	1939
Tmem29	82	65	42	91	40	103
Tmem63b	4798	2984	2429	3841	3116	4549
Zfp518a	858	547	480	606	475	953
Zfp606	1023	700	639	821	739	1180
Arpc2	5786	4249	3687	5613	3589	7126
Pitpnm1	1070	593	386	839	545	914
Tpcn2	142	97	69	104	82	186
Lenep	317	185	145	247	165	344
Letmd1	476	307	246	386	236	481
1110057K04Rik	1467	994	919	1310	866	1492
3000002C10Rik	63	36	34	50	32	82
Ankmy2	1044	781	600	941	545	1178
Catsperg1	27	10	10	10	8	28
Ccdc71l	1156	704	576	887	656	1016
Chmp4c	488	417	330	505	393	347
Dph6	415	271	222	333	260	465
Faxc	70	46	39	64	32	65
Ganab	3320	1952	1772	2745	1636	3234
Gas2l3	187	124	135	171	112	193
Jagn1	597	324	312	520	265	523

Leprel1	116	90	75	133	58	105
Mrps7	2539	1548	1525	2203	1357	3123
Nckap1l	373	223	155	309	121	369
Nr3c2	680	535	405	662	520	683
Nudt1	205	99	105	155	79	197
Ppef2	7	6	3	7	5	9
Rnpep	1276	779	620	1053	569	1260
Serpinb9	1394	864	728	1204	820	1391
Taf4a	1428	956	728	1034	905	1308
Tfcp2	635	461	331	576	371	552
Vgll4	265	166	155	238	144	236
5430405H02Rik	67	45	52	59	46	100
A230056J06Rik	10	10	9	8	11	19
Anxa6	9404	5745	4745	8629	5075	9196
Cdon	419	350	223	384	275	375
Ckm	71278	47285	37292	63313	44076	72922
Cript	1435	963	827	1251	826	1710
Ddt	1259	776	808	1028	623	1497
Hebp2	22	53	29	45	19	90
Kat6a	3564	2547	1703	3093	2129	2880
L1td1	1	2	1	3	1	2
LOC171588	4	3	1	1	2	4
Map4k2	3077	1690	1388	2157	1676	3170
Nudt11	86	71	54	88	62	105
Olfml3	867	505	480	742	406	1079
Orai2	33	24	36	47	16	64
Pwwp2b	395	204	246	355	199	337
Rbm41	712	558	410	591	460	745
Rnf182	4	3	1	3	2	2
Rraga	2093	1355	1121	1880	1193	2204
2-Sep	7370	5352	4637	6545	4325	8136
Siah2	454	247	185	424	173	305
Snord33	1	2	1	1	2	3
Snrpd3	1341	1008	946	1193	844	1601
Supt4a	328	254	212	291	210	462
Thoc3	690	484	421	578	451	849
Thop1	626	399	280	460	327	583
Trerf1	109	84	51	102	38	119
Trim32	676	406	370	718	342	646
Usp19	6543	4109	3225	5495	3460	5659
Yy1	2161	1496	1169	1781	1299	2060
Zbtb17	920	540	423	661	512	777
Zmym5	3115	2167	1634	2243	1863	3302
2310001K24Rik	13	6	1	8	10	6
Atp6v0b	2203	1192	1051	1722	1105	2297

Crmp1	17	15	11	17	11	27
Gm13247	1	2	3	1	3	5
Gm14548	16	1	2	13	1	11
Mia	2	0	2	1	2	3
Plcd4	2	0	1	0	2	0
Ppl	665	544	487	639	460	746
Yeats2	868	533	391	601	489	985
Zbtb32	2	0	1	0	2	0
Kidins220	5286	3816	3271	4291	3483	5477
Clk2	1076	814	600	912	634	1149
Gm15708	39	29	26	29	24	44
Spc24	110	42	55	68	69	105
Cyth2	1409	869	750	1138	835	1509
Ak2	1791	1248	1099	1570	996	1974
Fcgr3	468	339	235	441	213	618
4930552P12Rik	30	15	21	22	31	26
Apc2	325	197	146	262	198	303
Csnk1a1	7919	5857	5077	7752	4829	7802
Nfe2l2	4475	3183	2493	4124	2453	4388
Psmg3	157	84	96	126	74	157
Rpp21	330	176	138	234	181	323
4833417C18Rik	26	8	14	9	14	23
4930592A05Rik	0	0	1	1	0	1
Adtrp	0	0	1	1	0	1
Amacr	1399	853	760	1106	772	1348
Apln	536	343	360	507	238	627
Asxl1	1958	1246	1054	1464	1216	1794
BC002163	935	538	775	820	528	1148
BC147527	11	8	3	11	0	10
Bckdha	8865	4821	4729	6705	4780	9972
Calcoco1	7176	5130	3407	5762	4617	7526
Ccl3	4	2	0	2	0	3
Cep170b	598	367	245	507	292	437
Chsy3	22	13	8	22	5	29
Cnot3	914	731	505	849	616	1030
Cntn6	1	3	0	3	2	1
Col26a1	12	6	3	8	3	17
Cpsf1	2268	1282	986	1726	1118	1889
Crb2	0	0	4	1	2	0
D2Wsu81e	285	202	185	256	134	350
Eif2b3	515	419	336	427	333	622
F630206G17Rik	8	10	9	3	10	13
Fam173b	459	284	262	377	242	459
Fbrsl1	1527	973	765	1181	921	1300
Fbxo2	4	3	4	2	4	4

Ggn	15	5	4	12	7	11
Gm14295	66	79	42	68	64	98
Gm17769	62	47	30	36	52	79
Gnb1l	84	55	56	75	49	117
Hist1h4h	144	77	86	95	70	160
Ltn1	2400	1501	1235	2021	1303	2127
Map10	373	334	244	365	264	384
Map3k2	1716	935	785	1226	879	1495
Mapkapk5	2042	1358	1202	1743	1384	2149
Mef2a	13286	9182	7156	11215	8089	11814
Mgat2	1101	742	666	1113	625	1217
Mir325	0	0	1	1	0	1
Mir5128	0	0	1	1	0	1
Mob2	1208	768	616	1098	640	1271
Myl1	842	568	832	913	536	1455
Myl3	151307	100785	95608	135798	93684	188145
Ndufs5	905	522	754	807	509	1107
Nhlrc3	162	109	92	116	109	148
Olfr1029	0	0	1	1	0	1
Oma1	1666	1138	938	1407	1005	1591
Pcdh20	68	43	29	55	20	71
Reep6	109	81	60	81	80	138
Reps1	1311	909	718	1079	724	1383
Rfx8	12	19	9	11	8	22
Scaf4	1301	934	626	1015	764	1244
Scamp2	1117	629	575	955	575	1156
Setmar	137	64	61	92	67	105
Slc18a2	38	35	14	21	23	43
Slc25a25	368	179	286	306	226	496
Smim24	45	34	25	47	34	44
Sytl2	59	43	45	53	53	72
Top3a	289	193	202	226	180	331
Trpm4	1118	593	479	804	562	1138
Vmn1r42	0	0	1	1	0	1
Xpo7	3201	2134	1814	2863	1893	2858
Ykt6	2703	1806	1414	2467	1576	2759
Zfp280c	593	494	370	451	394	733
Zfp692	322	217	139	222	159	335
Zkscan14	344	188	191	221	163	392
Serpinb8	117	61	48	79	48	105
Gm10790	7	3	3	2	2	8
Sorbs2	26939	21196	19482	25189	19537	27448
Trmt2a	828	485	443	725	434	890
Ppp1r35	140	79	62	107	77	146
1700101I11Rik	55	23	24	29	28	68

1700123O20Rik	358	216	235	348	197	428
2410015M20Rik	2266	1501	1524	2107	1279	3012
4930449E18Rik	0	1	0	0	0	2
C730002L08Rik	0	1	0	0	0	2
Cep95	682	527	387	534	448	718
Cfl2	16739	10382	10004	14176	11228	17305
Chd1	1736	1246	1010	1476	1086	1728
Dirc2	3094	2063	1610	2896	1477	2958
Emp3	219	142	120	216	105	260
Etaa1	286	183	156	270	158	307
Etnppl	0	1	0	0	0	2
Gli3	201	139	109	178	93	210
Gm5148	0	1	0	0	0	2
Idh2	78882	52943	42581	59275	46276	78520
Mogs	361	177	178	307	156	281
Myh13	3	1	0	3	0	3
Myom1	92704	72333	58975	87950	61138	87526
Plbd2	2256	1302	953	1880	1248	1918
Plxnb2	8062	5895	4098	7668	4185	7469
Proser1	964	654	517	738	586	947
Prr14l	726	513	399	581	421	759
Sdc4	798	691	457	937	428	997
Tcerg1	1535	1234	911	1405	970	1609
Thsd7b	3	9	4	8	2	14
Tubb2b	71	45	36	62	20	79
Vmn1r5	0	1	0	0	0	2
Zfp78	78	64	65	67	50	106
Snora75	3	0	1	1	1	4
Uxs1	179	152	101	159	104	198
Elac1	1006	649	522	731	579	1012
Wfdc17	57	55	61	62	31	152
Ccl5	23	5	7	13	4	16
4921536K21Rik	41	14	15	29	24	29
Aip	1336	848	743	1227	758	1404
Aldh1a1	1670	947	1175	1227	728	2323
Anxa11	2792	1702	1260	2305	1376	2380
Chmp2b	2385	1700	1674	2162	1500	2665
Cox17	2968	2192	2270	2593	1958	3700
Cox8b	6598	4248	4350	5410	3536	7867
Ddx24	4689	3578	2974	4430	2683	5278
Grik3	29	32	18	26	18	38
Lrrtm3	18	10	6	12	7	14
Mef2c	5140	3639	3261	4738	3673	5498
Mir29c	74	38	29	32	41	100
Mmadhc	4064	2379	2343	3403	2281	4573

Mysm1	1251	809	651	914	720	1546
Naga	823	559	394	700	446	924
Nebl	5571	4259	3081	5879	3426	3983
Otud7b	3901	2776	2384	3504	2696	4258
Pabpn1	2671	1805	1581	2080	1693	3335
Prrc1	1671	1104	792	1425	890	1455
Pus10	631	383	360	455	373	801
Tamm41	276	201	163	258	169	333
Tgs1	1024	746	573	907	615	989
Tnfrsf11b	3	5	0	3	1	8
Trp53bp1	1445	1061	806	1340	886	1289
Dnajc4	878	633	468	745	497	887
Ndufa9	19400	11561	11599	16935	11008	22269
Ormdl2	174	112	124	149	84	215
Spata5l1	73	43	43	56	43	64
Cyhr1	4522	2881	2428	3858	2523	4083
Dpyd	603	450	442	558	431	612
Elfn2	25	12	9	13	17	12
Gdi1	3831	2479	1909	2968	2090	3720
Jmjd7	275	161	100	221	124	282
Kcnv2	838	353	267	365	478	500
Ppfia2	40	18	14	18	13	41
Sh3kbp1	6694	5158	4272	6424	4193	6886
Tfap2a	10	13	2	9	4	11
Traip	37	17	16	31	12	45
Txnip	28005	14665	16339	20225	15619	34416
Zdhhc1	812	475	326	582	432	827
1700034I23Rik	3	6	2	6	4	5
Adamts1	1890	1175	1705	1620	1369	2986
Banf1	1608	1022	895	1480	869	1760
Basp1	70	38	41	57	23	78
BC055324	36	32	16	28	12	48
Brip1	78	44	33	50	18	96
Colec12	1450	749	516	957	751	973
Dbr1	301	221	195	294	170	408
Dyrk3	7	4	13	4	4	17
Emc3	2979	2044	1883	2664	1718	3197
Iqgap1	4616	3178	2074	4223	2392	4816
Pacs1	1784	1239	900	1560	913	1767
Pkib	32	34	21	32	26	51
Ankrd40	10039	6972	6414	8661	6147	10623
Oxtr	11	3	5	8	1	9
Dnttip1	586	472	331	524	387	725
Top3b	1086	753	566	981	630	1170
Amtn	2	0	4	2	2	1

Snora44	7	2	5	8	2	9
Mapk8ip1	210	140	114	198	104	258
Pcnt	7097	4899	3223	5812	4142	5701
Kdm1a	1543	968	794	1329	935	1486
Vezf1	5252	3738	2959	4385	3352	4910
Col4a3bp	3798	2391	2088	3090	2141	3637
Fam103a1	381	182	188	282	165	344
Wdr7	1425	1026	915	1313	930	1370
1700001G17Rik	5	1	1	2	1	7
1700028I16Rik	0	0	1	0	0	2
1700085C21Rik	0	0	1	0	0	2
1810024B03Rik	7	3	4	0	6	6
Aph1a	823	460	494	693	479	902
Bsdc1	2469	1556	1382	2113	1529	2545
C130021I20Rik	7	0	2	4	2	1
C1s2	0	0	1	0	0	2
Carf	537	315	304	357	307	566
Cdh2	11584	11036	7621	11841	9129	10635
Cdk1	53	29	22	34	10	100
Csf3r	49	37	34	45	27	54
Dnmbp	834	641	411	808	485	671
E130218I03Rik	0	0	1	0	0	2
Ebi3	13	11	6	12	9	17
G630093K05Rik	0	0	1	0	0	2
Gm14379	10	1	1	4	0	6
Gm8709	0	0	1	0	0	2
Gphb5	0	0	1	0	0	2
Gpr108	1292	833	661	1163	737	1294
Hectd3	1571	1038	750	1337	936	1538
Il27	0	0	1	0	0	2
Klhl2	837	431	385	663	396	852
Lap3	2808	1911	1753	2480	1577	3066
Ldlrad1	0	0	1	0	0	2
Mid1	137	94	55	119	48	133
Mir684-2	0	0	1	0	0	2
Nkap	878	558	532	899	413	823
Osgepl1	3521	2751	2202	3027	2297	3882
Pcdh18	467	295	213	466	170	396
Pcx	1816	1051	791	1485	948	1318
Pdk4	23691	9302	13302	11366	9864	38625
Phf12	2153	1488	985	1812	1301	2138
Pibf1	551	447	317	504	341	567
Rnpc3	1568	1051	782	984	838	1849
Samsn1	29	18	11	16	13	29
Taok1	6555	4074	3858	5603	3699	6243

Tmem97	110	76	68	100	58	111
Unc5d	0	0	1	0	0	2
Chst2	98	53	35	91	36	97
Snord17	3	1	0	2	1	0
Brd8	1489	1167	822	1256	1014	1762
Gab1	4089	2778	2148	3591	2624	4026
Yod1	379	208	178	252	192	358
Zfp248	144	94	92	111	93	196
Depdc5	1060	684	578	949	586	940
Nme1	503	316	347	460	247	580
2010315B03Rik	867	403	470	389	506	891
4930538K18Rik	15	16	12	13	15	27
4930555G01Rik	4	0	3	1	2	3
4932438A13Rik	4156	2721	2269	3658	2395	3655
4933427D14Rik	318	197	148	251	198	309
5031434O11Rik	31	4	16	12	22	27
A330032B11Rik	10	6	3	2	9	5
Adam33	79	31	23	28	36	63
Adamts10	1869	1089	746	1348	854	1701
Adcy7	632	434	385	660	269	667
Aldh4a1	5668	3408	2783	4778	2995	4667
Anapc13	1602	1414	1211	1446	1124	2127
Ang	155	120	94	175	57	169
Ankrd28	1817	1390	1189	1545	1279	1896
Arfrp1	2081	1288	1154	1685	1051	2202
Armcx3	318	259	151	306	157	313
Atad2	464	301	176	369	186	581
Atxn7l2	197	77	67	113	99	176
AW011738	455	328	299	368	333	435
B4galt5	521	364	316	467	290	545
Btbd10	643	407	338	562	377	660
C87436	530	360	242	456	307	539
C920006O11Rik	108	107	69	74	87	197
Ccdc14	293	194	163	220	147	337
Ccdc32	553	388	315	595	303	584
Ccdc71	844	534	429	747	485	841
Cdan1	862	494	365	646	455	860
Cenpa	1226	815	631	976	577	1369
Ckap5	3797	2702	2010	3207	2399	3321
Clic5	19353	14365	14194	19775	12465	19829
Col5a1	1890	1191	1037	1730	781	2567
Cpne2	202	152	95	191	108	236
Cpne4	4	2	3	0	3	5
Ctdnep1	3927	2337	1934	3242	1937	3580
Dcbld2	1488	1106	936	1612	870	1693

Diap3	23	10	11	10	6	45
Dvl1	1984	1068	885	1481	975	1672
Ercc2	518	339	243	451	298	515
Etnk2	16	8	5	11	6	20
Fam114a1	644	498	334	605	353	798
Fam122a	792	595	462	650	502	836
Fam96b	1008	666	560	762	615	1009
Fbxo10	992	634	380	802	499	753
Fbxo48	14	15	10	12	7	21
Fgf2	53	28	22	39	27	36
Fzd7	613	398	310	519	344	524
Galnt7	664	433	343	634	397	619
Golph3l	1175	750	667	995	622	1196
Gpr107	2012	1113	1075	1791	980	1695
Gpr110	6	1	1	4	1	6
Gpr158	14	14	8	22	8	16
Hyal2	866	509	430	773	455	849
Irs3	16	19	5	12	13	13
Klhl3	89	46	46	46	56	79
Klra7	7	2	2	4	0	6
Kpnb1	7609	5386	4394	7022	4485	7140
Limk1	279	164	123	250	133	278
Lpcat2	44	39	11	43	17	59
Lpcat4	122	65	47	98	49	90
Lrp3	496	339	201	368	213	524
Lrrc14	351	168	130	256	141	266
Mapkap1	2101	1429	1334	1896	1246	2155
Mettl18	191	127	100	146	90	212
Mir7646	25	28	26	23	24	35
Mir7650	6	3	1	3	2	4
Mpv17l	103	98	73	90	57	154
Mrpl39	3281	2273	1808	2656	2022	3194
Msr1	121	92	65	116	52	129
Myadm	11510	7480	5925	11455	5885	11739
Myrf	99	51	50	58	96	68
Ncf1	183	132	109	137	100	220
Nfkbib	712	474	340	554	367	710
Nr1h4	30	18	12	27	13	34
Nr2c1	573	384	322	483	363	637
Nudt3	7116	5179	3960	6392	3994	7227
Oard1	1016	696	586	833	676	1140
Ociad1	7286	4570	4517	6344	4058	7408
Ola1	2215	1781	1549	2019	1512	2552
Omd	93	95	63	114	63	85
Padi2	45	18	20	30	15	39

Pars2	220	147	110	162	126	212
Pcna	749	407	357	590	311	720
Pdhx	6333	4257	4525	5630	4044	6773
Pdrg1	3264	1912	2187	2713	1893	3428
Pelo	734	513	495	691	383	859
Phlpp2	603	366	332	475	304	626
Pik3cd	358	165	136	250	150	367
Pla1a	521	287	344	434	245	759
Plac8	17	6	9	23	2	18
Ppp1r3e	64	43	23	60	40	52
Prkci	998	646	557	858	560	946
Psma1	4746	3036	2922	3793	2677	5239
Psma5	755	583	464	603	468	894
Ptprz1	61	47	34	47	38	62
Ranbp9	2988	1943	1649	2593	1624	2903
Rbx1	2369	1418	1220	1877	1133	2426
Rilpl2	222	132	108	196	106	246
Rpl14	2849	2280	1791	2349	1727	3602
Rpl8	8857	5606	4991	7829	4885	10072
Rps16	285	242	200	301	164	441
Saysd1	148	81	72	123	75	158
Sec62	6502	4648	4131	5698	4050	8412
Setd1a	1581	1089	788	1326	940	1700
Sh3bp4	801	534	391	669	481	827
Ska1	6	4	1	5	0	11
Slc39a13	1038	755	520	898	547	1052
Smad2	1257	936	708	1146	742	1240
Snrnp200	5677	4026	2969	5005	3318	5097
Snrpd2	423	259	245	348	193	470
Srsf11	3739	2749	2069	2849	2169	4307
Ssb	3671	3215	2337	3154	2572	4296
St6galnac2	645	501	380	578	284	845
Stam2	1184	837	636	1051	742	1262
Taldo1	3010	1935	1793	2709	1783	3325
Timm8b	2505	1662	1878	2209	1514	2988
Tlr6	31	19	16	26	14	27
Tmed1	1078	546	508	865	474	914
Tmem42	273	178	166	242	151	267
Tnfaip8l2	42	23	16	46	10	47
Tnik	1761	1289	1262	1582	1430	1997
Tomm40l	2896	1701	1496	2664	1454	2937
Ttf1	700	407	381	565	376	642
Tulp3	521	319	258	411	294	442
Ubxn1	2717	2170	1674	2348	1900	3444
Usp36	1240	830	576	982	701	1094

Vamp3	3545	2132	2006	3075	1884	4046
Zbtb16	5976	3544	2552	5203	2427	5153
Zcchc5	58	43	23	60	38	48
Zfp661	162	111	87	126	89	166
Zfp791	123	91	83	122	60	139
Zmiz1	4803	3485	2101	4355	3081	4245
AA465934	372	25	28	21	179	36
Cdc34	3595	2363	2101	3011	1942	3876
Fam83f	3	0	1	0	2	1
Gal3st3	637	411	297	617	306	475
Gatad2b	621	534	332	572	476	440
Hsf3	3	0	1	0	2	1
Plin3	9319	5516	5670	7840	4764	9834
Tmem147	2000	1042	1042	1566	1005	2175
Ube2j1	2013	1320	1267	1960	1197	2225
Aars2	994	595	529	837	524	902
Abca8b	1530	993	793	1068	795	1745
Cyp27b1	6	3	0	3	3	6
Gnai3	1624	931	927	1489	817	1781
Ppp1r7	1673	1159	1010	1493	956	1750
Ranbp10	1990	1401	1038	1693	1143	1906
Tmod1	26074	18556	15204	23555	15091	25699
Snora41	3	2	4	4	0	10
Arel1	3432	2305	1933	2736	2175	3248
Npas3	5	7	1	6	2	10
Prr11	53	34	21	29	22	88
Bcdin3d	106	73	50	92	53	132
Cd2bp2	1814	1182	975	1615	1011	1964
Cs	74745	48799	42526	69337	39739	67802
Dap	523	331	322	492	296	602
Mrpl48	1161	714	620	978	681	1198
Nr1h5	3	1	0	0	2	1
Ntn4	470	292	261	395	292	494
Nup62-il4i1	24	5	10	25	6	18
Plcd3	585	408	263	464	310	553
Slc51a	25	20	7	19	9	24
Smyd2	4166	2995	2537	3911	2469	5078
Ablim2	905	630	466	647	701	900
Aco2	99075	66937	56334	90018	57078	87259
Arpp21	23	39	39	40	29	39
Bace2	222	167	128	176	106	374
C4a	12	7	5	5	7	11
Camk2g	2829	1860	1461	2591	1603	2855
Ccdc138	52	36	28	47	25	72
Cdca5	10	1	5	6	0	18

Cela1	6	5	8	8	4	14
Cers5	1243	732	632	1037	602	1418
Copb2	7630	5655	4534	6933	4696	7591
Csrp3	34768	21622	20647	29744	20116	41207
Ctbp1	7022	4043	3432	6016	3706	6868
D030045P18Rik	7	5	5	7	5	4
D330023K18Rik	79	27	41	46	38	63
Efna2	60	28	24	31	38	63
Efnb3	3924	2749	2342	3581	2965	3640
Ephx2	13539	8514	8866	11197	8359	17290
Fam13b	1076	684	528	941	616	1049
Fam53a	1008	624	524	868	567	1041
Fam83g	29	29	12	27	18	25
G3bp2	4441	3133	2672	4163	2497	4512
G6pdx	301	232	184	311	165	391
Gm10649	3	0	0	2	0	0
Got2	21739	14588	12250	20470	11867	19529
Gpr113	6	1	1	6	0	0
Hdlbp	32614	23016	17663	28299	21131	26079
Lrrc73	7	1	3	2	1	7
Mapre3	1717	1144	1015	1435	1011	1759
Mbtps1	2894	1841	1510	2588	1568	3038
Meis3	166	93	87	110	89	163
Mrps9	3870	2753	2549	3094	2463	4487
Mutyh	44	39	38	37	37	52
Mvd	144	91	64	125	83	140
Naa35	3189	1971	1679	2691	1757	2844
Nae1	1778	1319	1052	1524	1099	1878
Nelfcd	1279	748	665	1084	689	1324
Nhp2	416	271	277	336	225	498
Pcdhgc3	3428	2340	1781	3185	1764	3892
Pde4dip	108847	78997	59264	96852	71016	84873
Plin5	3963	1999	1904	2894	1848	3637
Pmp22	2268	1832	1289	2393	1155	2362
Polr2d	492	288	273	436	264	531
Ppapdc1b	404	298	239	319	226	481
Ppib	2063	1357	1295	2028	1052	2601
Rars2	887	692	590	729	600	1036
Riok2	1449	1022	968	1286	868	1638
Rpl31	145	80	83	145	60	173
Rpl3l	12458	7414	6281	8683	7235	11501
Rpl6	1903	1136	977	1585	992	2068
Spop	10524	6557	5790	8931	5559	10158
Srp68	3396	2171	1746	2728	1903	3185
Stk32b	55	38	22	63	22	27

Tcp11l2	7226	4509	3232	5517	3307	7228
Tmc4	63	33	32	32	39	57
Tubb6	629	384	348	602	234	656
Wdr45	1288	815	588	971	736	1363
Xab2	952	650	435	839	481	1054
Txndc11	659	410	374	611	331	610
Kank2	5302	3243	2932	4419	3201	4391
1300002E11Rik	368	296	275	294	270	454
Akr1b3	1472	618	713	1250	613	1397
Art3	15897	11849	9145	16259	9245	17998
Bola3	2719	2394	2165	2537	2009	3550
Btbd19	102	64	65	74	65	143
Ciart	340	239	120	284	187	332
Dlx5	8	6	3	7	2	14
Ethe1	924	538	513	707	453	991
Exoc7	1714	1117	880	1525	958	1777
Fam168b	3296	2055	1673	3038	1898	3016
Gm2011	8	1	2	1	6	7
Gm6644	1472	618	713	1250	613	1397
Izumo1	7	3	3	2	3	13
Kif24	22	14	8	12	9	23
Mettl17	596	345	300	508	339	572
Mir6963	7	3	5	6	3	11
Pcdhb3	29	10	3	8	16	25
Phrf1	1541	1078	640	1345	849	1521
Ribc1	14	14	7	14	15	14
Serp1	1681	1105	1002	1519	793	1894
St8sia2	40	25	10	31	17	43
Xaf1	1114	284	174	339	581	695
2310002F09Rik	14	7	1	10	7	10
2410018L13Rik	1	6	2	4	3	6
Ch25h	23	11	8	15	5	22
Chml	856	713	475	881	442	913
Efcab11	5	1	6	2	4	5
Gcat	417	232	146	292	188	333
Grhl1	59	45	43	53	30	75
Igj	40	62	92	123	62	82
Kcnk3	10206	6098	5614	9160	6364	9301
Nrxn2	166	104	89	171	73	191
Rassf5	111	47	43	98	40	101
Rbbp4	3699	2437	2066	3391	2186	3872
Sp110	312	223	160	220	182	433
Tmppe	449	294	270	409	255	534
Vwa1	635	389	389	566	268	732
Wdsub1	872	577	494	710	575	946

1110012L19Rik	369	232	183	333	188	397
1500011K16Rik	568	305	331	402	314	568
1700042O10Rik	3	0	1	3	0	0
2300009A05Rik	205	120	176	203	130	288
2900056M20Rik	306	232	150	203	231	362
4930507D05Rik	8	3	4	3	5	6
4930577N17Rik	94	51	42	75	48	98
4933433G19Rik	3	0	2	1	3	3
9330188P03Rik	22	12	15	11	15	23
Abhd11	1328	742	712	1078	731	1388
Acot1	298	89	144	107	162	367
Afap1l2	218	114	80	178	82	228
Ankrd12	2530	1872	1416	2072	1734	2884
Apbb3	501	310	201	383	258	523
Arid3a	148	86	87	141	81	159
Arih2	1963	1203	1068	1614	1228	1968
BC068157	4	0	0	1	0	2
Bcl2l2	2853	1820	1714	2621	1580	2739
Bend7	343	243	188	302	225	370
Brinp2	8	5	5	9	6	8
C1qtnf1	1162	767	561	971	574	1380
Camkk2	634	460	390	615	299	731
Ccdc15	101	83	49	81	68	89
Cdk3-ps	14	5	5	9	4	18
Cep44	392	212	183	268	191	370
Cep55	28	14	13	11	7	59
Cfd	43	67	29	65	32	48
Chst4	4	0	5	3	4	0
Col1a2	5880	3740	3278	5544	2307	8226
Col4a4	335	227	212	239	202	507
Crocc	329	198	104	194	179	251
Cryz	459	252	283	438	235	508
Ctu2	198	78	80	147	88	176
Cyp4f13	348	213	158	229	213	387
D830015G02Rik	3619	2841	1519	1759	2340	4117
Ddx49	708	425	339	577	375	745
Dgcr2	2572	1460	1252	2520	1257	2278
Dpy19l4	681	498	380	580	431	646
Dusp28	493	293	259	442	299	450
E030011O05Rik	54	32	50	58	35	49
Egr3	71	62	18	67	32	49
Epcam	3	1	1	2	2	3
Epha3	14	5	5	11	6	5
Epm2aip1	3937	2560	1926	3294	2190	3408
Fam178b	2	1	2	1	4	2

Fam198b	2908	1878	2032	2539	1658	4101
Fam65a	4007	2882	1793	3557	2273	4103
Fbxo25	643	403	321	549	368	650
Foxred2	54	39	29	48	24	56
Fxyd2	32	32	30	29	29	40
Gap43	11	4	1	11	1	9
Gareml	9	4	5	10	5	8
Glt8d1	1242	816	737	947	695	1419
Gm10406	3	4	4	1	4	11
Gm16907	25	10	15	18	8	25
Gm6981	12	11	9	18	7	16
Gnl1	1484	944	739	1236	826	1568
Golga7	961	481	451	847	441	887
Gpc1	16497	9803	8239	14647	9325	11221
Gpr123	1	5	1	2	4	4
Gsdmc4	4	2	3	4	3	1
Gsk3b	4708	3300	2634	4272	2822	4251
Gstp1	776	436	380	667	309	741
Hhat	110	48	47	69	55	81
Hic2	198	110	108	137	126	224
Hoxb4	90	47	43	51	56	107
Ilvbl	826	450	442	628	497	835
Ing5	824	479	423	600	460	768
Inha	198	116	82	124	86	207
Kdm2b	410	244	195	340	202	351
Klra5	4	0	0	1	0	2
Lamtor1	2134	1217	1209	1881	1113	2326
Lphn3	73	58	20	61	30	61
Mamld1	249	213	131	201	172	248
2-Mar	3106	1999	1768	2707	1721	3594
Mbtps2	947	615	509	838	504	884
Mdm4	1873	1207	935	1318	1164	2121
Megf10	60	56	35	76	22	63
Mir1903	36	14	21	40	16	31
Mir568	163	75	145	131	93	190
Mir7-1	4	10	4	2	4	12
Morn2	232	132	156	183	141	228
Mrpl2	2414	1425	1304	1890	1326	2751
Mrpl32	1217	932	890	1040	826	1486
Mul1	2300	1395	1314	2067	1240	2072
Myl6b	72	29	32	58	43	52
Nabp2	1043	858	657	995	760	1223
Nap1l2	5	1	1	2	2	2
Ncf2	118	75	69	102	66	147
Ndufa1	3854	2303	2516	3460	2198	4627

Ndufs7	8409	4761	4145	6839	4273	8855
Nol9	619	402	319	545	313	578
Nudt16l1	480	230	225	396	218	470
Nudt17	91	66	56	67	51	109
Olfr921	4	1	0	0	0	4
Pcbd1	23	18	14	22	9	26
Pcdhb7	60	24	21	26	44	49
Pfn4	89	67	43	75	56	106
Pigz	41	27	20	29	25	53
Pin1	1118	657	584	944	588	1195
Ppifos	4	0	1	0	1	3
Ppm1d	624	461	369	553	385	776
Ppp6r2	2611	1732	1312	2077	1539	2368
Prr5l	59	25	19	39	26	57
Psmb1	4229	2628	2474	3613	2358	4975
Psmd2	8748	5757	4703	7228	4806	8691
Ptges2	1499	898	814	1244	794	1417
Rab3gap2	1861	1095	993	1431	1023	1781
Rexo1	1064	673	459	932	581	971
Rgs7bp	457	287	260	385	264	417
Rnf220	1233	823	629	1084	689	1322
Rock1	8479	5775	4084	6720	4882	7745
Rpl32	3669	2485	2511	3228	2060	4371
Rpl34	690	863	680	535	357	1776
Rpl34-ps1	684	861	676	533	355	1773
Rps15a	1829	895	1184	1460	927	1864
Rps24	5677	4438	3623	5242	3624	7522
Scd4	105	48	116	29	162	137
Scn7a	2567	1606	1369	2184	1201	2684
Scyl3	665	408	351	511	357	807
Sec16b	207	111	97	154	112	217
Sema6d	2141	1464	1096	1911	1376	2021
Setdb1	1359	810	629	1029	686	1261
Sharpin	1011	600	479	804	510	1103
Slc30a3	4	0	1	3	1	3
Slc7a5	268	137	129	222	132	194
Smarca2	4546	3465	2803	4350	3246	4856
Smarcd1	1744	1102	886	1463	983	1506
Snapin	3071	2247	2148	2806	2238	3675
Snf8	742	493	491	644	461	946
Snhg12	128	86	75	104	72	130
Specc1l	3558	2524	2033	3248	2180	3229
Spns1	908	501	368	750	443	820
Spopl	595	372	280	532	309	598
Ssh1	253	200	166	257	182	289

St6gal1	511	335	307	434	305	621
Stbd1	355	343	372	419	292	479
Stk16	961	576	482	878	494	952
Stx18	577	435	368	546	334	630
Tbc1d15	2111	1388	1125	1730	1168	2092
Tbc1d24	1520	968	864	1285	903	1394
Tceb3	2577	1815	1523	2347	1493	2598
Tfap4	135	100	64	108	88	156
Ticam1	598	359	224	468	262	498
Tln2	6180	4289	3316	5801	3775	4737
Tmem101	456	276	258	413	216	440
Tnip3	184	141	99	163	134	196
Tnni3k	6949	4346	3555	6446	3694	6999
Tpgs2	487	396	316	462	351	598
Trim72	12425	5623	7043	10644	6168	9281
Trp53	540	339	272	437	255	687
Tstd3	1195	884	983	1141	787	1489
Usp39	883	562	509	792	459	871
Wdr44	844	631	441	742	475	848
Yipf6	1716	1131	1056	1503	945	1845
Zbtb2	411	278	212	419	214	424
Zfp317	1435	963	772	1164	827	1421
Zfp748	407	237	218	356	232	407
Zfp784	295	178	129	183	197	305
Zfp811	18	18	32	21	21	30
Zpbp	60	30	35	52	34	64
Adamts3	300	212	177	246	179	315
Ermard	264	181	180	217	170	366
Pfkfb4	144	77	85	95	77	151
Prpf31	740	579	402	603	469	772
9530080O11Rik	3	3	3	2	6	4
R3hcc1l	845	518	438	724	482	858
Trim56	638	383	339	463	382	587
Ascc3	1019	736	581	948	621	928
Cxxc1	1740	1164	796	1366	1003	1847
Dhh	23	24	12	24	14	22
Eri2	201	140	151	162	145	291
Gpr153	836	526	383	808	298	775
Hoxa7	3	5	9	8	5	11
Ilkap	1526	1082	945	1294	865	1771
Nid1	9505	6241	5780	9875	4563	11595
Nkapl	30	20	22	17	21	36
Nubpl	365	231	182	290	207	408
Ost4	710	525	500	669	428	820
Pola1	336	257	198	319	173	372

Rab33b	820	551	436	647	457	845
Rpn2	3493	2224	1923	3132	1724	3502
Thrsp	1789	1118	1244	1711	686	3011
Zfpm1	890	563	401	820	472	820
Armc8	1716	1163	973	1468	1021	1662
Lef1	3	9	3	8	7	5
Prkaca	11909	8055	6262	11271	6863	12044
Psat1	116	45	100	69	66	143
Tagap	16	13	12	19	9	24
Tfpi2	65	36	50	50	39	70
Zfp592	1929	1300	990	1902	1080	1858
S100b	58	45	42	40	31	86
0610043K17Rik	22	18	17	14	22	35
1810062O18Rik	5	2	6	4	7	6
2310039H08Rik	261	153	131	174	118	295
4632428C04Rik	225	136	130	150	160	190
5730508B09Rik	182	122	127	153	120	247
9530051G07Rik	7	15	6	9	8	11
9930104L06Rik	361	250	176	324	202	389
Acn9	212	129	160	179	112	260
Adamtsl2	500	313	206	597	121	345
Aldh3b2	2	0	0	0	2	1
Amz2	2606	1692	1515	2157	1477	2694
Anp32a	5739	4594	3969	5440	4140	7348
Anpep	685	537	309	677	305	665
Armc7	77	29	46	50	41	94
B230216G23Rik	6	5	7	6	7	4
BC053749	147	115	62	115	77	148
Bdnf	418	235	312	231	392	307
Cbfa2t2	1104	725	593	955	640	986
Ccar2	1152	759	555	958	664	1190
Ccl27a	67	40	32	39	26	82
Cd151	6372	3703	3306	5390	3488	6447
Cd33	99	61	43	82	38	93
Cdc25b	208	135	103	174	94	209
Cdpf1	393	198	199	261	210	354
Cdr2l	313	218	158	258	152	334
Cdyl2	336	184	157	283	209	267
Ckmt2	95351	62926	60522	87544	56436	117649
Clcc1	1878	1211	1094	1606	1102	1801
Clec4d	15	7	17	17	8	15
Cog2	906	538	457	692	456	900
Coq4	614	298	279	432	269	556
Cox7b	22830	16750	15549	19900	14048	26807
Crls1	1037	675	662	785	737	981

Cyfip1	2834	1754	1468	2509	1382	2662
D130017N08Rik	83	65	40	73	63	83
D6Ertd527e	2	0	0	2	1	0
Dad1	1387	958	692	1278	752	1480
Dalrd3	1096	642	485	817	532	1016
Dbi	3037	2025	2335	2742	1854	4475
Dcaf6	5721	4047	3306	4791	3725	5362
Dctn1	6164	4375	3095	5190	3699	5473
Dctn5	1247	753	691	1079	728	1275
Dennd6b	888	489	399	563	497	973
Dera	205	118	112	169	116	225
Dhx34	450	250	190	368	192	372
Dicer1	2771	1897	1484	2231	1629	2761
Dnaic2	5	7	1	3	4	5
Dnmt3b	110	55	41	63	63	112
Dtx3l	1534	1013	919	1396	928	1685
Dynlt1c	33	22	19	21	20	35
Ears2	354	225	169	257	208	315
Epha7	323	276	194	323	203	326
Erap1	2442	1596	1377	2194	1393	2227
Eya1	60	59	43	74	43	82
Fads3	3006	1580	1468	2376	1717	2672
Fam160a2	1111	717	597	928	629	1044
Fam207a	486	348	261	429	288	562
Fkbp11	36	18	22	24	31	35
Gabarapl1	4660	3163	2867	3957	2898	5681
Gamt	116	85	73	113	61	163
Gas1	1231	834	629	1156	634	1099
Gchfr	68	51	36	75	36	76
Hcn3	7	7	2	5	4	12
Hist1h3g	2	1	2	1	3	3
Hist2h3c1	4	1	1	6	2	0
Hist2h3c2	4	1	1	6	2	0
Hsd11b2	5	1	1	3	0	7
Ifng	0	1	2	4	0	1
Igf2os	7	8	2	2	5	9
Igsf3	670	468	354	669	332	600
Il2rb	47	21	8	40	4	29
Kazald1	92	46	48	59	39	98
Kcnd3os	29	12	14	17	22	26
Kctd5	425	276	244	359	250	498
Kdm4b	1656	1047	825	1543	940	1515
Klre1	7	3	1	4	5	5
Lax1	8	11	13	12	9	22
Lix1	153	126	113	125	116	177

Lrrc24	76	30	33	61	44	54
Lrrn3	27	29	21	25	22	33
Ly6g6d	5	5	8	9	5	10
Ly96	66	42	67	81	34	76
Madd	706	439	282	532	424	638
Mapk4	193	129	128	146	241	102
Mapk8ip2	3	2	1	3	4	1
5-Mar	4384	2959	2739	3891	2416	4825
Mcm3ap	2125	1256	906	1525	1052	1954
Mcm7	380	228	195	307	198	438
Mdh2	46826	28503	26958	43173	25939	50817
Med28	1071	578	520	861	429	1064
Meis2	1079	823	702	962	910	1086
Mettl23	856	455	472	697	370	877
Mir143	2	0	1	0	0	5
Mir1930	2	0	0	0	2	1
Mir6950	10	6	7	7	4	12
Mirlet7e	4	1	1	4	3	1
Mrpl52	472	307	344	395	284	554
Mrps18a	1833	1079	1125	1433	1216	1984
Msra	1684	1025	885	1418	985	1682
Mtbp	148	95	101	106	86	238
N6amt1	1152	927	749	875	756	1485
Nacad	43	22	19	33	23	27
Ndufa12	4136	2822	2668	3502	2250	4981
Nfe2l1	26537	16794	14535	22074	15722	23135
Nfkbid	17	5	6	8	6	13
Nme2	7678	4080	4724	6602	3969	8992
Nsdhl	266	252	173	297	182	352
Ocln	23	15	12	13	12	26
Orai1	467	275	183	399	226	432
Pafah1b2	2125	1318	1235	1923	1168	2359
Papd7	947	637	578	909	546	880
Parp1	2854	1881	1515	2580	1597	2484
Paxip1	1006	712	516	803	560	1056
Pcsk5	136	115	102	145	88	201
Phactr3	31	21	18	29	15	28
Pla2g7	236	162	147	218	130	240
Poln	75	40	42	45	41	77
Polr2b	3455	2411	1854	3089	1996	3203
Polr3gl	1168	874	700	972	695	1346
Pot1a	688	453	330	574	381	741
Prdx6	8985	5187	5470	7216	4616	9731
Prpf19	10532	6163	5285	8948	5930	10045
Psmd7	4693	3415	2873	3706	3074	5091

Ptprcap	5	2	5	5	3	8
Rab40c	621	299	224	433	292	564
Rad54l	12	8	8	8	7	13
Retsat	1813	982	1031	1141	1213	1584
Rgs4	1158	543	565	922	476	1020
Rhou	423	213	216	353	209	325
Robo1	175	116	68	175	79	168
Rpsa	1794	1445	927	1463	1118	1892
Rpusd4	624	399	371	558	352	729
Rusc1	693	457	362	618	400	723
Scamp1	2538	1572	1409	2276	1324	2366
Sec23a	2968	2100	1686	2764	1803	2653
10-Sep	749	499	477	598	484	951
Sertad4	327	231	142	314	134	297
Setd6	532	336	267	431	312	562
Sh2d1b1	4	3	2	4	2	6
Slc31a2	1039	545	661	1173	472	1049
Slc35a2	459	251	224	382	212	502
Slc35d3	4	3	2	4	2	6
Slc39a8	57	47	46	63	39	56
Slc7a2	321	248	195	334	180	309
Slc7a3	3	5	4	6	4	6
Smarca5	2225	1399	1221	1832	1260	2114
Snord45c	14	6	8	8	5	15
Snx7	369	284	225	349	247	434
Ssu72	1151	826	761	1008	717	1282
Stag2	4110	2782	2177	3674	2416	4316
Syn3	42	22	18	26	33	36
Syp	124	63	64	85	69	143
Tada2b	1064	619	623	944	626	859
Taf13	571	415	373	509	381	712
Tbcc	365	218	185	318	194	380
Tc2n	10	10	7	10	6	11
Tdrd9	4	12	4	5	5	19
Tecta	4	2	1	3	5	1
Tfdp1	868	537	484	718	475	844
Tgm7	10	7	6	5	7	18
Tmem80	534	292	264	353	301	609
Tnfrsf18	5	0	1	4	2	2
Trim11	765	570	361	660	467	821
Tubb5	4255	2759	1976	3885	2006	4603
Ube2m	4700	3620	3108	4275	3352	5751
Ubn2	3357	2049	1698	2363	1900	3330
Usf2	2248	1348	1170	1795	1271	1990
Usp1	1027	768	591	987	617	1177

Usp53	480	378	229	388	335	384
Vbp1	2469	1474	1487	2030	1263	2683
Vps37b	885	521	372	726	471	807
Vps4a	2862	1854	1597	2423	1586	2834
Zfp609	2012	1500	1026	1704	1344	1651
Zfp839	600	398	352	473	336	660
Acadvl	37198	20378	20074	27990	23872	35881
Atf6b	596	355	297	518	334	573
B3galt4	54	41	25	36	36	73
Bambi	668	428	366	614	384	541
Brca1	35	29	12	17	14	70
Cdc16	1775	1097	907	1389	953	1728
Dcaf17	623	396	376	447	370	693
Dcxr	267	149	113	197	132	285
Dexi	815	427	430	735	357	881
Echdc2	1145	650	549	723	740	1171
Eid2	376	236	203	310	214	427
Fbxl4	584	382	304	486	353	620
Flrt1	23	21	18	23	21	18
Fuk	259	161	113	208	122	231
H2afy2	235	166	130	173	144	248
Herc3	1946	1434	1058	1891	1283	1874
Il1rn	3	7	8	4	3	12
Mcts1	964	597	653	786	532	1136
Mrpl4	2440	1489	1269	2069	1305	2611
Ms4a1	2	3	6	5	2	9
Mtg1	871	536	497	697	497	857
Ntn3	39	28	21	27	32	27
Nup188	989	605	406	817	496	759
Paxbp1	2114	1391	1122	1534	1173	2319
Pcdhb13	22	16	9	24	13	20
Pdcl	1044	610	510	857	517	1129
Pitpnb	2974	2213	1590	2652	1902	3196
Pomgnt1	2610	1611	1445	2392	1539	2524
Rbms3	907	627	493	952	462	965
Rcor3	1969	1360	1184	1536	1243	2112
Romo1	1100	608	609	850	600	1237
Setx	3539	2308	1965	3054	2057	3282
Slc16a1	17446	11734	10630	15869	10758	15839
Slc25a24	598	393	293	460	272	668
Slc44a1	1512	967	754	1336	719	1446
Sp4	1237	832	658	986	816	1294
Spcs2	1774	1094	928	1520	959	1885
Sv2b	44	34	44	50	33	71
Tspyl2	969	795	576	678	649	1219

Ttc30b	954	641	562	776	538	1048
Txn2	6432	4053	3488	5314	3325	6553
Zbtb44	2375	1337	1321	1924	1354	2492
Zbtb8os	229	145	164	183	120	291
Asprv1	1	2	2	2	3	2
Atp8a1	3881	2346	2044	3138	2513	3611
Dhdh	1102	721	750	1003	865	1061
Gm8221	2	0	2	2	0	4
Sgce	777	565	409	712	396	975
Shb	993	653	504	933	494	879
2610507B11Rik	23716	16912	14967	21375	15912	21672
4933421O10Rik	175	127	130	128	128	206
Ago3	553	281	264	469	242	445
Atg14	742	461	401	587	461	787
Calhm2	189	105	74	198	72	163
Ccpg1os	158	116	77	105	123	177
Cox8a	1993	1373	1223	1769	1108	2186
Dnajc19	605	459	395	505	368	736
Frmd4a	1626	1187	941	1330	999	1760
Gm16515	564	334	303	498	296	603
Gmip	212	153	105	188	110	262
Gxylt2	71	40	18	61	24	41
Hnrnpr	2034	1375	1243	1731	1201	2154
Ipcef1	15	7	5	15	4	15
Lrrc25	23	15	23	26	13	25
Mfsd7a	153	109	87	119	88	166
Nlrc3	47	25	26	31	37	47
Nradd	26	16	15	16	16	39
Pik3c3	1132	866	645	1187	716	1146
Ptp4a1	1159	678	687	792	705	1211
Rnf13	5769	4123	3431	5207	3451	5793
Rps15	6457	4419	4257	5871	3981	8046
3-Sep	64	25	28	49	24	45
Sgsm3	457	276	175	364	210	368
Slc24a5	16	13	2	6	6	18
Slc25a4	186634	107021	101993	160272	97629	208522
Slc39a12	1	2	2	2	2	3
Smap1	981	699	547	846	566	1195
Spock3	4	0	0	2	0	1
Tbp	466	361	267	375	320	466
Thtpa	780	492	473	658	480	727
Tmem259	3762	2128	1743	3037	1971	3605
Tubb4a	116	73	81	87	62	144
Zfc3h1	2052	1352	1179	1567	1311	2421
Zfp169	282	167	130	198	171	294

2210408F21Rik	599	354	358	472	298	667
Aim2	192	93	101	86	120	185
Amn1	275	171	153	230	137	350
Bgn	9585	7587	5794	11503	4351	12519
Cyp4b1-ps2	3	2	0	2	2	0
D830046C22Rik	66	49	36	45	52	54
Fam105a	102	50	58	99	35	128
Foxc1	355	321	187	284	239	511
Mir6367	943	502	459	803	496	858
Ndufs6	6838	5160	5557	6159	4513	9079
Pacrgl	143	71	62	115	72	132
Pigk	1497	972	830	1329	780	1487
Rbpj	1067	658	577	880	660	1074
Rlf	1066	794	777	925	691	1313
Rnf10	16127	9968	8726	13995	8659	14584
Rnf135	279	145	146	247	143	271
Rnf169	990	663	646	794	646	1266
Trim40	6	4	4	0	5	8
Gm6225	13	2	4	1	12	9
Kansl3	3974	2700	2174	3447	2349	3675
Mas1	54	37	27	31	32	59
Rnase6	15	12	8	24	7	13
Slfn8	112	129	135	194	49	295
Klk7	11	6	4	10	3	14
Rgs18	60	49	27	46	43	47
Ttc30a2	20	6	5	15	9	13
4930432K21Rik	14	12	5	6	14	18
Aldh7a1	1321	991	876	1382	906	1409
Atpif1	4112	3685	2852	3720	2242	6236
Dennd1b	1546	967	959	1256	957	1535
Dnajc21	2385	1825	1591	1984	1739	2524
Dock11	375	218	176	319	169	417
Egflam	918	638	548	849	477	995
Eif3l	3281	2223	1888	2942	1795	3363
Fgd1	501	370	268	495	296	532
Gm6034	4	0	1	0	2	5
Gpr125	1197	799	716	1086	726	1100
M6pr	2445	1529	1429	2084	1365	2423
Mir1668	124	64	54	93	49	111
Rab9	973	715	599	873	618	1182
Scfd2	440	309	252	389	228	477
Sec24c	4596	2773	2206	3818	2287	4125
Smarca5-ps	14	13	7	10	13	19
Spry2	890	630	440	836	470	973
Tada1	395	255	216	357	224	426

Tmcc2	1715	964	810	1390	885	1395
Tmem206	288	183	157	241	159	269
Ttc37	766	491	401	726	423	590
Unc13c	5	7	6	15	5	4
Zfp518b	337	281	255	382	256	397
Zfpl1	354	230	199	292	186	369
Zscan18	60	62	49	60	39	88
Il1rl1	72	38	36	51	31	71
5730405O15Rik	13	11	9	9	13	10
Paip1	4355	2974	2821	3492	2829	4712
Scd1	983	1628	680	2643	594	1050
Slc2a6	6	0	1	3	3	3
Ube4a	2720	1692	1370	2208	1494	2467
Ccdc124	1387	910	769	1126	838	1568
Pwp1	797	598	496	697	493	879
Abcd2	548	432	326	575	272	592
4732416N19Rik	1	3	1	3	2	2
D17Wsu104e	460	279	288	461	212	448
Itpkc	233	151	138	199	133	226
Mrps27	2134	1473	1242	1840	1220	2244
Pxylp1	329	229	164	311	161	299
Sstr4	66	30	18	57	23	28
Wdr47	710	442	397	648	396	749
Ccdc148	6	1	1	2	5	3
Gm10125	8	8	3	5	2	12
Fbxo32	11269	7617	8108	7307	7748	14397
0610010K14Rik	439	363	233	409	257	561
1600029O15Rik	1	1	4	1	0	5
1700108F19Rik	4	2	0	4	2	2
1810062G17Rik	12	8	5	8	4	13
2410137M14Rik	14	8	11	8	14	19
9330158H04Rik	441	432	353	482	263	652
A730046J19Rik	1	0	4	1	6	0
Acap1	38	20	15	38	17	32
Acly	2175	1291	1061	1776	1159	2225
Adam19	925	600	592	653	934	729
Akap7	1895	1505	1121	1540	1280	2071
Akr1b10	2147	1476	1229	2076	1273	2241
Alg14	273	197	164	239	152	299
Armcx6	34	24	11	28	10	33
Arrb2	450	297	223	383	248	492
Atxn3	1020	612	585	854	566	1155
Bclaf1	6104	4553	3702	5187	3834	6622
Brms1	490	349	247	371	287	504
Ccl25	28	37	36	44	26	39

Cdc37	5239	3791	3066	4696	3380	5900
Cdca7	50	38	36	45	27	61
Cdh6	19	11	2	17	9	12
Cdk5	820	434	408	602	426	886
Cenpj	408	269	232	357	242	367
Ces1d	7679	3694	2481	4275	3801	5622
Ciita	86	46	25	97	30	57
Cnep1r1	2146	1372	1253	1802	1212	2183
Cops6	3622	2436	2068	3263	2035	4177
Coq5	5992	4035	3836	5127	3876	5928
Cpt1a	3182	1970	1716	2468	1764	3727
Cpxm1	338	202	146	276	130	330
Crhr2	1209	713	569	854	579	1259
Cyb5r1	972	611	556	818	515	1003
Denr	2441	1941	1649	2092	1605	2988
Dgkk	3	1	2	2	3	0
Ell	667	431	335	513	413	710
Emb	48	50	26	45	22	67
Emc8	892	577	486	768	477	873
Epb4.1	7934	6170	4592	6678	5073	8512
Eps15l1	2394	1794	1281	1878	1522	2468
F8	185	132	134	180	120	184
Fam117a	236	155	84	168	130	248
Frat1	92	50	61	64	63	116
Galnt4	447	259	244	382	207	544
Gas2l1	681	357	313	545	321	553
Gdi2	11260	7840	6562	10105	7186	12280
Glipr1	27	18	13	21	11	28
Gm13363	330	191	204	258	215	286
Gm5512	1029	1002	928	1021	739	1627
Gorab	207	126	111	172	113	237
H2-Oa	3	1	7	8	0	3
Hars2	1359	907	718	1153	739	1340
Hinfp	209	135	118	175	115	259
Hnrnpab	7495	5038	4515	6257	4562	7676
Hsf2bp	9	6	12	18	8	9
Hsf4	177	109	80	111	101	168
Hspa14	1004	655	561	822	532	1078
Kcna3	1	3	1	2	2	3
Kifc3	3689	2211	2040	3437	1760	3436
Klrb1c	20	5	1	11	2	10
Lrrc57	986	732	634	879	628	1067
Lsm1	349	228	223	316	201	348
Mapk11	143	79	51	104	61	115
Mcmbp	1713	1121	855	1599	928	1687

Mien1	836	414	508	740	388	971
Minpp1	701	475	379	665	354	663
Mir133a-1	29	15	20	11	17	38
Mir7225	3664	2997	1866	2890	2472	3653
Mmp27	4	6	11	11	4	15
Mrpl17	1454	913	852	1216	818	1468
Mrpl9	2974	1834	1831	2547	1613	3154
Msh3	624	468	406	525	481	726
Nde1	1668	1061	1010	1577	940	1834
Nptn	5486	3495	3242	5197	2979	6148
P2ry2	1117	625	600	1076	621	961
Parvb	4743	3079	2619	4491	2469	4321
Pbxip1	5982	4036	3349	5245	3722	6356
Plxdc2	1614	1385	851	1797	738	1710
Poglut1	790	490	438	697	436	858
Ppp2r5d	1935	1242	975	1802	1102	1803
Prox2	50	41	26	42	41	55
Pura	399	323	262	434	241	501
Rev1	1782	1113	891	1471	957	1625
Rnf24	285	180	205	253	209	322
Rrp7a	767	523	419	667	446	720
Rspo3	17	14	14	16	13	17
Sdhb	31824	19126	18477	27423	18008	35629
Sdhc	18460	9923	9287	15698	9584	17694
Sec61b	171	87	106	140	73	181
Sgk3	1148	783	554	954	607	1115
Shfm1	2534	1864	1872	2501	1661	3314
Slc29a2	426	251	172	383	191	299
Slc39a9	1490	980	828	1376	818	1365
Smc4	1953	1326	1091	1450	1215	2419
Smek2	2228	1545	1307	1929	1469	2418
Smpd5	14	12	5	14	10	14
Smpdl3b	38	35	31	45	23	43
Snora24	5	5	5	7	1	7
Snord57	3	2	3	2	2	7
Snx2	1669	1105	909	1327	1046	1836
Srfbp1	424	324	266	392	298	382
Stau1	3287	2642	1995	3067	2093	3493
Stxbp5l	1	2	2	3	1	3
Tbx20	6498	5158	4341	7078	4420	5546
Thoc2	3775	2532	2065	2933	2254	4533
Tlr11	11	10	4	20	4	8
Tmem43	1316	845	709	1175	641	1322
Tppp3	707	427	313	568	360	731
Trappc3	1487	941	896	1323	854	1691

Trim35	4573	2944	2493	4333	2784	4229
Vcl	8831	6231	4879	8556	5195	7401
Wasf3	46	31	14	38	28	40
Wdr77	1937	1215	1106	1677	1024	1947
Xlr4a	19	16	2	6	7	23
Zdhhc16	487	302	273	407	265	573
Zfp652os	10	2	1	4	5	7
Zfp931	132	119	111	125	98	173
Zfp960	117	104	79	104	94	161
Zp2	20	7	8	10	13	19
Ano3	18	18	10	25	11	21
Ncald	519	324	246	464	233	587
Rab15	35	28	11	30	18	26
Zfp281	1274	824	688	1103	672	1251
AU019823	656	505	453	606	402	782
Atp5l	78	36	42	50	42	95
4930402H24Rik	3473	2362	1691	2639	2037	3352
Abhd11os	1	1	3	2	3	2
Alg8	183	103	89	137	95	161
Atg2a	2281	1178	1032	1780	1044	1930
Bbs7	369	297	205	326	231	378
Bptf	3671	2914	2148	3243	2521	3565
Ccdc77	383	282	227	312	235	419
Cd3e	7	11	8	14	4	17
Chrdl1	34	26	15	39	19	32
Elf2	3046	2020	1627	2849	1730	3072
Eml1	4732	2921	2577	4222	2434	4415
Hdac4	798	481	358	554	489	632
Hyal1	327	195	147	267	199	287
Mex3c	1181	739	671	1037	684	1261
Mir5101	6	3	4	3	9	4
Mrpl46	1094	765	682	905	644	1250
Mrpl49	1629	1148	1064	1532	950	1765
Myo18b	19099	12992	10291	16611	11674	16331
Ncapg2	193	144	109	142	106	300
Nwd1	21	10	5	17	10	16
Pigb	105	102	79	109	83	156
Pou4f1	18	7	5	14	9	13
Rnf14	5297	3257	2878	4305	2960	5018
Snx30	591	393	316	437	403	507
Sost	26	21	6	11	9	34
Spr	1929	1116	901	1714	740	1837
Stk32c	67	35	28	60	29	65
Thbd	4888	2888	2009	4523	2212	4417
Tspan33	72	32	44	59	27	71

Uimc1	946	707	579	772	646	1183
Uprt	38	51	23	62	21	34
Usp2	8311	6027	4578	7567	4408	8782
Vps9d1	1055	654	505	847	548	974
Zrsr1	1620	1248	844	1320	1145	1745
1700025G04Rik	2744	1962	1838	2655	1787	2669
A830010M20Rik	288	219	225	226	194	390
Anln	111	75	59	59	45	219
Atp10a	116	80	80	143	79	104
Bmx	150	125	92	91	97	209
Chmp7	2164	1268	1194	1783	1077	2192
Dag1	22418	15267	12969	22400	13299	17615
Fam195a	2582	1429	1374	2018	1247	2596
Gsr	984	578	597	825	538	988
Guca1a	2	3	0	5	1	1
Hrh1	8	9	12	14	3	14
Lepr	445	202	100	253	169	308
Mir7578	28	17	23	26	25	31
Nqo2	2233	1470	1366	2093	1324	2056
Pex11g	109	49	51	62	50	105
Sec61g	4	7	5	4	4	8
Ttll12	335	264	189	329	215	375
Ak1	24247	16107	13161	21234	13214	23191
Cilp2	44	18	33	56	28	30
Drp2	18	22	12	18	10	27
Dscc1	3	2	3	1	1	10
Gemin6	116	62	74	72	67	128
Mir8101	2	0	3	4	2	1
Sh3rf3	61	37	29	52	25	56
Dgkd	2504	1547	1304	2110	1460	2500
1700028J19Rik	17	14	10	16	5	33
1810011H11Rik	12	6	12	11	11	7
2310035C23Rik	1177	684	610	958	655	1202
2610301B20Rik	544	350	302	448	273	587
2810403A07Rik	2855	1976	1672	2372	1935	3098
4930415F15Rik	9	7	3	4	9	4
4930546K05Rik	2	0	0	2	0	1
4933402J15Rik	1	1	0	0	2	1
4933408B17Rik	26	22	14	10	17	54
9430016H08Rik	811	568	584	713	581	1009
Ackr4	181	142	146	158	75	309
Ahctf1	2991	1999	1610	2523	1710	2908
Akap12	3012	2118	1755	2649	1737	3170
Ampd2	293	153	138	203	146	332
Angptl1	201	139	123	192	103	208

Ankrd34a	62	57	50	69	42	71
Arntl2	228	129	126	150	121	246
Arpp19	2829	1850	2031	2511	1673	3309
Asb15	5584	4133	2732	4441	3958	4208
Atf2	2355	1672	1300	2065	1414	2268
Atp2c2	4	0	0	1	3	1
B4galt3	598	351	261	431	299	551
Bbs12	74	69	57	71	51	95
C330006A16Rik	3094	1938	1678	2719	1787	3132
C630031E19Rik	1	1	0	2	1	0
Capzb	10142	7495	5839	8882	6610	11627
Ccdc136	118	76	56	107	52	105
Ccdc177	1	0	1	1	1	1
Ccdc57	89	99	37	86	50	102
Ccdc6	1739	1229	1058	1506	1131	1708
Ccdc88a	776	620	454	775	438	982
Cdip1	3248	1999	1870	2928	1673	3194
Cebpb	994	623	489	729	576	1104
Cenpm	7	5	1	3	3	11
Cetn2	922	693	622	924	606	1088
Cgnl1	2924	2355	1593	2273	2033	3633
Chd1l	978	615	478	831	590	904
Chrnb1	318	183	188	231	153	371
Cisd1	5899	3493	3493	5134	3124	6883
Clec4a3	78	58	43	64	28	104
Cox10	3552	2270	2036	3079	1921	3611
Cpe	11768	7838	6667	11431	6811	12055
Crk	5966	3574	3304	5181	3168	5503
Cxcl13	5	3	16	12	8	4
D17H6S53E	681	376	362	670	346	616
D830013O20Rik	58	56	47	70	59	62
Dap3	4192	2561	2275	3630	2316	4427
Dapk1	2135	1425	1051	1577	1478	1628
Dos	86	66	30	83	46	84
Dpf3	428	283	219	334	279	349
Dph3	1504	926	901	1340	866	1648
Dtnb	90	73	49	77	51	98
Dusp7	2411	1643	1311	2311	1295	2189
E030003E18Rik	57	56	45	61	32	81
E230016K23Rik	1	1	0	1	1	1
E330009J07Rik	37	26	24	33	22	36
Enpp5	1088	792	657	1056	683	1200
Ep300	3927	2856	2079	3413	2455	3542
Epb4.1l3	561	437	372	555	395	646
Epor	61	32	30	42	26	83

Eri3	1774	1138	910	1462	995	1917
Erich6	11	5	8	8	8	14
Faim3	4	6	9	7	9	9
Fam149a	259	178	149	244	148	295
Fam86	377	276	232	325	254	454
Fbxl21	17	9	10	11	11	14
Fbxo46	208	132	96	155	115	187
Flnb	4121	2355	1817	3424	1936	4107
Furin	4983	3062	2206	4608	2657	4235
Gapdh	161	99	78	120	54	196
Gdpd5	769	426	299	602	398	668
Ginm1	4779	3681	3105	4103	3045	5630
Gja1	16989	13704	10373	14049	13029	15948
Gk5	119	59	49	71	52	113
Gm10012	6	2	0	4	0	3
Gm10635	147	124	94	174	82	135
Gm1720	2	0	0	1	1	1
Gm17757	12	11	2	3	11	17
Gm17762	40	29	27	27	26	48
Gm18853	12	11	2	3	11	17
Gm19277	128	80	55	133	91	68
Gmcl1	394	225	246	347	234	427
Gnai1	274	174	153	226	154	263
Gnrh1	45	35	20	33	22	69
Hdac6	839	457	442	685	462	835
Hhip	2	0	0	2	0	1
Hist1h2af	3	3	3	7	2	3
Hpgd	333	282	210	292	169	466
Hsd3b7	804	406	326	634	395	689
Idnk	361	232	221	352	207	394
Inhbb	124	75	73	93	66	132
Islr2	47	31	19	29	27	43
Isoc2b	145	81	82	112	75	140
Itfg3	4523	2846	2359	4044	2293	4167
Kars	4732	3199	2579	3951	2651	4823
Katnbl1	342	244	186	313	174	435
Kmt2e	6035	4828	3152	5460	4122	6423
Limch1	12833	9504	7431	11731	9371	12560
Lpar5	1	4	0	4	2	1
Lta	1	0	1	1	1	1
Mall	282	167	136	227	148	302
Mbd2	4206	3442	2875	4089	2874	5508
Mblac2	604	398	307	491	380	616
Med30	465	322	247	360	292	545
Med9os	14	12	14	14	18	17

Mettl25	199	137	103	155	144	202
Mir3069	1	0	1	1	1	1
Mir7017	1	1	0	2	1	0
Mir99a	5	3	5	3	5	9
Mnd1	36	21	17	28	17	45
Mob1b	902	654	496	878	567	712
Mrps28	867	527	505	794	475	935
Myl2	346269	259715	237463	319240	187109	436636
Naa16	432	341	242	409	281	489
Ndufa7	3699	2093	1984	3199	1727	4198
Neb	252	346	367	354	269	486
Necab3	14	4	7	5	4	16
Nfx1	2307	1531	1289	1895	1317	2366
Npl	43	23	18	33	19	49
Nup107	521	354	303	514	300	556
Oas2	1075	636	447	777	366	1227
Parpbp	18	10	12	8	4	31
Pcdhb8	22	3	4	11	8	15
Pes1	1148	757	623	961	682	1066
Pet100	314	208	242	299	164	392
Phkg1	2371	1505	877	1776	974	2333
Pigyl	454	227	301	439	227	505
Pik3r1	9262	5747	4265	7846	6416	6710
Pla2g15	415	208	234	313	211	391
Plekha4	176	107	52	112	64	173
Pmepa1	1463	1100	884	1714	693	1381
Poc5	583	343	291	510	269	528
Pold3	694	461	330	546	425	713
Poldip2	6094	3933	3586	5500	2990	6595
Polr3e	545	327	321	438	302	551
Ppp2cb	2051	1220	1283	1922	1175	2191
Ppp2r1b	327	229	206	285	231	367
Ppp3r1	5306	3418	3125	4651	3082	5090
Prtn3	5	0	1	3	1	4
Rab26	1	1	0	0	2	1
Rab36	37	24	16	30	12	55
Radil	60	39	25	56	22	51
Rassf10	117	64	31	80	71	83
Rnf103	1922	1259	1099	1752	997	1948
Rnf146	2179	1481	1362	1921	1331	2224
Rnf217	481	270	240	359	258	432
Rps20	1326	607	737	1106	518	1601
Rps6	246	179	155	202	156	269
Rrm1	1026	664	560	868	566	1003
S100a1	14466	11180	10235	11797	10352	17034

S100a4	95	43	46	47	40	143
Scaf8	2140	1448	1195	1977	1298	1807
Scrg1	1	0	1	2	1	0
Sec22b	1142	702	659	1041	573	1124
Sfmbt1	902	517	471	653	500	866
Sirpb1b	1	1	0	2	1	0
Ska3	18	4	5	8	3	24
Slc35b4	553	352	244	457	249	526
Slitrk3	5	3	0	2	3	2
Sln	369	258	258	280	353	220
Smim8	1440	935	1026	1146	865	1780
Snord32a	6	3	2	3	5	6
Snord47	20	10	11	15	12	23
Snrpe	518	316	317	451	286	621
Sptbn1	73581	51363	41114	61991	47693	82140
Stab1	2640	1660	1204	2027	1294	3066
Stat5b	3666	2272	1774	3150	1962	3059
Sycp2	42	34	23	33	36	48
Syt17	28	20	13	37	9	30
Tbc1d9	348	208	158	282	148	337
Thoc6	277	157	141	211	136	268
Tmem141	652	429	355	523	408	718
Tmem194	351	222	174	240	192	367
Tmem8b	1465	880	751	1235	782	1291
Tnrc6c	3971	2739	1952	3266	2611	3818
Top2b	5437	3947	3182	4758	3267	5666
Tox3	219	133	117	198	159	169
Tpd52	998	638	513	751	560	1005
Tpm4	5006	3032	2873	4377	2377	6308
Trappc1	1104	732	570	953	604	1219
Tssc4	316	188	148	280	154	320
Ttc4	1079	733	580	893	621	1074
Txnrd2	1275	628	639	1002	642	1255
Ube2n	1211	808	679	1085	598	1293
Unc13a	79	73	39	70	47	85
Upf2	2351	1677	1447	2053	1586	2664
Usp11	611	357	225	466	280	493
Vkorc1l1	1573	962	887	1368	840	1509
Zfp112	288	162	133	196	152	265
Zfp330	616	401	324	451	341	658
Zfp655	1742	1084	1103	1421	1052	1815
Zfp938	413	247	207	305	216	406
Znf41-ps	181	151	123	171	132	238
Tmem151a	36	15	19	23	19	29
2810013P06Rik	761	395	460	490	473	747

Smim6	7	5	3	5	6	3
Ehbp1	4568	3405	2633	3769	3127	4393
Fam19a3	147	93	70	111	73	145
Fem1a	11622	7077	5701	9965	6182	9633
Ikbke	91	52	35	54	46	83
Lrp6	4237	2826	2314	3590	2552	3882
Ptk6	3	6	4	4	6	7
Snora81	21	18	15	19	11	39
Serf1	107	86	74	90	83	108
1600010M07Rik	3	1	2	3	2	3
Abhd13	1481	1011	803	1381	789	1411
Adcy8	37	21	23	37	19	42
Ap1m1	1780	1174	849	1415	965	1705
Cetn3	1704	1367	1040	1441	1243	2069
Csnk1g1	985	702	560	801	592	1056
Errfi1	945	665	812	893	673	1123
Fos	93	52	63	80	53	118
Gm1631	3	3	1	1	4	1
Helb	836	526	422	684	483	866
Hist1h2bh	6	2	1	6	0	2
Klf15	2289	1498	1354	1831	1416	2316
Mir1a-1	3	0	3	3	3	2
Mir8091	11	3	4	6	7	9
Six5	533	348	214	430	273	440
Smox	707	452	340	569	424	703
Tomm20	1151	761	738	1034	676	1405
Vrk1	588	377	297	490	337	619
Fntb	522	315	242	444	278	507
Bcan	4	1	0	2	0	5
Timp2	5475	3879	3094	5486	2611	5660
Coq9	15534	9643	8521	12449	8729	15101
1110038B12Rik	71	44	52	53	39	89
1700034F02Rik	0	1	1	1	1	1
1810020O05Rik	1	0	1	0	1	2
2310045N01Rik	388	267	210	350	241	402
5830415F09Rik	114	62	47	71	62	95
6430548M08Rik	1025	644	509	1033	498	773
6430706D22Rik	1835	1270	880	1430	987	2210
A630072M18Rik	666	470	328	522	440	696
A930024E05Rik	27	19	16	16	19	41
Acot8	200	118	115	178	114	213
Acox3	1753	1159	927	1423	1022	1951
Adk	6696	4373	3756	5242	4132	6484
Agrp	0	1	1	1	1	1
Akr7a5	1093	644	514	899	583	1062

Alcam	186	168	87	191	109	163
Aldh1a7	3	10	5	8	6	10
Ap3s1	274	180	190	253	191	307
Arf4	3125	2234	2120	3041	1849	3432
Arhgap36	8	6	5	9	3	7
Arhgef17	4413	2977	2053	4186	2588	3796
Arl4c	511	347	302	496	254	522
Atg16l1	1619	1077	917	1365	922	1659
BC026585	228	157	168	215	156	285
Becn1	2867	1833	1647	2385	1674	3259
Bik	164	85	95	113	95	198
C1qa	1291	742	709	1093	511	1454
C2cd3	817	560	333	777	423	741
Capn3	103	68	66	108	61	112
Ccdc127	2462	1707	1415	2168	1416	2523
Ccny	5442	3660	3257	5242	2947	6366
Ccr1l1	0	0	3	0	0	3
Ccr4	1	1	0	2	0	1
Cd82	320	207	187	275	195	359
Cdh20	53	56	28	73	33	60
Ceacam2	1	2	1	0	1	5
Cenpv	401	272	211	312	225	416
Cep57	808	499	445	626	456	953
Chchd2	7997	4012	4493	6617	3332	9911
Chtf8	1636	900	869	1443	841	1343
Clcn2	198	111	85	118	109	231
Cmtm4	1702	888	886	1370	836	1528
Cops5	2844	1697	1791	2538	1615	3223
Cox16	527	342	342	465	311	655
Cped1	1938	1443	1196	1746	1166	2137
Crb3	12	4	1	5	1	10
Crebbp	1563	1142	650	1269	1009	1454
Cspp1	458	298	236	365	258	439
Ctage5	4465	3008	2203	3287	2948	3788
Ctbp2	869	582	517	817	546	892
Ctss	606	466	367	540	251	850
Cwc25	603	377	349	526	351	659
Dcun1d5	2198	1358	1306	1803	1187	2366
Dhrs13	153	86	84	127	73	185
Dmp1	1	0	1	0	1	2
Dnajc8	2975	2326	1703	2410	1887	3370
Dsg1c	2	0	0	1	0	2
Duoxa1	9	0	1	7	0	6
Ebp	531	341	253	480	250	584
Ecsit	3863	2295	2008	3090	2004	3745

Eef1b2	4463	2724	2705	3883	2544	5085
Ell3	7	2	1	1	3	9
Esrra	4263	2150	2089	3315	2100	3591
Etfb	21134	12712	11644	17015	11951	23619
Exd1	26	11	16	9	15	30
Fa2h	2	0	0	1	0	2
Fam133b	704	476	397	597	403	839
Fau	116	120	59	114	106	120
Fbxl19	201	94	94	159	96	146
Fgf18	194	163	170	203	130	255
Fpr1	2	0	0	1	0	2
Fstl4	96	57	75	70	111	75
Gda	1306	825	1072	1149	804	1996
Gfod2	148	66	54	125	59	124
Gjb2	1	1	0	1	0	2
Gldn	1	0	1	0	1	2
Gm10364	6	6	4	7	6	2
Gm15055	41	20	27	33	24	49
Gm9776	66	28	30	43	29	76
Gmpr	5063	2922	2396	4125	2413	5426
Guk1	1474	841	913	1195	872	1674
Gzmm	229	79	92	158	111	178
Haus8	538	350	346	501	379	531
Hsbp1l1	146	96	87	108	91	151
Hspbap1	148	89	85	121	68	160
Ifltd1	86	98	71	127	104	66
Il10ra	204	88	84	142	82	215
Ing3	672	477	415	590	432	798
Irx6	1	0	1	2	0	1
Itsn2	2437	1615	1243	2085	1516	2351
Kcnd3	889	630	567	747	717	891
Kcnn4	14	8	7	15	5	9
Kcns3	15	5	7	8	5	14
Kif5b	11997	8270	8106	10118	8241	12280
Klhl41	4855	3426	2823	5363	2691	4874
L3mbtl2	618	403	265	521	341	593
Lama2	6738	4982	3572	6768	3867	5616
Leprotl1	1443	985	843	1397	778	1402
Lor	2	0	0	1	0	2
Mapk10	820	575	583	761	635	878
Mapk7	305	199	144	281	156	312
Melk	19	8	11	6	4	44
Mfsd4	177	79	86	143	66	200
Mir21	1	1	0	0	1	2
Mir5122	0	1	1	1	1	1

Mir7025	13	2	5	11	1	7
Mllt3	783	549	429	652	500	867
Mpp4	130	53	58	74	68	137
Mrps18b	461	378	318	499	277	501
Ms4a6c	317	146	149	248	114	294
Mtdh	4281	2994	2684	3946	2635	5028
Mvb12b	1232	782	613	1109	672	997
Ndufb11	8316	4498	4833	7461	3985	9347
Nfat5	5841	4054	2739	4549	3640	6064
Nlrp12	1	1	0	1	0	2
Nme3	448	247	267	349	199	515
Npcd	367	226	146	288	196	354
Opn1sw	2	0	0	1	0	2
Parp2	783	528	415	578	462	823
Pdf	2478	1526	1263	1989	1284	2437
Pi4kb	1487	891	836	1219	754	1556
Pla2g12a	1586	905	1016	1231	1040	1759
Ppt1	2770	1954	1740	2351	1886	3200
Preb	2125	1338	1029	1667	1094	2091
Pttg1ip	5252	3330	2809	4749	2827	5551
Qars	2867	1856	1511	2406	1564	2790
Reep4	162	102	77	128	74	163
Rfc2	676	400	301	556	334	685
Rhbdl1	137	84	77	95	61	169
Riiad1	6	15	15	16	13	19
Rnf166	1539	911	924	1194	989	1407
S100a11	591	571	307	571	356	837
Sac3d1	143	95	85	154	74	156
Sall3	3	2	1	4	1	3
Samd10	117	59	62	92	62	125
Sart1	2566	1791	1401	2114	1593	2891
Sccpdh	2993	2007	1670	2778	1770	3064
Sdccag3	2108	1363	1177	1781	1119	2201
Sf3a2	988	644	461	871	519	998
Sfrp4	15	13	3	16	7	15
Sfxn4	401	273	198	316	234	458
Sgtb	71	64	48	76	47	72
Sirt3	1472	743	763	1214	780	1373
Slc22a8	0	2	2	1	1	4
Slc25a10	485	360	264	503	253	551
Slc25a11	20412	11646	11033	17888	11111	20378
Slc29a3	327	218	166	297	180	345
Slc6a2	1	0	3	1	1	4
Smad6	647	254	191	457	246	509
Snx12	895	562	535	962	458	909

Sowaha	16	11	4	10	6	23
Sppl2b	494	291	234	453	250	461
Syt9	4	1	2	5	1	0
Tagln3	40	22	13	26	18	32
Tango6	457	337	227	388	290	393
Tex15	4	3	3	3	3	7
Thrb	1664	1073	809	1284	1065	1608
Tnfaip8l3	17	10	6	13	13	13
Tor1aip1	7471	5127	3628	6193	4386	6575
Trpv4	336	172	114	266	123	331
Tssc1	282	190	153	273	129	277
Ttc33	1394	936	824	1185	816	1448
Tyrp1	1	4	5	2	3	5
Ubxn6	2019	1376	1067	1546	1186	2099
Upf1	3080	1832	1487	2727	1687	2762
Upf3b	1192	915	681	842	862	1529
Vip	4	2	1	2	0	8
Vps13b	1731	1257	870	1428	1058	1636
Ywhaq	898	573	491	887	443	960
Zfp280b	538	348	297	522	289	452
Zfp408	258	168	119	231	121	287
Zfp451	884	644	488	766	561	829
Zfyve21	1464	847	610	1184	642	1286
Zmym3	1181	816	560	908	730	1261
Zmynd11	8104	5887	4889	7064	5294	8009
1110004E09Rik	820	506	430	622	440	841
1110037F02Rik	1811	1237	915	1548	981	1768
2210016L21Rik	675	422	393	599	372	763
2610002M06Rik	916	627	443	815	562	856
4930581F22Rik	114	69	65	74	77	140
A530072M11Rik	12	6	10	9	5	15
Acat1	28194	19650	18012	22948	18405	30104
Adam22	1311	738	493	810	782	948
Apc	4829	3501	2874	4219	3046	4896
Apeh	1459	921	653	1262	746	1436
Asap1	2132	1453	1060	1960	1200	2124
Ash2l	1708	1227	965	1500	1129	1802
Atp11b	2139	1515	1337	2036	1316	2087
Brwd3	239	145	120	206	126	252
C130030K03Rik	8	4	0	5	3	7
Calcr	0	2	0	1	1	1
Cat	12042	8025	7074	9471	8908	12109
Ccdc61	252	159	151	199	165	287
Ccndbp1	1270	881	690	1057	744	1481
Cdc7	75	45	40	74	29	92

Ces1e	29	25	27	30	26	43
Chpf	657	381	295	576	273	581
Chst3	108	69	55	91	66	112
Cntnap4	0	2	0	1	1	1
Cog4	3256	2095	1855	2751	1791	3366
Crebzf	1750	1011	853	1272	882	1757
Cxx1c	261	171	134	252	122	294
Dcaf10	1064	617	556	837	601	1107
Ddx6	8529	5976	5296	7748	5454	8197
Dennd5b	2510	1638	1443	2133	1534	2733
Dleu7	1	0	1	1	0	2
Dnajc9	593	433	340	541	355	693
Drc1	1	2	1	1	0	5
Dtd1	440	351	281	447	236	617
E130310I04Rik	77	38	26	53	43	40
E230016M11Rik	29	21	21	12	20	60
Fam64a	25	10	5	9	3	41
Fbxo42	1046	672	507	919	552	906
Fbxw11	2575	1586	1448	2333	1420	2291
Foxo6	87	65	47	73	55	81
Gabrg3	3	0	0	1	2	1
Glod4	1907	1335	1227	1781	1193	2228
Glt8d2	163	96	95	142	64	183
Gm14023	36	26	36	37	22	67
Gm15663	71	43	27	59	43	59
Gm16973	173	130	104	141	96	208
Gm21284	4	6	1	1	1	9
Gm3646	121	78	114	137	58	159
Hfe2	9972	5746	5712	8032	5988	10430
Hist1h2bb	0	5	3	3	2	7
Hmg20b	1519	1056	775	1219	802	1652
Hopx	12584	7886	6429	10486	5402	13925
Ift52	1078	780	682	960	715	1269
Insl6	9	3	6	8	3	12
Irf2	1292	1082	757	1250	845	1546
Kcmf1	5145	3438	3228	4519	3240	5040
Kif19a	14	10	8	12	10	17
Lama4	5114	3441	2736	4768	2693	5648
Lamc1	12073	8304	6534	11426	6103	11895
Lcp2	123	72	40	103	45	94
Letm1	5425	3628	3217	4685	2983	5871
Lmtk3	4	4	1	2	2	8
Lrrc14b	5061	3189	3104	4326	3158	4718
Mad1l1	388	239	177	293	234	381
Map2k7	3331	2232	1649	2794	2001	3321

Mcm6	529	321	264	429	268	596
Meis1	1098	771	692	853	817	1033
Mir6927	1	0	1	1	0	2
Mir7658	31	7	10	16	10	21
Mmp2	2655	1652	1314	2649	955	2582
Msc	120	61	30	86	26	106
Nedd1	2068	1366	1145	1867	1209	1836
Nek8	129	87	58	75	85	160
Nip7	493	359	309	443	295	535
Nlrp3	8	8	6	11	9	7
Nrl	1	0	1	1	0	2
Nudt22	492	311	245	457	207	584
Ogfrl1	1948	1608	1487	1716	1334	2634
Oscar	20	12	8	9	15	15
Pcnp	1599	1157	970	1346	1001	1740
Pdap1	2176	1674	1533	1941	1465	2994
Pdcd2l	466	242	211	325	231	500
Pdcd4	1071	697	575	734	711	1225
Pdzrn4	187	111	79	184	87	163
Pex11a	272	167	167	191	178	276
Pex7	1697	898	845	1455	866	1565
Plau	202	121	136	167	80	341
Pld5	16	12	11	21	9	18
Plekhg4	13	5	6	10	4	10
Plxnb3	211	132	76	157	109	163
Polr3c	1046	666	595	841	618	1040
Ppm1a	6687	4394	4120	5669	4277	6401
Ppp1r3b	2046	1236	921	2429	1020	1289
Pstk	981	772	641	839	673	1303
Rabgap1	2631	1894	1475	2420	1703	2690
Rasl10b	3546	1904	2135	2970	1938	3307
Rdx	9593	6275	5426	8514	5445	9092
Rest	391	296	250	361	304	407
Rpa3	254	132	150	195	138	297
Rplp2	1236	610	799	1174	566	1155
Rps8	2282	1354	1268	1799	1126	2873
Sap130	1110	886	608	1015	778	1168
Sars2	389	293	208	369	251	401
Sfpq	5799	3920	3107	5030	3217	5662
Shf	89	39	25	62	40	71
Siae	892	595	465	784	537	742
Skida1	295	199	142	247	169	255
Slc35f6	2466	1689	1516	2474	1218	2629
Slitrk5	6	7	4	8	3	6
Smek1	2270	1767	1365	2203	1504	2513

Snora70	21	7	5	11	10	18
Snord53	3	8	4	7	9	3
Snord64	5	1	1	2	3	4
Snw1	3196	2282	1873	2690	1842	3598
Ssx2ip	758	540	491	690	489	767
St3gal2	1385	860	763	1340	731	1391
Tfpt	406	277	243	308	280	483
Tmem116	133	83	61	89	85	109
Tmem131	4096	2924	2200	3799	2649	3966
Tnnt3	21	19	17	16	13	32
Trappc4	782	430	459	655	438	835
Trps1	582	332	351	483	286	621
Tspyl3	66	31	42	52	30	66
Tube1	62	29	36	39	25	72
Tubg1	882	566	481	751	500	829
Tusc1	69	50	41	63	32	78
Ube2r2	7305	5105	4579	6531	4485	8852
Ugt1a7c	85	49	37	66	34	106
Uqcrb	7737	5748	6642	7575	5138	10106
Vps18	756	401	334	643	389	637
Zbtb39	686	410	340	567	378	572
Zfp110	1622	1148	993	1429	904	1854
Zfp532	1213	767	698	1046	792	1187
Zfp61	136	97	80	125	79	167
Zfp639	1416	953	833	1168	825	1519
Zfp747	92	80	57	122	49	107
Zfp942	678	539	489	644	430	986
Zzef1	2361	1494	1026	1873	1391	2151
Fkbp14	606	347	327	501	306	683
Gm12250	530	81	52	296	154	221
Lacc1	209	111	124	159	111	256
Mastl	46	24	24	26	23	66
Rab1	8474	5896	5137	7764	4798	8941
Rnf32	7	9	2	3	4	11
Scarf2	299	191	102	238	146	290
Tgfa	47	35	27	46	37	44
Agbl5	346	202	190	237	177	389
Fbxw5	2868	1636	1403	2332	1553	2765
Hace1	469	326	246	396	238	505
Rc3h2	2934	1656	1635	2257	1526	3019
Sobp	1279	1009	817	1230	791	1414
0610005C13Rik	6	4	4	7	1	9
0610007P14Rik	207	157	122	157	138	265
0610009O20Rik	6638	4146	3451	5620	3624	6330
0610031O16Rik	2	2	1	1	1	3

0610038B21Rik	18	10	7	12	11	13
1110001J03Rik	935	495	516	845	427	979
1110032A03Rik	519	436	360	437	357	664
1110034G24Rik	1275	670	554	968	677	1126
1110046J04Rik	33	18	21	26	18	41
1110059E24Rik	549	348	307	453	303	635
1500004A13Rik	16	5	5	7	8	10
1600002H07Rik	111	72	85	104	68	125
1600012H06Rik	887	603	539	760	554	1002
1600014C10Rik	2113	1375	1133	1802	1283	1903
1600020E01Rik	172	101	90	134	87	198
1700001C19Rik	1	0	0	0	0	1
1700001P01Rik	5	2	0	1	1	5
1700003E16Rik	10	9	1	8	5	11
1700003M07Rik	5	7	1	10	0	8
1700008J07Rik	87	56	54	63	54	93
1700008O03Rik	5	0	3	2	0	6
1700009P17Rik	41	41	24	36	23	56
1700010I14Rik	3	3	3	3	4	2
1700011E24Rik	0	0	2	2	1	0
1700018L02Rik	11	14	10	10	15	16
1700019A02Rik	0	1	0	1	0	0
1700020D05Rik	38	23	16	21	22	36
1700024F13Rik	1	0	0	0	0	1
1700024P16Rik	0	1	0	1	0	0
1700028K03Rik	1	0	3	2	0	4
1700030F18Rik	1	3	2	3	3	2
1700030K09Rik	287	177	152	190	184	317
1700034H15Rik	34	12	18	6	24	42
1700048O20Rik	12	9	5	7	8	16
1700056E22Rik	22	11	10	13	13	25
1700061G19Rik	0	1	1	2	0	1
1700065D16Rik	1	0	0	0	0	1
1700066M21Rik	238	140	152	210	131	275
1700074H08Rik	1	0	0	0	0	1
1700084J12Rik	0	0	1	0	0	1
1700094D03Rik	383	248	155	248	234	397
1700095B10Rik	3	3	3	1	4	4
1700096K18Rik	133	100	78	96	111	146
1700109H08Rik	91	59	26	49	47	101
1700110K17Rik	0	0	1	0	1	0
1700113A16Rik	14	12	8	7	13	20
1700124L16Rik	3	0	1	3	1	1
1810009A15Rik	416	307	240	339	246	526
1810022K09Rik	246	195	143	194	161	315

1810026J23Rik	3499	2179	1962	3181	1950	3195
1810030O07Rik	1153	761	639	992	676	1133
1810041L15Rik	18	11	18	13	17	25
1810043H04Rik	413	236	188	322	178	403
1810044D09Rik	56	22	36	42	30	63
2010016I18Rik	3	2	3	5	0	5
2010106C02Rik	1	0	0	0	0	1
2210039B01Rik	6	3	5	3	2	10
2210407C18Rik	98	69	54	97	57	103
2210408I21Rik	185	136	97	148	109	207
2300005B03Rik	0	1	0	1	0	0
2310022A10Rik	1834	1030	898	1544	995	1610
2310040G24Rik	356	269	251	271	235	472
2310043O21Rik	0	0	1	1	0	0
2310061J03Rik	125	77	60	103	54	127
2310068J16Rik	27	16	12	18	14	25
2410004I01Rik	2	1	0	2	1	1
2410016O06Rik	177	107	92	138	95	173
2410017I17Rik	0	0	2	1	0	1
2410089E03Rik	783	470	315	559	448	711
2510002D24Rik	606	423	309	479	348	682
2610001J05Rik	1182	627	668	999	596	1271
2610008E11Rik	1724	1252	1090	1388	1075	2043
2610020C07Rik	17	11	7	9	12	19
2610035D17Rik	464	113	115	291	126	324
2610037D02Rik	1	2	0	0	1	2
2610044O15Rik8	602	401	329	454	322	698
2610507I01Rik	1015	710	643	797	713	1050
2700046G09Rik	87	62	63	54	65	119
2700070H01Rik	6	7	5	6	5	8
2700094K13Rik	342	194	136	251	141	383
2700097O09Rik	298	179	184	219	189	310
2810021J22Rik	255	144	125	162	180	203
2810032G03Rik	2	1	3	2	2	3
2810055G20Rik	221	223	221	198	206	345
2810403D21Rik	42	29	26	27	26	50
2810408I11Rik	0	1	3	0	1	3
2810442I21Rik	14	17	13	12	18	21
2810459M11Rik	0	1	0	1	0	0
2900008C10Rik	117	81	52	89	61	120
2900052N01Rik	46	18	37	21	47	41
2900076A07Rik	74	52	36	55	34	90
3110001I22Rik	100	50	57	87	41	94
3110002H16Rik	1804	1099	856	1191	1024	1805
3110009E18Rik	25	19	30	25	21	39

3110021A11Rik	15	10	7	10	9	16
3110035E14Rik	1	0	1	1	0	1
3110040N11Rik	257	186	165	207	157	313
3110043O21Rik	1463	917	868	1355	800	1389
3110045C21Rik	17	17	11	10	11	30
3110056K07Rik	335	202	215	270	176	385
3300005D01Rik	1	0	1	1	0	1
3632451O06Rik	1128	677	553	939	578	1063
3830408C21Rik	60	21	28	37	27	50
4632434I11Rik	29	21	18	19	19	43
4732471J01Rik	18	10	8	16	11	16
4831440E17Rik	251	189	130	169	177	315
4833422C13Rik	113	64	51	79	52	135
4833439L19Rik	5398	3580	3191	4790	3180	5438
4921504A21Rik	138	145	82	148	117	152
4921507L20Rik	0	1	0	0	0	1
4930404H11Rik	6	1	5	3	4	7
4930404N11Rik	4	1	0	1	1	3
4930429F24Rik	1	0	3	3	1	1
4930431P03Rik	1	0	0	1	0	0
4930451G09Rik	41	36	13	33	22	49
4930455H04Rik	5	2	8	4	4	8
4930479D17Rik	15	7	13	8	13	19
4930481A15Rik	1355	751	562	790	832	1093
4930511M06Rik	3	4	3	3	4	5
4930513N10Rik	12	5	7	6	12	4
4930520O04Rik	40	22	25	34	22	37
4930524B15Rik	1	0	0	0	0	1
4930529M08Rik	39	29	14	24	25	42
4930539J05Rik	19	9	10	4	9	27
4930545L23Rik	1	0	0	0	0	1
4930548H24Rik	1	0	0	0	0	1
4930556G01Rik	0	1	0	0	0	1
4930558J18Rik	0	0	1	0	0	1
4930563E22Rik	49	20	13	9	30	38
4930578M01Rik	11	6	4	4	6	14
4930579F01Rik	0	1	0	1	0	0
4930594C11Rik	5	4	7	0	8	12
4931414P19Rik	242	151	138	181	153	239
4931431C16Rik	1	3	2	1	2	3
4931440P22Rik	19	13	6	9	14	21
4932435O22Rik	20	13	12	13	12	27
4932438H23Rik	3	2	0	3	2	0
4932702P03Rik	0	1	0	0	0	1
4933405D12Rik	1	0	0	0	0	1

4933406I18Rik	4	0	0	0	2	1
4933406M09Rik	2	0	0	1	0	1
4933407L21Rik	1	0	0	0	0	1
4933411E08Rik	2	2	1	1	2	3
4933411K20Rik	1019	718	578	832	644	1038
4933413L06Rik	1	0	1	0	0	2
4933417D19Rik	21	12	7	16	6	20
4933421I07Rik	0	0	1	0	0	1
4933424G06Rik	4	0	0	1	0	3
4933426M11Rik	6636	4524	3577	6133	3831	6062
4933427D06Rik	0	1	0	1	0	0
4933427E11Rik	1	2	0	1	0	2
4933432I03Rik	1	1	1	1	0	2
4933439K11Rik	0	2	0	1	0	1
5031425F14Rik	3	1	5	1	5	6
5033406O09Rik	23	4	7	13	7	20
5330413P13Rik	8	9	8	7	9	10
5330426P16Rik	228	169	136	242	111	232
5430403N17Rik	0	1	1	0	1	2
5430425K12Rik	3	1	0	0	1	4
5430428K19Rik	1	0	1	1	1	0
5730420D15Rik	1	3	2	2	1	3
5730559C18Rik	1	0	0	0	0	1
5830416I19Rik	7	12	1	6	8	10
5830416P10Rik	3	1	3	4	2	1
5830418K08Rik	664	483	435	525	434	780
5930403L14Rik	658	439	389	644	384	600
6030407O03Rik	0	1	2	1	1	2
6330410L21Rik	41	45	35	41	37	67
6330419J24Rik	9	5	4	9	6	7
6530402F18Rik	337	216	215	291	210	386
6820431F20Rik	47	41	26	36	38	55
8430429K09Rik	49	51	52	59	44	81
8430437L04Rik	8	0	5	6	2	7
9030617O03Rik	2811	1769	1298	2125	1766	2187
9030619P08Rik	1	1	2	0	0	6
9030625G05Rik	0	2	0	0	0	2
9130008F23Rik	33	29	14	24	19	46
9130019O22Rik	119	82	42	82	51	130
9130019P16Rik	0	3	4	3	3	1
9130023H24Rik	194	127	110	163	135	156
9130230L23Rik	1	0	0	0	0	1
9130401M01Rik	477	258	248	355	250	451
9230110C19Rik	36	22	19	28	16	41
9230116L04Rik	6	2	4	2	3	8

9330020H09Rik	25	43	38	35	32	58
9330133O14Rik	120	74	69	88	74	139
9330151L19Rik	267	197	157	230	163	292
9330159F19Rik	20	18	104	23	51	106
9430060I03Rik	9	4	5	8	3	8
9430076C15Rik	13	12	9	13	14	12
9530026P05Rik	49	38	29	51	32	53
9630028H03Rik	2	1	1	1	1	2
9830147E19Rik	291	194	160	228	156	334
9930111H07Rik	0	0	1	0	1	0
A130010J15Rik	290	212	168	257	189	319
A230028O05Rik	2	1	1	1	0	3
A2m	12	9	7	7	6	16
A330049N07Rik	2	0	1	1	0	2
A330076C08Rik	1	0	1	0	0	2
A430088P11Rik	0	0	1	1	0	0
A4galt	186	113	139	154	92	292
A530050N04Rik	1	2	0	1	1	1
A530088E08Rik	0	1	1	1	0	2
A530099J19Rik	12	5	5	13	4	8
A630023P12Rik	1	0	0	1	0	0
A730017C20Rik	3	0	0	1	0	3
A730017L22Rik	149	102	67	91	110	158
A730036I17Rik	0	5	2	5	2	2
A730090N16Rik	1	2	1	1	1	2
A930003A15Rik	2	1	0	0	1	2
A930007I19Rik	15	14	6	6	15	19
A930012L18Rik	14	14	0	6	8	13
A930013F10Rik	175	111	124	135	107	235
A930041C12Rik	1	0	0	0	0	1
AA415398	157	102	99	117	113	171
AA467197	11	3	6	5	2	19
AA474331	36	19	25	30	14	44
AA987161	508	365	282	438	331	530
Aarsd1	1474	908	689	1190	705	1584
Aasdhppt	1805	1095	1194	1633	1027	1955
Aatk	249	155	100	188	125	254
Abca14	0	2	0	0	0	2
Abca3	1337	976	731	1109	806	1482
Abca6	886	518	564	701	469	1072
Abcc10	302	136	126	192	156	274
Abcc3	55	25	43	62	24	66
Abcd1	1663	1058	771	1405	881	1481
Abcd4	401	266	187	324	212	421
Abhd14a	468	245	265	358	246	449

Abhd16a	1574	1025	848	1273	895	1757
Abhd2	637	401	305	595	332	496
Abl1	3210	2098	1418	2783	1797	2870
Acad10	281	195	153	198	167	330
Acadl	59342	41142	38500	48393	39397	66374
Acads	6559	3679	3246	5041	3652	6318
Acap3	723	349	247	468	325	565
Acat2	122	85	69	117	71	125
Acbd5	2143	1427	1253	1915	1344	2183
Acd	326	217	132	230	170	318
Acer3	482	271	258	393	243	507
Aco1	1765	1193	951	1747	961	1709
Acot4	22	11	16	11	14	35
Acot7	2386	1437	1150	1815	1290	2436
Acox1	20287	13634	11245	16306	12422	19805
Acox2	16	5	9	9	12	10
Acoxl	1	2	0	1	3	0
Acp2	2080	1406	1116	1802	1183	2086
Acpp	1	1	3	2	2	2
Acsf3	1072	680	555	905	568	1162
Acsl4	1277	844	713	1105	666	1410
Actg1	564	258	271	375	252	632
Actl6a	984	641	503	830	553	1021
Actl7b	1	0	1	0	1	1
Actn2	95344	70337	55236	93338	60484	101130
Actr1b	2827	1713	1486	2364	1570	2552
Actr2	4539	2961	2736	4016	2719	5029
Actr3	3845	2523	2186	3501	2035	4241
Actr5	196	109	74	149	89	172
Actr6	611	383	375	531	365	590
Actr8	1655	1093	1045	1429	1079	1792
Acvr1	967	722	494	901	556	959
Acvr1b	713	519	402	660	481	670
Acvr2a	2171	1460	1177	1921	1329	1896
Acvr2b	64	36	42	42	36	76
Acy3	888	599	420	677	521	939
Acyp1	313	218	245	291	195	398
Acyp2	2518	1527	1542	2032	1464	2694
Adam1b	0	1	0	0	1	0
Adam21	2	3	3	2	1	7
Adam30	0	1	0	0	0	1
Adam32	13	16	9	11	11	19
Adam5	13	10	2	10	5	15
Adam6b	0	0	1	0	0	1
Adam9	3724	2382	2013	3363	2177	3576

Adamts4	10	5	3	5	3	15
Adamts8	48	24	31	45	19	61
Adat3	28	18	9	15	18	24
Adck4	300	188	141	261	150	261
Adck5	169	68	57	106	73	154
Adcy9	2304	1586	1336	2114	1511	2059
Adcyap1r1	441	279	169	396	198	363
Add1	7184	4953	3968	6515	4249	7426
Add2	9	4	3	4	4	11
Add3	3653	2528	1805	3496	2030	3543
Adhfe1	4141	2757	2285	3434	2399	4207
Adipor1	8841	6004	5025	7864	5070	9239
Adnp	3828	2691	2193	3235	2421	3951
Adnp2	676	382	344	548	343	685
Adora2a	387	185	159	261	178	391
Adra1b	873	531	409	734	503	671
Adra1d	158	53	86	141	71	117
Adrb3	1	3	1	2	0	3
Adss	845	514	445	665	431	970
Aebp1	771	492	389	628	410	819
Aebp2	2516	1585	1439	2025	1598	2376
Aff1	4550	3296	2786	4238	2911	4453
Aftph	1646	1119	880	1432	982	1658
Agap3	2258	1384	982	1884	1206	2020
Agbl1	3	2	3	3	4	3
Agmat	19	10	10	8	13	19
Agpat3	5545	3233	3125	4258	3391	5257
Ahdc1	1944	1032	727	1849	853	1387
Ahi1	159	105	86	135	81	172
Ahnak	44232	28740	21273	36247	27053	40292
AI413582	120	94	58	113	39	162
AI504432	52	35	16	45	16	49
AI607873	1120	737	617	925	609	1274
AI661453	22	22	14	28	14	29
AI839979	77	24	41	48	35	78
Aicda	0	1	0	0	1	0
Aif1l	95	46	52	67	60	72
Ajap1	6	5	9	8	4	13
Ak5	1	1	2	2	1	2
Akap2	11574	8210	8017	10444	7717	13025
Alas1	9696	6688	6204	11374	5568	9115
Aldh9a1	2630	1406	1316	1500	1277	3067
Aldoart1	18	6	5	12	8	9
Alg1	500	305	247	431	223	519
Alg12	336	230	189	285	185	374

Alg2	558	370	286	498	321	529
Alg5	468	327	295	438	254	608
Alkbh1	212	140	127	225	121	208
Alkbh4	112	79	70	97	58	132
Alox12b	1	0	0	0	0	1
Alox5ap	221	153	122	182	106	275
Alyref	680	497	393	622	388	750
Amdhd1	0	1	0	1	0	0
Amer1	685	442	397	570	412	674
Amfr	7875	4595	4370	6676	4533	7077
Amn	1	0	0	1	0	0
Ampd1	0	1	0	0	0	1
Amy1	669	396	338	363	358	797
Amz1	17	12	10	16	9	18
Anapc10	282	213	190	263	200	324
Anapc5	9851	6212	5028	7857	5509	9473
Angpt4	2	4	4	3	6	4
Ankdd1b	35	30	20	25	23	46
Ankrd7	0	1	1	1	1	0
Anks6	107	60	46	75	58	95
Ankzf1	1066	590	464	722	573	1087
Ano7	1	1	0	1	1	0
Anxa10	0	0	1	1	0	0
Anxa2	3004	1888	1474	2817	1265	3359
Anxa8	20	14	13	19	11	22
Aoc2	123	65	71	87	82	107
Aox2	0	0	1	0	0	1
Aox3	26	28	18	23	19	43
Ap1s1	777	418	412	637	400	792
Ap1s3	8	2	4	8	4	5
Ap2a1	2858	1708	1293	2591	1442	2561
Ap2m1	1158	757	657	1071	631	1279
Ap3s2	1696	1003	951	1409	939	1654
Ap4e1	995	612	585	714	655	1140
Ap5z1	343	186	182	228	203	373
Apbb1	3132	1739	1437	2316	1679	2711
Aph1c	104	49	67	58	64	133
Apip	1766	1209	1115	1407	1140	1926
Aplp2	19274	14124	11373	17519	12162	21214
Apob	1	0	0	1	0	0
Apobec4	0	0	1	0	0	1
Apoc2	2	4	1	4	1	2
Apoe	7388	6662	4451	7757	4549	8690
Apof	0	0	1	0	1	0
Apon	7	6	2	9	4	4

Aqp2	1	2	0	0	2	1
Aqp3	1	0	1	1	0	1
Areg	0	0	1	0	0	1
Arfgap2	2232	1502	1077	1952	1149	2103
Arfgap3	560	332	296	463	269	595
Arfip1	1480	1053	796	1390	808	1510
Arfip2	807	542	423	704	439	860
Arg2	2	1	0	4	0	0
Arhgap1	1084	748	581	994	570	1195
Arhgap11a	162	98	81	104	73	203
Arhgap15os	0	1	0	0	0	1
Arhgap20	1912	1278	1244	1707	1600	1318
Arhgap22	49	29	24	38	19	55
Arhgap30	241	117	111	180	74	274
Arhgap32	1308	926	687	1058	853	1362
Arhgap40	0	1	1	1	1	0
Arhgef10l	2050	1142	1104	1816	997	1862
Arhgef11	1971	1296	1016	1590	1240	1978
Arhgef18	1093	647	572	926	584	1028
Arhgef33	4	2	4	2	2	6
Arhgef37	159	120	110	140	97	225
Arhgef7	5650	3556	3042	4864	3505	5096
Arid1a	3543	2626	1765	3258	2154	3491
Arid4b	2434	1745	1295	1988	1594	2595
Arl2bp	2821	1894	2110	2688	1875	3407
Arl5b	611	395	379	533	352	630
Arl6	348	235	209	320	186	385
Arl6ip5	4791	3122	2963	4594	2740	5228
Armc5	456	291	168	315	233	461
Arnt	3088	1995	1575	2657	1821	3010
Arrb1	1851	1156	831	1608	936	1702
Arsb	561	481	276	571	287	591
Arsi	28	13	19	14	22	36
Arsk	470	354	257	426	274	501
Artn	5	0	3	3	4	3
Asah1	2048	1456	1202	1875	1124	2325
Asb10	2612	1422	1190	2019	1453	1997
Asb2	21404	14565	10603	19266	13313	19176
Asb3	467	257	241	378	256	455
Asb4	279	169	211	221	176	390
Asb7	675	404	352	565	362	625
Asb8	3492	2278	2048	3189	2152	3193
Ascl3	1	0	0	0	0	1
Asf1a	872	608	494	806	481	897
Asf1b	56	27	16	36	25	42

Aspm	72	34	27	42	15	95
Atcay	3	4	1	5	2	1
Atcayos	461	396	378	356	371	780
Atf7ip2	0	1	0	0	0	1
Atg12	1701	1174	1067	1521	1076	1763
Atg16l2	419	206	168	334	197	342
Atg4b	1118	624	535	938	501	1031
Atg4c	290	197	172	274	173	299
Atg4d	1922	981	990	1647	854	1715
Atg9a	4048	2450	1767	3559	2138	3404
Atp13a1	1064	624	494	861	530	963
Atp13a2	732	479	341	639	423	683
Atp1a2	33148	26765	18304	35899	18926	36635
Atp1a4	22	0	15	1	23	2
Atp1b4	5	0	0	0	2	2
Atp2a1	37	15	21	25	22	28
Atp4b	0	1	0	0	1	0
Atp5a1	192312	119810	105471	171502	113086	186544
Atp5c1	54111	36374	33853	46075	32314	57493
Atp5h	15273	9217	8878	12811	8055	18330
Atp5j	10785	7781	7932	10055	6825	12943
Atp5k	5442	3326	4317	5271	2873	6747
Atp5o	27021	18632	16186	23673	15658	29766
Atp6ap1	6155	4000	3212	5721	3374	6138
Atp6v0d1	1740	961	889	1529	824	1713
Atp6v0e2	469	303	250	458	192	474
Atp6v1a	1542	955	852	1368	808	1561
Atp6v1d	4942	3865	3274	4526	3163	5896
Atp6v1e1	3799	2440	2085	3197	2102	3819
Atp7a	882	546	437	715	444	861
Atp9a	3237	1789	1468	2624	1514	2958
Atp9b	1324	931	728	1162	761	1376
Atxn10	3910	2797	2415	3743	2378	4404
Atxn2l	3537	2317	1571	2636	2116	3490
AU015791	7	4	4	4	3	12
Aurkaip1	1916	1190	1024	1511	1023	2209
Avil	98	52	49	40	61	105
Avl9	762	453	442	600	464	797
Avpr1b	0	0	2	2	0	1
Axl	4953	3432	2698	5336	2053	5114
B130034C11Rik	14	14	19	11	19	20
B230118H07Rik	2349	1661	1624	1966	1614	2680
B230119M05Rik	0	1	1	1	0	1
B230208H11Rik	14	8	10	16	3	15
B230216N24Rik	7	8	11	4	10	18

B230217O12Rik	43	23	32	29	27	50
B230319C09Rik	9	8	6	8	6	11
B3galt1	164	114	82	186	54	154
B3galt5	2	0	1	1	0	2
B3gat3	894	534	464	727	488	906
B3gnt4	0	1	0	0	0	1
B3gntl1	229	166	140	185	142	284
B430319G15Rik	49	43	49	39	50	64
B4galnt1	102	40	37	71	42	74
B4galt7	284	166	143	239	160	273
B630005N14Rik	1111	693	618	910	635	1222
B930003M22Rik	72	31	32	34	32	75
B9d2	187	122	82	127	105	184
Baalc	71	51	51	85	31	90
Bag1	7357	5325	4521	6183	4624	8295
Bag6	7232	4663	3554	6146	4027	6469
Baiap2	603	370	210	462	295	494
Baiap2l2	3	0	1	2	2	1
Bap1	2186	1213	1127	1893	1081	1890
Batf3	11	3	0	6	2	5
Baz1a	410	269	202	352	210	414
Baz2a	3540	2550	1812	3040	2157	3464
Bbs2	814	544	365	647	506	762
Bbs5	190	129	123	161	129	226
BC021614	0	0	1	1	0	0
BC030499	43	30	25	31	38	42
BC030867	9	6	2	4	2	12
BC037032	191	132	138	182	148	188
BC037034	205	140	118	167	102	241
BC037704	22	42	9	18	24	37
BC039771	1	2	2	3	1	2
BC048403	101	78	62	76	65	129
BC051226	28	20	21	18	19	47
BC051537	1	0	0	0	0	1
BC051628	1	1	1	0	2	2
BC052040	153	111	88	116	119	158
BC094916	1	0	1	2	0	0
BC107364	2	0	1	0	1	2
Bcap31	2047	1394	1206	1825	1166	2414
Bcas1	11	7	8	6	8	18
Bckdk	3845	2494	1985	3287	2105	3632
Bcl10	894	516	582	750	503	1051
Bcl11a	184	134	124	142	145	191
Bcl2	575	379	529	582	312	851
Bcl2l11	922	516	478	608	642	867

Bcl2l12	263	160	131	210	142	288
Bcl2l14	2	1	0	2	0	1
Bcl6	1236	542	436	792	570	917
Bcl7a	877	594	479	706	537	963
Bcorl1	404	243	194	350	220	318
Bdkrb1	0	0	1	1	0	0
Begain	11	5	4	7	1	14
Bend6	43	38	33	44	23	58
Best1	3	6	6	8	4	5
Bfsp1	20	6	13	12	17	11
Bhlhb9	941	710	571	805	622	1053
Bin1	276	188	171	258	146	300
Birc6	5806	3854	2976	5047	3312	5143
Blk	5	5	2	2	6	6
Blm	118	81	59	93	72	134
Blmh	1802	1228	961	1663	956	1819
Bloc1s4	261	168	133	208	146	295
Bloc1s6	1257	800	777	1075	792	1356
Blvra	572	343	350	493	299	613
Bmp15	0	5	0	1	0	7
Bmp2	111	55	31	59	49	90
Bmp5	55	46	31	57	47	41
Bmper	252	137	204	199	153	368
Bmpr1a	6630	4661	4060	6423	4335	6084
Bnc1	5	1	3	3	4	2
Bnipl	3	0	0	1	0	2
Bod1	3519	2206	2068	3169	1794	3856
Bod1l	3444	2521	2082	2785	2275	3836
Bola2	180	124	95	140	99	224
Bphl	994	538	562	747	513	1049
Bpifb1	5	5	1	5	3	3
Braf	2000	1337	1116	1541	1227	2118
Brap	1508	1012	869	1276	996	1488
Brca2	118	95	87	76	81	175
Brd1	2248	1565	1280	1859	1412	2366
Brd2	4386	3151	2299	3551	2732	4738
Brd3	2193	1362	1128	1901	1106	2318
Brdt	53	33	31	41	27	67
Bscl2	615	443	297	527	310	667
Btbd16	7	1	6	5	3	10
Btbd18	0	0	1	0	0	1
Btbd2	1573	809	641	1165	747	1292
Btbd7	1131	796	556	1017	678	952
Btf3	1397	915	884	1174	805	1577
Btg3	39	31	21	26	28	45

Bud31	1154	760	725	977	647	1334
Bzrap1	1	3	3	2	1	4
C030006K11Rik	1328	720	792	1141	747	1352
C030034L19Rik	9	10	8	9	5	16
C030039L03Rik	57	27	32	43	35	51
C130050O18Rik	15	5	7	10	5	17
C1d	1625	978	946	1301	902	1772
C1qc	1056	693	563	905	433	1315
C1qtnf4	94	43	39	69	44	74
C1s1	1946	1201	1087	1727	919	2124
C2	46	13	24	29	18	38
C230029M16	2	0	3	0	1	6
C230037L18Rik	22	14	9	9	13	25
C230052I12Rik	56	59	30	63	41	69
C230091D08Rik	1	1	1	1	1	1
C2cd2l	716	403	406	561	367	816
C2cd4c	3	0	4	3	3	2
C330021F23Rik	2	0	0	0	0	2
C430002E04Rik	1	0	0	0	0	1
C530008M17Rik	140	74	79	97	106	95
C6	13	2	6	6	5	13
C8g	53	29	26	45	32	46
C920021L13Rik	32	16	15	16	12	41
Caap1	247	156	140	189	147	287
Cab39	8714	6014	5378	7828	5671	8353
Cabin1	1710	1065	821	1480	884	1567
Cacfd1	1205	663	608	1070	564	1185
Cacna1c	17217	12754	10352	14488	12097	16652
Cacna2d2	211	174	133	264	131	156
Cacna2d3	33	20	27	29	27	31
Cacna2d4	0	1	1	2	0	0
Cacnb1	233	165	127	159	174	211
Cacng1	1	0	2	1	2	0
Cacng5	1	0	0	0	0	1
Cacng7	56	41	39	54	31	74
Cactin	662	482	340	591	352	682
Cadm4	487	297	246	354	279	487
Cage1	36	19	26	36	33	22
Calb1	1	0	0	1	0	0
Calm2	3751	2470	2097	3108	2135	4298
Calr3	667	410	370	486	416	726
Camk1d	252	160	185	293	120	255
Camk2d	9755	6255	5784	8113	6314	9117
Camsap2	2319	1511	1269	1989	1372	2255
Camta1	7539	6416	5568	7432	5433	8809

Capn6	14	10	5	14	4	12
Capn7	3239	2295	1849	2615	2030	3402
Capza2	8249	5198	4897	7077	4724	8231
Car11	190	135	88	142	111	185
Car14	8130	5441	4522	8083	3837	8531
Car9	2	3	2	2	2	5
Card6	485	342	259	442	285	505
Carns1	2334	1497	943	1620	1194	2503
Cars	1018	576	605	850	546	1024
Casp9	720	467	391	640	406	755
Casr	2	1	0	2	0	1
Cast	7663	5669	4549	7148	4828	7509
Catsperd	10	4	6	6	8	9
Cbfb	1539	1054	946	1412	909	1736
Cbr2	414	275	189	322	198	488
Cbx3	298	199	187	281	168	315
Cbx4	595	387	349	576	351	574
Cbx8	186	85	69	121	79	164
Cc2d1b	842	530	418	661	458	905
Ccar1	4390	3456	2872	4088	2892	4820
Ccbl1	1302	801	668	1142	705	1275
Ccbl2	1884	1163	1092	1611	1019	1912
Ccdc109b	45	36	28	43	16	73
Ccdc116	5	3	4	4	2	7
Ccdc126	293	216	151	264	170	284
Ccdc154	1	2	0	1	2	0
Ccdc157	698	404	273	486	354	698
Ccdc159	152	90	77	83	103	149
Ccdc163	184	141	97	133	123	203
Ccdc166	61	52	40	55	46	75
Ccdc22	601	354	306	502	319	602
Ccdc24	15	10	6	16	7	14
Ccdc28a	370	274	185	293	246	400
Ccdc34	350	271	209	282	216	439
Ccdc40	3	6	3	3	6	3
Ccdc50	2810	1688	1501	2277	1505	2963
Ccdc51	276	192	146	235	165	310
Ccdc53	594	436	398	503	426	732
Ccdc80	4846	3897	3429	5066	2708	6109
Ccdc81	0	3	0	1	0	2
Ccdc91	1555	1074	880	1376	974	1555
Ccer1	0	1	0	0	1	0
Ccl11	67	42	142	47	67	241
Ccl17	2	1	2	3	1	1
Ccnd1	2870	1520	2098	2163	1243	4236

Ccne2	194	141	85	177	86	189
Ccnl1	1497	1020	843	1069	827	1927
Ccnl2	4483	2563	2131	2937	2510	4945
Ccr1	62	23	16	61	14	43
Ccr8	1	0	0	1	0	0
Ccrl2	824	486	383	724	384	755
Ccrn4l	3578	2428	1508	3795	1601	2738
Ccser1	33	28	11	30	24	25
Cct6b	20	4	4	8	8	11
Ccz1	1574	1119	982	1307	1022	1848
Cd163	999	631	615	933	439	1204
Cd163l1	7	10	5	12	2	15
Cd207	6	2	3	8	0	6
Cd209c	2	1	0	2	1	1
Cd22	8	11	9	10	10	15
Cd244	17	9	4	15	5	16
Cd276	229	186	147	222	117	309
Cd300c	2	0	0	1	0	1
Cd36	101315	59676	59848	80399	62484	109554
Cd3g	9	14	4	14	5	11
Cd44	672	472	331	708	289	664
Cd48	83	74	53	75	35	131
Cd59a	3453	2434	2065	3081	2171	3888
Cd59b	28	24	16	28	22	28
Cd63	1763	1131	1038	1521	938	1958
Cd68	279	182	118	196	131	350
Cd97	3308	2065	1387	2821	1457	3005
Cdc123	2045	1383	1171	1746	1246	2066
Cdc27	1847	1371	1070	1684	1153	1868
Cdcp1	7	4	8	3	9	7
Cdh10	3	0	1	2	1	1
Cdh16	36	27	19	27	37	27
Cdh17	0	1	0	0	0	1
Cdh19	546	381	325	428	282	703
Cdh22	77	75	69	97	45	126
Cdhr1	2	1	0	1	0	3
Cdk10	564	330	209	382	259	589
Cdk11b	3285	2648	2190	2937	2236	4321
Cdk14	622	426	379	559	361	719
Cdk16	6981	4921	3566	6213	4244	6776
Cdk2ap1	392	203	197	386	149	348
Cdk2ap2	488	277	243	414	210	502
Cdk4	1508	958	759	1167	805	1532
Cdkl2	371	252	254	352	255	362
Cdkl4	4	1	0	3	1	2

Cdkn2aip	644	439	297	534	363	633
Cdkn3	11	2	10	3	4	25
Cdo1	201	131	62	160	95	149
Cdrt4	3	1	0	2	1	2
Cdsn	43	12	30	31	35	11
Cdyl	507	300	241	397	260	468
Ceacam15	6	7	5	12	3	6
Cecr2	1148	699	617	743	762	1106
Celf4	26	31	13	30	11	37
Celf5	4	7	6	2	9	9
Celsr3	4	0	1	0	4	2
Cenpb	6600	3728	3634	5386	3702	6847
Cenpf	1340	1528	493	1932	852	1130
Cenpo	93	76	65	72	66	119
Cenpp	92	58	60	77	60	108
Cep112	276	190	123	200	163	301
Cep152	232	139	115	207	142	199
Cep162	454	352	288	374	344	515
Cep19	655	484	386	597	421	703
Cep41	99	72	48	84	48	103
Cep70	363	256	289	290	269	511
Cep83os	382	289	200	274	256	430
Cep85l	603	382	306	484	410	543
Cept1	1292	765	716	1142	624	1317
Cercam	104	89	67	105	56	146
Ces2g	123	91	60	116	66	112
Cggbp1	2958	1922	1576	2621	1628	3070
Cgn	3	1	1	4	1	0
Chchd5	276	190	149	199	166	317
Chchd7	1741	1230	1070	1487	1009	2007
Chd2	3001	2186	1786	2382	2284	2990
Chka	550	289	321	477	235	576
ChkbCpt1b	32828	18733	16607	23392	20243	30942
Chl1	151	141	92	114	99	215
Chmp1a	1897	1002	886	1489	940	1689
Chmp2a	2774	2223	1725	2538	1725	3595
Chmp4b	3895	2986	2399	3485	2497	4623
Chmp6	666	378	357	598	326	669
Chn2	169	114	77	159	66	177
Chodl	1	0	1	2	0	0
Chp1	3996	2579	2400	3654	2339	4213
Chrac1	1068	712	608	870	589	1224
Chrm3	23	16	17	22	13	33
Chst1	132	77	63	107	53	154
Chst12	194	151	85	182	93	232

Chst8	4	2	5	2	0	11
Chsy1	671	482	400	662	382	672
Ciao1	1620	1216	975	1441	987	1792
Cib2	18	6	19	14	11	28
Cipc	3991	2565	2027	3411	2405	3683
Cit	92	48	34	55	39	106
Ciz1	1458	1161	681	1217	913	1585
Ckap2l	46	39	20	33	13	91
Clasp2	4597	3264	2768	4031	2756	4937
Clasrp	696	516	307	483	435	799
Clcn3	3005	1855	1560	2196	1850	2769
Clcn6	338	248	164	310	223	315
Clcnka	0	0	1	0	0	1
Clec18a	113	58	34	54	61	89
Clec5a	54	39	32	39	21	78
Clec9a	37	15	9	42	6	21
Clgn	2	1	0	1	1	1
Clic3	6	4	0	2	5	2
Clic6	8	5	11	11	9	4
Cln6	646	456	391	559	457	681
Clpp	1631	1004	794	1277	840	1680
Clptm1	4525	2969	2339	4074	2588	4351
Clta	2293	1439	1201	1879	1227	2357
Cma1	11	8	8	9	6	13
Cmas	1384	969	773	1137	809	1479
Cmc1	538	381	399	447	361	690
Cmtm5	6	1	5	4	6	5
Cmtm7	246	127	127	215	101	224
Cnbp	15595	10552	9546	12995	9599	17102
Cngb3	363	277	173	289	223	351
Cnih1	2498	1593	1425	2268	1310	2792
Cnksr1	472	242	270	353	267	482
Cnksr2	11	7	11	11	6	15
Cnnm1	1	0	3	1	2	2
Cnot11	1151	679	646	1040	633	1124
Cnot6l	3413	2147	1728	3022	1941	3245
Cnpy2	771	576	469	691	456	962
Cnr1	1	1	0	0	0	2
Cntn4	2	3	1	2	1	3
Cntnap1	236	142	97	174	130	216
Cntrl	1191	852	668	836	780	1362
Cntrob	231	160	117	166	141	244
Coa3	1715	1097	914	1445	846	1839
Cog1	1053	698	504	943	587	1017
Col11a1	4	5	3	7	2	6

Col16a1	537	294	208	423	206	504
Col19a1	6	7	1	7	2	9
Col6a1	3414	2310	1758	3322	1357	4275
Col6a2	2896	1830	1389	2750	996	3599
Col6a5	3	1	0	2	1	2
Colq	263	173	204	282	117	344
Commd2	385	227	227	339	186	393
Commd4	354	211	176	275	172	401
Commd8	1553	946	849	1270	885	1507
Comp	89	145	78	176	72	104
Copa	8570	7069	4498	8856	4703	9094
Copg2	2047	1411	1165	1948	1180	1883
Cops4	3577	2574	2094	3172	2172	3753
Cops7a	2619	1768	1303	2096	1533	2539
Copz1	1806	1064	950	1537	903	1903
Coq10a	10643	5910	5635	7948	5843	10540
Coq6	1910	1032	1041	1616	943	2022
Cotl1	281	177	112	209	109	345
Cox14	1973	1116	1073	1686	998	2085
Cox18	292	197	182	236	182	346
Cox4i1	53546	35264	31623	45162	31879	62962
Cox6a1	1348	928	957	1279	770	1663
Cox6b1	16977	11692	11732	15646	10244	21116
Cox6b2	20	18	14	10	21	31
Cox6c	15961	11853	10638	12891	8998	22130
Cox7a1	10762	7303	7821	10534	6291	13825
Cox7a2	9717	6311	5994	8147	5169	11292
Cplx3	0	0	1	0	0	1
Cpne5	91	42	41	77	62	46
Cpsf3l	689	371	348	566	344	681
Cpt1b	31421	17956	15880	22407	19459	29044
Cpz	7	18	3	16	6	14
Cr1l	1663	1111	941	1521	963	1767
Cr2	2	3	3	4	1	5
Crbn	2829	2002	1491	2419	1800	2878
Crcp	349	284	248	327	253	429
Crkl	2352	1420	1322	2247	1188	2091
Crtac1	1	1	2	1	0	4
Crtap	986	648	526	869	486	1049
Crtc1	586	327	236	438	305	483
Crtc2	958	580	422	725	522	960
Crtc3	1013	700	426	980	537	824
Cry2	2050	1278	988	1824	1062	1734
Crygn	0	2	2	4	1	0
Crym	4	5	1	3	0	7

Csf2rb2	59	32	24	41	28	56
Csgalnact2	1833	1208	1096	1449	1082	2018
Csnk2a1	4205	2552	2695	3651	2419	4540
Cst6	37	18	34	34	20	42
Cstf1	691	445	395	624	391	671
Ctcf	3067	2072	1662	2673	1898	3082
Cth	237	97	94	123	117	204
Ctnnd2	14	7	6	14	2	16
Ctsa	5767	3640	2898	4994	3139	5886
Ctsb	20988	13806	12239	20198	11393	23077
Ctsc	1802	1150	878	1526	817	1937
Ctsw	20	13	5	15	8	17
Ctu1	123	51	58	101	47	97
Ctxn1	45	20	9	27	10	38
Cux1	5036	3650	2915	4857	3362	4579
Cwc15	2136	1650	1437	1992	1399	2641
Cwc22	698	482	420	549	468	805
Cwc27	840	650	625	697	600	1051
Cwf19l1	337	201	166	273	175	305
Cwf19l2	1423	1043	846	1177	899	1626
Cxcl16	394	283	185	305	188	467
Cxcl17	0	1	0	0	1	0
Cxcr2	2	3	6	9	0	3
Cxcr3	10	7	2	15	0	8
Cxcr6	19	13	8	28	6	13
Cxx1a	373	273	178	335	199	366
Cxx1b	629	360	272	555	270	611
Cyb5	1990	1405	1225	1647	1245	2268
Cybb	572	393	271	462	240	686
Cyfip2	9858	5871	5344	7789	7388	7594
Cyp2ab1	21	14	8	16	10	25
Cyp46a1	28	16	10	14	20	29
Cyp4a10	2	0	0	1	0	1
Cyp4f17	156	79	79	83	89	156
Cyth4	280	172	138	223	116	315
D10Wsu102e	471	335	248	436	237	488
D130043K22Rik	1	1	0	0	1	1
D17Wsu92e	9154	5309	4857	7332	5785	7308
D2hgdh	1171	838	596	1019	771	963
D330045A20Rik	1	3	2	2	2	4
D330050G23Rik	3	8	4	3	8	7
D430020J02Rik	13	10	11	10	7	26
D4Ertd617e	86	49	59	72	58	97
D630003M21Rik	394	292	184	497	150	350
D630010B17Rik	3	0	0	1	0	3

D630023F18Rik	0	0	1	0	0	1
D630029K05Rik	0	2	0	2	0	0
D630045J12Rik	74	58	30	75	41	56
D8Ertd738e	2243	1375	1384	1988	1210	2613
D930015E06Rik	1373	871	723	1034	753	1454
D930015M05Rik	1	1	0	2	0	0
D930028M14Rik	0	1	0	0	0	1
Dact1	470	272	158	423	204	308
Daf2	1	0	0	1	0	0
Daglb	482	264	250	422	247	455
Dancr	94	53	54	74	45	114
Dapk3	638	453	287	510	364	683
Dbhos	0	4	0	1	2	1
Dbn1	324	227	190	330	147	413
Dcaf11	8229	5055	4061	6937	4574	7924
Dcaf12	1946	1211	1027	1699	969	1919
Dcaf5	2398	1637	1538	2148	1690	2205
Dcaf8	8681	6460	4920	7966	5794	8995
Dcp1a	922	527	484	707	519	888
Dcstamp	0	1	0	0	0	1
Dct	154	146	138	167	143	171
Dctn3	905	538	529	738	436	1048
Dcun1d3	655	474	440	552	449	735
Dda1	1211	765	652	1030	678	1169
Ddb1	10296	6768	5442	9560	5623	9254
Ddc	185	98	91	108	132	165
Ddi2	1270	937	707	1169	827	1242
Ddn	24	18	21	28	19	22
Ddost	2288	1469	1202	1967	1109	2398
Ddrgk1	2329	1602	1258	1820	1443	2557
Ddx10	624	503	366	553	456	682
Ddx11	80	67	44	76	39	93
Ddx17	13879	8733	6826	10035	7586	14417
Ddx19a	789	577	423	728	419	812
Ddx20	668	499	392	668	387	759
Ddx23	2760	1835	1514	2354	1658	2614
Ddx28	369	245	221	338	187	465
Ddx4	26	11	18	20	17	29
Ddx42	2629	1835	1414	2364	1575	2751
Ddx46	3672	2643	2033	2923	2284	3878
Ddx5	27311	16365	14278	21418	15576	28309
Ddx55	375	262	229	305	224	426
Ddx56	854	496	450	689	444	819
Decr1	19164	11244	12352	14252	11592	21267
Defb19	0	2	1	1	1	1

Dennd1c	88	71	32	72	45	108
Dennd5a	9130	6370	5219	8534	5554	9377
Depdc1a	9	0	1	0	1	9
Derl1	2678	1469	1302	2257	1322	2517
Des	80827	56473	45334	72168	50429	86009
Dgcr14	431	297	229	346	252	456
Dgki	8	4	2	11	0	3
Dgkq	663	405	301	528	306	686
Dhcr7	277	161	134	211	137	274
Dhodh	618	378	327	505	323	632
Dhrs1	1333	898	782	1244	748	1484
Dhrs4	2731	1709	1657	2250	1667	3049
Dhrs7	1564	1050	863	1393	856	1703
Dhx40	1342	1079	873	1220	858	1650
Dhx8	1107	748	605	1001	632	1066
Disp2	4	2	3	5	1	5
Dkk1	0	2	0	0	1	1
Dkk3	708	703	606	1023	624	479
Dlc1	5879	3965	3501	5248	3698	6226
Dleu2	390	260	212	319	247	414
Dlg4	219	135	98	192	101	198
Dlg5	701	491	339	603	385	694
Dlgap3	9	3	2	5	2	10
Dlgap4	2391	1618	1315	2015	1501	2493
Dmap1	624	368	305	452	340	660
Dmpk	22133	13255	10003	16602	11672	20853
Dmtf1	1441	958	826	1182	884	1580
Dnaaf2	152	110	68	117	87	152
Dnaaf3	361	277	204	303	248	398
Dnah11	0	2	0	0	0	2
Dnajb13	2	3	0	3	1	2
Dnajb14	324	186	194	299	180	279
Dnajc14	1910	1336	1211	1707	1269	2136
Dnajc22	0	0	2	1	0	1
Dnajc30	1115	799	652	1000	694	1196
Dnajc6	13	11	14	16	6	20
Dnase1	0	0	1	1	0	0
Dnase1l1	527	336	287	404	324	591
Dnase1l2	83	38	38	58	42	67
Dnd1	54	20	34	33	25	59
Dnlz	956	571	539	805	513	1044
Dnmt1	1246	870	578	1060	696	1178
Dntt	1	0	0	1	0	0
Dock2	177	135	79	162	79	197
Dock7	931	638	464	837	462	934

Dohh	892	505	351	704	425	789
Dok3	75	57	43	64	42	96
Donson	228	112	115	155	106	239
Dopey1	1598	1082	872	1343	992	1626
Dot1l	2529	1826	1172	1836	1481	2655
Dpcd	386	247	220	320	210	400
Dpf1	4	4	4	3	3	9
Dph1	342	174	148	277	167	281
Dpp7	127	73	63	90	67	148
Dpy30	773	533	504	677	513	859
Dqx1	86	51	30	48	49	87
Dram1	252	174	175	247	144	284
Drd1a	3	4	10	7	5	11
Drg1	1857	1181	1190	1634	1024	2094
Dscr3	1253	652	581	953	615	1137
Dtl	22	10	15	11	6	47
Dtwd1	305	173	142	216	179	265
Dtx4	463	252	203	431	150	424
Dus1l	780	467	415	613	378	850
Dus3l	907	582	407	688	468	916
Dusp19	251	163	130	217	132	272
Dut	675	429	395	564	382	753
Dync1i1	1	1	2	2	1	1
Dync1i2	4054	3020	2489	3884	2560	4565
Dynlt1f	37	28	23	28	28	48
Dyrk1b	1011	591	346	716	529	881
Dzank1	87	65	49	71	61	82
Dzip1l	229	160	129	209	118	247
E130012A19Rik	80	22	19	53	25	59
E130102H24Rik	175	95	105	126	102	211
E130307A14Rik	202	130	94	156	108	220
E130309D02Rik	553	318	286	496	320	452
E130309D14Rik	14	10	12	9	15	15
E2f5	216	162	110	174	119	252
E2f8	71	38	38	36	53	74
E4f1	642	408	288	493	324	617
E530011L22Rik	185	125	92	129	124	197
Eaf1	1250	720	720	1109	722	1233
Eapp	920	635	584	791	564	1112
Echdc3	1044	670	588	810	624	1150
Edf1	2821	2104	1809	2538	1793	3408
Eea1	3104	2309	1834	2664	2196	3163
Eef1d	4580	2987	2543	3881	2561	5023
Eef2	66986	43148	31856	59971	38394	61923
Efcab14	2171	1486	1233	2062	1299	2113

Efcab5	53	32	25	30	34	53
Efhb	8	3	4	7	4	4
Efhc1	38	23	23	27	25	36
Efna5	1159	797	521	863	682	1090
Eftud2	1695	1128	857	1461	967	1748
Egfem1	4	1	2	5	1	1
Egfl6	2	1	0	1	1	2
Egln2	1659	960	838	1349	860	1728
Egr1	577	399	305	468	299	658
Ehhadh	692	415	521	485	429	1038
Ei24	3545	2248	2202	3193	2015	3716
Eid3	9	4	7	6	6	8
Eif1	10542	5298	6167	8799	5303	11243
Eif2ak4	545	355	290	424	311	619
Eif2b1	1021	662	585	866	548	1193
Eif2b5	2786	1808	1508	2326	1512	2955
Eif2d	1500	931	766	1228	817	1519
Eif3b	5709	3737	3027	4801	3214	5536
Eif3d	2528	1731	1337	2267	1344	2646
Eif3g	2246	1576	1320	1945	1323	2432
Eif3h	5559	3800	3247	4676	3314	6103
Eif3k	2860	1782	1657	2423	1590	3169
Eif3m	3225	2191	1868	2763	1797	3577
Eif4a1	1468	836	1026	1242	842	1855
Eif4b	11647	8337	6304	10268	7448	10795
Eif4e3	3836	2529	2258	3447	2217	3932
Eif5a	8244	5486	4698	7458	4785	8698
Elac2	1075	709	569	919	654	1045
Elavl4	6	5	5	4	5	7
Elk4	1111	815	606	834	806	1152
Elof1	552	371	317	520	284	637
Elovl1	791	467	405	687	384	816
Elovl2	3	3	1	3	1	5
Elovl3	0	2	0	1	1	0
Elp2	1371	972	803	1188	761	1552
Elp4	457	327	287	406	301	464
Emc10	2241	1330	908	1884	1048	1987
Emc4	874	522	474	752	412	904
Emc7	1643	1089	1060	1565	935	1908
Eme1	9	2	4	5	2	12
Emilin2	3357	2520	1707	4077	1437	3315
Eml4	594	381	320	471	291	683
Emp1	3339	2183	1880	3477	1375	3859
Endog	1091	633	508	871	522	1130
Eno1	1695	867	806	1447	661	1598

Eno1b	1696	868	807	1447	661	1599
Eno3	69545	41945	36078	64099	30337	75140
Enpp4	2545	1599	1346	2259	1471	2419
Enthd2	376	254	167	319	191	339
Entpd5	10546	6644	5603	8517	7062	9167
Eomes	5	1	1	4	0	5
Ep400	2128	1549	1111	2042	1213	2135
Epb4.1l5	503	316	250	403	273	545
Epg5	975	682	446	856	576	902
Ephb2	17	21	13	24	11	25
Epn1	6874	4059	3536	5901	3768	6663
Eppin	0	0	1	0	0	1
Eps8	769	534	402	729	367	863
Epyc	11	8	7	10	8	12
Erc1	3042	2139	1484	2526	2121	2339
Ercc3	976	727	553	853	591	1041
Ercc4	571	403	331	526	337	623
Ergic3	1725	1122	892	1449	895	1750
Erp27	5	2	1	3	2	5
Erv3	2	0	1	0	1	2
Esd	2937	2124	1860	2642	1714	3643
Espn	88	77	30	66	64	64
Esrp2	18	8	12	8	14	17
Esyt2	3392	2072	1705	3150	1701	3277
Etfa	28673	18478	17920	23574	17343	29910
Evc	391	297	208	380	241	378
Evi2a	135	108	70	138	65	140
Exo1	11	6	5	6	2	17
Exoc5	2006	1196	1141	1688	1127	2053
Exoc6b	1384	1013	773	1227	879	1394
Exosc2	307	211	144	252	164	327
Exosc5	257	130	84	163	120	230
Exosc6	100	78	54	88	51	129
Exosc8	439	400	267	375	321	542
Ext2	1778	1208	932	1609	957	1773
Extl2	1030	800	586	969	592	1229
Eya4	32	26	14	26	13	39
F10	14	3	5	3	6	14
F3	1431	932	692	1168	1046	1112
F420014N23Rik	18	11	9	12	11	17
F630111L10Rik	3	1	0	1	1	2
F8a	209	148	115	197	103	242
Fabp1	0	0	1	0	0	1
Fabp3	38228	22325	28591	26772	27529	43194
Fadd	615	367	379	553	373	602

Fahd1	2409	1549	1561	2082	1415	2751
Fam101a	2	1	0	1	1	2
Fam102a	755	336	272	561	337	483
Fam104a	662	450	405	609	412	717
Fam107b	331	193	199	276	169	366
Fam114a2	2257	1616	1428	1922	1364	2601
Fam118b	594	302	284	474	281	559
Fam126b	1504	892	788	1195	911	1467
Fam129c	39	25	27	45	20	40
Fam132b	7	3	0	1	5	5
Fam134b	16800	11409	10689	12575	11186	18822
Fam135a	2047	1339	1158	1655	1222	2130
Fam161a	71	53	46	45	68	84
Fam174a	1610	969	991	1440	862	1686
Fam179a	262	148	127	204	158	210
Fam180a	8	19	3	11	7	16
Fam183b	12	2	3	3	8	5
Fam188a	692	506	414	599	403	818
Fam189b	212	112	76	154	102	197
Fam192a	1065	735	682	987	643	1203
Fam195b	1054	647	564	845	538	1123
Fam196b	3	0	0	1	1	1
Fam199x	595	328	322	488	301	615
Fam19a5	230	144	118	151	153	216
Fam203a	107	58	69	76	68	104
Fam204a	639	548	420	563	440	789
Fam208a	2290	1365	1096	1640	1310	2407
Fam20c	541	295	263	469	235	513
Fam212b	1861	1361	740	2358	820	1228
Fam216a	278	161	138	231	129	301
Fam219b	600	381	324	491	329	611
Fam221a	30	12	14	13	18	35
Fam222b	903	658	529	889	510	910
Fam228b	90	78	49	62	54	130
Fam229b	109	78	67	85	66	146
Fam32a	1248	866	661	1055	680	1409
Fam43b	0	1	0	0	0	1
Fam45a	785	532	478	655	462	854
Fam47c	0	0	1	1	0	0
Fam50a	944	633	603	847	538	1043
Fam57a	60	36	22	53	20	61
Fam58b	482	270	246	370	265	451
Fam69a	315	204	251	312	253	329
Fam71d	1	0	3	3	0	3
Fancd2	22	11	8	9	12	27

Fancl	279	172	128	230	133	289
Fap	249	187	132	216	114	351
Far1	1738	1090	909	1502	876	1757
Fasl	4	1	1	6	0	1
Fasn	1591	1246	619	1845	667	1281
Fastkd3	426	238	257	351	213	473
Fat2	6	3	6	6	6	5
Fbn2	63	51	46	68	31	100
Fbxl12	369	222	169	261	195	361
Fbxl12os	18	7	14	14	14	17
Fbxl14	762	486	394	695	428	741
Fbxl17	2627	1855	1434	2466	1549	2684
Fbxl8	74	24	30	43	32	59
Fbxo11	3102	2157	1848	2639	1959	3278
Fbxo18	3763	2524	2079	3374	2207	3572
Fbxo28	1438	894	855	1270	825	1437
Fbxo3	5319	3416	3130	4696	3217	5144
Fbxo33	532	314	257	396	279	586
Fbxo38	1767	1262	871	1425	1028	1801
Fbxo39	7	5	1	2	2	12
Fbxo44	546	346	278	414	341	548
Fbxo7	584	416	338	504	336	725
Fdx1	1394	893	993	1269	857	1737
Fermt2	11241	7823	7130	10280	7708	11949
Fermt3	169	103	59	119	64	173
Ffar2	1	0	4	4	0	1
Fgd2	88	54	36	65	36	97
Fgf16	593	477	327	573	359	685
Fgf17	7	0	2	6	1	2
Fgf21	0	1	1	1	1	0
Fgf6	6	2	6	5	6	2
Fggy	227	103	116	157	105	219
Fh1	18807	12068	10368	15981	10261	18949
Fhdc1	14	8	10	15	12	11
Ficd	557	367	284	529	298	551
Fign	945	652	678	772	715	1083
Fignl2	133	71	57	93	62	145
Filip1l	2505	1984	1478	2291	1667	2806
Firre	2841	1911	1338	1706	1679	3674
Fiz1	773	453	398	623	447	725
Fkbp15	1250	872	676	1073	804	1232
Fkbp9	6534	4074	3367	5728	3470	6409
Flii	6846	4631	3477	6224	3867	6621
Flot2	3288	2440	1657	2917	2020	3273
Flt3	31	11	2	24	7	9

Fmn2	10	4	5	12	5	5
Fmnl2	806	535	397	645	429	914
Fmo5	1302	955	727	1108	837	1331
Fndc3a	1730	1125	793	1253	974	1733
Fndc3c1	1	1	1	2	1	1
Fndc4	150	88	72	112	77	140
Fnta	2853	1740	1630	2385	1559	2864
Fopnl	2634	1685	1567	2339	1529	2763
Fosl1	4	0	2	1	1	5
Foxj2	2695	1872	1539	2316	1725	2653
Foxk1	1378	913	704	1246	833	1220
Foxm1	72	49	29	54	18	116
Foxn2	723	406	375	631	363	695
Foxo4	3049	1617	1565	2465	1813	2591
Foxq1	10	8	10	6	13	9
Foxred1	3527	2129	2129	3022	1922	3696
Fras1	43	33	36	42	47	34
Frat2	113	53	49	80	50	120
Frk	139	93	102	123	82	164
Frmd4b	1053	689	494	807	569	1086
Frzb	240	170	182	311	84	311
Fsbp	27	13	13	16	16	31
Fsd1	0	2	1	0	1	3
Fstl3	208	98	93	139	108	194
Fth1	52821	36621	31991	43427	31409	61589
Ftl1	3375	2290	1947	2744	1960	3714
Fto	3893	2456	2079	3580	2310	3608
Ftsj1	432	279	240	365	251	429
Ftsj2	398	239	204	311	220	413
Fuca2	7022	5063	5400	6174	5758	7813
Fuom	760	554	400	646	420	858
Fus	5761	4014	3481	4453	3694	6493
Fut1	2	0	1	0	2	1
Fut10	250	168	119	261	121	228
Fut2	30	19	24	35	11	41
Fut8	1181	881	771	1065	778	1264
Fuz	478	284	201	364	251	446
Fxn	431	269	273	370	239	462
Fxyd7	23	24	7	23	18	22
Fyn	2588	1666	1208	2267	1295	2563
Fyttd1	5292	3539	3167	4721	3271	5012
Fzd1	486	301	218	483	202	405
Fzd2	215	151	81	211	105	153
Fzr1	1021	639	523	906	570	977
G530011O06Rik	58	15	10	48	7	28

G630090E17Rik	233	139	123	217	108	223
Gab2	1543	1098	900	1501	866	1600
Gabarap	7879	5405	4475	6878	4648	8659
Gabpb1	699	541	408	628	426	861
Gad1	2	1	1	2	3	0
Gadd45gip1	1142	821	691	972	688	1306
Galk2	459	285	256	405	242	499
Galnt14	0	1	0	0	1	0
Galnt3	3	3	3	4	0	6
Galt	1362	911	683	1103	677	1468
Gan	91	69	52	80	64	87
Ganc	952	667	538	764	626	962
Gar1	191	123	162	172	129	234
Gas5	1274	1032	841	1158	839	1530
Gas8	209	198	136	227	119	259
Gata1	0	2	1	3	0	1
Gatad1	3448	2413	2399	3202	2452	3780
Gatm	152	112	61	81	77	196
Gba	665	474	354	621	345	768
Gcc1	670	445	347	601	397	652
Gcc2	1794	1268	1041	1552	1166	1906
Gch1	277	153	183	248	138	331
Gck	675	280	613	448	621	515
Gclm	928	730	524	791	650	1022
Gcsam	20	4	1	12	3	7
Gdap2	624	459	348	521	349	721
Gdf11	38	21	20	26	20	47
Gdf2	3	0	1	1	1	2
Gdf5	1	0	2	0	2	1
Gdpgp1	99	47	53	90	34	95
Gemin2	283	159	147	225	151	297
Gfer	366	275	222	314	212	424
Gfi1	4	1	1	6	1	0
Ggact	493	315	293	375	309	513
Ggt1	4	0	1	2	0	3
Ghr	10901	7826	6366	9939	6847	11512
Gimap3	41	34	19	44	21	47
Gimap7	5	2	3	6	2	4
Gins2	30	19	10	15	12	41
Git1	3109	2042	1571	2540	1941	2908
Gjc2	19	13	12	22	9	21
Gkap1	532	355	304	402	316	579
Glb1	862	588	473	803	441	864
Glcci1	743	454	369	524	429	794
Gli1	86	49	39	83	28	81

Glmn	214	158	148	204	141	270
Glp2r	2	0	0	0	0	2
Glrb	9	5	8	7	9	7
Glrx3	1782	1057	973	1460	1007	1725
Glt25d1	2243	1481	1140	1984	1225	2263
Glt28d2	346	244	172	351	190	283
Gltpd1	222	118	109	196	105	209
Gltscr1	804	529	252	651	451	632
Glud1	7550	5194	4026	6424	4336	7591
Glyctk	86	50	53	60	53	104
Glyr1	3271	2233	1786	2839	1879	3250
Gm10033	468	348	278	386	313	504
Gm10400	7	5	0	7	2	3
Gm10532	6	2	3	3	5	3
Gm10536	3	8	3	4	2	9
Gm10584	1	1	1	2	0	2
Gm10782	0	1	1	0	0	3
Gm10785	6	3	3	6	1	8
Gm10814	15	6	6	6	7	14
Gm1123	1	1	1	0	1	2
Gm11517	23	22	7	19	9	37
Gm11696	88	55	45	57	59	78
Gm11944	65	36	26	45	40	55
Gm12185	134	32	22	94	38	63
Gm12191	410	166	347	421	179	520
Gm128	3	0	0	0	1	2
Gm13139	190	134	139	165	138	227
Gm13157	405	304	229	333	235	479
Gm13293	0	0	1	0	1	0
Gm14164	1	0	0	1	0	0
Gm14322	38	40	26	33	32	48
Gm14327	3	3	4	1	1	12
Gm15008	0	0	1	1	0	0
Gm15401	0	2	1	3	0	1
Gm15455	4	1	0	0	0	6
Gm15706	46	19	19	24	26	48
Gm15800	3033	1987	1379	2419	1833	2588
Gm15880	8	2	2	2	1	9
Gm15910	0	2	0	0	0	2
Gm15987	1	0	0	0	0	1
Gm16039	580	320	314	460	316	540
Gm16062	37	21	27	51	15	39
Gm166	374	255	196	321	193	385
Gm16938	7	2	5	3	3	11
Gm16982	2	0	0	1	0	1

Gm16998	3	7	4	3	2	11
Gm17746	0	1	1	0	0	2
Gm19461	438	309	218	409	245	390
Gm1966	79	84	59	122	31	96
Gm19689	34	20	10	15	20	36
Gm19710	3	0	1	2	1	2
Gm1976	109	72	70	81	78	124
Gm20110	1	0	0	1	0	0
Gm20554	46	25	28	41	18	56
Gm21057	1	3	1	3	1	2
Gm21949	5465	3511	3083	4558	3454	5143
Gm2373	0	3	0	0	1	3
Gm2518	13	10	8	8	10	16
Gm266	15	6	9	18	4	11
Gm3219	52	20	15	28	19	44
Gm3417	0	1	0	0	0	1
Gm3448	0	1	0	0	0	1
Gm3558	9	10	11	8	14	12
Gm3604	90	79	67	71	69	139
Gm3776	2	0	1	0	1	2
Gm4489	0	0	1	0	0	1
Gm4566	0	0	1	0	0	1
Gm4925	20	12	11	10	12	23
Gm5082	1	0	0	1	0	0
Gm5088	0	0	1	0	0	1
Gm5127	3	2	1	2	2	2
Gm5420	5	3	2	2	5	3
Gm5431	32	41	20	32	19	54
Gm5483	0	1	0	0	0	1
Gm5547	2	0	1	2	0	2
Gm5617	201	108	109	160	107	209
Gm5643	546	375	308	387	331	620
Gm614	10	5	8	9	7	12
Gm6277	25	13	18	21	14	24
Gm6307	74	49	24	61	37	65
Gm694	20	10	14	9	16	22
Gm7173	1	1	0	0	0	2
Gm7334	39	31	21	26	28	45
Gm7788	1	0	0	0	0	1
Gm7854	1	4	2	4	0	3
Gm8179	1	0	0	1	0	0
Gm8579	1	0	0	0	0	1
Gm8580	1	0	0	1	0	0
Gm8787	0	2	1	2	0	2
Gm973	2	1	1	0	1	3

Gm9920	5	1	2	3	4	1
Gm9994	6	4	0	2	2	6
Gmeb1	644	387	337	559	337	616
Gnat1	1	1	0	0	1	1
Gnat3	16	9	9	8	7	22
Gnb2l1	9618	5405	4785	8003	4500	10128
Gnb5	1065	715	659	1068	560	1186
Gng13	1	1	2	1	1	2
Gng8	15	15	3	14	8	12
Gnptg	412	258	220	301	215	467
Golga2	4939	3526	2711	4188	3297	4548
Golga5	1665	1172	1016	1382	1125	1707
Gopc	1557	980	923	1306	893	1752
Gorasp2	3227	2240	1971	3026	1884	3350
Gosr1	1710	1112	932	1474	956	1656
Gosr2	2106	1272	1143	1921	1062	2005
Gp49a	115	80	52	75	53	151
Gp9	18	5	11	10	13	16
Gpaa1	744	363	293	604	314	639
Gpalpp1	1525	1101	916	1365	1031	1582
Gpat2	0	0	3	1	2	0
Gpatch1	822	614	505	638	669	886
Gpatch11	949	628	589	813	554	1037
Gpatch3	179	78	74	116	78	161
Gphn	1779	1306	997	1596	1146	1739
Gpm6a	198	187	88	128	166	251
Gpn1	838	477	472	681	453	847
Gpr101	0	0	1	1	0	0
Gpr124	715	454	315	582	326	741
Gpr132	22	15	10	21	9	22
Gpr135	13	5	3	8	4	9
Gpr137b	171	118	106	147	90	232
Gpr15	1	3	0	2	2	1
Gpr150	1	0	1	1	0	1
Gpr156	5	6	0	6	0	8
Gpr161	46	17	12	32	13	30
Gpr17	2	0	2	0	2	3
Gpr173	21	13	9	18	10	22
Gpr179	2	0	0	1	0	1
Gpr180	877	507	444	716	443	862
Gpr182	197	150	54	151	102	207
Gpr19	124	84	76	102	75	133
Gpr20	20	11	18	23	15	21
Gpr33	0	0	2	1	0	1
Gpr35	20	23	15	19	15	28

Gpr62	4	0	0	2	1	0
Gpr64	61	39	49	45	40	77
Gpr68	7	8	6	9	5	9
Gpr88	14	4	7	7	6	12
Gprin1	0	0	1	1	0	0
Gprin2	0	0	1	0	1	0
Gps1	304	173	144	223	149	299
Gpsm1	6848	4105	3316	5864	3230	6440
Gpx1	2362	1853	1459	2171	1433	2786
Gramd4	2133	1297	1112	1684	1284	2095
Grem2	0	2	1	1	0	2
Gria2	0	1	0	1	0	0
Gria3	86	68	63	71	65	113
Grik5	365	227	141	267	209	303
Grin1os	5	2	2	0	0	9
Grin2d	47	31	12	35	27	39
Gripap1	1504	1156	832	1223	978	1580
Grk4	138	101	100	131	86	156
Grm1	2060	1232	1074	1316	1369	1936
Grn	4158	2424	1874	3236	1887	4437
Grpel1	3709	2216	2370	3054	2128	4158
Grtp1	64	40	46	51	42	79
Gse1	1410	888	527	1145	813	1064
Gskip	434	267	251	400	238	464
Gsta3	1092	743	572	1094	497	1190
Gstm1	7929	5224	4058	6498	4450	8488
Gstm5	1353	1052	777	1267	791	1582
Gsto2	22	24	25	27	29	29
Gstp2	56	46	40	75	23	64
Gstz1	1507	1027	892	1261	891	1657
Gtf2a2	1530	1031	977	1195	916	1823
Gtf2h4	346	205	171	301	172	359
Gtf2h5	1083	706	738	978	655	1328
Gtf2ird1	554	379	420	457	467	598
Gtf3a	618	408	373	529	367	718
Gtf3c6	770	641	533	651	607	947
Gtpbp1	1513	1036	696	1389	777	1324
Gucd1	1872	1215	1044	1560	1098	1996
Gucy2c	1	0	0	0	0	1
Gucy2g	16	7	7	9	10	11
Gxylt1	1250	833	673	1020	715	1400
Gyg	8061	5654	5322	7186	4981	8940
Gys1	5848	4035	3145	5472	3691	4850
H2afv	2060	1293	985	1631	1055	2037
H2afx	272	133	110	199	130	223

H2afy	1749	934	919	1472	805	1860
H2-DMb2	12	4	5	7	7	7
H2-Eb2	1	2	1	2	1	2
H2-Ke2	912	621	547	804	524	1073
H2-Ke6	1683	1130	946	1308	1070	1880
H2-M5	7	0	3	2	3	7
H6pd	1881	1117	1009	1680	1052	1737
Habp2	0	1	2	1	1	1
Haus2	953	638	568	815	589	955
Hc	3	3	3	2	1	7
Hcn2	1281	871	681	1052	752	1312
Hcn4	245	200	151	304	163	175
Hcrtr1	1	1	0	0	1	1
Hdac11	1000	659	508	841	589	974
Hdac8	194	140	117	170	95	273
Hddc3	165	111	96	139	97	187
Hdgf	6241	4062	3800	5549	3713	6407
Hdgfrp3	891	632	551	748	564	993
Hdhd3	43	40	34	61	33	45
Heatr2	506	333	261	490	290	445
Heca	1927	1189	1077	1648	1237	1737
Hecw1	12	1	2	6	4	7
Helz	901	752	429	840	610	726
Hemgn	1	2	0	1	0	2
Hemk1	652	397	365	520	349	684
Hepacam	2	0	1	0	0	4
Herc2	4959	3405	2494	4501	3070	4428
Herpud2	2255	1640	1328	2023	1452	2461
Hexb	1151	765	626	951	579	1338
Hexim1	1518	868	773	1302	816	1265
Hexim2	59	33	35	46	35	62
Hfe	1561	1097	1091	1450	949	2022
Hiat1	1551	889	828	1326	783	1619
Hic1	612	333	226	533	244	485
Hid1	134	95	59	93	72	146
Hira	849	529	422	766	423	855
Hist1h2ac	3	0	1	3	0	2
Hist1h3f	1	2	1	2	2	1
Hist2h2be	502	430	337	466	435	525
Hivep1	2211	1713	1195	2199	1482	1821
Hivep2	2451	1825	1229	2056	1562	2338
Hjurp	2127	1530	1007	1665	1167	2567
Hk1	8587	6625	4871	9198	5606	7938
Hk1os	6	3	3	3	1	9
Hlf	3407	2472	1599	3297	1735	3122

Hmbox1	545	400	285	440	381	466
Hmces	220	149	126	209	114	255
Hmg20a	1589	1106	917	1341	1007	1652
Hmgb1	714	467	428	603	421	763
Hmgn1	1184	770	738	970	728	1255
Hmgn2	1369	1145	930	1190	1052	1685
Hmgn5	1257	1008	783	1049	968	1427
Hnrnpa2b1	19158	13500	12207	16461	12117	21198
Hnrnpd	3413	2322	2011	2920	1960	3831
Hnrnpdl	4459	2979	2626	3465	2772	5239
Hnrnpk	4140	2747	2432	3767	2595	4265
Hnrnpu	11399	8423	6688	10206	7150	11706
Hoxb5	20	7	6	8	11	14
Hoxb8	1	0	0	1	0	0
Hoxd3	9	1	0	3	4	4
Hoxd9	5	9	4	7	7	5
Hp	21	26	39	36	28	45
Hp1bp3	6712	4632	3949	6034	4157	6995
Hpcal4	2	3	2	5	3	0
Hprt	2733	1718	1535	2299	1382	2987
Hps3	695	484	359	570	394	770
Hps4	375	222	138	301	157	319
Hps6	248	138	101	194	104	223
Hs2st1	722	490	445	608	489	762
Hs3st1	192	107	82	118	108	178
Hs3st3b1	105	76	56	101	63	96
Hsd11b1	485	259	529	358	364	914
Hsd17b10	4243	2926	2417	3517	2496	4638
Hsd17b12	1158	855	734	1144	635	1289
Hsd17b7	521	790	449	761	384	904
Hsdl2	13807	9226	8676	10802	9948	13270
Hspa1l	332	186	178	282	262	204
Hspb11	142	121	75	117	85	177
Hspb3	310	189	193	339	128	325
Hspb6	32576	25916	20764	25983	27517	36265
Hspb9	2	0	0	0	0	2
Htatip2	654	358	434	463	439	682
Htr2a	53	37	36	47	48	35
Hypk	2163	1552	1511	1862	1380	2663
I830077J02Rik	11	6	11	14	8	7
Iars2	6894	4235	3797	5533	4120	6619
Ica1l	1	0	7	3	0	8
Ick	1364	927	774	1062	885	1481
Icmt	2223	1369	1201	2005	1256	2069
Icos	18	10	17	16	10	28

Icosl	599	370	281	498	273	615
Ict1	1434	1044	846	1220	867	1738
Id4	30	13	16	17	17	34
Idh3a	38268	26991	24742	36392	24391	38803
Idh3g	19555	13395	10693	16481	11500	20062
Ier5	1895	946	748	1686	731	1561
Ifi27l2a	397	227	183	355	90	581
Ifnab	1	0	0	0	0	1
Ift43	654	486	387	518	449	794
Ift80	488	291	246	425	258	460
Igf2	1347	1003	841	1276	689	1675
Igf2r	8784	5040	4561	6710	6411	6282
Igfbp2	3	0	1	2	3	0
Igfbp4	3009	2047	2126	2876	1509	4364
Igfbp6	330	236	214	386	183	351
Igflr1	160	74	70	112	60	174
Igsf11	27	21	29	33	24	28
Ikbip	923	696	521	823	564	1033
Ikbkb	1431	882	656	1129	760	1420
Ikzf5	1318	835	701	1060	775	1261
Il10rb	6384	4498	3745	5472	4243	6226
Il11	7	3	2	2	4	6
Il11ra1	1663	971	826	1336	803	1666
Il12a	13	6	5	9	2	14
Il13ra2	2	0	3	0	0	5
Il17b	1	0	0	1	0	0
Il17d	136	72	55	91	53	135
Il18	10	3	9	5	7	16
Il18rap	24	17	8	20	2	32
Il1bos	0	0	1	0	0	1
Il23r	4	0	0	3	0	2
Il2ra	217	133	114	184	114	208
Il6	2	1	1	1	0	3
Ilf2	1369	919	763	1248	678	1491
Imp3	886	484	430	690	429	842
Imp4	519	393	301	480	329	509
Impdh2	326	178	152	277	155	300
Impg2	12	6	8	9	8	10
Inca1	201	138	141	164	134	237
Incenp	222	123	103	180	88	258
Ing1	945	665	513	846	551	1006
Ing4	1090	591	516	821	535	1058
Inhba	29	18	27	39	15	29
Inip	556	409	388	604	347	620
Ino80c	1716	1244	1144	1646	939	2226

Ino80d	2351	1414	1192	1692	1352	2377
Ino80e	470	297	218	364	245	467
Inpp4a	1263	758	670	969	775	1149
Inpp5e	1373	985	746	1203	862	1337
Insig1	664	395	384	423	403	729
Ints1	2085	1193	843	1733	975	1675
Ints12	585	376	358	482	356	600
Ints3	1658	964	746	1400	896	1227
Iqce	272	195	134	202	180	299
Iqgap3	54	28	15	23	11	92
Iqsec2	1658	1124	770	1530	871	1591
Irak1	4718	2950	2583	3863	2647	4760
Irak3	397	311	236	415	226	461
Irf6	57	62	36	55	38	75
Irf9	1186	652	446	865	475	1244
Irx1	281	180	124	265	157	234
Irx4	1205	896	743	1096	909	1138
Isg15	124	81	47	105	35	166
Ism2	2	0	1	0	0	4
Isy1	649	387	362	560	380	642
Itch	2526	1643	1347	2259	1396	2373
Itga10	108	67	57	106	64	98
Itga5	2490	1751	1320	2378	1417	2566
Itgae	22	12	2	21	4	17
Itgb2l	1	0	0	1	0	0
Itgb4	148	119	83	125	104	164
Itih2	32	30	15	54	10	20
Itih3	12	6	2	8	6	6
Itm2c	2638	1575	1197	2317	1063	2539
Iws1	2269	1445	1290	1788	1322	2348
Jade3	209	128	111	169	122	219
Jak2	2742	1877	1571	2312	1776	2887
Jakmip2	1	0	0	0	0	1
Jam2	16241	11719	8758	14318	9545	16530
Jarid2	1508	1062	1011	1326	1082	1679
Jazf1	187	120	110	203	99	186
Jkamp	1075	746	628	933	618	1297
Jmjd4	917	661	542	951	506	869
Jph1	1442	954	911	1138	1070	1336
Kat2b	6762	4325	3869	5775	4071	6365
Kat6b	1887	1307	1071	1447	1215	1940
Kat7	2101	1391	1104	1883	1163	1926
Katnb1	338	257	181	303	197	380
Kbtbd11	151	106	73	154	75	128
Kbtbd4	930	540	499	778	523	842

Kcnab3	15	18	9	14	13	17
Kcnd1	11	13	0	6	3	16
Kcne1l	11	7	4	11	3	9
Kcnf1	1	0	2	0	2	2
Kcng3	0	1	0	1	0	0
Kcnh2	4065	2571	1693	3557	2165	3383
Kcnip1	62	42	20	55	20	68
Kcnj12	1888	1223	1048	1911	1126	1640
Kcnj5	4340	3090	2451	4816	2680	3571
Kcnk1	9	4	8	6	8	10
Kcnk4	2	0	0	0	0	2
Kcnk5	23	18	12	18	16	28
Kcnq4	131	56	38	58	74	93
Kcp	122	65	44	79	43	123
Kctd13	209	179	123	174	163	250
Kctd6	561	357	367	478	328	641
Kctd9	4100	2701	2664	3327	2740	4229
Kdelr1	4430	3092	2316	4023	2408	4472
Kdm4c	1080	707	596	909	618	1061
Kdm5b	1012	629	543	867	565	1054
Kdm6b	2352	1533	1094	1898	1359	2084
Kera	1	1	1	2	1	0
Khdc3	0	1	1	1	0	1
Khsrp	2026	1430	948	1669	1131	1968
Kif11	109	82	53	73	32	197
Kif13b	1175	780	476	838	642	1096
Kif18b	19	12	11	11	2	38
Kif21b	99	68	54	77	76	98
Kifc2	520	314	208	321	283	486
Kifc5b	25	13	11	12	6	36
Klb	1	0	1	3	0	0
Klf1	3	2	2	1	0	6
Klf12	1115	769	438	874	626	1058
Klhdc7a	119	46	123	50	148	112
Klhl11	352	202	155	242	160	367
Klhl15	92	72	41	86	44	108
Klhl23	3263	2232	1828	3317	1712	3092
Klhl25	227	120	109	202	94	223
Klhl26	556	306	253	437	264	552
Klhl28	436	197	204	345	208	391
Klhl32	131	56	59	102	60	119
Klhl33	426	246	164	248	240	438
Klhl38	1547	989	917	988	1178	1734
Klk8	195	119	86	148	96	204
Klra1	2	0	0	0	0	3

Klra15	3	0	0	1	1	2
Klra17	2	0	0	1	0	1
Klra2	22	12	6	24	11	9
Klra22	3	0	0	1	0	2
Klra33	2	0	0	0	0	2
Klra4	2	0	0	0	0	2
Klrb1b	19	4	3	18	2	8
Klrc1	3	3	0	3	2	1
Klri2	2	2	1	3	1	2
Kmt2b	1872	1238	827	1517	1010	1864
Kmt2c	4743	3236	2556	3806	3008	4634
Kng2	1	0	0	0	0	1
Krba1	1438	921	678	1001	843	1514
Krcc1	2766	2187	2047	2526	2008	3208
Kri1	733	539	451	632	433	850
Krt10	61	44	26	52	30	61
Krt14	0	0	2	1	1	0
Krt19	10	4	2	7	7	3
Krt79	4	0	0	1	1	2
L3hypdh	262	209	174	265	148	324
Lactbl1	3	0	0	0	0	3
Lag3	22	13	16	19	12	24
Lage3	422	251	206	318	215	454
Lair1	64	72	67	85	50	119
Lalba	0	1	0	0	0	1
Lamp1	23578	16651	14475	21120	15005	23327
Lamp3	0	0	1	0	0	1
Lancl1	1143	670	581	908	535	1225
Lancl3	149	108	79	160	66	167
Laptm4a	17346	11062	9523	15212	9684	18267
Larp6	86	50	51	73	42	92
Lcmt2	478	254	236	366	259	392
Lcn5	0	1	0	1	0	0
Ldhal6b	8	10	8	9	6	13
Ldhb	45551	28243	25805	38621	25998	47469
Ldoc1	7	4	0	5	2	4
Lect1	17	12	41	9	23	54
Lefty1	39	23	21	37	19	32
Lemd2	1121	602	538	854	571	1091
Lep	1	1	0	2	0	0
Lgals2	0	1	2	3	0	0
Lgals8	2227	1612	1343	1841	1544	2440
Lgmn	4100	2733	2088	3478	2062	4284
Lilra5	103	51	64	71	43	147
Lime1	804	358	335	570	335	716

Lincrna-cox2	1	0	0	0	0	1
Lingo1	68	35	14	49	23	44
Lingo4	1	1	0	0	0	2
Lipc	3	3	0	2	3	0
Lipt2	183	96	91	122	97	171
Llph	132	77	64	94	51	155
Lmbr1	1144	710	636	937	656	1147
Lmbrd1	2985	2019	1878	2692	1772	3193
Lmna	3742	2441	1917	3513	1950	3655
Lmo4	1037	714	618	911	605	1214
Lmod2	37156	30767	26015	34735	24947	45463
Lmx1a	0	1	1	1	0	2
Lmx1b	0	0	1	0	1	0
LOC100503280	1	0	2	0	1	2
LOC101056043	402	241	186	308	217	359
LOC102308570	416	305	239	337	246	526
LOC102636514	1666	1139	1076	1307	1107	1870
Loh12cr1	445	187	193	324	177	382
Loxl1	744	493	489	732	321	1022
Loxl4	38	42	33	41	35	44
Lpar2	10	4	5	7	4	11
Lpin1	10525	7221	6627	11676	7024	8474
Lpin3	282	188	206	233	139	470
Lpl	335556	217518	203355	243847	221363	348967
Lpp	2914	2015	1960	2698	1816	3104
Lrfn2	18	8	5	16	5	16
Lrfn3	32	22	30	36	21	47
Lrg1	1446	869	757	1221	744	1508
Lrif1	1338	1013	955	1030	909	1781
Lrig1	2400	1717	1285	2279	1296	2343
Lrig2	897	582	487	731	531	903
Lrmp	95	43	51	70	49	102
Lrp11	169	127	147	162	123	223
Lrp2bp	103	70	59	82	80	97
Lrpap1	2551	1807	1478	2237	1456	2788
Lrrc10b	19	11	7	11	9	24
Lrrc16a	326	210	93	284	129	255
Lrrc16b	186	126	73	116	129	155
Lrrc28	394	239	225	350	218	406
Lrrc38	1	0	1	3	0	0
Lrrc40	614	397	354	536	350	661
Lrrc41	1059	683	552	1029	509	957
Lrrc45	427	282	217	289	268	487
Lrrc46	11	10	8	9	13	12
Lrrc47	1400	981	750	1263	841	1404

Lrrc48	258	157	146	201	149	268
Lrrc66	0	1	0	0	1	0
Lrrc69	20	9	8	9	9	20
Lrrc71	30	23	21	27	21	32
Lrrc75a	207	143	106	213	113	153
Lrrc8b	140	110	74	106	100	169
Lrrc8d	366	299	231	349	226	484
Lrrc8e	12	7	12	4	18	6
Lrrfip1	3671	2978	2357	3290	2375	4591
Lrrn4	7	1	10	8	11	2
Lrrn4cl	203	157	174	253	116	266
Lrtm2	6	10	9	8	5	15
Lsamp	51	37	14	44	21	52
Lsm12	662	481	482	605	485	683
Lsm2	271	179	144	253	145	272
Lsm4	1019	650	473	820	539	1045
Lsp1	1641	1389	791	1711	891	1820
Luc7l	2794	1958	1341	2056	1532	3066
Luc7l2	11373	8105	6158	8068	7351	13781
Ly6h	0	1	0	0	1	0
Ly75	65	28	28	50	35	48
Lypd6	10	11	10	6	11	15
Lypd6b	5	2	2	4	4	2
Lypla2	970	584	481	771	482	995
Lyplal1	98	28	80	44	68	132
Lyrm5	3077	2066	2160	2637	2122	3739
Lyst	730	514	360	685	410	655
Lyz1	15	20	9	18	13	22
Lzts2	2255	1337	1093	1974	1247	1930
Mab21l3	4	0	1	2	1	2
Macf1	17865	13264	11038	15688	11943	18289
Macrod1	5671	3285	2690	4357	2903	5524
Mad2l1bp	606	362	285	510	300	541
Maea	2945	1748	1586	2565	1653	2828
Mageb3	0	0	1	0	0	1
Magi3	2915	2218	1855	2310	2176	3078
Magoh	687	526	465	566	466	820
Magohb	187	137	123	160	118	247
Malat1	130832	88601	89157	71927	106945	152019
Malt1	873	737	547	688	597	1131
Mamdc2	225	161	86	207	82	225
Maml3	209	161	92	223	106	193
Man1a2	2156	1346	1120	1860	1173	1977
Man2a1	2512	1621	1470	2283	1264	3024
Man2b1	1946	1199	940	1584	1060	1963

Man2c1os	5	9	5	4	6	10
Manbal	395	249	208	354	189	407
Maneal	18	13	13	24	11	18
Manr	4	4	2	4	6	1
Maob	3011	1993	1848	2691	2062	2945
Map1a	520	400	426	637	365	565
Map2k3	3062	1729	1508	2488	1583	2737
Map3k10	859	447	384	660	395	741
Map3k14	231	129	104	168	158	184
Map3k8	213	141	110	183	91	246
Map3k9	14	13	8	11	7	19
Map4	20215	14567	11677	18477	12242	19644
Map4k5	2245	1622	1363	1974	1483	2470
Map7	1832	1136	835	1436	1080	1611
Map9	202	118	101	137	108	216
Mapk13	0	0	1	1	0	0
Mapk15	6	3	4	3	3	7
Mapk1ip1	1532	995	729	966	948	1697
11-Mar	0	3	0	1	0	3
Marcksl1	128	92	77	134	68	119
Mark2	2125	1419	1126	1940	1246	2084
Mars	2023	1104	1004	1668	1027	1805
Marveld2	40	31	13	29	25	33
Mast4	2763	2185	1567	2817	1584	2915
Mau2	2363	1464	1216	1931	1366	2352
Mb21d1	93	57	54	65	52	105
Mb21d2	555	320	275	478	305	485
Mbd4	168	105	62	122	86	168
Mbd5	836	603	510	829	604	751
Mbd6	1314	833	550	847	787	1353
Mbnl1	12728	8840	7262	10732	8147	13378
Mbnl3	78	37	52	69	25	94
Mc2r	2	1	0	1	2	0
Mc5r	1	0	0	1	0	0
Mccc2	3410	2483	2014	2878	2099	3713
Mcee	2327	1706	1719	2076	1558	2887
Mcemp1	0	0	3	2	0	1
Mcl1	12786	8725	7956	10393	7860	14450
Mcm10	32	11	6	13	5	43
Mcm4	558	339	262	430	290	576
Mcm9	145	100	90	119	89	175
Mcmdc2	3	10	5	4	5	10
Mcpt4	12	14	16	21	8	23
Mctp1	115	66	36	72	57	100
Mcts2	335	221	217	282	201	384

Mdc1	1149	884	575	953	730	1142
Mdfi	24	18	11	15	18	21
Mdga2	60	25	20	46	23	38
Mdh1	98982	60526	53055	86362	51975	100984
Mdm1	299	207	185	222	212	362
Me1	4583	2515	2559	3256	3021	3993
Me2	405	278	240	312	259	445
Meaf6	541	367	266	387	311	562
Mecp2	4152	2812	2173	3441	2482	3951
Med11	166	100	91	130	92	188
Med12	2315	1531	1019	1796	1326	2289
Med12l	521	323	244	427	281	460
Med15	1142	855	587	1057	645	1171
Med17	741	529	395	620	440	771
Med19	725	488	443	650	390	820
Med20	665	407	379	568	359	670
Med23	1080	663	546	895	621	1038
Med4	974	609	590	796	585	1077
Med6	397	258	225	319	210	497
Med8	737	589	475	638	463	913
Meig1	35	22	23	15	25	49
Memo1	1763	1099	1199	1596	1155	1942
Mettl20	1114	652	739	733	686	1338
Mettl21a	383	237	217	332	203	391
Mettl7a1	2070	1397	1045	1785	1273	2008
Mettl7a2Higd1c	1	0	0	1	0	0
Mettl7b	0	1	0	1	0	0
Mex3a	19	16	18	25	11	28
Mex3b	125	56	53	95	51	122
Mex3d	310	184	146	264	159	289
Mfap1b	116	37	69	105	42	114
Mfap3	1064	762	681	1063	587	1264
Mfn2	41302	25532	21603	36250	23669	33181
Mfsd12	106	70	58	103	64	74
Mfsd2b	112	65	60	60	69	124
Mfsd3	150	80	57	115	58	134
Mfsd5	522	336	250	472	280	501
Mfsd6	1658	1158	1088	1773	965	1712
Mfsd7b	451	254	219	330	230	481
Mgat4b	2786	1691	1392	2452	1457	2750
Mgp	5312	3982	2743	6487	2215	4609
Mgst3	6680	4173	3484	5635	3717	6650
Mink1	2489	1660	1288	1984	1526	2371
Minos1	6264	4685	3795	5419	3669	7427
Mios	1188	815	780	1081	670	1405

Mipep	3792	2249	1969	3324	1981	3447
Mipol1	103	94	73	114	86	117
Mir1191b	1	0	0	0	0	1
Mir125a	3	0	0	1	1	2
Mir140	1	0	0	1	0	0
Mir155	0	0	1	0	0	1
Mir17hg	161	87	90	94	95	180
Mir1892	320	161	140	240	135	330
Mir1901	2	0	0	1	0	1
Mir1906-1	9	9	6	2	6	17
Mir1906-2	9	9	6	2	6	17
Mir1947	1	0	0	0	0	1
Mir221	1	0	0	0	0	1
Mir24-1	17	3	7	11	9	12
Mir27a	0	0	1	0	0	1
Mir29b-2	27	5	4	11	6	25
Mir3058	6	1	1	3	2	5
Mir3072	0	0	1	1	0	0
Mir3082	476	406	276	421	322	521
Mir30b	1	0	0	0	0	1
Mir30c-2	2	0	0	0	0	2
Mir3103	6	5	4	8	5	5
Mir3112	5	4	5	6	1	10
Mir324	0	1	0	0	0	1
Mir331	5	1	0	1	2	3
Mir343	19	12	13	18	15	17
Mir345	0	2	0	0	1	2
Mir3471-1	0	1	0	0	0	1
Mir410	2	0	0	0	1	1
Mir425	0	2	2	2	0	4
Mir452	0	1	0	0	0	1
Mir5134	1	0	0	0	0	1
Mir5710	0	3	1	1	0	3
Mir6369	4	1	3	4	2	4
Mir6387	1	1	1	2	0	1
Mir668	1	0	0	0	0	1
Mir674	2	0	0	0	1	1
Mir677	20	5	14	10	10	20
Mir680-2	3	5	1	7	0	4
Mir6907	1	1	2	1	2	2
Mir6919	1	0	0	0	0	1
Mir6937	2	1	0	3	1	0
Mir6938	0	1	1	1	1	0
Mir6955	8	2	6	4	8	7
Mir6958	10	9	3	7	6	9

Mir6966	0	1	1	0	0	2
Mir6985	0	1	0	0	0	1
Mir6986	1	0	0	1	0	0
Mir6991	4	1	2	2	1	4
Mir6997	3	3	4	3	3	4
Mir7000	1	0	1	3	0	0
Mir7001	1	1	0	1	1	0
Mir7013	3	7	7	7	7	5
Mir7014	8	0	1	2	1	7
Mir7015	0	0	1	1	0	0
Mir7016	1	0	0	0	0	1
Mir7027	3	1	3	4	1	4
Mir7030	3	3	1	0	4	4
Mir7037	3	0	0	1	1	1
Mir7048	3	0	0	1	1	1
Mir7052	44	24	36	34	26	58
Mir7058	0	1	0	1	0	0
Mir7059	3	0	0	2	1	1
Mir7064	1	0	0	0	0	1
Mir7069	6	5	2	5	2	8
Mir7082	24	13	8	21	12	17
Mir7084	3	2	5	6	4	3
Mir7087	3	1	2	3	3	1
Mir7236	0	0	1	0	0	1
Mir761	10	6	5	7	9	8
Mir7647	0	1	1	0	1	1
Mir7654	32	21	21	28	17	44
Mir7657	28	25	20	21	24	44
Mir7667	1	1	1	0	1	2
Mir7670	29	17	3	14	13	23
Mir7673	5	7	3	9	5	4
Mir7676-1	0	3	3	1	4	1
Mir7676-2	0	3	3	1	4	1
Mir8097	60	24	19	46	16	55
Mir8107	7	4	1	6	2	7
Mir8108	44	30	30	48	25	40
Mir8119	1	0	0	0	0	1
Mirg	11	7	6	10	5	10
Mirlet7a-1	1	1	0	1	1	0
Mis12	541	359	361	525	312	619
Mknk1	1170	759	576	885	649	1203
Mknk2	9387	5049	4306	6963	5261	7726
Mkrn2	1831	1178	1051	1544	1129	1854
Mlip	3770	2684	2462	3448	2322	4479
Mllt10	1823	1269	826	1418	1173	1708

Mllt6	5801	3520	2571	4746	3259	5085
Mlst8	566	334	248	448	302	485
Mlycd	3404	1874	1702	2425	1845	3310
Mmp13	32	12	8	16	9	28
Mmp25	14	2	5	8	2	12
Mmp3	120	52	119	118	67	176
Mmrn1	512	537	280	540	388	482
Mms19	1181	762	466	946	604	1129
Mms22l	47	21	19	26	20	55
Mnat1	659	466	365	524	448	694
Mnda	97	76	68	92	38	165
Mog	1	0	0	1	0	0
Mogat1	0	1	1	3	0	0
Mon1a	445	299	201	386	240	450
Mon1b	565	390	320	470	352	590
Mos	3	1	0	2	1	2
Mpc2	9963	5410	5749	8281	4528	12053
Mphosph6	230	164	170	193	160	273
Mpl	11	16	3	7	13	8
Mplkip	220	134	133	146	126	275
Mpp5	2783	2006	1676	2207	1840	3130
Mppe1	717	461	459	656	406	757
Mr1	1021	865	628	1079	664	1120
Mrc2	1116	756	562	1112	448	1185
Mrgprb1	5	4	1	2	2	8
Mroh2a	1	2	1	0	1	3
Mroh5	0	0	1	0	1	0
Mroh8	7	1	1	3	3	4
Mrpl11	1608	1033	1097	1440	977	1873
Mrpl12	4791	2827	2688	4029	2369	5066
Mrpl13	2367	1669	1728	2157	1447	2880
Mrpl20	1189	610	654	910	576	1299
Mrpl23	10	6	5	8	5	11
Mrpl24	2275	1588	1392	1841	1447	2696
Mrpl30	4634	2952	3105	3912	2632	5610
Mrpl40	1024	731	627	896	623	1155
Mrpl53	1929	1144	1076	1596	1020	2085
Mrpl54	878	550	598	786	493	1065
Mrps14	1575	1019	1001	1345	900	1967
Mrps16	1011	614	665	985	488	1265
Mrps21	1228	1073	1121	1266	974	1680
Mrps24	1431	780	820	1181	728	1571
Mrps30	1940	1299	1141	1618	1156	2235
Mrps31	2119	1479	1314	1913	1260	2241
Mrps33	345	266	248	311	256	431

Mrps34	1613	1063	1083	1310	1038	2045
Mrps35	3229	2474	2118	2753	2165	3884
Ms4a4d	751	459	439	752	267	852
Ms4a6b	226	140	139	213	89	277
Msh2	1238	918	683	1017	786	1292
Msh5	14	5	8	3	12	17
Msh6	660	440	375	528	399	710
Msmp	5	1	3	1	2	9
Msrb2	2441	1650	1435	2062	1420	2700
Mst1r	171	81	51	147	42	123
Msx1os	1	0	0	0	0	1
Msx2	21	10	17	16	14	28
Mt2	888	548	417	555	558	864
Mtch2	6378	4016	3892	5479	3535	6765
Mtcp1	519	310	286	396	272	579
Mterf1a	43	38	29	34	33	50
Mtfr1l	6669	4174	3636	5604	3683	6479
Mthfr	838	455	468	571	388	1002
Mthfs	36	25	26	39	20	43
Mtif3	783	622	491	699	558	907
Mtmr1	1948	1309	1125	1643	1186	2009
Mtmr3	3912	2464	2125	3418	2480	3578
Mtor	3614	2461	2032	3252	2216	3221
Mtpn	3383	2223	2037	3059	1837	3656
Mttp	546	290	298	385	371	492
Mturn	1930	1140	891	1600	974	1710
Mtus1	11521	8579	7386	10193	7898	12420
Muc13	7	9	4	8	5	11
Mucl1	7	4	1	0	3	11
Mus81	755	390	312	619	386	583
Musk	4	6	3	5	2	10
Mx1	42	22	21	34	12	59
Mx2	129	61	63	83	52	136
Mxd1	567	348	271	465	303	563
Mxra7	649	471	386	660	291	779
Mxra8	1299	920	625	1294	574	1332
Myadml2	2496	1455	1383	2225	1160	2488
Mybpc1	9	4	3	5	3	11
Mybpc2	1588	606	647	917	838	1153
Mybpc3	181796	127651	99830	169425	99755	175625
Mycn	109	60	63	101	53	111
Myeov2	1183	649	723	1025	590	1387
Myg1	536	346	289	505	255	581
Myh14	6947	4897	2836	5397	4132	5650
Myh3	0	0	2	0	0	2

Myh6	1265572	947089	585984	1099897	772149	1034846
Myl12a	21368	12892	13556	16041	12541	25305
Myl6	1683	1297	1213	1460	1152	2333
Mylk3	33972	22658	15867	26921	20256	28493
Myo18a	8822	6655	4841	8013	5941	7815
Myo1a	6	5	4	2	7	6
Myo1c	7918	5113	4551	7695	4214	8363
Myo1f	170	96	64	125	54	180
Myo1g	104	46	33	88	24	107
Myo5c	600	399	363	380	352	860
Myo9a	1996	1323	1041	1756	1188	1964
Myo9b	2317	1479	1070	1775	1314	2108
Myof	480	312	275	392	232	657
Myoz2	42464	31124	25902	36909	27623	45319
Myzap	23515	16492	15301	21029	14143	26024
Mzf1	14	9	5	10	4	20
N4bp1	1690	1110	950	1509	1041	1656
N6amt2	763	484	468	644	435	899
Naa10	1117	844	601	968	627	1272
Naa38	674	457	399	587	439	719
Nab1	1290	891	1069	1598	675	1466
Nacc1	1907	1231	959	1798	962	1695
Nadk	1662	1012	877	1392	832	1703
Nadk2	3717	2529	2074	2874	2621	3334
Nagk	681	473	347	530	434	710
Nagpa	473	310	240	421	268	457
Naif1	77	76	46	80	50	90
Nalcn	52	29	40	40	30	61
Nampt	16463	9752	7771	11885	11539	12718
Napa	3492	2145	1778	2895	1869	3627
Napsa	19	4	3	14	5	10
Nat2	87	61	66	61	51	132
Nat6	891	574	429	735	496	891
Nav2	2873	2150	1464	2661	1695	2688
Nbeal1	2010	1307	981	1820	1125	1827
Nbr1	8492	5715	4637	7560	4843	8182
Ncapd2	1058	736	521	890	575	1090
Ncapd3	1295	828	588	935	803	1265
Ncf4	41	19	25	27	21	53
Nck1	1845	1199	967	1705	946	1929
Nckap1	6533	4411	3638	5955	3725	6240
Ncoa2	3810	2556	1991	3440	2410	3298
Ncr1	10	5	1	4	4	8
Ndel1	3024	1983	1722	2579	1774	3066
Ndnl2	527	366	337	472	285	617

Ndp	1	1	3	1	1	3
Ndrg2	54787	37239	29216	49184	33031	56645
Ndrg3	1078	732	600	1002	579	1155
Ndufa10	24455	17420	15861	22177	15273	27047
Ndufa2	3142	1780	1943	2815	1479	3860
Ndufa3	4057	2578	3084	3737	2329	5164
Ndufa6	6930	4729	5081	5963	4079	9215
Ndufab1	853	501	548	776	465	890
Ndufaf3	751	435	409	633	324	829
Ndufaf7	1311	845	771	1158	694	1449
Ndufb10	17667	12898	12139	16526	11307	21388
Ndufb5	13362	7554	8247	11490	6857	15960
Ndufb6	4721	2479	2774	4065	2178	5470
Ndufc1	4592	2688	3372	4165	2296	5878
Ndufs2	42376	25284	23017	35004	23100	44493
Ndufs4	8569	5688	5417	7206	4757	10551
Ndufs8	7253	4829	4469	6288	4033	8458
Ndufv2	22781	15324	14508	19793	13320	25798
Ndufv3	6580	4426	4707	6073	3761	8797
Neat1	60035	44765	36269	27092	41540	100650
Nedd4	33268	23155	18748	29063	22199	29206
Nefh	1	0	0	0	0	1
Nefm	18	18	5	8	13	20
Neil1	191	92	88	141	95	163
Neil2	93	55	53	64	65	100
Nek11	0	0	1	1	0	0
Nek3	132	93	83	103	83	148
Nek5	4	0	1	1	1	4
Nek6	159	87	76	127	73	171
Nelfa	575	398	324	514	326	599
Nemf	2085	1641	1277	1759	1362	2459
Neurl1a	61	40	40	56	35	75
Nexn	45036	38834	32500	42281	34721	56901
Nf2	6648	4545	3689	6871	3896	5476
Nfatc2	1051	750	470	766	668	958
Nfatc3	2128	1398	1198	1998	1248	1862
Nfib	17341	12396	10261	13473	11049	21929
Nfix	9005	6487	4573	7979	5415	7991
Nfs1	4094	2646	2278	3501	2314	4125
Nfu1	1677	1198	1097	1436	982	2037
Nfyb	761	507	453	724	437	825
Ngb	4	2	0	3	3	1
Ngdn	930	570	500	698	526	984
Ngef	34	15	9	36	11	20
Ngrn	950	533	531	764	501	1008

Nhej1	124	79	54	79	63	156
Nim1k	236	168	152	178	159	279
Ninl	74	70	53	83	61	88
Nipal2	35	35	28	36	23	60
Nipal4	1	3	1	0	3	2
Nipbl	5016	3470	2637	4172	2910	5207
Nisch	8971	6077	4519	8171	5230	8473
Nit2	934	483	478	750	443	941
Nkd2	62	44	43	70	35	57
Nkg7	3	7	3	7	3	3
Nkiras2	1576	978	874	1398	906	1408
Nkx2-3	0	2	0	2	0	0
Nkx3-1	2	0	0	0	1	1
Nkx6-2	5	1	3	3	5	3
Nlgn1	1	0	0	0	0	1
Nlk	1084	660	652	899	651	1062
Nlrp1c-ps	4	12	3	8	2	16
Nlrx1	725	387	366	586	367	626
Nme4	143	81	58	98	55	151
Nme5	111	81	50	88	71	114
Nmrk1	177	112	100	130	101	220
Nmt1	3888	2872	2366	3428	2295	4571
Nmt2	1033	734	662	957	583	1183
Nol6	988	634	480	816	543	906
Nom1	1017	744	620	898	688	1129
Nop9	531	324	317	446	276	566
Noxo1	39	22	17	25	20	46
Npb	8	5	1	4	5	8
Npc2	2155	1430	1228	1900	1139	2492
Npepps	6904	4225	3885	5424	4297	6490
Nphp1	575	390	379	514	317	700
Nphp3	512	274	292	325	314	606
Nploc4	3704	2350	1928	3150	2190	3176
Nr1h3	510	283	244	372	309	470
Nr1i3	2	1	2	0	4	0
Nr2c2	2460	1620	1166	2018	1330	2375
Nr2c2ap	151	69	65	108	75	140
Nr2f6	1034	490	548	879	475	963
Nrk	49	30	27	51	13	53
Nrtn	394	175	107	301	112	335
Nrxn3	4	1	1	0	2	4
Nsa2	834	574	579	801	453	1138
Nsd1	4910	3396	2891	4137	3336	5026
Nsg1	197	122	111	179	86	250
Nsmaf	3894	2331	2103	2877	2316	3746

Nsun4	1796	1057	1029	1596	1017	1725
Nt5c2	2081	1396	1242	1931	1210	2135
Nt5c3b	974	614	485	809	529	942
Nt5dc3	3891	2581	2279	4031	2435	3198
Ntf5	23	12	12	17	3	39
Ntn5	5	2	2	3	3	5
Ntng2	281	154	176	186	170	309
Ntrk3	32	19	20	23	18	38
Nub1	2859	2174	1541	2372	1864	3162
Nucb1	3906	2674	2148	3328	2313	3895
Nudcd3	3999	2538	2087	3641	2101	3777
Nudt10	48	24	39	39	31	53
Nudt2	644	407	405	567	370	734
Nudt5	238	144	138	203	127	248
Nudt6	780	436	372	548	453	742
Nudt7	2793	1679	1726	2002	1717	3345
Nudt8	1890	1235	1136	1561	1157	2015
Nufip2	4430	2704	2436	3765	2414	4362
Numb	1772	1224	891	1425	1068	1835
Nup133	976	543	464	806	510	820
Nup155	955	694	474	810	556	1010
Nup214	1108	782	517	1032	671	952
Nup85	886	473	416	710	404	834
Nup88	2579	1841	1497	2242	1548	2839
Nup93	800	528	492	686	453	884
Nusap1	60	69	26	43	24	136
Nxf1	3397	2115	1753	2750	1941	3454
Nxf3	1	0	0	1	0	0
Nxnl1	7	2	0	2	2	6
Nxpe5	15	4	3	6	2	15
Nxt1	157	84	74	117	77	169
Ocrl	1175	830	636	1032	771	1169
Odf2	1854	1266	865	1413	1135	1906
Ofd1	596	437	316	407	424	689
Ogdh	119298	81654	65441	106084	79257	95456
Oip5	17	15	10	12	16	22
Olfr56	8	5	2	5	3	8
Olfr920	9	18	21	15	15	34
Omp	0	3	0	3	0	0
Onecut2	0	3	2	1	0	6
Ophn1	671	481	399	483	422	900
Opn1mw	1	1	1	0	2	2
Opn3	96	67	47	92	43	116
Opn4	162	106	57	152	71	141
Oprk1	0	1	1	2	0	1

Orc5	579	417	332	523	341	659
Ormdl3	1946	1092	1115	1749	984	1977
Osbp	7088	5112	3931	6181	4556	6647
Osbp2	1438	939	784	1151	887	1393
Osbpl7	289	170	148	185	175	302
Osbpl8	7100	5035	4266	6239	4730	6777
Osbpl9	4934	3664	3330	4383	3120	5945
Osgin1	222	115	102	184	123	183
Osr1	59	44	53	60	34	87
Osr2	3	2	2	1	3	5
Ostc	1325	782	770	1167	696	1342
Ostn	0	2	0	0	0	2
Otub1	2222	1439	1108	1882	1160	2445
Otud1	936	638	464	839	522	932
Otud3	344	272	228	320	237	374
Otud4	6389	3863	3323	4909	3476	6257
Otulin	903	599	654	816	574	1154
Ovca2	574	384	327	523	335	549
Ovol3	0	2	1	1	1	1
Oxa1l	3668	2429	2033	3255	2204	3704
Oxct2a	1	0	0	0	0	1
Oxld1	705	444	409	544	358	850
Oxr1	4779	3281	2918	4331	2949	4702
P2ry6	124	87	61	99	58	166
P4htm	642	394	323	604	295	599
Paf1	2679	1986	1580	2298	1620	3032
Paip2	8762	5886	5233	7444	4916	9611
Pak1	102	59	49	93	39	112
Pak1ip1	1265	875	834	1142	733	1466
Pam	48052	34178	32092	49721	30829	45305
Pank2	1509	889	798	1206	822	1571
Pank3	2563	1450	1359	2169	1406	2367
Panx1	55	47	48	72	25	79
Papd4	494	303	226	376	233	506
Papolb	3	3	0	2	1	4
Pappa2	2	7	2	9	1	5
Paqr5	0	2	0	1	0	1
Park2	306	205	146	224	199	324
Parp16	228	143	145	177	146	265
Pate2	1	1	2	1	0	4
Pax5	0	5	1	4	1	3
Pbrm1	4109	2648	2219	3395	2391	3963
Pcbp1	5793	3728	3242	5353	3106	5691
Pccb	5490	3718	3240	4559	3197	6053
Pcdh10	3	3	1	2	3	2

Pcdhb11	66	13	23	26	27	46
Pcdhb14	142	80	65	106	69	133
Pcdhb17	283	154	124	206	130	264
Pcdhb20	181	113	77	122	112	147
Pcdhb22	113	46	52	87	55	97
Pcdhb9	25	10	6	18	9	14
Pcgf5	1847	1298	1264	1617	1271	2049
Pcgf6	175	130	138	178	121	227
Pcid2	555	329	290	431	329	538
Pcmt1	2828	1741	1612	2591	1633	2735
Pcmtd2	5292	3412	2686	3982	3072	5789
Pcnx	6226	4138	3255	5243	3870	5519
Pcp4	29	28	27	32	20	54
Pcsk1	7	7	5	9	9	4
Pcsk2	1	1	0	2	0	0
Pctp	228	181	165	222	131	294
Pcyox1l	94	45	40	78	40	72
Pcyt1b	12	15	10	10	10	20
Pdcd1lg2	1	1	2	1	0	4
Pdcd2	363	241	217	260	219	432
Pdcd5	669	351	355	507	311	785
Pdcd6ip	5032	3630	2878	4604	3033	5008
Pdcd7	587	392	374	505	382	643
Pddc1	401	204	173	319	172	403
Pde1a	484	373	263	501	247	511
Pde4b	1344	812	972	1035	895	1566
Pde5a	456	335	251	451	208	524
Pde6h	2	1	0	1	0	3
Pde7a	4602	2909	3391	3123	3073	6274
Pde9a	198	117	99	133	111	205
Pdgfa	685	455	303	562	372	615
Pdgfc	117	75	84	82	71	161
Pdhb	25223	16730	15317	21689	14821	26840
Pdk2	19331	14059	9638	16621	12026	17903
Pdlim2	202	154	111	191	97	231
Pdlim5	31465	22440	22722	31132	22472	34955
Pdp1	2542	1917	1331	2039	1643	2844
Pdzd11	691	525	393	638	397	851
Pdzd4	110	68	59	98	50	111
Pdzd8	2323	1650	1378	2116	1470	2257
Pdzk1	0	0	1	0	1	0
Pdzrn3	2035	1412	957	1761	1420	1505
Pebp1	3153	1927	1711	2720	1662	3412
Pemt	32	7	14	16	20	17
Per3	3899	2503	1646	2422	2257	3787

Pex13	1874	1194	1298	1564	1243	1979
Pex19	5741	3533	3155	4831	3200	6025
Pex26	440	277	216	380	249	351
Pex3	1004	740	617	876	618	1121
Pfdn1	1831	1360	1314	1681	1285	2288
Pfdn2	1136	909	762	1030	745	1403
Pfkm	46012	28644	23225	41292	26535	42114
Pgam1	231	189	171	345	160	179
Pgam2	18088	12026	11520	16056	9761	22782
Pgd	1242	887	664	1104	727	1321
Pgf	164	91	144	163	104	209
Pgp	542	271	317	522	244	517
Pgr	1	0	0	1	0	0
Pgrmc2	2863	1898	1689	2477	1592	3083
Phactr2	5286	3946	3249	4422	3602	6234
Phactr4	2226	1430	1000	1877	1229	1914
Phax	1042	822	650	860	663	1265
Phc3	3382	2099	1652	2546	1899	3234
Phf14	1895	1360	1174	1553	1248	2100
Phf20l1	4913	3168	2602	3845	2908	4844
Phf23	1089	696	567	977	605	1059
Phf8	1035	701	477	948	566	877
Phldb3	9	9	6	5	9	14
Phospho1	2817	1582	1344	2187	1604	2316
Phyh	32229	24479	22141	28092	22059	36551
Phyhip	937	664	510	892	579	878
Phyhipl	1	2	0	1	1	2
Phykpl	340	246	242	289	243	426
Pi15	158	108	112	175	80	201
Pi16	3599	2074	1853	3431	1671	3109
Pi4k2a	1264	793	673	1095	674	1307
Pi4k2b	216	172	135	171	131	306
Pias4	564	392	308	477	355	554
Picalm	13227	9226	7892	12730	7560	15013
Pick1	989	561	475	741	537	907
Pidd1	34	21	23	27	20	48
Pigc	561	353	329	514	274	670
Pigh	228	154	126	174	133	276
Pigp	2068	1284	1266	1633	1167	2426
Pigq	1846	1095	814	1526	868	1687
Pigt	1204	765	516	923	664	1050
Pigu	1177	1004	629	1096	777	1367
Pigv	508	240	221	386	230	449
Pik3ap1	65	54	23	75	20	59
Pik3ca	5011	3328	2940	4212	2939	5251

Pik3r4	1440	960	732	1350	818	1358
Pim2	135	70	48	99	52	133
Pip4k2c	1924	1200	1055	1740	1058	1904
Pip5k1a	708	515	394	693	454	669
Pira6	48	14	10	36	6	34
Pirt	368	208	111	252	246	197
Pithd1	784	455	396	631	387	778
Pitpnm2	4903	3286	2214	3817	2958	4685
Pitpnm2os1	0	1	0	0	0	1
Pkd2	3213	2094	1727	3025	1561	3638
Pkn1	2166	1297	1067	1698	1268	2092
Pknox1	570	324	324	513	270	552
Pkp3	4	4	3	8	1	5
Pkp4	9224	5907	4629	7320	5374	9577
Pla2g6	401	232	183	319	230	351
Pla2r1	349	222	217	275	200	451
Plaur	75	20	24	34	27	62
Plcb3	1373	851	583	1122	681	1248
Plcg2	593	447	337	594	330	666
Plch2	1	0	2	1	1	1
Plcz1	1	1	1	1	0	2
Pld4	241	156	108	205	74	295
Plek2	15	4	8	13	6	10
Plekha3	1554	1042	961	1370	881	1705
Plekha6	6520	5058	3192	5803	4104	6570
Plekhb1	170	79	63	91	78	161
Plekhf1	475	248	218	344	302	353
Plekhg6	1	0	0	0	0	1
Plekhm2	5732	3517	2554	4554	3095	4855
Plekhm3	671	375	348	489	402	654
Plekhn1	80	66	24	56	48	76
Plekho1	1952	1320	1297	1904	1247	2113
Plin1	7	18	8	26	6	4
Plk3	85	44	75	53	50	150
Plod1	4608	2817	2542	4338	2369	4714
Plod2	2127	1302	1169	1723	1271	2145
Plod3	1496	968	722	1341	760	1397
Plp1	426	250	197	297	218	428
Plrg1	1120	700	636	931	596	1207
Plscr1	479	282	258	391	254	486
Pmm2	1113	802	716	970	700	1282
Pms2	250	171	140	223	145	273
Pnisr	5353	3928	2959	3651	3362	6335
Pnliprp1	1	2	0	1	1	1
Pnmal2	418	195	157	374	140	367

Pnpla1	1	0	0	0	0	1
Pnpla7	1408	846	667	1169	715	1373
Pnpo	750	447	413	576	429	798
Pole2	53	38	26	40	36	61
Pole4	597	344	338	508	315	572
Polr1d	1074	652	664	993	553	1153
Polr2e	2099	1389	1111	1715	1289	2157
Polr2g	1117	591	599	962	526	1166
Polr2i	413	279	283	367	247	516
Polr3a	938	749	572	838	673	1037
Polr3f	1026	771	617	986	603	1095
Polr3h	239	139	110	178	139	234
Pop1	150	80	92	130	72	159
Pop4	403	302	260	332	298	476
Pop5	442	309	247	368	258	456
Pop7	150	112	91	153	68	194
Popdc2	19329	13321	11493	17828	11650	21781
Pou2f1	1313	851	686	873	939	1350
Pou3f2	0	1	0	1	0	0
Pou6f2	0	1	0	0	0	1
Ppa1	2037	1325	1270	1690	1237	2221
Ppapdc3	973	566	584	963	579	842
Ppfibp2	1491	1226	914	1281	971	1800
Ppig	3352	2715	2327	2909	2377	4500
Ppil2	1522	1112	803	1309	958	1569
Ppil3	389	297	268	356	253	504
Ppm1g	2182	1551	1289	1881	1376	2253
Ppm1k	7893	5709	3923	6280	5320	7206
Ppp1cb	27111	17666	16113	22306	16762	26514
Ppp1r12a	2482	1873	1452	2198	1645	2508
Ppp1r13b	1953	1269	883	1642	1093	1821
Ppp1r14b	250	226	187	276	162	350
Ppp1r15a	984	706	566	847	613	1027
Ppp1r17	0	1	0	0	1	0
Ppp1r36	4	2	2	6	3	1
Ppp1r37	3155	1762	1514	2455	1817	2562
Ppp1r3d	1162	727	546	1034	615	936
Ppp1r3fos	2	4	1	2	1	4
Ppp1r42	14	7	6	5	12	15
Ppp2r2b	1	2	1	2	1	1
Ppp2r3c	825	511	483	735	478	869
Ppp2r5b	1620	931	767	1400	844	1345
Ppp2r5c	6879	4699	3850	6102	4194	6440
Ppp4c	817	469	446	718	412	868
Ppp4r1	1772	1130	915	1555	1035	1713

Ppp4r2	1175	729	637	1007	657	1078
Ppp6r1	4922	2997	2385	4214	2541	4521
Ppp6r3	6237	4048	3331	5510	3595	5659
Pradc1	215	131	110	193	123	200
Praf2	166	99	86	130	80	201
Pramef12	0	1	0	0	0	1
Pramef8	709	409	364	547	390	645
Prdm2	3378	2266	1830	3134	1890	3451
Prdm5	353	259	216	299	204	423
Prelp	2278	1798	1245	2651	1165	2321
Prf1	7	0	0	2	0	5
Prim1	89	63	56	69	61	110
Prim2	95	72	55	79	60	124
Prkaa1	1539	1057	843	1325	937	1601
Prkacb	4322	3156	2588	4017	2710	4646
Prkcb	192	131	90	138	104	234
Prkcd	577	349	288	466	275	578
Prkcz	78	50	43	50	68	53
Prkdc	868	538	502	660	543	962
Prkg1	1683	1193	869	1549	950	1557
Prkra	580	435	357	487	393	695
Prkrip1	345	257	226	285	247	413
Prkx	1298	843	783	1117	798	1291
Prlr	423	198	264	269	307	337
Prmt6	269	176	155	240	154	295
Prodh	1301	780	581	1023	665	1275
Prpf18	2520	1532	1391	2062	1454	2466
Prpf4	1012	668	601	966	600	1051
Prpf6	2316	1607	1323	2041	1420	2443
Prph2	0	0	1	0	1	0
Prps2	876	565	542	737	633	836
Prr12	1462	1039	647	1281	822	1311
Prr14	1721	1194	869	1436	928	1815
Prr24	433	224	202	315	215	441
Prr3	133	102	70	115	74	146
Prr33	252	173	159	191	197	233
Prr7	10	5	3	4	4	11
Prrc2b	9749	6635	4900	9385	5539	7622
Prrc2c	5489	4255	2783	4494	3719	4954
Prrg1	816	487	418	529	505	902
Prrt2	276	215	151	212	204	318
Prss12	10	1	6	3	4	10
Prss36	256	142	126	191	154	230
Prss46	1	0	0	0	0	1
Prss53	83	46	33	57	42	69

Psap	47388	26550	23019	37909	26463	42322
Psma2	2635	1742	1647	2245	1592	3179
Psma6	4482	3107	2998	3966	2812	5560
Psma7	3226	2323	1906	2660	1867	4038
Psmb2	3068	2387	1858	2642	1981	3784
Psmc2	4421	2799	2557	3778	2571	4907
Psmc3	7897	5257	4311	6471	4697	8533
Psmd1	8046	5851	5000	7254	5248	8313
Psmd11	2520	1729	1500	2179	1636	2379
Psmd13	2730	1709	1546	2334	1468	3132
Psmd14	2334	1737	1506	2077	1504	2646
Psmd5	840	508	408	688	449	844
Psmd8	3374	2569	2056	2943	2185	3769
Psmg4	140	98	112	131	90	177
Pstpip2	598	448	409	512	575	481
Ptcd1	1294	741	682	1106	760	1129
Ptcd2	2723	1847	1879	2525	1743	2892
Ptchd3	10	12	12	15	10	16
Ptdss1	2568	1592	1734	2563	1484	2802
Ptdss2	850	470	455	768	429	821
Ptgdr2	1	2	1	2	2	1
Ptgfrn	3460	2111	1550	3271	1562	3282
Ptgir	308	213	118	317	127	287
Ptgis	418	256	276	443	165	500
Ptgs2	51	46	53	70	25	97
Pth1r	326	164	132	255	126	325
Ptk2b	545	471	356	723	261	650
Ptk7	142	99	59	119	80	140
Ptpmt1	752	454	486	605	401	891
Ptpn11	14665	9332	8574	13694	9224	11354
Ptpn13	337	241	169	309	200	287
Ptpn3	3007	1982	1639	2157	2161	2538
Ptpn7	35	21	16	22	15	43
Ptprd	965	722	549	813	624	1020
Ptprm	6984	4065	3091	5405	3717	5940
Ptprn2	4	6	1	4	3	6
Ptprv	22	10	8	8	13	27
Pttg1	2964	1902	1625	2449	1602	3396
Pus7l	130	66	47	87	66	116
Pusl1	169	101	111	150	87	183
Pvrl1	140	90	58	143	59	111
Pwwp2a	1053	739	522	703	648	1133
Pxt1	0	0	2	0	1	1
Pycr1	11	12	9	17	9	12
Pycr2	387	225	167	339	172	389

Pygm	56496	38960	28790	52553	32014	50365
R3hdm4	2564	1667	1076	2101	1297	2385
R74862	83	47	26	58	43	74
Rab11fip2	676	434	389	495	440	760
Rab11fip5	3747	2427	2197	3538	2147	3615
Rab12	9855	6685	6370	8542	6271	11272
Rab18	6073	3790	3677	5001	3747	5945
Rab19	15	11	15	21	5	21
Rab20	476	339	273	382	300	573
Rab26os	16	3	5	8	4	12
Rab2a	7170	4846	4350	6422	4263	7620
Rab33a	5	3	7	7	5	6
Rab38	2	0	1	1	0	2
Rab3c	1	0	2	0	0	4
Rab3ip	3525	2396	2213	3171	2136	3648
Rab42	10	5	12	14	10	7
Rab4a	299	102	189	263	109	350
Rab6a	6032	4425	3695	5536	4018	6645
Rabgap1l	4848	4091	2689	4872	2816	5637
Rabggta	626	350	284	505	328	571
Rabl3	294	221	166	255	164	343
Rad21	6002	4137	3178	5354	3619	6168
Rad23a	3019	1928	1543	2406	1875	2879
Rad50	748	673	515	697	598	930
Rad51c	77	51	48	78	51	74
Rad9b	90	52	45	65	50	81
Raet1a	2	1	2	1	1	3
Raet1b	2	1	2	1	1	3
Raet1d	2	1	2	1	1	3
Raf1	5212	3353	2692	4605	3006	4475
Rai2	1947	1348	1087	1600	1256	1880
Raly	2816	1778	1358	2501	1401	2923
Ranbp3	1529	875	724	1240	808	1446
Rap1a	2162	1501	1580	1913	1391	2576
Rap1gds1	2266	1535	1242	2085	1216	2303
Rap2a	1094	708	559	1030	583	965
Rap2b	442	271	219	398	181	474
Raph1	7861	5721	4605	6499	5618	7403
Rara	558	352	228	457	300	513
Rarb	1076	666	661	875	783	908
Rarres2	641	403	372	584	317	765
Rasd2	321	195	173	293	136	389
Rassf2	275	204	192	279	159	325
Raver1	1581	1106	774	1463	879	1518
Rb1	2304	1600	1285	1967	1420	2214

Rbbp6	4107	2899	2186	3347	2589	4310
Rbbp9	844	626	508	725	547	986
Rbfa	1714	1167	932	1420	962	1866
Rbfox1	3051	2342	1615	2770	1694	3250
Rbm15b	417	258	227	380	228	377
Rbm24	14684	10307	8593	13375	8598	15314
Rbm27	1775	1189	1010	1572	1068	1740
Rbm33	3064	1880	1642	2199	1945	3126
Rbm34	253	184	172	212	174	286
Rbm39	8853	6387	4860	7460	5429	9773
Rbm3os	7	7	2	9	3	7
Rbm4b	215	164	121	189	124	259
Rbm8a	1455	975	945	1207	940	1702
Rbmx	2924	2052	1612	2241	1947	3256
Rbmxl1	196	120	97	187	92	204
Rbpms	6828	5095	3723	6198	4159	7220
Rc3h1	4039	2155	1983	3380	1850	3730
Rcbtb2	1069	716	600	978	594	1071
Rce1	205	119	96	174	92	192
Rcl1	224	127	127	205	92	232
Rcor2	282	153	134	236	141	266
Rd3	7	7	5	5	3	13
Rd3l	1	1	1	1	2	0
Rdh10	328	181	204	270	188	318
Rdh18-ps	1	0	2	0	0	4
Rdh5	135	75	53	107	65	118
Rdm1	814	477	408	643	437	784
Redrum	0	1	0	0	0	1
Rell2	5	2	1	1	2	5
Reln	389	284	176	346	223	334
Relt	87	43	32	69	36	64
Rem2	1	1	0	0	1	1
Rer1	3421	2629	2500	3578	2223	4345
Rere	4270	3048	1948	3622	2609	4049
Rev3l	1163	718	554	1083	579	1053
Rfc3	127	83	74	92	66	157
Rfc4	245	168	170	222	136	331
Rfesd	432	344	307	366	322	517
Rfwd3	919	578	477	795	513	894
Rfx1	719	348	226	612	264	564
Rfx2	227	129	74	235	88	118
Rfx4	0	0	1	1	0	0
Rfx7	974	674	554	812	598	1031
Rfxap	614	299	310	470	302	598
Rgag1	6	7	5	12	3	7

Rgma	1643	1200	768	1606	932	1471
Rgmb	909	550	369	748	417	793
Rgs14	22	7	12	18	8	19
Rgs22	8	7	5	9	6	8
Rgs9	11	7	7	6	7	13
Rhbdl2	1	2	1	1	1	2
Rhoa	8499	6614	5097	7918	5059	9743
Rhod	110	59	78	96	63	137
Rhox2c	1	0	0	1	0	0
Rhox2h	4	2	2	2	3	3
Rhox6	0	1	0	1	0	0
Ric8b	2460	1695	1398	2003	1659	2401
Rims1	3	0	1	2	1	2
Rin2	1800	1214	920	1685	884	1808
Ring1	568	341	274	397	306	583
Riok1	533	406	330	463	341	642
Ripk2	257	209	142	239	146	321
Rlim	2077	1319	1208	1718	1229	2243
Rmnd1	1005	995	926	1019	738	1621
Rmst	21	7	5	9	10	19
Rnase1	6	5	2	3	9	3
Rnase10	20	9	11	13	5	25
Rnase4	2314	1470	1583	2228	1134	2788
Rnaseh2a	462	251	252	347	244	449
Rnaseh2b	417	290	250	397	220	448
Rnf111	1427	885	735	1077	890	1408
Rnf113a1	62	49	48	73	35	71
Rnf113a2	434	297	246	314	271	484
Rnf121	573	342	288	483	310	524
Rnf126	744	419	316	532	394	735
Rnf130	2409	1701	1532	2208	1494	2837
Rnf149	466	336	242	374	248	549
Rnf168	1402	939	843	1398	767	1403
Rnf185	1174	757	668	1079	653	1130
Rnf187	4728	2706	2327	4064	2307	4319
Rnf19a	1747	1181	1011	1542	1075	1829
Rnf2	1072	785	755	982	657	1427
Rnf216	1626	1125	801	1491	914	1428
Rnf222	17	4	4	5	9	15
Rnf31	1297	756	475	1009	612	1207
Rnf40	2453	1786	1350	2112	1515	2551
Rnf41	647	412	356	568	366	623
Rnpepl1	2819	1598	1205	2212	1464	2535
Rogdi	497	342	244	401	262	523
Ropn1	2	1	2	2	0	5

Rora	3456	2204	1764	3029	1966	3239
Ros1	1	2	0	3	0	1
Rp2h	787	530	453	698	461	801
Rpa1	3402	1944	1620	2941	1707	3085
Rpap1	799	464	331	614	390	677
Rpf1	823	661	523	651	578	1094
Rpgrip1l	304	192	171	266	191	251
Rpia	512	360	309	450	321	524
Rpl14-ps1	33	16	15	22	18	32
Rpl18a	1646	1393	953	1457	1108	2050
Rpl19	790	763	758	862	644	1120
Rpl22l1	351	143	219	291	145	403
Rpl23	3668	3580	2489	3323	2795	4622
Rpl23a	13	8	8	10	7	13
Rpl30	411	166	350	423	182	523
Rpl39l	2	3	4	1	3	5
Rpl41	2553	2281	1825	2426	1728	3406
Rpl5	462	313	270	411	261	499
Rpl7	6091	4826	3927	5569	4101	7572
Rpl7a	874	502	522	785	462	873
Rplp1	3951	2105	2229	3395	1950	4286
Rpp25	83	63	48	82	48	98
Rpp40	132	142	105	114	131	157
Rprm	2	2	1	3	1	1
Rps15a-ps4	9	4	3	3	7	6
Rps17	107	79	72	87	67	128
Rps19bp1	192	172	136	190	135	221
Rps19-ps3	43	27	12	33	14	38
Rps27l	1094	758	795	924	708	1321
Rps3	9762	6035	5345	8231	4931	10498
Rps5	6429	3610	3148	5325	2957	6963
Rps6kc1	616	441	352	632	356	602
Rps6kl1	142	74	53	130	50	111
Rps7	84	23	44	72	30	73
Rrbp1	6121	4676	3396	5250	4196	6527
Rrnad1	641	361	258	375	322	637
Rrp1	4026	2886	2256	3468	2429	4524
Rs1	20	12	9	12	11	21
Rsc1a1	948	679	553	853	623	969
Rsrc1	762	532	448	616	462	841
Rtcb	3132	2009	1728	2697	1786	3103
Rtkn	263	160	131	211	130	256
Rtn3	5836	4178	3411	5499	3415	5891
Rtn4rl1	551	238	200	327	306	364
Rtn4rl2	81	49	47	73	39	81

Rufy2	779	508	441	521	523	831
Rundc3a	2	1	0	0	1	2
Rundc3b	24	15	9	16	11	28
Runx1	101	85	46	99	48	102
Rxfp2	2	0	0	0	1	1
Rxfp3	1	2	1	0	2	3
S1pr2	244	144	94	224	82	215
S1pr5	79	44	21	55	33	71
Saa1	1	0	1	0	0	2
Sae1	1423	959	792	1292	748	1471
Safb2	2116	1328	911	1346	1239	2041
Sall4	123	67	53	101	63	112
Samd11	2	4	0	1	2	5
Samd14	108	57	47	88	44	113
Samd3	7	1	0	5	0	2
Samd8	1790	1131	1031	1620	1014	1857
Samm50	9633	5568	5612	8203	5147	10257
Sap30bp	538	419	337	476	346	613
Sar1b	4326	2432	2589	3496	2392	4519
Sarm1	15	7	2	6	9	7
Satb1	1416	962	870	1139	1016	1464
Sav1	1827	1260	1022	1648	1036	1972
Sbf2	4574	3178	2083	3816	2824	4138
Sbk1	5027	3232	2572	4618	2636	4548
Sbno2	765	423	351	582	370	784
Scaf1	2900	1823	1314	2454	1520	2726
Scamp3	1366	831	564	1079	673	1231
Scamp5	496	270	280	443	216	560
Scap	1375	871	739	1196	797	1345
Scg5	2	2	2	5	2	1
Schip1	5465	3512	3084	4558	3454	5145
Scimp	9	8	4	8	5	11
Scly	637	402	329	517	380	615
Scn11a	2	2	1	0	4	2
Scn4a	1108	572	625	633	825	898
Scn5a	17272	13180	10498	17913	11105	15344
Scn8a	10	4	0	1	4	12
Scpep1	690	414	368	611	326	669
Scrib	1407	824	672	1184	725	1206
Scrn2	389	160	162	236	189	316
Sct	0	1	0	0	0	1
Sctr	0	0	1	0	0	1
Scyl1	3030	1983	1572	2493	1745	2967
Sdc2	3400	2499	1865	3322	1709	3668
Sdcbp2	49	29	30	25	33	60

Sdccag8	360	357	186	350	234	484
Sdf2	1302	797	668	1110	661	1337
Sdhd	18720	10822	10875	16069	10102	19543
Sdr39u1	2713	1721	1476	2250	1533	2697
Sec11a	1070	694	601	948	592	1185
Sec14l4	1	0	0	1	0	0
Sec14l5	120	54	48	94	59	90
Sec23b	770	405	409	636	384	702
Sec24b	1915	1396	920	1645	1200	1870
Sec31b	1366	851	611	990	797	1186
Secisbp2	1124	772	551	836	647	1159
Sectm1a	1	1	1	0	0	4
Selo	759	467	383	632	430	745
Sema3b	1424	985	830	1190	896	1423
Sema3e	21	17	11	11	17	28
Sema4a	147	102	64	137	53	152
Sema4b	655	428	276	633	330	494
Sema4g	10	8	5	6	10	12
Sema5a	484	306	244	504	237	424
Sema5b	47	34	31	47	23	52
Senp3	1708	1107	951	1350	974	1846
Senp7	987	699	490	815	582	1038
Sephs1	961	582	500	838	516	963
Sepp1	19618	15397	11754	16683	12053	24303
1-Sep	75	57	44	78	40	84
11-Sep	1647	1054	731	1402	844	1639
8-Sep	3459	2243	1686	2881	1712	3546
Serinc1	18766	13516	11372	16240	11946	19161
Serpina1e	5	5	0	0	4	5
Serpina3c	9	6	23	6	11	28
Serpina3f	14	4	2	7	2	14
Serpina3h	8	3	6	6	5	9
Serpina3n	666	289	468	421	292	1029
Serpinb1b	0	0	1	1	0	0
Serpinb2	4	0	0	1	1	2
Serpind1	18	9	10	11	9	23
Serping1	4659	3208	2543	4624	2147	4896
Sertad2	2159	1302	1164	1723	1281	2175
Sesn1	9661	5746	4905	7801	5559	9352
Setd1b	1755	1253	791	1605	914	1563
Setd5	3416	2289	1650	2759	2032	3362
Setdb2	403	282	178	271	237	465
Sf3b1	11912	7993	6488	10325	6747	12826
Sf3b2	7880	5836	4555	6747	5086	8310
Sfmbt2	18	9	16	11	10	32

Sfswap	1708	1187	832	1338	957	1943
Sft2d1	101	95	62	94	77	124
Sft2d2	2278	1400	1184	1894	1265	2373
Sft2d3	236	177	170	206	155	315
Sfxn5	235	165	143	175	145	306
Sgcz	1	0	0	1	0	0
Sgpl1	1105	686	563	949	648	1037
Sgta	3081	2010	1556	2427	1823	3007
Sh2b1	2741	1538	1362	2193	1444	2395
Sh2d5	11	0	2	6	1	5
Sh2d7	1	0	0	0	0	1
Sh3d21	299	201	129	261	157	247
Sh3pxd2a	4115	2915	2182	3891	2402	3736
Sh3pxd2b	313	229	163	334	137	341
Shc3	10	9	11	12	8	12
Shc4	50	28	26	42	30	34
Shcbp1	30	43	34	56	19	53
Shisa9	9	4	2	5	5	7
Shmt2	1597	1012	797	1325	881	1493
Shoc2	2226	1550	1253	2002	1356	2075
Shq1	107	64	28	79	53	83
Shroom1	49	22	13	21	28	37
Sidt2	3290	2061	1676	2792	1893	3188
Siglec5	0	1	0	0	1	0
Siglece	59	29	21	43	23	56
Sin3a	1892	1322	982	1577	1180	1978
Sipa1l3	453	312	188	395	234	376
Sirpa	1795	1107	993	1529	1116	1671
Sirt1	1314	761	678	999	704	1267
Sirt2	3574	2299	1936	3015	2223	3535
Sirt5	1307	860	846	1193	803	1497
Siva1	322	179	157	242	161	335
Skap1	5	8	1	10	3	4
Skint3	8	9	4	7	4	11
Skp1a	7772	4700	4775	6431	4696	8763
Slamf7	28	28	13	37	6	43
Slc11a1	101	71	86	110	61	142
Slc12a6	3543	2414	2031	2972	2197	3652
Slc14a1	30	20	16	23	13	43
Slc15a4	515	267	252	437	263	397
Slc16a9	223	150	196	175	143	376
Slc17a5	437	302	273	395	249	545
Slc17a9	25	12	17	14	9	36
Slc1a3	137	130	74	197	61	158
Slc1a4	140	69	67	135	47	116

Slc22a12	7	3	2	2	4	6
Slc22a18	8	2	7	4	7	9
Slc23a2	1373	844	696	1123	754	1436
Slc23a3	6	1	3	5	3	2
Slc25a12	10943	6650	6194	10529	5922	9705
Slc25a17	814	500	454	724	408	882
Slc25a18	2	0	5	1	0	7
Slc25a20	7398	3939	4516	5636	4736	7619
Slc25a3	68446	39962	36493	60351	35036	71141
Slc25a31	30	11	18	18	15	35
Slc25a33	1300	707	539	891	537	1394
Slc25a35	293	249	188	303	211	325
Slc25a51	3639	2223	2138	3307	2080	3327
Slc26a1	0	1	0	0	0	1
Slc26a3	3	2	6	9	3	2
Slc27a1	6617	3485	2429	3793	3341	6433
Slc27a5	2	5	3	1	6	3
Slc2a2	8	4	5	4	7	9
Slc2a3	158	86	78	94	86	162
Slc30a1	1712	941	958	1232	925	1843
Slc30a5	1258	804	730	1146	668	1313
Slc30a6	364	271	236	371	218	391
Slc33a1	990	535	519	892	506	901
Slc35a1	716	443	414	605	382	771
Slc35c2	534	356	287	483	306	542
Slc35e1	6092	4080	3923	5953	4056	5304
Slc35f1	121	65	48	75	55	138
Slc35f5	6493	3559	3790	6023	3668	5916
Slc35g2	175	88	86	160	75	161
Slc38a10	4467	2742	2210	3718	2623	4110
Slc38a7	789	529	380	640	435	766
Slc38a9	1156	785	687	954	642	1332
Slc39a3	757	364	327	614	326	683
Slc39a6	274	187	198	250	150	342
Slc39a7	2495	1572	1382	2104	1325	2600
Slc3a1	19	6	3	3	7	18
Slc3a2	1684	1128	928	1600	937	1729
Slc40a1	1198	810	738	991	767	1350
Slc41a1	3739	2512	1891	3563	2159	3370
Slc43a2	799	570	539	726	448	972
Slc45a4	784	506	458	724	515	739
Slc4a1ap	853	671	530	811	530	978
Slc4a9	0	1	3	1	1	2
Slc6a14	0	1	0	1	0	0
Slc6a9	139	77	61	112	53	144

Slc7a8	116	84	44	99	57	108
Slco1a4	60	36	22	31	29	66
Slco1c1	16	8	3	14	4	13
Slco4a1	3	2	1	5	0	1
Slfnl1	2	0	1	1	1	1
Slirp	907	538	654	805	502	1081
Slit1	10	2	7	7	3	9
Slit2	436	460	241	526	226	517
Slitrk2	15	15	12	23	7	16
Slitrk4	122	85	59	131	49	110
Sltm	2789	2159	1820	2176	2097	3465
Slu7	2099	1634	1178	1809	1392	2351
Smarcal1	1647	1047	817	1431	957	1386
Smarcc2	5283	3889	2819	4481	3264	5224
Smarcd3	2380	1467	1238	2124	1133	2303
Smc1a	3312	2460	1813	2923	2066	3591
Smc5	1959	1358	1054	1456	1184	2121
Smco1	934	650	593	778	681	966
Smim15	1277	800	668	1079	642	1333
Smim19	1744	1164	1118	1328	1132	1947
Smim20	2159	1282	1272	1941	1108	2360
Smndc1	1644	1113	1070	1498	955	1802
Smpd2	1057	598	581	852	525	1230
Smpd4	1090	681	557	862	638	1077
Snai1	79	54	34	81	39	69
Snai3	134	36	82	78	79	133
Snap47	1418	905	841	1348	786	1478
Snapc1	444	348	317	551	265	427
Snapc5	699	547	487	674	466	897
Snd1	3201	1989	1478	2630	1629	2933
Snhg3	145	122	106	128	103	179
Snhg6	157	105	109	133	88	191
Snhg8	176	101	125	147	109	215
Snhg9	0	0	2	0	1	1
Snip1	436	259	221	364	232	436
Snn	1341	815	675	1054	733	1387
Snora21	1	0	1	0	0	2
Snora23	2	2	3	3	2	2
Snora2b	4	3	1	5	3	1
Snora34	26	13	8	17	9	25
Snora65	19	15	4	17	5	22
Snora68	23	18	9	11	10	32
Snora78	1	0	3	1	1	2
Snord12	15	7	6	6	8	14
Snord1a	8	1	0	0	2	6

Snord1b	1	1	0	0	0	2
Snord2	38	23	20	30	17	42
Snord22	31	11	17	13	18	30
Snord35b	0	2	0	0	2	0
Snord4a	6	2	0	0	3	4
Snord55	1	0	0	1	0	0
Snord7	15	5	10	18	2	17
Snord71	8	4	1	7	1	8
Snord83b	1	4	2	2	2	3
Snord95	3	4	3	3	3	7
Snrnp27	1488	1049	1126	1270	931	1933
Snrpa1	786	537	430	599	483	849
Snrpf	184	122	127	149	103	241
Snx1	1152	773	700	1072	668	1294
Snx15	462	275	237	352	240	470
Snx17	2252	1245	1085	1915	1095	2138
Snx18	1588	936	745	1361	734	1438
Snx24	430	247	239	330	246	450
Snx33	538	381	315	557	306	536
Snx6	1435	913	842	1202	819	1512
Sos1	1648	1134	999	1441	1168	1650
Sox30	9	8	6	2	4	23
Sp3	3247	2303	1643	2713	1945	3387
Spa17	140	126	107	130	123	150
Spag8	2	7	3	1	4	10
Spag9	15061	9813	8167	12239	9566	14677
Spast	1864	1258	1184	1684	1187	1878
Spata1	134	96	72	89	98	152
Spef2	0	0	2	1	1	1
Sphkap	17	11	9	23	10	8
Spi1	121	79	43	102	53	129
Spin1	1836	1093	999	1648	1013	1708
Spock1	2	3	1	3	1	3
Sprn	29	33	20	32	17	42
Spsb1	2021	1148	863	1591	1160	1631
Sptlc1	934	593	509	743	498	1007
Spty2d1	806	503	503	648	473	902
Sqrdl	2012	1341	1180	1885	980	2210
Sra1	1438	950	886	1165	848	1743
Srp54b	517	462	367	462	395	656
Srp54c	517	462	367	462	395	656
Srp9	1017	677	642	936	616	1124
Srpx	171	84	105	116	75	220
Srrm2	18975	14264	9566	13800	12089	20305
Srrm3	1	0	0	0	0	1

Srsf10	2509	1554	1503	2158	1415	2658
Srsf12	1	1	2	1	2	2
Srsf4	1586	1058	870	1360	909	1631
Ssh2	10042	6698	4763	9068	5408	9359
Ssh3	366	201	152	248	189	332
Ssr2	1292	778	675	1119	610	1336
Ssr3	10603	7264	6219	8991	6452	10972
St3gal1	2365	1371	952	1864	1262	1915
Stac3	53	26	22	38	19	60
Stag1	2292	1563	1219	1986	1409	2325
Stambpl1	137	104	110	153	85	174
Stamos	5	0	2	2	3	3
Stard10	1036	606	530	796	584	980
Stat1	3852	2053	1695	3596	1621	3452
Stat3	6165	3878	3403	5894	3333	5495
Stat4	12	5	8	13	3	15
Stil	30	8	7	12	5	37
Stim1	952	579	504	818	515	950
Stk3	565	478	352	492	400	709
Stk31	1	2	0	0	1	2
Stk32a	28	30	22	28	24	41
Stk33	2	2	2	3	1	3
Stk38	2415	1693	1532	2148	1490	2806
Stk38l	912	627	565	945	563	906
Stmn1	85	51	42	54	52	103
Stom	6752	4079	3835	5605	3819	6664
Stoml2	2262	1546	1195	1810	1246	2457
Stra13	401	284	204	340	219	423
Strada	515	285	216	398	236	476
Strip2	8061	5335	4331	7458	4932	6535
Stub1	3863	2494	1881	3178	2056	4125
Stx17	2172	1605	1406	1865	1533	2518
Stxbp3b	0	0	1	1	0	0
Stxbp5	1526	1015	774	1239	866	1553
Styx	196	103	116	160	85	256
Suco	1244	1009	752	1134	880	1407
Sugp2	1504	953	721	1193	805	1441
Sugt1	1943	1395	1199	1838	1087	2120
Sumf2	240	159	146	222	146	259
Sumo3	1836	1309	1097	1648	1138	2080
Sun1	3127	2065	1598	2765	1753	3070
Sun2	4127	2465	2154	3706	2198	3529
Suox	1135	681	651	951	709	1031
Supt20	805	572	451	589	511	908
Surf4	5905	3672	3198	5310	2889	5779

Susd3	21	13	7	17	7	18
Suv420h1	2557	1657	1320	1932	1521	2521
Sv2a	329	190	162	308	161	259
Swi5	4002	2981	2406	3415	2518	4601
Swsap1	273	154	147	213	129	302
Swt1	725	476	373	582	404	751
Syap1	2525	1689	1459	2094	1550	2626
Syn1	10	25	7	17	11	16
Syngr1	1673	944	965	1359	852	1807
Syngr3	2	2	2	3	1	3
Synpo	6710	4455	3529	5871	3835	6217
Sys1	610	270	264	450	274	558
Syt1	0	0	1	0	0	1
Syt11	954	614	526	796	590	895
Syt13	1	3	1	2	4	0
Syt16	1	0	0	1	0	0
Syt3	102	50	44	69	57	95
Syt5	18	12	8	19	8	18
Syvn1	973	553	471	781	486	876
T2	26	19	20	19	27	31
Tacr3	0	1	0	0	0	1
Taf10	451	270	241	348	210	513
Taf5l	562	380	285	503	289	604
Taf6l	158	95	78	138	82	143
Tagap1	334	184	184	261	180	314
Tal2	6	6	9	13	4	9
Tapt1	2020	1188	1095	1663	1155	2045
Tarbp2	351	171	138	252	158	317
Tardbp	6332	3958	3490	4943	3641	6990
Tas2r135	4	0	1	1	2	3
Tatdn1	439	361	350	426	327	611
Tbata	2	0	1	1	1	1
Tbc1d10c	270	125	106	170	132	222
Tbc1d14	800	480	410	698	448	755
Tbc1d19	985	594	578	797	556	1005
Tbc1d20	1089	596	566	982	597	955
Tbc1d25	211	113	92	169	101	199
Tbc1d8	578	319	234	388	282	552
Tbca	1007	547	642	844	512	1229
Tbcd	653	398	339	520	323	748
Tbcel	1162	785	764	1005	795	1321
Tbl2	531	369	260	467	318	432
Tbl3	703	408	347	612	349	690
Tbx3os2	0	1	0	0	1	0
Tbx4	2	1	1	1	0	4

Tceal1	453	360	228	383	261	539
Tceal5	4	4	0	4	1	3
Tceb1	2149	1302	1458	1952	1147	2512
Tcf21	290	189	123	307	111	245
Tchhl1	8	0	5	3	4	5
Tcn2	2408	1508	1178	2169	1137	2458
Tcp10b	2	4	2	5	2	1
Tcta	2009	1018	1101	1509	1055	2146
Tcte2	5	3	13	5	7	10
Tcte3	0	1	0	0	0	1
Tctex1d4	2	0	3	2	1	3
Tctn3	439	260	213	387	203	412
Tdp1	315	232	189	276	186	343
Tdrkh	234	177	123	212	147	246
Tead3	664	359	288	505	292	636
Tectb	7	6	5	3	8	10
Tekt2	27	27	19	18	24	47
Tekt5	0	0	1	0	0	1
Telo2	387	227	189	366	178	360
Tenm2	1	1	2	1	3	1
Tepp	2	0	0	1	0	1
Terc	1	0	1	1	0	1
Terf2	982	696	504	807	525	1097
Terf2ip	852	588	543	930	508	828
Tert	3	0	1	3	1	1
Tet2	2148	1449	1224	1438	1298	2946
Tex22	0	0	2	2	0	0
Tex261	1678	1012	877	1444	963	1533
Tfap2e	3	3	0	1	1	5
Tfr2	2	1	1	2	1	1
Tgds	394	275	239	337	240	456
Tgfb2	604	422	362	563	389	576
Tgfbr1	1592	1015	824	1454	817	1462
Tgfbr2	3385	2297	2049	3142	1985	3843
Tgfbr3	4633	2607	1830	3617	2322	3671
Tgfbrap1	1451	1036	793	1252	866	1447
Tgif1	200	113	96	189	88	199
Tgm5	1	0	2	0	1	2
Tha1	250	168	120	215	124	253
Them6	355	210	142	319	138	314
Them7	3	1	1	0	5	1
Thg1l	196	147	109	152	132	215
Thra	2010	1357	1168	1933	1302	1889
Thrap3	3150	2291	1730	2830	1986	2918
Thyn1	606	424	378	481	374	718

Tifab	59	36	19	65	13	61
Tigd2	793	610	512	792	482	840
Tigd4	6	1	6	5	7	2
Tigd5	152	81	55	138	67	110
Timm10b	601	365	318	502	300	668
Timm13	893	474	418	734	391	934
Timm17a	4653	3040	3188	4014	2755	5656
Timm17b	619	440	305	542	376	634
Timm22	914	519	599	842	452	988
Timm50	1305	679	678	1063	652	1261
Tinag	0	0	1	0	1	0
Tktl1	0	1	0	0	0	1
Tlcd2	161	111	78	163	78	146
Tle2	530	317	209	350	253	577
Tlk1	2863	2172	1819	2718	1873	2951
Tlr1	49	27	27	49	30	40
Tlr5	29	25	15	29	14	39
Tlx1	7	4	2	4	3	6
Tm2d3	352	227	185	287	215	352
Tm4sf4	1	3	1	3	1	2
Tm7sf2	124	46	42	82	54	82
Tm7sf3	1114	768	576	924	607	1252
Tmco2	1	0	0	0	0	1
Tmed10	6016	4039	3482	5148	3523	6214
Tmed2	1336	663	742	1113	689	1283
Tmed3	772	442	378	657	346	806
Tmem104	559	332	257	526	284	468
Tmem106a	235	176	132	215	119	313
Tmem109	4398	2642	1998	3788	2052	4042
Tmem120a	429	265	213	342	205	486
Tmem123	3627	2410	2279	3466	2027	4040
Tmem132b	0	2	0	1	0	2
Tmem14a	143	104	70	119	91	135
Tmem150c	208	161	166	165	183	258
Tmem151b	0	2	0	1	0	2
Tmem163	54	48	69	71	61	50
Tmem167	939	605	549	840	501	1091
Tmem167b	1928	1411	1172	1783	1151	2063
Tmem168	1557	1015	925	1462	885	1489
Tmem169	3	1	3	2	4	1
Tmem17	104	63	66	83	64	129
Tmem170	168	111	83	113	99	195
Tmem180	127	57	37	77	50	117
Tmem181a	451	315	270	361	290	485
Tmem183a	1044	663	581	937	565	1109

Tmem184b	1847	1215	919	1731	944	1802
Tmem192	528	370	356	458	350	632
Tmem196	17	10	12	18	9	18
Tmem209	659	397	339	523	348	670
Tmem216	128	70	56	103	52	118
Tmem218	469	352	292	435	292	523
Tmem219	179	122	97	156	103	200
Tmem220	130	91	109	85	118	174
Tmem222	839	498	435	665	441	823
Tmem223	474	275	232	435	243	435
Tmem230	499	301	272	453	246	492
Tmem234	781	608	421	678	494	874
Tmem242	2878	1799	1587	2471	1561	3066
Tmem243	357	298	243	355	245	455
Tmem245	3990	2964	2258	3666	2613	4005
Tmem260	663	437	378	549	392	702
Tmem30c	0	0	2	0	2	0
Tmem38a	19936	12264	11046	18030	10888	18881
Tmem5	1012	808	702	841	742	1198
Tmem57	2618	1891	1417	2179	1671	2634
Tmem59	6045	3476	3319	5157	3148	6436
Tmem60	766	475	496	732	390	856
Tmem63a	1064	668	597	971	588	1064
Tmem66	10438	5608	5548	7931	5294	10322
Tmem68	562	351	285	483	282	592
Tmem74	1	0	0	0	0	1
Tmem87a	1318	795	794	1134	748	1407
Tmem89	1	0	1	2	0	0
Tmem9	341	194	175	298	170	335
Tmem98	438	260	259	373	230	452
Tmpo	3091	2055	1433	2596	1600	3059
Tmprss5	5	1	2	3	3	4
Tmtc3	1323	924	687	1134	800	1369
Tmx4	4735	3228	2700	4594	2560	4573
Tnf	9	4	12	10	6	12
Tnfaip8	2915	2353	1879	2546	1999	3708
Tnfrsf23	155	95	132	178	84	165
Tnfrsf25	151	64	59	85	61	148
Tnfsf11	2	2	2	4	2	1
Tnfsf14	7	3	0	7	2	2
Tnni3	153726	115094	92800	136786	99949	187798
Tnnt1	659	559	349	631	387	742
Tnrc6b	5097	3166	2836	3940	3307	4941
Tob2	4615	2658	2389	3399	2556	5038
Tollip	4142	2647	2312	3748	2370	4092

Tomm22	2657	1690	1844	2359	1574	3210
Tomm34	889	589	503	742	517	938
Tomm6os	20	17	6	15	10	19
Tomm7	1579	853	1119	1487	809	1938
Topbp1	1028	652	563	905	577	1060
Topors	1031	757	603	894	670	1188
Tor1b	1386	891	732	1162	736	1512
Tox2	42	36	15	43	21	42
Tox4	1901	1255	1165	1838	1080	1915
Tpd52l2	2467	1562	1375	2059	1372	2584
Tpgs1	307	163	128	229	123	308
Tph1	2	0	1	1	2	1
Tpm1	354570	281529	232389	342127	227397	443360
Tpp1	3281	2316	1726	2829	2032	3112
Tradd	81	56	38	72	42	85
Traf1	32	17	18	18	16	49
Traf2	679	287	294	537	267	542
Traf4	175	113	90	149	98	179
Traf5	162	96	79	150	85	153
Tram1l1	8	10	7	15	3	14
Trappc11	1794	1133	902	1459	1049	1750
Trappc12	1595	1136	856	1401	896	1670
Trappc5	1148	674	639	903	598	1196
Trappc6a	197	94	91	134	88	187
Trappc9	1750	1016	743	1454	854	1550
Trdmt1	369	258	274	345	260	424
Trdn	17217	13962	11960	15098	11645	22374
Trem1	3	0	0	1	0	3
Trhde	24	32	6	42	7	30
Triap1	602	348	341	504	330	626
Trib1	1226	854	726	1325	700	1209
Trib3	121	71	49	123	59	94
Trim28	2445	1487	1099	2170	1190	2278
Trim29	2	3	0	3	0	2
Trim33	1550	1039	905	1368	968	1583
Trim46	43	25	20	33	23	35
Trim54	8355	4883	4462	5729	5666	8256
Trim63	7936	4788	5046	5407	5522	8221
Trim65	220	122	80	175	105	196
Trim67	46	37	33	45	45	39
Trim68	664	496	405	515	460	839
Trim8	1691	1129	825	1491	929	1547
Trio	2824	1987	1584	2229	1802	3258
Trip10	4919	3284	2583	4040	2969	5063
Trit1	308	188	193	258	170	330

Trmt13	283	181	130	199	165	275
Trmt61b	119	72	67	101	69	121
Trpm1	2	1	1	0	3	1
Trpm2	25	20	5	24	10	22
Trpm7	6498	4406	3338	5056	3805	7380
Trrap	2889	1939	1288	2372	1687	2616
Tsc2	5933	4174	3263	5360	3469	5666
Tsc22d4	6325	3953	2868	5356	3268	6033
Tsen54	232	130	125	210	117	223
Tsfm	1160	645	576	920	584	1108
Tsg101	1968	1317	1142	1686	1232	2136
Tshr	0	1	1	0	2	0
Tslp	57	37	49	60	22	81
Tspan10	2	1	0	0	2	1
Tspan12	2696	1597	1726	2229	1475	2990
Tspan18	431	278	255	463	208	452
Tspan3	7724	4995	4516	6940	4249	7771
Tspan31	2417	1576	1367	2263	1218	2568
Tspan6	375	211	251	313	190	511
Tspear	1	0	0	0	0	1
Tsr3	224	125	113	166	98	252
Tssk4	8	6	5	3	6	12
Tstd1	16	25	6	17	10	23
Ttc1	1528	1066	937	1273	960	1634
Ttc16	16	8	5	7	9	14
Ttc18	4	5	2	4	4	6
Ttc26	174	101	100	153	95	162
Ttc36	7	0	0	4	0	5
Ttc39c	273	150	125	158	149	273
Ttc7b	3016	1983	1911	2547	1995	2966
Ttc9c	1080	734	641	946	684	1029
Ttk	17	8	8	4	2	34
Ttll13	12	4	6	2	11	12
Ttll6	2	0	0	0	1	1
Ttpal	1045	648	763	882	639	1276
Tub	9	6	2	8	5	6
Tuba1c	265	139	142	232	112	258
Tubgcp2	1202	872	663	1068	728	1278
Tubgcp5	762	449	397	618	419	727
Tubgcp6	898	560	427	799	496	800
Tug1	3438	2259	1944	2991	1944	3424
Tusc3	567	378	298	529	274	643
Twf2	4811	3014	3092	3706	2911	5712
Twistnb	460	282	261	392	238	524
Txndc15	1744	1091	999	1436	996	1827

Txndc16	1626	1207	920	1438	1091	1647
Txndc17	1474	1158	1035	1381	885	1983
Tymp	266	161	162	217	143	289
Tyrobp	156	105	100	131	83	209
Tysnd1	602	326	260	501	278	568
U2af1	252	167	170	206	153	292
U2af2	1742	946	821	1434	848	1595
Ubac1	2811	1754	1469	2439	1669	2544
Ubac2	1256	715	627	997	690	1176
Ube2b	15185	8847	8124	11555	8324	15224
Ube2cbp	43	30	18	34	30	38
Ube2e1	1305	800	785	1126	727	1379
Ube2g2	2532	1574	1525	2302	1502	2618
Ube2k	2704	1838	1619	2304	1586	3039
Ube2s	208	84	88	162	82	183
Ubfd1	1616	940	862	1380	958	1449
Ubl4b	0	1	0	0	1	0
Ubl5	2014	1511	1336	1977	1168	2617
Ubn1	2694	1657	1417	2244	1567	2671
Ubox5	352	258	166	299	209	357
Ubp1	2814	2045	1702	2257	1721	3282
Ubr1	2703	1778	1481	2165	1653	2685
Ubr3	13590	9763	8003	11785	9532	12776
Ubr4	8894	6490	4428	8259	5689	7678
Ubr7	1715	1144	1031	1602	973	1700
Ubtd1	354	201	193	295	191	339
Ubxn8	622	499	364	549	408	765
Uck1	345	252	197	353	185	336
Ucma	0	1	0	0	0	1
Ucp2	4277	2368	1831	2873	2050	4498
Ufd1l	2071	1372	1140	1764	1181	2133
Ufm1	1581	1046	1010	1313	1022	1648
Ufsp2	1034	681	622	832	639	1111
Ugcg	1608	982	957	1204	987	1675
Ugp2	16224	10373	12763	14470	12620	17518
Uhrf2	1306	802	637	1178	675	1263
Unc45a	632	349	323	539	302	586
Unc5a	57	34	50	39	34	99
Unk	620	407	297	450	377	657
Uqcrc1	44626	27065	23992	38550	24358	47128
Uqcrc2	43766	28033	25472	39069	25988	44397
Uqcrh	18883	13011	12859	17908	11088	23391
Urm1	604	303	364	521	298	579
Uroc1	1	0	0	1	0	0
Usp20	1693	1049	808	1339	981	1471

Usp22	1875	1274	979	1546	1078	1910
Usp27x	43	30	16	43	20	41
Usp3	718	572	405	572	447	840
Usp34	4416	2937	2604	3990	2879	4410
Usp4	4321	2981	2414	3890	2488	4415
Usp45	1222	827	704	971	768	1381
Usp48	2217	1421	1071	1680	1252	2216
Usp49	376	239	222	247	276	422
Usp54	2660	1982	1518	1742	1882	3083
Utp11l	630	535	445	556	426	833
Utp23	307	171	187	245	183	334
Uxt	334	215	162	266	179	365
Vamp1	544	262	265	327	325	546
Vangl1	476	295	247	414	229	487
Vars	1320	757	583	1088	642	1272
Vasn	444	270	191	463	192	374
Vdac1	46122	31028	27910	41895	27903	45744
Vgf	0	1	0	1	0	0
Vipas39	1368	1004	773	1293	773	1384
Vkorc1	277	205	138	247	135	300
Vmn1r181	0	0	1	0	0	1
Vps25	2752	1823	1571	2211	1628	3234
Vps26b	2868	1825	1627	2606	1589	2841
Vps29	1360	901	931	1224	817	1586
Vps33a	1848	1167	905	1616	1017	1637
Vps36	935	658	604	845	610	938
Vps37a	2078	1221	1214	1845	1095	2021
Vps37c	933	566	452	773	506	851
Vps41	2768	1887	1664	2338	1744	2804
Vps45	444	278	255	397	245	436
Vps51	590	357	239	478	275	542
Vps54	2330	1482	1370	2087	1272	2408
Vtcn1	1	1	1	2	2	0
Vti1a	753	559	486	668	538	846
Vwa7	719	404	304	468	425	598
Wasl	3130	2293	1848	2760	1970	3514
Wbp1	1289	786	649	1129	644	1337
Wbp4	1827	1280	1152	1632	1104	2052
Wbscr25	4	1	2	1	3	5
Wdr1	5126	3673	3357	5182	3081	5158
Wdr11	1672	996	849	1402	851	1578
Wdr13	3228	2252	1771	2769	1983	3476
Wdr16	1	0	2	0	1	2
Wdr20	570	452	341	529	387	546
Wdr26	8900	5917	5193	7597	5378	9538

Wdr31	22	11	7	15	9	19
Wdr4	307	168	182	242	163	323
Wdr41	1361	969	802	1205	904	1300
Wdr46	307	187	175	296	152	324
Wdr48	1856	1298	1036	1631	1141	1923
Wdr54	24	16	13	19	14	25
Wdr55	430	283	240	306	288	441
Wdr59	545	373	281	487	305	584
Wdr5b	231	155	120	190	147	227
Wdr6	868	475	355	718	426	744
Wdr73	277	169	128	232	130	285
Wdr74	342	240	253	340	212	447
Wdr81	711	395	364	523	371	715
Wdr90	159	83	69	99	90	161
Wdr91	411	259	243	330	254	461
Wdyhv1	501	315	285	443	277	548
Wee1	1586	973	712	1367	788	1493
Whsc1l1	2259	1484	1116	1838	1301	2110
Wipi1	3955	3067	2873	3700	2767	4514
Wisp1	90	60	57	80	69	69
Wisp3	6	4	3	8	0	5
Wiz	1105	757	502	904	645	1053
Wnk4	802	518	291	507	399	832
Wnt10b	3	6	5	7	2	6
Wnt16	1	2	2	3	1	2
Wnt2b	7	6	2	12	3	2
Wnt8a	1	0	0	0	0	1
Wnt8b	0	1	0	0	0	1
Wnt9b	40	27	13	23	15	51
Wrb	635	297	309	456	312	545
Wrnip1	1383	816	716	1192	724	1375
Wt1	248	118	142	137	144	273
Wwc1	5	2	3	4	5	0
Xcr1	27	10	3	31	1	7
Xkr5	2	3	1	2	4	1
Xpnpep1	1793	1380	1020	1708	1096	1825
Xpo6	2708	1867	1209	1656	1583	3353
Xrcc1	738	526	390	686	433	754
Xrcc2	358	177	204	315	210	308
Xrcc4	414	292	240	372	236	482
Ybx1	26011	19389	13996	21415	16924	28431
Yeats4	934	668	560	728	608	1058
Yif1a	2021	1164	991	1596	1014	1901
Yif1b	1045	625	567	906	542	1001
Ypel4	39	25	13	35	17	30

Ythdf1	2258	1463	1227	1943	1257	2177
Zbed5	24	10	6	22	5	18
Zbtb11	914	618	445	766	550	797
Zbtb12	217	153	117	181	156	206
Zbtb14	699	534	372	621	421	703
Zbtb24	415	303	241	361	251	496
Zbtb25	265	153	130	175	155	296
Zbtb3	69	33	34	57	36	60
Zbtb33	974	598	461	829	480	913
Zbtb6	559	395	352	500	346	579
Zbtb7a	2973	2134	1528	2879	1809	2794
Zbtb7b	913	570	463	898	473	849
Zbtb7c	120	76	49	116	52	88
Zc2hc1a	769	568	435	709	514	781
Zc3h10	351	235	206	334	205	369
Zc3h11a	5979	3869	3276	4982	3306	6904
Zc3h13	2566	2087	1715	2333	1722	3041
Zc3h18	1419	1042	764	1220	932	1417
Zc3h3	74	37	27	46	37	62
Zc3h4	1853	1343	968	1497	1259	1884
Zcchc10	284	161	124	204	149	295
Zcchc12	15	5	3	8	4	16
Zcchc17	1007	794	640	939	595	1198
Zcchc7	938	671	508	634	613	1051
Zcchc8	1178	868	617	1068	739	1220
Zdhhc15	69	53	43	52	31	115
Zdhhc23	2	7	1	5	5	3
Zdhhc7	1298	778	736	1253	704	1118
Zeb1	3908	2746	2311	3356	2373	4207
Zfand2a	587	400	369	585	368	612
Zfand3	3942	2724	2416	3704	2451	3853
Zfand4	420	270	208	240	249	487
Zfml	3451	2397	1888	3279	2000	3216
Zfp1	472	344	305	386	284	580
Zfp11	306	220	178	307	159	313
Zfp113	520	380	319	389	359	583
Zfp143	705	560	445	660	452	781
Zfp148	2838	1910	1503	2384	1723	2731
Zfp212	399	224	227	314	206	469
Zfp229	463	301	282	372	274	513
Zfp236	1522	1203	852	1458	1012	1507
Zfp263	748	504	453	584	459	855
Zfp275	1020	665	479	919	540	987
Zfp276	884	562	379	746	460	818
Zfp277	700	442	377	518	417	765

Zfp3	422	263	225	353	214	438
Zfp319	575	316	255	472	292	469
Zfp329	983	576	581	791	508	1121
Zfp334	332	227	150	243	203	346
Zfp345	0	3	1	2	0	2
Zfp35	468	323	350	497	289	555
Zfp354a	157	132	79	145	97	184
Zfp358	901	612	452	764	517	908
Zfp365	47	43	41	48	26	80
Zfp36l2	1320	865	631	1309	616	1329
Zfp385a	473	317	268	467	233	527
Zfp389	7	3	7	4	3	15
Zfp418	101	64	39	77	47	107
Zfp449	341	229	271	473	179	337
Zfp454	57	39	31	44	23	77
Zfp458	73	45	39	43	57	63
Zfp512	1571	951	877	1224	903	1615
Zfp516	754	448	392	668	419	675
Zfp524	260	144	131	210	151	237
Zfp53	244	191	170	225	144	327
Zfp560	300	149	192	219	149	345
Zfp57	127	68	45	83	63	111
Zfp572	1	1	0	0	0	3
Zfp579	331	229	151	200	234	355
Zfp59	207	151	122	182	126	223
Zfp595	227	146	154	189	153	263
Zfp605	181	133	110	163	142	178
Zfp607	182	102	84	144	80	208
Zfp628	293	175	125	274	151	237
Zfp646	1214	750	591	1004	648	1192
Zfp653	232	148	92	159	123	248
Zfp68	2139	1652	1305	1818	1494	2564
Zfp689	102	81	44	82	69	108
Zfp709	249	179	153	244	133	307
Zfp710	837	585	443	740	500	791
Zfp715	2249	1515	1186	1776	1285	2596
Zfp719	379	296	218	351	206	432
Zfp763	320	233	208	267	203	383
Zfp768	1629	991	781	1437	848	1421
Zfp777	528	407	277	448	356	559
Zfp780b	474	289	245	381	250	476
Zfp804b	1	0	0	0	0	1
Zfp808	135	97	89	100	80	173
Zfp810	483	330	346	334	296	741
Zfp82	130	72	70	100	78	128

Zfp85os	10	7	10	8	7	14
Zfp865	571	383	234	478	312	546
Zfp867	624	388	413	562	351	693
Zfp868	447	271	228	341	248	436
Zfp870	651	426	394	602	400	647
Zfp874a	411	310	295	385	252	512
Zfp879	58	50	51	49	46	85
Zfp93	218	145	116	154	127	243
Zfp936	3	0	2	2	0	3
Zfp94	74	41	29	45	35	91
Zfp949	557	356	355	380	365	660
Zfp963	272	184	138	189	174	274
Zfp97	268	168	138	217	135	273
Zfpm2	1181	825	490	1029	664	919
Zfx	1754	1057	882	1448	968	1761
Zfyve20	1570	950	789	1360	854	1361
Zfyve26	1140	645	511	901	586	998
Zglp1	10	2	4	6	5	5
Zic1	1	0	1	1	0	1
Zic2	0	1	0	0	1	0
Zkscan5	866	599	475	755	530	837
Zkscan8	1354	812	681	1100	778	1313
Zmat1	688	610	397	533	517	817
Zmat2	1860	1432	1200	1678	1246	2225
Zmym2	2561	1953	1510	2467	1722	2666
Zmynd10	6	3	2	5	1	8
Zmynd12	2	4	0	0	2	4
Znf512b	1166	783	625	882	710	1341
Znhit1	1007	600	571	851	521	1132
Znhit3	297	166	140	232	139	280
Znrf1	3095	1764	1352	2254	1630	2929
Zpr1	1120	676	609	896	619	1214
Zrsr2	715	503	432	556	456	865
Zscan12	385	306	252	336	266	502
Zscan25	295	154	145	221	132	296
Zscan29	572	415	383	545	339	661
Zswim2	0	1	0	0	0	1
Zswim3	159	88	83	124	92	164
Zswim8	2647	1684	1313	2191	1520	2567
Zufsp	313	226	172	263	185	338
Zw10	946	495	485	818	516	796
Zwilch	54	33	18	21	23	70
Zfp869	1549	1048	887	1280	963	1619
Lima1	2234	1554	1204	1915	1291	2404
Psen1	1746	1197	1028	1713	1003	1714

Phb	2525	1522	1375	2063	1429	2551
Pvrl3	394	264	223	400	203	382
Naa30	1850	1146	1056	1519	1112	1842
Ppp2r2d	1773	1391	1337	1669	1273	2243
Kctd15	65	35	30	63	26	57
Cpsf6	2890	1897	1601	2470	1647	2996
2810008D09Rik	181	97	106	140	99	194
Bivm	593	466	387	516	433	680
Caprin2	420	355	216	356	242	523
Ccnc	705	437	375	584	385	713
Exoc1	934	687	513	691	563	1148
Nono	2515	1928	1376	2337	1534	2599
Oraov1	678	471	373	578	419	683
Tsn	2829	1766	1593	2409	1510	3091
A730008H23Rik	1069	749	506	768	594	1226
Hgsnat	1154	714	601	946	638	1143
Atg10	1061	757	646	927	670	1159
Cog7	1356	887	670	1170	764	1287
Tbx18	330	189	174	276	161	345
1700027A15Rik	1	0	0	0	1	0
2810468N07Rik	32	16	9	28	13	21
4930515G01Rik	9	2	2	2	5	7
4931409K22Rik	1	0	0	0	1	0
5430427O19Rik	20	13	11	16	10	23
A930006K02Rik	25	14	13	17	19	21
Abhd12b	1	0	0	0	1	0
Acad9	2045	1309	1215	1850	1184	2061
Adamts17	35	25	15	36	12	36
Angpt1	1640	1530	1088	1446	1244	2134
Apoa1	1	1	0	0	2	0
Apoo-ps	22	15	11	22	14	16
Arl6ip6	704	427	333	605	384	616
Asb14	4560	2806	2342	3327	2837	4461
Atp5f1	36164	25779	22131	31588	22310	40685
Atp5g1	2636	1227	918	1805	1055	2337
B230219D22Rik	4892	3095	2637	4262	2770	4682
B3gnt5	37	39	20	35	25	50
Camkk1	61	37	39	87	21	47
Cand2	5462	3633	2856	5047	3397	4570
Ccbe1	683	551	364	608	409	775
Ccdc107	503	363	291	387	325	579
Ccdc142	109	53	63	68	62	123
Cd27	7	5	3	11	3	2
Cdc26	339	203	201	286	182	366
Ctnna1	34763	24389	16911	31784	18862	34110

Dcps	425	280	209	336	233	443
Ddx47	1587	1027	942	1458	917	1591
Dnal1	607	403	303	478	349	635
Dtymk	460	317	221	361	247	503
Eaf2	32	19	33	37	19	41
Evi5	1684	1130	956	1359	963	1917
Ewsr1	5329	3381	2623	3983	2975	5652
Exosc9	942	703	573	837	558	1144
Fbxw4	787	351	380	569	378	705
Foxc2	70	50	29	48	46	70
Gadd45a	610	355	296	289	425	629
Gata4	4389	3265	2488	4280	2735	4196
Gbp8	17	20	11	20	12	22
Gjb3	1	0	0	0	1	0
Gm15413	2	0	0	1	1	0
Gm20556	1	0	0	0	1	0
Gm20735	6	4	1	5	5	1
Gm5535	1	0	0	0	1	0
Gm6793	169	94	95	122	106	163
Gm765	1	0	0	0	1	0
Gng7	64	42	27	51	46	45
Gpc6	1407	1041	621	1486	608	1386
Gpn2	163	106	78	145	71	179
Gsk3a	3101	2207	1612	2645	1870	3117
H2afj	422	233	187	287	222	419
H2-T24	1110	430	487	650	602	865
Hadh	25221	16549	14934	19932	16884	25297
Hars	1756	1203	996	1465	1009	1969
Higd2a	2568	1640	1547	2336	1220	3143
Hmox2	2381	1706	1556	2249	1387	2864
Hpgds	125	80	70	88	61	170
Igbp1	1877	1345	1131	1576	1271	2013
Itk	18	15	5	16	11	15
Jtb	1817	1046	956	1378	949	1963
Kif9	24	8	7	10	13	19
Klhl10	23	18	13	21	15	25
Lepre1	675	451	354	557	390	706
Lmf1	990	564	517	800	559	925
Lrrc39	7423	5460	4651	6234	5304	8015
Lypd2	39	25	10	32	9	44
Macrod2	137	69	69	118	71	114
Mast2	10817	7300	5457	9248	6151	10542
Mettl24	38	17	16	24	18	37
Micu2	4773	3474	3212	4363	3231	5270
Micu3	563	376	295	452	330	597

Mier3	1258	814	662	1028	731	1282
Mir1843	1	0	0	0	1	0
Mir6922	1	0	0	0	1	0
Mir7033	1	0	0	0	1	0
Mro	1	0	0	0	1	0
Mrps26	961	538	502	719	480	1038
Myo5a	784	534	501	642	448	991
Naalad2	692	512	397	703	343	781
Ndufa4	21775	13098	13632	19154	10995	26054
Ndufa5	10164	7798	8040	9375	6858	14367
Ngf	194	173	89	207	99	206
Nlrp1b	30	18	19	22	7	51
Orc6	286	183	139	192	147	343
P4hb	9699	6258	5669	8774	5211	10639
Pef1	982	597	474	858	470	972
Phgdh	12	4	11	14	6	11
Poll	467	260	270	363	256	492
Prdm15	386	246	171	339	229	298
Ptrh1	58	29	32	47	30	54
Pus1	467	299	316	406	308	506
Rab32	123	59	36	73	48	118
Rangrf	326	171	137	255	157	282
Rbl2	3972	2736	2187	3534	2521	3750
Rbm25	7004	5482	4387	5148	4815	9345
Rdh14	2391	1753	1534	2159	1587	2615
Rpl21	961	691	706	888	642	1180
Rpl36al	373	259	287	380	214	484
Rps9	5315	4043	3274	4704	3215	6456
Rsrp1	10408	5819	4666	6797	5111	11165
Scarna3b	1	0	0	0	1	0
Sell	15	20	15	17	16	25
Slc39a11	119	70	70	106	46	146
Slc6a6	11360	7765	4883	9091	6255	11040
Smurf1	1760	1262	1052	1639	1150	1750
Spata2l	232	117	66	161	103	171
Spata4	1	0	0	0	1	0
Spo11	1	0	0	0	1	0
Ssr1	3443	2191	2017	3050	1916	3617
Stx1a	64	45	39	60	37	71
Stxbp2	141	72	43	79	37	174
Svep1	869	704	547	1031	431	1013
Tcf7	46	26	13	38	17	39
Tctex1d1	1	0	0	0	1	0
Tdo2	1	0	0	0	1	0
Tex40	1	0	0	0	1	0

Tjap1	1209	733	505	915	672	1037
Tmem45a	158	103	81	163	61	170
Tmem51os1	8	7	7	7	9	8
Tmem9b	1588	1045	1003	1442	901	1810
Tnfsf18	3	1	4	1	1	8
Tpbg	30	11	10	23	11	22
Trim41	1359	718	704	996	725	1337
Tsx	3	2	2	3	2	3
Ttyh1	41	32	22	33	30	41
Tubgcp3	1186	897	718	990	814	1310
Tulp2	1	0	0	0	1	0
Tunar	1	0	0	0	1	0
Ubxn10	550	355	235	419	306	512
Usb1	410	265	206	344	248	377
Wdr45b	1629	1112	964	1522	903	1753
Zfp14	561	324	261	381	283	605
Zfp667	253	189	139	218	146	288
Zik1	179	76	77	121	60	197
Engase	225	111	85	139	109	211
Khdrbs3	2999	2011	1795	2736	1733	3226
6820408C15Rik	167	109	80	133	105	152
Fam109b	27	15	13	23	9	31
Tnk1	1	2	4	2	2	4
Zfp758	735	542	469	648	447	907
Eif4a3	1429	1008	914	1300	797	1776
Nanos1	25	16	11	20	8	32
Gm5141	81	82	57	65	68	120
Gpr155	1488	1042	783	1433	821	1422
Hnrnpa1	799	542	469	588	493	952
Lrr1	2	2	1	1	0	5
Rad51b	10	20	13	13	10	30
Tmem208	369	187	184	282	171	368
Ubap1	1757	1203	1051	1568	1075	1834
Ppp1r8	763	478	395	599	435	772
0610037L13Rik	892	552	505	714	475	1025
2010204K13Rik	9	5	2	6	3	9
4430402I18Rik	681	545	417	570	452	848
Acss3	47	29	35	44	33	46
Afg3l1	3091	1945	1778	2754	1768	3103
Akap8l	2222	1415	1381	1680	1420	2551
Arxes1	3	0	2	1	0	5
Atp5b	222536	136798	120043	193169	116643	224364
Atpaf1	323	211	214	282	249	287
Aup1	1915	1310	1091	1643	1071	2155
B430212C06Rik	76	54	37	49	60	69

Ccdc65	7	14	7	15	4	14
Cidec	19	41	10	41	22	13
Degs2	6	7	2	5	2	10
Fam26f	3	8	2	6	2	7
Fam65c	23	11	5	20	6	17
Fbxl18	119	66	54	92	71	96
Gabrb3	13	13	9	14	9	17
Gtf2ird2	485	318	226	393	271	473
H13	1600	1010	763	1379	804	1550
Insrr	6	8	2	3	3	13
Itgb1	24712	18374	15825	23152	16404	26920
Lrrc27	349	218	225	301	230	353
Lrrc49	295	241	155	273	189	303
Man1b1	1617	881	800	1304	820	1524
Ndfip2	1020	617	561	864	537	1073
Pcdhb5	54	33	22	49	25	47
Psmb10	1010	593	567	778	559	1103
Rae1	886	596	523	761	512	982
Sds	14	7	4	14	7	5
Sec63	3902	2772	2431	3273	2616	4333
Sh3bp5l	1170	620	527	906	617	989
Sh3glb1	19698	15035	13138	17379	13932	22827
Slc7a6os	450	302	259	360	247	538
Spg20	1872	1369	1148	1644	1265	1999
Stard6	19	7	12	10	14	17
Susd1	40	15	14	27	15	34
Tab1	883	554	477	761	484	896
Tbc1d9b	3620	2414	1959	3205	2100	3595
Tmsb15b1	5	1	3	1	2	7
Ywhah	1996	1359	1113	1929	1040	2097
Zcchc18	2	3	2	4	2	2
Zfp703	1215	677	449	1160	533	820
Zmym1	375	258	191	334	198	387
3110082I17Rik	112	96	56	73	81	142
Flrt3	48	24	20	27	25	50
Pcyox1	5234	3300	2855	4529	3113	4928
Prr15	29	22	15	19	27	24
Klhl21	4284	2514	2427	3930	2390	3843
Grin3b	11	10	9	8	11	15
Slc24a2	169	132	124	154	145	167
Spef1	316	192	180	281	171	321
Smg9	334	210	135	251	162	334
Ccm2	629	395	299	487	345	623
Brox	1477	815	881	1235	795	1546
Chmp1b	1995	1403	1221	1659	1341	2162

Dmxl1	3180	2185	1866	3065	1879	3110
Fzd9	51	21	20	45	15	41
Htr2b	11	8	12	10	13	11
Me3	3431	2045	1726	2874	1746	3365
Mir5113	96	73	70	94	55	126
Psd4	52	41	16	59	19	44
Rbms2	5660	3791	2979	5035	3194	5618
Scp2	6783	5011	4278	5973	4672	7235
Zfp959	241	171	146	159	159	317
Fn1	3179	2449	1590	3163	1479	3602
1700093K21Rik	3	3	1	1	2	5
Agpat2	1284	784	681	906	731	1422
Apol9b	11	3	4	9	0	12
Cnn2	1735	1132	935	1537	922	1821
Cnst	4187	2911	2456	3817	2798	3904
Creb1	2430	1604	1189	2171	1278	2327
Ier5l	94	56	40	83	43	85
Ifitm10	16	11	14	20	10	16
Itpka	5	6	2	5	2	8
Klc2	2735	1775	1314	2336	1603	2366
Man1c1	1198	695	682	1130	656	1053
Nans	362	216	191	312	171	388
Napg	1391	918	698	1146	748	1453
Nfatc2ip	298	136	157	211	133	324
Nudt9	2022	1312	1279	1634	1272	2305
Polr2f	1069	638	551	826	538	1183
Rab5a	2547	1854	1582	2297	1677	2703
Slc25a27	493	317	203	315	251	556
Snrnp70	3548	2482	1812	2596	2112	4018
Spag7	2784	2109	1694	2414	1716	3344
Sytl3	10	2	3	1	3	13
Trp63	13	22	14	10	21	24
Ubc	8428	4847	4374	6415	4254	9210
Vmn1r90	5	5	2	4	5	4
Zswim6	598	343	260	508	317	463
Smim12	908	479	530	782	439	938
Rad54b	40	24	17	25	20	46
Parva	3171	2118	1694	2867	1617	3430
Smarcb1	717	477	371	649	367	735
Igf1r	3371	2344	1620	2949	1957	3158
Ndufa13	10840	7179	6721	9331	6227	12714
Szrd1	2821	1707	1484	2526	1564	2500
1700021K19Rik	2382	1537	1207	1992	1406	2201
3110070M22Rik	17	14	9	12	7	27
4833419F23Rik	51	46	26	41	33	64

5830454E08Rik	22	15	11	15	13	26
6330418K02Rik	33	24	15	36	17	27
Aar2	1040	552	520	913	483	952
Acp5	12	13	9	21	4	15
Agtrap	2620	1714	1565	2268	1561	2805
AI427809	5	8	6	8	5	9
Aida	1493	1092	841	1369	860	1643
Ankra2	1064	704	617	859	663	1129
Ankrd26	274	219	168	222	212	294
Arf1	8092	4972	4643	7164	4418	8324
Aurka	36	24	18	20	15	55
BC005537	9725	6673	5540	7994	6370	9770
Birc2	1825	1166	1109	1613	1061	1949
Cachd1	255	130	98	220	109	191
Ccdc101	357	206	198	269	195	386
Ccdc130	267	188	149	183	179	308
Cep170	1423	976	681	1206	773	1447
Clybl	3501	2404	2292	3033	2120	4235
Cnot2	2311	1443	1209	1928	1306	2264
Coasy	573	376	287	438	350	574
Cops8	3413	1935	1814	2835	1761	3361
Csnk2a2	710	477	391	579	444	729
Cxxc5	1255	829	713	1260	661	1214
Cyb5b	3832	3095	2488	3871	2634	4045
Cyb5d1	97	72	73	107	75	85
D15Ertd621e	1443	981	833	1102	848	1729
D430042O09Rik	687	440	317	544	420	589
Dnajc1	1706	1188	937	1466	1046	1731
Dolk	808	477	414	756	404	726
Dsp	73098	60591	45168	73536	57228	62884
Eci2	5719	3718	3211	4227	3510	6338
Efemp1	319	194	296	203	207	563
Eif1ad	1110	713	648	1008	600	1187
Eogt	1006	666	580	934	565	1033
Fam118a	351	216	221	286	201	409
Fcer2a	2	4	6	3	4	7
Frs3	178	87	89	136	92	162
Gabpb2	1937	1263	1012	1510	1137	2011
Gid8	3474	2445	2003	3113	2159	3572
Gm10560	92	58	47	64	40	124
Gm8234	2	3	2	2	2	4
Gm8369	3	0	3	2	0	5
Got1	40930	25063	23748	35405	23084	41639
Gpr137	989	490	402	760	426	873
Haus5	152	96	85	110	96	162

Hells	82	38	35	51	26	99
Hist1h1e	33	12	14	31	19	11
Hoxa2	17	14	11	16	11	20
Hs3st4	5	2	1	4	1	4
Krtcap2	679	450	437	598	395	785
LOC100504703	71	48	46	61	36	92
Lrfn4	291	148	101	223	137	217
Lrrc58	4358	2779	2427	4011	2517	4019
Mcm3	258	163	120	195	129	279
Mrpl21	824	535	581	726	544	928
Mypop	147	78	64	123	79	110
Ndrg4	18734	17977	15648	25727	9684	28141
Ndufb9	16140	10808	10236	14034	9033	19790
Nprl3	715	425	346	602	387	643
Nsun6	234	142	130	146	130	296
Nudt13	1092	760	696	975	610	1336
Oaf	341	244	198	301	151	461
Oas3	21	19	9	25	4	29
Pcdhb4	50	36	13	44	27	35
Pde6d	1294	882	766	1235	714	1387
Phex	7	3	1	4	4	4
Plec	13646	8822	6238	12663	7995	10085
Prdm4	821	573	470	716	476	913
Prpf40a	1983	1543	1295	1786	1395	2225
Prrg2	195	116	92	152	104	187
Ptpdc1	584	353	299	430	336	604
Ptpru	266	149	89	161	163	201
Rab3il1	404	247	165	308	205	383
Rbm45	825	552	471	682	501	885
Rilp	1569	1081	739	1202	889	1673
Rin1	98	62	31	80	49	75
Rn45s	510692	377009	205031	54081	755901	101306
Ropn1l	16	7	1	6	5	15
Rusc2	2877	1768	1223	2394	1471	2533
Sars	2773	1722	1704	2364	1575	3061
Sec24d	2143	1380	929	2061	998	1827
Selplg	109	70	46	104	40	111
Sepn1	1192	697	538	980	573	1120
Sf3b5	514	242	249	386	241	478
Slc12a4	3154	1860	1457	2547	1580	2991
Slc35b1	934	507	509	768	432	1013
Smarca1	107	49	77	81	58	126
Smpdl3a	3331	2332	1936	3103	2034	3348
Snhg4	164	101	107	122	96	208
Soat2	8	2	4	2	3	11

Sox12	724	431	352	505	454	665
Stxbp4	472	339	264	443	325	398
Tbx5	1905	1330	965	1786	1123	1683
Tdrd6	12	3	4	1	2	19
Tlcd1	237	136	113	168	136	230
Tmed5	3129	1976	1708	2263	1874	3470
Tmem11	805	569	516	743	485	912
Tmem132c	6	2	1	5	1	4
Tmem175	480	338	234	380	281	503
Tnfsf9	17	13	6	12	7	22
Tspyl1	4515	2907	2519	3982	2604	4498
Twist1	68	42	37	68	27	73
Txnrd3	868	657	492	778	578	872
Ubl7	1836	1236	1017	1532	1120	1876
Unc50	778	467	468	685	423	828
Vac14	969	578	499	779	507	1001
Vnn3	5	3	1	6	0	4
Vps72	868	628	483	727	541	936
Wasf1	34	12	11	27	12	22
Wdfy3	3291	2279	1695	2999	1917	3115
Wdr25	172	110	94	153	99	167
Wibg	237	172	126	176	139	291
Zbtb1	714	488	421	604	471	727
Zcchc6	3180	2479	1796	2772	2032	3535
Zdhhc3	1241	735	680	1103	671	1182
Zfp426	651	463	392	607	406	676
Zfp64	310	209	137	254	150	332
Zfp821	273	221	164	241	178	322
Gm11110	6	1	3	2	4	5
Gm4944	337	231	254	251	242	444
Gna13	2161	1376	1320	1954	1315	2134
Haus1	522	394	385	431	372	691
Maged1	5108	3385	2688	4715	2904	4697
Moxd1	59	30	33	39	32	66
Mpp3	136	78	68	86	55	184
Rab17	29	15	21	26	19	27
Rhno1	268	182	153	213	157	311
Slc2a4	23213	13446	10969	19733	11764	20580
Snora17	13	8	4	11	3	14
Snora31	25	16	19	17	12	41
Spats2l	531	402	271	493	315	526
Tesk1	1218	742	646	1016	751	1073
Usp7	4734	3417	2712	3954	3053	5067
Zfp825	316	236	193	230	212	398
Ccdc84	253	183	131	195	153	284

Enox2	540	351	312	447	307	598
Gm561	570	362	409	510	307	751
Plekha1	3868	2676	2056	3263	2286	3997
Stk25	3224	2096	1632	2619	1909	3089
Xpc	867	649	466	687	517	1027
2310015B20Rik	11	8	4	8	8	9
Fam21	1884	1330	1068	1567	1160	2073
Map3k3	2093	1298	964	1845	1072	1869
Mrpl33	2196	1435	1413	1903	1246	2638
Psmd6	3090	1921	1770	2600	1733	3286
Rwdd1	649	464	481	585	457	773
Trpc4ap	5765	3757	2909	5116	3183	5445
Znhit6	529	335	328	387	374	556
Adamtsl3	680	427	233	567	308	582
U2af1l4	1101	721	543	860	609	1156
Fdps	347	219	163	275	165	381
Gpr111	20	8	6	15	10	11
Bet1	626	360	362	544	283	722
Brf2	189	112	74	146	94	168
Chp2	42	23	16	22	21	47
1700026D08Rik	2	0	0	0	2	0
4930426L09Rik	7	7	2	8	2	8
4930427A07Rik	18	10	9	16	6	20
A230001M10Rik	0	3	1	1	0	4
AA413626	4	6	0	5	3	3
Adamts14	42	19	19	30	10	52
Adamtsl4	3283	2034	1325	2607	1696	2901
Ank3	5313	4220	3130	5231	3645	5093
B130024G19Rik	72	45	26	36	43	76
Bag4	516	293	251	392	283	486
BC031181	1104	678	675	980	626	1154
Carm1	2541	1664	1396	2117	1563	2490
Cd302	462	296	274	440	224	513
Chchd6	204	107	122	165	104	218
Chrna10	0	3	1	1	0	4
Cmtr1	2852	1796	1493	2566	1611	2567
Cnga4	2	0	0	0	2	0
Dgcr8	789	565	436	728	467	799
Etv3	1731	1125	957	1431	1040	1753
Fbp2	1175	573	742	709	618	1531
Fbxo36	41	41	32	34	35	61
G730013B05Rik	0	2	3	3	1	2
Ifnlr1	275	186	185	174	177	395
Itgb7	50	22	16	46	18	30
Klf14	2	5	2	2	5	3

Ltb	5	4	2	5	4	3
Mrps17	1579	998	963	1305	941	1741
Nes	2137	1269	1282	1810	1168	2324
Ntan1	822	487	455	696	411	888
Numa1	5694	3897	2642	4815	3522	4850
Pggt1b	797	546	436	720	491	754
Psmd3	4182	2695	2178	3469	2377	4170
Rhpn2	440	253	189	376	181	425
Rps3a1	2912	1655	1627	2487	1415	3117
Serpinf1	1040	925	665	1176	553	1345
Sh2d4a	475	327	349	378	405	481
Slc7a1	2444	1802	1302	2778	1341	2038
Smu1	1906	1317	1113	1683	1092	2120
Snx20	21	14	8	19	6	24
Son	18875	12371	9757	14152	11346	19885
Tex264	1036	618	529	924	514	989
Thap7	328	175	135	239	179	267
Timm44	4256	2976	2588	3597	2675	4760
Tnfrsf11a	106	69	45	97	52	94
Uba1	10493	7071	5222	9725	5777	9659
Vhl	581	356	310	516	291	590
Zfp219	1122	644	537	922	570	1041
Zfp882	210	147	111	216	119	181
Zfp940	54	44	32	40	40	65
2310002D06Rik	14	6	7	10	6	14
Pqlc1	492	234	226	343	254	434
Zfp101	385	232	240	283	220	474
2310069B03Rik	25	24	31	17	40	30
4632427E13Rik	13	9	7	11	10	11
Armc6	220	115	101	193	107	174
B930059L03Rik	16	19	9	19	8	24
Ccnt1	417	319	238	391	259	439
Chd4	9688	7420	5671	8407	6555	10414
Csrnp2	182	70	92	142	72	168
Dynlt1b	2	1	5	5	3	1
Klf5	10	8	1	8	7	5
Lamtor2	1015	724	604	865	633	1139
Med25	2117	1312	916	1763	1150	1775
Mettl10	217	160	165	186	146	294
Mir7012	1	0	2	0	3	0
Mslnl	2	3	3	2	4	3
Msmo1	409	235	271	359	204	494
Pilra	19	21	16	22	13	30
Prdx3	11553	6231	6398	9707	5831	11552
Rbm12b2	412	230	196	318	202	411

Tbc1d23	1391	967	805	1117	866	1567
Zdhhc8	1405	831	674	1285	670	1261
Zfp407	590	419	328	566	382	519
Zfp85	144	119	83	108	110	165
Arl13b	715	432	332	546	334	783
Mir23b	12	4	1	6	4	8
Fads6	15	8	11	15	12	10
Eif3i	2686	1621	1404	2201	1393	2801
2010111I01Rik	2543	1559	1380	2091	1343	2719
Dennd4a	680	477	417	598	406	776
Fbf1	901	658	426	730	559	889
Gm5176	2	2	3	4	1	3
Il7	20	26	16	25	14	33
Lhfpl2	258	149	172	253	138	264
Plagl2	479	322	266	411	280	502
Snx8	424	241	204	336	204	429
Tsen2	280	209	135	222	179	287
Uckl1	1265	781	703	1118	659	1317
Zfp775	565	330	304	464	331	520
Kmo	26	21	22	32	11	38
Ogfr	1675	1018	804	1376	801	1741
Ppp2r5a	5299	3747	2902	4654	3290	5290
Cfi	8	2	0	8	1	1
Paqr7	921	613	513	824	504	975
Chic2	452	303	253	357	270	503
Fcgr4	37	12	12	26	7	35
Irf8	221	135	98	198	85	231
Atp6v1c1	2473	1712	1569	2295	1485	2734
Cherp	1670	1160	781	1440	930	1613
Echs1	13361	7765	7221	11250	7156	13125
Metap2	5676	3975	3538	4723	3677	6423
Odf3l1	7	2	2	3	4	5
Zc3h12d	15	16	8	14	10	20
2310010J17Rik	74	28	55	46	40	94
Acot12	2	2	3	3	1	4
Adat1	139	112	77	139	85	143
Adcy5	4106	2666	1937	3861	2493	2916
Alg6	431	292	215	294	237	526
Amica1	37	8	7	25	7	23
Ankrd46	2020	1280	1079	1700	1075	2137
Arid5a	363	227	189	324	177	374
Asb12	299	175	182	239	159	346
Atrip	382	237	208	326	209	387
Bank1	18	22	41	33	24	43
Bcl7b	1344	918	664	1109	758	1379

Capn5	152	89	74	121	71	161
Cask	778	543	443	710	422	866
Chchd10	13705	8828	7362	11581	7574	14240
Chd3	1769	1461	1286	1955	1036	2293
Chtop	4055	2445	2119	3396	2170	4012
Commd3	2541	1793	1721	2348	1604	2941
Commd9	559	312	331	462	299	590
Cstad	14	13	15	9	9	33
Cyp27a1	1325	932	982	1122	736	1986
Dbp	3662	2535	1631	2938	2014	3670
Ddx50	2187	1659	1479	1894	1470	2705
Degs1	3141	1982	1787	2914	1737	3060
Dhdds	1835	1222	1064	1637	1144	1782
Dynlt3	3378	2423	2175	3072	2129	3837
Eif4h	5997	3479	3187	5016	3205	5828
Fanci	32	22	14	30	6	44
Frem1	32	11	10	24	9	25
Gata6	3585	2597	1794	3077	2362	3194
Ggnbp2	4472	2885	2605	3613	2597	5017
Gm6936	7	3	0	5	0	6
Gm9054	14	2	5	7	4	12
Gm9079	23	21	22	19	23	33
Gne	1026	741	573	999	602	1009
Hist1h2bg	1	4	2	5	2	1
Hsbp1	4082	3102	2541	3716	2514	4851
Hsd17b4	5406	3412	3280	4452	3468	5478
Iglon5	9	7	5	8	3	13
Il33	385	306	195	362	217	416
Klf7	353	207	166	237	209	346
Lin52	696	374	347	564	340	668
Lmnb2	267	135	147	235	111	276
Lrrc52	46	44	32	58	41	34
Malsu1	462	313	299	365	269	599
Man2c1	1606	931	661	1223	791	1476
Med7	711	461	488	643	410	851
Mir3064	1762	1140	979	1329	1054	1954
Mir7035	1	3	3	5	2	1
Mir7219	157	85	80	107	95	150
Mkln1	3188	2107	1965	2844	1931	3376
Mrpl43	1513	865	921	1230	879	1585
Mrps23	4907	3348	3519	4638	2942	6040
Nap1l3	105	66	49	92	59	90
Nfxl1	429	272	202	333	227	442
Ngly1	1931	1220	1160	1591	1142	2110
Nos1ap	120	108	62	134	80	106

Odf4	1	2	2	0	1	5
Pelp1	881	653	473	805	484	980
Phf6	585	377	286	463	340	568
Pla2g16	5889	4266	3894	5476	3888	6495
Plekha8	739	493	453	617	409	902
Polm	149	101	64	85	78	191
Ppcs	275	174	180	180	167	376
Prcc	1580	1058	770	1410	839	1530
Psd2	3	8	3	2	6	8
Ptar1	152	80	69	144	66	120
Rab11a	4253	2540	2493	3590	2272	4634
Rorb	67	32	40	39	50	61
Rpl28	15	8	5	13	5	13
Rps6kb2	775	506	359	568	425	826
Sema6c	339	197	107	259	146	291
Sf3a3	752	564	505	757	431	909
Slc38a6	209	110	138	162	121	232
Smo	470	302	230	382	216	540
Snord66	3	2	2	5	0	3
Sowahc	162	119	103	173	91	172
Spg7	4523	2884	2340	3660	2648	4431
Stk11	4107	2727	2128	3238	2463	4187
Supt5	4381	2907	2001	3516	2406	4286
Taf1	2115	1467	1178	1769	1318	2201
Tbxas1	54	41	22	59	26	44
Ten1	145	100	98	162	92	129
Tmem63c	12	3	4	4	4	13
Ttbk2	1436	953	816	1288	911	1322
Ubap1l	3	4	2	2	4	4
Uvrag	1307	888	757	1179	834	1244
Wdr37	2599	1694	1481	2360	1482	2607
Zfp105	80	77	86	87	64	139
Zfp119b	164	93	95	112	88	199
Ccdc173	94	70	64	57	72	129
Ccdc55	1092	850	685	863	728	1397
Cd4	42	17	15	29	19	32
Cycs	2587	1368	1548	2107	1173	3066
D3Ertd254e	626	446	393	547	414	680
Dpp8	6130	4267	3642	5484	3972	6105
Efcab4b	49	17	17	21	26	41
Hnrnpul1	3621	2691	1923	3325	2270	3484
Ilf3	1491	1070	820	1354	963	1399
Kdm5a	2587	1723	1474	2253	1540	2657
Lpar3	100	91	50	142	33	106
Mccc1os	93	61	49	87	46	95

Nfrkb	819	577	438	646	488	919
Peg3	689	489	290	459	442	686
Psph	201	104	116	147	126	188
Rita1	258	185	152	250	146	275
Tmem205	772	405	376	538	374	819
Tmem240	9	4	6	10	5	6
Traf3ip3	16	12	8	17	9	14
Atrn	2682	1841	1473	2483	1644	2475
Gm14325	628	429	409	543	437	650
Lrig3	114	84	61	100	63	129
Prkar1a	21964	14673	14790	21104	14276	22292
Josd2	1255	633	621	945	684	1094

WT314.CPM	WT361.CPM	WT366.CPM	TG363.CPM	TG364.CPM	TG365.CPM	WT314.TPM
105.374888	100.161702	132.110235	734.551388	941.334524	700.989709	96.3404698
0.13142291	0.04008071	0.23667187	2.96938409	5.75097702	2.29056927	0.0500912
30.6215376	26.0524636	26.9332583	52.4900499	68.7835108	54.2688718	44.461574
25.2069138	25.0504458	25.1345521	50.696047	48.6094486	76.9731958	46.2297548
66.473707	67.936809	68.1141628	103.464477	119.903307	112.665803	85.4611738
33.6968337	32.9463463	31.8086987	51.9332904	50.7090116	62.6760162	12.3897927
4.31067139	3.48702205	4.82810605	7.60904672	14.1035865	11.3018198	2.81762267
30.5163993	29.6196471	33.0393924	45.2831073	52.3521479	57.8683378	24.0840399
25.4960442	25.0905265	23.2411772	34.7974698	50.5264409	53.211686	68.2471235
32.4088892	31.7038442	35.9267892	52.7993608	51.074153	69.6735794	6.99582087
38.9800346	47.2952416	38.4355109	87.1019332	61.6176109	73.3737298	25.1961439
12.1171921	10.5412276	11.3129152	19.7340317	18.5309259	21.2444007	7.42996627
36.1675843	38.0365969	43.1216139	58.3050938	64.3105287	66.2754822	27.0187929
484.976816	465.697807	452.800613	787.227025	757.440187	861.832979	429.902172
11.0395243	7.8558198	8.47285278	16.3316125	16.7508616	19.5075954	6.17348
4.04782557	5.37081558	5.91679663	8.56791033	12.1409515	12.8120852	1.48141972
10.6715401	8.73759549	7.99950905	20.940344	20.1740622	14.1713241	18.9056306
0.63082996	0.80161427	0.42600936	1.97958939	3.56012863	1.76197636	0.18976638
32.9608654	28.7378714	32.9447237	42.3137232	61.9371096	56.8614942	62.031602
42.4233148	47.9766138	51.7364698	85.1532749	129.533911	68.9436178	24.7949433
12.0120538	11.4230033	12.306937	16.7027855	23.6429055	28.544017	12.5269979
26.9942654	25.2508494	33.370733	43.2416557	43.7256824	58.1703909	21.9778285
1.73478239	2.56516565	0.99402183	4.7015248	4.47298212	4.90836271	3.5546629
64.6600709	54.790335	54.9078728	88.865005	79.5551821	104.434856	40.940845
93.4942569	113.789145	216.128747	68.5742137	61.8914669	55.8294795	65.7677675
85.6614516	69.1793111	91.1660025	108.351588	161.803282	177.959612	50.415392
22.2893252	23.3670558	27.9746145	31.3950505	53.2650014	50.9714589	12.7471731
4.31067139	3.88782919	5.06477792	6.83576961	9.44803367	10.4963449	4.96533035
86.6602657	83.6083679	87.2845839	116.022497	136.060813	138.164118	159.294105
261.189888	235.47419	232.648444	352.088532	440.862595	370.367431	138.345851
25.9165975	23.487298	22.7678334	31.5187748	40.8501939	44.5276597	17.2254236
0.05256916	0.08016143	0	0.24744867	0.3651414	1.43475218	0.16280274
95.7021618	99.6406532	109.437071	149.613654	145.919631	157.696884	99.7622883
1.49822115	1.28258282	1.84604055	3.3405571	3.14934456	3.82600581	1.35809698
10.5664018	12.2646983	14.72099	23.9406592	16.979075	27.5371734	6.22476463
19.6345825	15.9120432	16.0463525	8.35139274	7.57668401	12.0066103	24.6725173
13.9571129	13.6675232	12.685612	19.5175141	21.8628412	22.1505599	9.09133687
15.6656107	14.7897832	17.0877087	20.9094129	29.3482399	28.820899	4.03060487
25.2857675	21.523343	25.371224	35.8181955	34.8710035	39.7954946	11.961217
74.4642198	152.747598	87.9945995	56.913195	56.82513	50.6442348	289.177726
74.4642198	152.747598	87.9945995	56.913195	56.82513	50.6442348	289.177726
1.31422908	0.84169498	1.27802807	2.78379758	2.64727513	2.69330672	2.43403041
24.0503922	26.8941586	32.4240455	36.8079902	49.8874435	47.6488749	13.5111323
17.8209464	15.0703482	18.9337492	25.5800067	24.8752577	34.9878163	12.9185785

38.8748963	37.1548212	40.849564	51.1290822	58.4682663	71.7879511	16.6801432
47.0494011	40.120794	50.1271011	87.6586927	54.7712097	97.0848973	81.2921463
28.5976248	27.6957729	30.9093456	40.3341338	40.3024818	46.8434	21.2018505
38.5069121	36.1928841	37.5834922	45.4996249	65.0408115	59.9575384	59.6642667
41.3982161	36.1928841	34.5540923	52.4281878	52.762932	55.4519131	17.8410527
49.4150135	44.168946	39.9028765	62.9447564	61.6632536	72.593426	32.7723819
102.352161	88.618457	95.3787617	127.466998	137.293166	160.037796	64.5557573
14.0359666	12.1043754	13.2536245	7.54718455	8.76339355	8.25611779	12.1471463
17.0061243	13.3468775	14.9103275	22.0847941	22.9582654	24.4411292	15.6298584
20.4494045	22.0043116	22.8151678	30.9929464	30.0785226	32.1938252	9.34633119
11.9069155	10.4610662	11.928262	15.4036799	19.7176355	19.8096485	6.83027169
4.52094804	4.44895917	3.92875296	6.99042504	6.84640121	9.43915906	1.88525965
48.3110611	41.8442646	42.0329233	62.6045145	72.3436394	58.9255236	27.3666171
65.4486083	57.1550971	55.1445446	81.3796826	85.3974444	85.8334197	37.8187618
103.350975	100.402187	93.6273899	127.559791	146.010916	165.273382	55.4264471
48.8367527	53.5478329	53.2985041	66.5946243	75.2647706	105.592726	87.2135481
6.57114541	5.81170342	7.10015596	12.867331	11.9583808	8.88539506	1.95390992
150.295238	138.919752	157.576128	187.13306	327.531834	232.052286	209.400856
40.9513782	36.2730455	30.6726737	47.015248	54.3147829	60.8888687	55.8786592
33.1974266	26.693755	24.5192052	43.7674842	40.0286257	41.9350373	32.6184122
75.4893185	62.0850248	57.8899383	87.9061414	97.6296812	95.4739475	109.608275
57.7472259	49.0187123	59.8306476	72.6261858	78.5054005	100.130599	76.2042406
80.9302269	72.9068174	95.0947555	109.032072	122.00287	162.479391	35.9218994
104.92805	119.600848	120.134639	147.108237	178.371573	181.684934	84.7520227
6.07173836	12.6655054	8.33084966	4.67059372	3.9709127	4.6818229	10.0985291
134.366781	116.033665	127.045457	162.140744	201.375481	194.899756	67.8509412
21.7636336	21.4832623	21.7738116	27.8379758	30.9000908	34.4340523	16.4188298
21.0013807	21.7237466	17.135043	26.9100433	28.207173	36.5484239	9.13284031
107.819354	75.6323059	82.2671404	66.6255554	54.725567	51.7265917	42.4847247
3.75869518	3.04613421	3.31340611	5.44387082	6.02483306	6.56965471	3.68278451
19.9237129	15.791801	20.306446	26.5079392	25.3773272	38.788651	18.2770038
2.99644231	5.77162271	2.84006238	1.48469204	2.05392036	1.18304127	3.6131136
5.46719298	4.8497663	4.16542483	8.59884142	6.52690249	12.2834923	3.52089322
52.8582937	45.3713674	52.2571479	62.078686	73.1195649	94.3412484	162.260407
44.3158047	41.4835382	52.4938197	55.6450205	76.1776241	76.8976825	21.7845575
44.3420892	44.5697531	36.7314735	66.0687959	53.4019294	61.9208835	30.4394079
42.5284531	40.2410361	34.8854329	56.6966774	50.8459396	56.3077302	23.5991973
1.2879445	2.00403566	1.32536245	0.43303518	0.91285349	0.35239527	1.72471105
22.7887323	21.242778	16.8983712	36.5605416	25.8337539	31.1366394	12.6829484
18.7671913	20.3610023	21.7264772	27.6523893	28.0246023	29.9535981	19.2840225
4.88893219	4.08823275	4.40209669	6.52645877	7.89618273	8.18060452	7.7770595
10.3298406	8.73759549	10.7922371	14.0427122	14.879512	16.9653152	15.8053057
0.2891304	0.52104927	0.56801248	1.02072578	1.18670954	1.35923891	0.27772985
21.8424873	21.4431816	29.0633051	28.8277705	43.1779703	44.049409	17.8428001
24.8389297	21.8439887	23.477849	34.6118832	30.2610933	32.8482735	17.6094447

55.9073052	56.3935636	58.363282	71.2961491	79.7377528	82.1332694	34.7689797
267.62961	258.240036	264.64648	343.799001	369.61438	413.51068	141.91043
7.3071137	7.33477053	6.62681223	12.1559161	10.0870311	10.9242534	9.85522005
16.4541481	16.1124467	14.9103275	23.1673821	23.2321214	21.5212827	17.1961701
3.39071103	4.04815204	3.78674985	5.50573299	6.11611841	8.35680216	5.35892361
29.5701544	24.4893158	28.2586207	31.1476018	45.3688187	55.4519131	28.6630225
36.0887306	34.2289291	35.0747704	42.4683786	51.4392944	51.6007362	17.2684579
317.728023	316.95828	317.092965	403.09389	459.941233	518.675498	195.018571
71.0472242	72.546091	77.8650437	93.2262879	99.3184602	103.704894	47.1701618
453.067334	954.241621	581.502773	366.935452	403.526887	338.853225	1756.67384
453.067334	954.241621	581.502773	366.935452	403.526887	338.853225	1734.68106
169.246421	203.770346	149.245278	112.001456	126.156353	120.645038	67.5266324
2.8650194	2.92589207	3.50274361	5.53666408	6.11611841	4.63148071	8.44514709
23.9715385	21.6435852	22.2471553	28.1782177	31.2195895	38.3104002	18.4834731
39.0326037	35.0706241	43.8316295	51.2218755	54.7712097	55.3512287	19.9157495
159.652549	165.533346	148.913938	195.762832	259.615534	219.466741	52.9054889
0.68339912	1.00201783	1.13602495	2.22703806	3.33191526	1.48509436	0.79504716
133.394252	125.29231	116.915901	160.532327	177.595647	188.632155	198.394193
50.6241043	49.8203266	48.2337262	112.187042	49.2028034	103.956605	123.979422
22.1053332	17.9962403	17.4190493	23.4457618	33.684294	29.4501763	13.2069004
32.1723279	71.2635082	44.0683013	30.1578071	29.5308106	24.2397605	39.7735574
4.20553306	4.40887846	2.88739676	7.39252913	6.11611841	6.39345707	1.87177248
1.23537534	1.96395495	1.37269682	2.13424481	3.788342	4.22874326	0.65647978
17.0586935	19.1585809	15.5256744	25.2706958	22.3192679	26.9834094	23.2897863
11.0658089	6.97404411	11.928262	4.26848962	4.29041142	7.27444525	16.8786048
9.04189609	9.53920976	13.6322994	14.3829542	17.3442164	21.068203	6.13637633
9.19960358	8.57727264	6.24813724	12.8364	13.6471597	11.7297283	5.42610395
2.20790486	2.04411638	2.27204991	0.95886361	1.36928024	0.95650145	2.69550649
12.6691684	11.3027611	9.98755272	15.4964732	16.6139336	18.6517783	10.8150782
4.07411016	4.16839418	6.34280599	6.2790101	8.94596425	11.7297283	3.23203564
26.3371508	26.6135936	30.8620112	33.4983642	40.4394098	45.6100166	19.7171657
1.10395243	0.6012107	0.66268122	1.7630718	1.55185094	2.16471381	0.71908879
5.46719298	5.01008916	5.96413101	7.26880479	12.8712343	8.40714434	5.95276962
32.9082962	40.681924	29.5839832	48.4071468	52.1239345	44.6535151	19.1833377
102.483584	99.3200075	108.963727	129.23007	137.156238	169.401441	130.386541
7.25454453	6.89388268	9.04086526	12.496158	16.2031495	9.79155433	3.75794093
131.896031	134.430712	162.924912	183.452261	206.943887	222.462101	48.6129775
110.763227	114.029629	97.6508116	135.014183	152.811675	155.834223	116.50475
5.70375422	6.61331769	5.86946226	3.74266119	4.1534834	3.09604417	125.717451
0.31541498	0.12024214	0.18933749	0.49489735	0.86721082	0.75513273	0.43795971
10.1195639	4.80968559	5.63279039	9.99074021	14.6969413	14.146153	25.2377442
10.1195639	4.80968559	5.63279039	9.99074021	14.6969413	14.146153	24.4888497
10.1458485	9.13840262	11.3602495	13.7952636	15.290296	15.782274	6.96870696
19.3454521	18.8780159	14.2949807	24.1262457	26.1076099	25.8003681	10.482838
0.15770749	0.20040357	0.18933749	0.34024193	1.87134966	0.50342182	0.15089656

2.91758856	5.65138057	3.171403	1.39189879	1.87134966	2.24022708	1.56881017
0.10513833	0.16032285	0.04733437	0	0	0	4.21685793
499.380767	455.116499	443.097066	282.338937	372.535511	319.924565	216.587534
468.654091	513.433937	470.409	374.884741	352.178878	282.646179	176.535073
0.36798414	0.48096856	0.5206781	0.80420819	1.00413884	1.78714745	0.11730282
24.8126451	27.0945622	28.8266332	36.4368172	33.684294	39.5941259	27.4190997
0.18399207	0.16032285	0.09466875	0	0	0.02517109	1.17226454
573.792417	509.826673	554.19084	825.426914	811.207258	704.614346	727.369756
20.5019737	15.1104289	17.0403743	20.1361358	28.0246023	36.1456864	20.5151432
55.7495977	58.2773571	130.169526	48.6236644	41.6261194	42.4132881	47.8580084
5.59861589	4.80968559	3.59741235	7.08321829	8.99160692	7.32478743	1.32444486
307.161621	318.801993	294.277797	412.558801	434.929048	413.561022	140.992979
262.661824	262.40843	249.215474	327.7767	386.228314	349.12303	102.328134
112.550579	103.96937	113.555161	120.012607	175.724298	201.066674	100.791702
1.13023701	0.56112999	1.37269682	1.67027855	2.28213374	3.0205309	0.85477731
146.483974	125.933601	135.802316	165.17199	221.869042	191.476488	107.429069
47.8379386	47.7762102	46.3876856	52.7065675	73.2564929	73.8771516	39.2352973
26.2057279	20.2006795	20.9691273	31.9827411	28.0246023	34.7864475	54.3801205
1.10395243	2.48500422	0.94668746	0.5876906	0.5477121	0.57893509	1.31302747
39.3743033	40.6017625	41.8435858	49.5206658	61.2068268	51.827276	13.9733117
45.4986108	45.0507217	47.1450356	56.20178	67.2773026	60.7378422	16.7820837
15.2450574	15.6715589	14.1529775	21.1568616	23.5516202	18.9034892	13.5236071
600.602691	616.040563	596.271097	755.213352	894.915923	930.575228	172.163662
70.7843784	59.7202628	57.5112633	74.1108778	83.5260948	105.69341	65.747523
27.3622495	25.6917372	24.0458615	32.9416047	33.8668646	35.7177779	19.4886969
27.5725261	25.2107686	20.6377867	41.3857907	30.03288	33.5782352	12.0741239
14.7982195	17.0343031	14.9576619	21.68269	21.2694864	21.4961116	9.08757227
17.1901164	20.0403566	25.4185583	14.1973677	15.3815814	12.4848611	8.23029991
15.3239111	14.9100253	12.9696182	21.5589657	22.1823399	17.9469878	9.57625277
76.146433	78.8788437	68.2088316	94.8656353	90.78328	109.519416	91.7270411
0.44683789	0.36072642	0.56801248	0.21651759	0.04564267	0.10068436	0.25626358
7.93794366	6.93396339	6.48480911	9.09373876	11.8214528	11.8304127	5.4293372
49.4150135	54.0688822	52.5411541	63.6871024	66.9578038	77.3507621	14.5799948
0.13142291	0	0.04733437	4.48500721	0.13692802	0	0.12486812
0.63082996	0	0	0	0	0	3.13056795
22.1316177	21.2026973	19.0757523	29.9412895	24.2362603	31.9924565	45.3488812
50.702958	50.5818601	66.4101254	68.295834	77.8207604	88.1995023	49.7986206
4.46837888	4.80968559	4.59143419	6.83576961	7.48539866	6.79619453	6.17987799
0	0	0	0.06186217	0.13692802	0.10068436	0
0.89367578	0.52104927	0.5206781	1.1753812	1.32363757	2.03885836	1.19152089
0.23656123	0.12024214	0.28400624	0.64955277	0.7302828	0.50342182	0.18562019
0.21027665	0.16032285	0.23667187	0.03093108	0	0.05034218	2.59826599
43.6586901	43.4074125	34.9801017	54.9026745	54.9994231	52.2048424	9.99476611
33.0922883	33.828122	34.0334142	40.4887893	45.5970321	46.289636	16.7071023
0.13142291	0.08016143	0.04733437	0	0	0	0.31430637

12.5903146	10.621389	8.85152776	6.71204528	8.03311075	6.46897034	2.71991991
23.9715385	23.4071365	29.5366488	17.8781667	19.2155661	18.8279759	13.5759398
0.42055331	0	0.94668746	0.09279325	0	0	0.78125538
1.83992072	1.60322853	3.31340611	4.39221396	3.14934456	5.73900871	1.06418341
14.2199587	11.5833261	16.2830243	17.9709599	20.0827769	23.0567192	6.56541463
43.5009826	46.6138695	47.7603824	36.6842659	35.6012863	33.7292617	46.1727648
18.6357684	16.352931	17.3243805	25.4872134	20.630489	26.5303297	17.5091099
58.7986091	63.6481727	86.148559	115.744117	103.335016	81.3529656	21.6910352
11.8280617	11.463084	10.6975683	14.7231961	17.4355017	15.7571029	15.5414541
0	0	0	0.06186217	0.1825707	0.07551327	0
310.473478	331.868306	337.541414	247.881709	261.213027	215.892446	78.9518001
22.1841869	17.2747874	15.8096806	29.9722206	21.2694864	30.0291114	30.8732528
22.052764	17.194626	15.6676775	29.755703	21.1782011	29.7522294	32.9186351
14.824504	13.2266354	12.4962745	18.8988925	21.9997692	16.8394598	16.0483164
64.2658021	49.1389545	56.1859008	65.4192431	80.1028942	91.2955465	96.6979206
0.49940705	0.48096856	0.56801248	1.23724337	0.86721082	1.18304127	0.42691635
0.23656123	0.68137213	0.37867498	0.77327711	1.59749362	1.03201472	0.32297084
157.760059	145.332666	160.368856	123.662475	120.724875	99.9795728	87.4821202
17.531816	21.1626166	26.8859239	16.2078881	11.1368126	15.530563	5.01553141
11.7754926	10.4610662	14.5789869	15.6820597	17.4811444	20.7409788	18.176387
16.0598794	13.5472811	13.6322994	21.7136211	17.3898591	21.9491912	15.8689935
233.328231	252.388251	262.279761	332.230776	364.411115	309.453391	376.304535
33.1448575	31.0625528	28.6372957	24.4664876	24.7383297	19.8599907	26.0995276
21.553357	21.0824552	22.7204991	27.8070447	26.1988953	34.7612764	12.337812
4.12667932	3.84774847	3.21873737	6.83576961	4.92940887	6.24243053	4.36876334
24.7075067	23.8480244	26.8385895	27.899838	38.8419162	38.6879666	21.6119622
1.02509868	1.12225997	1.56203431	0.74234602	0.41078407	0.47825073	0.54544938
61.006514	60.0809892	61.6766881	70.9868383	79.3269687	91.3962308	18.3768457
20.8436732	20.3209216	15.7623462	28.5493907	22.3649106	29.4753474	12.7266077
0.2891304	0.48096856	0.37867498	0.43303518	0.59335477	4.02737453	0.42664531
5.23063175	5.61129986	4.9227748	6.30994118	9.0372496	9.16227706	9.32733935
6.91284497	6.17242984	7.28949345	8.78442792	9.99574577	11.0501089	20.1342416
0.05256916	0.12024214	0.09466875	0	0	0	0.05867435
0.31541498	0.44088785	0.33134061	0.09279325	0.1825707	0.05034218	0.14840096
1.18280617	1.08217926	0.94668746	3.12403951	1.78006431	1.68646309	1.26478092
7.99051282	8.17646551	6.57947786	11.6919498	10.6347432	10.6725425	11.5951087
372.636514	359.684321	356.238492	447.696513	486.550913	549.057004	76.5347146
15.3501957	11.7837297	11.7389245	10.0216713	8.58082285	7.82820925	10.7515994
70.1535484	69.3797147	57.6532664	87.6586927	81.5634597	86.0347885	36.2406262
11.1709472	9.33880619	9.94021834	15.5274043	13.4189464	13.8944421	9.35015752
0.02628458	0.20040357	0.09466875	0	0	0	0.02555925
16.2175869	15.8719625	16.6143649	11.1661214	13.5558744	9.3384747	22.9614991
19.0037525	18.4772088	19.6437648	26.9100433	21.8628412	28.6195303	12.8223997
0.55197621	0.40080713	0.66268122	0.77327711	1.41492292	1.48509436	0.55642717
125.903146	119.841333	122.31202	144.973992	165.819837	177.556875	33.5180554

30.5163993	30.3811807	31.8560331	43.1179314	35.8294997	42.4636302	16.8457861
43.3169906	43.327251	40.7075608	45.0047275	67.9163	64.9917565	29.1709533
6.20316127	6.85380197	9.32487149	7.11414937	16.3400776	17.6197636	1.95020196
33.801972	33.4273149	33.2760643	48.2215603	38.5680601	44.4773175	46.4861772
106.636548	102.80703	100.064865	129.013552	131.952973	139.624041	56.2150048
5.54604673	7.77565837	6.1534685	8.19673732	11.7758101	9.8922387	6.83222286
0.47312247	0.20040357	0.23667187	0.61862168	0.95849617	0.85581709	0.7185149
5.38833924	4.56920131	3.171403	8.25859949	5.84226237	7.37512961	5.77187044
57.1163959	44.730076	41.748917	56.7894706	65.3603102	78.710001	42.7076076
7.51739035	8.05622337	8.66219027	9.86701587	11.8214528	13.6175601	19.4622496
35.5367544	29.21884	25.8919021	39.282477	39.2070576	44.0997511	22.2816957
34.3802328	40.2811168	34.8854329	37.3338187	58.6051944	60.8636976	66.9694785
57.4580955	62.4056705	69.155519	87.5349684	67.8250146	105.718582	158.662849
6.41343792	6.73355983	6.62681223	8.25859949	8.62646552	14.8006014	5.40135227
0.81482203	1.08217926	1.04135621	1.82493397	1.59749362	1.93817399	1.02284227
25.2857675	24.3690737	21.9158147	11.3517079	21.1782011	15.1529967	13.1344828
32.4877429	33.9082834	28.3059551	25.3634891	23.7798335	23.283259	32.6958268
0.13142291	0.08016143	0.04733437	0.24744867	0.45642675	0.35239527	0.16421625
0.91996036	0.32064571	1.08869058	1.26817445	1.96263501	2.01368727	0.48021078
15.0084961	14.4691375	12.0702651	21.4043103	15.5185094	22.8805216	23.5229626
23.5247006	31.864167	26.2705771	23.4457618	15.4272241	17.6701058	10.4893092
14.955927	15.9521239	12.6382776	18.3112019	23.2777641	19.3817399	6.19342086
0.73596829	0.80161427	0	0	0.1825707	0.05034218	5.54030257
21.3167957	21.3630202	21.2531335	29.2917368	29.0743838	25.3221174	8.12859174
17.3478239	18.3569667	20.306446	26.074904	22.593124	26.2031056	13.5469757
6.46600709	5.93194556	6.39014036	8.53697925	7.80489738	11.4276752	3.48448073
84.3209379	76.9950502	79.4744124	94.4635312	104.704296	106.473714	35.5194772
16.4015789	11.8238104	9.56154336	18.2802708	17.0247177	21.3954272	13.6025831
6.88656039	5.65138057	6.95815284	8.01115081	9.95010309	10.9242534	10.8280565
0	0	0	0.18558651	0.09128535	0.02517109	0
6.07173836	8.13638479	7.24215908	12.5270891	15.6097948	7.55132725	5.39786928
31.6466363	31.1026335	35.4061111	35.4160914	46.4642429	51.7769338	32.1268582
9.35731107	7.37485124	7.24215908	10.2072578	12.3691649	12.3841767	7.07238853
0.39426872	0.32064571	0.18933749	0.55675952	0.63899745	1.18304127	0.56674868
248.757281	569.025886	228.625022	201.361358	217.076561	179.268509	544.366938
248.757281	569.025886	228.625022	201.361358	217.076561	179.268509	540.499322
3.39071103	3.16637635	3.45540923	4.17569637	5.43147829	6.29277271	2.05795429
49.5464364	50.3413759	50.1744354	37.2100943	35.1905022	43.772527	30.868055
8.75276569	9.25864476	7.95217467	9.99074021	13.327661	14.6495749	2.84650522
11.1709472	11.6234068	13.8689713	13.3003662	19.7176355	21.0178608	7.03669167
8.64762736	8.33678836	10.0348871	20.1052047	9.72188972	13.2651649	18.3814339
8.64762736	8.33678836	10.0348871	20.1052047	9.72188972	13.2651649	18.9239986
1.5770749	1.40282496	2.22471553	2.59821108	3.37755793	2.99535981	0.7060247
103.771528	99.0795232	83.5451685	136.993772	117.347317	122.381844	46.9027092
224.391474	215.714399	211.300641	175.781352	165.226483	166.255055	204.085342

0	0	0	0.15465542	0.04564267	0.05034218	0
0.84110661	0	0	0	0	0.02517109	1.21405703
21.5007878	21.3229395	34.1754174	45.3759006	33.9125073	33.6285774	33.1672095
0.07885374	0.08016143	0.04733437	0	0	0	0.03119684
16.0335948	14.5492989	13.9636401	18.6823749	18.8960673	23.283259	16.0899593
30.8055297	34.7900591	30.2466644	23.5385551	26.381466	24.1390761	8.53446968
99.4082878	89.0593449	94.4794086	124.683201	106.301789	144.054153	58.1006663
541.357244	665.780728	682.798331	479.988565	469.480552	441.828157	629.500327
9.72529521	7.77565837	10.5555652	12.8982621	13.190733	13.1393094	2.7897316
0.18399207	0.16032285	0.09466875	0	0	0.05034218	0.17948548
61.2167907	54.4296086	54.5765321	84.9058262	79.1900407	62.1977655	35.6767978
161.939308	140.803546	133.908941	198.825009	209.865018	173.932238	111.416181
35.4316161	34.950382	38.9088547	39.6845811	48.97459	57.1887184	49.1138518
38.4017738	30.1006157	34.6487611	34.7356076	54.3604256	58.6738127	32.9311773
0.49940705	0.88177569	0.94668746	0.30931084	0.45642675	0.17619764	0.40864033
29.5964389	24.8901229	30.6253394	31.580637	38.9788442	45.2576213	77.5265266
27.1519728	22.5253609	21.1111304	33.9004683	29.2569545	30.4570199	41.0310174
24.4183764	22.3249573	24.992549	29.6010476	32.1780857	30.3059934	17.5761343
26.7577041	26.1727058	26.0812396	33.9623305	32.7714405	33.5027219	11.696375
53.3051316	51.4636358	37.299486	64.3057241	62.0740376	63.9093996	25.516487
53.2262778	37.4353862	42.1749264	61.9240306	52.2152199	66.8795883	19.7186317
0	0	0	0.06186217	0.04564267	0.10068436	0
0.52569163	0.36072642	0.28400624	0.71141494	0.7302828	1.33406781	0.64532949
56.1964356	44.9304796	44.2576388	68.6979381	53.9039989	73.8519805	24.844334
73.6493978	78.558198	84.3025184	105.320342	102.969874	90.7669535	45.8494778
25.2857675	27.7358536	25.9865708	34.0241926	29.6220959	43.9738957	13.6714728
48.9156064	37.5556283	45.3463294	54.5933637	52.3521479	73.9274938	37.4571149
6.75513748	6.93396339	6.48480911	8.32046166	9.76753239	10.4711738	2.53363796
50.9395192	47.4154838	53.2038353	60.2537521	63.1238192	70.101488	24.6639872
32.8294425	33.1066692	27.1225958	43.6437598	33.95815	47.6992171	92.1095724
4.94150135	4.48903989	3.73941547	9.92887804	6.16176109	5.4872978	2.48708736
15.2450574	12.5452633	11.9755964	17.5997869	17.8919285	18.4755807	8.41943755
14.5879428	15.7116396	13.5376307	16.517199	20.4022756	24.1390761	14.5142055
289.866366	249.903247	247.937446	221.466563	177.413077	199.103328	284.696313
42.6335914	30.0605349	35.3114423	43.8293463	46.8293843	53.8409633	35.4541432
71.7569079	64.0890605	69.0608503	50.0155632	61.2524695	44.4018042	76.5961333
15.9547411	12.9861511	12.0702651	22.2703806	14.8338693	21.898849	12.240326
1.15652159	1.2024214	0.89935309	1.39189879	3.14934456	2.01368727	0.97569368
0.60454538	0.40080713	0.75734997	0.27837976	0.13692802	0.25171091	1.1145915
3.28557271	3.44694134	4.97010917	2.07238264	2.69291781	2.29056927	1.20841633
66.3685687	65.6121276	81.7464623	75.8430185	108.903422	102.823906	35.773383
17.0586935	20.6014866	21.1111304	23.0745888	28.1615303	26.8323828	6.79838689
78.0652075	66.6943069	61.91336	86.5761048	94.6172647	83.2156263	55.5209412
5.78260796	7.2546091	6.53214348	3.95917878	4.8837662	4.35459871	1.78834008
24.2343843	21.7638273	21.7264772	29.013357	26.3358233	32.7224181	15.7815094

10.3561252	9.73961332	10.1295558	12.2487094	12.9168769	17.3680527	6.37088027
28.5450557	20.5614059	25.4185583	33.5911575	29.6677386	36.4980817	73.281734
1.91877446	3.96799061	4.59143419	6.65018311	4.5186248	7.55132725	0.71095216
37.9812205	37.9564355	43.5949576	41.0455488	61.3893975	59.1772346	30.1016312
0	0	0.04733437	0.09279325	0.09128535	0.17619764	0
19.6871517	17.9962403	20.8271241	20.3835845	31.8129443	29.1732943	21.8439934
18.4517763	20.2407602	15.3836712	25.5490756	20.493561	28.2167928	34.2775797
82.4547326	77.8367452	74.4096345	103.062373	85.7169431	111.23105	40.5734756
72.9397141	79.680458	83.4978341	73.6778426	37.472636	49.9897864	21.2670905
0.02628458	0	0.04733437	0.09279325	0.1825707	0.20136873	0.02330811
1.31422908	1.40282496	1.37269682	2.3507624	1.68877897	3.22189963	1.48721284
0.89367578	0.52104927	0.99402183	1.36096771	1.23235222	2.21505599	0.28951812
4.81007844	3.12629563	3.31340611	5.59852625	6.80075853	5.4872978	2.91797576
163.516382	153.38889	153.600041	231.117061	184.898475	202.098688	160.793555
8.91047318	8.65743406	9.46687461	11.3517079	12.6430209	12.8875985	5.54427804
42.7387298	37.2349826	46.6716918	44.0458639	64.6300274	65.1679542	57.5789194
2.78616565	3.72750633	3.31340611	1.7630718	2.60163246	1.30889672	2.18900155
22.6047402	20.7217288	21.5844741	25.0232471	45.5513894	25.4479728	20.9803079
50.387543	45.1709638	52.7304916	39.5608567	37.3357079	40.7016539	14.4877987
19.7397208	19.0784195	16.6616993	28.7968394	18.7591393	30.4066777	11.1457696
56.0387281	45.0908024	48.5650668	50.5723227	77.5469044	80.4971485	38.6313614
0.13142291	0.04008071	0.42600936	0.03093108	0	0.02517109	0.12042525
0.10513833	0.04008071	0.09466875	0.21651759	0.41078407	0.276882	2.82668498
0.52569163	0.32064571	0.94668746	0.37117301	0	0.10068436	0.2931043
30.0432768	36.9143369	41.2282389	40.5815825	49.2028034	54.7722937	78.3614033
36.8772681	36.1928841	44.9676544	46.6131439	49.7505155	55.1750311	49.8468718
0.07885374	0.12024214	0	0	0	0	0.03892681
0.07885374	0.12024214	0	0	0	0	0.10428176
23.1041473	22.365038	17.4190493	11.5991566	19.3981368	11.6793861	9.73913272
457.745989	482.611868	456.587363	617.044199	590.981352	619.208835	359.405602
15.5604723	18.8780159	18.9810836	26.4151459	20.9499877	23.7363387	8.29407263
23.5772697	26.5334322	27.1225958	28.8587016	33.6386513	38.4362557	17.1779195
0	0	0.04733437	0.15465542	0.09128535	0.10068436	0
117.229234	91.1034612	100.727546	144.417232	115.384682	150.44761	269.557887
21.9739103	19.0383388	21.5844741	26.6316635	26.2901806	27.4616601	11.1144763
0.47312247	0.56112999	2.31938428	0.37117301	0.04564267	0.47825073	0.24962853
13.9833974	11.1023576	10.6502339	15.5892665	14.6969413	19.8348196	27.9733184
28.3610636	26.97432	30.7200081	30.9620153	37.609564	47.674046	11.4955837
77.592085	72.3456874	63.2860568	82.1220286	90.9658507	97.0093841	45.8980924
14.5616582	13.2667161	10.3188933	17.5069937	16.3857202	18.5259229	10.4782718
0.55197621	0.48096856	0.47334373	0.12372434	0.13692802	0.30205309	0.92814347
48.9156064	51.1429901	59.9253163	72.0384952	64.264886	67.4081813	38.1132109
28.5713403	28.6977907	30.2939988	30.6836356	41.534834	43.8983824	25.2170994
98.830027	106.454374	129.696182	76.1523294	86.721082	92.7806408	51.8427602
183.75551	161.405032	152.322013	221.157252	200.097486	216.421039	201.872842

41.7924848	43.0867668	54.7185353	46.7987304	80.3767502	70.8817918	17.8693224
12.4326071	11.5432454	11.7862589	15.8985773	14.8338693	16.6884332	22.5312966
0.07885374	0	0.14200312	0	0	0	2.87942164
3.8638335	3.72750633	4.2127592	5.62945733	4.92940887	7.04790543	2.11858836
3.02272689	2.52508494	6.53214348	2.7528665	1.87134966	1.13269909	2.64307169
22.9464398	21.2026973	19.2177555	25.6109377	27.8420316	27.990253	23.3139883
13.5891287	12.9861511	12.3542714	18.4039951	14.6056559	19.6082798	24.0395966
84.9254833	70.5821361	79.7110842	92.1127688	94.6629074	111.180708	18.5730032
8.04308198	8.17646551	5.39611853	9.68142936	10.9542419	10.4711738	4.82894908
28.7553323	24.7298001	28.4479582	31.0857396	35.3274302	39.9465212	18.3494912
41.4507853	33.9483641	35.4061111	46.0563844	45.0036773	49.2094826	17.6522664
19.8974283	21.4431816	21.2057991	24.7448674	26.5640367	28.7453857	15.9817669
162.491284	190.984599	184.272714	155.18125	133.230968	122.482528	277.616194
0.13142291	0.08016143	0	0.18558651	0.27385605	0.42790854	0.1952249
156.261838	137.316524	160.41619	181.658258	189.280172	223.418602	93.1090138
32.5140275	30.7819878	22.0104835	40.6125136	41.260978	33.5530641	72.97969
43.1855676	38.6378076	35.4534454	44.5098302	51.6675078	53.3123704	100.338593
67.603944	65.692289	93.1067118	61.3982022	57.8292689	50.7700902	75.0103485
8.17450489	7.01412482	6.43747474	9.43398069	10.0413884	10.5466871	13.6702002
68.7078964	64.7303519	80.6104373	63.3159294	50.663369	50.6945769	68.5278092
47.6013774	42.6057982	45.298995	41.5713772	29.3482399	32.7727603	32.6218846
83.4535467	102.486384	122.264686	69.1309732	93.9782673	51.3238542	94.4817167
16.8747014	16.9942224	16.1883556	22.3013117	18.3027126	26.4799876	12.1928965
7.09683704	6.57323697	5.58545602	7.98021973	8.76339355	10.7480558	3.20644737
40.2942637	42.8462825	38.2461734	30.0650139	32.7257978	33.6537484	23.6863909
32.9608654	50.3413759	35.2641079	61.9549617	57.6466982	44.049409	33.0225563
0.05256916	0.08016143	0.04733437	0	0	0	1.47832376
443.2369	419.404584	473.864409	673.957394	491.160823	634.865253	163.932025
0.02628458	0.04008071	0	0.21651759	0.09128535	0.12585545	0.03568651
32.2774663	32.3852163	28.7792988	38.0452336	39.0244869	40.3492586	38.8245321
0.02628458	0	0.04733437	0.21651759	0.04564267	0.17619764	0.01689624
0.15770749	0.16032285	0.09466875	0	0.09128535	0	6.43070834
2.36561235	2.4048428	2.03537804	3.09310842	3.6057713	3.92669017	2.66098276
19.0826063	16.633496	16.7563681	25.1469715	19.3981368	25.0955776	26.7546949
19.5557287	21.0824552	20.1171086	25.9511797	26.7466074	24.5166425	10.5314704
10.1721331	10.0201783	10.3662277	7.79463323	8.17003878	6.36828598	7.84331587
0	0	0	0.03093108	0.09128535	0.07551327	0
0.05256916	0.04008071	0.09466875	0	0	0	0.10380482
75.3841802	100.402187	80.184428	137.334014	117.895029	92.4030745	44.6244169
22.4470327	23.1666523	27.406602	33.2199845	28.344101	31.7659166	14.4673997
0.18399207	0.20040357	0.14200312	0.37117301	0.82156815	0.35239527	0.17057582
7.07055246	7.05420553	7.24215908	10.300051	9.63060437	9.11193488	14.6722692
13.2474292	11.5432454	9.89288397	15.8676462	13.9210158	17.7959612	6.95808717
0.5782608	0.6012107	0.66268122	0.55675952	1.73442164	1.86266072	0.79169325
0.07885374	0.04008071	0.04733437	0	0	0	0.08329933

348.165569	337.239121	423.926645	489.26789	406.037234	688.429334	322.533747
6.54486083	6.25259127	6.1534685	8.69163467	8.26132413	8.83505288	3.46890463
0.34169956	0.52104927	0.66268122	0.03093108	0.13692802	0.30205309	0.36221494
26.4685737	34.3491713	26.7439208	37.1482322	40.5306952	35.742949	27.404669
2.78616565	3.36677991	3.02939988	3.77359228	4.65555282	5.66349544	2.30600502
0	0	0	0.06186217	0	0.12585545	0
41.7662002	48.0166945	47.6183793	40.4887893	36.1033557	29.7018872	23.7803015
0.07885374	0.24048428	0.28400624	0.40210409	0.82156815	0.40273745	0.14364948
3.83754892	5.29065415	4.78077168	5.19642215	7.07461458	9.16227706	2.2024007
4.7312247	4.00807133	4.44943107	3.06217734	3.24062991	1.33406781	3.89871169
4.99407051	5.69146128	8.75685902	10.2072578	6.70947319	15.4047076	2.47635708
166.644248	179.641757	153.836712	127.652585	147.197626	101.76672	106.256687
167.196224	174.551506	180.154624	212.651204	262.62795	199.279526	180.042851
15.6656107	12.4250211	12.9222838	17.0739585	17.5724298	20.2878992	11.2959097
14.6930811	14.5092182	17.4190493	11.8466053	11.6845247	11.5283596	13.5141577
26.7314195	35.1908662	40.8022296	34.6737454	53.5845001	57.6669691	12.2817472
7.75395159	6.81372125	9.51420899	10.8258795	10.3608872	12.2079791	6.27126929
65.527462	66.9748719	74.9776469	56.0780557	60.202688	43.9235535	18.6415766
0.02628458	0.08016143	0.09466875	0	0	0	0.58997324
0.26284582	0.56112999	0.18933749	0.43303518	1.14106687	0.93133036	0.38415223
1.99762821	1.08217926	1.60936868	3.18590168	2.46470444	2.41642472	1.44169272
0.13142291	0.16032285	0.23667187	0.30931084	0.45642675	0.553764	0.34060955
210.907483	144.97194	150.523306	244.819532	192.292589	252.390528	313.487264
13.4839904	19.5593881	15.857015	20.569171	26.2901806	20.1620438	20.3388001
18.3992072	18.5172895	17.0877087	17.8472356	29.2113118	28.2167928	7.70408324
115.520736	108.899298	112.561139	153.727489	126.201996	147.074684	63.1292453
21.7110644	19.9601952	14.9103275	28.271011	25.5598978	22.5029552	9.66830194
0.02628458	0.04008071	0.09466875	0.06186217	0.45642675	0.276882	0.12414495
43.0015756	41.5236189	38.9088547	55.088261	47.5140244	50.6190637	48.0833585
272.465973	267.939568	260.670392	337.76744	317.307875	363.168499	207.149542
0.55197621	0.36072642	0.28400624	1.02072578	0.5477121	1.00684363	0.57077293
15.6918952	15.2707518	18.8864149	21.0640684	19.6719928	24.9697221	49.5371984
4.67865553	5.09025058	5.91679663	6.2790101	8.39825215	7.72752489	8.58070528
11.2760855	8.73759549	9.32487149	14.0427122	11.9583808	13.8189289	32.2286668
82.9278551	87.496197	84.3025184	97.4019842	123.919862	101.968089	9.3419674
28.5187711	32.705862	30.0573269	26.9409744	23.7341909	19.4824243	16.459822
40.2154099	36.9544176	32.5187143	41.7260326	51.1197957	46.6672024	11.9753941
0.18399207	0.40080713	0.18933749	0.46396626	0.7302828	0.60410618	0.23408715
0.05256916	0.28056499	0.28400624	0.03093108	0.04564267	0.02517109	0.04002931
2.18162028	2.32468137	1.7513718	3.40241926	4.01655538	2.71847781	1.5128934
0.44683789	0.08016143	0.09466875	0.64955277	0.86721082	0.42790854	0.80089408
22.7361631	25.2909301	23.8091897	18.8060992	19.9002062	17.4435659	6.72700775
0.23656123	0.16032285	0.23667187	0.30931084	0.63899745	0.65444836	0.37951721
38.454343	37.6357898	34.5540923	45.5614871	42.8128289	48.781574	22.6920557
45.3934725	48.6179052	48.3757293	37.9215093	38.8875589	39.0907041	20.8913343

14.9822115	15.1104289	10.9342402	15.8985773	23.2321214	18.2238698	5.82565758
34.0911024	29.8200507	37.2521516	28.5803218	27.6594609	21.8736779	11.6116229
41.4507853	39.1588569	57.1799227	33.0034669	33.1365819	38.3104002	132.047227
14.7193657	12.5051825	11.549587	16.9193031	13.8753731	25.9010525	19.3716873
27.835372	31.5435213	32.4240455	36.8389213	39.0244869	38.788651	11.1641314
45.7877412	51.7842815	59.120632	42.3137232	41.5804767	41.0540492	61.7206277
132.106307	144.010003	143.233813	128.147482	89.5965705	104.157974	85.7541526
18.1889305	16.072366	10.6975683	19.1463411	23.5516202	20.5396101	3.95384289
70.7843784	67.656244	60.4933288	83.0499611	77.4099763	87.0919743	30.3237569
1.73478239	3.44694134	3.6920811	2.01052047	1.64313629	1.15787018	1.42568609
36.4041456	23.6877015	25.7025646	42.0044124	36.9249239	36.2211997	29.4723066
22.4207481	26.8941586	40.092214	24.2809011	20.9956304	17.2673683	56.8434633
232.119141	238.640567	262.469099	196.536109	209.910661	168.470111	80.6441143
28.2296407	29.9402928	32.8973893	34.4262967	51.8500785	35.5919224	4.08190352
25.0492063	22.8059258	21.1111304	31.209464	26.0163246	30.4066777	21.6630083
0	0	0	0.12372434	0.04564267	0.02517109	0
29.5964389	27.8961764	22.8625022	35.5398158	34.2776487	33.2258399	16.3527046
0.05256916	0	0.04733437	0.24744867	0.13692802	0.12585545	0.06823033
1.05138327	0.32064571	1.18335933	0.37117301	0.50206942	0.07551327	1.02768012
0.07885374	0	0.09466875	0	0	0	0.28370772
10.9606705	11.8238104	10.1295558	15.1871624	12.9168769	15.22851	7.0998289
20.4231199	21.3630202	20.3537804	18.0328221	16.1575069	12.7869141	14.6615621
2.26047402	2.76556921	2.9820655	3.09310842	4.01655538	5.78935089	0.78791981
43.2381368	38.998534	45.2516606	44.3551748	60.3852587	60.964382	56.059964
21.3956495	23.8881051	26.9805926	26.5698014	32.0411577	34.5599077	7.06042162
3.23300354	4.44895917	3.73941547	1.97958939	3.10370188	2.21505599	2.10702484
35.0110628	40.120794	32.0927049	46.2419709	43.8169677	43.8228691	28.9479672
3.18043438	3.16637635	4.16542483	1.02072578	2.69291781	2.26539818	10.319837
0	0	0	0.03093108	0.1825707	0.02517109	0
45.1306267	48.0567752	29.5839832	50.0464943	58.8790504	55.2757155	22.3331841
134.892473	111.143818	126.240773	140.519916	153.952742	191.124093	98.5146125
39.8474258	20.0403566	19.8331023	39.4989946	34.8253608	45.3583057	74.0239471
198.895429	154.350827	163.966268	237.64352	186.952396	253.800109	280.306279
5.57233131	3.88782919	5.20678104	7.17601154	6.02483306	7.97923579	6.85080486
37.6658055	40.0005518	37.3468203	32.2611208	30.443664	30.3059934	12.5036703
20.5019737	21.0022937	16.23569	16.0841638	12.9168769	14.5992327	8.01270368
55.1187677	71.9047996	65.4634379	82.7715814	63.6715312	130.386251	40.9262379
16.5067173	23.1666523	21.0164616	24.4046255	24.418831	32.1938252	12.7638585
0.7096837	0.6012107	0.89935309	0.21651759	0.27385605	0.50342182	0.46437316
8.35849696	8.33678836	8.47285278	7.0522872	5.88790504	5.46212671	5.29372315
0.23656123	0.36072642	0.33134061	0.12372434	0.04564267	0.12585545	0.7289216
0.10513833	0.32064571	0.09466875	0.03093108	0	0.05034218	4.67687879
10.9081014	11.8638911	13.1589557	12.8982621	18.9873527	17.0408285	5.7965768
0.5782608	0.48096856	0.5206781	0.12372434	0.22821337	0.32722418	0.28523303
80.9039423	75.6323059	76.63435	91.7106647	89.8704265	103.176301	28.7323563

63.7663951	68.497939	81.3677873	61.33634	55.5927778	51.9783026	24.3611781
1.47193657	1.16234068	1.46736556	1.82493397	2.37341909	2.49193799	3.54448491
1.70849781	1.32266354	1.04135621	0.77327711	0.91285349	0.67961945	3.66663195
0.05256916	0.12024214	0.09466875	0.27837976	0.22821337	0.25171091	0.05503388
0	0	0	0.09279325	0.13692802	0	0
3.78497976	3.16637635	4.26009358	4.91804239	5.11197957	6.24243053	1.56079892
0.26284582	0.20040357	0.23667187	0.21651759	0.63899745	1.03201472	0.168918
645.207626	655.560146	651.699648	816.054795	842.518133	850.858383	427.32701
15.6130415	13.8679268	15.3836712	20.0742737	16.4770056	22.2512443	17.0757298
89.0784472	77.7565837	70.8595565	98.051537	86.4472259	118.581009	129.109423
0	0	0	0	0.09128535	0.10068436	0
6.41343792	8.05622337	5.72745914	8.0420819	9.17417762	11.2263065	3.36210164
84.6363529	78.7185208	112.703142	105.784308	117.621173	142.342519	34.4082433
280.351348	225.413931	261.901086	324.281487	319.042296	329.28821	125.830004
0	0	0	0.06186217	0.04564267	0.05034218	0
0.52569163	0.40080713	0.23667187	0.89700144	0.82156815	0.70479054	17.618379
14.1411049	10.7015504	13.6322994	14.5376096	17.3898591	20.2627281	11.6724733
8.04308198	8.61735335	7.14749033	10.5474997	10.0413884	11.2514776	5.40010085
28.5976248	24.4091544	26.5545833	20.5073088	23.8254762	17.4939081	9.36001427
46.4711404	41.2430539	32.6607174	35.1686428	30.8088054	25.0704065	25.8045673
38.4280584	39.3993411	38.8615203	45.9945222	51.9870065	45.2324502	5.83702464
3.07529605	3.48702205	3.55007798	4.1138342	4.65555282	6.14174616	1.78249911
1.31422908	1.08217926	1.18335933	1.51562313	2.69291781	1.98851618	0.41248931
42.3707456	38.1969197	41.4175764	53.3251892	42.9497569	56.6097833	35.8447505
3.49584936	4.12831347	3.12406862	4.48500721	5.38583562	5.73900871	3.0447996
3.02272689	3.3266992	2.31938428	4.97990456	3.69705665	4.35459871	6.04686393
6.33458418	7.77565837	8.14151217	5.75318167	4.65555282	5.38661344	17.3355784
17.6369543	21.4832623	16.8983712	22.2085185	36.9249239	20.6654656	18.0998544
0.84110661	1.04209854	0.37867498	1.29910554	1.32363757	1.56060763	2.36531801
5.62490047	5.77162271	5.68012477	7.98021973	5.70533434	12.0066103	4.63201476
63.266988	70.3015711	52.6831572	74.4201886	79.3269687	80.4468063	37.8637842
0.13142291	0.36072642	0.09466875	0.03093108	0.04564267	0.05034218	0.0201754
0.15770749	0.84169498	0.37867498	0.68048385	2.00827769	0.85581709	0.22380655
0.05256916	0	0.09466875	0.09279325	0.31949872	0.22653982	2.85809259
156.209269	147.657348	127.471467	170.801447	167.508616	227.924228	166.091698
38.1652126	55.8324336	44.9676544	33.436502	37.2444226	37.3538988	45.1517819
0.02628458	0.04008071	0.04733437	0.15465542	0.09128535	0.20136873	0.97434975
1.62964406	1.28258282	0.99402183	1.67027855	2.37341909	2.66813563	0.99501851
0	0	0	0.03093108	0.09128535	0.05034218	0
54.5405069	56.4336443	47.8077168	62.2642725	63.1238192	72.2410307	75.3031591
15.9810256	15.1905903	22.2471553	12.8982621	14.1492292	12.6862298	15.9716939
5.94031545	6.25259127	5.25411541	4.63966263	3.65141398	4.00220344	3.11942495
334.23474	366.137316	397.845406	307.764288	280.519879	279.600477	420.858915
43.2381368	43.9284617	64.706088	55.3047786	71.6133567	78.3072636	65.6235435
65.7377387	66.814549	62.1973662	69.0072489	89.0488584	85.4810245	38.6987525

2.02391279	2.64532708	2.50872177	1.42282987	1.64313629	1.48509436	3.05468564
0.07885374	0	0.14200312	0.34024193	0.22821337	0.17619764	0.23441221
11.0132397	9.29872548	6.29547162	13.1147797	12.0496661	12.3841767	10.4761539
0.13142291	0.16032285	0.14200312	0.21651759	0.45642675	0.47825073	0.16255582
0.84110661	1.04209854	1.60936868	1.91772722	1.82570699	1.98851618	0.53353038
0.97252952	2.1242778	1.04135621	0.95886361	0.59335477	0.50342182	1.93130039
0	0	0	0.06186217	0.09128535	0.02517109	0
7.07055246	6.77364054	7.10015596	7.94928865	8.99160692	12.182808	9.19649411
0	0	0	0.06186217	0.09128535	0.02517109	0
41.6610619	55.0709	66.8834691	56.0161935	86.584154	83.3666528	69.7650425
14.325097	13.7076039	13.1116213	14.0736433	19.3068514	22.4022708	8.26393785
2.10276653	1.92387424	1.46736556	1.97958939	1.73442164	9.16227706	0.72458685
9.25217274	9.94001689	11.1235777	11.2898457	13.6015171	15.6564185	5.27893968
17.7420926	24.1686701	19.0757523	25.1469715	27.2030341	25.8758814	10.1324186
126.27113	116.474553	112.561139	125.147167	166.321907	162.303194	34.7660622
108.71303	116.634876	115.732542	132.354109	141.172793	147.603277	55.2271796
37.4555288	34.0285256	30.1519956	37.7359228	42.7671862	47.9005859	22.5430735
0.2891304	0.36072642	0.23667187	0.27837976	0.7302828	1.15787018	0.16826306
242.396412	230.784747	254.185583	260.718109	317.216589	354.711012	77.2086867
58.4569096	39.1989376	39.9028765	34.7974698	36.3315691	34.7109343	23.864334
6.99169872	6.17242984	6.20080287	7.11414937	9.81317507	9.79155433	6.07446171
0.52569163	0.96193712	0.75734997	0.37117301	0.45642675	0.276882	0.53994193
0.18399207	0.12024214	0.14200312	0.27837976	0.3651414	0.50342182	0.21851922
0.02628458	0	0.18933749	0	0	0	0.06677786
474.594406	441.328734	474.52709	629.695013	643.196572	517.945536	359.371308
51.0709421	45.010641	47.2870387	50.665116	60.7504001	71.8382932	64.3401972
28.7553323	33.0265077	30.8620112	22.7034158	25.9250392	25.9765657	3.91736451
9.43616481	9.53920976	9.98755272	8.59884142	6.84640121	5.38661344	4.75123337
0	0	0	0.09279325	0.04564267	0.02517109	0
0	0	0	0.09279325	0.04564267	0.02517109	0
0.13142291	0.16032285	0	0.03093108	0	0	0.40597906
0	0	0	0.09279325	0.04564267	0.02517109	0
0	0	0	0.09279325	0.04564267	0.02517109	0
1.07766785	0.88177569	0.99402183	1.60841638	1.18670954	2.26539818	1.47790943
62.951573	64.2894641	50.4584417	70.9249761	79.6008247	71.5865823	78.2201446
0.07885374	0.04008071	0.18933749	0	0	0.02517109	0.04231657
0.65711454	0.9218564	0.5206781	0.37117301	0.1825707	0.42790854	1.55331119
12.6428838	11.2626804	11.3129152	13.238504	15.6554374	17.4939081	9.72695192
12.3011842	12.2646983	10.6975683	9.4030496	9.90446042	6.39345707	2.72359412
40.4519712	39.0786954	46.6243575	42.2518611	59.5180478	62.4998185	12.5786224
10.1458485	12.4250211	9.70354648	12.2796404	14.1948718	16.638091	22.342515
24.8126451	26.132625	25.0398834	21.7445522	20.630489	18.6517783	12.2514403
16.8221323	15.9521239	15.4310056	22.1157252	19.2612087	19.7089641	7.911675
68.8918885	61.2834106	51.0264542	68.5123516	79.7833954	80.7740305	29.9802322
41.5033544	39.559664	39.3821984	47.9122495	49.111518	48.5298631	28.3159187

0.02628458	0	0	0.06186217	0.09128535	0.12585545	0.03525608
0.23656123	0.16032285	0.14200312	0.21651759	0.45642675	0.78030382	0.31765299
86.8705423	93.4682233	119.708629	83.9778937	84.3476629	67.2319836	59.7343762
0.26284582	0.48096856	0.14200312	0.15465542	0.04564267	0.07551327	0.06353199
0.5782608	0.96193712	1.2306937	1.60841638	1.36928024	1.68646309	1.4333899
119.594846	143.368711	127.660804	167.801132	139.529657	196.611391	241.217831
19.9499975	19.8399531	22.4364928	11.5682255	11.2737407	20.6402945	17.431813
9.75157979	11.463084	17.2770462	7.6399778	10.0413884	9.11193488	5.29235313
9.83043354	7.93598123	8.52018715	11.7228809	10.5891005	12.5352032	6.86578623
4.62608637	3.88782919	4.49676544	2.59821108	3.69705665	2.64296454	2.01927684
0.36798414	0.36072642	0.5206781	0.52582843	0.7302828	1.25855454	0.39643292
1.23537534	1.28258282	0.66268122	0.68048385	0.68464012	0.35239527	1.82514065
23.2881393	20.401083	18.8864149	26.9100433	23.7798335	28.3929905	11.9196811
0	0	0	0.15465542	0	0.02517109	0
13.5628441	11.463084	14.8629931	14.4138852	19.0329954	19.3565689	9.33402391
16.1124485	18.5573702	18.9337492	17.6925802	28.9374558	25.498315	11.2308382
0	0.04008071	0	0.12372434	0.04564267	0.10068436	0
1.36679825	1.60322853	1.32536245	1.51562313	3.33191526	2.51710908	1.2362175
0.10513833	0.12024214	0.23667187	0.06186217	0	0.02517109	0.13987403
0	0	0	0.09279325	0	0.05034218	0
0	0	0.04733437	0.12372434	0.04564267	0.10068436	0
18.3203534	17.9561595	15.3836712	16.888372	24.0536896	29.9787692	25.7746044
0	0.24048428	0	0	0	0	0
325.140275	348.782367	402.957518	270.059296	313.108749	265.957746	341.114332
0.21027665	0.08016143	0.18933749	0.30931084	0.50206942	0.40273745	0.19940181
5.99288462	7.61533552	6.29547162	7.91835756	8.35260948	10.9997667	6.47329581
1.60335948	0.96193712	1.2306937	1.67027855	2.28213374	2.39125363	0.58670836
25.1806292	23.4071365	25.4658927	22.1157252	19.8089208	15.4298787	13.0715438
35.6418927	31.8240863	31.6666956	39.4062013	42.0369034	39.9465212	26.7650108
0.55197621	0.56112999	0.7100156	0.21651759	0.27385605	0.37756636	1.20575781
3.36442645	5.17041201	5.86946226	6.03156142	6.38997446	8.75953961	3.85180471
15.7970336	14.7497025	14.4369838	20.6619643	15.7923655	21.9240201	14.0489121
10.8292476	10.3809047	11.1235777	8.6297725	7.7592547	8.30645998	3.59491429
437.743423	445.457047	513.530613	388.958384	374.452503	339.935582	243.734676
13.3262829	15.9120432	11.5969214	15.4655421	17.9832138	20.4389258	4.73477945
41.1879394	35.0706241	34.8380986	43.705622	42.6759009	51.1476566	31.9699237
0.02628458	0.16032285	0.04733437	0.18558651	0.31949872	0.22653982	0.06189325
3.39071103	3.12629563	4.07075608	4.14476529	4.38169677	7.90372252	3.39705723
2.36561235	6.13234913	2.69805926	0.80420819	1.41492292	3.04570199	1.34408674
66.1320074	49.2191159	50.5057761	66.7802108	77.8664031	65.9734291	93.0935683
0.07885374	0.08016143	0.14200312	0.18558651	0.45642675	0.25171091	0.01206738
0.18399207	0.04008071	0.04733437	0.03093108	0	0	0.05428067
27.9667949	24.9702844	20.685121	34.0241926	26.8378927	33.0999844	17.037534
0.63082996	0.9218564	0.56801248	0.43303518	0.31949872	0.32722418	0.68746993
0	0	0	0	0.09128535	0.07551327	0

0	0	0	0	0.09128535	0.07551327	0
67.9982127	66.9347911	69.8182003	76.7709511	90.2355679	80.1447532	176.605546
104.29722	106.414294	142.523797	96.0719476	90.9202081	92.1513635	193.31099
6.4397225	6.21251056	7.24215908	8.41325491	7.39411331	11.7800705	7.80735155
14.1936741	12.9059897	12.4962745	14.2901609	17.2072884	20.791321	4.56558309
6.38715334	6.57323697	4.78077168	8.59884142	6.61818784	9.41398797	6.07803238
6.12430752	7.2546091	6.29547162	7.42346021	8.58082285	10.6473714	20.6101106
124.299787	135.99386	133.766938	145.035854	168.330184	177.078624	72.1148203
0.15770749	0.20040357	0.33134061	0.40210409	0.41078407	0.62927727	0.45075058
0.15770749	0.20040357	0.33134061	0.40210409	0.41078407	0.62927727	0.44197308
0.15770749	0.08016143	0.09466875	0	0	0.05034218	0.43304433
130.292671	155.954055	172.912465	131.766419	122.641867	107.30436	146.69591
3.10158063	2.76556921	3.36074049	4.17569637	3.83398468	5.23558689	5.87779693
5.25691633	4.16839418	4.26009358	5.66038841	13.0081623	4.6566518	5.01029087
18.846045	30.3410999	29.0633051	31.4259816	31.8129443	42.5643146	16.8832584
22.1579023	22.8860873	28.0219489	25.3944202	31.3565175	37.9831761	30.1004282
11.433793	11.3027611	17.7030555	11.9393985	27.5681755	22.4777841	8.49228332
5.57233131	5.57121914	4.63876856	7.6399778	6.70947319	7.02273434	1.42605666
11.8543463	11.743649	8.85152776	10.300051	6.34433179	5.71383762	4.59773218
2.78616565	2.80564993	3.171403	2.04145156	2.05392036	1.83748963	2.80170606
6.62371458	6.61331769	7.62083406	2.3507624	7.02897191	3.22189963	15.0049861
83.1907009	76.9148887	69.7235315	58.7071979	71.5220713	54.4198984	81.6741247
19.0563217	20.0804373	15.9516837	21.3424481	20.9956304	30.2808223	6.28676314
16.7169939	13.8679268	20.685121	18.373064	21.6802705	30.6332175	10.3411644
0	0.08016143	0.09466875	0	0	0	0
54.5930761	53.0668644	49.4644199	61.7384441	66.9121612	62.07191	21.2616702
0.7096837	0.72145284	0.66268122	1.05165686	1.64313629	1.03201472	1.00132137
13.2737137	11.7837297	29.9626581	16.6718544	31.3565175	50.0401286	5.96091724
13.3525675	15.9922046	15.47834	13.9189879	9.53931902	9.74121215	10.579015
20.3442662	20.2407602	20.685121	24.1881079	27.0204634	24.3656159	18.6209063
0.07885374	0.08016143	0.04733437	0.12372434	0.22821337	0.32722418	0.04026743
10.0669948	11.8638911	11.5969214	8.32046166	7.9418254	9.18744815	24.7782826
65.8691616	71.3837503	77.2496968	85.2460681	78.6879712	97.7896879	76.7763463
17.1112627	17.1545453	16.1410212	23.3529686	19.1242807	20.8920054	5.09293324
28.1507869	25.9322215	26.1285739	31.0548086	30.443664	37.6307808	19.9284972
39.7422875	40.3211975	38.1041703	31.5497059	27.4312475	36.0701732	18.2836236
0.52569163	0.36072642	0.23667187	0.18558651	0.1825707	0.12585545	0.38141805
13.1685754	11.3027611	13.1589557	14.2282987	15.5185094	19.7341352	17.0104798
49.5464364	47.4956452	52.4938197	43.0560692	43.360541	36.1708575	34.7531113
73.0185678	63.5680112	64.2327442	68.6979381	90.2355679	90.4145583	79.6455986
77.3555238	80.2015072	76.8236875	86.9472778	97.8578946	96.3801068	42.6732235
128.452751	114.751082	118.572605	144.046059	149.799258	148.887002	45.5000313
83.3484084	89.3799906	92.3966962	78.967058	73.0739222	67.7857476	56.6753089
0.10513833	0.24048428	0.23667187	0.5876906	0.22821337	0.47825073	0.1279743
397.449159	371.067243	379.337666	303.24835	316.395021	308.97514	138.319688

3.31185729	2.4048428	2.50872177	3.55707469	3.83398468	4.90836271	4.57004653
22.0001948	23.6877015	31.2880206	33.776744	35.646929	29.097781	16.1831115
0.07885374	0.08016143	0.09466875	0.03093108	0	0	0.2207337
433.19619	434.35469	450.7179	483.514709	838.54722	498.966534	253.915918
100.144256	101.324043	113.034483	88.8959361	86.3102979	83.4925083	47.6302464
2.36561235	2.44492351	2.22471553	1.45376096	1.55185094	1.661292	1.76883787
0	0.04008071	0	0.03093108	0.27385605	0.07551327	0
26.4422891	27.1747236	38.8615203	42.7776895	39.1157722	36.9008192	59.7349269
0.07885374	0.08016143	0.09466875	0	0.04564267	0	0.04491764
47.3385315	46.5337081	50.6951136	39.4062013	38.9788442	41.6833264	36.1363673
5.86146171	4.40887846	3.64474673	6.43366552	5.61404899	8.15543343	7.12393422
54.5667915	45.2110446	48.7070699	44.819141	39.2983429	36.9259903	63.3609421
22.6047402	21.4832623	26.4125802	30.0031517	27.2486768	29.5256895	8.44724174
45.2094804	43.4875739	55.9018946	38.3854755	42.0369034	36.5484239	56.6060304
15.3501957	16.4731731	12.4016057	20.3835845	17.7550005	18.6266072	7.40298377
7.25454453	6.77364054	7.52616532	5.78411275	5.70533434	4.48045417	4.03014419
1.70849781	2.76556921	1.04135621	1.29910554	0.50206942	1.05718582	1.53168209
14.2462433	16.7136574	15.6203431	15.3727489	23.2777641	23.1322325	6.5962224
0.02628458	0.12024214	0	0	0	0	0.04731941
147.114803	122.646983	125.483423	164.584299	159.201649	164.115512	71.061549
34.5379403	25.8119793	30.0573269	38.2617512	31.7673016	46.7930579	45.1627512
0.18399207	0.20040357	0.33134061	0.37117301	0.50206942	0.60410618	0.29023184
0.02628458	0.04008071	0.04733437	0.12372434	0.09128535	0.20136873	0.07729217
0.02628458	0.12024214	0	0	0	0	0.04908938
5.04663968	3.44694134	3.6920811	6.2790101	5.43147829	5.66349544	10.0709299
0.02628458	0.12024214	0	0	0	0	0.08761183
0.02628458	0.12024214	0	0	0	0	0.02988247
5.36205466	4.92992773	5.53812165	6.80483853	7.07461458	7.22410307	3.31195279
0.02628458	0.12024214	0	0	0	0	0.00834723
2.1553357	1.8036321	1.98804367	2.53634891	3.74269933	2.84433326	1.9868805
7.3071137	8.4570305	8.33084966	11.0733282	11.365026	9.28813252	5.62181421
17.1375472	20.8820516	22.4838272	21.9610698	25.0121858	31.1618105	12.9408081
0	0	0	0.03093108	0	0.10068436	0
213.719933	208.980839	179.491943	261.986283	243.366742	237.464071	77.6824981
53.3577007	52.8664608	58.5052851	73.0282899	63.1694618	63.9849129	16.7170418
0.05256916	0	0.09466875	0	0	0	0.0567833
0.05256916	0	0.09466875	0	0	0	0.06408279
16.0598794	16.2727696	15.47834	17.4451315	21.5889851	21.4457694	17.5801467
20.3968354	18.7176931	17.3717149	21.2805859	25.2403991	24.4159581	5.7517631
350.899165	354.714312	384.355109	455.955113	462.497223	433.521697	244.935681
8.98932692	7.01412482	8.85152776	9.27932527	10.7260286	13.8441	37.0252904
0.10513833	0.08016143	0.14200312	0.03093108	0	0.02517109	0.05702246
44.9729192	53.4275908	75.877	46.1182466	46.4642429	39.5689548	62.4457546
18.846045	18.316886	16.7563681	21.651759	21.7715558	23.4846277	10.01046
0.13142291	0.16032285	0.09466875	0.03093108	0	0.05034218	0.09673147

302.640673	294.232516	327.553862	436.344805	331.867888	397.199813	115.224363
21.3956495	20.2407602	17.513718	26.4770081	20.5392036	28.3678194	15.111422
0.10513833	0.08016143	0.04733437	0.03093108	0	0	0.07080328
72.9134295	70.9027818	80.9891123	58.2432316	64.8582408	62.6005029	40.2954256
34.7482169	35.1908662	45.4409981	33.034398	30.3523787	28.8964123	50.9676044
16.3227252	14.6695411	20.5431179	15.4036799	9.63060437	12.7617431	9.35457924
4.4420943	5.65138057	5.91679663	3.15497059	4.19912608	3.95186126	15.1786272
38.7697579	39.6799061	47.6657137	36.8079902	31.4021602	34.081657	46.5650211
16.7695631	13.7476846	15.5730087	12.4342959	11.5932394	12.1576369	10.3815585
1.99762821	2.84573064	2.74539364	3.3405571	3.56012863	4.10288781	2.82505106
9.80414895	10.0201783	12.306937	7.76370214	7.48539866	8.93573725	30.9503769
0.02628458	0	0.04733437	0.12372434	0.27385605	0.05034218	0.07340991
0.05256916	0.04008071	0.04733437	0	0	0	0.05867435
0.05256916	0.08016143	0	0	0	0	0.16446824
0.05256916	0.08016143	0	0	0	0	0.02900635
0.05256916	0.08016143	0	0	0	0	0.03625998
27.519957	30.9022299	31.5720268	26.3532838	24.692687	22.1505599	31.4918224
21.6584953	28.1767414	18.6970774	19.8577561	18.1201419	12.0821236	6.07393818
0.34169956	0.36072642	0.23667187	0.55675952	0.59335477	0.67961945	0.89315394
30.1221306	29.6196471	29.820655	35.5088847	35.6925716	36.8253059	12.6213251
31.9094821	31.9443285	29.0159707	27.8379758	26.0619673	20.8416632	27.7232952
23.3144239	19.2387424	19.3597586	28.3328732	21.4520571	29.1229521	9.67113987
3.78497976	3.04613421	2.9820655	2.31983132	1.87134966	2.4667669	6.01312987
56.7746964	53.9887208	46.5770231	68.4814205	62.028395	61.6943436	17.487511
3.78497976	4.04815204	4.87544043	4.67059372	6.48125981	6.77102343	5.98205427
3.1541498	4.08823275	3.50274361	5.07269781	9.53931902	3.34775508	3.44194915
3.96897183	3.16637635	2.08271241	1.94865831	2.23649106	1.83748963	1.27482862
261.952141	259.562699	278.515451	245.06698	210.321445	187.474285	161.066698
84.5837837	91.143542	82.7878185	103.928443	102.878589	104.107632	37.2865215
42.5021685	48.6980666	44.92032	63.8108268	43.5887544	67.1816414	20.3492277
30.0432768	26.7338357	30.2939988	33.2818466	34.9166462	37.9328339	26.3735186
0.26284582	0.16032285	0.04733437	0.06186217	0	0.05034218	0.32759594
29.5175852	27.5755307	29.5366488	31.0857396	36.3315691	39.0403619	17.6439909
0.26284582	0.20040357	0.23667187	0.03093108	0.09128535	0.12585545	0.21791624
0.18399207	0.24048428	0.61534685	0.12372434	0.04564267	0.15102655	0.21405116
2.52331984	2.60524636	2.36671865	3.43335035	3.37755793	3.82600581	2.09270509
15.3239111	15.7517203	13.5849651	19.3009966	16.1575069	21.4457694	6.37928018
0.07885374	0.04008071	0	0	0	0	0.04850924
11.5915005	11.9039718	12.1175995	10.3309821	9.26546297	7.80303816	5.58586247
0.07885374	0.04008071	0	0	0	0	0.16130539
21.421934	20.4812445	19.407093	24.2190389	25.6968259	25.1207487	17.7963575
128.426466	129.380542	111.756455	114.166632	99.0902468	81.1012547	67.8334217
40.1628408	32.5856199	61.6766881	36.1584375	24.0536896	35.8436333	108.816416
37.192683	31.9443285	34.2700861	40.7362379	37.5182786	49.8135888	18.6311472
3.31185729	3.80766776	2.69805926	4.14476529	4.8837662	4.88319162	4.57262557

4.33695597	3.24653777	3.21873737	4.88711131	4.60991015	5.78935089	7.01299984
15.139919	12.2246175	10.5082308	7.51625347	9.85881774	10.7480558	10.4487673
2.52331984	2.68540779	2.84006238	1.82493397	1.59749362	1.98851618	7.14523149
2.81245024	2.64532708	2.9820655	3.83545444	3.56012863	4.50562526	2.53158864
6.99169872	6.77364054	7.85750593	8.0420819	9.67624704	10.8487401	9.59376566
2.575889	2.44492351	2.41405303	3.12403951	3.51448595	4.02737453	1.50479803
2.1553357	1.48298639	1.7513718	2.66007324	2.55598978	2.89467545	3.19199808
0.02628458	0.04008071	0.04733437	0.15465542	0.09128535	0.15102655	0.01801824
1.81363613	1.92387424	1.7513718	1.23724337	1.27799489	1.08235691	0.63908811
25.443475	28.65771	33.5127361	23.5694862	24.6470444	22.452613	33.6300684
0.10513833	0.04008071	0.09466875	0	0.04564267	0	0.19369603
5.25691633	5.450977	6.6741466	7.08321829	7.7592547	8.18060452	17.5702414
5.36205466	6.0521877	6.24813724	4.33035179	4.01655538	4.58113853	25.3255695
114.075084	117.797216	118.761942	130.776624	145.234991	149.76799	59.3561765
23.997823	22.084473	21.9631491	24.0025214	27.2943195	34.6605921	15.5735722
20.3968354	18.3970474	25.7025646	16.1769571	16.2031495	18.022501	44.675288
78.7486066	79.1994894	79.9950905	89.978524	94.5259794	97.3366083	41.9838792
0.10513833	0	0	0	0	0	0.0655025
0.42055331	0.52104927	0.5206781	0.30931084	0.1825707	0.20136873	0.31474865
12.3011842	9.29872548	8.80419339	13.4550216	12.6886636	13.6930734	17.8397836
2.83873482	2.76556921	4.16542483	5.41293974	4.38169677	4.20357217	2.76810084
0.10513833	0	0	0	0	0	0.59959984
59.560862	63.4878498	76.9183562	46.3656953	61.0242561	52.4313822	15.9030722
25.3383367	28.09658	32.8973893	24.3118322	20.4479183	24.2901027	28.1015541
0.10513833	0	0	0	0	0	3.17565844
12.1697613	12.7857475	13.3009588	11.1042592	10.4065298	7.52615616	11.5582995
30.5163993	28.938275	26.7912552	37.6740606	34.6884328	32.7727603	15.330703
0.47312247	0.32064571	0.37867498	0.15465542	0.09128535	0.25171091	0.31309913
45.9717333	38.3572426	42.0329233	40.7981001	57.0077007	63.3052934	28.8097051
0.49940705	0.28056499	0.28400624	0.92793253	0.45642675	0.72996163	0.26304297
2.2867586	2.32468137	1.98804367	2.7528665	3.19498723	3.70015035	3.03730524
173.688516	169.621579	212.484001	222.889393	272.988838	205.270246	113.864203
3.36442645	3.40686063	3.26607174	3.61893685	5.24890759	5.4872978	5.11579035
8.14822031	5.85178414	6.91081847	9.09373876	7.57668401	12.2834923	10.3991632
0.02628458	0	0.04733437	0.06186217	0.13692802	0.15102655	0.01600873
1.02509868	0.80161427	0.7100156	1.1753812	1.78006431	1.35923891	0.30455085
42.3181765	48.0166945	46.2930169	39.5299256	39.7547697	33.5530641	33.0042729
104.533781	123.488678	105.129643	95.2368083	93.7956966	84.1469567	60.8347456
523.03689	581.170342	721.139174	464.182781	515.625296	443.9677	463.808138
30.7003914	28.9783557	34.1754174	38.4782688	34.3232914	41.8846951	41.1114797
131.554331	137.436766	117.483914	170.801447	155.230737	147.880159	41.680517
5.99288462	5.05016987	5.63279039	6.68111419	7.12025726	8.48265761	4.2771339
0.91996036	1.00201783	0.85201872	1.51562313	1.46056559	1.40958109	1.97607368
0.05256916	0.16032285	0.04733437	0	0	0.02517109	1.1692197
0.02628458	0	0.04733437	0.18558651	0	0.17619764	0.05253847

37.5869518	45.852336	44.0683013	38.6019931	35.7382143	25.6493416	27.1907538
89.3675776	86.9350671	136.133657	95.3605327	74.1237038	68.2891694	54.0796645
45.9454487	39.0786954	41.0862358	49.0876307	49.4310167	52.9096329	33.4450407
13.1422908	6.85380197	6.95815284	11.8466053	12.6886636	14.3223507	20.9332954
13.1422908	6.85380197	6.95815284	11.8466053	12.6886636	14.3223507	21.1397381
35.7733156	42.0446682	45.7723388	30.6527045	39.0244869	28.7453857	30.305381
19.3717367	15.1505096	18.649743	14.8778515	14.4687279	12.6358876	12.5981713
23.2618548	23.1265716	28.3532895	27.0646987	34.095078	31.3883503	46.8795459
74.4905044	68.1372125	56.7539133	90.4424903	80.3311075	75.110535	32.4166265
2.62845816	0.84169498	2.27204991	0.55675952	1.73442164	0.50342182	0.6833909
2.52331984	2.28460066	3.78674985	3.77359228	4.24476875	4.4049409	2.50648802
0	0	0.04733437	0.03093108	0.22821337	0.07551327	0
0.60454538	0.88177569	0.85201872	0.5876906	0.41078407	0.20136873	18.7223154
27.4673878	21.8439887	21.7738116	28.6112529	28.2528157	30.8849285	14.2586255
2.54960442	3.08621492	4.73343731	4.60873155	3.19498723	10.2194629	1.94202587
66.1057228	63.4076884	63.4280599	63.6871024	86.9036527	89.1308326	63.7243163
3.81126434	2.80564993	4.63876856	2.938453	1.87134966	2.59262236	8.41563817
74.8584885	88.618457	83.0244904	76.4616402	68.190156	55.9553349	102.48829
10.9606705	16.5934153	11.6915901	17.8781667	18.1201419	15.6060763	2.8123811
55.6181748	53.94864	57.2745914	61.6765819	65.3603102	74.3050601	71.5424755
1.31422908	1.56314782	1.18335933	1.67027855	2.14520571	2.4667669	1.1297801
222.919537	205.934705	229.098366	240.272662	282.75637	283.879562	100.071261
12.879445	11.5833261	11.9755964	10.0835335	9.2198203	9.41398797	12.3570474
54.2513765	65.3716433	61.2033444	53.6345	48.5181632	46.566518	49.8048105
32.0146204	34.2289291	40.0448796	27.7761136	30.8544481	28.1664506	32.4600197
18.1889305	18.4371281	26.5072489	19.455652	32.269371	34.912303	12.0271626
14.824504	12.1043754	11.549587	16.2078881	15.3815814	17.5442503	5.53137029
0	0	0	0	0.1825707	0	0
51.7017721	44.9705603	46.0090106	53.4179825	56.6425593	63.2046091	12.5128136
28.0982178	25.5314143	23.7145209	31.1476018	28.4353864	35.2395272	20.4596048
0	0.04008071	0	0.12372434	0.09128535	0.05034218	0
1.02509868	1.2024214	0.99402183	1.73214072	1.73442164	1.53543654	0.89538602
43.4484135	37.8361933	43.5476232	35.632609	36.6510678	30.3563355	37.219751
15.770749	15.4711553	18.2237336	19.2391344	18.1657845	26.052079	19.4477067
38.4017738	42.3252332	40.6602265	36.9317146	34.0037927	27.8895686	12.9482702
44.1318126	49.3794387	49.3697511	69.780526	53.2193587	53.5389102	35.4470102
102.69386	95.9933083	109.247733	94.4944623	80.8788196	78.6848299	47.0061318
1.31422908	1.08217926	1.84604055	1.79400289	3.05805921	1.98851618	1.18168106
85.6614516	75.5521445	64.1380755	91.3085606	87.5426501	94.5677883	48.0570476
0.10513833	0	0.18933749	0	0.04564267	0	0.22098654
663.869679	631.07083	818.789985	592.330263	561.222328	509.639076	620.87309
86.6339811	74.1894002	71.2382315	93.0097703	88.8662877	96.1032248	48.0408311
27.204542	30.0605349	26.5545833	22.8890023	23.8711189	22.7043239	17.2608484
2.83873482	0.88177569	1.84604055	0.43303518	0.91285349	1.43475218	3.28221408
0.36798414	0.6012107	0.37867498	0.46396626	0.86721082	1.28372563	0.33147981

1203.67613	1221.66014	1271.96927	1409.93161	1643.86657	1659.50485	1680.86677
0.18399207	0.44088785	0.23667187	0.12372434	0.09128535	0.10068436	0.08410867
9.56758772	8.97807977	9.18286837	11.4445012	11.1368126	12.6358876	9.09039936
34.7219324	32.9463463	30.2466644	28.8587016	25.7881112	26.4799876	19.1759948
9.33102648	7.89590051	6.1534685	10.7949484	7.39411331	16.1850114	8.22074706
45.8928796	46.4535467	41.3229077	52.9230851	56.2774179	51.6762495	40.0857435
9.93557186	8.69751478	9.75088085	6.12435468	8.35260948	7.27444525	9.9541677
49.6778593	52.1049272	53.5825103	57.284368	59.0616211	72.8199658	40.9089154
0.02628458	0	0	0.06186217	0.1825707	0.05034218	0.9456924
14.1673895	13.0663125	13.8216369	17.2286139	16.8877897	16.8142887	11.2427888
14.7719349	15.0302675	12.6382776	16.3934746	19.3068514	17.6197636	5.09237436
29.5964389	25.9723022	22.5311616	34.0241926	33.1822245	29.3243208	13.1558459
0.36798414	0.28056499	0.56801248	0.06186217	0.22821337	0.22653982	0.85498496
210.197799	234.031285	206.472535	193.040897	164.998269	177.229651	158.381197
0	0	0	0.03093108	0.13692802	0	0
1.20909076	0.84169498	0.89935309	1.39189879	1.59749362	1.76197636	1.6262374
5.25691633	5.25057344	8.09417779	3.06217734	2.32777641	6.01589071	5.68083775
7.14940621	6.69347911	6.20080287	8.16580624	8.53518017	9.16227706	9.14350584
28.5713403	30.8220685	29.44198	32.6632249	32.6801551	46.0379251	43.7706948
0.60454538	0.64129141	0.47334373	0.86607036	1.04978152	1.00684363	0.87208898
1.39308283	1.68338996	1.13602495	0.64955277	0.86721082	1.05718582	0.76953611
73.5968286	69.8606832	78.3857218	66.3471757	56.688202	61.5433171	39.5735898
2.91758856	2.24451994	3.59741235	3.03124625	4.47298212	5.73900871	2.30632835
30.2798381	27.1747236	21.821146	37.5812673	34.3232914	28.2923061	12.6268553
0.02628458	0.08016143	0.18933749	0.03093108	0	0	1.00479818
41.1879394	41.6037804	39.9028765	49.1185618	47.1032403	49.0081139	46.7606495
5.151778	2.88581135	7.76283718	3.09310842	3.92527003	2.99535981	6.08897987
1.76106697	1.44290568	1.60936868	2.01052047	2.41906176	2.79399108	1.82180744
5.7300388	9.0582412	6.01146538	10.26912	17.6637151	5.81452198	2.75854864
299.118539	291.346705	290.349044	362.233927	331.913531	380.058301	217.996607
1.23537534	1.12225997	1.18335933	0.61862168	0.5477121	0.93133036	1.29495841
11.0132397	10.621389	11.6915901	11.9703296	14.1948718	16.4367223	3.87748855
16.9535552	16.7136574	16.7090337	20.8784819	19.1242807	21.7478225	10.1936763
23.8401156	21.7237466	23.0045053	26.9719054	25.8793966	30.0794535	23.1179249
5.25691633	3.96799061	3.88141859	6.34087227	5.02069422	6.79619453	6.38917868
0	0	0	0.06186217	0.09128535	0	0
1.39308283	1.28258282	1.42003119	2.07238264	2.05392036	1.96334509	2.37510482
164.488912	160.242692	197.810345	151.376726	145.508847	124.823439	99.9338782
10.0407102	10.901954	9.32487149	11.6919498	11.9583808	15.2033389	12.9019463
14.2988124	11.9440526	13.5376307	11.4445012	10.9085993	8.73436852	6.61805777
0.26284582	0.08016143	0.04733437	0.34024193	0.3651414	0.35239527	0.35726157
0.02628458	0.04008071	0	0.06186217	0.09128535	0.17619764	0.07477568
0.02628458	0	0.04733437	0.12372434	0.09128535	0.10068436	0.02155786
649.097744	750.912163	636.268643	802.599774	1092.63999	754.32725	260.167664
0	0	0	0.03093108	0.09128535	0.02517109	0

120.435953	121.725126	138.784382	110.98073	109.72499	92.7302986	81.1274934
0.49940705	0.56112999	0.33134061	0.71141494	0.68464012	1.08235691	1.49368531
2.81245024	2.1242778	2.60339052	2.90752192	3.65141398	4.43011199	2.05644289
45.1569113	42.4855561	36.7314735	49.1185618	51.0285103	49.0332849	39.4288256
51.3863571	59.5599399	58.6946226	51.1600133	47.6509524	39.8710079	33.8868862
55.9861589	47.6960488	36.9681454	60.7177183	55.5014925	60.0330516	30.1771508
26.1531587	25.6115758	24.7085427	26.1676973	33.5017233	34.2075124	17.4252582
248.573289	252.428332	258.58768	279.555139	298.913877	353.5028	184.009709
0	0	0	0.09279325	0.04564267	0	0
1.55079032	2.20443923	1.98804367	0.74234602	0.86721082	1.71163418	1.47976518
35.6156081	35.230947	33.2287299	30.095945	29.8503093	26.8072117	36.1560572
0.91996036	1.24250211	1.2306937	0.80420819	0.50206942	0.67961945	1.60195581
29.5701544	26.2929479	23.7145209	34.4572278	28.4353864	34.7612764	13.7147808
44.5260813	46.4535467	38.9088547	57.0987815	50.2069422	48.781574	23.4070046
0	0	0	0	0.09128535	0.05034218	0
1.89248988	1.96395495	0.85201872	0.9897947	1.04978152	0.830646	0.50964337
2.1553357	2.00403566	4.16542483	6.89763178	3.51448595	3.27224181	2.65384038
43.6324055	43.8483003	35.7374517	50.293943	47.9704511	49.6877333	14.6192493
13.2999983	10.9420347	10.3662277	10.26912	17.4355017	20.8164921	7.96362804
2505.81431	2257.78666	2325.25374	3074.92095	3038.88928	2964.34903	3361.10279
0.26284582	0.32064571	0.28400624	0.03093108	0.13692802	0.17619764	1.11838406
2.12905111	1.60322853	1.27802807	3.12403951	1.78006431	2.94501763	2.88580263
0	0	0	0.06186217	0.04564267	0.02517109	0
0	0	0	0.06186217	0.04564267	0.02517109	0
0.91996036	1.2024214	1.13602495	1.42282987	2.00827769	1.6361209	0.74775678
31.4626442	32.2248935	29.44198	25.4872134	26.8835354	25.5989994	13.6723941
0.10513833	0.12024214	0	0.03093108	0	0	0.25317749
27.0731191	23.5273787	26.9805926	21.311517	21.4976998	21.2695718	23.0226958
89.3150084	75.5120638	73.9836251	92.5148729	91.2853495	101.464667	39.7373103
6.51857625	5.25057344	5.77479351	1.14445012	6.29868911	1.86266072	10.7467363
84.2683688	92.1856405	84.7285278	74.915086	72.9826369	74.2043758	110.546118
16.1387331	14.8298639	19.028418	18.1256154	22.0454119	22.5532974	5.60939753
7.78023617	7.65541623	8.66219027	9.18653202	10.3152445	11.4528463	9.45128931
1.2879445	1.00201783	1.13602495	1.23724337	1.78006431	2.56745127	0.66872816
0	0	0	0.03093108	0.04564267	0.05034218	0
26.1268742	27.8961764	33.5127361	30.6217734	38.9788442	38.8138221	18.7562326
0.02628458	0	0	0	0.09128535	0.20136873	0.68411791
2.60217358	2.7254885	2.55605615	3.03124625	4.65555282	3.67497926	1.37771072
0	0	0	0.03093108	0.04564267	0.05034218	0
3.52213394	4.12831347	3.88141859	4.67059372	5.47712097	5.33627126	1.74754597
24.2869534	18.5172895	19.028418	24.1571768	28.8918131	24.4411292	24.5434717
48.9156064	54.3093665	40.5655577	50.1392875	61.0698988	69.0443022	55.4566646
0.2891304	0.16032285	0.47334373	0.18558651	0.09128535	0.07551327	0.57417039
0	0	0	0.03093108	0.04564267	0.05034218	0
14.6142274	14.0683304	12.2596026	11.5991566	10.9085993	10.1691207	12.6789852

13.0108679	11.9440526	11.0762433	9.2174631	9.81317507	9.6153567	15.3777808
39.9788487	40.120794	42.884942	38.1380269	33.7299366	29.5005185	14.4498558
13.4839904	11.3428419	11.3602495	13.8880568	14.9251546	17.0659996	15.6422635
12.2223305	12.6655054	11.2182464	10.4237754	9.99574577	7.62684052	7.27916109
0.15770749	0.28056499	0.23667187	0.24744867	0.7302828	0.50342182	0.13017628
0	0	0	0.09279325	0	0.02517109	0
578.234512	543.37423	605.453966	821.993563	673.412023	665.976721	264.329508
0.07885374	0	0.09466875	0.18558651	0.27385605	0.12585545	1.50719727
0.26284582	0.68137213	0.23667187	0.30931084	0.04564267	0.02517109	0.50005508
6.49229167	6.53315626	7.9048403	6.89763178	3.92527003	3.70015035	2.28117903
2.2867586	2.60524636	8.04684342	4.14476529	10.0413884	9.6153567	3.15195282
3.91640267	3.88782919	3.73941547	4.5159383	5.34019294	5.78935089	2.59316791
0	0	0	0	0.04564267	0.07551327	0
0	0	0	0	0.04564267	0.07551327	0
0	0	0	0	0.04564267	0.07551327	0
0	0	0	0	0.04564267	0.07551327	0
2.26047402	1.32266354	1.7513718	2.96938409	2.37341909	2.69330672	6.19306738
0.02628458	0.04008071	0	0.09279325	0	0.276882	0.05219731
0.26284582	0.24048428	0.18933749	0.09279325	0.04564267	0.12585545	0.30724837
0	0	0	0.06186217	0	0.05034218	0
134.839904	149.060173	150.049963	186.5763	153.770171	191.048579	126.83404
1.41936741	1.2024214	1.37269682	1.63934746	2.14520571	2.24022708	2.01777019
14.6142274	14.9901868	15.47834	14.4448163	11.1824553	8.48265761	25.9092307
0.15770749	0.16032285	0.23667187	0.06186217	0.04564267	0.07551327	0.13849336
0.05256916	0.04008071	0	0.03093108	0.22821337	0.20136873	0.09655718
12.5903146	9.94001689	9.70354648	13.7024703	12.5517356	14.8761147	11.2914564
34.6693632	34.2289291	29.820655	21.6208279	31.7673016	26.1779345	22.9618416
95.5707389	89.9010398	87.0952464	85.6172411	77.5469044	61.1909218	49.1013345
0.44683789	0.16032285	0.28400624	0.03093108	0.1825707	0.15102655	0.38480127
24.2606689	29.4593242	29.3946457	25.9202486	21.9084839	18.9286603	23.8951038
0	0	0	0.03093108	0	0.07551327	0
1.36679825	1.12225997	0.89935309	1.05165686	2.00827769	2.76881999	1.82531023
0	0	0	0.03093108	0	0.07551327	0
0	0	0	0.03093108	0	0.07551327	0
0.2891304	0.48096856	0.80468434	0.68048385	0.82156815	1.28372563	0.42975572
23.7612618	20.9221323	20.8744585	26.074904	26.6096794	26.3037899	23.4220803
0	0	0	0.03093108	0	0.07551327	0
37.3503905	33.3471534	35.0747704	38.911304	53.5388575	38.5369401	38.2985605
82.4810172	90.9431384	89.8879744	92.4530108	126.521494	103.553868	17.5933416
0.02628458	0.04008071	0.04733437	0.15465542	0.09128535	0.12585545	0.09429191
2.20790486	1.76355138	1.79870618	2.50541782	2.51034711	3.42326835	2.17990113
999.287225	1216.93062	1146.72252	963.503274	934.396837	731.069162	202.856513
2.18162028	1.56314782	2.60339052	1.45376096	0.82156815	1.6361209	1.52456096
4.52094804	4.60928202	3.97608734	5.47480191	5.61404899	6.34311489	7.56036797
119.463424	120.161978	118.572605	104.237754	107.397214	85.2041425	52.0526614

0.15770749	0.04008071	0.14200312	0.34024193	0.1825707	0.30205309	1.06000687
0.89367578	1.28258282	0.89935309	1.5774853	1.46056559	1.661292	1.99310924
0	0	0.09466875	0.06186217	0.04564267	0.30205309	0
0	0	0	0	0	0.10068436	0
8.41106613	9.81977475	8.94619651	7.11414937	6.89204388	7.24927416	7.18013492
0.21027665	0.24048428	0.23667187	0.52582843	0.41078407	0.40273745	0.33126637
28.7290477	29.9002121	30.578005	27.0956298	25.7881112	20.0613594	12.8991819
5.20434717	6.57323697	8.23618091	12.496158	7.34847063	8.05474907	3.23003615
0	0	0	0	0	0.10068436	0
72.0460383	75.9128709	112.371802	71.4817356	67.0947319	68.9184467	69.6152115
14.7456503	12.5452633	9.94021834	15.5274043	16.2944349	15.9333005	7.93059437
7.85908991	9.81977475	15.9990181	10.6093619	18.0744992	20.9926898	3.3899538
8.33221238	8.57727264	6.05879975	10.7949484	11.1368126	8.63368416	7.44534165
0.36798414	0.52104927	0.28400624	0.27837976	0.13692802	0.12585545	0.25693469
4.02154099	2.28460066	1.65670306	3.61893685	4.29041142	4.53079635	8.3310616
0	0	0	0	0	0.12585545	0
48.626476	54.9506579	48.470398	59.3876817	63.6715312	58.1703909	30.6066643
111.788326	99.9212182	83.8765091	95.5461192	70.0615057	73.6002696	100.32943
0	0	0	0	0	0.12585545	0
91.4440596	77.9569873	74.3149657	83.5139274	99.4097456	114.528463	30.3190599
26.8628424	27.3751272	28.1166176	24.3736944	21.8171985	22.6539818	15.4748856
1.97134362	1.96395495	2.13004679	2.50541782	2.41906176	4.02737453	1.31525259
51.7543413	50.5016987	49.9377636	49.2732172	35.3274302	40.7771672	20.2011243
22.5258865	23.4071365	24.6612084	20.5073088	18.7134966	18.9538314	42.7218376
0.10513833	0.08016143	0.18933749	0.30931084	0.45642675	0.17619764	0.25169113
42.3707456	42.8863632	44.5889794	51.4693242	47.5140244	53.8913055	58.5666771
29.9907077	34.2289291	24.5192052	34.0860548	34.3232914	41.5071288	24.2881106
10.0669948	9.25864476	7.47883094	11.1970525	11.6845247	11.5031885	3.22039918
43.3958443	36.4734491	37.5834922	35.1686428	32.8627258	29.4250052	27.9470899
0.7096837	0.44088785	0.5206781	0.83513927	1.96263501	0.60410618	2.60313531
1.41936741	1.64330924	1.42003119	2.01052047	2.05392036	2.44159581	1.02134779
25.0754909	28.7779521	32.1400393	22.9199334	23.7798335	24.1642472	31.623174
54.2776611	49.6600037	57.5112633	51.5311863	42.5389728	37.6056097	29.9625738
0.02628458	0	0.09466875	0.12372434	0.13692802	0.12585545	0.01027927
0.2891304	0.16032285	0.18933749	0.06186217	0.04564267	0.12585545	0.19374909
0.39426872	0.36072642	0.33134061	0.15465542	0.27385605	0.12585545	0.57830111
0.86739119	1.28258282	1.2306937	1.45376096	1.55185094	2.16471381	1.41099319
393.664179	341.367435	349.848351	497.464628	378.788558	471.706242	1122.52586
1.47193657	0.88177569	1.08869058	1.91772722	1.46056559	1.98851618	1.82246795
2.68102733	2.80564993	2.4613874	3.03124625	3.65141398	4.55596744	10.5966438
84.2946533	84.4500628	100.301537	74.1418089	78.0033311	75.1608772	66.8285212
73.9122436	63.8485762	63.8067349	65.1099323	53.2193587	44.7038573	46.378948
29.7541464	37.315144	29.8679894	31.7662235	22.1823399	23.0315481	27.0515118
0.55197621	0.72145284	0.47334373	0.83513927	1.04978152	1.00684363	1.44587661
4.94150135	3.68742562	5.53812165	3.37148818	2.60163246	3.95186126	3.68926813

37.5869518	29.9402928	27.6432739	39.9938919	38.4767748	37.8824917	36.9313772
4.99407051	4.88984702	5.11211229	4.17569637	3.33191526	3.67497926	3.10899385
9.40988023	10.2606626	12.8749495	11.9393985	15.9749362	13.8944421	24.7281803
4.38952514	5.21049272	5.30144978	4.02104095	4.06219805	2.64296454	1.35545664
36.9561218	40.6017625	48.091723	36.6842659	59.5180478	71.1586738	47.5872417
0.07885374	0.04008071	0.09466875	0	0	0.02517109	0.08240976
0.02628458	0	0	0.06186217	0.09128535	0.07551327	0.78423272
1.05138327	0.16032285	0.09466875	0.15465542	0.09128535	0.20136873	0.7173127
0.02628458	0	0	0.06186217	0.09128535	0.07551327	0.98933974
24.3658072	21.6836659	18.9337492	27.2193541	24.6014017	27.3358046	20.3040416
22.683594	29.2589207	28.542627	20.569171	21.9541265	23.1574036	31.3897132
0	0	0.14200312	0	0	0	0
0.36798414	0.24048428	0.56801248	1.1753812	0.50206942	0.553764	0.12240641
0.52569163	0.76153355	0.37867498	1.11351903	1.04978152	0.72996163	0.26205005
0.07885374	0.16032285	0.04733437	0.24744867	0.22821337	0.22653982	0.09112955
1067.62714	1043.10056	950.000867	578.318482	1088.71472	493.479236	550.943917
60.4545378	61.363572	66.9781379	55.7996759	55.6384205	46.1889517	35.0405808
49.8092822	37.5155476	44.0683013	44.7263478	59.5180478	59.1268924	26.1225987
0.26284582	0.16032285	0.23667187	0.12372434	0.13692802	0	0.2408505
0.2891304	0.20040357	0.18933749	0.40210409	0.45642675	0.50342182	0.12219346
0.13142291	0.08016143	0.14200312	0	0.04564267	0.05034218	0.06423001
28.650194	27.8560957	22.7678334	32.1992587	31.6760163	31.7407455	30.3177858
0	0.04008071	0.09466875	0	0	0	0
0.07885374	0.20040357	0.04733437	0.06186217	0	0	0.10405677
40.9250936	38.717969	40.470889	46.7677994	43.9538958	51.1728277	30.6196113
15.1136344	14.4290568	10.8869058	16.2388192	16.4313629	18.5259229	11.1914567
30.9895218	24.9702844	22.2471553	33.9004683	32.269371	30.0291114	11.4980363
0.02628458	0	0	0.09279325	0	0.12585545	0.0735779
0.07885374	0.08016143	0.04733437	0	0.04564267	0	0.14216747
16.7958477	18.3569667	19.2650898	14.5066785	15.0620827	14.8509436	20.3426863
0.15770749	0.28056499	0.23667187	0.06186217	0.09128535	0.10068436	0.06549694
0.05256916	0	0	0.12372434	0.04564267	0.12585545	0.02114321
119.463424	120.442543	107.354358	113.300562	70.3810044	92.277219	33.9855458
11.0132397	18.597451	15.6676775	18.001891	18.3483552	22.7798372	28.6340786
158.390889	138.037976	148.203922	122.208714	117.849386	131.015528	144.487131
58.4831942	57.7963885	57.8899383	63.3468605	69.9702204	72.6437681	43.9985426
42.1078998	44.1288653	43.8789638	36.4368172	37.9290627	36.2463708	19.0530696
8.67391194	9.21856405	8.33084966	9.77422262	10.4521725	13.4665336	8.22532462
0	0.08016143	0.04733437	0	0	0	0
0	0.08016143	0.04733437	0	0	0	0
50.702958	61.8445406	65.7001098	49.953701	49.5679448	48.2529811	71.7872476
12.8005913	13.7877654	16.1410212	12.0940539	11.9583808	9.81672543	5.42671107
0.02628458	0	0.09466875	0	0	0	0.01783336
0.02628458	0.08016143	0	0	0	0	0.039868
0.02628458	0.04008071	0	0.09279325	0.22821337	0.05034218	0.16280274

12.1434767	10.5011469	14.0109744	9.83608478	9.63060437	9.51467234	18.3394275
0	0.12024214	0	0	0	0	0
0.02628458	0.04008071	0.04733437	0	0	0	0.03850724
1.81363613	1.96395495	2.27204991	2.38169349	2.60163246	3.80083472	2.55745749
0.02628458	0.04008071	0.04733437	0	0	0	0.0381871
0.10513833	0.12024214	0.23667187	0.37117301	0.41078407	0.22653982	0.13211522
5.51976215	5.33073486	3.26607174	3.9282477	2.19084839	3.52395272	14.9385924
20.8436732	18.4772088	19.2177555	19.7649628	34.8710035	22.0750467	25.4595692
0.02628458	0.08016143	0	0	0	0	0.02846706
135.549588	143.84968	162.546237	128.456793	120.907445	119.336142	73.1271508
0.02628458	0	0	0.03093108	0.04564267	0.15102655	0.08078779
3.83754892	3.76758705	2.55605615	3.80452336	4.8837662	5.68866653	1.08141375
31.3312213	22.0443923	25.1818865	29.9722206	30.1241653	37.6559519	30.7353823
0.02628458	0.04008071	0.04733437	0	0	0	0.03091687
0	0.12024214	0	0	0	0	0
0.02628458	0.08016143	0	0	0	0	0.07451574
5.28320091	8.37686907	7.24215908	5.32014649	5.43147829	4.6818229	7.11768929
0.07885374	0.08016143	0.09466875	0.12372434	0.68464012	0.10068436	0.12535494
15.5341878	13.4270389	13.4902963	19.6412385	14.4687279	19.0293447	6.56285378
12.4851763	11.7837297	9.79821522	14.7850583	13.7840878	13.9196132	6.60165649
37.5869518	36.0726419	40.4235546	40.395996	43.497469	53.6144235	18.3918258
0.05256916	0.16032285	0.33134061	0.09279325	0	0.02517109	0.22406649
105.190896	103.047514	109.200399	96.6596382	88.2729329	81.7305319	84.8243071
9.01561151	9.01816048	9.70354648	13.3622284	11.4106687	10.3201472	5.88666389
0.05256916	0.12024214	0.14200312	0.03093108	0	0.02517109	0.14040848
203.968354	194.391459	246.706752	187.349577	173.807305	173.328131	166.064215
0.02628458	0.08016143	0	0	0	0	0.01862893
0.21027665	0.20040357	0.23667187	0.03093108	0.09128535	0.12585545	0.25607599
0.02628458	0.08016143	0	0	0	0	0.02986859
63.4509801	70.8226203	70.8122221	59.6969926	59.0616211	54.5457538	55.1073125
28.7553323	22.4852801	22.5311616	39.4062013	28.207173	26.052079	83.4542695
0.52569163	0.32064571	0.42600936	0.52582843	0.95849617	0.830646	0.77385179
47.7328003	48.3774209	49.7484261	61.9858928	48.8833046	65.4951784	91.3784534
7.25454453	7.01412482	7.71550281	11.7847431	5.84226237	13.2399938	6.10342332
0	0.12024214	0	0.09279325	0.1825707	0.15102655	0
9.5938723	11.9440526	8.80419339	8.53697925	6.16176109	8.28128888	4.06724752
240.214792	232.548298	198.331023	277.173446	257.105187	274.641772	117.329294
0.02628458	0	0.09466875	0	0	0	0.01596898
0.02628458	0.04008071	0.04733437	0	0	0	0.02059804
70.3638251	74.1493195	73.8889564	64.4603795	65.4059529	53.1865149	51.5728167
2.60217358	2.04411638	2.13004679	2.66007324	5.84226237	2.4667669	2.12780791
79.5634286	75.2715795	85.012534	73.9252913	56.3230606	68.9687889	102.613359
3.23300354	2.36476208	2.69805926	3.06217734	4.24476875	4.55596744	4.65554518
0.21027665	0.40080713	0.37867498	0.21651759	0.04564267	0.12585545	0.15991814
57.8786488	58.7583256	57.5585976	73.1520142	61.2068268	72.1906885	78.5816857

6.15059211	5.41089629	4.68610293	7.11414937	6.61818784	7.50098507	8.20493866
0.02628458	0.08016143	0	0	0	0	0.61833734
0.02628458	0.08016143	0	0	0	0	0.83515693
0.13142291	0.20040357	0.04733437	0.15465542	0.3651414	0.42790854	0.40343214
34.4590865	40.0406325	43.642292	39.1896837	53.2193587	52.5824088	16.1972308
0.02628458	0.08016143	0	0	0	0	0.01920761
1.23537534	1.08217926	1.79870618	2.04145156	1.50620827	2.66813563	1.24842334
32.3300354	37.0746598	42.5536014	24.8995228	35.7382143	29.2236365	16.386516
0.39426872	0.08016143	0.09466875	0.03093108	0.13692802	0.02517109	0.47776436
0.10513833	0.24048428	0.09466875	0.06186217	0	0.05034218	0.13327893
45.5774646	40.8823275	37.7254953	45.7470736	45.2775333	60.3854469	13.8657651
112.314017	115.873342	125.67276	135.663735	140.625081	144.557575	86.9020137
0.02628458	0.08016143	0	0	0	0	0.01030067
68.0507819	61.1230877	57.8899383	63.3159294	82.2480999	81.0005703	43.7213862
37.008691	37.4754669	35.9267892	39.282477	47.7878804	44.049409	22.7099005
0.02628458	0.04008071	0.04733437	0	0	0	0.0429573
5.67746964	6.17242984	4.9227748	8.10394407	6.84640121	6.84653671	3.04746161
48.7316144	48.0968559	40.9442327	57.1297126	50.7090116	56.9873496	27.9674719
17.715808	18.7176931	21.3951366	15.9295084	16.8877897	13.9447843	6.93376647
0.02628458	0.08016143	0	0	0	0	0.02794745
0.39426872	0.56112999	0.47334373	0.30931084	0.1825707	0.22653982	0.55088878
233.591077	229.582326	289.970369	286.359978	274.038619	389.547802	852.980671
18.6357684	18.9581774	19.4544273	22.2703806	21.2238437	25.6493416	13.9515674
22.368179	20.8018902	19.7857679	19.7031007	15.8836508	15.6060763	11.8581426
21.8162028	20.7217288	22.9098366	21.8682765	15.01644	14.5740616	17.9713398
8.96304234	7.81573909	7.66816844	7.39252913	13.4189464	12.5855454	18.7424918
3.89011808	3.76758705	4.2127592	4.7015248	5.56840632	5.4872978	2.24256558
81.7713335	82.4460272	86.4325652	89.7620064	127.388705	91.0438355	86.2324726
57.7472259	89.6605556	81.6044592	55.3666408	75.356056	42.1364061	27.6104479
5.86146171	3.84774847	4.16542483	5.78411275	5.70533434	7.57649834	17.7921583
1.83992072	1.76355138	1.79870618	2.53634891	2.32777641	2.71847781	1.29502182
30.1484151	32.425297	31.5246925	29.1989435	26.4271087	22.5281263	33.1506628
83.374693	85.2115964	100.396205	79.2454378	77.6838324	70.4538832	95.9915616
13.6416979	14.5893796	18.0817305	12.9601243	12.3691649	11.5031885	11.4692015
2.49703526	2.64532708	2.65072489	4.20662745	2.92113118	3.59946599	1.87685804
17.4003931	15.5513167	13.6322994	20.1052047	17.4811444	20.0361883	11.7502868
5.17806258	5.69146128	5.30144978	6.83576961	6.20740376	7.77786707	5.50325605
0.05256916	0	0.04733437	0	0	0	0.11767078
0.05256916	0.04008071	0	0	0	0	0.03446253
1.60335948	0.84169498	1.32536245	2.04145156	1.87134966	1.86266072	0.99662909
14.1673895	16.2326889	11.7862589	12.0940539	10.8629566	10.6473714	8.20973897
4.62608637	4.64936274	5.91679663	5.41293974	6.16176109	9.21261925	25.3200149
23.7349772	30.2208578	27.406602	35.4160914	27.3856048	37.681123	41.7764721
0.05256916	0.04008071	0	0	0	0	0.04478209
90.8657988	104.370177	128.465488	82.4004084	95.7583316	86.1103017	105.110916

0.05256916	0	0.04733437	0	0	0	0.06335673
4.62608637	3.80766776	3.36074049	5.16549107	5.15762224	5.56281107	3.30068436
34.7745015	32.1848127	51.9731416	34.6118832	23.3234068	33.1754977	14.694002
179.208278	163.088422	123.116704	199.288976	179.193141	200.739449	143.376617
26.6788504	34.108687	34.9801017	27.4668028	26.1988953	25.3724596	42.4669418
79.4057212	77.1954537	68.8715128	84.5037221	96.9906838	84.524523	59.5377563
8.20078947	11.4230033	11.5022527	8.0420819	8.71775087	7.1485898	6.06523882
0.05256916	0	0.04733437	0	0	0	0.12390575
159.100573	199.882517	184.556721	139.251741	163.537704	143.399704	35.3061928
0.05256916	0	0.04733437	0	0	0	0.19726099
0.05256916	0.04008071	0	0	0	0	0.04012923
0.05256916	0.04008071	0	0	0	0	2.17990113
0.05256916	0.04008071	0	0	0	0	1.27340759
0.05256916	0	0.04733437	0	0	0	2.04149471
0.05256916	0	0.04733437	0	0	0	2.07442204
111.315203	121.484642	136.370329	126.693721	164.952626	159.484032	33.6305876
0.10513833	0.04008071	0.04733437	0	0	0.02517109	0.05389238
39.1377421	41.8041839	34.6487611	45.6233492	46.8750269	44.1752644	15.6869671
0.05256916	0.04008071	0	0	0	0	0.03306277
29.7541464	33.5074763	25.5132271	25.4253512	27.0204634	19.4824243	19.9112742
22.6047402	18.6776124	16.3776931	31.8590168	24.418831	18.8279759	8.5280018
3.70612601	3.56718348	6.39014036	5.6913195	4.3360541	11.2514776	2.66685257
37.0612601	42.5657175	43.3582857	37.7668538	32.9996538	32.0176275	89.9533607
0.05256916	0	0.04733437	0	0	0	0.85742778
30.7529605	29.0985978	26.1285739	33.3437088	37.5639213	32.3196806	14.148042
0.05256916	0	0.04733437	0	0	0	1.05421448
0.05256916	0.04008071	0	0	0	0	0.13439307
0.05256916	0	0.04733437	0	0	0	0.03234763
0.05256916	0	0.04733437	0	0	0	0.07395869
0.05256916	0.04008071	0	0	0	0	0.09063719
0.05256916	0.04008071	0	0	0	0	0.06823033
14.2725278	15.9521239	18.6024086	13.2694351	12.1865942	13.5923891	13.3634697
33.801972	30.5014228	38.2461734	41.5713772	35.646929	47.3216508	13.3622409
33.801972	39.7600676	32.2347081	29.4154611	33.5017233	22.3015865	31.6732705
0.07885374	0	0.04733437	0.09279325	0.1825707	0.17619764	2.1676545
0.05256916	0.08016143	0	0.03093108	0.31949872	0.20136873	0.053746
38.4280584	34.0686063	35.5481142	34.0241926	28.070245	28.0405952	22.8529305
26.1794433	21.4031009	19.7384336	24.0025214	29.7133812	29.0474388	15.6792791
0.84110661	0.9218564	0.85201872	0.55675952	0.45642675	0.57893509	1.3397309
0.02628458	0	0	0.03093108	0.09128535	0.10068436	0.03474181
16.1387331	17.8359174	17.135043	14.1355055	14.879512	12.9379407	7.42888978
0.02628458	0	0	0.03093108	0.09128535	0.10068436	0.03606679
2.575889	2.24451994	1.9407093	3.06217734	2.46470444	4.25391435	2.89086889
25.6274671	28.4973871	27.406602	25.4562823	22.2736253	19.8348196	17.8936662
5.20434717	5.65138057	5.4434529	6.03156142	7.30282796	7.72752489	2.83897492

20.4231199	23.9682665	20.5904523	19.5484452	16.6139336	17.1163418	57.3669389
283.794628	306.978183	317.660978	294.958819	229.810867	215.439366	86.9970654
11.7229234	10.2205819	12.9222838	12.6198824	13.8753731	17.9218167	9.92420733
39.9262795	45.9324974	42.2222608	47.4792143	56.0492046	48.0516124	44.7673967
0.31541498	0.32064571	0.18933749	0.27837976	0.50206942	0.90615927	0.45419953
7.09683704	5.89186485	4.26009358	2.56727999	6.07047574	2.7436489	9.01033342
1.62964406	2.1242778	2.08271241	2.84565975	2.92113118	2.44159581	0.51975481
0.2891304	0.40080713	0.23667187	0.46396626	0.5477121	0.65444836	0.21612524
7.93794366	8.81775692	12.5436089	8.47511708	7.30282796	6.11657507	7.39555947
0.36798414	0.32064571	0.14200312	0.15465542	0.04564267	0.15102655	1.03960643
75.2790418	65.4918855	61.6766881	80.5754744	79.0987553	79.8427001	19.4093673
3.65355685	4.12831347	5.34878416	5.44387082	4.65555282	8.10509125	2.76141013
81.8764718	90.6625734	81.4151217	86.8854156	51.3023664	64.9414144	29.7337932
0.07885374	0	0	0	0	0	0.34205895
0.21027665	0.20040357	0.23667187	0.46396626	0.27385605	0.47825073	0.22032405
0.07885374	0	0	0	0	0	0.09893397
30.9895218	36.5135298	41.6542483	37.4266119	51.3480091	45.1065948	21.8874282
0.07885374	0	0	0	0	0	0.02301888
0.07885374	0	0	0	0	0	0.15071973
0.07885374	0	0	0	0	0	0.10383275
1.94505904	1.88379352	2.08271241	2.53634891	3.05805921	2.66813563	2.50987562
0.07885374	0	0	0	0	0	0.20878923
0.07885374	0	0	0	0	0	0.23021629
0.36798414	0.24048428	0.47334373	0.55675952	0.63899745	0.67961945	0.10272697
0.60454538	0.64129141	0.80468434	0.34024193	0.45642675	0.40273745	1.50617405
0.07885374	0	0	0	0	0	0.13760432
6.80770665	5.69146128	6.1534685	7.85649539	7.07461458	8.91056616	14.0198103
0.07885374	0	0	0	0	0	0.08540117
2.70731191	2.76556921	2.88739676	3.99010987	3.37755793	3.8511769	0.56520433
87.711649	97.0754875	98.9288397	88.3391766	138.206019	126.811956	26.6873196
0.10513833	0.04008071	0.14200312	0.03093108	0.04564267	0	0.17415595
1.89248988	2.04411638	1.70403743	1.42282987	1.14106687	1.30889672	1.36420448
100.827655	121.885449	185.834749	101.175577	100.870311	113.471277	315.853997
10.3561252	10.2205819	9.79821522	13.9808501	12.7343062	11.1256221	6.53689134
0.65711454	0.48096856	0.75734997	0.37117301	0.50206942	0.22653982	0.4087661
28.9130398	29.499405	28.163952	25.5800067	25.4686125	21.9995334	13.897405
0.60454538	0.40080713	0.7100156	0.83513927	0.82156815	1.107528	1.00479818
25.3383367	22.926168	28.4952926	25.5800067	39.0701296	30.9100995	35.8750164
0.10513833	0.16032285	0.23667187	0.03093108	0.09128535	0.05034218	2.92304924
12.5114609	13.7877654	16.4723618	10.3928443	12.0953088	11.7297283	19.8638363
37.7446592	36.6337719	33.1813955	39.1587526	42.1738315	45.987583	24.2502552
100.380817	77.4760187	92.2546931	110.485833	91.8787042	130.310737	245.098554
9.51501856	8.29670764	7.76283718	8.0420819	6.48125981	5.00904708	4.32698219
67.9719281	58.2372764	52.5884885	75.1934658	83.5717374	60.4357891	83.3574524
28.7027632	24.6496387	31.5720268	26.3842148	21.6802705	21.9240201	44.7852902

4.91521677	4.80968559	4.73343731	6.49552769	6.38997446	5.78935089	2.61706738
17.9260847	14.3889761	17.2770462	17.7235113	26.7466074	18.8028049	54.1449764
5.07292426	5.49105772	5.68012477	4.39221396	4.1534834	3.8511769	136.38755
21.7636336	23.767863	20.5431179	25.332558	26.655322	26.5303297	19.8977074
0	0.04008071	0.14200312	0.06186217	0.3651414	0.17619764	0
0	0.04008071	0	0.03093108	0.09128535	0.10068436	0
14.7982195	12.8258282	14.2476463	14.4757474	21.9541265	16.6632621	6.33284729
0.86739119	0.9218564	1.27802807	1.51562313	1.68877897	1.38441	0.25691692
1.2879445	1.36274425	1.04135621	0.83513927	0.7302828	0.85581709	0.96391774
28.7553323	24.7698808	24.6612084	24.8067295	20.8587024	18.4252385	11.2924477
0.02628458	0	0.04733437	0.03093108	0.22821337	0.10068436	1.03721102
317.412608	313.511339	383.645094	369.255283	429.908353	438.706942	131.17776
71.3363546	108.177845	86.7165715	88.6175563	65.9080223	52.5320666	83.7071579
0.34169956	0.32064571	0.23667187	0.52582843	0.5477121	0.553764	0.12843633
27.0205499	22.5654416	26.4599145	29.9722206	29.1200265	31.2373237	42.7052207
50.3612584	57.034855	65.9367817	54.0984663	43.0410423	45.6100166	42.2683951
91.5754825	93.6686269	100.538208	89.5764199	78.9618273	75.4629303	49.3928303
0.73596829	0.44088785	0.5206781	0.89700144	0.86721082	1.03201472	0.78423272
22.3944636	22.1646344	23.5725178	25.7965242	26.2901806	28.4433326	14.5950013
1.44565199	0.80161427	0.5206781	0.40210409	0.7302828	0.50342182	1.38213739
0.44683789	0.24048428	0.42600936	0.09279325	0.22821337	0.22653982	0.80443003
0.52569163	0.68137213	0.94668746	0.49489735	0.27385605	0.40273745	0.97656922
18.5043455	19.4792266	20.3537804	20.7856886	25.7881112	24.0132207	15.7086005
0.15770749	0.16032285	0.28400624	0.21651759	0.50206942	0.50342182	0.14301056
9.67272605	8.93799906	12.685612	7.6399778	16.2944349	22.3267576	5.17380994
31.0683755	32.7860234	30.0099925	29.9103584	23.2777641	25.1710908	13.3095732
15.0084961	13.2266354	13.5849651	15.7129908	16.9334323	18.4000674	34.7392097
11.4863622	9.17848334	11.549587	11.1351903	13.2820183	16.8898019	19.8461832
29.7541464	28.1767414	33.0393924	38.6638553	37.746492	32.2441674	27.4700447
22.9990089	22.8860873	20.4957835	25.3016269	25.5142552	28.1161085	5.25679166
0.05256916	0.04008071	0	0.12372434	0.04564267	0.17619764	0.03969573
112.287733	122.52674	127.045457	108.166002	106.073576	90.0369919	72.0482193
0.84110661	0.24048428	0.89935309	0.34024193	0.41078407	0.30205309	0.944824
140.09682	155.032199	166.853665	127.80724	139.164515	119.361313	56.2818973
37.7183747	37.2750633	40.3288858	35.7254023	33.3191526	27.2099492	32.0641642
9.27845732	10.4209854	12.2122683	18.1256154	11.365026	12.0317814	7.04762509
1.49822115	1.32266354	1.42003119	1.94865831	1.59749362	2.64296454	1.91810767
15.4290494	14.2687339	12.685612	16.5790611	16.0205788	19.4824243	20.7294113
2.94387314	1.84371281	2.60339052	3.55707469	2.96677386	3.87634799	3.27381515
29.0970319	28.4573064	23.3358459	23.5694862	22.0454119	22.0750467	11.3158387
105.926864	94.630564	92.9647087	115.218289	116.20625	114.327095	36.6663195
6.96541414	7.61533552	7.95217467	8.87722117	9.53931902	9.64052779	6.05377517
41.7924848	49.0187123	45.1096575	51.7167728	66.7295905	47.371993	25.2340233
41.0565165	35.9924805	39.6662046	32.4776384	36.6967105	28.6950436	40.291883
21.0013807	16.5132539	16.6143649	24.3427633	20.0371342	22.1505599	44.4475429

27.9405103	31.7038442	29.9626581	24.6830052	26.9291781	24.2145894	19.7453581
0.44683789	0.48096856	0.99402183	0.71141494	1.00413884	1.51026545	0.40236305
30.7529605	32.986427	27.2172645	24.1881079	27.1117488	25.3976307	4.60235427
0.34169956	0.32064571	0.18933749	0.09279325	0.27385605	0.05034218	1.32277228
19.2403138	21.0423745	19.3124242	18.0637532	15.7010801	15.4298787	6.94698716
1.70849781	1.84371281	1.65670306	1.05165686	1.55185094	0.98167254	4.77709762
30.4638301	39.6398254	33.6074049	28.0235623	33.1822245	22.9812059	17.7245921
0.05256916	0.08016143	0.04733437	0.09279325	0.1825707	0.22653982	0.20033359
28.2822099	30.7819878	33.5127361	27.2502852	27.4312475	22.729495	27.1670285
82.6650093	75.8327095	79.5217467	73.2757385	63.3063899	66.3006533	36.8657997
21.8162028	25.1306072	27.9272801	21.4043103	20.9956304	19.6334509	24.7564375
3.91640267	5.09025058	4.07075608	4.05197203	2.96677386	2.21505599	2.85341138
12.6954529	11.7837297	10.271559	14.0427122	15.01644	13.9951265	7.89736112
44.5523659	43.1268475	45.2043263	41.880688	38.1572761	31.6148901	16.6260687
24.7337913	25.0504458	30.5306706	27.9617001	39.1157722	31.2373237	7.02740279
11.3023701	9.69953261	13.5849651	11.0733282	8.62646552	5.96554853	4.66465011
0.13142291	0.20040357	0.37867498	0	0.1825707	0.07551327	0.18279444
0.31541498	0.24048428	0.23667187	0.09279325	0.22821337	0.07551327	1.57808794
0.02628458	0	0	0	0.09128535	0.15102655	0.19605818
0.31541498	0.08016143	0.14200312	0.40210409	0.41078407	0.37756636	9.6460625
10.3561252	9.81977475	8.85152776	8.2276684	6.66383051	8.15543343	7.31648595
126.060854	152.86784	138.737047	127.590722	122.413654	90.7166114	65.7885605
1.65592864	1.64330924	1.2306937	0.86607036	0.77592547	1.28372563	1.19685266
1.05138327	1.00201783	0.61534685	0.61862168	0.59335477	0.47825073	1.03429165
3.81126434	2.96597278	2.88739676	5.01083564	3.56012863	4.63148071	7.63679532
14.6142274	17.5152717	16.23569	11.7228809	13.5102317	14.0202976	13.869177
1.65592864	2.60524636	1.37269682	3.3405571	3.10370188	2.06402945	0.49065596
0.2891304	0.16032285	0.18933749	0.12372434	0.13692802	0.02517109	0.18283223
75.6995951	80.1213458	78.669728	75.8120874	107.351571	97.5128059	26.6980539
11.8806309	15.8719625	10.4608964	16.517199	12.6430209	21.0178608	20.5129158
42.4495994	48.3373402	44.3049732	39.4989946	40.4850525	35.0633295	16.6222695
33.2762804	37.876274	30.2466644	45.1284519	34.5515048	42.5391435	25.042377
0.05256916	0.04008071	0.04733437	0.06186217	0.1825707	0.20136873	0.08894479
9.51501856	8.73759549	9.27753712	11.3207768	11.9127381	10.8235691	4.00123138
1.02509868	0.64129141	1.04135621	0.83513927	1.23235222	2.64296454	0.21441192
0.13142291	0.12024214	0.42600936	0.15465542	0.77592547	0.60410618	0.12892358
13.2999983	13.3869582	14.2949807	14.9087826	16.4770056	18.7021205	13.8524411
1.23537534	1.08217926	1.13602495	1.60841638	1.14106687	2.56745127	0.71536874
5.7300388	6.09226842	6.72148097	7.73277106	7.16589993	8.38197325	3.46232259
0.52569163	0.64129141	0.42600936	1.1753812	0.63899745	0.80547491	0.53745995
1.60335948	2.4048428	0.94668746	1.02072578	0.68464012	1.28372563	0.74619214
3.65355685	4.08823275	4.54409981	6.06249251	4.5186248	5.53763998	2.92496223
0.52569163	0.52104927	0.28400624	0.21651759	0.22821337	0.276882	0.28242022
2.575889	2.20443923	1.08869058	1.79400289	0.77592547	0.98167254	1.96021592
0	0	0	0	0.13692802	0	0

12.3537534	14.3088146	12.685612	11.9703296	20.7217743	18.3497252	6.9577185
22.3418944	20.4411638	19.6910992	25.3016269	23.1408361	25.9513947	15.4104936
72.6768683	69.7003604	69.0135159	82.0910975	75.7668401	87.4695406	35.8269364
0.02628458	0	0.04733437	0.09279325	0.27385605	0.02517109	0.0041577
121.960459	138.919752	120.087304	134.828596	160.479644	160.037796	30.9431574
15.5604723	21.1626166	20.4011148	18.8988925	11.228098	13.7434156	13.2049231
30.8318143	29.77997	29.7733207	30.0031517	23.7798335	20.6402945	21.7196109
19.0037525	17.2347067	17.0403743	15.5892665	15.290296	13.0889672	11.62932
2.99644231	5.01008916	2.03537804	5.35107757	8.6721082	2.71847781	2.35043524
160.835355	162.928099	151.469994	149.644585	120.861803	127.592259	42.8037684
17.216401	21.6836659	23.6198522	25.2706958	25.9706819	24.3152737	11.274395
0.34169956	0.16032285	0.66268122	0.68048385	0.41078407	1.00684363	0.35787332
2.20790486	1.8036321	5.11211229	2.31983132	1.41492292	1.76197636	1.92234698
45.5248954	44.3693496	48.7544043	37.1173011	42.9041142	38.5872822	40.7239007
198.343453	209.942776	240.647953	197.278455	193.753154	133.708835	66.9510556
49.5201518	47.7361295	39.5242015	33.4983642	42.9953996	38.0335183	33.2021225
4.46837888	4.48903989	5.68012477	5.6913195	6.89204388	6.36828598	6.80710098
55.1976215	59.2793749	66.7888004	45.4996249	57.8749116	48.1522968	50.0536972
30.6478222	25.6917372	32.4240455	34.1169859	30.8544481	43.4704739	28.6847969
2.44446609	2.00403566	2.41405303	2.44355565	4.06219805	3.2974129	2.12982862
8.91047318	9.6594519	9.6562121	8.59884142	6.29868911	7.34995852	12.330374
0.10513833	0.20040357	0.09466875	0.06186217	0	0.05034218	0.08263037
0.05256916	0.12024214	0.09466875	0.18558651	0.1825707	0.22653982	0.0451912
11.6966388	11.6234068	10.6975683	15.2180934	12.0953088	14.423035	7.03630492
30.2009843	37.9163547	32.8973893	29.9412895	23.1864788	29.7270583	39.5974484
5.67746964	6.17242984	6.10613413	5.1036289	4.97505155	3.64980817	14.5906828
92.4691582	91.9451562	80.9891123	101.051852	110.774772	100.306797	37.7368339
0.26284582	0.16032285	0.14200312	0.24744867	0.50206942	0.45307964	0.20108531
0.07885374	0.24048428	0.04733437	0.03093108	0.04564267	0.02517109	0.04916444
0	0	0	0.03093108	0.09128535	0	0
50.9132347	57.5158235	50.6951136	49.4897348	44.5016079	40.0472055	14.8838356
8.43735071	9.73961332	10.5082308	8.50604816	7.43975598	6.69551016	4.92310369
0.73596829	0.76153355	1.13602495	1.29910554	1.00413884	1.6361209	0.50979568
0.84110661	0.76153355	1.18335933	0.52582843	0.82156815	0.37756636	0.25774382
46.8654091	50.7822637	56.7065789	56.8513328	52.7172893	81.3529656	11.5817707
0.02628458	0.08016143	0	0.09279325	0.13692802	0.12585545	0.0184261
566.432735	495.277374	494.691533	579.895967	588.973075	743.730221	500.837603
11.0658089	8.65743406	9.94021834	13.207573	10.6347432	13.2148227	15.1331929
54.8033527	52.2652501	50.4111073	62.4498591	57.3271995	64.2114527	46.3945567
1.73478239	1.48298639	1.32536245	2.59821108	1.78006431	2.16471381	1.19321549
0.02628458	0.08016143	0	0.12372434	0.09128535	0.12585545	0.03911623
0	0	0	0	0.09128535	0.02517109	0
0	0	0	0	0.09128535	0.02517109	0
18.662053	16.072366	11.1235777	17.9400289	20.8130597	19.7341352	12.5503104
19.0826063	13.7476846	22.4364928	22.6415537	20.8587024	26.0269079	23.0439006

0.02628458	0	0	0.03093108	0.22821337	0.02517109	0.04707693
0	0	0	0	0.09128535	0.02517109	0
0	0	0	0	0.09128535	0.02517109	0
9.46244939	6.93396339	8.42551841	8.35139274	13.6015171	10.7732269	16.7031385
0	0	0	0	0.09128535	0.02517109	0
32.1460434	30.7018264	34.1754174	37.9833714	34.232006	42.4888013	31.9057051
0	0	0	0	0.09128535	0.02517109	0
3.75869518	3.48702205	3.73941547	5.16549107	4.42733945	4.70699399	4.30318808
149.033578	146.735491	146.783891	179.276564	157.375942	189.790025	205.536168
0.47312247	0.16032285	0.75734997	0.06186217	0.3651414	0.20136873	1.16334422
8.12193573	9.57929047	11.3602495	15.0325069	11.5475967	10.5466871	9.63670648
5.88774629	4.12831347	5.39611853	5.13455998	7.85054005	7.80303816	2.71021386
52.2011792	38.2370004	54.2925259	45.4996249	32.7714405	39.342415	64.081218
17.2689701	25.6516565	16.9930399	25.8893175	23.2321214	25.2466041	21.1037731
0.15770749	0.28056499	0.37867498	0.12372434	0.1825707	0.05034218	0.3381617
0.05256916	0	0	0.06186217	0.1825707	0.07551327	0.07790077
0.10513833	0.16032285	0.23667187	0.15465542	0.1825707	0.830646	0.11106577
0	0	0	0.06186217	0.04564267	0	0
0	0	0	0.06186217	0.04564267	0	0
0	0	0	0.06186217	0.04564267	0	0
35.3790469	37.595709	39.2401953	31.3950505	32.6345124	31.866601	29.7754848
0	0	0	0.06186217	0.04564267	0	0
1.20909076	1.08217926	1.08869058	1.36096771	1.78006431	1.83748963	1.16324256
269.627239	310.224721	336.405389	264.337046	268.196357	229.812059	142.168548
127.296229	131.304417	130.832207	120.229124	112.828692	95.7760006	83.8554671
0.07885374	0.04008071	0.18933749	0.06186217	0	0	2.57228333
11.2235164	12.3849404	12.6382776	10.26912	8.39825215	10.4208316	21.8449679
14.5879428	15.3509132	16.5196962	12.496158	13.5102317	12.2834923	8.97420952
50.9658038	55.5518686	48.7544043	59.2020952	60.2483306	61.8201991	35.6261242
10.2247023	10.340824	10.7449027	12.6817445	10.6803859	15.6815896	8.50576519
0	0	0	0.06186217	0.04564267	0	0
11.6440697	13.1865547	14.2949807	10.0526024	12.5517356	8.43231543	5.1127132
3.39071103	2.36476208	2.27204991	4.7015248	2.96677386	3.70015035	1.94049445
3.39071103	2.36476208	2.27204991	4.7015248	2.96677386	3.70015035	1.94049445
172.505709	164.531328	174.143158	146.273097	142.815929	145.186852	302.109798
9.19960358	7.33477053	11.0762433	7.6399778	6.84640121	7.04790543	5.77262867
8.48991987	9.41896762	8.99353088	12.3724337	10.0870311	10.8235691	3.59238809
65.2120471	75.5922252	62.8600474	70.2444923	86.2190126	85.4055112	33.9603818
0.21027665	0.12024214	0.04733437	0.03093108	0	0.07551327	0.18837666
10.8292476	8.49711121	8.75685902	12.0321918	9.76753239	13.8441	12.6044331
4.28438681	3.60726419	3.26607174	2.3507624	3.14934456	2.81916217	3.24924197
23.8401156	18.9581774	18.9810836	24.621143	23.9167616	25.7500259	9.17805265
0	0	0	0.03093108	0.04564267	0.02517109	0
25.6011825	24.2087508	22.5311616	29.3535989	25.4686125	30.8345863	23.2239893
51.5440646	54.9907386	46.813695	56.8513328	62.2109657	60.7630133	25.0359719

0	0	0	0.03093108	0.04564267	0.02517109	0
8.96304234	6.97404411	8.66219027	9.49584286	9.95010309	11.5283596	4.69364628
0	0	0	0.03093108	0.04564267	0.02517109	0
167.301362	160.242692	164.581615	185.864885	183.711766	216.723092	50.8528495
13.19486	13.8278461	12.8276151	17.1667517	14.6056559	16.4870645	280.714399
50.3349739	48.6179052	49.3224167	51.5002552	68.053228	56.9118364	43.824934
1.55079032	1.88379352	2.4613874	1.20631228	1.09542419	1.58577872	1.13358767
2.23418944	2.7254885	2.27204991	2.66007324	4.01655538	3.37292617	0.68912028
128.715596	120.602866	98.3608272	157.47015	124.148075	138.541684	139.526711
115.468167	137.837573	146.594553	119.053743	117.621173	95.3984343	174.168321
6.67628374	6.37283341	7.9048403	5.72225058	5.75097702	4.95870489	3.1722663
28.019364	33.4673956	30.0573269	27.3430785	25.6511832	24.5166425	29.383348
2.89130398	2.08419709	2.60339052	2.81472866	4.06219805	3.70015035	5.98965213
14.1673895	13.7076039	14.2949807	16.9811652	15.8836508	17.6952769	17.9036766
14.2988124	16.9541417	18.649743	21.8064144	17.4355017	21.621967	10.8440959
55.2501906	55.1510614	42.3169295	60.6558562	56.0948472	66.0237713	48.6410541
25.3909059	23.0464101	21.442471	28.271011	29.7590239	25.1710908	16.0642986
5.88774629	5.57121914	5.82212789	4.76338697	4.5186248	4.22874326	11.3871831
0	0	0	0.06186217	0	0.02517109	0
108.660461	122.646983	129.222838	113.207768	101.281095	87.9226203	72.7745641
0.05256916	0.16032285	0	0.03093108	0	0	0.07307623
0	0	0	0	0.04564267	0.05034218	0
0	0	0	0	0.04564267	0.05034218	0
0.18399207	0.04008071	0.09466875	0.03093108	0	0.05034218	0.13880653
31.9883359	33.547557	47.6657137	32.4467074	58.376981	57.088034	21.6431749
0	0	0	0	0.04564267	0.05034218	0
1.94505904	1.60322853	3.40807486	4.1138342	2.69291781	3.27224181	1.79034017
3.1541498	2.36476208	2.65072489	4.42314504	3.74269933	3.12121526	10.2617686
0	0	0	0.06186217	0	0.02517109	0
0	0	0	0	0.04564267	0.05034218	0
0	0	0	0	0.04564267	0.05034218	0
0	0	0	0	0.04564267	0.05034218	0
19.9237129	18.6776124	16.7090337	16.3316125	16.7508616	11.5535307	22.809906
112.130025	113.428419	124.110726	103.588201	103.106802	91.4717441	109.909462
2.60217358	2.20443923	7.19482471	2.47448674	1.87134966	2.84433326	2.13781103
50.9658038	55.1911422	57.0852539	61.1816846	62.4848217	66.4013376	44.8369057
4.81007844	4.28863632	4.40209669	5.876906	5.15762224	6.3179438	2.45939316
0	0	0	0.06186217	0	0.02517109	0
0	0	0	0.06186217	0	0.02517109	0
0.13142291	0	0.09466875	0.15465542	0.27385605	0.20136873	0.1062927
102.904137	102.326061	79.1904061	128.333068	111.961481	102.647708	32.0552879
13.6416979	16.4731731	19.4544273	14.2282987	12.7799489	12.7869141	15.2259928
25.6800363	28.4573064	22.9571709	31.1785329	24.8296151	38.9396775	39.6141365
6.75513748	7.29468981	7.66816844	10.26912	7.30282796	9.79155433	2.41798397
5.20434717	4.92992773	5.30144978	6.18621685	6.80075853	6.56965471	7.93321028

0	0	0	0.06186217	0	0.02517109	0
0	0	0	0	0.04564267	0.05034218	0
13.3262829	14.8298639	17.9870618	12.7126756	12.8712343	11.7548994	12.4394091
7.25454453	8.73759549	8.70952464	10.5784308	9.08289227	11.0501089	3.3249822
0	0	0	0.06186217	0	0.02517109	0
0.15770749	0.12024214	0.14200312	0.06186217	0.04564267	0.05034218	0.34205895
85.2671829	85.6124035	84.5865247	99.4743669	94.1151953	100.130599	113.130249
0	0	0	0.06186217	0	0.02517109	0
0.97252952	0.96193712	0.7100156	1.14445012	1.04978152	1.88783181	2.04588313
0.47312247	0.72145284	0.80468434	0.37117301	0.1825707	0.50342182	0.28538646
89.6829926	91.3439455	75.4509907	99.3506425	94.160838	109.192192	189.970362
0	0	0	0.09279325	0	0	0
0	0	0	0	0.04564267	0.05034218	0
22.867586	24.6496387	27.1225958	20.6928953	22.2736253	19.8348196	22.5411614
5.91403087	4.92992773	5.34878416	5.66038841	7.34847063	8.00440689	7.18069169
0	0	0	0	0.04564267	0.05034218	0
0	0	0	0.06186217	0	0.02517109	0
0	0	0	0	0.04564267	0.05034218	0
117.255519	112.706966	117.957258	106.805034	90.1899253	99.7782041	120.434047
1.05138327	0.76153355	0.80468434	1.48469204	1.27799489	1.20821236	0.86405218
20.6333966	20.3610023	20.7324554	17.3523383	18.8504247	15.505392	24.0729902
31.2523676	40.401359	34.6960955	38.1070958	13.190733	23.9628785	29.3629501
27.3885341	23.2868944	22.199821	29.8484963	28.0246023	28.4433326	21.7912133
5.04663968	5.29065415	6.1534685	6.15528576	7.30282796	7.45064289	8.20396014
0	0.04008071	0	0.12372434	0	0.07551327	0
0	0.04008071	0	0.12372434	0	0.07551327	0
365.381969	376.037252	351.221048	489.731857	437.348109	386.426586	193.157469
3.8638335	5.21049272	3.6920811	2.938453	3.788342	2.7436489	2.2604355
0.78853745	0.44088785	0.80468434	0.5876906	0.31949872	0.25171091	0.6377562
0	0	0	0.03093108	0	0.05034218	0
0	0	0	0.03093108	0	0.05034218	0
0	0	0	0.03093108	0	0.05034218	0
4.12667932	4.12831347	4.44943107	5.01083564	5.79661969	5.53763998	4.00325618
2.91758856	3.16637635	3.64474673	3.43335035	4.79248085	4.80767835	3.67185507
5.67746964	5.37081558	5.4434529	5.96969926	7.02897191	8.02957798	8.45424833
0	0	0	0.03093108	0	0.05034218	0
3.10158063	3.68742562	2.93473113	3.09310842	4.38169677	6.19208835	8.31132074
1.07766785	1.24250211	1.08869058	1.51562313	1.59749362	1.76197636	1.77071217
0	0	0	0.03093108	0	0.05034218	0
19.2928829	19.1185002	17.8450586	24.5592809	19.6263501	23.1825747	13.5792288
1.70849781	2.44492351	2.08271241	3.30962601	1.96263501	3.5742949	1.78249911
0	0	0	0.03093108	0	0.05034218	0
69.864418	71.9047996	74.4569688	64.7078282	63.7171739	57.6166269	31.1571687
21.7110644	20.1605988	23.6198522	24.1262457	25.8337539	27.5875156	26.9632745
96.5432684	95.191694	97.2721366	91.9271823	83.2522387	73.2478743	32.316311

0.97252952	0.6012107	0.85201872	1.26817445	1.32363757	1.13269909	0.39237501
152.765989	151.74558	158.712153	142.313919	132.044258	118.203443	62.794484
51.1235113	43.9685424	56.8959164	56.4182976	59.3811198	63.9345707	70.8653128
14.3776662	16.4330924	15.8096806	13.7024703	12.2778795	12.45969	10.2494098
0.34169956	0.40080713	0.23667187	0.24744867	0	0.17619764	0.27810781
0.07885374	0.08016143	0.09466875	0.03093108	0	0.02517109	0.02348116
0	0	0	0	0	0.07551327	0
0	0	0	0	0	0.07551327	0
0	0	0	0	0	0.07551327	0
0	0	0	0	0	0.07551327	0
146.352551	157.116396	171.303096	152.985143	134.326392	97.6386613	85.2528191
0.47312247	0.88177569	0.61534685	0.43303518	0.41078407	0.30205309	0.12248968
4.02154099	5.05016987	4.16542483	5.01083564	5.84226237	6.29277271	2.62723198
1.20909076	1.48298639	0.80468434	1.42282987	2.09956304	1.76197636	1.63613155
0	0	0	0	0	0.07551327	0
41.2667932	44.4094303	32.0927049	38.4782688	28.0246023	29.7270583	29.1208886
21.237942	23.1666523	24.613874	26.9409744	24.1906176	30.6080465	48.9727836
0	0	0	0	0	0.07551327	0
1.91877446	0.96193712	1.46736556	2.25796915	1.91699234	2.24022708	2.06348004
54.7507836	64.4097062	64.8480911	50.8197714	60.5678294	39.6696392	20.77414
0.78853745	0.9218564	0.89935309	2.07238264	0.77592547	1.25855454	1.16639208
40.6622478	46.6539502	42.884942	35.6635401	37.9290627	38.3104002	34.7235804
7.46482119	0.6012107	4.82810605	8.25859949	2.55598978	18.8028049	4.3546046
389.484931	364.413845	406.22359	336.282748	321.643929	322.492016	439.733439
8.85790402	9.73961332	7.3841622	14.0736433	12.9168769	7.6771827	5.52280507
13.3525675	12.8258282	11.8335933	12.4342959	8.80903622	9.21261925	10.6827987
0.13142291	0.20040357	0	0.27837976	0.1825707	0.30205309	0.29689328
0.23656123	0	0.04733437	0.21651759	0.3651414	0.25171091	0.40108368
75.2001881	79.5201351	82.8351529	75.2243968	64.7213128	63.4059778	56.2982147
11.8280617	11.8238104	15.6203431	10.8877416	10.9998846	9.79155433	27.851961
7.54367493	5.69146128	6.39014036	7.98021973	7.25718528	9.71604106	6.27546172
159.468557	170.062466	182.616011	146.304028	162.168423	113.874015	34.9942663
9.75157979	8.89791834	10.1295558	12.0321918	10.5891005	12.5855454	3.83938332
2.33932777	1.76355138	3.21873737	1.60841638	2.14520571	1.25855454	3.2262291
7.67509784	5.81170342	6.43747474	9.00094551	7.71361203	8.35680216	4.59448699
72.5717299	53.1470258	96.8461273	50.5723227	75.0365573	42.8411966	80.4494142
46.7077016	35.7119155	47.1923699	49.0257685	70.1984337	43.4704739	54.0301121
1.26165992	0.84169498	1.08869058	1.45376096	1.50620827	1.73680527	1.15825141
23.5772697	22.2848766	21.6791429	26.7244568	24.7839724	27.6881999	31.7990374
75.9098718	60.5619577	59.0259632	70.7084585	77.5469044	84.1469567	33.3846588
6.28201501	5.85178414	5.96413101	7.98021973	6.84640121	7.72752489	7.86158206
68.4450506	67.7764861	81.9357998	82.4932016	78.8248993	93.6364579	27.9932539
14.6667966	15.2707518	15.857015	13.3622284	12.0953088	12.5100321	13.4798469
0	0	0.09466875	0.06186217	0.13692802	0.10068436	0
0.49940705	0.32064571	0.5206781	0.71141494	0.5477121	0.90615927	2.39106572

2.18162028	1.76355138	1.32536245	2.72193541	1.87134966	3.0205309	2.0489397
45.2883342	50.8223444	50.6951136	45.1903141	42.7215435	37.8824917	35.6044681
35.4841852	29.2990014	29.8679894	35.6016779	37.0162092	38.8641642	49.6083215
33.3025649	28.8180328	28.2112863	33.9932616	42.2651168	32.143483	25.2329125
9.54130314	8.25662693	7.33682782	10.640293	8.90032157	12.182808	6.28019135
0.44683789	0.80161427	0.37867498	0.71141494	1.36928024	0.72996163	1.33645528
14.8507886	16.4731731	15.3836712	18.4039951	20.0371342	17.4939081	4.83415408
136.443263	127.616991	115.637873	162.821227	160.707858	129.429749	89.8084664
0.15770749	0.12024214	0.33134061	0.40210409	0.22821337	0.47825073	0.32809736
0.02628458	0.04008071	0.14200312	0.09279325	0.31949872	0.15102655	0.03778325
4.25810223	3.76758705	2.84006238	5.41293974	3.9709127	5.16007362	5.6039524
0.05256916	0.12024214	0.28400624	0.03093108	0.04564267	0.05034218	0.04088181
12.2223305	10.0201783	9.8455496	11.2279836	12.7799489	16.3108669	4.44585099
1.52450574	1.64330924	1.42003119	0.95886361	1.36928024	0.88098818	0.66366741
0.23656123	0.48096856	0.23667187	0.55675952	0.7302828	0.42790854	0.0904601
4.78379386	4.12831347	5.49078728	5.35107757	5.93354771	7.34995852	6.70709981
1.60335948	1.16234068	1.37269682	1.88679614	2.00827769	1.98851618	1.83305238
184.09721	192.066778	172.486455	184.844159	147.836623	99.753033	59.830873
0.42055331	0.48096856	0.42600936	0.61862168	0.50206942	1.03201472	0.32684668
85.6877362	75.9930323	72.2322533	70.6465964	67.6880866	62.4746475	38.8296151
3.81126434	4.76960488	3.97608734	3.00031517	3.69705665	2.81916217	2.97433081
7.25454453	6.93396339	5.96413101	5.56759516	3.83398468	5.99071962	16.4188298
6.99169872	7.09428625	7.47883094	4.88711131	5.61404899	6.49414144	11.0573266
5.86146171	5.77162271	6.43747474	4.97990456	4.56426747	4.6818229	4.94840566
168.983575	170.944242	201.644429	160.532327	162.670493	126.484731	81.916037
100.85394	73.2675438	146.594553	122.889198	168.375827	126.811956	95.9355672
258.929414	289.823638	269.427251	334.395952	304.482283	324.782585	164.971114
24.2080997	20.7217288	22.0104835	18.9916857	20.2197049	17.0659996	16.0419349
0.13142291	0.48096856	0.18933749	0.09279325	0	0.17619764	0.10422542
4.17924848	6.6533984	6.91081847	4.29942071	3.37755793	5.16007362	6.7048041
1.15652159	1.64330924	1.65670306	0.30931084	1.04978152	1.25855454	1.50666223
0.07885374	0.08016143	0.09466875	0	0	0.05034218	3.21535417
1.20909076	1.08217926	1.18335933	0.74234602	0.91285349	0.72996163	3.26503955
8.83161943	8.33678836	8.23618091	9.65049828	9.08289227	13.2651649	4.25003541
41.3982161	39.3993411	40.8968983	44.6335545	49.5679448	46.3903204	18.7319505
2.33932777	2.80564993	1.7513718	1.94865831	1.78006431	0.57893509	1.20465806
29.8855693	29.0585171	22.3891585	31.9827411	30.169808	34.8619608	16.6478037
0.44683789	0.56112999	0.47334373	0.52582843	0.82156815	1.05718582	0.66863634
27.835372	27.4552886	32.5187143	21.5280346	23.5059775	28.3174772	11.4841823
2.65474275	3.08621492	3.02939988	2.41262457	2.09956304	1.93817399	4.04926148
11.0132397	10.6614697	11.0762433	14.0736433	11.4106687	14.2216663	21.216274
0	0.04008071	0	0.06186217	0.04564267	0.07551327	0
0.05256916	0.12024214	0.18933749	0	0.04564267	0.05034218	0.06815801
1.15652159	1.08217926	0.99402183	1.5774853	1.50620827	1.56060763	0.67225271
0.05256916	0.08016143	0.04733437	0	0	0.02517109	0.09128046

0.05256916	0.08016143	0.04733437	0	0	0.02517109	0.04723253
0.05256916	0.08016143	0.04733437	0	0	0.02517109	0.07569992
21.4482186	20.0804373	18.6970774	21.0022062	21.3607718	32.1686541	13.740398
15.2187728	12.585344	23.4305147	17.6616491	22.0454119	27.4113179	14.4597286
55.1450523	53.2271872	59.2153007	62.6973077	61.6176109	70.4538832	123.098778
81.9027564	84.2496593	84.3971872	80.5136122	71.7959274	63.5318333	29.6992547
0	0	0.04733437	0.06186217	0.04564267	0.07551327	0
14.824504	14.3889761	12.6382776	17.8163045	16.9334323	15.7319318	8.06519791
1.05138327	1.00201783	0.7100156	1.51562313	1.59749362	1.107528	0.56384992
0.02628458	0.04008071	0.04733437	0.18558651	0.13692802	0.05034218	0.0930638
8.96304234	9.77969404	9.46687461	9.34118744	13.4189464	12.4848611	13.060581
5.41462382	5.93194556	5.34878416	4.73245589	4.8837662	3.09604417	7.32297356
0.86739119	1.00201783	0.85201872	0.52582843	0.5477121	0.67961945	0.50647584
7.5962441	9.29872548	7.28949345	6.61925202	7.39411331	4.98387599	6.83514053
0.05256916	0.16032285	0.04733437	0.09279325	0.27385605	0.276882	0.0684482
0.63082996	0.56112999	0.47334373	0.49489735	1.09542419	1.20821236	0.83742268
0.89367578	1.16234068	0.7100156	0.92793253	1.36928024	2.01368727	0.46510122
0.02628458	0	0	0.03093108	0.09128535	0.07551327	0.01665987
0.9988141	0.96193712	1.42003119	2.1651759	1.91699234	1.08235691	1.52158728
176.264405	162.206647	151.754	153.325385	138.251662	121.098118	113.574743
8.27964322	9.73961332	8.56752152	9.34118744	12.0040235	11.629044	11.4058172
4.36324055	5.450977	5.68012477	5.50573299	6.29868911	8.25611779	4.33413554
0.44683789	0.52104927	0.47334373	0.74234602	0.68464012	0.80547491	0.85274604
98.830027	106.374213	106.028996	109.372314	120.633589	134.363283	121.321944
166.039702	193.509684	205.715185	176.554629	164.67877	118.60618	130.326547
8.75276569	10.060259	12.9222838	12.124985	11.9127381	15.6312474	5.47679252
0.02628458	0	0.04733437	0.06186217	0.04564267	0.15102655	0.02781448
55.854736	62.6060741	64.3747474	53.8510176	49.248446	54.042332	53.2758488
9.12074983	8.29670764	7.28949345	8.78442792	10.2239591	12.0569525	14.1589835
5.96660003	5.49105772	4.82810605	5.876906	6.52690249	8.60851307	3.44529335
0.60454538	0.48096856	0.7100156	0.86607036	1.04978152	0.85581709	0.45579751
24.5235147	34.0285256	31.2880206	23.6004173	29.7590239	19.5831087	21.7859509
60.7962374	58.9587292	55.5232196	81.5652691	71.1569299	56.6349544	59.735857
0.23656123	0.20040357	0.04733437	0.49489735	0.1825707	0.35239527	0.37630933
7.3071137	7.21452839	8.14151217	6.12435468	5.65969167	6.39345707	6.67315012
1.34051366	1.32266354	4.49676544	0.95886361	1.82570699	1.30889672	2.20703987
165.592864	164.811893	168.936377	203.835845	166.276264	228.427649	154.572539
0.13142291	0	0	0.12372434	0.45642675	0.05034218	0.14668587
41.214224	55.63203	58.4106164	43.5509666	46.0534588	40.6764828	23.4878888
0.13142291	0	0.04733437	0	0	0.02517109	0.21740055
0	0.04008071	0	0.09279325	0.04564267	0.05034218	0
3.1541498	3.36677991	2.9820655	3.86638553	4.3360541	4.17840108	1.59011951
8.0167974	8.89791834	9.79821522	7.6399778	6.61818784	7.19893198	13.8124369
24.6023684	30.7819878	30.578005	22.022932	25.4686125	24.9697221	70.6472183
4.41580972	4.40887846	3.40807486	5.16549107	5.65969167	5.08456035	4.79946246

51.4914954	51.3834744	74.0782938	51.6239796	47.6509524	46.9944266	33.901393
6.41343792	9.13840262	13.1116213	8.25859949	4.06219805	7.75269598	1.28005615
85.5825978	101.965335	88.0892683	105.753377	122.87008	97.2107528	19.0713055
12.6954529	11.9039718	10.3662277	13.1766419	14.4230852	14.7754303	9.14076553
10.9606705	10.621389	10.4608964	11.382639	12.5060929	15.2788521	11.6896485
32.6454504	37.9163547	36.5894704	34.5809522	28.5723144	27.3861468	21.8880234
7.78023617	8.89791834	5.49078728	5.1036289	4.60991015	6.99756325	2.70190159
0.02628458	0.08016143	0.09466875	0	0	0.02517109	0.02137158
6.33458418	6.73355983	4.78077168	7.73277106	7.48539866	7.34995852	1.95484449
21.8950565	18.5172895	17.5610524	23.1055199	23.7341909	22.3519287	31.07181
12.6165992	8.53719192	6.62681223	11.7228809	11.8214528	12.7617431	10.5891595
23.8664001	24.4492351	27.5012707	23.1055199	21.7715558	19.2307134	17.2498764
10.618971	9.21856405	6.6741466	12.6817445	10.2239591	10.8990823	4.7073857
17.7683772	17.194626	24.1405303	18.8988925	12.8712343	15.2788521	14.558469
0.05256916	0.04008071	0.09466875	0	0.04564267	0	0.18114671
26.705135	24.7298001	21.7738116	29.3226678	26.244538	30.6332175	22.7730905
0.65711454	0.44088785	0.09466875	0.52582843	0.59335477	1.18304127	1.08042815
9.64644146	3.28661849	1.51469994	4.14476529	1.14106687	2.26539818	30.3741308
0.05256916	0.08016143	0.23667187	0.03093108	0.04564267	0.02517109	0.05136349
153.843656	179.160788	174.143158	174.822488	231.271433	196.082798	127.072707
0.42055331	0.36072642	0.61534685	0.34024193	0.31949872	0.10068436	1.09926638
20.6333966	21.242778	16.23569	19.3319276	14.2405145	13.2399938	9.62644173
0.15770749	0.08016143	0.14200312	0.09279325	0.04564267	0	0.2076655
0.63082996	0.36072642	0.18933749	0.09279325	0.3651414	0.17619764	0.61982731
49.7041439	54.0288015	56.6119102	54.0366041	43.5431117	34.5347366	28.5256145
40.5045403	39.2390183	44.92032	36.2512307	36.6054251	34.8871319	26.0782146
0.34169956	0.32064571	0.23667187	0.71141494	0.27385605	0.57893509	0.18483133
6.41343792	7.61533552	5.82212789	5.72225058	5.70533434	3.97703235	6.07234069
39.0063192	31.3431178	34.9327673	38.571062	41.2153353	43.3949606	30.9541718
14.0622512	17.2747874	19.0757523	13.3931595	14.0123011	13.9699554	28.0851344
1.13023701	0.64129141	0.80468434	1.26817445	1.18670954	1.43475218	0.59364633
88.8156014	73.0270596	73.3682782	68.2339718	71.2938579	62.0467389	82.4956852
0.31541498	0.20040357	0.37867498	0.09279325	0.1825707	0.17619764	0.54229445
2.81245024	2.88581135	3.59741235	2.938453	4.60991015	5.13490253	7.80142621
0.81482203	1.28258282	1.56203431	0.49489735	1.18670954	0.60410618	1.70386289
6.67628374	5.69146128	5.68012477	4.67059372	4.29041142	5.23558689	4.88603026
13.9045437	12.3849404	12.7802807	15.8676462	14.4230852	16.5625778	13.9591494
0.44683789	0.24048428	0.85201872	0.77327711	0.59335477	1.15787018	0.55578059
8.0167974	8.73759549	8.85152776	7.85649539	6.48125981	6.36828598	5.82872524
33.5391262	32.9062656	25.5605615	25.9511797	30.169808	18.4755807	8.76477658
27.519957	22.3249573	23.9985271	29.1370813	25.3316845	33.3516954	42.9123749
0.52569163	0.64129141	0.66268122	0.77327711	1.00413884	0.98167254	1.46485384
50.5452505	46.2130624	39.9502109	57.5936788	49.0658753	54.1430164	48.6477267
69.8907026	70.0610868	74.0782938	78.9979891	75.2647706	92.9820095	48.7019466
24.8126451	25.2909301	20.1171086	32.7250871	26.6096794	24.8690377	8.14406851

9.51501856	7.93598123	7.57349969	10.9805349	8.8546789	11.3018198	3.76198516
24.2343843	20.4812445	27.1699301	32.9106736	23.6429055	29.9787692	13.2228213
86.6865503	91.5443491	100.632877	95.4533259	72.4805675	66.5271931	66.5050991
90.6029529	101.12364	92.8700399	82.8643746	87.1318661	78.156237	84.6378451
0.2891304	0.12024214	0	0.30931084	0.1825707	0.50342182	0.33147981
0.23656123	0.32064571	0.28400624	0.09279325	0.1825707	0.15102655	0.19500126
0.49940705	0.28056499	0.75734997	0.24744867	0.3651414	0.25171091	0.50054674
0.21027665	0.28056499	0.28400624	0.15465542	0.31949872	1.15787018	0.44197308
2.8650194	2.60524636	2.13004679	3.49521252	3.65141398	3.09604417	1.98850272
1.55079032	1.16234068	1.79870618	2.3507624	1.68877897	2.24022708	1.51220324
13.4577058	13.4671197	16.7090337	19.6103074	14.1492292	19.6334509	73.6582252
34.4853711	32.4653777	37.9621672	36.5605416	36.2402837	54.6212671	11.6357597
12.9845833	12.5452633	13.064287	14.0117812	14.7425839	17.7959612	13.4837433
6.78142207	6.49307555	5.72745914	8.2276684	6.93768656	8.40714434	4.83991468
0.42055331	0.72145284	0.85201872	0.46396626	0.50206942	0.15102655	0.37011271
5.09920884	4.76960488	4.26009358	3.30962601	4.29041142	3.44843944	2.40655366
0.05256916	0.12024214	0.04733437	0.18558651	0.13692802	0.17619764	0.0503974
0	0.20040357	0.04733437	0.27837976	0	0.47825073	0
1.70849781	1.64330924	1.84604055	2.28890023	3.01241653	1.98851618	1.94145862
16.6644248	16.5132539	17.5610524	14.5994718	14.9707973	13.1393094	4.52454676
29.5964389	30.8621492	31.4300237	27.0646987	25.8337539	26.5051586	27.2832614
0.15770749	0.08016143	0.04733437	0.03093108	0.04564267	0.02517109	0.12926047
55.3290444	60.4016349	66.6941316	56.0780557	51.8957212	48.4291788	45.8558301
11.2235164	9.85985546	12.4016057	12.6508134	12.2322368	16.084327	7.34594023
272.544827	198.35945	247.700774	306.124941	257.242115	299.082901	638.086265
0.02628458	0.04008071	0	0.06186217	0.04564267	0.12585545	0.06762049
0.05256916	0	0	0	0	0	0.08389704
0.02628458	0	0	0.03093108	0.04564267	0.10068436	0.03141528
0	0.04008071	0.04733437	0	0	0	0
0.02628458	0.04008071	0	0	0	0	0.07035786
0	0.08016143	0.09466875	0.06186217	0.22821337	0.15102655	0
0.2891304	0.52104927	0.47334373	0.64955277	0.82156815	0.553764	0.77648509
0	0.08016143	0	0	0	0	0
0.05256916	0	0	0	0	0	0.05628629
0	0	0.09466875	0	0	0	0
0.02628458	0	0	0	0.09128535	0.10068436	0.14040848
1.20909076	1.40282496	1.2306937	1.48469204	2.09956304	1.76197636	1.01409867
14.4302353	10.7015504	10.3188933	14.8778515	14.9251546	13.9447843	19.5918917
1.70849781	1.2024214	0.94668746	0.68048385	1.14106687	0.88098818	2.37093614
0.02628458	0.04008071	0.09466875	0.12372434	0.04564267	0.20136873	0.09923933
0	0.04008071	0.04733437	0	0	0	0
0.02628458	0	0.04733437	0	0	0	0.0595436
0.02628458	0.04008071	0	0	0	0	0.0534556
8.77905027	10.7817119	7.57349969	10.3309821	10.1326738	13.0637961	9.37519242
0	0.04008071	0.04733437	0	0	0	0

10.0144256	10.4209854	7.62083406	8.815359	14.5143706	12.6610587	13.6192322
0.05256916	0	0	0	0	0	0.12861417
0.05256916	0	0	0	0	0	0.03797289
0.05256916	0	0	0	0	0	0.05962641
0.13142291	0.04008071	0.28400624	0.40210409	0.22821337	0.25171091	0.12648915
0.26284582	0.04008071	0.18933749	0.34024193	0.31949872	0.32722418	0.43043563
4.81007844	3.64734491	5.39611853	4.5159383	6.75511586	6.79619453	1.79475313
0.02628458	0	0.04733437	0	0	0	0.05538939
0.02628458	0.04008071	0	0	0	0	0.02256389
0.05256916	0	0	0	0	0	0.04685398
0.05256916	0	0	0	0	0	0.04407614
0	0.04008071	0.04733437	0.03093108	0.09128535	0.15102655	0
0.18399207	0.40080713	0.28400624	0.12372434	0.1825707	0.12585545	0.21283668
1.10395243	1.24250211	0.80468434	1.02072578	2.00827769	1.78714745	1.22046882
0.02628458	0.04008071	0	0	0	0	0.05336687
0.05256916	0	0	0	0	0	0.04581908
30.8580989	33.9483641	31.9980362	29.1061503	29.8046666	24.2649316	77.6713126
0.02628458	0.04008071	0	0	0	0	0.04419731
138.966583	154.831795	159.611506	150.943691	112.189694	117.67485	59.0365601
754.23607	920.573822	793.229424	832.293614	580.255324	606.321236	244.798588
4.10039474	2.80564993	2.84006238	2.3507624	4.1534834	8.28128888	7.39816003
6.93912955	5.81170342	8.09417779	5.78411275	5.38583562	5.33627126	8.99685745
55.7495977	43.9685424	45.0149888	46.1182466	41.1240499	36.2211997	57.42961
223.918351	109.620751	324.051118	182.153155	550.861441	285.21363	348.715495
0	0.04008071	0.04733437	0	0	0	0
139.544844	148.699446	127.61347	168.172305	155.048166	162.353536	89.5844412
1.41936741	0.80161427	0.89935309	0.89700144	0.5477121	0.60410618	1.13557309
2.81245024	2.96597278	3.83408422	2.38169349	2.55598978	2.29056927	2.86821922
348.139284	313.912146	319.270346	329.663496	546.205888	346.530408	208.966467
0.52569163	0.64129141	0.37867498	0.30931084	0.41078407	0.22653982	0.30542429
0.65711454	0.64129141	0.66268122	1.23724337	0.82156815	0.85581709	0.87184224
0.86739119	0.9218564	0.94668746	0.46396626	0.68464012	0.67961945	0.63746883
7.33339828	6.37283341	5.72745914	8.69163467	6.70947319	8.70919743	8.32560383
4.07411016	2.96597278	2.88739676	4.48500721	4.19912608	4.4049409	5.21863766
8.38478155	9.94001689	8.18884654	6.43366552	7.66796935	7.47581398	5.6248861
1.68221323	1.68338996	1.79870618	1.94865831	2.69291781	2.39125363	3.31373054
8.80533485	9.29872548	9.32487149	7.98021973	8.21568145	6.26760162	9.49443496
71.625485	68.0570511	74.1256282	61.7693752	68.4183694	55.0743467	41.029455
0	0.12024214	0.09466875	0	0	0.02517109	0
19.2928829	18.3569667	17.4663837	18.744237	21.9541265	25.4228017	7.6875243
11.433793	13.3468775	14.1529775	11.2279836	9.58496169	11.07528	15.8580393
0	0.04008071	0.04733437	0	0	0	0
115.179037	118.799234	118.856611	109.712556	107.488499	84.3734965	32.109576
22.3418944	22.2447959	21.3004679	22.9508645	25.3316845	29.374663	15.2514009
12.3537534	13.5072004	15.0049963	14.1664366	15.4728667	19.6586219	8.99265968

7.3071137	6.37283341	6.86348409	7.94928865	8.99160692	8.05474907	8.83705842
35.0899165	38.3171619	33.0393924	31.9208789	31.5390882	28.4936748	32.8801058
36.7458451	32.986427	30.8146769	37.1482322	37.0162092	43.2691051	14.8670915
54.2250919	58.4376799	59.6413101	52.5828432	47.422739	49.3101669	22.8418583
0.07885374	0.08016143	0.14200312	0.03093108	0	0.05034218	0.09913733
0.05256916	0	0	0	0	0	0.09449976
0.05256916	0	0	0	0	0	0.21507386
268.470717	256.236	213.004679	297.154926	290.333054	277.611961	272.884316
7.49110577	5.450977	5.96413101	7.20694262	8.26132413	8.38197325	5.55379356
21.8687719	20.8018902	29.44198	23.507624	29.4395252	34.7612764	15.6168982
0.44683789	0.52104927	0.28400624	0.24744867	0.13692802	0.30205309	0.52685321
113.943661	130.542883	165.954312	96.6905693	139.75787	95.1970655	83.6397259
0	0.04008071	0.04733437	0.06186217	0.13692802	0.07551327	0
11.8280617	9.94001689	10.0822215	13.949919	9.17417762	17.5694214	37.7783127
14.0359666	14.1484918	17.3717149	12.6817445	12.2778795	12.8875985	22.7869824
0.15770749	0.32064571	0.18933749	0.43303518	0.3651414	0.35239527	0.15729413
488.314958	496.720279	542.641253	428.086206	477.650591	385.319058	278.549078
33.8545412	30.2609385	26.4125802	32.7869493	39.2983429	34.8619608	13.1014748
55.4341827	44.2090267	45.2516606	59.6351304	54.5429963	55.5022553	24.8896382
0	0.08016143	0	0	0	0	0
27.519957	27.6156114	28.163952	29.1061503	35.5100009	32.3700228	14.5044197
0	0	0.09466875	0	0	0	0
0.76225287	0.68137213	0.61534685	0.49489735	0.3651414	0.45307964	2.33404933
10.0144256	13.5873618	12.8276151	9.74329153	7.48539866	11.629044	8.14527884
182.099582	185.293137	180.864639	161.831433	172.894452	129.756973	77.1997008
0.05256916	0.04008071	0.04733437	0.15465542	0.09128535	0.12585545	0.07455894
121.224491	117.596813	148.061919	121.404506	99.5923163	105.869608	151.550469
131.896031	137.717331	159.990181	131.580832	116.114965	120.393327	148.637929
0.02628458	0	0.04733437	0	0	0	0.02913778
5.41462382	4.72952416	10.6028996	3.46428143	6.66383051	4.32942762	2.08848481
19.5031596	15.5913975	17.5610524	16.6099922	13.0081623	14.0202976	24.2212466
5.70375422	5.89186485	5.25411541	4.14476529	5.20326492	4.1280589	5.64965064
23.7875464	26.6135936	23.9511928	23.4766929	20.0371342	20.0110172	22.9306188
0.15770749	0.24048428	0.33134061	0.43303518	0.1825707	0.62927727	0.14117911
22.0790486	21.7638273	26.1759083	20.6310332	19.9914915	18.7776338	12.7913687
51.8857642	49.3794387	50.6477792	43.4581733	45.3688187	44.4269753	31.1438132
0	0	0.09466875	0	0	0	0
0.10513833	0.04008071	0	0.12372434	0.13692802	0.17619764	0.20254199
0.02628458	0.04008071	0.04733437	0.09279325	0.13692802	0.10068436	0.02085861
13.0634371	13.0663125	10.8869058	14.8159893	12.3235222	18.1986987	5.25149859
0	0.04008071	0.04733437	0	0	0	0
12.248615	12.4651018	12.5436089	13.8571257	14.9707973	15.253681	9.11408177
0.05256916	0	0	0	0	0	0.07651051
4.62608637	3.68742562	4.11809046	5.1036289	4.79248085	5.94037744	2.82527376
54.5405069	61.4838141	72.5635939	55.2429164	59.7919039	42.8160255	30.1757571

7.80652075	7.73557766	8.80419339	8.13487515	9.72188972	12.4848611	4.49710472
15.5079032	19.1185002	17.9397274	27.157492	16.1575069	21.2695718	42.6784918
69.1284497	68.7384232	70.1968753	66.6874176	56.82513	56.7608098	45.8713396
134.393066	125.29231	116.537226	153.696558	136.380312	148.861831	60.0551812
28.8604706	26.6135936	29.3473113	31.2713262	32.0411577	34.9626452	20.4486468
6.83399123	7.61533552	7.24215908	9.12466985	8.17003878	8.86022397	5.31970782
71.3889238	83.16748	64.5167505	97.5566397	72.8913515	87.6205672	74.9283734
0.05256916	0.08016143	0	0.12372434	0.22821337	0.07551327	0.07221458
0.21027665	0.12024214	0.14200312	0.24744867	0.68464012	0.17619764	0.19628259
0.02628458	0	0.04733437	0	0	0	0.01034541
0	0	0.09466875	0	0	0	0
43.9741051	58.5980028	53.9611853	39.4062013	52.4890759	39.896179	27.480396
223.024675	248.901229	230.613066	223.755463	207.537242	151.353769	80.7168051
14.4565199	13.6675232	13.3956276	17.7853734	15.1990107	16.5374067	5.85093397
151.267767	163.088422	153.221366	141.540641	138.799374	120.947091	112.42019
0.05256916	0	0	0	0	0	0.18639734
28.3873482	25.2909301	30.0099925	26.4151459	21.7259132	23.4594567	25.992384
0.02628458	0.04008071	0	0	0	0	0.04001685
20.6859658	21.3229395	22.3891585	17.6925802	19.8089208	17.4939081	13.5974408
1.20909076	1.04209854	0.94668746	1.5774853	1.50620827	1.48509436	1.139055
88.8156014	84.0893364	95.8521055	82.5859949	84.3020202	65.671376	67.8406602
0.86739119	0.64129141	0.61534685	1.02072578	0.82156815	1.38441	1.20166124
5.04663968	5.25057344	3.6920811	2.87659083	3.46884328	4.25391435	1.95952389
0	0	0.09466875	0	0	0	0
0.02628458	0	0.04733437	0	0	0	0.04795457
0	0.04008071	0.04733437	0	0	0	0
0	0.08016143	0	0.03093108	0.13692802	0.12585545	0
0.02628458	0	0.04733437	0	0	0	0.06335673
0.05256916	0	0	0	0	0	0.08523139
0.2891304	0.12024214	0.33134061	0.15465542	0.04564267	0.12585545	0.08729827
0.73596829	0.68137213	0.33134061	0.86607036	0.82156815	1.03201472	1.56031051
12.879445	11.182519	20.0224398	17.0739585	19.2155661	18.8028049	6.64357387
21.1853728	18.9581774	20.8271241	17.630718	14.9707973	18.8783181	14.0388703
0.02628458	0	0.04733437	0.12372434	0.04564267	0.07551327	0.01526396
0	0.08016143	0	0.03093108	0.13692802	0.12585545	0
0.94624494	0.88177569	0.5206781	1.20631228	1.09542419	1.20821236	0.61570612
38.2966355	46.3733852	44.3996419	34.0241926	38.7962735	38.7383088	27.6224707
42.9227218	43.0867668	37.5834922	40.395996	35.3730729	29.0222677	22.7252688
4.25810223	5.09025058	4.35476232	5.93876817	5.61404899	5.66349544	7.64324835
60.375684	49.8604073	55.3812165	63.7489646	65.7710943	62.6005029	59.5138479
0	0.04008071	0.04733437	0	0	0	0
0.36798414	0.32064571	0.28400624	0.27837976	0.04564267	0.15102655	0.23592745
21.2116574	20.7618095	16.7563681	24.1571768	18.1201419	30.5577043	16.5063029
3.73241059	3.88782919	3.6920811	4.02104095	4.47298212	6.06623289	2.14508007
0.02628458	0.08016143	0	0.03093108	0.1825707	0.15102655	0.03504473

0.07885374	0.16032285	0.14200312	0	0.04564267	0.07551327	0.04956867
0.02628458	0	0.04733437	0.09279325	0	0.15102655	0.06267747
0.10513833	0.08016143	0.04733437	0.12372434	0.1825707	0.25171091	0.12596882
21.9213411	18.036321	15.7623462	20.7856886	21.4064144	25.4228017	13.584627
146.957096	159.200593	133.340929	158.89298	172.164169	183.119686	212.243744
0.05256916	0	0	0	0	0	0.05484613
0.05256916	0.12024214	0.23667187	0.06186217	0	0.05034218	0.09923933
9.62015688	7.21452839	6.6741466	11.6610188	7.71361203	10.798398	7.43411008
0.02628458	0.12024214	0.14200312	0	0	0.05034218	0.03115653
2.47075067	2.7254885	3.171403	2.62914216	4.29041142	4.30425653	2.83929819
32.4088892	32.2248935	24.2825334	30.9001531	31.8129443	45.7610431	26.6792173
50.9395192	50.2612144	36.0687923	44.6954167	63.2607472	59.3534322	27.4630074
0	0.08016143	0	0	0	0	0
3.28557271	2.96597278	2.27204991	1.60841638	1.91699234	2.66813563	2.32189065
4.25810223	3.40686063	2.88739676	4.79431806	4.83812352	4.25391435	6.9497982
0.02628458	0.04008071	0	0	0	0	0.04236303
0	0.08016143	0	0	0	0	0
0.02628458	0.04008071	0	0	0	0	0.03479821
0.05256916	0	0	0	0	0	0.0512203
0.07885374	0	0.09466875	0.03093108	0	0	0.17012456
0	0	0.09466875	0	0	0	0
1.26165992	2.68540779	0.5206781	0.68048385	0.91285349	0.98167254	5.26747441
0.05256916	0	0	0	0	0	0.23426988
0.05256916	0	0	0	0	0	0.03438881
0.02628458	0.04008071	0	0	0	0	0.03866932
0.02628458	0	0.04733437	0	0	0	0.0448133
0.02628458	0.04008071	0	0	0	0	0.09773113
62.8464347	62.1651863	67.3568129	59.3876817	54.862495	52.3306978	44.6971617
0.02628458	0	0.04733437	0.09279325	0.04564267	0.10068436	0.19253618
0.10513833	0.04008071	0.04733437	0.09279325	0.13692802	0.276882	0.12025635
0.05256916	0.12024214	0.09466875	0.06186217	0	0	0.1090875
0.05256916	0.04008071	0.09466875	0.24744867	0.04564267	0.15102655	0.03397996
0.02628458	0	0	0.06186217	0	0.10068436	0.01385033
10.5664018	10.5011469	10.4608964	10.2072578	6.11611841	8.58334197	15.9380071
0.05256916	0	0	0	0	0	0.20545394
0.02628458	0	0.04733437	0	0	0	0.02035034
0.76225287	0.80161427	0.89935309	0.92793253	2.46470444	0.78030382	1.80707889
14.9296424	16.1525274	16.5196962	13.9808501	13.464589	12.6610587	15.8810536
0.42055331	0.40080713	0.09466875	0.5876906	0.41078407	0.62927727	0.57225436
0.2891304	0.44088785	0.23667187	0.40210409	0.5477121	0.65444836	0.18086881
0	0	0.09466875	0	0	0	0
0.10513833	0.08016143	0.04733437	0.12372434	0.1825707	0.25171091	0.01330859
44.4997967	49.0187123	46.5770231	42.8086206	42.1281888	36.4477395	18.2701615
0	0.04008071	0	0.03093108	0.27385605	0	0
0	0.08016143	0	0	0	0	0

0	0.04008071	0.04733437	0	0	0	0
4.23181765	4.04815204	4.44943107	5.19642215	4.79248085	5.94037744	7.58493804
0	0.08016143	0	0	0	0	0
0	0.08016143	0	0	0	0	0
0.02628458	0.04008071	0	0	0	0	0.04788316
0.02628458	0	0.04733437	0	0	0	0.03242919
35.1950548	36.1127226	28.2112863	29.5701165	28.9374558	26.328961	42.6695662
0.05256916	0.12024214	0.09466875	0	0.09128535	0	0.10499116
1692.6482	1911.93018	2220.69211	1653.29738	1621.36473	1461.9873	1651.84812
544.195978	539.005432	586.66222	531.396027	420.140821	457.786629	734.775858
46.2871483	49.5397616	42.7902732	41.2311353	39.6634843	40.0723766	8.5668185
0.02628458	0.04008071	0	0	0	0	0.06691684
15.6393261	15.9922046	15.1943338	17.8781667	17.0247177	20.1872148	33.505004
0	0.12024214	0.09466875	0.03093108	0	0	0
77.4869467	80.6824758	75.5456594	61.1507535	74.6714159	66.2503111	29.6817413
7.67509784	9.89993618	11.8335933	13.238504	10.4065298	12.6107165	7.96676637
98.6723195	104.290016	95.7574367	92.0818377	94.0695526	69.9756325	12.40666
2.94387314	3.36677991	2.79272801	2.13424481	2.60163246	2.1898849	1.9033809
2.83873482	2.16435852	1.9407093	3.21683276	2.41906176	3.90151908	1.94651486
1.34051366	1.08217926	0.80468434	1.05165686	2.00827769	1.81231854	0.53747316
1.78735155	1.12225997	1.18335933	1.7630718	1.46056559	2.7436489	1.11981605
57.0901113	60.6020384	58.741957	52.149808	55.9122765	42.8160255	21.6282108
23.2355702	19.7597916	24.3298678	27.4049406	22.1366972	30.0542825	14.8932307
80.9827961	82.60635	81.7464623	112.032387	76.5427655	100.860561	30.9771926
41.1353703	34.389252	41.6542483	35.3542293	32.4519417	32.9237868	31.4895449
98.7774578	98.0775053	97.1301335	95.7935679	83.8912362	76.6711427	70.5182432
0	0.08016143	0	0	0	0	0
0	0.04008071	0.04733437	0	0	0	0
9.90928728	11.0622769	12.6382776	11.1351903	14.0123011	15.9333005	9.09027763
0.05256916	0	0	0	0	0	0.25878102
0.07885374	0.12024214	0.33134061	0.09279325	0.09128535	0	0.07002586
3.36442645	1.96395495	2.22471553	3.3405571	2.64727513	4.48045417	5.85441442
4.17924848	5.17041201	4.07075608	4.73245589	5.47712097	7.04790543	2.90560564
162.832983	186.936447	190.28418	147.541272	159.338577	154.802209	177.690625
0	0	0.09466875	0	0	0	0
0	0.08016143	0	0	0	0	0
30.1221306	29.6196471	31.6193612	24.8995228	27.9333169	26.5051586	60.7550845
26.7839887	28.6176293	29.0633051	23.5694862	27.5225329	20.8668343	30.0591367
0.65711454	0.68137213	0.56801248	0.95886361	0.82156815	1.05718582	0.66958646
31.4363597	36.6337719	31.0513487	31.6115681	23.9624042	28.3174772	29.5018303
26.2320125	35.7119155	33.9860799	25.9511797	22.2279826	31.4638635	17.4682823
38.1652126	35.0706241	31.9507018	41.1692731	38.3398468	42.1867482	24.5526913
0.34169956	0.36072642	0.56801248	0.06186217	0.1825707	0.37756636	0.25534273
111.367772	101.965335	122.217351	91.8653202	106.48436	90.9683223	50.6448164
1.78735155	1.12225997	1.32536245	1.88679614	1.96263501	2.16471381	2.49309103

0.02628458	0.08016143	0.09466875	0	0.04564267	0	0.01844724
0	0	0.09466875	0	0	0	0
0.05256916	0	0	0	0	0	0.0751251
54.3565148	46.5337081	41.4649108	52.9230851	68.0988707	49.2598248	13.245722
34.4853711	38.3171619	41.9855889	37.9215093	33.3191526	24.2145894	12.4275878
6.78142207	7.29468981	8.99353088	6.21714793	3.4232006	7.40030071	10.7040177
0.02628458	0.04008071	0	0	0	0	0.03950067
12.4326071	12.865909	14.6736556	14.3829542	15.7010801	17.6449347	8.53697739
27.204542	32.5856199	36.163461	27.8379758	28.5266717	24.5669847	30.9715362
0.05256916	0	0	0	0	0	1.62802743
0	0.08016143	0	0	0	0	0
0.02628458	0	0.04733437	0	0	0	0.83515693
0.05256916	0	0	0	0	0	1.53112103
0	0.04008071	0.04733437	0	0	0	0
0.02628458	0	0.04733437	0	0	0	0.66986545
0.02628458	0	0.04733437	0	0	0	0.90573357
0.05256916	0	0	0	0	0	2.21748563
17.3478239	15.1104289	16.5196962	19.9196182	17.252931	20.6906367	573.549662
1.13023701	1.5230671	0.94668746	1.73214072	1.64313629	1.68646309	32.9191022
0.02628458	0.24048428	0.28400624	0	0	0.12585545	0.91867262
0	0.08016143	0	0	0	0	0
0.13142291	0.12024214	0.09466875	0	0.09128535	0.05034218	5.10373678
0	0.04008071	0.04733437	0	0	0	0
0	0.08016143	0	0	0	0	0
0.02628458	0.04008071	0	0	0	0	1.2133412
0.07885374	0.28056499	0	0	0.04564267	0.05034218	1.91011139
0.34169956	0.28056499	0.23667187	0.34024193	0.63899745	0.52859291	0.25994779
107.372516	100.642671	119.235286	89.9475929	93.8413392	98.1924253	171.583563
43.1592831	47.2150802	47.0503668	40.767169	40.9871219	38.2852292	46.9298804
33.6705491	37.4353862	41.6542483	33.8076751	35.5556436	25.9262236	38.1200249
12.9320142	13.1464739	13.7269682	10.9805349	10.3152445	11.9310971	26.0189844
0.2891304	0.16032285	0.23667187	0.46396626	0.50206942	0.32722418	0.33445764
5.04663968	4.5291206	4.9227748	5.41293974	5.79661969	6.97239216	4.39081081
5.02035509	4.20847489	4.59143419	4.97990456	6.07047574	6.49414144	7.40364854
0.05256916	0	0	0	0	0	0.10305622
0.07885374	0	0	0.12372434	0.13692802	0.07551327	0.0654414
0.2891304	0.12024214	0.09466875	0.34024193	0.22821337	0.45307964	0.15274842
133.630813	134.991842	155.54075	129.044483	120.679232	113.471277	72.2353539
12.8005913	11.5031647	10.5555652	14.7231961	11.1824553	16.5374067	32.4870846
0.02628458	0	0.04733437	0	0	0	0.02875988
4.04782557	4.48903989	4.02342171	5.1036289	5.47712097	5.18524471	3.29012985
1.47193657	2.04411638	1.27802807	2.22703806	1.59749362	2.97018872	2.00959636
0.78853745	1.24250211	0.99402183	1.88679614	1.55185094	1.05718582	0.40191927
0.05256916	0	0	0	0	0	0.05041716
0.02628458	0	0.04733437	0	0	0	0.02779044

60.9013757	69.2193918	39.3821984	55.3357097	44.2277518	34.2578546	53.8877078
5.12549342	9.9800976	6.20080287	6.21714793	5.84226237	3.07087308	6.65598793
1.05138327	1.92387424	1.27802807	0.71141494	1.04978152	1.05718582	1.33417185
3.60098769	5.17041201	5.49078728	2.25796915	5.29455027	2.29056927	6.81366622
20.3968354	23.3670558	19.407093	16.888372	21.7259132	12.6862298	11.0183919
0.02628458	0.04008071	0	0	0	0	0.03009222
0.05256916	0	0	0	0	0	0.09319867
1.83992072	1.64330924	1.65670306	2.1651759	2.28213374	2.44159581	5.67653952
1.47193657	1.56314782	1.79870618	1.33003662	1.04978152	1.00684363	1.38935057
1.31422908	1.64330924	1.51469994	1.36096771	2.96677386	2.26539818	3.27762912
0.05256916	0	0	0	0	0	0.12810176
23.997823	25.0103651	28.7792988	22.6415537	20.3566329	22.9560348	16.4876066
50.8869501	55.3113843	43.3582857	53.6654311	59.9744746	61.0147242	30.7479658
155.657293	162.006243	174.521833	142.93254	151.53368	125.226177	126.01805
72.0460383	67.7364054	67.2148097	77.8844701	73.5759917	87.3688563	24.3091595
33.9859641	28.8180328	27.7379426	31.7662235	21.6802705	21.6974803	16.3229405
90.8657988	91.9050755	75.9716688	103.155166	101.281095	95.5494608	47.0496481
32.5665967	28.0164186	29.6313175	36.5296105	34.1407207	33.8551172	18.287004
0.02628458	0.04008071	0	0	0	0	0.0872552
48.5476223	54.1891243	61.2033444	52.1188769	45.0949626	41.280589	56.4520832
0.05256916	0	0	0	0	0	0.04528668
26.9679808	23.5674594	27.7379426	28.1472866	34.3689341	28.9719256	13.1852653
40.9776628	44.4094303	44.3049732	43.6746909	33.821222	33.1251555	39.6891302
36.8246989	23.767863	25.8919021	30.1887382	36.1033557	39.342415	31.5455966
2.36561235	2.52508494	3.50274361	1.60841638	2.69291781	1.73680527	1.17802514
0	0	0.09466875	0	0	0	0
63.6875413	60.0409085	76.7763531	52.9540162	57.1902714	61.1657507	78.9341758
43.8426822	47.0948381	35.2641079	34.9830563	38.3398468	34.2326835	20.8969833
22.0001948	19.3189038	18.0343961	24.1262457	20.1284196	26.3793032	10.8627707
176.684958	180.483452	195.538295	166.873199	165.317768	139.120619	77.5654431
17.531816	15.2707518	11.9755964	18.5586505	15.5185094	20.5396101	7.97709217
0	0.08016143	0	0	0	0	0
25.1806292	25.1306072	20.4484492	22.394105	18.7134966	18.1483565	12.1775422
27.7828028	24.5694772	30.95668	28.9824259	29.0287411	44.049409	52.855822
52.2800329	45.291206	51.3577948	56.6038841	53.5388575	63.2549513	25.9288037
6.62371458	5.65138057	7.24215908	6.15528576	3.9709127	4.90836271	5.72224047
0.5782608	0.48096856	0.09466875	0.24744867	0.27385605	0.10068436	1.39384811
22.5258865	20.5614059	20.306446	25.0541782	22.1366972	26.8072117	56.4085675
15.9547411	15.3909939	15.1469994	13.1457108	12.8712343	13.1644805	39.6288321
3.96897183	5.33073486	5.11211229	4.57780047	6.38997446	7.80303816	4.64833393
38.7434733	36.6738526	39.8082077	32.1683276	36.4228544	31.1366394	11.2789911
97.5157979	92.8269319	102.147577	100.495093	106.98643	136.653852	36.0718596
9.67272605	9.81977475	9.135534	10.2381889	13.1450903	11.6793861	20.2265014
49.4675827	47.8964523	46.056345	54.0675352	52.762932	58.6989838	76.9395619
0.31541498	0.36072642	0.5206781	0.18558651	0.3651414	0.12585545	0.2051808

109.475283	104.610662	88.562612	122.858267	120.81616	108.311204	26.6612584
66.8942603	66.6542262	72.0902502	73.801567	79.6464674	83.3918239	33.190332
25.8640283	22.645603	15.9990181	28.1472866	28.0246023	22.8301794	29.8763787
0.02628458	0.04008071	0	0.03093108	0.04564267	0.17619764	0.0166988
0.02628458	0	0.04733437	0	0	0	0.03404292
0	0.04008071	0.04733437	0	0	0	0
39.9788487	34.950382	38.2461734	43.6437598	38.3854894	50.2666684	91.0717633
12.5903146	11.7035683	10.6502339	15.0325069	12.0496661	15.2788521	24.3695355
0.44683789	0.40080713	0	0.03093108	0.22821337	0.10068436	3.97534697
18.3992072	21.523343	24.8032115	19.2700655	17.8462858	16.9401441	30.1708836
315.41498	362.690374	356.948507	289.731466	321.507001	263.667176	96.3161508
7.09683704	6.73355983	5.01744354	6.55738986	4.10784073	2.99535981	4.90200805
67.3410982	72.3456874	66.7888004	62.5735834	62.9868911	53.5640813	36.5147933
0.52569163	0.40080713	0.42600936	0.86607036	0.50206942	0.72996163	0.33054271
63.1881343	64.5700291	55.2392134	71.2961491	64.8582408	76.494945	21.522237
11.8806309	14.3889761	16.3776931	15.0015758	27.2943195	13.9447843	9.22170104
0.05256916	0	0	0	0	0	1.81146714
0.02628458	0.04008071	0	0	0	0	0.54497528
0.13142291	0.08016143	0.14200312	0	0.09128535	0.05034218	0.10531786
24.3395226	18.8780159	22.1051522	25.0541782	24.281903	28.0154241	16.1552792
32.7243042	39.0786954	43.4529545	43.0251382	39.2527003	54.596096	43.1602797
202.995824	206.054947	265.593167	221.002597	153.496315	181.861131	203.209331
7.09683704	7.89590051	6.81614972	6.71204528	6.16176109	4.53079635	8.04583527
0	0	0.09466875	0	0	0	0
0.15770749	0.12024214	0.18933749	0.40210409	0.27385605	0.22653982	0.08109342
0.39426872	0.44088785	0.28400624	0.09279325	0.31949872	0.22653982	0.74429495
79.6422824	67.4959211	65.7001098	66.4709	89.0945011	98.5951628	27.3282556
0.39426872	0.48096856	0.33134061	0.18558651	0.1825707	0.30205309	0.62799886
115.862436	130.783367	100.680212	110.857006	98.9533188	83.3918239	45.0875813
0.05256916	0	0	0	0	0	0.03504473
29.6227235	24.7698808	23.4305147	30.0650139	29.4851679	31.4638635	18.7368363
0.02628458	0	0.04733437	0	0	0	0.03208936
9.96185644	9.13840262	8.6148559	7.14508046	8.39825215	7.4254718	10.784241
2.78616565	3.04613421	3.55007798	3.89731661	3.87962735	4.25391435	1.99197862
0.18399207	0	0.04733437	0.27837976	0.41078407	0.07551327	0.21517667
22.2104715	22.4051187	21.1111304	22.5487604	26.9291781	28.0405952	35.4003162
0.18399207	0.48096856	0.33134061	0.21651759	0.09128535	0.15102655	0.14601024
1.52450574	1.64330924	1.56203431	1.05165686	2.92113118	3.34775508	0.96502221
116.388128	118.719073	96.562121	134.673941	129.442626	121.677053	79.2738767
0	0.08016143	0	0	0	0	0
207.858472	212.227377	225.074944	233.962721	232.184286	294.124196	84.9124086
0.02628458	0	0.04733437	0	0	0	0.02874702
0.02628458	0	0.04733437	0	0	0	0.6495665
73.4916903	77.3958573	82.7404841	87.751486	92.2894883	85.3299979	75.5473131
73.4916903	77.3958573	82.7404841	87.751486	92.2894883	85.3299979	91.2240513

0.02628458	0	0.04733437	0	0	0	0.02386163
0.02628458	0.08016143	0.09466875	0	0.04564267	0	0.02599316
110.789512	122.767225	102.147577	111.908663	89.7791412	78.986883	39.1131827
0.02628458	0	0	0	0.09128535	0.10068436	0.04770555
0	0	0.09466875	0	0	0	0
6.17687669	6.85380197	6.95815284	7.73277106	8.94596425	7.85338034	7.34313148
0.02628458	0.04008071	0	0	0	0	0.07628361
0.02628458	0	0.04733437	0	0	0	0.02330811
44.0266743	46.1729817	43.5476232	48.1287671	49.6592301	57.7676535	13.397309
7.51739035	8.7776762	6.95815284	7.17601154	5.43147829	6.09140398	13.8492664
0.02628458	0.04008071	0	0	0	0	0.03899762
18.0049384	17.9160788	17.4190493	17.6616491	13.6015171	13.0386251	15.9718463
16.9798397	16.9942224	16.5196962	17.2286139	18.804782	24.6928401	8.33849375
1.2879445	1.2024214	2.4613874	2.10331373	3.10370188	1.96334509	0.81338335
0	0	0.09466875	0	0	0	0
64.1080946	72.9869788	48.091723	82.1529597	79.6008247	62.7011873	28.5432168
3.89011808	5.09025058	4.26009358	3.71173011	3.10370188	3.34775508	2.36575897
0	0	0.04733437	0.03093108	0.04564267	0.10068436	0
44.0266743	49.3794387	52.2098135	48.0359738	41.8543327	32.8482735	80.5642219
80.4308198	68.497939	66.1734535	82.276684	84.5302336	79.5909892	24.7864561
27.2571112	28.8981943	28.0692832	22.8890023	27.5681755	22.0750467	19.1023906
15.7444644	12.1444561	13.4429619	13.5168838	18.667854	18.576265	11.5502078
93.7308182	92.9872548	110.62043	88.3082455	90.9202081	77.5521309	75.458723
60.0339845	58.8785678	65.084763	56.3564355	54.9537804	48.6808897	37.4878454
5.20434717	9.41896762	9.89288397	11.9703296	8.53518017	11.4276752	3.97651546
774.133499	812.315816	759.243344	933.43826	902.857749	939.762676	404.608464
0.15770749	0.08016143	0.5206781	0.34024193	0.3651414	0.70479054	0.15671913
0.07885374	0.08016143	0.04733437	0.24744867	0.13692802	0.12585545	0.0381871
1.39308283	0.48096856	0.94668746	0.55675952	0.68464012	0.57893509	3.52094568
0.15770749	0.16032285	0.18933749	0.03093108	0.09128535	0.10068436	0.44045948
0.21027665	0.36072642	0.28400624	0.06186217	0.22821337	0.15102655	0.2316329
0.02628458	0.04008071	0	0	0	0	0.0940162
13.2211446	12.2646983	10.5555652	15.0943691	14.2861572	14.120982	6.03704049
0.02628458	0.04008071	0	0	0	0	0.01207418
0	0	0.09466875	0	0	0	0
0.13142291	0.20040357	0.14200312	0.03093108	0.04564267	0.10068436	0.10047982
15.5604723	15.791801	21.2057991	20.4454467	19.5350648	23.0315481	6.26663265
131.212632	130.422641	134.855629	124.559476	111.09427	105.768924	200.6381
49.9932743	52.9867029	42.0802577	43.8602774	41.397906	39.7451524	24.7194973
0.05256916	0	0	0	0	0	0.07637421
86.0557203	93.4281426	89.3672963	88.4010387	76.4058375	67.60955	23.318351
7.93794366	8.53719192	7.99950905	7.33066696	6.66383051	6.09140398	9.59522687
27.5988107	27.4953693	24.7558771	27.8689069	35.3274302	30.2556512	10.5652382
31.72549	28.4973871	30.2466644	26.1367662	24.9665431	27.4113179	42.8595525
4.81007844	4.64936274	3.97608734	4.36128288	2.28213374	3.39809726	2.74701126

0.63082996	0.9218564	0.5206781	0.37117301	0.50206942	0.42790854	0.35447175
9.93557186	8.7776762	9.23020275	10.8568106	11.5475967	11.2263065	12.9229546
10.6715401	10.8217926	12.0229308	11.9703296	13.053805	15.5809052	6.44022591
2.75988107	1.68338996	2.4613874	2.84565975	2.87548851	3.72532144	1.61769136
21.0802345	21.2026973	21.4898054	19.1463411	16.0205788	19.0041736	15.276742
9.35731107	10.621389	11.3129152	8.41325491	15.153368	17.745619	4.65690026
11.4075084	11.8238104	14.2949807	9.77422262	10.9085993	10.3453183	17.8106408
0	0.08016143	0	0	0	0	0
25.5748979	22.2848766	22.7204991	27.899838	27.4768902	26.2786188	21.9777984
36.2201535	35.4714312	36.2107954	34.3025724	42.7671862	51.9027893	17.130323
52.7005862	53.8684786	47.6183793	48.9329752	70.1071484	64.6141902	27.8058448
0	0.08016143	0	0	0	0	0
13.1160062	14.7497025	15.0523306	12.4033648	11.501954	11.8304127	8.18394149
12.8005913	14.1084111	18.3184024	14.8159893	16.6595763	26.9078961	12.0591258
16.086164	17.2347067	16.6616993	15.9295084	14.0123011	11.7045572	14.4797406
7.56995951	6.41291412	6.62681223	7.11414937	8.90032157	9.56501452	8.04536925
0.10513833	0.24048428	0.42600936	0.12372434	0.13692802	0.07551327	0.1315746
5.94031545	5.57121914	6.10613413	6.80483853	5.56840632	10.1942918	7.47218552
59.4294391	52.3053308	64.1854099	56.6348152	45.6883174	48.5298631	62.0299981
8.67391194	9.9800976	6.01146538	8.56791033	5.24890759	4.85802053	3.25430724
3.23300354	3.36677991	3.97608734	4.73245589	4.1534834	4.43011199	1.54759758
62.2156048	61.2834106	61.4873506	67.924661	70.6092178	72.0144909	32.1467511
5.4409084	5.25057344	6.34280599	7.0522872	6.34433179	7.45064289	7.47420902
3.52213394	4.68944345	3.50274361	3.89731661	2.41906176	2.31574036	1.52623967
67.5250903	58.5980028	61.2506787	69.780526	65.1777395	82.1836116	55.2709592
202.969539	144.250487	221.856207	142.65416	149.023333	176.776571	100.03612
0.91996036	1.12225997	0.99402183	0.92793253	0.41078407	0.62927727	0.72162485
0.18399207	0.16032285	0.14200312	0.18558651	0.31949872	0.42790854	0.14423248
7.56995951	6.0521877	9.51420899	5.41293974	4.3360541	7.75269598	2.52287699
1548.47727	1538.21761	1755.06388	2081.87849	1924.47774	1828.32735	2259.06911
21.737349	22.7658451	19.7857679	18.7751681	20.1740622	15.6312474	7.45576307
6.99169872	5.01008916	5.58545602	6.77390745	7.39411331	7.82820925	13.4796566
7.83280533	9.29872548	9.70354648	6.34087227	7.9418254	7.57649834	3.11449876
34.1962407	28.7779521	27.6906082	35.0449184	35.9664277	34.2326835	17.695329
47.496239	43.4474932	47.6657137	51.407462	48.7920193	59.8820251	61.2561411
2.73359649	6.17242984	4.07075608	3.12403951	3.9709127	1.88783181	3.85249808
0.39426872	0.36072642	0.28400624	0.18558651	0.1825707	0.22653982	0.29744257
30.2798381	27.6556921	29.2053082	40.3032027	27.8420316	34.5095655	12.5711454
160.467371	208.940758	184.888061	161.955157	177.869503	116.743519	93.3194067
33.5916953	33.3872341	36.0214579	32.8488114	29.7133812	26.3541321	10.7698142
0.13142291	0.16032285	0.18933749	0.37117301	0.45642675	0.15102655	0.33845833
0.10513833	0.16032285	0.14200312	0.09279325	0	0.05034218	0.12003189
0.84110661	1.12225997	0.85201872	0.61862168	0.91285349	0.35239527	0.22895268
0.02628458	0.04008071	0.04733437	0.09279325	0.04564267	0.15102655	0.02382626
3.39071103	2.56516565	2.93473113	4.88711131	3.51448595	3.37292617	6.05519252

86.449989	86.3338564	92.2546931	87.8752103	72.2067114	70.8566207	95.1872175
0.2891304	0.36072642	0.33134061	0.15465542	0.13692802	0.22653982	0.40237652
52.1748946	54.3093665	54.3398603	58.7071979	64.4474567	60.3351047	21.9555488
85.9768666	85.8929685	87.8525964	84.2253423	76.6340509	65.6462049	41.7938545
0.44683789	0.32064571	0.5206781	0.27837976	0.1825707	0.276882	1.24229593
38.1914971	34.7499784	31.9507018	38.1689579	38.2029187	45.2072791	50.6714708
38.1652126	35.6718348	31.8560331	34.7665387	28.2528157	27.4868312	54.4771791
16.7432785	18.9581774	16.1410212	21.5898968	18.1657845	21.1437163	5.88304066
16.0335948	16.8338996	18.4130711	14.1355055	14.6969413	14.8006014	50.1628144
5.80889254	5.37081558	6.34280599	6.71204528	5.97919039	9.26296143	16.7198417
0	0.04008071	0	0	0.13692802	0.07551327	0
0.18399207	0.24048428	0.18933749	0.06186217	0.1825707	0.07551327	0.32666878
6.99169872	6.69347911	9.32487149	6.24807901	6.89204388	5.26075798	8.876847
0.13142291	0.04008071	0.04733437	0.12372434	0.27385605	0.17619764	0.28657346
14.2725278	12.304779	14.1056432	20.7856886	12.3691649	16.9401441	30.4435451
8.70019653	7.05420553	8.33084966	9.37211852	9.17417762	10.7480558	8.70578511
67.6565132	70.3015711	73.3682782	65.9450716	61.2068268	57.6669691	45.6498711
11.6440697	10.5412276	12.0702651	13.8571257	13.5102317	13.1644805	235.43833
115.652159	88.0974077	92.9647087	122.518025	104.476082	120.418499	179.423695
9.25217274	7.97606194	8.99353088	7.98021973	7.30282796	6.26760162	11.9012058
95.0976164	102.005415	94.3374055	91.9271823	89.2770718	72.7696236	52.9260754
11.7229234	14.3488953	8.75685902	8.35139274	9.63060437	10.0684363	6.96647053
2.89130398	2.56516565	2.27204991	4.20662745	2.41906176	3.70015035	2.91461853
92.1011741	86.4140178	62.1973662	88.5866252	99.3184602	95.4487764	34.3493933
30.4638301	37.2750633	40.849564	30.5908423	33.821222	28.0405952	40.3748156
1.20909076	0.48096856	0.56801248	0.86607036	1.46056559	1.33406781	1.47832376
3.4432802	2.48500422	4.02342171	2.81472866	2.05392036	2.51710908	1.29027844
84.0055229	91.9451562	101.53223	80.2043014	82.1111718	81.1767679	111.759347
24.8652142	25.8921408	23.3358459	27.4358717	26.5640367	31.4890346	29.0518151
14.6930811	11.0622769	14.8629931	14.1973677	18.0288565	16.9904863	23.5567887
16.7695631	12.8258282	17.9397274	22.4250361	21.2694864	14.951628	9.7499808
42.7650143	49.3794387	48.1863918	40.4578582	43.5431117	38.6376244	20.6937549
25.3120521	27.6957729	26.6965864	27.8070447	30.6262347	33.4272086	78.7884495
0.60454538	1.16234068	0.89935309	0.86607036	0.31949872	0.40273745	0.50844377
53.1474241	48.9385509	39.9975452	57.284368	54.7712097	53.3627126	31.8308256
7.99051282	7.77565837	9.41954024	6.15528576	7.16589993	7.34995852	2.61669835
114.101369	108.097684	137.837694	104.299616	107.488499	98.6203339	61.326241
44.2895201	42.8462825	33.8440767	38.911304	36.9705665	21.8233358	64.5368883
27.4673878	25.2909301	22.7678334	21.651759	23.7341909	19.5327665	7.14371235
94.4405019	101.003397	106.218333	89.1124537	84.8040896	90.1376763	141.231877
56.9324038	56.6741285	54.813204	49.953701	49.111518	48.8822584	66.9659339
0.21027665	0.12024214	0.14200312	0.06186217	0.09128535	0.07551327	0.32851639
0.36798414	0.52104927	0.37867498	0.09279325	0.50206942	0.12585545	0.40941299
1.41936741	1.12225997	0.94668746	1.85586505	1.27799489	1.78714745	2.64880435
46.2082945	46.2932238	41.7015827	56.0161935	48.4725206	48.8822584	37.4716117

30.2535535	27.2148043	31.8086987	31.9208789	35.4187156	35.4912381	19.442462
2.575889	3.20645706	2.41405303	1.94865831	5.52276364	4.43011199	3.5625179
0.47312247	0.24048428	0.33134061	0.15465542	0.31949872	0.15102655	0.59238869
0.26284582	0.04008071	0.09466875	0.06186217	0.04564267	0.05034218	0.29938121
2.65474275	2.4048428	3.36074049	2.28890023	1.82570699	2.16471381	7.24890114
47.1019703	44.8903989	45.9616762	43.3653801	41.671762	35.4912381	13.132569
0.21027665	0.08016143	0.28400624	0.09279325	0	0.12585545	9.52697531
20.4231199	18.9180967	18.6970774	19.455652	25.3316845	23.7111676	16.180895
29.9118539	35.8321577	36.4474673	31.6424992	31.7216589	21.8736779	20.9209436
9.22588816	13.4671197	14.4843182	11.1661214	10.9998846	6.69551016	8.46971342
2.89130398	2.08419709	2.60339052	3.03124625	2.96677386	4.00220344	3.10798733
16.5330019	17.9962403	20.2591117	19.1463411	23.4603348	21.8736779	14.9867193
17.0061243	15.5913975	14.4843182	15.6511286	18.7134966	22.2009021	25.4319578
14.6667966	15.8318817	14.6263213	15.1562313	18.0288565	20.4892679	8.29672891
0.49940705	0.72145284	0.61534685	0.27837976	0.27385605	0.52859291	0.29961613
202.575271	149.420899	229.051031	158.429013	178.097717	144.708601	185.553984
19.9237129	16.7537381	15.857015	19.826825	19.1242807	23.3084301	33.0921719
66.3159995	72.7464946	74.2676313	66.842073	65.1777395	52.1545002	34.2076263
0.07885374	0.12024214	0.09466875	0.06186217	0.04564267	0	0.13789939
17.3741085	17.0743838	21.8684804	15.8367151	13.9666585	17.2170261	11.438908
196.372109	200.60397	210.779963	177.235113	199.002062	142.493545	45.3200849
11785.8487	13197.4967	13423.1288	15017.1033	17000.8922	15774.924	23031.0785
13.3525675	10.340824	12.5909432	14.5685407	13.2363757	15.6564185	30.7608271
45.5774646	53.5077522	44.8729857	45.2521762	46.0078161	22.5281263	13.8847569
6.54486083	6.81372125	6.10613413	5.84597492	5.15762224	4.83284944	4.90277519
12.2223305	10.7416312	13.8689713	9.27932527	11.501954	9.81672543	8.5878213
1.73478239	2.92589207	1.89337492	1.20631228	2.09956304	1.35923891	0.573085
0.07885374	0.20040357	0.14200312	0.21651759	0.31949872	0.25171091	0.07126755
7.28082912	6.61331769	9.89288397	9.61956719	8.3069668	11.6542151	11.3603712
26.9416962	20.7217288	20.4957835	21.8992076	28.1158876	32.0176275	20.8459078
5.23063175	6.37283341	6.43747474	5.01083564	5.15762224	4.27908544	3.32651666
57.7472259	49.4596002	58.2686132	47.1080413	51.6218651	45.156937	49.8703361
4.75750928	7.97606194	38.2461734	9.2174631	54.725567	37.1525301	2.40040876
60.9013757	70.5420553	66.2681223	63.2849983	57.0077007	51.0973144	19.1442262
40.6622478	33.6677991	30.0099925	41.849757	37.1987799	42.9167099	24.1288038
51.1235113	63.2072848	46.1510137	51.5930485	49.4310167	25.3221174	29.7448935
18.5831992	24.9702844	21.4898054	22.394105	26.381466	28.0154241	10.1484616
19.0826063	18.316886	21.1584648	16.6718544	17.0247177	16.4618934	18.0957142
27.2833958	28.09658	30.8146769	26.6007324	24.8296151	23.4594567	28.0937511
449.413777	434.074125	468.941634	533.220961	454.509755	615.458342	614.601739
0.21027665	0.08016143	0.09466875	0.37117301	0.45642675	0.10068436	0.25034388
20.7385349	19.7197109	22.2944897	18.5277195	18.804782	18.6769494	63.8217469
0.02628458	0	0	0	0	0	0.14681983
11.1446626	12.5051825	12.4962745	11.2898457	11.4106687	9.0615927	8.26248586
0.07885374	0.04008071	0.04733437	0.06186217	0.13692802	0.15102655	0.14955136

4.94150135	4.04815204	4.02342171	5.75318167	4.42733945	5.23558689	15.8657896
0.02628458	0	0	0	0	0	0.07655605
0.10513833	0.04008071	0.14200312	0.15465542	0.09128535	0.22653982	0.10355408
0	0.04008071	0	0	0	0	0
0	0	0	0.03093108	0	0.02517109	0
0	0	0	0	0	0.02517109	0
0.05256916	0	0.14200312	0.03093108	0	0.02517109	0.13300327
0	0	0	0.03093108	0	0	0
0	0	0	0	0.04564267	0	0
0	0	0	0.03093108	0	0	0
0	0	0.04733437	0	0	0	0
0	0	0	0	0.04564267	0	0
0	0	0	0.03093108	0	0	0
0	0	0.04733437	0	0	0	0
1.8662053	1.68338996	2.50872177	1.33003662	1.64313629	1.78714745	1.39286239
0.10513833	0.04008071	0.04733437	0.09279325	0.22821337	0.10068436	0.15356915
0	0	0	0.03093108	0	0	0
0	0.04008071	0	0	0	0	0
0	0	0	0.03093108	0	0	0
0	0	0	0	0	0.02517109	0
0	0	0	0.03093108	0	0	0
0	0	0.04733437	0	0	0	0
0	0	0.04733437	0	0	0	0
0	0	0.04733437	0	0	0	0
0	0	0	0	0.04564267	0.02517109	0
0	0	0	0	0.04564267	0	0
4.8626476	4.60928202	4.07075608	4.17569637	3.51448595	3.67497926	9.58647093
0	0	0	0.03093108	0	0	0
0	0.04008071	0	0	0	0	0
0	0	0	0	0	0.02517109	0
0	0.04008071	0	0	0	0	0
0.10513833	0.04008071	0.09466875	0	0	0.07551327	0.12867851
0.02628458	0	0	0	0	0	0.03277629
0.02628458	0	0	0	0	0	0.16659866
1.55079032	1.2024214	1.60936868	1.60841638	2.23649106	1.78714745	3.02802707
0.07885374	0.16032285	0.14200312	0.03093108	0.13692802	0.05034218	0.19350176
0.23656123	0.32064571	0.04733437	0.24744867	0.41078407	0.32722418	1.02799956
0.78853745	0.6012107	0.56801248	0.52582843	0.50206942	0.47825073	1.75702414
0.07885374	0.04008071	0.04733437	0.03093108	0	0.02517109	0.34760586
0	0	0	0	0	0.02517109	0
0	0	0	0	0.04564267	0	0
0	0.12024214	0.04733437	0	0.04564267	0	0
1.83992072	2.16435852	1.70403743	1.20631228	1.78006431	1.35923891	4.09599257
8.98932692	9.25864476	9.75088085	8.66070358	8.62646552	7.02273434	12.204785
0	0	0	0.03093108	0	0	0

6.59742999	8.69751478	5.68012477	5.93876817	4.5186248	6.14174616	8.57655575
0	0	0	0	0	0.02517109	0
0.07885374	0.08016143	0	0.06186217	0.22821337	0.10068436	0.35204608
0	0	0	0	0	0.02517109	0
1.44565199	0.84169498	1.13602495	0.74234602	0.77592547	1.03201472	3.8995475
11.118378	10.6614697	9.89288397	12.3105715	10.7260286	13.1393094	31.7779162
0.15770749	0.04008071	0.14200312	0.30931084	0.04564267	0.276882	0.25654422
1.47193657	0.96193712	1.08869058	0.83513927	2.37341909	1.88783181	4.68296056
0.02628458	0.08016143	0	0.09279325	0.09128535	0.10068436	0.02096742
2.02391279	1.84371281	1.65670306	2.50541782	2.19084839	2.31574036	8.6718834
27.9405103	27.9362571	29.2999769	24.4355565	39.572199	32.7727603	14.109067
0	0	0	0	0	0.02517109	0
0.76225287	0.44088785	0.5206781	0.37117301	0.31949872	0.42790854	2.36663124
0	0	0	0.03093108	0	0.02517109	0
39.4268725	39.7600676	40.4235546	39.7773743	48.3355925	48.3788366	108.139714
0	0	0.04733437	0	0	0	0
93.678249	102.085577	93.3907181	86.3286561	86.721082	89.8607943	184.831004
2.73359649	3.52710277	2.60339052	2.69100433	1.04978152	2.51710908	7.75862723
9.98814103	10.2205819	10.0348871	9.65049828	11.9583808	12.9631118	12.3293096
1.70849781	2.28460066	2.08271241	1.5774853	1.73442164	1.61094981	9.65348826
0.13142291	0.12024214	0.04733437	0.15465542	0.1825707	0.20136873	0.17882949
3.54841852	4.00807133	3.31340611	4.29942071	4.1534834	4.75733617	18.8317923
2.75988107	3.52710277	2.31938428	1.7630718	2.60163246	2.39125363	1.68427133
1.07766785	1.00201783	1.84604055	1.54655421	1.64313629	1.86266072	1.12290904
18.8986142	23.0864908	27.2645989	18.0637532	30.3980214	32.9237868	18.0541948
0.44683789	0.84169498	0.56801248	0.83513927	0.68464012	1.00684363	1.156847
0.05256916	0	0.14200312	0	0.04564267	0.02517109	0.06029731
10.4612635	12.0642947	10.5082308	8.6297725	9.72188972	9.56501452	4.44112774
0	0	0	0	0	0.02517109	0
46.7602708	53.1069451	56.2805696	42.0662745	53.6757855	39.7199813	82.780247
16.3752944	21.1626166	20.5904523	17.5069937	16.2944349	15.6312474	13.9108725
0.76225287	0.52104927	0.42600936	0.46396626	0.50206942	2.01368727	0.78127583
0.02628458	0.08016143	0.09466875	0.03093108	0	0.02517109	0.10992664
0	0	0	0	0	0.02517109	0
0.86739119	1.04209854	1.18335933	1.08258795	1.55185094	1.40958109	0.75119779
69.3912955	61.5639756	51.9731416	85.7409655	62.302251	64.0352551	29.0802844
0	0	0	0	0	0.05034218	0
0	0	0	0.03093108	0	0.02517109	0
0.02628458	0	0	0	0	0	0.06855766
0.02628458	0	0	0	0	0	0.01228172
0	0	0.04733437	0	0	0	0
1.31422908	1.12225997	1.37269682	1.11351903	2.09956304	1.83748963	2.10153867
0.5782608	0.56112999	0.7100156	0.5876906	0.5477121	0.22653982	0.34984071
0.23656123	0.6012107	0.14200312	0.30931084	0.3651414	1.08235691	0.3923822
0.02628458	0	0	0	0	0	0.03208936

0	0	0	0.03093108	0	0	0
0	0	0	0	0	0.05034218	0
0	0	0	0	0.04564267	0	0
0.02628458	0	0	0	0	0	0.04236303
0	0.04008071	0	0	0	0	0
0.2891304	0.16032285	0.14200312	0.09279325	0.09128535	0.17619764	0.17513689
0.02628458	0	0	0	0	0	0.05867435
0	0.04008071	0	0.03093108	0.22821337	0	0
0	0	0	0	0	0.02517109	0
0	0	0.04733437	0	0	0	0
0	0	0	0	0.04564267	0	0
0.02628458	0	0	0	0.04564267	0.07551327	0.03967124
0.21027665	0.16032285	0.42600936	0.06186217	0.09128535	0.20136873	0.26382393
0	0	0.04733437	0	0	0	0
0	0	0	0	0.04564267	0	0
0	0	0.04733437	0.03093108	0.04564267	0.05034218	0
0	0	0	0	0	0.02517109	0
3.49584936	4.60928202	5.4434529	3.3405571	3.51448595	3.72532144	3.10899385
0.02628458	0	0	0	0	0	0.03740959
0	0.04008071	0	0	0	0	0
0	0	0	0	0.04564267	0.02517109	0
0	0	0	0	0	0.02517109	0
0	0	0	0	0	0.02517109	0
0	0.08016143	0.04733437	0.09279325	0.04564267	0.15102655	0
0	0	0	0	0	0.02517109	0
0	0	0	0.03093108	0	0	0
0	0	0	0	0	0.02517109	0
0	0.08016143	0	0.06186217	0.1825707	0.02517109	0
0	0	0	0	0	0.02517109	0
0	0	0	0	0.09128535	0	0
0.02628458	0	0	0.09279325	0.09128535	0	0.04824237
0.07885374	0	0.04733437	0	0	0.02517109	0.16198258
0	0	0	0	0.04564267	0	0
0.02628458	0	0	0	0	0	0.05350007
0	0.04008071	0	0	0	0	0
0.18399207	0.24048428	0.14200312	0.12372434	0.5477121	0.37756636	0.99590616
30.3849764	29.5795664	37.2048172	32.2301898	32.3150137	47.2461375	13.7920201
0	0	0.04733437	0	0	0	0
0	0	0	0	0	0.02517109	0
0	0	0	0	0.04564267	0.02517109	0
0	0.04008071	0	0	0	0	0
0	0.04008071	0	0	0	0	0
0	0	0	0	0	0.05034218	0
0	0	0	0	0	0.02517109	0
0	0	0	0.03093108	0	0.02517109	0

0	0	0	0	0	0.02517109	0
0.21027665	0.08016143	0.28400624	0.06186217	0.09128535	0.12585545	0.44657697
0.31541498	0.08016143	0.14200312	0.15465542	0.41078407	0.37756636	0.22730044
0	0	0	0	0	0.02517109	0
0.10513833	0.08016143	0.14200312	0	0.13692802	0.02517109	0.55919203
0	0	0.04733437	0	0	0	0
10.6715401	15.0703482	14.0109744	13.3931595	16.842147	17.468737	8.75836157
0	0.04008071	0	0	0	0	0
0	0	0	0.03093108	0	0	0
0	0	0	0	0.04564267	0.02517109	0
0.05256916	0.04008071	0.04733437	0.15465542	0.13692802	0.05034218	0.27191156
0	0	0.04733437	0	0	0	0
0	0	0	0.03093108	0	0	0
2.54960442	2.56516565	3.12406862	3.09310842	3.69705665	3.34775508	2.01804823
0	0	0	0.03093108	0	0	0
0	0	0	0	0	0.02517109	0
0.13142291	0.04008071	0.23667187	0.12372434	0.31949872	0.30205309	0.17166867
0	0	0	0	0.04564267	0	0
0	0	0	0	0	0.02517109	0
0	0	0	0	0	0.02517109	0
0	0	0	0.03093108	0	0	0
0.05256916	0.08016143	0.04733437	0	0.04564267	0.02517109	0.06657048
0	0	0	0.03093108	0	0	0
6.28201501	7.01412482	6.20080287	5.2892154	5.38583562	6.11657507	2.84249915
0	0	0	0.03093108	0	0	0
0.02628458	0	0	0.03093108	0.04564267	0.07551327	0.03594583
64.8703475	58.2773571	64.6587536	52.5519121	56.0492046	57.1635473	56.0416249
0	0	0.04733437	0	0	0	0
2.41818151	2.28460066	2.27204991	2.13424481	3.65141398	3.59946599	3.15197212
0.02628458	0.08016143	0.04733437	0.03093108	0.1825707	0.12585545	0.05621249
0	0	0	0	0.04564267	0	0
2.54960442	2.1242778	3.6920811	1.7630718	2.78420316	2.01368727	2.352994
14.6667966	13.8278461	10.9342402	13.1457108	16.979075	16.9904863	14.0940112
44.420943	42.766121	43.263617	41.0764799	41.3522633	31.5645479	21.5975697
0	0	0	0	0	0.02517109	0
0.07885374	0.12024214	0.04733437	0.15465542	0.09128535	0.17619764	0.05800398
0.78853745	0.84169498	1.13602495	0.43303518	0.7302828	0.78030382	3.08180911
0.05256916	0.04008071	0.04733437	0	0	0.02517109	0.05604103
0	0	0	0	0.04564267	0	0
0.02628458	0	0	0	0	0	0.05515187
0	0	0	0.03093108	0.04564267	0	0
19.3191675	17.9962403	18.6970774	21.311517	20.2653476	21.1688874	16.5554138
0.07885374	0	0.04733437	0.12372434	0.04564267	0.12585545	0.11249052
31.4363597	32.1046513	32.1400393	29.0752192	28.070245	27.4113179	7.8217504
0	0	0	0.03093108	0	0	0

0	0	0	0	0.04564267	0	0
0	0.04008071	0	0	0	0	0
0.02628458	0.24048428	0.09466875	0.34024193	0.09128535	0.22653982	0.04024223
0.02628458	0	0	0	0	0	0.05751975
15.5341878	17.7156753	16.9930399	15.0015758	15.1990107	13.3658492	10.4698309
0.26284582	0.28056499	0.47334373	0.12372434	0.1825707	0.25171091	0.22739421
0.02628458	0	0	0.06186217	0	0.05034218	0.04890272
0	0	0	0	0	0.02517109	0
31.1209447	34.5495748	36.5894704	34.4881589	33.0909392	21.6723092	22.276064
12.064623	9.29872548	10.2242246	9.99074021	9.31110564	8.45748652	7.01281807
0	0	0	0.03093108	0	0.02517109	0
0	0	0.04733437	0	0	0	0
3.4432802	2.76556921	2.74539364	3.46428143	4.01655538	3.49878163	4.5121735
0.34169956	0.6012107	0.42600936	0.71141494	0.77592547	0.57893509	0.36860321
0	0.04008071	0	0	0.04564267	0.07551327	0
0	0	0	0	0.04564267	0.02517109	0
0	0	0	0	0	0.02517109	0
0.02628458	0	0	0	0	0	0.0338815
0	0	0	0	0	0.02517109	0
0	0	0	0	0	0.02517109	0
0	0	0	0	0	0.02517109	0
0.10513833	0.12024214	0.18933749	0	0.09128535	0.10068436	0.18282042
0	0.04008071	0	0	0	0	0
0.65711454	0.56112999	0.80468434	0.74234602	0.86721082	1.03201472	1.07608908
5.30948549	5.81170342	4.02342171	3.64986794	3.788342	4.78250726	4.88347024
2.31304318	1.92387424	1.98804367	2.31983132	3.10370188	2.64296454	1.78124751
4.07411016	3.96799061	4.54409981	4.26848962	4.92940887	5.63832435	8.86008704
0	0	0	0.03093108	0	0.02517109	0
0	0	0	0	0	0.02517109	0
0	0	0	0	0	0.02517109	0
0.10513833	0.04008071	0.09466875	0	0.09128535	0.02517109	0.26963138
0.47312247	0.44088785	0.47334373	0.68048385	0.77592547	0.60410618	0.58284366
2.49703526	2.32468137	2.13004679	2.50541782	3.37755793	3.19672854	1.19087191
0	0.04008071	0	0.06186217	0	0.05034218	0
0.36798414	1.5230671	1.27802807	1.20631228	2.51034711	1.05718582	0.23518787
41.9239077	43.6879775	41.6069139	41.0146177	37.1074946	35.4157248	17.5093544
0.02628458	0	0	0	0	0	0.09787988
0.07885374	0.04008071	0	0	0.04564267	0	0.10422542
5.65118505	4.80968559	4.82810605	4.33035179	4.7011955	4.17840108	6.28170056
0.26284582	0.16032285	0.23667187	0.15465542	0.13692802	0.10068436	0.30162797
0.60454538	0.84169498	0.47334373	0.43303518	0.50206942	0.45307964	0.54557835
0	0	0.04733437	0	0	0	0
0.5782608	0.88177569	0.85201872	0.64955277	0.59335477	0.20136873	0.99700904
0.02628458	0	0	0	0	0	0.01823797
0.86739119	0.72145284	1.32536245	0.83513927	1.27799489	1.661292	1.69364226

0	0	0	0	0	0.02517109	0
0	0	0	0.03093108	0.04564267	0	0
3.02272689	3.04613421	3.59741235	3.80452336	4.06219805	3.75049253	2.68043298
0	0	0.04733437	0	0	0	0
0.15770749	0.12024214	0.23667187	0.09279325	0.09128535	0.07551327	0.32342205
0.02628458	0	0	0	0	0	0.06712639
0	0	0	0.03093108	0	0	0
0	0	0	0.03093108	0	0	0
34.7745015	31.3831985	38.1988391	37.457543	39.6178417	41.3057601	95.2724203
80.1942586	80.5221529	90.8346619	80.049646	77.8207604	71.4355558	52.8131659
29.7278618	38.5576462	44.5889794	34.7665387	31.9042296	28.1664506	15.6917608
0	0	0	0	0	0.05034218	0
100.801371	99.8811374	88.4679433	92.4839418	93.0654138	71.6872667	30.5939294
8.83161943	5.01008916	3.31340611	8.38232383	10.9085993	4.70699399	2.98112307
62.0841819	61.083007	63.948738	60.1918899	55.1363511	54.042332	35.2748098
13.9833974	12.9861511	13.7269682	12.5889513	11.0455273	12.0317814	10.2613582
0.44683789	0.16032285	0.18933749	0.43303518	0.31949872	0.47825073	0.30149488
0	0.04008071	0	0	0	0	0
33.4602724	27.0544815	34.3647548	30.5908423	25.9250392	27.6126866	36.5786046
66.7628374	57.7162271	54.9078728	67.0585906	69.4681509	62.9780693	59.4179672
20.0025666	18.0764017	16.8037024	18.8679614	20.0371342	23.7363387	26.0306864
36.9824064	36.1127226	39.9028765	40.2413406	30.443664	23.8621941	19.8508263
553.448151	539.807046	518.595391	480.390669	488.833046	433.773408	159.581608
5.75632338	5.85178414	5.96413101	5.59852625	7.89618273	8.18060452	3.37565946
1.68221323	2.76556921	3.02939988	3.00031517	2.60163246	4.02737453	1.129123
0	0	0	0	0	0.02517109	0
0	0	0.09466875	0.06186217	0.04564267	0.07551327	0
51.5966338	53.0267836	49.3224167	55.1501232	39.709127	42.3377748	22.2714899
0.60454538	0.44088785	1.37269682	0.52582843	0.41078407	0.60410618	0.57040606
31.856913	26.8139972	27.3119333	24.7448674	26.4271087	25.8255392	44.3850712
0.60454538	1.04209854	0.66268122	0.49489735	1.46056559	1.38441	0.94388189
11.5126468	11.6634876	10.6975683	9.68142936	9.81317507	9.56501452	7.13076013
0.05256916	0.12024214	0.18933749	0.06186217	0.04564267	0.02517109	0.04498572
2.91758856	1.72347067	4.02342171	1.82493397	6.93768656	3.72532144	2.86555048
296.358658	293.631305	319.365015	340.767755	289.967913	385.520427	383.833968
0	0	0	0	0	0.02517109	0
0.5782608	0.6012107	1.46736556	1.63934746	0.82156815	1.23338345	1.07585995
46.2871483	42.4454753	38.9088547	55.1191921	45.5513894	44.7290284	30.2874495
47.811654	43.4474932	48.2337262	51.0672201	55.2732791	48.1019546	68.3662096
44.2106663	38.1969197	33.1813955	49.1185618	39.1614149	44.880055	28.554518
0	0	0.04733437	0	0	0	0
0	0	0.04733437	0	0	0	0
17.0324089	16.5132539	16.3776931	15.0325069	15.0620827	14.0706398	15.2808911
19.3454521	17.5553524	18.4604055	15.2180934	17.3442164	15.782274	9.27675683
3.62727227	5.49105772	4.44943107	4.5159383	6.29868911	5.86486416	1.84039351

3.12786522	3.16637635	3.45540923	4.54686938	4.79248085	3.2974129	0.97621418
111.394057	108.378249	99.2128459	98.8248141	95.4844755	93.2840626	76.5758413
12.4063225	12.304779	12.4489401	13.7334014	11.9127381	16.8394598	19.0062263
32.040905	30.3811807	27.2172645	31.9208789	32.4062991	36.8253059	12.9806813
26.8102733	25.5714951	31.1933518	24.7448674	24.6470444	23.6104832	25.4040376
0	0.12024214	0.04733437	0	0.04564267	0	0
0	0	0	0.03093108	0	0	0
22.3418944	28.4973871	20.4484492	30.5599112	24.3731883	28.8964123	9.39354199
0	0	0	0	0	0.02517109	0
23.0778627	22.2447959	23.5251834	25.332558	16.5682909	16.059156	22.6662462
0	0.04008071	0	0	0	0	0
0	0	0	0	0.09128535	0	0
11.118378	13.0262318	9.6562121	13.949919	13.6471597	11.6542151	11.664621
0	0	0	0	0	0.02517109	0
13.6154133	12.5452633	9.23020275	13.7643325	14.4687279	14.7250881	5.91355746
3.73241059	6.25259127	5.06477792	4.76338697	2.41906176	4.75733617	2.63766778
30.4638301	27.7759343	30.1519956	26.7863189	26.9748208	23.7111676	11.4295215
76.3041405	72.9869788	69.0135159	69.8114571	66.3188064	63.45632	82.8599481
0	0.04008071	0	0	0	0	0
63.6086876	70.9027818	62.7180443	61.5219265	49.4766594	63.1039247	68.1362267
0	0	0	0	0	0.02517109	0
0	0	0	0.03093108	0	0	0
0	0	0	0	0.04564267	0	0
0	0	0	0	0	0.05034218	0
3.02272689	1.60322853	3.73941547	2.10331373	2.41906176	1.6361209	2.91039535
3.49584936	3.00605349	2.69805926	3.18590168	6.48125981	2.76881999	1.80744761
0.02628458	0	0	0.03093108	0.04564267	0.05034218	0.02709949
0.73596829	0.48096856	1.70403743	0.9897947	1.91699234	1.35923891	0.30869164
46.8391245	45.8122553	53.2038353	45.3759006	45.1406053	40.9281937	33.1487482
25.8640283	24.8099615	20.7797898	28.5803218	25.3316845	25.4228017	40.9567444
0	0	0	0.06186217	0	0	0
1.05138327	1.16234068	1.08869058	1.08258795	1.46056559	1.73680527	2.93975238
130.05611	124.490695	134.287616	140.427122	160.434002	137.509669	134.371389
13.2737137	14.6294603	15.9043493	13.2694351	13.3733037	11.1759643	30.8112686
0	0	0	0	0	0.02517109	0
0	0	0.04733437	0	0	0	0
5.67746964	4.08823275	6.20080287	4.48500721	4.29041142	4.63148071	11.9538124
43.8163976	36.4333684	47.3817074	53.4489135	50.0243715	39.7703235	20.7671267
0	0	0	0	0	0.02517109	0
305.742254	357.680285	316.950962	297.526099	335.428017	206.327432	72.8851207
7.80652075	6.85380197	8.99353088	9.43398069	8.35260948	10.2194629	19.6291919
0.02628458	0	0.04733437	0.12372434	0.09128535	0	0.05223971
0.63082996	1.04209854	0.85201872	0.27837976	0.27385605	0.90615927	1.25273539
0	0	0.09466875	0.12372434	0	0.12585545	0
125.982	127.336426	103.425605	110.39304	108.401352	92.2017057	95.1014658

31.72549	24.6496387	25.6078958	29.755703	34.2776487	31.4890346	16.8699521
40.2416945	39.3191797	35.0274361	41.2002042	23.1864788	31.9924565	42.7689594
81.7713335	79.0792473	88.7046151	79.8021973	74.0780611	69.4722107	35.4275432
0	0.04008071	0	0.09279325	0	0.02517109	0
0	0	0	0	0	0.05034218	0
1.20909076	1.36274425	1.42003119	1.14445012	0.91285349	1.00684363	2.77758294
0	0.04008071	0	0	0	0	0
0.10513833	0.04008071	0	0.03093108	0	0	0.21985328
6.96541414	7.49509338	6.05879975	6.68111419	10.6803859	6.97239216	5.69755169
0.02628458	0	0.04733437	0.03093108	0.22821337	0.05034218	0.02663922
0.36798414	0.76153355	0.5206781	1.45376096	0.68464012	0.35239527	0.27506849
13.6679825	8.17646551	7.9048403	14.0117812	18.1201419	8.25611779	6.97386514
229.043844	283.891692	263.605124	256.820792	244.18831	151.806849	77.3140072
44.1843817	37.4353862	32.3767112	37.828716	46.0534588	45.8113853	20.6929952
0	0	0	0	0	0.02517109	0
0	0	0	0	0	0.02517109	0
0	0	0.04733437	0	0	0	0
0	0	0	0	0	0.02517109	0
0	0	0	0	0.04564267	0	0
0	0.04008071	0	0	0	0	0
40.9250936	43.1268475	45.298995	46.0254533	33.684294	32.2693384	17.924477
8.51620445	8.33678836	10.9342402	8.56791033	12.5517356	12.4345189	6.48273025
11.6703543	16.8739803	13.4902963	14.2901609	11.6845247	8.38197325	9.18377131
15.8758873	17.3950296	24.5665396	16.9502342	27.8876743	22.9308638	5.18924226
5.12549342	4.60928202	4.73343731	5.44387082	7.25718528	4.6818229	3.52243856
130.870932	137.557008	97.0827992	112.156111	98.9076761	113.169224	92.9689222
6.09802294	2.16435852	6.29547162	2.07238264	4.97505155	17.3177105	7.83985462
393.61161	413.192073	432.020823	397.186053	339.033788	366.189029	106.786269
199.079421	227.377886	228.625022	215.68245	194.163938	174.737713	64.5371471
17.7683772	17.5954331	14.5789869	14.5685407	16.5682909	12.2079791	13.0310517
14.2199587	11.8638911	11.4075839	11.753812	14.9251546	16.5122356	14.7791555
0.05256916	0.20040357	0.28400624	0.15465542	0.3651414	0.37756636	0.11692197
0	0	0	0.06186217	0	0	0
7.38596744	7.33477053	9.75088085	8.75349684	9.40239099	9.79155433	5.5498435
56.5907043	57.4757428	60.0673194	51.036289	52.0782919	51.7769338	36.999773
0	0	0	0.03093108	0	0	0
0.73596829	1.12225997	0.94668746	0.5876906	0.5477121	0.80547491	0.64723161
5.20434717	6.53315626	11.0762433	7.11414937	4.38169677	5.58798217	3.42003828
85.0569062	93.0273355	83.6871716	83.0499611	84.0738069	53.2368571	31.4584613
5.88774629	4.80968559	4.40209669	5.66038841	5.79661969	6.67033907	7.67028044
0.86739119	0.44088785	0.33134061	0.27837976	0.50206942	0.37756636	0.59327195
0	0	0.04733437	0	0	0	0
0.05256916	0.08016143	0.04733437	0.06186217	0.09128535	0.22653982	0.08597204
34.0385332	28.6176293	27.4539364	37.0245078	36.1946411	30.1549668	32.5557752
0	0	0	0	0.04564267	0	0

0.02628458	0	0	0	0	0	0.03655889
1.26165992	0.72145284	3.45540923	0.68048385	1.27799489	1.68646309	0.39482476
21.5270724	26.3731093	20.8744585	25.2397647	14.1948718	19.7593063	10.0052244
253.22566	280.124105	255.889621	257.779656	231.317076	201.922491	165.16514
88.526471	98.959281	103.236268	106.310136	105.845363	119.487168	125.702993
58.430625	52.1049272	51.263126	46.0254533	73.1652076	73.9526649	32.6231507
6.49229167	5.97202628	4.63876856	6.37180335	6.52690249	7.04790543	5.94233056
0	0	0	0	0.09128535	0	0
31.5940671	25.0905265	27.027927	33.8386061	26.8835354	33.5027219	96.8635516
40.6359632	43.0066053	44.5416451	37.2719565	39.8916977	36.5484239	22.4725929
8.38478155	8.61735335	8.75685902	9.09373876	10.3152445	11.4025041	5.91691941
12.4851763	13.1063932	11.4549183	12.2796404	8.8546789	9.69086997	4.60583001
3.4432802	3.36677991	3.50274361	4.5159383	3.33191526	4.75733617	3.00972773
0.44683789	0.6012107	0.5206781	0.83513927	0.82156815	0.65444836	1.17550582
0	0	0	0	0.04564267	0	0
0	0	0	0.03093108	0	0	0
61.1642215	71.3436696	68.5401722	58.3669559	61.2981122	58.4976151	21.4972824
118.806309	119.640929	96.940796	85.555379	105.708435	102.144287	98.6988173
1.47193657	1.56314782	1.32536245	1.60841638	0.86721082	0.88098818	1.20079916
22.6047402	24.2087508	29.8679894	27.6833204	29.6677386	30.1046246	7.51210156
3.02272689	2.68540779	1.7513718	2.47448674	1.78006431	1.661292	2.41755953
146.06342	158.318817	168.463034	146.737064	147.60841	122.683897	103.431103
31.9357667	30.9423106	26.5545833	38.0143025	29.9872373	34.2326835	22.4455922
42.975291	36.2730455	37.1101485	43.4581733	45.0949626	42.941881	112.692477
0	0.04008071	0	0	0	0.15102655	0
0	0	0	0	0	0.02517109	0
174.63476	186.695962	185.456074	174.88435	206.350532	239.704298	83.9403265
1.36679825	0.96193712	0.47334373	0.95886361	1.50620827	1.48509436	0.7519605
17.4529622	19.6796302	22.0104835	18.713306	17.2985737	16.1598403	7.90153652
44.2895201	38.7580497	37.4414891	38.9731661	39.8916977	27.0337516	26.5973087
98.514612	109.700912	100.632877	102.196302	98.9076761	72.1151752	46.3862487
0.73596829	0.56112999	0.7100156	0.80420819	0.86721082	1.20821236	1.16392911
31.856913	31.9042478	30.7673425	32.8488114	36.6967105	38.3355713	14.0991652
43.2381368	34.108687	35.9267892	41.0146177	26.4727513	27.6630288	31.1407572
65.1594779	67.6963247	60.9193381	75.1625347	66.5013771	71.7124378	32.8695381
18.0049384	17.9160788	17.4663837	19.3319276	19.4437794	20.9171765	18.454274
54.3302303	61.7643791	52.9671635	64.8315525	69.3312229	59.579972	29.149724
134.209074	147.657348	131.210882	127.961895	129.716482	110.551431	71.8651713
4.57351721	5.21049272	4.11809046	3.77359228	4.29041142	3.0205309	2.95157808
50.387543	55.63203	59.3573038	50.293943	52.5347186	41.7588397	146.409357
15.4027648	14.5492989	13.4429619	20.7238264	15.3815814	15.4802209	27.2676924
73.7808207	78.558198	77.3443656	80.6682677	62.9412485	59.4289455	34.6335347
27.9405103	29.3390821	29.1106394	31.95181	30.5805921	32.7475892	40.1635897
68.8130348	69.7404411	81.3204529	73.8634291	63.99103	60.4357891	57.4397626
0.02628458	0	0	0	0	0	0.05830198

0	0	0	0.03093108	0	0	0
35.2213394	28.5775486	32.9447237	38.5091999	31.9498723	42.2622615	55.5586665
28.7027632	25.1706879	30.2466644	31.5497059	29.3482399	34.9374741	30.921768
13.4314212	13.9881689	14.2949807	18.0637532	12.5973782	17.3680527	9.41303913
1.07766785	1.00201783	0.7100156	0.61862168	0.59335477	0.78030382	0.71452315
20.0288512	13.1865547	13.064287	17.259545	18.4852833	17.9973299	28.5892634
0	0	0	0	0.04564267	0	0
0.26284582	0.36072642	0.23667187	0.46396626	0.31949872	0.47825073	0.47215186
0	0.04008071	0	0	0	0	0
0.02628458	0	0	0	0	0	0.02426682
5.46719298	4.20847489	4.49676544	6.37180335	4.5186248	5.99071962	8.7481186
0	0	0	0	0.04564267	0	0
0.02628458	0	0	0.03093108	0	0.07551327	0.12414495
59.1665933	64.2093026	67.7354879	72.6261858	61.1155415	80.8747149	61.5717757
26.1005896	23.2868944	20.685121	26.074904	24.8752577	27.2351203	34.1298417
2.41818151	2.56516565	1.84604055	2.56727999	2.55598978	3.62463708	0.81897172
167.879623	174.110618	175.421187	164.831748	160.251431	139.019935	146.011142
48.9681756	51.8243622	45.9143419	41.8188259	49.0202327	39.6444681	49.5262903
3.54841852	3.48702205	3.40807486	3.5261436	4.10784073	5.31110017	1.19381962
8.27964322	7.49509338	7.10015596	8.78442792	9.63060437	7.92889361	8.15816805
252.805106	285.655243	278.988795	268.883915	243.68624	176.776571	82.8762599
11.8806309	13.6675232	10.3188933	10.8877416	11.501954	9.08676379	10.1845836
13.2211446	12.1845368	12.685612	15.063438	12.5060929	16.2856958	26.2979373
50.0984126	48.0567752	45.1096575	48.5618022	44.2733945	35.9443177	12.0496757
5.62490047	4.68944345	4.02342171	5.44387082	5.52276364	6.36828598	4.62578684
5.78260796	5.21049272	6.43747474	5.96969926	8.03311075	7.27444525	5.02042524
86.5551274	85.0512735	97.7928147	85.3079303	82.2937425	73.9274938	51.0518866
11.1972318	8.97807977	10.7449027	11.3517079	10.1326738	14.3726929	62.5452455
16.6644248	14.5492989	15.2416681	17.9400289	17.8919285	18.3245541	6.74118566
234.432184	271.546832	232.648444	247.324949	225.611741	168.797335	77.0596368
1170.61013	1206.91044	1185.44204	1212.40571	1018.88143	805.726618	773.630542
0.34169956	0.20040357	0.66268122	0.30931084	0.22821337	0.22653982	0.21245034
1.15652159	0.56112999	0.37867498	0.40210409	0.59335477	0.50342182	4.37327924
114.180223	124.811341	118.19393	110.98073	110.957342	96.2290803	69.3692501
0	0	0	0	0	0.02517109	0
31.0946601	29.21884	25.0398834	34.4881589	27.4768902	33.8299461	13.9408612
39.4268725	38.9584533	37.0628141	48.0669049	39.846055	39.2417306	12.8169845
99.6974182	126.695135	111.283111	109.279521	104.019656	75.5636147	54.5431054
56.6958426	60.0008278	56.0438977	50.8197714	55.1363511	46.6420313	20.2437797
0	0	0.04733437	0	0	0	0
0	0	0	0.03093108	0	0.02517109	0
61.7424823	65.692289	60.6353319	57.5318167	57.5554128	50.1156419	30.320622
0.23656123	0.08016143	0.37867498	0.15465542	0.04564267	0.12585545	0.43450732
7.5962441	7.69549695	7.76283718	7.08321829	8.76339355	11.1256221	5.44847467
16.4541481	17.194626	20.7797898	16.0223016	15.0620827	15.6060763	19.8992754

0	0	0	0	0	0.02517109	0
10.0932794	10.3007433	9.6562121	9.8051537	12.2322368	12.8120852	4.6148234
0.02628458	0.24048428	0.14200312	0.06186217	0.04564267	0.05034218	0.03026216
8.22707406	8.41694978	8.23618091	7.73277106	8.26132413	5.61315326	4.72712943
0.23656123	0.12024214	0.09466875	0.18558651	0.5477121	0.25171091	0.18154446
0	0	0	0	0.04564267	0	0
0	0	0	0	0.04564267	0	0
6.09802294	4.68944345	5.11211229	5.13455998	7.21154261	6.92204998	6.93273389
50.7818117	45.3713674	55.3338821	49.953701	46.2360295	38.0838604	33.752047
0	0	0	0.03093108	0	0	0
0	0	0	0	0.04564267	0	0
68.7604656	65.1712398	59.4519726	57.8101964	62.028395	54.4702406	28.7175367
22.9727244	19.7597916	19.6910992	25.6109377	23.2777641	21.1940585	12.6929519
0	0.04008071	0	0.03093108	0	0.10068436	0
17.4529622	16.9942224	14.1056432	18.2184086	12.1409515	8.40714434	7.76644295
161.124485	176.47538	152.984694	151.871624	147.654053	131.015528	61.2781628
54.9084911	62.0850248	61.0140069	52.6137743	76.4058375	74.7077976	16.8342728
0.07885374	0.08016143	0.04733437	0.12372434	0.13692802	0.12585545	0.10961435
0.02628458	0	0	0	0	0	0.01759428
6.4397225	7.61533552	6.34280599	6.49552769	8.1243961	9.79155433	6.1979683
21.553357	28.2969836	22.2944897	19.1463411	23.2777641	21.6471381	39.707687
0.02628458	0	0	0	0	0	0.02933717
12.1171921	9.69953261	11.4075839	14.1355055	10.8629566	13.6175601	11.4461643
0	0.24048428	0.14200312	0	0.04564267	0.07551327	0
4.20553306	4.5291206	5.06477792	3.3405571	4.42733945	3.39809726	5.44398589
2.75988107	2.96597278	3.171403	2.53634891	2.41906176	2.06402945	1.60882625
0.39426872	0.40080713	0.42600936	0.27837976	0.3651414	0.15102655	0.3517893
0.05256916	0.04008071	0.09466875	0.03093108	0	0.02517109	0.02737637
0	0	0	0	0.04564267	0.02517109	0
0	0	0	0	0	0.05034218	0
0.02628458	0.04008071	0.04733437	0.12372434	0.22821337	0.02517109	0.03895038
0.23656123	0.16032285	0.42600936	0.43303518	0.50206942	0.32722418	0.68900446
72.8345757	72.9468981	77.1076937	64.4603795	69.1942949	68.1884851	88.742494
0	0.04008071	0.04733437	0.03093108	0.09128535	0.07551327	0
0.97252952	0.96193712	0.99402183	1.23724337	0.82156815	1.68646309	0.29367589
62.452166	64.4898676	66.5047942	57.3771612	56.7338447	61.9460545	122.824461
0.02628458	0.24048428	0.14200312	0.24744867	0.22821337	0.17619764	0.08417158
0	0	0.04733437	0	0	0	0
42.8175835	48.1770173	38.8141859	44.5098302	52.8542173	45.9372408	17.1675252
0.02628458	0.08016143	0	0.09279325	0.27385605	0.02517109	0.03827803
0	0	0	0.03093108	0	0	0
6.54486083	5.17041201	7.76283718	4.91804239	6.43561714	4.6818229	2.01795384
11.0395243	8.57727264	11.3602495	12.3415026	10.5891005	12.3841767	17.4026901
0	0	0	0.03093108	0	0	0
0	0	0	0.03093108	0	0.02517109	0

51.1497959	55.1911422	41.3229077	68.2649029	71.3395006	39.0907041	40.1996737
9.96185644	13.0663125	10.4608964	9.68142936	9.85881774	9.84189652	2.52197688
0.97252952	0.84169498	0.61534685	0.74234602	0.59335477	0.50342182	1.20291309
38.9800346	40.2009554	42.1749264	44.1695883	44.1364665	45.8113853	173.395281
3.5747031	3.36677991	3.40807486	4.48500721	4.29041142	4.00220344	4.7686823
40.8462399	49.0187123	49.9377636	42.0353435	42.3107595	41.6833264	24.7665942
9.19960358	7.93598123	8.94619651	8.53697925	10.0413884	11.5787018	4.85807882
1.41936741	0.88177569	1.46736556	1.60841638	2.51034711	1.33406781	0.94620777
0	0	0	0	0	0.02517109	0
0.13142291	0.48096856	0.18933749	0.09279325	0.1825707	0.15102655	0.05515187
49.4938672	60.4016349	62.2447006	50.7579092	55.7297058	48.9074295	28.2393279
1.31422908	1.92387424	1.27802807	1.5774853	0.5477121	1.03201472	0.49827277
8.41106613	7.09428625	7.00548721	10.9186727	7.48539866	9.03642161	4.64730503
1.94505904	1.8036321	1.42003119	2.50541782	1.64313629	2.44159581	3.3231314
18.1100768	15.9120432	14.5316525	15.6820597	12.5060929	13.4161914	17.8948225
0	0	0	0	0.04564267	0	0
0	0	0	0	0	0.02517109	0
0	0	0	0	0	0.02517109	0
0	0	0	0	0.04564267	0	0
28.9130398	27.816015	37.3468203	30.2506004	26.6096794	26.9330672	32.4187863
51.0709421	46.1729817	49.5117542	43.5509666	45.7796028	42.9922231	17.8804612
30.6741068	28.7378714	30.9093456	27.0956298	27.0204634	26.0017368	29.2464405
22.7624477	24.6095579	20.0224398	22.3013117	27.1117488	26.6813563	9.32361195
0	0	0	0.03093108	0	0	0
0.02628458	0	0	0	0	0	0.02369458
0.02628458	0	0	0	0	0	0.07349381
0	0	0	0	0	0.05034218	0
1.49822115	1.2024214	1.18335933	1.11351903	0.63899745	1.23338345	1.44595809
0	0	0.04733437	0	0	0	0
0	0	0	0.03093108	0	0	0
32.7505887	24.1686701	30.4833363	25.5490756	41.671762	36.2463708	32.5321258
0.47312247	0.44088785	0.42600936	0.46396626	0.1825707	1.53543654	0.2668959
1.8662053	1.56314782	1.13602495	1.82493397	1.36928024	2.99535981	1.24442707
70.3638251	69.4999568	70.1495409	67.8318677	67.4598733	54.5709249	107.125116
27.0731191	27.2148043	27.406602	26.1676973	24.6470444	21.9491912	17.1863767
0.02628458	0.04008071	0.04733437	0.12372434	0.09128535	0.07551327	0.03262663
0	0.04008071	0	0.06186217	0.09128535	0	0
0.44683789	0.36072642	0.23667187	0.15465542	0.09128535	0.32722418	0.86352324
0	0	0	0	0.04564267	0	0
160.809071	177.196833	224.364928	188.555889	301.69808	181.936645	206.416965
0	0	0	0	0.04564267	0.02517109	0
2.73359649	2.80564993	3.40807486	3.80452336	2.55598978	4.43011199	5.52265621
8.20078947	10.901954	8.28351529	8.87722117	6.52690249	7.29961634	11.8580437
0	0	0	0.03093108	0	0	0
0.05256916	0.04008071	0.04733437	0	0.04564267	0	0.03854185

0.34169956	0.6012107	0.42600936	0.15465542	0.45642675	0.276882	0.62856548
0	0.04008071	0.04733437	0.03093108	0.09128535	0.10068436	0
0	0	0	0	0	0.02517109	0
0	0.04008071	0	0	0	0	0
0	0	0	0	0.04564267	0	0
0	0	0	0.03093108	0.04564267	0	0
0.76225287	0.80161427	1.13602495	0.74234602	1.46056559	1.61094981	0.62308901
3.81126434	4.16839418	4.11809046	3.24776384	3.83398468	2.21505599	3.82780258
4.10039474	4.16839418	4.07075608	3.71173011	3.14934456	3.27224181	3.90346498
0.76225287	1.68338996	1.60936868	0.9897947	1.23235222	0.50342182	0.52370273
0	0	0	0	0	0.02517109	0
6.25573043	5.65138057	5.25411541	7.79463323	6.16176109	6.01589071	2.70455661
0	0.04008071	0	0	0	0	0
39.5057262	40.1608747	38.577514	35.0449184	36.1489984	33.1754977	31.8253363
0	0	0	0	0	0.02517109	0
0	0	0.04733437	0	0	0	0
0.02628458	0.08016143	0.04733437	0.03093108	0	0	0.01058553
0.10513833	0.12024214	0	0	0.04564267	0.05034218	0.02626387
0.02628458	0	0	0	0	0	0.04659934
52.8582937	53.5077522	58.4579507	52.5519121	50.3895129	44.628344	22.3353272
4.02154099	4.8497663	3.97608734	4.67059372	5.11197957	5.61315326	2.242759
1.78735155	2.00403566	2.13004679	2.13424481	2.51034711	2.81916217	0.45722309
3.78497976	2.7254885	3.02939988	2.62914216	2.05392036	2.76881999	2.22654965
0.02628458	0	0	0	0	0	0.01163087
0	0	0	0	0.04564267	0	0
86.5814119	95.5123397	109.484405	112.063318	101.509309	114.301923	52.4975298
0.21027665	0.32064571	0	0.30931084	0.13692802	0.52859291	0.59820543
0.02628458	0.08016143	0.18933749	0	0.04564267	0.05034218	0.03952494
50.5715351	49.5397616	48.3283949	53.1086716	52.5347186	58.2962464	86.2809124
55.6444593	54.4696893	57.2745914	66.7183487	53.4019294	68.2891694	56.890136
0	0.04008071	0	0	0	0	0
0	0	0.04733437	0	0	0	0
0.10513833	0.16032285	0.09466875	0.03093108	0.09128535	0.07551327	0.11039843
0	0	0	0	0	0.02517109	0
239.163408	267.699084	249.310143	242.530631	226.981021	199.531237	77.0617874
0.07885374	0	0.04733437	0	0	0.02517109	0.20393367
0	0	0	0.03093108	0	0	0
8.06936657	9.21856405	10.271559	7.4543913	8.4895375	7.9540647	11.0292037
4.75750928	11.9039718	5.06477792	7.20694262	4.56426747	2.59262236	7.61254551
0	0	0	0	0	0.02517109	0
28.7816169	24.3690737	29.820655	34.5809522	26.0619673	32.1938252	24.7161307
0.55197621	0.40080713	0.18933749	0.46396626	0.22821337	1.20821236	0.20696533
63.8715334	65.5319662	58.6472882	59.7897858	58.6051944	52.5320666	41.5601629
0.55197621	0.76153355	0.23667187	0.40210409	0.3651414	0.32722418	0.21873158
97.3580904	88.5382956	70.1968753	109.465107	88.957573	91.2703754	50.4433369

14.7982195	13.7877654	17.3717149	17.2286139	17.0247177	18.1986987	13.8982295
7.22825995	6.29267198	6.91081847	8.25859949	6.89204388	8.28128888	6.77565055
9.8830027	9.09832191	13.8216369	12.7436067	11.2737407	15.505392	8.35792027
0.47312247	0.64129141	0.7100156	0.46396626	0.27385605	0.47825073	0.31191795
0.18399207	0.12024214	0.09466875	0.21651759	0.1825707	0.30205309	0.26065407
47.7328003	44.8102374	39.5242015	55.4903651	49.248446	44.3514621	14.6636945
0	0.04008071	0	0	0	0	0
0.2891304	0.32064571	0.18933749	0.18558651	0.1825707	0.15102655	0.14977301
7.88537449	6.37283341	7.76283718	7.23787371	6.02483306	5.83969307	5.1583222
23.4721314	27.6156114	24.8978802	24.3736944	32.0411577	28.6195303	10.5388558
0.05256916	0.04008071	0.18933749	0.24744867	0.09128535	0.15102655	0.06144967
0.02628458	0	0	0	0	0	0.05059566
46.18201	41.4835382	43.3109514	42.6539651	39.2070576	37.7062941	33.2218599
1.73478239	1.72347067	2.31938428	1.91772722	2.41906176	2.76881999	2.46616357
35.4316161	36.6738526	36.3527985	33.2509155	32.8627258	32.7224181	14.7877769
0.07885374	0.04008071	0.14200312	0.03093108	0	0.05034218	0.24175595
0	0	0.04733437	0	0	0	0
0.05256916	0.24048428	0.04733437	0.18558651	0.09128535	0.276882	0.03537243
0.60454538	0.40080713	0.66268122	0.64955277	0.59335477	1.08235691	0.96293159
0.05256916	0.04008071	0.14200312	0.03093108	0	0.02517109	0.0467858
18.1889305	19.3990652	18.6970774	14.5994718	19.2155661	14.7754303	7.88177501
2.2867586	2.36476208	2.55605615	2.38169349	3.24062991	3.19672854	2.79316837
4.59980179	5.450977	5.58545602	4.23755854	4.24476875	4.78250726	5.13869388
0.13142291	0.24048428	0.47334373	0.37117301	0.3651414	0.42790854	0.06904347
0	0	0.04733437	0	0	0	0
1.13023701	0.88177569	1.04135621	1.94865831	1.04978152	1.00684363	1.18729265
31.4100751	36.7139333	39.6188703	32.6632249	29.4851679	31.4890346	34.5069441
0.05256916	0.16032285	0.09466875	0.15465542	0.22821337	0.17619764	0.02729503
0	0	0	0	0	0.02517109	0
0.02628458	0	0	0	0	0	0.02243792
28.7816169	26.8540779	32.7080518	27.157492	27.3856048	24.5166425	30.5228679
0	0	0	0	0	0.02517109	0
0	0	0.04733437	0	0	0	0
0.15770749	0.56112999	0.94668746	0.27837976	1.27799489	1.00684363	0.12755124
12.0383384	10.7817119	9.75088085	12.3105715	7.21154261	8.2309467	20.7559156
0.13142291	0.12024214	0.23667187	0.21651759	0.45642675	0.22653982	0.08152521
23.4721314	18.1164824	17.6083868	22.2703806	22.2736253	25.0955776	13.0484493
0.05256916	0	0	0.03093108	0.09128535	0.07551327	0.04066208
20.8173887	19.2788231	20.1644429	20.0742737	24.8296151	22.9560348	19.8175914
1.20909076	1.60322853	0.75734997	0.95886361	0.68464012	0.93133036	1.31122599
0.73596829	0.56112999	0.42600936	1.05165686	0.59335477	0.72996163	3.35932525
1.07766785	0.80161427	0.75734997	0.86607036	0.31949872	0.70479054	3.27120399
0.05256916	0.04008071	0	0.12372434	0.09128535	0.05034218	0.0821291
0.86739119	1.00201783	0.56801248	0.80420819	1.46056559	1.20821236	1.08493546
39.0063192	47.1349188	43.642292	39.282477	39.3896283	37.8069784	48.2954007

14.5090891	13.3468775	14.2949807	14.8159893	15.7923655	16.61292	12.1359009
10.2509868	9.29872548	7.76283718	10.0216713	11.3193833	11.2263065	5.0079398
6.78142207	7.01412482	5.77479351	7.02135612	3.10370188	5.36144235	5.67027598
161.229624	171.024403	184.414717	154.469835	161.164284	141.587386	201.04977
0.2891304	0.28056499	0.14200312	0.18558651	0.1825707	0.10068436	0.36072306
0.02628458	0.04008071	0.18933749	0.03093108	0	0.05034218	0.06522017
0	0	0	0.03093108	0.04564267	0	0
0.26284582	0.28056499	0.09466875	0.06186217	0.1825707	0.12585545	0.57986549
54.1199536	60.241312	67.3568129	64.7387593	66.3188064	76.3942607	24.5245943
0.02628458	0	0.04733437	0.06186217	0.09128535	0.07551327	0.03299491
3.83754892	3.84774847	5.72745914	3.49521252	2.28213374	4.58113853	3.85261968
0.15770749	0.04008071	0	0.21651759	0.22821337	0.07551327	0.29010714
1.78735155	1.04209854	1.13602495	1.23724337	2.14520571	2.4667669	3.60501374
54.8559219	51.4235551	57.3219258	63.7489646	58.4682663	58.5982995	70.1195836
0.05256916	0	0.04733437	0.06186217	0.1825707	0.07551327	0.06313901
0.97252952	1.2024214	0.80468434	1.23724337	1.00413884	1.78714745	1.58307524
4.23181765	3.68742562	4.63876856	3.15497059	2.92113118	3.95186126	4.83579655
0.10513833	0.28056499	0.04733437	0.40210409	0.1825707	0.22653982	0.18165843
37.9023667	42.9665246	40.9915671	44.819141	46.5555282	44.8297128	77.7943072
0.23656123	0.44088785	0.33134061	0.37117301	0.50206942	0.52859291	0.71540637
73.1237061	75.1513374	76.9656906	66.9657974	62.6673924	72.593426	43.3492382
148.087333	130.703206	161.59955	139.004293	135.011032	122.079791	143.943626
90.5240992	93.0273355	96.1834461	106.12455	103.471944	102.44634	25.5861362
1.76106697	1.64330924	1.08869058	2.22703806	1.55185094	2.29056927	4.98677614
48.2584919	38.1167583	45.3936638	50.6032538	46.0991015	51.0469722	37.6251769
50.702958	66.0129347	63.191388	55.8924692	56.2317753	46.541347	41.4461623
30.7792451	34.9103012	33.6074049	32.2920519	28.8918131	25.4228017	28.1193408
0.63082996	0.80161427	0.61534685	0.5876906	0.59335477	1.61094981	0.94918204
0.02628458	0.08016143	0.09466875	0	0.04564267	0.02517109	0.01457219
5.96660003	9.61937118	7.52616532	5.47480191	6.20740376	7.29961634	3.46245444
0.34169956	0.72145284	0.37867498	0.80420819	0.3651414	0.78030382	0.28309925
14.3513816	10.9821154	11.0762433	12.7745378	14.7882266	15.782274	14.4373633
2.47075067	2.52508494	2.17738116	3.18590168	3.56012863	2.69330672	3.30862936
37.192683	36.9143369	38.956189	33.065329	34.0494353	35.2395272	11.9776916
23.6561235	27.8961764	26.5072489	21.311517	24.692687	23.6104832	22.3806555
21.8424873	20.401083	17.9397274	21.2805859	21.8628412	25.1962619	15.7684232
14.009682	14.6695411	17.7503899	10.8258795	15.9292935	12.3590056	20.2934727
3.25928812	4.48903989	3.59741235	2.84565975	4.56426747	1.45992327	2.45658606
25.6274671	34.389252	28.0219489	27.899838	34.8253608	42.5139724	32.2859971
14.009682	16.1926082	15.7623462	16.9193031	16.8877897	17.4183949	18.3882379
10.8555322	12.2246175	10.1295558	13.1457108	11.9127381	13.8441	24.0134045
6.64999916	5.33073486	7.76283718	5.41293974	9.63060437	8.88539506	4.1525503
0	0	0	0	0.04564267	0	0
0.02628458	0	0	0	0	0	0.05194433
0	0	0	0	0.04564267	0	0

0	0	0.04733437	0	0	0	0
0.13142291	0.40080713	0.33134061	0.34024193	0.22821337	0.65444836	0.14668587
11.7229234	11.6234068	14.2949807	10.8258795	9.63060437	11.2263065	19.8897082
2.44446609	2.60524636	2.22471553	2.81472866	2.55598978	3.47361054	3.38458334
0.86739119	0.80161427	0.89935309	0.74234602	0.3651414	0.70479054	0.86020825
43.8689668	46.2531431	45.8670075	44.7263478	44.8211066	32.0427986	32.7520666
10.1458485	9.94001689	7.3841622	9.77422262	12.0496661	11.3269909	6.93754449
95.2290393	94.3099183	90.1246463	88.4010387	93.1566991	69.5728951	17.5084214
56.1964356	61.4838141	62.9547162	50.8197714	59.2898345	54.3695562	50.9972345
87.396234	88.899022	100.206868	91.6178715	84.0281642	65.0672698	44.4534412
31.8306284	40.8021661	36.0214579	28.8896327	39.0244869	28.7957279	26.0803342
62.8201501	57.1150164	61.6766881	54.345915	62.439179	45.735872	39.7552843
55.8021668	60.2813927	60.6353319	67.955592	71.6133567	57.641798	30.0316626
48.8630373	55.8725143	48.0443887	56.542022	58.513909	53.5640813	32.5918397
0.15770749	0.12024214	0.04733437	0.06186217	0.04564267	0.05034218	0.20361082
69.6804259	73.3477053	77.0603593	80.5754744	73.8954904	94.4419328	143.984863
9.38359565	8.29670764	6.29547162	6.77390745	5.47712097	7.6771827	15.0640609
0.65711454	0.48096856	0.66268122	1.02072578	0.59335477	0.85581709	0.63145211
3.36442645	3.36677991	3.36074049	3.37148818	4.1534834	5.41178453	2.4883031
0.63082996	0.6012107	0.80468434	0.52582843	0.91285349	1.40958109	0.80677993
0	0	0	0	0	0.02517109	0
28.965609	26.97432	35.8794548	26.4151459	28.2984583	27.3609757	56.9208079
0.73596829	0.72145284	0.7100156	0.46396626	0.68464012	0.50342182	1.01214072
1.34051366	0.80161427	0.85201872	0.5876906	0.77592547	0.72996163	0.34573701
202.259856	214.712381	169.551724	165.605025	202.653476	146.873315	46.9089967
65.2120471	71.0230239	65.6527754	59.5423371	62.9412485	60.5364735	13.8530758
0.07885374	0	0.04733437	0.03093108	0.1825707	0.17619764	0.12658875
0.02628458	0	0	0	0	0	0.03855341
0	0	0	0.03093108	0	0	0
3.41699561	2.56516565	3.31340611	4.17569637	2.96677386	4.43011199	2.50222114
0.02628458	0	0	0.03093108	0.04564267	0.07551327	0.01023021
0.15770749	0	0.33134061	0	0.13692802	0.05034218	0.08931539
0	0	0	0	0.04564267	0	0
0	0.04008071	0	0	0	0	0
0.02628458	0	0.14200312	0	0	0.02517109	0.03075422
0.02628458	0	0	0	0	0	0.03210538
0	0	0.04733437	0	0	0	0
46.6025633	46.413466	54.1505228	49.3041483	43.5431117	39.9465212	26.350002
0.26284582	0.16032285	0.37867498	0.12372434	0.31949872	0.07551327	0.99546569
22.7624477	27.816015	28.2586207	26.0439729	29.3025972	32.5210494	47.8435861
40.0051333	40.3211975	43.4056201	47.8194562	42.5389728	48.2026389	131.907521
36.4304302	43.7681389	49.0384105	44.78821	30.3980214	37.5300964	30.5761981
0.15770749	0.08016143	0.18933749	0.21651759	0.41078407	0.22653982	0.24282096
0.02628458	0.08016143	0	0.18558651	0	0.10068436	0.0926615
416.137497	538.123656	510.737885	489.855581	437.53068	346.404552	128.890966

0	0	0	0	0	0.02517109	0
0	0	0	0	0.09128535	0	0
1.83992072	2.00403566	1.08869058	1.36096771	0.63899745	1.35923891	1.03411345
0	0	0	0.03093108	0	0.02517109	0
2.99644231	1.32266354	3.55007798	2.47448674	3.74269933	4.05254562	3.96056591
0.02628458	0	0	0	0	0	0.06469525
0	0	0	0	0	0.02517109	0
0	0	0	0	0.04564267	0	0
0.05256916	0.04008071	0.09466875	0	0	0.05034218	0.08851629
0.02628458	0	0	0	0	0	0.02727188
0.02628458	0	0	0	0	0	0.04456485
1.34051366	1.5230671	1.46736556	1.48469204	1.68877897	2.56745127	2.18498418
2.12905111	1.68338996	2.08271241	1.45376096	2.82984583	3.27224181	3.21337061
85.6877362	97.1155682	89.698637	88.3082455	82.3850279	64.9665854	33.5748065
0.05256916	0.48096856	0.37867498	0.77327711	0.7302828	0.12585545	0.04389562
40.6096786	44.6499146	37.0154797	43.334449	26.244538	34.2326835	100.155689
0	0	0	0	0	0.02517109	0
2.52331984	3.76758705	3.88141859	4.05197203	3.46884328	4.88319162	2.71123408
34.2488099	35.1908662	30.1046613	37.9524403	30.8544481	42.8411966	72.2969194
673.200705	693.797147	701.069399	690.443662	562.865465	566.928479	405.273873
81.8764718	91.4241069	89.4146307	77.1730551	86.6754393	73.0465056	58.9513139
12.0120538	14.2286532	13.8216369	13.2694351	16.4770056	16.5625778	5.88001943
8.85790402	7.21452839	5.53812165	6.65018311	5.38583562	6.26760162	1.79132808
3.18043438	3.96799061	4.2127592	2.72193541	3.10370188	3.54912381	2.6565917
0.94624494	0.84169498	0.5206781	0.89700144	0.86721082	1.43475218	0.46468386
0	0	0	0	0	0.02517109	0
29.9381385	29.3791628	31.9033674	29.7866341	34.0494353	37.0770168	70.3609683
36.3252918	37.5556283	29.9153238	41.0764799	36.2859264	38.1845448	18.7732128
0	0	0	0	0.04564267	0	0
11.4863622	11.2626804	11.3129152	13.238504	11.09117	16.638091	17.3899724
38.2703509	35.5115119	41.5122452	43.4891044	39.572199	43.8983824	27.9329097
10.1195639	9.33880619	8.52018715	10.4856376	10.9085993	10.3956605	23.2690104
0.7096837	1.36274425	1.13602495	1.26817445	1.68877897	1.33406781	0.82444979
0	0	0	0.03093108	0	0	0
0	0	0	0	0.04564267	0	0
0	0	0	0.03093108	0	0	0
55.6181748	57.0749357	59.2153007	63.8726889	54.7712097	77.7534996	67.9349917
46.6025633	54.9506579	50.7897823	47.3245589	48.4268779	41.3812733	15.862056
58.8248937	56.5939671	53.5351759	64.8006215	58.5595517	67.6598922	25.0686732
9.46244939	9.89993618	9.46687461	10.8568106	10.1783165	11.6542151	8.64471621
29.0181781	28.2569028	23.0991741	31.0238775	29.3938825	31.7659166	25.9295179
14.9033578	14.6695411	12.8276151	14.7850583	17.6637151	15.9836427	19.2108094
0.84110661	0.64129141	0.66268122	1.05165686	0.59335477	1.35923891	0.53311572
0.86739119	0.68137213	0.5206781	0.30931084	0.41078407	0.67961945	0.95248373
5.86146171	9.09832191	6.39014036	6.52645877	5.02069422	5.4872978	6.32575191

0	0	0	0	0	0.05034218	0
8.22707406	7.33477053	8.66219027	6.09342359	6.20740376	7.7023538	24.134433
39.6108645	40.4815204	36.7314735	42.7158273	33.3647952	27.5875156	32.3359275
16.1913023	16.8338996	16.1410212	15.0015758	14.2405145	14.8761147	18.8275491
35.247624	35.4714312	17.892393	35.972851	13.9210158	19.9355039	13.3740383
0.10513833	0.04008071	0.14200312	0.09279325	0.1825707	0.25171091	0.04643949
3.20671896	2.64532708	3.83408422	1.97958939	2.78420316	2.71847781	1.8728728
11.2235164	13.6675232	9.60887773	9.65049828	9.53931902	10.5970292	3.59695109
164.541481	149.380818	166.616993	170.615861	106.210504	330.89916	72.3512476
49.8092822	46.0527395	40.4235546	44.4788991	20.9499877	44.049409	11.2408378
0	0	0	0.03093108	0	0.02517109	0
0.5782608	0.84169498	0.47334373	1.23724337	0.68464012	0.75513273	0.32665801
0	0	0	0.03093108	0	0	0
2.54960442	2.48500422	2.55605615	1.67027855	2.09956304	2.24022708	2.78348375
64.2658021	66.5740647	75.0723157	67.553488	55.6384205	60.9895531	90.7275354
18.6094838	14.5492989	12.4489401	17.3523383	16.8877897	19.6082798	12.6120263
28.2559253	26.132625	29.5839832	26.7244568	24.692687	24.5418136	30.602087
478.983931	452.030284	473.01239	512.404341	731.515148	451.695225	1778.25187
0.10513833	0.52104927	0.66268122	0.18558651	0.09128535	0.35239527	0.473717
0.02628458	0	0	0	0	0	0.03120188
318.201145	262.167945	274.539364	211.506754	273.445264	258.230221	117.633961
5.65118505	6.45299483	6.20080287	6.12435468	7.25718528	7.9540647	2.8056053
17.3215393	16.7938189	17.3717149	19.5484452	20.2197049	19.7844774	8.15752992
0.10513833	0.04008071	0	0.12372434	0.13692802	0.12585545	0.05613888
39.1903112	34.5094941	31.1933518	37.8905782	37.1074946	40.9030226	27.2391651
0.07885374	0.04008071	0.04733437	0.09279325	0.04564267	0.17619764	0.08428189
1.05138327	0.72145284	0.7100156	1.36096771	0.95849617	1.25855454	1.06955648
62.7150118	54.7101736	59.7833132	64.7078282	76.1319815	56.2322169	48.1899186
46.7865553	49.0187123	49.6537573	48.5308712	42.4020448	41.7336686	21.5365209
60.6385299	62.2854284	64.3747474	57.0059882	51.3480091	56.9370075	57.9517349
0	0.04008071	0	0	0.13692802	0.02517109	0
16.6644248	17.2347067	46.8610293	7.4543913	31.4934456	18.4504096	11.6288337
5.30948549	5.37081558	4.2127592	3.86638553	4.1534834	4.20357217	4.52141693
0	0	0	0	0.04564267	0	0
3.52213394	4.60928202	3.36074049	4.45407613	4.1534834	5.23558689	5.74476611
0	0	0	0	0	0.02517109	0
0	0	0	0.03093108	0	0	0
1.34051366	0.76153355	0.33134061	1.08258795	1.50620827	1.13269909	3.96094354
89.2887239	93.7087076	94.8107492	94.0923582	83.9825215	69.4218685	60.61353
0	0.04008071	0	0	0	0	0
0.02628458	0	0	0	0	0	0.04718055
0.02628458	0	0	0	0	0	0.00493834
0.02628458	0	0	0	0	0	0.0485703
35.0373473	34.7900591	31.9507018	48.7783198	33.7299366	36.9259903	10.6486139
44.2895201	48.2972595	41.9382545	43.2725868	44.0908238	34.358539	28.8876128

0	0	0	0.03093108	0	0	0
6.59742999	8.13638479	6.91081847	7.29973588	7.43975598	10.1942918	26.8124218
0.36798414	0.08016143	0.42600936	0.15465542	0.13692802	0.20136873	0.27985675
0.10513833	0.32064571	0.09466875	0.12372434	0.04564267	0.05034218	0.07400125
13.5628441	13.9080075	12.1175995	16.0841638	14.9707973	13.9951265	9.00473678
3.96897183	2.32468137	2.74539364	3.49521252	5.79661969	3.07087308	7.21960564
132.448007	118.879396	98.9761741	136.344219	115.339039	166.557108	134.905659
0	0	0	0.03093108	0	0	0
34.4590865	39.0386147	40.7548952	33.0034669	36.0120704	34.2075124	19.6061829
0	0	0	0	0.04564267	0	0
0.44683789	0.64129141	0.28400624	0.12372434	0.50206942	0.276882	1.23110407
0	0	0	0	0	0.02517109	0
2.18162028	2.44492351	0.94668746	1.70120963	3.51448595	2.49193799	0.89993052
134.577058	144.250487	136.322994	125.116236	135.056675	117.246941	104.292767
78.2491996	80.5221529	90.0299776	78.3793674	75.9037681	68.4905382	46.0418921
114.390499	114.06971	124.347398	109.032072	109.04035	94.5426172	46.4659517
29.7278618	29.6998085	24.3298678	32.4157763	34.232006	29.4753474	13.1545146
22.0001948	21.7237466	21.1111304	22.9508645	22.1366972	27.6126866	65.3216369
14.7193657	14.0282496	12.2596026	13.578746	11.6845247	10.1187785	7.04321664
0	0	0.04733437	0	0	0	0
3.81126434	4.48903989	6.34280599	4.79431806	6.61818784	7.07307652	1.65975918
8.17450489	6.73355983	7.81017156	6.03156142	7.12025726	5.4872978	12.4607495
12.8268758	10.621389	12.2122683	12.9601243	13.5102317	14.1964952	41.3463197
0.34169956	0.12024214	0.18933749	0	0.09128535	0.20136873	1.28219645
0.02628458	0.04008071	0.14200312	0.03093108	0	0.02517109	0.03624977
0	0	0	0	0	0.02517109	0
0	0	0	0	0.04564267	0.02517109	0
0	0.04008071	0	0	0	0	0
0	0	0	0	0	0.02517109	0
11.4600776	11.7837297	14.9576619	12.8054689	14.9707973	16.4870645	7.79913445
0	0	0	0	0.04564267	0.02517109	0
0.02628458	0.04008071	0.18933749	0.12372434	0.1825707	0.12585545	0.02176213
0	0	0	0	0.04564267	0	0
0.10513833	0.08016143	0.09466875	0	0.1825707	0.40273745	0.12059462
4.67865553	3.84774847	4.97010917	4.17569637	5.34019294	6.84653671	5.28226158
3.99525641	4.76960488	3.83408422	4.82524914	4.5186248	6.44379925	3.28119391
1.41936741	1.48298639	1.27802807	1.14445012	0.50206942	1.28372563	0.60550698
0	0	0	0	0	0.05034218	0
0.05256916	0	0.04733437	0.09279325	0.09128535	0.07551327	0.05071537
2.02391279	1.40282496	1.42003119	1.60841638	3.05805921	2.1898849	1.59730497
0	0	0	0	0	0.05034218	0
0	0.04008071	0	0	0	0	0
0.34169956	0.16032285	0.37867498	0.27837976	0.1825707	0.07551327	0.14694886
0.65711454	1.04209854	0.85201872	0.52582843	0.45642675	0.70479054	0.27785639
0.23656123	0.40080713	0.5206781	0.12372434	0.41078407	0.22653982	0.39969872

0	0.04008071	0	0	0	0	0
0.97252952	0.76153355	0.47334373	0.83513927	1.59749362	0.95650145	0.65965126
20.0551358	16.6735767	16.4250275	12.6817445	21.5433425	31.1114683	22.3740559
24.313238	32.2248935	24.6612084	21.4043103	26.7466074	22.8301794	32.2581628
0	0	0	0	0	0.02517109	0
155.263024	197.918562	221.430197	154.036799	204.342255	130.461764	60.1142493
104.165797	95.8730661	83.1191591	120.012607	101.554951	103.704894	33.9391359
10.9081014	11.7035683	10.6028996	14.7541272	15.6097948	9.8922387	9.43685983
4.8626476	4.40887846	4.11809046	4.17569637	3.69705665	3.2974129	2.11160994
12.6428838	13.2266354	13.5849651	11.6300877	12.5973782	9.28813252	18.4117304
0	0.04008071	0	0	0	0	0
0	0	0	0	0.04564267	0	0
0	0	0	0	0	0.05034218	0
245.208862	232.748702	237.47655	256.913586	276.548966	271.294017	329.264973
2.65474275	2.44492351	2.65072489	2.78379758	2.69291781	3.92669017	2.65753495
6.4397225	5.77162271	4.97010917	5.41293974	3.9709127	4.55596744	4.45818773
0	0	0	0	0	0.02517109	0
0.02628458	0	0	0	0	0	0.00799839
0.02628458	0	0	0	0	0	0.04484455
22.0264794	19.5593881	21.4898054	28.0235623	20.7217743	24.7935245	5.05955646
5.70375422	5.89186485	4.9227748	4.5159383	3.46884328	5.73900871	1.07384664
0.5782608	0.80161427	0.94668746	1.45376096	0.7302828	1.08235691	0.40444707
16.4804327	18.9581774	19.028418	14.9397137	17.6637151	15.1781678	9.89704008
3.96897183	3.24653777	3.50274361	3.43335035	4.65555282	5.03421817	2.93276037
0.02628458	0	0	0	0	0	0.10524891
59.2980162	63.0870427	64.7534224	64.0582754	69.605079	78.6093167	150.494585
9.01561151	10.4610662	9.41954024	7.54718455	7.07461458	9.51467234	34.7907407
38.3754892	36.8341755	45.7723388	38.1689579	35.783857	35.2898693	19.7867949
32.0934742	32.6257006	34.128083	28.1163556	29.3938825	30.1549668	18.401441
52.5428787	59.9206663	68.2088316	61.0270292	54.451711	45.4841611	42.8499532
62.2418893	61.2834106	68.5401722	62.3879969	48.1073792	57.4907715	18.3690197
131.133778	142.406774	142.429129	121.899403	130.355479	123.791425	129.942503
0	0	0	0.03093108	0	0	0
12.0120538	8.7776762	8.52018715	8.53697925	11.3193833	16.6884332	4.90705245
0.02628458	0	0	0	0	0	0.12248968
15.4290494	14.6294603	12.9696182	14.4138852	10.7716712	12.0066103	8.51144486
6.83399123	6.73355983	5.58545602	7.48532238	6.98332923	7.65201161	3.36144786
32.5928812	36.3532069	38.8141859	34.890263	28.7092424	31.1618105	27.3929177
41.5559236	39.8001483	44.5416451	43.9530707	33.5930086	34.1319992	24.1323282
13.510275	11.7035683	14.1529775	10.4856376	12.5973782	10.244634	14.9092652
0.26284582	0.40080713	0.18933749	0.34024193	0.50206942	0.47825073	0.30148656
4.02154099	3.00605349	2.74539364	3.55707469	3.87962735	4.4049409	1.96858418
0	0.04008071	0	0.06186217	0	0.05034218	0
33.5391262	34.5896555	35.3587767	30.9929464	32.6801551	27.5623445	20.0233866
2.2867586	3.16637635	1.7513718	2.84565975	2.19084839	4.27908544	2.3195341

35.1950548	40.4414397	35.1221048	38.3545444	43.9082531	40.6513117	10.2010644
95.2027547	104.249935	86.0538902	89.978524	89.1857864	81.5795054	15.657452
0.02628458	0.08016143	0.04733437	0.12372434	0.09128535	0.10068436	0.03442563
101.721331	106.895262	95.284093	94.1542204	98.8620335	73.0465056	31.0155051
60.1391228	61.2433299	58.363282	53.3870514	55.9579192	51.7265917	37.0616138
0.02628458	0	0	0	0	0	0.07121493
2.33932777	3.52710277	4.30742795	3.99010987	4.3360541	4.35459871	0.7134543
14.2988124	16.4731731	13.8689713	12.5580202	12.1865942	13.3406781	6.20597008
0.02628458	0	0	0	0	0	0.01426194
0	0	0	0	0.04564267	0	0
0	0	0	0.03093108	0	0	0
0	0	0	0	0.04564267	0	0
0	0	0	0	0	0.02517109	0
0	0	0	0	0	0.02517109	0
0	0	0	0.03093108	0	0.02517109	0
0	0	0	0	0.04564267	0.02517109	0
0.02628458	0.04008071	0.04733437	0.12372434	0.1825707	0	0.0218212
0	0.20040357	0.14200312	0.09279325	0	0.02517109	0
0	0	0.04733437	0	0	0	0
71.8094771	63.2473655	68.3035003	56.20178	69.5137936	52.657922	16.5523791
0.36798414	0.24048428	0.28400624	0.09279325	0.31949872	0.12585545	0.06413301
3.54841852	0.40080713	4.07075608	0.86607036	2.41906176	1.13269909	0.59531346
400.577024	459.886104	467.190262	392.144286	406.448018	383.557082	345.206041
21.8687719	16.914061	20.6377867	19.2391344	14.3774425	17.0911707	23.6322851
168.484168	167.858027	210.495957	166.625751	164.770056	130.537277	147.375189
110.158682	110.382284	100.538208	111.197248	88.0447196	81.6046765	116.721952
0	0	0	0.03093108	0	0	0
71.0735088	70.1412482	84.8231965	66.8730041	70.5179325	58.6234706	59.0646581
87.8167873	90.9832191	93.2960493	85.8646898	89.8247839	72.5179127	27.9134683
54.6193607	59.0388906	57.7479351	59.0165087	47.3770964	47.1957953	25.8622255
76.6458401	88.4982149	91.4973431	73.8943602	83.6630228	65.4196651	53.0465219
0	0	0	0.03093108	0	0	0
0.02628458	0	0	0.06186217	0	0.05034218	0.01770569
82.3758789	82.886915	77.3443656	75.6265009	79.6464674	67.1816414	20.2612244
0	0	0	0	0	0.02517109	0
1.26165992	0.88177569	0.66268122	0.95886361	0.50206942	0.60410618	2.79090416
47.6013774	66.9748719	61.6293537	47.1389724	49.1571607	53.7402789	48.4644727
0.36798414	0.40080713	0.7100156	0.24744867	0.45642675	0.25171091	0.09933788
25.5748979	23.1666523	25.8919021	21.0640684	31.5847309	34.2075124	7.75831272
0	0	0	0	0	0.02517109	0
22.6310248	20.9221323	20.1171086	26.074904	23.2777641	22.7043239	29.0647763
4.4420943	6.25259127	6.57947786	4.73245589	4.79248085	4.43011199	6.93547995
7.80652075	7.93598123	8.47285278	9.55770503	8.90032157	9.56501452	22.1825828
0.02628458	0	0	0	0	0	0.0331309
87.5539415	98.5183932	104.088286	98.8248141	56.2317753	90.5655848	130.93331

0	0.04008071	0	0	0	0	0
105.453742	120.041736	106.502339	103.247959	102.376519	86.6388947	56.4427956
99.5659953	100.56251	84.5865247	83.6995139	84.621519	85.8837619	55.1619637
0	0	0.04733437	0	0	0	0
0	0	0	0.03093108	0	0	0
217.005506	253.069624	198.851701	188.153785	227.209235	169.753837	92.672243
0	0	0	0	0.04564267	0	0
157.392075	179.681838	199.609051	202.072773	218.674055	187.902193	202.66885
47.0231166	46.4936274	47.4763762	51.0053579	54.1322122	53.0354884	18.0180692
8.09565115	6.97404411	6.01146538	8.01115081	8.07875343	8.15543343	7.23660273
0.7096837	0.32064571	0.56801248	0.46396626	0.04564267	0.47825073	0.32965469
0.07885374	0	0	0.03093108	0.09128535	0.12585545	0.12118169
0	0.04008071	0.04733437	0.09279325	0	0.07551327	0
0.39426872	0.12024214	0.14200312	0.18558651	0.04564267	0.15102655	0.59617197
0.2891304	0.24048428	0.33134061	0.37117301	0.63899745	0.35239527	0.40491008
0	0	0	0.03093108	0.04564267	0	0
34.7482169	40.8422468	33.7020736	32.694156	36.4228544	24.1390761	28.789016
6.28201501	6.37283341	5.15944666	7.17601154	6.84640121	7.6771827	10.4411637
0	0.04008071	0	0	0	0	0
104.165797	106.253971	109.058396	96.1338098	102.650375	91.2703754	103.135966
64.1869484	68.6181811	70.3388784	66.4709	64.0366726	52.4062111	77.1685
0.49940705	0.40080713	0.56801248	0.55675952	0.5477121	0.830646	0.24708485
0.02628458	0	0	0	0	0	0.03857653
0.21027665	0.16032285	0.14200312	0.34024193	0.04564267	0.52859291	0.55258503
0.02628458	0	0	0	0	0	0.04912688
0.31541498	0.44088785	0.33134061	0.40210409	0.59335477	0.553764	0.57119541
0	0	0	0.03093108	0.04564267	0	0
1.70849781	1.84371281	1.42003119	1.54655421	2.82984583	2.29056927	1.162715
0	0	0	0	0	0.05034218	0
0.81482203	0.72145284	0.5206781	0.86607036	0.5477121	1.35923891	0.64327834
0.78853745	1.36274425	0.99402183	0.61862168	0.77592547	0.90615927	0.40717866
22.6047402	22.6055223	20.9217929	20.2907913	18.3027126	19.6082798	27.7491679
0	0	0	0.03093108	0	0	0
3.28557271	2.56516565	2.60339052	3.5261436	3.33191526	3.72532144	3.71631319
23.498416	27.816015	27.1225958	23.1983132	24.9209004	22.1505599	36.7821705
19.1877446	25.3310108	19.6910992	21.3424481	16.4770056	18.1483565	8.4859311
26.9679808	25.0504458	19.9751054	30.4361869	27.2030341	26.2282766	13.1353907
0.9988141	1.00201783	0.37867498	0.95886361	1.18670954	1.18304127	0.9152319
53.594262	61.8846213	53.4878416	60.4084075	65.0864542	62.9277271	36.4633323
45.5774646	42.2450718	38.1041703	49.1185618	46.1447442	43.4453028	24.0113012
1.20909076	1.44290568	1.37269682	0.64955277	1.18670954	1.15787018	0.55209515
0	0	0	0.03093108	0	0	0
94.7559168	100.522429	88.9886214	106.248274	75.4016987	57.7928246	63.6362985
78.9063141	82.60635	84.5865247	77.9154012	71.3395006	74.5567711	123.354546
5.49347756	5.61129986	4.73343731	6.55738986	6.29868911	5.94037744	5.01686466

0	0	0.04733437	0	0	0	0
150.952352	161.886001	158.90149	150.881829	154.911238	113.29508	145.514413
0.02628458	0	0	0	0	0	0.01258209
1.60335948	1.64330924	1.32536245	1.33003662	2.14520571	2.59262236	2.15653221
8.22707406	6.13234913	7.00548721	8.75349684	6.52690249	9.71604106	10.9273166
0.18399207	0.08016143	0.23667187	0.24744867	0.41078407	0.20136873	0.17625277
0.18399207	0.12024214	0.04733437	0.09279325	0.3651414	0.22653982	0.28204861
125.324885	126.053843	126.998123	120.693091	115.932394	102.496682	58.8514728
2.10276653	2.16435852	1.98804367	2.31983132	1.18670954	1.43475218	1.44065155
637.164544	584.056154	686.253741	734.798837	852.970305	629.503811	442.35755
0.31541498	0.24048428	0.61534685	0.68048385	0.31949872	0.65444836	0.26026476
398.263981	417.240225	418.009849	384.194997	383.352825	333.189729	151.254413
4.91521677	5.97202628	5.82212789	7.02135612	6.02483306	6.64516798	3.28743154
55.1713369	51.9446044	48.9910761	54.2840528	60.8416854	59.6303142	33.1566121
43.8426822	43.2871703	47.6657137	40.7362379	41.3066206	41.1547335	51.0538863
56.6958426	61.4036527	64.327413	50.6032538	55.7753485	54.2185297	44.0070999
22.5521711	28.4973871	33.0867268	21.6208279	26.1532526	24.6928401	22.8659252
22.5521711	28.4973871	33.0867268	21.6208279	26.1532526	24.6928401	22.9038927
411.248564	419.124019	448.209179	384.102204	390.883866	377.390165	315.902237
147.614211	156.835831	168.510368	145.901924	144.641636	131.871345	125.31179
49.7829976	48.2571788	57.227257	50.1392875	45.8252454	41.557471	24.7154253
28.9918936	31.3431178	26.8385895	28.5803218	28.0246023	21.7478225	39.7148449
61.9790435	71.0631046	70.1495409	64.4913106	64.4474567	52.5824088	35.8477784
238.874278	250.263974	266.634523	213.795654	238.300405	214.533207	147.358238
36.2990073	36.9544176	33.7020736	40.4887893	39.3896283	38.7634799	20.5146875
0.02628458	0	0	0	0	0	0.03215354
25.1280601	24.3289929	17.7030555	28.3328732	22.593124	27.0840937	16.2596064
47.7853694	45.6920131	44.2103044	44.2314504	41.1240499	38.8138221	38.4320439
21.3167957	17.0743838	21.1111304	21.9610698	19.9002062	25.6241705	64.7863908
13.9571129	12.2646983	14.2003119	9.34118744	11.5475967	13.7685867	3.36888923
10.4086943	9.09832191	7.52616532	8.41325491	4.38169677	8.18060452	7.84764407
17.715808	14.5092182	15.7150119	19.1772722	16.3400776	19.683793	9.69641481
2.2867586	1.56314782	2.84006238	2.78379758	3.05805921	3.0205309	4.4865407
1.44565199	1.56314782	1.46736556	1.88679614	2.00827769	2.06402945	2.76968644
0.05256916	0.12024214	0	0.03093108	0	0.02517109	0.05010291
9.64644146	9.81977475	6.48480911	9.18653202	7.21154261	5.53763998	7.32031625
0	0	0	0	0	0.02517109	0
11.2498009	10.4209854	10.4135621	15.0943691	8.76339355	13.3155071	14.2093091
0	0	0	0.03093108	0.04564267	0	0
0.07885374	0.28056499	0.47334373	0.12372434	0.13692802	0.15102655	0.05556488
29.8067156	26.9342393	35.5007798	28.4256664	27.5225329	25.6241705	30.5505792
59.9025616	59.3595363	52.0678104	53.2942581	59.3811198	38.0838604	23.1598993
0	0	0	0	0	0.02517109	0
1.41936741	1.56314782	1.84604055	1.82493397	2.73856048	1.83748963	1.43732719
57.8786488	59.8805856	52.1624791	61.8003063	66.1362357	63.7080309	31.5447087

0.18399207	0.52104927	0.37867498	0.71141494	0.59335477	0.35239527	0.03652329
211.144044	237.959195	233.547797	195.886556	226.113811	187.801509	106.511093
0.78853745	10.340824	13.3482932	2.72193541	8.35260948	1.33406781	0.49543208
5.20434717	4.68944345	4.16542483	5.59852625	5.24890759	5.58798217	4.15425857
2.94387314	2.52508494	1.89337492	1.79400289	1.32363757	2.31574036	3.10180592
12.9582988	10.1805012	8.66219027	13.8571257	11.0455273	11.905926	6.34321571
3.31185729	3.12629563	2.9820655	2.66007324	2.82984583	1.661292	1.9033809
21.6584953	17.1545453	17.3717149	20.3217223	20.5848463	23.9125363	35.6348599
38.4017738	46.132901	43.3109514	37.1791632	36.057713	39.7199813	23.4295882
30.7792451	29.6998085	29.5366488	27.8070447	29.5308106	24.4159581	12.4592314
19.79229	16.7136574	17.1823774	22.5796915	18.1657845	21.0933741	13.8233611
21.9476257	24.7698808	22.0104835	22.7962091	19.0329954	19.5327665	10.5972794
75.4893185	91.2237034	88.9886214	74.2964643	84.1194495	73.9023227	52.7534828
0	0	0	0.03093108	0	0	0
24.6023684	27.6957729	27.027927	24.7448674	22.5018386	23.1070614	13.6241354
58.4569096	66.9748719	67.498816	61.0888913	52.762932	55.2757155	54.0305831
0.07885374	0.04008071	0.09466875	0.12372434	0.1825707	0.15102655	0.03545043
0.07885374	0.08016143	0	0.03093108	0	0.02517109	0.05738288
89.6829926	94.7107254	91.8760181	90.2259727	88.0447196	66.7537329	39.3218223
40.399402	38.7981304	36.6841391	46.5512818	36.5597825	45.9372408	26.8222489
201.418749	211.345601	245.807399	182.957363	207.445957	176.852084	133.005447
128.137336	112.666885	122.596026	135.849322	137.749592	134.28777	61.9559351
0	0	0.04733437	0	0	0	0
3.49584936	3.00605349	3.73941547	2.84565975	3.69705665	6.24243053	4.83758037
11.7754926	13.7877654	12.5909432	11.5682255	9.67624704	11.6542151	7.00792346
3.23300354	6.0521877	4.63876856	4.60873155	2.87548851	3.2974129	1.99540143
2.89130398	5.25057344	3.97608734	3.89731661	2.32777641	2.81916217	2.00447129
0.60454538	0.32064571	0.23667187	0.77327711	0.50206942	0.50342182	0.23117582
0	0.04008071	0	0	0	0	0
26.4948583	24.6496387	26.5545833	25.5181445	22.730052	21.7981647	19.5069335
3.65355685	3.96799061	4.54409981	4.36128288	5.11197957	4.90836271	4.4918013
113.444254	126.093924	128.749495	114.97084	112.828692	100.785048	60.3893324
0.02628458	0.04008071	0.04733437	0.03093108	0.22821337	0.05034218	0.00669796
0.02628458	0	0	0	0	0	0.02865735
52.1748946	56.6741285	60.8246694	63.8417578	58.8790504	70.0259747	38.7286288
0.21027665	0.32064571	0.14200312	0.12372434	0.1825707	0.12585545	0.20878923
0.02628458	0	0	0	0	0	0.02032462
0.52569163	0.32064571	0.14200312	0.09279325	0.13692802	0.30205309	0.69184597
0.02628458	0.04008071	0	0.03093108	0.09128535	0.07551327	0.04961966
0.02628458	0	0	0	0.1825707	0	0.02352985
0.44683789	0.76153355	0.33134061	0.27837976	0.13692802	0.52859291	0.30881933
0.07885374	0.04008071	0.09466875	0	0.04564267	0.02517109	0.07679986
659.664146	609.106599	780.969821	632.231362	844.435125	1030.60514	2517.80791
24.7337913	24.3690737	28.0692832	28.7349772	26.7922501	30.0794535	17.4994116
119.121724	118.478588	128.134148	112.434491	111.824553	109.066337	65.8025969

31.5940671	35.0706241	31.7613643	33.065329	28.9374558	27.1092648	17.8845706
12.8005913	11.7837297	18.0343961	8.41325491	21.5889851	24.1894183	19.7587064
0.10513833	0.36072642	0.18933749	0.15465542	0.13692802	0.05034218	0.05510461
5.62490047	7.2546091	8.14151217	5.78411275	7.48539866	3.59946599	6.89810318
228.728429	254.953417	253.380899	229.787025	228.259016	204.137547	113.786141
11.6177851	13.1865547	12.0702651	12.2177783	14.4687279	14.976799	19.4549835
38.2440663	34.7900591	25.4185583	43.9840018	36.4228544	33.5782352	25.3362595
24.9966371	19.3990652	18.3657367	25.7655932	22.1366972	23.9880496	26.9291221
5.70375422	4.88984702	5.20678104	6.49552769	6.07047574	5.83969307	10.563692
0.02628458	0	0	0	0	0	0.01391025
31.4363597	28.5775486	34.0334142	27.0956298	29.1656692	27.7385421	47.8899575
2.26047402	2.24451994	1.89337492	2.66007324	2.60163246	2.97018872	3.99884972
0.05256916	0.04008071	0.04733437	0	0	0.02517109	0.0954111
0.55197621	0.52104927	0.56801248	0.37117301	0.13692802	0.45307964	0.3188781
1.44565199	1.32266354	1.9407093	1.29910554	0.86721082	1.30889672	0.77056418
0.07885374	0.04008071	0.09466875	0.09279325	0.13692802	0.17619764	0.06838754
0	0.04008071	0	0	0	0	0
14.009682	10.7015504	11.5022527	13.207573	12.7799489	16.9401441	37.3373371
18.9248988	22.1646344	21.1111304	17.4760626	18.6222113	18.1231854	18.5278511
24.8652142	26.2528672	28.8266332	26.5079392	24.3731883	19.2055423	19.738644
14.5616582	14.0282496	14.5316525	15.4964732	17.9375712	15.6815896	9.88242002
2.97015773	2.92589207	3.02939988	3.74266119	3.10370188	4.32942762	6.84891651
47.7853694	38.1969197	53.1091666	50.8816336	50.5720836	55.0995178	148.93029
80.167974	68.2574547	66.2207879	64.0582754	85.8082285	101.942918	43.3834559
0	0	0	0.03093108	0	0	0
0.02628458	0	0	0	0	0	0.09082921
52.5691633	50.6620216	57.6532664	51.1600133	50.7090116	43.772527	55.1755327
0	0	0.04733437	0	0	0	0
0.02628458	0	0	0	0	0	0.07556649
47.4699545	60.2012313	44.2576388	64.1819998	56.3687033	56.8363231	21.0969287
0.10513833	0	0.04733437	0.03093108	0	0	0.17091584
32.6980196	32.7459427	29.2053082	36.4986794	41.671762	31.7910877	15.3871921
126.402553	137.997896	145.742535	132.415972	125.243499	106.926794	38.2691206
7.83280533	12.8258282	10.0348871	8.41325491	8.03311075	8.83505288	2.60055786
16.9272706	15.230671	15.099665	15.4964732	10.9542419	15.0019701	25.5325288
0.44683789	0.76153355	0.42600936	0.30931084	0.50206942	0.35239527	1.28765656
0	0	0.04733437	0	0	0	0
0.15770749	0.12024214	0.04733437	0.34024193	0.13692802	0.15102655	0.32809736
1.18280617	0.80161427	0.94668746	1.26817445	1.18670954	1.33406781	1.37473575
37.2715368	40.3211975	36.920811	36.8079902	34.1863634	31.3883503	17.0316482
74.0173819	62.205267	67.5461504	69.4402841	56.82513	57.9438511	50.0245157
5.91403087	4.88984702	6.62681223	5.2892154	4.83812352	4.45528308	2.9019442
0	0	0	0.03093108	0	0	0
0	0	0	0	0	0.02517109	0
0.13142291	0.12024214	0.14200312	0.06186217	0.04564267	0.07551327	0.11087428

1.41936741	1.68338996	1.42003119	2.78379758	1.96263501	1.43475218	1.02739127
27.0994037	25.4512529	26.8385895	30.3743247	28.6635997	30.2808223	11.9181382
0	0	0	0	0	0.02517109	0
9.48873397	9.13840262	9.70354648	8.25859949	9.40239099	6.56965471	6.68053442
0	0	0	0.03093108	0	0	0
5.86146171	4.97000844	5.58545602	6.80483853	6.38997446	6.16691725	7.11333313
3.70612601	2.88581135	2.13004679	3.89731661	3.19498723	4.53079635	3.32500871
56.8009809	57.3955814	55.570554	54.717088	49.6135874	45.0814237	54.7332049
4.31067139	4.40887846	4.07075608	3.77359228	3.33191526	3.52395272	3.01410737
0.89367578	0.88177569	0.80468434	0.64955277	0.68464012	0.57893509	0.6826228
65.7903079	67.1351947	61.4873506	62.790101	81.380889	75.0350218	18.7011304
40.1365562	30.1807771	44.2576388	39.6227189	25.9250392	29.1481232	24.9547437
0	0	0	0	0	0.02517109	0
1.44565199	1.60322853	1.70403743	0.89700144	0.95849617	1.51026545	1.79719999
49.3098752	40.8422468	40.1868827	46.7677994	54.588639	46.7678868	30.8621357
53.1211395	56.3935636	59.3573038	55.3357097	48.7007339	49.61222	25.4134954
115.547021	140.923788	142.097788	125.549271	127.662561	63.8590574	112.269237
0.02628458	0	0	0	0	0	0.01593732
0.31541498	0.16032285	0.33134061	0.46396626	0.68464012	0.30205309	0.45906306
41.8187694	44.1288653	40.092214	44.4788991	47.6965951	51.0973144	46.1283001
0.23656123	0.40080713	0.18933749	0.40210409	0.45642675	0.40273745	0.17634484
0	0	0	0	0	0.02517109	0
147.32508	146.855733	114.975192	140.148743	169.836393	155.859394	70.4124247
0	0.04008071	0	0	0	0	0
0.02628458	0	0	0	0	0	0.0500834
38.1914971	42.2450718	40.2815515	37.9833714	50.5720836	45.9372408	32.6364625
48.2847765	43.6879775	51.4524635	47.8503873	42.4476875	39.7199813	18.2021744
5.4409084	4.12831347	5.53812165	6.24807901	4.83812352	7.62684052	5.9908039
99.2768649	94.7107254	116.016548	116.11529	103.7458	127.290206	51.5029376
0	0	0	0.03093108	0	0	0
18.346638	19.5994688	16.5670306	16.517199	17.2985737	11.1256221	11.1768785
7.727667	8.13638479	8.14151217	8.28953057	6.02483306	6.46897034	7.22165107
1.68221323	1.88379352	1.65670306	2.53634891	2.46470444	1.71163418	1.19571567
0	0	0	0	0	0.05034218	0
0	0.04008071	0	0	0	0	0
0	0	0	0	0.04564267	0	0
4.81007844	6.3327527	4.9227748	6.74297636	5.61404899	6.24243053	6.98409273
0.7096837	0.80161427	1.2306937	0.55675952	0.63899745	0.65444836	0.65029635
0.02628458	0	0	0.06186217	0.04564267	0.02517109	0.05819646
0	0	0	0.03093108	0	0	0
0.02628458	0	0	0	0	0	0.08472607
62.3733122	58.0769535	60.5406631	68.6979381	58.513909	80.2706087	138.476142
100.722517	99.520411	100.727546	105.25848	89.4596425	76.0418654	149.984628
24.129246	26.3330286	25.6552302	25.2706958	25.9250392	33.3013532	22.6791788
0.34169956	0.48096856	0.47334373	0.71141494	0.41078407	0.60410618	0.85305315

50.7292426	36.1127226	41.7962514	55.3975719	45.6426747	47.5481906	31.9548586
17.3741085	21.0022937	20.6377867	17.9090978	14.3317999	19.4572532	47.3351694
81.2456419	69.5801182	71.3329002	83.7923072	79.5551821	83.1904552	104.948888
199.683967	192.18702	175.847196	245.469084	190.284311	209.196936	58.3611172
453.487887	459.445216	521.435454	490.226754	399.692903	319.82388	121.044088
7.62252868	6.69347911	5.68012477	5.93876817	9.26546297	8.83505288	17.3479574
24.9703526	24.0885087	23.9985271	23.7860038	22.1823399	19.8599907	24.3490352
27.9667949	27.1747236	20.1171086	34.8284008	25.1034711	29.374663	17.6665987
1.2879445	3.80766776	2.17738116	5.62945733	3.33191526	1.6361209	1.10215008
0.10513833	0.36072642	0	0.09279325	0.45642675	0.45307964	0.27658961
30.1746997	29.9002121	20.0697742	23.6932105	18.6222113	27.3861468	12.6282127
587.74953	573.835572	633.759921	537.303864	501.658638	505.057938	532.187524
0	0	0.04733437	0	0	0	0
152.608281	140.362658	158.617484	135.385356	144.36778	118.002074	59.4438666
28.5976248	25.6917372	25.0872177	26.8791122	20.7217743	22.5029552	12.071447
0.49940705	0.64129141	0.56801248	0.55675952	1.00413884	0.75513273	0.32357907
0.02628458	0	0	0	0	0	0.03153854
0.2891304	0.44088785	0.23667187	0.68048385	0.63899745	0.22653982	0.27009466
0.47312247	0.68137213	0.42600936	0.5876906	0.82156815	0.90615927	0.47733093
0	0.04008071	0	0	0	0	0
0.13142291	0	0	0.15465542	0.09128535	0.10068436	0.12472281
43.9215359	48.1369366	48.7544043	46.1801088	41.6261194	33.3265243	21.3547568
0	0	0.04733437	0	0	0	0
0	0	0	0	0.04564267	0.02517109	0
5.65118505	6.29267198	4.82810605	2.87659083	5.65969167	5.08456035	6.10959917
26.9679808	26.0123829	25.1345521	25.1779026	23.3690495	22.2260732	18.8943492
14.1411049	10.901954	13.064287	9.27932527	14.4230852	7.92889361	2.80184733
0.2891304	0.12024214	0.04733437	0.15465542	0.5477121	0.25171091	0.1683031
0.02628458	0	0	0	0	0	0.00709321
2.31304318	3.00605349	3.21873737	2.3507624	2.41906176	2.24022708	2.19682583
0	0.04008071	0	0	0	0	0
0.05256916	0	0	0.09279325	0.09128535	0.02517109	0.07486273
0	0.04008071	0	0	0	0	0
0	0.04008071	0	0	0	0	0
0	0	0	0	0.04564267	0.02517109	0
122.144451	119.520687	123.82672	143.922335	104.704296	170.357943	82.80272
8.83161943	8.09630408	5.11211229	7.08321829	5.52276364	5.36144235	2.88441864
98.4883274	100.362106	110.336424	91.6797336	92.7002724	90.0873341	37.1219598
0	0	0	0.03093108	0	0.02517109	0
300.169922	326.297087	334.180674	299.320102	288.370419	267.040103	315.86533
7.35968286	6.53315626	7.00548721	10.0835335	7.48539866	7.85338034	5.38134589
0	0	0	0	0	0.02517109	0
21.9476257	23.2468137	18.9810836	18.1565464	29.1200265	27.2351203	11.9697759
0.07885374	0.08016143	0.14200312	0.03093108	0.04564267	0.05034218	0.04825444
0	0.04008071	0	0	0	0	0

0	0.04008071	0	0	0	0	0
0.02628458	0	0	0	0	0	0.04416695
0	0	0	0	0	0.05034218	0
0.10513833	0.12024214	0.09466875	0.06186217	0.04564267	0.02517109	0.12590716
0.13142291	0	0.09466875	0.18558651	0.09128535	0.15102655	0.10168736
17.7420926	18.036321	18.2237336	16.4244057	14.5600132	15.5557341	11.7190284
132.211446	135.232327	166.569659	136.777254	131.085762	122.734239	79.6318634
17.0061243	13.7076039	14.6736556	18.8988925	15.8380081	17.4435659	8.85060262
72.8345757	65.972854	66.8361348	67.4606947	60.476544	55.8043084	43.3458837
0.02628458	0	0	0	0	0	0.02125854
0	0	0	0.03093108	0	0	0
0	0	0	0	0	0.02517109	0
34.4590865	33.547557	42.506267	29.4154611	38.0203481	27.2854625	17.6262986
94.177656	92.3058826	98.3134929	87.1328643	82.4763132	87.5450539	96.4068116
54.409084	51.864443	52.7304916	51.2837376	45.6883174	46.7427157	40.0107191
15.6656107	16.2326889	15.4310056	14.1355055	10.9085993	15.0271412	5.19899914
0	0	0.04733437	0	0	0	0
26.7839887	31.2629563	32.0453706	29.8484963	24.9209004	25.2466041	36.8761497
28.4399173	26.2528672	26.1759083	27.8070447	29.3938825	32.4707072	19.6832431
0.23656123	0.24048428	0.09466875	0.24744867	0.68464012	0.25171091	0.21372369
30.2798381	31.9844092	32.613383	27.4358717	27.9333169	28.3426483	22.5309489
0.15770749	0.24048428	0.37867498	0.5876906	0.27385605	0.276882	0.20002203
26.2845816	29.4593242	24.9452146	24.3118322	24.1449749	24.5669847	18.7648332
0	0.04008071	0	0	0	0	0
0.2891304	0.40080713	0.18933749	0.46396626	0.45642675	0.40273745	0.13883767
0.10513833	0.36072642	0.04733437	0.06186217	0.09128535	0.07551327	0.06533613
8.0167974	4.32871703	4.73343731	5.35107757	10.0870311	7.19893198	7.02243481
0.60454538	0.32064571	0.47334373	0.30931084	0.27385605	0.30205309	1.36444919
4.78379386	4.20847489	4.78077168	6.03156142	5.15762224	5.78935089	5.18559556
6.0191692	6.61331769	5.20678104	5.93876817	4.42733945	4.60630962	4.27097508
0.02628458	0	0	0.03093108	0.09128535	0.05034218	0.03529478
0	0	0	0.03093108	0	0	0
11.9857692	10.1404205	10.3188933	13.7334014	13.053805	10.5466871	13.5633811
0	0.04008071	0	0	0	0	0
101.116786	98.959281	100.112199	95.3914638	99.0446042	74.254718	56.2248522
0	0.04008071	0	0	0	0	0
2.89130398	2.76556921	4.40209669	3.12403951	4.74683817	5.18524471	3.64064805
20.9750962	21.3229395	19.7857679	24.3427633	23.2777641	23.3084301	31.7556018
2.23418944	1.76355138	1.65670306	2.25796915	2.19084839	2.76881999	5.33799032
27.7302336	25.8520601	24.3772021	24.0025214	23.7341909	22.2764154	23.4510795
0.15770749	0.28056499	0.18933749	0.15465542	0	0.15102655	0.19129524
24.8389297	25.5714951	24.5665396	28.1782177	27.5681755	29.2236365	20.9119731
0	0	0	0.03093108	0	0.02517109	0
4.52094804	4.16839418	3.97608734	3.77359228	5.43147829	7.27444525	3.20510529
27.4148187	27.1346429	28.7319644	29.755703	31.858587	32.0931408	14.5021163

0.34169956	0.24048428	0.18933749	0.71141494	0.3651414	0.30205309	0.21342662
95.3078931	56.9947743	104.987639	64.274793	88.2272903	66.3761665	120.256567
0	0.04008071	0	0	0	0	0
0.68339912	0.6012107	1.13602495	0.43303518	0.68464012	0.65444836	0.85436084
0	0	0	0	0	0.02517109	0
102.877853	80.7225565	112.608474	97.4947775	254.001485	72.6437681	66.7279757
0	0	0	0	0	0.02517109	0
0	0	0	0	0.04564267	0	0
0	0	0	0	0	0.02517109	0
0	0	0.04733437	0	0	0	0
3.94268725	5.33073486	5.20678104	3.37148818	4.10784073	4.1280589	4.81821304
0.02628458	0	0	0	0	0	0.01646367
1.13023701	0.88177569	0.80468434	0.95886361	1.50620827	1.28372563	2.030253
0	0.04008071	0	0	0	0	0
0	0	0	0	0.04564267	0	0
0	0	0	0	0	0.02517109	0
0	0	0	0.03093108	0	0.02517109	0
0.02628458	0	0	0	0	0	0.02299967
0	0.04008071	0	0	0	0	0
0	0	0	0.03093108	0.04564267	0	0
0	0.04008071	0	0	0	0	0
0	0	0	0	0	0.05034218	0
0	0	0	0	0	0.02517109	0
0.94624494	0.9218564	0.61534685	0.37117301	0.3651414	0.85581709	3.91056588
0	0	0	0	0	0.02517109	0
0.34169956	0.08016143	0.18933749	0.24744867	0.31949872	0.42790854	1.06767827
1.07766785	0.80161427	0.80468434	1.60841638	0.7302828	1.43475218	1.60181678
0	0	0	0	0	0.02517109	0
0	0.04008071	0	0	0	0	0
0	0	0	0	0	0.05034218	0
0.60454538	0.28056499	0.7100156	0.46396626	1.27799489	0.67961945	2.28957108
0	0.04008071	0	0	0	0	0
0.18399207	0	0.04733437	0.24744867	0.1825707	0.12585545	0.20177032
0.02628458	0.04008071	0.04733437	0.09279325	0	0.12585545	0.02636617
0	0	0.04733437	0	0	0	0
0.02628458	0	0	0	0	0	0.07958797
0	0	0	0	0.04564267	0.02517109	0
0	0	0	0	0	0.02517109	0
0.02628458	0	0	0	0	0	0.02673891
4.88893219	3.72750633	4.68610293	3.58800577	6.89204388	6.77102343	4.23102848
0.02628458	0	0	0.03093108	0	0.12585545	0.08308409
0.02628458	0	0	0	0	0	0.07929357
0	0	0	0	0	0.02517109	0
0.02628458	0	0	0	0	0	0.06329437
0	0	0	0.03093108	0	0	0

0.02628458	0	0	0	0	0	0.02422112
0.18399207	0	0.14200312	0.03093108	0.04564267	0.07551327	0.31435027
0	0	0	0	0	0.02517109	0
1.81363613	2.1242778	1.89337492	2.59821108	1.91699234	3.14638635	0.98823803
2.81245024	3.52710277	2.60339052	2.7528665	2.09956304	2.39125363	3.22137543
0.02628458	0.28056499	0.04733437	0.15465542	0.1825707	0.276882	0.025448
44.4472276	57.6360657	59.9253163	38.6947864	54.6799243	46.541347	29.2950641
0.02628458	0	0	0	0	0	0.0044832
0	0	0	0	0.04564267	0	0
1.26165992	1.16234068	1.56203431	0.77327711	0.91285349	1.18304127	0.99926837
0	0	0	0	0	0.02517109	0
0.05256916	0.08016143	0.09466875	0	0	0.07551327	0.31066224
0.78853745	0.72145284	0.7100156	0.34024193	0.63899745	0.57893509	2.85386465
0	0	0	0	0	0.02517109	0
0.49940705	0.32064571	0.37867498	0.12372434	0.31949872	0.32722418	1.50843776
0.23656123	0.36072642	0.09466875	0.30931084	0.41078407	0.37756636	0.18562019
0	0	0	0	0	0.02517109	0
0	0	0	0.03093108	0	0	0
0.63082996	0.96193712	0.99402183	0.43303518	0.91285349	0.37756636	1.10635842
0.68339912	0.40080713	0.7100156	0.43303518	0.3651414	0.45307964	0.67067155
0	0.04008071	0	0	0.04564267	0.10068436	0
0	0	0	0.03093108	0	0	0
0.10513833	0	0.04733437	0.03093108	0	0.02517109	0.1660609
0.02628458	0	0	0	0	0	0.09019226
0.65711454	0.56112999	0.56801248	0.46396626	0.91285349	1.05718582	1.21886056
0	0	0	0.03093108	0	0.02517109	0
0.13142291	0.52104927	0.18933749	0.27837976	0.50206942	0.52859291	0.21181516
0	0	0	0.03093108	0.04564267	0	0
0.02628458	0	0.04733437	0.06186217	0.09128535	0.05034218	0.02624779
0.49940705	0.52104927	0.42600936	0.27837976	0.27385605	0.35239527	0.49049963
0.02628458	0	0	0	0	0	0.05491638
9.38359565	7.81573909	6.81614972	7.91835756	6.48125981	6.24243053	4.53708078
0	0.04008071	0	0	0.09128535	0.07551327	0
0.55197621	0.48096856	0.66268122	0.40210409	1.04978152	0.90615927	1.57947222
0	0	0	0	0.04564267	0	0
106.137141	97.7568596	86.6219027	104.918238	106.758216	114.629148	184.68848
0	0	0	0	0	0.02517109	0
0	0	0.04733437	0	0	0	0
0	0	0	0.03093108	0	0	0
0	0	0	0	0	0.02517109	0
0	0.04008071	0	0	0	0	0
0	0	0	0	0.04564267	0	0
0	0	0.04733437	0	0	0	0
0.18399207	0.28056499	0.09466875	0.12372434	0.13692802	0.07551327	0.40335984
0.18399207	0.08016143	0.28400624	0.09279325	0.1825707	0.05034218	0.39417652

0	0	0	0	0	0.02517109	0
0	0	0	0	0	0.02517109	0
0.02628458	0	0	0	0	0	0.04109079
0.47312247	0.36072642	0.47334373	0.46396626	0.63899745	0.85581709	0.46468386
0.31541498	0.32064571	0.56801248	0.12372434	0.27385605	0.30205309	0.41421632
0	0	0.04733437	0	0	0	0
0	0	0	0.03093108	0	0	0
0	0	0	0	0	0.02517109	0
0.63082996	0.6012107	0.61534685	0.74234602	0.63899745	1.03201472	2.45759554
0.02628458	0	0	0.09279325	0.04564267	0.02517109	0.01844724
0	0.04008071	0	0	0	0	0
2.07648195	1.76355138	1.46736556	2.69100433	2.37341909	2.06402945	0.6420955
0.02628458	0	0	0	0	0	0.05408502
0.02628458	0	0	0.09279325	0.04564267	0.02517109	0.01844724
0	0	0	0	0	0.02517109	0
0.02628458	0	0	0	0	0	0.0595436
0	0	0	0.03093108	0	0	0
0.68339912	0.80161427	0.61534685	0.43303518	0.5477121	0.553764	0.66986545
0.21027665	0.04008071	0.23667187	0.43303518	0.13692802	0.22653982	0.17819767
0	0.04008071	0	0	0	0	0
0	0	0.04733437	0	0	0	0
0	0	0	0	0.04564267	0	0
0	0	0	0	0	0.02517109	0
0.42055331	0.48096856	0.5206781	0.24744867	0.41078407	0.276882	0.38944487
0.02628458	0	0	0	0	0	0.06367038
1.65592864	2.96597278	2.93473113	3.80452336	2.96677386	2.81916217	1.5299646
4.10039474	3.68742562	3.73941547	5.07269781	3.28627258	5.28592908	8.73859321
0	0	0	0.03093108	0	0	0
0	0	0.04733437	0	0	0	0
0.02628458	0	0	0	0	0	0.13711532
1.31422908	1.64330924	1.89337492	1.60841638	0.86721082	1.15787018	1.93114364
0	0	0	0	0	0.02517109	0
11.749208	13.3067968	11.8809276	12.2177783	10.7260286	7.80303816	9.72767048
0.02628458	0.04008071	0.04733437	0.03093108	0	0.22653982	0.02953931
0.15770749	0.08016143	0.09466875	0.03093108	0.09128535	0.05034218	0.63987148
0	0	0.04733437	0	0	0	0
0	0	0	0	0.04564267	0	0
0	0	0	0	0.04564267	0	0
0	0	0	0	0.04564267	0.02517109	0
0.02628458	0	0	0	0	0	0.03379248
0.13142291	0.08016143	0.09466875	0.06186217	0.04564267	0.02517109	0.14913517
0.63082996	0.40080713	0.75734997	0.83513927	0.63899745	0.93133036	1.23965462
0.02628458	0	0	0	0.13692802	0.02517109	0.01269135
0	0	0.04733437	0	0	0	0
0	0	0.04733437	0	0	0	0

0.05256916	0	0	0.06186217	0.09128535	0.05034218	0.11039843
0.02628458	0	0	0	0	0	0.04659934
0	0.04008071	0	0	0	0	0
0	0	0.04733437	0	0	0	0
0	0	0	0	0.04564267	0	0
0.02628458	0	0	0.03093108	0	0.07551327	0.03756255
0.02628458	0	0	0	0	0	0.1007948
0.52569163	0.68137213	0.56801248	1.05165686	0.86721082	0.70479054	0.45917232
0.02628458	0	0	0	0	0	0.03796168
0.02628458	0	0	0	0	0	0.06575366
0	0	0	0	0.04564267	0.02517109	0
0.34169956	0.32064571	0.80468434	0.24744867	0.41078407	0.25171091	0.46341025
0.07885374	0.04008071	0	0	0	0.02517109	0.33435225
0.02628458	0	0	0.03093108	0.04564267	0.07551327	0.04788316
0	0	0	0	0	0.02517109	0
0.10513833	0.16032285	0.18933749	0.03093108	0.04564267	0.12585545	0.17691082
0	0	0	0	0.04564267	0	0
0	0	0	0	0.04564267	0	0
0.10513833	0.04008071	0.04733437	0.06186217	0	0	0.64146717
0.02628458	0	0	0	0	0	0.08956418
0.18399207	0	0.04733437	0	0	0.05034218	0.30518616
0	0	0.04733437	0	0.09128535	0.10068436	0
0.02628458	0	0	0	0	0	0.02523826
0.02628458	0	0	0	0	0	0.0044832
0.02628458	0	0	0	0	0	0.09954657
0	0	0	0.03093108	0	0	0
0	0	0	0	0.04564267	0	0
0	0.04008071	0	0	0	0	0
0.5782608	0.24048428	0.5206781	0.24744867	0.27385605	0.30205309	0.3020401
43.1067139	48.3774209	54.7658696	45.2831073	55.8209912	63.0535825	107.946384
283.97862	298.801717	266.49252	326.817836	282.893298	345.649419	319.142733
0.07885374	0	0.23667187	0.06186217	0	0.05034218	0.10868803
0.52569163	0.56112999	0.42600936	0.40210409	0.3651414	0.25171091	0.5281896
7.49110577	6.17242984	8.04684342	6.37180335	5.47712097	6.56965471	7.22693957
843.761355	948.910886	1017.26301	742.562539	993.686672	628.849362	274.073511
0.76225287	0.64129141	0.80468434	0.49489735	0.3651414	0.57893509	0.6249683
66.6314145	70.7023782	65.4161036	64.9552769	57.8749116	56.7859809	35.8203596
23.2355702	21.0824552	22.2471553	20.4763778	17.8919285	19.1803712	20.3317102
39.9788487	41.6839418	34.0807486	32.5395006	33.5017233	35.1640139	12.8970298
0.31541498	0.52104927	0.28400624	0.12372434	0.50206942	0.10068436	0.28277208
0.07885374	0.24048428	0.14200312	0.03093108	0.13692802	0.07551327	0.16746636
173.662231	157.036235	187.775458	124.930649	200.416985	115.057056	110.991876
13.2474292	17.6755945	17.7503899	10.3619132	16.0205788	14.951628	14.4047867
76.8035476	89.7006363	89.7933057	87.8752103	64.9951688	78.3576058	67.4704839
29.9644231	30.9022299	25.0398834	26.1676973	25.2860418	24.6424979	12.3604915

0.05256916	0.04008071	0.04733437	0	0.04564267	0	0.21873158
48.757899	51.583878	44.1629701	48.0359738	29.1656692	44.9303971	23.1990742
0.60454538	0.36072642	0.56801248	0.64955277	0.91285349	0.80547491	0.48272288
0	0	0.04733437	0	0	0	0
0.02628458	0	0	0	0	0	0.02338439
3.94268725	3.44694134	2.4613874	2.25796915	2.28213374	3.27224181	1.48286895
0.15770749	0.04008071	0.28400624	0.40210409	0.1825707	0.30205309	0.11239222
0	0	0	0	0.04564267	0.02517109	0
95.4918851	104.690823	101.011552	96.1028787	92.608987	84.9776027	55.3357731
0	0	0	0	0	0.02517109	0
0.76225287	0.6012107	0.5206781	0.83513927	1.00413884	0.830646	0.6971609
0.02628458	0.08016143	0.04733437	0.03093108	0	0	0.02013371
0.31541498	0.16032285	0.14200312	0.21651759	0.09128535	0.07551327	0.34682472
0	0	0	0.03093108	0	0	0
3.28557271	4.28863632	2.36671865	3.21683276	1.78006431	2.81916217	4.23518726
0.94624494	0.76153355	1.37269682	0.61862168	0.50206942	1.00684363	1.02754328
0.05256916	0.28056499	0.23667187	0.15465542	0.41078407	0.52859291	0.04782974
15.2187728	15.2707518	13.7269682	15.9295084	15.6097948	18.7021205	12.910472
0	0	0.04733437	0	0	0	0
0.02628458	0	0	0	0	0	0.03570632
0.26284582	0.36072642	0.23667187	0.64955277	0.31949872	0.35239527	0.21791624
1.70849781	1.64330924	1.27802807	1.85586505	1.55185094	2.71847781	1.71450386
0	0.04008071	0	0	0	0	0
3.12786522	2.56516565	2.74539364	3.68079902	2.87548851	3.87634799	5.69809599
5.96660003	6.25259127	6.1534685	6.99042504	5.93354771	8.30645998	12.0244711
1.36679825	1.04209854	1.18335933	1.23724337	1.87134966	1.9130029	0.92247402
182.861835	191.185002	204.863167	196.381454	280.246023	197.039299	227.792454
0	0.04008071	0	0	0	0	0
0	0	0	0.03093108	0	0.02517109	0
3.46956478	2.7254885	3.31340611	3.27869493	2.09956304	1.58577872	1.7341236
36.7984143	28.4973871	26.6965864	27.0028365	29.0743838	23.8118519	17.5087353
38.5069121	43.2070089	37.3941547	48.4071468	47.9248085	36.5484239	29.7473562
0	0	0	0	0	0.02517109	0
0	0	0	0	0	0.02517109	0
0.07885374	0.04008071	0.14200312	0.09279325	0.31949872	0.12585545	0.07077082
0	0	0	0	0	0.02517109	0
149.638123	157.076315	170.498412	143.303713	138.845017	136.477655	53.6410587
58.4043404	60.9627649	66.8361348	63.3468605	79.418254	70.3531989	149.154843
35.9047385	34.7098977	28.1166176	35.261436	42.9041142	32.9489579	67.3130083
13.5891287	13.0663125	11.9755964	15.3727489	16.1118642	14.146153	32.0605228
9.75157979	6.73355983	6.81614972	7.23787371	9.40239099	11.5031885	17.4145459
4.70494011	4.64936274	5.25411541	5.32014649	5.93354771	6.24243053	6.14573835
15.770749	17.5553524	15.1469994	15.9913705	23.1408361	17.2170261	15.0484594
0	0.16032285	0	0	0.04564267	0	0
42.5284531	39.8001483	42.3169295	41.6023083	37.3813506	29.1984654	14.6012996

15.7444644	17.3950296	13.7269682	14.7231961	12.5060929	13.8189289	13.6450382
15.455334	15.3108325	17.5610524	13.1147797	14.0123011	14.0958109	12.8965092
0.5782608	0.40080713	0.47334373	0.55675952	0.59335477	0.90615927	0.50890498
0	0	0.04733437	0	0	0	0
0.7096837	0.48096856	0.5206781	0.43303518	0.41078407	0.35239527	1.040942
0	0	0.04733437	0	0	0	0
0	0.04008071	0	0.06186217	0	0.05034218	0
0	0	0.04733437	0	0	0	0
10.3561252	11.5432454	13.064287	10.7330862	13.8753731	15.4047076	8.51377381
0.26284582	0.32064571	0.18933749	0.15465542	0	0.22653982	0.45414607
0	0	0	0	0	0.02517109	0
16.086164	33.0265077	29.5839832	16.1150949	17.1616457	27.2854625	15.0500708
125.561447	137.476846	136.701669	146.551477	140.488153	153.417799	134.204866
0	0	0	0	0	0.02517109	0
1.05138327	1.32266354	0.7100156	1.29910554	1.36928024	1.71163418	2.42211237
45.7351721	39.1989376	33.8440767	65.9760027	29.7133812	55.5022553	104.67196
39.9262795	35.7519962	31.2880206	56.7894706	24.5101163	46.7175446	58.5275372
0	0	0.04733437	0	0	0	0
4.38952514	2.7254885	4.44943107	5.01083564	4.1534834	5.46212671	7.53633889
0.02628458	0.20040357	0.18933749	0.06186217	0	0.10068436	0.04365722
0.44683789	0.96193712	0.5206781	0.34024193	0.41078407	0.45307964	0.21626517
0	0	0	0	0.04564267	0.02517109	0
5.88774629	4.28863632	3.6920811	3.77359228	3.9709127	3.97703235	2.43653361
0	0	0.04733437	0	0	0	0
0.07885374	0.08016143	0.04733437	0.03093108	0	0.05034218	0.25791611
83.2958392	86.8949863	95.0474211	82.5241327	80.7875343	78.8610276	41.6491206
0	0	0.04733437	0.15465542	0	0	0
0.05256916	0	0.04733437	0.15465542	0	0.07551327	0.29098228
10.0144256	6.81372125	8.75685902	8.90815226	9.72188972	11.1759643	12.4307452
93.6256798	85.4921614	67.9721597	93.2262879	94.297766	86.5633814	51.7536898
29.5701544	35.8321577	35.0747704	31.6424992	47.5596671	37.9831761	6.65796694
2.18162028	1.48298639	1.70403743	2.38169349	1.64313629	2.91984654	2.22488033
1.60335948	2.08419709	1.79870618	1.85586505	2.32777641	2.66813563	5.46341516
54.6982144	57.7563078	57.1799227	49.8609078	49.5679448	50.3421817	36.483926
0.23656123	0.16032285	0.14200312	0.34024193	0.27385605	0.276882	0.13582815
139.965397	162.807857	120.844654	118.496984	131.679117	114.22641	71.6391671
1.05138327	0.64129141	0.99402183	0.52582843	0.7302828	0.78030382	1.67903612
67.0519678	72.7865753	69.7235315	67.4606947	61.6176109	61.1657507	40.5756541
15.6130415	14.6695411	11.170912	14.1973677	13.7840878	21.0430319	7.23591732
480.455868	395.636721	332.334633	523.663256	410.008147	460.630962	628.257176
20.9488116	23.8480244	23.9511928	25.7346621	22.9582654	29.9535981	35.3223607
0	0	0	0	0.04564267	0	0
0	0	0	0	0.04564267	0	0
66.7628374	71.8246382	76.4923469	64.1201376	64.3561714	65.6210338	87.581765
14.4302353	11.463084	14.2476463	17.630718	13.327661	15.8577872	16.0111514

0.81482203	1.04209854	0.7100156	0.5876906	0.50206942	0.78030382	0.5479713
30.0695614	30.1807771	23.9038584	31.8280857	33.9125073	28.3426483	25.2982474
0.36798414	0.24048428	0.18933749	0.18558651	0.09128535	0.15102655	0.32905671
36.9035526	28.4172257	30.5306706	39.9320297	30.0785226	39.2165595	26.0043621
51.9383333	52.5458151	67.9721597	51.6858417	45.6883174	55.6784529	67.8434579
0.02628458	0	0	0	0	0	0.1270891
0	0	0	0	0.04564267	0	0
107.556508	98.2378282	111.472449	121.064264	114.3349	121.702224	456.848238
37.8497976	64.2894641	50.5057761	43.1179314	55.0450657	18.7776338	20.6332888
0.07885374	0	0.04733437	0.06186217	0.09128535	0.15102655	0.2589547
26.38972	31.9844092	34.4594236	32.1064654	38.1572761	33.1754977	89.054223
0.07885374	0.04008071	0.14200312	0.18558651	0.13692802	0.15102655	0.24860986
0	0	0	0.03093108	0	0	0
0.21027665	0	0.28400624	0.06186217	0.09128535	0.07551327	0.96160125
0	0	0	0	0	0.02517109	0
0.02628458	0	0	0	0	0	0.1199759
0.02628458	0	0	0	0	0	0.14920437
0.02628458	0	0	0	0	0	0.13314096
0.10513833	0.16032285	0.04733437	0	0.09128535	0.07551327	0.59681748
0.02628458	0.08016143	0.04733437	0	0	0.02517109	0.13397309
0.36798414	0.20040357	0.09466875	0.15465542	0.13692802	0.07551327	1.78630787
0	0.12024214	0.14200312	0.03093108	0	0.02517109	0
0	0	0	0	0	0.02517109	0
0.10513833	0.12024214	0.04733437	0.06186217	0.04564267	0.02517109	0.69899004
0.65711454	0.36072642	0.66268122	0.40210409	0.50206942	0.276882	3.98927316
6.33458418	8.57727264	7.47883094	7.11414937	6.11611841	5.99071962	30.507888
0.02628458	0	0	0	0	0	0.14648538
0	0	0.04733437	0	0	0	0
40.3731174	42.6458789	44.2576388	35.6635401	41.3522633	38.0586893	19.0319229
7.35968286	5.49105772	8.47285278	6.58832094	10.3608872	10.1942918	8.38657818
25.8377438	27.0144007	30.7200081	24.7757985	23.7798335	24.4914714	35.9782942
1.97134362	0.68137213	1.98804367	0.5876906	1.55185094	0.93133036	3.19406043
0	0	0	0.03093108	0	0	0
0	0.16032285	0.09466875	0.09279325	0.1825707	0.17619764	0
6.7288529	7.97606194	8.85152776	9.06280768	7.34847063	12.0317814	18.0709257
10.5664018	10.5412276	8.56752152	7.76370214	7.53104133	9.81672543	6.37992288
0	0	0	0	0	0.02517109	0
124.956901	132.346515	144.60651	124.837856	117.073461	116.089071	107.495033
4.07411016	3.76758705	4.54409981	3.49521252	5.84226237	6.46897034	3.8087879
0	0	0	0.03093108	0	0.02517109	0
0	0	0.04733437	0	0	0	0
1.23537534	0.48096856	0.75734997	1.14445012	1.41492292	1.15787018	1.8553916
0.02628458	0	0	0	0	0	0.02382626
0.02628458	0	0	0	0	0	0.02589895
0.34169956	0.40080713	0.5206781	0.46396626	0.59335477	0.65444836	0.29931689

0.07885374	0	0.18933749	0.12372434	0.04564267	0.35239527	0.07349381
0.97252952	0.96193712	1.18335933	1.29910554	1.32363757	1.43475218	0.44341448
17.6632389	17.755756	15.857015	16.4244057	14.5143706	13.5420469	14.6093171
37.7183747	34.9103012	39.1455265	34.9211941	33.7299366	30.5577043	24.8133005
1.94505904	1.76355138	1.18335933	1.39189879	1.18670954	0.98167254	0.97895992
10.4086943	9.41896762	9.98755272	10.26912	12.4148075	11.2766487	31.6736381
32.98715	34.9103012	37.9621672	34.7046765	39.1614149	47.120282	47.5017008
0	0	0	0	0	0.02517109	0
20.922527	19.8800338	20.1644429	23.6313483	22.8213374	22.1253888	16.1528679
26.4422891	27.3350464	23.7145209	27.157492	44.3646798	21.1940585	11.2843059
7.62252868	8.17646551	5.49078728	9.46491177	7.9418254	8.2309467	6.95600678
602.889449	551.871341	451.001907	658.15161	549.902945	623.638947	104.042574
0	0	0	0	0.04564267	0	0
0	0.04008071	0	0	0	0	0
0.91996036	1.40282496	1.79870618	1.94865831	1.68877897	1.61094981	0.7302881
21.9476257	21.9642309	23.0991741	19.7031007	21.9084839	17.468737	11.7215487
0	0	0	0	0.04564267	0	0
0	0	0	0.03093108	0	0	0
1.47193657	1.28258282	1.42003119	1.67027855	1.78006431	2.03885836	3.88478605
11.3286547	9.57929047	10.6028996	12.4652269	11.3193833	12.3590056	29.9312667
9.8830027	10.7817119	13.8216369	12.6198824	12.0496661	14.7250881	11.6527534
0.05256916	0.04008071	0.09466875	0.03093108	0	0.02517109	0.04313017
9.51501856	7.97606194	10.0348871	10.3309821	9.95010309	13.1141383	15.2951144
4.12667932	3.44694134	3.26607174	5.01083564	4.10784073	4.07771672	12.0193001
55.5918902	54.0288015	47.3817074	59.0474398	45.6883174	75.3119038	209.891175
0.07885374	0.12024214	0.14200312	0.24744867	0.13692802	0.22653982	0.3566012
0	0	0	0	0.04564267	0	0
250.99147	267.137954	272.835326	240.365456	246.607372	207.837697	296.651372
0	0	0	0.03093108	0	0	0
49.4150135	46.2130624	52.5411541	40.7053068	46.6468136	47.120282	66.5734123
4.62608637	4.00807133	3.55007798	4.63966263	5.0663369	4.75733617	12.4237614
7.22825995	8.37686907	7.43149657	8.815359	8.71775087	9.76638324	6.73436707
26.9154116	28.2569028	37.4414891	22.6415537	23.7798335	31.7407455	8.4240058
2.68102733	2.4048428	1.60936868	1.20631228	1.59749362	2.11437163	3.10721104
0	0	0.04733437	0	0	0	0
0	0.04008071	0	0	0	0	0
6.54486083	6.29267198	5.34878416	5.90783709	4.1534834	4.48045417	1.58790795
0.15770749	0.08016143	0.14200312	0.12372434	0.1825707	0.40273745	0.19292125
0.89367578	0.68137213	0.42600936	0.5876906	0.3651414	0.50342182	0.51457774
41.9239077	41.1628925	42.127592	42.1281367	35.646929	34.6857632	16.6509412
3.8638335	3.00605349	2.93473113	3.24776384	2.09956304	2.71847781	1.83093962
11.2498009	9.41896762	11.5969214	8.28953057	9.53931902	9.59018561	2.9214979
0.47312247	0.52104927	0.5206781	0.52582843	0.27385605	0.22653982	0.28246157
0	0	0.04733437	0.09279325	0	0.05034218	0
0.02628458	0	0.04733437	0.06186217	0	0.15102655	0.02239105

104.612635	113.869306	119.329954	117.878362	87.8165062	91.7486261	69.4551402
4.04782557	4.60928202	4.07075608	5.62945733	4.65555282	5.51246889	2.4945317
0.07885374	0.12024214	0.09466875	0.15465542	0.1825707	0.15102655	0.09360565
6.4397225	4.32871703	4.07075608	5.22735323	6.34433179	6.3179438	6.65058481
11.3286547	14.1084111	11.0289089	10.5784308	9.49367634	11.4276752	19.7691533
0.15770749	0	0.09466875	0.06186217	0	0.02517109	0.28708519
0	0	0	0.03093108	0.04564267	0	0
0	0	0	0	0	0.02517109	0
0	0	0.04733437	0	0	0	0
0.63082996	0.36072642	0.61534685	0.46396626	0.1825707	0.42790854	0.59200997
0	0	0.04733437	0	0	0	0
0	0	0	0	0.04564267	0	0
2.52331984	2.52508494	2.36671865	3.15497059	2.87548851	3.39809726	4.42860832
0.02628458	0	0	0	0	0	0.07628361
295.517551	331.066692	316.619621	299.289171	301.332939	240.157378	132.612718
0.42055331	0.84169498	0.47334373	1.20631228	0.50206942	0.67961945	0.32053375
0	0.04008071	0.09466875	0	0	0.02517109	0
0	0	0	0.03093108	0	0	0
33.5391262	33.3070727	28.9686363	29.4463922	29.5308106	27.0589226	13.0330112
0.89367578	0.72145284	1.42003119	1.1753812	1.32363757	1.61094981	1.2133412
10.3824098	10.340824	8.28351529	12.8364	8.76339355	14.2971796	5.14404575
50.7555272	48.8583895	43.7369607	48.7473887	59.4724052	53.6647657	47.0189239
0.55197621	0.24048428	0.28400624	0.24744867	0.22821337	0.276882	0.58183919
0	0	0.04733437	0	0	0	0
23.2355702	23.2468137	19.7857679	20.8475508	20.8587024	16.2353536	11.374042
16.5592864	20.5213252	16.2830243	16.3934746	15.9749362	14.2468374	12.4580143
11.3023701	12.2246175	12.5436089	11.8775363	14.0123011	15.6312474	15.0119684
45.3146188	49.4596002	47.1450356	45.0665897	46.6011709	37.3035566	25.891035
278.958265	292.22848	292.57376	262.852354	284.262578	217.931304	148.593746
54.5667915	55.0308193	54.3398603	52.1188769	50.800297	45.433819	24.9116449
11.433793	11.1424383	11.7389245	9.77422262	10.6803859	10.0180942	8.3653054
0	0	0	0.03093108	0	0	0
14.0359666	15.7116396	12.0229308	12.4033648	13.1450903	10.798398	16.0317379
0.02628458	0	0	0	0	0	0.11821155
0	0	0	0.03093108	0	0.02517109	0
6.12430752	6.21251056	6.24813724	5.32014649	5.20326492	5.58798217	7.91524058
8.83161943	10.060259	12.2122683	9.00094551	14.6056559	15.1529967	6.75857992
75.5418877	68.5380197	65.8421129	78.7196094	80.8788196	78.0555527	40.7987986
0.02628458	0	0	0	0	0	0.03512129
47.7590849	35.8321577	33.7020736	46.1801088	39.1157722	46.1637806	52.4678808
58.9826012	43.327251	40.470889	73.4922561	39.7547697	55.9301638	41.0657641
5.28320091	5.69146128	5.68012477	4.36128288	5.15762224	4.37976981	3.22176564
0	0	0.04733437	0	0	0	0
9.54130314	14.1484918	9.04086526	13.1766419	12.3691649	12.4848611	9.71430348
0.05256916	0.08016143	0	0.03093108	0	0	0.05091614

0.02628458	0.04008071	0.14200312	0	0.04564267	0.02517109	0.03474181
29.8855693	29.9002121	27.5486051	32.1373965	15.9749362	24.1642472	31.0476237
0.02628458	0	0	0	0	0	0.03588565
13.4577058	13.3067968	8.37818403	13.207573	17.5267871	11.7800705	17.9136163
98.0677741	105.732922	105.602986	97.7731572	96.9906838	86.1606439	71.5140769
2.97015773	4.56920131	3.55007798	3.95917878	2.05392036	2.49193799	1.47188585
3.52213394	3.60726419	2.31938428	2.01052047	3.51448595	2.24022708	2.50717171
99.0140191	92.7868512	112.797811	96.5359139	97.4927532	72.6941103	34.1622878
3.12786522	2.52508494	1.84604055	2.1651759	2.09956304	1.48509436	1.46460152
6.88656039	6.45299483	6.05879975	5.07269781	3.9709127	6.97239216	3.59855956
29.7541464	28.3370643	32.5660487	24.590212	23.5059775	30.6080465	38.8450472
3.96897183	3.80766776	4.73343731	3.61893685	3.788342	2.94501763	1.68028545
95.6758772	99.1596846	92.11269	110.826075	107.534142	102.144287	71.321689
0	0	0	0.03093108	0	0	0
0.02628458	0	0	0	0	0	0.05645925
14.009682	12.1444561	10.7922371	11.7847431	6.25304644	12.8372563	21.0022521
8.3059278	7.49509338	7.47883094	10.1453956	8.90032157	8.88539506	7.1152095
60.8488065	51.5037165	49.9377636	60.6867873	58.240053	60.7378422	12.5916348
0	0	0.04733437	0	0	0	0
2985.11365	2881.92353	2715.62032	2386.27129	2612.54106	2454.48341	1449.92876
2.97015773	2.92589207	2.4613874	2.44355565	4.06219805	4.25391435	7.74701537
65.4223237	67.4558404	73.462947	62.7591699	63.3976752	61.0147242	26.8918566
33.6442645	29.3390821	27.4539364	35.7563334	30.169808	34.2830257	14.9850841
38.9800346	31.7439249	27.3592676	45.3759006	34.9622888	36.2463708	23.5242735
120.330815	133.18821	132.441576	117.878362	128.027703	97.5883192	33.5305043
32.7243042	33.4273149	35.8321204	36.6842659	26.8378927	26.4799876	23.5893691
0.21027665	0.32064571	0.23667187	0.12372434	0.13692802	0.20136873	0.11460385
0	0	0	0.03093108	0.04564267	0	0
61.0327986	62.0048634	60.3513257	55.3666408	60.613472	51.3490253	31.369968
16.9272706	15.6314782	16.7090337	13.5478149	14.3774425	14.4733772	11.2141245
27.2308266	26.8139972	24.4718709	22.9508645	23.5516202	24.5669847	21.8576569
0	0.04008071	0	0.06186217	0	0.05034218	0
20.9488116	24.6496387	26.7912552	19.8886872	23.4146921	18.2742119	25.0992877
8.91047318	8.65743406	8.04684342	5.50573299	6.66383051	9.13710597	2.43033459
0	0	0.04733437	0	0	0	0
4.12667932	2.92589207	3.31340611	2.56727999	2.60163246	3.09604417	1.72909952
0	0.08016143	0.09466875	0.03093108	0	0	0
1.73478239	1.72347067	1.56203431	1.36096771	1.46056559	1.25855454	0.68500121
0.86739119	0.20040357	0.18933749	0.5876906	0.5477121	0.98167254	0.70432584
58.8248937	54.790335	49.7957605	55.6759516	49.8418008	37.8069784	31.6584366
2.62845816	2.7254885	2.79272801	4.1138342	4.79248085	2.16471381	2.03825938
0.39426872	0.40080713	0.47334373	0.55675952	0.77592547	0.50342182	0.13504217
0	0	0	0.03093108	0	0	0
0.05256916	0.08016143	0	0.03093108	0	0	0.04508032
0.73596829	0.6012107	0.37867498	0.27837976	0.59335477	0.32722418	0.83670926

0	0	0	0	0	0.02517109	0
0	0	0	0.03093108	0	0	0
0.02628458	0	0	0	0	0	0.0329442
0.5782608	0.64129141	0.75734997	0.52582843	0.3651414	0.50342182	0.45461306
4.17924848	3.44694134	4.82810605	3.43335035	2.37341909	4.20357217	2.47994816
5.65118505	6.29267198	7.19482471	7.51625347	6.80075853	8.38197325	3.38127242
0.52569163	0.20040357	0.7100156	0.64955277	0.86721082	0.57893509	0.16413242
0	0	0	0	0	0.02517109	0
0	0.04008071	0	0	0	0	0
0	0	0	0	0	0.02517109	0
0	0	0.04733437	0	0	0	0
0.10513833	0	0.18933749	0.06186217	0.27385605	0.25171091	0.14040848
13.1422908	15.5913975	14.3896494	12.0012607	13.053805	11.8807549	8.96390903
72.4665916	62.7263163	60.3513257	79.2454378	61.2068268	76.746656	57.2287375
12.1697613	11.2626804	10.7449027	12.0940539	12.9625196	15.1529967	15.6212905
0	0	0.04733437	0	0	0	0
5.41462382	6.3327527	4.97010917	5.6913195	3.83398468	4.45528308	3.10531157
26.705135	26.7739165	27.4539364	25.4562823	22.1823399	24.8438667	28.1135958
105.006904	107.536554	64.7534224	112.68194	59.5636905	55.1750311	23.3233589
62.5573043	61.2834106	31.4300237	57.9029897	37.6552067	33.4523797	29.0694888
4.94150135	4.32871703	4.63876856	4.26848962	4.24476875	3.0205309	4.94669872
17.0061243	16.352931	17.0403743	15.5274043	19.3981368	22.9308638	7.45372321
0	0	0.04733437	0	0	0	0
71.3363546	75.8327095	75.5929938	70.9249761	68.3270841	64.1611105	36.4971611
25.9954512	20.6415673	22.7678334	24.8067295	25.7424685	26.8072117	16.4085927
0	0	0	0.03093108	0	0	0
90.2612534	79.0792473	104.419627	70.8012518	96.5798997	63.0787536	40.5565701
26.2845816	20.7217288	16.9457056	25.6727999	23.4603348	25.221433	9.29561772
0.31541498	0.48096856	0.33134061	0.18558651	0.27385605	0.22653982	0.17294599
13.9571129	14.5492989	13.3009588	14.5994718	9.81317507	10.6222003	4.98570029
0.42055331	0.52104927	0.7100156	0.27837976	0.50206942	0.37756636	0.32539954
5.88774629	4.60928202	8.42551841	7.11414937	6.89204388	10.4460027	2.78031011
44.4472276	42.2450718	57.9372726	48.097836	59.0159784	63.3052934	35.7238101
13.7205516	11.2225997	8.52018715	13.1766419	10.9085993	14.9264569	11.3675237
0.07885374	0.08016143	0.18933749	0.03093108	0.04564267	0.07551327	0.12406511
9.83043354	11.2626804	12.4962745	12.9601243	11.6388821	14.2468374	10.0003531
0	0	0	0	0	0.05034218	0
0	0	0.09466875	0.12372434	0.13692802	0	0
0.02628458	0.04008071	0.04733437	0.12372434	0.04564267	0.10068436	0.06225274
0.36798414	0.04008071	0.14200312	0.15465542	0.5477121	0.42790854	0.70889698
0	0.04008071	0	0	0	0	0
0	0	0	0	0	0.02517109	0
0.05256916	0.04008071	0.04733437	0.09279325	0.09128535	0.10068436	0.11311712
0	0.04008071	0	0.03093108	0	0.07551327	0
0	0	0	0	0	0.02517109	0

0.42055331	0.20040357	0.80468434	0.34024193	0.22821337	0.22653982	0.45088227
0	0.04008071	0	0.09279325	0	0.05034218	0
153.738518	171.264888	146.026541	127.652585	150.985968	127.36572	22.8804751
0	0.04008071	0	0	0	0	0
0.76225287	0.28056499	0.14200312	0.37117301	0.31949872	1.45992327	0.26790769
166.144841	173.108601	192.792901	181.44174	159.156007	116.944888	101.065409
134.419351	134.631116	134.240282	133.49856	119.127381	104.460027	57.5847355
74.8059194	76.874808	77.4863687	71.9147708	63.99103	70.8314496	39.1901412
44.1843817	47.2150802	49.9377636	41.7260326	45.1406053	38.788651	21.6633682
0	0	0	0	0	0.02517109	0
0.47312247	0.9218564	0.66268122	0.95886361	0.95849617	0.93133036	0.82680536
0	0	0	0	0	0.02517109	0
0.07885374	0	0	0.09279325	0.1825707	0.05034218	0.05617975
0	0	0	0	0	0.02517109	0
6.07173836	6.81372125	5.77479351	5.62945733	5.20326492	5.31110017	2.80917857
15.1662036	14.7497025	7.85750593	14.9706448	18.4396406	12.5352032	8.91094791
15.0347807	14.9100253	18.9810836	18.1256154	11.4106687	11.6542151	7.12309289
42.4495994	45.3713674	53.9611853	42.6230341	34.232006	46.6420313	82.2295642
95.623308	109.500509	116.063883	92.6385973	97.7209666	99.4006377	137.213589
49.5464364	42.5256368	38.5301797	56.5110909	40.9871219	53.1361728	35.3098899
2.07648195	1.8036321	1.13602495	1.7630718	1.96263501	2.76881999	3.03843277
4.81007844	4.40887846	4.40209669	6.18621685	4.47298212	6.26760162	6.58913564
418.792239	409.905455	402.389505	364.646552	394.124496	324.883269	333.965045
39.6108645	32.144732	34.1754174	37.9833714	40.1655538	42.8411966	27.2834388
0.9988141	1.12225997	0.75734997	1.14445012	1.46056559	1.18304127	0.77087355
0	0.04008071	0	0	0	0	0
3.75869518	2.52508494	5.34878416	4.29942071	5.43147829	5.08456035	10.7806717
0	0.04008071	0	0.06186217	0	0.07551327	0
39.4794416	37.1548212	35.8794548	31.3331883	38.7049882	31.312837	43.4890766
0	0	0	0.03093108	0	0	0
3.60098769	4.56920131	4.68610293	3.18590168	3.19498723	3.80083472	2.06035323
28.6239094	32.4653777	31.7613643	26.2295594	26.1076099	30.1549668	31.645013
12.380038	11.5031647	9.98755272	12.9291932	12.0040235	13.5420469	10.8991134
47.3385315	29.21884	26.1285739	21.8064144	72.4805675	47.2964797	80.4844039
40.7411016	24.2087508	22.9571709	19.084479	63.8997446	40.6764828	110.628168
0.21027665	0.12024214	0.18933749	0.49489735	0.1825707	0.25171091	0.10920328
0	0.08016143	0.09466875	0	0.04564267	0	0
0.07885374	0.12024214	0.04733437	0.06186217	0.04564267	0	0.07556649
0	0.04008071	0	0	0	0	0
0.81482203	0.9218564	0.47334373	0.43303518	0.31949872	0.67961945	0.41836717
0	0.04008071	0	0	0	0	0
139.728836	132.827484	140.06241	120.816815	121.135659	129.631118	33.08395
0	0	0	0	0	0.02517109	0
6.0191692	5.85178414	5.39611853	7.11414937	6.66383051	6.14174616	4.36077053
7.78023617	7.2546091	9.79821522	7.42346021	5.88790504	7.29961634	5.53500921

0.86739119	1.04209854	1.32536245	0.9897947	1.32363757	1.76197636	1.22172352
125.614016	120.442543	119.377289	119.455847	111.322484	101.464667	71.222144
35.7996002	35.1507855	28.9686363	38.7257175	36.1946411	35.5164092	30.1397961
30.595253	36.7139333	39.5715359	29.9412895	28.7548851	32.9993001	39.39655
0.02628458	0.04008071	0	0.03093108	0.04564267	0.10068436	0.04416695
3.49584936	3.3266992	4.63876856	3.37148818	3.01241653	3.04570199	4.81578946
2.10276653	2.32468137	1.70403743	1.73214072	1.50620827	1.43475218	4.38208405
0	0	0	0	0	0.05034218	0
0.02628458	0	0	0	0	0	0.03502564
0	0	0	0.03093108	0	0	0
0	0	0	0	0.04564267	0	0
0.02628458	0	0	0	0	0	0.06273862
0	0	0.04733437	0	0.04564267	0.07551327	0
15.3501957	12.585344	15.7623462	16.4862679	11.228098	24.8942088	7.250065
16.1650177	17.9160788	16.9457056	16.7337166	10.1783165	16.8394598	10.8710435
26.6788504	26.5735129	20.6377867	28.4256664	26.0619673	29.7018872	26.4472
102.457299	100.60259	98.455496	111.011661	123.919862	103.553868	41.4192021
70.4426788	85.6925649	73.5102814	72.8117723	71.567714	51.3993675	14.9772298
32.2248971	35.3111084	31.6193612	35.5707469	37.746492	35.8436333	16.7496249
3.20671896	3.24653777	2.4613874	3.18590168	3.65141398	3.8511769	2.1696527
0.02628458	0	0.09466875	0.15465542	0.09128535	0.02517109	0.02102913
0.02628458	0	0	0	0	0	0.02312373
67.603944	55.351465	59.8306476	72.2240817	62.028395	67.8109187	54.9129543
0.91996036	1.2024214	1.60936868	1.42282987	1.27799489	2.06402945	0.29318066
327.873871	324.77402	329.021227	305.722836	314.15853	253.749767	173.591551
27.6776645	30.6617456	31.71403	27.3430785	27.4312475	26.0269079	21.572271
203.337524	247.298001	164.96029	245.963982	205.62025	244.763687	348.375068
0	0	0	0	0.04564267	0	0
0.86739119	0.56112999	0.47334373	0.92793253	0.91285349	0.830646	0.494094
1.39308283	0.80161427	0.89935309	0.71141494	1.96263501	1.88783181	1.36440169
0	0	0.04733437	0	0	0	0
0.15770749	0.16032285	0.42600936	0.40210409	0.3651414	0.37756636	0.14560094
0	0	0	0	0	0.02517109	0
30.7529605	31.7038442	33.5127361	33.5911575	36.7423532	39.065533	9.14207625
0	0	0	0.03093108	0	0.02517109	0
878.483288	870.753496	947.066136	747.078477	949.230706	660.992845	293.696548
39.7948566	55.7121914	60.6353319	45.5924182	24.1449749	52.7586064	154.296235
0.23656123	0.12024214	0.23667187	0.21651759	0.09128535	0.05034218	0.41577856
22.1841869	19.8800338	21.442471	23.2601753	23.5972628	24.8942088	25.9441579
3.78497976	3.12629563	3.55007798	4.60873155	3.14934456	4.88319162	8.48005495
0.02628458	0	0	0.09279325	0.04564267	0.02517109	0.06021262
0.23656123	0.08016143	0.33134061	0.03093108	0.22821337	0.12585545	0.65918423
3.39071103	2.48500422	2.55605615	3.64986794	3.24062991	3.87634799	4.60611535
0.31541498	0.44088785	0.14200312	0.21651759	0.22821337	0.15102655	0.37753669
6.49229167	7.17444767	7.28949345	5.35107757	6.48125981	6.34311489	10.5191057

49.2310214	51.6239587	52.020476	44.6954167	49.3853741	42.4384591	23.8367637
36.9561218	33.0265077	40.6602265	27.5905271	34.3689341	34.358539	26.4296285
0.02628458	0.12024214	0	0	0	0.02517109	0.02325753
0	0	0	0.03093108	0	0.02517109	0
76.1990022	65.7323697	64.9427598	76.7709511	75.5386267	77.2752489	106.590186
0	0	0	0	0	0.02517109	0
27.9667949	25.5314143	18.9337492	29.7866341	26.0619673	27.9650819	30.9045785
28.8341861	30.0605349	27.5486051	30.4052558	34.1863634	31.2373237	7.15973515
89.9984076	78.5982787	96.1361117	78.4721607	79.3726114	74.9846796	48.6817275
0.02628458	0	0	0	0	0	0.01803845
121.855321	133.508856	119.28262	132.261316	99.8205296	102.798735	45.4878911
0	0	0	0	0.04564267	0	0
25.0229217	27.3350464	25.9865708	31.3022572	31.2652322	26.605843	5.1654019
8.3059278	7.73557766	9.32487149	10.1144645	9.17417762	9.79155433	3.68134753
58.7986091	47.1749995	48.0443887	63.2540672	54.6799243	60.3351047	60.6215531
5.94031545	6.85380197	9.56154336	7.4543913	8.8546789	10.0432652	7.04137638
10.2247023	9.13840262	7.95217467	9.24839418	10.2239591	12.3590056	4.5252271
3.12786522	3.64734491	2.55605615	1.91772722	2.82984583	2.44159581	1.14063838
2.02391279	2.56516565	3.6920811	1.36096771	2.55598978	2.29056927	1.13883289
0.05256916	0	0	0.12372434	0.09128535	0	0.03267636
195.031596	206.135108	202.638451	189.576615	176.819722	182.490409	79.8725408
6.38715334	8.37686907	6.39014036	9.49584286	7.12025726	8.13026234	2.55545728
413.325046	416.639014	365.184688	397.40257	342.730845	328.58342	298.649996
33.9333949	32.3852163	32.7553862	36.0656442	33.8668646	38.7131377	43.9261612
26.38972	28.938275	29.2053082	28.3638042	24.7383297	23.0315481	14.3699781
40.2942637	43.9284617	45.1096575	39.8392365	39.846055	35.1136717	22.5951774
78.3806225	84.6103857	85.6278809	79.4000932	72.2979968	71.5110691	40.3373417
0	0	0	0	0.04564267	0	0
35.3001932	30.1807771	36.9681454	35.7872645	37.2444226	43.1432497	45.8653282
0.05256916	0.04008071	0.09466875	0	0.04564267	0.02517109	0.06479303
18.3203534	15.0302675	15.8096806	15.4036799	15.5185094	12.5603743	9.81862806
42.7124452	37.2750633	36.5894704	51.7477039	42.0825461	40.9533648	33.9613294
0.42055331	0.12024214	0.18933749	0.12372434	0.1825707	0.12585545	0.20904375
0	0.04008071	0	0	0	0	0
0	0	0	0	0	0.02517109	0
69.2598726	74.3497231	63.9960724	76.2141915	78.2771872	75.5132725	84.5132991
42.0290461	44.168946	52.5411541	37.3956808	42.9497569	42.664999	38.4831685
9.8830027	10.9821154	9.32487149	10.1763267	11.9127381	12.8624274	16.0341269
0.94624494	0.52104927	0.75734997	0.89700144	1.46056559	0.90615927	1.76991973
0.02628458	0	0.18933749	0.03093108	0.04564267	0	0.03496851
36.9824064	34.950382	31.335355	34.1788481	40.7589085	39.2669017	10.7369249
0	0	0	0.03093108	0	0	0
8.20078947	8.97807977	7.3841622	6.89763178	6.07047574	7.4254718	5.95188668
3.73241059	3.20645706	3.07673425	3.9282477	2.82984583	5.66349544	2.66849966
8.51620445	10.7817119	8.70952464	8.84629009	5.52276364	8.53299979	6.00100663

29.6490081	28.2969836	26.0339052	25.0541782	24.555759	25.4479728	36.2329621
13.4577058	10.8618733	10.0348871	13.207573	13.2820183	14.0706398	17.1843563
28.8341861	32.7459427	24.992549	28.0854245	14.1948718	26.9834094	9.32393212
82.9804243	84.28974	80.9891123	76.368847	83.9368788	63.0284114	26.7515433
3.04901147	2.28460066	3.31340611	2.01052047	2.05392036	2.84433326	4.4481942
24.8126451	24.6095579	27.9272801	22.2703806	23.1864788	24.5921557	77.8280599
10.5401172	11.0622769	8.70952464	12.4652269	12.6430209	11.3018198	8.48540323
9.90928728	8.53719192	8.47285278	7.82556431	7.43975598	8.38197325	9.81529167
0.21027665	0.12024214	0.04733437	0.15465542	0.22821337	0.30205309	0.38335072
53.5154082	52.3454115	53.8665165	53.7272933	43.223613	45.0059104	29.1471999
0	0	0	0.03093108	0	0	0
49.0995985	46.7741924	51.8784729	37.6740606	45.8708881	49.8387599	95.413528
13.2737137	10.7416312	13.1116213	12.6817445	9.67624704	9.59018561	13.1584591
8.17450489	7.8558198	5.96413101	6.61925202	6.43561714	5.94037744	7.16828062
27.4148187	34.9103012	33.2287299	28.642184	30.7175201	26.4044743	25.5513478
21.553357	23.5674594	25.2765552	22.022932	20.1284196	18.3748963	19.4009597
12.380038	12.7055861	13.7743026	10.9496038	11.365026	11.9310971	11.5649623
0	0	0.09466875	0.06186217	0.1825707	0.02517109	0
0.07885374	0.04008071	0	0	0.04564267	0	0.04668956
6.49229167	7.93598123	5.11211229	8.56791033	7.25718528	7.04790543	4.62411924
29.149601	24.8500422	29.0159707	25.7965242	26.3358233	22.1002178	23.3748133
238.558863	180.323129	210.069948	219.332318	212.603579	289.845111	126.113027
19.0563217	18.3970474	18.2237336	21.7136211	22.0454119	19.8851618	12.2917573
131.817177	140.001931	142.192457	131.642694	125.060929	110.979339	43.7584835
147.824487	160.082369	146.215878	155.057525	134.006893	105.894779	54.027941
15.8496027	14.5092182	15.1469994	16.5790611	17.4355017	17.2673683	16.3203583
147.798203	126.214166	128.70216	164.584299	190.421239	115.912873	175.618616
103.193268	116.514633	98.3134929	136.313288	118.853525	112.338578	68.0878126
34.4853711	32.705862	31.3826893	35.8491266	36.6510678	36.573595	33.4539625
73.1762753	68.0169704	74.8829782	74.7604306	79.6921101	83.8449036	35.5855536
11.8543463	10.4209854	9.27753712	10.300051	13.190733	15.0271412	15.9969634
0	0	0	0	0.04564267	0.02517109	0
0	0	0	0.03093108	0	0.02517109	0
0	0	0	0.03093108	0	0	0
0.05256916	0.08016143	0	0.09279325	0.09128535	0.10068436	1.062927
0	0	0.04733437	0	0	0	0
0.02628458	0	0	0	0	0	0.78423272
0.10513833	0	0.04733437	0.03093108	0.1825707	0.17619764	2.62477891
0	0	0	0	0.04564267	0	0
0	0	0	0.03093108	0	0	0
0.34169956	0.16032285	0.23667187	0.18558651	0.13692802	0.15102655	8.03838542
0.02628458	0	0	0	0	0	0.90573357
0	0	0	0	0.04564267	0	0
6.04545378	6.49307555	4.9227748	5.59852625	7.34847063	7.50098507	10.8995057
0	0	0	0.03093108	0	0	0

0	0	0	0	0	0.02517109	0
0.02628458	0	0	0	0	0	0.73916188
0	0	0	0.03093108	0	0.02517109	0
0.15770749	0.20040357	0.09466875	0.09279325	0.13692802	0.05034218	5.2855137
0.23656123	0.20040357	0.09466875	0.46396626	0.13692802	0.32722418	8.15160212
0.31541498	0.36072642	0.56801248	0.9897947	0.3651414	0.40273745	9.29740964
0.07885374	0.12024214	0.23667187	0	0.04564267	0.12585545	2.38174383
0	0.04008071	0	0	0	0	0
10.0407102	9.41896762	9.6562121	8.78442792	8.07875343	7.62684052	307.066323
0	0	0.04733437	0	0	0	0
0	0	0	0	0.04564267	0.02517109	0
0	0	0	0	0	0.02517109	0
0	0	0	0	0	0.05034218	0
0	0	0	0	0.04564267	0	0
0	0	0.04733437	0	0	0	0
0.05256916	0	0.18933749	0.03093108	0	0.02517109	1.54956827
0	0	0	0	0	0.05034218	0
0	0	0	0.06186217	0	0	0
0	0	0	0	0	0.02517109	0
0	0	0	0	0	0.02517109	0
0	0	0	0.03093108	0	0.02517109	0
0	0	0	0	0	0.02517109	0
0	0	0	0	0.04564267	0	0
0	0	0	0	0	0.02517109	0
0.10513833	0.12024214	0.09466875	0.21651759	0.04564267	0.25171091	2.70766667
0.07885374	0.04008071	0.04733437	0.15465542	0.09128535	0.10068436	2.35269817
0	0	0	0	0	0.02517109	0
0.02628458	0	0	0	0	0	0.81401371
0	0	0	0	0	0.05034218	0
0.02628458	0	0	0	0	0	1.19087191
0.02628458	0	0	0	0	0	0.7225515
0.02628458	0	0	0	0	0	0.69899004
0.15770749	0.24048428	0.14200312	0.21651759	0.27385605	0.32722418	4.38457387
0	0.04008071	0	0	0	0	0
0.02628458	0	0	0	0	0	0.79391461
0.02628458	0	0	0	0	0	0.79391461
0	0	0.04733437	0	0	0	0
0	0	0	0	0.04564267	0	0
0	0	0	0.03093108	0	0	0
0.02628458	0.04008071	0	0.09279325	0	0.07551327	0.6495665
0	0	0	0	0	0.02517109	0
0.02628458	0.08016143	0.23667187	0	0.04564267	0.07551327	0.67691667
0	0.04008071	0	0	0	0	0
0	0	0	0	0	0.02517109	0
0	0	0	0	0.04564267	0	0

0.02628458	0	0	0	0	0	0.51860551
0	0	0	0	0	0.02517109	0
0.02628458	0	0	0	0	0	0.88091895
0.02628458	0	0	0	0	0	0.76556052
0	0	0	0.06186217	0	0	0
1.2879445	1.48298639	1.13602495	0.89700144	1.27799489	0.70479054	53.4075777
0	0	0	0.03093108	0	0	0
0	0	0	0	0.04564267	0	0
0.13142291	0.20040357	0.09466875	0.03093108	0.13692802	0.05034218	5.64097223
0	0	0.04733437	0	0	0	0
0	0	0.04733437	0.09279325	0.04564267	0	0
0.10513833	0.28056499	0.23667187	0.03093108	0.1825707	0.12585545	3.62293427
0	0	0	0	0	0.02517109	0
0.02628458	0	0	0	0	0	1.60767708
0.02628458	0	0	0	0	0	1.2609232
0	0	0.04733437	0	0	0	0
0.02628458	0	0	0	0	0	1.02074736
0	0.04008071	0	0	0	0	0
0.26284582	0.16032285	0.28400624	0.15465542	0.1825707	0.12585545	7.22551498
0.02628458	0	0	0	0	0	0.55437141
0	0	0	0	0	0.02517109	0
0	0	0	0	0	0.02517109	0
0	0	0	0	0.04564267	0	0
0	0	0	0	0.04564267	0	0
0.02628458	0.04008071	0.14200312	0.15465542	0.22821337	0.05034218	0.49850452
0	0	0	0.03093108	0	0	0
0.49940705	0.20040357	0.28400624	0.52582843	0.27385605	0.65444836	12.4676998
1.36679825	3.16637635	2.9820655	1.54655421	1.50620827	2.49193799	39.8091468
0	0	0	0	0.04564267	0	0
0	0	0	0	0	0.02517109	0
0	0	0	0	0.04564267	0	0
0	0	0.04733437	0	0	0	0
0.05256916	0	0	0.18558651	0.04564267	0	1.9486995
1.07766785	1.56314782	1.08869058	0.89700144	1.23235222	0.45307964	38.2114553
0.13142291	0.08016143	0.09466875	0.06186217	0.04564267	0.02517109	4.65993358
0	0	0	0	0.04564267	0	0
0.02628458	0	0	0	0	0	1.02074736
0	0	0	0	0	0.02517109	0
0.02628458	0	0	0	0	0	0.9456924
0	0	0	0.03093108	0	0	0
0	0	0.04733437	0	0.09128535	0.05034218	0
0	0.04008071	0.04733437	0.03093108	0.13692802	0.07551327	0
0	0	0	0	0	0.02517109	0
0	0	0	0	0	0.05034218	0
0	0	0	0	0	0.05034218	0

0	0	0	0	0.04564267	0	0
0	0	0	0	0.04564267	0	0
0	0	0.04733437	0	0	0	0
0.23656123	0.04008071	0.09466875	0.12372434	0.45642675	0.22653982	5.03272826
0.05256916	0.04008071	0.09466875	0	0	0.05034218	2.00959636
0	0	0	0.03093108	0	0	0
0	0	0	0.03093108	0	0.02517109	0
0	0	0.04733437	0	0	0	0
0	0.04008071	0	0	0	0	0
0	0	0.04733437	0	0	0	0
0	0	0	0	0	0.02517109	0
0.02628458	0	0	0	0	0	1.03721102
0	0	0.04733437	0	0	0	0
0	0	0	0	0	0.02517109	0
0	0	0	0	0	0.02517109	0
0	0	0	0.03093108	0	0	0
0	0	0	0	0.04564267	0	0
0.02628458	0	0	0	0	0	1.07178472
0	0	0	0	0	0.02517109	0
0	0	0	0	0	0.02517109	0
0.02628458	0	0	0	0	0	0.78423272
0	0.04008071	0	0	0	0	0
0	0	0	0	0	0.02517109	0
0	0	0	0	0	0.02517109	0
0	0	0.04733437	0	0	0	0
0	0	0	0	0	0.02517109	0
0.02628458	0	0	0.03093108	0.09128535	0.02517109	1.08995057
0.07885374	0.04008071	0.09466875	0	0	0.05034218	3.06224207
0	0	0	0.06186217	0	0	0
0	0	0	0	0	0.02517109	0
0	0	0	0	0.04564267	0	0
0.23656123	0.56112999	0.5206781	0.15465542	0.31949872	0.32722418	4.22455292
0	0	0	0	0	0.02517109	0
0.13142291	0.08016143	0.09466875	0.03093108	0.09128535	0.05034218	2.45446883
0	0	0	0.03093108	0	0	0
0.02628458	0	0	0	0	0	0.84614583
0	0	0	0	0	0.02517109	0
0	0	0	0.03093108	0	0	0
0.18399207	0.24048428	0.18933749	0.30931084	0.22821337	0.42790854	5.29587745
0	0	0	0	0.09128535	0	0
0	0	0.04733437	0	0	0	0
3.49584936	3.56718348	3.73941547	4.42314504	3.788342	5.2104158	6.5791093
0.63082996	0.48096856	0.47334373	0.55675952	0.41078407	1.68646309	0.38430528
67.9982127	71.9448803	80.7524404	69.2237665	61.1611841	64.9162433	52.0859188
36.0098769	38.3973233	37.8201641	32.6013628	32.8627258	35.7177779	30.0070518

0.7096837	0.64129141	0.47334373	0.71141494	0.31949872	0.276882	0.54242151
14.0885358	15.2707518	16.6143649	12.4342959	13.8753731	14.2216663	13.2673582
4.91521677	4.80968559	4.26009358	5.16549107	5.11197957	6.61999689	7.06546686
83.2169855	79.5602158	81.5571248	79.0598513	94.1151953	97.0093841	25.2162777
77.0663934	78.4780366	83.4031653	76.8018821	71.7959274	63.8842285	97.6934551
101.589908	108.538572	115.590539	103.062373	101.463666	81.9822428	60.8586869
0	0	0	0	0	0.02517109	0
45.235765	48.778228	44.0683013	47.0771102	54.3147829	57.0628629	58.0044499
23.4195622	21.8840694	18.6970774	18.373064	20.0371342	17.0911707	12.4940278
6.04545378	5.65138057	6.24813724	6.77390745	6.84640121	7.04790543	5.1285122
11.6440697	10.5412276	12.0702651	13.7334014	11.8670954	14.674746	10.7745983
0	0	0	0	0	0.02517109	0
58.5883325	64.6902712	69.3448565	61.2435468	54.6799243	55.5274264	57.7985842
0.78853745	0.96193712	0.66268122	0.77327711	1.09542419	1.38441	1.16147652
0.02628458	0	0	0	0.09128535	0.07551327	0.09319867
6.88656039	6.73355983	5.72745914	6.95949395	7.07461458	8.25611779	10.3111725
8.09565115	6.73355983	7.14749033	7.70183997	8.71775087	9.79155433	15.073502
138.887729	151.064208	151.327991	138.911499	133.413538	108.965652	45.2160517
21.5270724	27.7759343	27.1699301	21.4043103	24.5101163	20.6402945	23.9180296
19.6871517	20.2006795	18.2237336	16.3625436	18.4852833	17.0408285	6.14597492
0	0	0	0.03093108	0	0.02517109	0
16.086164	15.8318817	14.6736556	17.630718	15.7923655	20.9171765	16.0113649
7.41225202	8.89791834	9.94021834	8.41325491	6.93768656	6.92204998	18.2808443
0	0	0	0	0	0.02517109	0
0	0	0	0	0	0.02517109	0
0	0.04008071	0	0	0	0	0
43.0541447	45.010641	53.9138509	42.5302408	41.260978	40.6764828	21.0670005
37.9549359	36.6337719	39.4768671	33.6530196	35.6012863	32.8482735	143.745245
52.9108629	54.0688822	62.4340381	51.6858417	46.7837416	49.3605091	35.0338725
78.0652075	73.107221	89.5566338	72.7499101	70.2440764	76.218063	78.565215
17.5843851	16.4330924	16.8510368	15.4964732	14.3317999	15.782274	18.5357341
43.2644214	46.9345152	51.0264542	43.4891044	42.7671862	37.9328339	50.9136408
22.867586	23.3269751	30.3886675	22.8580712	20.904345	23.9377074	71.7271314
0	0	0	0	0.09128535	0	0
0	0	0	0	0	0.02517109	0
0.15770749	0.12024214	0.04733437	0.09279325	0.22821337	0.30205309	0.3566012
0.7096837	0.40080713	0.61534685	0.34024193	0.31949872	0.553764	0.62165816
93.047419	103.047514	113.98117	96.3503274	90.1442826	83.4925083	83.1739405
0.26284582	0.08016143	0.18933749	0.09279325	0.13692802	0.05034218	0.41461691
91.0497908	93.7087076	94.0533993	87.0710021	85.0779457	74.9846796	66.7344927
1.31422908	0.72145284	1.08869058	0.80420819	1.64313629	1.78714745	1.75798478
0.63082996	0.68137213	0.7100156	0.68048385	0.45642675	0.276882	2.86871747
0	0	0.04733437	0.03093108	0	0.07551327	0
34.1436716	40.962489	41.9382545	36.4986794	35.1448595	31.7407455	32.6563336
120.0154	111.504544	122.832698	108.97021	96.123473	110.224207	210.333913

65.5537466	65.8125312	66.3154567	62.6045145	62.0740376	51.7265917	51.7527802
0.34169956	0.28056499	0.42600936	0.37117301	0.22821337	1.28372563	0.36237195
40.2154099	41.8442646	42.4589326	42.22093	34.0037927	32.8482735	29.5998308
0.81482203	0.64129141	0.85201872	0.86607036	1.09542419	1.23338345	0.57850249
0	0	0	0.03093108	0	0	0
34.7745015	33.828122	35.3114423	34.7974698	29.8046666	29.3998341	35.9891165
16.585571	17.8759981	17.7977243	14.4138852	15.5641521	15.9333005	21.7925723
17.0849781	17.7156753	19.6910992	15.2180934	17.2985737	16.084327	27.4636033
41.7924848	44.1288653	43.4056201	35.3542293	38.5680601	40.4247719	16.2945438
1.94505904	1.24250211	1.13602495	0.40210409	1.59749362	1.107528	0.42202236
0	0	0	0	0	0.02517109	0
0	0	0.04733437	0	0	0	0
226.520525	238.039356	236.813868	229.106541	216.20935	178.035125	150.802298
0.05256916	0.12024214	0.04733437	0.18558651	0.04564267	0.22653982	0.09901014
77.6709388	77.2355345	67.3568129	71.2033559	65.3146675	64.1107684	46.1567722
0	0	0.04733437	0	0	0	0
519.015349	713.316454	641.096749	837.58283	569.757509	836.787744	209.746889
0	0	0.04733437	0.03093108	0.13692802	0	0
192.771122	194.471621	154.641397	220.198389	188.139105	210.405148	63.4335103
43.8426822	52.7462186	76.7763531	51.0053579	60.202688	30.4570199	181.803754
0.05256916	0.04008071	0.14200312	0	0	0.05034218	0.02042792
56.9586884	62.6461548	70.5282159	51.3765309	64.127958	46.8434	20.9648638
0.02628458	0	0	0	0	0	0.0097568
0	0	0	0.03093108	0	0	0
261.479018	313.270855	294.514469	291.803849	247.200726	196.057627	121.945647
11.1972318	12.1444561	12.2596026	11.3517079	10.5891005	8.93573725	4.78011124
4.07411016	3.96799061	4.40209669	3.58800577	3.10370188	3.62463708	5.22958968
0	0	0	0	0.04564267	0	0
40.4256866	40.7620854	49.4170855	40.6743758	35.0079315	39.2920728	84.3893295
23.7612618	28.4172257	26.2232427	23.9715903	25.7424685	19.8851618	18.7225776
0	0	0.04733437	0	0	0	0
5.51976215	4.88984702	3.88141859	5.75318167	5.56840632	5.78935089	5.13283448
0.10513833	0.16032285	0.14200312	0.09279325	0.09128535	0.05034218	0.12162096
0	0	0	0	0.04564267	0	0
1.15652159	1.36274425	1.08869058	1.42282987	1.55185094	1.661292	0.58436837
0.02628458	0.04008071	0	0	0.04564267	0.17619764	0.04468873
8.93675776	10.7015504	13.4429619	9.83608478	8.44389483	8.58334197	5.95273845
46.8916937	42.8062018	45.298995	44.4788991	37.9747054	40.1982321	44.9016973
2.81245024	2.20443923	2.08271241	1.91772722	1.64313629	1.9130029	4.26852228
0	0.04008071	0	0	0	0	0
12.064623	9.33880619	10.1768902	15.4964732	9.90446042	11.2011354	16.7519587
8.27964322	11.0622769	9.41954024	14.7850583	10.4978152	10.0180942	2.14834354
0.13142291	0.04008071	0	0.06186217	0.22821337	0.17619764	0.05216344
0	0	0.04733437	0	0	0	0
1.8662053	1.08217926	1.70403743	1.94865831	1.73442164	2.69330672	1.69480435

43.0278602	43.2470896	44.2103044	41.5404461	37.4269933	39.3927572	33.8382178
0	0	0	0.03093108	0	0	0
14.1673895	15.5913975	14.5789869	16.3625436	17.4811444	17.3177105	12.4906371
7.85908991	7.97606194	6.53214348	8.6297725	7.71361203	9.66569888	3.96123155
52.7005862	51.7442008	41.4175764	48.4071468	42.3107595	40.2234032	44.7848913
58.6409017	68.1372125	67.877491	62.8519631	58.6051944	50.7700902	19.575536
0.63082996	0.72145284	0.61534685	0.55675952	0.82156815	1.73680527	0.70121309
86.9493961	82.5261886	73.084272	76.028605	78.0946165	55.9049927	35.0342279
27.9667949	23.7277822	22.9571709	25.6727999	21.8171985	17.2925394	150.710874
0	0	0	0.03093108	0	0	0
7.49110577	7.41493195	4.78077168	6.8667007	3.74269933	4.43011199	3.08596039
22.368179	24.8901229	26.4599145	22.3631739	21.7259132	19.5831087	12.8342702
111.18378	120.763189	110.809767	108.506243	116.20625	82.0074139	36.8289958
0	0	0	0	0.04564267	0	0
0	0	0	0.03093108	0	0.02517109	0
26.074305	22.3249573	23.0045053	23.3838997	18.2570699	22.1002178	16.5695134
0.13142291	0.08016143	0	0.03093108	0	0.05034218	0.15972947
7.49110577	5.93194556	7.85750593	7.23787371	8.44389483	9.0615927	5.48563866
4.96778593	3.9279099	5.30144978	4.7015248	5.34019294	7.07307652	4.70539634
77.7760771	71.2234275	60.5879975	64.0892065	64.7213128	58.4221018	36.1277121
56.7484118	63.2473655	64.0907411	56.3873665	57.7379835	53.5137391	11.7193376
0	0	0	0	0	0.02517109	0
13.1685754	12.5051825	11.6915901	12.8054689	11.6388821	7.80303816	14.0443979
31.4889288	34.0285256	35.7374517	31.2713262	32.6345124	21.9240201	19.5830925
13.5891287	15.230671	12.9222838	13.8571257	17.7093578	17.5694214	2.56316106
19.5557287	23.0864908	23.2411772	18.8370303	18.7134966	19.6082798	25.2610718
49.6778593	47.8964523	51.4524635	48.9948374	42.6302582	43.7221848	30.4688863
0.05256916	0.04008071	0.14200312	0.06186217	0	0.02517109	0.03058601
0.94624494	0.52104927	0.85201872	0.80420819	0.95849617	1.43475218	1.23535486
4.59980179	5.33073486	5.25411541	5.44387082	6.02483306	6.67033907	7.46764405
14.9822115	12.4651018	19.8804367	11.8466053	14.2405145	14.5488905	14.3632592
38.4806275	42.4454753	50.0324323	38.2617512	38.9332015	41.0540492	20.515487
0	0	0	0	0	0.02517109	0
0	0	0.04733437	0	0	0	0
0	0.04008071	0	0	0	0	0
0	0	0	0.03093108	0	0	0
0	0.04008071	0	0.12372434	0	0.02517109	0
6.04545378	3.80766776	5.06477792	3.64986794	4.3360541	4.53079635	8.37047491
15.3501957	12.1444561	11.5022527	14.0117812	15.0620827	15.5809052	7.50806411
1.41936741	0.80161427	1.13602495	0.74234602	0.91285349	0.88098818	0.89430402
15.4027648	9.94001689	9.23020275	15.5892665	12.6886636	14.951628	5.42605484
22.6047402	21.4431816	22.6258303	24.0953146	45.323176	18.1483565	12.3364023
0	0	0	0.03093108	0	0	0
0.02628458	0	0	0	0	0	0.02683935
0.05256916	0.04008071	0.09466875	0.09279325	0.22821337	0.10068436	0.1096455

0.10513833	0.08016143	0.14200312	0.18558651	0.31949872	0.12585545	0.12325268
11.6966388	11.9440526	10.9815746	13.1457108	11.3193833	15.9333005	20.9186053
0	0	0.04733437	0	0	0	0
23.4195622	22.4051187	23.0991741	21.7136211	21.4520571	18.5510939	24.8795533
24.2343843	17.0743838	17.9397274	17.0120963	17.2985737	16.7136043	35.9994723
0.42055331	0.24048428	0.28400624	0.24744867	0.13692802	0.22653982	0.53533472
12.8531604	13.9881689	12.4016057	13.0838486	10.3152445	10.5970292	10.5312002
37.6658055	40.681924	39.6188703	37.9215093	35.783857	31.8414299	29.1620413
0.02628458	0	0	0	0	0	0.01187135
29.4913006	36.7540141	35.8321204	29.1680124	30.8544481	28.4685037	26.7033855
12.6165992	13.2667161	12.4016057	10.8258795	10.8173139	11.07528	17.8012688
23.1567164	24.7298001	22.2471553	21.7754833	20.7217743	19.9103328	21.5991386
23.6561235	28.0164186	29.2053082	22.4868982	23.2321214	24.1390761	29.2896635
30.4638301	27.0544815	26.6019177	31.3331883	31.858587	30.9352706	34.9259183
6.96541414	7.17444767	11.170912	8.38232383	10.0870311	11.2766487	9.43075655
4.10039474	5.17041201	3.97608734	5.50573299	4.38169677	5.81452198	4.2418203
0	0	0.04733437	0	0	0	0
0.23656123	0.20040357	0.04733437	0.09279325	0.04564267	0.12585545	0.58050527
6.78142207	4.8497663	6.95815284	4.85618022	4.60991015	5.89003526	19.292125
0.02628458	0	0	0	0	0	0.1061173
0.02628458	0.04008071	0.09466875	0	0	0.02517109	0.11799465
0	0	0	0	0	0.02517109	0
0	0	0	0	0.04564267	0	0
4.25810223	4.44895917	3.21873737	4.82524914	4.60991015	4.90836271	4.28361328
43.9215359	45.6118517	36.0687923	42.6539651	52.89986	48.2026389	43.0517373
0.52569163	0.40080713	0.42600936	0.37117301	0.22821337	0.30205309	0.1696533
15.4027648	14.4691375	15.0523306	15.434611	17.2072884	17.4435659	10.1164969
2.36561235	3.60726419	2.60339052	5.38200866	1.96263501	4.20357217	0.64022539
29.5964389	34.6297363	30.4360019	29.4463922	27.5225329	26.8323828	19.2323442
30.8843834	27.0544815	28.1166176	29.4773233	32.269371	36.8756481	70.8825731
2.75988107	2.96597278	3.45540923	1.79400289	2.87548851	2.44159581	5.02399089
17.6369543	19.0784195	20.0697742	14.7850583	17.2985737	16.8898019	15.8873538
19.6082979	25.8119793	25.371224	25.3944202	31.1283042	26.1024212	13.4003028
0.31541498	0.32064571	0.23667187	0.09279325	0.1825707	0.22653982	0.14079274
0.78853745	0.88177569	0.80468434	1.26817445	0.82156815	1.08235691	0.13743766
0	0	0	0.03093108	0	0	0
7.12312163	10.4209854	10.1295558	9.95980912	9.76753239	12.6862298	10.3180696
54.8296373	59.7202628	53.6771791	50.0155632	51.9413638	49.5870489	99.5876584
7.99051282	9.21856405	9.18286837	7.39252913	8.21568145	7.04790543	5.67141089
17.1112627	14.4290568	22.6258303	19.084479	13.7384451	12.2331501	18.3613646
3.39071103	3.84774847	2.13004679	2.28890023	2.28213374	3.0205309	7.46679906
0	0	0.04733437	0	0	0	0
5.86146171	7.05420553	6.53214348	5.66038841	4.56426747	5.58798217	10.4751494
23.183001	24.9302036	21.5844741	25.3944202	19.2612087	16.3863801	23.7914629
9.67272605	10.1805012	9.8455496	8.50604816	8.3069668	8.48265761	8.13510027

15.1662036	15.1505096	14.9103275	13.3003662	12.3235222	13.9196132	18.8830469
22.8150169	24.5293965	20.9691273	21.9610698	19.2612087	20.0361883	18.2413557
0	0	0	0	0	0.02517109	0
0.23656123	0.08016143	0.14200312	0.34024193	0.31949872	0.20136873	0.27652353
0.89367578	1.12225997	0.99402183	0.89700144	1.23235222	1.78714745	0.95644831
0.02628458	0	0	0	0	0	0.04345073
2.47075067	1.72347067	1.18335933	2.07238264	2.00827769	3.24707072	2.84598203
17.5055314	19.8800338	14.2003119	18.713306	15.6097948	30.5325332	13.6570528
21.9213411	18.597451	18.6024086	15.1562313	18.804782	17.4183949	28.5580977
0.02628458	0	0	0	0	0	0.02061124
0	0	0	0	0	0.05034218	0
0	0	0	0.03093108	0	0	0
107.083386	107.937361	150.807313	119.734227	190.558167	128.573932	75.9602951
50.2298355	50.1409723	52.5884885	49.211355	49.5223021	37.681123	23.1389261
229.227837	270.665057	246.754087	230.281922	232.093001	203.860665	220.796092
0.02628458	0	0	0.06186217	0.04564267	0.05034218	0.01291307
0.02628458	0	0	0	0	0	0.03921164
27.6513799	19.3990652	28.6372957	21.5898968	26.381466	11.3773331	21.858175
0	0	0	0	0.04564267	0	0
0	0	0	0	0.04564267	0.02517109	0
0	0	0	0	0	0.02517109	0
1.73478239	1.28258282	2.08271241	0.89700144	1.59749362	1.51026545	2.40060379
0	0	0	0	0	0.02517109	0
0.02628458	0	0	0	0	0	0.06959641
0.02628458	0	0	0.06186217	0.04564267	0.02517109	0.05562896
0	0.04008071	0	0	0	0	0
0	0	0	0	0.04564267	0	0
0	0	0.04733437	0	0	0	0
0.02628458	0	0	0	0	0	0.06443596
0	0	0	0	0.04564267	0	0
0	0	0	0.03093108	0	0.02517109	0
0	0.04008071	0	0	0	0	0
0	0	0	0.03093108	0	0	0
0.18399207	0.04008071	0.14200312	0.09279325	0.04564267	0.05034218	0.43367012
0	0	0	0.03093108	0	0	0
0	0	0	0	0	0.02517109	0
0	0	0	0.03093108	0	0	0
0.02628458	0	0	0	0	0	0.02208348
0	0	0	0.03093108	0	0	0
0.02628458	0	0	0	0	0	0.06848465
0	0	0	0.03093108	0	0	0
4.52094804	4.32871703	5.34878416	3.64986794	7.53104133	7.6771827	3.16384964
0.02628458	0	0	0	0	0	0.06698655
0	0	0	0	0	0.05034218	0
0	0	0	0	0	0.02517109	0

0.07885374	0.44088785	0.09466875	0.12372434	0	0.15102655	0.04114337
0	0.04008071	0	0	0	0	0
0	0	0.04733437	0	0	0	0
356.418927	390.145663	414.317767	347.850973	389.377658	292.714615	145.28558
0	0.04008071	0	0	0	0	0
0	0	0	0	0	0.02517109	0
52.2274637	55.9526757	56.1385665	48.4071468	51.6218651	48.22781	20.2404839
0.02628458	0.12024214	0	0	0	0.02517109	0.08373318
2.23418944	1.24250211	1.65670306	2.50541782	2.09956304	2.16471381	2.91992633
52.5428787	53.2271872	47.3343731	52.1807391	43.360541	40.0472055	18.5363893
30.2272689	33.3872341	30.578005	28.8587016	31.858587	23.4342856	8.15809662
13.694267	12.7857475	14.1056432	13.0219865	10.9085993	12.1072947	12.6573443
44.4997967	46.8142731	36.4948016	50.2320808	47.285811	47.0447688	22.5174544
37.823513	35.3912698	41.8435858	39.5917878	41.534834	47.0195977	53.9894358
0.07885374	0.04008071	0	0.03093108	0.09128535	0.22653982	0.05286962
0.02628458	0	0	0	0.09128535	0.05034218	0.00896891
0	0	0	0.03093108	0	0.02517109	0
0	0	0.04733437	0	0	0	0
96.1752842	98.0775053	112.419136	93.8758406	97.7209666	82.9890865	72.1999442
3.20671896	3.3266992	3.31340611	1.26817445	3.51448595	2.54228017	3.10711452
39.4005879	37.8361933	43.4529545	41.3857907	35.646929	31.1114683	25.6919824
0	0	0.04733437	0	0	0	0
0.02628458	0.08016143	0.14200312	0	0	0.05034218	0.03351073
22.9464398	25.1306072	23.1465084	21.1877927	21.6802705	19.4824243	24.5367499
0	0	0.04733437	0	0	0	0
0.55197621	0.48096856	0.28400624	0.71141494	0.41078407	0.88098818	0.25880582
0.02628458	0.04008071	0.09466875	0	0	0.02517109	0.02642033
0	0	0	0	0	0.02517109	0
0.15770749	0.20040357	0.09466875	0.03093108	0.13692802	0.07551327	0.33727491
0.47312247	0.80161427	1.70403743	0.68048385	0.41078407	0.85581709	0.26188405
186.042269	209.58205	208.933923	189.267304	182.022987	159.559545	117.310705
165.382588	208.860597	158.522815	175.255523	171.707742	111.004511	113.466116
61.5059211	60.8425227	41.748917	69.8423882	58.376981	57.4152582	56.762948
73.7282515	78.5982787	85.2018715	76.399778	125.243499	80.2454376	75.3472719
0.63082996	0.56112999	0.61534685	1.23724337	0.45642675	0.85581709	2.79596015
3.12786522	3.48702205	2.27204991	5.6913195	2.73856048	3.44843944	2.6813395
68.6816118	56.7943707	58.0792757	55.1501232	53.1280734	52.2300135	65.5104908
168.72073	176.555542	191.562208	169.74979	157.239014	149.390424	91.5676948
13.4577058	12.8258282	13.0169526	15.0015758	13.3733037	17.5694214	11.2873592
4.1529639	3.08621492	4.2127592	4.5159383	5.65969167	4.4049409	2.29875999
49.2835906	52.5458151	52.3518166	49.5206658	49.6135874	37.0518457	15.0362615
32.0146204	35.1107048	33.0867268	33.4983642	29.9872373	27.2099492	17.8419197
16.1124485	16.3930117	15.9516837	17.8472356	19.5350648	18.9034892	13.5838188
19.9499975	14.5492989	14.2003119	21.1568616	16.4770056	20.5396101	14.376753
79.6159978	100.923236	85.6278809	81.8436489	83.7999508	76.3439185	30.4781968

0.55197621	0.52104927	0.7100156	0.89700144	0.41078407	1.25855454	0.26381105
2.10276653	2.60524636	2.03537804	2.96938409	2.28213374	3.07087308	2.81431437
0	0	0	0	0.04564267	0	0
0	0	0.04733437	0	0	0	0
0	0	0	0	0	0.02517109	0
11.3286547	13.1464739	9.60887773	11.0423971	8.1243961	10.0432652	8.09709405
0.73596829	1.32266354	1.2306937	0.80420819	2.09956304	1.81231854	0.92671041
0.02628458	0	0	0	0	0	0.05119991
22.683594	20.2407602	21.6791429	23.507624	25.5142552	24.0887339	19.8132856
28.6239094	27.0945622	30.6253394	32.7869493	30.443664	34.1823414	6.80303223
192.718553	219.321663	197.715676	178.039321	206.259247	147.376737	51.8530227
2.70731191	2.96597278	1.79870618	2.47448674	1.78006431	1.76197636	1.06301229
0.89367578	0.56112999	1.04135621	0.68048385	0.63899745	0.57893509	0.41662363
0.89367578	0.56112999	1.04135621	0.68048385	0.63899745	0.57893509	0.4171005
0.89367578	0.56112999	1.08869058	0.68048385	0.63899745	0.57893509	0.41614786
0.89367578	0.6012107	1.04135621	0.68048385	0.63899745	0.57893509	0.41614786
0.89367578	0.56112999	1.04135621	0.68048385	0.63899745	0.57893509	0.40761388
0.91996036	0.56112999	1.08869058	0.68048385	0.63899745	0.57893509	0.42291393
0.89367578	0.56112999	1.04135621	0.68048385	0.63899745	0.57893509	0.4171005
0.89367578	0.56112999	1.04135621	0.68048385	0.63899745	0.57893509	0.41925999
0.89367578	0.56112999	1.04135621	0.68048385	0.63899745	0.57893509	0.41284759
0.47312247	0.36072642	0.66268122	0.43303518	0.77592547	0.85581709	0.41414222
3.07529605	1.96395495	2.50872177	2.96938409	3.4232006	3.5742949	1.42769047
0.02628458	0	0	0	0	0	0.10289133
172.768555	268.861425	184.272714	245.685602	247.383297	236.960649	268.374895
0	0.04008071	0	0	0	0	0
117.334372	114.63084	102.052908	114.228494	69.7876497	94.769157	66.7907911
25.3383367	22.0043116	23.1465084	25.9821108	26.9291781	25.9765657	14.9019299
0	0	0	0.03093108	0	0	0
10.9869551	8.09630408	8.66219027	9.43398069	11.0455273	11.6542151	8.4185283
91.4966287	99.1997653	88.7992839	93.9067717	80.6049636	68.4150249	31.756697
31.9094821	35.7119155	30.0573269	27.4977339	33.1365819	25.5738283	29.097577
18.7409067	16.5132539	17.7977243	21.0640684	17.8919285	21.1437163	25.543705
177.894049	192.507666	223.465575	188.710545	161.529426	181.433223	164.735178
0	0	0	0.03093108	0	0.02517109	0
3.33814187	4.40887846	3.36074049	5.2892154	3.92527003	4.50562526	4.08349979
16.9535552	21.0423745	16.9457056	23.7550727	21.2694864	18.1986987	26.2852147
8.48991987	7.21452839	6.91081847	10.5474997	6.84640121	9.21261925	17.5284286
29.1233165	30.6216649	29.2999769	36.4986794	29.8046666	34.4088812	23.7983461
55.8284514	68.9388268	65.084763	59.5423371	67.2316599	29.7522294	47.0345197
51.7806258	57.9166307	56.6119102	53.6963622	50.2525849	38.1090315	30.5559465
3.36442645	3.36677991	3.55007798	3.24776384	2.69291781	1.98851618	1.23481948
0.76225287	0.56112999	0.66268122	1.08258795	0.82156815	0.85581709	0.70640357
29.7541464	27.8961764	28.7319644	31.7043613	30.6718774	35.466067	12.4458229
16.9535552	16.4330924	17.2770462	14.8159893	15.5641521	14.6495749	9.63709776

117.360657	122.48666	111.425114	124.559476	130.72062	136.729365	330.035778
58.8248937	56.5939671	58.0792757	53.0468094	56.414346	48.3033233	71.2471547
0	0	0	0	0.04564267	0.02517109	0
78.2491996	74.510046	86.6692371	69.9042504	53.5388575	83.1904552	83.6722846
0.13142291	0.08016143	0.18933749	0.30931084	0.04564267	0.37756636	0.47215186
0.23656123	0.16032285	0.14200312	0.15465542	0.04564267	0.10068436	0.20116919
0.13142291	0.16032285	0.09466875	0.21651759	0.31949872	0.17619764	0.3366863
31.1997984	27.4552886	29.8679894	33.9932616	30.443664	36.0450021	41.5302002
39.9525641	43.0867668	43.8316295	35.0758495	42.2194741	34.7612764	58.7420473
1.62964406	1.32266354	0.99402183	1.05165686	2.46470444	2.24022708	2.5075718
10.7241093	13.7877654	9.98755272	12.4342959	12.2778795	15.4802209	9.76816456
8.33221238	9.6594519	8.18884654	9.24839418	9.35674832	11.5283596	6.51705416
37.6920901	43.5276546	38.2461734	44.3551748	40.1655538	46.6420313	16.0851836
2.91758856	3.36677991	2.93473113	2.38169349	5.47712097	3.90151908	1.91523645
0	0	0	0.03093108	0	0	0
97.2003829	107.215908	106.313002	95.484257	99.2728175	86.6640657	51.5069513
15.6130415	15.5513167	13.1589557	15.8676462	16.7508616	18.6014361	23.4922555
8.06936657	5.21049272	5.49078728	8.84629009	6.02483306	7.55132725	13.4575832
0.49940705	0.64129141	0.5206781	0.27837976	0.31949872	0.52859291	0.75937513
0.84110661	0.84169498	0.94668746	1.1753812	1.23235222	1.05718582	0.87978908
0.91996036	1.48298639	0.99402183	1.63934746	1.18670954	1.48509436	0.20987951
0.18399207	0	0.09466875	0.09279325	0.04564267	0.47825073	0.12232326
6.62371458	5.17041201	5.77479351	5.1036289	4.65555282	5.23558689	4.5102658
15.1662036	12.7456668	13.4902963	15.1871624	16.7508616	15.4047076	28.4113224
0.42055331	0.28056499	0.5206781	0.30931084	0.09128535	0.32722418	0.56940417
69.1810189	59.5599399	66.2681223	61.0579603	81.9286011	78.8861987	69.9695095
0	0	0	0.03093108	0	0	0
45.7351721	45.852336	40.2815515	45.2521762	53.3106441	47.0699399	26.1496436
0.07885374	0.04008071	0	0	0.04564267	0	0.13097166
0.18399207	0.08016143	0	0.15465542	0.04564267	0.32722418	0.1299508
123.852949	140.522981	129.743517	134.828596	155.276379	145.287536	47.718894
78.1703458	91.3439455	82.4091435	77.3895727	83.6173801	66.3761665	54.11694
0.02628458	0.08016143	0.09466875	0.15465542	0.09128535	0.10068436	0.00963691
32.6191658	30.9423106	29.1579738	36.3130929	22.4105533	21.8736779	32.5204118
14.4302353	15.4711553	14.5316525	14.1355055	14.1492292	8.45748652	5.00206698
1.18280617	1.64330924	1.37269682	1.5774853	1.73442164	2.41642472	1.41923431
14.5879428	12.7456668	10.7449027	13.6096771	15.1077253	16.1346692	12.1768786
176.054128	188.940482	188.438139	183.823434	235.470559	202.123859	148.989569
0	0.04008071	0	0	0	0	0
1.44565199	1.5230671	1.51469994	1.48469204	2.51034711	1.76197636	1.56223038
9.5938723	8.53719192	6.53214348	7.6399778	6.34433179	6.99756325	6.08400348
0.31541498	0.12024214	0.23667187	0.34024193	0.27385605	0.42790854	0.22143042
0	0	0	0	0	0.02517109	0
45.5248954	42.6057982	45.0149888	58.1813694	47.9704511	43.9990668	48.9581839
7.70138242	6.81372125	7.71550281	7.4543913	7.7592547	10.5718582	4.06515112

10.0144256	13.5072004	8.75685902	8.16580624	9.72188972	8.88539506	4.65709918
20.6859658	19.0784195	18.9810836	22.2085185	19.078638	26.0269079	19.9880231
11.0920935	13.5472811	8.47285278	13.5168838	12.5973782	14.146153	3.81360163
3.75869518	3.60726419	1.89337492	5.41293974	3.19498723	3.34775508	1.78734945
0	0	0	0	0	0.02517109	0
0.07885374	0.04008071	0.14200312	0.09279325	0	0	0.10207474
0.21027665	0.36072642	0.28400624	0.37117301	0.22821337	0.60410618	0.26261188
0	0	0	0	0	0.02517109	0
0.9988141	1.28258282	0.85201872	0.89700144	3.33191526	0.85581709	0.37951066
18.031223	13.6675232	10.6028996	19.5484452	16.1118642	14.5740616	14.6755353
17.7946618	22.926168	26.1759083	16.1150949	19.7176355	21.9240201	38.9060728
20.8436732	19.3589845	23.5725178	21.1877927	23.6885482	28.3174772	60.4929029
0.26284582	0.20040357	0.18933749	0.27837976	0.41078407	0.37756636	0.34042924
8.64762736	8.69751478	8.99353088	10.9186727	9.08289227	10.3201472	24.3184258
92.5742966	99.0795232	106.644343	113.764528	100.733383	121.802909	64.8595497
25.1017755	20.2006795	20.4011148	21.7136211	23.7798335	28.8460701	19.4839037
44.3683738	41.4033768	38.3408422	38.4473377	51.5762224	53.8661344	33.3078726
0	0.04008071	0	0	0	0	0
35.4841852	30.5415035	24.3298678	35.5707469	29.5308106	40.3492586	48.0700789
5.80889254	5.37081558	6.76881535	6.95949395	6.34433179	8.15543343	4.89220841
0.44683789	0.76153355	0.80468434	0.43303518	0.27385605	0.60410618	0.41191425
0.05256916	0.08016143	0.09466875	0.03093108	0	0.05034218	0.09429191
6.70256832	6.89388268	7.3841622	5.38200866	6.84640121	5.94037744	5.41198226
6.20316127	4.97000844	4.26009358	5.84597492	6.70947319	6.36828598	9.05517403
17.3741085	15.511236	13.9163057	16.7646477	19.4437794	17.6449347	19.9189232
0.2891304	0.32064571	0.33134061	0.34024193	0.41078407	0.67961945	0.41881463
0	0	0	0.03093108	0	0	0
0	0	0	0.06186217	0	0	0
0.47312247	0.20040357	0.23667187	0.34024193	0.59335477	0.52859291	1.30352196
83.7952463	104.209854	98.8815053	78.5340229	90.6007093	90.9431512	59.0980057
45.1569113	46.4535467	54.055854	41.3548596	44.3646798	43.6718426	70.6843053
0.10513833	0.16032285	0.04733437	0.18558651	0.27385605	0.15102655	0.2103257
0.07885374	0.08016143	0.04733437	0.03093108	0.04564267	0.02517109	0.0927506
9.12074983	9.6594519	7.85750593	8.19673732	6.84640121	7.9540647	11.157279
37.6920901	40.3211975	40.470889	34.0860548	33.95815	37.9076628	75.1559556
6.46600709	6.85380197	5.68012477	7.17601154	7.57668401	7.40030071	17.3269907
5.49347756	5.61129986	4.40209669	5.59852625	5.84226237	6.89687889	1.88528271
87.8167873	81.6844936	75.1196501	82.4622705	68.5552974	69.0694732	46.747164
12.2748996	11.9440526	10.9342402	11.7228809	14.6512986	13.9699554	7.10634357
348.559837	360.20537	364.474673	335.540402	323.287065	291.506403	344.974204
58.3780558	67.6963247	56.6119102	54.4077772	54.588639	55.8294795	58.1302532
5.86146171	8.01614265	7.52616532	6.8667007	8.53518017	9.38881688	4.09845087
0	0	0	0.03093108	0	0	0
0.15770749	0.16032285	0.09466875	0.12372434	0.22821337	0.37756636	0.27658961
18.9774679	19.5193074	17.2297118	19.084479	20.8587024	24.2901027	10.1485714

0	0	0	0	0	0.02517109	0
0.02628458	0	0	0.12372434	0.04564267	0	0.02589895
80.1416894	86.4540985	80.0897592	70.522872	84.5758763	55.9049927	37.2831902
0.21027665	0.24048428	0.28400624	0.18558651	0.1825707	0.10068436	0.10572476
33.1185729	36.6337719	36.3527985	33.1271912	31.2652322	29.5760317	19.0070197
403.126629	381.327906	326.465171	395.639498	417.767402	477.520764	177.133214
37.192683	51.864443	49.2750824	45.6852114	36.8336385	34.5095655	14.4092673
9.93557186	8.01614265	9.56154336	9.99074021	9.85881774	11.5535307	11.7430326
55.8021668	59.2392942	54.9078728	56.1399179	51.074153	46.6420313	57.8246243
1.23537534	1.32266354	1.60936868	1.51562313	2.87548851	1.20821236	1.81418543
77.3292392	65.8926926	76.0190031	80.7610609	80.5136782	84.247641	46.4387431
16.4015789	16.2326889	15.8096806	17.3523383	19.4437794	18.9538314	12.7998788
174.003931	190.744114	199.940392	195.546314	161.575069	113.169224	159.085535
75.1476189	80.1614265	79.9004217	73.801567	72.8913515	65.8224025	38.2232747
134.655912	125.612955	128.938832	125.394615	114.289258	105.768924	84.8866756
41.0828011	44.6499146	46.056345	37.3338187	41.7630474	37.681123	51.5975212
5.96660003	4.97000844	5.82212789	4.48500721	8.1243961	9.0615927	3.26863142
1.15652159	0.84169498	0.94668746	1.36096771	1.36928024	1.28372563	0.76826274
276.645222	266.095855	325.755155	276.585755	252.632205	209.97724	146.786392
0	0	0	0	0	0.02517109	0
4.38952514	3.52710277	5.82212789	5.6913195	5.93354771	5.46212671	2.0881359
0	0	0	0	0.04564267	0	0
0	0	0	0.06186217	0	0	0
1.07766785	0.6012107	0.42600936	0.52582843	0.41078407	0.52859291	1.01368336
5.23063175	6.73355983	5.11211229	3.15497059	5.56840632	4.95870489	3.69858659
60.0339845	66.3736612	60.39866	58.6144046	55.2732791	50.0904708	54.0188961
10.2772714	11.0221961	9.04086526	12.1559161	10.8629566	11.6793861	3.85054665
0	0	0.04733437	0	0	0	0
137.520931	148.138316	143.896494	139.097086	131.040119	89.508399	83.2808565
13.7731208	14.8699446	13.9636401	18.2802708	13.7840878	17.191855	32.8429938
0.68339912	0.44088785	0.94668746	0.95886361	0.91285349	1.00684363	0.35604433
166.065987	170.182709	191.79888	165.574094	164.039773	143.651415	117.357641
0	0	0.04733437	0	0	0	0
0	0	0.04733437	0	0	0	0
0	0.04008071	0	0	0	0	0
0	0.04008071	0	0	0	0	0
0	0	0	0.03093108	0	0	0
0	0.04008071	0	0	0	0	0
20.0025666	17.3549488	19.1704211	17.7853734	17.0247177	14.3475218	19.6143048
47.6802311	51.303313	48.2810605	44.3861059	45.5513894	39.7954946	44.17003
0	0	0	0	0	0.02517109	0
0.36798414	0.12024214	0.23667187	0.15465542	0.04564267	0.20136873	0.48273414
22.1841869	23.7277822	20.5904523	25.0232471	24.1449749	25.4731439	29.0552347
25.6274671	27.53545	26.3179114	32.5395006	27.2486768	28.8460701	51.016604
0	0	0	0.03093108	0	0.02517109	0

0	0	0	0.03093108	0.04564267	0	0
0	0	0	0.03093108	0.04564267	0	0
0.94624494	1.2024214	0.7100156	0.71141494	0.68464012	0.72996163	2.51363192
0	0	0	0	0	0.05034218	0
3.75869518	2.96597278	1.79870618	4.02104095	3.74269933	3.09604417	2.69437824
0	0	0	0	0	0.05034218	0
0	0	0	0	0.04564267	0	0
0	0	0	0	0.04564267	0	0
0	0	0	0.03093108	0	0	0
0	0	0	0	0	0.02517109	0
1.94505904	1.68338996	1.2306937	2.62914216	2.32777641	1.81231854	0.90470041
1.26165992	1.16234068	2.13004679	0.92793253	1.32363757	1.03201472	4.74883077
0.02628458	0	0	0	0	0	0.03437043
0.02628458	0	0	0	0	0	0.01514177
32.3037508	28.1767414	31.9033674	34.147917	31.5847309	36.5987661	108.116834
98.015205	76.874808	83.8291747	99.5671601	84.8497323	111.558275	252.688213
20.1602741	24.6496387	20.8744585	19.826825	18.0744992	19.1803712	20.7678033
165.671718	191.866374	192.13022	166.625751	169.060467	148.182212	156.195586
0.52569163	0.28056499	0.56801248	0.49489735	0.77592547	0.70479054	0.19820337
0	0	0	0.03093108	0	0.02517109	0
0	0	0.04733437	0	0.13692802	0.05034218	0
0.02628458	0	0	0	0	0	0.05012243
10.1195639	8.65743406	10.7922371	8.90815226	12.8712343	13.9699554	7.44160718
0.02628458	0	0.14200312	0	0	0.02517109	0.05856747
0.02628458	0	0	0	0	0	0.02663922
23.2881393	20.8419709	23.1465084	27.4049406	21.6802705	27.5120023	47.7987214
35.7733156	33.6677991	38.3408422	34.8284008	32.1780857	28.1664506	32.0241275
48.9156064	48.3774209	50.2691042	56.3255044	55.2276364	52.7837775	37.8002155
2.05019737	2.16435852	1.46736556	1.1753812	1.00413884	1.96334509	3.18878099
0	0	0.04733437	0.06186217	0.09128535	0	0
14.5616582	14.5492989	15.6203431	13.7952636	12.5060929	13.5168758	7.57036213
0.02628458	0	0	0	0	0	0.01862354
4.8626476	4.16839418	5.82212789	4.91804239	5.75097702	7.80303816	2.19863434
12.380038	13.8278461	11.4075839	12.496158	7.9418254	10.4460027	3.6700153
41.4770698	42.8462825	35.6901173	43.4581733	47.011955	41.6833264	16.428133
0.39426872	0.68137213	0.28400624	0.52582843	0.04564267	0.22653982	1.06822398
19.2665983	17.5954331	18.9810836	15.6201975	17.116003	15.8577872	12.1769807
11.6440697	8.89791834	10.6975683	11.0423971	9.67624704	15.505392	15.217969
7.67509784	6.3327527	7.28949345	7.14508046	5.47712097	5.23558689	8.79516082
2.05019737	2.96597278	2.08271241	3.27869493	2.05392036	3.62463708	1.67926097
0.44683789	0.48096856	0.23667187	0.68048385	0.22821337	0.88098818	0.40791807
9.85671812	11.6634876	11.0289089	9.34118744	12.0953088	5.18524471	13.8195738
6.20316127	5.37081558	4.82810605	4.79431806	3.4232006	5.36144235	7.88797904
0	0.04008071	0.09466875	0.09279325	0.04564267	0.12585545	0
0	0.04008071	0	0	0	0	0

36.6144222	32.5054585	39.2401953	35.5088847	29.8503093	30.3311645	45.471963
117.045242	126.574892	131.447554	111.25911	121.637728	94.1398797	31.9063445
19.6082979	16.8739803	20.1644429	15.7439219	17.116003	15.8577872	19.5888461
5.17806258	6.69347911	6.01146538	4.26848962	4.79248085	5.78935089	9.59007981
17.0061243	15.511236	14.6736556	13.1766419	13.6928024	14.8761147	14.7384637
251.806292	277.118051	277.237423	250.356196	243.0016	212.897086	175.366313
0.07885374	0.08016143	0.09466875	0.06186217	0	0.02517109	0.06134221
1.02509868	1.28258282	1.13602495	1.33003662	1.04978152	2.06402945	0.89379054
41.8713386	43.1268475	37.0628141	49.1494928	48.2899499	42.4384591	70.6004023
0.65711454	0.64129141	1.04135621	0.83513927	1.32363757	0.98167254	0.48424009
25.1017755	29.499405	26.6019177	30.467118	31.3108749	28.7453857	30.5234914
116.677258	122.767225	115.49587	107.640173	110.090131	94.3160774	90.4783338
0.81482203	0.52104927	0.7100156	0.74234602	1.00413884	1.107528	1.84927605
2.49703526	1.44290568	2.08271241	2.47448674	1.82570699	3.54912381	1.99320487
1.76106697	2.00403566	1.51469994	1.67027855	2.19084839	2.81916217	1.59517756
0.52569163	0.6012107	0.66268122	0.64955277	0.63899745	1.23338345	0.60156299
0	0	0	0	0	0.02517109	0
0	0	0	0	0.04564267	0	0
43.7112593	50.2612144	41.9382545	44.2314504	43.8169677	32.9489579	14.2457279
61.821336	63.327527	77.4863687	60.0372345	61.5719682	61.5936593	56.8182795
93.8096719	97.7969404	105.934327	100.680679	93.3392698	72.8199658	27.2773925
16.5067173	18.597451	15.4310056	17.3214072	18.3939979	21.1688874	10.0459822
0	0	0	0	0.04564267	0	0
103.876667	89.179587	99.8281928	94.9893597	102.878589	58.2710753	39.6476745
67.2359599	73.7084317	66.0787848	85.1532749	72.6631382	74.7077976	25.1409933
53.3839853	58.2773571	60.6826663	51.0672201	50.8915823	51.7014206	61.2893882
1.20909076	1.04209854	0.80468434	1.14445012	0.5477121	0.57893509	0.80449438
4.02154099	5.05016987	5.49078728	4.36128288	3.92527003	3.72532144	2.46591072
10.6978247	11.8238104	10.1295558	10.4856376	12.6886636	15.22851	7.52097211
34.2225253	35.0305434	35.9741235	33.5911575	32.5432271	29.1732943	23.0528146
0	0	0.04733437	0	0.13692802	0.02517109	0
16.8221323	15.0703482	17.2770462	15.434611	15.7467228	11.7800705	12.5860958
33.0134345	39.2390183	38.9088547	32.9725358	34.232006	33.0496423	19.675931
55.1976215	45.8122553	52.6358228	46.2110398	47.148883	42.664999	32.2610786
7.64881326	6.09226842	7.33682782	7.88742648	7.48539866	8.7847107	8.68369432
0	0	0.04733437	0	0	0	0
0	0.04008071	0	0.09279325	0.04564267	0	0
139.571129	169.220771	150.759978	170.708654	178.143359	163.863801	127.891615
0.21027665	0.20040357	0.33134061	0.37117301	0.31949872	0.37756636	0.30732178
0	0.04008071	0	0	0.09128535	0.07551327	0
550.136294	557.723125	558.261596	560.28566	439.904099	456.88047	464.600364
0	0	0	0.03093108	0	0	0
0	0.04008071	0	0	0	0	0
0	0.04008071	0	0	0	0	0
2.81245024	2.92589207	2.31938428	3.03124625	3.28627258	4.10288781	4.85250911

5.54604673	5.21049272	5.77479351	7.29973588	5.47712097	7.24927416	5.86631846
0.02628458	0	0	0	0	0	0.09057336
0	0	0	0	0	0.02517109	0
11.7229234	7.89590051	8.47285278	10.3309821	10.8173139	13.013454	6.76118793
0	0	0	0	0	0.02517109	0
9.5938723	11.2225997	11.0762433	13.8571257	11.09117	11.2011354	6.38522455
7.12312163	6.73355983	5.49078728	7.51625347	7.34847063	8.25611779	1.86566958
0	0	0.04733437	0	0	0	0
46.4711404	37.1147405	32.7553862	43.334449	42.5846155	48.0012702	38.6717426
0.02628458	0.04008071	0	0.12372434	0	0.10068436	0.04835119
4.91521677	4.04815204	3.45540923	3.12403951	3.01241653	3.70015035	5.84041991
5.65118505	6.3327527	4.63876856	4.39221396	3.74269933	5.00904708	5.55931762
0.23656123	0.24048428	0.23667187	0.24744867	0.5477121	0.32722418	0.15900103
0.02628458	0	0	0	0	0	0.03363341
1.60335948	0.68137213	1.27802807	0.64955277	5.65969167	0.70479054	2.59097232
42.7912989	43.8082196	40.5655577	42.2518611	37.1074946	33.9306304	20.013751
0	0	0	0	0	0.02517109	0
0	0	0	0.03093108	0	0	0
0	0	0	0.03093108	0	0	0
1.15652159	1.64330924	1.51469994	2.07238264	1.55185094	2.03885836	3.53688959
0	0.04008071	0	0	0	0	0
6.12430752	6.89388268	6.91081847	5.81504383	4.1534834	6.56965471	4.1865187
24.4183764	20.3209216	21.4898054	23.5385551	23.5516202	26.4044743	20.1352479
47.3911007	40.5616818	38.9088547	42.8704827	47.5140244	50.5687215	12.4498734
274.752732	295.434937	280.929504	257.31569	260.802243	226.162251	298.889259
2.12905111	1.8036321	2.13004679	1.1753812	1.91699234	1.78714745	1.28139576
0.18399207	0.28056499	0.14200312	0.24744867	0.31949872	0.42790854	0.0566939
1.94505904	1.5230671	1.56203431	1.82493397	1.64313629	3.12121526	2.42174258
6.83399123	5.85178414	5.82212789	6.71204528	7.16589993	8.45748652	10.9351483
0	0	0	0.03093108	0	0	0
0	0	0	0	0.04564267	0	0
0.02628458	0	0	0	0	0	0.13538333
17.4266776	17.9160788	20.4484492	16.6718544	16.7965043	14.976799	23.6601533
6.59742999	5.53113843	5.86946226	6.65018311	6.43561714	7.92889361	24.9861887
1.55079032	1.36274425	1.2306937	1.94865831	1.32363757	2.31574036	3.94398952
2.44446609	1.96395495	2.22471553	2.72193541	2.09956304	3.59946599	2.95190462
0	0	0	0.03093108	0	0	0
7.04426788	6.73355983	6.48480911	6.4645966	9.95010309	8.43231543	4.85883799
0.02628458	0	0	0	0	0	0.05177704
0.7096837	0.56112999	0.33134061	0.64955277	1.14106687	0.65444836	0.23784812
58.5094787	51.5437973	51.3104604	57.9648518	58.7421224	65.2686385	93.9288501
0	0	0	0	0.04564267	0	0
67.4725211	76.3537588	71.4749033	65.6357607	67.4142306	61.6943436	38.282997
6.22944585	6.3327527	5.53812165	5.41293974	4.97505155	4.45528308	4.40994756
3.8638335	3.56718348	2.41405303	4.20662745	3.83398468	3.87634799	4.16254569

46.3922866	51.2632323	50.6004448	47.35549	46.7837416	40.449943	58.0870021
0.05256916	0.08016143	0.14200312	0.18558651	0.1825707	0.12585545	0.02983395
39.2954496	34.6297363	33.9387455	42.0972056	36.9249239	40.1982321	47.711707
13.8782591	18.1565631	17.135043	21.651759	15.9749362	18.2238698	17.6660458
0	0.16032285	0	0.03093108	0	0	0
0.15770749	0.20040357	0.09466875	0.09279325	0.22821337	0.47825073	5.43440141
1.81363613	1.60322853	1.13602495	2.13424481	2.00827769	1.86266072	0.55140907
0.07885374	0	0	0.15465542	0.04564267	0.07551327	0.04082125
2.18162028	2.48500422	1.46736556	2.10331373	1.00413884	1.661292	1.3444554
0	0	0	0	0.04564267	0.02517109	0
14.2462433	16.6735767	17.513718	13.1457108	15.7010801	13.8944421	18.4415022
0.13142291	0.44088785	0.18933749	0.21651759	0.45642675	0.52859291	0.06167954
1.41936741	0.64129141	1.42003119	0.55675952	0.86721082	1.00684363	4.18383434
0.39426872	0.64129141	0.61534685	0.68048385	0.63899745	0.90615927	1.31417745
38.1652126	57.8765499	67.877491	46.8605926	49.0658753	45.6100166	156.405168
2.44446609	2.28460066	3.02939988	2.13424481	1.91699234	1.93817399	14.6942476
30.3061226	26.0925443	31.0986831	32.4157763	28.344101	38.6627955	85.520262
6.88656039	7.01412482	7.85750593	7.26880479	4.38169677	6.69551016	23.2392494
4.1529639	4.24855561	4.26009358	3.27869493	3.74269933	3.27224181	9.89339744
48.442484	48.1369366	46.2930169	46.9843169	44.0908238	38.7634799	25.7479806
134.025082	120.362382	125.152082	139.437328	126.521494	173.202276	549.249277
0.18399207	0.16032285	0.47334373	0.27837976	0.63899745	0.40273745	0.78286884
0.10513833	0.04008071	0.14200312	0.18558651	0.1825707	0.12585545	0.74558937
7.67509784	6.41291412	5.72745914	7.57811564	6.93768656	9.56501452	5.76355689
22.2367561	20.0804373	17.6557212	22.6415537	21.8628412	22.4274419	17.3703041
55.1976215	56.6340478	54.0085197	53.263327	54.0865696	41.6078131	19.4813726
3.04901147	3.9279099	3.50274361	2.84565975	2.96677386	2.64296454	3.27032954
32.0671896	32.425297	29.9153238	36.0347131	32.9996538	38.788651	82.9330251
94.9924781	92.0253176	98.2661585	81.2559583	104.476082	68.5912225	17.0610629
0	0	0	0.03093108	0	0.02517109	0
5.57233131	7.57525481	7.10015596	4.76338697	5.61404899	5.81452198	11.2484337
25.1017755	25.5714951	25.0872177	22.2394496	25.9250392	19.6586219	14.794812
50.0195589	47.0948381	44.2103044	47.8813184	57.1902714	50.9462878	10.9961703
0	0	0	0	0	0.02517109	0
40.8462399	44.6899953	38.2935078	46.5512818	44.5472505	46.9692555	62.8115698
68.2610585	62.3655898	56.4699071	76.0595361	65.9080223	68.163314	79.450759
114.443068	112.065674	120.513314	109.619762	104.247869	90.9431512	93.9258775
4.57351721	3.72750633	2.55605615	3.43335035	2.82984583	1.9130029	5.97088181
7.14940621	5.731542	5.63279039	5.78411275	8.71775087	7.72752489	7.12485812
10.2509868	10.8618733	11.3129152	9.52677394	10.3152445	8.73436852	3.45023559
0	0	0	0.03093108	0	0.02517109	0
2.94387314	2.84573064	2.22471553	2.10331373	2.09956304	2.41642472	3.08321633
1.97134362	1.36274425	1.08869058	1.63934746	1.82570699	2.34091145	2.84376843
0	0	0	0	0	0.02517109	0
7.67509784	7.73557766	8.09417779	7.73277106	6.16176109	5.94037744	4.29105767

15.2976265	15.9521239	18.1763993	17.7235113	18.2570699	19.9355039	52.3450665
0.21027665	0.24048428	0.23667187	0.12372434	0.04564267	0.17619764	0.6942735
3.52213394	3.9279099	4.02342171	4.91804239	3.74269933	4.85802053	1.61490802
57.3266726	54.790335	46.8610293	63.2231362	50.4807983	65.5455205	26.3239018
24.129246	27.816015	26.1285739	28.4256664	25.7881112	32.8482735	46.192412
3.28557271	2.48500422	2.93473113	3.71173011	5.11197957	2.76881999	7.05740599
126.875676	141.244434	146.357882	111.939594	138.023448	117.498652	30.8221916
22.052764	25.2909301	23.856524	20.6928953	22.593124	19.9858461	23.9900591
155.394447	168.419157	181.763993	158.954842	145.326276	139.624041	180.181743
0.81482203	0.64129141	0.85201872	0.43303518	0.59335477	0.70479054	32.1535417
0	0	0	0	0.04564267	0	0
0	0	0	0.06186217	0	0	0
43.8426822	57.5158235	47.8550512	64.5841039	58.650837	47.8250726	19.6778967
0	0	0	0	0	0.02517109	0
1.39308283	1.88379352	0.80468434	0.77327711	1.23235222	1.08235691	0.698274
1.97134362	1.68338996	1.79870618	1.67027855	1.23235222	1.40958109	1.13723915
0.02628458	0	0	0	0	0	0.02168873
10.1721331	9.21856405	11.7862589	9.03187659	8.1243961	9.53984343	21.7352325
54.0673844	58.7984064	60.4459944	49.7371834	59.1072638	46.9944266	40.7390423
0	0	0	0.03093108	0	0	0
11.3286547	13.0663125	19.1704211	15.063438	11.7758101	9.46433015	7.78112098
0.10513833	0.20040357	0.09466875	0.43303518	0.13692802	0.15102655	0.08626034
20.0814204	24.048428	23.0045053	18.9298235	22.456196	16.2353536	9.78697444
0.34169956	0.44088785	0.56801248	0.61862168	0.45642675	0.85581709	0.12658875
306.872491	309.904075	302.277306	326.384801	355.191295	367.598611	245.756202
0	0	0	0	0	0.02517109	0
123.826664	118.598831	110.904436	130.003347	132.226829	139.775067	54.3506763
0.76225287	1.28258282	1.13602495	1.29910554	1.09542419	1.58577872	0.73769993
0	0	0.04733437	0	0	0	0
32.6191658	36.9143369	29.6786519	29.3535989	32.269371	28.1412796	11.7394955
0.73596829	0.9218564	1.04135621	1.51562313	1.09542419	0.85581709	0.39906878
15.7970336	13.1464739	16.0936868	17.2904761	15.7923655	18.1986987	10.1519719
23.5772697	22.5654416	19.5017617	25.3944202	24.7383297	23.7866808	9.01303965
44.736358	45.2110446	49.7010917	40.0866852	42.6759009	42.4384591	23.9707963
105.664018	123.809323	117.673251	100.278575	116.480106	92.4534166	52.3520606
11.2760855	13.9080075	13.8689713	12.4342959	11.5932394	9.64052779	7.06653145
0	0	0.04733437	0	0	0	0
0	0	0	0	0.04564267	0	0
7.38596744	7.33477053	6.81614972	7.73277106	12.5060929	7.02273434	2.69826645
149.243855	154.791715	169.173049	145.654476	139.803513	136.880392	114.606284
24.1029614	24.4492351	26.0812396	25.1469715	26.5640367	32.3700228	43.137963
0.05256916	0.16032285	0.04733437	0.12372434	0.27385605	0.10068436	0.09025556
0.05256916	0.08016143	0	0.12372434	0.04564267	0.10068436	0.08919152
0	0	0.14200312	0	0	0.02517109	0
0	0	0	0.03093108	0	0	0

0.21027665	0.12024214	0.23667187	0.03093108	0.22821337	0.05034218	0.30369343
4.20553306	4.56920131	3.21873737	5.19642215	4.47298212	5.23558689	5.36451165
0.44683789	0.40080713	0.42600936	0.40210409	0.68464012	0.80547491	0.68369007
52.648017	54.790335	55.1445446	52.0879458	79.9203235	56.0811904	69.2883744
3.91640267	3.84774847	3.40807486	4.60873155	3.788342	5.10973144	7.62271712
3.5747031	2.7254885	2.08271241	3.27869493	0.5477121	1.78714745	2.83493139
57.2215342	63.6882534	55.570554	53.0468094	54.6342817	51.9027893	15.6683291
248.783565	278.72128	326.55984	261.089282	268.105071	207.460131	222.30334
2.60217358	4.20847489	2.9820655	4.39221396	3.28627258	4.9335338	3.18160982
1.49822115	1.8036321	1.2306937	1.45376096	2.87548851	1.71163418	1.9292125
0	0	0	0	0	0.02517109	0
281.218739	305.13447	274.160689	273.647302	260.300174	228.20111	184.604638
19.9237129	22.2047151	26.649252	22.6724847	21.3607718	11.6038729	34.8425798
0.42055331	0.28056499	0.37867498	0.37117301	0.5477121	0.85581709	0.28509652
22.9201552	24.6897194	22.2944897	21.0640684	31.0826615	27.6881999	10.3537254
0.13142291	0.08016143	0.14200312	0.12372434	0.31949872	0.25171091	0.08042407
0.89367578	0.44088785	0.66268122	0.9897947	1.14106687	0.72996163	0.33710158
78.4069071	76.8347273	87.805262	84.3181356	84.9866603	98.8217026	67.640349
7.25454453	6.29267198	6.20080287	7.14508046	8.80903622	7.7023538	5.07107286
37.0875447	45.6920131	46.43502	38.6329242	62.9412485	49.4611935	36.6911826
14.6142274	11.743649	12.306937	16.1150949	13.5558744	14.3223507	17.2311992
38.7960425	28.5374678	29.5366488	25.0232471	50.800297	40.3996008	19.8405633
2.73359649	3.08621492	3.02939988	2.31983132	3.05805921	1.96334509	0.88045506
38.7960425	41.6037804	35.8321204	46.6131439	37.472636	48.1522968	45.9647724
0.26284582	0.32064571	0.37867498	0.37117301	1.14106687	0.20136873	0.08589166
0.10513833	0.04008071	0.09466875	0.03093108	0	0.05034218	0.04282143
7.91165908	6.09226842	6.48480911	7.11414937	8.4895375	8.86022397	8.24730809
2.575889	1.96395495	2.36671865	2.50541782	3.01241653	3.32258399	2.2661252
1.41936741	0.88177569	0.99402183	1.14445012	1.59749362	1.93817399	0.81420457
0.18399207	0.12024214	0.47334373	0.12372434	0.13692802	0.15102655	0.18779707
0.02628458	0	0	0	0	0	0.00874332
10.303556	8.73759549	9.46687461	9.55770503	11.1368126	11.8052416	15.3803396
0	0	0	0.03093108	0	0	0
0	0	0	0	0	0.02517109	0
60.3231149	68.3376161	67.0728066	62.1714793	57.3728421	57.1635473	44.6010143
0.86739119	0.88177569	0.7100156	0.43303518	0.63899745	0.70479054	0.78948428
2.1553357	1.72347067	1.84604055	2.31983132	1.82570699	3.5742949	4.60539811
0	0	0	0.06186217	0	0	0
72.124892	61.2032491	72.5162595	72.5333925	81.9286011	79.6665025	27.0642081
1.39308283	1.40282496	2.13004679	1.54655421	2.23649106	2.51710908	0.56587671
0	0	0	0	0.04564267	0.02517109	0
0.5782608	0.56112999	0.09466875	0.34024193	0.13692802	0.32722418	0.43882005
0	0.04008071	0	0.03093108	0.04564267	0.05034218	0
0	0	0	0	0	0.02517109	0
1.41936741	0.44088785	1.08869058	1.05165686	1.09542419	2.1898849	1.58782922

8.33221238	9.81977475	8.14151217	8.01115081	7.98746808	6.19208835	8.42717876
3.67984143	3.12629563	2.60339052	2.53634891	2.46470444	2.64296454	3.76694212
0	0	0	0	0.04564267	0	0
0.02628458	0	0	0	0	0	0.02912458
7.62252868	8.97807977	10.3188933	7.48532238	7.62232668	7.9540647	5.329824
0	0	0	0.03093108	0	0.02517109	0
4.81007844	5.731542	5.96413101	5.38200866	6.34433179	7.57649834	7.0132278
36.1150152	34.950382	36.7788079	34.2407102	31.3108749	29.7522294	31.646824
82.2970251	103.207837	79.9950905	85.6791033	69.1486522	75.362246	59.8886014
10.5926864	8.17646551	9.08819963	10.3619132	5.93354771	6.77102343	13.1953944
6.83399123	6.69347911	8.04684342	8.6297725	8.26132413	9.01125052	9.80061059
20.0814204	18.3970474	19.028418	19.1463411	16.2487922	15.7319318	31.0756557
116.361843	125.412552	129.12817	119.208399	115.475967	100.558508	65.1309673
4.28438681	2.84573064	4.44943107	3.15497059	3.01241653	3.17155745	6.63421176
8.96304234	7.69549695	7.05282159	8.41325491	5.43147829	5.56281107	8.04723502
1.23537534	1.44290568	1.7513718	1.48469204	1.59749362	2.59262236	0.88014936
0.21027665	0.24048428	0.28400624	0.15465542	0.22821337	0.05034218	0.16616817
11.8017772	9.89993618	12.2122683	13.4550216	12.1865942	14.0706398	13.8616805
26.8628424	25.6516565	23.7145209	27.7451826	28.6179571	29.0726099	7.28623494
0	0	0	0	0.04564267	0	0
36.6144222	34.5896555	35.2167736	35.6016779	30.2154507	27.7888843	31.5088875
6.38715334	5.49105772	4.78077168	6.55738986	6.02483306	8.15543343	7.05809452
0.73596829	0.48096856	0.04733437	0.37117301	0.27385605	0.17619764	0.64699904
0	0	0	0.03093108	0.04564267	0	0
0	0	0	0.03093108	0.04564267	0	0
0.07885374	0.12024214	0.14200312	0.06186217	0	0.07551327	0.08666723
1.68221323	1.60322853	1.56203431	2.19610698	1.41492292	2.66813563	0.93836145
0	0	0	0	0	0.02517109	0
0.49940705	0.24048428	0.56801248	0.55675952	0.77592547	0.70479054	0.29829946
4.59980179	4.56920131	5.06477792	6.52645877	4.47298212	6.84653671	3.22826724
52.7531554	52.0648465	58.9786288	60.9342359	80.1941795	52.4565533	17.2269509
9.14703441	9.45904833	7.3841622	9.68142936	9.85881774	11.4780174	1.8976397
18.7934759	16.5533346	16.7563681	18.8679614	19.6263501	20.7661499	44.9897892
0	0	0	0	0	0.02517109	0
0.02628458	0	0	0	0	0	0.03129298
0.02628458	0	0	0	0	0	0.01206286
0	0	0	0	0.04564267	0	0
0	0	0.04733437	0	0	0	0
0.02628458	0	0	0.03093108	0.04564267	0.05034218	0.02509055
0.23656123	0.24048428	0.18933749	0.12372434	0.04564267	0.15102655	0.1588265
0	0	0	0	0.04564267	0	0
0	0	0	0	0.04564267	0	0
0.55197621	0.28056499	0.23667187	0.12372434	0.45642675	0.17619764	0.49160857
3.25928812	3.12629563	2.36671865	2.81472866	3.46884328	4.48045417	1.52701615
4.41580972	5.21049272	4.9227748	3.9282477	3.6057713	4.27908544	3.09381157

0.05256916	0.08016143	0.04733437	0.09279325	0.13692802	0.17619764	0.05728916
0.05256916	0.04008071	0	0.12372434	0.13692802	0.02517109	0.03902129
40.6622478	39.3191797	38.1988391	31.2403951	43.1323276	27.3861468	11.6887625
0.07885374	0.16032285	0.04733437	0.03093108	0	0.05034218	0.05208457
9.46244939	8.89791834	7.24215908	11.2898457	7.43975598	13.4161914	13.9125902
0.13142291	0.12024214	0.42600936	0.15465542	0.04564267	0.10068436	0.56016623
0	0	0	0.03093108	0	0	0
0.34169956	0.16032285	0.23667187	0.18558651	0.1825707	0.05034218	0.38578315
70.2586867	74.6302881	78.0543812	68.7598002	63.5802459	65.8475736	59.1713714
42.5810223	46.9345152	43.9736326	42.0353435	40.9871219	33.6537484	18.3637361
39.1377421	47.7762102	41.5595795	36.7461281	40.7589085	37.8069784	32.8146837
0.13142291	0.04008071	0.18933749	0	0.04564267	0.10068436	0.07958797
91.2337829	106.574617	104.088286	89.6382821	97.4927532	87.293343	39.8375667
47.5750928	48.8183088	48.6597355	48.9329752	42.3564021	34.6857632	14.4107739
99.644849	109.821154	96.8934617	91.9581134	101.144167	78.8106854	40.1694106
3.10158063	3.24653777	3.78674985	3.9282477	3.65141398	4.6566518	2.20460077
68.6553273	61.2433299	74.0782938	68.1721096	61.3437548	52.5068955	37.4682359
0	0	0	0	0	0.05034218	0
3.49584936	3.08621492	3.97608734	3.80452336	4.24476875	5.03421817	21.2229332
0	0	0.04733437	0.03093108	0.04564267	0.07551327	0
6.07173836	7.8558198	5.20678104	4.7015248	4.38169677	6.84653671	4.24912364
81.3770648	87.5362778	90.8346619	84.7821019	80.0572515	67.0054438	37.346601
7.56995951	7.21452839	5.77479351	8.2276684	7.80489738	7.6771827	4.92433927
7.43853661	7.17444767	6.76881535	7.33066696	9.81317507	7.90372252	5.17455348
9.01561151	10.2606626	10.8869058	8.72256575	9.08289227	8.2309467	8.84770541
5.33577007	6.09226842	5.39611853	7.54718455	5.47712097	6.64516798	6.4914659
15.6393261	18.4772088	21.442471	18.4039951	12.8255916	16.5877489	8.50093637
0	0	0	0.03093108	0	0	0
0.76225287	0.44088785	0.75734997	0.34024193	1.78006431	0.90615927	0.31802616
6.0191692	4.60928202	6.29547162	7.4543913	6.20740376	6.99756325	9.93008907
0.18399207	0.04008071	0.09466875	0.06186217	0.09128535	0.02517109	3.72024449
0.34169956	0.12024214	0.33134061	0.03093108	0.13692802	0.25171091	6.01433154
0.47312247	0.68137213	0.42600936	0.18558651	0.45642675	0.42790854	8.63826493
0.31541498	0.20040357	0.23667187	0.27837976	0.31949872	0.57893509	12.4465323
0	0	0	0	0	0.02517109	0
0.13142291	0.08016143	0.09466875	0	0.04564267	0.07551327	2.21748563
0.18399207	0.36072642	0	0.09279325	0.04564267	0.07551327	8.3361034
0	0	0	0.03093108	0	0	0
0.02628458	0	0	0.03093108	0.13692802	0.02517109	1.4290463
0.02628458	0	0	0.06186217	0.04564267	0.02517109	1.3397309
0.07885374	0.04008071	0.09466875	0.03093108	0.04564267	0.02517109	3.11163307
0	0	0	0	0.04564267	0.02517109	0
0.07885374	0.04008071	0.09466875	0	0	0.05034218	3.2698517
0	0.04008071	0	0.03093108	0.04564267	0.07551327	0
0	0	0	0	0	0.02517109	0

0	0	0	0	0	0.02517109	0
0.02628458	0	0	0	0	0	0.9456924
0.52569163	0.36072642	0.66268122	0.74234602	0.50206942	0.90615927	16.7031385
5.07292426	5.21049272	5.53812165	5.07269781	7.25718528	6.39345707	11.3656292
0.21027665	0.04008071	0	0	0.04564267	0.05034218	0.07084228
0.13142291	0.08016143	0.04733437	0.21651759	0.13692802	0.17619764	0.13758469
0	0.04008071	0	0	0	0	0
10.618971	10.9821154	12.7802807	10.1453956	8.03311075	10.3956605	19.4170865
15.6656107	15.0302675	14.0109744	16.8265098	19.1242807	14.7250881	25.2151458
19.2140292	17.3549488	16.3776931	20.4145156	19.7176355	19.130029	15.3271855
58.4043404	53.7081558	56.0438977	54.0675352	50.9828677	43.0677364	22.8149991
91.417775	92.9070933	92.3493618	84.8748951	85.3974444	84.6000363	85.3664259
0	0	0	0.03093108	0	0.02517109	0
50.2824047	49.0988737	45.4883325	54.345915	50.4807983	60.0078805	35.6268319
210.539499	184.411362	219.063479	171.141689	166.458835	195.050783	228.021132
98.8037424	103.488402	101.248224	91.1229741	101.235453	80.8747149	43.0738286
2.33932777	2.52508494	1.9407093	1.88679614	1.91699234	1.30889672	2.1095947
0	0	0	0.03093108	0	0	0
0.15770749	0.04008071	0.09466875	0.03093108	0.13692802	0	0.1299133
3.52213394	3.64734491	3.78674985	3.3405571	2.32777641	3.27224181	1.17495898
0	0	0	0	0	0.02517109	0
0	0.04008071	0	0	0	0	0
2.73359649	3.20645706	2.60339052	2.84565975	3.87962735	4.25391435	6.06890805
0.21027665	0.24048428	0.28400624	0.24744867	0.5477121	0.37756636	0.34251443
0.68339912	0.84169498	0.89935309	0.9897947	0.63899745	1.86266072	0.43746315
0	0	0	0	0	0.05034218	0
0	0	0	0	0	0.02517109	0
0	0	0	0	0.04564267	0.02517109	0
0	0	0	0	0.04564267	0.02517109	0
0.15770749	0.16032285	0.47334373	0.21651759	0.09128535	0.12585545	0.33262284
0.78853745	0.72145284	0.47334373	0.61862168	0.68464012	1.40958109	1.21947693
102.877853	99.3200075	115.353867	99.536229	90.8289227	89.6342545	72.6191357
0	0	0	0.03093108	0	0	0
0	0	0	0	0	0.02517109	0
308.633558	290.865736	253.806908	271.234678	252.540919	223.569629	63.3202325
0	0	0	0.03093108	0	0	0
13.2211446	12.5452633	13.4902963	14.1664366	15.1990107	15.1529967	12.0247074
15.0084961	14.2286532	13.9636401	13.3622284	12.8712343	11.9814392	8.45678135
4.52094804	2.96597278	2.17738116	2.44355565	2.14520571	2.97018872	2.6486634
0	0	0	0	0.04564267	0	0
41.1090857	34.7499784	42.0802577	32.5395006	29.7133812	39.8458368	16.3114302
51.4389263	54.9105772	55.0025415	63.0375497	53.1280734	61.5936593	17.7652403
0	0	0	0.03093108	0	0.02517109	0
73.5968286	76.7946466	68.6348409	72.4715303	64.7213128	56.5090989	106.54428
9.90928728	9.69953261	13.3009588	12.0012607	6.80075853	7.60166943	10.8619043

17.2952547	28.65771	15.5256744	22.9817956	24.9665431	25.5738283	5.03439154
0	0	0.04733437	0	0	0	0
4.52094804	4.68944345	3.83408422	6.18621685	4.47298212	5.36144235	2.84559257
97.1215292	93.8289497	98.7868366	99.2887804	88.6380743	73.7261251	58.0539147
1457.66404	1498.21706	1694.19188	1536.25416	1355.45051	1183.29298	716.407779
91.3389212	104.610662	91.2133369	83.9778937	90.5550667	84.7007207	61.9881039
103.95552	121.644965	118.477936	105.413135	106.256147	100.407481	39.5174821
5.83517713	6.3327527	7.10015596	7.79463323	6.11611841	8.98607943	4.88408228
87.7642181	80.7225565	88.4206089	75.1006725	70.4266471	81.0257414	70.6087969
125.955715	123.488678	126.85612	113.578941	154.728667	163.007984	186.424406
32.040905	29.499405	35.6901173	31.3022572	26.4271087	27.3106336	27.8770749
47.1808241	56.7142093	55.0972102	50.3867362	48.1986645	44.4521464	17.3424301
66.8416911	61.4838141	51.8311385	74.8222927	60.0201173	65.7468893	32.1093487
374.686711	386.378076	407.690955	371.049286	386.730383	276.881999	207.962222
0.02628458	0	0.04733437	0.03093108	0.13692802	0.05034218	0.01637146
0	0	0	0	0	0.02517109	0
43.9215359	44.9304796	48.3757293	45.9945222	40.0286257	37.8069784	67.4558294
48.8367527	44.9705603	45.3463294	51.4693242	52.4890759	49.5367068	26.3991518
18.0837922	19.2788231	19.9277711	18.2802708	16.0662215	14.2971796	7.38618921
0.31541498	0.48096856	0.42600936	0.74234602	0.68464012	0.37756636	0.306833
61.6899131	62.2854284	56.1859008	62.5117212	71.430786	65.2434674	26.769905
16.4804327	15.1905903	13.5849651	15.5274043	17.8919285	17.6197636	19.3569569
7.80652075	6.89388268	7.52616532	8.25859949	7.89618273	9.81672543	6.39839322
28.3084944	31.3431178	26.6965864	28.0544934	25.3316845	24.6173268	17.813459
105.296034	118.478588	126.240773	99.7218155	114.928255	91.1948621	89.1398533
0	0	0	0	0	0.02517109	0
0	0	0	0.03093108	0	0	0
15.6130415	14.8298639	13.3482932	17.9090978	16.9334323	16.5877489	8.56850774
4.28438681	4.24855561	4.35476232	4.63966263	5.15762224	5.51246889	9.72361279
55.3553289	61.644137	59.2626351	55.6450205	57.0077007	42.8915388	52.6148864
31.8306284	38.156839	34.2227517	33.8076751	32.1780857	25.4479728	15.0078778
0.34169956	0.36072642	0.18933749	0.18558651	0.04564267	0.276882	0.61334709
1.44565199	1.24250211	1.08869058	1.7630718	2.05392036	1.33406781	1.14019651
0	0	0	0	0.04564267	0	0
78.4594762	65.972854	91.8760181	67.8009366	69.2855802	72.4172283	82.8470625
0.10513833	0.08016143	0	0.18558651	0.13692802	0.07551327	0.04468097
208.226456	216.596174	233.595131	211.939789	206.076676	177.657559	122.344072
95.9912922	100.442267	113.081817	102.010716	88.4555036	88.9042928	55.6515328
0.26284582	0.04008071	0.14200312	0.06186217	0.09128535	0.07551327	0.19037029
0	0	0.04733437	0.03093108	0.13692802	0.02517109	0
0.13142291	0.20040357	0.23667187	0.34024193	0.13692802	0.40273745	0.18650546
40.4256866	49.7802459	39.4295328	46.5512818	26.9748208	33.7292617	20.3339421
0	0.04008071	0	0	0	0	0
0	0.04008071	0	0	0	0	0
0.13142291	0.08016143	0.09466875	0	0.04564267	0.07551327	0.13492884

0.42055331	0.36072642	1.13602495	0.89700144	0.7302828	1.03201472	0.88699425
0.23656123	0.24048428	0.33134061	0.12372434	0.1825707	0.20136873	0.28095328
51.3863571	52.0648465	56.9432508	48.4999401	52.3521479	45.1317659	52.6026561
8.77905027	7.29468981	6.43747474	9.15560093	7.62232668	9.23779034	6.48116048
0.60454538	0.40080713	0.33134061	0.27837976	0.31949872	0.35239527	1.64157926
0	0.04008071	0	0	0	0	0
0	0	0	0	0	0.02517109	0
0.02628458	0	0	0	0	0	0.02203807
0.18399207	0.24048428	0.09466875	0.27837976	0.31949872	0.25171091	0.33245907
115.88872	121.324319	127.897476	110.300246	116.525749	106.14649	27.2677371
162.806699	199.601952	175.657858	173.770831	164.724413	92.9316674	55.9749964
0	0	0	0.03093108	0.04564267	0	0
0	0	0	0	0.04564267	0	0
1.15652159	1.08217926	0.80468434	1.39189879	1.50620827	1.33406781	0.67626952
0.05256916	0	0.09466875	0	0	0.02517109	0.02423938
73.8596744	72.826656	65.3214348	64.3366552	63.3063899	58.2459042	25.9406983
0	0.20040357	0.04733437	0.09279325	0.13692802	0.22653982	0
0	0	0	0	0	0.02517109	0
113.601962	118.999638	114.596517	121.559161	133.367896	126.233021	35.6876238
37.2452522	40.2811168	36.0687923	37.0554389	34.0494353	31.3631792	40.2487355
7.85908991	7.77565837	9.18286837	8.35139274	9.31110564	10.6977136	4.49878754
82.7701476	82.886915	68.5401722	82.7406503	91.2397068	91.5975995	29.4164738
83.690108	70.1412482	75.6876625	89.1124537	77.592547	91.2200332	74.4559103
8.06936657	7.01412482	5.68012477	9.68142936	7.66796935	7.72752489	9.52352851
51.9909025	48.4175016	49.7957605	47.2008345	42.5389728	42.8915388	28.648516
0.05256916	0	0	0	0.13692802	0.12585545	0.04618103
0	0	0	0.03093108	0	0	0
0	0	0	0.03093108	0	0	0
0.02628458	0	0	0.06186217	0.09128535	0.02517109	0.07291053
0	0	0.14200312	0.03093108	0	0	0
25.9428821	25.5714951	26.649252	28.4256664	30.2610933	27.5371734	11.4218268
26.573712	23.1265716	19.1704211	25.2088336	26.3358233	25.9513947	24.9959482
28.9393244	27.2949657	28.2112863	33.5911575	31.1739468	32.0679697	11.9315974
2.36561235	2.20443923	2.4613874	2.96938409	3.24062991	2.39125363	1.01626646
157.733774	142.206371	151.280656	118.68257	155.093809	130.260395	58.1097436
1.47193657	1.36274425	0.75734997	0.74234602	1.04978152	0.830646	1.84771507
0	0	0	0	0.04564267	0	0
1.91877446	1.40282496	1.04135621	1.02072578	1.14106687	1.25855454	2.73726944
996.763905	838.047634	731.789408	916.518957	1052.33751	1053.41015	2572.419
116.834965	127.937637	110.62043	116.981361	150.210043	130.537277	229.226131
5.62490047	4.04815204	4.68610293	4.79431806	5.24890759	7.90372252	3.3556976
16.7432785	18.1565631	16.7563681	16.1150949	21.8171985	20.237557	8.99705954
27.2308266	32.3451356	27.9272801	26.8791122	26.244538	24.7431823	14.3923392
0	0.04008071	0	0	0	0	0
8.27964322	8.41694978	7.81017156	8.25859949	8.99160692	10.7228847	4.96610229

0.02628458	0.04008071	0.09466875	0.12372434	0.09128535	0.07551327	0.03337161
0	0	0	0	0	0.02517109	0
0.02628458	0	0	0	0	0	0.01852163
23.5509852	23.6476208	21.5844741	25.2706958	24.6470444	28.5691881	15.170918
312.497391	335.355328	353.114423	279.957243	347.340755	211.940585	76.7463275
7.09683704	7.01412482	5.15944666	8.25859949	6.93768656	7.9540647	8.04956537
41.9501923	42.4855561	37.5361578	45.8707979	44.3190372	44.4018042	24.6479599
108.0822	113.628822	107.59103	116.177152	134.828461	117.926561	111.433092
0.07885374	0.04008071	0.04733437	0.09279325	0.13692802	0.15102655	0.12592771
235.588705	246.69679	192.88757	239.313799	260.208889	264.170598	122.4526
0.15770749	0.04008071	0.04733437	0.06186217	0	0.05034218	0.04614789
0.94624494	1.08217926	0.7100156	0.86607036	0.59335477	0.50342182	0.2177236
0	0	0	0	0	0.02517109	0
0	0	0	0	0	0.02517109	0
0.15770749	0.36072642	0.28400624	0.30931084	0.63899745	0.37756636	0.07893668
0	0	0	0	0	0.02517109	0
74.6744965	76.0731938	81.4151217	75.286259	75.5386267	54.7722937	36.9681149
0	0	0	0	0.04564267	0	0
0.10513833	0.08016143	0.14200312	0.09279325	0	0.05034218	0.23512645
0	0	0.09466875	0.12372434	0.1825707	0	0
0	0	0	0	0.04564267	0	0
3.65355685	3.40686063	5.25411541	3.55707469	5.52276364	5.96554853	2.07057785
0.02628458	0	0	0	0	0	0.02096059
11.3286547	11.6634876	10.3662277	9.86701587	9.67624704	9.71604106	20.7768763
22.368179	27.1346429	27.6432739	20.4145156	26.6096794	20.9423476	22.3551176
28.5187711	26.1727058	22.7678334	35.4779536	26.655322	26.3541321	24.6809995
149.795831	153.509132	146.736556	141.447848	127.708204	134.262599	96.9539863
22.3156098	24.7698808	23.9038584	32.3539141	24.7383297	24.5166425	16.1290144
0.10513833	0.12024214	0	0	0	0.07551327	0.0929629
11.0920935	10.3809047	10.4608964	9.89794695	8.58082285	9.31330361	11.9707054
9.38359565	6.3327527	8.14151217	8.07301298	10.1326738	9.86706761	20.6082843
11.3549393	11.8638911	11.549587	9.77422262	11.3193833	9.28813252	7.56347945
38.0863588	34.5495748	37.8674985	45.4377627	37.5182786	41.7588397	15.7347119
17.9260847	20.2808409	23.5251834	19.5793763	17.0247177	16.9401441	25.3511161
0	0	0.04733437	0	0	0	0
14.5616582	14.5492989	10.0822215	13.3003662	9.49367634	9.48950124	3.94530722
8.20078947	9.21856405	7.33682782	7.67090889	6.29868911	6.82136562	2.57294306
35.6156081	36.5936912	44.6363138	33.2199845	30.7631628	39.0151908	130.834982
0	0	0	0	0	0.02517109	0
55.0661986	56.2733214	61.3453475	50.8507025	51.9870065	51.827276	64.8643908
220.001948	234.231688	195.538295	235.385551	253.088631	232.027115	75.6607096
40.9513782	45.291206	41.1335702	44.5098302	50.8915823	48.4543499	20.8817082
0.07885374	0.28056499	0	0.06186217	0.04564267	0.02517109	0.05772629
0.86739119	0.44088785	0.47334373	0.21651759	0.13692802	0.72996163	0.62990019
21.9476257	21.6035044	24.0458615	23.3220375	26.0163246	27.8140554	12.4096174

4.07411016	4.5291206	5.11211229	4.17569637	2.55598978	4.37976981	2.505051
2.49703526	2.7254885	1.42003119	2.1651759	0.95849617	1.68646309	1.93450694
0	0	0	0	0.04564267	0	0
0	0	0.04733437	0	0.09128535	0.07551327	0
45.7088875	42.8462825	40.5182233	43.8602774	34.0494353	38.4362557	44.4475429
60.6385299	67.0149526	59.4046382	72.2859438	70.1984337	70.0511458	64.2513821
40.1102716	35.9123191	39.7608734	42.592103	41.534834	43.0425653	121.451249
11.9069155	8.7776762	7.81017156	11.9703296	9.99574577	13.1393094	9.26561984
3.33814187	2.96597278	2.74539364	3.18590168	3.4232006	4.50562526	2.80845928
106.767971	95.6726626	99.2601803	102.876786	113.832831	116.99523	44.3564905
0	0	0	0	0	0.02517109	0
3.33814187	2.68540779	2.08271241	2.56727999	1.46056559	2.01368727	6.45612616
8.27964322	8.13638479	8.33084966	7.14508046	6.48125981	8.02957798	10.8907157
31.0158063	30.4613421	30.8146769	32.3848452	24.7839724	25.7500259	33.8156677
0.26284582	0.68137213	0.18933749	0.37117301	0	0.25171091	0.1566555
72.0460383	73.6282703	72.2795877	70.522872	64.7213128	62.5753318	47.729682
30.7792451	26.693755	22.0578178	22.9508645	23.9624042	23.837023	49.8699302
20.107705	16.072366	20.7797898	21.0949994	22.9582654	22.2260732	34.8652862
16.0073102	10.901954	13.5849651	16.146026	14.4687279	17.0911707	48.0527776
47.312247	42.0045875	45.4883325	41.6023083	44.1821091	37.0518457	16.1440377
0.05256916	0.08016143	0.09466875	0	0.09128535	0	0.05419897
0.07885374	0.04008071	0.14200312	0	0.09128535	0.02517109	0.04769376
89.5778543	94.7908869	98.7395022	102.6912	78.9618273	69.1953287	199.962172
0.05256916	0.12024214	0.04733437	0.06186217	0	0	0.06153788
33.8545412	42.9264439	39.5242015	33.1271912	34.3232914	34.2075124	30.8827455
20.0551358	17.0743838	17.8450586	20.3217223	22.4105533	20.1872148	16.3065153
9.27845732	7.81573909	6.43747474	9.2174631	9.81317507	9.76638324	9.7217989
25.4697596	27.254885	28.3059551	26.074904	31.995515	36.1456864	9.29775646
3.31185729	2.64532708	2.17738116	3.68079902	3.24062991	3.62463708	8.86509027
0	0	0	0	0	0.02517109	0
0.23656123	0.12024214	0.04733437	0.15465542	0.04564267	0.02517109	0.15821863
4.57351721	3.84774847	3.02939988	5.50573299	3.33191526	5.26075798	5.67415441
56.6169889	50.6219408	56.6119102	58.397887	43.0866849	43.9990668	46.435621
13.0897217	13.9480882	14.1529775	12.5889513	11.228098	11.1759643	13.6044722
10.5926864	10.6614697	10.7449027	9.61956719	8.94596425	9.64052779	21.5068503
1.05138327	1.76355138	1.27802807	1.02072578	1.36928024	0.57893509	2.09640044
0	0	0.04733437	0	0	0	0
0.02628458	0.04008071	0	0.06186217	0.13692802	0.02517109	0.04541461
0.36798414	0.40080713	0.47334373	0.40210409	0.22821337	0.17619764	0.27431419
13.4051366	14.2687339	13.7743026	13.3312973	6.75511586	13.290336	17.0993809
40.4519712	39.559664	42.0329233	39.2515459	36.057713	36.2463708	43.4072813
0	0	0	0.03093108	0	0.02517109	0
8.91047318	8.09630408	8.42551841	8.10394407	7.21154261	7.04790543	7.60645543
80.0891203	74.8306917	67.498816	72.4405993	62.9868911	61.1909218	41.859364
34.616794	36.9944983	40.5655577	42.8395517	36.8336385	43.6466715	35.1566745

132.342869	117.837297	137.175013	116.702981	114.83697	117.523823	186.943513
21.7899182	23.5674594	20.8744585	23.7550727	22.5474813	30.482191	18.1885268
2.60217358	2.08419709	2.13004679	2.59821108	3.10370188	3.19672854	3.65465055
11.6177851	10.060259	10.0348871	11.6610188	13.0994476	12.6107165	15.8703131
0.52569163	0.48096856	0.5206781	0.43303518	0.86721082	1.00684363	0.94153856
17.3741085	17.5553524	14.1529775	15.9913705	11.8670954	14.0706398	4.14378847
0.02628458	0	0	0	0	0	0.02420289
0.02628458	0	0	0	0	0	0.0203439
0	0.04008071	0	0	0	0	0
0	0	0	0	0	0.02517109	0
0	0	0	0	0	0.05034218	0
0	0	0	0.03093108	0.04564267	0	0
0.15770749	0.24048428	0.18933749	0.27837976	0.27385605	0.30205309	0.56658223
85.1094754	77.3156959	90.2666494	94.2160826	87.6795782	101.28847	56.1408293
0.05256916	0.04008071	0.04733437	0.03093108	0.13692802	0.15102655	0.17148556
11.4600776	10.5412276	9.135534	14.0117812	11.8670954	11.0501089	5.70687734
0	0.04008071	0	0.03093108	0	0.12585545	0
18.4780609	17.7156753	16.3776931	19.6721696	19.0329954	21.7226514	26.68706
20.7385349	18.1966438	17.4190493	21.2496549	24.1906176	20.4137547	11.0758107
0.21027665	0.04008071	0	0.03093108	0.04564267	0.02517109	0.088562
27.1519728	25.5714951	26.9805926	28.7659083	27.3399622	31.4890346	65.967445
0	0	0	0	0	0.05034218	0
6.75513748	4.88984702	4.68610293	6.77390745	6.52690249	6.74585234	10.7597138
0.02628458	0	0	0	0	0	0.09057336
567.168703	578.324612	592.200341	678.040297	631.146906	642.013843	866.719703
2.68102733	2.68540779	2.4613874	3.5261436	2.87548851	3.39809726	2.56023517
0	0	0	0	0.04564267	0	0
0	0	0	0	0	0.02517109	0
55.7758823	52.9466222	54.1978572	46.0873155	46.6468136	53.3375415	74.6496887
31.9883359	30.9423106	32.8500549	30.4361869	30.0785226	25.0704065	10.9304079
334.445017	359.724402	327.506527	332.478224	312.424109	249.420339	167.501193
434.825834	467.180794	540.274534	440.860743	459.165308	343.081968	170.48591
0.07885374	0.16032285	0.04733437	0.03093108	0.04564267	0.05034218	0.01833852
0.02628458	0	0	0	0	0	0.05477605
49.8355668	51.9846851	53.0144978	48.6236644	47.5596671	45.2576213	25.2278564
44.420943	46.3333045	53.3458384	39.7155121	46.0991015	44.6786862	86.5967896
0.02628458	0	0	0	0	0	0.06136172
0.36798414	0.32064571	0.42600936	0.27837976	0.31949872	0.12585545	0.72023933
0.02628458	0	0	0	0	0	0.04646466
23.6561235	22.2047151	22.4838272	24.6520741	23.3690495	30.8345863	15.4915351
4.83636302	4.00807133	4.2127592	5.13455998	4.74683817	5.81452198	3.69304099
4.78379386	4.72952416	5.68012477	4.42314504	10.4065298	5.78935089	3.00407833
11.5389313	10.1404205	10.7449027	12.124985	12.0496661	13.3406781	10.1185697
57.2741034	63.2473655	68.3035003	61.0270292	74.6257732	77.602473	21.7754677
5.41462382	4.28863632	4.35476232	5.01083564	4.8837662	7.52615616	3.52508227

0	0	0.04733437	0	0	0	0
0.10513833	0.20040357	0.14200312	0.09279325	0.13692802	0.02517109	0.16757546
0.13142291	0.24048428	0.33134061	0.12372434	0.09128535	0.17619764	0.21725366
20.5282583	18.2367245	15.2416681	25.5490756	16.7965043	20.3382414	22.8810169
2.39189693	2.56516565	2.17738116	2.53634891	2.92113118	3.44843944	1.82987636
0.44683789	0.80161427	0.37867498	0.80420819	0.82156815	0.60410618	0.11329883
4.07411016	4.16839418	3.88141859	4.94897348	4.24476875	5.4872978	6.64063819
6.04545378	6.57323697	6.10613413	5.16549107	5.56840632	5.38661344	7.31123538
97.2003829	99.6005725	98.9288397	97.0617423	89.6422132	80.7236883	65.367673
1.81363613	2.96597278	1.37269682	1.73214072	1.50620827	1.38441	0.61244841
56.5118505	57.6360657	57.9372726	70.6465964	62.028395	60.5113024	15.8555309
36.7195606	29.9803735	45.2043263	37.3956808	39.709127	49.4611935	16.5445664
931.946127	835.241984	1012.1509	884.350629	836.584585	680.450099	579.581075
1.78735155	1.24250211	1.42003119	1.45376096	0.86721082	0.95650145	0.99090906
13.2211446	10.8618733	11.4075839	14.1973677	13.1450903	12.7617431	8.75648699
0.05256916	0	0.09466875	0.15465542	0	0.15102655	0.05271072
14.0359666	15.9922046	15.0523306	10.640293	13.190733	14.2720085	5.36981744
30.0432768	25.2508494	24.5665396	21.7445522	22.8213374	25.8255392	18.3619776
65.4223237	67.5360018	66.0314504	61.0270292	66.0449503	51.9783026	20.7466404
268.812417	215.193349	228.719691	272.16261	298.137951	230.416165	127.455144
9.40988023	10.0201783	11.4075839	8.84629009	8.03311075	8.75953961	20.3373992
0	0.04008071	0	0	0	0	0
0	0	0	0	0	0.05034218	0
0	0	0.04733437	0	0	0	0
0.34169956	0.44088785	0.33134061	0.21651759	0.1825707	0.30205309	0.45782699
8.67391194	7.61533552	5.77479351	8.84629009	9.26546297	8.83505288	6.69020729
0.21027665	0.20040357	0.18933749	0.24744867	0.59335477	0.276882	0.33801358
0.10513833	0.08016143	0.14200312	0	0	0.12585545	0.27511052
25.5223288	29.6196471	32.0453706	29.3535989	24.418831	21.6723092	16.0108149
32.8820116	33.1467499	26.5072489	30.467118	54.6342817	28.5943592	22.1620279
52.0171871	54.0288015	71.3802346	53.0468094	56.6425593	48.0516124	37.0383347
0	0	0	0.03093108	0	0.02517109	0
14.0622512	16.2326889	14.9103275	15.5274043	13.190733	10.5970292	7.69670908
9.62015688	10.621389	10.5082308	11.7228809	12.5517356	10.9997667	4.75578754
2.47075067	3.48702205	4.49676544	2.22703806	2.55598978	3.34775508	2.47334936
52.8057245	53.0668644	51.9731416	43.9221396	50.663369	43.9487246	14.7413202
42.0290461	43.0066053	48.6597355	40.1176162	44.7298212	36.3973973	22.5399005
55.7495977	49.7000844	50.4111073	50.6032538	42.8128289	43.9738957	65.7643799
2.07648195	2.24451994	2.65072489	2.07238264	1.32363757	1.83748963	1.59205879
0	0	0	0	0	0.02517109	0
10.8555322	9.3788869	9.60887773	11.1042592	10.2239591	13.290336	6.7067741
0.94624494	0.88177569	1.08869058	0.89700144	1.18670954	1.9130029	0.75828857
4.54723263	4.16839418	5.20678104	5.07269781	5.34019294	6.97239216	3.21907564
0	0	0	0	0.04564267	0	0
0.13142291	0.08016143	0	0.15465542	0.13692802	0.15102655	0.20519171

648.046361	676.121552	640.102727	699.166228	424.659446	560.560193	772.280146
0.52569163	0.96193712	0.85201872	0.86607036	0.82156815	1.28372563	0.74645483
164.567766	174.230861	171.01909	167.677408	149.160261	135.772864	99.487682
18.346638	18.597451	17.892393	14.7541272	16.7508616	17.0156574	7.89418646
34.3802328	34.1487677	40.5655577	33.5602264	27.1573915	36.7246215	66.023285
67.2096753	69.6201989	75.6403282	66.5946243	64.7213128	56.811152	40.9139617
23.4195622	22.365038	16.9457056	23.9097281	25.1947565	23.3084301	11.75818
0	0	0	0.03093108	0	0	0
39.3743033	39.840229	43.4056201	32.9106736	37.3813506	40.173061	40.1049171
106.951963	97.9572632	87.0952464	106.310136	106.98643	115.132569	224.605598
371.138293	440.607281	452.137931	353.109258	409.460435	363.873289	346.703328
150.715791	149.821706	157.8128	138.602188	146.102202	129.329065	96.1002909
20.107705	20.1605988	21.2057991	23.5694862	22.1366972	22.9308638	13.5822526
13.9833974	19.1585809	13.6322994	13.4859527	12.8712343	14.397864	24.6657306
38.7960425	40.401359	45.2043263	37.7977849	39.0244869	35.8184623	32.2738031
13.1685754	14.1084111	15.6676775	13.3312973	12.2778795	11.4528463	12.0126207
4.54723263	2.76556921	3.64474673	2.90752192	2.64727513	2.84433326	9.81917513
7.07055246	7.2546091	7.10015596	6.00063034	5.61404899	6.34311489	2.93594797
102.615007	117.035683	132.48891	108.289726	116.799605	77.0738801	99.9820205
103.745244	115.7531	116.537226	97.3710531	109.131635	97.5128059	62.0132074
56.0650127	57.5559042	64.9900942	56.7585396	68.3727267	73.0968478	13.2939532
0.13142291	0.16032285	0.23667187	0.12372434	0.13692802	0.05034218	0.27670862
18.3729226	20.1605988	26.6965864	17.3214072	17.6180724	19.7844774	33.620532
0.07885374	0.16032285	0	0.03093108	0	0.05034218	0.29956716
0	0	0	0	0.09128535	0	0
44.6049351	40.5216011	41.6542483	45.6852114	43.9995384	55.7791373	113.204482
4.52094804	5.69146128	6.48480911	4.79431806	4.47298212	4.80767835	11.1052393
0	0	0	0.03093108	0	0.02517109	0
72.440307	78.9590051	74.8356438	65.9141405	73.7129197	64.5135058	27.4946202
265.842259	264.372385	330.961936	290.752192	225.748669	236.205516	183.108626
0.10513833	0.08016143	0.28400624	0.06186217	0.09128535	0.05034218	0.02899981
0.15770749	0.52104927	0.23667187	0.06186217	0.31949872	0.07551327	0.12683843
15.8758873	15.230671	12.8276151	13.0529175	14.5143706	11.4780174	5.27379204
0.13142291	0.04008071	0.14200312	0.06186217	0.04564267	0.02517109	0.02023635
26.705135	28.2969836	24.3298678	23.2911064	19.2612087	26.0269079	191.601163
48.0482153	51.864443	51.3104604	47.757594	46.6924562	38.9145064	36.3267455
118.254333	134.831519	136.464998	111.382834	129.990338	99.2999533	89.9899123
0	0	0	0	0.04564267	0	0
0	0	0.04733437	0	0	0	0
22.1316177	20.7217288	18.5550742	20.3835845	16.4313629	17.2421972	24.8264852
215.586139	261.8473	250.25683	221.37377	216.254993	200.462567	124.987369
31.6203517	31.9443285	29.4893144	30.095945	28.5723144	24.113905	7.58594051
2.12905111	1.2024214	1.65670306	2.1651759	2.05392036	2.08920054	1.30810491
19.7397208	19.1585809	18.9337492	17.1976828	18.3027126	16.5877489	6.71131456
0	0	0	0	0.04564267	0	0

70.7843784	85.9330492	80.0897592	72.5333925	72.8000662	67.2068125	41.1351486
0	0	0.04733437	0	0	0	0
54.1462382	61.8445406	58.363282	54.2531217	53.9496415	47.4475062	34.221801
0	0	0.04733437	0	0	0	0
1.23537534	0.80161427	0.7100156	1.20631228	1.04978152	1.661292	1.3373597
30.8055297	31.3030371	26.3652458	37.6121984	28.344101	34.6605921	27.5870797
0.05256916	0.20040357	0.18933749	0.12372434	0.04564267	0.02517109	0.03518855
0	0	0.04733437	0	0	0	0
0	0.04008071	0	0	0	0	0
36.0887306	38.5576462	32.4240455	34.7665387	28.2984583	33.0244712	25.9839981
2.52331984	2.20443923	2.13004679	2.19610698	3.51448595	3.0205309	6.01117819
0	0	0	0.06186217	0	0	0
4.28438681	3.44694134	3.26607174	4.57780047	3.6057713	4.98387599	3.14681915
0.55197621	0.36072642	0.47334373	0.43303518	0.77592547	0.78030382	0.46327573
1.5770749	1.2024214	2.50872177	2.07238264	2.09956304	3.19672854	0.7871124
564.145976	488.784298	564.888408	395.546705	555.836493	402.234032	166.131792
28.7290477	32.3050549	31.3826893	28.7040462	25.7424685	27.3358046	14.6249776
0.02628458	0	0	0	0	0	0.03610729
0	0	0	0	0	0.02517109	0
0	0	0	0	0	0.02517109	0
0	0.08016143	0	0.12372434	0	0.10068436	0
1.20909076	1.36274425	1.60936868	0.89700144	1.27799489	0.830646	0.77722697
0.02628458	0	0	0	0	0	0.02512977
0.02628458	0	0	0	0	0	0.02512977
0	0	0.04733437	0	0	0	0
0	0	0	0.03093108	0	0	0
0.15770749	0.24048428	0.28400624	0.12372434	0.09128535	0.15102655	0.15970302
41.7399157	46.8142731	50.6477792	46.644075	43.5887544	32.5713915	18.9391039
89.7092772	95.1516133	96.3727836	82.7097192	94.2521233	69.8749482	19.0471297
35.247624	37.7560319	35.1221048	38.1689579	38.7506308	42.8915388	97.4415806
43.2118522	42.5256368	40.9915671	39.5917878	38.7962735	32.8482735	21.0055325
2.575889	2.84573064	2.74539364	2.38169349	3.74269933	4.30425653	1.83040783
1.49822115	0.88177569	1.65670306	1.1753812	0.91285349	1.00684363	2.55970932
0.34169956	0.32064571	0.28400624	0.21651759	0.09128535	0.25171091	0.57456501
0	0.04008071	0	0.03093108	0.04564267	0.05034218	0
327.63731	368.742562	371.385491	302.846246	365.734753	278.492949	111.409005
21.8687719	25.8520601	23.7618553	23.1673821	19.9458489	20.1117016	16.6418331
0	0.04008071	0	0	0	0	0
0.02628458	0	0	0	0	0	0.01816584
59.4820083	57.4757428	61.9606943	68.8216624	63.2607472	68.1381429	50.4776031
11.749208	12.4651018	12.306937	11.6300877	10.0413884	8.96090834	6.5508811
6.17687669	5.1303313	5.49078728	5.44387082	4.60991015	3.8511769	3.60156449
35.6156081	15.6314782	15.9990181	36.6224037	16.6139336	37.0518457	18.484535
5.36205466	4.48903989	2.79272801	4.36128288	2.87548851	2.4667669	1.35509193
3.96897183	3.88782919	4.44943107	5.04176673	4.60991015	4.70699399	3.97477265

0.07885374	0	0.09466875	0.03093108	0.04564267	0	0.04496999
3.52213394	3.84774847	3.83408422	4.1138342	4.29041142	4.9335338	1.9571086
18.7409067	18.3569667	18.4604055	18.1874775	14.6056559	16.4115512	13.3754231
36.5355685	43.2470896	42.9796107	34.2407102	36.9249239	38.0838604	25.9092307
43.1067139	41.5236189	41.2282389	37.9833714	36.7423532	36.5987661	36.3291825
0	0	0	0.03093108	0	0.02517109	0
0	0	0	0	0	0.02517109	0
0	0	0.04733437	0.03093108	0.13692802	0	0
5.75632338	6.37283341	4.97010917	6.65018311	6.75511586	6.72068125	6.54730416
20.5019737	20.1205181	25.1818865	17.9090978	19.6263501	18.7524627	19.2034935
1.18280617	1.40282496	1.42003119	1.29910554	0.77592547	0.93133036	0.76293666
0.34169956	0.16032285	0.5206781	0.06186217	0.22821337	0.32722418	0.46808067
16.0073102	16.7537381	16.8510368	14.8778515	15.153368	14.3475218	11.4144604
13.694267	12.6655054	11.4549183	13.8571257	7.07461458	10.2698051	28.6603853
0	0	0	0	0.09128535	0	0
9.3047419	5.93194556	9.79821522	9.86701587	12.5973782	8.86022397	6.71129349
14.9822115	15.6314782	14.4843182	15.7439219	16.6595763	18.1483565	11.9436421
24.5760838	23.0464101	23.2885115	25.4562823	28.8005278	26.0017368	7.92293095
0.23656123	0.44088785	0.28400624	0.27837976	0.09128535	0.17619764	0.05565036
0	0	0	0.06186217	0	0	0
0.34169956	0.48096856	0.09466875	0.12372434	0.31949872	0.17619764	0.21867436
14.955927	13.5873618	12.8749495	13.4240906	13.6928024	8.7847107	8.36742063
13.19486	13.5873618	13.6796338	16.1769571	14.3774425	15.6060763	5.05752089
0.13142291	0.12024214	0.14200312	0.37117301	0.13692802	0.15102655	0.08437035
146.799389	151.74558	157.623462	170.58493	171.616457	175.316648	75.0742184
65.0543396	66.533984	64.2800786	63.5633781	53.3106441	60.133736	70.8322347
0.15770749	0.12024214	0	0.09279325	0	0.02517109	0.76861056
1228.46249	1476.05243	1718.66375	1174.9791	1504.70206	927.680553	251.066758
680.139835	658.486038	0.28400624	491.494928	0.45642675	0.45307964	92.8680706
18.8723296	22.4852801	24.0931959	22.8580712	16.2487922	15.22851	9.15575766
0	0	0.04733437	0	0	0	0
0.07885374	0.08016143	0	0	0.09128535	0.40273745	0.11930813
8.80533485	9.17848334	10.4135621	7.70183997	9.53931902	5.43695562	6.23347018
0.86739119	0.9218564	0.89935309	0.77327711	0.86721082	0.45307964	0.87619065
36.4304302	36.1928841	37.0628141	32.8797425	35.1905022	32.1686541	42.1815511
0	0	0	0	0.04564267	0.02517109	0
1.18280617	0.84169498	0.89935309	1.42282987	1.46056559	1.20821236	0.94261197
35.2739086	33.5074763	33.8914111	34.1169859	30.9913761	25.6996837	25.1164452
36.3515764	33.3070727	35.3587767	32.3848452	29.4851679	32.5462204	30.9452666
48.8630373	52.7462186	61.8660256	46.8296615	67.4142306	76.6963138	25.9433307
39.7160029	42.3252332	39.0508578	32.7560182	40.9871219	33.2761821	19.822114
50.2824047	55.0308193	61.7713568	56.2636422	65.0864542	64.6897034	42.8788604
22.3418944	24.1285894	24.2825334	20.4454467	21.6802705	21.5464538	8.67772993
264.317753	245.374127	257.025646	258.119898	220.13462	190.066907	180.031189
402.890067	428.863632	434.340207	371.636977	444.377081	304.192633	108.199668

3.96897183	3.28661849	4.26009358	3.5261436	3.19498723	2.81916217	1.80691656
18.662053	16.5934153	16.8510368	18.1256154	19.0329954	21.6471381	17.5136284
33.5128416	42.6859596	40.3288858	39.4371324	35.783857	27.0840937	20.0419289
88.9995935	90.8228962	85.0598684	96.9689491	98.9989615	93.3092337	27.8302383
42.8964372	42.3653139	38.3881766	40.8599623	36.9705665	33.3768664	20.6227471
0.02628458	0.04008071	0.09466875	0.12372434	0.04564267	0.10068436	0.01955812
0.23656123	0.16032285	0.14200312	0.09279325	0.04564267	0.12585545	0.19408576
0.21027665	0.40080713	0.18933749	0.37117301	0.45642675	0.42790854	0.1528846
15.6918952	14.7096218	14.0583088	15.9604395	17.2985737	16.914973	6.25979598
5.49347756	5.69146128	4.16542483	5.53666408	3.28627258	3.82600581	2.05570211
15.5604723	16.5934153	18.9810836	15.063438	16.0205788	13.1393094	15.8162831
0	0	0	0	0.04564267	0	0
24.5235147	23.2868944	24.613874	23.1055199	21.6802705	19.9858461	6.85618887
5.07292426	4.64936274	4.44943107	4.91804239	5.65969167	6.56965471	4.84436655
169.850967	186.695962	197.621008	179.771462	165.272125	145.589589	87.6692769
74.7533502	80.9229601	72.4215908	69.1000422	72.8457089	63.4814911	19.5499033
43.6324055	43.8483003	38.3881766	48.9020442	49.248446	44.7793706	25.0762881
8.12193573	6.57323697	6.57947786	8.44418599	8.17003878	8.70919743	15.297066
24.2606689	26.693755	24.4245365	26.5388703	29.8503093	28.4181616	3.61064772
1319.85398	1524.34969	1573.48923	1285.65052	1521.8637	925.99409	354.886898
2.07648195	2.68540779	2.93473113	2.10331373	2.32777641	1.81231854	1.84938463
8.75276569	8.53719192	8.09417779	8.19673732	6.98332923	6.8717078	5.58681418
45.5248954	48.1369366	49.5117542	43.2725868	46.2816722	40.7016539	37.5648797
2.44446609	1.24250211	2.9820655	1.08258795	2.73856048	1.08235691	5.54268652
27.0205499	26.97432	21.9158147	26.0130418	23.1864788	18.2490409	8.84738781
30.4638301	35.9523998	33.4180674	31.6424992	31.995515	26.0269079	36.7152264
22.9464398	22.9662487	23.1465084	25.2706958	24.0536896	29.2488075	14.793171
23.2355702	26.8139972	23.6671865	23.6004173	19.9002062	21.7226514	14.757908
5.94031545	6.69347911	5.72745914	6.65018311	7.30282796	7.17376089	2.89452317
23.8401156	21.8840694	25.4658927	20.6001021	22.456196	20.0865305	16.2153252
2.12905111	3.28661849	3.31340611	2.22703806	2.55598978	2.21505599	2.08856205
17.0061243	17.0343031	17.4190493	19.0535479	19.078638	19.5579376	14.4367394
62.1104664	60.241312	59.8779819	56.8822639	47.9704511	60.2344204	43.5408705
4.20553306	4.56920131	4.87544043	4.73245589	5.24890759	6.09140398	2.76440982
20.7122503	19.4792266	19.028418	16.8265098	16.4313629	19.6334509	19.3412144
5.78260796	6.6533984	5.58545602	6.21714793	7.57668401	7.7023538	11.7212579
6.28201501	5.81170342	5.91679663	6.74297636	6.98332923	7.57649834	12.5259926
8.8841886	10.8618733	8.33084966	7.85649539	8.8546789	7.07307652	6.77338553
1.55079032	2.00403566	1.89337492	2.19610698	2.00827769	2.41642472	0.93015884
9.90928728	7.77565837	8.42551841	8.97001443	11.2737407	11.0249378	6.17361101
1.68221323	2.04411638	2.03537804	1.67027855	1.00413884	1.45992327	1.48848222
21.8950565	21.7237466	23.4305147	19.9814804	21.0869157	17.0156574	13.203796
64.6863554	74.9910145	74.6463063	62.8210321	67.5968013	57.5662847	39.2996603
49.4938672	54.7502543	50.7424479	47.9741116	47.011955	43.8983824	21.2812369
148.376463	126.174085	140.583088	133.034593	132.455042	101.263298	57.1404825

0	0	0.04733437	0	0	0	0
44.4735121	46.9345152	48.0443887	47.7885251	37.6552067	40.8275093	29.8675776
3.02272689	3.76758705	4.68610293	3.46428143	4.74683817	5.99071962	2.53959979
1.26165992	1.60322853	1.51469994	1.63934746	2.00827769	1.93817399	1.30572758
1.39308283	1.36274425	0.89935309	1.63934746	1.50620827	1.9130029	0.78713058
3.96897183	5.01008916	4.11809046	3.71173011	4.10784073	3.17155745	5.96093897
9.77786437	12.304779	9.75088085	11.2589147	11.1368126	14.5237194	5.31133104
15.0347807	14.5893796	16.3776931	13.4550216	12.9168769	13.5420469	9.9120592
3.75869518	3.56718348	2.79272801	4.20662745	3.69705665	4.63148071	3.66663195
7.96422824	6.53315626	4.68610293	7.29973588	8.39825215	7.72752489	15.841501
15.4027648	16.1926082	14.8629931	18.001891	16.979075	17.0911707	19.8441026
17.0061243	17.9561595	20.0697742	14.1664366	16.5226483	16.9904863	9.80364819
15.4290494	14.7497025	14.6736556	12.6817445	14.2405145	11.9310971	9.69146545
24.3658072	27.3751272	33.2760643	21.5898968	29.1656692	24.6173268	28.292675
9.14703441	9.73961332	8.14151217	8.87722117	6.52690249	6.69551016	3.5584139
6.7288529	9.01816048	9.08819963	6.58832094	6.34433179	8.07992016	7.93378956
3.41699561	3.12629563	4.02342171	3.89731661	3.69705665	5.13490253	1.20808105
3.99525641	3.16637635	3.6920811	3.12403951	2.78420316	3.0205309	4.93670539
33.1448575	38.1969197	36.7788079	38.4164066	39.2983429	41.6329842	29.6602897
14.8507886	14.5492989	16.1883556	16.7646477	15.6097948	20.5647812	13.0508269
10.9869551	12.5051825	14.9103275	11.9393985	16.2031495	17.3932238	9.21507057
16.6381402	21.2026973	23.8091897	17.7235113	19.1242807	15.5557341	12.5792286
1.36679825	1.76355138	1.27802807	0.86607036	1.41492292	0.98167254	1.92182088
2.68102733	2.48500422	2.50872177	1.73214072	2.28213374	2.13954272	1.9632812
36.6669914	38.0365969	38.7668515	36.4677483	34.8253608	32.0679697	16.4572338
38.5594813	37.1147405	39.5715359	36.715197	37.1074946	30.6583886	43.4739591
0.02628458	0	0	0	0	0	0.01593732
0	0	0	0	0	0.05034218	0
39.5320108	45.5316903	49.9377636	40.767169	40.5306952	38.0586893	11.7077658
27.8616565	25.5314143	25.0398834	29.7247719	26.1076099	30.6332175	16.7936705
1.91877446	1.64330924	1.84604055	0.86607036	1.87134966	1.45992327	1.91140761
72.8082912	75.8727902	84.4445215	73.6159805	73.0282796	64.0604262	51.9331256
41.2667932	43.1669282	45.1096575	34.5809522	40.8045512	42.111235	19.1071387
2.52331984	2.00403566	1.65670306	2.59821108	2.28213374	3.17155745	2.20402713
70.9420859	72.7064139	71.5222377	82.6787881	75.629912	78.0303816	23.6174742
6.15059211	7.09428625	4.9227748	7.39252913	8.44389483	6.44379925	3.49624942
0	0.04008071	0	0	0	0	0
4.17924848	3.76758705	4.30742795	4.33035179	6.29868911	4.45528308	4.47280239
0	0	0	0	0.04564267	0	0
0.02628458	0	0	0	0	0	0.0366631
2.83873482	3.40686063	3.07673425	5.35107757	3.46884328	2.99535981	1.28685659
24.0503922	27.6556921	28.4952926	24.2809011	26.4271087	20.9675187	21.9884085
157.339506	167.096494	171.445099	160.408603	157.786727	120.997434	36.1312372
46.2345791	49.0187123	36.2581298	44.7263478	44.4559652	22.8301794	39.1540878
115.494452	108.217926	115.401202	97.2473288	112.828692	98.7461893	67.518596

32.1986125	32.6257006	28.2586207	36.6224037	33.4104379	32.5462204	29.0794305
14.009682	14.6294603	13.7743026	12.8982621	12.5973782	12.45969	14.9675439
0	0.08016143	0.04733437	0	0	0.02517109	0
0.39426872	0.36072642	0.5206781	0.34024193	0.27385605	0.276882	0.35502622
26.705135	26.4532708	22.5311616	25.6109377	33.4560806	25.7248548	22.5841675
0	0.08016143	0	0.12372434	0.04564267	0.02517109	0
4.7312247	5.77162271	5.11211229	5.07269781	7.16589993	6.0410618	4.55540142
1.60335948	1.68338996	2.27204991	1.33003662	1.73442164	1.40958109	0.66792646
62.8464347	62.3655898	55.1445446	55.7378138	46.4642429	60.6371578	146.856004
1146.53345	1242.1013	1314.09686	1098.45559	1169.04583	1018.57336	922.115377
88.8681706	82.0051393	98.3134929	90.6280768	83.1153107	70.4790543	33.9298141
0.15770749	0.48096856	0.42600936	0.61862168	0.82156815	0.20136873	0.28665862
1.60335948	2.32468137	1.65670306	1.70120963	1.14106687	1.56060763	1.55294223
79.0114524	86.8148249	95.8047711	77.111193	75.356056	86.0347885	252.359129
0.07885374	0.36072642	0.28400624	0.34024193	0.27385605	0.40273745	0.06189325
132.474292	145.092182	143.612488	123.198508	142.176932	115.787018	99.6641144
0.18399207	0.16032285	0.18933749	0.03093108	0.04564267	0.20136873	0.19786795
73.9122436	66.8546297	71.9955814	70.0589058	62.9868911	61.2160929	39.8044284
6.46600709	7.77565837	8.85152776	6.71204528	4.79248085	7.90372252	5.64580389
118.75374	119.961575	132.48891	130.312658	130.081623	148.433923	284.842552
339.517941	380.40605	385.01779	351.377117	375.958712	245.795702	279.117808
139.439706	146.975976	149.245278	140.952951	134.372034	120.040932	51.5720449
117.229234	134.831519	98.7868366	128.116551	83.3891667	97.0345552	35.2475841
60.4019686	67.936809	54.7658696	60.222821	56.688202	48.5802053	20.0757611
6.62371458	5.65138057	6.01146538	6.40273443	6.70947319	7.92889361	7.21200935
4.1529639	4.24855561	4.73343731	4.94897348	5.29455027	4.98387599	5.67944057
13.9045437	15.0703482	13.7269682	12.0940539	13.5558744	12.5352032	6.70050169
49.888136	50.2612144	46.9083637	47.9431806	46.9206696	39.4682704	24.8280806
21.0013807	25.3710915	22.0104835	23.4457618	28.344101	24.1894183	8.35333435
119.016586	128.13804	129.45951	117.352534	116.388821	109.720785	102.9641
10.4086943	9.94001689	10.3662277	9.37211852	9.17417762	8.70919743	10.5273274
1.31422908	1.5230671	1.37269682	1.36096771	1.78006431	2.06402945	1.10531254
70.8369475	72.7064139	71.6642408	71.0796316	67.1403745	57.9941933	67.3562338
0.21027665	0.20040357	0.14200312	0.15465542	0.27385605	0.42790854	0.21863862
2.83873482	2.20443923	2.4613874	2.31983132	2.28213374	1.6361209	2.57896955
54.7770682	62.4457513	56.0438977	50.3558051	59.2898345	47.4726773	28.7464525
1.15652159	1.28258282	1.04135621	0.83513927	1.04978152	0.90615927	1.13498262
1.89248988	1.72347067	1.56203431	1.82493397	2.05392036	2.4667669	1.2493551
0.05256916	0.08016143	0	0.21651759	0	0.07551327	0.12319365
0.2891304	0.20040357	0.28400624	0.21651759	0.1825707	0.12585545	0.12690669
1900.82209	2271.57442	1717.29105	1767.2475	1823.92692	1565.84322	938.924964
0.05256916	0.12024214	0.04733437	0	0	0.07551327	0.08461458
7.56995951	7.33477053	9.37220587	6.12435468	8.39825215	6.56965471	4.2409984
6.83399123	5.65138057	5.53812165	6.12435468	7.71361203	7.12341871	12.2221065
1.20909076	1.12225997	0.7100156	1.33003662	1.41492292	1.20821236	1.13251372

3.94268725	4.04815204	3.73941547	4.1138342	4.83812352	4.75733617	1.91732509
9.72529521	10.5412276	8.28351529	7.67090889	7.30282796	9.76638324	203.364281
33.6442645	33.547557	28.8266332	35.6944712	33.8668646	36.0450021	15.6727088
93.7833873	109.821154	107.875036	96.226603	98.5881774	88.400871	30.8066157
8.0167974	9.21856405	8.80419339	8.01115081	7.21154261	7.62684052	11.5510368
0.02628458	0.04008071	0.18933749	0.06186217	0	0.02517109	0.03989273
23.3932777	31.1827949	27.5486051	27.0028365	24.7839724	21.7226514	17.6210912
0.86739119	1.04209854	0.56801248	0.95886361	0.95849617	1.28372563	1.14524218
0.63082996	0.9218564	0.66268122	0.55675952	0.59335477	0.52859291	1.45738433
3.52213394	3.64734491	4.02342171	3.77359228	4.42733945	4.88319162	5.59918725
4.57351721	4.80968559	3.6920811	5.44387082	3.56012863	6.64516798	7.06849811
2788.00558	3856.60631	4222.74676	3393.88229	3263.86203	2659.55229	1579.31288
0.10513833	0	0.04733437	0	0	0.05034218	0.40700686
1.94505904	1.48298639	1.65670306	0.80420819	1.64313629	1.58577872	1.98280174
39.8474258	37.5155476	34.9327673	39.7155121	43.8626104	39.6696392	38.855934
6.86027581	6.6533984	6.34280599	8.38232383	6.80075853	7.45064289	4.77364868
0.68339912	0.6012107	0.47334373	0.55675952	0.95849617	0.88098818	18.9998201
76.9349705	81.2035251	85.2492059	69.5949395	81.9742438	71.4103847	84.7105459
1.15652159	1.40282496	1.60936868	1.20631228	0.77592547	1.20821236	1.55467674
0.44683789	0.56112999	0.23667187	0.27837976	0.09128535	0.42790854	0.39197577
1.89248988	2.88581135	1.79870618	1.51562313	1.87134966	1.9130029	1.4883028
8.38478155	8.73759549	6.72148097	7.6399778	9.26546297	10.6222003	17.8227277
1.13023701	1.00201783	0.61534685	0.89700144	1.18670954	1.53543654	1.47320436
22.3156098	25.2508494	21.7264772	19.084479	24.1906176	18.8279759	33.3720744
15.9547411	15.791801	16.5670306	14.6304028	15.0620827	13.7182445	10.6564017
0.10513833	0.12024214	0.09466875	0.09279325	0.1825707	0.25171091	1.74985261
4.28438681	4.5291206	3.45540923	2.90752192	4.60991015	2.79399108	3.51628802
0.15770749	0.16032285	0	0	0	0.12585545	0.1242649
0.07885374	0.08016143	0.04733437	0.06186217	0.04564267	0	0.118502
212.931396	225.053205	243.819356	210.052993	217.669916	190.696184	196.882722
1.2879445	0.9218564	1.08869058	0.77327711	0.95849617	0.90615927	34.626891
0.31541498	0.48096856	0.23667187	0.34024193	0.68464012	0.47825073	0.18140221
9.64644146	8.05622337	7.95217467	8.2276684	10.5434579	10.7732269	4.4245781
0.34169956	0.36072642	0.09466875	0.21651759	0.13692802	0.17619764	0.39658068
16.900986	15.0703482	12.8749495	16.9502342	17.2072884	16.3108669	10.8785726
0.26284582	0.36072642	0.47334373	0.18558651	0.1825707	0.32722418	0.22430095
15.5604723	15.0302675	14.8629931	16.7955787	16.7508616	16.9653152	19.4332789
83.059278	87.0553092	82.4564779	91.7415958	91.8330616	90.3893872	46.822669
96.3855609	91.1034612	113.697164	84.0088248	89.0488584	99.3754666	78.5261654
0.05256916	0.04008071	0.18933749	0.06186217	0.1825707	0.22653982	0.23049134
30.8318143	28.4573064	23.1465084	40.7362379	21.2238437	33.6537484	63.2290099
35.7733156	30.7819878	31.71403	29.9103584	37.7921347	42.0860639	19.6678518
57.5895184	61.1230877	53.7245134	59.6041993	48.5638059	48.0012702	28.6608665
4.91521677	4.08823275	4.26009358	6.03156142	2.92113118	7.34995852	6.79018893
56.5644197	58.4777606	60.8246694	50.5413916	57.9661969	52.2300135	50.6362398

1.05138327	1.08217926	0.7100156	0.80420819	0.50206942	0.85581709	1.31507328
97.3318058	107.817119	121.507336	102.381889	98.1317507	95.9270272	97.4740605
17.1638318	16.7938189	19.7857679	13.3312973	18.2570699	16.1346692	41.9925254
84.820345	113.428419	91.0713338	97.2163977	77.8664031	85.6572221	67.5957518
38.8486117	33.4673956	27.3592676	40.0557541	35.0535742	36.2211997	56.1073608
36.7984143	37.7159512	41.2282389	36.2202996	36.6510678	32.6720759	17.3234398
11.2498009	10.2205819	10.3662277	11.4754322	10.4978152	14.0202976	17.0740891
0.15770749	0.08016143	0.23667187	0.12372434	0.13692802	0.02517109	0.131373
0.15770749	0.12024214	0.37867498	0.15465542	0	0.17619764	0.27000875
19.5294442	16.7136574	16.3303587	19.5175141	17.8462858	21.3702561	15.4378556
4.78379386	4.88984702	4.35476232	5.07269781	5.75097702	5.46212671	4.34765571
62.3995968	79.3197315	87.9472652	59.7897858	73.5759917	72.1151752	170.005586
41.5822082	43.1268475	47.7603824	46.8605926	48.1073792	49.3605091	76.5491391
0.07885374	0.04008071	0	0.15465542	0.1825707	0	0.03428492
9.96185644	10.3007433	9.08819963	10.3928443	7.98746808	16.2605247	15.4942051
0.05256916	0.04008071	0.18933749	0.27837976	0.09128535	0.10068436	0.06216248
1.02509868	1.96395495	0.80468434	1.39189879	0.3651414	0.90615927	0.85247323
0.2891304	0.40080713	0.28400624	0.55675952	0.50206942	0.32722418	0.1183302
55.6181748	55.0709	50.4111073	55.9852625	46.4186002	43.3949606	34.989403
91.6280516	105.612679	82.7404841	89.2980402	94.9367634	66.778904	91.1649014
8.83161943	9.0582412	7.81017156	9.58863611	9.58496169	9.94258088	10.7605478
51.9120488	52.7862994	49.8904292	53.1086716	46.3273149	40.6261406	40.6030977
262.530401	290.785575	261.61708	244.231841	286.362141	195.831087	100.847723
0.21027665	0.24048428	0.23667187	0.15465542	0.45642675	0.42790854	0.1388547
0.18399207	0.08016143	0.09466875	0.21651759	0.27385605	0.15102655	0.09891224
103.456113	90.1415241	110.762433	107.423656	91.6961335	72.0144909	57.6829262
1.47193657	1.04209854	1.2306937	0.77327711	1.41492292	0.830646	0.91169536
7.38596744	5.93194556	7.00548721	6.55738986	5.47712097	5.63832435	6.93410991
412.299948	431.388717	471.971034	414.105356	445.746361	303.739553	67.1406356
32.8031579	34.8702205	32.3767112	38.199889	34.5515048	36.6742793	35.2460431
1.13023701	1.16234068	0.94668746	0.64955277	0.7302828	1.107528	1.95007376
13.7994054	13.8679268	13.4429619	14.7850583	13.0994476	18.4504096	14.8990374
245.340285	232.347895	258.161671	233.560617	221.047474	214.558378	175.304415
3.23300354	2.76556921	3.26607174	3.74266119	2.64727513	4.70699399	3.11163307
67.2622444	72.546091	71.1908971	67.2441771	71.4764286	51.6007362	25.537217
1786.79958	1769.88414	1630.38515	1603.77672	1544.0004	1454.10875	155.936763
0.91996036	0.56112999	1.04135621	0.71141494	0.63899745	0.57893509	0.42140946
0	0.08016143	0.04733437	0.03093108	0	0	0
13.7994054	15.1104289	14.4369838	14.9087826	15.4728667	17.9973299	10.1172367
28.019364	26.97432	36.8261422	30.8692221	33.3191526	38.2600581	13.743254
6.91284497	6.0521877	6.72148097	8.53697925	7.34847063	6.69551016	24.1266233
1.91877446	2.92589207	1.2306937	2.53634891	2.46470444	2.61779345	3.9316726
101.116786	109.620751	119.897967	103.433546	104.886867	92.2520479	53.2134544
31.0683755	35.1908662	35.8794548	36.6533348	36.3315691	38.788651	83.9900249
30.2798381	29.0184364	25.4658927	32.694156	27.6594609	33.1754977	26.1403529

225.390288	231.185554	249.736152	220.105595	225.794312	195.428349	125.211907
0.7096837	0.48096856	1.18335933	0.92793253	1.09542419	1.03201472	2.36551942
0.02628458	0	0.09466875	0	0	0.02517109	0.02793531
0.02628458	0.08016143	0.04733437	0.12372434	0.1825707	0.02517109	0.03121703
1116.51646	891.354982	1110.27505	1247.07945	1013.54124	1281.08267	4023.02841
17.7946618	21.7237466	16.3303587	18.001891	15.7010801	16.0088138	14.0393084
9.01561151	8.49711121	9.6562121	8.69163467	10.3608872	11.8807549	12.7720496
24.1818151	29.0985978	28.2112863	25.1469715	26.7466074	21.6974803	32.3823299
50.5189659	51.1029094	42.2695951	51.9951526	52.5803613	53.5892524	63.5139847
3.1541498	2.44492351	4.68610293	3.64986794	4.3360541	4.37976981	5.57979031
0.02628458	0	0.09466875	0	0	0.02517109	0.07503744
4.41580972	3.40686063	3.64474673	3.49521252	2.46470444	3.59946599	3.22880753
14.4565199	13.5472811	17.2297118	14.8469204	17.3442164	18.6266072	29.9482607
16.2175869	14.8699446	13.1589557	17.3214072	9.35674832	11.1004511	6.05300846
69.1284497	65.6522083	74.9303126	74.0180846	75.8124827	78.9617119	230.419113
156.840099	149.941948	164.155606	154.810077	142.176932	129.933171	179.98141
63.4246955	65.3716433	63.0020505	63.5633781	60.5221867	50.4932082	18.4312854
0.26284582	0.24048428	0.37867498	0.27837976	0.63899745	0.37756636	0.12335907
0.73596829	0.44088785	0.66268122	0.89700144	0.68464012	0.830646	0.34401955
195.320726	222.808685	213.525357	193.535794	219.495623	152.05856	65.5059543
5.4409084	5.53113843	5.39611853	4.63966263	4.60991015	4.9335338	6.53488771
30.6478222	15.4711553	22.9098366	23.9406592	18.7591393	15.9333005	17.3730443
13.7731208	16.352931	12.9696182	17.9400289	14.1035865	16.4367223	8.79126316
85.9768666	94.7508061	86.8585746	84.3490667	89.5052851	70.4035411	24.7468788
5.94031545	6.37283341	6.20080287	4.60873155	5.65969167	5.78935089	7.65721857
27.1256883	27.9763379	30.2466644	26.1676973	25.2860418	26.3541321	25.9339234
0.10513833	0.08016143	0.28400624	0.21651759	0.1825707	0.276882	0.04929635
1.05138327	1.8036321	1.18335933	1.23724337	0.5477121	1.20821236	1.67031385
69.2598726	69.7805218	59.2153007	63.161274	60.3852587	57.5662847	36.4642059
41.4770698	38.1167583	38.6721828	36.3130929	35.2817876	36.7749637	49.8901561
0.02628458	0	0	0.12372434	0	0	0.02751694
32.2248971	33.8682027	28.9686363	31.7662235	31.995515	20.4389258	20.1483476
3.65355685	2.76556921	1.9407093	4.48500721	2.32777641	3.64980817	2.88437709
0.05256916	0.08016143	0.09466875	0.03093108	0.09128535	0	0.06492386
16.5330019	16.352931	19.8331023	19.3937898	24.9665431	15.8577872	8.38672101
27.4673878	24.9302036	21.6791429	25.8893175	27.7963889	28.267135	30.6993614
9.19960358	7.65541623	8.42551841	7.94928865	7.16589993	7.17376089	5.53825767
0.07885374	0.12024214	0.09466875	0.12372434	0.13692802	0.20136873	0.18303724
22.1053332	21.523343	23.2885115	25.1160404	25.4686125	23.0567192	9.74104055
0.26284582	0.36072642	1.04135621	0.12372434	0.13692802	0.67961945	0.09697947
1.52450574	0.44088785	0.42600936	1.1753812	1.27799489	1.03201472	54.0552295
20.5282583	21.3229395	21.9158147	22.7962091	22.9582654	24.2397605	14.4695569
6.17687669	6.45299483	5.96413101	5.93876817	7.43975598	8.00440689	3.98002755
0.02628458	0	0	0.12372434	0	0	0.07193186
81.9553256	85.0913543	85.2492059	89.5454888	87.5882928	96.5563044	193.541975

260.585342	263.210044	234.163144	242.592494	252.130135	184.554438	137.876389
97.9889204	89.3399099	64.4220817	94.2779447	55.1363511	69.4722107	84.6528273
8.27964322	9.01816048	8.23618091	10.8877416	6.70947319	12.0821236	11.6753494
0.23656123	0.36072642	0.47334373	0.55675952	0.22821337	0.62927727	0.46079916
0.34169956	0.40080713	0.37867498	0.5876906	0.59335477	0.37756636	0.16746636
5.86146171	6.89388268	7.62083406	5.47480191	6.75511586	5.41178453	7.98467683
1.97134362	2.16435852	2.22471553	2.22703806	2.14520571	3.27224181	2.13786846
25.8640283	25.8119793	27.1225958	28.1782177	30.6262347	27.5371734	19.9931027
2.68102733	1.76355138	2.13004679	2.56727999	3.01241653	2.51710908	1.30171115
6.22944585	6.81372125	6.34280599	4.33035179	6.20740376	6.16691725	5.56232801
0.10513833	0	0.18933749	0	0.09128535	0.05034218	0.22966815
18.7671913	16.5934153	17.135043	19.0535479	19.2155661	19.9355039	18.5516192
45.0254884	49.7802459	48.0443887	41.3548596	47.6053097	41.9350373	27.0193853
38.95375	47.5357259	42.884942	40.024823	42.0369034	35.742949	30.9224846
0.55197621	0.6012107	0.99402183	0.30931084	0.63899745	0.60410618	1.23894381
38.2966355	35.6718348	40.7075608	33.7148818	33.684294	37.1525301	98.1103879
26.6525658	29.2589207	34.3174205	29.755703	23.5972628	27.5623445	26.0101247
41.2667932	46.9745959	44.4943107	43.2416557	42.6302582	34.912303	27.2944366
13.5365595	13.5873618	13.8216369	12.4652269	12.2778795	12.0569525	12.2569015
9.27845732	9.3788869	9.94021834	10.0526024	7.43975598	7.4254718	3.93216706
0.05256916	0.24048428	0.14200312	0.03093108	0.04564267	0.12585545	0.04040659
13.510275	15.1505096	14.0583088	11.9703296	12.9168769	13.290336	13.9999326
54.8033527	54.8304157	53.1565009	62.9138253	54.6799243	60.1085649	33.7224016
0	0.12024214	0	0.03093108	0	0	0
71.2312163	61.7643791	55.7125571	72.1931506	70.9287165	64.8659011	82.0104451
0	0.12024214	0	0.03093108	0	0	0
0.18399207	0.48096856	0.14200312	0.34024193	0.3651414	0.45307964	0.14980019
36.5092839	39.5997447	30.95668	40.1485473	35.2361449	43.0173942	25.9960823
35.5893236	32.7860234	32.1400393	33.8695372	32.6801551	23.5853121	23.3624338
21.2642266	22.4451994	24.3772021	24.0643835	22.4105533	28.8964123	37.3470427
19.9237129	20.681648	22.8151678	20.6001021	23.3690495	26.3037899	39.3102977
29.780431	32.5054585	32.7080518	34.2097792	25.6055405	25.5486572	18.1876998
27.1256883	26.6135936	26.2705771	31.4569127	24.555759	32.2693384	21.0615392
6.49229167	6.3327527	6.29547162	3.40241926	5.38583562	7.12341871	2.30970621
0.02628458	0.04008071	0.04733437	0	0	0.02517109	0.01890829
41.1879394	39.8803097	42.8376076	36.6842659	42.2194741	34.2830257	20.7045818
39.4531571	39.8001483	34.506758	40.0866852	39.2070576	45.433819	12.8374694
0.44683789	0.24048428	0.47334373	0.27837976	0.1825707	0.32722418	0.25143064
0.18399207	0.24048428	0.28400624	0.12372434	0.1825707	0.15102655	0.09764633
194.873888	206.21527	214.661382	193.937898	196.263501	170.307601	164.347713
0.15770749	0.08016143	0	0.06186217	0.27385605	0.17619764	0.25201992
0.02628458	0.04008071	0.04733437	0	0	0.02517109	0.02331656
1.99762821	1.5230671	1.9407093	1.7630718	2.55598978	2.41642472	0.69759325
14.6667966	14.6695411	16.0936868	16.1769571	17.2072884	16.9653152	17.2267655
0.86739119	0.76153355	0.99402183	0.83513927	1.27799489	1.23338345	2.81077318

1.70849781	1.96395495	1.84604055	1.88679614	2.05392036	2.71847781	0.62340946
45.9980179	45.1709638	40.1868827	41.6332394	49.7961581	53.1613438	24.316637
1.26165992	2.32468137	1.56203431	1.29910554	1.59749362	1.18304127	0.13967149
27.8879411	25.6115758	26.6965864	30.1887382	26.655322	31.035955	117.032273
26.3108662	26.2528672	26.8859239	28.3947353	28.3897437	29.928427	24.7487084
17.7946618	17.5553524	19.7384336	16.146026	15.9292935	17.4183949	17.1942715
2.20790486	2.44492351	1.7513718	2.3507624	2.64727513	2.7436489	1.13865831
86.8968269	97.9572632	97.319471	89.0815226	95.0280488	72.870308	38.682536
7.22825995	6.09226842	6.86348409	7.60904672	7.16589993	8.15543343	3.54255768
19441.2857	18315.1224	18140.8985	18499.5412	16362.3968	12939.9544	8308.20559
50.7292426	48.5377438	55.1445446	47.6338697	43.7713251	49.4360224	118.202544
0.55197621	0.68137213	0.89935309	0.43303518	1.27799489	1.18304127	0.66426402
0.81482203	0.16032285	0.61534685	0.06186217	0.7302828	0.22653982	2.49501825
1473.46108	1861.3884	2486.85329	1837.83223	2257.39541	2759.50669	2042.45126
0.63082996	0.40080713	0.85201872	0.30931084	0.50206942	0.553764	0.1403574
25.4960442	27.6556921	28.4006238	22.7034158	26.7009647	24.8186956	11.945207
2.26047402	2.20443923	1.98804367	2.31983132	2.87548851	2.59262236	1.30835325
0.44683789	0.72145284	1.32536245	0.43303518	1.00413884	0.30205309	0.24266824
12.5377454	13.1464739	9.51420899	13.4240906	15.01644	11.7800705	6.32072507
28.1770715	29.9002121	25.8919021	25.3944202	28.3897437	22.5029552	30.5166859
5.99288462	5.21049272	6.81614972	5.876906	3.92527003	5.46212671	18.6302605
0.44683789	0.52104927	0.33134061	0.68048385	0.5477121	0.52859291	1.16053123
0.47312247	0.9218564	0.89935309	0.49489735	0.91285349	0.25171091	0.2567149
2.575889	1.84371281	2.60339052	2.69100433	2.41906176	3.34775508	3.54647956
0.18399207	0.08016143	0.18933749	0.27837976	0.31949872	0.15102655	0.21767388
3.4432802	3.36677991	3.36074049	2.31983132	2.32777641	3.70015035	2.49163795
6.25573043	7.73557766	6.10613413	7.17601154	7.80489738	7.92889361	2.18488021
0	0.04008071	0.09466875	0	0.04564267	0	0
52.0960408	47.4555645	54.434529	67.2441771	46.2360295	57.9690222	114.516298
36.4304302	43.5276546	42.6482701	35.2923671	39.3896283	37.1021879	44.2770082
19.2140292	15.7517203	17.892393	19.0535479	20.4479183	19.3313978	22.4063289
58.3517713	60.7222806	56.7539133	70.3682166	56.8707727	65.3693229	33.3010789
0.15770749	0.16032285	0.09466875	0.09279325	0.04564267	0.10068436	0.08020006
50.8869501	53.7883172	52.399151	51.6549107	49.5679448	41.8846951	24.3541693
102.641291	114.189952	108.159042	108.011346	101.281095	85.908933	41.2685556
0.13142291	0.16032285	0.09466875	0.03093108	0.13692802	0.07551327	0.09902538
7.22825995	6.53315626	7.19482471	5.876906	6.57254516	6.01589071	4.7951323
49.7304285	53.7883172	52.777826	50.4176673	46.6924562	45.9372408	26.2897584
0.34169956	0.12024214	0.09466875	0.06186217	0	0.22653982	12.1158273
233.144239	223.930945	221.477532	248.840573	246.561729	248.136613	64.9366837
24.4709455	20.681648	18.5550742	24.2190389	24.7383297	22.1253888	6.80107857
1.15652159	1.40282496	0.7100156	0.80420819	0.5477121	1.107528	1.00622748
7.85908991	8.61735335	6.81614972	8.47511708	5.65969167	5.91520635	10.8203815
48.3636302	47.3353224	55.949229	51.1909444	42.4933302	43.772527	31.6039085
0.10513833	0	0	0.03093108	0.04564267	0.20136873	4.35980226

7.88537449	10.3809047	9.75088085	11.5063633	7.39411331	13.7434156	20.458245
17.0586935	14.7497025	19.9751054	14.4448163	14.6056559	16.8646309	60.8386255
56.1964356	54.0688822	51.4524635	51.4693242	51.4849371	44.9555682	22.9568449
4.57351721	3.88782919	3.83408422	4.45407613	5.11197957	4.80767835	6.18200691
0.42055331	0.36072642	0.47334373	0.49489735	0.68464012	0.52859291	0.63123517
196.293256	255.755031	224.459597	216.301072	224.607602	161.623574	86.5774831
0.05256916	0.08016143	0.09466875	0.12372434	0.27385605	0.05034218	0.03185885
19.3717367	15.1505096	18.7444117	24.2809011	16.4313629	19.5831087	22.9957887
0.21027665	0.28056499	0.23667187	0.15465542	0.09128535	0.20136873	0.05246881
32.671735	27.816015	29.0159707	34.147917	30.443664	33.7796039	52.006314
44.5260813	50.8624251	47.4763762	47.3245589	42.8128289	40.5506273	17.7884061
67.5513748	71.7444767	64.0907411	64.5222417	64.8582408	56.8614942	33.811212
1.10395243	1.08217926	1.27802807	1.45376096	1.09542419	1.68646309	0.92750601
28.9130398	26.0123829	30.19933	30.5289801	26.0619673	18.8783181	8.25700849
0.02628458	0	0.09466875	0.03093108	0	0	0.98933974
0.02628458	0.08016143	0	0	0	0.02517109	0.04619762
1.20909076	0.72145284	1.2306937	1.02072578	1.68877897	1.38441	2.45487621
0.31541498	0.40080713	0.47334373	0.27837976	0.22821337	0.30205309	0.14193213
0.02628458	0.08016143	0	0	0	0.02517109	0.01809938
11.3812239	11.9039718	13.064287	9.52677394	13.6928024	8.55817088	6.75520793
80.4571044	81.9249779	81.6044592	77.8844701	71.6133567	75.6894701	131.580202
0.02628458	0.08016143	0	0	0	0.02517109	0.00854806
0.02628458	0.08016143	0	0	0	0.02517109	0.06055281
0.02628458	0.08016143	0	0	0	0.02517109	0.03942801
25.4697596	19.7197109	17.9870618	20.6310332	25.4229698	25.2466041	21.6066449
7.91165908	7.17444767	5.91679663	6.55738986	5.24890759	6.44379925	4.04523137
22.7624477	26.2929479	25.2765552	29.3535989	21.4976998	32.0931408	27.1790796
97.7260746	104.209854	107.023017	98.793883	97.0819692	86.462697	60.2099562
363.673472	364.373764	382.035725	367.646867	338.942503	295.458264	144.066193
0.2891304	0.40080713	0.23667187	0.43303518	0.22821337	0.60410618	0.19107993
0.10513833	0.16032285	0.18933749	0.06186217	0.13692802	0.07551327	0.12372695
0.13142291	0.36072642	0.33134061	0.18558651	0.09128535	0.20136873	0.23435526
52.14861	46.6138695	55.1445446	49.1185618	47.0575976	44.3011199	47.1664523
54.9610602	56.6741285	49.1804136	55.5831584	60.9786134	59.30309	25.9587087
1.76106697	2.08419709	1.18335933	1.63934746	2.28213374	2.36608254	2.67115597
80.9302269	69.2995532	67.9248253	79.0907824	58.6051944	58.4221018	55.0615989
992.794933	1013.12019	1004.5774	1234.42864	1049.8728	1086.63599	934.568236
0.05256916	0.12024214	0.09466875	0.03093108	0.04564267	0.05034218	0.0506754
11.3023701	10.901954	9.70354648	8.13487515	10.2239591	9.91740979	10.0333983
12.7743067	12.1043754	12.3542714	14.5685407	12.3235222	14.699917	7.45722799
4.07411016	5.01008916	3.73941547	3.64986794	4.10784073	3.22189963	2.42461638
95.9912922	91.8649948	109.389736	97.5875707	93.1566991	79.3392783	128.755191
620.789249	637.123018	624.482384	574.049992	610.653345	507.675731	345.732915
0.23656123	0.72145284	0.66268122	0.40210409	0.7302828	1.08235691	0.37315522
35.3790469	42.0446682	39.0508578	41.9734813	41.260978	43.772527	75.7945133

12.2223305	15.1905903	16.7090337	13.9808501	11.8670954	13.013454	20.3836358
12.879445	14.2687339	15.1943338	14.5066785	15.5185094	17.1415129	20.7989907
0.05256916	0.16032285	0.37867498	0.06186217	0.1825707	0.07551327	0.19253618
0.02628458	0.04008071	0	0.03093108	0	0.12585545	0.01625147
7.83280533	7.77565837	7.57349969	6.34087227	7.12025726	7.07307652	13.9472422
2.54960442	2.48500422	2.93473113	2.01052047	2.14520571	2.4667669	2.10309747
326.37565	337.359364	387.810519	319.332514	367.195318	247.708705	82.720507
29.9644231	27.3751272	34.0334142	31.0238775	32.4519417	37.2280433	24.6172179
10.4349789	9.94001689	10.1295558	11.5682255	9.58496169	13.2399938	9.83053989
5.70375422	6.97404411	8.33084966	7.23787371	6.34433179	4.07771672	2.97921373
4.81007844	5.09025058	6.01146538	4.36128288	3.69705665	5.36144235	6.3100248
2.41818151	1.84371281	1.42003119	2.3507624	1.55185094	3.24707072	1.62044691
1.15652159	0.64129141	0.42600936	0.92793253	0.82156815	1.28372563	0.6725723
55.223906	58.1571149	61.2033444	50.324874	59.8375466	49.058456	80.6621983
94.8873397	95.2718554	87.5685902	106.371999	99.1358895	97.7393457	31.7142856
1.34051366	1.5230671	1.27802807	1.79400289	1.41492292	1.81231854	0.83964702
9.98814103	11.743649	9.51420899	11.753812	11.1824553	12.1324658	7.36710632
0.9988141	0.72145284	1.08869058	1.45376096	0.91285349	1.15787018	2.79916285
0.07885374	0.12024214	0.23667187	0	0.04564267	0.15102655	0.07113615
29.5701544	26.0925443	32.5660487	23.5385551	26.244538	29.8277426	14.0667837
0.02628458	0.04008071	0.04733437	0.03093108	0	0	0.05965407
1.52450574	1.72347067	1.98804367	1.39189879	2.51034711	2.59262236	1.88564754
0.02628458	0.04008071	0.04733437	0.03093108	0	0	0.01637146
36.8509835	31.2228756	38.2461734	36.9007835	32.5888698	25.8003681	18.4675401
18.1100768	15.9922046	13.9163057	19.2391344	16.2031495	18.3748963	16.8919483
0.13142291	0.32064571	0.18933749	0.12372434	0.1825707	0.10068436	0.05288411
0.02628458	0.04008071	0.04733437	0.03093108	0	0	0.02815547
0.34169956	0.20040357	0.28400624	0.30931084	0.50206942	0.40273745	0.16502015
2.73359649	3.80766776	3.50274361	2.04145156	2.96677386	3.24707072	2.8605375
4.10039474	3.64734491	4.11809046	3.61893685	2.87548851	3.5742949	1.90865773
342.514383	331.748064	358.415873	339.932616	432.053559	373.488646	602.001152
0.23656123	0.12024214	0.04733437	0.06186217	0.04564267	0.12585545	0.29304494
16.270156	18.3569667	16.7563681	18.744237	18.7591393	19.2558845	15.3632129
1.18280617	1.44290568	1.13602495	1.14445012	1.27799489	0.553764	2.04944671
29.9381385	31.9042478	28.5899613	32.9725358	32.6345124	33.1754977	9.51367294
0.07885374	0.12024214	0	0.06186217	0	0.02517109	0.5076875
0.2891304	0.32064571	0.28400624	0.09279325	0.09128535	0.35239527	0.41931115
14.7456503	14.5092182	13.2536245	13.1457108	13.0081623	12.0821236	7.44454679
103.692675	99.5604917	78.7643968	96.1956719	81.9742438	77.602473	36.9005737
0.34169956	0.24048428	0.42600936	0.24744867	0.1825707	0.25171091	0.34488122
37.508098	40.8021661	41.5595795	35.2923671	40.0286257	34.3837101	32.9501644
52.2537483	42.0045875	46.9556981	40.2413406	66.5926624	53.211686	82.3727331
1.8662053	1.72347067	1.79870618	2.13424481	1.82570699	2.49193799	4.45444187
0.07885374	0.08016143	0.09466875	0.03093108	0.09128535	0.32722418	0.05662496
1.20909076	0.96193712	1.46736556	0.80420819	0.86721082	1.15787018	0.9840738

0.07885374	0.20040357	0.18933749	0.03093108	0.09128535	0.12585545	0.3482333
23.6035543	24.6496387	27.027927	25.2088336	28.3897437	28.8964123	46.4957817
143.172116	159.922046	165.054959	150.386932	149.47976	123.91728	54.4336726
71.4677775	83.9690943	75.7349969	82.4313395	84.5758763	83.9455879	140.668511
2.83873482	1.84371281	2.84006238	3.18590168	2.28213374	3.62463708	3.24237395
0.15770749	0.12024214	0.14200312	0.06186217	0.13692802	0.07551327	0.04393561
0.15770749	0.12024214	0.04733437	0.24744867	0.09128535	0.20136873	0.30768939
2.89130398	3.40686063	2.79272801	3.40241926	3.83398468	3.47361054	0.65992902
2.39189693	2.00403566	2.79272801	1.48469204	2.28213374	2.13954272	2.15779668
25.7851746	27.2949657	25.2292208	25.0232471	40.7132659	23.8621941	7.59057259
2.05019737	1.8036321	1.51469994	1.45376096	1.46056559	1.53543654	3.97145883
20.0551358	24.0083472	24.235199	19.6412385	23.0951934	18.6517783	4.92881011
2.78616565	2.16435852	2.84006238	2.87659083	1.55185094	1.86266072	1.17729721
1.20909076	0.76153355	0.99402183	0.95886361	0.7302828	0.65444836	0.78967588
11.5389313	8.61735335	12.9222838	8.815359	11.7758101	8.13026234	5.25517677
0.42055331	0.80161427	0.75734997	0.40210409	1.46056559	0.90615927	0.33979965
7.07055246	6.17242984	5.30144978	7.57811564	5.70533434	8.15543343	7.93878175
31.6466363	34.5495748	31.335355	32.0755343	39.8004124	35.4157248	11.2016389
26.4160046	33.7479606	31.0986831	30.5908423	26.2901806	25.5234861	23.6561562
54.3302303	50.6219408	57.4639289	56.8822639	39.2983429	49.7129044	51.0064241
2.20790486	1.48298639	2.27204991	1.54655421	1.78006431	1.58577872	4.75510123
271.335736	333.431454	304.549356	261.058351	313.245677	243.404448	87.9260956
6.51857625	6.73355983	6.34280599	5.72225058	5.34019294	6.09140398	14.4983242
8.25335864	8.37686907	8.18884654	9.46491177	8.6721082	9.69086997	10.8678279
23.498416	20.7618095	21.4898054	11.7847431	21.7259132	23.0567192	32.2256348
3.75869518	4.44895917	2.60339052	3.68079902	2.46470444	2.79399108	2.40856808
23.2618548	23.1265716	23.9985271	24.7757985	25.7424685	26.3793032	17.7516434
0.9988141	0.80161427	0.99402183	1.11351903	1.14106687	1.23338345	2.1212406
0.10513833	0.16032285	0.09466875	0.09279325	0.27385605	0.22653982	0.05674572
58.6146171	61.9647827	65.7947786	62.8210321	65.4059529	75.5132725	169.109429
39.7948566	44.8102374	54.1031884	48.0050427	51.8957212	53.1865149	26.9473911
39.269165	46.0928202	54.055854	42.8395517	48.837662	65.7217182	18.4475389
10.7503939	11.7035683	10.9342402	12.1559161	13.1450903	11.9814392	16.6255355
1.65592864	1.56314782	0.99402183	2.1651759	1.64313629	1.51026545	2.76731301
17.0586935	19.3589845	16.3303587	16.7027855	14.9707973	15.782274	25.9225448
128.873304	116.674956	127.897476	114.69246	118.488384	108.713941	72.382376
38.32292	33.8682027	44.2103044	36.2821618	27.3399622	39.9968633	16.7547762
1.05138327	2.04411638	1.89337492	1.7630718	0.82156815	4.25391435	2.89020599
0.21027665	0.24048428	0.33134061	0.30931084	0.31949872	0.42790854	0.62662201
0.05256916	0	0.04733437	0.06186217	0.04564267	0.10068436	0.19139013
1.26165992	1.8036321	1.60936868	1.97958939	1.87134966	1.78714745	1.40754218
0.05256916	0.04008071	0	0.03093108	0.09128535	0.10068436	0.05281896
1.55079032	1.32266354	1.18335933	1.29910554	0.86721082	3.24707072	0.48561601
2.39189693	3.24653777	2.84006238	2.47448674	2.05392036	2.4667669	0.47507262
0	0	0.14200312	0.09279325	0.04564267	0.10068436	0

1.5770749	2.28460066	2.27204991	1.94865831	1.18670954	1.71163418	0.45819083
25.443475	30.5815842	32.8973893	26.5698014	27.7507462	26.2786188	37.5901309
22.9464398	26.8540779	27.3592676	23.0127267	25.9250392	20.9171765	10.3426831
0.02628458	0.08016143	0	0.03093108	0	0	0.00955103
11.0132397	10.5412276	9.27753712	13.3622284	9.17417762	12.5100321	28.6645404
0.02628458	0.08016143	0	0.09279325	0.04564267	0.07551327	0.16363126
24.0503922	24.2488315	26.0812396	23.6622794	29.0743838	29.097781	14.5826472
4.94150135	5.25057344	4.9227748	7.57811564	5.15762224	4.9335338	6.45129758
7.80652075	6.93396339	7.10015596	7.6399778	7.9418254	9.08676379	5.28624516
5.38833924	5.21049272	6.95815284	4.88711131	5.38583562	4.88319162	8.80036855
0.86739119	0.64129141	0.66268122	0.52582843	0.63899745	0.553764	0.20647341
67.3148136	66.1331769	61.2980131	58.4597492	64.3561714	55.980506	15.5265806
1.23537534	0.64129141	0.42600936	0.71141494	0.27385605	0.67961945	0.27751657
14.7193657	15.4310746	13.8216369	13.3931595	14.7425839	11.2263065	7.08478589
3.54841852	5.17041201	2.55605615	2.90752192	2.92113118	3.44843944	5.60455536
1.41936741	1.24250211	0.75734997	0.9897947	0.91285349	0.85581709	0.93273771
20.9750962	19.7998724	18.5077399	22.7962091	21.4064144	21.2192296	29.0748173
0.10513833	0.12024214	0	0.09279325	0.09128535	0.22653982	0.10435227
0.05256916	0	0.18933749	0.06186217	0	0.02517109	0.04267225
44.8940655	42.6859596	40.9915671	46.4275574	36.2402837	61.9963967	27.8137499
141.910456	149.982029	153.079362	140.798295	146.92377	115.912873	83.3198808
55.1976215	58.9186485	56.0912321	62.3570658	58.3313383	64.4631636	27.0576788
316.229802	340.926547	375.408913	316.208474	346.701757	265.83189	159.029501
6.33458418	6.81372125	4.9227748	9.65049828	5.75097702	5.96554853	4.31819646
0.18399207	0	0.23667187	0.03093108	0.09128535	0.10068436	0.18756233
2.41818151	4.36879775	4.40209669	6.2790101	3.83398468	3.54912381	1.52953766
29.6227235	28.4973871	29.1106394	29.3226678	24.281903	25.9765657	35.3877358
0.55197621	0.44088785	0.7100156	0.40210409	0.27385605	0.52859291	16.4688872
0.05256916	0	0.04733437	0	0	0.02517109	0.13191197
8.5950582	7.69549695	8.09417779	8.35139274	8.4895375	10.7732269	7.9683275
0.63082996	0.48096856	0.37867498	0.52582843	0.09128535	0.40273745	0.17257855
73.9648128	78.7986823	80.9417779	79.8021973	62.9412485	71.1586738	154.534699
78.0914921	75.9529516	69.0135159	77.9154012	83.9368788	80.1699243	76.8529142
446.548758	755.441284	147.919916	84.8130329	676.60701	79.2134229	8882.21983
0.05256916	0.08016143	0.18933749	0.27837976	0.09128535	0.12585545	0.04148844
69.6804259	63.9287376	49.1804136	52.0570148	48.2443072	63.3052934	9.30862062
1.13023701	1.28258282	2.55605615	1.79400289	2.14520571	2.21505599	5.20754159
0.21027665	0.44088785	0.37867498	0.30931084	0.50206942	0.57893509	0.12167849
0.15770749	0.32064571	0.28400624	0.24744867	0.04564267	0.15102655	0.18200118
13.8519745	15.6715589	17.892393	15.1253002	14.9707973	11.9814392	6.32155063
1.05138327	0.80161427	0.61534685	1.33003662	0.63899745	1.23338345	21.9853276
52.8057245	55.3113843	77.7230406	76.5544335	55.0907084	78.433119	57.5726072
150.742076	146.214442	145.742535	139.77757	135.969528	129.505262	124.175462
34.616794	30.6216649	33.4654018	36.5296105	32.9540112	38.4614268	97.235854
13.19486	17.1144646	15.8096806	14.9706448	10.2239591	15.0019701	12.7799509

87.711649	103.688805	70.670219	113.486148	58.650837	46.4406626	63.8858997
0.39426872	0.76153355	0.66268122	0.30931084	0.45642675	0.52859291	1.42062776
37.8760822	39.0786954	39.0035234	42.6230341	31.5847309	56.7356387	14.0085423
36.7458451	35.7920769	38.3408422	36.2512307	46.9663123	38.7131377	35.9317756
7.62252868	7.65541623	6.81614972	8.44418599	6.98332923	9.64052779	3.50480251
42.2656073	43.888381	50.6477792	41.880688	41.3066206	42.1615771	20.7725573
0.5782608	0.48096856	0.7100156	0.55675952	0.41078407	0.35239527	2.25999335
1.13023701	1.5230671	1.32536245	0.92793253	1.96263501	2.1898849	0.68175655
30.9895218	37.4353862	36.6368048	31.0548086	33.684294	30.9856128	151.636103
2.44446609	2.04411638	2.69805926	1.29910554	2.32777641	2.24022708	3.27701849
16.6118556	17.5954331	16.6143649	19.3628587	20.0827769	16.9401441	13.0850215
0.10513833	0.28056499	0.14200312	0.30931084	0.3651414	0.15102655	0.59959984
8.33221238	10.7015504	9.08819963	10.2381889	11.0455273	10.3956605	3.25904803
188.51302	219.401824	261.569746	201.454152	160.57093	232.455024	89.8520167
3.31185729	4.36879775	3.02939988	3.58800577	3.788342	5.31110017	3.04727059
64.2658021	60.7222806	79.1904061	60.3156142	63.4433179	62.07191	60.1725292
161.124485	145.733473	129.080835	182.122224	145.600132	155.582512	123.88511
24.2080997	27.4953693	25.4185583	24.8995228	22.5018386	22.9056927	10.9801305
0.34169956	1.24250211	0.47334373	0.52582843	0.1825707	0.62927727	0.21419218
0.36798414	0.40080713	0.47334373	0.43303518	0.22821337	1.03201472	0.24959777
89.3938622	117.917458	108.774389	93.5974609	124.604502	41.3812733	35.3610979
298.198579	329.142817	316.903628	396.25812	288.416062	366.264543	403.296772
28.1770715	26.4532708	23.7618553	30.1887382	26.4727513	29.3243208	23.4560032
70.1272638	77.716503	75.2616532	68.295834	69.7876497	67.3074969	77.1800713
13.6679825	12.1444561	11.549587	13.3622284	15.4272241	13.2399938	11.9855496
51.9383333	63.1271234	49.7010917	42.1899989	49.2028034	56.8614942	25.9222352
3.78497976	3.88782919	3.31340611	3.9282477	4.38169677	4.48045417	2.82496034
6.25573043	5.41089629	6.05879975	4.63966263	7.43975598	8.86022397	4.91335019
12.9845833	12.1444561	11.2655808	13.9189879	12.6886636	13.9196132	10.7106201
4.99407051	0	5.39611853	0	3.51448595	0	6.80308788
13.9045437	10.340824	10.7449027	12.6198824	12.6886636	14.2468374	5.15274873
224.417758	234.432092	241.499971	215.435002	221.777757	203.483098	160.965663
0.89367578	0.9218564	1.08869058	1.23724337	0.77592547	1.56060763	1.31003046
24.5497993	35.3912698	33.8914111	31.6734302	28.344101	23.3839434	35.0629398
28.6764786	30.1406964	28.0692832	30.7454977	27.8876743	37.2783855	14.1936128
37.2978214	36.5135298	39.1928609	36.8698524	34.7797181	31.9169432	31.5204668
1.94505904	1.44290568	1.42003119	1.33003662	1.46056559	1.20821236	1.54403769
28.5976248	27.3350464	23.0518397	30.9620153	28.6179571	27.3861468	22.1481819
0.02628458	0	0.14200312	0.06186217	0.1825707	0.07551327	0.6495665
22.368179	18.6776124	21.1111304	21.1568616	18.5309259	16.2101825	17.0483888
0.36798414	0.48096856	0.33134061	0.43303518	0.7302828	0.47825073	0.57089357
18.9248988	21.4431816	19.6437648	19.0226168	21.6346278	26.1527634	11.3039795
8.64762736	9.45904833	6.91081847	8.2276684	7.43975598	6.29277271	6.03280024
1.26165992	0.96193712	0.99402183	0.83513927	0.95849617	0.830646	0.51609095
17.5055314	20.7217288	16.1410212	17.7235113	17.4355017	13.290336	611.835965

3.94268725	3.9279099	3.21873737	4.20662745	3.46884328	5.33627126	5.65088606
980.230903	1071.83843	1161.49085	952.862981	1029.92696	890.47768	570.865046
567.273841	402.971491	198.94637	134.36463	574.869488	126.837127	357.607697
5.67746964	7.13436696	5.58545602	7.20694262	6.89204388	6.84653671	5.24757462
20.8962424	19.3990652	25.1345521	20.7238264	15.5185094	21.6974803	13.6585977
3.39071103	2.56516565	2.55605615	3.18590168	3.37755793	3.5742949	1.85128627
10.6715401	12.4651018	18.3657367	13.5168838	14.2861572	20.3634125	36.3124838
4.28438681	3.52710277	4.49676544	3.68079902	3.37755793	3.49878163	3.01989472
137.783777	145.733473	160.652862	129.724967	134.828461	140.731569	127.49536
45.6037492	43.2470896	46.813695	44.819141	44.4559652	34.4088812	10.4137381
0.10513833	0.04008071	0.09466875	0.06186217	0	0.05034218	0.09082921
52.9108629	57.034855	49.3697511	56.0161935	58.4682663	59.0765502	48.4832055
129.71441	132.747322	121.460001	124.683201	123.509078	101.640865	65.4476091
748.663739	806.103305	885.815458	712.714043	809.244623	662.553453	474.892055
22.0001948	21.242778	24.5665396	19.0226168	21.8171985	20.6402945	41.8220892
26.5474275	25.0504458	25.2765552	25.1469715	20.5848463	24.1894183	17.9469893
34.1436716	35.5515927	37.9621672	33.3746399	34.5515048	30.7339019	19.6414064
1.60335948	1.36274425	0.66268122	0.92793253	1.23235222	0.75513273	2.85289606
0.05256916	0.04008071	0.14200312	0.09279325	0	0	0.10095304
39.1114575	43.2871703	37.9148328	36.5296105	36.6510678	37.404241	31.281118
0.23656123	0.40080713	0.09466875	0.21651759	0.13692802	0.12585545	0.65545159
13.694267	13.3468775	13.9636401	14.7541272	12.9625196	18.1735276	5.70960982
14.1673895	14.0683304	16.3776931	14.4757474	13.2363757	12.2331501	12.0268973
0.81482203	0.68137213	0.66268122	0.68048385	1.14106687	1.00684363	0.55054393
23.9715385	24.4893158	20.7797898	26.2914216	26.8835354	23.1574036	27.1016913
23.3144239	21.9642309	22.9571709	23.3220375	18.3483552	19.960675	25.3287668
8.27964322	10.3007433	8.94619651	7.6399778	8.58082285	8.15543343	13.1026722
0.05256916	0.40080713	0.14200312	0.12372434	0.1825707	0.02517109	0.05628629
66.9205449	73.7885931	71.3329002	69.3165598	83.3435241	78.6596589	128.311782
18.346638	19.3189038	19.2650898	17.7235113	17.2985737	16.638091	13.6932106
55.854736	50.461618	48.470398	56.7894706	50.1612995	62.902556	22.561095
123.958087	149.50106	123.732051	144.324439	143.409284	148.207383	310.411674
0.02628458	0.08016143	0	0.06186217	0.1825707	0.02517109	0.03620894
0.44683789	0.36072642	0.61534685	0.49489735	0.1825707	0.30205309	0.18001324
17.4003931	14.9901868	12.7329464	18.3112019	15.01644	17.3932238	14.2713004
130.529232	127.136022	116.395223	140.334329	123.646006	145.916814	116.892012
14.4565199	14.1484918	9.135534	13.207573	9.81317507	9.79155433	9.01348008
70.8632321	70.4618939	66.7888004	77.2349173	66.2731637	83.7442192	37.2122552
1.07766785	0.88177569	0.85201872	0.71141494	0.86721082	0.70479054	2.5086493
27.0205499	23.1666523	24.235199	25.5490756	26.3358233	29.8025715	16.4161117
0.07885374	0.28056499	0.09466875	0.09279325	0.04564267	0.10068436	0.11566022
94.0725177	71.0230239	100.77488	92.5767351	104.43044	97.4121215	47.4253145
28.2559253	28.09658	23.0991741	33.0962601	28.5723144	26.2031056	41.6195753
0.89367578	0.56112999	1.04135621	0.68048385	0.68464012	0.57893509	0.41805752
82.5335864	80.7225565	78.1490499	87.9680035	70.4266471	60.7126711	35.9872112

0.13142291	0	0.09466875	0.15465542	0.1825707	0.10068436	0.10660989
18.1100768	20.2006795	16.6143649	17.7853734	15.8380081	15.8829583	39.595693
20.291697	20.7217288	19.0757523	19.1154101	19.5807075	15.7067607	17.2618457
0.23656123	0.20040357	0.23667187	0.37117301	0.31949872	0.276882	0.18080717
49.2310214	54.2692858	44.9676544	46.0873155	53.3106441	33.6789195	15.4577987
127.690498	150.703482	142.523797	144.943061	122.18544	114.427779	62.1328184
5.07292426	5.41089629	4.30742795	4.73245589	3.56012863	4.4049409	10.0822641
67.7616515	69.4999568	73.2736095	66.1306581	68.4183694	58.9255236	30.9528867
25.7851746	29.1787593	23.2411772	29.2917368	30.03288	26.6813563	8.46213934
1.36679825	1.24250211	0.89935309	0.92793253	3.51448595	0.70479054	0.47907856
0.63082996	0.56112999	0.28400624	0.74234602	0.91285349	0.45307964	19.5363291
0.18399207	0.20040357	0.09466875	0.27837976	0.22821337	0.22653982	0.20927456
62.9778576	79.4800544	61.7713568	67.3060393	66.090593	50.3925239	22.421387
9.22588816	9.17848334	11.0289089	9.09373876	8.6721082	8.30645998	4.92618644
56.9586884	66.0530155	59.6886444	60.3774764	68.6465828	70.4538832	45.5850342
20.6859658	21.0423745	26.7912552	18.4967884	24.2362603	18.4000674	19.0190434
31.6466363	35.5115119	38.2461734	32.5085695	32.3606564	31.4135214	33.8251325
13.3525675	18.0764017	13.7743026	13.3003662	13.190733	13.7182445	8.89409157
62.6098735	64.1291412	67.5934847	64.6768971	60.0657599	53.4130548	61.9658061
44.7889271	48.5377438	52.2098135	47.1080413	45.4144614	41.1295624	31.5699424
63.7926797	57.1150164	59.7359788	57.5318167	56.7794874	51.8524471	21.2084918
188.959857	225.614335	195.064951	196.164936	207.172101	143.550731	45.9272424
102.1156	106.574617	100.77488	93.659323	104.065298	85.7830776	69.5139173
4.54723263	3.24653777	26.2705771	4.14476529	9.31110564	9.38881688	4.10672773
0.02628458	0.08016143	0	0	0.04564267	0	0.04122249
29.3598777	31.0224721	36.2581298	30.2506004	27.6138182	29.8277426	49.8826473
0.49940705	0.24048428	0.09466875	0.61862168	0.3651414	0.32722418	0.44495069
3.33814187	3.16637635	3.64474673	2.38169349	3.14934456	3.09604417	18.1893087
0.26284582	0.52104927	0.37867498	0.15465542	0.27385605	0.35239527	0.28057192
59.6660003	61.1230877	60.2093225	64.3057241	63.99103	67.8612609	33.4579599
42.5547377	48.8984702	45.0149888	49.8918389	53.4932148	45.2324502	46.5830729
233.117955	184.812169	152.369347	229.415852	240.308682	174.863568	50.0825011
1319.77513	1386.07123	1386.23445	1291.58928	1204.96661	1171.89048	536.811798
33.9859641	34.7499784	34.4120892	34.6118832	33.0909392	26.0772501	23.0393624
0.94624494	0.64129141	0.42600936	0.5876906	0.3651414	0.57893509	1.15694903
78.3543379	83.0472379	86.9532433	77.0493308	75.4016987	77.1997356	30.652289
10.0407102	10.7416312	10.0348871	10.6712241	13.053805	10.9242534	16.871776
0.73596829	0.52104927	0.75734997	1.14445012	0.5477121	0.88098818	0.3527127
129.320142	140.082093	147.351903	162.480985	147.060698	146.722288	47.4064804
32.4614583	33.1467499	30.2466644	38.4164066	28.0246023	39.6444681	122.183458
3.23300354	3.56718348	3.21873737	4.67059372	3.10370188	3.92669017	2.32298715
20.7385349	17.8759981	20.8271241	20.2598602	21.4520571	23.9377074	24.0579842
42.0816152	43.6879775	46.1510137	40.8599623	41.8086901	38.9648486	29.9987297
71.3363546	75.3517409	72.7529314	68.295834	74.0324184	58.6989838	54.6429005
14.2725278	13.6675232	19.1230867	16.888372	15.7010801	20.237557	4.24079989

4.36324055	3.24653777	3.55007798	4.14476529	5.24890759	3.87634799	2.71351699
21.737349	21.0423745	18.7917461	25.8583864	20.2653476	22.1002178	6.87460677
41.1616549	42.9665246	42.4589326	39.8083054	40.9414792	35.8436333	40.427496
0.60454538	0.56112999	0.80468434	0.80420819	0.59335477	1.05718582	0.76794544
19.7134362	23.487298	22.6731647	20.1670669	20.2197049	19.3313978	16.3603502
19.7660054	21.5634237	21.4898054	16.5790611	18.7591393	21.2695718	50.0092313
10.9869551	9.0582412	9.23020275	11.382639	11.4563114	10.2194629	15.2729323
41.7399157	40.4815204	41.748917	42.8086206	43.5431117	48.0516124	52.6118745
0.60454538	0.56112999	0.47334373	0.46396626	0.45642675	0.35239527	0.76954366
10.9606705	12.2646983	12.0702651	12.2487094	13.6015171	13.3406781	13.2818493
48.1796382	49.058793	49.7484261	45.1284519	45.7796028	45.1065948	48.3887044
0.05256916	0.04008071	0	0.03093108	0	0	0.2194781
0.05256916	0.04008071	0	0.03093108	0	0	0.03242102
356.234935	302.048255	302.561313	318.435512	358.431925	385.621112	121.708407
18.4780609	10.9821154	20.306446	9.49584286	17.8919285	14.5488905	19.6984225
0.13142291	0.28056499	0.04733437	0.27837976	0.09128535	0.32722418	0.4014175
1.99762821	1.48298639	4.40209669	1.94865831	1.55185094	2.4667669	2.65616214
24.8126451	21.8840694	24.8032115	24.5592809	23.9624042	30.1046246	8.02033118
0.05256916	0.04008071	0	0.03093108	0	0	0.10853516
41.9239077	39.7199868	48.470398	45.0975208	43.8626104	53.3123704	95.325091
0.60454538	0.6012107	0.89935309	0.68048385	1.00413884	0.98167254	0.7569411
54.7770682	53.5077522	48.0443887	52.5828432	50.800297	38.1090315	13.5083118
1.02509868	1.00201783	0.99402183	1.14445012	0.95849617	1.61094981	0.93826272
0.10513833	0.08016143	0	0	0.04564267	0.05034218	0.072725
0.10513833	0.08016143	0.14200312	0.09279325	0.09128535	0.32722418	0.12734076
68.0507819	60.4817963	64.6587536	65.728554	74.0780611	70.4538832	43.929034
0.76225287	0.52104927	0.33134061	0.37117301	0.5477121	0.35239527	3.98484064
15.9810256	14.6294603	10.1768902	15.7129908	14.3774425	16.2856958	51.4456667
64.4760788	65.2113205	51.8784729	71.3889424	72.0697834	57.2390606	14.3144533
17.7420926	17.0343031	19.3124242	16.0841638	17.4811444	15.4298787	12.9690114
12.7480221	11.6634876	11.0762433	12.4342959	12.8255916	14.2971796	23.9546355
3.60098769	3.84774847	5.15944666	3.09310842	3.87962735	3.70015035	2.53819373
1.34051366	0.64129141	0.75734997	0.49489735	0.27385605	1.15787018	0.83409493
0.21027665	0.24048428	0.42600936	0.12372434	0.13692802	0.276882	0.41893865
13.5365595	16.7938189	15.7150119	14.5066785	15.01644	11.5031885	6.51034951
97.9889204	113.628822	117.862589	112.712871	105.754077	77.1493934	31.3340487
0.5782608	0.36072642	0.37867498	0.5876906	1.00413884	0.35239527	0.50817379
0.02628458	0.08016143	0.04733437	0.18558651	0.09128535	0.02517109	0.0269971
20.922527	17.6755945	20.0224398	21.9920009	21.8171985	20.8164921	77.9123872
0.5782608	0.52104927	0.85201872	0.55675952	0.91285349	1.03201472	0.60615074
3.99525641	4.20847489	2.93473113	3.06217734	3.14934456	3.32258399	2.61915238
0.89367578	0.96193712	1.08869058	1.14445012	1.32363757	1.15787018	1.26603407
42.2656073	36.7139333	44.3996419	44.6954167	44.8211066	44.6535151	89.3740623
13.8519745	15.9922046	17.135043	13.8261946	12.5060929	15.4802209	19.288465
24.1555305	18.316886	19.4544273	23.0745888	21.7259132	23.9880496	30.9739044

86.3185661	104.610662	97.0827992	88.8959361	95.5301182	79.8930423	79.3032151
8.98932692	10.0201783	5.72745914	12.3415026	8.07875343	8.68402634	11.9657359
0.9988141	0.84169498	1.13602495	0.86607036	0.63899745	2.4667669	0.59355579
47.1545395	48.8583895	45.298995	47.35549	61.5263255	46.9440844	17.1779195
23.0252935	23.8079437	24.6612084	25.4562823	25.8337539	26.5051586	10.1868002
32.9608654	30.3010192	32.9447237	34.3025724	36.4228544	34.081657	23.0732711
4.59980179	3.3266992	3.171403	4.23755854	3.6057713	5.31110017	8.65672276
0.10513833	0.04008071	0.04733437	0	0.09128535	0.02517109	0.5303677
0.07885374	0.08016143	0.09466875	0	0	0.10068436	0.05974644
0.21027665	0	0	0	0.04564267	0.05034218	0.38915028
0.07885374	0	0.09466875	0.09279325	0.09128535	0.12585545	0.04438032
32.8031579	32.7860234	30.2939988	29.9412895	28.5266717	29.4501763	25.437477
22.4207481	24.3289929	23.7618553	23.2911064	21.2694864	19.683793	14.5116249
27.5462416	33.266992	35.1221048	29.6629098	29.8046666	27.5623445	38.7766533
137.28437	157.276719	124.915411	148.902239	115.384682	113.647475	58.5353601
50.9132347	52.7061379	56.5645758	48.840182	49.3853741	49.1339693	41.3967499
6.80770665	6.97404411	8.18884654	6.52645877	7.07461458	5.7641798	6.08977499
44.6837888	41.3232154	33.8914111	42.4993097	43.1323276	45.8868986	34.9271699
1.26165992	0.6012107	1.42003119	1.1753812	1.27799489	1.71163418	8.36514906
22.8938706	20.3209216	19.3124242	20.7238264	17.2985737	18.6014361	33.4396833
13.9045437	13.1865547	14.8629931	13.1147797	11.7301674	12.8120852	9.42078291
0.34169956	0.08016143	0.14200312	0.43303518	0.22821337	0.25171091	6.14700062
73.5179749	74.1092388	82.219806	75.0388103	76.2232668	57.9690222	80.441374
28.5976248	28.2969836	32.5660487	25.9821108	28.481029	27.4616601	16.8430685
2.12905111	1.32266354	1.32536245	0.86607036	1.09542419	1.78714745	3.6994842
10.9081014	10.0201783	9.46687461	12.6198824	9.44803367	12.1072947	5.4088852
0.05256916	0	0.09466875	0.03093108	0.09128535	0.15102655	0.14258777
121.829036	112.666885	138.595044	106.155481	135.011032	94.6181304	60.6189407
21.4745032	16.6735767	21.7264772	17.9090978	19.4894221	16.5374067	24.5623596
9.01561151	8.97807977	10.2242246	10.6712241	9.95010309	10.8235691	14.3043642
1.10395243	1.44290568	1.42003119	1.1753812	1.04978152	0.95650145	1.83984843
38.2703509	38.8382111	27.785277	39.2206148	41.3066206	35.8688044	17.5142374
66.7628374	69.9408446	68.8715128	70.0898369	61.2981122	57.2642316	41.4148052
327.164188	389.825017	362.581298	350.541978	331.228891	297.094385	308.68888
4.36324055	4.32871703	4.11809046	3.5261436	5.61404899	5.99071962	2.72320812
0.63082996	0.52104927	0.18933749	0.12372434	0.50206942	0.35239527	0.56575147
0.07885374	0	0	0	0	0.02517109	0.14560094
23.8926847	26.4532708	25.8919021	25.1469715	22.7756947	21.6974803	8.66002056
76.0412947	77.9169066	64.4694161	83.9469626	80.7875343	73.0968478	130.922162
6.86027581	7.81573909	5.53812165	5.44387082	6.20740376	6.09140398	3.90419836
0.18399207	0.08016143	0.14200312	0.12372434	0.04564267	0.07551327	0.34362564
388.433548	359.884724	427.003379	355.119778	373.448365	338.777712	457.990399
10.0144256	10.5813083	10.4135621	9.55770503	12.0953088	13.2651649	12.5453143
64.213233	67.5760826	68.2088316	68.9453867	63.1694618	49.5115357	33.9533617
610.748539	828.86915	745.942385	643.737725	683.499054	628.396283	333.163743

69.049596	75.5521445	85.1072028	76.7709511	68.0988707	65.0169276	54.5126518
53.8045386	58.9186485	57.9372726	52.3353945	53.5388575	51.3238542	51.6424479
599.314746	606.581514	654.113701	582.463247	578.65783	521.771542	323.60755
1.5770749	1.28258282	0.94668746	2.22703806	1.14106687	1.45992327	35.7261574
0.31541498	0.32064571	0.42600936	0.37117301	0.3651414	0.65444836	0.42705313
2.41818151	2.08419709	2.55605615	3.09310842	2.19084839	3.04570199	2.949278
0.44683789	0.24048428	0.28400624	0.55675952	0.31949872	0.47825073	0.6344866
2.41818151	1.32266354	2.22471553	1.42282987	1.18670954	2.11437163	6.20152167
6.20316127	4.60928202	7.19482471	5.53666408	6.84640121	8.58334197	2.16806738
3.10158063	3.44694134	1.13602495	1.51562313	2.41906176	2.13954272	6.43070834
0.91996036	0.56112999	1.04135621	0.71141494	0.68464012	0.57893509	0.42936816
0.26284582	0.20040357	0.09466875	0.12372434	0.04564267	0.17619764	0.23173724
64.1343792	68.2574547	72.0429158	66.4399689	78.1859018	78.1058949	91.974961
0.31541498	0.44088785	0.28400624	0.34024193	0.45642675	0.60410618	0.29419939
0.05256916	0.04008071	0.18933749	0.06186217	0.04564267	0.02517109	0.02846706
5.94031545	5.731542	6.86348409	6.52645877	4.97505155	4.50562526	3.27624005
12.0383384	11.182519	8.37818403	8.32046166	10.5434579	9.11193488	6.58306754
45.6826029	41.7641032	45.8670075	46.1491777	38.1116334	37.8824917	25.4186288
133.604529	140.843626	140.204413	139.746639	155.96102	157.168291	38.5508792
4.59980179	3.3266992	3.21873737	4.26848962	3.6057713	5.31110017	7.59361646
85.3986058	77.2756152	86.3378965	91.4013539	83.5717374	94.5677883	126.015509
34.5379403	32.3050549	30.9093456	42.7467584	32.3150137	32.7475892	14.9742172
1.2879445	1.16234068	0.94668746	0.86607036	0.59335477	1.18304127	2.836226
0.21027665	0.48096856	0.33134061	0.74234602	0.27385605	0.37756636	8.43371585
28.1507869	30.0605349	29.2526426	30.1887382	25.1491138	24.4914714	33.9442515
1.07766785	0.96193712	0.85201872	2.50541782	0.7302828	0.72996163	0.78868992
122.381012	133.468775	123.306042	121.651954	116.662677	109.821469	118.251888
303.350357	319.643688	308.667447	320.167653	289.602771	231.599207	187.795559
0.31541498	0.40080713	0.18933749	0.40210409	0.86721082	0.17619764	0.16478433
0.5782608	0.28056499	0.89935309	1.05165686	0.86721082	0.47825073	0.47001855
16.0335948	14.8699446	14.1529775	15.6511286	16.2944349	17.8966456	4.84706794
3.39071103	1.88379352	2.22471553	1.94865831	1.91699234	2.34091145	2.07910119
51.202365	49.7000844	49.2750824	56.2636422	52.1239345	54.5205827	40.009645
38.3492046	30.4613421	33.5600705	39.5299256	32.8170831	40.2485742	47.3141879
17.0061243	13.4270389	14.9103275	17.1976828	15.7010801	17.468737	24.1899319
27.6250953	23.0864908	26.6019177	23.878797	21.1782011	25.2717752	63.8212886
75.2790418	88.9791834	83.5451685	92.0199756	91.4679202	84.9272605	85.3454526
14.0622512	13.0663125	11.7862589	13.207573	18.1657845	12.8372563	5.45407254
6.09802294	6.81372125	5.77479351	5.876906	5.20326492	5.33627126	7.75025628
256.090679	208.259386	252.292208	267.615741	238.985045	282.89789	1255.59902
29.9118539	29.4192435	28.9686363	31.6115681	19.7632782	27.5623445	25.4455705
20.9488116	20.1605988	19.8331023	18.4658573	18.3483552	18.8279759	9.18178886
0.60454538	0.32064571	0.56801248	0.49489735	0.27385605	0.32722418	0.54297464
65.5011775	86.0933721	71.0015596	102.072578	70.7005032	74.7581398	26.3617785
242.422697	254.352206	262.942442	243.05646	230.312937	222.462101	181.878022

4.28438681	3.76758705	5.25411541	3.99010987	4.42733945	7.37512961	4.72803545
0.05256916	0	0.04733437	0	0.04564267	0	1.08079132
0.05256916	0	0.04733437	0	0.04564267	0	0.02899327
4.57351721	3.84774847	5.63279039	4.7015248	6.07047574	5.53763998	6.98902717
16.8484168	16.9942224	16.3776931	18.001891	18.4396406	18.7776338	20.2958348
3.39071103	3.20645706	2.88739676	3.24776384	3.56012863	4.30425653	2.1178488
0.68339912	0.64129141	0.75734997	0.86607036	1.04978152	0.75513273	0.31630423
0.15770749	0.28056499	0.14200312	0.21651759	0.09128535	0.05034218	0.12478735
1.31422908	1.24250211	1.2306937	1.05165686	1.41492292	2.26539818	1.40224778
19.1877446	15.3909939	13.8216369	17.0120963	18.6222113	18.6014361	13.7908845
10.5926864	10.2606626	10.8395714	14.1045744	11.0455273	10.4460027	6.93305366
7.70138242	6.57323697	6.24813724	8.25859949	8.99160692	6.39345707	17.4462735
36.7458451	32.0244899	36.542136	37.6121984	34.9622888	42.4636302	48.4121177
26.4948583	26.41319	27.5959395	24.0953146	26.1532526	23.7866808	9.22857916
0.21027665	0.56112999	0.37867498	0.21651759	0.41078407	0.17619764	0.28391648
0.07885374	0	0.04733437	0.15465542	0	0.10068436	3.32622845
109.92212	113.308176	120.749986	106.031757	113.65026	95.9270272	40.121173
5.94031545	6.13234913	7.14749033	5.2892154	8.6721082	8.30645998	7.61708639
0.07885374	0.08016143	0.09466875	0.18558651	0.22821337	0.05034218	0.1778076
27.3096803	22.5253609	33.4654018	26.3532838	22.1823399	26.1527634	87.7990271
0.97252952	0.64129141	1.42003119	0.49489735	0.91285349	0.90615927	0.5204204
28.2822099	28.938275	37.3468203	30.9310842	32.132443	42.0860639	82.66956
22.6047402	22.6856837	22.6258303	25.2397647	17.4811444	17.9973299	22.3270455
244.39404	289.583153	270.70528	248.716848	280.20038	194.320821	131.992773
8.64762736	8.4570305	9.70354648	8.38232383	8.53518017	6.92204998	17.8842185
315.730395	376.47814	393.443309	395.577636	314.432386	253.951135	132.292633
40.7673861	36.8742562	29.44198	43.7365531	37.7008493	37.0266746	31.2567491
7.64881326	7.37485124	6.72148097	7.79463323	7.57668401	9.0615927	2.39270698
16.8221323	18.1966438	13.5376307	12.867331	16.1118642	14.5488905	9.16422475
43.474698	44.2891882	50.1744354	48.2524914	40.9414792	36.5484239	21.8541023
3.52213394	3.04613421	4.2127592	3.71173011	4.29041142	4.50562526	6.72689241
3.20671896	3.16637635	2.50872177	3.3405571	3.05805921	4.02737453	2.03092523
2.78616565	2.64532708	3.78674985	2.3507624	2.69291781	2.69330672	2.8319696
104.822912	106.334132	108.537717	95.484257	107.169	91.2955465	33.414547
63.3195572	72.4258489	66.1734535	66.5636933	63.99103	54.4702406	38.5554415
28.2296407	30.2208578	33.5600705	29.1680124	28.3897437	26.6813563	43.1391677
21.9476257	20.7618095	19.3597586	18.8679614	21.6802705	15.530563	15.9953573
47.6539465	43.5276546	36.3527985	36.2202996	51.9870065	54.4450695	26.8575771
0.02628458	0.04008071	0	0.06186217	0.04564267	0.05034218	0.05219731
4.65237095	4.92992773	4.68610293	5.13455998	6.11611841	5.13490253	9.16453604
8.80533485	8.29670764	7.28949345	8.72256575	8.94596425	9.76638324	7.88250016
7.3071137	8.05622337	6.76881535	7.79463323	8.58082285	8.53299979	17.7003655
3.36442645	3.24653777	2.36671865	2.25796915	3.92527003	4.83284944	1.55542454
4.12667932	3.72750633	4.11809046	4.63966263	4.65555282	4.45528308	4.93219936
9.46244939	11.1424383	10.5082308	7.88742648	10.9998846	8.68402634	8.00226409

3.81126434	3.3266992	3.92875296	4.20662745	1.96263501	2.91984654	3.18896275
72.6768683	72.5060103	76.4450125	75.6265009	69.3312229	57.8179956	46.0525992
0.02628458	0.04008071	0	0.06186217	0.04564267	0.05034218	0.06595598
3.4432802	3.00605349	3.88141859	3.68079902	3.74269933	4.53079635	2.52071452
0.10513833	0.6012107	0.5206781	1.1753812	0.41078407	0.25171091	0.01900749
0.02628458	0.04008071	0	0.06186217	0.04564267	0.05034218	0.02590938
0.02628458	0.04008071	0	0.06186217	0.04564267	0.05034218	0.11821155
4.78379386	3.56718348	3.97608734	4.73245589	4.38169677	5.16007362	3.67469048
5.62490047	5.41089629	5.86946226	5.78411275	7.62232668	5.99071962	2.31600738
1.91877446	1.8036321	3.45540923	2.47448674	3.28627258	2.91984654	2.45139273
110.237535	105.492437	84.633859	97.7422262	107.123358	127.466404	61.6748017
10.5401172	10.8618733	8.52018715	10.5165686	6.34433179	9.08676379	6.10346519
1.99762821	2.20443923	3.64474673	3.43335035	2.60163246	3.27224181	1.28681894
45.2620496	48.4175016	42.2222608	43.3963112	44.2277518	37.4545832	37.0729152
0.44683789	0.64129141	0.56801248	0.40210409	0.31949872	0.47825073	0.29191466
13.9833974	13.9881689	15.6203431	14.0117812	13.1450903	12.1072947	35.8609731
2023.3871	2049.60743	2443.96835	1907.30345	2031.82931	1864.14582	1421.69996
26.1794433	25.8921408	28.9686363	27.2812163	24.0993323	22.6539818	31.5361177
70.7318092	64.3295448	64.4220817	76.9874686	72.4349248	67.2571547	48.9534261
26.2582971	25.5314143	26.1759083	27.0337676	27.4312475	30.2808223	54.7679252
356.55035	401.127778	392.733293	358.676853	381.800974	301.82655	115.693049
31.7780592	29.6597278	23.6671865	29.3226678	32.132443	32.3196806	11.540339
80.2993969	69.5801182	75.9716688	71.1724248	90.8289227	84.2979832	109.32562
6.51857625	9.45904833	9.70354648	7.79463323	8.3069668	6.16691725	1.94608379
10247.5436	10413.9713	10276.6711	9248.64163	9580.03228	8369.31219	10109.4106
0.05256916	0.04008071	0	0	0.04564267	0	0.06837542
49.4412981	53.6279943	51.4051291	47.2317656	47.9248085	47.3468219	26.9342293
15.6393261	14.4691375	14.8629931	14.3829542	12.4148075	13.7937578	11.945899
1.10395243	0.76153355	0.47334373	0.83513927	0.86721082	1.38441	2.3568041
6.22944585	5.25057344	5.86946226	6.89763178	6.20740376	6.56965471	39.2803576
61.7687669	72.826656	87.7105933	65.4811053	67.3685879	68.99396	37.0850665
0.07885374	0	0	0.03093108	0	0	0.12750909
200.551358	210.103099	217.170104	195.886556	203.018617	177.028282	150.141691
18.9774679	17.3950296	17.9870618	15.6511286	16.7508616	17.0408285	5.45461868
0.05256916	0	0	0	0.1825707	0.02517109	0.06681255
0.07885374	0	0	0.03093108	0	0	0.07655605
21.3693649	23.0864908	24.5665396	22.4868982	24.1449749	29.1481232	48.0529952
33.0397191	35.7920769	35.8794548	29.7247719	35.2361449	31.1366394	36.0062378
18.5569146	16.2727696	18.1763993	17.9090978	13.464589	16.2101825	11.83854
162.175869	167.858027	180.864639	155.830802	162.077138	149.642135	106.488112
0.91996036	1.08217926	0.99402183	0.95886361	0.77592547	0.65444836	0.62141025
0.07885374	0	0	0.03093108	0	0	2.5384375
30.7003914	33.4673956	31.2406862	36.9007835	35.1905022	31.866601	66.352185
68.9970268	69.8206025	65.7474442	75.7502253	64.0823153	83.3918239	37.5291449
9.83043354	10.4209854	9.56154336	8.6297725	8.17003878	9.74121215	20.2960753

38.1652126	36.5536105	35.3587767	37.4884741	40.2568391	41.7084975	13.8804646
0.07885374	0	0	0.03093108	0	0	0.067763
23.3669931	21.4431816	26.4599145	18.6514438	22.5018386	23.3587723	46.4788594
59.3505854	58.1571149	57.227257	55.7687449	55.0450657	50.5687215	33.2049838
0.49940705	0.68137213	0.66268122	0.34024193	0.27385605	0.67961945	0.37014074
0.05256916	0	0	0.06186217	0.09128535	0.02517109	0.09154033
0.5782608	0.48096856	0.47334373	0.5876906	0.1825707	1.35923891	0.58825606
128.978442	135.953779	124.962745	123.569681	120.633589	114.578805	49.491038
0.78853745	0.84169498	1.70403743	1.33003662	1.55185094	1.25855454	0.7744731
38.138928	38.5175654	34.6487611	41.6951015	40.3937671	38.6879666	16.9161671
1.70849781	1.16234068	2.84006238	1.88679614	3.87962735	1.68646309	0.98144175
300.774468	287.939844	304.123347	291.896642	288.233491	232.102629	118.002879
28.2033561	26.8139972	25.7972333	25.1469715	25.9250392	23.3587723	37.5008155
70.6266709	73.7485124	81.4151217	68.295834	68.2357987	71.6369245	61.1440669
39.1903112	43.1268475	39.0508578	37.9524403	41.260978	31.9672854	23.2328232
1.62964406	2.84573064	1.98804367	1.73214072	2.05392036	1.51026545	12.3821092
0.13142291	0.20040357	0.18933749	0.24744867	0.22821337	0.25171091	0.08244498
26.6525658	22.7257644	25.4185583	26.2914216	27.5225329	27.7637132	27.1019878
25.6800363	28.8180328	32.2347081	26.7863189	27.1573915	25.2717752	13.8846454
0.15770749	0.40080713	0.28400624	0.40210409	0.45642675	0.30205309	0.13844367
89.2887239	91.143542	87.0952464	105.25848	93.4761978	91.5975995	51.3519422
74.5430736	85.8128071	89.5566338	75.6265009	76.1776241	78.8610276	120.379464
4.91521677	4.08823275	4.2127592	4.91804239	5.20326492	5.10973144	7.27931271
27.4936724	29.6998085	26.2232427	28.3638042	26.4271087	21.0933741	14.803083
190.326656	196.916544	184.462052	174.729695	184.944118	163.108669	202.719891
25.9428821	21.8439887	24.992549	25.6109377	23.5972628	30.8094152	14.550915
1.62964406	1.68338996	2.08271241	1.60841638	1.91699234	2.99535981	1.18099501
223.813213	226.015142	229.808381	208.320852	211.188656	205.773668	175.223941
0.52569163	0.32064571	0.5206781	0.52582843	0.7302828	0.57893509	0.48754423
0.02628458	0	0.09466875	0.12372434	0.09128535	0.02517109	0.07683045
0.26284582	0.32064571	0.5206781	0.09279325	0.31949872	0.32722418	0.70822779
19.0826063	18.8379352	19.3597586	21.651759	20.493561	20.4892679	19.6991319
0.84110661	0.6012107	0.75734997	0.80420819	0.5477121	0.35239527	0.48487905
24.6023684	26.41319	30.8620112	28.4256664	23.2321214	22.452613	25.34376
59.7185695	62.0048634	59.120632	63.4087227	65.5885236	66.5271931	72.87067
1.78735155	1.04209854	1.42003119	1.11351903	2.05392036	2.24022708	1.58782922
0.02628458	0.04008071	0	0.03093108	0.09128535	0.05034218	0.01794783
0.02628458	0.04008071	0	0.06186217	0.09128535	0.02517109	0.01600475
0.05256916	0	0	0.03093108	0.09128535	0.05034218	0.93878954
0.02628458	0	0.09466875	0.12372434	0.09128535	0.02517109	1.12819445
34.5116557	38.6378076	38.1041703	37.3338187	35.8751423	28.0405952	23.0382539
127.769351	145.052101	140.346416	128.271206	142.679001	103.780408	37.2982618
15.1924882	9.25864476	10.9342402	16.9502342	9.95010309	14.2216663	20.3668461
239.583962	261.727058	272.693323	229.230265	249.437217	230.038599	367.267585
65.0806242	63.6481727	70.5282159	59.480475	60.613472	63.7583731	140.657543

25.3909059	21.7237466	27.027927	21.0640684	22.5018386	23.9125363	40.8151396
10.9869551	12.3849404	9.46687461	11.4135701	8.44389483	9.16227706	8.4449767
63.2932726	72.1052032	71.5695721	63.8108268	68.1445134	58.3717596	28.2833711
11.8806309	9.81977475	11.3602495	11.2279836	8.8546789	9.3384747	9.02976132
8.77905027	7.41493195	7.3841622	9.03187659	7.62232668	9.94258088	6.4422813
9.43616481	9.41896762	9.41954024	9.55770503	8.80903622	6.74585234	3.01426334
72.5717299	67.8967283	68.5875066	82.7715814	74.0780611	70.0259747	53.6897058
72.0723229	72.3857681	78.6223937	76.0904672	67.5511586	61.1657507	31.8227797
1.20909076	0.56112999	0.61534685	0.89700144	0.63899745	1.661292	0.8659619
256.222102	266.977631	280.88217	252.614165	252.449634	231.825747	241.287702
2.23418944	2.44492351	2.69805926	2.44355565	2.82984583	3.37292617	1.26971012
0.26284582	0.52104927	0.18933749	0.37117301	0.31949872	0.62927727	0.24944563
0.63082996	1.28258282	0.89935309	1.02072578	1.23235222	1.23338345	1.11919507
13.7731208	12.5452633	15.6676775	12.9601243	8.99160692	14.699917	30.0865283
84.9780525	77.9569873	96.7041242	89.5764199	89.5509278	102.949762	456.933628
147.561641	153.068244	141.861116	165.883405	185.446187	136.704194	69.9922385
21.6322107	12.6655054	17.0403743	13.3312973	17.9375712	13.8441	8.28632059
6.51857625	6.3327527	6.62681223	7.20694262	7.07461458	7.55132725	7.64532918
0.63082996	0.56112999	0.5206781	0.37117301	0.95849617	0.98167254	0.78066262
77.0401088	74.7505302	83.6398372	80.6064055	81.380889	92.5289299	57.5875531
0.02628458	0.12024214	0.18933749	0.06186217	0.04564267	0.05034218	0.02006461
0.10513833	0.08016143	0.04733437	0.09279325	0	0.02517109	0.09137774
1.62964406	2.1242778	1.84604055	1.39189879	1.91699234	1.35923891	1.3410828
42.0290461	35.9924805	32.4713799	36.5914726	29.9415946	34.1068281	14.7253367
257.483762	219.481986	227.346994	268.822053	243.138528	258.154708	224.742129
172.16401	157.998172	150.665309	157.377357	168.969182	203.835494	297.255749
10.7241093	8.13638479	7.81017156	9.99074021	8.90032157	11.4528463	12.1076558
0.65711454	0.9218564	0.56801248	0.77327711	0.31949872	0.50342182	0.46317404
53.7256849	45.9324974	52.1624791	53.9128798	93.8869819	33.5782352	29.3139336
11.9857692	11.8238104	10.6975683	9.68142936	12.5517356	8.55817088	29.3533834
3.96897183	3.44694134	4.02342171	4.88711131	3.4232006	4.88319162	7.44660244
6.99169872	6.53315626	4.02342171	6.09342359	7.98746808	6.56965471	9.07463351
2.47075067	3.56718348	3.78674985	2.87659083	2.51034711	2.79399108	0.88375232
110.999788	116.193988	116.063883	109.774418	108.036211	98.5448206	104.329164
1391.0852	1505.51175	1497.32822	1282.92858	1491.87647	1151.85429	874.682107
0.13142291	0.24048428	0.18933749	0.06186217	0.04564267	0.20136873	0.3345842
7.25454453	8.65743406	8.47285278	6.99042504	7.25718528	7.4254718	10.7243233
15.770749	13.8278461	15.2416681	14.9397137	13.6015171	11.8807549	9.92138082
53.8308232	50.5417794	56.6592446	57.5008856	56.2774179	60.5868156	85.5756379
0.15770749	0.12024214	0.04733437	0.24744867	0.31949872	0.05034218	0.20190607
5.99288462	6.21251056	4.82810605	7.54718455	5.47712097	6.46897034	9.65241278
4.88893219	5.57121914	6.62681223	4.7015248	5.70533434	4.43011199	6.27880184
42.9227218	42.0045875	41.8909202	43.0251382	39.2527003	34.4088812	33.4757626
135.023896	129.500785	144.748513	138.323809	123.463435	112.690974	47.6482745
58.2203483	56.2733214	50.6477792	65.3883121	54.3147829	60.133736	25.2285139

0.23656123	0.12024214	0.09466875	0.49489735	0.13692802	0.12585545	0.11299566
0.9988141	0.88177569	1.65670306	0.9897947	0.68464012	1.03201472	0.52585952
11.9332001	11.743649	10.9815746	11.7847431	9.99574577	9.28813252	4.92999254
12.6428838	12.9059897	13.2536245	12.124985	12.2322368	10.7480558	14.6042054
2.2867586	1.76355138	1.42003119	1.5774853	1.36928024	1.61094981	1.47930097
26.573712	26.8139972	24.2825334	29.6319787	27.2030341	27.6126866	15.4318683
1.68221323	2.28460066	2.17738116	1.48469204	1.46056559	2.03885836	6.4508673
1.07766785	1.48298639	1.04135621	0.80420819	0.82156815	1.20821236	0.80777893
0.49940705	0.72145284	1.2306937	0.43303518	0.5477121	0.78030382	0.3125696
38.1652126	36.4734491	36.5894704	30.2815315	42.4933302	27.5623445	47.9086121
52.7794399	50.181053	54.3871946	53.6345	52.7172893	65.117612	28.8878352
69.4438647	69.6201989	65.8421129	76.2760537	63.9453873	83.0897708	37.5717192
0.15770749	0.08016143	0.09466875	0.12372434	0.22821337	0.20136873	0.49786129
159.862826	176.074573	158.617484	166.161784	149.525402	135.67218	92.7473752
37.7446592	46.5337081	42.4115983	36.4368172	51.0285103	21.1437163	22.3217239
14.2725278	19.0784195	16.5670306	15.4036799	15.7010801	13.7685867	12.2651023
107.425085	87.9370849	103.425605	88.1845211	116.023679	127.13918	136.531454
0	0	0.04733437	0.12372434	0	0	0
28.6764786	29.21884	33.4180674	30.3743247	26.1988953	26.9582383	29.6029654
71.5992004	68.6983425	73.6996189	66.1925202	89.7791412	77.5269598	201.116527
424.022871	503.012951	420.707908	420.941125	453.368688	338.299461	70.9383116
68.2084894	68.9789075	64.8480911	74.915086	63.2151045	81.9822428	35.3777573
25.8640283	29.499405	25.0872177	30.0340828	26.0619673	31.6904034	17.1439095
56.9324038	62.5259127	65.9841161	56.4801598	60.3852587	53.8913055	72.9644539
40.8725245	46.0527395	44.4469763	39.8083054	43.2692556	38.2097159	37.9353242
64.0029563	72.3456874	67.4041472	65.9141405	64.8582408	56.8614942	42.1387912
2.20790486	1.96395495	1.98804367	2.41262457	2.28213374	2.56745127	1.04141026
12.0383384	16.2326889	10.4608964	21.4661725	10.7716712	13.2148227	19.4663874
31.4889288	28.4973871	30.578005	29.2298746	26.6096794	27.5623445	34.1791863
90.3663917	91.9050755	87.2372496	95.2368083	112.509193	86.6640657	53.8581614
0.18399207	0.28056499	0.23667187	0.18558651	0.3651414	0.42790854	0.84140109
0.18399207	0.20040357	0.18933749	0.12372434	0.09128535	0.15102655	0.26794618
60.4282532	84.570305	76.2083406	72.5952547	72.0241407	54.5457538	49.5615101
2.75988107	2.32468137	1.89337492	2.19610698	1.36928024	2.1898849	1.40642444
81.4296339	80.3217494	68.0668285	89.0196604	84.0738069	76.469774	38.5195948
69.365011	75.5521445	61.4400162	74.7604306	76.0406961	73.3485587	61.1554569
5.75632338	5.25057344	4.63876856	6.68111419	5.61404899	5.61315326	3.50416802
43.0015756	37.5556283	30.6253394	38.6947864	45.460104	38.6879666	52.0308548
6.04545378	4.32871703	3.6920811	4.57780047	5.47712097	6.56965471	2.10243485
25.1806292	23.3670558	21.7738116	22.2085185	20.904345	21.3954272	15.1590024
6.07173836	4.5291206	5.06477792	6.65018311	6.25304644	5.2104158	5.16334246
56.8009809	47.5357259	52.7304916	46.3966263	49.2028034	49.0332849	39.7050306
16.7432785	18.5573702	15.857015	15.063438	16.0662215	15.3795365	25.6825154
5.151778	5.1303313	5.86946226	6.00063034	3.69705665	4.07771672	3.78731621
2.73359649	3.36677991	2.88739676	2.1651759	3.4232006	2.01368727	8.58528456

28.2559253	28.6977907	32.8027205	29.3535989	26.7466074	26.1779345	38.9027094
5.75632338	5.49105772	5.20678104	7.60904672	4.8837662	6.34311489	3.95708099
13.0634371	12.865909	9.89288397	12.8364	13.8297304	13.4161914	4.31142863
34.5905094	32.6657813	31.0513487	33.776744	28.2984583	28.0154241	28.1905802
138.388322	140.723384	131.873563	149.892034	146.512986	150.095215	60.3308614
7.3071137	5.37081558	6.24813724	5.6913195	5.84226237	5.18524471	5.38799553
1.07766785	1.32266354	1.2306937	0.95886361	1.82570699	1.73680527	1.18872426
2.33932777	1.36274425	2.22471553	1.82493397	2.69291781	2.7436489	1.97560594
25.9428821	27.0544815	22.1524866	25.3634891	21.6346278	21.4709405	10.1912478
14.325097	16.5934153	17.0403743	13.7643325	13.3733037	15.9081294	10.4276586
26.7314195	26.7739165	31.9507018	25.6418688	24.3275456	28.0154241	54.4095705
0.02628458	0.04008071	0	0.03093108	0.04564267	0.07551327	0.01278979
101.563623	86.3739371	94.1007336	103.217028	96.4886144	106.901623	22.9016194
2.89130398	3.36677991	3.92875296	2.66007324	3.14934456	6.67033907	2.627704
81.4559185	93.3079005	80.1370936	79.4310243	107.032072	92.2017057	22.1751031
56.9061193	53.4275908	50.6004448	55.8924692	60.613472	57.9941933	34.7194103
151.767174	176.114654	168.415699	154.222386	156.965158	143.248678	110.377259
0.05256916	0.28056499	0.23667187	0.09279325	0.3651414	0.40273745	0.05851418
115.205321	107.616715	127.897476	95.7317057	115.795466	109.670443	117.440811
10.2772714	12.0642947	13.6322994	9.37211852	9.12853495	12.9882829	5.2635691
0.07885374	0	0	0	0.04564267	0	0.07132024
0.02628458	0.12024214	0.04733437	0.15465542	0.09128535	0.07551327	0.06379671
30.4375455	27.7759343	27.406602	26.9719054	30.6718774	36.1960286	27.5805935
1.81363613	1.04209854	2.41405303	1.79400289	2.32777641	2.34091145	3.6520072
0.13142291	0.04008071	0.04733437	0.06186217	0	0.05034218	0.09820874
31.856913	32.2248935	33.749408	29.013357	32.7257978	28.3929905	14.2382508
38.1126434	30.3410999	32.8973893	20.8475508	32.5432271	36.4225684	17.9490416
0.97252952	0.76153355	0.7100156	0.9897947	1.00413884	1.08235691	1.82047596
38.638335	39.0786954	39.3821984	41.4167218	34.5515048	31.2121526	21.2669095
2.60217358	3.40686063	2.17738116	2.84565975	1.41492292	2.39125363	1.31782266
0.05256916	0.08016143	0.14200312	0.09279325	0.13692802	0.17619764	0.07766556
4.46837888	4.00807133	4.73343731	3.80452336	3.4232006	4.20357217	12.1875186
168.247607	185.052653	203.206464	167.399028	180.105994	160.13848	137.164159
0.76225287	0.56112999	0.7100156	0.5876906	1.00413884	1.03201472	0.51219594
0.26284582	0.08016143	0.33134061	0.18558651	0.04564267	0.17619764	0.35509157
0.36798414	0.44088785	0.18933749	0.21651759	0.41078407	0.12585545	0.47990361
1.91877446	1.00201783	1.18335933	1.85586505	1.14106687	2.16471381	2.14259109
1.10395243	1.16234068	0.66268122	0.89700144	1.59749362	1.28372563	0.74363918
14.6142274	15.5913975	17.0877087	15.4036799	13.5558744	13.7434156	8.98586035
48.4687686	42.0045875	44.7309825	40.024823	49.0658753	60.0078805	61.6973266
2.75988107	2.7254885	2.65072489	2.53634891	1.68877897	2.56745127	8.91974076
149.664408	153.829777	167.137671	150.170414	148.79512	132.67682	79.9835152
27.8879411	25.9723022	27.3592676	27.6833204	23.5059775	23.3587723	10.6095188
1520.11621	1875.21625	1755.39523	1651.50338	1806.17192	999.871241	313.898679
24.9703526	24.0083472	21.1111304	27.188423	23.8254762	25.5486572	11.5638329

53.0948549	68.9789075	69.534194	53.3251892	60.613472	60.7126711	289.956046
0.05256916	0.12024214	0.14200312	0.09279325	0.09128535	0.276882	0.06633015
10.2509868	10.4610662	13.7743026	12.8054689	10.6347432	5.83969307	10.7592289
40.083987	37.6758705	41.5122452	42.6848962	43.360541	42.664999	23.6081613
14.824504	11.6634876	12.9222838	13.7024703	14.6969413	15.4550498	27.2291254
12.6165992	12.865909	12.2596026	10.0835335	13.7384451	10.1187785	5.56369863
0	0.04008071	0	0	0	0.10068436	0
0.15770749	0.08016143	0.14200312	0.09279325	0.22821337	0.276882	0.0908506
256.642655	285.013952	297.827875	263.440044	269.154853	234.795935	147.04786
0.07885374	0	0	0	0.04564267	0	0.22669947
29.9907077	26.693755	31.0040144	28.3947353	26.381466	25.9262236	35.6186321
50.1772664	54.9506579	49.5117542	50.0774254	51.211081	40.3996008	15.9286651
0.52569163	0.44088785	0.66268122	0.24744867	0.50206942	0.47825073	0.46131337
0.52569163	0.64129141	0.61534685	0.34024193	0.59335477	0.45307964	0.45398576
0.39426872	0.56112999	0.14200312	0.21651759	0.27385605	0.30205309	0.36677044
66.6576991	62.3655898	62.9073818	66.3471757	72.8457089	68.3143405	49.0032342
3.94268725	5.61129986	4.30742795	5.07269781	3.56012863	3.09604417	3.61005333
83.0329934	87.0152285	85.5805465	78.4721607	90.8745654	65.0924409	36.1534217
0.73596829	0.9218564	1.2306937	0.95886361	0.31949872	0.830646	1.17150184
24.9966371	24.4492351	22.7678334	24.1881079	20.5392036	21.3702561	16.6139735
11.9069155	12.8258282	12.2596026	9.8051537	11.5932394	11.905926	20.9878305
2.70731191	2.80564993	3.40807486	2.1651759	1.91699234	3.24707072	3.90314059
87.7642181	85.2917578	92.0653556	96.1647409	92.3807737	96.4807912	65.8250617
34.932209	32.3050549	32.0453706	29.8175652	27.1573915	33.9054594	69.7097176
2.31304318	1.72347067	1.89337492	1.63934746	1.59749362	1.76197636	2.16075729
20.0551358	16.1926082	15.9990181	20.8166197	15.290296	22.4274419	3.57547946
9.35731107	11.8638911	9.75088085	8.2276684	10.2239591	9.13710597	9.11721293
0.15770749	0.12024214	0.04733437	0.24744867	0.13692802	0.15102655	0.9433802
196.66124	215.594157	208.318576	188.524958	192.475159	191.073751	216.148067
11.1972318	11.5031647	8.66219027	12.9910554	10.1783165	12.0821236	5.4910438
0.39426872	0.20040357	0.94668746	0.46396626	1.32363757	0.45307964	0.16559764
20.0551358	15.3509132	15.6676775	20.0433426	21.5433425	15.8326161	13.0287585
186.646814	181.725954	167.563681	182.338742	165.363411	139.82541	140.419618
57.3792417	59.7603435	61.3926819	65.9141405	64.4474567	62.3236209	38.6196322
0	0.04008071	0	0	0	0.10068436	0
0.05256916	0	0	0.09279325	0.04564267	0.02517109	1.1692197
1.18280617	1.2024214	1.32536245	0.77327711	1.14106687	1.107528	47.4396517
0.84110661	0.96193712	1.51469994	0.86607036	0.7302828	0.95650145	2.13467497
0.10513833	0	0.18933749	0.24744867	0.13692802	0.10068436	0.10486275
61.5847748	73.0270596	78.9063999	57.4080923	68.1445134	69.3715263	51.2836951
15.4290494	13.7076039	13.9636401	12.3724337	11.0455273	15.1026545	16.4767603
6.04545378	5.41089629	5.39611853	4.91804239	5.70533434	4.27908544	2.03223814
23.5772697	25.2107686	23.4305147	22.394105	26.2901806	30.5828754	11.0293411
0.34169956	0.20040357	0.09466875	0.18558651	0.31949872	0.47825073	0.64605261
12.9845833	13.0663125	13.4429619	11.1661214	13.1450903	11.4780174	7.88476028

10.5138327	11.1023576	8.6148559	9.65049828	10.4065298	6.51931253	3.38770359
31.6729209	31.5034406	31.2406862	31.0857396	29.7590239	25.8255392	25.0695682
11.4075084	9.01816048	10.4135621	10.9186727	9.12853495	7.1485898	6.91679658
3.31185729	3.64734491	2.69805926	3.5261436	2.28213374	2.31574036	1.88522394
18.4517763	21.242778	19.3124242	17.1667517	18.7591393	17.8463034	23.8224657
7.33339828	7.13436696	4.9227748	9.92887804	5.56840632	7.1485898	16.048011
0.13142291	0	0	0.03093108	0	0.02517109	0.09290246
16.9798397	15.7116396	16.9930399	19.7031007	20.493561	15.1529967	9.90755446
67.919359	68.8586654	65.0374286	74.6676373	62.7130351	82.3598092	35.1384021
70.8369475	73.107221	69.2028534	78.7505404	66.6383051	86.3871837	37.4395311
69.2598726	69.8206025	65.9841161	76.0904672	63.7628166	83.4925083	37.8234743
94.8347706	121.564803	105.602986	90.5352835	117.849386	82.1836116	18.0898142
25.6537517	20.2808409	17.8450586	19.3009966	28.5266717	24.113905	9.85456325
103.140698	101.16372	95.0474211	97.1236045	100.550812	76.192892	41.2659027
0.10513833	0	0.14200312	0.03093108	0.04564267	0.05034218	0.10555122
255.854118	263.771174	219.063479	258.73852	224.379389	185.359913	91.2751749
63.0567114	69.9007639	75.3563219	62.9447564	68.0988707	60.7378422	28.5162094
39.1377421	38.2770812	37.2048172	37.3956808	35.4187156	33.0748134	31.9283918
20.291697	16.7136574	14.6736556	17.9400289	14.5600132	13.8189289	15.6707918
7.04426788	10.340824	8.28351529	10.7330862	8.21568145	10.0684363	6.56044855
0.44683789	0.16032285	0.47334373	0.24744867	0.31949872	0.22653982	1.31396685
55.0661986	48.9786316	67.2148097	50.6341849	56.7794874	48.4795209	59.2190504
17.7683772	23.4071365	17.2770462	19.7649628	18.6222113	13.5923891	12.2386228
31.856913	34.0686063	34.9327673	35.3542293	28.3897437	28.4181616	27.8556773
1.15652159	0.80161427	1.08869058	1.26817445	1.59749362	1.00684363	1.41972487
0.13142291	0.08016143	0.14200312	0.24744867	0.1825707	0.12585545	0.1252085
0.18399207	0.48096856	0.04733437	0.12372434	0.1825707	0.15102655	0.17583968
0.39426872	0.32064571	0.42600936	0.49489735	0.31949872	0.65444836	0.24371052
23.3407085	20.7618095	15.0523306	22.4250361	23.6885482	20.3130703	16.1723846
1.97134362	2.16435852	2.03537804	1.5774853	2.23649106	1.40958109	0.54276742
50.9132347	51.7442008	50.2217698	54.9954678	48.97459	62.2984498	49.0984708
0.07885374	0	0	0	0.04564267	0	0.09429191
10.7503939	11.6234068	10.8395714	9.46491177	10.1783165	10.2698051	12.4122686
24.0241076	29.1386785	28.8266332	22.7343469	27.2943195	24.8690377	44.0604754
0.44683789	0.28056499	0.37867498	0.30931084	0.41078407	0.78030382	0.51688909
19.5294442	17.9962403	16.5196962	19.6412385	21.9084839	18.1986987	10.4896077
104.849196	105.452357	113.602495	101.85606	100.322599	96.9338708	157.85905
14.9296424	16.3128503	17.4190493	14.5376096	14.1948718	15.3291943	16.2991626
2.47075067	1.8036321	1.32536245	1.91772722	1.18670954	4.17840108	1.38484899
145.274883	178.599658	146.357882	153.108867	153.313744	119.034089	64.7876868
0.07885374	0	0	0.03093108	0.04564267	0.12585545	0.04667826
0.07885374	0.04008071	0.18933749	0.03093108	0	0.10068436	0.01170568
48.4687686	50.1008916	51.7364698	53.0777405	43.9995384	40.9785359	39.5934096
30.1484151	26.0524636	30.95668	28.8277705	27.2030341	23.8118519	18.3164203
25.6011825	22.1245537	22.9571709	24.24997	24.2362603	28.8712412	5.49093532

1.39308283	3.12629563	3.26607174	2.78379758	2.73856048	3.92669017	1.67730089
0.76225287	1.04209854	1.7513718	0.86607036	1.82570699	1.83748963	0.56003166
2.47075067	2.04411638	1.79870618	2.04145156	2.00827769	1.25855454	2.58880764
61.7950515	56.9947743	58.5999539	52.5828432	60.9329708	49.9394442	10.9206843
1.73478239	2.28460066	2.65072489	2.22703806	2.78420316	2.84433326	0.64837573
1.89248988	1.92387424	2.03537804	1.94865831	2.73856048	2.29056927	2.66251294
9.01561151	9.61937118	8.14151217	8.59884142	11.6388821	10.0180942	23.3410895
19.1877446	15.1104289	14.2476463	12.5889513	22.7756947	20.5647812	12.1837973
4.07411016	3.72750633	3.21873737	4.05197203	4.01655538	4.6566518	4.39294752
0	0.12024214	0.04733437	0.03093108	0.1825707	0.10068436	0
52.5165941	42.7260403	45.67767	53.6654311	50.2525849	49.5618779	140.883281
81.1930727	81.5241708	84.1605153	78.3175053	76.9991923	74.5064289	48.5209039
481.375828	486.860424	483.520621	502.135221	576.877766	514.34607	580.157598
25.6537517	23.767863	20.2117773	19.9196182	21.9541265	21.8233358	7.68880477
24.3920918	25.3310108	23.3831803	23.8169349	23.5972628	19.5579376	10.5866548
60.1654074	61.8846213	56.0912321	60.2537521	57.8749116	44.7038573	28.3729595
2.31304318	1.88379352	2.08271241	2.19610698	2.82984583	2.4667669	1.1411622
23.0515781	22.2848766	21.821146	21.2805859	20.9956304	19.3565689	13.6225392
1.52450574	1.5230671	2.03537804	1.63934746	2.09956304	2.29056927	2.15471452
15.5604723	14.9901868	20.685121	14.7541272	14.9251546	16.2101825	39.0459419
0.36798414	0.6012107	0.37867498	0.83513927	0.31949872	0.57893509	0.30140581
1.39308283	1.2024214	1.13602495	1.14445012	0.86721082	1.05718582	0.85850766
0.10513833	0.12024214	0.23667187	0.18558651	0.04564267	0.02517109	0.14740879
4.99407051	4.92992773	8.28351529	5.25828432	5.65969167	4.63148071	4.78775307
0.5782608	0.36072642	0.5206781	0.46396626	0.91285349	0.60410618	0.67465705
66.6839836	68.3776968	73.7942876	64.3985174	94.9824061	71.1586738	34.5943746
0.05256916	0.20040357	0.04733437	0.18558651	0.09128535	0.17619764	0.02493006
182.704127	204.53188	178.355918	184.627642	151.259824	180.199839	79.5937565
62.8990039	69.9408446	64.9900942	64.0273443	61.6632536	57.2642316	46.8025701
111.972318	107.456392	107.070352	91.5250782	117.986314	89.2063459	118.132029
1.39308283	0.72145284	0.85201872	1.29910554	0.77592547	1.71163418	2.99234014
12.4063225	10.2606626	10.6502339	12.3415026	11.6845247	12.9631118	8.38942602
10.3561252	8.61735335	12.1175995	10.3619132	7.85054005	9.18744815	7.18372295
109.054729	92.2658019	85.8172184	106.526654	83.8455935	69.270842	37.6212759
26.5999966	30.5815842	32.9447237	31.9827411	20.8587024	28.0657663	18.604565
0.42055331	0.36072642	0.23667187	0.18558651	0.13692802	0.37756636	0.20418998
1.44565199	0.80161427	0.80468434	0.74234602	0.68464012	1.00684363	0.90642993
55.5656056	51.7842815	50.9317854	58.1813694	49.9787288	64.0855973	20.2933534
13.9045437	11.6634876	11.7389245	12.8364	13.2363757	15.4298787	14.4452005
31.3575059	34.2690098	36.920811	33.0962601	29.3938825	31.6148901	38.8250761
0.97252952	0.72145284	0.85201872	0.92793253	0.91285349	1.30889672	1.01856254
0.5782608	0.64129141	0.66268122	0.61862168	0.82156815	0.90615927	1.2475801
14.2725278	13.1464739	11.928262	16.1150949	13.6928024	13.8189289	17.9808168
0.26284582	0.12024214	0.33134061	0.18558651	0.1825707	0.12585545	0.30262157
4.91521677	4.44895917	2.88739676	5.44387082	3.788342	5.18524471	3.9885322

16.4015789	19.6395495	25.2765552	20.8784819	15.9292935	17.5945925	9.21626551
0.68339912	0.84169498	0.66268122	0.71141494	0.45642675	0.52859291	0.88417989
131.238916	130.663125	140.535754	135.045114	120.177163	114.880859	89.190352
6.70256832	6.3327527	6.34280599	7.67090889	6.52690249	7.47581398	6.77896083
0	0	0.04733437	0	0	0.10068436	0
4.4420943	3.44694134	5.20678104	4.63966263	4.60991015	5.81452198	1.4684363
7.33339828	6.3327527	6.91081847	8.41325491	6.29868911	8.43231543	17.4191032
13.19486	11.9039718	12.7329464	15.6201975	9.67624704	7.27444525	25.6614911
0.86739119	0.80161427	0.75734997	0.49489735	0.3651414	2.59262236	0.81589148
1.55079032	1.44290568	1.18335933	1.7630718	1.23235222	2.03885836	1.45035089
65.6063158	66.2934997	77.3443656	66.9039352	65.9993077	60.1589071	16.1723406
3.62727227	3.68742562	4.02342171	3.00031517	3.33191526	3.47361054	0.89297419
21.0802345	23.2868944	17.513718	25.8583864	12.2322368	15.1278256	18.1280425
13.6154133	13.8278461	13.4902963	12.4652269	11.228098	13.2651649	43.6580199
12.6954529	13.8679268	11.5022527	13.0529175	14.3774425	14.699917	11.373241
0.05256916	0.12024214	0.04733437	0.12372434	0.13692802	0.10068436	0.04918324
0.65711454	0.44088785	0.66268122	0.43303518	0.45642675	0.47825073	0.57009826
4.10039474	3.68742562	4.49676544	2.04145156	7.02897191	6.39345707	6.08362948
21.1590882	21.242778	24.992549	20.6619643	18.2570699	22.175731	25.2399815
53.5679774	53.1470258	57.7006008	45.5614871	52.5347186	53.488568	15.1092732
10.6452556	11.7837297	9.94021834	13.4550216	10.4065298	12.1072947	3.44867171
0.05256916	0.08016143	0.09466875	0.12372434	0.13692802	0.10068436	0.17962872
3.07529605	2.08419709	2.31938428	3.12403951	2.41906176	3.32258399	3.6224982
7.54367493	8.65743406	9.89288397	8.815359	5.97919039	7.97923579	2.86674944
18.8197605	15.9922046	15.5256744	22.1775874	14.7425839	19.3817399	30.7985764
0.05256916	0.20040357	0.09466875	0.06186217	0	0.10068436	0.02822962
40.5571095	39.6799061	40.8022296	39.282477	37.2444226	35.6170935	36.3065604
0.26284582	0.32064571	0.18933749	0.34024193	0.09128535	0.07551327	0.31006308
1.23537534	0.9218564	1.51469994	0.9897947	0.91285349	1.05718582	1.69418886
0.05256916	0.04008071	0.04733437	0	0.04564267	0.22653982	0.07332621
0.15770749	0.28056499	0.18933749	0.21651759	0.41078407	0.276882	0.56081759
0.05256916	0.04008071	0.04733437	0.03093108	0	0.02517109	0.04345073
41.845054	39.8803097	42.6482701	40.0866852	46.1447442	48.3284944	24.7406662
0.07885374	0.08016143	0.09466875	0.15465542	0.04564267	0.17619764	0.05282619
27.2833958	30.2609385	28.7319644	25.7346621	29.9872373	23.560141	28.1529956
63.1881343	75.2715795	70.291544	64.3366552	70.4266471	57.2390606	26.9187234
95.8335847	94.9912904	93.0120431	99.1959871	102.057021	105.189989	150.586786
0.18399207	0.12024214	0.28400624	0.30931084	0.09128535	0.40273745	0.34706984
1.13023701	1.44290568	0.94668746	1.29910554	1.50620827	1.48509436	1.85335428
0.68339912	0.6012107	0.61534685	0.34024193	0.63899745	0.52859291	0.66533393
27.4673878	27.6156114	26.1759083	34.4572278	24.9665431	29.4753474	28.9036138
496.305471	552.432471	478.171837	411.228765	518.637713	455.470889	240.302068
0.05256916	0.04008071	0.04733437	0.09279325	0.13692802	0.05034218	0.11842925
2.41818151	2.56516565	2.55605615	2.3507624	1.41492292	2.44159581	1.69617307
0.21027665	0.24048428	0.33134061	0.24744867	0.3651414	0.42790854	0.12304632

15.0873499	15.9120432	14.6263213	13.578746	14.3774425	13.6175601	13.8559556
58.1677792	59.5198592	66.0787848	59.2020952	55.7297058	54.9233202	34.4163423
0	0.04008071	0.04733437	0.03093108	0.04564267	0.07551327	0
0.05256916	0.04008071	0.04733437	0.03093108	0	0.02517109	0.23049134
0	0.04008071	0.14200312	0.03093108	0	0.02517109	0
48.7053298	40.0807133	35.0747704	46.272902	39.0701296	51.0469722	40.4896451
17.6632389	14.6695411	14.5789869	15.6201975	11.9583808	14.5488905	29.0419086
16.585571	14.9100253	13.064287	14.4448163	11.365026	14.2216663	15.9817919
8.38478155	10.2606626	19.8804367	13.578746	11.228098	21.7729936	12.7178919
9.64644146	16.633496	10.6975683	10.8568106	12.2322368	20.2878992	4.97905055
7.5962441	10.9821154	8.14151217	11.6919498	10.4065298	8.35680216	7.54557332
79.2217291	74.7505302	60.9666725	72.7808412	64.3105287	59.7058275	60.8163004
23.2881393	26.0925443	23.477849	23.2911064	24.5101163	18.7524627	9.25386972
0.02628458	0.20040357	0	0.09279325	0.09128535	0.20136873	0.01821214
31.4363597	34.3090906	35.3587767	29.8794274	32.4062991	30.9100995	18.2773935
27.3096803	23.0063294	26.7912552	25.1160404	21.2694864	24.1894183	27.2159102
80.798804	91.4641877	103.898949	93.8139785	93.8869819	114.90603	247.099968
0.02628458	0.08016143	0.04733437	0.03093108	0	0.02517109	0.02280393
5.65118505	5.57121914	6.05879975	6.06249251	5.84226237	7.55132725	4.86318077
26.1005896	28.5775486	28.4479582	32.8797425	15.8380081	23.9377074	23.4854482
0.02628458	0.08016143	0.04733437	0.09279325	0.09128535	0.07551327	0.023791
19.7660054	20.4812445	35.3587767	15.6511286	25.0578284	24.390787	17.407821
7.93794366	8.17646551	9.79821522	8.87722117	10.3152445	9.71604106	4.66732496
47.0231166	47.1349188	43.263617	47.5101454	54.862495	46.3903204	16.5271329
487.132152	525.177586	473.107059	470.338067	466.011709	418.318359	261.762174
120.541091	108.217926	118.288598	140.458053	105.708435	133.885032	138.716973
0.78853745	0.88177569	0.66268122	1.05165686	0.27385605	0.37756636	0.79489596
0.42055331	0.6012107	0.56801248	0.5876906	0.5477121	0.830646	0.27733513
55.6444593	49.2992773	60.1619882	61.7693752	58.513909	58.6738127	95.6025951
6.09802294	6.29267198	6.86348409	6.03156142	6.11611841	4.90836271	6.64554269
35.4841852	36.7940948	38.2935078	37.8596471	38.6593455	42.8411966	27.8073551
44.7626425	43.2070089	40.0448796	43.334449	41.8999754	31.9924565	15.1263761
6.09802294	5.41089629	5.91679663	6.34087227	5.84226237	7.4254718	9.036489
153.764803	147.537106	154.736066	177.389768	146.147844	174.284633	141.374084
0.65711454	0.88177569	0.61534685	0.5876906	1.14106687	1.03201472	0.29871369
36.0361614	31.3831985	27.4539364	33.7148818	37.0162092	33.2258399	19.969425
82.244456	80.2015072	73.5102814	84.2562734	84.5302336	85.1034581	55.0374354
174.293061	185.533622	188.154133	168.790927	179.603925	153.392628	67.3756154
372.452522	406.698997	407.122943	342.438033	410.373289	326.141824	219.309596
0.02628458	0.04008071	0	0	0.1825707	0.02517109	0.08785121
0.18399207	0.24048428	0.23667187	0.12372434	0.13692802	0.17619764	0.21714886
0.36798414	0.40080713	0.14200312	0.15465542	1.18670954	0.20136873	0.26386259
71.6517696	72.5861717	68.256166	78.7196094	66.7295905	85.4055112	38.6381109
70.0221255	70.1011675	66.2207879	76.7709511	63.8997446	83.7442192	37.7343767
166.696817	176.194815	198.993704	179.493082	230.039081	184.453754	214.650275

12.7743067	12.585344	13.4429619	11.1970525	12.8712343	11.1759643	18.9758607
47.5488082	44.4094303	49.6537573	47.5720075	38.9788442	44.0745801	51.0449819
0.39426872	0.68137213	0.56801248	0.40210409	0.45642675	0.37756636	0.34157445
16.4015789	29.4192435	22.7204991	31.4259816	23.6429055	22.6791528	20.5466564
0.26284582	0.12024214	0.04733437	0.09279325	0.1825707	0.02517109	0.13802765
4.94150135	5.731542	5.77479351	4.91804239	7.85054005	6.21725944	1.81364112
6.59742999	5.25057344	4.9227748	7.29973588	4.8837662	7.09824762	6.78481628
217.426059	211.946812	214.898054	214.940104	183.894336	193.918084	210.75602
268.549571	276.116034	319.128343	277.389963	266.142436	247.180112	340.427705
726.768683	821.494299	781.727171	726.478375	772.456627	604.685115	241.555611
310.920316	332.629839	327.080518	305.908423	344.373981	223.670313	92.7555773
36.2727227	39.3592604	28.3532895	34.5809522	46.0078161	34.9626452	43.2685398
103.140698	103.849128	101.153555	103.124235	115.110826	115.333938	187.753717
15.9021719	13.3869582	15.0049963	16.0223016	15.9749362	16.6884332	12.4180611
43.6324055	45.8122553	39.9028765	45.6233492	51.5762224	43.2439341	37.5879431
2.31304318	2.52508494	1.42003119	2.47448674	1.32363757	1.25855454	1.09184321
11.9857692	11.5432454	11.7862589	13.578746	12.2322368	13.0386251	10.7888263
3.49584936	4.72952416	3.92875296	3.77359228	4.38169677	5.94037744	4.9069662
63.0304268	61.8044598	67.5934847	62.3261347	57.8292689	57.8935089	59.1516632
0.02628458	0.08016143	0	0.03093108	0.04564267	0.12585545	0.09429191
4.17924848	4.88984702	5.58545602	3.61893685	6.75511586	6.8717078	1.57668871
0.42055331	0.56112999	0.47334373	0.80420819	0.13692802	0.98167254	0.28983474
11.1709472	11.0622769	11.9755964	11.9393985	11.5475967	14.2468374	8.78794548
34.0911024	42.5256368	41.5595795	36.8079902	38.9332015	32.3196806	22.0534868
0.36798414	0.48096856	0.5206781	0.30931084	0.22821337	0.42790854	1.84110259
83.4798313	79.2395701	88.2312714	85.7100344	75.0821999	71.6117534	56.0788843
9.8830027	8.7776762	8.37818403	7.79463323	8.21568145	8.35680216	20.1495528
145.196029	162.928099	147.351903	154.655421	141.172793	120.695381	84.0189992
26.7577041	29.1386785	28.7792988	24.2190389	26.0619673	27.6630288	58.6600456
21.8162028	14.8699446	14.6736556	14.7850583	15.1077253	16.084327	13.2608396
239.321116	219.882793	207.703229	250.387127	258.154968	219.391228	166.576385
9.01561151	10.340824	9.32487149	9.92887804	10.6803859	11.2011354	5.10114005
7.17569079	6.57323697	7.85750593	7.08321829	7.66796935	9.46433015	2.13991148
2.52331984	3.28661849	2.88739676	3.03124625	3.87962735	3.22189963	3.60389959
39.2165958	34.1888484	29.8679894	37.828716	37.88342	37.127359	24.2104891
4.10039474	2.84573064	2.60339052	3.86638553	3.51448595	3.90151908	4.37310593
45.1043421	44.5697531	45.5830013	42.8395517	44.4103225	38.1090315	35.1324276
0.52569163	0.28056499	0.28400624	0.27837976	0.27385605	0.276882	0.49505068
0	0.04008071	0.14200312	0.03093108	0	0.02517109	0
13.5365595	17.5152717	17.4190493	13.1457108	14.0579438	16.1598403	39.5676797
49.2835906	49.7802459	48.9437417	51.8095661	46.0991015	37.9076628	17.1882796
0.26284582	0.40080713	0.42600936	0.12372434	0.1825707	0.40273745	0.32994912
0	0.04008071	0.04733437	0.03093108	0.04564267	0.07551327	0
23.2881393	21.8840694	20.1644429	27.4358717	21.1782011	23.0567192	14.2654171
37.4555288	35.4714312	30.5306706	34.9830563	32.5432271	46.8685711	20.8551647

67.6302286	65.9327733	69.534194	63.8726889	66.1362357	58.0948776	51.1474886
59.7448541	54.2692858	47.1923699	61.8930995	50.663369	63.7583731	105.767005
10.1721331	10.7416312	11.6915901	10.26912	10.3608872	8.65885525	6.35841627
46.7339862	42.766121	43.7842951	46.0254533	43.8626104	54.4450695	225.074792
0.07885374	0.04008071	0.09466875	0.09279325	0.13692802	0.12585545	0.02967106
0.44683789	0.32064571	0.28400624	0.80420819	0.27385605	0.37756636	0.37710259
102.904137	91.4241069	97.698146	87.3803129	93.2023418	90.0369919	58.6584882
0.21027665	0.40080713	0.09466875	0.12372434	0.09128535	0.22653982	0.07149203
14.1411049	14.7096218	13.9163057	13.9808501	12.9168769	11.8807549	13.4410299
0.02628458	0.04008071	0	0.06186217	0	0.07551327	0.0896891
0.18399207	0.12024214	0.23667187	0.18558651	0.04564267	0.10068436	0.26050323
331.816559	327.459427	323.719777	304.145351	330.635536	263.74269	97.2696646
123.879233	114.711001	114.691186	129.137277	122.459296	130.663133	173.983515
0	0.08016143	0.09466875	0.03093108	0	0.02517109	0
28.2559253	37.7961126	38.5301797	33.2199845	23.0495507	36.6239372	29.6441315
19.9499975	13.4671197	17.1823774	12.3105715	16.1575069	16.5877489	35.7313882
0.65711454	0.64129141	0.37867498	0.9897947	0.68464012	0.553764	0.57050287
0.52569163	0.24048428	0.7100156	0.71141494	1.46056559	0.12585545	0.63170023
4.1529639	4.48903989	4.82810605	3.37148818	3.6057713	4.60630962	6.63219267
10.1984177	9.94001689	8.89886214	7.14508046	7.66796935	10.6977136	7.95889899
11.5126468	11.0221961	11.2655808	12.1559161	12.0953088	13.013454	5.68675601
26.7839887	32.986427	29.5839832	22.1466563	40.9414792	38.3355713	8.94470624
100.196825	107.255989	123.211373	106.155481	100.68774	96.9086997	149.201827
0.81482203	0.9218564	1.42003119	0.83513927	1.73442164	1.38441	0.71120927
91.4703441	83.5282064	79.1430718	85.7100344	84.4845909	62.5753318	53.6020719
0	0.08016143	0.09466875	0.03093108	0	0.02517109	0
3.46956478	3.12629563	3.31340611	2.3507624	3.01241653	3.17155745	6.21870696
14.1411049	14.1484918	12.0702651	13.207573	15.2446534	16.3863801	10.1043256
13.6154133	10.1003397	11.3129152	13.7334014	11.365026	14.0706398	25.1974805
60.2442611	74.510046	62.1500318	64.986208	62.3478937	53.0606595	20.9541989
6.33458418	6.0521877	7.05282159	5.2892154	5.56840632	6.29277271	8.05509724
24.6023684	22.6055223	18.9810836	24.7448674	24.3275456	23.6356543	14.2330173
0.55197621	0.84169498	1.2306937	0.34024193	0.59335477	0.95650145	0.2832317
9.06818067	8.61735335	7.71550281	7.51625347	7.57668401	7.72752489	4.51484407
9.93557186	9.53920976	10.6975683	11.2898457	12.0496661	10.2194629	9.77798774
129.057296	114.029629	149.103275	127.126756	109.314206	121.878422	71.4846682
5.28320091	4.08823275	3.92875296	4.1138342	5.84226237	5.58798217	7.11768929
5.70375422	8.65743406	8.33084966	6.09342359	7.57668401	6.14174616	5.29788804
7.56995951	6.45299483	7.81017156	6.37180335	9.72188972	8.73436852	24.6282447
301.668144	374.353862	366.415382	313.23909	342.228775	294.67796	142.591267
48.6790452	48.5377438	45.6303356	47.2936278	52.4434333	54.8981491	121.403383
11.3549393	13.2266354	11.2655808	13.8261946	13.4189464	12.3590056	10.1872607
13.510275	13.6274425	14.0583088	13.3003662	10.9542419	12.8875985	20.0935203
0.07885374	0.04008071	0.09466875	0.15465542	0.09128535	0.10068436	0.10688158
13.1685754	15.1905903	14.342315	11.753812	14.6056559	12.2583212	6.22086286

9.40988023	9.57929047	7.3841622	9.31025635	10.0413884	10.244634	4.20491979
62.3995968	65.2113205	56.4225727	61.7693752	58.650837	48.7312319	28.2294778
17.9786538	17.5553524	20.7797898	20.4145156	13.5102317	16.361209	11.7139933
218.503727	217.037062	211.347976	202.567671	222.964466	166.078857	168.745197
2.1553357	1.40282496	4.11809046	1.79400289	3.69705665	4.27908544	1.63814254
0.23656123	0.20040357	0.23667187	0.37117301	0.1825707	0.35239527	0.28273754
3.20671896	3.84774847	2.13004679	4.23755854	3.4232006	3.24707072	1.59883109
71.8094771	70.1412482	73.2736095	84.7511708	80.5136782	67.4836945	45.7875819
0.18399207	0.24048428	0.28400624	0.18558651	0.04564267	0.20136873	0.23654734
50.9132347	47.9365331	53.1091666	53.2942581	51.0285103	60.1589071	21.8607969
48.7316144	55.1911422	52.5884885	50.324874	49.6592301	44.5528308	51.1916413
0.02628458	0.04008071	0	0	0.09128535	0.07551327	0.02154341
43.2644214	52.3454115	52.777826	57.2225058	45.8708881	59.0513791	50.6699182
0.02628458	0	0	0	0.13692802	0	0.04676879
0.02628458	0	0	0	0.13692802	0	0.04135504
12.0120538	10.6614697	12.685612	15.0943691	9.81317507	14.699917	18.3104904
0.73596829	0.44088785	0.37867498	0.40210409	0.27385605	0.50342182	1.5589596
0.60454538	0.88177569	0.94668746	0.64955277	1.23235222	1.15787018	0.34001446
0.18399207	0.04008071	0.18933749	0.03093108	0.04564267	0.15102655	5.69809599
0.34169956	0.20040357	0.14200312	0.21651759	0.22821337	0.553764	0.32018081
0	0.04008071	0.04733437	0.03093108	0.13692802	0.02517109	0
0.10513833	0	0.09466875	0.06186217	0.3651414	0.02517109	0.15148901
0	0.04008071	0.09466875	0.06186217	0	0.15102655	0
77.1715317	88.3779727	64.8480911	63.4087227	73.1652076	74.2798891	27.64762
22.9990089	19.5193074	22.6258303	19.1772722	20.5392036	19.9858461	44.9430495
0	0.04008071	0.04733437	0.03093108	0.13692802	0.02517109	0
0.02628458	0.08016143	0.04733437	0	0	0.05034218	0.05422182
51.0446576	51.583878	40.3288858	57.5008856	48.0160938	50.8204324	24.0624963
106.373702	116.234068	108.869058	98.5464343	115.293396	91.0186645	50.2608664
1.31422908	0.9218564	1.7513718	1.33003662	1.14106687	2.44159581	1.89920506
0.49940705	0.84169498	0.56801248	0.55675952	0.41078407	0.47825073	0.20982905
258.377438	259.723022	278.515451	259.913901	234.648991	238.672283	206.446319
377.210031	373.71257	381.136372	362.202996	364.958827	309.679931	253.257671
71.1786471	67.2153561	58.9312945	75.5337077	68.7378681	69.547724	30.3016499
26.5474275	27.2949657	27.9272801	26.9719054	30.3523787	31.2121526	20.0773274
175.60729	176.675784	165.433634	168.172305	163.400776	143.953468	80.8649772
6.28201501	5.01008916	6.72148097	6.95949395	5.61404899	7.80303816	10.9938433
26.6788504	33.2269113	28.6846301	32.2611208	32.132443	31.9924565	44.7066367
23.4195622	23.8881051	22.1524866	25.2088336	21.6346278	28.9719256	10.3818828
2.78616565	3.80766776	1.46736556	2.84565975	1.87134966	1.81231854	62.5371636
0.02628458	0	0.04733437	0.06186217	0	0.07551327	0.01893055
35.4053315	38.3973233	41.8909202	35.261436	35.3274302	36.1960286	58.4097379
0.02628458	0	0.04733437	0.06186217	0	0.07551327	0.07556649
56.669558	59.2392942	67.120141	58.4288181	56.2774179	54.3695562	60.5970593
13.2211446	14.7497025	13.2536245	15.4036799	12.3235222	8.65885525	4.6622172

0.7096837	1.00201783	1.08869058	0.9897947	0.59335477	0.57893509	0.81477769
87.711649	73.7084317	80.7524404	88.3082455	84.6671616	87.6960805	117.908097
56.8535501	52.9065415	53.5351759	57.0059882	59.7462612	59.2527478	41.5336582
2.44446609	1.5230671	2.08271241	1.97958939	1.50620827	1.58577872	3.82388667
33.6179799	28.2168221	29.8679894	30.6527045	42.4020448	28.7202146	31.002171
42.0027615	39.1989376	39.9975452	43.4272423	43.4518263	43.4704739	82.0133433
153.817372	159.120432	148.582597	155.243112	175.76994	170.383114	36.7361433
61.1905061	68.7384232	68.2088316	57.8720586	66.2731637	59.1772346	46.1773257
155.920138	156.475105	157.339456	175.936007	160.890428	172.82471	172.767037
0.13142291	0.12024214	0.04733437	0.06186217	0.04564267	0.07551327	0.16985495
0.05256916	0.04008071	0.04733437	0.12372434	0.04564267	0.07551327	0.07614812
0.07885374	0.16032285	0.04733437	0.30931084	0.1825707	0.02517109	0.04814606
68.103351	71.5841539	66.362791	69.1000422	74.8996292	78.156237	105.72313
82.0341793	78.7986823	86.0538902	76.8946754	81.0613903	71.8886354	72.6919258
63.3984109	64.0088991	64.0907411	66.2853135	82.8414546	60.1085649	23.8959613
0.02628458	0.04008071	0.09466875	0	0.04564267	0.02517109	0.02314037
10.8555322	11.743649	12.3542714	10.3619132	9.35674832	11.6038729	19.4712796
0.05256916	0.04008071	0.04733437	0	0	0.05034218	0.02186944
114.732199	118.478588	114.217842	112.063318	107.169	102.773564	111.877409
169.114998	174.110618	197.005661	222.363565	206.715674	163.461064	29.869461
38.8748963	43.2470896	43.5949576	39.4989946	41.8999754	34.7612764	53.6134026
0.9988141	0.56112999	0.61534685	1.05165686	0.63899745	1.08235691	0.57565823
18.7409067	17.3148681	17.1823774	17.2904761	14.5600132	16.638091	7.84179074
169.772113	180.162806	192.224889	178.441425	177.276149	138.138946	98.0083651
1.13023701	1.04209854	0.80468434	1.36096771	0.82156815	1.45992327	2.10281717
11.1972318	9.73961332	11.1235777	9.61956719	8.99160692	10.244634	7.94974391
2.20790486	1.48298639	1.60936868	1.42282987	2.00827769	3.12121526	2.71174448
0.02628458	0.08016143	0.04733437	0.06186217	0.09128535	0.10068436	0.03156951
0.02628458	0	0.04733437	0.09279325	0	0.05034218	0.01622278
90.8657988	86.9751478	97.1774679	78.3484363	106.073576	64.1862816	118.24978
17.4266776	18.8379352	19.5017617	16.7646477	18.0744992	16.1094981	9.37252061
0.44683789	0.80161427	0.89935309	0.52582843	0.45642675	0.60410618	0.46127444
15.3764803	17.2747874	14.7683244	15.4036799	14.5600132	13.1393094	21.3142458
0.05256916	0	0	0.06186217	0	0.07551327	0.08655058
37.2452522	34.8301398	33.2287299	33.3437088	31.8129443	32.3700228	29.8764384
1.10395243	0.6012107	0.7100156	0.80420819	1.18670954	1.13269909	0.72585259
69.6015722	70.7424589	69.912869	71.1724248	63.3976752	60.1085649	30.1389658
261.163603	251.787041	232.601109	265.110323	211.964581	203.961349	125.38367
9.77786437	9.29872548	6.1534685	10.4856376	9.81317507	8.55817088	7.3067303
18.9774679	14.2286532	17.0403743	20.4763778	17.0247177	18.1231854	13.3957629
17.8735155	15.8719625	17.4190493	17.5069937	12.9168769	15.7571029	11.7298328
256.905501	314.032388	293.615116	248.345675	274.814545	269.154474	943.749899
13.7468362	14.1484918	15.099665	13.8571257	13.3733037	11.7800705	14.4222147
70.1009793	69.7003604	65.5107723	75.3790523	63.8084593	83.693877	37.1468467
54.2776611	53.6279943	61.8660256	51.9951526	67.7337293	65.5455205	130.318084

86.7391194	90.6224927	81.9831341	93.9686339	91.4679202	92.8058119	70.7142203
6.83399123	11.6634876	8.47285278	13.0838486	11.9127381	6.77102343	10.2638684
13.0634371	12.1043754	12.8276151	14.3520231	14.2861572	13.2148227	17.9352528
8.27964322	6.17242984	7.52616532	9.12466985	6.98332923	8.58334197	24.143899
0.31541498	0.32064571	0.28400624	0.21651759	0	0.35239527	0.93651092
1.23537534	1.04209854	1.32536245	1.42282987	1.50620827	1.40958109	1.09270894
23.1567164	21.9642309	23.0045053	22.3322428	25.4686125	26.328961	70.4658463
24.3395226	22.6856837	22.1051522	24.4664876	22.86698	27.8643976	18.1108148
3.96897183	3.24653777	4.73343731	4.63966263	3.9709127	5.03421817	15.3888583
86.9493961	58.5980028	84.6811934	88.7103496	56.6425593	58.7996682	43.2901571
79.5897132	66.4538226	57.9372726	78.7814715	63.0781765	82.586349	89.5272866
0.15770749	0	0.04733437	0.03093108	0.41078407	0.05034218	0.14112747
29.7541464	33.547557	33.6074049	30.838291	28.6635997	29.651545	43.8263807
2.31304318	1.8036321	1.27802807	3.24776384	1.78006431	1.71163418	1.17358427
32.3300354	38.1969197	44.3996419	35.2923671	33.7755793	35.8939755	58.0320708
19.4505904	21.4431816	23.5725178	20.7547575	18.8960673	19.130029	19.486995
32.7505887	35.4714312	33.2760643	33.5292953	30.4893067	29.7522294	18.7474557
39.2954496	35.0305434	26.2232427	26.6625946	25.8337539	37.3035566	61.4700062
494.255273	517.722573	520.725438	490.597927	505.44698	398.206657	219.421229
14.1673895	14.2286532	14.8156588	13.8571257	11.501954	13.7182445	21.2516971
2.07648195	2.52508494	0.61534685	1.60841638	0.95849617	1.45992327	1.03215351
26.7839887	25.3310108	28.0692832	25.9202486	21.8171985	25.9010525	25.3007405
18.2940688	20.1205181	17.5610524	17.7544423	16.6139336	16.8142887	11.7628725
24.7863605	22.8460066	25.1345521	23.0436577	24.0536896	19.8096485	10.7711509
11.5652159	15.4310746	19.5017617	13.3312973	12.6886636	14.9264569	5.51776846
9.8830027	8.49711121	8.18884654	8.78442792	9.58496169	11.4276752	10.4582454
4.99407051	4.60928202	4.78077168	4.08290312	3.788342	4.73216508	13.9638238
2.83873482	4.00807133	3.92875296	3.5261436	5.02069422	3.97703235	0.76988859
2.54960442	2.04411638	3.21873737	2.13424481	2.37341909	2.11437163	9.5818542
84.0318075	90.1816048	89.2252932	89.4526956	81.9286011	72.9458212	30.1583901
31.541498	31.864167	27.9746145	33.4674331	33.3647952	32.143483	25.7829937
21.737349	19.4391459	24.5665396	19.0535479	20.8587024	20.1368727	33.680784
33.8545412	32.2248935	30.6253394	32.0136722	29.0287411	28.1664506	21.0703443
0.10513833	0	0.09466875	0.15465542	0.1825707	0.05034218	3.95735898
30.7003914	27.53545	28.4479582	32.2301898	28.6635997	33.2258399	8.70747431
336.863198	376.51822	366.557385	323.941245	370.527233	291.657429	183.105883
1.05138327	1.08217926	1.13602495	0.71141494	0.45642675	1.30889672	2.76292517
26.9416962	29.3791628	25.1345521	27.528665	28.9374558	31.8917721	46.4188454
50.7555272	51.303313	49.0384105	49.3041483	59.7006185	54.6464382	25.1523148
111.81461	134.150147	107.638364	113.82639	117.57553	91.6479417	62.6144044
27.8616565	23.2868944	27.9272801	33.6220886	23.3234068	29.9787692	30.2823227
0.49940705	0.36072642	0.28400624	0.5876906	0.41078407	0.52859291	0.42931644
33.7756874	37.5155476	32.5187143	33.3437088	33.7299366	28.6447014	18.9877303
20.8699578	24.0885087	21.5844741	23.2911064	17.3898591	19.6586219	14.3911568
355.078413	335.315247	353.871773	333.406157	335.519302	289.668913	291.127476

45.8928796	45.291206	44.3049732	38.8803729	45.323176	41.7084975	32.7156665
0.73596829	0.24048428	0.56801248	0.49489735	0.31949872	0.35239527	0.67539322
0.05256916	0.04008071	0.04733437	0.03093108	0.1825707	0.07551327	1.9486995
0.94624494	1.00201783	0.61534685	1.02072578	0.41078407	0.553764	37.9517213
12.7480221	11.9440526	14.0583088	10.7330862	12.7799489	11.629044	12.4061
3.60098769	3.08621492	2.84006238	2.938453	4.29041142	3.97703235	3.67239284
36.6144222	38.1167583	43.9262982	39.2206148	31.995515	37.6559519	36.1063148
0.05256916	0.04008071	0.04733437	0.03093108	0.1825707	0.07551327	1.08995057
0.02628458	0.04008071	0.09466875	0.06186217	0.13692802	0.07551327	0.02155786
1790.71598	1853.41234	2131.60882	1784.5689	1929.3615	1446.02883	1256.41324
4.62608637	4.88984702	5.68012477	5.32014649	4.19912608	3.64980817	3.5580153
59.1140241	63.2072848	67.5934847	57.3462302	72.8457089	76.9228536	35.552269
15.3501957	14.8298639	15.2890025	14.7850583	17.0247177	18.0728432	28.6244944
0.73596829	0.36072642	0.66268122	0.55675952	0.41078407	0.40273745	1.37240727
74.8584885	84.6905471	96.4201179	74.0799467	88.546789	72.870308	54.6706189
0.05256916	0.04008071	0.04733437	0.06186217	0	0.17619764	0.07070597
0.68339912	0.76153355	0.47334373	0.86607036	0.59335477	0.93133036	0.55108245
8.98932692	3.84774847	4.35476232	3.15497059	7.12025726	3.72532144	14.662015
0.02628458	0.04008071	0.09466875	0.03093108	0.04564267	0	0.02079117
0.9988141	0.9218564	0.80468434	1.33003662	1.27799489	0.830646	1.84846382
0.10513833	0	0	0.06186217	0.09128535	0.10068436	0.05749404
37.2978214	35.0706241	33.749408	50.4795295	26.1076099	42.941881	61.7400212
0.07885374	0.08016143	0.09466875	0.12372434	0	0	0.1956605
19.5820133	20.4812445	17.6557212	18.9298235	17.3898591	16.5122356	11.5693738
22.1316177	21.6836659	18.7917461	20.0433426	23.4146921	25.2969463	10.6650707
130.240102	112.426401	118.572605	130.188934	121.683371	139.850581	211.441007
1.83992072	1.96395495	1.32536245	2.19610698	1.27799489	2.71847781	3.75437518
2.99644231	2.60524636	2.65072489	3.18590168	2.92113118	3.42326835	1.21173678
0.05256916	0.08016143	0	0.03093108	0	0.02517109	0.0477587
0.02628458	0.16032285	0	0.12372434	0.09128535	0.10068436	0.07043492
30.8318143	30.0204542	35.2641079	32.6013628	32.8170831	38.9145064	127.634871
4.96778593	9.33880619	6.29547162	8.84629009	6.11611841	8.7847107	5.9403904
44.1843817	43.046686	42.2695951	44.8500721	36.8336385	38.1342026	35.7947706
40.6885324	42.1649103	49.606423	42.22093	44.6385359	34.7361054	37.9806811
0.49940705	0.28056499	0.5206781	0.5876906	0.31949872	0.75513273	20.3639097
53.0160012	44.9705603	50.1271011	58.5525424	47.5596671	54.7722937	48.7438509
41.214224	46.4535467	47.6183793	41.9734813	56.3230606	48.9326006	19.6174138
65.0017704	59.960747	61.4400162	64.2438619	55.9579192	51.9531315	47.8433866
16.1913023	17.0743838	14.3896494	15.6201975	14.6969413	13.0386251	14.0223587
51.018373	56.8745321	59.0732976	46.7059372	61.4806829	44.2004355	21.7532326
7.25454453	7.61533552	5.77479351	6.89763178	8.26132413	8.13026234	6.99045097
10.8029631	10.7817119	10.0822215	10.8258795	10.9998846	13.1896516	24.3596417
214.245625	212.868668	197.715676	213.177032	196.811213	163.13384	83.8935718
21.3956495	19.6395495	18.6970774	20.9094129	23.0495507	21.345085	17.5304641
0.02628458	0.40080713	0	0.27837976	0.5477121	0.02517109	0.06522017

18.9511834	11.6634876	14.72099	13.5478149	10.9998846	15.4802209	22.4421138
401.023862	446.218581	412.519061	347.943766	462.405938	329.464408	148.836949
1.44565199	1.08217926	1.60936868	1.60841638	1.36928024	1.93817399	1.3301578
55.7758823	51.3834744	52.5884885	53.0468094	50.2525849	44.4773175	27.1561455
41.8713386	42.4454753	39.713539	41.4785839	37.5182786	36.0701732	32.2446282
6.15059211	6.77364054	6.6741466	6.49552769	7.39411331	7.97923579	6.10460751
70.5741017	67.4157597	80.8944436	70.2754234	82.795812	83.4421661	271.912628
122.854135	138.398703	139.873072	131.79735	122.048512	116.139413	78.1516661
0.65711454	0.24048428	0.28400624	0.40210409	0.31949872	0.17619764	0.49604353
42.2130381	40.7220047	47.3817074	39.7773743	37.0162092	43.5459871	47.1585278
41.2667932	33.3471534	35.0274361	39.7773743	35.4187156	44.2004355	47.2890496
72.8608603	85.131435	91.9233525	67.7390745	79.2356833	83.1904552	70.9348329
102.509868	119.11988	114.359845	109.743487	99.6836016	101.036759	97.700672
15.7444644	16.5132539	18.9337492	15.5583354	15.5641521	15.4802209	9.43884904
81.929041	74.6703688	87.0005777	94.9893597	80.5136782	86.9157767	213.922283
35.0110628	30.5415035	32.3767112	33.1890534	34.3689341	38.5872822	70.3259729
21.6847799	23.1666523	22.4364928	25.0232471	21.5433425	26.4548165	19.4192327
19.5294442	18.1164824	16.0936868	20.940344	18.5309259	19.4320821	13.7694994
21.3956495	20.4411638	19.407093	22.1157252	21.3607718	23.1574036	28.7457253
69.5227185	66.4939033	69.2028534	75.1625347	73.3021356	72.039662	40.9170641
31.226083	28.0164186	23.9038584	30.7454977	28.2528157	31.6652323	21.8588884
59.7711387	68.0971318	73.841622	67.7700055	65.3603102	51.3490253	64.7054458
0.13142291	0.16032285	0.28400624	0.18558651	0.13692802	0.45307964	0.10308926
2.23418944	2.24451994	1.84604055	2.31983132	2.41906176	2.66813563	3.07776018
12.5903146	15.0302675	14.9576619	10.9186727	13.8297304	13.5923891	22.6160741
118.41204	148.659365	134.098279	118.40419	136.654168	112.716145	29.3876456
69.864418	71.0230239	66.1734535	76.3379159	64.0823153	84.0462723	36.4374819
20.1602741	21.0423745	17.8450586	19.2700655	17.7093578	17.1163418	6.21046751
25.6011825	19.3990652	19.9751054	27.6214582	20.493561	23.7615097	17.1932746
1.07766785	1.24250211	1.18335933	1.20631228	1.14106687	1.83748963	1.36540156
3.94268725	7.69549695	6.76881535	5.876906	4.83812352	4.90836271	9.13452889
171.060057	177.798044	173.196471	143.705817	188.686817	144.079324	196.946117
60.7699528	65.0910783	73.2262751	67.3679014	72.7087808	74.8839952	132.27578
186.778237	266.89747	216.933432	189.019856	208.587024	212.796402	162.044729
0.91996036	0.56112999	1.04135621	0.74234602	0.63899745	0.62927727	0.43233729
66.1845766	72.1853646	73.1789408	68.5742137	67.7337293	59.7058275	48.1633658
2.47075067	1.68338996	1.60936868	1.97958939	2.00827769	2.97018872	1.39701082
1.47193657	0.84169498	2.60339052	0.40210409	3.6057713	3.0205309	1.02103676
0.02628458	0	0	0.03093108	0.09128535	0	0.04054671
11.0658089	10.901954	10.7922371	7.48532238	9.85881774	11.7548994	4.35471805
6.7288529	8.33678836	7.66816844	8.16580624	8.80903622	8.30645998	17.3656259
0.02628458	0	0	0.03093108	0.09128535	0	0.02682815
0.34169956	0.12024214	0.28400624	0.12372434	0.3651414	0.05034218	0.56485952
191.877446	198.399531	212.199994	184.163675	202.881689	168.621137	245.139273
0.02628458	0.08016143	0.04733437	0.09279325	0.04564267	0.10068436	0.07477568

52.3588866	43.327251	34.3647548	53.9128798	34.6884328	57.7928246	59.0865821
374.581573	350.345515	324.808468	331.79774	416.71762	401.629925	337.298581
14.4565199	12.2646983	13.1589557	12.496158	11.1368126	12.5855454	23.0116434
40.3468328	41.3632961	35.5007798	38.5401309	34.7797181	35.1388428	35.7390923
46.5237095	43.0867668	40.9442327	46.8296615	45.186248	48.4795209	41.6173812
54.9347756	58.4376799	58.5999539	55.3975719	53.4475721	50.8456035	25.5565325
0.49940705	0.56112999	0.56801248	0.46396626	0.86721082	0.75513273	0.78272555
23.7086926	21.1225359	23.856524	19.8577561	23.6885482	19.5831087	14.8274512
0.60454538	0.32064571	0.56801248	0.49489735	0.31949872	0.32722418	0.54217849
41.2667932	32.9062656	23.3831803	34.3953657	35.8751423	38.8389932	21.9435168
9.04189609	9.85985546	10.5555652	7.14508046	10.6803859	8.43231543	14.2261329
4.12667932	5.85178414	5.58545602	3.68079902	5.43147829	4.35459871	7.96859675
46.050587	42.4855561	50.4111073	44.8810032	48.2899499	33.8802883	16.0584393
459.139072	459.966265	505.957114	483.54564	463.364434	341.143794	292.377963
0.39426872	0.28056499	0.42600936	0.71141494	0.3651414	0.37756636	0.38522614
35.9310231	31.1827949	36.1161266	33.0034669	27.2943195	34.358539	47.7500179
85.7928745	84.3298207	97.7454804	85.7718966	87.5882928	75.1860483	61.1413691
100.091687	108.698894	100.538208	108.846485	96.1691157	78.2820925	28.0216699
30.7266759	35.3912698	32.1873737	31.95181	32.4975844	25.9010525	29.4111817
54.7770682	60.241312	61.3926819	57.7792653	56.0492046	49.4108513	21.9123548
26.889127	26.7338357	27.5959395	26.6625946	34.5515048	27.4113179	18.9749485
32.2248971	37.9965162	38.1041703	34.9521252	40.2568391	42.2874326	64.0458848
11.9332001	12.8258282	10.6502339	12.7436067	12.1865942	14.0706398	8.73591138
0.26284582	0.08016143	0.18933749	0.21651759	0.27385605	0.30205309	0.30247923
34.1699561	38.0766776	33.8914111	38.4473377	35.8751423	40.173061	26.3304593
2.52331984	2.04411638	1.70403743	2.31983132	3.01241653	2.24022708	1.34176918
9.64644146	7.93598123	9.41954024	10.5474997	9.53931902	9.91740979	20.4335061
0.10513833	0.16032285	0.09466875	0.21651759	0.09128535	0.20136873	0.65955983
0.07885374	0.12024214	0.14200312	0.06186217	0.22821337	0.22653982	0.06904841
6.04545378	5.97202628	5.63279039	7.73277106	7.16589993	5.23558689	5.47598266
0.34169956	0.20040357	0.47334373	0.21651759	0.22821337	0.276882	0.54039566
0.81482203	0.84169498	1.08869058	0.64955277	0.7302828	0.80547491	0.560765
14.6930811	14.7096218	15.2416681	12.8364	13.9210158	13.9951265	18.9797569
1.78735155	1.68338996	1.70403743	0.83513927	2.19084839	1.25855454	0.93637723
129.950972	127.977717	140.535754	126.848376	126.658422	115.107398	97.5258344
0.60454538	0.36072642	0.80468434	0.34024193	0.31949872	0.62927727	0.98146179
34.3539482	32.6257006	33.5127361	38.5401309	33.2735099	36.5987661	40.8999309
10.2247023	12.024214	12.1175995	9.86701587	10.8629566	10.2949762	5.28309513
1.13023701	1.12225997	1.46736556	1.05165686	1.14106687	0.88098818	1.19602274
12.380038	8.49711121	8.28351529	11.8775363	9.08289227	12.1576369	16.0257335
57.1426805	63.4878498	59.6413101	59.6660615	60.9786134	44.5276597	30.4516661
25.3383367	23.9281858	24.5192052	24.3118322	20.2653476	23.2580879	17.0120824
12.6165992	13.1464739	11.8809276	15.5583354	11.7758101	14.2216663	22.7133186
12.3274688	13.7476846	12.4489401	11.2898457	11.501954	12.2331501	10.9672808
3.07529605	4.12831347	4.97010917	4.05197203	5.15762224	4.75733617	3.08484164

0.55197621	0.36072642	0.42600936	0.43303518	0.50206942	0.80547491	0.83258246
5.12549342	5.17041201	4.02342171	5.16549107	3.788342	3.52395272	6.65952271
72.6505837	78.0371487	91.6393463	72.1931506	78.8705419	73.3989009	43.458381
0.21027665	0.6012107	0.42600936	0.55675952	0.22821337	0.80547491	0.16127168
8.12193573	7.45501267	7.95217467	6.89763178	6.93768656	7.37512961	5.47860181
67.7616515	73.387786	84.1131809	67.955592	73.5759917	66.7033907	40.1705018
0.15770749	0.12024214	0.04733437	0.12372434	0.13692802	0.25171091	0.12943391
239.05827	259.402376	268.669902	240.643835	244.233952	222.965523	234.042786
3.20671896	4.80968559	3.92875296	4.67059372	2.00827769	3.14638635	3.77548805
12.1697613	10.4610662	10.4135621	11.1970525	11.7301674	13.6679023	10.5358031
0.39426872	0.84169498	0.56801248	0.27837976	0.63899745	0.45307964	0.37214747
30.2009843	28.7779521	29.0159707	25.5490756	28.3897437	27.713371	6.17593103
59.7448541	64.8906748	61.3453475	61.9240306	58.1031249	52.3055268	43.5548273
1.20909076	1.64330924	0.85201872	0.77327711	0.91285349	1.28372563	3.92845396
34.0385332	28.938275	30.1519956	30.5599112	28.070245	27.5623445	24.5656852
58.1677792	55.912595	48.6597355	59.0783709	63.1694618	54.1681875	85.4724177
0.26284582	0.28056499	0.09466875	0.09279325	0.22821337	0.15102655	5.84609849
83.0855626	99.0795232	89.319962	84.4418599	83.7086655	84.4993519	83.5833431
0.05256916	0	0.14200312	0.03093108	0.22821337	0.10068436	0.0970673
12.8531604	13.3468775	12.0702651	14.0117812	7.71361203	11.9310971	5.54997596
0.36798414	0.32064571	0.37867498	0.18558651	0.27385605	0.32722418	0.51182443
1.52450574	2.1242778	1.60936868	2.10331373	2.32777641	1.81231854	1.53049275
0.26284582	0.28056499	0.33134061	0.52582843	0.09128535	0.52859291	0.58460985
112.708286	120.081817	139.163057	118.063948	123.280864	100.105428	72.988029
267.130203	274.873532	330.67793	259.759245	291.839262	247.331139	116.518611
68.6816118	69.4598761	65.0374286	74.7913617	62.8043204	82.5108358	35.6988334
1.07766785	0.9218564	0.80468434	0.77327711	0.86721082	0.70479054	1.23147614
9.06818067	11.7035683	10.3662277	11.0423971	11.5932394	11.7548994	2.671396
137.888915	149.020092	147.304569	137.024703	144.139567	119.235457	76.4457193
0.91996036	0.9218564	0.89935309	0.92793253	1.41492292	1.05718582	0.9764633
45.4723263	51.2632323	47.3817074	46.7987304	49.4766594	36.3218841	23.2305814
0.26284582	0.08016143	0.14200312	0.18558651	0.09128535	0.05034218	6.36703796
21.6059261	24.7698808	27.7379426	23.5385551	23.4146921	20.8416632	13.7442596
12.4063225	10.7817119	11.8335933	10.8877416	9.85881774	10.9997667	20.0217304
19.476875	17.5954331	15.47834	16.5481301	15.9749362	15.5809052	8.91349584
35.3001932	36.8742562	34.2700861	34.3025724	35.0992169	29.0726099	14.2916454
25.5748979	11.2225997	16.0463525	22.7034158	15.153368	24.0635628	109.19859
0.42055331	0.48096856	0.42600936	0.89700144	0.3651414	0.45307964	0.34308547
12.879445	12.2646983	11.6915901	10.5784308	11.8214528	11.2263065	14.4543444
1.39308283	1.12225997	1.37269682	0.95886361	1.50620827	0.80547491	1.79856223
32.1986125	28.5374678	27.6906082	29.7247719	36.1489984	30.6835597	27.0894694
148.560455	113.50858	133.67227	115.651324	164.724413	157.268976	99.2798785
24.4972301	19.5994688	19.2650898	27.7761136	22.0910546	20.2627281	13.4956545
11.4863622	10.901954	12.1649339	11.753812	12.6886636	13.4917047	11.7092481
0.31541498	0.48096856	0.47334373	0.30931084	0.5477121	0.07551327	0.14426715

198.790291	184.89233	211.442644	202.072773	180.790635	165.525093	93.6377496
12.3274688	13.1464739	13.8216369	13.1147797	15.7467228	14.3475218	10.6798945
98.5671812	107.536554	99.2601803	93.659323	102.330877	86.8402634	48.3657366
0.21027665	0.28056499	0.23667187	0.27837976	0.27385605	0.40273745	0.25878102
0.5782608	0.84169498	0.66268122	0.43303518	1.41492292	0.90615927	1.15963593
1.39308283	1.44290568	1.79870618	1.63934746	1.23235222	0.93133036	1.13156554
0.05256916	0	0.09466875	0	0	0.05034218	0.07651051
0.05256916	0	0.09466875	0	0	0.05034218	0.03496851
24.944068	23.206733	23.5725178	19.084479	24.5101163	37.3538988	34.5568642
108.345046	104.5305	114.40718	102.6912	106.758216	93.938511	52.6462359
11.433793	12.9460704	15.47834	12.5889513	11.0455273	12.1576369	18.2595178
46.050587	47.2150802	51.8784729	47.7885251	44.5016079	42.6398279	51.3518733
0.23656123	0.16032285	0.09466875	0.09279325	0.09128535	0.15102655	0.20472718
0	0	29.6313175	0	34.8253608	28.1664506	0
60.7699528	66.6943069	65.1320973	57.4699545	62.5761071	59.026208	23.1802271
15.5341878	15.791801	15.7623462	17.4760626	16.5682909	17.2170261	50.6739817
532.341632	579.647275	699.88604	838.294245	402.796604	862.08469	1571.06316
0.02628458	0	0	0	0.09128535	0.02517109	0.0218212
62.7412964	45.9725781	46.1983481	62.9447564	50.3438702	57.0125207	175.429723
35.0110628	43.6879775	42.2695951	44.1077261	29.7133812	35.8436333	13.3567808
2.02391279	1.96395495	1.89337492	2.84565975	2.37341909	1.78714745	2.0055267
17.4529622	16.633496	15.5730087	16.9811652	19.3981368	18.022501	15.3652045
0.02628458	0	0	0	0.09128535	0.02517109	0.07193186
0.5782608	1.24250211	0.75734997	0.77327711	1.32363757	1.13269909	1.09756077
68.8656039	71.4639117	64.0434068	72.2859438	79.418254	68.6919069	70.0559494
0	0.04008071	0.04733437	0.06186217	0	0.07551327	0
35.3790469	37.3552248	33.6074049	41.4476529	47.0575976	29.2739786	11.1946889
9.09446525	7.73557766	8.47285278	11.5991566	8.03311075	8.7847107	4.67245923
17.1112627	18.4772088	15.7150119	20.6001021	17.2072884	18.3245541	10.7233379
1.41936741	1.32266354	1.18335933	1.26817445	0.41078407	1.35923891	0.62806701
118.885163	112.546643	114.785855	125.363684	115.475967	132.727162	151.096591
1.5770749	1.60322853	1.37269682	2.22703806	1.55185094	1.6361209	1.85056355
78.4594762	79.3197315	70.6228846	82.4932016	68.8291535	58.0445355	68.1906372
33.3288495	36.5135298	39.5715359	37.0554389	37.3813506	43.7221848	14.5298257
19.9499975	23.0464101	24.2825334	20.940344	21.1782011	19.5075954	32.8680648
4.7312247	5.731542	4.68610293	4.29942071	4.29041142	4.73216508	2.41503756
13.2474292	18.7978545	16.6143649	15.8367151	14.9707973	12.9379407	6.48734388
102.378446	109.340186	103.47294	105.196617	97.4014679	89.4328857	52.117372
316.308656	308.821896	263.605124	335.076435	257.652899	391.385291	421.958256
12.0120538	11.9039718	12.2596026	11.3207768	11.501954	10.1691207	13.273865
11.6966388	14.5092182	13.6796338	11.8156742	13.0994476	11.2514776	15.8981401
2.81245024	2.08419709	1.84604055	2.1651759	1.82570699	1.78714745	2.07192349
38.4806275	39.3592604	40.2342171	40.3650649	35.4643583	33.5027219	11.2911454
51.3600725	53.2672679	47.3817074	48.1287671	45.460104	47.5481906	89.0546002
6.7288529	9.73961332	12.2122683	8.16580624	13.5102317	11.3773331	6.64887453

78.6434683	73.5080281	74.2676313	63.2231362	103.517586	82.7373756	29.2189512
0.34169956	0.08016143	0	0.15465542	0.50206942	0.15102655	0.43070174
236.166966	278.120069	297.780541	254.006064	262.856164	227.924228	103.178419
51.1760805	50.9826673	48.0443887	49.953701	45.186248	44.2759488	30.2868629
4.12667932	4.16839418	3.171403	4.20662745	4.79248085	4.20357217	4.22966573
92.9422807	84.9711121	81.6991279	75.4718455	88.0903622	78.2065792	40.2673715
1.62964406	1.64330924	1.56203431	1.39189879	1.00413884	1.58577872	1.08816571
0.5782608	0.40080713	0.66268122	0.52582843	0.5477121	0.98167254	0.37377961
0.02628458	0.08016143	0	0	0.09128535	0.12585545	0.01798297
0.13142291	0.12024214	0.09466875	0.09279325	0.09128535	0.05034218	0.11574349
3.18043438	3.24653777	2.4613874	4.33035179	3.01241653	3.04570199	1.66870193
0.68339912	0.48096856	0.5206781	0.80420819	0.3651414	0.95650145	0.27852477
0.18399207	0.16032285	0.04733437	0.18558651	0.27385605	0.17619764	0.13910679
0.10513833	0.36072642	0.09466875	0.15465542	0.09128535	0.10068436	0.159472
0.15770749	0.16032285	0.28400624	0.03093108	0.1825707	0.17619764	0.71188653
0.39426872	0.32064571	0.37867498	0.21651759	0.27385605	0.32722418	0.71771298
0.78853745	0.76153355	0.61534685	0.83513927	0.95849617	0.90615927	2.01589603
0.13142291	0.44088785	0.37867498	0.46396626	0.45642675	0.32722418	0.15253103
16.8747014	17.7156753	19.1230867	16.4553368	16.4770056	16.2856958	18.9468323
4.8626476	9.25864476	6.1534685	8.78442792	5.88790504	8.68402634	7.44015661
1.23537534	0.88177569	1.13602495	1.02072578	0.27385605	1.15787018	1.80659469
3.8638335	3.52710277	4.49676544	3.89731661	3.05805921	3.34775508	4.37240576
0.10513833	0.08016143	0.09466875	0.06186217	0.1825707	0.20136873	2.35989297
52.2800329	54.8304157	63.2860568	50.7888403	53.5388575	53.3375415	30.5412581
9.98814103	8.21654622	10.2242246	11.5372944	10.2239591	9.81672543	15.7859765
69.8381334	71.3436696	66.5521285	76.3379159	64.401814	83.9204168	37.6850288
16.2175869	16.5533346	15.3363369	14.7231961	14.3317999	15.0271412	121.33783
4.4420943	2.7254885	3.73941547	3.68079902	2.73856048	2.94501763	4.0933699
0.34169956	0.24048428	0.33134061	0.27837976	0.41078407	0.52859291	0.22754276
0.44683789	0.12024214	0.18933749	0.06186217	0.13692802	0.30205309	0.43381763
1.36679825	0.56112999	0.89935309	0.77327711	0.77592547	0.75513273	2.53330934
9.90928728	9.61937118	8.56752152	9.31025635	7.57668401	8.38197325	4.32538277
10.4086943	8.4570305	10.2242246	11.3517079	10.7716712	10.1439496	4.877534
0.15770749	0.64129141	0.47334373	0.21651759	1.04978152	0.553764	0.08431873
8.56877362	8.81775692	7.99950905	5.75318167	8.8546789	8.02957798	10.152111
0.44683789	0.48096856	0.28400624	0.49489735	0.7302828	0.42790854	0.6941082
0.5782608	0.76153355	1.37269682	0.9897947	1.36928024	0.98167254	0.85638973
44.736358	41.9244261	51.7838041	47.726663	49.4310167	51.8524471	58.218434
19.2403138	16.7537381	18.1763993	20.4763778	18.6222113	19.8851618	53.3100623
0.26284582	0.16032285	0.33134061	0.21651759	0.31949872	0.47825073	0.89814362
70.0221255	70.2214096	66.1734535	75.6883631	64.1736007	83.3163107	36.3492616
5.20434717	4.8497663	4.9227748	4.60873155	4.3360541	4.35459871	9.69748858
11.8280617	11.8238104	13.4902963	13.2694351	14.5143706	12.9379407	33.0722143
0.02628458	0	0	0.06186217	0.04564267	0	0.04130192
0.02628458	0	0	0.06186217	0.04564267	0	0.03090201

92.1537433	103.167756	97.0827992	89.7001443	96.9450411	84.8769183	43.9751578
58.4831942	63.2473655	56.8959164	50.7269781	60.202688	54.8226358	15.8400598
53.8308232	47.2150802	43.7369607	54.6552258	52.8085747	49.3856802	43.9882788
50.1509818	50.0207301	44.3996419	51.0053579	35.7382143	45.7610431	40.5077303
0.05256916	0	0	0.12372434	0	0.02517109	0.06081048
0.10513833	0.12024214	0.14200312	0.06186217	0.13692802	0.05034218	0.15449149
20.922527	22.7257644	24.2825334	17.9400289	20.9499877	23.0818903	22.2365067
57.8260796	52.5057344	49.5117542	57.0059882	46.7837416	43.1432497	21.4910502
191.719739	190.623872	209.975279	182.029431	188.412961	178.8406	250.697951
63.4772647	63.9287376	62.5760412	64.9552769	58.4226237	52.7837775	26.1011103
35.8521694	36.5536105	40.5655577	33.6530196	34.9622888	36.1205153	55.9762998
68.7078964	69.4999568	65.6054411	74.8532238	62.8956058	82.7373756	33.4658005
7.49110577	7.05420553	7.33682782	7.0522872	9.35674832	8.05474907	9.23766066
0.02628458	0.08016143	0.04733437	0.03093108	0.13692802	0.10068436	0.0751251
0.05256916	0.08016143	0.09466875	0.15465542	0.04564267	0.12585545	0.14834391
0.15770749	0.80161427	0.04733437	0.40210409	0.04564267	1.18304127	0.29956716
4.78379386	0.52104927	0.66268122	1.1753812	8.44389483	0.50342182	3.3174289
29.1758856	26.5735129	26.9332583	25.3634891	25.5142552	25.9513947	46.7152242
5.23063175	4.80968559	4.63876856	5.59852625	4.7011955	6.24243053	0.70811806
5.23063175	4.80968559	4.63876856	5.59852625	4.7011955	6.24243053	0.70788304
0.39426872	0.48096856	0.33134061	0.24744867	0.27385605	0.37756636	8.76914773
7.70138242	8.49711121	8.37818403	8.44418599	8.94596425	9.71604106	19.7298172
1.76106697	2.1242778	2.08271241	1.73214072	2.87548851	2.4667669	9.79221497
86.5025582	92.2257212	103.141599	97.309191	87.0405807	76.3942607	53.5783826
151.162629	145.372747	148.866603	151.500451	142.176932	115.862531	36.3826893
0.10513833	0	0.04733437	0.06186217	0.04564267	0.17619764	0.0997009
10.3298406	8.41694978	9.37220587	10.4856376	9.35674832	11.3269909	6.10893975
19.8711437	20.681648	17.3717149	22.9817956	20.4022756	19.8599907	15.9921563
113.07627	121.484642	114.217842	107.887622	98.7707481	116.441466	25.070147
0.39426872	0.24048428	0.28400624	0.49489735	0.41078407	0.35239527	0.76073048
5.57233131	5.53113843	4.49676544	5.25828432	3.83398468	4.60630962	7.41332337
0.52569163	0.48096856	0.80468434	0.49489735	0.77592547	0.98167254	0.34949502
34.5116557	31.3831985	32.9447237	36.0347131	31.6303736	39.1410462	23.6911337
151.004922	135.232327	108.632386	137.859842	165.226483	131.06587	46.7710082
0.44683789	0.40080713	0.47334373	0.43303518	0.13692802	0.37756636	0.75760251
24.3920918	21.242778	17.6557212	25.5181445	21.3607718	22.6791528	39.2095751
107.687931	115.111808	103.946283	109.526969	104.795581	87.3940274	40.9681419
0.31541498	0.24048428	0.37867498	0.12372434	0.41078407	0.17619764	0.82444979
7.70138242	5.85178414	5.49078728	7.0522872	6.11611841	8.43231543	5.63624751
14.6142274	15.3909939	13.7269682	12.2177783	13.9210158	13.8189289	6.7284039
0.68339912	0.24048428	0.33134061	0.30931084	0.22821337	0.40273745	1.299133
0.07885374	0	0.04733437	0.03093108	0	0.02517109	0.0437364
18.2152151	16.6735767	16.7090337	18.9298235	18.0744992	19.1552001	25.3930534
5.33577007	5.29065415	5.77479351	4.54686938	5.11197957	4.9335338	2.74020527
16.4541481	13.7877654	10.1768902	14.7541272	10.4065298	21.6974803	15.2601343

0.07885374	0	0.04733437	0.03093108	0	0.02517109	0.08627963
91.9697512	98.959281	95.9941086	94.8347042	95.2562622	76.2432341	35.3512152
5.51976215	4.92992773	5.68012477	4.63966263	6.25304644	7.47581398	7.62534585
19.1877446	17.7958367	22.2471553	16.1150949	16.2487922	21.1688874	33.5795214
0.07885374	0.04008071	0	0.03093108	0	0.02517109	0.0735779
234.721314	246.496387	220.388841	241.942941	314.523672	214.810089	108.371816
18.9774679	19.5994688	19.028418	16.0841638	20.2653476	16.5122356	11.6598981
18.7671913	17.9561595	14.0109744	16.8574409	14.7882266	14.5237194	18.9419379
108.029631	115.472535	118.051926	105.196617	99.7292443	111.986183	153.842906
105.480026	96.594519	112.703142	96.5049828	101.418023	94.1398797	33.7338988
2840.91644	3025.33232	3000.19457	2960.25942	2889.63774	2047.34102	413.400493
47.1545395	44.449511	46.4823543	48.9639063	49.9787288	48.8319162	53.3365268
0.13142291	0.04008071	0.04733437	0.12372434	0.1825707	0.10068436	2.70197829
0.05256916	0.04008071	0.09466875	0.03093108	0.09128535	0.17619764	0.03382803
10.5926864	8.85783763	9.135534	10.5165686	9.76753239	11.3773331	29.9950863
18.4254917	19.1585809	20.8744585	18.9916857	16.979075	17.6197636	12.1737147
97.8312129	105.532518	99.8281928	95.7007746	100.231314	85.4055112	70.9818993
423.181765	361.367711	329.92058	331.48843	360.987914	328.935815	459.948486
28.8867552	26.132625	32.2347081	27.4049406	26.0619673	27.159607	19.8968144
47.7590849	48.8583895	51.3577948	49.0566996	50.7546543	59.8820251	124.436603
0.02628458	0	0	0.03093108	0.04564267	0.02517109	0.05363393
0.02628458	0	0	0.03093108	0.04564267	0.02517109	0.02882433
0.02628458	0	0	0.03093108	0.04564267	0.02517109	0.06855766
0.5782608	0.20040357	0.42600936	0.27837976	0.31949872	0.32722418	1.22172352
63.582403	64.0088991	62.9547162	68.4195583	67.0034465	68.6667358	58.3054103
50.6241043	44.5296724	41.7015827	45.901729	49.0658753	52.5572377	25.9927477
38.0863588	38.1167583	38.2935078	43.705622	39.0701296	40.0472055	18.958487
173.136539	162.006243	176.32054	167.429959	145.098063	159.710571	335.650363
252.121707	269.021747	239.559262	265.852669	271.802128	284.836064	431.352128
0.42055331	0.36072642	0.33134061	0.24744867	0.27385605	0.32722418	0.42237822
54.2776611	61.5238949	54.0085197	58.1504383	60.9329708	63.5821754	41.2020252
0.05256916	0.08016143	0	0.03093108	0.04564267	0	0.07066712
20.8962424	18.6776124	18.9337492	18.0946843	23.9167616	22.2009021	13.42193
13.1160062	13.4270389	11.8809276	10.9805349	14.6056559	8.98607943	8.07073305
101.931608	109.700912	96.6567898	98.0206059	104.567368	82.0577561	52.490606
55.8021668	51.7842815	48.7070699	53.603569	46.3273149	44.7793706	48.6542901
0.02628458	0	0	0.03093108	0.04564267	0.02517109	0.04345073
0.07885374	0	0	0.06186217	0.1825707	0	0.04202162
0.02628458	0	0	0.03093108	0.04564267	0.02517109	0.51860551
0	0.04008071	0	0.03093108	0.09128535	0	0
0.05256916	0.08016143	0	0.03093108	0.04564267	0	0.06495665
0	0.04008071	0	0.03093108	0.09128535	0	0
0.02628458	0	0	0.03093108	0.04564267	0.02517109	0.02803273
17.5055314	17.755756	14.9103275	16.6718544	17.8006431	20.5396101	10.5749426
3.99525641	3.00605349	3.55007798	3.15497059	3.65141398	5.4872978	6.15921655

94.0725177	103.488402	79.9004217	95.5151881	87.9077915	71.9389776	28.2815251
0.02628458	0	0	0.03093108	0.04564267	0.02517109	0.12861417
0.55197621	0.52104927	0.37867498	0.43303518	0.3651414	0.35239527	0.33534858
5.09920884	4.8497663	5.49078728	6.80483853	4.38169677	6.21725944	4.58323812
20.5545428	20.8018902	18.3184024	24.3427633	19.4894221	21.2444007	6.90107578
58.5620479	55.7522721	48.470398	61.4909954	57.9661969	56.257388	24.2102369
1.49822115	0.96193712	1.32536245	1.20631228	2.09956304	1.40958109	2.56867817
5.49347756	5.97202628	4.82810605	4.1138342	5.47712097	4.88319162	15.1524018
0	0.16032285	0.33134061	0.34024193	0.09128535	0.276882	0
0.10513833	0	0.14200312	0.09279325	0.04564267	0.25171091	0.03823251
6.38715334	9.49912904	11.8809276	8.97001443	7.34847063	7.80303816	7.51279868
32.35632	29.9803735	37.0628141	28.271011	29.2569545	33.8551172	107.265609
0.26284582	0.16032285	0.23667187	0.18558651	0.09128535	0.17619764	0.24848177
6.22944585	7.97606194	6.48480911	7.17601154	5.24890759	5.83969307	7.74036504
0	0.16032285	0.14200312	0.18558651	0.09128535	0.15102655	0
0.02628458	0	0	0.03093108	0.04564267	0.02517109	0.02498333
0.9988141	0.68137213	1.18335933	0.83513927	0.82156815	0.67961945	0.45069516
69.1021652	70.4618939	66.1734535	75.7502253	63.8084593	82.6618623	37.3125849
96.0175768	91.143542	99.9228615	103.371683	94.0239099	112.112039	94.83802
175.134168	185.693945	176.32054	178.750736	176.317652	137.283129	88.5468271
0	0.04008071	0	0.03093108	0.09128535	0	0
56.3541431	61.8044598	57.7952695	66.0687959	67.0490892	56.6601255	74.5266955
277.170913	255.795112	292.621094	246.21143	258.01804	259.237065	478.559064
0	0.08016143	0	0	0	0.17619764	0
6.36086876	10.2205819	8.6148559	11.4754322	8.39825215	8.60851307	3.34313946
1.41936741	1.24250211	1.2306937	1.05165686	0.91285349	1.25855454	1.3475291
0.15770749	0.08016143	0.09466875	0.09279325	0.04564267	0.07551327	0.18730218
2.99644231	2.80564993	3.97608734	2.44355565	2.82984583	3.09604417	9.88006402
0.13142291	0.12024214	0.14200312	0.12372434	0.04564267	0.07551327	0.03804703
0.15770749	0.12024214	0.18933749	0.12372434	0.1825707	0.02517109	0.09074377
7.75395159	9.29872548	9.41954024	7.23787371	8.1243961	8.38197325	14.972841
39.8474258	42.1649103	42.2222608	37.5194052	40.5763378	37.7062941	13.6120551
6.78142207	7.45501267	8.23618091	6.21714793	6.20740376	7.55132725	4.01433039
56.6432735	63.6080919	67.9248253	55.7378138	58.0118396	60.7378422	30.1526903
2.73359649	2.56516565	2.27204991	2.28890023	1.68877897	2.4667669	1.60459133
187.619344	194.191056	241.452637	160.006499	180.334208	225.960882	74.1677106
0.2891304	0.28056499	0.09466875	0.27837976	0.31949872	0.35239527	0.24664502
4.12667932	5.05016987	5.11211229	6.30994118	4.01655538	5.83969307	4.74669116
12.6165992	13.1865547	15.857015	11.8156742	13.7840878	12.1576369	5.10289304
10.6715401	12.6254247	9.27753712	11.8156742	12.7799489	11.5031885	5.83173461
18.6883376	18.8780159	19.5017617	15.9295084	18.4396406	18.0980143	16.1107598
152.424289	163.288826	168.226362	146.304028	157.604156	143.97864	81.5831933
0.05256916	0.24048428	0.04733437	0.21651759	0.13692802	0.15102655	0.07939146
12.56403	11.3428419	12.306937	14.5066785	12.2778795	12.8875985	16.1273798
0.42055331	0.32064571	0.33134061	0.15465542	0.41078407	0.276882	0.47834186

8.17450489	7.05420553	6.34280599	9.09373876	6.57254516	8.48265761	9.58289551
180.759068	180.643775	167.185006	190.133375	195.989645	185.611624	48.3216578
963.119641	1094.7646	1063.22469	887.753048	1123.22058	805.32388	78.5420536
0.07885374	0.20040357	0.09466875	0.09279325	0.09128535	0.05034218	0.10530636
0.60454538	0.6012107	0.80468434	0.74234602	0.77592547	0.90615927	0.45052175
0.21027665	0.24048428	0.14200312	0.24744867	0.27385605	0.30205309	0.58594153
0.02628458	0.08016143	0.14200312	0.09279325	0.13692802	0.12585545	0.02649653
27.3096803	27.4152079	28.8739676	26.0439729	26.9748208	24.6928401	23.2642965
19.0037525	15.2707518	21.3004679	14.8778515	15.0620827	19.960675	12.4648851
31.2786522	29.499405	25.7972333	30.838291	30.2154507	32.4707072	23.3522823
27.1519728	25.8520601	24.1878646	24.2190389	23.4146921	24.0635628	30.3884799
0.15770749	0.04008071	0.09466875	0.06186217	0.04564267	0.07551327	0.25152705
70.5215326	72.0651224	67.3568129	78.2556431	64.4474567	83.970759	36.843029
0.05256916	0.08016143	0	0.03093108	0.09128535	0.12585545	0.08083857
0.02628458	0.04008071	0	0	0.04564267	0.10068436	0.02788685
0.13142291	0	0.23667187	0.06186217	0.13692802	0.02517109	0.39597958
103.456113	105.452357	102.194911	86.6070358	100.915954	101.313641	36.0816365
0.26284582	0.12024214	0.18933749	0.12372434	0.09128535	0.17619764	0.09762727
38.454343	43.888381	49.5117542	34.9521252	40.8958366	45.2072791	107.398702
0.86739119	0.52104927	0.56801248	0.74234602	0.41078407	0.40273745	2.92304924
47.9956461	57.1150164	56.2805696	53.5726379	51.074153	44.4018042	45.6727866
3.60098769	4.5291206	2.65072489	4.48500721	1.87134966	2.51710908	1.31336768
6.28201501	6.97404411	7.47883094	5.72225058	6.57254516	6.26760162	5.88414737
41.9764769	42.9264439	35.8794548	43.9530707	38.3398468	48.3033233	163.793321
544.169694	505.417794	514.051291	559.667038	459.621735	403.920495	312.89061
0.05256916	0	0.04733437	0.18558651	0	0.02517109	1.91961443
248.231589	307.459151	293.709785	250.7583	299.324661	226.262936	89.0623398
0.02628458	0.04008071	0	0.03093108	0	0.10068436	0.04774097
0.18399207	0.28056499	0.18933749	0.12372434	0.22821337	0.12585545	0.16715543
24.1818151	24.4893158	24.4245365	25.7965242	24.555759	28.2923061	12.4526451
43.1592831	45.1709638	45.7723388	42.3137232	37.5639213	44.7038573	35.9646563
0.73596829	1.32266354	1.2306937	0.64955277	0.77592547	1.13269909	1.31430535
19.1351754	19.2387424	16.9457056	15.434611	16.8877897	18.4000674	5.558722
0	0	0.04733437	0.03093108	0.09128535	0	0
22.368179	23.8881051	19.7857679	23.0127267	20.493561	16.7891176	19.0946713
12.7480221	13.6274425	13.6322994	13.7643325	5.93354771	14.4733772	12.0606866
0.76225287	0.56112999	0.99402183	0.71141494	1.18670954	0.98167254	1.9049085
0.34169956	0.28056499	0.23667187	0.40210409	0.45642675	0.32722418	1.25902422
0.5782608	0.32064571	0.42600936	0.37117301	0.27385605	0.37756636	0.2627704
0.44683789	0.36072642	0.42600936	0.5876906	0.63899745	0.40273745	0.65266891
7.83280533	8.61735335	7.66816844	8.53697925	8.4895375	9.56501452	3.47542815
0.21027665	0.76153355	0.61534685	0.37117301	0.50206942	0.276882	0.19665775
73.5968286	70.7023782	67.9721597	73.8634291	77.318691	76.2935763	60.3822379
41.5559236	44.8903989	42.2222608	40.2104095	42.2651168	37.5804386	32.3788213
0	0	0.04733437	0.03093108	0.09128535	0	0

12.56403	14.4691375	12.2596026	10.6712241	11.6388821	13.3155071	24.5125884
6.93912955	8.25662693	7.76283718	10.9805349	3.788342	4.05254562	8.59163462
66.3685687	73.107221	74.9303126	68.295834	70.6548605	60.2847625	59.1531459
0.21027665	0.08016143	0.23667187	0.18558651	0.09128535	0.07551327	8.43371585
9.43616481	7.49509338	7.19482471	8.47511708	5.84226237	7.17376089	9.92103263
34.8796398	33.0265077	33.5600705	36.003782	30.443664	27.159607	18.8253915
42.2130381	34.7499784	33.370733	39.3443391	38.3854894	41.7084975	40.6443038
4.38952514	5.61129986	5.06477792	3.46428143	4.7011955	4.98387599	3.16886483
0.13142291	0.32064571	0.14200312	0.27837976	0.1825707	0.32722418	0.08494991
8.41106613	10.5813083	7.28949345	9.55770503	9.17417762	2.86950436	18.6566334
0.23656123	0.24048428	0.47334373	0.24744867	0.59335477	0.42790854	0.21922869
111.630618	127.376507	149.718622	112.465422	131.770402	112.539947	115.237208
100.959078	92.5463669	101.20089	96.7524315	82.1568145	94.6936437	65.2068393
0	0.12024214	0.04733437	0.06186217	0	0	0
16.2438715	32.5054585	31.335355	20.4145156	27.2030341	22.5784685	87.9242865
0	0.08016143	0.04733437	0	0.13692802	0.10068436	0
18.2414997	18.4371281	18.271068	20.7238264	20.2653476	18.6266072	6.43348938
30.5689685	34.3491713	34.0807486	27.3121474	33.1365819	31.1366394	41.3885656
142.225871	142.005967	143.565154	149.644585	162.350994	149.113542	194.285666
170.350374	194.231136	187.160111	174.111073	177.869503	157.042436	103.597864
6.09802294	6.3327527	6.10613413	6.80483853	7.02897191	6.74585234	9.28390998
1.55079032	1.12225997	0.75734997	0.80420819	0.86721082	1.15787018	0.77875984
0.07885374	0.20040357	0.23667187	0.30931084	0.04564267	0.32722418	0.06934624
16.0073102	14.5492989	12.4962745	15.6201975	14.1492292	17.6449347	55.3932302
32.8031579	39.279099	39.5715359	36.3130929	33.5017233	33.4272086	14.520579
7.43853661	6.25259127	7.24215908	7.36159805	8.71775087	7.29961634	15.2675374
6.62371458	6.77364054	6.86348409	5.44387082	8.6721082	8.83505288	5.76089051
36.3515764	41.5636997	40.5182233	36.9317146	38.0659907	35.189185	25.1304595
12.56403	11.6234068	14.2476463	11.3207768	9.99574577	13.290336	26.7992902
15.9547411	15.2707518	12.7329464	16.4862679	17.1616457	14.7754303	28.1633475
5.88774629	5.01008916	5.53812165	5.78411275	5.15762224	7.55132725	19.5186811
0.15770749	0.08016143	0.14200312	0.15465542	0.13692802	0.25171091	0.11912396
2.44446609	2.32468137	2.36671865	2.56727999	3.10370188	2.61779345	3.4690016
0.02628458	0	0	0	0.04564267	0.05034218	0.03166277
0.21027665	0.24048428	0.28400624	0.12372434	0.5477121	0.37756636	0.41996463
0.94624494	0.84169498	1.2306937	0.71141494	0.86721082	0.88098818	2.59535314
0.18399207	0	0.09466875	0.18558651	0.13692802	0.15102655	0.27101119
0.02628458	0	0	0	0.04564267	0.05034218	0.01236199
30.3324072	27.2148043	30.4833363	26.2604905	29.9415946	25.5738283	25.3018664
2.8650194	2.1242778	2.4613874	2.10331373	2.60163246	1.78714745	2.15742447
15.3764803	15.4310746	14.5316525	15.6201975	15.8836508	17.7959612	18.1212157
12.8005913	10.901954	11.0289089	11.4135701	12.8712343	13.9951265	8.84926522
39.1640267	42.7260403	38.0568359	41.9425502	44.0908238	42.5391435	18.1129592
84.50493	83.7686907	96.7514585	91.2776296	98.9533188	93.4350892	39.2607811
45.7088875	47.7361295	41.8435858	46.7987304	47.4683817	50.6190637	70.2449862

1.18280617	0.64129141	0.75734997	0.64955277	1.09542419	0.37756636	3.34159209
0.10513833	0.08016143	0.23667187	0.09279325	0.09128535	0.07551327	0.10036221
0.07885374	0	0.04733437	0	0.04564267	0.02517109	0.09646063
0.55197621	0.24048428	0.5206781	0.49489735	0.5477121	0.65444836	0.13813919
2.49703526	2.00403566	1.60936868	2.50541782	2.19084839	2.51710908	1.01095034
0.13142291	0.32064571	0.28400624	0.40210409	0.27385605	0.276882	0.23082227
73.7282515	74.9509338	72.705597	70.863114	71.7502847	64.0604262	21.949546
46.0768716	72.1052032	53.4878416	62.3261347	57.0077007	30.2556512	55.3685251
5.83517713	6.3327527	8.23618091	5.90783709	6.80075853	10.521516	1.65828464
0.10513833	0	0	0.06186217	0.04564267	0.12585545	0.20845084
0	0	162.830243	0	154.135313	157.319318	0
4.04782557	5.17041201	5.01744354	3.86638553	4.24476875	4.4049409	2.21253147
31.4363597	28.4973871	31.9033674	37.5194052	31.3108749	30.5073621	32.3020881
13.6154133	12.0642947	11.6442558	14.2901609	12.6430209	14.0454687	27.4843805
15.2713419	17.3950296	16.6143649	17.3832693	14.5143706	12.9379407	11.8086016
10.5138327	7.93598123	8.80419339	8.84629009	7.25718528	8.43231543	15.0074874
0.26284582	0.52104927	0.37867498	0.46396626	0.7302828	0.35239527	0.52324722
533.70843	649.347636	584.39017	574.761407	558.757624	481.044717	209.356314
0.23656123	0.32064571	0.18933749	0.34024193	0.45642675	0.25171091	0.24420411
59.061455	51.4636358	46.7663606	48.8711131	61.6632536	60.7126711	26.5719044
0.02628458	0	0	0	0.04564267	0.05034218	0.11566022
162.464999	148.539123	180.580633	130.900348	124.558859	188.732839	342.361828
66.5525607	57.7963885	44.5416451	65.5120364	59.4267625	59.8065118	32.8277288
17.5843851	12.9861511	12.1175995	14.1973677	10.2696018	25.4731439	21.5322516
3.39071103	5.69146128	3.97608734	2.96938409	4.01655538	4.20357217	5.26705635
216.269538	233.590397	218.21146	230.622164	238.665546	250.452354	409.217851
1.91877446	1.96395495	1.84604055	3.5261436	2.05392036	1.40958109	1.48369693
34.3802328	31.3030371	27.6906082	36.1275064	18.9873527	28.1664506	10.6096954
5.17806258	5.77162271	4.73343731	5.72225058	5.52276364	6.29277271	3.20154042
5.96660003	5.69146128	6.57947786	6.00063034	7.53104133	6.84653671	3.22031942
5.7300388	6.6533984	4.11809046	5.01083564	4.24476875	5.2104158	6.40719569
47.8379386	39.1989376	42.2222608	48.1906292	46.0991015	44.8045417	49.3626705
0	0.04008071	0	0.06186217	0.04564267	0	0
0.07885374	0.04008071	0	0	0.04564267	0.02517109	2.79596015
0.2891304	0.64129141	0.33134061	0.43303518	0.31949872	0.17619764	0.22286639
602.416327	810.752668	500.797667	624.869764	665.51584	391.158752	206.277683
0.49940705	0.40080713	0.85201872	0.55675952	0.77592547	0.830646	0.3183519
2.60217358	2.24451994	2.41405303	2.81472866	2.23649106	3.32258399	0.99010906
0	0.04008071	0	0.06186217	0.04564267	0	0
71.3889238	72.1853646	67.2148097	77.2039862	64.9951688	85.2796557	38.4963717
68.1559202	66.2133383	56.7539133	65.728554	70.1984337	69.7994349	28.5674605
5.83517713	6.29267198	6.72148097	5.90783709	4.97505155	5.86486416	24.2792049
16.1124485	16.914061	15.2890025	17.1358207	19.3524941	16.3108669	10.0127615
4.1529639	2.88581135	2.84006238	3.09310842	3.788342	4.6818229	6.84210045
2.1553357	2.16435852	1.32536245	1.67027855	1.36928024	1.73680527	0.99101312

20.5019737	23.0063294	21.4898054	20.940344	26.3358233	23.3587723	12.3820106
5.54604673	4.32871703	4.87544043	4.67059372	3.83398468	4.53079635	8.12016433
7.0179833	6.53315626	8.14151217	7.36159805	7.53104133	9.08676379	6.23230173
6.20316127	6.09226842	6.24813724	7.0522872	7.66796935	6.0410618	4.21217643
45.6300337	40.8021661	46.1510137	48.3762157	44.9123919	48.6808897	80.7209665
48.4161994	45.4916095	45.67767	47.6648008	42.6302582	39.7451524	16.7638901
2.99644231	2.7254885	3.12406862	3.49521252	3.24062991	3.32258399	3.92872857
26.7314195	26.2528672	24.4245365	28.3638042	20.630489	21.9491912	16.3174411
0.02628458	0	0	0	0.04564267	0.05034218	1.00479818
0	0.04008071	0	0.06186217	0.04564267	0	0
8.0167974	6.21251056	6.6741466	7.0522872	7.80489738	8.5078287	11.0874282
5.54604673	4.04815204	3.78674985	5.22735323	4.60991015	5.4872978	4.16860049
104.165797	93.3880619	92.7753712	101.082783	95.4388329	69.3463552	36.4695151
0.42055331	0.6012107	0.42600936	0.55675952	0.3651414	0.17619764	0.57836612
97.9889204	87.3358742	91.2606713	92.4530108	90.4181386	74.5819421	110.528726
21.3693649	20.962213	20.7797898	19.0226168	19.5807075	19.7844774	19.0392057
13.9308283	14.9100253	13.2062901	12.8982621	12.5973782	13.013454	8.01381476
9.98814103	8.69751478	9.08819963	9.34118744	11.0455273	10.3956605	3.68800056
66.6314145	62.9668005	60.8246694	72.6261858	66.7752331	65.0169276	41.0212522
153.738518	154.751634	147.63591	152.119072	143.317999	126.811956	64.7053381
0.76225287	0.88177569	0.37867498	0.46396626	0.27385605	0.78030382	1.42576867
29.149601	30.3410999	29.3946457	28.3638042	25.5598978	28.6195303	30.6211058
7.727667	9.17848334	7.3841622	8.59884142	7.12025726	6.01589071	5.64029908
38.8486117	39.5195833	37.5834922	35.7872645	36.1033557	36.296713	20.0307417
14.4039507	11.9440526	13.8689713	15.0943691	13.464589	15.4550498	323.305337
0.7096837	0.20040357	0.09466875	0.74234602	0.22821337	0.52859291	0.46939477
29.5701544	31.0224721	29.5366488	27.899838	29.9872373	26.052079	29.4806311
22.9727244	25.3710915	23.7618553	27.4049406	22.6387667	27.713371	24.6943721
13.6679825	12.1043754	11.928262	14.7231961	12.1409515	14.5237194	5.80247846
13.5628441	12.7055861	12.5909432	14.3520231	12.1409515	16.1094981	9.92298296
0.21027665	0.52104927	0.18933749	0.86607036	0.1825707	0.25171091	0.06671724
114.206507	95.2317747	90.8346619	111.104455	109.953203	103.579039	54.7763727
0.26284582	0.20040357	0.56801248	0.06186217	0.27385605	0.35239527	0.44106367
15.3764803	12.7456668	12.4962745	15.063438	15.8380081	13.869271	14.5756078
28.3610636	23.3269751	32.3293768	24.8376606	22.2279826	29.4501763	104.029
0.02628458	0	0	0.06186217	0	0.02517109	0.10771706
0	0.04008071	0.09466875	0.03093108	0.04564267	0.12585545	0
146.773104	164.691651	160.936868	142.128332	163.492061	130.13454	18.5624582
0.5782608	0.40080713	0.7100156	0.46396626	0.50206942	0.37756636	0.27191156
151.057491	168.659641	176.604546	163.501711	124.467574	166.255055	124.645129
0.02628458	0.08016143	0.09466875	0.03093108	0	0.05034218	0.02927041
38.5594813	38.0766776	38.3408422	39.2206148	33.9125073	33.804775	10.9150169
0.02628458	0	0	0.06186217	0	0.02517109	0.01284344
262.504117	229.542245	209.54927	248.995228	242.81903	268.60071	179.796988
86.2397124	73.1473017	78.291053	87.411244	80.8331769	86.2361572	78.786983

6.33458418	6.81372125	7.52616532	6.03156142	7.16589993	5.33627126	5.71039318
125.482593	113.789145	130.595535	115.032702	110.957342	117.851047	57.8594075
0.02628458	0	0	0.06186217	0	0.02517109	0.02402207
21.1590882	24.6897194	24.4718709	22.7034158	20.630489	21.4961116	12.4650138
0.02628458	0	0	0.06186217	0	0.02517109	0.03078367
0.47312247	0.40080713	0.23667187	0.37117301	0.27385605	0.22653982	0.43304433
6.78142207	5.69146128	4.87544043	6.52645877	6.52690249	6.51931253	7.75291005
1.52450574	1.92387424	1.70403743	1.7630718	1.41492292	1.15787018	1.29462368
15.9547411	18.3970474	20.4011148	17.9709599	20.4479183	21.1940585	9.51130595
57.5106646	57.595985	57.3219258	51.8404972	59.2898345	49.4863646	20.451148
46.8128399	37.3953055	45.4409981	46.7987304	38.8875589	55.5525975	84.3379348
1.23537534	1.56314782	1.27802807	0.86607036	1.36928024	1.18304127	1.56036805
53.5416928	52.8664608	62.0553631	48.5618022	52.8542173	54.872978	69.8632154
13.3788521	14.5893796	15.1469994	13.6715392	13.2363757	12.5352032	10.3028975
40.9513782	35.6718348	41.2755733	36.3130929	39.709127	33.5530641	32.731276
87.1333882	86.8148249	83.6871716	93.0407014	94.3890513	87.4191985	27.2675852
90.9446525	91.2637841	91.7813494	90.2878349	89.2770718	76.6711427	19.9303573
2.49703526	2.44492351	1.89337492	2.84565975	2.82984583	2.34091145	1.45007665
0.02628458	0.04008071	0.14200312	0	0	0.07551327	0.01698101
0.2891304	0.24048428	0.33134061	0.21651759	0	0.32722418	1.3321618
12.4063225	13.9881689	15.1469994	12.124985	13.4189464	12.3338345	14.4951974
1.65592864	1.84371281	1.7513718	1.54655421	1.50620827	1.43475218	3.97190809
0.18399207	0.16032285	0.09466875	0.06186217	0.31949872	0.30205309	0.26541839
1.18280617	1.28258282	1.79870618	1.94865831	0.86721082	2.26539818	0.46419935
0.73596829	0.80161427	1.04135621	1.14445012	0.77592547	1.107528	0.93927926
8.72648111	7.77565837	6.53214348	7.33066696	7.30282796	6.24243053	4.65444772
0.31541498	0.20040357	0.42600936	0.06186217	0.22821337	0.35239527	0.74775678
325.087706	330.98653	368.261422	317.321993	340.722567	286.950436	63.7504013
400.524455	371.107324	377.444291	431.767005	375.59357	438.304205	1052.53634
0.76225287	0.44088785	0.5206781	0.49489735	0.22821337	1.61094981	0.95294094
4.99407051	5.17041201	5.30144978	4.14476529	5.38583562	4.27908544	4.43819319
0.42055331	0.32064571	0.23667187	0.37117301	0.22821337	0.15102655	0.24068148
0.21027665	0.20040357	0.14200312	0.03093108	0	0.276882	0.15338601
0.05256916	0.12024214	0.14200312	0.06186217	0.13692802	0	0.07378897
2.18162028	3.60726419	4.87544043	3.00031517	3.28627258	2.64296454	1.85587202
45.7614566	47.6960488	46.6716918	48.0359738	47.9704511	32.5713915	13.8665633
50.1772664	55.4717072	51.7838041	51.8714282	49.8418008	44.7541995	46.6421817
0	0.04008071	0	0.03093108	0.04564267	0.02517109	0
1.49822115	1.60322853	0.99402183	1.48469204	0.68464012	1.15787018	1.57588295
2.83873482	3.48702205	3.50274361	3.15497059	3.4232006	4.60630962	3.76840206
0.15770749	0.04008071	0.09466875	0.03093108	0.04564267	0.10068436	0.19496842
26.5999966	26.7739165	28.163952	26.0130418	26.4727513	23.4342856	28.9367578
2.39189693	2.28460066	2.08271241	2.01052047	3.28627258	2.69330672	2.50083102
31.6992055	29.2589207	34.0334142	33.5602264	30.5805921	38.3355713	34.3769249
246.969929	253.670834	295.413822	252.18113	268.880997	211.009254	156.455038

14.7193657	15.791801	10.8869058	15.3418178	13.9666585	16.4367223	10.3245317
287.369331	309.342945	334.701352	349.923356	307.631628	351.237402	947.532807
0.13142291	0.12024214	0	0.09279325	0	0.05034218	0.11599402
7.99051282	8.09630408	10.5555652	9.34118744	8.8546789	11.2514776	6.67213425
60.1654074	71.5841539	56.2805696	55.088261	58.8334077	60.4861313	31.8061612
5.4409084	6.13234913	6.62681223	4.48500721	5.56840632	6.06623289	2.51636413
39.9262795	51.7442008	48.1863918	37.1173011	46.3729575	45.4841611	47.9540793
5.54604673	5.1303313	5.39611853	5.62945733	5.52276364	6.74585234	4.59647513
44.736358	43.1268475	48.6124011	53.4489135	36.0120704	34.8116186	22.7123171
25.2331984	25.8921408	23.8091897	28.4565975	25.9706819	26.1024212	37.5973204
2.02391279	3.16637635	2.27204991	3.06217734	2.05392036	3.54912381	1.615545
4.54723263	4.12831347	4.11809046	4.02104095	3.56012863	3.77566363	5.28007851
12.1960459	12.8258282	12.8749495	11.2898457	11.501954	11.9310971	28.8573372
372.189676	338.922511	364.900682	333.406157	340.539996	322.064107	340.025504
7.78023617	7.17444767	7.3841622	6.74297636	7.21154261	6.36828598	9.62330469
3.54841852	4.32871703	5.72745914	3.46428143	5.70533434	2.24022708	1.17316976
0.10513833	0.04008071	0.09466875	0.18558651	0.1825707	0.05034218	0.13755526
102.615007	97.2758911	101.863571	103.742856	88.9119304	87.7967648	93.5027387
0.07885374	0.04008071	0	0	0	0.05034218	0.04679147
0.55197621	0.68137213	0.56801248	0.37117301	0.63899745	0.45307964	0.74858578
0.02628458	0	0	0.03093108	0	0.05034218	0.02478115
0	0	0.04733437	0.06186217	0.04564267	0	0
757.153659	691.592707	675.74551	747.140339	802.215651	766.308689	659.932256
7.28082912	7.81573909	6.72148097	6.58832094	7.25718528	5.91520635	7.50023667
0	0.04008071	0	0.03093108	0.04564267	0.02517109	0
1.47193657	0.64129141	1.08869058	0.83513927	1.27799489	1.93817399	1.08666164
15.2450574	18.597451	13.3009588	13.3622284	14.6056559	14.951628	29.3224122
20.2654125	21.7237466	22.0104835	19.7340317	19.1242807	20.237557	61.8213981
143.671523	103.608644	102.526252	111.073523	131.90733	140.857424	112.552839
21.1853728	19.0383388	18.1763993	20.197998	20.9956304	22.2009021	13.7084129
0.05256916	0.04008071	0.09466875	0.09279325	0.09128535	0.10068436	0.10918011
0	0.04008071	0	0.03093108	0.04564267	0.02517109	0
27.2308266	27.3350464	31.1460175	30.0031517	29.8503093	32.143483	75.4497603
73.1762753	74.9108531	79.9950905	71.7910465	78.8248993	60.9895531	33.7475815
15.6918952	11.5432454	12.6382776	11.9703296	12.1409515	12.3086634	69.2984274
0.02628458	0	0	0.03093108	0	0.05034218	0.9456924
0.05256916	0.08016143	0.04733437	0.09279325	0.09128535	0.10068436	1.8913848
6.80770665	8.29670764	6.20080287	6.83576961	6.70947319	5.56281107	3.77762182
0	0.08016143	0.09466875	0.09279325	0.13692802	0.05034218	0
17.9260847	18.1164824	18.4604055	19.9814804	17.5267871	21.4961116	9.70726668
184.149779	172.868116	185.503408	195.329797	175.359156	214.432523	845.282225
99.9339794	96.9953261	113.64983	94.8347042	96.1691157	98.0413988	40.1075346
0.9988141	1.36274425	1.27802807	1.20631228	1.09542419	0.70479054	0.60772673
1.2879445	1.32266354	1.98804367	0.92793253	1.59749362	1.28372563	2.325496
21.8687719	22.7257644	23.1465084	20.569171	22.3192679	19.8348196	14.2145306

70.6266709	71.423831	67.9248253	76.9256065	64.7669554	84.3734965	38.1105278
49.4675827	60.9627649	63.948738	55.7687449	64.7669554	68.3395116	21.3148874
13.7731208	12.9460704	10.2242246	11.4445012	12.0953088	10.1439496	19.2224254
0.76225287	0.52104927	0.37867498	0.71141494	0.50206942	0.90615927	1.15259915
54.4353686	57.0749357	64.8007567	55.088261	60.0657599	48.1774679	14.0056756
5.70375422	7.45501267	6.95815284	8.25859949	6.38997446	7.62684052	14.0671745
77.8023617	87.3759549	72.9896033	79.6475419	80.2398222	60.2847625	31.1587767
22.0264794	23.1265716	23.0045053	20.7238264	23.5516202	18.6266072	11.1572124
33.2237112	35.9924805	28.9213019	33.2509155	30.9000908	26.4296454	25.2592148
4.65237095	5.61129986	5.20678104	6.74297636	4.42733945	6.16691725	4.93811443
5.4409084	4.40887846	3.59741235	3.27869493	7.34847063	5.41178453	18.3861412
83.0067088	90.4220891	102.762924	76.5853645	91.2853495	88.8791217	185.293585
13.3788521	13.3869582	10.6502339	14.9087826	13.7840878	12.6107165	6.53469863
43.8163976	48.5778245	54.8605384	46.9224548	62.7586778	50.5938926	13.5764828
34.1436716	29.0585171	24.235199	32.9725358	31.5847309	30.9100995	38.0916103
0.73596829	0.80161427	1.13602495	1.05165686	0.77592547	1.30889672	1.59911042
18.3203534	14.8298639	15.0523306	17.0430274	16.7965043	18.8783181	21.0432099
4.41580972	5.21049272	5.53812165	5.01083564	6.16176109	5.81452198	4.91072273
16.2175869	15.9120432	14.6263213	14.321092	14.4230852	14.2971796	17.39477
138.651168	139.520963	159.138162	136.344219	138.525518	131.166554	102.981137
17.8998001	16.633496	17.6557212	18.4658573	17.7550005	20.3130703	29.6902534
34.6430786	34.1487677	28.7792988	32.1373965	32.4975844	41.3561022	134.748388
0.42055331	0.80161427	0.42600936	0.46396626	0.3651414	0.42790854	0.4662045
371.243431	435.116223	431.310807	373.245393	394.672208	373.992068	135.867352
1.62964406	1.40282496	2.17738116	1.51562313	2.37341909	2.26539818	0.71337255
0.23656123	0.20040357	0.18933749	0.09279325	0.22821337	0.15102655	0.23690698
43.8689668	33.5074763	32.4240455	34.1169859	28.1158876	38.0838604	37.6327216
0.13142291	0.20040357	0.09466875	0.09279325	0.09128535	0.10068436	0.18258684
4.07411016	4.16839418	5.01744354	4.82524914	4.65555282	5.33627126	8.6901464
12.3537534	13.3869582	14.2003119	12.5270891	12.3235222	11.7045572	8.56213291
0.15770749	0.08016143	0	0.18558651	0.04564267	0.17619764	0.33965009
18.1363613	22.8059258	18.5077399	16.7955787	17.252931	20.237557	39.0597601
46.996832	41.1228118	41.0862358	43.6128288	50.8459396	44.603173	10.5429181
18.1363613	19.8800338	13.0169526	18.1256154	22.4105533	16.4870645	4.03380796
30.5426839	25.7718986	24.8505459	27.7451826	27.9789596	32.143483	23.1417872
50.1509818	51.583878	53.6298447	52.6137743	45.186248	46.7175446	31.9110312
0.02628458	0	0	0.03093108	0	0.05034218	0.04307239
0.07885374	0.20040357	0.09466875	0.06186217	0.13692802	0.05034218	0.05585444
3.62727227	4.08823275	3.83408422	3.64986794	4.47298212	4.9335338	2.7915626
0.05256916	0.24048428	0.04733437	0.06186217	0.1825707	0.276882	0.02383951
0	0.04008071	0	0.03093108	0.04564267	0.02517109	0
31.1997984	29.0585171	30.7200081	29.7866341	28.481029	26.5303297	18.071143
157.470929	181.365228	163.918934	175.224592	198.499992	170.861365	31.1148228
20.8436732	17.8359174	17.7503899	19.6103074	19.8545635	21.9240201	29.1070303
69.4438647	76.3938395	75.7823313	73.3376007	67.4598733	65.9230869	62.4170883

0.47312247	0.32064571	0.14200312	0.21651759	0.1825707	0.30205309	0.54729433
3.25928812	3.04613421	2.60339052	2.19610698	2.41906176	3.09604417	2.77455753
31.5152134	34.1888484	31.6666956	31.4878437	33.7299366	24.4159581	20.7884047
30.4901147	27.816015	32.3293768	28.7968394	27.1117488	28.3174772	47.0045474
82.7701476	90.4220891	90.1719807	77.6679525	84.9410177	83.970759	66.7445635
3.70612601	3.24653777	4.30742795	4.36128288	4.60991015	3.82600581	2.33512716
147.272511	145.693393	144.369838	148.252687	140.625081	115.283596	60.475426
13.19486	25.4512529	23.0991741	21.4352414	23.6885482	24.1894183	6.25138572
15.4290494	15.2707518	12.685612	16.8574409	15.5185094	15.1529967	10.3391558
31.72549	31.7038442	30.4360019	30.6527045	30.443664	26.0772501	13.4661085
1.13023701	1.5230671	1.65670306	1.05165686	1.87134966	2.24022708	0.95253344
29.7278618	27.53545	27.406602	26.0439729	26.8835354	26.0772501	28.9650782
9.98814103	11.2626804	10.7922371	10.300051	9.08289227	9.71604106	16.2802743
111.998602	106.734939	100.490874	106.866896	98.0404653	92.3023901	30.5578769
0.97252952	0.72145284	0.7100156	0.49489735	0.59335477	0.85581709	1.60550748
0.31541498	0.32064571	0.33134061	0.18558651	0.13692802	0.35239527	0.3482333
1.07766785	1.04209854	1.13602495	1.1753812	1.14106687	1.51026545	1.26032047
4.57351721	5.25057344	4.2127592	6.92856287	4.3360541	4.73216508	4.93361222
0.07885374	0.04008071	0.09466875	0.09279325	0.04564267	0.17619764	0.24957471
0.05256916	0.08016143	0.04733437	0.06186217	0	0.02517109	1.60767708
23.2092856	19.2788231	23.3358459	21.2496549	23.7798335	26.4799876	8.95068641
14.824504	14.2286532	13.7269682	13.0219865	12.8712343	13.4413625	17.1242658
20.3442662	18.1565631	15.7623462	17.5379248	19.9914915	21.9240201	11.2533761
33.5654108	29.4593242	35.0274361	29.6010476	32.0868003	29.4753474	25.0748536
17.6369543	18.6375317	19.7384336	20.4763778	19.9458489	20.0110172	32.3463665
8.91047318	9.94001689	14.0109744	11.3517079	12.1865942	12.8372563	6.12878866
5.46719298	6.37283341	6.48480911	5.19642215	6.34433179	4.85802053	4.2142008
2.49703526	1.68338996	2.27204991	2.07238264	2.46470444	3.0205309	3.10267797
0.34169956	0.12024214	0.33134061	0.18558651	0.31949872	0.10068436	0.26863499
0.60454538	0.64129141	0.66268122	0.5876906	0.45642675	0.47825073	0.56996644
0.07885374	0.04008071	0.04733437	0.03093108	0.04564267	0.02517109	0.16674265
26.573712	31.2228756	31.2880206	25.8893175	30.1241653	26.5555008	22.0612356
21.6322107	22.1245537	23.3831803	20.7547575	24.3275456	16.3108669	5.12092207
2.20790486	1.72347067	2.22471553	1.23724337	1.68877897	2.24022708	4.10159074
281.139885	379.684597	340.570814	339.592374	302.337077	274.641772	175.960236
100.301964	103.688805	101.53223	100.371368	110.683486	66.502022	39.6439144
0.07885374	0.08016143	0.09466875	0.06186217	0.22821337	0.12585545	0.1051342
16.6118556	15.9521239	13.2062901	15.8367151	15.9749362	18.2742119	14.2653832
32.6191658	36.5536105	40.5182233	26.7863189	37.472636	36.1960286	51.453959
41.4770698	36.1928841	29.0633051	35.6016779	31.1739468	31.5142057	9.65891658
0	0.12024214	0.09466875	0.06186217	0	0.02517109	0
71.5992004	71.7444767	67.0254723	76.6162956	65.0408115	84.7258917	34.4895639
0.21027665	0.52104927	0.42600936	0.34024193	0.22821337	0.25171091	0.40862325
0.2891304	0.20040357	0.37867498	0.18558651	0.1825707	0.25171091	0.23809422
0.07885374	0.04008071	0.04733437	0.03093108	0.04564267	0.02517109	0.0843925

0.44683789	0.36072642	0.28400624	0.34024193	0.22821337	0.276882	0.82012034
0.07885374	0.04008071	0.04733437	0.03093108	0.04564267	0.02517109	2.60704392
3.4432802	3.52710277	3.26607174	3.77359228	4.79248085	3.24707072	2.99581363
0.47312247	0.24048428	0.5206781	0.37117301	0.3651414	0.22653982	0.12868566
0.97252952	0.72145284	1.04135621	0.37117301	0.82156815	0.98167254	1.8288717
5.88774629	7.09428625	5.53812165	6.55738986	8.07875343	6.19208835	3.17216179
15.6918952	15.6314782	12.0702651	13.578746	16.4313629	17.9469878	14.1560947
1.15652159	0.80161427	0.94668746	0.86607036	0.68464012	0.85581709	0.90981083
3.28557271	3.72750633	2.50872177	3.12403951	4.97505155	3.14638635	1.10296178
22.6573094	21.2026973	17.892393	23.7860038	19.9914915	23.4846277	12.5045581
13.1422908	13.1464739	11.4549183	8.93908334	12.8712343	12.4093478	7.63923537
0	0.04008071	0	0.09279325	0	0	0
0	0.04008071	0	0.09279325	0	0	0
69.4175801	89.8208784	70.9542252	62.8519631	76.8166216	72.8199658	379.095998
0.34169956	0.24048428	0.14200312	0.09279325	0.22821337	0.22653982	0.41222489
0.5782608	0.40080713	0.75734997	0.06186217	0.68464012	0.52859291	2.3737514
0	0	0.04733437	0.03093108	0.04564267	0.02517109	0
1.31422908	2.96597278	2.50872177	2.31983132	2.96677386	2.71847781	1.21886056
30.6215376	37.595709	35.3587767	33.3746399	43.5431117	35.4912381	16.6706168
4.23181765	3.80766776	4.68610293	4.73245589	4.74683817	4.75733617	3.78138803
136.706109	137.67725	138.784382	128.642379	144.413423	109.14185	60.1008339
0	0	0.04733437	0.03093108	0.04564267	0.02517109	0
0.02628458	0.12024214	0.04733437	0	0	0.07551327	0.01465856
30.1484151	30.2208578	31.1460175	30.498049	27.2943195	27.4616601	17.355347
33.3025649	31.5435213	32.7553862	31.3641194	30.03288	29.7018872	32.0649644
0.23656123	0.32064571	0.23667187	0.64955277	0.27385605	0.17619764	0.17527673
2.52331984	2.00403566	1.37269682	2.01052047	2.37341909	2.66813563	5.7427721
60.7436682	57.31542	53.2985041	60.0990967	52.2152199	46.566518	19.1487785
27.835372	20.962213	27.8326114	23.1055199	21.5889851	25.6493416	38.5624016
0	0	0.04733437	0.03093108	0.04564267	0.02517109	0
29.7015773	34.3090906	38.5301797	31.7352924	31.3108749	31.7659166	23.794042
43.0804293	49.0988737	53.3931728	40.2104095	47.011955	47.8502437	98.0458694
1.44565199	1.84371281	1.42003119	1.54655421	1.73442164	2.21505599	1.54719579
0.07885374	0.04008071	0.04733437	0.03093108	0	0.05034218	0.20033359
278.30115	301.647448	318.797003	289.669604	287.959635	254.429386	70.6897216
240.924475	242.287912	263.794461	217.136211	265.320868	207.334275	176.056967
36.8772681	36.0726419	36.4001329	38.8494418	41.671762	36.7497926	12.7722024
3.65355685	3.36677991	7.14749033	4.05197203	3.69705665	4.20357217	3.07382551
53.9622461	48.8583895	53.8665165	58.6762668	51.3480091	58.0445355	83.137558
8.17450489	8.17646551	7.57349969	7.23787371	7.48539866	7.12341871	11.520451
43.0804293	45.6920131	49.7957605	42.6230341	45.7796028	41.003707	43.0728687
7.0179833	6.09226842	7.62083406	8.47511708	6.52690249	7.90372252	21.4089667
102.457299	97.1957297	98.2188241	90.5971457	119.948949	111.407248	33.6333035
0.07885374	0.04008071	0.09466875	0	0.27385605	0.12585545	0.29725925
1.26165992	1.56314782	2.22471553	1.1753812	1.59749362	1.40958109	4.5594387

8.17450489	8.17646551	7.57349969	7.23787371	7.48539866	7.12341871	11.4939672
0.73596829	1.08217926	0.7100156	0.37117301	1.23235222	0.42790854	4.57004653
135.917572	146.014038	95.8047711	135.849322	77.7294751	122.734239	160.256351
7.09683704	6.3327527	5.96413101	5.96969926	7.02897191	8.73436852	4.47728533
2.70731191	2.24451994	2.74539364	2.28890023	2.82984583	3.80083472	6.83553105
0.5782608	0.32064571	0.33134061	0.24744867	0.27385605	0.42790854	0.29753014
93.5731107	85.0512735	91.1186681	87.6586927	101.098525	100.659192	95.7478949
14.2199587	14.1484918	12.9222838	13.238504	12.4604502	12.2331501	5.7887075
0.07885374	0	0.09466875	0.09279325	0.13692802	0.07551327	0.07038353
1.65592864	1.8036321	1.56203431	2.13424481	1.73442164	1.93817399	1.27240774
10.8555322	13.4671197	14.2476463	14.9087826	16.4313629	11.4528463	6.37513813
48.0482153	48.497663	44.3523076	47.4173521	51.1197957	52.5572377	52.2227225
40.0577024	42.6859596	39.4768671	43.0251382	40.0742684	47.674046	162.797334
12.5903146	12.7456668	10.9342402	11.8775363	14.0123011	13.869271	7.35332846
178.13061	168.779884	172.155115	167.893925	176.865365	133.482295	102.326627
28.1507869	26.2528672	31.4300237	26.9409744	26.8835354	26.0017368	8.53020638
175.659859	173.669731	181.290649	200.216908	189.325815	182.037329	153.268273
3.04901147	2.32468137	2.74539364	3.03124625	2.09956304	4.32942762	5.48501593
0	0.08016143	0.14200312	0.03093108	0.04564267	0.02517109	0
60.8488065	62.8465584	54.7658696	62.4498591	61.2068268	67.5843789	27.6866093
0.36798414	0.32064571	0.09466875	0.34024193	0.31949872	0.42790854	0.1666295
0	0.04008071	0	0	0.04564267	0.05034218	0
0.02628458	0.04008071	0.14200312	0	0.04564267	0.05034218	0.06171505
19.7397208	19.6395495	23.3831803	24.2809011	22.2279826	21.3954272	17.3659186
0	0.04008071	0	0	0.04564267	0.05034218	0
21.737349	25.6516565	21.5371397	23.7241416	26.9748208	23.9125363	12.1697844
1128.71251	1476.33299	1355.98779	1094.21804	1460.70252	1001.07945	619.581506
1.89248988	1.72347067	1.98804367	1.82493397	2.46470444	2.24022708	2.01484334
16.5592864	15.511236	15.7623462	13.7952636	16.2944349	14.0202976	15.7824163
2.68102733	5.57121914	4.73343731	5.07269781	4.56426747	5.26075798	3.20749267
6.04545378	3.64734491	4.26009358	4.1138342	3.788342	4.37976981	6.43070834
0.9988141	0.88177569	1.2306937	0.95886361	1.14106687	1.58577872	0.58210318
228.754714	266.296259	277.09542	233.37503	265.640367	213.324995	202.483555
67.3410982	70.5019746	66.6941316	59.5732682	65.2233822	66.502022	46.885244
1.73478239	1.64330924	1.42003119	0.71141494	1.73442164	1.56060763	0.77905057
1.91877446	2.00403566	2.41405303	2.19610698	2.19084839	2.84433326	1.74708488
10.8029631	8.05622337	7.00548721	8.10394407	7.9418254	7.29961634	8.03838542
0.52569163	0.9218564	0.7100156	0.64955277	0.41078407	0.60410618	0.35568077
0.02628458	0	0	0	0	0.07551327	0.03493052
0.02628458	0	0	0	0	0.07551327	0.07861502
4.83636302	4.8497663	4.49676544	4.88711131	4.92940887	6.0410618	8.049322
26.3634354	23.3670558	22.578496	27.7761136	26.381466	23.9628785	30.583217
42.5021685	42.6458789	39.4295328	47.6648008	44.5016079	41.2302468	26.9739439
70.4426788	72.5861717	66.8361348	78.9361269	71.9328554	73.3485587	82.6981686
10.6978247	10.1003397	8.42551841	9.74329153	8.58082285	14.4985483	8.27735071

2.97015773	2.28460066	2.60339052	3.00031517	2.51034711	3.49878163	2.08274589
454.670693	412.951589	433.772195	431.024659	418.771541	349.022346	309.511388
23.6298389	23.0063294	19.6437648	26.3532838	23.1408361	22.3519287	8.84382254
11.5915005	14.8699446	13.7269682	12.2177783	11.9583808	12.45969	16.6330931
3.67984143	3.56718348	3.92875296	2.66007324	2.96677386	4.05254562	3.54588093
430.357455	474.956452	514.098626	410.888523	474.638175	421.439574	735.265276
0.31541498	0.24048428	0.28400624	0.21651759	0.1825707	0.22653982	0.41222489
15.2450574	14.8699446	13.4429619	15.5274043	17.8462858	14.3726929	14.0429625
0.78853745	0.80161427	0.99402183	0.64955277	0.86721082	0.62927727	1.15314555
0.60454538	0.72145284	0.94668746	0.89700144	0.82156815	0.95650145	1.54713694
798.446737	905.623716	882.928061	837.459105	802.261294	733.686956	707.517664
68.9707422	63.2473655	65.7947786	76.2451226	56.7794874	81.6298476	24.5835936
0.13142291	0.16032285	0.09466875	0.03093108	0	0.17619764	0.05326079
17.0849781	15.3509132	12.8749495	16.9502342	14.6969413	17.9973299	10.6986445
14.9822115	12.4250211	12.8749495	14.321092	12.8255916	17.0659996	24.3393343
49.0470294	42.9665246	44.3049732	44.78821	37.7921347	44.4269753	58.8797927
47.0231166	46.2932238	46.1510137	46.2419709	43.0410423	41.4567866	51.8455936
3.4432802	3.9279099	3.45540923	4.17569637	3.46884328	4.50562526	4.00391061
142.304725	136.474829	152.984694	157.037115	153.907099	152.964719	41.0661181
0.10513833	0.24048428	0.23667187	0.24744867	0.09128535	0.02517109	0.09387895
11.3023701	11.6634876	12.8749495	9.4030496	11.1824553	12.0066103	13.9234873
67.2096753	61.8445406	60.8720038	68.5742137	67.5511586	66.9047594	25.3911693
7.54367493	7.13436696	7.14749033	5.96969926	8.26132413	5.46212671	6.43294978
0	0.12024214	0.09466875	0	0.04564267	0.05034218	0
15.0084961	12.585344	14.0109744	16.3934746	14.4687279	14.5488905	22.1334205
4.65237095	5.450977	5.20678104	4.29942071	6.75511586	6.29277271	9.16453604
0.13142291	0.04008071	0.14200312	0.06186217	0.22821337	0.20136873	0.31126371
0.07885374	0.04008071	0.04733437	0.06186217	0	0.02517109	0.13770253
23.2881393	21.6035044	20.6377867	21.7136211	21.2238437	17.6952769	19.6740593
8.06936657	11.3428419	9.32487149	9.2174631	8.17003878	8.43231543	2.18581428
0.05256916	0.04008071	0	0.06186217	0.04564267	0.07551327	0.06162634
313.52249	314.312953	347.481633	304.763973	324.017348	274.742456	129.987272
20.2128433	20.3209216	19.6910992	19.3937898	17.252931	19.0796869	41.7317697
15.0347807	16.072366	13.7743026	13.6096771	18.7134966	16.9653152	16.811541
4.25810223	5.25057344	6.05879975	3.9282477	8.62646552	5.61315326	5.33422811
30.8843834	30.2609385	21.4898054	33.9932616	27.6594609	28.7453857	13.8643712
0.36798414	0.48096856	0.28400624	0.46396626	0.5477121	0.45307964	0.4635938
0	0	0.04733437	0	0.04564267	0.05034218	0
2.31304318	2.00403566	1.89337492	2.47448674	2.82984583	2.01368727	4.65763238
0.23656123	0.16032285	0.56801248	0.18558651	0.22821337	0.25171091	0.14074994
7.25454453	8.21654622	6.91081847	5.53666408	7.39411331	7.24927416	7.89886738
5.07292426	5.61129986	4.73343731	6.06249251	5.29455027	5.78935089	5.38683467
0	0.04008071	0	0.06186217	0	0.02517109	0
0.13142291	0.24048428	0.18933749	0.21651759	0.31949872	0.22653982	0.2423025
40.3205482	34.0285256	36.7314735	44.8500721	34.8710035	40.0220344	42.6489693

23.5772697	24.6496387	26.2232427	24.6830052	23.0495507	21.345085	43.3710179
0.86739119	0.44088785	0.7100156	1.02072578	0.59335477	0.88098818	0.3463012
40.1628408	48.2571788	42.6009358	42.0972056	43.1323276	36.7749637	31.5850927
75.6995951	80.8828794	71.9482471	73.430394	83.7086655	87.0919743	36.768791
20.1339895	22.8460066	36.163461	20.6928953	36.3315691	32.8231024	35.3366039
159.363419	122.246175	106.786346	125.858582	122.413654	107.581242	346.880646
25.1543446	26.2528672	23.8091897	23.5385551	24.3275456	22.3015865	11.5290144
50.5189659	53.2672679	57.4165945	50.7269781	51.3023664	48.504692	133.475393
7.04426788	7.37485124	7.43149657	7.57811564	9.63060437	7.09824762	2.43697658
496.410609	621.531621	653.498355	692.732562	508.18554	374.243779	150.533413
1.60335948	0.9218564	1.18335933	0.83513927	0.77592547	1.40958109	1.50238686
5.151778	3.52710277	4.68610293	4.05197203	2.82984583	4.6818229	6.24278769
0.68339912	0.48096856	0.56801248	0.55675952	0.45642675	0.40273745	1.19342196
0	0	0.04733437	0	0.04564267	0.05034218	0
0.60454538	0.40080713	0.47334373	0.92793253	0.45642675	0.50342182	0.64391072
7.99051282	5.77162271	6.01146538	7.42346021	7.43975598	7.29961634	12.1048628
3.96897183	4.32871703	2.74539364	2.7528665	3.92527003	3.0205309	1.75626146
0.52569163	0.96193712	0.89935309	0.77327711	0.31949872	0.70479054	0.49638814
0	0.04008071	0	0.06186217	0	0.02517109	0
0	0	0.04733437	0	0.04564267	0.05034218	0
0	0	0.04733437	0	0.04564267	0.05034218	0
210.460645	231.626442	213.288685	215.187553	217.487345	174.360146	46.2048471
2.31304318	2.56516565	3.6920811	3.27869493	2.60163246	3.87634799	1.8819499
0.15770749	0.08016143	0	0.09279325	0.22821337	0.12585545	0.16638314
144.80176	166.89609	165.528303	145.685407	164.4962	131.040699	67.7117206
14.7456503	14.6695411	15.8096806	16.6718544	14.9707973	17.2421972	7.03652697
0.7096837	0.68137213	1.37269682	0.95886361	1.14106687	1.18304127	1.14758179
2.39189693	2.76556921	2.69805926	2.87659083	3.51448595	2.64296454	5.52591557
388.249556	397.159788	441.29836	382.834029	408.319368	340.967596	288.717303
12.4851763	9.0582412	11.4549183	11.3207768	12.0040235	13.0386251	14.7351011
1.15652159	1.00201783	0.47334373	0.64955277	0.3651414	1.03201472	0.75675626
0.23656123	0.08016143	0.37867498	0.06186217	0.09128535	0.276882	0.22839927
18.662053	17.2347067	16.4250275	17.5379248	14.3774425	16.2101825	8.65719173
6.04545378	5.61129986	6.48480911	6.06249251	6.02483306	8.05474907	31.0727504
0.34169956	0.04008071	0.28400624	0.24744867	0.13692802	0.07551327	11.4519464
0.02628458	0.04008071	0.04733437	0.03093108	0	0.15102655	0.9456924
4.57351721	4.36879775	3.83408422	4.48500721	4.3360541	5.56281107	13.4488371
53.278847	53.3474293	41.0389014	56.20178	49.9787288	53.488568	9.85115311
2.75988107	3.28661849	2.74539364	3.03124625	2.92113118	4.02737453	1.73802928
100.012833	102.887191	111.519783	93.7830474	106.301789	93.0575228	101.027437
0	0	0.04733437	0	0.04564267	0.05034218	0
137.599785	156.835831	145.269191	139.77757	153.222459	112.338578	78.6927493
0	0	0.04733437	0	0.04564267	0.05034218	0
23.183001	21.4031009	30.0099925	24.4664876	21.7259132	22.1253888	65.7991271
20.6596812	16.5132539	20.6377867	18.5586505	18.7591393	26.2031056	23.3681773

7.83280533	8.17646551	8.52018715	7.39252913	7.39411331	7.55132725	25.050341
4.49466346	5.05016987	5.49078728	5.6913195	5.29455027	5.66349544	2.19010382
89.1835855	91.9050755	93.2013806	87.2565886	90.509424	79.817529	55.9758681
0	0.04008071	0	0.06186217	0	0.02517109	0
0	0.04008071	0	0.06186217	0	0.02517109	0
38.32292	43.9685424	43.0742795	32.1683276	44.5928932	39.1410462	12.3303166
6.20316127	7.17444767	6.6741466	5.16549107	6.20740376	6.61999689	5.95389238
0.18399207	0.12024214	0.09466875	0.09279325	0.09128535	0.10068436	0.52464986
83.8478155	91.0232998	86.6692371	80.1733703	89.1857864	75.8656678	48.2567857
126.087138	136.514909	135.186969	123.662475	128.210273	119.386484	224.024022
4.4420943	3.40686063	4.82810605	4.14476529	5.43147829	4.80767835	9.34470945
0.07885374	0.12024214	0.23667187	0.12372434	0.3651414	0.15102655	0.07097912
8.41106613	7.37485124	7.47883094	8.07301298	8.71775087	8.86022397	20.9982313
0.05256916	0.08016143	0.04733437	0.12372434	0.04564267	0.10068436	0.15780879
61.3744981	62.9267198	57.7479351	56.8822639	68.9204388	69.7994349	39.2772795
3.5747031	4.08823275	4.78077168	4.26848962	4.97505155	4.6818229	1.88445666
1.15652159	0.88177569	1.08869058	1.26817445	1.09542419	1.33406781	1.55810114
13.2737137	10.901954	9.70354648	12.1868472	16.7965043	9.46433015	8.25917525
6.88656039	6.85380197	8.52018715	6.34087227	7.57668401	6.16691725	5.05352605
6.57114541	7.65541623	7.19482471	6.83576961	8.39825215	8.38197325	6.10819561
0.18399207	0.08016143	0	0.09279325	0.13692802	0.22653982	0.21405116
0.60454538	0.52104927	0.75734997	0.5876906	0.7302828	0.17619764	0.92731217
0.10513833	0.24048428	0.42600936	0.24744867	0.04564267	0.17619764	0.17863079
0.05256916	0.28056499	0.09466875	0.37117301	0.09128535	0.15102655	2.04149471
29.5175852	29.8200507	42.6009358	34.0241926	37.3813506	39.5689548	15.1971495
14.2462433	13.9080075	14.7683244	13.0529175	13.2820183	13.2148227	7.41426062
514.336694	425.496852	451.333247	491.866101	533.973652	483.536655	531.851651
4.38952514	4.32871703	5.34878416	4.08290312	3.37755793	4.83284944	2.61105833
15.4816186	17.8759981	17.7977243	17.0739585	13.2820183	16.3108669	54.1872276
1.07766785	1.2024214	1.9407093	1.29910554	0.82156815	1.25855454	1.12386633
60.8225219	52.2251694	68.1614972	52.9540162	72.1610687	72.2662018	82.9930791
0.52569163	0.6012107	0.94668746	0.71141494	0.91285349	0.85581709	0.43246189
190.904917	209.381646	238.186565	223.693601	201.238553	161.699088	125.960719
68.4976198	62.0850248	50.3164386	66.1925202	64.9951688	64.1359394	27.4638249
0.15770749	0.04008071	0	0.03093108	0	0.07551327	0.30285911
10.1458485	8.73759549	9.8455496	9.77422262	11.09117	10.6977136	14.6359282
0.63082996	0.40080713	0.99402183	0.37117301	0.63899745	0.57893509	0.35693108
17.6895234	13.8679268	14.6263213	15.6201975	13.3733037	13.3155071	6.06398586
166.617963	167.617543	179.018599	166.31644	146.147844	164.191026	207.346186
0.02628458	0.04008071	0.04733437	0.03093108	0.09128535	0.07551327	0.03049174
3.99525641	3.56718348	3.6920811	2.81472866	3.56012863	3.59946599	1.54956827
25.443475	23.6476208	26.0339052	25.3634891	28.4353864	26.8323828	8.67102057
14.2988124	17.3148681	21.063796	17.5997869	18.7134966	21.2444007	2.94991596
19.1351754	20.0804373	22.6258303	18.0328221	15.290296	22.7798372	15.229524
1.81363613	1.96395495	2.17738116	1.45376096	1.50620827	2.06402945	3.25784784

5.38833924	6.21251056	6.72148097	5.6913195	6.11611841	8.63368416	3.40644757
0	0	0.04733437	0.06186217	0	0.02517109	0
55.7233131	54.5899315	58.363282	50.8507025	53.7214282	53.3627126	69.3091087
0	0	0.04733437	0.06186217	0	0.02517109	0
1.23537534	1.32266354	1.32536245	0.86607036	0.91285349	1.40958109	1.59832518
0.9988141	0.84169498	1.7513718	1.11351903	1.73442164	1.45992327	0.96435247
52.4377404	55.63203	49.9377636	43.4272423	56.0492046	47.4223351	11.3473051
54.2513765	54.3494472	59.9253163	61.4600644	50.5720836	43.4453028	27.6635723
0.2891304	0.24048428	0.75734997	0.21651759	0.09128535	0.52859291	0.05591478
3.89011808	5.97202628	4.68610293	5.75318167	6.57254516	4.30425653	5.73685855
0	0	0.04733437	0.06186217	0	0.02517109	0
16.270156	16.5934153	17.2770462	14.6613339	17.0703603	14.5740616	13.2466172
13.9833974	14.2286532	13.4902963	14.4138852	14.4687279	16.361209	19.2848751
170.718358	153.308728	158.806822	168.265098	149.023333	129.127696	85.1180979
54.6982144	57.1550971	59.6886444	57.8101964	57.0533434	44.2507777	26.4472412
5.99288462	6.57323697	7.95217467	6.2790101	6.43561714	5.68866653	3.14298285
9.77786437	10.3007433	8.37818403	9.34118744	11.9583808	10.2194629	6.34542043
135.39188	127.977717	123.779386	143.551162	129.899052	141.713241	232.290173
3.33814187	3.28661849	2.74539364	2.78379758	2.51034711	2.94501763	4.29842084
29.3598777	30.4613421	33.4654018	26.5388703	26.9291781	32.8734446	18.6332068
6.17687669	6.81372125	7.52616532	6.80483853	7.66796935	8.07992016	5.4853592
77.6446542	83.9690943	74.1729626	79.678473	75.9494108	64.8910722	16.0931146
6.36086876	2.7254885	5.86946226	3.64986794	2.92113118	5.86486416	3.02768758
27.0731191	26.0123829	27.8799457	30.6527045	26.3358233	29.651545	31.0095018
0.02628458	0.08016143	0.14200312	0.03093108	0	0.07551327	0.02484818
4.20553306	4.60928202	3.31340611	3.89731661	3.46884328	3.39809726	5.04864246
17.1901164	17.1144646	16.9457056	15.3727489	17.0247177	15.0774834	27.0288127
12.3537534	11.9841333	10.6975683	13.0529175	12.6886636	12.45969	14.503037
3.78497976	2.68540779	5.39611853	3.83545444	3.788342	6.11657507	1.63897699
2.2867586	1.96395495	1.79870618	1.91772722	1.91699234	3.24707072	1.34650211
18.3203534	21.0423745	21.2057991	18.4967884	18.1201419	19.3062267	24.3598028
0.07885374	0	0	0	0.13692802	0.07551327	0.29408727
10.7766785	14.0683304	13.7269682	12.9910554	14.1035865	15.0019701	13.0913129
10.5401172	10.340824	7.00548721	9.8051537	5.47712097	9.18744815	5.99840438
38.8223271	38.1969197	37.7254953	42.5611719	38.8419162	40.9533648	19.7960738
56.984973	66.5740647	72.9896033	56.2327111	61.9371096	64.3121371	64.3070834
0	0.04008071	0	0.03093108	0	0.05034218	0
0	0.04008071	0	0.03093108	0	0.05034218	0
8.96304234	9.0582412	8.6148559	9.06280768	9.2198203	10.8990823	15.1024211
33.6705491	36.9143369	28.6846301	37.2100943	35.5556436	34.1571703	16.8082787
0.2891304	0.08016143	0	0.09279325	0	0.12585545	0.54878039
9.14703441	10.1805012	9.135534	8.07301298	9.72188972	8.18060452	7.4521695
166.460256	181.766035	156.014094	170.925171	168.832254	122.910437	48.6276727
34.9059244	35.2710277	28.0219489	32.2920519	31.3108749	27.5875156	10.8872778
0	0.04008071	0.04733437	0.03093108	0	0.10068436	0

1676.27291	1679.94302	1723.63386	1698.92073	1596.48948	1334.9488	528.494837
99.8288411	96.9953261	99.3548491	91.061112	94.0695526	92.6296143	37.9605693
35.8784539	35.7920769	36.1161266	33.3127777	37.88342	44.905226	19.9181232
0.86739119	1.60322853	0.80468434	0.95886361	1.36928024	1.6361209	0.73787683
0.05256916	0.12024214	0.18933749	0.09279325	0.13692802	0.25171091	0.0474591
4.36324055	4.64936274	4.63876856	5.16549107	3.87962735	6.24243053	3.5207704
16.585571	19.2788231	17.7503899	18.8988925	22.4105533	17.1415129	10.9877524
7.56995951	5.93194556	5.53812165	6.03156142	7.25718528	8.10509125	7.78496848
33.6442645	33.7880413	30.3886675	32.6013628	30.4893067	28.2167928	15.8324806
0.02628458	0.04008071	0.04733437	0.03093108	0.13692802	0.05034218	0.04711142
0	0.04008071	0.04733437	0.03093108	0	0.10068436	0
21.8424873	18.3970474	15.1469994	19.9505493	19.6719928	20.9926898	12.0766523
42.6335914	41.1228118	48.1390574	41.1692731	42.4476875	39.7954946	36.6887405
88.0007794	95.1115326	87.0005777	95.4533259	95.6670462	97.0597263	28.5885161
0.15770749	0.08016143	0.37867498	0.21651759	0.41078407	0.22653982	0.09860529
72.3614533	69.9809254	73.841622	75.1625347	73.6216343	81.6801898	123.285098
0.39426872	0.44088785	0.33134061	0.68048385	0.3651414	0.42790854	0.52595761
0.07885374	0.28056499	0.04733437	0.15465542	0.27385605	0.17619764	0.31471656
0.26284582	0.04008071	0.18933749	0.09279325	0.04564267	0.17619764	14.290463
22.1053332	18.5573702	16.1410212	22.394105	20.0827769	19.5831087	13.5036847
37.7446592	43.046686	41.7015827	40.5506514	37.88342	35.9946599	15.0423476
21.9476257	24.4893158	25.0398834	21.4352414	24.4644737	20.4137547	26.0535733
13.4839904	14.7096218	14.342315	11.9703296	13.190733	13.8944421	6.21974619
9.51501856	8.21654622	10.2242246	7.51625347	8.6721082	9.18744815	9.86823407
97.6735054	107.255989	109.437071	94.8347042	102.55909	96.430449	152.595863
183.045827	176.515461	158.333478	152.33559	203.885828	109.695614	59.3708774
0.10513833	0	0.14200312	0.06186217	0.09128535	0.22653982	0.10439462
0.18399207	0.08016143	0.14200312	0.12372434	0.04564267	0.10068436	0.29249486
3.46956478	3.60726419	2.60339052	2.25796915	1.55185094	4.17840108	2.90703254
0.5782608	0.6012107	0.5206781	0.52582843	0.45642675	0.40273745	0.35564501
78.8537449	77.1152923	78.1963843	79.3691621	74.9909146	65.2434674	47.3078102
16.9535552	13.1865547	15.9990181	14.5685407	16.8877897	19.0796869	31.7110618
255.801549	277.438697	324.193121	259.944832	304.801782	222.285903	50.6873358
0.21027665	0.20040357	0.18933749	0.24744867	0.1825707	0.35239527	0.15356915
32.7505887	32.425297	29.7259863	30.838291	29.8503093	27.8643976	11.8163436
121.539906	123.488678	131.305551	116.239015	125.471713	109.645272	41.7049023
0.13142291	0.24048428	0.14200312	0.06186217	0.09128535	0.17619764	0.16329884
5.30948549	6.37283341	4.73343731	5.50573299	3.33191526	5.46212671	6.52437511
0.21027665	0.04008071	0	0.15465542	0.22821337	0.10068436	0.44311513
144.092077	165.693669	166.143649	152.644901	156.326161	132.475451	92.5036555
19.3980213	20.2006795	19.5490961	19.6412385	16.3857202	18.6014361	14.5355674
3.02272689	3.9279099	3.73941547	3.74266119	3.788342	4.43011199	2.33143587
11.2235164	9.81977475	10.1768902	11.011466	11.09117	12.0066103	13.5400023
1.07766785	1.12225997	0.94668746	0.80420819	1.27799489	1.71163418	4.48399731
2.1553357	1.60322853	1.56203431	2.38169349	1.96263501	1.9130029	0.95115094

45.2883342	42.4855561	48.9910761	56.4801598	46.5098856	43.8983824	27.1438277
0.02628458	0.08016143	0	0.06186217	0.04564267	0.07551327	0.03964678
14.0622512	14.6695411	12.8276151	15.063438	12.8712343	9.76638324	24.912592
19.9237129	18.6776124	15.3836712	24.3736944	23.5516202	13.013454	5.92785713
57.300388	50.3012951	48.7544043	58.2432316	57.3728421	52.2300135	24.1414572
7.54367493	6.6533984	6.20080287	6.34087227	7.62232668	8.73436852	3.17279232
193.980213	205.413655	189.574164	200.433426	214.657499	215.615564	666.553757
4.02154099	4.60928202	2.4613874	3.03124625	5.11197957	4.88319162	7.43125661
35.7996002	38.0766776	39.5242015	44.1695883	31.8129443	27.6126866	14.3021306
2.36561235	2.80564993	2.60339052	2.78379758	2.28213374	3.77566363	5.80505266
0.07885374	0.04008071	0	0.06186217	0	0	1.85501202
106.505125	109.01954	118.856611	134.36463	101.281095	124.773097	148.305237
11.2760855	8.73759549	7.52616532	9.31025635	7.80489738	7.80303816	3.34275279
0.49940705	0.68137213	0.66268122	0.40210409	0.63899745	0.45307964	1.66010134
27.0731191	30.5415035	24.4718709	21.9610698	27.9333169	26.1779345	20.9674884
571.40052	589.026162	647.912898	607.857667	576.512625	477.143198	217.008955
38.2703509	44.0086232	39.334864	41.4167218	41.7630474	28.7957279	23.86114
93.1788419	101.12364	105.224311	100.12392	100.459527	77.2500778	38.3979469
0.63082996	0.80161427	0.66268122	0.49489735	1.00413884	1.08235691	1.48400962
69.2598726	73.9088353	77.1550281	72.9973588	74.1693464	57.7676535	39.7674641
25.5486134	26.7739165	28.3059551	23.507624	22.9126227	28.267135	11.1938548
83.0329934	76.0331131	67.4041472	80.7301298	78.9161846	82.1584405	119.991776
3.33814187	3.36677991	2.36671865	2.56727999	2.32777641	3.0205309	1.925271
0.23656123	0.04008071	0.04733437	0.09279325	0.27385605	0.20136873	6.50296349
4.99407051	3.96799061	4.87544043	5.13455998	5.43147829	4.95870489	3.41199269
26.1005896	30.1406964	33.4180674	23.9715903	30.5349494	28.3174772	68.6633697
79.9051282	80.0812651	82.7878185	85.8337587	77.3643337	63.0284114	27.0167957
8.77905027	11.4230033	10.1295558	10.3309821	10.3152445	6.44379925	6.63738129
136.679825	145.773554	135.612979	134.179043	122.68751	133.406781	116.555188
17.0849781	16.7136574	13.6322994	19.6103074	14.9707973	17.2421972	8.60605398
0.21027665	0.20040357	0.09466875	0.40210409	0.1825707	0.15102655	0.1287107
0.15770749	0.16032285	0.14200312	0.09279325	0.41078407	0.20136873	0.0801834
0.26284582	0.28056499	0.37867498	0.18558651	0.31949872	0.20136873	0.17446306
0.21027665	0	0.04733437	0.09279325	0.04564267	0.02517109	0.10830667
0	0	0.04733437	0.03093108	0	0.05034218	0
21.6584953	22.7658451	18.9337492	24.6830052	25.1034711	19.2307134	16.0963052
0.18399207	0.16032285	0.14200312	0.09279325	0.13692802	0.12585545	0.36011967
0.55197621	0.24048428	0.18933749	0.49489735	0.5477121	0.35239527	0.38452413
103.66639	98.5985546	89.4619651	100.928128	69.8332923	97.7141746	26.5050827
55.4078981	54.0688822	55.6178883	56.7585396	60.9329708	58.2962464	34.6610411
81.0616498	79.119328	81.8884654	79.7403351	73.9411331	74.254718	34.1465298
15.2976265	13.0262318	9.46687461	15.8676462	8.4895375	8.70919743	10.4339901
14.0359666	15.0302675	16.4250275	14.5685407	14.7882266	12.4345189	9.73079697
8.51620445	10.1404205	9.8455496	8.47511708	9.2198203	8.25611779	10.3556138
12.7217375	11.463084	10.1295558	12.2177783	9.99574577	9.03642161	8.76750094

4.31067139	4.97000844	3.171403	4.94897348	4.42733945	4.70699399	4.34006653
3.10158063	3.24653777	2.69805926	2.59821108	2.69291781	2.71847781	6.51350716
0.15770749	0.12024214	0.28400624	0.15465542	0.09128535	0.12585545	0.28538646
18.9248988	16.8338996	17.6557212	16.7955787	16.705219	16.059156	15.3824253
30.2535535	31.0625528	40.9915671	25.332558	42.9953996	23.006377	10.4323401
0	0.08016143	0.09466875	0.12372434	0.13692802	0.02517109	0
0	0.08016143	0.09466875	0.12372434	0.13692802	0.02517109	0
24.5235147	30.7419071	24.8032115	25.4872134	28.6179571	32.3951939	43.3202229
2.54960442	1.96395495	1.89337492	3.43335035	1.09542419	3.19672854	4.85808963
154.84247	175.272959	165.433634	198.237319	209.226021	136.024575	64.713534
123.300973	128.538847	119.187951	118.280466	121.409515	107.153334	25.5929862
7.91165908	5.09025058	7.19482471	4.60873155	5.65969167	7.50098507	302.444251
0.15770749	0	0.04733437	0.06186217	0.04564267	0.02517109	6.22326613
17.5581005	19.7197109	19.028418	18.9916857	17.8462858	15.0774834	17.8393404
1.99762821	1.72347067	1.2306937	1.97958939	1.55185094	2.29056927	8.22784232
157.470929	165.413104	168.368365	163.965677	157.51287	135.017731	61.8798163
2.12905111	1.68338996	2.13004679	1.7630718	2.37341909	2.76881999	1.36787651
12.6954529	12.9861511	12.306937	11.9084674	13.053805	9.99292306	8.92025309
15.8758873	17.0343031	17.4663837	14.7850583	16.2944349	15.505392	24.4132485
2.36561235	2.24451994	1.46736556	1.97958939	2.41906176	2.7436489	3.42058954
15.5341878	16.1926082	17.135043	17.8472356	16.979075	17.7959612	21.0907249
95.0187627	112.666885	107.165021	101.237439	105.79972	85.6823932	48.2603501
21.8162028	18.1164824	14.8156588	23.9097281	18.3939979	18.0980143	20.3410363
15.9547411	16.072366	16.7563681	19.0535479	14.0123011	10.9242534	14.6196253
2.49703526	2.96597278	2.4613874	3.21683276	2.37341909	3.37292617	1.61447487
1.94505904	1.68338996	2.41405303	1.42282987	1.59749362	2.08920054	2.46949879
23.183001	20.6014866	22.7204991	23.6313483	19.6719928	28.8964123	69.0850762
0.15770749	0.08016143	0.14200312	0.15465542	0.09128535	0.276882	0.31141445
3.28557271	2.1242778	3.12406862	3.06217734	3.4232006	3.32258399	2.14757826
38.7171888	33.9082834	33.9860799	38.0761647	30.03288	30.8849285	31.6697873
0.02628458	0.16032285	0	0.03093108	0	0.05034218	0.02558977
1.05138327	0.96193712	1.79870618	0.80420819	2.41906176	1.53543654	1.25050235
60.4282532	65.7323697	59.7359788	60.5012007	57.4184848	56.3832435	65.0140654
22.5784556	26.6135936	25.8919021	23.2911064	24.8752577	21.6723092	23.1516281
0.76225287	0.24048428	0.33134061	0.5876906	0.45642675	0.72996163	0.2541782
30.8843834	26.8139972	25.6078958	30.8073599	28.1158876	30.5325332	23.2566399
239.557677	241.967266	239.937937	262.140939	252.586562	257.52543	258.305314
18.6357684	16.4330924	13.4429619	18.9916857	18.804782	15.530563	10.3480985
121.90789	84.2496593	215.087391	111.815869	298.45745	104.460027	152.638819
0	0.04008071	0.09466875	0	0.09128535	0.12585545	0
244.840878	266.176017	230.329059	262.481181	232.366857	189.060063	80.0722472
7.20197537	5.85178414	4.02342171	7.33066696	4.74683817	7.47581398	7.76217658
0.39426872	0.24048428	0.5206781	0.24744867	0.22821337	0.37756636	1.53355525
0.02628458	0.08016143	0.09466875	0.06186217	0.1825707	0.07551327	0.06877763
6.99169872	8.05622337	8.94619651	6.21714793	7.98746808	7.47581398	34.7676508

2.73359649	1.76355138	2.27204991	1.91772722	2.00827769	1.98851618	2.5039074
10.5401172	4.72952416	5.01744354	5.2892154	6.29868911	6.0410618	18.3538366
64.1343792	66.8546297	70.1022065	72.5643236	69.9702204	71.4103847	32.2195654
3.8638335	3.68742562	5.06477792	3.83545444	4.38169677	2.89467545	6.73300659
0.13142291	0.12024214	0.33134061	0.09279325	0.13692802	0.15102655	0.05163569
1.65592864	1.56314782	1.70403743	2.47448674	1.00413884	2.29056927	1.0794954
11.2235164	12.3448597	13.7743026	10.2072578	12.7343062	11.2263065	12.3245622
103.193268	110.462446	107.922371	102.536544	102.193949	96.6569888	67.2355817
13.19486	10.5011469	10.271559	12.4342959	10.8629566	14.0202976	11.6879637
0.86739119	0.72145284	0.47334373	0.68048385	0.59335477	1.28372563	0.71356212
5.36205466	4.88984702	4.68610293	5.01083564	4.3360541	4.07771672	1.02955933
14.2199587	10.5011469	7.85750593	9.68142936	13.9210158	13.3155071	10.9094174
66.4211378	74.3096424	64.8007567	64.8006215	70.7005032	56.2070458	12.8899817
60.691099	66.253419	66.5047942	66.2234513	58.1487676	56.4587567	32.5126025
20.8436732	23.8480244	19.9751054	16.2388192	23.1864788	20.0110172	0.61118589
39.3743033	32.9463463	30.0573269	38.5401309	37.4269933	34.5347366	29.935367
0.05256916	0	0.14200312	0	0.04564267	0.05034218	1.83734524
0.13142291	0.20040357	0.14200312	0.27837976	0.22821337	0.15102655	4.40459475
0.60454538	0.6012107	0.56801248	0.27837976	0.50206942	0.62927727	2.54134522
2.91758856	3.12629563	2.60339052	1.67027855	2.64727513	3.12121526	1.38657464
44.5786505	53.2672679	48.8017386	45.5614871	49.248446	42.4384591	44.6620857
60.7699528	58.9988099	65.7947786	64.9243458	66.4557344	66.3006533	33.7827714
6.22944585	6.49307555	7.05282159	6.95949395	5.29455027	5.4872978	5.43343271
11.1446626	11.9440526	11.549587	14.3520231	10.6347432	12.7114009	34.5579257
0.05256916	0.12024214	0	0.06186217	0	0.02517109	0.24221124
10.5926864	10.4209854	7.76283718	9.89794695	4.47298212	20.7409788	2.57228333
2.97015773	3.80766776	2.65072489	3.95917878	1.55185094	2.36608254	4.74018292
0.05256916	0.08016143	0.04733437	0.03093108	0.13692802	0.12585545	0.05452063
61.7950515	54.2692858	44.2103044	58.1504383	46.5555282	42.5894857	30.5302813
5.25691633	4.40887846	5.15944666	4.5159383	4.8837662	3.95186126	3.59558755
17.1638318	21.9642309	19.8331023	19.5484452	24.0536896	20.4137547	25.6364624
1.91877446	1.72347067	1.98804367	1.39189879	1.96263501	1.56060763	2.77611892
42.7124452	44.6899953	37.678161	42.0972056	37.1074946	37.7818073	29.2468543
17.8472309	22.1646344	20.3537804	17.8781667	19.5350648	18.4000674	14.8974785
58.0889254	54.1891243	51.8311385	62.3570658	51.4392944	61.9460545	69.8371765
9.35731107	8.05622337	7.62083406	7.76370214	8.07875343	7.09824762	3.708021
283.452928	295.074211	303.034656	289.2675	277.005393	255.184519	245.048617
17.531816	14.6695411	16.5196962	16.5790611	17.5267871	18.6769494	10.8865037
40.6359632	44.2891882	49.3224167	38.1689579	44.1821091	42.9670521	46.8735271
66.9993986	70.5420553	62.1500318	63.2540672	71.2025726	50.7952613	20.2019664
25.6011825	28.938275	28.542627	27.7761136	24.5101163	25.1207487	13.1891133
0.07885374	0.08016143	0.04733437	0.03093108	0.04564267	0.05034218	0.22857968
0.23656123	0.12024214	0.04733437	0.03093108	0.13692802	0.12585545	0.14878246
123.064411	142.366693	153.410703	121.868472	154.500454	108.764283	40.5666619
185.700569	187.417415	178.16658	185.617436	182.114272	141.889439	87.5225475

7.85908991	7.17444767	7.81017156	7.42346021	9.08289227	8.63368416	2.6952366
4.23181765	4.36879775	5.4434529	5.35107757	4.5186248	5.68866653	4.43022697
1.94505904	2.16435852	1.79870618	2.1651759	1.78006431	2.81916217	2.40826122
114.443068	130.502802	137.885029	125.672995	133.77868	92.7051275	180.757289
0.26284582	0.12024214	0.42600936	0.24744867	0.1825707	0.15102655	0.72827954
1.31422908	1.8036321	1.32536245	1.54655421	1.27799489	2.34091145	2.82048611
0.02628458	0.04008071	0.14200312	0.06186217	0.04564267	0.17619764	0.025448
2.83873482	3.08621492	2.88739676	2.69100433	2.87548851	4.48045417	1.11069327
0.49940705	0.80161427	0.75734997	0.86607036	0.1825707	0.47825073	0.68874554
0	0	0.04733437	0	0	0.07551327	0
49.572721	53.8684786	45.0149888	47.726663	46.5555282	45.0814237	20.7997186
0.07885374	0	0.09466875	0.06186217	0	0.02517109	0.06546361
0.34169956	0.84169498	0.61534685	0.37117301	1.09542419	0.830646	10.0721938
2.1553357	2.04411638	2.08271241	2.07238264	2.23649106	2.86950436	1.05802184
15.1924882	14.1484918	17.4190493	15.2799556	12.7343062	14.8006014	28.4605622
18.7671913	18.316886	16.7563681	16.2388192	17.3442164	16.4367223	8.07798338
1.26165992	1.32266354	1.60936868	1.26817445	1.14106687	1.13269909	1.73998873
17.1901164	18.597451	16.1883556	18.2802708	18.804782	18.9790025	24.3807725
22.9201552	23.5273787	20.306446	21.1568616	21.5889851	19.3817399	15.0822423
63.4772647	61.6842177	67.2148097	66.3162446	54.3147829	59.0765502	65.7222202
17.9523693	19.6395495	19.0757523	15.4655421	19.2612087	17.6952769	14.9140027
0.05256916	0.04008071	0.14200312	0.06186217	0	0.05034218	0.05410777
0	0	0.04733437	0	0	0.07551327	0
71.9934691	89.7807977	77.6283718	73.7087737	80.0116088	70.37837	36.3094416
50.0195589	50.8624251	49.4644199	47.757594	49.6135874	43.5963293	36.2381936
31.2786522	31.5435213	32.3293768	28.5803218	30.7631628	29.651545	19.4523206
0.05256916	0.12024214	0.04733437	0.06186217	0	0.05034218	0.15220611
0.07885374	0.04008071	0.09466875	0.03093108	0.04564267	0.05034218	0.0755961
2.26047402	1.8036321	1.13602495	1.54655421	1.23235222	1.661292	1.185257
4.99407051	5.57121914	4.35476232	5.38200866	5.38583562	5.81452198	1.66258618
105.532595	121.40448	115.069861	103.247959	122.550582	91.8493105	21.3294457
19.8974283	26.97432	22.4364928	20.3835845	18.0288565	24.8438667	44.2549656
0.73596829	0.76153355	0.85201872	0.86607036	0.31949872	0.65444836	1.51438043
0.05256916	0	0.04733437	0	0.13692802	0.07551327	0.0495623
41.7662002	45.7721745	44.8256513	40.8290312	42.1738315	41.4064444	72.3170244
434.852119	477.882344	442.339716	442.345436	445.153007	365.886976	141.985371
280.141071	294.553162	303.271328	297.680755	268.42457	251.106802	111.553531
76.9349705	74.1894002	72.6109283	83.9469626	79.4638967	74.2798891	50.2072107
16.5067173	13.3468775	13.8689713	17.6925802	11.365026	19.3817399	16.3237059
1.10395243	1.16234068	1.2306937	1.1753812	1.32363757	1.56060763	1.24695176
0.02628458	0.04008071	0.09466875	0.09279325	0	0.12585545	0.01868849
747.244372	834.280047	695.436609	751.254174	647.441341	698.824995	539.765596
10.4875481	10.7817119	9.37220587	12.4033648	10.8173139	10.2949762	5.92028755
53.9359615	50.5016987	54.6712009	55.7068827	55.8209912	57.9438511	69.5981725
8.72648111	10.5412276	11.4549183	8.97001443	6.61818784	11.5787018	13.8098006

1.78735155	1.84371281	1.46736556	2.10331373	2.32777641	1.58577872	2.38174383
0.81482203	1.16234068	1.08869058	0.37117301	1.09542419	1.00684363	0.57867042
29.2284548	30.7819878	26.1759083	25.7965242	31.7673016	35.5919224	19.3740116
9.83043354	8.49711121	9.98755272	9.09373876	8.94596425	13.2651649	11.789632
10.8555322	10.3007433	11.6915901	11.2279836	9.44803367	9.3384747	49.6426644
0.02628458	0.08016143	0.09466875	0.03093108	0.09128535	0	0.06762049
0.07885374	0.04008071	0.04733437	0.06186217	0.04564267	0	0.41047074
121.066783	128.338444	102.28958	144.695612	74.4888452	96.7576732	85.829738
0.63082996	0.48096856	0.66268122	0.30931084	0.86721082	1.08235691	0.9925209
20.5545428	19.0383388	15.1943338	22.8890023	18.667854	18.3245541	18.6321375
6.59742999	7.73557766	7.47883094	6.21714793	6.80075853	6.77102343	5.89520742
0.07885374	0.08016143	0.04733437	0.06186217	0	0.05034218	0.12527354
289.840082	312.028353	286.088951	295.299061	274.495046	257.424746	143.516334
1.23537534	1.12225997	1.2306937	1.33003662	1.82570699	1.15787018	1.6791294
0.31541498	0.36072642	0.5206781	0.34024193	0.31949872	0.80547491	7.49208738
5.23063175	4.28863632	4.73343731	4.85618022	3.19498723	4.53079635	3.70608445
2.18162028	2.20443923	2.55605615	3.5261436	2.09956304	2.34091145	1.41916722
1.36679825	2.28460066	1.42003119	1.48469204	1.32363757	1.45992327	1.19641085
18.3729226	17.9561595	19.4544273	18.3112019	20.9956304	20.7661499	12.093261
63.9766717	56.9947743	58.1739445	61.5219265	66.5470198	63.0787536	74.6416027
0.13142291	0.24048428	0.37867498	0.34024193	0.3651414	0.25171091	0.19253618
116.703543	121.324319	129.554179	123.384095	130.583692	139.095448	316.194297
6.59742999	8.29670764	5.58545602	6.65018311	8.03311075	8.10509125	5.69149433
5.59861589	7.97606194	5.91679663	3.9282477	7.30282796	5.94037744	1.07523422
30.0169922	28.5775486	28.2586207	29.0442881	36.8792812	28.0657663	60.4433656
0.31541498	0.04008071	0.23667187	0.24744867	0.04564267	0.10068436	0.19143761
13.8256899	15.8318817	16.7090337	18.2493397	14.4230852	17.4939081	34.692847
4.8626476	3.72750633	3.55007798	4.94897348	3.83398468	4.9335338	7.22770986
1.36679825	1.04209854	0.66268122	1.05165686	0.5477121	0.93133036	0.61731001
19.1877446	20.0804373	17.7977243	20.1361358	13.0994476	18.6769494	32.3084452
0.34169956	0.28056499	0.42600936	0.30931084	0.59335477	0.45307964	0.48660773
3.28557271	2.88581135	3.26607174	3.5261436	3.69705665	3.42326835	3.20765579
5.12549342	5.37081558	4.54409981	5.59852625	5.56840632	5.53763998	6.52439191
0.55197621	1.00201783	0.66268122	0.40210409	0.82156815	0.57893509	0.1938907
21.237942	19.0383388	21.6318085	23.7241416	22.1366972	20.8164921	28.1169499
40.2679791	40.7220047	38.7195172	38.6947864	43.4518263	28.4685037	9.98869023
7.85908991	9.9800976	7.81017156	9.24839418	9.90446042	9.0615927	13.9940451
8.80533485	9.69953261	7.95217467	8.69163467	6.93768656	8.38197325	7.10516917
0	0	44.3049732	0	43.9538958	36.9259903	0
2.73359649	2.56516565	2.55605615	2.78379758	3.24062991	2.91984654	3.12666511
7.54367493	6.3327527	7.00548721	7.23787371	6.20740376	5.4872978	6.90206916
24.8652142	24.048428	32.2347081	23.7550727	27.7963889	23.4342856	15.0357145
23.1041473	21.1225359	20.5431179	18.7751681	21.5889851	19.9858461	21.5912629
0.31541498	0.08016143	0.18933749	0.09279325	0.41078407	0.37756636	0.14604182
15.0873499	15.4711553	14.5316525	16.7646477	14.7425839	17.1163418	10.686817

104.113228	97.1957297	98.9761741	88.3391766	95.7126889	97.537977	152.801654
119.647416	118.999638	107.307024	121.868472	118.94481	128.775301	69.039114
14.5353737	13.7076039	11.4549183	11.4754322	15.290296	17.0659996	15.7005815
81.8501873	74.9509338	75.9716688	90.5662146	50.4351556	121.173631	42.6250016
71.3363546	72.0651224	67.6408191	77.6060903	64.7669554	83.416995	38.4426045
31.6466363	30.3010192	29.3946457	33.0962601	27.3856048	24.3656159	13.740147
0	0.08016143	0.04733437	0.06186217	0.09128535	0.05034218	0
20.5019737	18.7978545	20.0224398	21.2496549	20.3566329	22.1002178	18.6953131
7.14940621	7.89590051	9.04086526	8.84629009	8.99160692	8.45748652	13.4137475
25.1806292	22.8059258	23.856524	26.7553879	23.4146921	26.7065274	31.7230617
22.6310248	23.2868944	26.7912552	24.4664876	21.7715558	21.2444007	23.5409859
17.3478239	16.4731731	15.9043493	18.2802708	17.6637151	17.6701058	10.523847
3.04901147	3.72750633	2.88739676	2.62914216	3.14934456	2.79399108	1.64853518
28.5713403	29.499405	25.7025646	23.8478659	26.0619673	28.2923061	22.9487195
1.81363613	2.4048428	1.9407093	1.7630718	1.32363757	2.11437163	0.93120436
823.916496	630.5097	800.234911	678.349608	955.575038	845.396257	1771.32674
11.3286547	11.182519	9.70354648	11.382639	9.85881774	8.15543343	4.00872909
29.2284548	40.2811168	38.1041703	31.5497059	35.0079315	32.9993001	37.8958541
0.36798414	0.40080713	0.14200312	0.18558651	0.31949872	0.22653982	0.25752265
17.1375472	14.8298639	14.0583088	15.9913705	17.7093578	16.361209	21.0061214
75.2264727	82.0051393	97.4141398	83.8541693	77.8207604	76.2432341	89.2564853
20.3179816	24.6496387	25.7025646	25.6109377	25.3773272	24.7683534	10.0200313
1.70849781	1.44290568	1.08869058	1.45376096	1.32363757	2.24022708	2.04699335
52.8845783	53.1470258	56.1859008	54.5005704	57.6010555	60.6119867	39.5433532
6.28201501	5.33073486	7.19482471	5.2892154	5.97919039	5.68866653	3.96424888
33.5128416	34.2690098	32.6607174	32.2611208	31.7216589	30.2808223	44.828612
418.792239	396.15777	422.648617	353.851604	436.891683	355.239605	153.315092
35.5367544	30.5014228	30.578005	31.1476018	35.0992169	37.5804386	32.4293833
0.02628458	0.08016143	0.14200312	0.06186217	0.04564267	0.02517109	0.02996602
0.36798414	0.36072642	0.56801248	0.52582843	0.27385605	0.17619764	0.3355569
60.2705457	62.1251056	54.7185353	54.4077772	57.8292689	54.1178453	183.403162
11.749208	13.1063932	11.928262	13.4550216	14.9707973	11.7045572	8.36104313
9.96185644	8.33678836	8.09417779	10.1144645	9.40239099	9.48950124	17.8552268
0.34169956	0.04008071	0.37867498	0.09279325	0.1825707	0.25171091	0.4760775
33.6442645	31.583602	29.1579738	33.776744	33.3191526	33.804775	36.3573616
23.6561235	23.487298	23.9511928	27.6214582	18.4852833	31.5393768	6.37194485
23.3669931	15.791801	17.6557212	25.1160404	15.290296	22.7043239	54.2915452
73.9122436	71.7845574	82.4091435	78.2865742	69.2399376	65.8475736	41.5895857
34.8796398	30.1406964	36.0687923	35.2923671	34.9166462	37.8573206	15.5664903
0.86739119	0.84169498	0.89935309	0.89700144	0.63899745	0.62927727	0.78510313
2.68102733	2.36476208	3.73941547	2.25796915	2.82984583	2.54228017	0.43995724
24.944068	25.6115758	26.9332583	26.6935257	27.9789596	27.9147397	49.2951713
209.277839	201.966714	208.697251	201.144841	200.553913	178.437863	182.340811
7.75395159	10.3809047	9.46687461	8.35139274	13.1450903	9.3384747	4.57563666
12.7743067	10.621389	10.7449027	11.011466	9.72188972	10.6222003	16.9854579

28.3084944	27.7358536	26.649252	25.8893175	30.5805921	32.2189963	15.3464943
367.668728	351.708259	326.512505	372.317461	325.75177	266.712878	166.610017
16.2438715	18.036321	21.821146	14.692265	16.6139336	19.8096485	58.4437905
0.15770749	0.24048428	0.28400624	0.09279325	0.31949872	0.10068436	0.21640073
76.4092788	83.0071572	76.4450125	76.8018821	77.318691	67.9619453	61.5341314
31.3049367	28.0564993	30.2466644	33.1271912	26.7009647	23.1322325	12.6135929
11.9069155	17.2347067	17.4663837	17.1358207	16.1118642	17.5190792	9.1406052
2.31304318	2.16435852	2.08271241	1.7630718	0.95849617	5.58798217	2.01317088
0.5782608	0.24048428	0.5206781	0.30931084	0.13692802	1.38441	0.47731303
2.73359649	1.92387424	2.08271241	1.94865831	2.60163246	3.32258399	2.92945102
0.05256916	0.08016143	0.09466875	0.03093108	0.09128535	0.02517109	0.08467029
121.119352	122.085853	125.720095	125.611133	116.845247	102.345655	113.622331
8.20078947	6.73355983	6.48480911	8.35139274	7.16589993	8.07992016	11.4258599
98.4620429	107.977442	107.733033	97.0308112	109.451134	86.739579	17.2919628
0.42055331	0.40080713	0.23667187	0.37117301	0.50206942	0.50342182	13.0242194
103.508683	94.0293533	99.6861897	118.899088	95.2106195	104.157974	132.034043
111.840895	118.638911	137.553688	115.83691	120.040235	107.480558	103.255336
2.26047402	2.04411638	1.89337492	2.01052047	2.51034711	2.61779345	1.47753384
1.76106697	2.36476208	2.74539364	2.25796915	2.23649106	3.32258399	1.57304658
38.32292	40.2811168	29.0159707	57.8101964	36.9705665	26.5806719	53.0315201
5.7300388	5.1303313	4.82810605	6.71204528	5.02069422	5.71383762	8.7454424
0.55197621	0.76153355	0.18933749	0.64955277	0.68464012	0.60410618	0.9764633
6.28201501	5.29065415	4.16542483	5.13455998	4.60991015	4.4049409	3.51220131
24.8389297	26.8540779	27.8326114	25.8893175	27.1573915	20.9423476	51.1964564
0.2891304	0.36072642	0.42600936	0.40210409	0.22821337	0.17619764	0.29834581
233.538508	215.874722	254.658927	237.210485	208.404453	210.606517	236.885753
1.41936741	1.36274425	1.27802807	1.33003662	2.73856048	1.00684363	0.41163851
43.1855676	42.8062018	47.5710449	39.9938919	44.4559652	41.0540492	80.3471772
40.399402	43.2470896	42.4115983	42.4993097	37.7008493	38.1090315	27.8344092
15.7970336	17.475191	15.1943338	13.6406081	18.0744992	21.4457694	6.50211257
69.1021652	65.972854	69.155519	66.1306581	62.6217497	63.4059778	100.513271
5.59861589	5.731542	6.24813724	6.12435468	6.07047574	7.07307652	5.05626015
0.44683789	0.44088785	0.94668746	0.46396626	0.3651414	0.553764	0.50682449
0.68339912	0.72145284	0.89935309	0.77327711	0.86721082	1.05718582	0.98121137
107.766785	103.167756	84.633859	139.963156	66.7752331	124.445873	186.331478
0.02628458	0.04008071	0.33134061	0.06186217	0	0.12585545	0.02323233
0.15770749	0.04008071	0.09466875	0.09279325	0.04564267	0.30205309	0.15250692
14.8770732	13.8278461	14.9576619	15.774853	15.8380081	15.505392	24.6597623
0.10513833	0.20040357	0.09466875	0.06186217	0.1825707	0.05034218	0.20895884
649.676005	701.332321	701.164068	637.737095	712.938579	539.164766	293.424069
3.54841852	3.20645706	3.26607174	3.09310842	3.87962735	4.35459871	4.71305986
0.94624494	1.00201783	1.18335933	0.95886361	0.68464012	0.93133036	1.99745902
24.1555305	26.8540779	15.4310056	37.7668538	19.5350648	18.7776338	28.5636586
282.033561	277.358536	278.278779	242.468769	280.109095	259.539118	73.7589529
17.4266776	14.4691375	12.9696182	16.5481301	16.6595763	15.7319318	25.8554253

0.02628458	0.04008071	0.18933749	0.09279325	0	0.02517109	0.00675637
2.05019737	1.04209854	1.98804367	1.60841638	2.00827769	2.39125363	4.68342904
1.83992072	2.92589207	1.46736556	2.44355565	1.04978152	1.6361209	1.59063457
20.4231199	16.8739803	18.6970774	16.517199	17.3442164	18.0980143	35.4373076
0.15770749	0.12024214	0.33134061	0.15465542	0.13692802	0.12585545	0.18114671
97.3580904	101.965335	101.390227	101.701405	95.3931902	84.4490097	44.5055001
0.07885374	0.28056499	0	0.09279325	0.04564267	0.07551327	0.04699665
37.7709438	34.108687	43.0742795	39.9010987	36.2859264	30.4570199	30.0322648
9.77786437	8.13638479	10.9815746	9.34118744	11.6845247	10.7480558	10.1022952
17.5843851	20.7217288	22.3891585	18.1874775	18.7591393	19.130029	10.9888732
34.7745015	40.1608747	42.0802577	34.5500211	37.7008493	37.0518457	40.687839
5.57233131	6.25259127	6.29547162	7.0522872	5.02069422	7.92889361	2.12353609
18.6094838	18.9581774	16.3303587	16.6718544	16.6139336	16.7891176	19.2188329
5.20434717	6.89388268	6.01146538	6.30994118	7.12025726	6.54448362	1.9181685
2.60217358	3.40686063	3.07673425	3.18590168	3.01241653	3.97703235	2.63509986
0.36798414	0.6012107	0.28400624	0.55675952	0.50206942	0.50342182	0.26675531
35.3527623	34.5495748	34.8854329	32.6013628	34.3689341	31.4890346	40.0430681
8.33221238	7.93598123	8.89886214	8.97001443	8.21568145	5.4872978	3.77226969
103.298406	99.8811374	109.10573	96.1338098	108.492638	87.3185141	52.9270864
3.02272689	2.84573064	2.69805926	3.21683276	1.64313629	2.49193799	1.84238032
52.2274637	48.6980666	50.7897823	51.6858417	54.451711	55.4015709	20.3338916
2.99644231	2.92589207	1.51469994	3.12403951	1.64313629	1.45992327	6.34719264
129.924687	128.338444	113.034483	128.735173	122.459296	92.554101	179.182589
23.3669931	24.7298001	27.6432739	24.4664876	31.0826615	25.9513947	9.69787907
5.62490047	4.88984702	5.53812165	6.24807901	5.65969167	5.81452198	7.69240684
10.1984177	11.1023576	11.928262	8.72256575	10.8173139	10.8235691	10.5457094
44.8152117	42.7260403	36.163461	45.0975208	39.3896283	29.5508606	20.5787495
9.09446525	7.93598123	7.57349969	7.42346021	6.84640121	8.18060452	9.49242783
15.4290494	19.5994688	17.7977243	21.7136211	15.1077253	20.6402945	9.87657193
15.1924882	12.6655054	16.7563681	17.4451315	14.9251546	15.9836427	48.7149334
0.13142291	0.08016143	0.18933749	0.21651759	0.22821337	0.12585545	0.34798205
0.07885374	0.08016143	0.04733437	0.09279325	0.22821337	0.05034218	0.35726157
23.813831	20.401083	25.1818865	25.9511797	22.3192679	26.2786188	83.7100827
257.431193	240.684683	227.725669	255.3361	220.682332	198.876789	191.261334
42.9227218	41.9244261	43.1689482	51.8714282	50.5720836	35.9191466	9.56145563
8.62134278	9.49912904	10.4135621	8.815359	8.58082285	8.63368416	19.9175858
54.3039457	48.4175016	71.427569	56.5110909	61.5263255	70.7811074	44.1536837
0.07885374	0.04008071	0.23667187	0.15465542	0.13692802	0.17619764	0.14771918
138.572314	132.386596	148.156588	142.839747	121.637728	126.887469	98.2117449
9.33102648	8.29670764	8.04684342	8.78442792	8.53518017	10.8487401	18.5601745
17.1638318	14.6695411	14.6263213	14.6613339	14.0123011	14.3726929	8.2678727
53.594262	51.7842815	49.0384105	52.0879458	40.3937671	51.273512	41.5206279
1.65592864	0.76153355	1.32536245	0.89700144	1.04978152	1.20821236	7.82113176
51.0709421	64.3295448	59.5466413	48.5308712	56.688202	57.7173113	133.20753
11.7754926	9.85985546	9.04086526	9.61956719	11.5475967	6.46897034	7.16656053

34.7482169	33.1066692	39.0035234	32.9416047	30.7175201	35.9946599	34.2384069
4.28438681	4.04815204	4.2127592	3.46428143	3.05805921	4.53079635	6.81981431
6.88656039	7.09428625	6.62681223	7.23787371	6.66383051	8.63368416	16.8822203
25.5748979	20.9221323	21.9158147	22.2085185	23.8711189	17.2170261	9.12509729
1.31422908	1.36274425	0.99402183	1.02072578	1.87134966	1.53543654	1.99587472
87.711649	78.9990858	74.4096345	93.4118744	84.7128043	78.9617119	77.3028592
0.9988141	0.76153355	1.65670306	1.29910554	1.36928024	1.33406781	0.44389994
4.81007844	6.3327527	6.62681223	4.60873155	5.93354771	5.33627126	4.62404568
1.5770749	2.04411638	3.02939988	2.04145156	1.04978152	2.31574036	2.03718321
8.35849696	10.901954	11.549587	8.19673732	9.63060437	10.0180942	5.14586123
33.486557	35.8321577	35.0274361	34.4572278	39.0701296	37.7062941	24.3541095
33.3025649	35.3111084	39.9975452	35.2305049	34.6884328	31.5645479	37.3576683
2.49703526	2.84573064	3.40807486	3.86638553	2.14520571	3.90151908	1.9486995
8.20078947	7.57525481	10.0822215	10.1144645	6.84640121	6.06623289	1.83852378
0.68339912	0.44088785	0.80468434	0.83513927	0.5477121	0.90615927	0.60843674
0	0.08016143	0.04733437	0.06186217	0.04564267	0.07551327	0
14.5353737	15.9922046	13.7743026	13.7643325	12.9625196	14.1964952	8.73540091
242.028428	262.248107	233.642465	232.23058	256.192333	197.467208	37.361324
34.8007861	35.9123191	34.0807486	30.1887382	34.3689341	33.7544328	18.3734524
0.18399207	0.12024214	0.33134061	0.21651759	0.04564267	0.15102655	0.22827058
0.65711454	0.80161427	0.47334373	0.34024193	0.68464012	0.57893509	0.69506143
0.21027665	0.08016143	0.14200312	0.09279325	0.09128535	0.12585545	0.03348891
0.23656123	0.16032285	0.14200312	0.18558651	0.45642675	0.17619764	0.22354722
6.78142207	7.8558198	9.60887773	7.70183997	9.40239099	9.51467234	17.3730131
38.0600742	32.8661849	41.6069139	35.3542293	34.1863634	35.5667513	30.8027313
16.4541481	14.9901868	19.6910992	16.4244057	16.3857202	23.0315481	7.90267652
28.3084944	30.5815842	23.3358459	27.7451826	30.0785226	30.5828754	20.1685291
152.161443	152.587275	151.422659	153.20166	148.29305	123.942451	50.594415
1.5770749	3.08621492	4.02342171	3.46428143	1.78006431	5.16007362	3.73155223
25.1543446	24.9702844	29.2526426	22.2085185	24.692687	26.9330672	31.128922
0.13142291	0.16032285	0.23667187	0.21651759	0.13692802	0.30205309	0.11769232
0.34169956	0.24048428	0.23667187	0.40210409	0.59335477	0.17619764	0.29561248
0.97252952	1.48298639	1.2306937	1.20631228	0.7302828	1.08235691	0.57920207
4.02154099	4.04815204	4.63876856	4.33035179	4.8837662	4.85802053	5.4179426
28.0719332	29.6597278	28.8266332	29.1061503	25.9706819	26.0269079	18.6528965
119.410854	105.933325	116.915901	111.661214	117.940672	136.905563	44.6026076
24.8389297	23.2468137	22.199821	25.8893175	26.2901806	23.2077457	16.0810251
5.96660003	6.6533984	6.24813724	5.13455998	6.48125981	5.53763998	9.59744111
0.05256916	0	0.14200312	0.12372434	0	0.15102655	0.0754778
1.76106697	1.32266354	1.08869058	1.36096771	1.68877897	1.9130029	1.09105459
30.5689685	33.9884448	32.1873737	31.8280857	31.2195895	27.5120023	36.7153353
82.1656022	83.4079643	81.462456	79.0598513	80.1941795	74.1540336	44.7515455
24.9966371	24.8500422	21.821146	23.4766929	22.2279826	21.3199139	16.7230069
0.91996036	1.2024214	0.7100156	0.74234602	0.91285349	0.75513273	0.51765132
0.05256916	0.08016143	0.09466875	0	0.09128535	0.05034218	0.21725366

0.63082996	0.48096856	0.42600936	0.71141494	0.50206942	0.67961945	0.68079841
2.575889	2.80564993	2.22471553	2.7528665	3.28627258	2.66813563	2.11125433
0.34169956	0.36072642	0.47334373	0.46396626	0.1825707	0.78030382	0.26976189
12.0120538	12.7456668	11.549587	11.3207768	11.5932394	10.6977136	9.42840459
45.1831959	36.8341755	40.6602265	48.6855266	37.88342	44.9807393	131.599853
149.585554	148.579204	156.345434	152.490245	145.691418	126.207849	66.9174641
79.0640216	77.3958573	81.7464623	92.7623216	68.9660815	58.195562	37.3355929
25.5223288	31.0224721	31.9033674	30.126876	27.2030341	24.8186956	26.1264343
0.07885374	0.08016143	0.04733437	0.12372434	0.09128535	0.10068436	0.09536394
49.3887289	44.4094303	45.4883325	50.8816336	45.186248	52.0034737	85.5152227
26.6525658	22.3249573	23.9038584	24.8376606	26.1988953	27.0337516	75.0372641
9.04189609	9.77969404	9.46687461	9.24839418	9.58496169	6.99756325	16.3985446
10.4086943	8.05622337	6.86348409	9.52677394	8.6721082	9.91740979	16.8200826
0.23656123	0.08016143	0.18933749	0.15465542	0.09128535	0.45307964	0.46006657
18.5043455	19.8800338	20.0697742	18.2802708	18.94171	17.2170261	10.152991
0.2891304	0.32064571	0.37867498	0.43303518	0.45642675	0.35239527	0.51520606
0.47312247	0.40080713	0.14200312	0.27837976	0.1825707	0.32722418	0.09387134
4.62608637	4.04815204	5.20678104	5.22735323	3.83398468	6.44379925	4.28876342
74.4379352	72.5861717	68.9188472	73.7087737	76.6796935	48.3536655	27.3696514
1.36679825	1.56314782	1.42003119	1.08258795	1.73442164	2.29056927	0.87378321
158.496027	191.385406	154.972737	135.354425	167.462974	164.895816	103.904532
13.6679825	10.8618733	11.928262	11.7847431	9.17417762	12.3086634	15.1379282
0	0.12024214	0.09466875	0	0.09128535	0.02517109	0
35.2213394	33.6277184	36.6368048	34.7665387	33.821222	30.3563355	35.9047882
32.5928812	39.279099	28.9686363	33.3746399	33.2278672	26.9078961	22.0096007
11.118378	11.5031647	9.46687461	10.4547065	12.2322368	12.3338345	18.231834
135.497018	148.098235	143.42315	128.178413	140.807652	131.166554	105.239052
17.5581005	15.0302675	19.2650898	19.5793763	17.9375712	18.4000674	17.824536
15.9021719	16.3930117	17.3243805	16.9811652	16.2487922	20.1620438	9.59215617
9.8830027	8.09630408	8.37818403	9.06280768	10.4521725	9.46433015	14.6542202
1.31422908	0.76153355	0.94668746	1.20631228	0.91285349	1.48509436	1.48927937
29.8855693	30.0204542	31.5720268	31.209464	27.7051036	26.7820406	17.5720148
23.8664001	24.8099615	24.3298678	23.6004173	22.6387667	22.1253888	10.3695315
0.81482203	0.52104927	0.7100156	0.68048385	0.91285349	0.90615927	2.20522078
18.5569146	17.9962403	14.342315	17.5069937	11.4563114	17.2170261	18.9327777
36.0361614	30.9022299	39.5715359	36.8389213	32.4519417	29.7774005	17.334843
69.3912955	74.1493195	58.8839601	78.0700566	63.5802459	75.362246	59.4643433
49.2047368	48.8984702	48.6124011	54.9954678	45.0036773	36.0450021	23.9902073
11.4863622	6.61331769	10.1768902	10.7021551	10.4978152	10.2949762	13.2059189
8.41106613	7.73557766	9.135534	6.95949395	9.81317507	6.16691725	6.72492375
46.4185712	44.3292689	49.5590886	44.0458639	43.0866849	44.9807393	109.61999
15.7444644	10.2205819	11.8809276	13.3003662	10.2696018	10.8235691	23.7777426
151.556898	168.218754	168.321031	151.19114	162.214066	142.59423	52.6248428
133.683382	135.352569	150.901981	134.550216	142.450788	114.553634	44.462456
11.5915005	10.060259	10.6028996	11.2589147	10.5434579	13.2651649	16.5361072

0.31541498	0.12024214	0.18933749	0.21651759	0.41078407	0.276882	1.45876182
5.25691633	3.40686063	3.171403	3.74266119	1.82570699	9.11193488	2.46528976
37.1401139	39.3191797	37.2521516	35.7872645	40.2111964	30.2556512	24.0895835
13.4577058	15.3909939	12.6382776	13.8261946	17.5724298	13.9196132	3.1369309
3.02272689	3.3266992	2.4613874	2.81472866	2.96677386	4.22874326	2.8172627
1.36679825	1.28258282	1.70403743	1.5774853	1.41492292	1.96334509	2.32381399
695.279754	688.746977	800.32958	702.321198	663.233707	656.9403	421.57397
2.20790486	2.24451994	2.60339052	2.50541782	2.19084839	3.24707072	2.23955017
8.75276569	8.49711121	7.95217467	8.41325491	9.31110564	9.76638324	20.6303071
57.1952497	53.6680751	55.9965633	59.1711641	45.5513894	51.0469722	70.1414604
0.78853745	0.80161427	1.18335933	0.74234602	0.95849617	1.56060763	2.44513625
1.5770749	1.28258282	1.08869058	1.42282987	0.77592547	1.13269909	1.40153469
240.293645	273.190142	254.374921	258.088967	262.947449	188.858695	65.4089181
21.7110644	21.0824552	21.7264772	19.3937898	21.8171985	19.0796869	24.591505
31.0946601	29.9002121	26.0812396	29.2917368	30.5349494	33.3013532	48.2099364
0.73596829	1.08217926	1.27802807	0.86607036	0.59335477	1.00684363	0.58575092
0.02628458	0.16032285	0.04733437	0.18558651	0.04564267	0.10068436	0.04082989
30.9895218	31.3030371	25.4185583	31.5497059	33.4560806	29.374663	17.71036
2.05019737	1.84371281	2.08271241	2.01052047	2.32777641	2.4667669	3.72103301
4.17924848	3.9279099	5.11211229	4.26848962	4.74683817	5.66349544	9.54698997
0.5782608	0.48096856	0.89935309	0.74234602	0.86721082	0.72996163	0.89882836
0.13142291	0.08016143	0.09466875	0.15465542	0.1825707	0.12585545	0.12840871
100.669948	98.2378282	95.9467742	89.0505915	91.9243469	96.5311333	76.0877755
20.6859658	22.1245537	17.4663837	18.9298235	21.2238437	15.6060763	19.4278981
179.655116	127.376507	185.692746	195.855625	141.355364	203.785151	1061.6882
10.6715401	9.3788869	13.2536245	8.47511708	13.9210158	7.27444525	5.70307467
8.17450489	6.57323697	8.42551841	7.42346021	8.4895375	9.53984343	20.5123107
7.14940621	7.17444767	6.34280599	6.83576961	8.17003878	7.7023538	23.9938638
1.05138327	1.04209854	0.66268122	1.02072578	1.36928024	0.98167254	1.14019651
8.80533485	10.6614697	12.7802807	8.72256575	13.327661	13.6930734	9.70836995
0.15770749	0.32064571	0.33134061	0.37117301	0.3651414	0.276882	0.13914263
0.05256916	0.08016143	0.09466875	0.09279325	0.13692802	0.10068436	0.27306617
0.05256916	0.08016143	0.09466875	0.09279325	0.13692802	0.10068436	0.27306617
47.9430769	48.6179052	48.1863918	48.2834225	45.0036773	43.2439341	37.5347584
0.73596829	1.00201783	0.66268122	0.61862168	0.63899745	0.72996163	0.51578297
28.7290477	28.0564993	28.8266332	42.6230341	35.1905022	18.5510939	17.5237203
0	0.16032285	0.09466875	0.06186217	0.04564267	0.02517109	0
25.1806292	23.0464101	24.7085427	24.4664876	25.5142552	27.8392265	32.9974215
47.8379386	48.8183088	45.9143419	46.6750061	43.8626104	43.9990668	42.1003208
0.15770749	0.24048428	0.23667187	0.03093108	0.13692802	0.25171091	5.75884329
0.52569163	1.00201783	0.23667187	0.71141494	0.95849617	0.60410618	0.28404189
7.56995951	7.09428625	5.49078728	7.57811564	7.07461458	7.60166943	23.0353731
1.20909076	1.72347067	1.18335933	1.67027855	1.55185094	1.53543654	0.86092137
8.12193573	7.2546091	8.04684342	7.6399778	6.80075853	7.02273434	4.24773167
0.10513833	0	0	0.03093108	0	0.02517109	0.07648776

14.7193657	15.9120432	12.6382776	15.2799556	10.5434579	13.6175601	8.1236108
23.2355702	29.6998085	24.8032115	26.0130418	24.0993323	22.175731	11.8308973
4.17924848	4.04815204	3.21873737	3.12403951	5.84226237	4.25391435	2.52651995
4.4420943	4.92992773	5.34878416	3.58800577	5.11197957	4.48045417	9.988876
3.70612601	2.80564993	5.01744354	3.03124625	3.28627258	3.72532144	3.2710313
0.10513833	0	0	0.03093108	0	0.02517109	0.91215721
0.10513833	0	0.04733437	0.03093108	0	0.05034218	0.09736122
31.226083	30.8621492	35.9741235	29.1061503	32.6345124	30.0542825	10.2573597
30.1484151	27.0144007	27.3119333	28.0235623	29.6220959	32.5713915	31.414065
36.0098769	40.681924	43.3582857	34.7046765	37.472636	40.0975477	14.5260848
9.56758772	11.2225997	7.24215908	10.9186727	7.7592547	6.34311489	2.13224434
161.913023	184.090716	188.722145	157.346425	177.458719	163.83863	144.679194
0.44683789	0.44088785	0.75734997	0.46396626	0.63899745	0.85581709	0.46362189
2.05019737	2.04411638	2.08271241	2.938453	1.09542419	3.19672854	1.93740923
13.2211446	13.5072004	13.1589557	11.2279836	12.9625196	12.7869141	15.8483405
19.9762821	18.7978545	19.407093	19.2082033	16.7508616	18.1986987	16.8936686
240.503922	258.680923	226.87365	254.006064	240.354325	176.650715	85.7115532
87.4488031	90.7828155	92.7753712	90.6280768	78.2771872	86.5633814	125.263271
2.47075067	2.52508494	1.7513718	2.50541782	2.87548851	2.41642472	5.597098
41.7399157	37.0746598	43.3582857	37.7049917	35.9664277	40.8778515	32.9311991
0.23656123	0.04008071	0.09466875	0.15465542	0.22821337	0.20136873	0.42998793
51.4914954	58.1571149	59.6413101	49.5206658	53.9039989	55.2757155	119.864487
1.81363613	1.40282496	1.08869058	1.5774853	1.00413884	1.107528	2.77151078
0.18399207	0.32064571	0.23667187	0.18558651	0.1825707	0.17619764	0.12349783
0.21027665	0.16032285	0.23667187	0.46396626	0.13692802	0.20136873	0.57934309
0.18399207	0.20040357	0.09466875	0.15465542	0.13692802	0.35239527	0.67998427
14.7193657	13.7877654	8.99353088	12.9601243	11.1824553	9.91740979	11.3423517
783.254249	855.162098	832.658957	736.902151	871.409946	670.507518	264.936648
101.195639	108.658814	94.148068	87.2256575	101.235453	96.6318177	67.0046741
59.9551307	58.8785678	51.3104604	65.4811053	48.2899499	43.2439341	45.8819071
6.99169872	6.0521877	6.86348409	6.8667007	5.52276364	5.68866653	6.0572536
277.828028	282.047979	312.406862	302.784383	295.673247	335.656496	169.888996
28.9393244	25.6115758	25.2765552	27.4977339	28.6635997	29.0726099	21.6321719
0.15770749	0	0.09466875	0.15465542	0.09128535	0.15102655	0.11566022
0.23656123	0.12024214	0.33134061	0.18558651	0.04564267	0.22653982	0.27019783
149.480416	152.186468	156.108762	147.417547	138.845017	143.097651	206.735095
121.303344	131.424659	101.248224	125.549271	129.670839	123.942451	44.17642
537.677402	583.29462	519.684082	485.587091	592.168062	436.164662	223.832856
630.540829	698.686994	547.043349	638.603165	590.43364	498.639309	306.509561
110.973504	123.208113	128.560157	112.217974	123.782934	103.60421	41.4890749
0.10513833	0.04008071	0.04733437	0.06186217	0	0.05034218	0.23090515
5.38833924	6.29267198	3.97608734	7.70183997	4.47298212	5.53763998	5.77692905
19.16146	18.7577738	19.1704211	20.0433426	20.3109903	20.7158078	42.2341115
0.34169956	0.20040357	0.23667187	0.52582843	0.22821337	0.276882	0.13142463
80.3782507	87.8569235	82.0304685	78.7505404	81.7003878	76.0922076	85.5376516

0.23656123	0.16032285	0.14200312	0.15465542	0.09128535	0.15102655	0.22217418
1.97134362	1.48298639	2.31938428	2.44355565	1.41492292	2.76881999	5.93969366
10.3298406	13.1865547	10.1768902	12.8982621	11.8214528	11.8807549	5.98453321
39.5057262	38.5576462	38.1988391	38.6638553	35.4643583	35.2898693	49.5659212
0.02628458	0.04008071	0.04733437	0.06186217	0	0.10068436	0.04428862
68.0244973	65.2514012	64.4694161	68.3886272	63.8084593	53.0103173	34.1248168
125.876862	132.667161	131.589557	126.508134	126.61278	112.993027	84.9797523
64.397225	58.5579221	67.0254723	63.6871024	59.4724052	55.2505444	21.7703958
0.15770749	0.24048428	0.14200312	0.12372434	0.27385605	0.32722418	0.28496492
8.48991987	8.89791834	8.52018715	7.98021973	9.49367634	6.19208835	6.81916872
9.43616481	10.8217926	11.0289089	9.77422262	10.6347432	8.28128888	7.68516742
90.9183679	98.4783125	109.910414	93.2262879	98.3143214	88.9294639	105.77185
113.312831	112.546643	89.6513026	91.6797336	103.700157	99.4258088	511.490473
8.8841886	10.7416312	11.0762433	12.0012607	8.62646552	6.84653671	17.0343215
0.52569163	0.28056499	0.23667187	0.27837976	0.5477121	0.57893509	0.24094074
32.5665967	39.8001483	40.5655577	23.4148308	36.9705665	42.1364061	10.8669498
41.7662002	33.5876377	37.299486	44.2005194	34.5971474	41.9853795	95.0548423
30.910668	26.9342393	26.2232427	31.6424992	27.6594609	30.5577043	44.4592181
26.3108662	26.97432	25.7972333	26.5079392	25.6055405	32.3951939	33.6143031
7.35968286	7.2546091	10.1768902	7.23787371	7.43975598	7.72752489	4.64430832
6.96541414	6.0521877	6.43747474	5.22735323	6.89204388	5.66349544	4.95821271
59.4820083	57.7563078	55.0972102	55.2429164	69.3768656	59.6051431	26.2021839
3.49584936	2.44492351	3.21873737	2.47448674	2.87548851	2.79399108	5.85811102
3.04901147	2.68540779	2.79272801	2.69100433	1.82570699	2.89467545	2.20437993
44.2895201	39.3993411	40.3288858	43.4891044	43.3148983	44.880055	25.0827397
37.7183747	38.9584533	41.5122452	35.2923671	39.5265563	36.3470552	22.1721924
24.313238	22.084473	26.7439208	23.6932105	22.1823399	22.3770998	30.9974216
68.5764735	66.0930962	74.1256282	74.5748441	65.5885236	82.4101514	286.798599
240.162223	206.25535	237.665887	239.406592	203.246831	195.025612	188.445741
40.1365562	37.7560319	30.7200081	41.4167218	36.1489984	38.9648486	15.8561144
10.2509868	11.0622769	11.928262	9.49584286	9.58496169	11.3269909	18.6744323
119.121724	125.29231	123.022036	120.538435	115.612895	109.192192	111.70552
42.9490064	43.5677353	46.8610293	44.2623815	40.3937671	40.9030226	22.4285537
3.5747031	4.88984702	3.73941547	4.60873155	4.74683817	4.25391435	2.71270575
7.46482119	8.73759549	6.43747474	8.28953057	8.17003878	8.35680216	3.40287156
5.12549342	5.09025058	6.43747474	4.39221396	5.84226237	4.75733617	5.62831295
59.9551307	58.8785678	51.3104604	65.5120364	48.2899499	43.2439341	45.7531058
51.9909025	53.6680751	60.7300006	44.4170369	70.9287165	65.6462049	44.8200884
100.564809	102.045496	114.927858	91.2157674	103.335016	103.15113	96.1840895
0.05256916	0	0.04733437	0.12372434	0	0.05034218	1.8913848
1.68221323	2.00403566	1.08869058	1.85586505	1.50620827	2.1898849	2.27761668
4.67865553	5.97202628	4.78077168	5.47480191	4.19912608	4.10288781	2.37630493
0.36798414	0.24048428	0.09466875	0.30931084	0.04564267	0.15102655	0.86567228
4.31067139	3.76758705	7.47883094	4.67059372	5.56840632	7.45064289	2.84421836
24.2080997	23.4472173	36.163461	18.7751681	39.3896283	36.4225684	13.1352459

74.3853661	74.2695617	83.4031653	80.8229231	68.6009401	68.7674202	44.3119177
29.0444627	31.3030371	30.6726737	27.9926312	29.4395252	28.1664506	11.3079769
0.02628458	0.04008071	0.04733437	0.12372434	0	0.05034218	0.04304356
251.201747	268.460617	250.730174	230.003542	280.017809	206.327432	183.567143
0.10513833	0.04008071	0.04733437	0.06186217	0.04564267	0.02517109	0.18789506
152.555712	146.454926	145.6952	154.315179	144.732922	176.826913	109.679198
1.97134362	1.64330924	1.84604055	2.41262457	2.19084839	1.71163418	1.89659113
98.0414895	87.9370849	83.4504997	91.9271823	84.2107349	77.602473	51.7062774
119.989115	131.584982	117.105239	131.271521	119.994592	90.615927	34.9769851
26.9942654	29.0985978	28.542627	26.1986283	33.8668646	30.5828754	26.3541
190.773494	215.754479	188.532808	188.834269	211.234299	153.115746	73.7463756
0.07885374	0.12024214	0	0.06186217	0	0.05034218	0.29230492
1.44565199	1.76355138	1.7513718	1.5774853	1.32363757	1.35923891	3.72696479
27.0731191	27.4953693	29.1579738	27.528665	26.5640367	24.5418136	18.496592
74.8847731	76.8347273	74.1256282	82.8334436	76.6796935	79.1379096	45.1926198
10.0669948	8.69751478	9.79821522	7.36159805	9.76753239	9.0615927	13.2417274
39.7948566	38.8782919	38.4355109	45.4377627	43.7256824	35.9191466	13.2158171
2.52331984	2.20443923	1.46736556	2.04145156	1.18670954	2.06402945	2.99102713
0.84110661	0.76153355	0.80468434	0.61862168	1.00413884	0.45307964	0.33829141
0.21027665	0.36072642	0.18933749	0.09279325	0.09128535	0.32722418	0.31103789
0.18399207	0.04008071	0	0.09279325	0.27385605	0.07551327	0.11281944
15.5867569	1.04209854	1.2306937	6.80483853	0.45642675	4.37976981	13.4369628
0.02628458	0.08016143	0	0.09279325	0	0.07551327	0.02923049
0.02628458	0.04008071	0.04733437	0.12372434	0	0.05034218	0.03000797
125.614016	132.306434	149.860625	123.445957	130.903191	127.617431	180.354197
19.79229	20.7618095	21.3951366	19.8886872	18.7591393	19.1552001	16.1950615
2.73359649	2.36476208	2.9820655	2.84565975	2.82984583	3.37292617	4.07056401
66.0268691	58.9587292	63.4280599	69.1000422	63.3520325	67.6347211	68.9749758
42.0027615	38.5977269	42.4589326	43.7365531	43.360541	43.3446184	26.5674041
122.302158	121.40448	97.2248023	101.54675	112.144052	105.240331	15.0915852
10.3824098	12.2246175	12.4016057	11.6610188	12.5060929	13.6175601	13.3340147
0.10513833	0.08016143	0.04733437	0.06186217	0.04564267	0.05034218	3.67469048
71.1786471	58.5178414	74.9303126	74.0799467	66.3188064	78.3827769	163.5151
26.573712	26.693755	29.1579738	28.6731151	24.281903	24.113905	27.1686006
32.0671896	37.1147405	32.7553862	32.0446033	31.4478029	32.143483	23.8972408
5.17806258	4.20847489	2.88739676	4.67059372	4.42733945	4.85802053	8.21030164
0.05256916	0.12024214	0.09466875	0.03093108	0.09128535	0.05034218	0.04434971
42.4758839	39.7600676	46.056345	41.509515	34.6427901	43.7473559	69.0499978
0.05256916	0	0.04733437	0.06186217	0	0.10068436	0.01741324
2.99644231	4.40887846	1.51469994	2.87659083	2.05392036	2.61779345	3.62741589
21.4482186	20.5213252	21.5844741	22.0538631	21.5433425	24.3152737	35.7456267
31.4100751	35.5515927	31.1933518	34.5500211	34.4145767	35.4408959	36.9634269
5.09920884	4.72952416	4.73343731	4.17569637	3.74269933	5.10973144	5.33144195
35.1162011	38.8382111	37.6308266	40.0866852	34.5058621	44.326291	36.4352262
32.5665967	31.7439249	37.6308266	35.3851604	37.4269933	35.742949	35.1772522

51.7280567	42.0847489	45.2516606	42.0972056	49.3397314	38.8641642	26.9612995
3.99525641	4.5291206	5.53812165	3.64986794	5.52276364	6.69551016	3.26148704
50.0458435	47.4555645	50.4584417	45.9635912	46.9663123	46.9189133	29.7114018
35.6156081	36.4734491	41.3229077	34.8284008	37.0162092	34.6102499	13.7568832
0.07885374	0.04008071	0.09466875	0	0.09128535	0.05034218	0.05300034
0	0.08016143	0.14200312	0.03093108	0.09128535	0	0
31.4889288	33.0665884	29.0633051	33.3746399	33.7299366	32.5965626	15.6904045
85.9242974	101.203801	89.4619651	93.1025635	87.7708635	79.4651338	33.9009604
11.9857692	11.2225997	9.70354648	10.7330862	13.053805	12.1576369	14.9155799
15.6393261	14.950106	15.1943338	15.6511286	15.7467228	17.7707901	33.8608094
44.2895201	46.5337081	39.5242015	42.0972056	43.7713251	36.6491083	20.8700762
0.23656123	0.12024214	0.04733437	0.37117301	0.09128535	0.15102655	0.21270259
82.5335864	81.6043322	69.9602034	89.978524	74.9909146	84.0966145	46.1541124
7.85908991	7.57525481	9.135534	8.16580624	8.94596425	9.59018561	7.76567768
15.9284565	17.3148681	16.7563681	15.8676462	15.8380081	14.8257725	12.9554829
18.4780609	16.5132539	17.0877087	18.9298235	14.8338693	14.3475218	5.80928805
18.5831992	18.5172895	20.1171086	19.1772722	20.2197049	21.8233358	19.5885859
1.2879445	1.5230671	0.7100156	0.52582843	1.23235222	1.18304127	37.9644227
4.57351721	3.44694134	4.2127592	5.04176673	3.4232006	5.2104158	2.36563055
0.36798414	0.20040357	0.33134061	0.21651759	0.3651414	0.57893509	1.0794954
43.2381368	42.1649103	36.7788079	39.6845811	37.88342	37.5049253	35.4626725
12.7480221	13.9881689	13.1116213	14.1973677	13.6471597	15.0019701	23.5210674
32.3826046	37.9965162	32.6607174	38.571062	32.7257978	38.6124533	18.6152083
23.997823	24.6496387	23.0045053	21.4971035	23.7798335	21.9995334	44.7161973
0.07885374	0.08016143	0	0.06186217	0.04564267	0	3.01439453
2.23418944	2.68540779	2.22471553	2.44355565	3.37755793	2.39125363	0.3547574
0.21027665	0.20040357	0.09466875	0.18558651	0.04564267	0.12585545	0.10151079
12.7480221	11.3428419	12.2596026	12.8364	12.6430209	13.7182445	24.2904482
13.6679825	14.5893796	15.9043493	11.9703296	14.5600132	14.2468374	11.8580437
65.8954462	60.241312	56.7539133	60.593994	57.5554128	53.9164766	58.2855597
28.1770715	24.6897194	24.1878646	25.3016269	28.8461704	28.3174772	12.281702
9.64644146	11.1023576	9.70354648	7.17601154	10.0870311	10.4460027	6.47836936
0.26284582	0.08016143	0.18933749	0.24744867	0.09128535	0.37756636	0.82024341
0.23656123	0.16032285	0.18933749	0.12372434	0	0.25171091	0.08920526
147.456503	145.733473	160.558193	146.767995	140.853294	138.063433	51.7667869
55.4867519	55.7121914	59.5939757	53.4489135	56.8707727	50.5435504	26.9777927
0.07885374	0	0.04733437	0	0.22821337	0.05034218	0.22563889
27.2833958	27.1346429	25.4185583	28.8277705	27.7051036	28.3426483	35.2618872
0.18399207	0.16032285	0.42600936	0.40210409	0.27385605	0.276882	4.28713889
99.2242957	115.672938	108.774389	98.3917789	104.293512	101.414325	43.2571703
4.04782557	4.56920131	4.40209669	4.63966263	4.47298212	5.2104158	4.29085392
14.3776662	15.511236	13.1589557	13.8880568	14.5600132	18.1986987	26.1337107
84.3472225	98.8791196	89.1306245	86.8544845	68.2357987	98.3182808	195.233141
19.5031596	23.5674594	22.3891585	19.7958939	21.0412731	20.1368727	38.5426946
0.23656123	0.12024214	0.28400624	0.15465542	0.13692802	0.17619764	0.29068998

3.23300354	3.96799061	2.9820655	4.26848962	3.05805921	4.05254562	6.743198
0.02628458	0.16032285	0.09466875	0.06186217	0.22821337	0.12585545	0.02701978
3.94268725	5.21049272	4.16542483	3.86638553	4.79248085	6.26760162	5.14182436
1041.71054	977.20787	1036.6701	1050.2031	889.210589	885.292436	1214.78471
27.9667949	27.7358536	28.6846301	28.3638042	25.6511832	25.2717752	26.3063194
377.131177	443.012124	445.227113	371.296735	418.178186	388.389932	111.744948
2.75988107	2.96597278	1.60936868	1.5774853	3.10370188	1.68646309	8.18453788
17.3215393	14.5492989	16.4250275	18.5895816	15.0620827	18.4252385	98.3256797
7.12312163	7.01412482	5.72745914	8.07301298	7.16589993	6.64516798	5.23339928
26.705135	21.3630202	20.1171086	22.6106226	24.1449749	26.882725	29.2331081
0.15770749	0.16032285	0.04733437	0.06186217	0.27385605	0.22653982	0.1642582
36.2990073	35.9924805	37.5834922	36.0965753	35.8751423	31.3883503	17.6451584
0.52569163	0.6012107	0.99402183	0.34024193	0.95849617	0.42790854	0.43014771
69.864418	63.6481727	70.8122221	74.17274	65.953665	76.8473403	42.5088852
22.8938706	22.4451994	25.8445677	23.0127267	25.0121858	27.9650819	67.4024905
1.81363613	2.04411638	1.42003119	1.39189879	1.59749362	1.6361209	3.15141247
68.9707422	80.2816687	76.1136719	64.4294484	70.8374312	76.3690896	123.439493
0.94624494	1.48298639	0.75734997	1.20631228	1.32363757	1.23338345	1.86999596
47.312247	52.4656537	46.6243575	52.2426013	43.634397	41.4567866	22.7099765
14.0622512	13.9080075	10.3662277	15.3108867	8.6721082	10.4963449	17.2539065
244.026056	305.896004	291.579738	293.752507	265.138298	221.102862	163.211307
11.3812239	13.6274425	14.2476463	10.5165686	11.0455273	14.2216663	19.1374344
40.7673861	42.5657175	44.4469763	37.7668538	44.9123919	37.5804386	62.4939137
2.54960442	2.1242778	1.51469994	1.97958939	1.73442164	3.54912381	2.27076341
0.60454538	0.56112999	0.75734997	0.5876906	0.3651414	0.57893509	1.03503353
1.2879445	0.64129141	1.27802807	0.9897947	1.14106687	1.68646309	3.1167627
64.8440629	67.375679	78.5277249	71.1105626	65.6798089	60.7126711	22.8234174
0.10513833	0	0	0.09279325	0.09128535	0.05034218	0.19253618
30.2009843	32.986427	25.7498989	30.4361869	31.9498723	32.8986157	26.2669174
12.7480221	11.9039718	11.6915901	7.98021973	10.2696018	14.4985483	8.82289545
1.70849781	1.5230671	1.32536245	1.60841638	2.28213374	1.51026545	1.36600014
47.7590849	47.2150802	54.055854	47.5101454	47.1945257	45.4086479	81.3690602
17.0324089	12.024214	10.6028996	16.5481301	10.8629566	16.7387754	9.47283247
9.35731107	11.3829226	9.60887773	9.37211852	12.1409515	11.6038729	4.73687599
67.0782524	77.1954537	77.0603593	76.4925713	69.2855802	61.6440015	30.3348756
0.13142291	0.08016143	0.09466875	0.12372434	0.13692802	0.17619764	0.11856026
1.81363613	2.04411638	1.7513718	2.1651759	2.09956304	2.08920054	1.31745509
66.6576991	67.8967283	61.534685	71.6982532	68.4640121	67.8109187	29.3631191
16.5592864	16.8739803	14.342315	17.9709599	15.3359387	18.1483565	15.1679006
16.6644248	16.6735767	16.6616993	15.3108867	15.7923655	15.5557341	8.61413287
1.23537534	2.28460066	2.08271241	1.14445012	1.46056559	2.03885836	0.65364034
123.222119	113.628822	126.619448	129.786829	123.828577	132.853017	133.512669
0.26284582	0.76153355	0.5206781	0.34024193	0.41078407	0.42790854	8.6901464
0.68339912	0.6012107	0.85201872	0.49489735	0.68464012	0.60410618	0.65645236
0.13142291	0.40080713	0.09466875	0.06186217	0.09128535	0.25171091	0.06690292

0.02628458	0	0.09466875	0.03093108	0.04564267	0.10068436	0.03071016
268.549571	259.562699	243.346012	216.270141	244.142667	261.150067	121.739016
0.07885374	0.12024214	0.04733437	0.03093108	0	0.10068436	0.04819417
0.34169956	0.52104927	0.18933749	0.06186217	0.04564267	0.57893509	0.61742399
87.080819	96.6746804	91.0713338	82.6478571	96.5798997	79.817529	28.1774061
5.67746964	7.17444767	5.34878416	6.89763178	3.37755793	5.66349544	4.88065004
64.397225	77.5160994	78.7170624	72.0694262	81.7460304	80.5474907	71.8105534
27.6513799	23.5674594	24.5192052	27.559596	22.2736253	20.6906367	21.5038308
246.260245	274.552886	274.681367	251.779026	260.57403	232.706735	169.812025
19.3717367	23.3670558	19.028418	21.9610698	20.0827769	24.2901027	11.4010874
0.89367578	1.12225997	0.80468434	1.14445012	1.27799489	0.93133036	0.96531604
7.56995951	8.05622337	7.14749033	7.67090889	7.12025726	6.11657507	5.31738157
50.8080963	43.5276546	45.8196731	49.1185618	45.5513894	54.872978	53.031396
0.07885374	0.08016143	0.09466875	0.09279325	0.04564267	0.02517109	0.0790985
45.3671879	37.7560319	39.2875296	44.4170369	38.4311321	47.7999015	81.4336213
218.162028	212.107135	211.584648	202.413015	191.379735	209.373134	95.9979842
141.805318	153.869858	160.700197	143.891404	147.836623	136.225944	44.9749436
25.6274671	24.9702844	24.613874	25.3634891	26.5640367	28.0657663	7.97093901
44.420943	43.3673317	50.7897823	46.4894196	43.7713251	40.0220344	41.0729293
180.759068	162.246727	166.096315	181.967569	211.553797	155.230117	57.8469342
22.0001948	18.3569667	23.2411772	20.9094129	16.5682909	21.2192296	54.5892787
12.064623	9.3788869	8.23618091	10.9186727	11.5475967	10.2698051	26.9315249
105.532595	73.5881895	113.081817	111.506559	87.8165062	118.807549	181.442684
66.0005845	73.3076246	66.6467973	63.9964133	73.5759917	55.2253733	35.2181213
2.8650194	3.52710277	3.171403	2.84565975	3.14934456	2.54228017	2.66418551
58.0363563	61.3234913	59.3573038	54.2221906	62.0740376	51.9279604	14.231737
144.51263	128.498767	125.720095	140.798295	143.546212	140.505029	206.518893
62.6624426	64.4097062	58.0319414	65.8522783	58.2856956	48.9829428	25.46223
27.3359649	25.8520601	28.2586207	29.6319787	31.4021602	25.9262236	22.3976446
0.05256916	0	0	0	0.13692802	0.02517109	0.16746636
92.6005811	101.845092	64.9900942	64.986208	77.318691	95.6501452	25.6456707
30.9632372	33.6677991	33.1340611	35.1067806	30.169808	25.9262236	28.5432344
2.73359649	3.00605349	3.40807486	3.77359228	1.68877897	2.34091145	2.4687843
0.65711454	0.48096856	0.66268122	0.5876906	1.46056559	0.35239527	0.55513711
51.7806258	50.5818601	40.8022296	52.7993608	56.9164154	44.326291	15.9774189
1.49822115	1.32266354	1.70403743	1.42282987	1.46056559	1.05718582	0.56038889
1.36679825	1.44290568	1.79870618	1.67027855	1.96263501	1.6361209	0.94116756
3.1541498	1.8036321	2.55605615	2.28890023	1.78006431	2.4667669	3.01439453
155.315593	162.647534	140.015076	143.396506	163.263848	119.260628	49.7304745
4.65237095	5.29065415	4.9227748	4.85618022	6.43561714	5.2104158	11.2920176
3.4432802	1.96395495	1.98804367	3.61893685	2.64727513	2.41642472	2.05118771
0.39426872	0.36072642	0.42600936	0.27837976	0.50206942	0.67961945	0.4545741
0.5782608	0.9218564	0.66268122	0.64955277	0.63899745	0.50342182	0.89882836
0.23656123	0.28056499	0.14200312	0.18558651	0.13692802	0.17619764	0.16717613
1.47193657	0.84169498	1.13602495	0.9897947	1.59749362	1.56060763	0.95244556

213.220526	217.237466	225.311616	216.022692	198.728206	201.695951	169.314852
88.7104631	94.0293533	95.9941086	98.1752613	101.737522	95.599803	26.7615791
73.2288445	80.1213458	80.3264311	74.0490156	74.3975598	72.6941103	22.2227157
12.1697613	11.8638911	10.7922371	11.7847431	10.6347432	9.84189652	15.045063
15.1924882	15.6715589	13.6796338	12.9910554	14.1492292	14.2971796	8.18170684
59.3243008	60.2012313	65.9841161	61.3054089	65.7710943	69.7490927	25.7616413
75.7784489	79.680458	75.498325	75.5027766	73.0739222	70.1518302	54.7217595
1.13023701	0.80161427	0.75734997	0.71141494	0.77592547	0.830646	2.02134838
57.3529572	55.7522721	53.0618322	47.1389724	54.862495	54.3947273	31.3000348
0.36798414	0.44088785	0.14200312	0.21651759	0.45642675	0.10068436	0.46503056
17.8472309	0.16032285	0.04733437	18.8060992	17.252931	0.07551327	15.4838687
11.5652159	12.4651018	17.892393	14.2901609	14.3774425	17.0156574	32.8249613
7.22825995	7.09428625	5.4434529	8.25859949	6.89204388	6.67033907	1.26570626
58.3254867	55.2713036	58.5999539	57.4699545	51.4392944	53.4130548	23.6645801
112.603148	114.430436	122.832698	116.022497	115.978036	141.234991	182.565636
0.26284582	0.32064571	0.37867498	0.34024193	0.22821337	0.57893509	0.52070513
4.1529639	4.56920131	3.45540923	4.73245589	4.06219805	4.70699399	6.73774481
42.4495994	41.2831347	52.4464854	43.7674842	40.9414792	42.9167099	70.6022703
0.10513833	0.12024214	0.04733437	0.09279325	0.09128535	0.20136873	0.42376991
0.05256916	0.12024214	0.04733437	0.15465542	0.09128535	0.07551327	0.06815801
0.86739119	0.68137213	0.66268122	0.49489735	0.68464012	0.70479054	1.00100649
48.1533536	51.022748	44.5416451	48.6236644	60.1570453	45.3834768	19.8501393
217.08436	231.586361	259.865708	194.649313	217.943772	248.111442	156.255429
14.6142274	14.5092182	12.5909432	12.5889513	16.2487922	16.4870645	24.2405006
81.0353652	72.0651224	90.6926588	84.1634802	76.9535496	67.7102343	62.9792687
58.2203483	73.1473017	56.5645758	57.9648518	65.9993077	51.2483409	35.432883
126.691684	121.083835	146.547219	125.642064	122.961366	121.400171	126.928805
166.381402	181.52555	192.224889	164.831748	168.512755	172.421972	70.1108918
0.21027665	0.16032285	0.28400624	0.30931084	0.27385605	0.25171091	0.16264833
66.789122	79.961023	69.9602034	72.2550128	72.3892821	58.3465886	30.4972562
16.086164	14.3889761	13.7269682	15.2180934	16.1575069	16.2856958	11.3646939
36.3515764	36.0726419	34.4594236	33.2509155	39.0244869	41.6581553	24.4130377
3.39071103	3.68742562	2.9820655	3.9282477	3.33191526	3.92669017	3.77932289
6.07173836	6.01210699	5.63279039	7.54718455	5.79661969	6.09140398	8.11745151
73.3076982	70.0210061	73.8889564	74.5748441	64.4474567	65.7720604	75.421554
275.19957	254.151803	254.564258	181.812913	275.955611	265.83189	438.343205
46.18201	50.6219408	51.2157917	51.4693242	41.260978	46.289636	56.1289347
1.44565199	0.52104927	0.37867498	0.83513927	0.50206942	0.57893509	3.48805679
3.70612601	4.12831347	3.73941547	3.9282477	3.65141398	5.23558689	6.43070834
0	0	104.040952	0	82.2480999	91.8744815	0
12.56403	14.5492989	13.8689713	13.238504	13.327661	11.3773331	3.80430518
9.51501856	9.6594519	10.8395714	10.0216713	10.5434579	6.79619453	21.5747583
67.0782524	69.8206025	60.9666725	70.3991477	73.6216343	66.1244556	39.1301089
20.3179816	22.8460066	23.477849	23.0436577	20.3566329	28.0405952	40.5459832
39.1903112	38.0766776	36.8734766	41.9116191	40.3481245	38.7131377	17.030526

36.4304302	43.2470896	32.1400393	34.7356076	36.5141398	33.4272086	16.0854751
0.36798414	0.48096856	0.18933749	0.43303518	0.22821337	0.15102655	0.51066317
0.23656123	0.48096856	0.47334373	0.24744867	0.59335477	0.62927727	0.72984079
3.1541498	3.00605349	3.97608734	3.30962601	3.69705665	4.25391435	5.11049669
0.63082996	0.72145284	1.08869058	0.5876906	1.00413884	1.28372563	0.74522936
328.662409	355.916734	377.53896	331.519361	361.033557	299.032559	283.232043
20.0288512	21.7638273	18.2237336	18.9916857	22.730052	22.8301794	37.8686225
108.581607	122.767225	101.011552	111.073523	97.4927532	103.352499	130.285709
1.26165992	0.72145284	2.08271241	0.92793253	0.82156815	1.51026545	0.61623877
35.563039	34.4293327	30.3413331	32.9106736	33.8668646	27.3609757	43.0516991
24.4709455	27.6957729	28.0692832	29.2608057	25.9250392	30.0039403	11.8907437
3.25928812	2.7254885	2.22471553	2.13424481	2.28213374	2.84433326	3.00002947
13.2999983	13.1865547	12.685612	13.4859527	11.1824553	11.629044	14.1537121
19.4243058	15.4310746	19.8804367	16.6409233	17.7093578	25.3976307	11.9344386
126.507691	129.941672	157.150119	136.189564	127.890775	122.885265	159.049328
0.39426872	0.24048428	0.33134061	0.34024193	0.22821337	0.20136873	0.37518718
0.44683789	0.52104927	0.28400624	0.34024193	0.3651414	0.32722418	0.641938
0.10513833	0	0	0	0	0.05034218	1.8913848
0.21027665	0.40080713	0.28400624	0.5876906	0.3651414	0.20136873	6.59559829
0.15770749	0	0	0.03093108	0.13692802	0.15102655	6.53970339
1.20909076	1.44290568	1.56203431	1.54655421	1.09542419	2.13954272	1.72384955
50.5189659	51.583878	55.1445446	48.9948374	51.5305798	47.7243882	13.7621884
221.473885	246.416225	194.260267	241.942941	255.781549	212.091611	86.0764868
0.2891304	0.44088785	0.42600936	0.34024193	0.27385605	0.276882	0.15475343
16.086164	16.5934153	16.8510368	17.4142004	13.2820183	15.1278256	11.7761625
51.4126417	57.2753392	49.8904292	50.1702186	53.9039989	44.9555682	17.2119123
44.5786505	45.4114481	44.9676544	40.8599623	42.9953996	43.7473559	20.0745101
2.10276653	2.32468137	2.41405303	2.78379758	2.55598978	2.34091145	5.24955783
25.6011825	22.2848766	20.7797898	25.1160404	20.4479183	18.1986987	14.7863785
6.99169872	6.69347911	5.96413101	5.1036289	6.84640121	6.06623289	10.2367948
1.23537534	1.64330924	1.56203431	1.5774853	1.32363757	2.11437163	1.64710241
26.5211429	27.4552886	31.0986831	28.9514948	25.4686125	25.221433	22.4907616
160.388517	160.763741	160.605528	160.006499	155.322022	136.679023	46.6978249
3.33814187	3.48702205	2.79272801	3.89731661	3.51448595	3.37292617	1.88614309
109.527852	109.340186	119.992636	109.001141	106.301789	103.931434	176.178578
53.3314162	48.4575823	53.5825103	54.8098812	51.5762224	58.4976151	128.171977
109.396429	124.77126	129.45951	113.764528	117.940672	109.871811	98.2187453
0.05256916	0.12024214	0.14200312	0.12372434	0	0.05034218	0.06595598
0.73596829	0.88177569	0.33134061	0.64955277	0.1825707	0.67961945	0.96962754
0.2891304	0.28056499	0.47334373	0.21651759	0.27385605	0.30205309	11.789632
882.478544	948.55016	1014.70696	870.029537	923.853379	846.252074	1024.21538
7.88537449	6.45299483	7.14749033	6.15528576	5.65969167	7.6771827	5.92146256
19.0563217	16.2727696	18.6024086	17.7853734	17.8006431	14.5992327	14.1624044
170.718358	163.649552	184.935396	178.410494	163.948488	142.543887	64.445997
2188.58569	2373.98065	2487.04263	2483.24023	2399.93748	2780.87694	7616.68463

1567.16561	1624.0705	1519.67005	1826.17121	1833.64881	1458.43817	384.649
77.0401088	74.7104495	76.1610063	75.1934658	74.6714159	65.2434674	34.3823534
429.227218	409.224082	476.562468	467.059372	449.215205	493.756118	631.849983
54.6193607	40.120794	45.4409981	45.2521762	48.5181632	57.1383762	26.6886597
31.6203517	32.5856199	32.3293768	29.3226678	36.0120704	37.7566363	17.7393766
18.4780609	15.5513167	16.5670306	17.3832693	18.2114272	18.8279759	9.75990492
66.0268691	67.4558404	52.4464854	63.4705848	58.8790504	51.4245386	39.8567465
0.21027665	0.32064571	0.42600936	0.30931084	0.31949872	0.10068436	0.30191119
0.2891304	0.72145284	0.66268122	0.43303518	1.32363757	0.42790854	0.26375016
0.02628458	0.12024214	0.14200312	0.06186217	0.09128535	0.02517109	0.02340141
0.05256916	0.12024214	0.14200312	0.03093108	0.09128535	0.07551327	0.17666781
0.05256916	0.12024214	0.14200312	0.03093108	0.09128535	0.07551327	0.20095964
3.10158063	4.00807133	3.55007798	2.3507624	2.92113118	4.02737453	5.81028778
44.1580972	48.6179052	43.1689482	43.1179314	45.9165308	39.2417306	23.2285315
7.04426788	6.89388268	5.91679663	6.40273443	6.38997446	5.46212671	5.79693856
2.12905111	2.00403566	2.88739676	1.79400289	2.37341909	1.98851618	4.75262204
38.1652126	38.6778883	40.092214	40.6125136	39.709127	43.3194473	30.3063567
0.31541498	0.72145284	0.61534685	0.61862168	0.5477121	0.75513273	0.43870665
0.68339912	0.96193712	1.08869058	0.46396626	0.7302828	1.00684363	0.71544038
1.20909076	2.44492351	1.65670306	1.08258795	1.46056559	1.88783181	0.74530759
0.10513833	0.08016143	0.14200312	0.12372434	0.13692802	0.17619764	0.07694536
4.81007844	6.89388268	5.96413101	6.92856287	4.42733945	8.28128888	17.8847967
0.39426872	0.72145284	0.37867498	0.43303518	0.27385605	0.45307964	0.84318728
3.07529605	2.36476208	2.74539364	2.01052047	2.37341909	2.84433326	3.7676158
162.806699	152.627356	157.8128	175.843214	147.654053	182.28904	281.695951
2.02391279	1.76355138	1.56203431	1.70120963	1.64313629	1.40958109	2.018608
0.21027665	0.04008071	0.14200312	0.06186217	0	0.17619764	0.30262157
0.7096837	0.28056499	0.42600936	0.5876906	0.86721082	0.42790854	1.13631626
10.5926864	8.73759549	8.37818403	12.7436067	7.66796935	10.1691207	11.6110012
22.2367561	19.7197109	19.0757523	24.1262457	18.2114272	23.3336012	10.5576931
44.7100734	44.1288653	40.0448796	46.7987304	40.7589085	32.8986157	12.1176857
29.7541464	31.3030371	39.713539	31.5187748	30.4893067	31.9421143	54.6924255
29.8855693	28.6176293	30.3413331	30.3433936	27.3856048	25.8758814	15.415803
385.489674	390.426228	392.685959	367.801523	423.290165	291.934311	132.461753
3.75869518	2.68540779	4.68610293	3.68079902	1.96263501	3.87634799	1.19598295
16.900986	16.9541417	16.4723618	16.7027855	15.5641521	14.7250881	6.62333087
19.79229	19.1585809	19.8331023	19.1772722	21.8171985	21.8485068	7.11687739
0.7096837	0.6012107	0.94668746	0.24744867	0.59335477	0.93133036	0.33091124
243.316372	200.964696	241.689309	187.040266	235.790058	217.453054	545.637645
0.10513833	0.04008071	0.04733437	0	0.04564267	0.07551327	0.06650164
1.44565199	1.40282496	1.2306937	1.26817445	1.36928024	0.95650145	1.8913848
13.9833974	10.1003397	10.7449027	11.6610188	12.4148075	13.9447843	21.2757266
58.0100717	68.2574547	62.5287068	62.6354456	61.0242561	53.9164766	36.3260131
105.585164	115.913423	101.20089	99.9074021	105.160723	99.0230713	83.6804515
68.9707422	72.0651224	78.4330562	65.7904162	73.1195649	68.1129718	54.4855624

15.1136344	12.865909	12.6382776	14.9397137	15.3359387	13.6930734	33.6456533
0	0	0.09466875	0.03093108	0	0.10068436	0
0.07885374	0	0.04733437	0.06186217	0.09128535	0.07551327	0.09583768
19.6082979	22.4852801	26.4599145	20.3217223	21.9084839	21.4709405	55.0150048
34.8796398	33.7078799	35.784786	36.2512307	36.8336385	37.4294121	39.8391688
0.21027665	0.16032285	0.04733437	0.06186217	0.04564267	0.17619764	0.19486995
59.8499924	66.7343876	62.8127131	68.7288692	63.4889605	68.2136562	40.5727982
45.6563183	49.3794387	45.7250044	46.272902	44.3646798	42.4888013	24.4049386
14.7456503	15.791801	14.6736556	15.3108867	13.2820183	13.4161914	6.75332717
0.21027665	0.08016143	0.04733437	0.12372434	0.04564267	0.07551327	0.1771545
2.07648195	2.52508494	2.03537804	2.25796915	1.87134966	1.71163418	1.44654316
28.650194	31.1427142	27.5486051	27.157492	31.6760163	34.5599077	30.6492002
14.4828045	14.3488953	13.4429619	12.8982621	11.3193833	14.8006014	7.89685824
7.51739035	6.61331769	6.1534685	5.66038841	7.80489738	9.01125052	9.6040866
36.5355685	30.8220685	31.6193612	34.3644346	36.9249239	34.2326835	31.7312197
9.40988023	8.7776762	7.10015596	8.47511708	9.58496169	9.6153567	10.3006424
0.05256916	0.08016143	0	0.06186217	0.09128535	0.07551327	0.13467452
0.07885374	0.04008071	0.18933749	0.03093108	0.04564267	0.10068436	0.11663921
0.36798414	0.36072642	0.18933749	0.34024193	0.68464012	0.25171091	0.82748085
6.09802294	5.69146128	3.55007798	4.57780047	11.0455273	2.99535981	1.79253194
23.1304318	18.1565631	21.2531335	24.621143	17.9375712	25.0452354	60.5243138
6.38715334	5.57121914	6.1534685	5.90783709	4.65555282	5.86486416	8.74950798
37.4555288	44.168946	40.5655577	41.9116191	43.9538958	43.495645	19.019841
109.238721	101.043478	117.957258	125.394615	117.073461	106.750596	97.5046474
0.13142291	0.16032285	0.23667187	0.21651759	0.1825707	0.25171091	0.16255582
1.60335948	1.00201783	0.99402183	0.89700144	1.32363757	0.95650145	0.42647663
0.10513833	0.08016143	0	0.03093108	0	0.07551327	0.11267119
0.94624494	0.9218564	1.08869058	1.05165686	0.63899745	0.78030382	0.65862162
52.5165941	44.3292689	53.4878416	51.6858417	50.2525849	58.0948776	138.156508
0.36798414	0.64129141	0.56801248	0.52582843	0.3651414	0.35239527	0.50408688
1406.35654	1628.80003	1571.40652	1456.35917	1527.06697	1269.02572	1415.94671
19.0037525	17.2347067	15.0049963	19.0226168	17.8462858	18.299383	5.17174875
16.6381402	16.8338996	19.4544273	17.5997869	18.9873527	20.0361883	16.7792184
122.748996	129.78135	133.719604	115.218289	124.193718	124.445873	52.1197639
22.8150169	25.6917372	25.0398834	33.4674331	16.705219	30.759073	39.587623
0.34169956	0.16032285	0.23667187	0.18558651	0.27385605	0.15102655	0.45286678
4.70494011	5.57121914	3.64474673	4.79431806	3.01241653	4.50562526	1.70583401
4.81007844	4.32871703	3.78674985	4.67059372	4.24476875	5.41178453	2.93251838
15.4816186	15.1104289	15.1943338	17.9709599	14.1492292	16.9653152	43.4367799
196.240687	237.478226	245.239387	245.809326	227.163592	252.289843	363.44942
84.8991987	79.9209422	77.5337031	84.2562734	82.2024572	60.6371578	46.7083156
24.313238	25.3310108	17.3717149	32.1064654	23.3234068	18.5259229	10.3974921
3.36442645	3.20645706	2.79272801	2.96938409	4.29041142	3.37292617	2.67597746
63.8452488	65.2914819	65.4634379	62.047755	62.3478937	59.7058275	37.2707959
65.9742999	78.558198	70.1495409	69.2237665	71.2938579	61.5684882	29.5191623

7.96422824	6.77364054	7.14749033	8.56791033	7.89618273	7.40030071	10.2769231
34.4590865	42.5256368	43.0269451	27.6214582	45.6883174	37.2280433	12.7389825
140.09682	136.154183	141.624444	181.936637	142.085646	125.603743	38.2968441
0.52569163	0.28056499	0.80468434	0.40210409	0.45642675	0.42790854	0.38821058
4.07411016	4.48903989	4.02342171	4.48500721	5.02069422	4.4049409	1.71825511
10.5664018	11.4230033	12.1175995	11.0423971	9.12853495	11.1507932	26.1125732
11.1709472	10.7416312	12.685612	11.1042592	12.3691649	13.8189289	9.72270026
114.916191	134.831519	114.73852	133.374835	119.173024	84.0462723	72.6487257
131.344054	156.595347	161.031537	132.570627	166.869619	116.819033	49.4601348
3.1541498	2.84573064	3.40807486	3.55707469	3.65141398	3.27224181	3.52689671
29.8855693	29.21884	33.7967424	32.1992587	36.0120704	30.7339019	15.0632785
0.78853745	1.04209854	0.56801248	0.49489735	0.41078407	2.13954272	0.87572061
1.02509868	0.64129141	0.89935309	0.80420819	0.86721082	1.35923891	1.83197681
1.02509868	1.04209854	0.85201872	1.08258795	0.59335477	0.78030382	0.86961729
123.274688	136.635152	149.48195	128.982621	164.724413	144.557575	68.1120644
0.07885374	0.08016143	0.04733437	0.06186217	0.09128535	0	0.08483784
43.6849747	41.4835382	42.2222608	41.849757	41.9912608	36.6491083	14.4293739
200.945627	200.04284	177.125224	215.001966	183.939979	216.974803	189.598015
14.1411049	12.2246175	11.9755964	13.6715392	13.7384451	13.9699554	17.7786284
18.5831992	22.2047151	23.5251834	18.6205127	21.4520571	19.7844774	15.1752697
0.18399207	0	0.14200312	0.03093108	0.09128535	0.10068436	0.3547278
0.05256916	0.16032285	0.09466875	0.03093108	0.09128535	0.07551327	0.1642582
21.7110644	22.084473	18.8864149	24.1571768	20.8587024	22.0498756	16.1993446
37.6920901	34.3491713	37.2048172	38.4782688	39.4809136	37.8069784	29.6706427
6.88656039	5.97202628	7.47883094	5.41293974	6.70947319	6.46897034	7.11205396
0.60454538	1.00201783	0.33134061	0.64955277	1.00413884	0.75513273	0.6594128
1.23537534	0.96193712	1.37269682	0.9897947	0.95849617	1.107528	2.95447988
17.5843851	17.0343031	19.5490961	15.8985773	15.6097948	18.7524627	38.6883442
0.07885374	0.16032285	0.09466875	0.18558651	0.13692802	0.12585545	0.08927406
0.21027665	0.08016143	0.14200312	0.15465542	0	0.45307964	0.26504723
44.8414963	46.6138695	53.1091666	45.0047275	47.6509524	43.7976981	32.955207
94.3879327	92.6265283	79.5690811	93.4737365	78.1859018	79.5909892	33.0982853
17.4266776	18.1164824	18.649743	17.1667517	21.9997692	19.0545158	19.7844994
24.4709455	22.7257644	24.3298678	23.3220375	22.593124	21.3954272	16.0681413
0.60454538	1.00201783	0.47334373	0.74234602	0.5477121	0.42790854	0.413377
2.81245024	1.68338996	1.84604055	2.28890023	3.05805921	2.11437163	1.55993152
0.15770749	0.04008071	0.18933749	0.06186217	0.1825707	0.05034218	0.1566555
37.1401139	34.9904627	38.6721828	38.1689579	35.1905022	30.8345863	48.7739714
45.9717333	42.6057982	38.8615203	41.9425502	46.2816722	47.0699399	87.5276956
0.10513833	0.12024214	0.23667187	0.03093108	0	0.22653982	0.2727766
27.4673878	26.8941586	31.2880206	31.0548086	24.3731883	24.5166425	33.7862756
51.7543413	48.3373402	43.3109514	57.0059882	41.8086901	54.8226358	105.60521
88.3687635	87.4160356	82.0304685	91.4632161	84.5302336	96.0780537	55.2236052
45.3671879	65.1712398	142.902472	51.9642215	72.8457089	92.9064963	18.0507442
0.34169956	0.20040357	0.18933749	0.15465542	0.59335477	0.30205309	0.27940912

0.81482203	0.40080713	0.61534685	0.77327711	0.50206942	0.93133036	25.8898647
1.73478239	2.32468137	1.27802807	1.82493397	1.96263501	2.34091145	1.30794068
9.43616481	7.33477053	8.99353088	9.58863611	8.4895375	9.94258088	13.4535215
142.33101	132.787403	131.163548	124.992511	119.44688	137.836893	182.986262
0.9988141	0.84169498	1.08869058	0.64955277	1.59749362	1.28372563	0.96245339
0.36798414	0.64129141	0.28400624	0.30931084	0.22821337	0.45307964	0.45677279
36.8509835	40.8422468	34.7434298	45.8707979	37.7008493	36.296713	24.6604297
1.34051366	1.44290568	1.37269682	0.9897947	0.95849617	1.56060763	0.69750346
89.7092772	87.4561163	77.2023625	75.8120874	74.4432025	89.5587412	124.846459
0.07885374	0.12024214	0.23667187	0.06186217	0.04564267	0.15102655	0.25317749
36.1412998	28.6176293	39.9028765	36.9935767	34.9166462	40.0220344	15.0813985
0.2891304	0.12024214	0.14200312	0.27837976	0.27385605	0.22653982	0.44238769
20.2391279	18.2768052	21.7264772	20.1670669	18.667854	17.4435659	19.0082358
8.54248904	10.8618733	7.05282159	7.57811564	8.1243961	8.48265761	5.65623873
189.695826	213.710363	226.589644	212.589342	228.943656	228.855558	142.232369
10.5664018	9.25864476	11.5969214	11.4754322	9.2198203	13.3910203	6.71989798
3.10158063	3.00605349	2.88739676	3.00031517	2.92113118	2.16471381	120.448188
6.38715334	7.2546091	6.95815284	6.95949395	5.65969167	6.16691725	4.60554708
14.0885358	15.6314782	20.7324554	16.3934746	15.7010801	14.2720085	9.52171179
13.2211446	13.9480882	15.0049963	12.7745378	14.4230852	12.0066103	20.041179
131.31777	125.773278	137.364351	144.107921	127.890775	147.175368	125.843395
0.31541498	0.24048428	0.28400624	0.34024193	0.1825707	0.50342182	0.57631441
17.0061243	15.6314782	13.6322994	15.7129908	16.3857202	17.745619	28.2079206
129.845833	116.835279	107.070352	126.817445	92.8828431	110.425575	208.44947
48.2584919	51.9446044	49.985098	44.6026235	47.4683817	49.61222	70.7416447
9.43616481	10.4209854	12.8276151	9.09373876	12.3235222	14.3475218	5.65978008
0.97252952	0.84169498	0.61534685	0.52582843	1.32363757	1.15787018	0.15980671
0.18399207	0.16032285	0.28400624	0.15465542	0.22821337	0.40273745	0.63490773
7.25454453	8.4570305	7.99950905	8.01115081	6.52690249	7.17376089	5.17758314
43.3432751	40.401359	34.3647548	37.3028876	37.9747054	36.0953443	13.8798927
13.6416979	13.5072004	11.8809276	14.4448163	14.8338693	12.8875985	7.37577376
27.3622495	26.3731093	26.9805926	26.7244568	27.9333169	30.9352706	23.7220673
0.21027665	0.16032285	0.18933749	0.21651759	0.13692802	0.07551327	0.15824567
0.05256916	0.04008071	0.04733437	0.06186217	0.04564267	0.10068436	0.02312373
16.0598794	15.9922046	18.1763993	15.4036799	15.5185094	15.9081294	7.57064122
35.4579006	36.5536105	33.5127361	36.4677483	33.95815	28.7705568	19.8195694
26.5474275	26.3330286	27.9746145	25.4872134	25.4229698	25.3724596	32.2493318
0.15770749	0	0.33134061	0.03093108	0.27385605	0	0.20666444
33.0397191	29.2990014	29.1106394	29.3226678	31.6760163	25.0704065	20.8819436
0.2891304	0.24048428	0.28400624	0.77327711	0.13692802	0.17619764	0.20056079
43.290706	49.8604073	50.7424479	43.8912085	49.8874435	41.834353	39.1692923
2.70731191	2.60524636	2.36671865	2.19610698	2.05392036	2.56745127	6.81443373
4.99407051	4.08823275	5.39611853	5.38200866	5.93354771	4.73216508	5.76881296
0.76225287	0.64129141	0.66268122	0.61862168	0.31949872	0.72996163	0.6206008
542.934319	573.875652	486.266014	595.578027	539.131274	583.742768	164.39692

0.05256916	0.08016143	0	0.09279325	0.04564267	0.07551327	0.03936767
62.5573043	52.4656537	45.5830013	53.5726379	57.0077007	61.3419484	42.5495853
2.73359649	2.24451994	2.27204991	2.62914216	2.92113118	2.66813563	3.91794767
91.3126366	86.6545021	102.857593	98.1752613	89.8247839	110.828313	42.2551178
92.4165891	84.009175	93.2960493	95.2677394	86.994938	102.748393	134.027093
13.2474292	12.7857475	12.7802807	13.238504	10.3608872	12.3338345	13.1164589
184.491479	184.651846	222.234882	197.98987	186.039542	170.811022	164.075397
25.3383367	21.7638273	19.2650898	19.9196182	21.1325584	21.068203	16.5004068
0.15770749	0.08016143	0.14200312	0.30931084	0.09128535	0.12585545	0.24529085
9.98814103	11.8638911	11.0289089	9.83608478	8.39825215	11.7800705	9.68173204
11.0920935	10.8217926	12.9222838	11.0423971	10.0413884	11.07528	8.50441529
0.13142291	0.24048428	0	0.15465542	0.09128535	0	0.16790361
13.3788521	17.194626	32.7553862	12.4033648	11.2737407	29.7018872	17.915876
17.5581005	20.2407602	18.0817305	16.6718544	17.3898591	18.1231854	16.5729675
11.8280617	10.901954	8.52018715	12.7436067	11.0455273	10.3704894	12.7818849
3.36442645	3.60726419	3.45540923	3.43335035	3.05805921	2.89467545	5.94318171
21.5270724	22.7658451	23.477849	22.3322428	21.7715558	19.4320821	13.4390154
10.2772714	8.89791834	10.271559	10.8568106	10.2696018	10.6473714	15.0383191
6.09802294	5.69146128	5.58545602	6.95949395	4.1534834	4.45528308	1.51741694
12.064623	11.6634876	12.306937	9.68142936	10.6347432	12.9127696	24.5974594
11.4075084	9.0582412	8.28351529	10.7330862	10.7716712	9.96775197	10.8935496
15.1662036	15.8318817	18.649743	14.7231961	18.1201419	13.1393094	20.1549088
16.5592864	19.0383388	16.9457056	13.6406081	18.4396406	16.7387754	15.6785846
67.9982127	53.7883172	51.7838041	60.3774764	62.6673924	62.2229365	41.9048929
0.07885374	0.04008071	0	0.03093108	0.09128535	0.10068436	0.40615
20.4494045	20.2006795	21.6791429	19.8577561	20.2197049	18.4252385	13.3131748
23.2355702	22.4852801	25.0872177	21.9920009	22.6844093	21.9240201	51.6325719
4.31067139	3.56718348	3.45540923	4.48500721	4.5186248	3.70015035	4.22699867
1.41936741	1.88379352	1.42003119	1.73214072	1.14106687	1.18304127	0.40791525
29.0181781	28.4973871	27.2645989	28.7040462	30.1241653	31.0107839	9.44459492
19.0300371	20.9221323	14.8629931	20.197998	18.4852833	20.3885836	16.1548676
33.5654108	35.6317541	38.2461734	32.5704317	34.6427901	34.081657	30.5506494
9.09446525	9.94001689	9.98755272	8.66070358	9.95010309	12.9631118	18.3734524
16.4804327	19.3990652	18.0343961	16.2697503	19.0329954	22.729495	26.4396992
16.5592864	15.7116396	18.0343961	15.0325069	19.1699234	19.9858461	11.0662285
23.8664001	22.2447959	26.0339052	21.9610698	21.4520571	24.1642472	43.8698961
27.5988107	26.97432	28.9686363	30.095945	26.9748208	31.3883503	12.8958055
0.68339912	0.80161427	1.04135621	0.95886361	0.91285349	1.00684363	0.37870536
8.3059278	9.81977475	11.2655808	8.84629009	10.5891005	12.5352032	8.95989345
0.26284582	0.20040357	0.18933749	0.09279325	0.1825707	0.22653982	0.28980209
55.3290444	63.0068812	57.0852539	55.7687449	55.6384205	54.3695562	57.6027279
219.765387	217.47795	226.542309	239.994283	222.371111	241.869012	570.776565
0	0	0.09466875	0	0	0.02517109	0
0	0	0.09466875	0	0	0.02517109	0
2.33932777	1.68338996	1.37269682	1.42282987	2.00827769	1.38441	5.17948454

2.23418944	3.12629563	3.07673425	2.01052047	3.56012863	1.81231854	1.32223079
9.96185644	11.5833261	12.4962745	13.0529175	10.6347432	12.8875985	15.7342702
72.8608603	69.3797147	78.3383874	70.9868383	67.8250146	69.7994349	91.9335921
29.8330002	33.4273149	33.9387455	29.0442881	30.9000908	31.5393768	26.0394362
59.9025616	61.6040563	64.327413	58.7381289	56.5056313	60.4609602	45.2891975
8.38478155	8.7776762	6.24813724	7.70183997	9.2198203	8.7847107	3.88742839
0	0	0.09466875	0	0	0.02517109	0
2.20790486	1.5230671	2.17738116	2.07238264	2.05392036	2.56745127	5.0531291
0.21027665	0.6012107	0.18933749	0.52582843	0.1825707	0.52859291	0.15660781
1.89248988	2.00403566	1.42003119	2.38169349	2.23649106	1.56060763	0.95584434
0	0	0.09466875	0	0	0.02517109	0
0	0	0.09466875	0	0	0.02517109	0
17.9523693	16.5132539	14.0583088	16.517199	14.4230852	14.2720085	24.3738834
0	0	0.09466875	0	0	0.02517109	0
21.6847799	19.1585809	21.821146	21.0640684	21.4520571	24.3656159	16.1846686
1.18280617	1.12225997	1.04135621	0.9897947	0.86721082	1.03201472	1.02291225
18.5043455	12.3849404	14.5789869	18.0637532	16.4770056	14.976799	21.713279
1.99762821	2.04411638	1.65670306	1.82493397	0.86721082	2.11437163	2.24705211
0.34169956	0.24048428	0.09466875	0.24744867	0.22821337	0.07551327	0.23529189
5.83517713	6.85380197	6.86348409	6.65018311	5.84226237	5.33627126	9.63304488
4.04782557	3.88782919	3.36074049	2.59821108	3.788342	3.77566363	4.59762806
50.072128	53.4676715	49.3224167	54.6552258	55.8209912	51.3238542	12.786243
1.41936741	1.72347067	1.37269682	2.07238264	1.50620827	1.56060763	0.4204095
341.331577	329.784109	393.490643	321.343034	380.568622	289.845111	226.803852
8.35849696	9.81977475	10.2242246	8.815359	8.07875343	9.13710597	34.1396536
7.70138242	8.53719192	8.37818403	8.66070358	8.35260948	9.48950124	15.8202984
2.10276653	2.00403566	2.60339052	2.10331373	1.91699234	1.88783181	8.6029543
5.80889254	3.36677991	3.21873737	4.54686938	4.24476875	5.31110017	8.40938782
1.2879445	1.48298639	1.2306937	1.23724337	1.00413884	1.20821236	0.7915215
3.18043438	1.84371281	3.50274361	3.5261436	2.64727513	3.47361054	11.2444467
6.57114541	8.09630408	8.37818403	6.61925202	8.3069668	6.16691725	5.69896166
0.2891304	0.12024214	0.23667187	0.30931084	0.13692802	0.37756636	0.37054894
94.5193556	98.6386353	94.4320743	89.5145578	92.4264163	90.4145583	51.7797295
17.2689701	15.791801	15.47834	16.146026	15.1077253	14.1964952	10.5282217
0.78853745	0.36072642	0.66268122	0.80420819	0.41078407	0.25171091	0.75507339
5.41462382	6.85380197	4.87544043	4.23755854	4.60991015	6.46897034	7.87121757
10.5664018	10.8217926	11.9755964	9.8051537	13.5558744	12.8120852	10.2098924
0.7096837	0.56112999	0.56801248	0.95886361	0.41078407	0.80547491	0.70181538
13.0371525	13.1464739	13.4429619	15.1871624	9.40239099	11.6542151	16.9211211
0.05256916	0.08016143	0.28400624	0.03093108	0.09128535	0.12585545	0.09626809
3.89011808	5.53113843	3.97608734	3.83545444	4.19912608	4.02737453	7.0447434
2.73359649	1.88379352	2.03537804	3.15497059	2.64727513	1.9130029	6.03604393
0.7096837	0.20040357	0.33134061	0.12372434	0.41078407	0.45307964	0.44157967
131.475477	131.384578	126.004101	120.97147	131.177047	114.780174	21.1162628
74.9110577	76.7545659	78.9537343	75.2243968	69.2399376	74.3302312	70.328161

25.5748979	28.8581135	27.6906082	31.4569127	31.1283042	25.0200643	21.7864875
1.81363613	1.88379352	2.08271241	1.82493397	2.14520571	2.51710908	4.83353895
21.6059261	23.487298	26.5545833	23.3220375	23.5059775	29.6012028	55.4673898
46.8128399	49.2591966	43.9736326	48.8711131	53.9952842	45.5345033	9.57376206
9.46244939	9.61937118	10.6975683	8.2276684	10.1326738	9.16227706	21.1035096
0.02628458	0.04008071	0	0	0.13692802	0.02517109	0.01320745
7.49110577	5.49105772	7.00548721	5.78411275	12.3691649	4.98387599	3.19963665
77.9863537	72.8667367	73.2262751	90.3806281	70.7461458	76.2432341	28.9836118
6.57114541	7.21452839	8.33084966	4.79431806	6.93768656	8.15543343	5.11185082
12.6954529	14.2687339	18.0817305	10.7021551	15.6097948	14.8257725	20.5018622
38.7434733	33.6677991	31.4773581	37.3338187	35.920785	37.2028723	29.9395581
12.9057296	13.0663125	13.7743026	11.9703296	14.3317999	16.5625778	10.9940035
0.07885374	0.04008071	0.18933749	0.06186217	0.09128535	0.05034218	4.01919271
30.9369526	25.6516565	22.9571709	33.6220886	26.0163246	26.0269079	17.6062892
16.0073102	16.352931	17.3243805	17.1667517	14.6969413	14.423035	14.6294411
15.139919	13.6675232	13.1116213	15.1871624	15.0620827	14.8006014	8.81086585
1.23537534	1.72347067	1.18335933	0.86607036	1.55185094	1.18304127	4.2095166
1.2879445	0.72145284	1.04135621	0.83513927	1.00413884	0.830646	0.9173354
0.55197621	0.44088785	0.18933749	0.61862168	0.77592547	0.22653982	0.41109551
1.36679825	1.44290568	1.04135621	1.39189879	1.36928024	1.661292	1.56114301
0.2891304	0.44088785	0.23667187	0.43303518	0.50206942	0.30205309	0.42561848
0.02628458	0.04008071	0	0.06186217	0.09128535	0	0.05937866
25.259483	25.0905265	25.0872177	24.5592809	24.6470444	21.9995334	13.1236159
0.02628458	0.04008071	0	0.06186217	0.09128535	0	0.05381346
83.6638234	96.1937118	92.4440306	84.4109289	90.9202081	82.7373756	102.140442
0.44683789	0.44088785	0.47334373	0.37117301	0.7302828	0.57893509	0.74368736
41.4245007	32.9463463	36.8734766	43.9221396	34.0494353	40.7519961	105.351313
23.4195622	24.2087508	19.3124242	22.4250361	21.2694864	18.9538314	23.0295865
5.80889254	7.57525481	5.11211229	5.01083564	6.98332923	8.60851307	7.04953642
32.8031579	36.4333684	37.1101485	32.6013628	36.1489984	31.5142057	6.37553543
27.6776645	28.1366607	28.9213019	24.7757985	27.8876743	27.3358046	19.5822322
12.4063225	14.1885725	15.6676775	11.0733282	15.2446534	12.6862298	10.0506435
0.21027665	0.04008071	0.14200312	0.24744867	0	0.30205309	0.17570241
0.02628458	0.04008071	0	0.03093108	0.09128535	0.02517109	0.02756412
226.467955	229.983133	200.177064	253.944202	231.819145	213.324995	64.7277839
7.62252868	8.49711121	7.76283718	7.0522872	9.26546297	9.69086997	6.03920148
122.065597	126.695135	126.004101	117.043223	118.853525	118.404811	80.2369949
0.07885374	0.08016143	0.09466875	0.09279325	0.22821337	0.07551327	0.30573891
0	0.04008071	0.04733437	0	0	0.02517109	0
12.3011842	10.2606626	10.2242246	11.382639	11.5475967	12.4848611	20.8130809
0	0.04008071	0.04733437	0	0	0.02517109	0
69.1021652	82.7666729	69.0135159	70.8321829	79.7833954	55.6029397	37.3455538
0	0.04008071	0.04733437	0	0	0.02517109	0
19.0826063	21.523343	21.0164616	20.1361358	24.6014017	21.1688874	30.6545913
0	0.04008071	0.04733437	0	0	0.02517109	0

0	0.04008071	0.04733437	0	0	0.02517109	0
634.325809	504.255454	568.91183	641.170445	542.919616	652.384332	1299.76788
21.1065191	24.6496387	21.442471	25.3016269	16.1118642	20.4892679	13.1095679
11.9069155	16.7938189	13.1589557	15.8676462	13.2820183	16.0339849	20.6603608
0.07885374	0.08016143	0	0.03093108	0	0.22653982	0.04737752
0.52569163	0.40080713	0.61534685	0.49489735	0.31949872	0.42790854	1.15973099
0	0.04008071	0.09466875	0.03093108	0.04564267	0.10068436	0
0	0.04008071	0.04733437	0	0	0.02517109	0
9.85671812	8.25662693	8.23618091	10.1453956	8.94596425	9.51467234	18.0637875
54.9084911	55.1109807	89.5092995	66.9348663	75.1734853	35.8688044	152.137596
270.310638	280.564993	251.061515	246.025844	265.640367	243.253422	142.528889
1.07766785	0.80161427	1.13602495	0.64955277	1.27799489	0.70479054	0.86901464
15.5604723	16.7136574	17.7030555	19.5175141	15.01644	18.9286603	19.7970844
0	0.04008071	0.04733437	0	0	0.02517109	0
240.188507	235.955159	258.114336	241.91201	236.292127	212.897086	87.8908357
0.10513833	0	0.14200312	0.03093108	0	0.10068436	0.05439381
0	0.04008071	0.04733437	0	0	0.02517109	0
0.34169956	0.48096856	0.09466875	0.43303518	0.22821337	0.50342182	0.76837508
18.2677842	19.1185002	19.407093	19.1772722	19.8089208	21.4205983	14.0457017
16.6381402	15.9120432	11.0289089	14.7231961	11.4106687	13.8944421	26.0604249
0.07885374	0.08016143	0.18933749	0.06186217	0.41078407	0.07551327	0.10905667
189.958672	189.421451	195.632964	176.86394	189.097601	177.330335	62.9312514
22.9990089	20.6014866	22.0104835	19.8577561	22.3192679	19.5327665	16.851961
3.1541498	3.68742562	2.79272801	2.78379758	3.01241653	2.89467545	7.2390713
0.63082996	0.44088785	0.56801248	0.30931084	0.82156815	0.276882	1.52206114
0.05256916	0	0	0.03093108	0.04564267	0.05034218	0.06769167
0	0.08016143	0.04733437	0.09279325	0	0.07551327	0
163.306106	195.193074	165.575637	155.459629	186.815468	147.326395	57.3063553
2.70731191	3.04613421	3.59741235	2.50541782	2.60163246	3.14638635	2.67513311
34.0385332	34.5896555	34.7907642	35.0139873	37.1074946	37.1525301	85.7648537
44.2106663	46.5737888	43.5002888	47.5410765	45.1406053	33.0999844	15.4300306
0.02628458	0	0.04733437	0.03093108	0.04564267	0.05034218	0.06450059
534.128984	513.674421	493.460839	577.607067	546.525387	521.645686	360.691206
61.5584902	66.9347911	60.9666725	65.5429675	62.3478937	50.2666684	22.8054496
0.05256916	0	0	0.03093108	0.04564267	0.05034218	0.03614788
21.1590882	21.8840694	20.2117773	25.9821108	18.9873527	22.7043239	12.7192143
18.9511834	18.2768052	14.8629931	15.9295084	15.0620827	17.5442503	25.3501406
42.4495994	48.2571788	42.127592	36.715197	51.9870065	53.7402789	22.1441236
0.02628458	0.20040357	0	0.06186217	0.04564267	0.02517109	0.03832365
0.65711454	0.36072642	0.56801248	0.18558651	0.45642675	0.62927727	0.5501975
17.7420926	22.0443923	20.9691273	16.3006814	28.7548851	21.4205983	15.4254731
0.07885374	0	0.04733437	0.06186217	0.04564267	0.10068436	0.05530999
1.76106697	1.60322853	1.37269682	1.45376096	1.18670954	1.51026545	2.05954808
3.8638335	2.76556921	4.07075608	3.74266119	2.69291781	3.07087308	5.90083724
20.2391279	18.0764017	16.0463525	19.3009966	18.5309259	20.4389258	10.3009058

18.5831992	15.8719625	16.0936868	18.9607546	14.8338693	20.6151234	33.4302264
0.05256916	0	0	0.03093108	0.04564267	0.05034218	0.10456436
139.991682	135.43273	132.536245	145.035854	110.363987	127.340549	59.2764843
0.5782608	0.96193712	0.56801248	0.61862168	0.3651414	0.70479054	0.09596119
3.70612601	2.96597278	3.07673425	3.99010987	3.33191526	3.54912381	4.53818756
2.12905111	2.36476208	3.40807486	2.56727999	2.55598978	1.78714745	77.7443844
0.07885374	0.04008071	0	0	0.13692802	0.10068436	2.60704392
1.68221323	1.60322853	1.89337492	2.28890023	1.00413884	2.61779345	1.17556508
140.123105	108.258007	95.7101023	136.06584	88.1360049	91.6479417	59.4143954
16.9535552	18.4371281	19.1230867	19.7340317	20.493561	17.9469878	21.3584288
19.5820133	18.3970474	20.9217929	18.1874775	19.2612087	17.8463034	7.06931933
0.31541498	0.12024214	0.09466875	0.30931084	0.04564267	0.37756636	0.36263393
0.31541498	0.12024214	0.09466875	0.30931084	0.04564267	0.37756636	0.35709625
114.416784	127.336426	148.014585	118.002086	110.40963	136.024575	231.155024
1.26165992	0.80161427	0.89935309	1.08258795	1.18670954	1.23338345	0.74147009
1.52450574	1.5230671	1.84604055	1.88679614	1.50620827	2.06402945	1.69305984
164.962034	159.320835	152.180009	157.253632	156.600017	134.363283	75.1846601
30.2535535	30.8220685	32.613383	32.9416047	31.0370188	35.0633295	39.038741
45.8403104	45.1709638	45.6303356	47.35549	45.6883174	50.8707746	29.8991078
19.4243058	21.0022937	21.3004679	21.2187238	13.190733	22.0498756	3.23527365
12.7480221	14.4290568	13.064287	12.496158	12.5517356	12.5100321	26.980048
2.91758856	2.76556921	3.97608734	3.00031517	2.92113118	4.88319162	1.79619684
6.64999916	4.04815204	8.28351529	6.52645877	10.1326738	5.08456035	8.68179941
0.49940705	0.48096856	0.7100156	0.52582843	0.7302828	0.72996163	1.04430306
0.47312247	0.44088785	0.37867498	0.30931084	0.27385605	0.45307964	0.85742778
0.2891304	0.24048428	0.14200312	0.18558651	0	0.276882	0.12096066
39.2165958	36.9544176	40.7075608	34.6737454	34.3689341	41.003707	52.2867403
6.3082996	6.61331769	7.19482471	6.4645966	6.25304644	5.7641798	3.00617452
14.1673895	15.1905903	12.7802807	16.5790611	14.2861572	14.397864	21.8271524
8.98932692	8.53719192	9.8455496	8.815359	8.39825215	8.13026234	5.76186076
0.13142291	0.12024214	0.42600936	0.09279325	0.27385605	0.50342182	0.25139595
11.2760855	8.7776762	6.86348409	9.86701587	7.98746808	6.67033907	6.65566677
0.65711454	0.76153355	1.18335933	0.86607036	0.50206942	0.72996163	2.0070875
13.8519745	20.4812445	15.6203431	20.3526534	16.5226483	17.1163418	16.5639457
2.47075067	1.84371281	2.22471553	2.59821108	2.41906176	2.36608254	4.26596036
1.5770749	1.8036321	1.7513718	1.39189879	2.19084839	2.26539818	1.73724674
11.3549393	11.3829226	12.8276151	11.0423971	14.1035865	7.19893198	4.60707463
12.6691684	11.2225997	11.170912	12.1559161	9.99574577	10.4208316	13.8933277
0.52569163	0.32064571	0.37867498	0.27837976	0.50206942	0.276882	1.67031385
0	0	0.09466875	0.03093108	0	0	0
263.765777	321.607643	338.630105	263.780286	308.772695	291.506403	682.87998
0	0	0.09466875	0.03093108	0	0	0
0	0	0.09466875	0.03093108	0	0	0
5.30948549	26.7739165	21.5371397	21.4661725	3.74269933	16.7387754	3.01253034
31.1735138	30.4212614	32.5660487	30.6836356	32.132443	25.775197	9.95018929

29.5964389	29.0985978	26.3652458	29.3535989	30.5805921	30.205309	7.79521756
0.86739119	0.48096856	0.75734997	0.80420819	0.7302828	0.95650145	0.93982894
0.42055331	0.6012107	0.33134061	0.43303518	0.27385605	0.37756636	0.34925775
53.6205466	59.3595363	62.7180443	54.9026745	65.4515956	66.0237713	147.899042
6.96541414	7.21452839	5.68012477	6.77390745	4.83812352	6.44379925	5.95435957
15.7181798	14.7096218	17.3717149	14.5376096	14.6512986	15.4047076	33.1800137
19.3191675	18.7577738	18.649743	22.6106226	18.667854	19.2307134	9.56021567
243.447795	232.748702	258.161671	247.201225	243.229814	198.952302	110.585259
0.13142291	0	0.14200312	0.06186217	0.13692802	0.20136873	0.34024912
26.38972	22.1646344	20.5431179	24.0643835	25.2860418	24.5669847	26.5806141
58.0363563	56.9546935	64.2327442	60.1609588	56.414346	52.7586064	40.0762179
1.89248988	2.1242778	2.03537804	1.63934746	2.19084839	3.0205309	1.6110334
17.9523693	15.9922046	15.0523306	13.9808501	13.327661	18.0728432	9.89451181
0.2891304	0.20040357	0.5206781	0.5876906	0.5477121	0.17619764	1.8045355
0.10513833	0	0.09466875	0	0	0.10068436	0.11964109
2.75988107	2.7254885	4.2127592	3.68079902	3.01241653	1.71163418	2.08145615
4.8626476	3.60726419	3.97608734	3.68079902	3.56012863	4.05254562	5.33728597
5.62490047	6.6533984	8.70952464	5.78411275	7.25718528	5.96554853	6.10817392
0	0.08016143	0	0	0	0.02517109	0
0	0.08016143	0	0	0	0.02517109	0
0	0.08016143	0	0	0	0.02517109	0
8.09565115	8.65743406	7.99950905	7.4543913	7.89618273	7.60166943	13.860449
34.616794	30.5815842	31.9507018	36.5605416	33.7755793	32.6720759	22.8466223
15.770749	13.8278461	19.5490961	14.9087826	17.9375712	20.2627281	7.15450584
158.653735	161.525274	161.268209	172.131484	160.342716	178.286836	236.537206
0	0.08016143	0	0	0	0.02517109	0
534.207837	560.488694	510.453879	618.992858	551.591724	544.677235	452.711175
25.2857675	22.1245537	21.7738116	21.7754833	20.0827769	23.1574036	44.8936242
34.6956478	32.9062656	30.0099925	35.0139873	35.8751423	32.7224181	36.5569983
101.668762	101.925254	108.869058	105.351273	95.8952596	94.1147086	91.3477042
17.0061243	16.4731731	18.1763993	16.3006814	16.5682909	15.5557341	26.2834384
21.8687719	23.3670558	19.6437648	21.8682765	23.1864788	23.9880496	19.4416764
216.74266	222.688443	212.768007	215.063829	219.130481	179.771931	86.0139837
0.49940705	0.40080713	0.42600936	0.27837976	0.5477121	0.80547491	2.20150375
3.33814187	3.60726419	4.40209669	3.9282477	2.00827769	3.90151908	2.06393722
50.9395192	40.3211975	43.0742795	58.1195073	36.5141398	50.0401286	111.174958
30.2272689	38.8382111	35.5481142	38.6019931	33.4104379	24.9193799	36.2871177
39.1377421	36.7139333	43.6896263	39.3752702	46.6924562	40.7016539	33.4684541
66.6576991	61.6842177	63.570063	57.0059882	68.9204388	55.2757155	31.9394366
24.2869534	25.6917372	26.0339052	25.9511797	33.1365819	22.5784685	22.7661858
0.44683789	0.76153355	0.23667187	0.68048385	0.50206942	0.57893509	0.93357849
67.7090823	77.716503	76.2083406	72.6571168	66.9121612	70.0511458	65.1415835
72.2300304	68.8185847	68.3981691	68.6360759	78.1402591	74.4560867	145.325547
94.2039406	93.7487883	92.9647087	99.165056	91.1940641	106.473714	118.986364
95.0976164	94.9512097	67.8301566	63.7180335	83.5260948	92.2017057	93.8535811

6.83399123	8.53719192	8.47285278	6.83576961	9.12853495	10.0180942	7.48761383
8.25335864	8.57727264	8.6148559	8.35139274	8.07875343	7.1485898	6.70399209
0.60454538	0.52104927	0.28400624	0.43303518	0.27385605	0.45307964	0.4423035
0.15770749	0.36072642	0.09466875	0.21651759	0.1825707	0.37756636	0.34604709
9.96185644	10.7015504	10.4135621	10.5165686	11.0455273	11.7800705	15.1381271
189.064996	177.517479	250.635505	212.125376	169.288681	192.886069	373.635582
0.36798414	0.28056499	0.14200312	0.09279325	0.22821337	0.30205309	0.24719911
2.73359649	1.36274425	2.88739676	1.67027855	2.41906176	2.01368727	5.79042136
1.62964406	1.72347067	1.37269682	1.39189879	1.27799489	1.48509436	2.15399199
26.0217358	29.77997	26.6965864	28.642184	25.1034711	23.8873652	20.3659669
8.48991987	8.29670764	8.66219027	8.38232383	7.9418254	7.27444525	9.33536536
0.97252952	1.2024214	0.89935309	1.26817445	1.09542419	1.15787018	0.54685408
0.05256916	0.20040357	0.09466875	0.12372434	0.04564267	0.05034218	0.1676847
0.07885374	0.08016143	0.09466875	0	0.13692802	0.05034218	0.05879953
0.21027665	0.28056499	0.14200312	0.15465542	0.31949872	0.35239527	6.1982731
29.0444627	31.1427142	27.5012707	28.642184	37.472636	27.9399108	9.30461269
0	0.08016143	0.14200312	0.06186217	0.22821337	0.05034218	0
52.4114558	49.4996809	44.0209669	55.3975719	53.6301428	46.1889517	40.1340608
16.4541481	14.8298639	14.4843182	15.9295084	14.6056559	12.1324658	8.95976723
0.10513833	0	0.14200312	0.09279325	0	0.05034218	0.07088133
6.28201501	8.09630408	8.94619651	9.89794695	7.85054005	7.60166943	3.95100075
4.88893219	5.69146128	5.4434529	4.39221396	5.93354771	4.30425653	8.0007475
0.73596829	0.96193712	0.37867498	0.5876906	0.45642675	0.67961945	0.75878564
2.62845816	3.52710277	3.12406862	2.81472866	3.46884328	1.98851618	2.56203519
6.22944585	5.731542	6.86348409	7.91835756	7.43975598	5.36144235	4.18357913
266.026251	241.526378	251.724196	225.425742	264.681871	224.400275	276.958294
0	0.04008071	0.04733437	0.03093108	0	0	0
0.15770749	0.08016143	0.14200312	0.09279325	0.3651414	0.12585545	0.5314635
0	0.04008071	0.04733437	0.03093108	0	0	0
0.02628458	0.04008071	0	0	0.09128535	0.05034218	0.03602638
0	0.04008071	0.04733437	0.03093108	0	0	0
17.8998001	12.2246175	13.1589557	18.3112019	13.6928024	15.1781678	22.0620271
1.05138327	0.64129141	1.18335933	0.5876906	0.68464012	1.107528	0.29323795
480.613575	503.694324	496.253567	543.521012	468.339485	360.953443	122.166755
0.52569163	0.32064571	0.94668746	0.49489735	0.59335477	1.03201472	0.96629727
0.10513833	0.04008071	0.18933749	0.06186217	0.09128535	0.07551327	0.10211526
1466.04883	1387.07324	1802.54026	1404.98264	1332.94867	1564.2071	2925.60512
33.8545412	30.6216649	36.3054641	36.3749551	29.5764532	28.5943592	20.4714591
30.1221306	29.6998085	33.4180674	30.095945	29.8046666	28.0154241	23.3658584
6.75513748	5.85178414	4.82810605	6.43366552	6.43561714	6.3179438	10.9740507
0.34169956	0.08016143	0.23667187	0.09279325	0.31949872	0.12585545	0.34630989
60.0865536	65.9327733	71.048894	63.5015159	62.0740376	60.7378422	41.0975657
0.13142291	0.08016143	0.14200312	0.09279325	0.04564267	0.32722418	0.10226953
561.859217	590.629391	650.563623	500.062839	671.221175	501.987065	376.095271
8.85790402	8.05622337	9.37220587	10.4856376	8.35260948	9.51467234	17.8073025

8.09565115	7.29468981	7.28949345	6.4645966	8.80903622	9.53984343	10.0134387
27.2571112	24.1686701	24.8505459	26.7863189	27.5681755	26.5051586	17.96026
29.5175852	29.7398892	29.2053082	28.1472866	30.443664	25.0704065	20.149792
30.6478222	30.2208578	32.992058	29.2298746	31.4934456	27.8643976	10.1477953
0	0.04008071	0.04733437	0.03093108	0	0	0
63.7926797	61.2433299	59.6886444	64.2438619	59.7919039	50.2414973	21.9512365
54.9610602	59.8004242	56.8012477	53.232396	57.9661969	51.2986831	31.0187108
58.6146171	50.5016987	53.6771791	55.0573299	47.3314537	51.2986831	55.1132959
60.4545378	64.3295448	66.1734535	54.9645367	67.9163	57.4404293	55.666651
46.8128399	47.5758066	48.8017386	43.6128288	48.5181632	43.8732113	49.6449569
24.2869534	27.5755307	32.5187143	20.4454467	30.0785226	27.7385421	14.0671745
62.6887272	65.4518048	67.498816	63.5633781	67.5055159	53.6395946	22.1508368
167.038516	178.880223	175.373852	171.481931	174.172447	144.305864	128.95914
26.1005896	18.8780159	26.6965864	25.7655932	21.9997692	29.2488075	99.9326037
23.498416	24.4492351	25.5605615	23.878797	23.6429055	21.7981647	12.6631129
5.83517713	6.13234913	7.47883094	6.00063034	4.47298212	6.97239216	13.4553935
17.4529622	16.633496	17.0403743	18.5586505	16.0205788	19.9355039	40.9395047
0.05256916	0.08016143	0.18933749	0	0.27385605	0.20136873	0.05385853
19.0037525	23.0063294	21.6318085	21.5589657	23.7798335	22.4022708	21.5549473
12.248615	14.7497025	13.2536245	13.4859527	12.5060929	11.4780174	4.60041462
199.762821	200.123001	191.562208	161.769571	192.429517	202.878992	420.959374
0.02628458	0.08016143	0.04733437	0.03093108	0.09128535	0.10068436	0.02179901
0.21027665	0.12024214	0.09466875	0.03093108	0.1825707	0.12585545	0.66899437
15.5867569	15.0703482	12.0229308	13.0529175	17.252931	15.9836427	13.9532018
0.13142291	0.12024214	0.04733437	0.06186217	0.09128535	0.07551327	0.08512984
257.904315	232.949105	249.594149	244.664876	237.752693	215.665906	242.498502
0.10513833	0.20040357	0.37867498	0.21651759	0.09128535	0.15102655	0.0381192
135.339311	130.222237	123.968723	134.704872	114.928255	117.951732	25.7959779
14.4565199	14.6294603	13.8689713	15.1562313	13.1450903	11.7297283	6.11601173
182.83555	163.008261	192.319558	182.988294	197.358926	189.815196	226.950823
2.83873482	2.08419709	3.59741235	3.18590168	3.37755793	2.97018872	5.81672111
2.73359649	2.52508494	2.31938428	2.28890023	3.05805921	3.17155745	3.58794886
5.28320091	4.68944345	6.34280599	4.88711131	6.84640121	6.19208835	2.50595653
0.23656123	0.40080713	0.23667187	0.52582843	0.27385605	0.276882	1.33355703
181.205906	180.042564	255.463611	215.527795	197.860995	249.999274	1214.61105
0	0.08016143	0	0.03093108	0	0	0
0.02628458	0	0.04733437	0	0	0.02517109	0.04162271
0	0	0.09466875	0	0.04564267	0	0
0.02628458	0.04008071	0	0.09279325	0.04564267	0	0.0474241
0.13142291	0.24048428	0.18933749	0.18558651	0.22821337	0.276882	0.83733181
1.91877446	1.84371281	2.13004679	1.67027855	2.46470444	2.51710908	2.57228333
0.05256916	0	0	0.06186217	0	0.05034218	0.15703805
249.361826	306.497214	311.933518	246.366086	269.474352	296.817503	889.331341
74.516789	67.0550333	76.7763531	72.9354966	68.053228	65.8727447	49.113842
3.73241059	5.450977	4.87544043	4.85618022	4.65555282	5.86486416	6.03144375

0.02628458	0	0.04733437	0.06186217	0	0.05034218	0.01350705
405.229395	385.937188	386.674494	353.913466	428.858572	319.446314	296.566648
8.72648111	7.05420553	6.39014036	5.2892154	8.3069668	6.79619453	5.83650948
20.4756891	19.6395495	19.0757523	20.2907913	17.252931	18.0728432	31.289955
206.491673	215.073107	217.264772	239.684972	218.0807	218.2837	414.775408
0.02628458	0	0.04733437	0	0	0.02517109	0.08993998
31.0158063	29.0985978	24.4245365	28.3328732	24.1449749	27.1092648	11.9424549
2.18162028	2.1242778	1.89337492	2.07238264	1.82570699	1.661292	5.09302282
0.02628458	0	0.04733437	0	0	0.02517109	0.06089686
7.28082912	6.89388268	6.20080287	7.94928865	7.43975598	6.77102343	8.43022342
0.13142291	0.16032285	0.18933749	0.09279325	0.13692802	0.37756636	0.51528112
0.10513833	0.16032285	0.04733437	0.12372434	0	0.07551327	0.10867272
0	0.04008071	0.04733437	0.06186217	0.04564267	0.02517109	0
102.877853	97.4362139	89.5566338	92.174631	83.2522387	98.6203339	114.20051
56.9061193	52.145008	62.6233756	62.6663766	56.0035619	62.8522138	127.145968
41.7924848	45.9725781	47.0030325	44.6026235	46.9206696	35.3905537	33.9356995
12.56403	17.0343031	15.7150119	15.774853	19.9458489	13.4665336	6.39458828
14.5353737	16.1926082	12.5436089	14.0117812	15.0620827	11.1507932	4.44189572
232.776255	305.054309	254.706261	299.938724	213.744646	222.361416	130.470454
99.5134261	89.179587	95.0000867	93.999565	84.895375	90.7166114	142.211809
24.2343843	20.0403566	20.5904523	20.197998	22.456196	18.3245541	23.3797835
0.02628458	0	0.04733437	0	0	0.02517109	0.05059566
0.02628458	0	0.04733437	0	0	0.02517109	0.03895038
0	0	0.09466875	0	0.04564267	0	0
98.9877345	93.7487883	104.845636	99.8146088	92.4264163	89.3070303	46.6629048
13.9833974	14.0683304	12.0702651	12.0940539	10.0413884	14.8509436	9.04105929
16.4541481	15.7517203	17.5610524	14.1045744	17.0703603	15.4550498	5.1544474
61.5847748	61.0028456	57.605932	61.9858928	55.5471351	53.1361728	42.9385854
6.15059211	5.49105772	5.11211229	5.22735323	5.43147829	4.80767835	2.7454584
177.079227	196.195091	181.148646	179.245633	181.56656	161.170495	150.325059
0.7096837	0.44088785	0.61534685	0.49489735	0.22821337	1.45992327	0.59892765
2.1553357	1.8036321	3.07673425	2.87659083	2.55598978	2.44159581	1.91195824
0	0	0.09466875	0	0.04564267	0	0
0.02628458	0.04008071	0	0.09279325	0.04564267	0	0.03084273
0.05256916	0.04008071	0	0.18558651	0	0	0.0422101
0.13142291	0.16032285	0.18933749	0.09279325	0.09128535	0.15102655	0.13589832
0	0.08016143	0	0.03093108	0	0	0
34.432802	30.2208578	32.2347081	31.4878437	29.8503093	30.3563355	54.596422
0.02628458	0	0.04733437	0	0	0.02517109	0.05894325
0.02628458	0.04008071	0.09466875	0.03093108	0.09128535	0.10068436	0.07747841
3.02272689	2.4048428	3.21873737	2.938453	2.05392036	2.66813563	2.4350723
37.7709438	42.4454753	39.2875296	37.5812673	42.4476875	32.7727603	18.9830071
71.7043387	88.7787799	69.1081847	102.103509	82.4306706	63.0032404	38.24498
6.28201501	4.97000844	5.20678104	5.81504383	4.97505155	7.24927416	20.9392274
0	0.04008071	0.04733437	0.03093108	0.09128535	0.02517109	0

0.34169956	0.32064571	0.33134061	0.34024193	0.45642675	0.42790854	0.45682628
0.02628458	0	0.04733437	0	0	0.02517109	0.03289365
18.5569146	22.2447959	19.3124242	21.3733792	17.5724298	17.1415129	16.7592473
48.9681756	53.5879136	42.3642639	47.0771102	45.3688187	44.7290284	16.9814453
0.44683789	0.80161427	0.33134061	0.37117301	0.5477121	0.40273745	0.20218613
3.49584936	3.08621492	5.06477792	3.9282477	3.9709127	4.9335338	3.22505358
1.05138327	1.48298639	1.79870618	1.45376096	0.82156815	1.33406781	0.36501821
16.6907093	15.7116396	18.1763993	15.7439219	15.290296	16.2856958	12.9102112
0.05256916	0	0	0.06186217	0	0.05034218	0.06663946
13.694267	14.9100253	15.2890025	13.3622284	12.6430209	14.8509436	12.3131167
15.7181798	16.1124467	13.1589557	14.8159893	13.1450903	14.0706398	12.5589928
15.2713419	13.9080075	16.0463525	14.1664366	14.0123011	14.146153	38.4782857
35.4579006	31.2629563	36.2581298	36.5605416	30.7175201	29.6767161	27.9388906
6.25573043	4.80968559	4.07075608	4.54686938	3.51448595	5.51246889	13.6652552
31.7780592	34.7900591	41.9855889	33.3127777	33.1365819	35.4157248	41.1578951
0.13142291	0.04008071	0.04733437	0.03093108	0	0.10068436	0.02732286
0.07885374	0	0.14200312	0.12372434	0.04564267	0.12585545	0.09641242
63.5035493	60.2813927	58.741957	66.2853135	62.7130351	63.4814911	37.728488
18.5306301	14.6695411	8.99353088	15.1253002	16.1575069	15.4802209	5.41525248
42.8964372	43.8483003	42.6482701	43.0560692	48.0617365	45.2072791	70.6252759
0.02628458	0.04008071	0.09466875	0.03093108	0.09128535	0.10068436	0.05529414
2.49703526	1.84371281	2.50872177	2.62914216	2.46470444	2.56745127	2.838835
0.02628458	0.04008071	0.18933749	0.09279325	0.04564267	0.17619764	0.0337216
105.427457	114.390356	110.289089	110.516764	123.737291	114.578805	27.7947965
5.23063175	5.37081558	6.76881535	5.04176673	4.01655538	6.44379925	6.95873278
0.15770749	0.44088785	0.04733437	0.18558651	0.1825707	0.47825073	0.37827696
0	0.04008071	0.04733437	0.06186217	0.04564267	0.02517109	0
109.081014	108.618733	106.407671	102.567475	127.662561	113.345422	72.2845059
0	0.08016143	0	0.03093108	0	0	0
7.93794366	6.13234913	6.1534685	6.06249251	6.52690249	6.14174616	3.55300753
199.289698	192.146939	179.586611	227.09602	178.599786	201.016331	216.124249
12.7743067	16.5934153	11.3602495	16.7646477	14.6969413	12.7617431	12.4168623
262.609255	234.31185	280.787501	265.759876	251.993207	311.190196	933.854753
36.1675843	34.669817	35.5954485	32.0136722	34.5515048	34.358539	44.8033148
0	0	0.09466875	0	0.04564267	0	0
0	0.08016143	0	0.03093108	0	0	0
0.34169956	0.40080713	0.56801248	0.37117301	0.22821337	0.40273745	0.40153318
33.4339879	28.2569028	28.0219489	32.8797425	26.5640367	36.5232528	23.7510482
102.378446	109.420347	95.7574367	93.8449095	107.534142	89.1560037	52.0523877
21.5007878	25.0905265	24.235199	23.5694862	21.9541265	21.0933741	14.0200411
0.39426872	1.24250211	0.61534685	1.29910554	0.82156815	0.60410618	0.74372109
41.9764769	42.0446682	40.9442327	41.6023083	42.2194741	34.5599077	23.1615724
2.47075067	2.60524636	1.79870618	2.66007324	2.00827769	1.40958109	0.62017706
126.928245	138.959833	148.913938	138.478464	139.849155	161.97597	515.845358
1.8662053	1.08217926	1.08869058	1.29910554	0.77592547	1.38441	1.76695159

5.94031545	6.73355983	6.39014036	5.62945733	4.47298212	7.07307652	5.12642005
0.18399207	0.04008071	0.14200312	0.06186217	0.1825707	0.276882	0.23335904
123.45868	133.548937	110.099752	118.930019	122.550582	104.686567	69.2617222
12.9057296	13.9480882	17.4663837	14.6613339	14.5143706	18.4252385	11.2365758
46.1294408	48.5778245	51.7364698	59.4495439	53.7670708	42.8663677	14.8147718
14.4302353	13.6675232	16.6616993	16.2078881	19.078638	13.1393094	13.2873876
0.07885374	0.12024214	0.09466875	0.06186217	0.13692802	0.02517109	0.30818091
11.6966388	13.2667161	15.3836712	12.5580202	12.5973782	12.2834923	20.6618427
0.2891304	0.24048428	0.04733437	0.30931084	0.13692802	0.32722418	10.4026164
0.10513833	0	0.14200312	0.12372434	0.13692802	0.10068436	4.51277778
707.607223	751.553454	781.916509	700.805575	746.257732	649.263117	452.72254
30.3586918	29.9402928	34.9801017	29.6319787	28.8461704	31.2121526	44.0015884
0.10513833	0.12024214	0.23667187	0.12372434	0.09128535	0.10068436	0.08436482
83.7163925	88.2978113	79.048403	86.6998291	75.492984	76.0670365	53.8286624
9.01561151	8.7776762	8.56752152	9.49584286	11.8670954	7.57649834	12.3019128
0.05256916	0.12024214	0.09466875	0.09279325	0.13692802	0.12585545	0.07934248
0.13142291	0.12024214	0.18933749	0.12372434	0.1825707	0.25171091	0.0894645
20.6859658	18.2367245	19.9277711	21.7754833	18.6222113	22.2009021	13.9305463
174.608476	167.256816	181.101311	174.265729	168.740968	149.365253	85.0302458
10.9081014	9.49912904	8.99353088	10.9496038	7.30282796	8.70919743	7.64902253
2.91758856	3.84774847	4.26009358	4.14476529	2.28213374	3.19672854	1.14723341
0	0.08016143	0	0.03093108	0	0	0
7.54367493	6.97404411	6.62681223	6.8667007	5.43147829	7.09824762	13.9819189
0.02628458	0.24048428	0	0.09279325	0	0.05034218	0.02886314
0.05256916	0.12024214	0.09466875	0.18558651	0	0.15102655	0.052646
51.6492029	55.8725143	46.3403512	50.1702186	49.2028034	46.0882673	28.3962739
43.3432751	38.5175654	37.5834922	46.0254533	40.0742684	40.2989164	52.1092779
7.96422824	7.8558198	8.94619651	8.93908334	8.71775087	8.93573725	6.99893903
23.5772697	21.1626166	23.9038584	19.084479	23.0951934	22.4022708	15.2925381
25.0492063	24.3289929	28.2586207	22.6724847	29.3938825	30.7087308	11.7156663
60.9539448	64.4497869	58.8839601	56.7894706	63.3063899	54.6212671	26.2364754
1.5770749	1.8036321	1.79870618	1.85586505	1.55185094	2.34091145	0.35314159
0.13142291	0.12024214	0.33134061	0.15465542	0.09128535	0.15102655	0.22328848
0	0.04008071	0.04733437	0	0.04564267	0	0
0.26284582	0.16032285	0.47334373	0.15465542	0.31949872	0.22653982	0.63860063
0.07885374	0.04008071	0	0.03093108	0.04564267	0.12585545	0.10467784
5.151778	5.69146128	5.91679663	5.72225058	7.66796935	5.08456035	5.950986
0.31541498	0.36072642	0.18933749	0.46396626	0.3651414	0.276882	0.22763569
23.9452539	23.5674594	26.3652458	26.7863189	23.5516202	27.6630288	22.5322127
0.15770749	0.12024214	0.09466875	0.15465542	0.1825707	0.17619764	0.13364825
2.07648195	1.68338996	2.22471553	3.18590168	1.50620827	2.08920054	3.49639338
0	0.04008071	0.04733437	0	0.04564267	0	0
0.02628458	0.04008071	0	0	0	0.02517109	0.03498753
19.5557287	20.5213252	22.4838272	17.8781667	21.2694864	19.6082798	8.32512094
33.486557	36.1127226	31.9507018	39.4371324	36.2402837	31.9924565	14.4263469

0.02628458	0.04008071	0	0	0	0.02517109	0.0500834
0.23656123	0.08016143	0.04733437	0.09279325	0.1825707	0.02517109	0.12285369
0.36798414	0.72145284	0.89935309	0.43303518	0.82156815	0.37756636	1.14980737
139.124291	134.550954	134.808294	135.385356	134.508962	116.29044	37.8786326
5.91403087	5.05016987	4.73343731	8.0420819	4.7011955	4.75733617	7.0306578
50.7292426	53.4275908	45.2043263	51.4383931	45.0949626	44.5780019	41.5648596
58.9563166	65.3315626	58.1739445	60.2846832	59.6549759	52.9599751	20.3701155
3.65355685	2.92589207	4.82810605	2.19610698	5.88790504	5.10973144	5.69342967
0.60454538	0.6012107	0.33134061	0.52582843	0.41078407	0.37756636	0.97370831
0.36798414	0.12024214	0.28400624	0.27837976	0.45642675	0.30205309	0.72780854
43.3958443	44.2090267	44.7783169	40.395996	40.9414792	44.4269753	52.1724789
6.78142207	6.41291412	7.43149657	6.12435468	6.48125981	6.44379925	6.36410721
72.2825995	79.0792473	76.9183562	70.9249761	77.2274056	68.5660514	21.6800882
375.922087	395.877205	271.320626	408.970796	286.179571	263.239268	201.207593
0.02628458	0.04008071	0	0	0	0.02517109	0.22484994
84.7940604	84.570305	92.8700399	86.3595872	89.5052851	99.9544017	79.7289204
1.31422908	1.2024214	1.08869058	1.23724337	1.82570699	1.18304127	0.23462888
0	0.08016143	0	0.03093108	0.09128535	0.02517109	0
23.0778627	23.0464101	23.0991741	23.1983132	23.5059775	26.5303297	9.63837815
126.008284	134.31047	129.743517	131.982936	153.359387	128.07051	46.2617283
31.0420909	6.53315626	8.47285278	23.1055199	6.89204388	6.69551016	23.035082
24.2869534	21.8439887	20.8744585	26.3842148	22.1366972	22.7798372	23.23807
0.47312247	0.48096856	0.37867498	0.40210409	0.68464012	0.553764	0.48331002
33.0397191	36.7139333	35.2167736	33.5602264	34.3689341	31.5142057	43.4357892
0.31541498	0.6012107	0.56801248	0.64955277	0.68464012	0.42790854	0.21991593
119.095439	115.713019	114.643852	116.331808	110.181417	104.736909	38.9175096
0.02628458	0.28056499	0.23667187	0.12372434	0.13692802	0.10068436	0.04965798
0.02628458	0.04008071	0	0	0	0.02517109	0.05746835
0.34169956	0.20040357	0.28400624	0.24744867	0.22821337	0.20136873	0.50360969
69.3912955	70.3416518	78.4803905	67.4606947	65.1777395	74.0030071	40.7026373
0.02628458	0	0.04733437	0.09279325	0.04564267	0	0.01358409
22.0001948	23.1265716	20.685121	20.3835845	22.3192679	19.3817399	38.391604
6.41343792	6.93396339	6.57947786	6.99042504	6.29868911	5.05938926	6.42018345
76.0938639	83.8889329	81.9357998	69.3784219	76.1776241	83.8952458	121.361803
27.7828028	29.3791628	32.7080518	28.1782177	27.6594609	29.0222677	66.9021527
0	0.08016143	0.04733437	0.06186217	0	0.10068436	0
0.97252952	0.84169498	1.04135621	1.08258795	1.50620827	0.80547491	2.06541848
15.5341878	17.194626	17.9870618	14.4138852	17.0703603	15.9836427	22.2644911
308.002728	308.180604	289.54436	312.342089	296.220959	242.070381	168.61723
11.5915005	10.2205819	9.60887773	11.5682255	10.9085993	11.3269909	10.1283656
83.4009776	75.7525481	62.5760412	81.4106137	80.1028942	73.4995852	56.8691125
0.31541498	0.16032285	0.18933749	0.40210409	0.1825707	0.276882	0.44917637
0.84110661	0.76153355	1.04135621	0.83513927	0.45642675	1.81231854	0.99700904
0.36798414	0.40080713	0.33134061	0.34024193	0.04564267	1.00684363	0.29221005
1.02509868	0.84169498	1.08869058	0.83513927	0.91285349	0.830646	0.56270501

19.7134362	17.5553524	16.3776931	17.9709599	16.2944349	16.1346692	16.0020944
74.4642198	81.9249779	91.9706869	87.411244	77.5469044	69.4218685	49.6543928
66.2108612	73.0270596	70.7175534	61.9549617	71.7959274	65.2686385	61.9937019
245.314001	265.093838	235.583175	237.36514	257.470328	207.837697	134.147968
0.18399207	0.36072642	0.04733437	0.21651759	0.09128535	0.12585545	0.30895647
7.83280533	6.73355983	6.6741466	7.42346021	7.34847063	8.20577561	10.6463949
207.753333	229.502164	217.359441	209.032267	213.835931	196.586219	113.659031
17.9523693	18.2367245	19.5964304	15.774853	18.667854	17.9469878	36.7545924
140.911642	140.082093	150.049963	144.571888	133.367896	129.555605	184.852694
7.09683704	7.05420553	5.4434529	9.24839418	4.5186248	7.97923579	197.305824
0	0.04008071	0.04733437	0	0.04564267	0	0
2.02391279	1.00201783	1.51469994	2.13424481	0.82156815	2.39125363	45.4279396
0.26284582	0.08016143	0.18933749	0.06186217	0.13692802	0.20136873	10.5421448
44.736358	46.8543538	42.7902732	44.3861059	44.0908238	39.1662173	20.8955051
49.5201518	47.8162909	55.4758852	49.6134591	56.3687033	55.5022553	53.9183556
39.3480187	39.1187761	36.5894704	43.1488625	32.4519417	32.5210494	16.9128081
3.52213394	4.20847489	2.79272801	3.83545444	3.37755793	4.43011199	9.1867262
94.9136243	83.5682871	72.7529314	84.1634802	94.7998354	87.0416321	44.5791664
1038.89809	1150.39663	1214.31601	1096.19762	1205.46868	847.636484	461.714345
102.089315	98.4382318	92.11269	100.618817	106.438717	101.691207	116.44229
50.0984126	52.1049272	47.6657137	53.6963622	49.4310167	54.872978	85.414147
4.8626476	4.48903989	5.15944666	5.53666408	4.42733945	5.78935089	1.45722813
242.895819	267.618922	261.901086	261.02742	258.930894	205.899523	126.277467
0	0.04008071	0.04733437	0	0.04564267	0	0
22.683594	23.3670558	26.2705771	23.0436577	20.1740622	24.5669847	94.7048002
0.89367578	0.80161427	1.04135621	0.9897947	0.95849617	1.15787018	1.6883713
1.36679825	1.40282496	0.99402183	1.33003662	1.55185094	1.45992327	2.4022761
23.6561235	22.1646344	24.1878646	24.3427633	24.6470444	25.0704065	16.3400268
59.560862	55.7121914	55.9965633	63.4705848	54.5429963	62.7263584	74.3468627
15.7970336	20.8820516	16.1410212	16.3316125	21.9541265	9.13710597	22.3015332
64.6863554	71.143266	74.6463063	63.5943092	71.430786	64.3121371	39.6740366
42.5547377	45.1709638	48.4230636	46.3038331	37.2444226	44.8297128	53.227591
19.2403138	17.6355138	20.2591117	18.4039951	21.1782011	21.2192296	12.0606674
125.824292	127.136022	119.28262	116.39367	123.326507	113.244738	124.028206
63.7926797	60.3615542	66.5047942	59.9444412	73.9867757	67.7605765	26.5973571
19.2665983	18.1966438	17.8450586	17.4142004	17.7550005	16.9904863	11.3011489
0.5782608	0.56112999	0.37867498	0.71141494	0.50206942	0.60410618	0.66017538
1.55079032	1.24250211	1.70403743	1.23724337	1.68877897	2.1898849	5.54695602
79.5371441	87.6565199	83.7345059	84.101618	85.8538712	93.661629	41.7223916
38.2966355	35.6317541	35.9741235	35.8491266	32.7257978	35.6170935	33.8495016
0.49940705	0.96193712	0.7100156	0.24744867	0.91285349	0.62927727	0.35539109
11.8280617	10.340824	10.6975683	9.15560093	12.5973782	13.9196132	9.00939835
1.76106697	2.00403566	2.03537804	1.85586505	1.00413884	2.08920054	1.26314119
77.3555238	71.984961	61.9606943	59.0783709	70.9743592	69.4973818	59.664485
0.49940705	0.52104927	0.66268122	0.68048385	0.63899745	0.62927727	0.32960199

0	0.04008071	0.04733437	0	0.04564267	0	0
18.8723296	19.1585809	17.513718	19.2391344	20.5848463	19.2810556	15.2133397
30.6478222	36.4333684	37.299486	28.5803218	34.7340755	34.7612764	28.4346072
34.1436716	37.7961126	35.3587767	38.3545444	34.7797181	39.9716922	53.3769337
26.5211429	37.876274	26.4599145	33.436502	21.4976998	28.7202146	24.2292185
20.7385349	20.8018902	22.3418241	21.5898968	17.5724298	20.6906367	22.7016952
18.3729226	18.6776124	21.6318085	15.6201975	19.5807075	19.6082798	12.8430432
5.28320091	6.01210699	5.72745914	5.53666408	5.56840632	4.53079635	6.8209624
12.064623	12.2246175	10.8395714	12.8364	12.0040235	12.7114009	25.8015308
57.7472259	57.8364692	59.5939757	59.2330263	49.6135874	56.9370075	61.8846527
8.3059278	8.17646551	10.0348871	10.3928443	9.49367634	8.68402634	10.4210453
45.9454487	45.3312867	47.9023855	42.4065165	47.9704511	41.9350373	25.0968479
42.2918919	43.9284617	44.3523076	43.2416557	46.0991015	47.9257569	15.7776909
15.770749	13.9881689	13.3956276	16.2078881	14.6969413	15.3040232	20.8902274
44.0792434	50.8223444	51.3577948	37.2100943	45.5513894	54.1681875	23.0532233
44.5523659	51.1029094	50.3637729	46.9533859	47.9704511	43.6970137	15.5515061
11.9857692	10.2205819	9.56154336	10.5165686	11.4106687	12.3590056	6.74425713
0	0.04008071	0.04733437	0	0.04564267	0	0
0.13142291	0.12024214	0.04733437	0.03093108	0.04564267	0.12585545	0.07449847
33.7494028	32.3852163	32.4240455	33.2818466	27.8876743	31.9672854	24.8481177
13.7731208	10.5412276	11.2655808	12.124985	8.76339355	11.9310971	24.7226058
0.13142291	0	0.04733437	0.06186217	0	0.05034218	0.06291047
0.10513833	0.04008071	0.09466875	0.15465542	0.04564267	0.12585545	0.10012781
119.174293	134.951762	131.40022	133.343904	152.172678	123.363516	59.8088853
3.4432802	3.00605349	3.78674985	4.14476529	3.46884328	3.62463708	3.33236864
2.39189693	1.8036321	2.08271241	2.66007324	2.37341909	2.06402945	2.44646513
13.0108679	15.9922046	15.6676775	14.9087826	7.39411331	17.5442503	31.8957979
33.4077033	31.583602	24.3298678	34.4881589	29.8046666	31.1618105	30.8431332
8.56877362	7.37485124	8.23618091	8.16580624	7.02897191	7.27444525	4.15295348
188.197605	182.247003	197.668342	181.843844	186.724182	167.664636	95.9847231
23.7349772	21.6836659	20.4011148	22.6106226	17.4355017	21.5716248	15.4152631
84.9780525	85.3719192	91.8760181	76.9256065	84.2563776	88.1743312	162.298829
112.077456	113.668903	119.377289	108.753692	114.973898	103.654552	93.9059601
19.8974283	17.8359174	16.8983712	20.7547575	17.5267871	19.9355039	10.2723068
0.26284582	0.08016143	0	0.18558651	0.1825707	0.17619764	0.2280393
5.20434717	5.25057344	7.95217467	4.54686938	5.38583562	6.56965471	2.46616357
0.05256916	0.16032285	0.14200312	0	0.13692802	0.10068436	0.01731245
23.4721314	26.7338357	24.7085427	27.2193541	20.3109903	22.6791528	20.1213123
80.2468278	86.1334528	83.9238434	78.7505404	81.4721744	78.3576058	28.8847323
11.2235164	14.3088146	12.5436089	14.1045744	12.0953088	14.5237194	7.26816427
2.99644231	3.9279099	4.11809046	2.59821108	3.46884328	3.77566363	3.92032487
11.3286547	10.5011469	12.4489401	12.124985	10.9085993	13.567218	42.1862297
0.02628458	0.04008071	0	0	0.04564267	0.07551327	0.12248968
0.31541498	0.36072642	0.37867498	0.24744867	0.41078407	0.60410618	0.60524314
0.02628458	0.04008071	0	0.03093108	0	0	0.02730662

1.76106697	1.5230671	1.84604055	0.89700144	2.82984583	2.34091145	1.34474862
1.36679825	1.64330924	1.08869058	1.29910554	0.91285349	1.33406781	1.74346629
4.96778593	4.64936274	4.54409981	4.60873155	5.52276364	5.36144235	11.6753494
2.23418944	3.44694134	3.6920811	2.90752192	3.6057713	3.8511769	1.34832316
0.02628458	0	0.04733437	0.03093108	0	0	0.01689624
87.4488031	90.582412	110.809767	85.9574831	89.6878558	96.8583575	168.33176
21.6584953	18.4772088	22.199821	20.940344	19.7176355	17.9973299	10.5136978
402.627222	387.660659	461.510137	436.406667	410.692787	486.733383	2065.10671
5.88774629	5.65138057	6.20080287	6.21714793	5.79661969	7.12341871	14.7590027
0.02628458	0.12024214	0	0.12372434	0.13692802	0	0.02171803
21.4482186	23.1666523	19.028418	22.4868982	27.7507462	18.3497252	4.89182251
38.3492046	38.6378076	40.6602265	35.0139873	37.746492	38.9145064	21.0556631
32.2511817	32.705862	32.7080518	30.8073599	36.8792812	35.5164092	28.6405776
110.447812	112.586724	114.596517	116.795774	105.023795	98.1672543	95.3824089
13.510275	14.1484918	16.8510368	14.8778515	14.7425839	17.8211323	14.7167591
0.34169956	0.96193712	0.18933749	0.68048385	0.22821337	0.22653982	0.26709012
0.05256916	0.32064571	0.09466875	0.37117301	0.09128535	0.15102655	0.05897027
132.448007	157.677526	131.826229	165.079197	158.243153	126.333705	141.503665
60.0602691	51.9045237	69.2028534	61.2744779	60.202688	70.5797387	31.2508902
0.02628458	0.04008071	0	0.03093108	0	0	0.03272625
91.10236	93.3079005	73.1789408	121.033333	54.9081377	107.430216	41.9041833
69.0758806	69.5400375	66.4574598	75.5646388	69.5137936	70.4790543	37.5303165
26.3108662	28.377145	24.5192052	27.0337676	27.8876743	28.7202146	69.2910554
6.07173836	6.13234913	5.82212789	5.2892154	4.5186248	6.5948258	8.034038
4.8626476	3.76758705	5.53812165	3.58800577	6.07047574	6.14174616	10.3721102
35.6418927	34.3090906	44.4943107	39.8392365	32.132443	35.4157248	33.1686592
6.51857625	5.77162271	5.68012477	7.85649539	5.93354771	5.81452198	7.06921838
85.950582	91.3038648	90.6926588	87.0091399	89.3683571	78.9113698	56.6193222
0.02628458	0.04008071	0	0	0.04564267	0.07551327	0.02202297
0.05256916	0.08016143	0	0.09279325	0	0.10068436	0.00999178
99.1717266	116.755118	113.507827	93.628392	110.774772	106.951965	240.466428
9.173319	9.94001689	10.6975683	9.4030496	9.17417762	9.11193488	12.2707338
10.3298406	14.1084111	12.4489401	10.5784308	11.3193833	12.2331501	15.581186
81.1930727	85.0512735	83.6398372	95.2677394	86.7667247	80.648175	33.2292707
54.724499	61.1230877	55.8545602	59.7279236	55.1819937	47.4978484	25.7525192
3.4432802	3.24653777	3.02939988	4.33035179	2.73856048	3.67497926	4.71681295
0.26284582	0.20040357	0.09466875	0.18558651	0.13692802	0.40273745	0.28454462
61.9001898	57.7963885	60.4459944	61.1507535	53.0824307	56.7356387	69.215348
0.13142291	0.08016143	0.04733437	0.12372434	0	0.05034218	0.13065234
248.100166	243.089526	196.058973	250.974817	222.462397	166.255055	56.5698565
62.3733122	61.6040563	47.1450356	64.7078282	71.8872127	47.6488749	17.85639
826.282109	779.650034	878.005286	800.527391	796.464674	731.95015	656.349829
0.63082996	0.6012107	0.61534685	0.64955277	0.59335477	0.35239527	0.62007634
0	0.08016143	0	0.06186217	0.04564267	0.02517109	0
0.02628458	0.20040357	0.18933749	0.09279325	0.13692802	0.05034218	0.07090086

0	0.08016143	0	0.06186217	0.04564267	0.02517109	0
23.3407085	21.6035044	19.5964304	23.0127267	20.9499877	24.5166425	16.5233478
0.65711454	0.36072642	0.09466875	0.49489735	0.22821337	0.17619764	0.71899691
0.02628458	0.04008071	0	0.09279325	0	0.02517109	0.17148556
0.02628458	0	0.04733437	0.03093108	0	0	0.17962872
0.02628458	0.04008071	0	0.03093108	0	0	0.02007087
5.36205466	4.20847489	4.9227748	4.73245589	5.75097702	5.43695562	2.3586201
2.18162028	1.08217926	1.2306937	1.08258795	2.23649106	2.06402945	9.09282439
108.213623	116.594795	120.702651	117.166947	121.272587	85.7327354	48.5962302
5.46719298	4.88984702	5.77479351	5.41293974	3.46884328	5.61315326	9.45959925
70.0221255	73.107221	66.4101254	71.234287	77.5012617	71.9138065	24.0880301
70.8895167	76.1934359	80.4684342	69.1928354	77.6381897	69.0694732	18.1551559
1.15652159	0.76153355	1.04135621	1.29910554	0.7302828	1.35923891	0.90747648
8.70019653	8.73759549	7.3841622	10.2072578	7.80489738	8.7847107	5.21579137
29.5964389	31.4232792	36.3527985	27.8070447	32.1780857	31.7659166	35.6522776
87.2122419	78.9990858	81.9831341	76.4925713	85.8995138	73.9023227	56.061719
144.407492	161.044306	168.699706	132.323178	153.450672	160.616731	107.191479
14.7982195	12.9059897	15.1469994	16.4553368	9.90446042	13.013454	10.6830593
0.02628458	0	0.04733437	0.03093108	0	0	0.02776644
106.925678	110.70293	105.460983	97.2473288	108.994707	100.407481	33.6897895
0	0.08016143	0	0	0.04564267	0	0
0.02628458	0.04008071	0	0.03093108	0	0	0.97434975
0.02628458	0	0.04733437	0.03093108	0	0	0.64307083
142.173302	136.033941	135.092301	141.942746	142.31386	151.907533	225.28304
0.02628458	0.04008071	0	0.03093108	0	0	0.06937118
0.02628458	0.16032285	0.37867498	0.06186217	0.1825707	0.12585545	0.00706438
35.5104698	33.4673956	31.1933518	37.3338187	33.1822245	35.2143561	32.9461015
0	0.08016143	0	0	0.04564267	0	0
29.780431	29.0985978	29.0159707	29.5391854	28.2984583	25.5234861	34.6457087
3.70612601	2.24451994	2.22471553	2.7528665	2.64727513	3.90151908	4.93592747
4.46837888	3.84774847	4.73343731	4.85618022	4.7011955	4.6818229	4.96692602
27.3359649	31.7840056	27.3592676	29.5082544	28.070245	24.113905	7.96941929
30.411261	29.5394857	33.0393924	28.9824259	30.5349494	28.4936748	27.4146262
35.0373473	31.6236828	40.5182233	38.2308201	35.0992169	39.9716922	56.2475998
0.23656123	0.16032285	0.33134061	0.15465542	0.7302828	0.15102655	0.20321761
197.213216	200.684131	156.440103	215.713381	211.416869	164.694447	35.9722692
16.2175869	15.8719625	16.9457056	16.9193031	15.7467228	19.406911	24.076135
26.573712	23.7277822	24.7085427	25.8583864	26.0163246	18.5510939	17.7780862
10.1984177	10.901954	12.9696182	9.49584286	9.85881774	12.0317814	16.5678276
0.47312247	0.16032285	0.09466875	0.24744867	0.13692802	0.20136873	0.45897205
1.65592864	1.32266354	1.18335933	1.5774853	1.46056559	1.71163418	3.78630491
0.13142291	0.16032285	0.18933749	0.06186217	0.13692802	0.15102655	0.14457528
26.5999966	24.4091544	23.0045053	24.0334524	25.0578284	21.0178608	20.048912
0.23656123	0.44088785	0.33134061	0.34024193	0.22821337	0.60410618	0.3264319
100.117971	108.538572	149.623953	92.2055621	148.79512	87.1423165	25.4886244

71.3626392	75.5922252	68.6348409	78.5649539	74.1693464	74.0281781	34.2273537
2.05019737	1.72347067	1.70403743	2.10331373	1.55185094	1.20821236	1.08335907
0.02628458	0.12024214	0.09466875	0.18558651	0.09128535	0.05034218	0.10576823
27.3359649	26.4933515	27.6906082	27.188423	26.655322	23.4594567	13.54656
6.64999916	6.01210699	6.43747474	6.71204528	6.84640121	7.07307652	11.1665697
13.4577058	15.9120432	13.0169526	13.7334014	16.4313629	15.2788521	8.91316369
2.31304318	2.24451994	2.4613874	2.10331373	1.59749362	2.4667669	7.11826835
8.0167974	6.37283341	6.43747474	7.23787371	5.88790504	9.6153567	25.2103604
1.49822115	2.24451994	1.32536245	1.94865831	0.59335477	1.661292	0.77249816
0.02628458	0.04008071	0	0.03093108	0	0	0.03144601
2.81245024	4.20847489	4.16542483	3.99010987	3.46884328	4.78250726	5.78223355
17.0324089	16.8338996	15.5256744	14.9397137	14.7425839	16.6884332	24.5267746
14.5879428	11.8238104	12.2596026	12.2177783	14.1948718	15.2033389	19.850073
1.34051366	1.24250211	1.46736556	1.67027855	1.18670954	1.661292	1.66142921
0.02628458	0.04008071	0	0.09279325	0	0.02517109	0.03013453
193.165391	164.531328	170.735084	177.204182	197.26764	186.064703	30.3859548
0.10513833	0.08016143	0.09466875	0.09279325	0.22821337	0.10068436	2.14356945
231.803726	260.284152	219.584157	240.860353	267.694287	146.646775	54.5626485
2.62845816	2.80564993	2.88739676	2.19610698	2.96677386	2.36608254	2.68393503
0.15770749	0.16032285	0.18933749	0.06186217	0.09128535	0.20136873	0.17482669
13.9833974	13.9480882	12.8276151	13.7024703	15.01644	14.8257725	19.6730123
57.300388	58.5980028	53.2985041	58.3669559	55.0450657	46.7678868	35.9921545
0.02628458	0.04008071	0	0.03093108	0	0	0.01578863
23.6561235	20.8820516	22.2471553	25.9202486	21.3607718	23.5097988	26.5853813
3.75869518	4.76960488	4.26009358	5.07269781	4.29041142	4.53079635	2.74750909
99.644849	96.9552454	90.2193151	97.1545356	97.5840386	76.2432341	106.690658
16.7432785	17.9160788	18.3657367	19.826825	16.8877897	19.4824243	67.1534625
5.02035509	6.3327527	6.01146538	4.82524914	5.84226237	5.26075798	8.51778982
8.09565115	7.37485124	7.81017156	7.98021973	8.6721082	8.43231543	5.86687846
77.5395159	84.1294171	74.5516376	74.0799467	78.7792566	72.1655174	34.4418838
102.483584	102.606626	112.277133	107.578311	100.368242	92.6296143	108.214639
111.18378	107.576634	100.01753	111.444696	87.1318661	102.345655	85.648288
0.36798414	0.28056499	0.47334373	0.43303518	0.31949872	0.553764	0.58309532
72.0723229	76.9950502	70.5755502	73.6469115	74.7170585	59.5296298	35.0487026
3.1541498	3.24653777	3.73941547	2.87659083	4.74683817	3.70015035	1.2939051
4.4420943	5.01008916	4.97010917	4.7015248	6.57254516	4.63148071	2.72651708
9.27845732	9.25864476	8.47285278	8.01115081	8.07875343	9.0615927	6.74402865
0.10513833	0.28056499	0.28400624	0.15465542	0.1825707	0.15102655	0.02842616
61.1379369	55.9927564	52.0678104	65.2955188	59.4267625	54.2437007	30.1812502
188.80215	207.537933	214.42471	198.54663	222.279826	224.727499	73.8241617
195.951556	194.872428	188.958817	180.946843	196.628643	172.220603	161.634965
13.6416979	16.6735767	13.9163057	13.7643325	16.1118642	11.3269909	4.61431996
13.8782591	12.7456668	12.8749495	17.0120963	9.95010309	15.807445	7.83075185
2.02391279	1.76355138	1.89337492	1.85586505	1.46056559	1.73680527	1.11048339
112.498009	119.11988	102.526252	120.785884	118.670954	113.018198	158.362668

1.26165992	2.08419709	1.51469994	1.85586505	1.59749362	1.96334509	1.63146935
41.845054	47.6960488	47.4763762	45.1284519	40.7132659	44.0997511	23.2939424
322.091264	346.978735	343.505545	294.340198	369.933879	287.302831	80.4840159
0.05256916	0	0	0.03093108	0	0.07551327	0.15996787
17.8735155	17.5553524	20.9217929	19.5793763	20.2653476	19.8851618	15.1310784
142.988124	146.935895	162.877578	143.736748	154.272241	129.656289	119.477641
6.96541414	7.97606194	7.57349969	8.25859949	7.80489738	4.43011199	11.0157577
0.05256916	0	0.23667187	0.18558651	0.09128535	0.10068436	0.04434971
0.13142291	0.20040357	0.18933749	0.15465542	0.3651414	0.17619764	0.12604289
0.07885374	0.08016143	0.04733437	0.03093108	0.13692802	0	0.05672486
0.13142291	0.12024214	0.23667187	0.12372434	0.27385605	0.22653982	0.77292168
0.15770749	0.08016143	0.04733437	0.06186217	0.04564267	0.10068436	0.38392289
0.81482203	1.00201783	0.85201872	0.95886361	0.63899745	0.67961945	0.69003793
90.47153	96.7548418	84.3498528	84.2253423	90.8745654	83.7190481	69.5397364
0.05256916	0.16032285	0.18933749	0.27837976	0.09128535	0.12585545	0.05475273
1.23537534	0.76153355	0.28400624	0.5876906	1.68877897	0.70479054	0.70174899
29.465016	23.8079437	23.9985271	30.4052558	25.1947565	26.5555008	43.8226386
28.7027632	22.6055223	25.2292208	24.2809011	22.8213374	25.1710908	30.3997121
86.6602657	82.0853008	96.0414429	77.9154012	85.0779457	88.677753	81.0475741
0.05256916	0.28056499	0.09466875	0.12372434	0.09128535	0.07551327	0.02779645
0.07885374	0.20040357	0.23667187	0.15465542	0.13692802	0.07551327	0.10507693
32.8031579	32.3852163	28.3059551	32.5704317	23.0951934	31.7659166	20.7110297
0.15770749	0.12024214	0.04733437	0.09279325	0.09128535	0.07551327	0.07331227
2.26047402	1.84371281	1.04135621	1.42282987	1.46056559	1.68646309	3.22097214
36.2464381	38.7981304	38.4355109	37.828716	38.0659907	31.6400612	20.1041641
9.51501856	9.73961332	12.0229308	7.88742648	11.501954	9.48950124	5.62434505
9.96185644	10.901954	11.7862589	8.93908334	9.76753239	11.5031885	36.6502024
29.2284548	30.3410999	29.2053082	29.1989435	27.9789596	27.159607	14.3882247
51.9120488	56.19316	58.5526195	47.1699034	52.3521479	58.0193644	146.320841
108.529038	116.153907	117.105239	108.537175	116.023679	99.6271775	29.6774279
0	0.04008071	0.04733437	0.06186217	0	0.05034218	0
9.80414895	9.85985546	9.18286837	10.2381889	8.76339355	7.82820925	5.94904318
3.75869518	3.44694134	3.40807486	3.30962601	1.91699234	4.10288781	6.70744925
30.2798381	24.4893158	24.7085427	25.0232471	24.281903	25.9010525	43.6031548
9.5938723	7.61533552	9.6562121	9.89794695	9.12853495	9.86706761	6.30292305
2.20790486	1.40282496	1.89337492	2.59821108	1.91699234	1.76197636	2.44314564
0.02628458	0	0.04733437	0.09279325	0	0.02517109	0.02438646
0.97252952	0.84169498	0.85201872	0.86607036	1.04978152	1.15787018	0.67043169
34.7219324	37.2750633	34.7434298	31.3331883	38.9788442	30.7087308	9.10890598
0.52569163	0.6012107	0.75734997	0.49489735	0.68464012	0.98167254	0.57314691
102.588722	82.2857043	106.407671	114.321287	78.1859018	120.368156	286.518889
5.51976215	5.93194556	3.78674985	4.97990456	2.46470444	5.91520635	2.11437099
4.10039474	3.40686063	4.40209669	4.17569637	3.9709127	4.83284944	3.71552037
69.9958409	71.143266	78.9537343	72.718979	71.0200019	65.3441518	65.7136466
5.57233131	5.05016987	4.82810605	5.25828432	5.24890759	6.29277271	4.31563839

23.7086926	23.4472173	19.0757523	26.446077	21.9997692	22.1253888	16.05008
7771.69368	10254.5302	9184.66708	8550.92917	11211.3015	9955.26711	15186.8985
61.1905061	60.4817963	64.5167505	62.4807901	58.4226237	75.7649834	137.978701
25.8114592	26.2929479	25.0398834	24.1881079	25.9250392	23.1825747	14.3783142
23.9715385	23.0464101	16.0463525	24.4046255	23.1864788	20.3885836	12.8332735
1.91877446	2.28460066	2.17738116	1.70120963	2.32777641	1.71163418	1.22282289
11.5915005	15.1104289	13.1589557	11.0733282	13.327661	12.736572	29.0270458
24.3920918	22.8860873	23.856524	23.878797	21.9084839	21.5716248	8.63507066
48.3110611	56.473725	52.4938197	51.778635	54.1322122	59.9323673	190.026397
0.49940705	0.52104927	0.99402183	0.34024193	1.04978152	0.276882	0.52939107
6.49229167	6.21251056	6.1534685	5.59852625	4.60991015	6.97239216	2.00326013
6.67628374	6.81372125	5.86946226	6.43366552	8.62646552	6.16691725	3.78364586
20.2128433	19.3189038	18.1290649	17.6616491	22.456196	21.3954272	26.3744785
5.78260796	6.0521877	5.82212789	5.35107757	5.34019294	5.61315326	12.2172352
43.6849747	41.8041839	41.6542483	38.4164066	38.5680601	43.8480402	14.8133572
8.35849696	8.7776762	9.08819963	7.26880479	7.48539866	9.43915906	8.36728826
24.2343843	25.6115758	24.8978802	22.7343469	26.4727513	21.5967959	8.86265035
4.04782557	5.41089629	5.30144978	5.35107757	4.7011955	5.91520635	4.36076215
60.2968303	50.7021023	54.7185353	58.0576451	55.8209912	60.7881844	92.8385458
338.177427	327.780073	319.980362	340.953341	314.204173	274.641772	236.663311
4.4420943	5.29065415	4.44943107	4.76338697	3.56012863	4.53079635	6.9755437
2.8650194	1.60322853	2.31938428	2.41262457	2.23649106	3.0205309	3.07838036
108.187338	118.759153	129.412176	112.094249	132.774541	132.198569	114.48441
110.815796	117.476571	130.027523	106.526654	112.96562	119.940248	282.709764
0.7096837	0.48096856	0.66268122	0.34024193	0.09128535	1.98851618	0.4695217
68.9444577	65.972854	60.39866	63.3159294	71.1112872	71.4355558	15.7216404
123.984372	133.709259	100.727546	132.756214	107.260286	95.5494608	55.5053087
1.15652159	0.80161427	1.18335933	0.71141494	1.09542419	0.95650145	0.23446401
13.1685754	12.2646983	12.7329464	11.8156742	12.2322368	11.8555838	9.20509965
103.140698	98.9993618	87.0479121	92.6695283	92.8828431	89.2063459	27.1246904
5.80889254	6.0521877	4.54409981	4.33035179	5.29455027	5.46212671	16.8987698
4.1529639	5.37081558	3.50274361	5.6913195	1.87134966	3.70015035	2.72619243
114.627061	116.193988	102.431583	107.423656	110.500916	98.368623	58.9042618
119.6737	121.644965	121.033992	117.723707	118.716597	107.858124	99.4531761
6.86027581	5.731542	5.72745914	6.30994118	4.92940887	5.63832435	4.51794045
0.60454538	0.04008071	0.09466875	0.49489735	0.04564267	0.52859291	0.42996015
0.60454538	0.04008071	0.09466875	0.49489735	0.04564267	0.52859291	0.70398045
69.8907026	68.8185847	70.0548721	68.5123516	76.7709789	74.1288625	28.1933281
0.36798414	0.28056499	0.37867498	0.24744867	0.09128535	0.42790854	0.94668682
35.0373473	37.5556283	34.2227517	34.51909	36.2402837	30.6332175	13.1152604
0.94624494	0.16032285	0.47334373	0.30931084	0.50206942	0.50342182	0.46171819
366.643629	366.498042	391.123925	419.208985	400.560113	371.298761	252.752749
4.02154099	4.80968559	6.10613413	4.54686938	5.52276364	6.21725944	4.82066818
24.7337913	25.0103651	23.9511928	24.1262457	24.1449749	29.5760317	51.1954022
1.44565199	1.76355138	1.13602495	1.48469204	1.50620827	1.9130029	2.13451393

56.0387281	51.1830708	54.1978572	57.2534369	54.1778549	58.2710753	24.5352007
0.07885374	0	0.09466875	0.09279325	0.13692802	0.05034218	0.10344303
0.18399207	0.24048428	0.09466875	0.24744867	0.27385605	0.17619764	0.49740285
33.1448575	32.4653777	30.2466644	33.9004683	32.3606564	34.6857632	34.2301529
14.9033578	15.230671	14.2949807	15.5274043	15.4272241	16.2353536	6.24886311
28.5713403	28.4573064	29.6786519	25.3944202	32.6801551	23.8118519	14.0168036
1.15652159	1.28258282	0.99402183	1.54655421	0.77592547	0.60410618	0.89939977
0.02628458	0	0.04733437	0.03093108	0	0.07551327	0.04395563
20.7648195	18.9581774	21.5844741	21.1568616	21.6346278	22.0750467	22.977203
0.02628458	0	0.04733437	0.03093108	0	0.07551327	0.10239981
0.05256916	0	0	0	0	0.02517109	0.11125793
10.5926864	9.21856405	10.1768902	11.1351903	9.81317507	11.1256221	17.0386289
0.05256916	0	0	0	0	0.02517109	0.04756441
2.47075067	3.80766776	1.84604055	1.79400289	2.19084839	3.09604417	2.00028651
0.05256916	0	0	0	0	0.02517109	0.04658246
0.05256916	0	0	0	0	0.02517109	0.04171721
0.05256916	0	0	0	0	0.02517109	0.01844989
0.05256916	0	0	0	0	0.02517109	0.0880316
0.05256916	0	0	0	0	0.02517109	0.33845833
0.05256916	0	0	0	0	0.02517109	0.16198258
0.05256916	0	0	0	0	0.02517109	0.10918011
0	0.08016143	0	0	0.13692802	0.02517109	0
4.38952514	3.48702205	4.30742795	3.24776384	3.19498723	4.53079635	1.59858335
0.05256916	0	0	0	0	0.02517109	1.62802743
0.05256916	0	0	0	0	0.02517109	1.62802743
0.05256916	0	0	0	0	0.02517109	1.71485556
39.9525641	39.6799061	40.3762202	36.4058861	38.9332015	38.9396775	36.8716585
0.05256916	0	0	0	0	0.02517109	0.16616817
77.27667	90.2216855	84.8705309	86.3595872	87.5426501	90.7669535	268.17422
0.05256916	0	0	0	0	0.02517109	0.04258747
9.85671812	8.09630408	8.33084966	9.09373876	9.40239099	9.84189652	8.37041175
59.1403087	61.083007	61.2980131	53.3870514	56.0035619	62.902556	43.5684847
8.83161943	9.89993618	9.37220587	8.75349684	11.5475967	9.99292306	7.54704157
13.9045437	13.2266354	14.2003119	13.8571257	13.327661	11.6038729	4.30831397
61.9264744	53.0668644	63.0020505	62.7591699	47.1945257	57.7173113	104.415912
7.14940621	7.09428625	7.05282159	7.79463323	6.93768656	4.88319162	5.02919111
0.55197621	0.40080713	0.66268122	0.37117301	0.3651414	0.57893509	0.90150117
41.1353703	41.5636997	41.7962514	40.0866852	45.4144614	32.1686541	31.6479829
524.429973	508.223444	601.051869	593.6603	533.836724	611.556823	2001.64575
55.9073052	67.9768897	61.1086756	54.9954678	61.5263255	58.8751815	45.5481739
259.428821	241.766862	242.257321	258.367347	274.22119	252.21433	271.941265
7.38596744	8.4570305	7.3841622	7.54718455	6.70947319	7.29961634	3.22856717
161.229624	156.394943	164.20294	161.120018	163.400776	130.260395	117.189438
160.204525	183.249021	149.576619	163.1924	175.0853	123.111805	34.7783206
0.02628458	0	0.04733437	0	0.04564267	0	0.02809396

0.02628458	0	0.04733437	0	0.04564267	0	0.05249558
91.4703441	91.7848334	87.8999308	102.753062	86.2190126	96.3801068	119.099867
0.02628458	0	0.04733437	0	0.04564267	0	0.02550856
2.94387314	2.20443923	1.7513718	2.90752192	1.09542419	4.05254562	3.81482698
0.02628458	0	0.04733437	0	0.04564267	0	0.0293238
10.2509868	7.89590051	8.23618091	8.38232383	8.80903622	11.4276752	35.3236092
1.05138327	0.80161427	0.85201872	0.83513927	1.09542419	1.20821236	0.16503807
16.1387331	18.8379352	14.9576619	18.2802708	18.6222113	16.3863801	9.38098104
0.02628458	0	0.04733437	0	0.04564267	0	0.02461044
0.23656123	0.64129141	0.61534685	0.37117301	0.91285349	0.52859291	0.10826108
0.05256916	0.12024214	0.04733437	0	0.1825707	0.15102655	0.17618379
10.5926864	8.7776762	10.6975683	10.0216713	11.4563114	10.8235691	18.1229053
225.337718	232.267733	242.588662	232.756409	215.113926	216.21967	231.835419
27.9142257	24.5293965	25.8445677	25.9511797	25.2860418	23.1322325	19.6078445
0	0	0.09466875	0.06186217	0.04564267	0.02517109	0
35.5893236	38.7981304	30.4833363	36.6533348	37.609564	36.573595	17.3865397
136.206702	135.512892	119.992636	116.239015	128.027703	127.290206	51.7934887
15.7444644	14.2286532	10.7449027	13.6406081	11.2737407	12.9631118	7.55144931
89.4727159	96.1135504	84.9651996	88.5247631	89.3683571	80.1195821	105.800537
0.97252952	1.72347067	1.46736556	1.23724337	1.73442164	0.60410618	1.0449548
10.934386	12.865909	11.7862589	11.3517079	10.9085993	11.0249378	12.6426024
746.061565	662.854836	732.168083	670.802424	785.829931	827.751322	1216.8615
17.5581005	14.5492989	14.9576619	17.3832693	16.842147	16.0088138	12.7962859
0.07885374	0	0.09466875	0.03093108	0.1825707	0.07551327	0.12148693
0.10513833	0.16032285	0.14200312	0.12372434	0.22821337	0.17619764	0.12696364
15.2713419	13.1464739	15.5730087	15.5583354	11.9583808	13.5168758	17.740938
14.5090891	12.1444561	14.2476463	16.3625436	11.3193833	16.2101825	13.3099025
16.1124485	17.6355138	12.7329464	17.5069937	13.7840878	11.8052416	8.93072997
3.94268725	5.01008916	4.63876856	4.23755854	5.24890759	5.31110017	5.26244545
24.7600759	25.6115758	26.5545833	24.4664876	24.7839724	23.7866808	44.8388398
59.1665933	67.656244	75.877	58.8927844	67.1860172	65.6210338	49.590697
44.9992038	54.50977	55.3812165	50.6032538	53.0824307	42.4888013	21.3194668
0.52569163	0.32064571	0.37867498	0.61862168	0.31949872	0.52859291	2.77185704
67.1308215	77.6764223	74.6463063	69.9042504	76.9535496	61.1405796	36.208177
0.05256916	0	0	0.03093108	0	0	0.16531384
160.388517	159.922046	156.061428	162.171675	142.9985	146.29438	159.060325
0.76225287	0.56112999	0.47334373	0.5876906	0.5477121	0.45307964	0.5038923
73.5179749	67.8566475	67.3568129	56.7894706	66.8665185	74.0533492	29.029521
11.5389313	11.6234068	10.3662277	12.3415026	9.95010309	8.96090834	10.1622785
78.7486066	79.1594087	71.1908971	80.6682677	67.4598733	69.4218685	35.6191573
73.2025599	80.4820722	79.427078	64.9243458	87.2231514	94.9453546	104.611698
70.9683704	67.4959211	68.4455034	70.5538031	79.418254	67.7857476	99.1599801
8.48991987	8.85783763	9.37220587	10.8568106	10.1783165	7.82820925	11.1254354
0.05256916	0	0	0.03093108	0	0	0.07181137
0.05256916	0	0	0.03093108	0	0	0.05817013

0.05256916	0	0	0.03093108	0	0	0.04054671
8.43735071	7.29468981	9.46687461	9.27932527	7.89618273	9.81672543	8.50188376
47.3385315	46.413466	46.2930169	56.5729531	44.1821091	46.7678868	17.665811
0.05256916	0	0	0.03093108	0	0	0.13070545
21.0539499	22.0043116	19.6910992	21.4661725	20.1284196	17.6952769	12.8549972
26.074305	24.2889122	25.371224	26.8481811	25.7424685	27.0337516	59.0126056
0.13142291	0.12024214	0.37867498	0.24744867	0.09128535	0.10068436	0.09612419
8.85790402	12.9059897	12.2596026	12.9291932	9.67624704	13.9699554	8.4588162
25.7326054	25.2508494	29.4893144	30.2196693	25.0121858	29.5508606	19.6739483
47.1808241	47.7361295	48.6597355	47.4792143	48.7463766	54.7219515	84.1335384
1.49822115	1.48298639	0.94668746	1.26817445	1.23235222	1.98851618	1.18013643
0.05256916	0	0	0.03093108	0	0	0.03921164
3.78497976	4.08823275	4.02342171	3.9282477	6.75511586	2.86950436	2.41655011
0.18399207	0.24048428	0.23667187	0.34024193	0.31949872	0.17619764	0.08685116
3.12786522	4.00807133	1.84604055	3.27869493	2.92113118	1.76197636	1.79216462
0.26284582	0.44088785	0.37867498	0.18558651	0.31949872	0.35239527	0.16612525
39.8474258	41.0025697	38.9088547	37.6431295	33.1822245	42.5139724	60.0674913
7.67509784	8.17646551	9.89288397	10.6712241	9.17417762	7.90372252	31.5591065
97.9100666	106.734939	92.7280368	108.258795	102.832946	101.791891	59.573212
0.05256916	0	0	0.03093108	0	0	0.0506754
3.36442645	3.84774847	3.64474673	4.08290312	3.83398468	3.87634799	16.5619853
20.8962424	22.0443923	24.3772021	20.9094129	21.0412731	21.5716248	17.7576003
0.13142291	0.20040357	0.42600936	0.34024193	0.09128535	0.10068436	0.08386422
14.3776662	13.7877654	13.3956276	11.9703296	14.1492292	13.013454	33.9209013
49.3098752	54.2692858	58.5052851	51.8404972	50.0243715	51.9279604	57.3930011
0.63082996	0.64129141	0.85201872	0.34024193	0.68464012	0.75513273	0.26752817
99.6974182	109.01954	111.330445	105.938963	106.575645	90.9934934	81.6321175
21.7636336	20.6014866	17.0403743	22.394105	15.290296	17.7204479	11.20974
13.4839904	12.9059897	9.79821522	13.8880568	8.17003878	11.1507932	9.11564901
18.5569146	17.475191	20.2117773	19.1463411	17.0703603	16.7387754	34.2131129
0.05256916	0.08016143	0.04733437	0.09279325	0.04564267	0.10068436	0.04310126
0.05256916	0	0	0.03093108	0	0	0.0794405
43.8163976	38.998534	32.992058	37.9215093	40.7132659	43.9990668	23.7535803
3.33814187	3.24653777	4.59143419	3.46428143	4.3360541	4.43011199	7.44484921
0.05256916	0	0	0.03093108	0	0	0.11125793
103.850382	103.127675	109.815746	107.083414	106.119219	87.1674876	57.836851
12.9582988	13.5472811	14.5789869	13.3312973	11.5932394	13.4413625	12.1097754
0.21027665	0.20040357	0.18933749	0.18558651	0.13692802	0.15102655	0.35900675
0.02628458	0.04008071	0	0	0.04564267	0	0.04718055
48.8893219	48.2571788	44.3049732	49.4588037	51.3936517	48.0516124	17.055636
0.05256916	0	0	0.03093108	0	0	0.03933155
978.233275	999.973715	936.131896	984.691066	962.740938	780.731724	353.831567
3.02272689	3.64734491	2.9820655	3.27869493	3.37755793	2.08920054	0.9985572
31.5677826	35.1107048	33.5600705	30.9620153	39.0244869	35.9443177	14.1711573
0.52569163	0.16032285	0.42600936	0.43303518	0.31949872	0.60410618	0.55389391

0.10513833	0.32064571	0.09466875	0.15465542	0.09128535	0.12585545	0.15060207
2.94387314	3.00605349	2.36671865	3.03124625	2.78420316	3.34775508	4.26177121
0.26284582	0.20040357	0.28400624	0.40210409	0.22821337	0.276882	0.14820715
36.7458451	36.3532069	37.2048172	38.5091999	36.1033557	29.9535981	15.0563227
0.55197621	0.24048428	0.23667187	0.77327711	0.09128535	0.45307964	0.52383582
1.8662053	1.60322853	1.70403743	1.5774853	1.87134966	2.34091145	9.11337908
23.4458468	27.2148043	27.9746145	27.528665	24.8296151	21.7981647	16.7773964
7.91165908	9.81977475	10.6975683	8.32046166	11.4563114	6.19208835	6.23596395
120.777653	140.763465	130.690204	122.146852	140.579438	107.75744	43.8348981
2.36561235	1.12225997	3.171403	1.29910554	1.50620827	2.79399108	289.381875
0.05256916	0	0	0.03093108	0	0	2.14356945
125.719154	118.999638	90.5033213	120.940539	104.384797	88.8791217	71.8814629
19.8448591	17.3950296	16.9457056	20.0742737	13.464589	16.9401441	31.0831293
0.49940705	0.84169498	0.37867498	0.24744867	0.86721082	0.35239527	0.28114003
116.098997	124.410534	165.386299	131.457108	173.807305	129.882829	138.964964
81.9027564	88.9391027	79.1430718	77.8226079	87.9534342	72.2410307	88.8213084
36.6669914	41.4434575	35.4534454	38.3854755	42.3107595	38.9145064	8.4138418
26.4685737	27.254885	25.9865708	26.7553879	27.0661061	29.9787692	47.1304461
0.02628458	0.04008071	0	0	0.04564267	0	0.06826654
0.13142291	0.16032285	0.18933749	0.09279325	0.27385605	0.25171091	0.04603886
0.13142291	0.08016143	0.04733437	0.12372434	0.09128535	0.15102655	0.13618611
0.34169956	0.52104927	0.47334373	0.15465542	0.86721082	0.65444836	0.34136059
1.47193657	2.36476208	2.69805926	1.42282987	1.55185094	2.59262236	0.32657991
4.33695597	3.16637635	2.88739676	3.89731661	3.87962735	3.80083472	7.3839031
0.13142291	0.04008071	0.09466875	0	0.09128535	0.10068436	0.19909314
0.47312247	0.68137213	0.94668746	0.77327711	0.63899745	0.32722418	0.4139941
3.02272689	3.04613421	2.65072489	2.7528665	2.78420316	4.07771672	2.08494914
99.3031495	99.9212182	108.064374	104.70172	102.285234	116.391124	127.400189
44.2632355	51.7842815	47.3817074	47.9741116	45.2318907	43.2439341	31.298592
52.8845783	52.2652501	50.4584417	56.6966774	51.3936517	55.3260577	21.0966659
11.1709472	12.4250211	10.9342402	13.1147797	13.3733037	10.5718582	13.3189622
0.18399207	0.24048428	0.23667187	0.52582843	0.09128535	0.20136873	0.17841838
0.05256916	0.08016143	0.04733437	0.09279325	0.04564267	0.10068436	0.10439462
162.832983	166.495283	176.746549	177.204182	159.247292	141.360846	42.0411969
0.94624494	1.16234068	1.2306937	0.92793253	0.95849617	1.00684363	0.71540637
0	0	0.09466875	0.03093108	0.04564267	0.05034218	0
0.05256916	0	0	0.03093108	0	0	0.10826108
8.22707406	9.45904833	7.9048403	7.82556431	10.3608872	9.41398797	16.5936662
22.9990089	22.8059258	24.2825334	21.0949994	23.0039081	22.3519287	14.6190434
1.94505904	3.04613421	1.60936868	2.07238264	1.50620827	2.1898849	0.55631566
0.65711454	0.6012107	0.42600936	0.68048385	0.82156815	0.553764	0.45959894
116.466981	125.653036	126.051435	118.466053	121.090016	110.375233	45.2293153
73.6493978	69.3797147	70.7648877	78.3793674	62.1196803	61.9712256	41.9140376
15.2976265	14.9901868	13.5849651	16.0841638	15.7467228	14.9012858	11.6195972
0.02628458	0.04008071	0	0	0.04564267	0	0.50239909

10.2247023	9.45904833	9.04086526	10.1763267	9.72188972	10.8235691	15.1608821
0.2891304	0.16032285	0.47334373	0.18558651	0.1825707	0.32722418	0.62269183
23.2355702	21.1626166	24.1878646	25.5490756	22.730052	24.0635628	19.7730302
0.81482203	0.32064571	0.99402183	0.43303518	1.27799489	0.90615927	0.55887849
0.23656123	0.08016143	0.04733437	0.09279325	0.22821337	0.22653982	0.35376757
4.59980179	3.84774847	4.49676544	3.64986794	3.65141398	4.50562526	3.71165554
0.18399207	0.24048428	0.09466875	0.15465542	0.04564267	0.17619764	0.26573175
0	0.16032285	0.04733437	0.03093108	0.13692802	0.12585545	0
59.0877395	57.1550971	40.8968983	76.3069848	50.5264409	43.8983824	21.9067015
23.3932777	21.1225359	24.6612084	22.0538631	26.3358233	24.8690377	112.222165
11.4600776	10.0201783	10.0348871	11.011466	8.35260948	9.94258088	9.55619916
4.07411016	3.04613421	2.9820655	3.55707469	4.19912608	3.52395272	9.65852512
2.8650194	2.80564993	2.79272801	3.24776384	2.87548851	3.14638635	2.161416
1.99762821	1.5230671	1.51469994	1.7630718	1.36928024	2.54228017	1.48011458
0.65711454	0.9218564	0.37867498	1.29910554	0.63899745	0.45307964	0.17294289
34.932209	29.77997	32.0453706	31.1476018	35.0535742	36.019831	26.8755075
21.6847799	19.9201145	20.4484492	22.1157252	22.1366972	21.3702561	40.0402595
50.702958	59.3194556	51.5944666	49.953701	58.7877651	61.8705413	36.8424009
186.383968	201.846472	199.277711	188.741476	194.802936	174.334975	244.89878
9.8830027	10.3007433	8.14151217	10.3619132	11.5932394	8.75953961	3.50782872
20.2391279	20.962213	21.9158147	20.3217223	22.456196	16.6884332	20.6835648
87.8430719	95.5524204	86.1012246	79.5547486	90.78328	86.8150923	24.1232768
0.02628458	0.04008071	0	0	0.04564267	0	0.04882846
3.49584936	3.84774847	3.92875296	3.30962601	3.74269933	3.27224181	6.7186505
2.97015773	2.48500422	2.36671865	1.88679614	1.36928024	3.49878163	2.12166436
0.34169956	0.20040357	0.33134061	0.30931084	0.31949872	0.42790854	0.21121579
6.38715334	10.1805012	8.99353088	7.02135612	7.43975598	8.86022397	16.043759
4.23181765	4.76960488	3.78674985	3.83545444	3.6057713	4.25391435	8.02592281
0.13142291	0	0.09466875	0.21651759	0.04564267	0.07551327	0.10837055
10.1984177	10.7817119	12.0702651	9.46491177	11.365026	10.0684363	12.3642955
40.6359632	33.0265077	39.5242015	38.942235	37.1074946	31.035955	18.4621636
2.44446609	1.88379352	1.98804367	2.47448674	2.14520571	2.41642472	7.61854618
43.2118522	39.4394218	39.8555421	45.3759006	42.2194741	41.0540492	26.0203901
0.13142291	0.16032285	0.42600936	0.18558651	0.22821337	0.42790854	0.13363899
15.5867569	15.1104289	16.9930399	16.4862679	15.8836508	18.0980143	8.4817839
0.05256916	0	0.09466875	0.03093108	0.04564267	0.12585545	0.0286382
3.02272689	2.20443923	4.02342171	3.00031517	3.24062991	3.97703235	5.42576272
0.21027665	0.04008071	0.18933749	0.06186217	0.27385605	0.276882	0.59751065
0	0.08016143	0	0	0.09128535	0.05034218	0
12.56403	9.61937118	10.5555652	10.0526024	10.5891005	10.0684363	3.07664757
10.8555322	10.7015504	10.5555652	9.52677394	11.0455273	9.6153567	9.12674413
49.6515747	54.5498507	47.1450356	48.2524914	61.5263255	50.3925239	44.676749
73.5968286	67.4558404	73.841622	72.9973588	70.2897191	61.1154085	39.827435
12.380038	12.2246175	11.4075839	14.8159893	9.26546297	14.6495749	13.7176795
3.70612601	4.28863632	3.02939988	3.89731661	3.24062991	4.95870489	2.45526638

100.801371	90.7026541	115.732542	98.3917789	99.5923163	93.1833783	53.4498623
2.99644231	2.16435852	1.9407093	1.60841638	4.01655538	2.66813563	3.12489663
87.2910957	80.0011037	68.9661816	83.9469626	79.5095394	84.9776027	113.416794
7.20197537	6.97404411	8.75685902	11.3207768	5.52276364	8.2309467	5.83062238
17.7683772	18.9180967	18.1763993	18.4967884	18.2114272	21.2695718	9.67109863
0.05256916	0.04008071	0.09466875	0.03093108	0.1825707	0.07551327	0.46768788
16.7169939	12.5051825	13.9636401	16.9811652	15.3815814	13.9951265	7.89409477
35.6418927	27.8961764	22.9571709	23.5385551	37.2444226	32.8734446	39.3681287
4.67865553	4.76960488	3.55007798	4.94897348	4.92940887	4.4049409	3.48877197
158.154328	144.97194	157.576128	162.975883	157.147729	165.22304	82.5198807
3.65355685	4.16839418	4.87544043	3.12403951	4.1534834	4.22874326	4.81610161
9.12074983	8.7776762	9.04086526	9.83608478	8.39825215	10.521516	8.13509221
85.5563133	79.8808615	85.0598684	93.7211852	71.6589993	72.0648331	48.5322412
0.13142291	0.04008071	0.18933749	0.27837976	0.13692802	0.07551327	0.12673844
6.70256832	5.17041201	6.05879975	7.08321829	5.93354771	6.39345707	3.78626328
60.375684	63.4076884	60.9193381	58.1195073	58.7421224	59.30309	69.4468126
0.49940705	0.68137213	0.47334373	0.89700144	0.50206942	0.52859291	0.42901495
0.31541498	0.36072642	0.28400624	0.43303518	0.41078407	0.32722418	0.27808468
27.9930795	26.693755	26.9332583	28.6731151	24.692687	24.113905	17.6558504
164.830612	200.804373	189.053486	146.304028	182.798912	192.130936	123.702368
132.737137	123.168032	118.761942	132.818076	133.459181	127.919484	176.975897
43.5798364	41.4033768	44.92032	45.0356586	45.5513894	45.45899	22.0200628
160.940493	153.869858	141.529775	144.540957	144.459066	144.356206	41.3735706
6.12430752	6.17242984	6.05879975	5.62945733	6.80075853	7.37512961	6.82311039
3.52213394	2.76556921	2.60339052	3.95917878	3.14934456	2.81916217	3.87461743
0.2891304	0.40080713	0.23667187	0.43303518	0.3651414	0.32722418	0.21166305
15.9810256	15.0302675	15.7623462	17.3523383	16.7508616	15.530563	5.95019125
475.750928	464.856112	373.799544	473.86421	399.692903	357.605687	206.962697
21.3167957	22.2848766	23.1938428	21.3733792	20.7217743	21.1185452	13.8889599
15.2450574	13.6675232	12.1649339	15.1253002	14.1492292	8.65885525	18.72395
1.23537534	1.16234068	0.75734997	0.95886361	1.32363757	1.38441	0.83816775
24.4972301	22.1646344	26.5072489	21.3424481	23.7341909	24.1390761	20.5183847
143.802946	162.366969	173.764484	136.127702	164.359272	152.889206	224.951441
48.8104681	57.4356621	62.8127131	55.9852625	59.4724052	43.5711582	12.3302275
34.5379403	25.0504458	55.4758852	18.6205127	40.7589085	42.7405122	19.9103458
5.88774629	4.44895917	5.34878416	5.93876817	5.61404899	5.51246889	4.70898551
4.94150135	6.6533984	4.9227748	5.44387082	5.34019294	7.12341871	11.9700314
0.05256916	0.04008071	0.09466875	0.09279325	0.04564267	0.10068436	1.53112103
365.250547	361.728437	393.679981	346.087901	356.104148	359.367664	515.34673
50.8606655	52.4255729	59.9726507	52.7993608	46.0078161	55.6784529	75.0055493
15.139919	14.7497025	15.1469994	13.9189879	15.4272241	13.1644805	9.50009747
8.64762736	11.4230033	11.0762433	9.74329153	11.4106687	12.182808	17.0483726
16.8747014	19.1986617	16.8037024	15.3727489	18.7591393	15.7319318	9.65057212
42.1867535	42.3252332	47.8550512	43.0560692	45.2775333	37.5300964	24.6096492
142.147018	140.803546	148.7246	147.324754	123.326507	138.69271	141.832262

15.0610653	20.2407602	17.1823774	10.7021551	15.3359387	21.7729936	7.74604977
11.5915005	10.5011469	11.5969214	11.382639	10.4521725	9.76638324	12.3248256
295.149567	265.013676	293.189107	299.784068	271.254416	234.946962	349.34893
0.52569163	0.28056499	0.37867498	0.37117301	0.27385605	0.35239527	0.37761059
20.7385349	17.5553524	15.0523306	21.1259305	17.4355017	18.5007518	23.7316599
38.7960425	34.8702205	32.4713799	34.3335035	35.2361449	31.4386925	21.0459546
104.218366	107.576634	113.034483	101.763267	108.446995	99.0230713	41.5475942
1.02509868	1.36274425	0.80468434	1.11351903	0.91285349	0.78030382	0.69977016
746.140419	796.644257	856.799487	740.335501	831.655176	681.079376	801.706269
14.2988124	14.4691375	14.8156588	14.321092	13.9210158	12.8372563	21.7691682
6.88656039	6.13234913	6.34280599	6.71204528	6.61818784	7.47581398	11.8651097
163.779228	179.761999	171.066424	163.811022	216.574492	166.557108	22.1245341
0.05256916	0.04008071	0.09466875	0.12372434	0.04564267	0.07551327	0.06417873
54.0148153	53.9085593	48.0443887	54.0056731	47.5140244	46.7427157	16.4489739
24.0241076	23.0063294	22.6731647	23.878797	22.86698	26.7065274	37.2475755
31.226083	26.6135936	25.371224	26.1058351	27.6138182	25.4479728	12.7455481
31.9620513	31.2228756	32.2820424	31.0857396	29.1656692	30.482191	54.492971
1.49822115	0.80161427	0.94668746	0.9897947	0.82156815	1.98851618	0.55286633
0.60454538	0.48096856	0.56801248	0.43303518	0.59335477	0.42790854	0.35065503
17.1901164	17.4351103	18.6970774	16.5481301	16.7508616	17.0408285	31.4561201
6.0191692	4.64936274	10.6502339	5.84597492	8.3069668	9.56501452	10.4294066
18.7934759	18.0764017	19.8331023	15.1562313	19.4437794	18.8028049	13.9247621
5.67746964	5.81170342	4.44943107	5.56759516	5.38583562	6.34311489	6.24004043
66.6576991	57.595985	60.9193381	60.8105116	65.5885236	68.2891694	38.058988
2.10276653	1.92387424	2.36671865	2.25796915	1.73442164	3.04570199	8.01334372
5.09920884	3.96799061	4.73343731	5.32014649	4.5186248	5.16007362	9.40133698
1.49822115	1.16234068	1.04135621	1.39189879	1.09542419	1.71163418	1.28840202
0.15770749	0.08016143	0.33134061	0.06186217	0.31949872	0.35239527	0.61439889
38.0863588	38.1167583	42.884942	35.6944712	35.0079315	42.1364061	43.6444795
7.99051282	7.33477053	6.86348409	6.40273443	6.80075853	7.50098507	6.81874899
14.0359666	10.7416312	21.9631491	9.34118744	21.4520571	21.8736779	10.4567547
13.5891287	13.3869582	11.5969214	14.8778515	12.5973782	13.6427312	11.6410231
2.60217358	3.16637635	3.02939988	3.03124625	2.55598978	4.00220344	2.30666712
33.7494028	29.9002121	28.9686363	33.8695372	31.0370188	32.6720759	16.55047
21.3693649	18.2768052	18.6024086	24.2809011	15.7010801	13.869271	8.53160228
0.13142291	0.32064571	0.42600936	0.12372434	0.31949872	0.22653982	0.13823535
6.80770665	8.97807977	6.91081847	6.99042504	6.66383051	7.34995852	4.08123857
117.991487	97.9572632	96.4201179	106.062688	122.641867	101.691207	66.2857629
335.154701	335.796216	324.666465	342.963862	310.644044	289.241005	161.987282
0.31541498	0.16032285	0.14200312	0.27837976	0.04564267	0.15102655	0.27848611
0.26284582	0.12024214	0.28400624	0.24744867	0.31949872	0.276882	0.25357683
18.3992072	19.7998724	20.306446	15.1253002	24.8752577	14.2468374	16.8911664
27.6776645	25.0504458	28.0692832	28.3328732	27.9789596	28.5691881	12.3703615
6.22944585	6.25259127	6.57947786	5.22735323	5.97919039	6.44379925	16.8035047
0.18399207	0	0.09466875	0.06186217	0.22821337	0.15102655	0.54896291

1920.5881	1876.37859	1970.10394	1723.44908	1992.85046	1677.37632	1225.25535
30.8318143	31.8240863	38.1988391	33.1890534	29.3938825	32.7224181	47.8630766
5.96660003	5.93194556	5.72745914	5.2892154	5.47712097	5.61315326	2.50561413
70.9158013	72.7865753	72.4215908	74.9769482	74.3519171	76.8473403	17.5661143
1.23537534	1.48298639	0.85201872	1.1753812	1.55185094	1.38441	1.12024941
20.2654125	26.1727058	24.8978802	23.0436577	27.7963889	24.6928401	14.0058648
13.3788521	13.5873618	10.8395714	12.2487094	15.3359387	13.0637961	8.8657382
266.735935	266.817308	235.819847	260.903695	251.810636	215.51488	84.2266755
42.2393227	42.1248296	42.4589326	42.6230341	47.1945257	43.1432497	37.5787211
17.7683772	20.6415673	18.6970774	13.5478149	16.2487922	23.006377	3.35532482
2.62845816	2.76556921	1.98804367	2.81472866	2.32777641	3.0205309	5.83548851
0.44683789	0.9218564	0.18933749	0.92793253	0.41078407	0.553764	0.4757269
20.4756891	20.2808409	19.8804367	20.4763778	16.1118642	20.4389258	46.0011184
101.747616	106.734939	98.8815053	106.928758	93.9782673	91.3962308	84.8441444
1.02509868	0.9218564	1.51469994	1.33003662	1.55185094	1.05718582	0.64855864
9.06818067	11.1023576	8.89886214	10.9496038	6.20740376	9.38881688	8.73462353
31.7517746	32.1046513	28.8266332	29.9103584	35.9664277	31.9672854	8.8861767
0.97252952	1.28258282	0.85201872	0.83513927	0.50206942	1.25855454	1.03495523
40.7673861	40.962489	47.0977012	41.7569637	45.186248	48.22781	89.4531716
22.2630407	23.0864908	19.028418	21.1259305	18.9873527	20.7409788	16.4556192
2.47075067	3.08621492	2.17738116	3.24776384	1.78006431	1.81231854	2.17676119
0	0.04008071	0.09466875	0.03093108	0	0.02517109	0
36.6669914	33.1467499	35.9741235	33.9004683	39.572199	37.8824917	15.4509785
0.44683789	0.72145284	0.75734997	0.68048385	0.27385605	0.57893509	0.40132908
11.8543463	10.0201783	9.8455496	11.0423971	9.95010309	8.73436852	8.80464317
29.0707473	30.9022299	31.9507018	27.2502852	30.0785226	30.1046246	53.4764167
3.23300354	4.5291206	2.60339052	3.12403951	2.96677386	3.27224181	1.15034486
24.313238	29.2990014	30.3886675	25.9511797	27.0204634	26.5303297	48.917806
2.73359649	3.28661849	2.50872177	2.7528665	2.14520571	2.76881999	2.32785822
0.60454538	0.48096856	0.42600936	0.55675952	0.5477121	0.67961945	0.54537718
4.91521677	4.16839418	3.88141859	3.00031517	4.60991015	4.30425653	6.64755367
12.4588917	10.340824	12.4962745	11.2279836	15.4728667	11.3773331	11.1817893
102.325876	98.7187968	115.117195	112.12518	100.68774	119.99059	196.043442
36.2464381	34.5094941	34.506758	33.034398	39.6178417	38.2097159	47.5493126
13.7731208	15.511236	13.4429619	14.1973677	12.5517356	13.3910203	8.44111014
0.68339912	1.2024214	1.60936868	1.02072578	1.14106687	0.80547491	13.818051
37.5606672	36.7540141	32.7553862	36.4058861	31.8129443	33.6034063	46.8134601
21.0539499	27.2949657	21.8684804	24.7757985	29.3482399	20.8668343	10.6403581
280.745617	285.494921	253.380899	264.801012	252.997346	260.29425	535.775318
0	0.04008071	0.09466875	0.03093108	0	0.02517109	0
175.922705	169.741821	163.918934	188.8652	161.985853	187.096718	155.101733
26.3634354	28.4172257	27.8326114	27.4049406	28.6179571	30.6835597	23.0110612
35.0110628	28.4172257	35.9741235	28.0235623	36.7423532	29.097781	24.8425276
9.12074983	9.13840262	9.75088085	9.55770503	9.17417762	7.4254718	4.87004756
1.13023701	1.72347067	1.13602495	1.82493397	0.41078407	1.05718582	0.52993572

55.039914	49.9405687	59.0732976	50.4795295	52.9455027	52.7082642	107.4693
2.1553357	1.64330924	2.41405303	2.07238264	2.46470444	2.36608254	2.72797767
50.4926813	57.7963885	52.1151447	52.7684297	55.1363511	44.3514621	15.3917153
14.8507886	15.230671	12.8749495	13.578746	16.979075	15.3543654	6.6205361
14.3776662	10.8217926	13.9163057	14.7231961	12.7343062	14.3223507	21.2031191
0.07885374	0	0	0.06186217	0.09128535	0.02517109	0.29319339
7.91165908	8.93799906	10.5082308	8.38232383	9.40239099	7.65201161	5.51937043
35.1950548	31.3030371	30.6726737	31.0238775	32.4062991	29.1732943	43.4884771
0.39426872	0.20040357	0.14200312	0.40210409	0.3651414	0.22653982	1.18066861
70.8632321	73.107221	70.0075378	73.7087737	65.2233822	65.117612	68.4182702
54.3302303	58.4777606	56.0912321	58.5216114	54.6799243	47.371993	16.2100904
44.4472276	42.1248296	43.4056201	47.4792143	38.5680601	37.4294121	24.0529259
20.0551358	19.5994688	16.23569	19.3937898	14.7882266	18.2490409	12.0113353
17.8209464	18.1164824	24.235199	19.5484452	21.9997692	22.3519287	35.1331205
0.10513833	0.08016143	0.18933749	0.12372434	0.13692802	0.02517109	0.08710746
4.38952514	3.80766776	6.76881535	5.2892154	4.7011955	6.3179438	8.73822858
89.8669847	89.7807977	83.7345059	85.8646898	85.8538712	80.3964641	61.5353815
0	0.04008071	0.04733437	0	0.04564267	0.07551327	0
22.4733173	23.1666523	22.2471553	21.8373455	22.6844093	20.0110172	10.6164426
36.0098769	37.3552248	36.6841391	37.457543	40.2111964	37.7566363	22.3322444
50.2824047	54.9907386	45.4409981	54.2840528	49.1571607	38.7634799	28.1251601
154.868755	156.354862	116.395223	138.973361	141.172793	123.288003	75.8401391
0.15770749	0.24048428	0.23667187	0.18558651	0.27385605	0.30205309	0.13177681
23.8926847	25.0103651	21.5844741	25.0541782	23.7341909	17.6197636	17.6175825
150.1901	140.162254	146.783891	143.458369	147.836623	123.741083	95.3179441
0.15770749	0.12024214	0.04733437	0.12372434	0.09128535	0.05034218	0.24328026
43.0541447	39.4795026	35.1694392	39.8083054	35.3730729	36.8253059	33.891571
7.54367493	7.73557766	6.95815284	6.09342359	7.30282796	7.37512961	15.8967553
9.09446525	10.1805012	8.94619651	8.47511708	8.90032157	9.03642161	3.29584519
2.81245024	3.00605349	2.50872177	3.00031517	3.19498723	2.97018872	2.41518355
0.21027665	0.12024214	0.14200312	0.27837976	0.09128535	0.22653982	9.1867262
874.120047	892.958211	772.73364	809.652061	898.430409	670.633373	224.242043
21.3430803	20.3610023	20.4011148	21.7445522	19.4894221	24.3404448	41.4094779
0.10513833	0.12024214	0	0.09279325	0.04564267	0.17619764	0.06250992
114.863622	105.773002	90.7399932	117.105085	96.123473	115.912873	78.2790959
25.1543446	23.3670558	22.5311616	23.5694862	20.5848463	23.2329168	33.6478287
18.1889305	19.2788231	21.1111304	19.6103074	17.116003	18.4755807	29.2573976
50.2824047	50.1008916	50.7424479	54.0366041	50.9828677	53.2620282	60.9912992
0.84110661	1.48298639	1.18335933	1.23724337	1.09542419	0.70479054	0.55392373
3.60098769	4.28863632	4.02342171	3.5261436	3.46884328	3.87634799	8.17260707
109.396429	119.640929	144.985185	95.0821529	144.230852	110.601773	110.918392
0.13142291	0.04008071	0	0.09279325	0	0.02517109	0.59876241
30.910668	29.77997	29.1106394	30.6217734	28.207173	26.7316985	14.6135517
62.9252885	60.3214735	71.0015596	68.0174542	67.779372	67.8612609	161.713401
0.21027665	0.12024214	0.14200312	0.09279325	0.13692802	0.15102655	0.46139611

36.9298372	41.8041839	37.1574829	42.7467584	38.2942041	40.449943	25.2590026
87.6327952	90.1014434	87.3319183	78.8124026	83.2978814	91.2703754	20.8966682
268.786132	278.240311	237.855225	248.005433	283.623581	208.920054	108.265432
0.05256916	0	0	0	0.04564267	0	0.12784708
0.05256916	0	0	0	0.04564267	0	0.04079105
9.14703441	8.81775692	8.47285278	7.76370214	8.26132413	8.75953961	4.14040056
23.6035543	20.401083	24.3298678	24.3118322	22.6844093	24.9697221	90.9413557
14.4302353	15.8719625	14.6736556	14.1664366	16.3400776	17.1666839	6.81424219
1.34051366	1.2024214	0.85201872	0.86607036	2.37341909	0.90615927	1.24229593
0.81482203	1.44290568	1.2306937	0.74234602	1.46056559	1.78714745	0.94884321
10.4349789	10.060259	9.6562121	9.34118744	10.1326738	8.91056616	9.2533208
44.6312196	48.8183088	44.9676544	44.1695883	44.3646798	43.772527	32.4497556
1.15652159	0.64129141	0.94668746	0.95886361	1.18670954	1.05718582	1.41334249
14.0622512	13.3869582	13.3482932	13.3312973	13.053805	12.1324658	15.3727836
35.4316161	26.8139972	29.44198	36.344024	34.6884328	26.7568696	21.6390286
0.2891304	0.56112999	0.28400624	0.34024193	0.27385605	0.70479054	0.25454405
58.9037475	74.229481	75.4036563	60.3465453	65.6798089	71.3348714	222.39533
4.91521677	4.60928202	5.49078728	5.53666408	5.0663369	5.53763998	10.037917
4.8626476	3.88782919	4.35476232	4.63966263	3.05805921	4.22874326	3.05987922
0	0.08016143	0.04733437	0.03093108	0	0.02517109	0
5.38833924	5.1303313	7.81017156	5.59852625	5.84226237	8.45748652	3.36299798
9.69901063	6.69347911	7.99950905	6.80483853	13.5102317	6.94722107	9.3791754
0	0.08016143	0	0.09279325	0.04564267	0	0
0	0.08016143	0.04733437	0.03093108	0	0.02517109	0
81.1667881	80.0011037	90.59799	85.6481722	77.7294751	77.023538	67.2469602
57.799795	59.1992135	53.7245134	55.6450205	53.4932148	53.8157922	22.6765998
0.78853745	1.08217926	1.32536245	0.89700144	1.23235222	1.43475218	0.65066189
0.05256916	0	0	0	0.04564267	0	0.03370392
58.7197554	61.5639756	52.9198291	61.2435468	55.9579192	47.1706242	15.4558391
0.05256916	0	0	0	0.04564267	0	1.71485556
0	0.08016143	0.04733437	0.03093108	0	0.02517109	0
0.15770749	0.04008071	0.09466875	0.06186217	0.22821337	0.15102655	3.97775773
76.0675793	87.8168428	71.2855658	81.8127178	78.8705419	85.8585908	14.577011
3.1541498	5.29065415	3.92875296	4.60873155	2.69291781	3.70015035	12.3272364
43.290706	37.876274	39.4768671	41.880688	39.0244869	33.9054594	20.9605712
113.391685	100.161702	83.3084966	129.663105	88.546789	98.7461893	19.5780351
3.62727227	3.00605349	2.93473113	3.64986794	3.69705665	3.22189963	1.46272911
225.653133	216.716417	219.489488	248.005433	210.23016	238.269546	405.044982
0	0.08016143	0.04733437	0.03093108	0	0.02517109	0
1.26165992	1.88379352	1.18335933	2.38169349	0.45642675	2.24022708	0.8826823
7.12312163	5.53113843	5.96413101	5.44387082	6.84640121	5.08456035	8.89143857
20.7911041	22.645603	22.8625022	21.8373455	22.1823399	25.7500259	32.6069891
1.26165992	1.5230671	1.98804367	1.67027855	0.95849617	1.45992327	1.10004989
32.8031579	27.1747236	30.8146769	30.6527045	29.3482399	35.8939755	25.8470982
54.3302303	55.0308193	50.6004448	54.6242947	51.9413638	45.7610431	18.8596398

0.81482203	0.24048428	0.80468434	0.37117301	0.86721082	1.03201472	0.24346844
0.44683789	0.52104927	0.7100156	0.24744867	0.27385605	0.78030382	0.43554598
71.8620462	75.1513374	86.148559	76.8018821	72.4805675	73.1220189	70.3262264
0.13142291	0.20040357	0.09466875	0.21651759	0	0.07551327	0.1259935
0.49940705	0.40080713	0.18933749	0.34024193	0.63899745	0.42790854	0.3659283
0.05256916	0	0	0	0.04564267	0	0.02580023
11.9594846	10.8618733	10.7922371	11.1351903	11.365026	9.13710597	5.55529199
0.05256916	0	0	0	0.04564267	0	0.03760648
5.62490047	4.64936274	5.20678104	5.38200866	5.0663369	3.87634799	3.2086071
0	0.08016143	0.09466875	0.12372434	0.04564267	0.05034218	0
833.773214	822.335994	958.852395	756.698045	889.621373	812.623496	894.294165
0.05256916	0.04008071	0	0.03093108	0.13692802	0.02517109	0.05711109
0.36798414	0.12024214	0.61534685	0.37117301	0.13692802	0.32722418	0.61664327
62.8464347	52.0247658	58.1739445	67.1204528	54.1778549	60.9895531	46.4245883
0.21027665	0.12024214	0.14200312	0.18558651	0.04564267	0.12585545	0.18076482
16.0335948	16.5934153	13.2536245	17.9709599	14.5600132	16.2605247	8.05820067
16.2438715	15.6715589	17.0403743	16.8574409	14.0123011	15.253681	14.0430309
0.73596829	1.2024214	0.99402183	0.9897947	0.86721082	0.67961945	0.89895074
8.77905027	6.73355983	8.6148559	8.44418599	6.61818784	7.37512961	23.8385858
51.1235113	48.6579859	50.0324323	49.953701	44.2277518	48.6053764	43.6722336
101.405916	107.296069	94.4794086	103.062373	95.8952596	89.8104521	52.4517395
5.28320091	5.29065415	4.16542483	4.42314504	3.4232006	5.53763998	8.63441801
46.8391245	46.4936274	48.849073	51.3146687	50.2069422	47.4475062	58.2588828
24.3395226	23.3670558	21.5371397	23.6622794	23.3234068	25.9262236	17.3965408
13.4577058	12.1444561	12.2122683	14.7541272	11.6845247	13.7937578	7.74164747
21.1065191	24.0083472	22.9098366	21.1259305	21.0869157	22.2764154	33.509791
141.805318	171.104565	179.491943	146.334959	156.554374	163.058326	278.664028
22.1579023	20.962213	19.4544273	20.1052047	28.2528157	18.8028049	13.9574849
1.81363613	1.88379352	0.99402183	1.39189879	1.04978152	1.68646309	2.07151669
7.85908991	7.45501267	5.77479351	7.82556431	7.66796935	7.29961634	10.3042969
0.18399207	0.04008071	0.23667187	0.06186217	0.09128535	0.17619764	0.37233216
0	0.12024214	0	0	0	0.05034218	0
41.9239077	41.9645068	42.127592	39.3443391	65.6798089	31.8414299	34.769423
10.4086943	12.8258282	11.6915901	11.2279836	10.7716712	10.7228847	8.27073888
16.5330019	17.3549488	17.8450586	16.3006814	17.1616457	15.505392	13.7022884
10.6715401	11.2626804	9.41954024	8.19673732	9.81317507	11.2263065	2.25424589
37.9023667	37.1949019	37.1574829	36.4058861	33.9125073	36.7497926	50.1247644
4.70494011	5.05016987	4.40209669	3.24776384	3.6057713	5.89003526	7.67397861
196.503532	208.219305	172.486455	245.530947	134.919747	154.651182	32.2852565
54.5142223	52.3053308	50.884451	54.4696393	48.0160938	47.8754148	55.8746925
163.253537	165.493265	168.605037	171.017965	154.454811	146.822973	92.1789279
1.83992072	2.1242778	1.98804367	1.73214072	2.19084839	1.48509436	40.9226894
6.15059211	6.89388268	9.60887773	6.83576961	7.07461458	10.6222003	2.69771558
0.5782608	0.52104927	0.18933749	0.43303518	0.22821337	0.90615927	0.44252607
14.009682	13.9080075	13.4429619	12.9291932	12.8255916	13.290336	27.5749601

22.7361631	21.2828587	20.2591117	22.8271402	18.1657845	19.7593063	30.0192267
2.94387314	4.16839418	3.02939988	2.31983132	3.33191526	3.49878163	2.02087355
21.4482186	24.9702844	25.6078958	23.0745888	27.0661061	17.4435659	29.4140023
53.8045386	68.4578582	45.67767	57.6864721	54.5429963	45.6603588	12.2521035
64.3446559	74.1493195	69.9602034	63.3777916	73.5759917	61.4929749	33.3524873
18.9248988	18.7176931	21.1111304	20.1361358	16.705219	18.5510939	17.604981
0.02628458	0	0.09466875	0	0	0.05034218	0.20350343
16.1124485	16.7537381	20.0224398	15.9604395	16.0662215	17.6701058	51.1951196
0.2891304	0.28056499	0.28400624	0.43303518	0.3651414	0.25171091	0.10063706
1.02509868	0.76153355	0.99402183	0.71141494	0.68464012	1.00684363	1.09184861
9.93557186	9.0582412	8.47285278	9.00094551	9.85881774	6.89687889	11.2850871
0.2891304	0.56112999	0.5206781	0.34024193	0.68464012	0.57893509	0.70879551
7.91165908	7.89590051	8.04684342	7.33066696	8.71775087	6.34311489	3.48890268
33.6442645	37.876274	33.2287299	32.6322939	38.9788442	27.5371734	11.1990567
12.5377454	13.7877654	14.1056432	12.2487094	14.1035865	11.7045572	2.17827573
10.5401172	11.182519	9.23020275	13.578746	10.0413884	9.66569888	4.4445261
3.52213394	4.64936274	4.30742795	3.80452336	3.87962735	3.72532144	7.41579102
10.5926864	9.61937118	11.170912	10.4547065	8.71775087	14.5488905	11.4367849
0.10513833	0.08016143	0.23667187	0.12372434	0.22821337	0.17619764	0.079736
6.96541414	7.49509338	7.33682782	6.55738986	6.93768656	6.8717078	5.95435957
38.8486117	41.6438611	43.0269451	42.3446543	40.2568391	35.0129874	29.4715874
0.26284582	0.76153355	0.66268122	0.09279325	0.68464012	0.553764	0.17993028
61.5847748	70.1412482	66.6467973	63.4705848	67.4598733	58.1200487	28.8035741
1.97134362	1.5230671	1.08869058	1.7630718	1.87134966	1.6361209	1.49273638
172.137725	168.058431	179.34994	193.257414	169.10611	184.755807	133.995256
153.160257	138.438784	144.843182	138.416602	148.11048	173.98258	174.938083
0.02628458	0.04008071	0.04733437	0	0	0.05034218	0.60100078
30.6741068	25.5314143	29.8679894	29.1061503	29.2569545	32.1686541	126.128347
21.0276653	19.1986617	20.8744585	22.9817956	19.3524941	22.0750467	10.539985
1.05138327	0.88177569	1.42003119	1.08258795	0.7302828	1.05718582	0.64630235
13.6416979	12.7055861	14.0109744	12.4342959	11.5932394	13.8441	28.5748084
11.2498009	14.0282496	17.2297118	12.124985	13.464589	13.869271	25.9166023
3.04901147	2.60524636	2.08271241	2.22703806	2.09956304	4.37976981	1.93454919
10.6452556	12.1043754	10.6028996	11.7228809	11.8670954	11.8052416	8.37170323
0.31541498	0.32064571	0.09466875	0.15465542	0.27385605	0.20136873	0.61002767
0.36798414	0.44088785	0.56801248	0.27837976	0.50206942	0.37756636	0.54696183
0.76225287	0.68137213	0.66268122	0.77327711	0.1825707	1.53543654	0.47647047
2.89130398	1.92387424	3.12406862	3.09310842	2.51034711	3.12121526	11.4277531
0.31541498	0.40080713	0.42600936	0.21651759	0.1825707	0.47825073	1.06881579
88.2899098	84.9711121	90.2193151	82.1529597	85.8082285	84.6503785	53.020985
1.20909076	0.48096856	0.99402183	0.9897947	1.00413884	1.13269909	2.37600469
27.9142257	36.2329648	28.542627	27.0646987	30.169808	30.5828754	57.7296048
0.18399207	0.24048428	0.33134061	0.21651759	0.59335477	0.17619764	0.11667952
8.06936657	9.61937118	8.89886214	5.84597492	8.17003878	10.3956605	3.27890294
1.44565199	0.96193712	1.13602495	1.11351903	1.27799489	1.6361209	1.82690578

1.89248988	2.04411638	2.03537804	1.36096771	2.37341909	2.97018872	2.39158574
0.02628458	0.04008071	0.04733437	0	0.04564267	0.02517109	0.05690892
0	0.04008071	0.09466875	0	0.04564267	0.02517109	0
36.693276	32.2649742	34.6487611	39.3752702	32.3606564	37.1525301	56.7104791
31.4363597	35.0706241	37.2048172	36.4368172	31.2652322	30.6583886	20.0865165
0.13142291	0	0	0	0	0.07551327	0.20479963
0.02628458	0.04008071	0.04733437	0	0.04564267	0.02517109	0.02470499
27.3885341	29.8601314	32.0453706	28.6112529	32.5888698	32.6720759	23.0267975
30.595253	27.4152079	36.4001329	30.5289801	31.2652322	37.8573206	59.3603846
0.7096837	0.88177569	0.99402183	0.89700144	1.41492292	0.70479054	0.24055019
58.11521	54.50977	66.741466	60.8105116	64.3561714	62.9277271	130.085052
100.696232	105.732922	112.040461	89.9475929	114.15233	98.6706761	48.457993
3.70612601	3.96799061	3.12406862	3.24776384	2.73856048	3.82600581	3.99969067
241.345029	252.829139	212.057991	258.955037	254.09277	230.844074	53.2576567
1.41936741	1.48298639	1.46736556	1.02072578	0.82156815	1.88783181	2.5127225
16.1913023	18.316886	14.9103275	15.1562313	16.6595763	14.9264569	7.06999167
157.523498	169.220771	167.043003	159.387877	167.326046	142.493545	48.3857314
0.02628458	0.04008071	0.04733437	0	0.04564267	0.02517109	0.03734442
188.828435	200.644051	173.101802	172.657312	218.902268	134.640165	91.572267
37.5606672	38.998534	36.6841391	36.9007835	35.6012863	35.6674357	21.7657087
51.2286496	49.900488	50.7424479	56.8822639	44.1364665	42.941881	33.4047189
16.3490098	14.4691375	13.5376307	15.2799556	15.7467228	16.1094981	18.1401387
5.04663968	5.93194556	4.49676544	4.97990456	4.5186248	4.80767835	2.51107586
128.295043	127.01578	144.748513	118.930019	129.807767	132.047543	115.313326
0.21027665	0.24048428	0.14200312	0.18558651	0.1825707	0.12585545	0.1023387
0.07885374	0.28056499	0.47334373	0.34024193	0.09128535	0.15102655	0.06317002
88.1322023	88.4982149	87.3792527	85.9884142	85.1235884	82.0829272	39.8561276
0.07885374	0.24048428	0.09466875	0.18558651	0.1825707	0.15102655	0.0466444
8.5950582	8.05622337	9.89288397	9.03187659	7.07461458	8.53299979	7.88467051
1.20909076	0.96193712	1.18335933	1.33003662	0.86721082	0.75513273	0.16017575
79.2480137	62.766397	59.2626351	62.8828942	74.3062745	75.7649834	50.307695
1.97134362	1.24250211	1.08869058	0.9897947	3.28627258	1.00684363	2.65001717
28.6239094	26.2929479	23.5251834	31.7662235	29.9872373	21.8736779	14.7587806
37.6132363	39.9604711	41.5122452	38.7257175	38.1116334	36.8756481	24.6711625
23.8926847	29.4192435	22.6258303	21.0022062	26.244538	24.5921557	47.9140482
0.10513833	0.08016143	0.04733437	0.06186217	0.13692802	0	0.07274557
74.0173819	64.8505941	63.5227287	71.60546	71.3395006	69.3211842	61.0548708
0.18399207	0.32064571	0.28400624	0.34024193	0.13692802	0.12585545	0.05403308
33.2762804	30.7419071	35.5954485	36.0965753	33.4560806	34.8367897	35.8330843
28.3084944	30.1807771	33.2287299	31.3950505	28.8918131	26.8575539	26.4043953
441.738679	449.585361	474.811096	437.241807	464.870642	390.730843	382.429173
11.5126468	10.5412276	10.0348871	11.9703296	9.49367634	8.55817088	8.25754984
0.94624494	0.84169498	0.94668746	0.74234602	1.14106687	1.23338345	1.06341525
78.3806225	65.8526119	81.1784498	73.5541183	62.5304644	77.7283285	84.2917462
0	0.04008071	0.09466875	0	0.04564267	0.02517109	0

0.05256916	0	0.04733437	0.03093108	0.13692802	0.02517109	2.00959636
5.80889254	5.85178414	6.29547162	7.20694262	4.38169677	7.80303816	9.91064534
8.48991987	7.29468981	7.28949345	6.18621685	8.21568145	10.6222003	9.66551323
5.4409084	4.00807133	3.88141859	4.23755854	3.24062991	4.70699399	3.0728454
14.7193657	12.6655054	15.4310056	14.4138852	11.3193833	14.2971796	18.9039195
11.8806309	11.9440526	10.6502339	12.9910554	11.1368126	12.45969	10.1278055
0.42055331	0.52104927	0.28400624	0.43303518	0.3651414	0.62927727	0.2046367
0.02628458	0	0.09466875	0.03093108	0	0.02517109	0.02069082
84.9780525	85.0913543	100.064865	84.101618	94.434694	105.038962	116.931834
2.97015773	3.3266992	1.84604055	3.9282477	2.19084839	2.99535981	3.01648004
10.7766785	11.3027611	10.6028996	11.8775363	11.8214528	10.9997667	5.5777246
2.20790486	1.40282496	2.36671865	1.85586505	3.14934456	1.83748963	2.47675149
372.215961	328.341203	308.099434	349.490321	394.717851	323.196806	158.236769
0.05256916	0.08016143	0.04733437	0	0.1825707	0.10068436	0.18399738
43.6324055	52.6259765	41.8435858	44.076795	47.4683817	39.896179	26.6341713
54.3565148	60.0409085	52.6831572	41.7878948	57.8749116	58.1452198	13.9297212
17.5055314	18.0764017	18.1763993	16.6099922	15.4272241	18.6266072	20.126183
140.359666	162.166566	170.829752	144.479094	156.417446	148.257725	199.303439
5.33577007	5.37081558	4.49676544	4.63966263	3.9709127	5.36144235	12.166205
25.3909059	25.5314143	26.2705771	22.9817956	26.2901806	24.3656159	24.534219
65.5800312	63.7283341	60.4933288	69.7495949	65.2690249	63.7080309	36.3823522
130.476663	147.336702	148.771935	132.106661	132.683255	140.98328	127.841555
76.6458401	90.9030577	91.3080056	74.0180846	90.8289227	81.755703	86.1762202
41.2667932	47.1349188	41.0862358	42.1281367	45.7339601	35.2898693	19.1979694
125.35117	131.985789	148.014585	145.685407	128.894913	151.983046	162.436695
0.2891304	0.52104927	0.7100156	0.49489735	0.5477121	0.65444836	0.21712029
0.13142291	0	0.04733437	0.06186217	0.13692802	0.10068436	3.53335623
77.6709388	76.7946466	80.3264311	78.9361269	81.0613903	84.8517472	81.9083756
58.5357633	67.095114	63.9960724	62.078686	53.5845001	64.5890191	27.1646196
49.5990056	49.8203266	47.713048	49.4588037	52.9455027	51.6762495	16.7862037
52.2274637	57.9967921	63.0493849	48.4071468	58.4226237	57.6669691	62.361237
14.5353737	15.9521239	15.4310056	14.2282987	15.7467228	13.4161914	4.91253172
0.13142291	0	0.14200312	0.21651759	0	0.15102655	0.1013029
64.1869484	67.3355983	56.5172414	62.6973077	65.3603102	50.4932082	27.4589784
3.81126434	3.40686063	2.41405303	3.55707469	3.51448595	3.59946599	5.13748049
6.75513748	6.53315626	6.29547162	7.08321829	5.20326492	8.80988179	14.7693659
2.44446609	2.52508494	1.65670306	1.97958939	1.96263501	2.08920054	4.89407427
0	0.04008071	0.09466875	0	0.04564267	0.02517109	0
37.6920901	34.3090906	34.3174205	38.199889	37.7008493	35.6422646	18.4839362
11.0395243	11.6234068	8.85152776	10.3928443	12.4148075	11.0249378	23.7545954
54.1725228	51.303313	56.0912321	53.1086716	51.074153	50.1408129	15.0115414
0.02628458	0.04008071	0.14200312	0.06186217	0.13692802	0.07551327	0.04239096
7.09683704	5.49105772	5.72745914	6.95949395	6.66383051	6.21725944	10.8518203
78.0389229	75.3517409	77.5337031	73.2138764	74.7170585	73.6002696	89.5113598
47.2596778	55.912595	47.0503668	44.2005194	54.9994231	43.2942762	19.0988001

5.07292426	4.32871703	5.39611853	4.60873155	4.19912608	4.80767835	4.96848162
12.4063225	10.5412276	11.5969214	13.2694351	12.7343062	10.8739112	9.96158298
16.5330019	20.1605988	18.0817305	15.5583354	19.4437794	16.6632621	15.5693439
28.9130398	26.97432	32.8027205	29.4463922	27.7963889	36.4225684	18.4645763
124.168364	143.208388	137.03301	130.498244	139.484014	114.075384	66.0980552
9.54130314	9.45904833	9.56154336	9.37211852	8.58082285	8.86022397	22.7741183
3.78497976	3.60726419	2.93473113	3.83545444	3.51448595	3.92669017	4.18635624
1.78735155	2.32468137	1.70403743	2.53634891	2.05392036	1.86266072	3.74711368
12.6428838	11.0221961	16.1410212	14.1973677	14.2405145	13.9447843	31.5629664
746.666111	749.06845	733.209439	802.537911	711.204158	847.359602	1030.90932
16.1913023	16.0322853	15.47834	16.1150949	14.4230852	14.6244038	27.1695222
546.798152	612.874186	668.59802	589.144361	666.839478	455.798113	71.4893526
194.111635	198.840418	198.757032	196.690765	189.097601	236.834794	462.87311
0.47312247	0.48096856	0.23667187	0.21651759	0.63899745	0.20136873	0.23053724
1.18280617	0.88177569	0.42600936	1.1753812	0.13692802	0.70479054	0.71896118
32.0671896	24.5694772	26.8385895	33.0962601	28.3897437	26.882725	17.5317635
23.3144239	23.6476208	23.6198522	24.0334524	21.9084839	21.2192296	28.8812066
74.5430736	68.1372125	75.9716688	71.8219776	63.1694618	73.4995852	66.3664077
7.38596744	7.53517409	7.52616532	7.29973588	7.30282796	6.46897034	4.13602436
10.0669948	9.09832191	11.0762433	9.89794695	8.39825215	9.91740979	7.49988213
12.4851763	14.9100253	19.2177555	10.5474997	14.7882266	17.5190792	17.9787314
224.838311	224.091268	239.275256	219.610698	228.898014	207.334275	179.883189
7.14940621	8.13638479	7.62083406	7.94928865	6.38997446	7.07307652	21.5678504
45.6300337	47.2952416	48.6597355	48.6236644	45.7796028	52.8844618	79.7407834
0.13142291	0.28056499	0.33134061	0.15465542	0.3651414	0.10068436	0.41866591
7.14940621	7.45501267	10.0348871	8.38232383	8.3069668	9.53984343	23.384394
0.02628458	0	0	0	0	0.05034218	0.02903254
0.26284582	0.48096856	0.04733437	0.27837976	0.31949872	0.37756636	0.82656919
0.02628458	0.08016143	0	0.03093108	0.04564267	0	0.02975802
0.68339912	0.6012107	1.13602495	0.92793253	0.91285349	0.85581709	2.23826528
1.05138327	1.2024214	1.51469994	1.23724337	1.50620827	1.35923891	3.7011271
0	0.04008071	0	0	0.04564267	0.02517109	0
0.2891304	0.40080713	0.42600936	0.34024193	0.31949872	0.276882	0.23809422
0.44683789	0.32064571	0.14200312	0.12372434	0.45642675	0.22653982	1.59361577
3.73241059	4.5291206	5.25411541	4.45407613	4.92940887	4.98387599	3.78433727
0	0.08016143	0.04733437	0.03093108	0.09128535	0.05034218	0
1.39308283	1.24250211	1.13602495	0.9897947	2.14520571	1.23338345	5.2114303
28.6764786	39.1187761	35.784786	31.0548086	33.5930086	33.9306304	71.88425
59.5345774	65.0109169	63.9960724	60.3465453	68.5096548	67.6850633	111.870617
0.02628458	0	0.09466875	0	0.09128535	0	0.12179372
0.18399207	0	0.18933749	0.06186217	0.22821337	0.22653982	0.20859573
1.07766785	1.24250211	1.13602495	1.29910554	1.64313629	0.95650145	0.60458391
7.80652075	6.29267198	7.00548721	6.58832094	8.3069668	7.77786707	25.6365151
0.52569163	0.52104927	0.61534685	0.5876906	0.7302828	0.57893509	2.53676857
35.247624	43.5276546	30.4833363	35.632609	37.746492	29.8529137	21.4837566

35.8258848	28.0564993	31.9507018	34.5809522	33.4104379	31.9672854	32.094674
15.0873499	16.1926082	14.9103275	16.9502342	16.9334323	14.6495749	16.9012206
4.91521677	4.56920131	5.15944666	3.64986794	3.92527003	5.91520635	11.6300044
0.02628458	0.08016143	0	0	0	0.05034218	0.08506228
75.331611	86.8549056	88.6099464	75.9976739	81.5178171	83.8449036	122.055696
4.4420943	5.33073486	6.05879975	4.7015248	4.56426747	5.28592908	10.530908
6.83399123	5.97202628	6.91081847	7.79463323	6.80075853	6.46897034	8.95066471
0	0.20040357	0.04733437	0.15465542	0.04564267	0.10068436	0
0.02628458	0.04008071	0.14200312	0.06186217	0	0.05034218	0.07193186
0.18399207	0.12024214	0.33134061	0.30931084	0.1825707	0.22653982	0.48929303
5.28320091	5.69146128	5.49078728	5.1036289	5.79661969	6.54448362	3.46999295
21.8687719	22.0443923	19.1704211	22.394105	20.2197049	17.3680527	13.0052244
0.39426872	0.32064571	0.56801248	0.30931084	0.7302828	0.47825073	0.80250104
25.5748979	27.8560957	25.5605615	25.0541782	26.0619673	24.5921557	39.2293367
0.07885374	0.08016143	0.14200312	0.06186217	0.13692802	0.17619764	0.13760432
0.18399207	0.52104927	0.42600936	0.09279325	0.31949872	0.45307964	0.23568041
0.05256916	0.04008071	0.09466875	0	0.04564267	0.07551327	0.13904234
0.10513833	0	0.23667187	0.03093108	0.04564267	0.15102655	0.26382393
0.02628458	0	0.09466875	0.09279325	0	0.05034218	0.06870415
0.42055331	0.28056499	0.42600936	0.34024193	0.31949872	0.30205309	0.83178119
0.02628458	0	0	0	0	0.05034218	0.07249953
0.05256916	0	0.09466875	0.06186217	0	0.02517109	0.04800827
0.02628458	0.04008071	0.14200312	0	0.04564267	0.07551327	0.0414083
0.34169956	0.44088785	0.37867498	0.55675952	0.13692802	0.60410618	0.20047772
0	0.04008071	0.09466875	0.03093108	0.04564267	0.07551327	0
0	0.12024214	0.09466875	0.03093108	0.09128535	0.02517109	0
0.05256916	0	0.04733437	0.03093108	0.04564267	0.07551327	0.0683028
0.07885374	0	0.28400624	0.06186217	0.09128535	0.10068436	0.16732112
0.10513833	0.16032285	0.14200312	0.09279325	0.27385605	0.15102655	0.12156348
6.75513748	7.93598123	6.53214348	7.23787371	5.29455027	7.19893198	6.55050354
0.02628458	0.04008071	0.04733437	0.06186217	0	0.07551327	0.04230729
0.02628458	0.08016143	0	0.09279325	0	0.05034218	0.06201262
0.05256916	0.04008071	0	0.03093108	0.09128535	0.05034218	0.05347782
0.84110661	0.52104927	0.33134061	0.49489735	0.59335477	0.45307964	0.65307098
0.02628458	0	0	0	0	0.05034218	0.10063706
2.44446609	2.60524636	2.79272801	1.26817445	3.33191526	4.32942762	2.09550061
0.05256916	0.04008071	0.04733437	0	0.04564267	0.05034218	0.069899
2.83873482	2.96597278	3.45540923	3.00031517	3.14934456	3.72532144	2.46457239
11.4075084	10.9420347	11.6915901	7.91835756	16.0205788	13.0386251	8.82646242
0.10513833	0.16032285	0.09466875	0.12372434	0.27385605	0.10068436	0.08483784
0.23656123	0.24048428	0.09466875	0.12372434	0.1825707	0.17619764	0.17591603
0	0	0.04733437	0.03093108	0.04564267	0	0
0.31541498	0.28056499	0.28400624	0.12372434	0.5477121	0.12585545	0.31003817
0	0	0.04733437	0	0.04564267	0.02517109	0
8.96304234	9.57929047	9.60887773	9.4030496	8.71775087	8.60851307	6.97922197

3.70612601	3.36677991	3.92875296	3.49521252	3.56012863	3.19672854	1.15654321
14.0622512	13.7476846	14.9576619	13.4859527	13.0994476	14.0202976	18.9450934
3.54841852	3.3266992	3.45540923	3.74266119	3.05805921	2.7436489	2.73604042
0.10513833	0	0.09466875	0.18558651	0.04564267	0.05034218	0.19082221
0.44683789	0.52104927	0.37867498	0.30931084	0.68464012	0.60410618	0.60465731
80.8513731	66.533984	64.4694161	73.5850494	76.2232668	71.5362402	67.2818328
51.5703492	47.5357259	46.5770231	50.9434957	46.6011709	54.6464382	35.9972889
29.7015773	28.2569028	29.0159707	29.9412895	29.2569545	31.0862972	12.1638775
18.846045	21.6435852	15.3836712	18.8370303	16.7965043	17.4939081	9.72336119
54.093669	55.7923529	48.5177324	62.078686	53.5388575	49.8387599	21.9257749
11.2235164	12.4651018	11.1235777	11.9393985	9.44803367	11.4780174	6.1622811
38.9274654	31.3030371	33.8914111	35.4160914	34.3689341	39.4934415	54.1745111
0	0.04008071	0	0	0.04564267	0.02517109	0
1380.99192	1345.14882	1525.87085	1277.36099	1468.96384	1299.83513	1638.55197
64.71264	60.0409085	51.8784729	58.6762668	60.9329708	65.8475736	24.383804
126.61283	145.613231	124.820742	123.600613	136.882382	118.706864	62.3650535
58.8248937	43.7681389	59.5466413	45.2521762	68.9660815	58.9003526	65.477367
1.89248988	1.72347067	1.37269682	1.63934746	1.68877897	1.28372563	2.54122393
26.4422891	26.6536743	32.3293768	22.4559671	35.2361449	33.2006688	21.0794806
130.844647	121.96561	124.58407	139.932225	127.845132	126.107165	116.747141
4.25810223	3.68742562	3.64474673	3.09310842	3.6057713	4.02737453	3.36925857
0.34169956	0.44088785	0.42600936	0.40210409	0.41078407	0.553764	0.35879489
58.6146171	50.3814566	56.5172414	52.4900499	48.9289473	73.2227032	17.6955572
42.7124452	37.6758705	31.7613643	42.9014138	38.2029187	36.8756481	30.7802682
441.370695	437.080178	507.708485	408.042863	499.787288	416.2795	791.094904
28.334779	23.5674594	23.856524	24.621143	30.0785226	16.2353536	14.2317873
83.5324005	77.8367452	76.0190031	80.0805771	76.9079069	71.6872667	59.2199104
33.5128416	40.962489	40.1868827	36.8079902	46.2360295	37.6559519	12.4229593
584.490242	578.525015	548.037371	611.600328	568.525156	612.739864	950.163862
0.13142291	0.24048428	0.18933749	0.12372434	0.22821337	0.30205309	10.7178472
35.3001932	35.7519962	33.2760643	34.147917	35.6012863	30.4066777	13.0518986
2.36561235	4.97000844	2.55605615	3.74266119	3.19498723	1.93817399	1.29015548
43.7112593	45.9324974	49.3697511	46.7987304	43.5887544	42.8160255	32.8649907
0.05256916	0	0.04733437	0.03093108	0.04564267	0	0.02292588
2.05019737	2.36476208	1.60936868	2.10331373	2.14520571	2.34091145	2.5604658
16.1124485	18.2367245	18.1290649	18.373064	17.5724298	14.120982	9.44197416
0.02628458	0.04008071	0.09466875	0	0	0.07551327	0.07442949
1.94505904	1.96395495	1.13602495	1.7630718	1.55185094	1.30889672	1.33185675
60.9013757	59.960747	58.4579507	55.2119853	57.4184848	59.4289455	132.798139
16.6907093	16.1124467	18.4130711	15.5892665	17.252931	16.1346692	41.2474727
85.8980128	93.1074969	94.2900711	92.9788392	89.8247839	79.7671869	22.3189835
165.776856	184.210958	149.576619	168.141374	210.686587	150.951032	32.3355477
54.6456452	48.8583895	55.6652227	55.5831584	51.4392944	58.5227862	77.7745354
53.7256849	50.3413759	46.0090106	54.1293974	52.1695772	50.2414973	58.3157402
92.7845732	101.203801	104.324958	93.0716324	96.7624704	96.0025404	104.851734

316.860632	287.13823	278.846792	315.52799	313.154391	295.030356	351.255954
15.9284565	14.3088146	13.9636401	14.6613339	16.1575069	15.7319318	7.85054241
155.420731	138.919752	142.33446	155.521491	143.135428	116.743519	63.1116654
6.09802294	7.2546091	5.86946226	6.12435468	6.48125981	5.58798217	1.49431524
48.3373456	43.2470896	40.1395484	46.0563844	45.7339601	45.156937	22.0924204
88.1059177	77.2756152	98.4081616	70.0279747	97.4471105	112.942685	51.4333914
1.78735155	1.92387424	2.03537804	2.04145156	1.82570699	2.29056927	1.35341432
7.22825995	7.29468981	7.43149657	6.68111419	6.43561714	7.37512961	4.5933631
98.9351653	115.232051	102.762924	103.093304	111.550697	86.6892368	29.4073456
173.898792	206.976803	180.911974	169.718859	205.255108	161.094981	48.25949
21.0276653	19.7597916	18.6970774	21.0949994	20.1740622	21.3954272	10.386769
0.02628458	0	0.09466875	0.03093108	0.04564267	0	0.01632159
32.6454504	28.9783557	32.2347081	35.9109888	24.8296151	38.4362557	57.2540484
1.78735155	1.60322853	1.60936868	2.01052047	1.68877897	1.78714745	1.37037971
7.46482119	6.49307555	6.81614972	6.12435468	7.66796935	8.48265761	10.6428972
1.65592864	2.32468137	1.2306937	2.19610698	1.91699234	1.6361209	1.31026722
18.4780609	19.7197109	18.1290649	18.744237	18.0288565	17.0911707	14.2836902
13.4839904	12.9861511	16.2830243	15.3727489	14.3317999	15.0271412	25.2406532
75.8047335	75.9128709	80.5157686	80.420819	71.4764286	70.9573051	50.0976846
0.2891304	0.44088785	0.04733437	0.27837976	0.1825707	0.47825073	1.35773113
1.23537534	1.56314782	1.46736556	1.33003662	1.14106687	1.33406781	0.83979798
0.2891304	0	0.23667187	0.21651759	0.22821337	0.25171091	0.59644006
0.47312247	0.32064571	0.56801248	0.71141494	0.3651414	0.45307964	0.34460479
0.05256916	0.04008071	0	0.06186217	0.04564267	0.05034218	0.06292278
0.05256916	0.04008071	0	0.03093108	0	0.02517109	0.05775221
22.867586	22.1646344	23.9511928	22.7034158	22.4105533	20.9171765	23.2628534
6.86027581	6.81372125	5.49078728	6.80483853	4.7011955	6.21725944	19.3812341
32.4351738	30.7819878	34.3174205	39.0659594	30.3980214	32.7979314	29.8439041
50.1772664	51.5037165	54.3398603	57.655541	48.9289473	56.3077302	32.9033026
372.426237	421.047893	412.519061	392.206148	433.103341	315.796506	86.9185409
3.70612601	3.3266992	2.88739676	3.15497059	3.19498723	4.37976981	1.74203626
14.5616582	14.9100253	13.064287	13.7334014	16.0662215	15.253681	5.69198341
0.39426872	0.28056499	0.28400624	0.09279325	0.22821337	0.45307964	0.35541866
60.9539448	62.5259127	57.9372726	64.4603795	69.3312229	55.4519131	13.1680465
64.8177783	62.4056705	52.9198291	64.5841039	66.7295905	56.53427	17.5635472
19.8711437	17.9561595	18.2237336	18.744237	19.1242807	21.0933741	14.3452803
44.7889271	48.8984702	46.5770231	46.7677994	50.9828677	48.4543499	133.308114
2.12905111	1.44290568	1.98804367	1.5774853	2.41906176	2.1898849	1.53427798
0.49940705	0.44088785	0.66268122	0.52582843	0.27385605	0.52859291	1.26483911
0.49940705	0.12024214	0.66268122	0.43303518	0.3651414	0.276882	0.76990207
2.70731191	2.00403566	2.03537804	2.3507624	2.32777641	2.69330672	4.06108497
0.2891304	0.68137213	0.37867498	0.24744867	0.59335477	0.32722418	0.60875896
79.5108595	78.7586016	88.3732745	85.1223438	75.2647706	77.1745645	112.509501
6.17687669	5.93194556	6.76881535	5.53666408	7.21154261	7.32478743	7.14185472
37.7972284	33.547557	34.6960955	36.8079902	34.0494353	30.5828754	18.9611618

58.9037475	58.5980028	47.0030325	59.1711641	63.8997446	49.2094826	32.7825691
0.02628458	0.04008071	0.04733437	0.03093108	0.09128535	0.05034218	0.01435426
416.190066	435.316627	511.779242	429.725553	451.999408	407.998211	393.142223
71.8094771	81.6844936	80.9891123	76.4616402	70.5635751	78.0555527	95.9513663
33.4602724	35.5916734	35.9741235	29.6010476	36.3315691	34.912303	55.1265435
33.0660037	28.2969836	32.613383	32.8178804	24.1449749	32.5462204	34.163138
0.10513833	0.32064571	0.18933749	0.27837976	0.1825707	0.25171091	0.10276801
28.1245024	27.5755307	29.7733207	26.3223527	26.2901806	29.5005185	86.4429387
17.2689701	16.3128503	16.6143649	18.8060992	17.7093578	16.0088138	6.56052077
55.6970285	54.1089629	60.3039913	63.7489646	51.1197957	63.3556356	37.8623811
3.49584936	4.24855561	4.49676544	3.74266119	3.788342	3.77566363	3.24709267
101.274493	100.883155	88.4206089	91.4632161	94.9367634	91.5975995	184.219474
32.2774663	32.2649742	38.0095016	28.7659083	40.6676232	28.267135	13.0333551
0.02628458	0	0	0	0	0.05034218	0.12683843
1.73478239	1.8036321	1.65670306	2.38169349	1.46056559	1.86266072	1.58901816
0.39426872	0.36072642	0.14200312	0.68048385	0.22821337	0.22653982	0.21828609
0	0.04008071	0.09466875	0	0.09128535	0	0
0.7096837	0.96193712	0.66268122	0.68048385	0.63899745	0.75513273	1.2996192
4.1529639	2.4048428	3.64474673	3.55707469	2.96677386	2.89467545	3.16231534
26.573712	25.8921408	29.3473113	28.2400799	27.9333169	29.1732943	23.071136
2.18162028	2.56516565	1.18335933	2.07238264	1.46056559	1.83748963	2.85885802
3.23300354	4.08823275	3.21873737	4.60873155	2.92113118	3.77566363	3.14378826
9.01561151	8.73759549	6.43747474	9.68142936	6.80075853	9.38881688	2.59711876
0.02628458	0	0.09466875	0.03093108	0.04564267	0	0.01741324
0.52569163	0.28056499	0.14200312	0.24744867	0.3651414	0.553764	1.42272308
0.91996036	0.72145284	0.7100156	0.86607036	1.09542419	0.78030382	1.64168338
11.7229234	9.85985546	10.6502339	11.2279836	11.09117	11.8807549	33.004556
0	0	0.04733437	0.03093108	0.04564267	0	0
194.532189	170.503354	216.365419	178.998184	202.927332	175.744556	169.432796
7.28082912	6.25259127	6.6741466	6.58832094	7.85054005	7.24927416	6.88294517
6.28201501	4.44895917	3.45540923	3.77359228	6.52690249	5.51246889	5.54251458
3.4432802	2.96597278	3.50274361	2.66007324	4.56426747	3.70015035	1.48001193
0.02628458	0	0	0	0	0.05034218	0.09319867
0.02628458	0	0.09466875	0	0.1825707	0.02517109	0.03830082
72.755722	75.8727902	74.1729626	71.4508046	68.0075853	74.9343374	127.326185
2.54960442	2.60524636	3.36074049	2.81472866	2.55598978	2.41642472	3.39933901
5.02035509	4.24855561	4.54409981	5.32014649	4.56426747	4.88319162	12.7281377
0.02628458	0.12024214	0.09466875	0.06186217	0.04564267	0.05034218	0.02539774
1.39308283	1.72347067	1.79870618	1.70120963	0.95849617	1.6361209	1.00509449
0.07885374	0.16032285	0.09466875	0.06186217	0.09128535	0.10068436	0.24670237
0.5782608	0.36072642	0.56801248	0.30931084	0.50206942	0.50342182	0.90111837
199.710251	209.02092	215.93941	208.846681	204.798682	185.2844	76.9214766
1.8662053	1.48298639	2.50872177	1.14445012	1.23235222	2.7436489	2.1773023
16.5067173	17.3549488	19.3124242	18.4039951	19.0329954	18.5259229	22.8291964
51.7017721	54.1089629	48.2337262	51.3146687	49.7505155	60.2595915	28.3996482

13.3262829	12.024214	11.3602495	12.8982621	11.0455273	11.1004511	4.9640212
17.4529622	14.950106	19.5490961	15.6820597	15.9292935	17.468737	39.3547496
0.02628458	0	0	0.06186217	0	0	0.03531416
8.38478155	7.29468981	6.76881535	8.0420819	7.21154261	8.63368416	5.88635856
66.5262762	76.6343238	61.2506787	74.3583265	73.530349	65.7720604	25.7941724
0.13142291	0.08016143	0.33134061	0.18558651	0.09128535	0.12585545	0.06889553
88.8681706	87.0152285	83.1664935	80.7301298	89.2314291	78.6596589	19.3401751
28.8604706	32.8261042	32.1400393	32.6632249	31.9042296	32.7979314	11.1635063
8.8841886	9.6594519	10.1295558	8.07301298	9.76753239	9.31330361	9.84410968
2.83873482	2.28460066	2.55605615	2.10331373	3.28627258	3.04570199	2.52919337
15.6918952	14.9901868	14.8629931	16.517199	16.2031495	15.0774834	18.5914425
28.7553323	30.2609385	27.7379426	27.8070447	22.6844093	31.9169432	66.2447733
147.561641	144.490971	177.598568	139.808501	147.288911	161.120152	309.888383
0.05256916	0	0.04733437	0	0	0.05034218	0.10322164
16.585571	19.6395495	17.7503899	19.4865831	14.2861572	17.2925394	14.6914445
1.62964406	2.00403566	1.89337492	1.7630718	1.64313629	2.61779345	6.46197596
0.02628458	0	0	0	0.04564267	0.02517109	0.0341514
0.02628458	0	0.14200312	0.03093108	0.04564267	0.02517109	0.05190241
0.10513833	0.12024214	0.09466875	0.15465542	0.09128535	0.15102655	0.05697195
4.02154099	3.44694134	3.31340611	4.45407613	4.65555282	2.86950436	1.06344399
107.635362	132.426677	112.419136	122.579887	114.061044	99.5264932	59.1769677
433.669313	464.69579	496.158898	477.730596	427.62622	415.448854	309.962772
12.6165992	12.9059897	13.4429619	13.0838486	14.6056559	13.3910203	22.2547945
0.02628458	0	0	0	0.04564267	0.02517109	0.1073574
57.5369492	60.3615542	58.6472882	64.7696904	52.0782919	52.1796713	17.1480333
146.483974	147.977993	150.617975	159.449739	149.068976	155.607684	112.522253
1.26165992	1.12225997	1.2306937	0.9897947	1.00413884	1.25855454	0.89496666
0	0.12024214	0	0.03093108	0	0.02517109	0
38.638335	34.4293327	34.8854329	37.1482322	32.8627258	33.8299461	51.0980608
46.5762787	47.5758066	44.8256513	45.7470736	44.5928932	43.0929075	30.4522052
4.54723263	3.60726419	4.87544043	4.17569637	4.42733945	5.43695562	7.76894233
13.8519745	14.9901868	13.3009588	12.6198824	15.01644	12.45969	16.8522292
18.3992072	19.8800338	21.1111304	17.8781667	20.8130597	18.022501	35.3335623
16.0073102	16.5533346	12.7329464	14.5685407	13.7384451	14.9012858	11.9108923
0.07885374	0	0	0	0	0.05034218	0.08769148
0.63082996	0.64129141	0.47334373	0.52582843	0.50206942	0.52859291	0.72086408
15.8233182	19.5593881	20.6377867	15.9913705	17.7093578	18.9538314	35.9117479
0.21027665	0.04008071	0.04733437	0.15465542	0.09128535	0.17619764	0.40254825
9.33102648	12.865909	16.7563681	12.6817445	7.02897191	15.3543654	15.8976425
0.89367578	0.64129141	0.61534685	0.77327711	0.1825707	1.61094981	1.41884545
44.2369509	38.0766776	37.8674985	47.757594	35.8751423	42.7656833	47.888859
0	0.08016143	0.09466875	0.03093108	0.04564267	0.02517109	0
176.632389	184.411362	203.537804	184.070882	174.674516	178.186152	180.737599
0.2891304	0.28056499	0.14200312	0.24744867	0.22821337	0.37756636	0.47666976
1.5770749	1.12225997	1.9407093	1.11351903	1.50620827	2.59262236	0.83119884

0.81482203	0.80161427	0.47334373	0.49489735	0.5477121	0.80547491	0.6921943
0.02628458	0.08016143	0.04733437	0.06186217	0.04564267	0	0.04356848
2.10276653	2.1242778	2.27204991	2.1651759	1.91699234	1.86266072	2.88372571
1.94505904	2.20443923	1.98804367	2.87659083	1.14106687	2.71847781	9.46068423
0.55197621	0.36072642	0.5206781	0.30931084	0.63899745	0.35239527	1.12725271
0.5782608	0.76153355	0.94668746	0.71141494	0.5477121	0.70479054	0.83220931
315.572687	320.966352	306.111391	339.283063	306.444918	340.615201	508.610568
202.62784	203.048893	212.436666	219.023007	200.50827	223.972366	165.027732
15.1136344	14.2687339	16.0936868	17.1358207	12.9168769	17.6197636	24.7998477
3.04901147	3.08621492	2.50872177	3.00031517	2.96677386	3.34775508	3.48092472
36.0098769	39.6398254	36.4948016	38.4473377	36.8792812	32.3196806	10.121864
291.680003	302.529224	285.00026	369.379008	295.034249	261.502463	79.4230055
28.3873482	35.5115119	35.7374517	28.1472866	33.1365819	33.1251555	45.9032717
14.2199587	14.7096218	9.75088085	13.7643325	12.4604502	10.3956605	3.29983232
0	0.16032285	0.04733437	0.03093108	0	0.07551327	0
0.94624494	1.16234068	0.99402183	1.1753812	1.00413884	1.18304127	1.30942025
22.3156098	25.3710915	21.8684804	19.8886872	28.1158876	24.9948932	6.83997918
0.02628458	0.08016143	0	0.06186217	0	0	0.03114144
32.35632	42.0847489	40.4235546	32.694156	38.2029187	37.9076628	65.1539256
0.39426872	0.12024214	0.14200312	0.27837976	0.13692802	0.15102655	0.60212625
0.02628458	0	0	0.03093108	0.04564267	0	0.010201
0.15770749	0.52104927	0.14200312	0.55675952	0.27385605	0.17619764	0.23341954
44.1580972	46.413466	54.5291978	43.2416557	42.3107595	52.5320666	181.572941
82.8227168	88.9791834	77.2023625	83.5139274	92.3807737	83.5176794	24.7413455
0.81482203	0.6012107	0.75734997	0.5876906	0.82156815	1.08235691	0.5868471
80.5359582	82.1654622	90.59799	77.4205038	80.4680355	85.908933	76.0173239
35.747031	37.9965162	39.713539	39.8392365	39.435271	38.8641642	17.7255033
459.559626	480.968559	517.270029	443.180575	531.006878	411.446651	329.623291
0.86739119	0.96193712	1.13602495	0.71141494	0.95849617	0.93133036	0.3941556
0.05256916	0.52104927	0.23667187	0.12372434	0.1825707	0.276882	0.03280137
1.76106697	2.52508494	1.37269682	1.97958939	1.41492292	2.81916217	1.93905247
0.49940705	0.68137213	0.37867498	0.55675952	0.5477121	0.67961945	0.50782817
0	0	0.04733437	0.03093108	0.04564267	0	0
1.07766785	1.08217926	1.98804367	1.54655421	0.5477121	1.38441	2.11774331
9.09446525	10.5412276	7.81017156	12.1559161	8.71775087	8.68402634	6.74250026
368.089281	385.015332	384.970456	369.44087	374.22429	342.075124	175.993042
186.199976	179.160788	168.273696	168.821858	176.500223	214.457694	147.523115
44.0003897	43.9685424	39.9975452	40.4269271	45.8252454	47.7243882	43.1636157
29.8592848	27.6156114	28.7792988	26.5079392	27.6594609	28.5943592	21.5177752
114.732199	118.518669	135.092301	111.877732	144.915492	128.951498	97.2627924
122.275874	112.867289	105.792324	124.002717	119.173024	113.093711	66.6421367
639.503871	591.952054	686.443078	660.842615	659.901791	691.198154	865.371315
1.2879445	2.04411638	1.9407093	1.67027855	2.69291781	1.56060763	0.87431939
0.26284582	0.04008071	0.09466875	0.24744867	0.09128535	0.20136873	0.21167572
4.10039474	3.20645706	3.07673425	2.938453	3.4232006	3.34775508	1.77085702

0.13142291	0.16032285	0.04733437	0.21651759	0.04564267	0.15102655	0.0450519
0.9988141	0.72145284	0.85201872	0.80420819	0.82156815	1.23338345	0.32665007
0.05256916	0.04008071	0.04733437	0.06186217	0.1825707	0	0.02505633
2.70731191	3.16637635	2.41405303	4.54686938	2.09956304	2.41642472	0.99633417
48.1533536	48.778228	54.7185353	50.6341849	50.6177263	57.1383762	144.02271
69.3387264	73.9088353	75.0723157	76.368847	67.1403745	66.6530485	51.1740832
61.4796365	55.1911422	53.3458384	60.6249251	52.4890759	64.0352551	145.609166
5.46719298	5.1303313	4.02342171	6.77390745	3.83398468	5.28592908	8.37562513
665.131339	567.462738	752.616532	719.333295	589.794643	782.871267	2351.57622
177.079227	202.447683	238.707243	198.608492	192.064375	200.890476	761.400388
125.876862	133.869582	148.629931	128.67331	125.334785	137.862064	268.030132
136.837532	134.029905	166.522324	114.10477	158.790865	141.083964	53.4369794
0.05256916	0.04008071	0	0.03093108	0	0.10068436	0.08150454
0.65711454	0.56112999	0.33134061	0.43303518	0.1825707	0.67961945	1.1064536
41.4507853	41.5236189	37.1574829	30.8692221	41.4435487	42.388117	32.5665608
89.8932692	84.7306278	69.912869	90.4424903	94.3434087	71.6620956	32.1206697
13.0108679	13.2667161	12.3542714	12.8054689	12.8712343	15.1278256	8.56850774
2.8650194	4.00807133	3.55007798	3.71173011	3.4232006	3.97703235	2.5087588
21.4482186	20.4411638	19.5490961	21.7445522	19.078638	23.5097988	33.1697725
0	0.08016143	0.04733437	0.03093108	0.09128535	0.05034218	0
1081.6631	1023.58126	904.228529	1128.52061	966.027211	1098.56709	1839.03064
102.982991	110.823172	104.182955	123.631544	73.7585624	101.942918	60.712085
15.4290494	14.5092182	16.1883556	15.9604395	14.6056559	17.745619	15.7546986
61.2430752	67.2955176	67.5461504	62.9138253	64.6756701	60.133736	53.4554064
28.7553323	27.4552886	26.0339052	32.1064654	26.7922501	26.882725	23.3262431
27.4148187	26.8540779	30.1519956	26.1367662	26.7009647	28.1412796	37.4287321
1.31422908	1.56314782	1.27802807	0.92793253	1.41492292	1.38441	0.2251964
97.4369442	95.2317747	92.4440306	98.5155033	98.7707481	99.4258088	94.9368212
24.6812222	26.6135936	25.4185583	24.8376606	26.7009647	28.3174772	13.7424559
71.5466312	75.1513374	66.8834691	75.657432	73.1652076	72.9458212	155.594561
8.46363529	8.37686907	8.18884654	8.66070358	8.39825215	9.53984343	7.87633353
19.5557287	20.8820516	18.1290649	21.1877927	21.0869157	18.9790025	11.4817543
17.531816	18.7176931	15.3363369	17.3523383	20.1740622	17.0911707	6.9450817
4.17924848	3.36677991	4.73343731	2.41262457	5.29455027	5.91520635	5.28961524
1092.17694	1066.86843	1134.27358	1067.55544	1116.41982	946.961608	1367.49638
43.7638284	40.8422468	39.9975452	42.4993097	42.9953996	44.2004355	54.2133133
94.493071	73.9889967	74.4096345	74.4820508	105.891005	76.7969981	72.4941877
143.697808	148.058155	149.671288	140.736433	146.649914	134.010888	96.9304728
38.8748963	41.5236189	41.5595795	38.5091999	43.497469	45.5093322	18.8113482
1.97134362	2.04411638	1.46736556	2.3507624	2.23649106	1.58577872	2.70652708
0.23656123	0.36072642	0.42600936	0.21651759	0.68464012	0.32722418	0.11112975
17.0849781	23.5674594	20.4957835	20.0124115	20.0827769	17.8966456	9.60248201
45.4197571	43.5677353	48.7544043	39.9938919	56.7338447	47.1706242	54.7942012
8.38478155	8.29670764	8.23618091	8.6297725	7.57668401	7.32478743	3.33018825
0.07885374	0.12024214	0.09466875	0.03093108	0.13692802	0.20136873	0.18165843

0.15770749	0.32064571	0.28400624	0.30931084	0.27385605	0.276882	0.10600069
0.02628458	0.04008071	0.04733437	0.03093108	0.09128535	0.05034218	0.0506754
1.8662053	1.68338996	2.74539364	1.29910554	2.51034711	1.86266072	1.70939832
0.02628458	0.08016143	0.04733437	0	0.09128535	0.02517109	0.00827846
0.97252952	0.9218564	0.85201872	0.86607036	0.91285349	0.72996163	0.84704951
17.7683772	18.5172895	17.892393	18.8679614	17.9375712	20.1117016	15.3338936
51.9909025	50.5818601	45.0149888	46.5512818	52.9911454	55.3260577	61.0069117
91.5229133	91.9852369	92.3020275	91.6178715	91.9243469	82.6870334	38.9489066
7.54367493	7.73557766	6.24813724	6.83576961	5.24890759	8.05474907	6.03733494
2.20790486	1.76355138	1.42003119	1.82493397	1.91699234	2.21505599	1.28644796
761.359192	838.047634	809.133773	849.491297	615.080685	814.284788	893.819087
21.7899182	22.0443923	25.7025646	20.9094129	24.8752577	20.1620438	12.6089338
10.618971	11.3428419	11.8335933	10.4856376	10.2239591	11.07528	11.5518282
0.05256916	0	0.09466875	0	0.04564267	0.05034218	0.0582228
30.3586918	28.1767414	31.0513487	30.9310842	29.8959519	32.9237868	23.4749309
42.4233148	36.7540141	45.4409981	41.509515	33.8668646	43.6718426	200.75751
29.3598777	27.4552886	29.5366488	28.1782177	27.5225329	27.2602914	26.2828438
13.0108679	9.29872548	10.6028996	10.5784308	9.35674832	11.1256221	17.4135702
0.07885374	0	0	0	0	0.05034218	0.6584343
1.65592864	1.8036321	1.9407093	1.14445012	1.32363757	2.24022708	2.35406522
9.5938723	7.65541623	6.53214348	6.24807901	7.30282796	8.60851307	11.4609792
0.02628458	0	0	0.03093108	0.04564267	0	0.05284066
3.1541498	2.36476208	2.9820655	3.09310842	2.92113118	3.22189963	1.91580189
52.8845783	50.181053	48.7070699	51.4383931	48.6550913	46.2392939	28.7524115
67.7090823	74.5501267	68.2088316	68.0174542	76.3601948	75.8908389	33.2974968
25.6011825	27.9763379	22.6731647	27.559596	26.5640367	25.3724596	14.948711
100.775086	111.905351	104.182955	105.041962	104.476082	93.8881688	53.4475087
1.73478239	2.44492351	2.88739676	3.18590168	2.32777641	2.21505599	0.72988263
64.8440629	68.8586654	65.3214348	67.3679014	66.3644491	57.5411136	17.5047528
14.9822115	16.0322853	15.6203431	16.4244057	16.4313629	15.9836427	11.5594568
12.4063225	15.230671	13.4429619	14.1973677	13.7840878	10.9997667	5.82813812
865.840404	904.461375	939.161296	932.29381	848.360395	804.040155	765.57269
58.4831942	58.9186485	55.7125571	58.6453357	61.8001816	60.2344204	56.7790716
2.44446609	2.08419709	2.27204991	1.63934746	1.64313629	2.81916217	1.45088762
0.02628458	0	0	0.03093108	0	0.02517109	0.02279585
0.02628458	0.12024214	0.14200312	0.06186217	0.09128535	0.17619764	0.06323214
105.716587	110.061639	117.76792	115.775048	104.339154	99.5768353	57.0578181
0.05256916	0.08016143	0.18933749	0.06186217	0.13692802	0.17619764	0.0481701
65.4486083	69.6602796	71.7115752	66.1925202	78.1859018	71.3852136	23.4959116
19.1351754	14.3088146	14.5789869	20.8475508	12.3235222	11.5787018	18.8544328
7.49110577	6.45299483	7.99950905	6.77390745	6.29868911	7.34995852	7.45627289
4.17924848	4.32871703	4.54409981	4.05197203	4.47298212	3.64980817	4.04783304
58.1414946	59.1992135	69.2975222	54.5933637	67.5968013	73.2227032	62.035442
49.0995985	45.3713674	48.7544043	46.644075	47.0575976	44.7038573	39.0018285
26.38972	26.3330286	24.7085427	25.4253512	23.5516202	25.1710908	37.6907832

4.99407051	5.65138057	5.68012477	6.00063034	5.97919039	5.43695562	4.76534549
87.8693564	89.0593449	87.0952464	88.6484874	92.0156323	73.7764672	29.0826001
79.1428753	73.4679474	83.2138278	74.543913	88.8662877	83.1904552	147.808113
3.10158063	3.16637635	3.55007798	2.87659083	3.33191526	2.91984654	2.41279359
48.941891	43.8082196	38.1041703	39.9320297	47.1032403	50.5687215	61.5945418
10.6452556	9.9800976	8.94619651	9.89794695	11.0455273	6.79619453	9.60338081
23.7349772	21.803908	27.8326114	25.1160404	23.5516202	27.8140554	18.9893055
58.8511783	68.8185847	67.3094785	67.6153501	63.1238192	54.7471226	35.772313
182.49385	167.377059	217.690782	188.803338	198.545635	206.780511	106.229855
483.136895	508.223444	423.97398	493.196138	463.273149	381.316855	82.0967148
139.439706	133.74934	142.287125	133.096455	137.658307	126.258192	73.6345947
12.6691684	10.7416312	13.2536245	16.3625436	9.76753239	13.1644805	15.3369689
529.555466	531.630581	634.327933	577.607067	497.687725	536.72317	459.59376
0.15770749	0.20040357	0.28400624	0.15465542	0.13692802	0.20136873	0.42122544
2.97015773	3.24653777	1.79870618	2.13424481	1.91699234	3.14638635	1.32700884
6.62371458	5.57121914	5.77479351	3.71173011	6.29868911	6.74585234	2.4143899
31.6729209	33.7880413	34.5540923	31.2403951	33.0909392	31.866601	26.179066
0.02628458	0.04008071	0.09466875	0.03093108	0	0.05034218	0.04777644
30.8055297	30.1006157	31.5720268	31.4259816	27.9789596	37.2028723	39.6882052
1562.98636	1586.35455	1470.39496	1600.12685	1405.74874	1355.46324	1868.9914
0	0.04008071	0	0.06186217	0	0	0
1.60335948	2.64532708	3.59741235	2.1651759	1.68877897	3.0205309	2.17325877
13.6679825	16.914061	15.2416681	15.434611	16.2487922	16.2856958	9.19430392
33.486557	41.0827311	34.6014267	35.8491266	36.5141398	32.2945095	16.042143
15.5079032	15.3909939	16.1410212	15.8676462	17.7550005	16.0339849	15.6523016
41.5559236	37.5556283	46.2456825	42.9014138	42.1738315	46.012754	110.871863
24.9177834	26.9342393	30.2466644	23.9406592	27.1117488	35.0633295	14.0922596
24.3395226	23.3670558	21.6318085	24.8067295	23.3234068	24.2145894	37.8565538
110.000974	111.82519	119.850633	112.032387	105.754077	110.853484	174.643182
98.4357583	112.266078	105.792324	111.351903	107.214643	111.432419	38.0758936
294.098184	364.373764	374.651563	291.27802	331.913531	357.026752	92.2831802
26.3371508	24.2889122	25.0872177	27.9926312	24.9665431	25.9765657	10.9752508
1.31422908	2.08419709	1.42003119	2.78379758	1.04978152	1.61094981	0.5404865
79.4057212	77.8367452	67.7354879	74.7294995	71.6589993	68.717078	31.1482602
40.5045403	33.3872341	37.0628141	39.3443391	30.8544481	35.4912381	94.8298713
1.83992072	1.88379352	1.32536245	1.88679614	2.09956304	1.71163418	1.71094483
147.456503	135.112084	147.777913	147.108237	137.110595	127.088838	121.797008
18.4254917	17.0343031	15.1943338	14.7231961	17.6180724	15.8577872	18.0317062
38.9011808	39.6398254	43.7369607	38.7875796	42.1281888	45.9624119	39.1825786
80.6148119	90.9832191	74.5989719	87.040071	80.3311075	89.5587412	44.1922081
50.5452505	51.9846851	47.1450356	46.8296615	52.9911454	43.0929075	28.3759801
5.30948549	6.97404411	6.05879975	5.47480191	7.39411331	6.8717078	8.22674531
0.13142291	0.04008071	0.33134061	0.24744867	0.13692802	0.20136873	0.35333562
5.96660003	5.25057344	5.34878416	5.90783709	4.24476875	5.16007362	3.44366783
38.1652126	35.1507855	44.7783169	38.3545444	34.0494353	39.9213501	75.1197788

0	0.04008071	0	0	0.04564267	0.02517109	0
7.33339828	6.89388268	8.09417779	8.28953057	7.57668401	5.08456035	11.6353283
63.9503871	68.6582618	56.2332352	59.3876817	64.6300274	57.8683378	56.05845
1.73478239	1.12225997	1.37269682	1.45376096	0.86721082	1.48509436	1.54000998
0.02628458	0.12024214	0.04733437	0	0.09128535	0.05034218	0.09954657
3.4432802	4.36879775	4.9227748	4.33035179	4.1534834	5.00904708	7.84378764
12.7743067	9.21856405	8.89886214	12.6508134	9.35674832	10.9745956	13.3846863
12.248615	12.4250211	13.3482932	11.9084674	11.5475967	12.5352032	9.3676464
54.4353686	43.5677353	49.4644199	51.7167728	47.011955	55.6784529	40.2843223
34.4065174	35.230947	37.4414891	32.9106736	37.1074946	32.8482735	26.731652
0.02628458	0	0	0.03093108	0.04564267	0	0.03389936
32.4351738	30.1006157	29.7259863	32.8488114	33.2735099	30.1297957	19.6715272
0.07885374	0.08016143	0.04733437	0.06186217	0.1825707	0.07551327	0.17133326
38.4280584	37.315144	38.6248484	38.0452336	35.7382143	36.3973973	14.6125203
0	0	0.04733437	0	0.04564267	0.02517109	0
0	0.12024214	0.09466875	0.09279325	0.13692802	0.05034218	0
0.02628458	0	0	0	0.04564267	0.02517109	0.02412119
0.07885374	0.04008071	0	0.06186217	0.04564267	0.07551327	0.04058083
54.2776611	53.1871065	55.1445446	53.8510176	54.4973536	60.5616445	52.1170044
8.12193573	8.25662693	9.56154336	6.4645966	9.95010309	7.82820925	5.75634089
17.3741085	15.9120432	20.2591117	17.4760626	12.9168769	19.5327665	8.76794185
52.9371474	55.1911422	55.9965633	57.2225058	56.6425593	56.3832435	42.5195226
0.89367578	0.9218564	1.04135621	0.43303518	0.63899745	1.35923891	0.47243752
23.7612618	29.5795664	30.1046613	26.6007324	28.6179571	24.5418136	43.8413298
5.07292426	6.13234913	4.16542483	5.90783709	5.20326492	5.46212671	2.89037426
6.62371458	7.49509338	7.43149657	6.49552769	7.12025726	6.72068125	2.08429389
0	0.12024214	0.09466875	0	0.09128535	0.17619764	0
226.126256	209.742372	195.017617	197.525904	207.811098	201.393898	300.670564
50.3349739	53.7883172	46.2456825	56.2945733	48.97459	36.5232528	8.41922914
0	0	0.04733437	0	0.04564267	0.02517109	0
127.007099	111.905351	103.662277	114.197563	122.094155	120.594696	72.8733177
0.23656123	0.32064571	0.14200312	0.21651759	0.3651414	0.30205309	0.26807029
13.7731208	13.8679268	12.0702651	14.3829542	12.3691649	11.1004511	9.27267795
26.7314195	24.048428	27.2172645	25.5800067	27.7963889	28.3426483	30.4329008
71.2837854	64.8105133	74.0309595	65.4501742	66.227521	69.5980662	49.4473519
3.28557271	3.00605349	2.9820655	2.96938409	3.01241653	4.07771672	4.50329715
58.9826012	52.1049272	49.6537573	63.9345511	50.3438702	54.0171609	28.9885687
12.2223305	11.6234068	12.0229308	11.4445012	11.8214528	14.5488905	41.7636784
0.47312247	0.52104927	0.42600936	0.52582843	0.31949872	0.37756636	0.34054943
0.2891304	0.04008071	0.14200312	0.21651759	0.04564267	0.10068436	0.40959926
0	0	0.09466875	0.06186217	0	0.05034218	0
26.2845816	31.9443285	28.7319644	29.6010476	28.9830985	24.8690377	17.7301029
1.41936741	2.4048428	1.51469994	1.48469204	2.05392036	2.29056927	2.70239883
0.07885374	0	0.04733437	0.06186217	0	0.02517109	0.13849336
19.0300371	18.8780159	16.5196962	19.8577561	21.4520571	16.4115512	5.78795728

0.05256916	0	0	0	0.13692802	0	0.10305622
4.31067139	2.60524636	4.54409981	2.47448674	3.05805921	4.83284944	6.00590072
41.1090857	39.559664	38.0095016	43.6437598	32.4062991	37.6307808	20.0151798
5.20434717	5.731542	6.43747474	5.16549107	5.70533434	5.33627126	11.0336244
20.5808274	19.2387424	19.4544273	18.2184086	18.3027126	19.8851618	11.5540262
54.409084	54.790335	54.9552071	51.8095661	54.2234976	51.0973144	23.976164
60.7699528	64.2894641	43.8789638	65.1099323	62.7130351	51.273512	19.8661113
15.4290494	13.1063932	11.2655808	16.3934746	14.7425839	11.5031885	9.37844918
0.02628458	0.16032285	0.09466875	0.09279325	0.04564267	0.05034218	0.0166858
20.5545428	21.7237466	18.5077399	18.8679614	22.6844093	22.6539818	13.1609891
7.22825995	8.37686907	7.43149657	7.17601154	6.29868911	8.15543343	3.53901299
0.02628458	0.12024214	0	0	0.04564267	0.05034218	0.02864458
0.89367578	0.9218564	1.13602495	0.74234602	1.55185094	1.03201472	0.78338977
6.07173836	7.77565837	5.06477792	7.39252913	7.80489738	5.36144235	11.7337569
0.05256916	0.04008071	0	0.03093108	0	0.10068436	0.06629596
0.34169956	0.24048428	0.37867498	0.37117301	0.09128535	0.30205309	0.4068088
1.76106697	3.00605349	1.79870618	2.04145156	2.09956304	3.04570199	1.2720917
75.2790418	78.9189244	69.155519	82.0601665	70.8830739	61.1154085	28.772924
0.73596829	0.84169498	0.47334373	0.55675952	0.63899745	0.62927727	1.3487628
35.6156081	44.730076	38.3408422	39.0350283	36.2859264	49.7632466	35.4933189
91.8383283	93.9491919	94.6687461	87.8752103	89.2770718	92.7051275	103.210358
6.46600709	6.93396339	7.33682782	5.44387082	6.02483306	7.77786707	4.68727185
42.2656073	52.5057344	51.7838041	47.8503873	43.0866849	49.0332849	67.6296861
1.2879445	30.8220685	1.13602495	1.05165686	1.50620827	46.1889517	2.95041862
19.2140292	21.3229395	20.8271241	20.7856886	20.4022756	16.9904863	7.56493047
89.1835855	90.5022505	94.7634149	91.7415958	90.6919947	81.0760836	40.7154196
54.1462382	48.6179052	41.9855889	50.1702186	45.186248	43.9738957	43.6626868
59.9288462	61.1230877	58.741957	63.5943092	59.1072638	64.9162433	29.9775404
1.99762821	2.08419709	1.84604055	1.79400289	2.19084839	2.4667669	4.21685793
15.5604723	18.8780159	16.1883556	15.3108867	15.5185094	16.9904863	27.9104057
3.91640267	3.72750633	5.11211229	3.43335035	4.06219805	4.22874326	7.58050271
18.5043455	20.7618095	17.2770462	20.4763778	17.6180724	21.4457694	28.101916
35.2213394	35.6317541	33.3233986	32.8178804	36.5597825	31.0862972	13.097962
0	0.08016143	0.33134061	0.06186217	0.13692802	0.07551327	0
0.05256916	0.04008071	0.09466875	0.03093108	0.09128535	0.02517109	0.0648911
0.31541498	0.80161427	0.28400624	0.43303518	0.31949872	0.40273745	0.86414894
0.39426872	0.40080713	0.5206781	0.21651759	0.31949872	0.52859291	0.36690995
0.55197621	0.68137213	0.66268122	0.49489735	0.50206942	0.62927727	1.19192299
0.05256916	0	0.04733437	0.06186217	0	0	0.07105755
0.02628458	0	0	0.06186217	0	0	0.04784753
30.6215376	29.6196471	31.0040144	30.7145666	30.6718774	25.8507103	74.1026233
2.1553357	1.44290568	1.89337492	1.26817445	2.14520571	1.6361209	4.62965833
2.1553357	1.8036321	2.88739676	1.91772722	2.14520571	2.11437163	1.42865913
0.05256916	0.08016143	0	0.06186217	0	0.02517109	0.15858714
0	0.04008071	0	0.03093108	0.04564267	0	0

0.15770749	0	0.04733437	0.03093108	0.04564267	0.07551327	0.06314935
0.02628458	0.28056499	0.23667187	0.15465542	0.13692802	0.10068436	0.01596105
0.05256916	0	0	0	0.13692802	0	0.15858714
46.9705474	46.7741924	46.7663606	46.4584885	34.0494353	52.7837775	220.993765
0.05256916	0.08016143	0	0.03093108	0	0.05034218	0.07043492
0.02628458	0	0.09466875	0	0.04564267	0.02517109	0.02027336
0.97252952	0.56112999	0.94668746	0.52582843	0.68464012	0.98167254	0.86052878
0.02628458	0.04008071	0.04733437	0.03093108	0.04564267	0	0.06548583
0.68339912	0.96193712	0.66268122	0.61862168	0.7302828	1.20821236	1.78630787
0.23656123	0.16032285	0.18933749	0.18558651	0.3651414	0.20136873	0.10549831
0.05256916	0.24048428	0.09466875	0.12372434	0.09128535	0.22653982	0.2359893
0.26284582	0.28056499	0.33134061	0.12372434	0.41078407	0.22653982	0.40091698
1.73478239	1.64330924	2.31938428	2.07238264	2.00827769	2.08920054	5.42052044
0	0	0.04733437	0	0.04564267	0.02517109	0
0.02628458	0	0	0.03093108	0.04564267	0	0.01233118
0.13142291	0.08016143	0.28400624	0.12372434	0.04564267	0.17619764	0.11195523
0.23656123	0.08016143	0.23667187	0.21651759	0.22821337	0.25171091	0.2917156
0.02628458	0	0.09466875	0.06186217	0.04564267	0.05034218	0.05894325
0.34169956	0.52104927	0.56801248	0.24744867	0.5477121	0.80547491	1.17249942
0.02628458	0.08016143	0	0.03093108	0	0.02517109	0.02080462
2.99644231	2.20443923	3.26607174	2.13424481	3.69705665	3.52395272	5.01094156
2.70731191	1.96395495	2.03537804	2.3507624	2.32777641	2.69330672	4.26780257
0.07885374	0.04008071	0	0.06186217	0	0.02517109	0.06743141
0.07885374	0	0	0.03093108	0	0.02517109	0.1062927
0.26284582	0.44088785	0.42600936	0.27837976	0.27385605	0.35239527	0.29404245
0.9988141	1.72347067	1.27802807	1.05165686	1.78006431	1.661292	1.46065103
0.15770749	0.04008071	0.28400624	0.09279325	0.1825707	0.30205309	0.28454462
0.07885374	0.04008071	0.09466875	0.03093108	0	0.10068436	0.1855012
0.02628458	0	0	0.03093108	0.04564267	0	0.03073952
0.15770749	0	0.04733437	0.03093108	0.04564267	0.07551327	0.05092958
0.02628458	0.08016143	0.09466875	0.03093108	0.09128535	0.12585545	0.07161145
0.05256916	0	0.18933749	0	0.1825707	0.15102655	0.05929653
0.81482203	0.04008071	0.14200312	0.03093108	0.68464012	0.67961945	0.96538479
1.44565199	3.48702205	2.69805926	2.90752192	2.46470444	2.91984654	1.22045879
12.0120538	14.5893796	12.306937	14.2901609	12.8255916	13.8441	19.0338971
0.78853745	0.48096856	0.37867498	0.64955277	0.77592547	0.57893509	0.57026678
0.36798414	0.36072642	0.66268122	0.30931084	0.3651414	0.45307964	0.45910207
1231.19609	1237.93291	1198.31699	1318.99422	1237.09906	1308.26745	497.146079
10.0407102	10.7817119	9.08819963	10.3928443	10.1783165	10.8990823	6.12295759
55.9861589	68.8185847	53.5825103	57.6246099	63.5802459	48.0264413	26.3209238
0.02628458	0	0	0.06186217	0	0	0.05327845
0.02628458	0	0	0	0	0.05034218	0.02804496
78.9063141	94.349999	79.9004217	75.5337077	87.1318661	80.8495438	54.8592965
1.47193657	2.32468137	2.22471553	2.22703806	1.18670954	1.9130029	1.67731564
0.10513833	0	0	0.09279325	0.04564267	0.05034218	0.10301495

0.02628458	0	0	0.03093108	0	0.02517109	0.06317002
0.02628458	0	0	0.03093108	0.04564267	0	0.0470081
94.5193556	89.0192642	95.9941086	106.155481	91.4222775	94.5426172	88.6008704
49.9932743	50.2211337	50.7424479	52.5828432	52.9455027	52.2803557	215.717941
7.35968286	9.41896762	6.81614972	9.49584286	7.30282796	8.40714434	3.64051422
0.02628458	0.08016143	0.04733437	0.06186217	0	0.02517109	0.02314037
0.23656123	0.24048428	0.09466875	0.40210409	0.09128535	0.22653982	0.09407733
0	0.04008071	0	0	0.04564267	0.02517109	0
0.42055331	0.68137213	0.47334373	0.34024193	0.68464012	0.75513273	0.36603107
1272.59431	1344.06664	1248.53876	1629.88255	875.198288	1077.04581	1059.7317
13.0634371	13.2266354	14.1056432	13.9189879	13.464589	14.8006014	16.2815183
67.3936673	65.5319662	67.498816	68.0483853	65.1320968	75.5636147	177.293937
27.835372	33.2269113	29.3946457	27.8379758	28.070245	30.2808223	39.5937217
98.6986041	103.809047	81.6044592	98.5773654	96.8537558	74.8588241	35.1540396
82.0867485	77.0752116	71.6169064	68.2339718	74.4888452	78.986883	44.1289873
49.7829976	49.4195194	56.6119102	49.5206658	48.4725206	50.8456035	30.6178019
33.0660037	33.0265077	34.2227517	36.3749551	22.3649106	35.5415803	32.9390517
0.05256916	0	0.04733437	0.06186217	0	0	0.04840578
4.31067139	4.12831347	3.07673425	3.89731661	2.64727513	4.05254562	6.36089365
1.13023701	0.96193712	1.04135621	0.9897947	1.14106687	1.33406781	1.03565715
0.02628458	0.04008071	0.09466875	0.03093108	0.04564267	0.02517109	0.03073952
9.96185644	9.09832191	8.94619651	8.90815226	8.1243961	9.46433015	9.0101237
61.7424823	60.2813927	50.9317854	65.357381	78.8248993	42.0608928	23.4670404
18.3992072	20.2006795	17.2297118	19.1772722	17.8462858	16.2101825	7.93494771
0.05256916	0.08016143	0.04733437	0.03093108	0.04564267	0.05034218	0.02444671
152.713419	152.426953	145.269191	156.171044	167.73683	146.873315	24.5337287
0.02628458	0	0	0	0	0.05034218	0.05051617
0.02628458	0.12024214	0	0.03093108	0.04564267	0.02517109	0.06670859
34.1436716	33.5876377	29.9153238	45.0975208	28.207173	29.928427	18.4322377
3.12786522	2.1242778	3.02939988	3.18590168	1.64313629	2.66813563	6.91911656
523.615151	521.169515	522.524144	516.796555	553.600002	417.689081	170.899334
43.1329985	53.8684786	47.4290418	42.5611719	51.6675078	44.0242379	136.340987
0.02628458	0	0	0	0	0.05034218	0.09954657
0	0.04008071	0	0	0.04564267	0.02517109	0
0.07885374	0.16032285	0.14200312	0.06186217	0.13692802	0.10068436	0.41757846
11.6703543	12.1845368	13.7269682	12.8364	11.228098	11.3018198	6.44522461
20.7648195	20.2407602	20.4957835	19.2700655	23.3234068	22.3267576	13.2160759
34.4853711	30.4212614	34.2227517	32.9416047	33.5930086	36.8756481	16.1506304
56.1964356	60.241312	63.4280599	57.7792653	61.5719682	53.8913055	58.1592827
178.892863	190.062742	184.935396	173.708969	192.018733	164.946158	76.4362573
0.26284582	0.36072642	0.23667187	0.12372434	0.1825707	0.37756636	0.25993162
0	0.12024214	0	0	0	0.17619764	0
1.44565199	1.72347067	1.89337492	1.79400289	0.95849617	1.661292	0.89928543
0.05256916	0.04008071	0	0.15465542	0	0	0.0604106
1611.32371	1789.60385	2425.41328	2464.61972	2058.48463	1710.87904	1424.21139

5.91403087	7.69549695	5.25411541	7.20694262	4.83812352	5.53763998	6.63415578
0.23656123	0.40080713	0.23667187	0.27837976	0.3651414	0.37756636	0.03667007
3.52213394	3.9279099	3.59741235	3.46428143	3.69705665	4.6818229	1.68864377
22.8150169	17.1144646	21.9158147	21.0640684	22.2279826	22.2260732	12.2221477
0.60454538	0.36072642	0.56801248	0.52582843	0.31949872	0.45307964	1.63071986
90.6818067	95.472259	85.9592215	83.4211342	105.160723	96.8080153	91.0005897
774.790613	820.612523	812.068504	736.438184	810.750831	734.794484	1247.09204
68.4976198	64.9708362	63.8067349	72.9045655	66.7295905	65.6210338	33.7056032
42.1867535	38.1969197	49.7484261	42.9014138	35.8751423	44.5780019	74.0407954
0.10513833	0.28056499	0.14200312	0.03093108	0.09128535	0.25171091	0.23534157
5.83517713	6.01210699	6.01146538	6.77390745	5.20326492	7.07307652	14.5526733
6.51857625	5.49105772	5.68012477	6.15528576	5.65969167	7.17376089	15.7279652
14.1148203	16.7136574	13.3956276	15.2180934	15.7923655	15.7571029	13.5956314
24.2080997	21.5634237	27.027927	28.0235623	20.4022756	20.4389258	10.6103231
0.02628458	0	0	0.03093108	0	0.02517109	0.02824202
2.23418944	2.68540779	2.31938428	2.50541782	2.19084839	3.14638635	2.56263576
49.5201518	19.5994688	18.5077399	44.7263478	22.2736253	30.1297957	59.8885542
0.13142291	0.12024214	0.14200312	0.12372434	0.09128535	0.10068436	0.13156114
112.997417	144.010003	133.908941	125.116236	131.313975	116.819033	138.505086
0.55197621	0.9218564	0.85201872	0.5876906	0.59335477	0.78030382	0.37722032
0.02628458	0	0	0.03093108	0	0.02517109	0.03296109
0.42055331	0.56112999	0.37867498	0.55675952	0.68464012	0.37756636	0.21520881
9.3047419	9.61937118	10.2242246	9.49584286	8.07875343	9.79155433	2.68261932
2.10276653	1.96395495	1.70403743	1.73214072	1.64313629	2.91984654	2.63823932
0.02628458	0	0	0	0	0.05034218	0.06797789
0.02628458	0	0	0.06186217	0	0	0.07939146
0.02628458	0	0	0.06186217	0	0	0.01753192
0.07885374	0.08016143	0	0.15465542	0	0.07551327	0.06341921
34.3013791	38.9584533	40.0448796	36.0347131	39.6634843	33.0244712	9.52778653
11.2760855	16.352931	23.1938428	16.6099922	23.3234068	15.0271412	26.9674866
0.02628458	0	0	0.03093108	0.04564267	0	0.09006594
62.9252885	56.3935636	61.4400162	60.5012007	80.0572515	50.7700902	47.4572002
11.3023701	11.7837297	14.9576619	11.0423971	13.1450903	11.6038729	11.9965492
37.2978214	36.0325612	35.9741235	37.7359228	34.1863634	33.3516954	16.8454405
42.9490064	40.5216011	33.2760643	40.4578582	35.9664277	34.9374741	29.0993559
13.9045437	14.0683304	14.342315	13.949919	13.5558744	12.8624274	15.0059317
129.215003	133.268372	131.210882	122.95106	144.824207	108.965652	46.4083414
5.94031545	7.57525481	7.71550281	7.29973588	7.80489738	7.52615616	5.1463884
42.8175835	40.8823275	37.6308266	43.3035179	44.1821091	38.7131377	25.475739
1.99762821	1.36274425	1.2306937	1.67027855	2.28213374	1.33406781	1.04363407
3.81126434	3.20645706	3.55007798	3.49521252	2.82984583	3.44843944	3.89821367
90.5240992	97.5163754	87.2845839	100.773472	90.78328	70.2021723	86.4118592
41.845054	32.0244899	33.3233986	31.8590168	34.8253608	35.9191466	94.1829593
186.962229	156.074297	143.991163	193.845105	163.948488	155.079091	113.785145
0.07885374	0.04008071	0.09466875	0.09279325	0.13692802	0.07551327	0.04866833

0.86739119	0.76153355	0.56801248	1.02072578	0.77592547	0.70479054	0.68544372
30.1746997	32.705862	29.44198	31.580637	27.3399622	29.7018872	35.2889731
0.81482203	0.6012107	0.7100156	0.77327711	0.59335477	0.553764	1.78630787
66.5262762	58.9988099	46.9083637	63.5633781	49.111518	50.0904708	56.5535886
63.6875413	65.3716433	52.6831572	64.5531728	60.5221867	48.9829428	28.2787773
27.7039491	29.499405	27.2645989	23.4766929	30.9913761	26.3037899	26.7269976
15.7444644	12.9460704	14.0583088	15.1871624	13.0994476	16.638091	25.6287045
20.8173887	20.7217288	21.8684804	20.7238264	17.2985737	21.9491912	28.6936395
5.75632338	5.731542	5.20678104	5.07269781	6.29868911	6.39345707	1.91582795
24.8389297	22.5253609	20.3537804	23.7550727	21.5889851	19.0545158	13.4209792
2.44446609	1.76355138	3.171403	2.31983132	2.14520571	2.16471381	1.26385434
16.5067173	15.1905903	15.857015	16.3934746	14.3317999	14.699917	11.5583424
113.233978	122.687063	103.425605	124.745063	116.617034	111.583446	24.8394975
16.2964406	12.7456668	15.5256744	13.8571257	14.9707973	13.3658492	9.86402565
0	0	0.04733437	0	0.04564267	0.02517109	0
96.3592763	102.526465	95.7101023	92.2055621	103.198088	87.4191985	32.4500712
0.05256916	0.04008071	0	0.03093108	0.09128535	0.05034218	0.04831486
8.33221238	8.65743406	7.43149657	6.74297636	9.2198203	9.99292306	18.2500854
0.89367578	0.6012107	0.5206781	0.49489735	0.27385605	1.661292	0.45333627
0.02628458	0.16032285	0	0.03093108	0.04564267	0.05034218	0.01642582
2.05019737	1.92387424	1.89337492	1.36096771	1.96263501	3.17155745	0.90166322
2.70731191	2.4048428	2.36671865	2.44355565	2.14520571	2.31574036	1.46378554
15.8233182	17.9561595	19.2650898	15.6511286	17.4355017	17.191855	28.073143
87.8167873	102.726868	97.4614741	83.6995139	104.476082	86.0851307	99.9302165
0.07885374	0.16032285	0.04733437	0.09279325	0	0.10068436	0.03769466
130.97607	134.110067	119.613961	119.084674	126.886636	123.715911	100.767986
0.07885374	0.12024214	0.04733437	0.06186217	0.09128535	0.05034218	0.09512882
79.1691599	79.8407808	87.6632589	81.1940961	86.721082	68.9184467	32.9241773
46.4185712	51.1830708	39.7608734	50.665116	37.1531372	42.1615771	32.2448351
22.5521711	23.6476208	20.7797898	23.6004173	20.0371342	19.9355039	12.1800171
28.1507869	31.3030371	29.3946457	28.0544934	29.0743838	28.267135	17.0604128
0.07885374	0.08016143	0.04733437	0.12372434	0.04564267	0.10068436	0.20633289
0	0.04008071	0	0	0.04564267	0.02517109	0
0.02628458	0.08016143	0	0.03093108	0.09128535	0.05034218	0.04714596
0.05256916	0.08016143	0	0.03093108	0	0.05034218	0.0470597
0	0	0.04733437	0	0.09128535	0	0
66.473707	62.5659934	78.8117312	63.8108268	67.8706573	66.4516798	64.7165196
0.18399207	0.32064571	0.80468434	0.21651759	0.22821337	0.553764	0.07591055
18.2414997	16.0322853	16.6616993	17.4451315	16.1118642	20.3130703	50.8883875
0.2891304	0.32064571	0.23667187	0.37117301	0.31949872	0.30205309	0.5732398
5.88774629	5.57121914	8.47285278	5.78411275	6.89204388	5.96554853	5.70486601
19.7134362	19.2387424	20.2117773	20.6001021	19.3068514	21.8485068	35.2303232
23.5772697	24.0885087	22.4838272	22.3322428	16.7508616	26.9330672	13.3003121
0.02628458	0.08016143	0.04733437	0.03093108	0.04564267	0.02517109	0.0104293
10.1458485	10.7416312	8.23618091	11.011466	10.9998846	8.98607943	9.95689297

9.72529521	11.2225997	10.3662277	10.300051	9.67624704	9.43915906	5.37587457
206.360251	216.155287	171.823774	142.65416	207.354671	213.652219	99.1116827
11.8017772	13.7076039	10.5555652	10.9496038	12.5973782	10.5718582	13.7494669
59.7711387	59.6000206	65.6054411	60.0681656	65.816737	66.8795883	87.6704482
32.1986125	28.8180328	31.2880206	30.9929464	33.0909392	31.866601	15.599243
6.83399123	6.57323697	6.57947786	7.70183997	5.70533434	7.90372252	14.875304
96.4906992	91.8249141	96.6094554	91.4632161	93.7956966	89.7349388	57.0772009
8.20078947	7.13436696	6.20080287	6.74297636	7.53104133	6.16691725	3.82678047
14.9296424	11.9039718	10.9815746	13.3622284	14.9251546	12.182808	10.106924
14.9033578	13.4270389	15.3363369	13.7024703	13.3733037	14.397864	33.5129745
26.074305	24.9302036	23.477849	24.3118322	28.0246023	18.7021205	16.2322205
46.3922866	43.4074125	35.9267892	41.849757	39.2527003	39.3927572	13.8619934
34.4065174	31.9844092	25.5605615	31.2403951	31.9042296	33.7292617	27.3216398
0.10513833	0.08016143	0.18933749	0.15465542	0.09128535	0.17619764	0.0484605
15.4816186	16.3930117	16.5196962	17.5379248	15.8380081	17.5190792	9.92580506
2.07648195	1.68338996	1.27802807	1.85586505	0.91285349	1.71163418	1.75181365
3.60098769	2.76556921	2.13004679	2.69100433	3.56012863	3.17155745	3.352386
0.02628458	0	0	0.03093108	0.04564267	0	0.02552881
8.75276569	8.61735335	6.34280599	8.07301298	9.08289227	8.33163107	23.4548289
8.80533485	6.53315626	8.66219027	8.13487515	8.3069668	8.93573725	3.33842754
16.6118556	26.132625	28.5899613	25.1469715	22.86698	27.7888843	10.6924695
2.62845816	2.24451994	2.69805926	2.19610698	3.24062991	2.84433326	2.07308457
0.26284582	0.24048428	0.5206781	0.37117301	0.1825707	0.57893509	1.54584335
40.7411016	37.6357898	39.6188703	43.334449	39.5265563	40.5002852	48.2458757
437.086308	425.93774	305.259372	486.793404	335.65623	428.235768	191.128238
0.15770749	0	0.09466875	0.09279325	0.09128535	0.02517109	0.14071572
101.432201	107.376231	104.324958	108.475312	103.152445	87.9981336	32.96069
0.5782608	0.40080713	0.28400624	0.18558651	0.45642675	0.47825073	0.26825101
0	0.04008071	0	0.06186217	0	0	0
162.281007	154.631392	162.782909	156.109182	158.060583	145.035825	163.186162
23.2881393	21.9642309	19.8804367	23.3838997	20.8130597	17.5442503	16.8418788
0.05256916	0	0.04733437	0	0	0.15102655	0.12913069
8.96304234	7.57525481	6.24813724	9.00094551	8.35260948	7.12341871	5.07726683
5.151778	4.68944345	4.97010917	4.67059372	5.0663369	6.21725944	7.30677585
5.41462382	7.29468981	6.1534685	6.95949395	5.84226237	7.1485898	10.8229242
0.23656123	0.20040357	0.18933749	0.27837976	0.22821337	0.25171091	0.53589236
48.4950531	44.0086232	46.3876856	49.6134591	46.0078161	49.1339693	41.9987854
20.2128433	20.2006795	21.7264772	18.0946843	21.8628412	19.2055423	26.6301277
0.02628458	0	0.09466875	0.09279325	0.09128535	0	0.02317372
7.07055246	8.41694978	6.57947786	9.2174631	7.21154261	6.97239216	7.93878175
4.88893219	5.17041201	4.97010917	5.1036289	5.43147829	5.41178453	5.03414036
84.2420842	77.8768259	85.2965403	81.9364421	81.9742438	74.6322843	55.7943157
24.4183764	27.816015	25.9392364	24.4664876	26.4727513	23.837023	26.4576087
89.3675776	79.7205387	78.669728	85.7409655	93.2936271	79.7923579	40.5421998
1.76106697	1.40282496	1.37269682	2.04145156	1.73442164	1.33406781	1.11794878

188.775865	191.06476	191.04153	197.464042	179.649568	167.99186	81.026925
40.5571095	44.4094303	47.8550512	46.9224548	43.9082531	36.5484239	17.2416733
356.234935	327.539589	393.159303	370.276009	348.938248	305.199476	339.522361
115.88872	118.999638	111.567117	130.312658	104.886867	125.704428	38.6965921
2.60217358	2.08419709	3.07673425	2.84565975	2.28213374	3.2974129	1.3202823
255.643841	243.851059	253.948911	230.653095	229.628297	260.722159	223.056595
0.26284582	0.40080713	0.75734997	0.12372434	0.59335477	0.45307964	0.27635188
35.7733156	35.6317541	29.3473113	37.9833714	22.5018386	33.8299461	24.878323
22.3418944	22.365038	21.3478023	21.5280346	23.5516202	18.1231854	18.9795211
169.98239	178.759981	164.818287	171.203551	169.471251	147.980843	139.461404
108.450184	114.189952	125.862098	108.104139	117.529887	106.725425	60.9116221
2.60217358	4.08823275	2.84006238	3.40241926	2.55598978	2.76881999	4.53770581
57.9575025	52.8664608	48.6124011	53.1705338	52.2152199	47.7999015	41.5827328
29.2547394	27.0945622	29.7733207	31.3950505	27.8420316	30.8597574	23.7549896
10.7766785	10.6614697	10.2242246	11.1661214	13.190733	9.48950124	13.9428367
0.05256916	0.08016143	0	0.03093108	0	0.05034218	0.04043199
6.88656039	7.69549695	5.96413101	6.30994118	6.80075853	6.21725944	7.83649109
85.5825978	72.0651224	98.5501647	81.9364421	92.3807737	94.4419328	114.856754
5.07292426	4.40887846	4.11809046	4.94897348	4.19912608	5.43695562	6.79324964
1.41936741	1.36274425	1.37269682	1.45376096	1.23235222	1.83748963	1.10170765
3.8638335	3.04613421	4.73343731	3.55707469	2.92113118	4.17840108	11.542297
0.10513833	0	0.18933749	0.12372434	0.04564267	0.17619764	0.02936397
32.1460434	28.4172257	22.8625022	33.0962601	29.1656692	25.5486572	19.6324421
0.63082996	0.84169498	0.61534685	0.55675952	0.45642675	0.75513273	0.64387568
44.8414963	52.7462186	46.9556981	54.5005704	47.0575976	49.9142731	18.5442671
0.05256916	0.04008071	0.04733437	0	0.04564267	0.05034218	1.49551357
0.05256916	0.04008071	0	0.03093108	0	0.02517109	2.00959636
0	0.04008071	0	0	0.04564267	0.02517109	0
0.02628458	0	0	0.06186217	0	0	0.74775678
0.02628458	0	0	0	0.04564267	0.02517109	1.46152462
0.05256916	0.08016143	0	0	0.04564267	0.05034218	1.38294803
0.02628458	0	0	0	0	0.05034218	0.64307083
0.07885374	0	0.18933749	0.06186217	0.04564267	0.07551327	2.64275685
0.07885374	0	0	0.03093108	0.04564267	0.07551327	2.57228333
0.84110661	0.72145284	1.32536245	1.02072578	0.68464012	0.78030382	31.6588718
0.02628458	0	0	0.03093108	0	0.02517109	0.65619473
0.31541498	0.28056499	0.04733437	0.24744867	0.45642675	0.17619764	8.9730814
0.05256916	0	0	0	0.13692802	0	1.4290463
0.31541498	0.20040357	0.18933749	0.18558651	0.22821337	0.20136873	1.00742167
0	0	0.04733437	0.03093108	0.04564267	0	0
0	0	0.04733437	0	0.04564267	0.02517109	0
0.05256916	0	0.04733437	0.06186217	0	0	1.81146714
0.02628458	0	0	0.06186217	0	0	0.91867262
0.26284582	0.28056499	0.09466875	0.21651759	0.1825707	0.37756636	9.59807214
0.78853745	0.6012107	0.42600936	0.61862168	0.63899745	0.830646	31.6264344

0.10513833	0.04008071	0.04733437	0.12372434	0	0.12585545	4.28713889
0.23656123	0	0.09466875	0.09279325	0.1825707	0.20136873	8.63826493
0.13142291	0.24048428	0.23667187	0.12372434	0.13692802	0.20136873	2.94986621
0.05256916	0.04008071	0.04733437	0.03093108	0	0.05034218	2.17990113
0	0.04008071	0.14200312	0.03093108	0.04564267	0.02517109	0
0.02628458	0	0	0.06186217	0	0	1.02074736
0.02628458	0	0	0.03093108	0	0.02517109	1.23667468
0.26284582	0.36072642	0.09466875	0.12372434	0.3651414	0.15102655	10.2074736
0.07885374	0	0	0.03093108	0	0.02517109	3.21535417
0.65711454	0.48096856	0.7100156	0.40210409	0.59335477	0.62927727	22.0229738
0.18399207	0.08016143	0.23667187	0.09279325	0.3651414	0.20136873	6.61984682
0.21027665	0.24048428	0.23667187	0.24744867	0.09128535	0.45307964	7.45589372
0.2891304	0.20040357	0.23667187	0.24744867	0.22821337	0.15102655	11.05278
0.02628458	0	0	0.03093108	0	0.02517109	1.49551357
0.02628458	0.08016143	0	0	0	0.05034218	1.10874282
0.02628458	0	0	0.06186217	0	0	0.46939477
0.02628458	0.08016143	0.04733437	0.06186217	0	0.02517109	0.75655392
3.07529605	3.80766776	3.31340611	2.87659083	3.74269933	4.30425653	3.18136522
0.13142291	0.52104927	0.42600936	0.34024193	0.27385605	0.22653982	0.69747379
0.94624494	1.04209854	0.5206781	0.80420819	0.77592547	1.25855454	0.51629237
1.10395243	1.36274425	0.7100156	1.5774853	1.00413884	1.00684363	0.5902311
0	0.04008071	0.14200312	0.09279325	0.04564267	0.07551327	0
2.62845816	2.28460066	2.17738116	2.19610698	2.46470444	1.98851618	1.06645246
7.28082912	8.33678836	9.18286837	7.82556431	8.26132413	10.1187785	6.18079878
3.89011808	3.88782919	3.78674985	3.80452336	3.9709127	4.55596744	5.65841162
0.76225287	1.2024214	1.04135621	0.9897947	1.36928024	0.98167254	1.10153894
35.3264777	35.9123191	39.1928609	33.065329	37.0618519	35.2646983	20.6816751
0.10513833	0.04008071	0.09466875	0.06186217	0.04564267	0.07551327	0.29131182
143.408677	134.671197	146.87856	140.829226	135.695672	130.914843	215.251194
70.0221255	68.0570511	87.9945995	70.2444923	64.8582408	79.0875674	40.7793549
16.7695631	17.5954331	22.7204991	18.5895816	17.0247177	18.4755807	55.0710325
15.3501957	16.3128503	15.6676775	16.888372	15.9749362	16.9653152	51.4456667
25.8903129	23.8881051	24.6612084	23.0127267	23.1408361	24.8186956	70.2244757
39.8474258	41.8041839	49.3224167	41.0764799	37.8377774	46.4658337	40.235055
17.6632389	17.7958367	16.23569	15.5274043	21.2238437	17.8966456	63.1788889
19.3717367	17.5152717	23.856524	21.3424481	18.5309259	24.2145894	94.4109969
28.650194	34.2289291	36.8734766	28.6112529	34.4145767	32.1938252	48.8805585
0.5782608	1.36274425	0.18933749	0.80420819	0.50206942	0.45307964	1.12460718
107.398801	110.422365	115.92188	112.156111	107.899283	100.785048	60.5434891
0	0.04008071	0	0	0.09128535	0	0
3.4432802	2.52508494	3.36074049	3.64986794	2.37341909	4.02737453	4.36262451
53.4102699	55.0709	54.1505228	57.5008856	56.5056313	55.1246889	42.3297679
14.8507886	17.5553524	17.3243805	19.1463411	16.705219	16.5625778	11.8775751
13.1685754	12.1845368	14.72099	12.5889513	11.8670954	13.4161914	9.71880807
6.64999916	3.56718348	4.68610293	4.63966263	3.01241653	5.89003526	4.16211105

49.1521677	48.3373402	46.0090106	44.2005194	49.4766594	44.3011199	25.5371089
3.33814187	3.60726419	5.53812165	3.58800577	4.7011955	3.0205309	1.69792091
70.1009793	68.2574547	62.292035	75.8739496	72.8457089	61.5433171	30.5226893
0.7096837	1.12225997	0.66268122	0.68048385	0.86721082	0.70479054	0.59299565
11.9332001	13.6274425	12.4489401	12.1559161	12.0953088	16.0339849	8.28708937
0.02628458	0.16032285	0.04733437	0.03093108	0	0.10068436	0.01068401
30.910668	29.0184364	28.6846301	28.3328732	29.6220959	26.9834094	13.6680155
0.02628458	0	0	0.03093108	0.04564267	0	0.01893055
73.4654057	78.3577944	79.1904061	78.595885	73.9867757	69.1701576	74.1188858
8.77905027	8.49711121	8.33084966	10.4237754	7.43975598	9.36364579	8.34119062
12.7217375	15.1505096	12.5436089	12.0940539	12.8255916	13.4161914	10.4095747
11.7754926	9.81977475	8.70952464	11.0423971	10.0870311	11.1507932	11.630833
194.742465	195.633961	195.91697	199.814804	187.454465	175.442503	198.108599
15.9810256	15.791801	14.7683244	15.3727489	16.3400776	17.2925394	34.6006254
61.1905061	60.4016349	54.434529	61.3982022	60.9786134	60.964382	44.7821986
20.8962424	18.1565631	16.4723618	21.6208279	20.5848463	16.638091	13.8135994
116.046428	139.400721	108.443049	139.406397	114.973898	88.123989	31.8719997
17.3215393	15.3909939	15.3363369	14.5994718	17.6637151	18.576265	8.78490214
288.68356	288.340651	320.311703	287.813739	323.469636	330.647449	955.730307
74.4379352	74.5902074	95.3314273	78.8742648	77.2730483	77.4514465	125.511826
15.5867569	16.1926082	19.7384336	15.2490245	17.0247177	16.5877489	35.2440854
133.157691	141.124191	189.621498	147.510341	168.969182	170.53414	612.367828
1090.62615	1076.76836	1134.79426	1104.51809	1082.2791	944.016591	911.613874
189.43298	143.969922	196.484983	191.092238	165.454696	202.325228	493.043776
736.257417	631.551799	661.261192	738.788947	683.362126	710.529552	1120.215
46.050587	49.9405687	47.6657137	47.6029386	55.0450657	48.1019546	13.0612115
0.7096837	0.36072642	0.56801248	0.34024193	0.13692802	1.51026545	0.77065746
34.6693632	37.2750633	36.6368048	37.828716	34.232006	40.8275093	128.90736
3.28557271	2.52508494	3.26607174	3.40241926	2.14520571	4.32942762	4.409427
0.78853745	0.72145284	0.94668746	0.9897947	0.86721082	0.25171091	0.18587653
37.4555288	38.156839	28.6372957	39.8392365	34.6884328	35.3402115	12.3284803
26.0217358	26.97432	23.6671865	31.1166707	25.0578284	24.1390761	17.1554871
0.07885374	0.08016143	0.04733437	0	0.04564267	0.10068436	0.09010801
40.4256866	36.0325612	34.8380986	39.0350283	43.7256824	34.1571703	64.8978309
12.064623	12.2646983	9.23020275	11.011466	9.49367634	11.1004511	14.4761899
45.2094804	50.7021023	45.3463294	47.8503873	54.0409269	44.9807393	38.5528698
72.9922832	82.2055429	80.5157686	71.7601154	81.243961	73.2730454	98.608929
3.18043438	4.08823275	4.35476232	2.41262457	4.29041142	3.97703235	1.86152083
0.15770749	0.32064571	0.28400624	0.37117301	0.27385605	0.22653982	0.1177426
22.6310248	25.7718986	24.8505459	23.3529686	22.3649106	23.9125363	7.53619148
2.02391279	1.72347067	1.60936868	1.51562313	2.51034711	2.03885836	4.9417619
0.36798414	0.16032285	0.28400624	0.21651759	0.22821337	0.25171091	1.48564219
0.23656123	0.16032285	0.14200312	0.15465542	0.22821337	0.10068436	0.24399821
0.63082996	0.48096856	0.89935309	0.61862168	0.77592547	0.88098818	0.51912883
16.9798397	17.4351103	14.5316525	15.9913705	16.6139336	19.0545158	14.1348676

66.6576991	72.9468981	70.7175534	67.924661	68.9204388	65.4196651	38.1123542
8.77905027	9.33880619	9.51420899	8.815359	8.17003878	9.08676379	9.58009181
5.75632338	4.28863632	4.78077168	4.88711131	4.74683817	6.21725944	4.49656809
63.5298338	62.205267	72.1375845	66.099727	57.5097702	65.8475736	80.8690013
6.78142207	7.01412482	4.07075608	7.70183997	5.88790504	5.81452198	6.07070161
0.89367578	1.24250211	0.85201872	0.95886361	1.14106687	1.20821236	0.38486901
19.4505904	21.523343	19.1704211	21.8373455	19.1699234	15.7571029	19.8777117
3.62727227	4.40887846	4.40209669	2.78379758	4.5186248	4.17840108	2.63491019
14.2462433	14.2687339	12.5909432	13.7334014	12.0953088	13.0386251	11.6570031
269.758661	248.981391	265.119824	264.089597	246.881228	237.715782	306.256889
2.52331984	2.68540779	2.79272801	3.24776384	2.92113118	2.56745127	1.03028705
19.2665983	18.9581774	21.2057991	20.1670669	18.7134966	17.7707901	25.5347195
3.67984143	4.20847489	4.16542483	3.68079902	3.83398468	3.70015035	5.76375907
37.0612601	35.230947	36.163461	33.065329	34.6884328	36.3973973	56.3886738
0.05256916	0.24048428	0.14200312	0.09279325	0	0.17619764	0.01280635
48.0219307	51.2231515	46.6243575	49.5515969	50.800297	51.7265917	27.5666451
22.0790486	23.3670558	22.1524866	19.8886872	22.2279826	22.3267576	27.1038384
0.49940705	0.36072642	0.23667187	0.40210409	0.09128535	0.40273745	0.43220183
145.695436	131.825466	139.873072	141.076675	153.724528	142.166321	155.588897
0	0.04008071	0	0.06186217	0	0	0
28.650194	27.3751272	25.7025646	25.4872134	26.7009647	26.1779345	49.6421536
83.0855626	88.057327	89.0832901	92.3911486	80.5593209	76.494945	35.4075406
1.39308283	1.12225997	1.18335933	0.64955277	1.41492292	1.28372563	1.96216201
49.5201518	53.3073486	48.6124011	47.9431806	53.3106441	43.2691051	26.2012424
2.78616565	2.64532708	3.40807486	3.15497059	3.65141398	2.84433326	2.00545773
5.67746964	6.73355983	5.30144978	6.34087227	5.88790504	6.72068125	4.41944957
43.1329985	43.6879775	48.3283949	44.6026235	45.4144614	50.6694058	79.8244507
39.1377421	35.5515927	32.1400393	35.972851	34.5515048	40.9785359	29.654149
58.5883325	59.7202628	69.3921909	63.7798957	64.401814	67.1061282	54.4399882
23.7086926	20.8820516	19.1230867	23.2292443	22.9126227	21.2695718	16.6585265
0.42055331	0.40080713	0.5206781	0.12372434	0.1825707	1.30889672	0.62509923
7.54367493	6.93396339	8.04684342	7.51625347	6.38997446	7.27444525	33.8024413
304.822293	288.500974	278.894126	311.383225	291.565406	311.492249	258.947655
1.5770749	1.16234068	1.27802807	1.20631228	2.32777641	1.18304127	0.81850339
10.0669948	9.6594519	9.46687461	8.78442792	9.08289227	9.6153567	6.74045236
56.3541431	53.1871065	53.8665165	49.0876307	61.6176109	60.3351047	23.7550632
4.10039474	4.5291206	4.59143419	4.33035179	3.92527003	5.91520635	11.4781522
42.3707456	49.619923	46.6243575	47.1699034	49.5223021	35.2143561	27.7842451
0.34169956	0.32064571	0.56801248	0.34024193	0.50206942	0.52859291	0.11553235
26.2582971	23.0464101	18.6024086	24.7139363	22.5474813	24.6424979	8.84399453
9.77786437	9.0582412	9.32487149	10.26912	6.93768656	8.96090834	5.41227036
37.2452522	30.1006157	34.6960955	38.3854755	26.655322	43.0425653	59.2093159
2.05019737	1.92387424	1.56203431	1.20631228	1.82570699	2.03885836	1.99520784
0.2891304	0.44088785	0.23667187	0.43303518	0.45642675	0.276882	0.78684974
1786.40531	1916.41922	2028.65656	1873.24832	1960.30724	1536.09099	1582.96509

325.45569	334.874359	324.098452	314.723782	362.083339	254.98315	153.568044
20.8173887	18.9180967	24.0931959	21.0640684	19.5807075	20.2878992	31.1505872
150.426661	165.974234	158.806822	149.799241	162.031495	142.971796	81.6572971
0.02628458	0.08016143	0.04733437	0.03093108	0.04564267	0.02517109	0.02745819
0.2891304	0.16032285	0.09466875	0.18558651	0.13692802	0.35239527	0.18123954
29.0181781	30.6216649	26.9332583	31.95181	27.6594609	30.5577043	20.3715983
28.0982178	28.2569028	25.1345521	29.7247719	27.5225329	27.4868312	20.9458477
44.2632355	32.3852163	31.71403	37.1482322	34.9166462	31.4135214	33.8416026
72.2563149	87.1354706	87.4739214	74.7604306	92.7915577	89.6845966	260.737717
34.5642249	29.8200507	34.0807486	33.405571	29.9872373	30.5828754	40.6752355
11.7754926	10.8217926	11.2655808	12.9601243	11.1368126	11.629044	2.72327945
10.4086943	9.53920976	8.70952464	8.66070358	9.08289227	9.28813252	10.5056126
2.60217358	2.76556921	2.74539364	2.47448674	2.69291781	2.34091145	1.77832437
8.35849696	9.17848334	9.56154336	8.38232383	10.0870311	10.3453183	9.39781825
19.0563217	17.9561595	19.1704211	20.9094129	19.7176355	18.1231854	20.2883531
152.792273	142.887743	151.091319	146.458684	155.915377	162.529734	274.865497
165.408872	160.763741	140.583088	161.955157	168.512755	156.664869	74.1044636
3.41699561	4.04815204	4.07075608	2.938453	3.9709127	3.82600581	5.04521475
3.52213394	3.24653777	2.69805926	3.83545444	3.01241653	3.49878163	1.23916439
1.5770749	1.04209854	1.42003119	0.92793253	1.09542419	1.56060763	0.88435136
27.9405103	27.0144007	27.3592676	23.0745888	29.0287411	27.0085805	6.07360547
4.83636302	6.13234913	4.59143419	4.63966263	3.92527003	5.81452198	4.33742791
33.8282566	31.3030371	37.0154797	32.0755343	33.5930086	32.3196806	38.4945192
115.31046	134.791439	117.341911	119.332123	115.795466	117.121086	75.9190459
90.1823996	98.3580703	103.993618	94.3088758	99.6379589	86.185815	35.5465769
95.2027547	97.8771018	107.922371	95.5151881	99.5923163	93.9888532	34.1775871
0.02628458	0.08016143	0.04733437	0.03093108	0	0.05034218	0.06010008
60.5333915	59.8805856	59.7359788	66.0687959	59.1072638	62.7767005	29.7089695
16.7169939	15.230671	15.7623462	16.6718544	14.2405145	19.0041736	14.0066113
13.9571129	11.1424383	11.2655808	13.7024703	11.7758101	13.1644805	27.1008423
25.2069138	26.5735129	24.3298678	26.9719054	27.7507462	24.8186956	12.0262272
2.36561235	2.96597278	2.60339052	2.72193541	2.51034711	2.08920054	1.24492095
31.72549	28.6176293	28.542627	29.5701165	26.4727513	28.7705568	13.8530519
10.7503939	9.6594519	7.24215908	9.71236045	11.365026	8.86022397	7.68154121
3.31185729	2.36476208	3.02939988	3.03124625	2.69291781	3.75049253	5.13804217
7.80652075	7.73557766	7.85750593	9.00094551	8.8546789	7.19893198	7.40279215
95.6758772	108.498491	76.6816844	90.9064565	93.5674832	84.4993519	34.7605856
16.2964406	13.9881689	12.2122683	15.2180934	12.6886636	12.4093478	15.4356917
15.6656107	16.4731731	15.4310056	16.0223016	15.6097948	13.8189289	10.6434384
0.02628458	0	0.18933749	0.06186217	0.04564267	0.02517109	0.04250303
3.31185729	3.68742562	3.07673425	3.9282477	3.19498723	3.75049253	4.273572
0.2891304	0.20040357	0.56801248	0.40210409	0.31949872	0.45307964	0.27567339
1.07766785	0.68137213	1.27802807	1.20631228	0.95849617	1.18304127	1.60181678
7.5962441	6.73355983	7.9048403	7.67090889	5.93354771	7.12341871	23.3183778
213.93021	214.07109	203.348467	216.734107	202.699118	187.776338	338.329251

2.83873482	3.16637635	2.93473113	1.79400289	3.05805921	3.27224181	2.78363327
44.92035	47.8563716	46.8610293	46.0254533	44.9580346	43.3949606	37.9491732
111.551765	102.366142	128.37082	92.9788392	135.467459	96.1283959	49.3435657
22.6047402	27.6156114	28.2112863	25.6727999	31.1283042	16.361209	13.986872
42.8964372	44.9705603	49.0384105	37.6740606	47.011955	45.8617275	62.5069446
5.25691633	4.60928202	4.11809046	4.48500721	4.29041142	4.37976981	3.68310901
67.1045369	67.9768897	63.8540693	72.4405993	72.6174955	62.8270427	37.9159316
71.3363546	82.5662693	80.8944436	71.7291843	76.5427655	76.9731958	43.2324558
56.669558	47.2551609	53.3931728	54.9336056	45.5513894	50.442866	53.8013472
42.6073069	46.6940309	46.5770231	45.4686938	45.0949626	40.1982321	66.907434
37.9812205	36.1127226	34.6014267	39.2206148	31.9042296	32.5965626	54.6288862
2.575889	1.76355138	1.98804367	1.73214072	0.95849617	4.60630962	2.08333692
5.49347756	5.77162271	4.9227748	4.54686938	6.20740376	4.4049409	3.94371491
28.4924865	26.8540779	19.2177555	21.4971035	26.6096794	23.1070614	27.905876
110.60552	99.1196039	100.822215	107.671104	112.691764	103.050446	165.103238
42.975291	44.0887846	39.0981922	43.7984153	38.6137028	49.889102	69.7228656
8.8841886	8.97807977	7.24215908	8.47511708	7.7592547	7.45064289	5.99608115
0.02628458	0.16032285	0.28400624	0.21651759	0.09128535	0.02517109	0.02681697
16.3490098	17.194626	15.3363369	16.9193031	15.9749362	18.4755807	26.3324594
4.57351721	4.92992773	6.01146538	5.25828432	4.83812352	6.39345707	5.99326862
0.05256916	0.04008071	0.09466875	0.18558651	0	0.05034218	0.11391866
0.02628458	0	0	0.03093108	0	0.02517109	0.05288411
0.02628458	0	0	0	0	0.05034218	0.0670564
275.225854	383.933152	384.781119	289.236569	454.966182	374.772371	53.8170932
0.02628458	0.08016143	0.14200312	0.06186217	0.04564267	0.05034218	0.03085753
41.8976231	44.6899953	45.2516606	43.4891044	42.8584716	40.5002852	26.7359131
115.94129	101.404205	131.163548	126.631859	112.509193	125.98131	292.733276
117.544649	132.306434	144.417172	128.147482	136.608525	146.017498	192.748845
48.626476	53.6279943	43.8316295	51.0053579	48.7007339	52.1545002	38.2780258
14.9296424	15.511236	11.1235777	14.1355055	19.3981368	11.352162	8.48465118
0.02628458	0	0	0	0.09128535	0	0.02017794
341.410431	262.488591	419.571883	289.050982	548.670593	274.264206	1037.62075
0.34169956	0.12024214	0.23667187	0.43303518	0.22821337	0.22653982	0.22186626
0.02628458	0.12024214	0.14200312	0.12372434	0.04564267	0.02517109	0.03186674
1.49822115	0.96193712	1.56203431	1.5774853	0.68464012	2.16471381	1.00507369
34.2750945	34.7098977	38.4355109	39.0659594	32.4519417	40.0220344	57.3966028
0.84110661	0.96193712	1.04135621	1.05165686	0.95849617	0.553764	1.36099647
24.1818151	28.6977907	22.7204991	25.0232471	25.5142552	21.2192296	11.0874282
18.1889305	17.1545453	18.271068	22.6415537	14.1035865	19.8599907	10.9715241
5.49347756	7.65541623	7.19482471	6.06249251	6.93768656	8.53299979	3.80203124
0.18399207	0.24048428	0.23667187	0.34024193	0.22821337	0.20136873	0.03697631
0.02628458	0.04008071	0.04733437	0.09279325	0	0.05034218	0.04064923
6.64999916	6.01210699	5.53812165	7.14508046	6.66383051	5.81452198	12.0249018
1009.06509	963.299862	864.372987	1064.06023	1112.63148	812.623496	643.238387
18.6094838	14.2687339	15.2890025	19.7649628	14.879512	16.1850114	15.6404724

0.76225287	1.16234068	0.61534685	0.83513927	1.59749362	0.62927727	0.39839894
597.054272	640.690201	646.587536	608.166978	683.77291	502.868053	147.877647
6.46600709	6.13234913	5.72745914	6.61925202	5.93354771	6.97239216	7.53311548
0.07885374	0.04008071	0	0.06186217	0.13692802	0.02517109	0.10711896
1.02509868	1.60322853	0.75734997	0.80420819	1.04978152	1.18304127	0.31487461
0.02628458	0	0	0	0	0.05034218	0.06066706
3.36442645	4.04815204	4.16542483	3.30962601	3.83398468	3.59946599	1.1560824
8.54248904	10.621389	9.79821522	9.31025635	10.8629566	10.3956605	7.58338247
9.22588816	7.57525481	6.62681223	8.10394407	7.98746808	9.13710597	12.4982205
0.39426872	0.32064571	0.28400624	0.43303518	0.13692802	0.25171091	0.28354093
67.4199519	66.2133383	66.362791	69.4712152	65.9080223	57.2894027	51.935664
34.3276636	22.7257644	21.9631491	32.1373965	27.2943195	24.5669847	17.7408219
84.189515	84.009175	93.3907181	82.245753	84.2563776	85.1789714	122.168202
2.75988107	2.52508494	1.84604055	3.27869493	1.78006431	2.86950436	2.22405921
0.02628458	0	0	0.03093108	0.04564267	0	0.0202542
8.51620445	7.37485124	7.19482471	8.10394407	6.02483306	7.47581398	10.1835264
0.05256916	0.04008071	0.09466875	0.09279325	0	0.02517109	0.10414103
0.47312247	0.64129141	0.5206781	0.52582843	0.5477121	0.75513273	0.22607959
191.877446	174.671748	190.142177	198.330112	209.134736	177.179308	73.9627712
0.5782608	0.48096856	0.61534685	0.46396626	0.3651414	0.60410618	1.00266182
22.8150169	19.1986617	20.4484492	18.713306	19.7176355	27.436489	9.79444611
36.0361614	38.2370004	36.4474673	40.0557541	34.8253608	40.449943	21.4096676
12.3011842	14.8298639	12.4016057	12.124985	13.7384451	11.4276752	10.0452987
3.33814187	3.36677991	3.78674985	3.30962601	4.29041142	3.82600581	8.09415222
0.02628458	0	0	0.06186217	0	0	0.03853031
13.7994054	13.3468775	13.4902963	15.4036799	13.6015171	13.9699554	11.8752089
34.2750945	41.8041839	43.0742795	35.6016779	39.8004124	38.2852292	54.665213
61.7161977	63.3676077	66.6941316	62.418928	59.8831892	61.2160929	59.2594316
9.22588816	9.41896762	10.5082308	8.19673732	8.6721082	10.5970292	9.68746191
18.9248988	17.7156753	20.4957835	19.5175141	16.3400776	18.1735276	9.14679969
37.2189676	35.1507855	37.5361578	39.6845811	37.7921347	37.4797543	37.0157846
41.030232	38.717969	42.7429389	38.4164066	34.7797181	43.5963293	32.7728884
16.2964406	18.5573702	15.9043493	17.8472356	17.3898591	12.5855454	14.647462
3.91640267	2.92589207	3.78674985	4.39221396	3.10370188	3.90151908	6.08751933
1.5770749	1.24250211	1.2306937	1.70120963	1.14106687	1.61094981	0.98379016
22.2893252	23.9682665	23.8091897	26.5388703	22.1823399	24.4411292	14.8105396
0.68339912	0.28056499	0.18933749	0.24744867	0.31949872	0.45307964	0.83349161
1.18280617	0.32064571	0.94668746	0.64955277	1.00413884	1.25855454	1.23932281
2.81245024	2.36476208	4.54409981	3.00031517	2.41906176	5.31110017	0.84015359
16.1650177	13.5873618	13.8216369	15.4655421	14.5143706	11.5787018	10.4987673
2.78616565	1.96395495	2.27204991	2.44355565	2.14520571	3.17155745	5.61958024
48.2322073	48.0166945	49.8430948	48.9948374	48.1073792	43.6466715	13.8794987
0	0.08016143	0	0.03093108	0	0.07551327	0
12.1960459	10.8618733	11.170912	7.60904672	11.3193833	13.1896516	6.27122461
29.9381385	35.1507855	28.7792988	38.6019931	25.0121858	24.4914714	25.9093626

119.463424	118.598831	99.1655116	127.497929	92.7915577	98.5951628	67.7505086
32.4351738	28.8981943	28.7792988	31.209464	27.0204634	28.3929905	11.3041226
0.76225287	1.2024214	1.42003119	1.02072578	1.27799489	1.38441	0.36232862
59.9814153	55.912595	67.0728066	57.8101964	66.227521	67.2068125	58.6291507
35.5367544	38.0365969	41.4175764	35.5398158	40.8045512	44.2004355	76.1991032
25.2069138	25.8520601	27.9272801	24.2809011	26.9291781	24.2397605	27.2878287
26.4685737	26.7739165	31.1460175	29.5701165	25.1491138	33.6285774	95.3714771
46.9705474	46.7741924	46.7663606	46.4584885	34.0494353	52.7837775	211.633072
15.0873499	23.4071365	19.1230867	18.0328221	21.1325584	21.9995334	27.1214297
28.8867552	30.6216649	35.0274361	31.9208789	30.2154507	36.4729106	120.193001
19.0037525	14.7497025	20.306446	20.0124115	15.9749362	21.3954272	118.00513
476.933734	528.103478	507.897823	474.390039	469.115411	501.735354	820.571046
320.540473	307.018264	304.975366	335.540402	204.981252	331.478095	576.635942
33.3025649	31.7038442	38.2935078	33.9313994	33.2278672	31.9672854	33.0267834
29.7278618	26.3330286	33.8440767	32.6322939	26.7922501	34.7109343	138.800212
28.0719332	31.1827949	31.71403	29.6010476	29.3938825	36.2211997	175.652084
23.0515781	20.1605988	27.6906082	25.2397647	20.0827769	29.6263739	82.4522107
0.68339912	0.84169498	0.42600936	0.68048385	0.50206942	0.52859291	2.03652152
68.7604656	80.0812651	82.4091435	68.5123516	78.8705419	73.5750985	21.3296982
3.99525641	4.04815204	3.31340611	4.39221396	3.46884328	4.37976981	5.73968096
4.54723263	4.24855561	3.97608734	4.94897348	4.38169677	4.45528308	8.7668443
24.1555305	21.3229395	22.2944897	25.3944202	19.3981368	19.7844774	18.0949815
146.536543	123.60892	124.347398	121.80661	159.658076	89.5335701	227.62666
2.41818151	2.1242778	2.17738116	2.28890023	2.23649106	1.73680527	1.98465336
0.36798414	0.20040357	0.23667187	0.27837976	0.13692802	0.25171091	0.17197692
20.4756891	21.0423745	20.2591117	21.4043103	17.4355017	20.0865305	15.5817163
6.3082996	7.33477053	7.52616532	7.6399778	7.34847063	7.29961634	7.33540875
89.1310164	77.1954537	78.7170624	90.3806281	90.9658507	74.4560867	39.8948627
69.549003	75.6723866	79.9950905	77.111193	68.053228	69.9756325	52.0834229
0.07885374	0	0.04733437	0	0.04564267	0.05034218	0.17618379
0.55197621	0.36072642	0.94668746	1.08258795	0.59335477	0.47825073	2.57719227
0.05256916	0	0.04733437	0	0.04564267	0.02517109	0.24221124
36.9035526	34.4293327	32.7080518	41.0764799	33.4560806	34.3081968	20.5618641
7.54367493	5.97202628	6.20080287	5.41293974	7.34847063	8.33163107	17.3297023
24.7075067	25.2909301	24.992549	25.3634891	27.2486768	25.498315	39.0243114
24.2606689	25.0504458	21.6791429	23.7241416	22.86698	21.4205983	16.5519905
57.1689651	69.0189882	70.2442096	60.3465453	75.492984	69.9756325	94.5692402
72.124892	68.6181811	59.3099694	65.9760027	64.2192433	61.0398953	28.1523032
118.41204	129.901592	124.252729	118.40419	131.633474	104.409685	37.5069149
0.05256916	0.28056499	0.18933749	0.12372434	0.45642675	0.12585545	0.0370646
3.5747031	2.64532708	3.31340611	3.49521252	4.60991015	2.44159581	20.0131884
53.9885307	58.8785678	50.884451	56.7585396	52.3977906	48.1774679	38.9177222
0.05256916	0	0.04733437	0.03093108	0	0.02517109	0.01551999
1.10395243	1.00201783	0.94668746	0.80420819	1.14106687	0.88098818	0.31080524
15.9284565	22.6055223	20.7797898	19.1772722	20.0827769	23.1825747	44.6904731

20.1865587	16.8338996	21.3951366	18.8988925	18.3939979	18.022501	23.7213449
25.8114592	30.8220685	28.2586207	28.9205638	28.7548851	23.5349699	18.4055832
23.4458468	22.6856837	20.306446	22.0847941	20.2653476	21.1185452	17.9817926
111.341488	119.560768	116.25322	118.466053	125.517356	117.347625	52.6487834
1985.40587	1933.21304	1979.38148	2012.19075	1936.98383	1650.82082	1698.99809
4.49466346	4.56920131	4.16542483	4.73245589	4.38169677	5.13490253	9.09554281
0.21027665	0.08016143	0.33134061	0.30931084	0.27385605	0.17619764	0.20828205
53.8833924	51.583878	54.5291978	55.5831584	52.0326492	58.7996682	101.329378
244.762024	278.560957	271.273292	260.810902	249.984929	248.463838	236.690735
0.60454538	0.68137213	0.80468434	0.64955277	0.59335477	0.57893509	0.73220937
0.15770749	0.08016143	0.04733437	0.03093108	0.09128535	0.276882	0.17594277
0	0.04008071	0	0.06186217	0	0	0
90.3663917	103.488402	86.8585746	92.3602175	94.1151953	83.4421661	26.4775752
4.4420943	5.61129986	5.39611853	5.50573299	3.69705665	4.90836271	6.40418214
121.093068	124.570857	148.014585	115.218289	124.148075	137.786551	187.152706
19.3717367	23.4071365	24.2825334	25.9511797	17.2072884	20.1368727	10.840421
52.963432	51.7041201	53.1565009	57.6864721	46.5555282	46.1637806	38.9827837
15.6393261	13.6274425	12.0229308	14.0117812	14.1492292	11.0249378	8.03838542
0.02628458	0	0.09466875	0.03093108	0.09128535	0.05034218	0.07851903
0.18399207	0.28056499	0.28400624	0.21651759	0.22821337	0.17619764	0.18121964
5.51976215	4.72952416	4.82810605	4.91804239	5.88790504	5.36144235	6.32825094
0.55197621	0.52104927	0.33134061	0.52582843	0.13692802	0.50342182	0.66198468
6.33458418	5.57121914	6.62681223	6.83576961	6.93768656	5.94037744	7.66848446
0.02628458	0.04008071	0.04733437	0.03093108	0.04564267	0.07551327	0.03084273
0.02628458	0	0	0.03093108	0	0.02517109	0.0135669
0.73596829	0.32064571	0.14200312	0.49489735	0.22821337	0.32722418	0.54580125
282.532968	269.222151	264.883152	280.668658	282.938941	217.302027	134.744023
5.62490047	4.60928202	4.54409981	4.20662745	6.38997446	5.61315326	4.15134716
0.31541498	1.04209854	0.18933749	0.61862168	0.31949872	0.30205309	0.22524372
0.02628458	0	0	0.03093108	0	0.02517109	0.01249409
31.541498	18.4371281	28.0219489	32.4157763	25.6055405	25.1459197	26.2477891
0.10513833	0.04008071	0.04733437	0.03093108	0.13692802	0.12585545	0.05777815
8.8841886	8.65743406	10.2242246	9.55770503	8.44389483	8.10509125	7.66154183
20.8436732	19.3189038	18.8390805	22.3013117	16.2487922	17.1666839	14.2804584
41.3193624	36.1127226	39.0035234	40.6434447	33.95815	37.1021879	36.6536385
244.893447	245.694772	219.252816	232.014063	239.852256	205.521957	163.791442
220.422502	236.55637	246.848756	225.0855	247.292012	198.67542	172.73517
22.0264794	17.5954331	21.5844741	20.1361358	19.6719928	18.3497252	15.5974923
0.21027665	0	0	0.09279325	0.04564267	0.02517109	0.18599301
0	0.04008071	0.14200312	0.03093108	0.04564267	0.02517109	0
74.9899114	66.7343876	64.5167505	75.4718455	57.2815568	64.8155589	74.1884791
21.2116574	19.1986617	16.2830243	20.1052047	23.7798335	16.2353536	20.1772225
8.91047318	6.93396339	6.86348409	6.4645966	7.66796935	7.22410307	7.30077068
0.07885374	0	0	0	0	0.05034218	0.03306277
15.9021719	13.9881689	15.47834	15.6511286	16.0205788	15.9836427	9.26328225

9.173319	9.01816048	7.28949345	8.0420819	7.71361203	8.18060452	6.89710267
22.2630407	19.8399531	20.7797898	23.8478659	23.4146921	18.7272916	22.4056354
5.70375422	6.77364054	6.72148097	5.35107757	6.02483306	6.51931253	7.2042525
85.5825978	78.5982787	94.1007336	90.3187659	93.9782673	61.9208835	40.2042749
58.6146171	58.1170342	61.2980131	59.5732682	62.7586778	62.6760162	56.7714948
37.7446592	36.2329648	32.2347081	43.3963112	30.5805921	37.7818073	22.1026739
275.304708	225.133366	217.548779	242.097596	272.486768	239.226047	151.66683
0	0.08016143	0.04733437	0.06186217	0.04564267	0.05034218	0
1.23537534	1.08217926	1.32536245	1.36096771	1.18670954	0.75513273	0.71367956
29.5701544	28.09658	25.2292208	31.4569127	28.7548851	26.7316985	15.7067887
0.44683789	0.56112999	0.09466875	0.24744867	0.63899745	0.50342182	0.2860336
0.07885374	0.04008071	0.09466875	0.06186217	0.13692802	0.10068436	0.08601037
73.5179749	78.6383594	91.9233525	76.6472267	77.2274056	80.2454376	129.493817
8.48991987	9.69953261	8.28351529	8.41325491	9.08289227	7.55132725	3.63069182
68.734181	67.936809	68.256166	69.1619043	67.6880866	59.9323673	50.3331407
84.3735071	78.9990858	74.5043032	85.7409655	84.4845909	76.5704583	28.7701376
25.1280601	24.6095579	25.2765552	26.074904	22.9582654	22.9308638	12.0591549
0.02628458	0	0	0.06186217	0	0	0.03133873
42.5284531	45.3312867	36.542136	41.4785839	49.1571607	40.173061	17.9425523
95.2816085	74.1493195	85.2965403	95.6698435	78.2315445	92.4534166	82.024341
10.7241093	13.5072004	12.7802807	10.7330862	11.0455273	12.8120852	18.6875285
385.542244	392.710829	422.837955	419.487364	385.817529	451.745567	835.47945
156.077846	149.541141	121.886011	128.982621	157.969297	162.177338	107.625553
638.321065	720.370659	735.008145	693.22746	725.216459	571.383762	464.652639
0.02628458	0	0.14200312	0.12372434	0	0.07551327	0.60100078
0.05256916	0.04008071	0.04733437	0	0	0.07551327	1.02074736
0.13142291	0.20040357	0.14200312	0.21651759	0.1825707	0.17619764	2.74816596
0.07885374	0.16032285	0.04733437	0.12372434	0.04564267	0.17619764	1.49551357
0.02628458	0.08016143	0.14200312	0.12372434	0.09128535	0.07551327	2.92304924
0.39426872	0.20040357	0.18933749	0.15465542	0.3651414	0.20136873	18.2001179
17.0324089	19.5994688	17.9870618	14.1973677	19.5807075	18.1231854	23.2928955
11.9594846	9.81977475	12.9222838	10.6093619	9.85881774	12.182808	38.4996354
4.38952514	3.96799061	4.44943107	4.05197203	3.37755793	4.4049409	27.3264196
4.28438681	2.64532708	3.97608734	3.61893685	3.05805921	5.28592908	3.84803766
157.549782	181.886277	195.538295	168.32696	192.931586	201.595266	594.840521
24.7863605	27.4552886	27.5012707	25.2706958	27.4312475	30.9100995	29.6535842
1234.27139	1361.94264	1337.0067	1260.37982	1448.19643	967.098481	444.666768
0.02628458	0.04008071	0.04733437	0.06186217	0	0	0.016489
0.07885374	0.32064571	0.14200312	0.12372434	0.09128535	0.17619764	0.078551
40.8462399	36.7139333	31.3826893	44.5098302	30.03288	39.7954946	30.5980427
4.81007844	5.21049272	3.40807486	4.48500721	3.19498723	4.6818229	2.83844579
0.02628458	0	0.09466875	0.03093108	0.09128535	0.05034218	0.07900133
4.12667932	3.76758705	3.55007798	3.43335035	3.46884328	3.77566363	3.13644364
0.34169956	1.16234068	0.80468434	0.68048385	0.63899745	0.62927727	0.50820187
16.3752944	14.4691375	16.23569	17.0120963	15.2446534	12.0821236	10.0258541

4.38952514	5.81170342	4.07075608	4.94897348	4.3360541	3.90151908	1.4959302
22.4996019	20.0804373	19.6437648	22.9817956	19.1699234	17.191855	13.2228833
20.6596812	17.6355138	19.1704211	21.311517	18.7134966	20.4137547	34.6201147
11.3286547	12.9861511	11.0762433	11.6919498	11.3193833	10.5970292	5.45275486
1.41936741	1.5230671	0.80468434	1.5774853	1.09542419	1.56060763	0.73963419
0.10513833	0.68137213	0.7100156	0.40210409	0.22821337	0.50342182	0.11498808
1.91877446	1.40282496	1.56203431	1.60841638	1.59749362	2.16471381	3.98169388
0.89367578	0.9218564	1.18335933	0.24744867	0.68464012	1.53543654	3.42702325
0.39426872	0.40080713	0.28400624	0.34024193	0.45642675	0.47825073	0.3541139
0.13142291	0	0.14200312	0.03093108	0.1825707	0.17619764	0.37650517
25.6800363	23.9682665	20.7797898	25.6727999	20.2197049	21.0430319	13.7479257
58.8511783	64.9307555	61.4873506	51.6549107	60.5678294	64.4128214	35.4377454
49.888136	48.5778245	54.1978572	52.520981	47.3770964	58.9758658	154.499803
0.91996036	0.68137213	0.80468434	1.02072578	0.91285349	0.80547491	0.52784895
5.30948549	5.81170342	5.77479351	5.04176673	6.07047574	6.97239216	11.692197
0.13142291	0.24048428	0.28400624	0.12372434	0.09128535	0.25171091	0.09575206
71.1786471	77.1152923	66.362791	68.9453867	68.3270841	68.4905382	57.5871633
157.891482	144.851698	148.819269	137.148427	136.243384	156.513843	108.937577
14.6142274	14.2286532	13.9636401	14.0117812	13.8297304	17.3932238	28.5580977
40.8199553	43.4875739	39.8082077	40.9527555	38.9788442	39.3927572	32.2991269
9.67272605	9.73961332	9.6562121	10.6712241	7.16589993	9.3384747	5.55516589
0.07885374	0.04008071	0.04733437	0	0.04564267	0.07551327	0.12080229
64.2658021	64.8105133	66.1261192	62.9138253	64.6300274	60.133736	53.516276
0.2891304	0.52104927	0.23667187	0.43303518	0.68464012	0.20136873	0.17609607
0.02628458	0	0	0.06186217	0	0	0.01043777
80.2731124	71.7845574	82.5511466	88.5556941	76.8166216	78.4079479	72.8734073
8.35849696	7.73557766	8.6148559	8.93908334	8.07875343	8.96090834	8.4328464
0.02628458	0.12024214	0	0.03093108	0	0.05034218	0.05288411
9.40988023	8.97807977	9.75088085	9.15560093	9.35674832	11.2766487	10.1507654
12.4588917	14.1885725	9.94021834	14.7850583	8.4895375	10.4711738	3.08018972
42.3181765	42.9264439	41.6069139	42.5302408	48.1530218	41.9098662	9.55202548
0.31541498	0.64129141	0.5206781	0.40210409	0.5477121	0.32722418	0.3242374
42.0290461	52.9065415	43.2162826	48.840182	50.2069422	31.0107839	26.0849889
40.7673861	43.9284617	42.4115983	44.9119343	37.6552067	38.9145064	36.9819378
48.2847765	50.3012951	47.8550512	40.9218244	48.1073792	51.4245386	31.6199444
67.8930744	69.8206025	61.5820194	60.965167	64.8582408	65.5958627	122.31605
16.8221323	17.2347067	18.7444117	19.2700655	18.3483552	17.5442503	16.7371018
140.622512	135.633134	152.558684	132.508765	155.45895	121.14846	100.803661
0.2891304	0.56112999	0.18933749	0.30931084	0.1825707	0.35239527	0.45027239
35.9310231	36.3131262	42.0329233	34.4572278	37.1987799	38.1342026	72.3520847
13.5891287	14.8699446	14.2476463	12.6817445	14.6512986	13.3910203	22.4185854
8.72648111	8.37686907	10.3188933	10.0835335	9.53931902	9.26296143	8.31384411
0.23656123	0.20040357	0.18933749	0.18558651	0.27385605	0.10068436	0.28440479
2.20790486	1.96395495	2.22471553	2.04145156	2.14520571	1.71163418	1.26151214
475.777212	574.757428	515.092648	528.148263	538.264063	408.627489	156.791826

116.072713	117.075763	107.023017	119.177468	116.936533	86.4123548	68.9104781
0.13142291	0.20040357	0.33134061	0.21651759	0.04564267	0.20136873	0.15646492
124.194648	124.129969	135.754982	128.797035	137.110595	97.3366083	77.473475
3.18043438	2.7254885	4.2127592	4.7015248	2.41906176	3.95186126	2.39641426
0.18399207	0.04008071	0.09466875	0.18558651	0.09128535	0.15102655	0.23384394
2.75988107	2.64532708	3.88141859	2.66007324	2.37341909	3.27224181	1.33999678
39.3217341	36.6337719	31.0040144	32.8488114	34.0037927	35.5164092	8.77928424
23.997823	26.693755	25.4185583	25.7655932	26.1532526	20.8668343	9.22570193
20.2128433	23.1666523	20.0224398	20.4145156	21.4520571	18.6014361	26.6876131
21.0013807	22.084473	23.9038584	23.6622794	21.2694864	24.7935245	42.1158685
23.6035543	30.9823913	25.5132271	27.0337676	28.070245	28.6950436	21.6689534
137.021524	123.288274	131.258217	120.693091	128.438487	126.585416	221.421946
15.0084961	17.6355138	15.3836712	15.4964732	15.5641521	14.6244038	8.09509361
196.056695	232.788783	231.275747	204.918433	230.632435	193.389491	146.061673
55.6181748	45.1308831	47.713048	49.3350793	54.2234976	51.7769338	74.2355638
9.01561151	8.21654622	8.70952464	6.68111419	9.40239099	8.30645998	5.24425335
34.8007861	42.2851525	38.3408422	37.3647497	38.7506308	34.1068281	14.4260553
16.5592864	17.4351103	16.2830243	17.5997869	19.1242807	16.4618934	6.8585513
33.8808257	34.2690098	32.2820424	32.2920519	34.232006	38.0586893	27.3570398
33.4077033	38.5576462	32.4713799	36.4986794	34.095078	29.1481232	35.5057789
61.821336	59.2793749	63.9014036	55.3047786	66.6383051	55.4519131	18.6108355
0.21027665	0.12024214	0.14200312	0.21651759	0.22821337	0.17619764	0.06447633
75.6207414	68.9789075	70.1968753	70.6156653	68.3727267	67.886432	58.9396237
0.42055331	0.32064571	0.56801248	0.27837976	0.59335477	0.276882	1.05964298
22.2104715	28.65771	25.7025646	27.7761136	23.8711189	28.2923061	23.0447351
58.8774629	57.1550971	54.434529	60.8723738	56.414346	60.2595915	28.4623329
205.15116	214.63222	202.543782	207.176402	224.014248	219.391228	129.393345
0.02628458	0.08016143	0	0.03093108	0	0.02517109	0.03153854
4.65237095	4.72952416	4.07075608	4.67059372	3.4232006	4.37976981	4.16478366
1.47193657	3.48702205	2.69805926	2.90752192	2.46470444	2.94501763	1.11907914
395.030978	413.272234	371.05415	401.609198	374.315575	354.056564	823.229264
330.949168	288.060086	257.972333	292.731781	280.20038	264.321625	181.626399
1.55079032	0.64129141	1.08869058	0.46396626	1.41492292	1.00684363	0.40928996
76.461848	85.8128071	80.563103	85.8646898	73.9867757	73.3989009	96.031471
30.7529605	30.6617456	26.8859239	33.4983642	30.5805921	28.3426483	21.2001374
0.05256916	0.08016143	0.04733437	0.06186217	0	0.05034218	0.03103624
0.13142291	0.24048428	0.04733437	0.09279325	0.1825707	0.07551327	0.0807675
0.36798414	0.20040357	0.33134061	0.21651759	0.27385605	0.276882	0.35839935
2.41818151	1.32266354	2.50872177	1.97958939	1.27799489	2.24022708	5.00105805
0.02628458	0	0	0	0	0.05034218	0.10063706
15.0873499	13.9080075	11.5969214	15.6820597	12.6886636	14.4985483	3.0323064
0.76225287	0.56112999	0.7100156	0.61862168	0.91285349	0.830646	0.37843048
5.30948549	5.93194556	4.30742795	6.68111419	4.38169677	5.63832435	4.54992324
32.8031579	30.1406964	34.0334142	35.5398158	29.5764532	35.9946599	62.6504606
184.544048	187.577738	191.704211	187.627957	181.247061	173.655356	73.4863334

9.01561151	7.49509338	12.2122683	9.71236045	8.39825215	12.6862298	34.626891
92.232597	100.081541	108.821724	91.6797336	98.8163908	97.9407144	50.4366463
13.2737137	11.9841333	12.9222838	12.9291932	12.5060929	10.8487401	9.77576072
0.02628458	0	0	0.03093108	0	0.02517109	0.05417614
20.0025666	41.2029732	35.3587767	51.2528066	18.94171	37.0015035	27.7424549
336.311222	326.697894	302.41931	313.053503	336.067014	274.339719	135.285947
10.8555322	11.463084	10.7449027	10.26912	11.7301674	12.8624274	16.2439299
3.23300354	4.5291206	3.36074049	2.84565975	3.74269933	3.72532144	2.14240825
14.9033578	12.7055861	15.0049963	14.5994718	14.6512986	10.8487401	13.1111529
0	0.04008071	0	0.03093108	0.04564267	0	0
0	0	0.04733437	0	0.04564267	0.02517109	0
0.05256916	0.08016143	0	0.06186217	0.04564267	0	0.11213092
0.02628458	0	0	0	0	0.05034218	0.02798394
0.02628458	0.08016143	0.18933749	0.06186217	0	0.10068436	0.01881424
1.20909076	1.40282496	0.99402183	1.23724337	1.73442164	1.107528	1.70693932
6.09802294	5.49105772	6.05879975	6.06249251	5.93354771	6.67033907	17.5934473
23.3932777	20.3610023	20.7324554	21.7445522	22.9126227	22.729495	19.3486492
26.4422891	27.5755307	29.820655	28.2400799	25.1034711	26.4799876	38.0546623
5.51976215	4.36879775	4.02342171	3.9282477	4.97505155	6.34311489	3.97892973
0.55197621	0.32064571	0.33134061	0.49489735	0.41078407	0.52859291	0.61805435
121.56619	107.857199	110.904436	118.187673	108.949065	99.6523486	47.1646465
4.25810223	4.16839418	3.26607174	4.60873155	3.28627258	2.91984654	3.60850277
22.4207481	22.5253609	26.1759083	23.3529686	20.904345	23.4342856	75.9749891
29.4124469	26.6536743	29.0159707	31.1476018	27.7963889	29.5760317	19.047016
196.95037	205.734301	207.797898	191.308756	201.421124	190.998237	131.653818
18.2414997	15.230671	13.9636401	16.5481301	14.4230852	18.9790025	16.2110846
51.7806258	48.0968559	51.4051291	48.0669049	47.6053097	50.165984	27.4091203
9.98814103	11.1424383	8.18884654	10.3619132	11.2737407	9.48950124	7.20846362
28.0982178	27.3751272	26.6965864	30.1578071	27.4312475	28.3929905	32.7978398
56.2752893	47.1349188	63.2860568	60.8105116	41.6261194	55.4770842	60.2017776
75.4367493	72.5861717	76.5396812	77.9772633	75.3104133	80.5223196	40.026313
0.02628458	0.08016143	0.09466875	0.06186217	0	0.05034218	0.03373929
2.60217358	2.36476208	2.03537804	1.94865831	2.00827769	3.82600581	3.74935292
0.02628458	0.04008071	0.04733437	0.03093108	0.04564267	0.07551327	0.01103416
35.3001932	36.5536105	39.4295328	36.6842659	42.4476875	37.5300964	28.325488
0.21027665	0.08016143	0.18933749	0.03093108	0.31949872	0.276882	0.74343449
51.0709421	50.5818601	51.4997979	57.5318167	58.0574823	45.2576213	22.6397288
33.5391262	36.0726419	33.6547392	38.8803729	31.0370188	28.820899	10.9656339
2.1553357	1.64330924	2.03537804	1.45376096	2.09956304	1.78714745	2.23629382
23.0252935	18.9982581	19.1230867	16.6099922	26.0163246	14.8257725	26.914957
2.70731191	2.24451994	2.74539364	3.21683276	2.51034711	2.61779345	20.3803987
57.2741034	61.8846213	59.8779819	60.2846832	58.0118396	54.2185297	30.1474042
0.02628458	0	0	0	0.04564267	0.02517109	0.02448861
6.99169872	7.09428625	7.57349969	7.36159805	7.34847063	8.25611779	6.30043616
51.5703492	50.9826673	43.9262982	52.8302919	45.323176	41.6581553	24.787917

206.018551	218.399807	207.229885	214.599862	202.699118	188.682497	76.1733292
5.99288462	6.93396339	6.39014036	6.2790101	6.52690249	7.72752489	5.11406174
66.7102682	70.3817325	70.2442096	72.9045655	69.7876497	72.0144909	56.5136349
2.89130398	3.20645706	2.93473113	3.3405571	3.74269933	2.81916217	5.08904976
71.2312163	75.5120638	75.2616532	75.0388103	76.9535496	60.2092493	34.5298585
26.7839887	29.9402928	29.1106394	26.7863189	29.8959519	25.7500259	15.3896003
2.26047402	1.88379352	1.04135621	1.5774853	1.27799489	1.83748963	1.14005549
0.23656123	0.08016143	0.18933749	0.24744867	0.04564267	0.10068436	0.3152308
10.4612635	12.3448597	12.5909432	11.4135701	10.5434579	11.4025041	10.8496054
12.3011842	12.2246175	12.8276151	12.0940539	13.7840878	13.3658492	19.7998125
0.34169956	0.24048428	0.23667187	0.09279325	0.22821337	0.65444836	0.36892855
6.17687669	4.88984702	4.54409981	4.79431806	4.42733945	5.26075798	11.063078
0.02628458	0.08016143	0.04733437	0	0	0.20136873	0.01508494
3.8638335	2.48500422	2.84006238	3.46428143	2.96677386	3.64980817	3.43625636
2.12905111	2.16435852	1.9407093	1.5774853	2.60163246	1.61094981	10.8292594
7.93794366	7.89590051	7.71550281	9.68142936	6.84640121	8.30645998	3.8825948
2.39189693	2.76556921	3.97608734	3.37148818	2.92113118	3.5742949	2.08998021
9.62015688	9.21856405	11.4075839	9.74329153	13.053805	9.56501452	5.87088863
0.47312247	0.6012107	0.66268122	0.46396626	0.91285349	0.60410618	0.79774466
0.05256916	0	0.04733437	0.03093108	0.04564267	0	0.0986305
0.26284582	0.16032285	0.18933749	0.15465542	0.27385605	0.12585545	0.09240851
23.498416	24.7698808	25.5132271	22.022932	25.3773272	22.8301794	18.8555371
88.2899098	85.1715157	80.0424248	81.7199245	82.6132413	100.860561	22.6233235
9314.09921	10918.0665	10018.1307	9421.42267	11752.7605	6711.69517	218.439425
0.05256916	0.20040357	0.37867498	0.30931084	0.3651414	0.10068436	0.10533511
27.0994037	26.5334322	28.3532895	33.405571	18.3027126	25.6745127	41.6722834
35.1424857	30.4613421	35.5007798	39.4062013	31.995515	34.7864475	48.3841139
0.05256916	0	0.04733437	0.03093108	0	0.02517109	0.06405088
24.5760838	25.3710915	24.0458615	24.5592809	24.1906176	22.3519287	34.3976676
28.5450557	29.2589207	30.1046613	29.1680124	28.481029	34.2075124	23.4905794
50.4663968	43.3673317	52.7304916	53.0777405	48.0617365	51.1728277	117.478211
75.2527573	72.9468981	71.048894	74.2346021	69.7876497	67.4585234	32.7599964
99.2505803	109.139782	99.3548491	95.2368083	100.733383	100.105428	31.3887729
50.0195589	49.9806494	48.1390574	52.7374986	47.6965951	53.5389102	48.4850949
8.67391194	7.29468981	7.19482471	8.41325491	6.07047574	10.3704894	42.9581731
69.4175801	78.6784401	77.5810375	69.6877328	71.5220713	75.8153256	118.682744
78.1177767	64.4898676	69.3448565	76.2451226	70.5179325	75.2615616	39.9917664
111.078642	103.809047	110.809767	113.981045	114.928255	110.199036	101.897913
91.2337829	99.4803303	102.573586	100.12392	97.3558252	84.7258917	60.5561276
45.3934725	43.327251	38.2461734	47.2008345	44.9580346	41.0288781	33.3809236
29.4913006	35.3111084	37.9148328	30.1887382	34.2776487	33.3265243	38.9803066
0.02628458	0	0	0	0.09128535	0	0.06417873
71.8094771	43.0066053	54.434529	34.2407102	53.8583562	98.2931097	71.7675457
11.118378	10.8217926	10.3188933	10.8877416	11.9127381	11.1759643	5.90447065
1.78735155	1.5230671	1.46736556	1.63934746	1.27799489	2.36608254	1.35425261

1.78735155	1.5230671	1.46736556	1.63934746	1.27799489	2.36608254	1.34882223
1.78735155	1.5230671	1.46736556	1.63934746	1.27799489	2.36608254	1.36396808
1.78735155	1.5230671	1.46736556	1.63934746	1.27799489	2.36608254	1.36311773
1.76106697	1.44290568	1.46736556	1.5774853	1.23235222	2.36608254	1.87901203
1.78735155	1.5230671	1.46736556	1.63934746	1.27799489	2.36608254	1.35972689
99.145442	101.444285	83.7818403	86.5142426	104.293512	80.4971485	46.6832791
0.76225287	0.40080713	0.28400624	0.74234602	0.50206942	0.52859291	0.47010472
0.05256916	0.04008071	0	0.12372434	0	0.02517109	0.04535055
13.9833974	15.6314782	13.2536245	14.1045744	14.6969413	16.5877489	24.4192494
41.1879394	38.1969197	45.67767	45.2831073	36.6967105	49.3856802	213.042705
952.7898	802.896848	956.343673	970.339043	877.069638	1016.40865	1775.37507
0.23656123	0.20040357	0.75734997	0.12372434	0.13692802	0.60410618	0.85742778
32.5403121	38.0766776	39.1455265	31.7662235	38.2942041	35.0129874	23.0693043
344.748573	386.859044	402.720846	352.305049	409.095294	316.853691	179.993962
16.7958477	17.5152717	15.2416681	17.4760626	19.0329954	15.6815896	7.97907306
155.079032	149.140334	153.600041	165.048265	160.707858	104.787251	31.8779862
34.8270707	44.4094303	44.3049732	35.4160914	41.7630474	40.8275093	53.2210403
0.26284582	0.44088785	0.28400624	0.30931084	0.41078407	0.15102655	0.37761059
95.3078931	107.977442	92.7753712	93.999565	93.9782673	95.4991186	107.752996
21.9476257	16.2326889	21.6318085	19.5793763	21.1325584	22.5281263	32.5827759
27.7828028	31.1026335	22.3418241	28.0854245	23.9167616	25.0955776	15.3645088
0.5782608	0.52104927	0.23667187	0.27837976	0.31949872	0.553764	0.33765056
202.811832	187.176931	196.958326	205.660779	203.566329	203.53344	149.681284
0.07885374	0	0	0.03093108	0	0.02517109	0.13159703
26.9154116	29.2990014	28.3059551	24.3427633	25.3316845	30.759073	55.2900532
3.73241059	3.80766776	5.68012477	4.29942071	3.19498723	4.45528308	3.35720803
6.91284497	7.2546091	8.18884654	6.68111419	8.39825215	8.55817088	7.43091517
58.9300321	62.5659934	72.3742564	64.1510687	58.7877651	63.3556356	51.992961
26.4422891	29.77997	34.128083	30.9620153	28.1615303	27.2602914	22.4161212
6.12430752	5.41089629	7.19482471	8.19673732	5.52276364	6.41862816	5.8347159
35.7996002	39.9203904	39.2875296	38.7875796	36.4684971	35.2143561	31.1030709
4.81007844	5.65138057	6.01146538	5.96969926	5.0663369	6.51931253	6.22326613
0.05256916	0	0.04733437	0.03093108	0	0.02517109	0.09436109
1.91877446	2.00403566	1.7513718	1.63934746	1.55185094	1.98851618	2.24184197
98.4357583	98.5985546	89.9826432	98.1752613	103.92837	97.2359239	134.167146
15.3764803	17.5954331	20.5431179	18.7751681	16.7965043	20.4640968	36.9544634
5.94031545	5.05016987	4.97010917	5.19642215	5.65969167	4.05254562	7.22695218
31.7780592	28.09658	27.9272801	28.7040462	26.7922501	28.5188459	26.3193175
4.46837888	3.00605349	3.171403	4.02104095	4.19912608	3.52395272	6.07344676
5.151778	5.21049272	5.82212789	5.44387082	5.70533434	5.96554853	3.38367472
0.18399207	2.04411638	0.47334373	1.29910554	1.87134966	0.22653982	0.07569356
140.044251	142.246451	128.228817	148.159893	134.280749	107.505729	96.1358418
34.432802	34.389252	36.1161266	33.7148818	34.7340755	32.0931408	6.5834854
36.1150152	30.3010192	32.9447237	37.2100943	29.8503093	37.2783855	18.4926606
94.177656	96.7949225	97.8874835	108.104139	82.7501693	85.9592752	83.2113686

135.365595	144.851698	105.366314	132.972731	138.890659	93.4350892	38.1194152
0	0	0.04733437	0.03093108	0.04564267	0	0
88.6841785	85.2917578	90.8819963	90.1022484	79.2356833	85.2796557	90.1046924
13.7205516	12.9460704	12.4962745	11.8775363	12.9168769	12.4093478	10.3414348
13.8782591	12.0642947	10.0348871	10.5165686	12.1409515	11.3018198	6.63815054
0.42055331	0.20040357	0	0.30931084	0.41078407	0.17619764	0.27815986
22.683594	25.5314143	21.3478023	22.7962091	24.3275456	19.6334509	10.0885317
18.4517763	15.8318817	17.6557212	17.8781667	16.842147	14.7754303	14.5109523
26.6262812	28.7779521	29.0159707	28.271011	30.7631628	29.1732943	28.1031387
135.549588	137.957815	140.204413	151.129278	125.973782	116.894546	79.5470446
6.91284497	7.29468981	6.6741466	7.48532238	6.38997446	5.7641798	3.74010679
6.99169872	6.25259127	6.01146538	6.58832094	6.89204388	7.07307652	7.85745713
136.075279	122.125933	121.696673	130.529175	118.123242	113.672646	273.107277
9.64644146	10.3809047	13.064287	9.31025635	10.4521725	11.1004511	15.1870654
31.8043438	32.9062656	33.0867268	37.5812673	32.3150137	32.5462204	19.0808168
533.366731	565.458703	531.044331	537.365726	509.007109	501.156419	627.968881
125.929431	132.987807	138.263704	133.993457	130.218551	114.553634	92.7718267
16.2964406	14.9100253	12.8749495	15.6820597	14.1035865	12.0821236	5.54371408
45.4986108	42.766121	46.3876856	49.4278726	41.1240499	38.9145064	13.3120738
7.3071137	5.53113843	6.24813724	7.94928865	5.61404899	6.94722107	5.14012915
5.57233131	4.56920131	4.40209669	4.82524914	4.97505155	5.81452198	3.04174513
109.159868	121.885449	122.264686	109.743487	119.44688	108.084664	63.8306207
52.1748946	53.94864	59.2153007	51.778635	54.7712097	52.0789869	65.0609381
31.2786522	34.389252	27.1225958	31.4878437	30.8544481	26.4548165	11.4851312
14.7982195	11.6234068	11.6442558	13.6406081	11.501954	11.0501089	11.7205853
71.2049317	88.0974077	100.254202	64.6768971	89.5965705	91.2955465	210.905434
9.90928728	8.4570305	8.70952464	10.0216713	9.99574577	8.98607943	7.78291186
62.8464347	62.0048634	59.9726507	60.0063034	61.0698988	57.364916	33.8078796
2.78616565	2.64532708	2.74539364	3.21683276	2.96677386	2.7436489	4.92169736
5.41462382	4.76960488	4.07075608	4.85618022	4.19912608	4.22874326	2.96491924
0.05256916	0.08016143	0	0	0.04564267	0.05034218	0.05171458
1.20909076	2.32468137	0.99402183	1.29910554	2.09956304	1.68646309	0.21321363
23.9715385	22.365038	23.0045053	25.4253512	23.6885482	23.3839434	10.0373199
6.25573043	6.61331769	5.20678104	6.30994118	5.84226237	7.02273434	4.43368651
0.26284582	0.24048428	0.18933749	0.21651759	0.13692802	0.45307964	0.22860677
3.78497976	4.68944345	3.6920811	4.82524914	4.60991015	3.70015035	3.98975442
39.7948566	45.3312867	44.3049732	35.3851604	45.6883174	42.1364061	6.99482177
23.6298389	25.4111722	24.8032115	22.0538631	24.1449749	24.3152737	21.2310202
107.346231	106.454374	90.5033213	112.279836	108.720851	96.8583575	88.0127776
29.149601	27.0945622	26.2705771	28.2400799	30.03288	27.6630288	20.0722081
0.42055331	0.36072642	0.61534685	0.55675952	0.1825707	0.40273745	0.25156805
3.25928812	3.9279099	3.59741235	3.18590168	3.83398468	4.53079635	2.88289166
0.21027665	0.16032285	0.14200312	0.09279325	0.45642675	0.17619764	0.12107712
0	0	0.04733437	0.03093108	0.04564267	0	0
3.12786522	2.64532708	3.92875296	2.69100433	2.82984583	3.34775508	3.01756424

13.4577058	12.1845368	15.7623462	13.1766419	12.7799489	13.2399938	5.15907657
11.6703543	9.53920976	8.37818403	12.0321918	9.90446042	9.86706761	6.74040251
30.5426839	29.2589207	29.2999769	26.9100433	31.858587	27.0840937	22.7334441
24.6023684	29.1787593	23.0518397	29.8175652	30.2610933	20.9926898	9.84807428
10.1721331	12.304779	12.6382776	10.8568106	11.365026	11.0501089	6.28455587
3.81126434	3.48702205	2.4613874	3.95917878	2.64727513	3.97703235	2.70197829
2.05019737	2.28460066	2.36671865	1.94865831	1.82570699	2.34091145	1.82133352
3.73241059	4.48903989	4.16542483	4.5159383	4.47298212	4.22874326	2.3001526
0.65711454	1.2024214	1.2306937	0.64955277	1.04978152	1.00684363	0.19764901
0	0	0.04733437	0.03093108	0.04564267	0	0
0.05256916	0	0.04733437	0	0.04564267	0.02517109	0.04345073
13.3262829	14.5492989	12.9696182	12.496158	14.6969413	15.9081294	13.1891955
24.5497993	22.6856837	22.8151678	25.8583864	23.6885482	23.4342856	15.5241189
8.93675776	11.6234068	9.41954024	9.74329153	11.4563114	10.6725425	11.3699471
3.1541498	3.36677991	3.171403	1.7630718	2.37341909	4.45528308	1.09489926
0.21027665	0.16032285	0.14200312	0.12372434	0.27385605	0.25171091	0.12159222
7.49110577	6.21251056	7.57349969	7.42346021	6.48125981	6.14174616	14.8883174
5.20434717	4.28863632	4.54409981	5.56759516	4.3360541	5.16007362	10.0893839
0.02628458	0	0	0.03093108	0.04564267	0	0.03923556
99.5659953	109.981477	93.3433837	84.4418599	102.832946	101.842234	61.0514365
5.49347756	4.16839418	4.73343731	5.50573299	4.8837662	5.2104158	2.39021526
55.6444593	57.3555007	55.6652227	55.5522273	58.1944103	47.120282	17.7147815
28.7290477	26.6536743	23.477849	26.9719054	25.7881112	23.1574036	12.5940946
0.5782608	0.44088785	0.28400624	0.37117301	0.1825707	0.553764	0.93568508
0.10513833	0.08016143	0.04733437	0.03093108	0	0.12585545	3.89739899
29.3598777	31.3030371	24.5665396	27.9307691	27.3399622	26.7316985	19.0938363
11.118378	10.5011469	8.66219027	11.8775363	8.03311075	8.65885525	24.9329938
17.5055314	17.7156753	17.7977243	16.8574409	19.1699234	19.3062267	19.6822231
2.47075067	2.04411638	1.98804367	2.41262457	2.28213374	2.36608254	4.40266995
0.13142291	0.04008071	0.09466875	0.06186217	0.1825707	0.12585545	0.0699903
0.10513833	0.20040357	0.04733437	0.03093108	0.22821337	0.05034218	0.1760632
13312.0892	13909.0095	13603.4728	15415.5884	13027.4235	14895.3957	23430.9104
3.70612601	3.84774847	4.30742795	3.27869493	4.29041142	5.08456035	1.62060746
8.0167974	8.41694978	6.91081847	7.94928865	6.98332923	7.24927416	13.7639722
68.3661969	72.1052032	71.9482471	68.8216624	71.0200019	80.2957798	51.6721421
61.7424823	58.0368728	58.4106164	58.5216114	64.264886	62.0215678	41.913801
284.32032	268.861425	303.365997	280.111899	269.10921	274.213864	510.351959
103.902951	123.007709	91.4500088	110.98073	101.828807	92.1513635	80.983084
0.02628458	0.08016143	0.04733437	0.06186217	0.13692802	0.02517109	0.02152178
4.99407051	4.60928202	5.49078728	2.50541782	7.62232668	3.67497926	6.61882223
31.9620513	36.9143369	26.8859239	31.3331883	31.5390882	29.0222677	33.7931778
0.21027665	0.52104927	0.23667187	0.18558651	0.45642675	0.20136873	7.0473516
0.26284582	0.72145284	0.42600936	0.30931084	0.7302828	0.57893509	6.12448413
8.64762736	9.33880619	9.04086526	7.23787371	9.76753239	8.68402634	10.3609356
0.26284582	0.56112999	0.23667187	0.21651759	0.3651414	0.32722418	0.46599336

19.7660054	16.633496	18.4130711	15.6511286	18.1657845	18.6517783	9.6563352
6.28201501	5.49105772	4.82810605	4.94897348	5.43147829	5.36144235	3.27077951
19.7134362	19.5994688	22.2944897	21.2187238	17.8919285	19.7341352	30.7199443
52.963432	47.2150802	55.5232196	57.0987815	44.4103225	60.8888687	128.805937
3.28557271	3.12629563	3.45540923	3.30962601	3.28627258	3.90151908	1.89674031
0.49940705	0.28056499	0.28400624	0.27837976	0.3651414	0.32722418	0.09307089
12.1434767	10.1805012	11.0289089	12.0321918	11.8214528	11.2263065	11.2494784
0.55197621	0.6012107	0.80468434	0.34024193	1.00413884	0.88098818	0.82697413
0.13142291	0.08016143	0.18933749	0.03093108	0.09128535	0.17619764	0.12624084
393.821887	383.893072	341.233495	404.052753	363.361334	301.902063	159.919175
169.456698	184.3312	174.427165	168.141374	178.280287	161.29635	107.657171
4.52094804	4.44895917	3.40807486	3.89731661	4.24476875	3.5742949	8.34778743
16.6118556	15.1104289	12.4489401	15.0325069	16.2031495	15.3040232	16.368134
15.3501957	15.230671	17.2770462	15.8985773	14.1492292	15.6060763	8.23943324
44.8414963	32.3451356	33.8914111	27.7451826	53.036788	22.5029552	22.5504387
28.3610636	28.8180328	28.0219489	29.9103584	28.1158876	30.2304801	8.56422401
8.12193573	9.53920976	8.18884654	10.640293	10.1326738	6.77102343	5.39237144
44.6049351	48.5778245	46.813695	42.6848962	46.9663123	45.5345033	45.3382304
5.4409084	4.76960488	3.6920811	5.19642215	5.15762224	4.63148071	2.23535957
1.23537534	1.32266354	1.18335933	0.9897947	0.95849617	1.40958109	0.70354584
0.21027665	0.12024214	0	0.27837976	0.1825707	0.02517109	0.16956383
0.07885374	0	0	0	0.04564267	0.02517109	0.16982504
63.2407034	65.9327733	58.6946226	60.7177183	63.6715312	70.5797387	81.7774009
6.91284497	4.92992773	5.06477792	5.53666408	6.89204388	5.78935089	6.43805212
37.0875447	35.0706241	36.5894704	37.3956808	36.5597825	38.7131377	11.3026027
0	0	0.04733437	0.06186217	0	0	0
31.8831975	33.6277184	32.7080518	33.5911575	32.4062991	35.7681201	28.4791866
1.36679825	0.76153355	0.85201872	0.71141494	1.78006431	1.00684363	0.7363947
60.4282532	60.4417156	69.0608503	58.6453357	61.2981122	63.0787536	110.577401
0	0	0.04733437	0.06186217	0	0	0
0	0	0.04733437	0.06186217	0	0	0
19.7660054	21.1626166	20.5431179	18.4658573	22.0454119	23.6608254	26.3106239
5.36205466	6.09226842	3.64474673	4.94897348	4.56426747	4.6566518	2.74333856
18.2414997	20.1605988	17.135043	17.9400289	19.9002062	20.1620438	12.2540131
0.07885374	0	0	0	0.04564267	0.02517109	2.64275685
0.02628458	0.08016143	0	0	0.09128535	0	0.98933974
0.36798414	0.52104927	0.33134061	0.24744867	0.45642675	0.67961945	13.8507564
54.4616532	49.339358	59.6886444	49.4278726	48.4268779	58.9506947	170.825996
115.152752	115.352293	137.03301	115.496669	117.073461	120.871578	223.950185
180.364799	166.33496	184.130711	182.462466	173.396521	196.863101	647.981213
7.64881326	7.61533552	7.81017156	7.08321829	6.61818784	8.10509125	9.38954403
0.65711454	1.16234068	0.28400624	0.37117301	1.00413884	0.50342182	0.76446842
70.6792401	69.6201989	67.0254723	72.6261858	67.9619427	59.2527478	32.8748569
1.36679825	1.2024214	0.85201872	1.05165686	1.27799489	0.88098818	1.58331834
11.8017772	7.65541623	8.23618091	9.58863611	8.1243961	8.48265761	8.71532763

153.896226	158.799786	180.201958	167.893925	160.114503	145.010654	45.8497288
67.1571061	58.4777606	67.6408191	68.5432826	53.8583562	79.5909892	117.865565
232.74997	249.783005	254.564258	264.46077	245.511947	195.755573	138.146342
0.81482203	0.32064571	0.61534685	0.52582843	0.82156815	0.70479054	1.37864425
19.5031596	18.9180967	19.0757523	19.3009966	19.7176355	16.1850114	23.0400077
32.4614583	31.4633599	36.5894704	33.8386061	30.5805921	32.1686541	18.5558991
28.9393244	26.693755	46.2456825	35.1995738	31.4478029	40.9281937	34.4535761
0.13142291	0.16032285	0.23667187	0.18558651	0.13692802	0.10068436	0.17952843
0.10513833	0.08016143	0.04733437	0.09279325	0.04564267	0.05034218	2.21748563
0.10513833	0.12024214	0.09466875	0.03093108	0.09128535	0.12585545	2.73647163
14.640512	14.2687339	15.099665	15.2799556	19.3981368	12.0066103	12.129714
33.6705491	33.9082834	33.9860799	37.7668538	34.2776487	33.3013532	25.4959374
1.83992072	1.32266354	1.46736556	0.9897947	1.55185094	1.71163418	4.42624959
51.1497959	56.5138057	59.120632	49.6443902	61.0698988	49.5870489	18.4547389
62.5573043	57.2352585	56.6119102	59.6969926	53.4475721	57.0125207	91.867262
1.07766785	1.88379352	0.99402183	1.60841638	1.27799489	1.43475218	0.92447069
5.25691633	3.40686063	2.9820655	3.03124625	3.92527003	3.95186126	3.84841911
0.07885374	0	0	0	0.04564267	0.02517109	0.13899226
162.1233	140.923788	194.307601	179.276564	150.346971	191.778541	899.424241
175.213021	154.631392	197.242333	178.410494	173.350879	198.599907	983.190408
15.7444644	16.4731731	14.4843182	17.5379248	16.0205788	15.2788521	12.4297977
46.4185712	52.9466222	43.642292	48.4380779	51.211081	48.9829428	34.1142413
0.49940705	0.48096856	0.42600936	0.27837976	0.63899745	0.37756636	0.7985847
9.93557186	8.53719192	10.271559	9.2174631	10.4978152	10.5466871	35.4345153
0.07885374	0	0	0	0.04564267	0.02517109	0.08283437
7.727667	8.41694978	7.57349969	8.0420819	7.25718528	7.24927416	4.84527999
18.9774679	13.3468775	17.0877087	19.8886872	14.4230852	17.745619	26.3805194
16.0335948	13.1865547	15.4310056	13.7024703	13.0994476	15.7319318	30.0592497
0.31541498	0.36072642	0.33134061	0.49489735	0.31949872	0.32722418	0.69271544
11.8543463	12.1043754	13.6322994	13.4550216	12.1409515	13.567218	33.9607665
0	0.04008071	0	0.03093108	0	0.02517109	0
1.26165992	1.2024214	1.08869058	0.92793253	2.05392036	1.08235691	0.386955
124.43121	129.78135	147.399238	132.849007	141.401006	143.022138	76.8179997
59.1140241	56.9146128	67.5461504	58.7381289	53.2650014	64.3373082	150.65274
55.0136294	61.8044598	64.1380755	47.4482832	68.0988707	57.7424824	23.2862847
0.23656123	0.44088785	0.09466875	0.12372434	0.41078407	0.15102655	0.04558272
1127.26685	1325.91008	1493.54147	1358.46229	1278.26875	1111.22815	1097.03178
6.36086876	5.17041201	5.96413101	5.75318167	4.3360541	6.26760162	7.60993358
14.8507886	16.3930117	17.0877087	15.7439219	19.078638	15.782274	19.1632395
52.0171871	58.0769535	53.5825103	57.4080923	50.4807983	49.7129044	27.9454805
0	0.04008071	0	0.03093108	0	0.02517109	0
27.5988107	25.2107686	27.3592676	27.0956298	24.8296151	25.3221174	23.518787
7.09683704	5.61129986	5.20678104	6.49552769	6.07047574	6.51931253	5.91581346
0	0.04008071	0	0.03093108	0	0.02517109	0
2.78616565	3.00605349	1.89337492	2.7528665	3.87962735	1.93817399	1.67936705

0	0.04008071	0	0.03093108	0	0.02517109	0
155.263024	178.599658	173.717149	154.284248	168.147614	165.273382	127.72762
0	0.04008071	0	0.03093108	0	0.02517109	0
8.64762736	8.97807977	8.94619651	8.78442792	8.44389483	8.07992016	3.58593736
66.5788453	62.6862355	64.9900942	63.8726889	69.3312229	67.9871163	36.4406806
5.09920884	4.36879775	6.86348409	5.38200866	5.88790504	6.0410618	3.6224083
0.31541498	0.44088785	0.47334373	0.40210409	0.59335477	0.40273745	0.73987057
8.35849696	7.69549695	8.42551841	8.16580624	9.95010309	7.80303816	4.42729
38.1126434	36.2730455	56.848582	31.7352924	56.1861326	51.7014206	98.8815174
0	0.04008071	0	0.03093108	0	0.02517109	0
0.05256916	0	0.18933749	0.03093108	0.09128535	0.05034218	2.04149471
10.1721331	10.3809047	9.18286837	12.1559161	9.40239099	9.74121215	5.36238769
29.1233165	35.5115119	25.2765552	31.580637	26.244538	28.1916217	26.0234654
12.3274688	13.2667161	12.0702651	12.6508134	13.0994476	13.5923891	16.5260395
0	0.04008071	0	0.03093108	0	0.02517109	0
5.02035509	4.68944345	3.59741235	4.91804239	4.01655538	5.28592908	2.48184541
42.1341844	44.4094303	50.2691042	46.8915237	47.5140244	37.0015035	165.199126
15.5341878	16.1926082	12.685612	14.692265	16.842147	15.1781678	4.93257447
13.2211446	9.9800976	15.2890025	9.8051537	15.0620827	16.1346692	9.2312965
12.6954529	11.0221961	8.85152776	11.5991566	11.4106687	11.4528463	7.79235355
0.02628458	0.12024214	0	0.09279325	0	0	0.05385853
0.44683789	0.52104927	0.66268122	0.43303518	0.5477121	0.45307964	0.94000036
41.6084927	39.9604711	43.5949576	44.4788991	40.9871219	43.9738957	33.4972402
76.0938639	87.9370849	68.7295097	76.6472267	81.5634597	65.8224025	15.1369222
46.4974249	39.3191797	41.0389014	44.8810032	38.2029187	48.8067451	74.9896048
2.1553357	2.60524636	1.98804367	2.81472866	1.82570699	2.59262236	1.91543074
126.113423	119.600848	114.975192	118.806295	142.222574	114.503292	95.3183151
22.5521711	21.9241502	22.7204991	18.744237	21.6802705	23.9880496	8.06659028
26.889127	28.0564993	30.2466644	25.3944202	33.7299366	29.7018872	10.2406828
152.082589	170.302951	174.521833	173.616176	163.81156	179.369193	232.987341
28.1245024	23.767863	18.271068	25.9511797	24.8752577	23.006377	14.553422
3.73241059	3.88782919	3.26607174	3.21683276	3.74269933	4.6818229	3.1390876
8.33221238	7.41493195	6.86348409	7.6399778	7.53104133	8.65885525	30.5169842
12.5114609	12.304779	11.6442558	11.9393985	10.7716712	12.1072947	11.869008
38.5594813	39.840229	43.5002888	40.5197203	39.5265563	37.5552675	30.609504
1.65592864	1.44290568	1.60936868	1.54655421	1.46056559	2.06402945	3.17006749
27.4411032	31.3030371	28.4006238	29.1061503	24.8752577	29.651545	27.4475041
0.7096837	0.40080713	0.47334373	0.30931084	0.3651414	0.70479054	0.48690164
30.3849764	28.2168221	27.2645989	27.4358717	29.9415946	25.5738283	17.8914533
12.8268758	16.7136574	15.6203431	15.6201975	17.9375712	8.73436852	17.5317635
10.9081014	10.8618733	10.5082308	10.300051	11.8670954	11.7045572	9.88423689
1.83992072	1.84371281	1.84604055	1.97958939	1.46056559	1.6361209	0.48185569
87.2648111	78.2375523	83.8765091	84.9058262	74.6714159	81.4033078	55.5554298
4.91521677	4.97000844	6.39014036	5.2892154	5.11197957	4.85802053	1.78207241
15.6918952	12.9861511	14.7683244	16.0841638	12.0953088	13.1644805	30.4693085

3.04901147	3.60726419	3.55007798	4.1138342	2.64727513	2.64296454	3.31833704
66.7365528	62.0449441	72.1849189	68.1411786	61.9371096	78.6093167	123.320004
9.80414895	8.93799906	7.33682782	9.55770503	5.52276364	9.28813252	5.08512658
17.8735155	21.4431816	19.1704211	20.4763778	23.7341909	17.191855	10.6994903
5.38833924	3.96799061	4.97010917	4.79431806	3.6057713	4.95870489	14.0393526
0.18399207	0.24048428	0.14200312	0.21651759	0.22821337	0.22653982	0.19795496
33.5391262	31.2228756	29.3473113	32.5704317	25.9706819	31.7155745	35.6609467
36.6407068	34.6297363	34.4594236	37.2410254	37.4269933	35.0129874	26.3891888
37.5343826	38.3171619	34.4594236	31.9827411	41.3066206	32.9237868	20.7667379
16.6907093	18.4772088	15.6676775	17.8163045	16.9334323	13.8944421	12.3704932
6.96541414	6.6533984	7.33682782	7.36159805	6.57254516	5.94037744	13.2001372
1.76106697	1.8036321	2.4613874	1.82493397	2.09956304	2.51710908	2.85525155
0.26284582	0.40080713	0.42600936	0.24744867	0.50206942	0.47825073	0.20389056
247.180206	230.263698	224.6016	266.904326	231.636574	231.473351	227.261861
11.0132397	14.0282496	10.5555652	11.8775363	12.5517356	9.43915906	3.73505239
1873.51241	1895.21653	1765.19344	1958.33974	2011.74653	1835.52629	3239.35003
37.7183747	38.5977269	39.1455265	38.6947864	37.7008493	43.0425653	78.8050082
33.0922883	31.1026335	38.2461734	31.7971546	28.4353864	37.681123	132.943543
0.5782608	2.1242778	1.37269682	1.39189879	0.86721082	2.26539818	0.74070986
93.678249	102.085577	80.6104373	95.6698435	97.1732545	72.4927416	25.1140089
0.02628458	0.08016143	0.04733437	0.09279325	0.04564267	0.05034218	0.02185829
0.10513833	0.12024214	0.04733437	0.03093108	0.09128535	0.10068436	0.16520766
80.8776577	67.7364054	65.7001098	66.7183487	76.4971228	79.7923579	41.8867264
2.26047402	2.84573064	2.55605615	2.72193541	2.82984583	2.64296454	2.10842896
22.7887323	20.2407602	22.7204991	22.9508645	18.5309259	27.159607	32.1164984
0.86739119	0.96193712	1.70403743	1.45376096	0.7302828	1.61094981	0.55134678
10.3824098	8.17646551	11.6442558	10.9805349	9.08289227	8.48265761	9.62170376
18.7146221	22.365038	19.407093	18.2802708	20.9956304	18.7524627	6.77417419
0.10513833	0.12024214	0.04733437	0.09279325	0.09128535	0.05034218	0.07442949
55.0136294	54.3093665	53.0618322	58.1504383	54.451711	55.4770842	83.5473156
193.717367	214.511977	219.489488	202.443946	197.404568	204.791995	142.15453
11.9332001	9.89993618	8.75685902	13.1147797	7.89618273	7.6771827	11.7344919
0.02628458	0.08016143	0.04733437	0.03093108	0.09128535	0.07551327	0.78423272
35.247624	40.401359	44.7783169	36.9007835	38.5224175	40.2989164	142.774501
8.62134278	10.1805012	10.0348871	9.00094551	9.58496169	11.629044	30.4368302
18.1363613	19.3990652	19.9277711	17.8781667	20.5848463	21.3702561	19.0764779
16.4541481	15.9922046	13.2536245	14.2282987	14.9251546	14.674746	15.5730113
2.8650194	3.36677991	2.41405303	3.15497059	1.73442164	2.99535981	1.54257748
17.7683772	16.2727696	17.513718	22.2085185	15.6097948	16.2605247	13.6018737
171.980018	164.691651	152.653353	169.966308	157.923655	142.443203	80.636498
56.8009809	59.960747	55.3338821	55.088261	59.2898345	51.8524471	59.7967329
24.1818151	21.6435852	20.0224398	20.4454467	23.3690495	19.5579376	17.3243094
81.8764718	86.8549056	77.3443656	69.3784219	85.032303	83.1149419	36.1516991
0.34169956	0.24048428	0.04733437	0.24744867	0.45642675	0.15102655	1.82133352
57.9049334	47.7762102	49.7484261	53.263327	50.4351556	57.8179956	142.237454

0.44683789	0.6012107	0.5206781	0.52582843	0.50206942	0.67961945	0.31816659
0.02628458	0.08016143	0.14200312	0.03093108	0.13692802	0.12585545	0.02015264
0.42055331	0.04008071	0.09466875	0.40210409	0.04564267	0.276882	0.48351191
0.05256916	0	0.09466875	0.03093108	0.09128535	0.07551327	0.22683275
0.05256916	0	0.04733437	0	0.09128535	0	0.04120928
17.4792468	21.803908	23.0518397	19.7649628	20.9956304	18.7776338	6.81284219
22.8150169	21.3630202	18.5077399	18.5895816	22.3192679	24.7935245	7.60056486
0.05256916	0	0.04733437	0	0.09128535	0	0.07245869
138.940299	152.948002	154.830734	132.725282	158.973436	137.862064	45.9237021
28.2822099	32.6257006	28.4006238	28.2091488	28.9374558	28.9215834	29.9542951
1.02509868	1.16234068	1.2306937	0.89700144	1.09542419	1.107528	1.4201451
2.89130398	1.68338996	2.60339052	2.10331373	3.14934456	2.64296454	5.24372066
37.0349755	34.8301398	35.5007798	35.1995738	38.1116334	37.9831761	35.856225
47.0756857	50.0207301	52.020476	48.5618022	45.460104	49.6877333	50.625928
12.3011842	13.5873618	11.1235777	13.6406081	9.72188972	15.5557341	22.2436918
0.78853745	0.6012107	0.99402183	0.68048385	1.41492292	0.65444836	0.77292168
8.54248904	7.89590051	6.91081847	8.10394407	9.0372496	7.62684052	2.3032623
208.147602	234.752738	240.316612	239.777765	220.408476	196.384851	252.97953
117.623503	127.57691	118.004592	127.559791	111.961481	110.450747	116.554961
4.12667932	3.36677991	4.54409981	3.89731661	3.37755793	3.95186126	12.0336259
8.67391194	7.05420553	6.53214348	7.23787371	8.26132413	8.13026234	42.442675
0.68339912	0.32064571	0.66268122	0.27837976	0.63899745	0.57893509	1.26761499
0	0	0.04733437	0.03093108	0	0.02517109	0
0	0	0.04733437	0.03093108	0	0.02517109	0
36.7721297	34.1888484	35.9741235	34.2097792	35.2361449	33.9306304	34.9381008
14.0885358	13.7476846	17.0403743	15.6820597	10.8629566	15.782274	10.9493636
51.4652109	49.9405687	49.8904292	45.2831073	55.5014925	45.156937	18.8662375
24.5760838	21.5634237	36.6841391	25.3634891	24.0993323	28.8964123	61.1047997
0.2891304	0.32064571	0.14200312	0.34024193	0	0.25171091	0.42690279
233.012816	193.229119	223.84425	207.39292	218.171985	251.006118	321.535417
188.618158	205.614059	161.268209	178.224907	210.732229	189.43763	163.409218
0.10513833	0.08016143	0	0.06186217	0	0.07551327	0.32560549
15.7181798	14.7096218	11.5969214	15.6820597	13.327661	10.9997667	5.79151142
0.5782608	0.52104927	0.37867498	0.68048385	0.22821337	0.72996163	0.36443994
24.0241076	29.2990014	23.9038584	26.2604905	28.1158876	25.9262236	20.2329343
0.02628458	0.12024214	0	0.09279325	0.09128535	0.02517109	0.01796789
0.31541498	0.24048428	0.14200312	0.24744867	0.13692802	0.42790854	0.28528096
59.6134312	51.3834744	46.6716918	53.3870514	51.0285103	47.5481906	32.29594
0	0	0.18933749	0.03093108	0.09128535	0	0
7.49110577	8.09630408	8.75685902	7.91835756	6.11611841	8.80988179	9.97144655
13.5365595	16.7938189	15.9043493	13.207573	15.1990107	15.6564185	8.23424862
0.21027665	0.40080713	0.42600936	0.09279325	0.45642675	0.32722418	0.2147148
12.064623	11.3829226	12.4016057	11.6610188	11.0455273	11.5535307	25.3147095
40.1365562	38.998534	36.2107954	36.5296105	42.0369034	32.7224181	19.8417693
0.10513833	0.12024214	0.18933749	0.06186217	0.1825707	0.10068436	0.20845084

0.39426872	0.20040357	0.18933749	0.37117301	0.31949872	0.276882	0.36359075
1.73478239	3.16637635	1.98804367	2.10331373	2.92113118	2.4667669	3.23742754
1.62964406	1.88379352	1.42003119	1.11351903	2.37341909	1.98851618	1.55500748
2.20790486	2.20443923	2.65072489	2.31983132	2.23649106	2.94501763	1.37520239
3.78497976	3.08621492	4.07075608	2.938453	3.19498723	4.02737453	21.2878621
63.082996	60.1611506	58.4579507	62.5117212	59.4724052	53.5389102	19.9479126
9.80414895	13.3869582	11.549587	11.2898457	12.0496661	9.66569888	6.7605812
45.1043421	37.4754669	37.1574829	37.9215093	40.1199111	37.6307808	10.2262029
53.6731157	54.4296086	56.8959164	53.9128798	63.1694618	54.0926742	59.4724023
349.216952	368.021109	338.724774	346.89211	369.203596	297.371267	146.2235
28.9393244	29.7398892	31.5246925	34.4262967	28.5266717	30.6332175	27.0650225
0	0	0.04733437	0.03093108	0	0.02517109	0
0	0	0.04733437	0.03093108	0	0.02517109	0
31.7517746	30.7819878	29.1579738	33.9623305	29.2113118	31.9924565	53.3536791
22.1316177	22.7658451	39.3821984	28.2400799	24.4644737	36.6239372	48.7806885
3977.0412	4039.53469	4525.54474	4200.37938	4275.98834	4735.81489	10835.3139
23.7875464	20.9221323	35.6901173	24.961385	23.2321214	27.8643976	114.337742
4.25810223	4.36879775	4.35476232	3.58800577	4.97505155	3.72532144	6.67804327
0	0	0.04733437	0.03093108	0	0.02517109	0
43.790113	45.6118517	44.3996419	43.5200355	45.8708881	40.0472055	57.4145771
1.78735155	1.72347067	1.37269682	1.70120963	0.91285349	1.78714745	0.83499745
2.8650194	3.24653777	2.84006238	2.50541782	3.65141398	3.47361054	3.47692068
34.4590865	36.4333684	33.9860799	33.3746399	33.0452965	34.8116186	32.2026686
0.31541498	0.76153355	0.42600936	0.34024193	0.3651414	0.553764	0.3858425
34.1962407	37.4353862	29.6313175	31.3950505	34.8710035	31.312837	20.0295704
29.3598777	25.2107686	27.2172645	29.5391854	26.244538	29.097781	30.6707994
3.60098769	2.56516565	2.88739676	2.84565975	3.05805921	2.64296454	4.97744092
0.9988141	1.40282496	0.66268122	0.64955277	1.04978152	1.08235691	0.64256355
9.67272605	7.17444767	13.5376307	9.46491177	10.3152445	12.4848611	5.58400346
1.18280617	1.36274425	1.18335933	1.45376096	1.55185094	1.107528	5.03272826
1.55079032	1.72347067	2.13004679	1.63934746	2.41906176	1.81231854	0.70002176
7.5962441	7.73557766	9.56154336	6.99042504	8.21568145	8.33163107	4.96918371
29.3861623	23.767863	22.6731647	24.8685917	25.6511832	28.6447014	16.936471
0	0	0.04733437	0.03093108	0	0.02517109	0
84.1369459	85.5322421	85.8645527	88.5556941	86.4015833	71.9389776	13.6160189
71.0472242	72.3857681	66.9308035	76.3069848	71.9328554	69.4470396	70.1744232
15.5867569	19.7998724	17.513718	13.949919	17.9832138	18.4504096	8.52349138
8.46363529	8.69751478	6.57947786	6.8667007	7.25718528	8.43231543	10.6243616
9.04189609	7.53517409	9.04086526	6.83576961	7.43975598	9.86706761	11.1669039
3.07529605	2.44492351	2.27204991	2.44355565	2.19084839	2.64296454	2.28760376
0.18399207	0.12024214	0.14200312	0.06186217	0.09128535	0.20136873	0.26541839
708.080345	849.550798	922.168256	779.123081	891.720936	690.896101	284.134577
21.7636336	19.4391459	20.9691273	22.4250361	19.8089208	22.4022708	14.4533836
3.67984143	3.16637635	2.93473113	3.30962601	3.51448595	3.67497926	9.58785055
1.44565199	0.9218564	1.13602495	0.89700144	1.27799489	1.71163418	4.38820048

9.40988023	8.65743406	11.1235777	10.7640173	8.99160692	10.7732269	12.1873668
59.560862	60.1611506	72.1375845	65.1717945	58.376981	75.8153256	193.006425
0	0.04008071	0	0	0	0.05034218	0
0	0.04008071	0	0	0	0.05034218	0
17.9260847	21.1225359	18.3184024	16.517199	20.4479183	18.0728432	16.0826662
439.977612	416.117965	473.533068	438.47905	512.475952	435.585727	366.384026
45.6300337	49.9405687	47.8077168	45.6542803	49.5679448	43.495645	14.0991534
81.3244956	82.6865115	76.2083406	89.5764199	67.4142306	74.4560867	43.6904075
5.75632338	5.69146128	5.68012477	6.68111419	4.79248085	6.54448362	14.4889416
7.51739035	7.33477053	7.3841622	8.35139274	7.21154261	7.72752489	4.38736303
0	0.04008071	0	0	0	0.05034218	0
5.28320091	5.57121914	5.15944666	5.50573299	4.24476875	5.28592908	1.53402845
0	0.04008071	0	0	0	0.05034218	0
2073.38037	2121.9932	2015.54494	1833.44002	2112.16042	1976.43405	2980.41795
9.48873397	7.09428625	8.42551841	9.49584286	7.12025726	7.07307652	8.38081484
0.07885374	0.04008071	0	0.09279325	0	0.07551327	0.03316508
2436.68586	2899.15823	2791.54465	2720.38886	2790.50185	2203.1249	1062.09226
59.2980162	52.1850887	45.1096575	58.1504383	56.9620581	48.2781522	40.3551544
211.906297	236.275805	193.976261	237.179554	191.014594	188.002877	77.8444003
25.3383367	26.2127865	24.4718709	22.8271402	26.7466074	23.837023	14.3466856
19.0826063	20.5614059	18.8864149	17.9709599	19.2155661	19.1048579	4.42698109
20.9750962	27.6957729	21.6318085	28.9824259	19.5350648	25.0955776	20.8605905
40.3468328	49.4596002	43.1216139	43.4581733	44.2733945	40.5002852	21.6757516
0.07885374	0.36072642	0.18933749	0.24744867	0.09128535	0.35239527	0.03085259
1.8662053	1.8036321	1.70403743	1.91772722	0.91285349	1.98851618	2.37554782
0	0.04008071	0	0	0	0.05034218	0
2.05019737	2.56516565	3.07673425	2.07238264	2.28213374	2.66813563	1.0052009
0.07885374	0	0.04733437	0.03093108	0.04564267	0.10068436	1.66311422
4.70494011	6.09226842	4.78077168	4.91804239	4.74683817	4.98387599	9.11398885
26.4422891	26.0123829	24.7085427	22.6106226	26.4271087	25.4731439	13.0140668
1.49822115	2.20443923	2.88739676	1.91772722	1.41492292	3.82600581	7.81557303
0.60454538	0.20040357	0.33134061	0.40210409	0.1825707	0.40273745	2.78542922
1.07766785	0.56112999	0.7100156	0.89700144	1.09542419	0.72996163	1.007101
35.1162011	33.9884448	35.1694392	37.9524403	34.5971474	35.3402115	40.9505545
43.8952514	37.9564355	55.6178883	37.9524403	33.2278672	58.472444	52.8508018
73.386552	68.217374	59.6413101	71.2961491	62.8043204	59.9071962	74.9667544
62.6887272	68.1372125	79.2377405	66.8730041	68.4640121	67.0809571	83.4452632
78.0126383	87.8569235	107.449027	80.2043014	89.3683571	93.1330361	437.760145
173.42567	170.26287	205.904523	167.337166	161.392498	198.020972	1168.86539
123.248403	143.408792	140.772425	137.024703	122.459296	132.853017	95.5134349
0.76225287	1.28258282	0.85201872	0.80420819	0.82156815	0.95650145	0.21091443
0.47312247	0.40080713	0.28400624	0.37117301	0.31949872	0.35239527	0.30050039
135.10275	145.853716	154.357391	146.551477	167.645544	138.390657	50.3485771
1.94505904	1.5230671	1.37269682	0.9897947	1.87134966	2.51710908	54.076411
106.82054	95.3520168	110.904436	105.25848	104.110941	115.107398	200.725028

32.8820116	32.425297	30.8146769	28.271011	32.8627258	38.9145064	10.8435316
21.6322107	22.4051187	18.649743	21.651759	20.3566329	23.2580879	27.6658283
146.431404	170.703758	145.837203	181.843844	156.371804	100.256455	120.219719
102.536153	111.26406	112.845145	108.382519	123.052651	107.178505	30.9667859
70.2061176	72.3456874	74.8356438	64.3366552	77.2730483	83.9455879	95.7971108
43.9215359	44.2491074	37.4888235	44.076795	40.6219805	36.6239372	22.3822404
16.585571	15.3509132	17.0403743	14.0736433	17.0247177	20.1620438	11.071697
7.25454453	8.05622337	7.71550281	7.98021973	7.71361203	8.38197325	15.2219168
26.9154116	29.9002121	27.1225958	28.0544934	28.070245	24.8942088	15.2185009
0.07885374	0.20040357	0	0.09279325	0.04564267	0.20136873	0.06846034
37.9812205	42.5256368	38.1515047	41.4476529	40.4394098	32.4455361	9.65541723
23.0778627	25.3710915	22.1524866	23.0436577	22.6844093	22.3267576	67.2162133
509.920884	463.373126	549.031393	523.817911	502.434563	560.535022	943.689423
4.57351721	4.48903989	5.86946226	4.60873155	3.83398468	5.41178453	5.44497932
1.91877446	1.72347067	2.03537804	1.73214072	1.96263501	1.61094981	3.55368439
118.858878	115.472535	114.927858	119.332123	115.156468	102.773564	44.6212415
15.8496027	18.036321	20.9217929	17.259545	19.6719928	15.4047076	8.7395022
0.65711454	0.48096856	0.42600936	0.40210409	0.77592547	0.30205309	0.3307297
100.696232	99.3600882	90.3613182	91.803458	95.3931902	93.6364579	92.7212783
7.22825995	6.45299483	4.73343731	6.83576961	5.65969167	7.09824762	12.7870195
22.0264794	14.1484918	12.6382776	11.2898457	21.8171985	12.5855454	10.6144053
1.05138327	0.72145284	0.66268122	0.55675952	0.59335477	1.03201472	0.39960903
175.94899	206.736319	202.212442	198.701285	191.379735	173.328131	71.9491252
0.26284582	0.52104927	0.09466875	0.27837976	0.1825707	0.276882	0.1967781
0.97252952	0.68137213	0.75734997	0.95886361	0.5477121	1.13269909	0.88222547
736.099709	587.78366	773.396321	625.581178	712.892937	866.288262	642.956041
21.3430803	19.0383388	15.4310056	18.001891	19.7176355	20.8164921	25.4222744
0.07885374	0.24048428	0.09466875	0.18558651	0.1825707	0.12585545	0.08226919
49.6778593	47.0948381	80.7051061	50.1083564	62.4848217	75.1608772	24.880325
42.2656073	40.962489	42.3642639	45.7780047	39.6634843	44.3011199	85.5300166
1.83992072	1.5230671	1.9407093	1.7630718	1.04978152	1.96334509	2.42668239
0.94624494	1.28258282	0.75734997	0.86607036	0.5477121	1.20821236	0.79555155
2.05019737	1.76355138	1.56203431	1.54655421	0.82156815	2.41642472	0.72348947
38.1126434	30.0204542	24.4245365	29.6010476	34.2776487	24.4914714	28.0690159
7.91165908	8.85783763	9.23020275	9.09373876	7.7592547	10.2698051	9.20419976
0.18399207	0.16032285	0.61534685	0.12372434	0.1825707	0.42790854	0.21725366
78.3017687	81.9249779	89.1306245	82.4004084	78.4141152	80.4719774	97.8400415
121.329629	127.376507	98.1714897	130.621969	109.177278	121.223973	40.3372057
46.8916937	49.6600037	42.6009358	48.2524914	41.671762	44.4773175	26.4218878
0.84110661	1.36274425	0.99402183	0.9897947	1.18670954	1.28372563	0.3407562
263.870915	279.442733	303.602669	267.89412	280.565522	267.392498	171.559609
0.2891304	0.12024214	0.23667187	0.24744867	0.04564267	0.22653982	0.15485506
15.4027648	18.9180967	15.6676775	16.2078881	17.6637151	18.2490409	30.292565
28.5450557	30.1807771	26.7912552	30.3433936	28.7548851	29.4501763	23.601721
0.05256916	0	0.18933749	0.06186217	0.09128535	0.02517109	0.12584556

0.18399207	0.08016143	0.23667187	0.24744867	0.09128535	0.22653982	3.84743234
5.51976215	5.61129986	5.39611853	6.12435468	4.74683817	6.49414144	3.90190335
186.541676	196.355414	152.558684	179.771462	189.051959	143.500389	47.8895457
40.5571095	38.7981304	37.5834922	41.1074109	42.6759009	37.404241	32.9107229
138.046623	149.821706	140.06241	135.632804	152.994246	123.590056	73.6942618
99.8288411	95.8329854	98.834171	95.5770503	97.7209666	91.5472574	45.5413579
10.0144256	7.29468981	8.89886214	8.72256575	7.53104133	8.65885525	17.2178487
37.4555288	41.1228118	43.3109514	40.6125136	42.4476875	34.4843944	12.892177
0.13142291	0.04008071	0.04733437	0.06186217	0.04564267	0.17619764	0.36168213
0	0	0.04733437	0	0	0.05034218	0
0	0	0.04733437	0	0	0.05034218	0
0.18399207	0.12024214	0.18933749	0	0.27385605	0.15102655	0.35556839
21.6322107	18.4371281	23.3831803	21.4352414	21.8628412	22.7043239	16.4618133
64.8966321	62.3655898	65.4161036	65.357381	69.7876497	64.0604262	59.7119928
0.18399207	0	0.09466875	0.12372434	0.09128535	0.02517109	0.11799465
0	0	0.04733437	0	0	0.05034218	0
14.1148203	12.6254247	14.3896494	11.0423971	14.0123011	14.2468374	5.96526235
304.480594	442.330752	360.735257	366.254968	416.671978	267.694551	160.338625
1.39308283	1.16234068	1.04135621	1.05165686	0.45642675	2.51710908	1.19170469
1.2879445	1.48298639	1.60936868	1.39189879	1.23235222	1.35923891	0.77861307
21.9213411	25.6917372	19.4544273	24.9923161	22.1366972	16.8898019	8.77919586
0	0	0.04733437	0	0	0.05034218	0
0.34169956	0.44088785	0.28400624	0.37117301	0.41078407	0.42790854	0.71391297
0	0	0.04733437	0	0	0.05034218	0
0.26284582	0.04008071	0.04733437	0.12372434	0	0.15102655	0.34242323
0	0	0.04733437	0	0	0.05034218	0
0	0	0.04733437	0	0	0.05034218	0
33.9596795	33.3872341	31.2880206	35.972851	33.6386513	32.5713915	39.6775319
41.2930778	41.6037804	35.5007798	41.3548596	42.7215435	38.7131377	22.193855
0	0	0.04733437	0	0	0.05034218	0
22.0001948	17.2747874	18.2237336	20.5073088	18.0744992	21.4457694	16.2172428
73.8071053	76.594243	82.977156	76.7090889	71.978498	77.1745645	79.6534142
0	0	0.04733437	0	0	0.05034218	0
3.60098769	3.76758705	2.60339052	3.68079902	2.19084839	3.34775508	2.06324834
0	0	0.04733437	0	0	0.05034218	0
23.0778627	22.365038	25.1818865	27.8070447	18.8504247	20.7158078	13.2476817
92.548012	110.262042	104.230289	93.628392	104.841224	97.7141746	116.294422
12.2748996	11.8238104	10.0822215	14.4138852	7.7592547	9.96775197	5.81103095
47.7328003	42.1248296	37.4414891	45.9326601	43.2692556	33.1754977	27.3365317
622.708024	372.830795	629.64183	351.562703	450.219344	972.233383	441.210284
56.5907043	59.6401013	46.6243575	56.0471246	59.3811198	53.8157922	31.5239414
14.4828045	17.9160788	15.0049963	15.5892665	15.5641521	14.2720085	10.7995132
41.214224	42.1248296	37.0154797	30.4361869	38.2485614	46.541347	25.9345439
0.76225287	0.72145284	0.5206781	0.49489735	0.59335477	0.72996163	1.01078884
172.295433	163.288826	182.616011	173.306865	168.832254	157.14312	33.9699356

2.89130398	3.04613421	3.21873737	3.09310842	2.64727513	2.79399108	5.30666104
0	0	0.04733437	0	0	0.05034218	0
2.575889	2.1242778	1.65670306	2.81472866	1.64313629	2.44159581	1.00962739
0.07885374	0.04008071	0	0.06186217	0.04564267	0	0.87294683
39.1377421	46.7741924	38.9088547	38.8494418	46.2816722	44.3514621	20.2438155
107.477654	111.344221	101.674233	111.073523	119.766378	101.338812	54.1164157
9.96185644	8.33678836	8.42551841	7.79463323	8.76339355	9.01125052	6.3685353
3.78497976	3.76758705	4.35476232	3.43335035	4.24476875	4.9335338	2.52942366
27.8616565	27.4152079	27.3592676	29.3535989	26.7466074	23.6608254	8.07074454
13.2211446	12.6655054	16.4250275	14.2282987	11.2737407	14.5992327	26.191468
22.7887323	16.1525274	22.2471553	12.0321918	23.0951934	22.4274419	20.2081338
0.39426872	0.64129141	0.56801248	0.40210409	0.68464012	0.67961945	0.64136054
0.10513833	0	0.14200312	0.03093108	0.09128535	0.07551327	0.12456578
109.238721	109.059621	107.401692	113.145906	109.314206	92.000337	16.826811
8.35849696	7.89590051	7.00548721	7.76370214	9.0372496	7.77786707	4.72060307
0.81482203	0.16032285	0.75734997	0.37117301	1.00413884	0.67961945	2.77648967
0.26284582	0.24048428	0.14200312	0.06186217	0.41078407	0.12585545	0.33510726
2.07648195	1.24250211	1.08869058	0.86607036	1.64313629	1.58577872	1.60513731
49.1258831	43.6478967	35.3114423	41.6951015	38.9788442	42.8160255	29.5306975
16.6118556	17.3950296	18.2237336	20.4145156	12.2778795	16.7891176	6.42054924
148.981009	136.595071	131.73156	147.78872	136.699811	117.473481	108.641594
42.1078998	56.6741285	57.3219258	44.7263478	51.3023664	53.5389102	252.499871
4.07411016	4.80968559	4.44943107	5.41293974	2.60163246	4.25391435	7.81772386
47.7590849	55.7121914	56.2805696	47.7885251	58.376981	47.7243882	22.3457584
54.6982144	51.6239587	54.6238665	52.1188769	47.9704511	55.426742	51.5497074
8.35849696	10.3809047	7.14749033	9.46491177	7.16589993	7.87855143	6.04125628
12.1960459	12.0642947	8.33084966	11.4135701	8.4895375	14.6244038	5.25048156
5.17806258	3.08621492	3.171403	3.49521252	4.5186248	4.43011199	4.81508758
11.9594846	13.1464739	14.1529775	11.382639	15.1990107	10.9494245	10.6592798
13.694267	14.5893796	14.9576619	14.4448163	13.2363757	13.7182445	7.96955053
16.900986	16.3128503	15.9990181	17.3832693	17.2072884	16.61292	16.1332246
13.9308283	14.4290568	11.4549183	14.1045744	14.0123011	13.567218	10.9169616
2.83873482	4.28863632	3.26607174	2.28890023	3.9709127	4.95870489	4.79969938
7.70138242	7.77565837	7.71550281	6.80483853	6.70947319	8.48265761	4.58777098
14.5353737	15.5513167	14.9103275	18.4039951	13.8297304	14.699917	19.0170145
22.1841869	21.4031009	20.306446	23.1055199	22.1366972	21.1688874	17.4630561
22.6573094	19.7998724	17.2770462	19.9814804	20.767417	21.6471381	8.68306796
32.2248971	32.6657813	29.8679894	30.1887382	26.3358233	34.4592234	58.9682006
99.8025565	108.298087	95.1420899	99.1959871	109.496777	83.5931927	37.1254365
508.685509	575.759446	671.864091	611.662191	568.935941	499.11756	212.016181
49.6778593	47.7361295	49.0857449	53.5107757	35.646929	64.6141902	14.4656496
5.30948549	6.09226842	4.49676544	5.90783709	4.92940887	5.94037744	5.6429326
0.10513833	0.08016143	0.14200312	0	0.13692802	0.12585545	0.07039637
103.219552	93.6686269	91.5446775	100.278575	88.409861	90.1125052	156.464632
39.1114575	44.3292689	44.3049732	49.8609078	39.709127	42.6146568	14.7508771

0.60454538	0.40080713	0.5206781	0.30931084	0.27385605	1.13269909	0.42066636
52.14861	42.8062018	41.8909202	45.8089357	44.5016079	42.0860639	38.1079013
13.6154133	13.5873618	11.5022527	13.949919	13.6015171	12.9631118	9.39133611
0.42055331	0.32064571	0.23667187	0.34024193	0.27385605	0.50342182	0.45127778
16.9272706	19.9601952	15.8096806	18.713306	16.1118642	20.0865305	14.0433237
20.8173887	23.8480244	21.8684804	20.1052047	22.9126227	21.0430319	30.0301946
26.4948583	26.693755	26.5072489	23.5694862	28.070245	25.3976307	95.3257942
26.074305	25.4111722	17.9870618	24.8067295	22.7756947	18.9538314	13.7662121
0.36798414	0.6012107	0.47334373	0.37117301	0.31949872	0.52859291	0.42527122
1.39308283	1.12225997	1.04135621	1.20631228	1.23235222	0.90615927	4.90399341
16.1124485	15.9521239	14.6736556	16.0532327	15.7010801	13.1896516	8.19718072
17.4529622	17.3549488	16.23569	19.6103074	18.1201419	15.5809052	9.44061538
30.8843834	30.0605349	31.5720268	30.7764288	28.3897437	30.1046246	26.5965586
52.8845783	44.6098339	50.884451	55.3975719	44.7298212	42.664999	32.9561517
0.15770749	0.04008071	0.04733437	0.12372434	0.04564267	0.15102655	0.11011487
0.36798414	0.56112999	0.37867498	0.68048385	0.3651414	0.40273745	0.12607466
22.7624477	20.401083	20.3537804	23.9097281	20.767417	21.3702561	30.1189476
0.42055331	0.76153355	0.23667187	0.37117301	0.59335477	0.32722418	0.440271
2.33932777	1.84371281	2.17738116	1.42282987	2.55598978	1.98851618	2.67195631
0.18399207	0.08016143	0.09466875	0.12372434	0	0.15102655	0.36361033
199.999382	215.874722	207.987235	217.198073	204.707396	179.721589	82.8080212
7.33339828	6.57323697	5.82212789	7.73277106	6.07047574	6.99756325	5.39924052
1.15652159	1.56314782	0.5206781	1.33003662	0.77592547	1.48509436	1.01053988
3.20671896	2.60524636	2.22471553	3.03124625	2.23649106	2.26539818	4.12484972
13.0371525	13.5873618	9.51420899	11.382639	9.72188972	13.1896516	7.98605742
9.22588816	6.73355983	6.1534685	7.91835756	6.43561714	6.69551016	9.91294961
55.223906	57.2753392	63.1440537	58.6453357	56.8707727	54.2437007	38.2745558
5.02035509	5.09025058	4.73343731	4.5159383	4.10784073	5.33627126	8.42431613
0.65711454	1.12225997	1.2306937	0.71141494	1.09542419	0.88098818	29.7717979
0.15770749	0.12024214	0.04733437	0.09279325	0.09128535	0.10068436	6.53970339
2.70731191	3.9279099	3.45540923	2.78379758	2.60163246	3.87634799	1.85691886
86.2397124	91.1034612	85.5805465	82.1529597	92.2894883	80.3964641	114.049481
3.18043438	3.68742562	3.07673425	3.58800577	2.37341909	3.24707072	1.9462624
302.535535	299.803735	280.45616	354.31557	268.607141	295.483435	216.55194
2.60217358	2.04411638	2.36671865	1.79400289	4.38169677	1.71163418	1.13746672
4.81007844	5.29065415	5.15944666	4.23755854	4.56426747	5.53763998	3.96369022
18.7146221	18.9982581	16.0936868	17.1358207	16.7508616	17.8714745	23.5406907
0.78853745	0.72145284	0.56801248	0.83513927	0.59335477	0.85581709	0.82444979
15.0610653	15.3909939	15.2416681	14.9397137	16.5682909	16.0339849	9.62841881
187.041083	207.578014	187.444117	197.71149	182.296843	181.911473	179.173534
26.705135	27.8961764	27.7379426	25.7655932	30.8544481	28.6950436	48.1473815
191.509462	183.16886	213.809363	196.226798	185.217974	186.467441	113.393371
58.2203483	71.3837503	73.3209439	62.4498591	69.0117242	64.2366238	61.822999
2.44446609	3.80766776	2.9820655	3.5261436	2.87548851	2.13954272	3.41551042
1.18280617	0.72145284	0.94668746	0.92793253	0.68464012	0.98167254	0.60820066

5.78260796	5.89186485	5.20678104	5.01083564	5.75097702	5.33627126	4.57257865
19.6871517	16.3128503	16.8983712	18.2493397	14.1948718	18.1231854	38.2269884
166.460256	170.623596	214.188038	174.142004	184.578977	170.483798	161.353708
85.7928745	76.6343238	103.520274	83.9160315	86.4015833	86.2864994	170.787893
19.2928829	20.5614059	23.4305147	21.3733792	17.4811444	21.621967	30.0263354
15.8496027	14.6695411	15.7150119	14.692265	13.8753731	15.7571029	4.94165557
9.40988023	6.61331769	6.43747474	7.73277106	6.84640121	9.23779034	4.26016577
13.694267	11.5031647	16.2830243	13.4240906	11.1824553	19.1048579	16.9726395
0.44683789	0.24048428	0.42600936	0.71141494	0.09128535	0.45307964	1.57071899
1.68221323	1.72347067	1.08869058	1.85586505	1.82570699	1.30889672	2.22227502
26.2320125	25.8921408	26.3652458	26.5388703	25.5598978	23.8118519	14.3736773
124.746624	121.685045	138.311038	117.321602	122.18544	131.871345	243.188381
19.8448591	23.3670558	21.9631491	18.6514438	21.3607718	22.5029552	39.8946984
1.60335948	1.88379352	1.60936868	1.45376096	1.73442164	1.56060763	0.48620874
78.53833	77.8768259	78.0543812	80.2043014	74.1237038	73.0716767	62.7119991
62.2681739	56.8344514	57.7479351	58.0576451	51.7131505	61.0650664	92.0504414
5.83517713	5.29065415	5.11211229	6.06249251	4.83812352	6.19208835	11.3302956
74.8847731	91.3840262	84.7758622	72.6571168	78.8248993	90.6662692	195.947466
232.80254	224.692479	236.245856	242.159458	222.964466	253.523227	660.751551
7.49110577	9.69953261	9.46687461	9.31025635	7.48539866	11.1004511	29.8008435
3.89011808	3.24653777	3.40807486	3.80452336	3.4232006	3.97703235	4.13083695
170.90235	186.295155	195.538295	176.245318	184.852833	211.739216	105.427296
41.5559236	43.6478967	37.299486	41.0146177	42.9041142	42.7908544	17.14205
21.0539499	21.4031009	18.5077399	20.6928953	21.9541265	20.8164921	14.0239515
0.15770749	0.16032285	0.04733437	0.15465542	0	0.276882	0.14456444
27.2833958	30.2609385	24.613874	27.7761136	24.9665431	26.4799876	27.6286227
33.0397191	37.5155476	33.5127361	35.4470225	33.8668646	31.2121526	8.84204811
149.21757	161.364952	140.535754	154.810077	151.442395	128.29705	54.1648831
11.118378	10.3809047	11.5969214	10.7640173	8.80903622	11.8304127	55.7415907
98.2780508	110.181881	97.9348179	88.122659	98.9989615	108.411888	65.6234129
96.4906992	128.859493	110.62043	97.5566397	117.392959	108.135006	113.060969
16.9535552	20.0804373	17.9870618	17.8781667	12.9625196	21.2695718	20.4023949
31.1209447	33.547557	30.1046613	32.5085695	33.8668646	31.7659166	18.4625574
79.1165908	77.5561802	84.8705309	83.7923072	81.380889	83.693877	151.696176
65.842877	66.6141454	88.8939526	68.3267651	69.1030095	75.2112194	305.672189
0.81482203	0.76153355	0.75734997	0.80420819	0.63899745	0.67961945	0.7667383
28.334779	21.8840694	24.0458615	26.7553879	21.6346278	23.006377	46.619392
7.17569079	7.13436696	7.85750593	7.48532238	6.89204388	6.72068125	16.7998409
1.10395243	0.9218564	0.75734997	1.42282987	0.45642675	1.18304127	2.40721702
46.2871483	51.6640394	59.7359788	48.9329752	65.2690249	50.2666684	15.4368558
76.1201485	68.1772933	70.8122221	82.4004084	66.3644491	73.9274938	63.778532
18.3992072	16.3128503	18.0343961	17.4760626	17.1616457	16.1598403	10.8391424
13.694267	12.7857475	12.2122683	12.7126756	13.4189464	11.1256221	9.93005051
71.4152083	86.9751478	79.2377405	72.6261858	86.721082	86.6892368	169.140702
32.5928812	33.266992	27.2645989	30.3743247	31.995515	27.5371734	14.2165775

93.1788419	85.4520807	94.9527524	95.113084	85.9907992	101.842234	107.786577
157.07666	142.046048	120.79732	160.934431	110.774772	129.706631	75.1464862
1.52450574	1.72347067	1.08869058	1.85586505	1.73442164	1.20821236	1.26562974
4.25810223	4.44895917	4.11809046	3.89731661	4.06219805	4.17840108	3.48536216
3.23300354	3.64734491	3.92875296	3.77359228	2.73856048	3.49878163	3.39184016
126.244846	139.681286	99.4495178	134.704872	140.625081	106.851281	41.0454381
9.77786437	1.00201783	1.32536245	0.64955277	8.17003878	0.90615927	61.4967481
94.493071	94.7107254	99.4495178	93.1334946	88.6380743	97.5631481	182.609767
0.07885374	0	0.04733437	0	0.09128535	0.02517109	0.06445748
16.7432785	16.4731731	14.0583088	19.084479	13.9666585	11.9562681	16.9131346
16.3227252	21.4031009	15.7150119	17.6925802	21.7259132	11.07528	20.952098
0.07885374	0	0.04733437	0	0.09128535	0.02517109	0.09783025
244.946016	221.085214	268.385895	242.4997	217.441702	247.532507	277.57189
52.5691633	41.7641032	49.3224167	48.4380779	45.8708881	54.7471226	148.173003
52.9108629	52.9065415	59.9726507	60.6249251	54.6342817	56.0056771	36.7547299
26.1268742	23.8480244	25.0398834	25.8893175	23.9167616	22.7043239	19.0866649
40.2154099	39.8001483	37.5361578	33.034398	36.2859264	43.9235535	15.9258397
0.15770749	0.12024214	0	0.09279325	0.13692802	0.15102655	0.15937319
42.6861606	37.315144	43.8789638	46.0563844	37.2900653	44.8297128	31.9079448
43.9741051	46.4535467	47.8077168	46.1801088	43.634397	44.049409	29.4513415
52.3063175	56.1530793	49.1330792	52.3663256	52.1695772	47.9760991	24.1090987
685.344182	743.737715	719.671808	728.581689	688.793604	646.871863	584.027479
0.07885374	0.08016143	0.18933749	0.12372434	0	0.25171091	1.7072677
90.2086842	92.3860441	91.4973431	84.6274464	99.2728175	81.755703	40.6224756
0.13142291	0.28056499	0.04733437	0.18558651	0.09128535	0.25171091	0.0546922
1.39308283	1.36274425	0.99402183	0.89700144	1.00413884	2.21505599	0.89058673
2.78616565	2.92589207	2.36671865	2.84565975	2.41906176	3.32258399	5.36313992
47.6802311	47.3754031	46.1510137	49.953701	46.1447442	49.4360224	33.2439582
1964.64106	1955.89873	2012.94155	2144.66859	1813.79425	1706.6503	1699.65804
13.7468362	13.2667161	15.2416681	15.2180934	13.5102317	15.1529967	22.7093887
30.5163993	28.6176293	29.3473113	30.2506004	31.0826615	30.1549668	70.9026817
0.07885374	0.04008071	0	0	0.09128535	0.02517109	0.06804982
12.3537534	11.7035683	12.3542714	12.2177783	13.327661	12.4345189	12.5360138
0.63082996	0.20040357	0.47334373	0.77327711	0.27385605	0.45307964	0.67103044
15.3764803	16.352931	12.4489401	14.3520231	14.1492292	13.9196132	12.4116278
0.65711454	0.80161427	0.33134061	0.5876906	0.41078407	0.60410618	1.04124163
109.501567	120.041736	120.087304	120.97147	112.691764	127.818799	166.91795
23.7875464	25.2508494	22.0578178	20.0124115	31.995515	22.6539818	15.4740522
2604.14493	2682.8827	2666.53457	2784.35434	2605.19259	2196.40422	2301.74288
0.60454538	1.56314782	1.84604055	1.23724337	1.32363757	0.98167254	0.13788225
5.83517713	6.69347911	6.05879975	5.44387082	4.83812352	9.41398797	3.82124532
0.31541498	0.28056499	0.23667187	0.15465542	0.31949872	0.276882	0.14394423
74.3590815	74.5501267	69.155519	80.1424392	73.1652076	71.8634643	49.1024936
1.36679825	1.44290568	1.32536245	1.45376096	1.14106687	1.81231854	1.35602933
0.26284582	0.04008071	0.23667187	0.18558651	0	0.45307964	0.33739288

0.15770749	0.20040357	0.37867498	0.24744867	0.1825707	0.35239527	0.34886302
32.671735	29.3390821	29.9153238	32.0755343	27.4768902	35.6926068	39.7680123
200.551358	226.656433	214.614047	214.445207	214.338001	191.073751	161.243196
913.862335	866.625182	977.312801	920.014169	918.148045	1037.22514	2297.8712
184.570332	162.046324	162.451568	186.081403	169.151753	172.875052	181.935673
0.18399207	0.20040357	0.23667187	0.21651759	0.22821337	0.10068436	0.23792261
2.07648195	1.08217926	1.9407093	1.42282987	1.73442164	1.58577872	9.922382
1.5770749	1.12225997	1.13602495	0.95886361	1.73442164	1.58577872	1.81061708
103.140698	110.181881	110.857102	110.764213	135.330531	91.6227706	78.4886455
355.866951	341.247193	419.666552	346.33535	381.527118	435.208161	336.419475
28.2822099	27.4152079	24.992549	29.1061503	28.1158876	26.4044743	20.6921118
26.4948583	25.0103651	24.8032115	26.8481811	25.8793966	26.2031056	32.3622267
0.76225287	1.16234068	0.56801248	0.83513927	0.82156815	0.62927727	0.38965847
116.729827	125.572875	126.477445	128.766104	113.969759	113.571962	61.0621675
7.91165908	9.29872548	8.70952464	9.61956719	7.53104133	9.84189652	7.39924774
0.07885374	0	0	0.06186217	0	0	0.12551805
571.40052	584.697445	579.84607	633.159294	541.641621	491.566233	596.149972
0.15770749	0.04008071	0.04733437	0.18558651	0	0	0.12450549
857.245346	922.497696	836.067031	875.318753	964.47536	656.436878	336.215328
0.18399207	0.04008071	0.14200312	0.06186217	0.04564267	0.17619764	0.32525259
45.1306267	45.852336	48.0443887	44.3861059	46.1447442	44.2759488	57.9303579
76.0675793	73.7885931	71.4749033	80.049646	71.567714	76.469774	42.5479422
4.36324055	3.72750633	4.11809046	3.40241926	4.06219805	4.10288781	5.80477207
101.721331	110.342204	120.655317	95.7007746	112.417908	112.942685	175.382955
1.15652159	1.56314782	1.79870618	1.14445012	1.68877897	1.30889672	1.62990303
3.78497976	3.64734491	3.02939988	3.86638553	3.788342	3.52395272	4.96792919
83.8215309	78.9990858	79.4744124	83.2355477	80.1941795	71.5865823	78.4526736
46.7339862	52.8664608	49.7957605	47.1389724	50.1612995	47.2713086	63.2400411
33.6179799	29.9803735	31.4773581	33.5292953	31.4478029	33.3265243	36.0898463
10.934386	10.8618733	13.1116213	10.3928443	10.2696018	12.5352032	26.645166
90.1035459	93.788869	84.3025184	98.5155033	80.5136782	97.9658855	47.0432526
2860.99786	3166.25611	2805.22429	2995.73737	3241.36019	2136.34599	540.888116
104.165797	80.1213458	90.1246463	89.5145578	84.3476629	91.5472574	124.987235
59.6134312	73.4278667	61.0140069	74.0180846	52.7172893	59.4541166	80.9819351
12.9320142	11.5432454	12.9222838	13.4859527	12.0496661	13.3658492	31.1715123
10.618971	11.9440526	11.3129152	9.86701587	10.3152445	12.1072947	17.6494984
54.2250919	54.3895279	61.2980131	62.7282388	48.0160938	65.4700073	138.193243
23.3144239	27.7358536	27.9272801	22.5487604	27.3856048	26.0772501	30.5518923
38.0863588	40.962489	45.8196731	39.7773743	39.6178417	41.2302468	29.1463759
3.81126434	3.20645706	3.92875296	4.48500721	2.73856048	4.35459871	7.44176144
327.453318	297.158408	297.307197	268.574604	330.224752	289.492716	502.280655
50.0195589	45.5316903	46.2456825	49.0257685	45.2775333	52.0538158	97.9011037
276.618937	262.809237	274.06602	276.245513	253.727629	255.687941	234.825727
89.2624393	87.0152285	82.6458154	84.3799978	86.85801	80.1699243	86.4556038
1.44565199	1.5230671	1.04135621	1.94865831	1.00413884	0.67961945	1.02816558

189.932387	180.723936	152.984694	170.646792	150.940325	181.936645	193.61791
1.65592864	1.32266354	1.51469994	0.9897947	1.78006431	1.43475218	1.72691656
16.5330019	15.3909939	16.4723618	18.6205127	10.6803859	16.5122356	23.1933231
33.8545412	32.6657813	27.8326114	30.0340828	33.5930086	34.3081968	50.0166204
25.0229217	26.0524636	20.5904523	25.9511797	21.9541265	26.5303297	23.067198
17.3215393	16.4330924	17.7030555	18.8988925	15.1077253	15.3543654	14.2352596
139.360852	129.981753	138.784382	136.684461	146.102202	110.52626	70.2567805
9.67272605	11.8638911	13.0169526	9.09373876	12.3235222	11.4276752	5.97752126
38.6909042	24.7698808	33.749408	38.6638553	27.9789596	35.1640139	67.5178507
417.845994	474.916371	432.872842	502.908498	421.966528	453.029293	654.056113
71.4677775	95.9532275	102.478918	78.4721607	91.6961335	89.3573725	329.907471
2.68102733	2.56516565	3.07673425	2.28890023	2.96677386	3.59946599	2.54335886
8.93675776	9.57929047	5.68012477	8.78442792	8.53518017	8.35680216	15.673411
0.21027665	0.24048428	0.14200312	0.21651759	0.09128535	0.35239527	0.36486288
24.2869534	21.5634237	24.2825334	21.8682765	20.6761317	24.944551	40.0132963
45.0517729	44.7701567	41.6542483	47.1699034	43.7256824	44.7290284	32.9612263
86.6339811	82.3658657	79.1904061	93.9686339	86.6297966	75.91601	41.7236509
0.21027665	0.04008071	0.09466875	0.03093108	0.27385605	0.17619764	0.64793031
38.6909042	24.7698808	33.749408	38.6638553	27.9789596	35.1640139	70.0148127
0.18399207	0.12024214	0.14200312	0.06186217	0.13692802	0.32722418	0.30150675
0.5782608	0.56112999	0.37867498	0.37117301	0.41078407	0.57893509	0.25272523
15.6656107	13.8278461	14.2003119	15.7129908	15.4728667	14.397864	23.614924
0.18399207	0.12024214	0.23667187	0.18558651	0.13692802	0.276882	6.34013498
0.76225287	0.40080713	0.14200312	0.24744867	0.7302828	0.62927727	0.49244928
40.5045403	43.2070089	30.2939988	41.6023083	38.7506308	38.2852292	18.440122
0.36798414	0.56112999	0.33134061	0.43303518	0.68464012	0.35239527	0.59780821
44.1843817	44.2891882	47.4290418	46.9843169	36.1946411	47.674046	44.1405501
1.05138327	1.00201783	0.47334373	0.95886361	0.77592547	1.08235691	0.47856434
29.281024	11.3829226	8.23618091	10.4856376	26.518394	17.4939081	27.5743229
0.36798414	0.28056499	0.04733437	0.30931084	0.31949872	0.25171091	0.35250555
0.02628458	0.24048428	0.09466875	0.12372434	0.13692802	0.15102655	0.04570511
0.60454538	0.44088785	0.37867498	0.46396626	0.22821337	0.553764	1.09398145
22.4996019	28.5775486	22.4838272	27.2502852	20.1740622	22.9812059	9.00046818
0.13142291	0.04008071	0.28400624	0.06186217	0.1825707	0.12585545	0.15540619
10.9606705	9.29872548	6.91081847	9.03187659	8.58082285	8.38197325	9.00774396
1.55079032	1.8036321	2.03537804	1.63934746	1.36928024	1.88783181	1.07482094
1.05138327	2.48500422	4.35476232	3.80452336	2.82984583	2.06402945	1.22373137
268.26044	244.412189	265.73517	283.328732	290.469982	234.116316	172.261967
4.36324055	4.16839418	4.2127592	5.2892154	3.33191526	4.80767835	1.60284923
2.91758856	1.88379352	2.03537804	3.03124625	1.82570699	2.54228017	2.09635426
97.2266675	97.6766982	97.7928147	104.887307	99.774887	97.4624637	53.975925
8.20078947	8.93799906	7.57349969	6.80483853	8.3069668	10.8990823	10.1795079
11.8017772	11.7837297	12.7802807	12.6508134	11.6388821	13.4413625	4.21147614
16.6907093	15.5913975	18.4130711	17.5069937	12.2322368	18.4252385	13.0047764
22.9201552	23.1265716	23.3831803	21.9610698	26.244538	23.8118519	25.7819663

9.69901063	9.29872548	8.66219027	10.300051	8.58082285	9.99292306	22.4496819
14.9296424	12.2246175	15.6676775	12.4342959	14.3317999	14.2971796	45.0387464
0.07885374	0	0.04733437	0.09279325	0	0	0.17946163
5.38833924	4.80968559	8.33084966	6.2790101	5.93354771	7.24927416	22.0450704
8.04308198	9.29872548	7.10015596	6.2790101	10.5434579	9.11193488	4.62141088
0.21027665	0.12024214	0.18933749	0.09279325	0.22821337	0.15102655	0.23448344
2.47075067	2.04411638	1.98804367	2.31983132	2.19084839	2.4667669	3.67022819
0.07885374	0	0.09466875	0.03093108	0.13692802	0.07551327	0.18182964
0.5782608	0.48096856	0.7100156	0.34024193	0.68464012	0.57893509	1.30753774
34.9059244	29.7398892	33.7020736	33.3437088	33.3647952	34.9374741	50.2647479
7.83280533	3.56718348	6.81614972	3.30962601	7.39411331	9.23779034	12.3000711
5.7300388	4.56920131	3.78674985	5.50573299	3.74269933	5.73900871	3.4402317
66.4999916	75.0310952	67.0254723	64.0892065	79.144398	72.593426	18.0955312
13.1685754	12.4250211	9.51420899	11.8466053	11.7758101	13.1644805	15.6245629
3.89011808	3.44694134	4.11809046	4.36128288	3.69705665	4.00220344	1.75566286
51.5966338	48.2170981	50.5531104	49.9227699	56.0492046	49.5367068	32.1781302
0.10513833	0	0	0.03093108	0	0.05034218	0.05572538
74.9899114	72.9468981	81.1311154	81.0703718	72.1154261	68.9436178	53.0101441
9.01561151	9.73961332	8.89886214	9.34118744	10.2696018	9.31330361	5.74858733
0.21027665	0.20040357	0.23667187	0.27837976	0.27385605	0.20136873	0.12544664
30.5426839	30.7419071	26.5545833	30.0340828	26.1988953	34.7361054	16.5357006
16.6644248	18.4371281	18.4604055	19.0226168	13.6471597	18.4000674	8.33926996
2.65474275	3.3266992	2.31938428	2.50541782	3.10370188	2.24022708	1.84255757
0.36798414	0.20040357	0.23667187	0.27837976	0.1825707	0.45307964	0.63090341
10.303556	8.49711121	8.66219027	8.28953057	8.71775087	9.31330361	18.018854
0.73596829	0.56112999	0.61534685	0.34024193	0.31949872	1.48509436	0.64816355
1.13023701	2.68540779	1.37269682	2.01052047	1.46056559	1.20821236	2.98296072
0.10513833	0	0.23667187	0.09279325	0.1825707	0	0.12861417
154.55334	149.901868	155.162075	171.481931	105.297651	207.057393	73.5223897
8.80533485	9.09832191	10.0348871	7.39252913	9.2198203	12.7617431	2.75907696
8.64762736	7.93598123	4.9227748	6.00063034	8.17003878	6.3179438	3.16722012
12.064623	10.1003397	13.3956276	13.5478149	10.7260286	12.7869141	11.7738138
5.20434717	3.12629563	3.78674985	4.54686938	4.01655538	4.43011199	4.15290362
9.14703441	8.53719192	7.47883094	7.08321829	9.72188972	9.74121215	13.0794068
95.123901	113.869306	71.9009127	54.4077772	106.803859	103.629381	281.07166
18.6094838	17.0343031	16.0463525	17.8472356	17.116003	18.7524627	21.6806738
67.603944	58.5178414	59.2626351	77.9463323	57.3728421	57.3397449	40.5486194
17.8998001	19.9601952	17.9870618	17.9400289	19.6719928	16.2605247	14.6759798
12.9582988	11.743649	12.2596026	13.6715392	13.6471597	11.3269909	21.2631738
1.41936741	1.28258282	2.36671865	1.79400289	1.59749362	1.23338345	4.55122215
1.8662053	2.48500422	0.85201872	2.07238264	1.46056559	1.23338345	1.13974112
0.07885374	0.04008071	0.04733437	0.06186217	0.09128535	0.07551327	0.09360565
0.36798414	0.20040357	0.23667187	0.34024193	0.27385605	0.12585545	0.22631955
103.482398	102.606626	91.1660025	101.886991	99.9574577	85.7830776	34.9354198
0.05256916	0.04008071	0.09466875	0.03093108	0.1825707	0.05034218	0.0500639

76.4355634	75.2715795	96.1834461	78.5340229	75.6755547	103.226644	28.2186507
105.322319	115.512616	84.8705309	110.021867	103.7458	103.276986	62.3465964
16.900986	16.1525274	15.1943338	16.9811652	16.7965043	16.361209	20.5923579
1.41936741	1.56314782	1.37269682	1.48469204	1.09542419	1.40958109	0.70999438
0.84110661	1.28258282	1.42003119	0.89700144	1.32363757	1.00684363	2.02342839
0.2891304	0.16032285	0.04733437	0.34024193	0.04564267	0.22653982	0.54371861
0.23656123	0.16032285	0.23667187	0.30931084	0.22821337	0.20136873	0.15103438
32.6454504	32.705862	34.8854329	29.2917368	31.7216589	35.7177779	31.6816333
0.07885374	0.16032285	0.18933749	0.03093108	0.1825707	0.276882	0.09868095
0.65711454	0.40080713	0.7100156	0.55675952	0.3651414	0.62927727	0.58164873
0.31541498	0.44088785	0.42600936	0.55675952	0.31949872	0.40273745	0.52638813
39.0063192	37.8361933	34.9801017	38.2308201	37.7008493	39.4682704	34.7783206
25.259483	19.2788231	21.3478023	26.1986283	20.1284196	22.3267576	30.4279208
433.616743	392.911232	389.9879	453.047591	425.617942	282.44481	298.669469
0.02628458	0.20040357	0.04733437	0.06186217	0.1825707	0.10068436	0.01351557
0.10513833	0.08016143	0.14200312	0.12372434	0.13692802	0.02517109	0.1234301
123.74781	132.266354	124.678739	132.137592	128.803628	107.002307	36.4856289
20.3968354	17.475191	17.9870618	20.6310332	14.1035865	18.6517783	67.0729794
2.89130398	1.92387424	2.22471553	2.13424481	2.51034711	2.03885836	2.4951602
5.20434717	4.40887846	5.11211229	4.23755854	5.75097702	5.63832435	2.00864529
2.36561235	1.88379352	2.03537804	1.5774853	2.55598978	2.69330672	2.25550955
21.7110644	18.036321	20.9217929	19.4247209	22.6844093	21.0178608	22.8070635
21.6584953	19.1986617	20.0224398	18.5586505	20.9956304	19.3313978	11.6767379
5.20434717	4.64936274	3.88141859	3.83545444	3.92527003	5.2104158	8.70321429
10.7766785	9.77969404	9.23020275	10.5165686	9.2198203	8.83505288	4.85381152
0.10513833	0	0	0.03093108	0	0.05034218	0.22348248
56.0912972	48.778228	57.227257	58.1813694	50.800297	58.5479573	115.709373
1.91877446	2.32468137	0.94668746	1.88679614	1.36928024	1.53543654	0.80771113
6.54486083	8.53719192	6.20080287	6.21714793	7.85054005	6.24243053	4.63189579
81.6399106	80.1213458	83.6871716	83.730445	78.5510432	90.4649005	50.3625318
24.8914988	24.6496387	24.0931959	25.9202486	23.0039081	22.2512443	12.6898954
49.2310214	48.3774209	44.2576388	40.767169	53.1280734	53.3878837	34.5120823
1.5770749	2.24451994	1.65670306	2.3507624	1.00413884	1.58577872	0.51349814
0.94624494	0.56112999	0.99402183	1.23724337	0.7302828	0.78030382	28.9381875
4.28438681	3.00605349	6.86348409	4.05197203	4.24476875	4.78250726	126.289814
0.10513833	0.40080713	0.18933749	0.06186217	0.1825707	0.30205309	2.38174383
6.09802294	5.29065415	7.3841622	5.66038841	6.43561714	5.73900871	23.0591087
63.4509801	57.1150164	61.7240225	58.4597492	60.5221867	69.2456709	153.396541
31.9883359	37.3552248	42.127592	32.1683276	37.7008493	37.404241	101.903282
60.4545378	55.912595	62.1973662	63.9345511	56.5969167	52.1545002	39.631911
1.89248988	1.16234068	1.51469994	1.79400289	1.96263501	1.30889672	4.51277778
27.4148187	34.389252	31.0986831	30.7764288	34.6884328	30.7842441	63.7569277
0.13142291	0.04008071	0.04733437	0.06186217	0.09128535	0.05034218	0.12338274
3.10158063	3.00605349	3.26607174	3.15497059	3.01241653	3.70015035	2.92304924
101.300778	92.3058826	119.093283	107.021551	100.322599	116.466637	502.717037

221.027047	190.824276	196.200976	211.537685	195.031149	222.890009	713.401404
16.270156	16.1124467	15.099665	16.8574409	14.2861572	14.5488905	10.0065572
12.6165992	9.21856405	10.6502339	12.2487094	9.95010309	11.8304127	24.3241923
2.39189693	2.64532708	2.65072489	2.07238264	2.32777641	2.7436489	4.29658193
0.10513833	0.04008071	0	0	0	0.10068436	0.16922917
0.60454538	0.72145284	0.66268122	0.68048385	0.41078407	0.65444836	1.79934661
1.5770749	0.96193712	0.99402183	0.80420819	2.00827769	1.23338345	1.10052054
2.33932777	2.68540779	2.03537804	2.31983132	2.55598978	2.66813563	4.3823357
1.07766785	1.08217926	0.94668746	0.89700144	1.14106687	1.33406781	1.20723004
29.3861623	26.3330286	27.6432739	29.1989435	26.8378927	30.0794535	18.8800733
0.10513833	0	0.04733437	0	0.04564267	0.07551327	0.14475427
16.4015789	18.4772088	17.4663837	17.1048896	17.5724298	19.5327665	13.8657982
68.6290427	69.4197954	62.1026975	64.2438619	70.2440764	59.6051431	38.6790589
1.55079032	1.00201783	0.89935309	1.20631228	1.18670954	1.43475218	0.85337785
111.157496	105.332114	117.105239	111.754007	107.625427	125.226177	262.504494
229.93752	230.744666	222.613557	223.569877	219.358695	218.76195	184.264122
39.4005879	35.9924805	38.5301797	38.4782688	36.2402837	35.6674357	49.3833596
48.9156064	43.888381	47.0030325	44.2623815	46.6924562	44.8297128	17.1258561
27.9667949	26.97432	21.7264772	28.8277705	26.518394	24.4411292	13.0353852
12.0120538	11.5031647	12.306937	11.9084674	12.0496661	10.4963449	5.04261103
32.4088892	32.986427	29.7733207	33.5292953	31.4478029	33.2761821	27.4267153
222.866968	231.466119	193.31358	207.856886	222.827538	194.950099	88.1299111
96.4381301	99.6005725	118.856611	99.8455399	94.0239099	110.022838	468.140256
18.1363613	34.5896555	32.1873737	16.5481301	16.2944349	44.7038573	64.4004173
17.9786538	34.5094941	31.9980362	16.4862679	16.2031495	44.628344	106.503741
48.0744998	35.8722384	56.0438977	45.159383	42.3107595	46.9189133	22.909555
149.21757	177.878205	171.492434	162.140744	165.409053	189.336945	417.224357
2.75988107	1.92387424	5.49078728	0.89700144	7.39411331	3.44843944	2.19799601
67.4725211	64.3696255	64.8007567	67.553488	54.8168524	67.5592078	22.5114255
17.4792468	16.352931	16.6143649	15.805784	16.2944349	20.3130703	9.12011312
5.4409084	4.44895917	4.59143419	4.76338697	5.11197957	5.46212671	2.86270242
56.2752893	58.6781642	51.8784729	59.109302	62.8043204	50.8707746	19.1038526
35.7207465	32.4653777	29.7733207	31.8280857	31.3108749	31.7407455	18.7539327
26.573712	24.048428	22.6731647	24.8685917	23.2777641	27.7637132	37.5155576
0.10513833	0	0.04733437	0.09279325	0.04564267	0.07551327	0.12854989
7.04426788	5.49105772	6.10613413	6.8667007	6.02483306	4.88319162	4.90586346
119.489708	138.879671	132.678248	134.550216	148.156122	122.230817	48.0348342
45.8403104	44.168946	41.9382545	45.2521762	44.8667493	37.9076628	35.5697918
80.7199502	90.0613627	101.674233	86.7926223	102.148306	92.5037588	103.072575
19.5031596	19.7597916	23.2411772	19.9196182	21.0412731	23.8118519	50.3863314
3.36442645	3.44694134	3.55007798	3.21683276	3.28627258	3.27224181	13.5830143
93.5205415	101.16372	96.2307804	100.464162	99.5010309	81.2774523	35.5397022
23.8664001	20.0804373	17.4190493	23.1983132	20.2197049	20.6402945	20.9887964
15.6393261	14.9100253	13.2536245	16.4553368	14.1035865	15.0523123	13.3879337
6.64999916	8.01614265	7.85750593	7.94928865	8.3069668	7.27444525	5.12592693

13.4314212	13.4270389	14.5316525	13.4240906	13.9210158	15.6312474	6.79506195
9.33102648	13.7476846	17.6083868	12.9601243	13.327661	12.0569525	11.500763
25.259483	23.0864908	22.8151678	27.157492	22.5474813	23.9628785	21.2733587
15.1662036	17.4351103	17.4190493	16.888372	15.2446534	15.8577872	14.7359758
55.4867519	55.63203	53.2511697	53.5107757	53.3106441	52.657922	35.9227978
39.9525641	38.7981304	40.8968983	39.7464432	41.2153353	35.0885006	12.137932
67.7353669	72.7464946	72.0902502	72.5952547	68.1445134	65.394494	36.0887095
3.54841852	4.00807133	3.02939988	3.3405571	4.01655538	3.92669017	4.10859264
15.7181798	14.3889761	10.6028996	14.4757474	11.9583808	12.5352032	13.5885639
162.438715	171.906179	156.960781	179.43122	172.301097	119.235457	32.7524127
11.9857692	11.0622769	12.2122683	12.7745378	9.85881774	11.07528	18.606618
4.83636302	5.65138057	4.68610293	5.04176673	6.11611841	4.9335338	3.13942779
182.651558	174.19078	168.273696	199.381769	168.60404	176.172465	146.90004
12.8005913	15.8719625	14.9576619	14.2901609	16.0205788	15.0523123	4.60213807
326.585927	225.373851	333.375989	329.230461	281.524018	233.612894	323.226339
14.1936741	13.5873618	12.8749495	13.5168838	11.6388821	17.2925394	18.5898421
31.4100751	35.4313505	46.5296887	35.2923671	35.920785	37.4797543	48.422788
23.2092856	22.5253609	24.0931959	24.4974187	20.9499877	21.9240201	25.3836185
22.1841869	25.2909301	20.8744585	22.9508645	21.6802705	21.345085	13.1992165
45.1043421	45.3312867	49.985098	46.4894196	43.1323276	46.4406626	21.245852
10.8029631	11.1424383	10.0348871	12.9601243	9.76753239	10.6725425	8.19033507
37.7183747	38.5977269	36.542136	36.003782	37.746492	35.7681201	22.9268732
10.6978247	9.49912904	10.3188933	11.011466	10.5891005	10.244634	5.67128558
7.75395159	7.13436696	6.10613413	5.66038841	8.99160692	7.6771827	8.83997651
0.47312247	0.72145284	1.51469994	0.64955277	0.95849617	0.75513273	0.32560549
1.5770749	1.2024214	1.65670306	1.60841638	1.55185094	1.61094981	1.00349155
7.88537449	8.49711121	8.37818403	7.60904672	8.17003878	7.92889361	2.51756819
6.93912955	7.2546091	8.52018715	6.71204528	7.7592547	9.21261925	6.36321964
3.78497976	3.08621492	4.02342171	2.938453	3.51448595	3.80083472	2.75519786
19.4505904	23.206733	19.028418	18.6514438	21.4064144	19.4320821	15.1455257
0.07885374	0.12024214	0.14200312	0.06186217	0.27385605	0.10068436	0.11157967
22.2104715	20.7618095	20.7324554	22.394105	21.9997692	21.5967959	13.6565683
16.7695631	15.3509132	16.0463525	14.321092	17.4355017	14.7754303	11.2807037
26.7839887	29.499405	27.5012707	29.3226678	28.344101	23.3587723	8.76406553
45.7351721	46.6539502	37.678161	42.2518611	45.7796028	46.4910048	44.1049764
0.60454538	0.96193712	0.56801248	0.74234602	0.63899745	0.553764	0.62486815
5.28320091	5.61129986	7.14749033	5.01083564	6.61818784	7.32478743	3.62166538
21.9739103	21.0824552	18.1290649	24.9923161	13.6015171	19.5075954	14.2337097
0.07885374	0.20040357	0.42600936	0.24744867	0.22821337	0.276882	0.09888327
40.1102716	43.3673317	44.7309825	40.024823	39.4809136	44.5780019	71.7343635
249.834949	250.143731	273.592676	305.444457	208.267525	291.858798	101.098053
0.78853745	0.80161427	1.04135621	0.52582843	0.95849617	0.90615927	1.32591924
9.5938723	9.25864476	8.6148559	8.97001443	9.44803367	10.2698051	19.6748411
18.662053	21.0423745	23.6671865	20.6928953	19.5350648	20.6402945	71.2293747
8.83161943	10.3007433	9.37220587	9.86701587	7.89618273	9.36364579	4.04099121

21.553357	22.084473	20.6377867	20.0124115	20.8587024	21.2695718	15.1877328
91.8120437	89.1395063	91.0239994	96.8761558	78.6879712	88.1491601	82.7042129
47.0231166	44.8102374	58.8839601	52.9230851	31.3108749	75.7901545	110.833692
23.3932777	22.5654416	18.9810836	25.3634891	21.5433425	20.6402945	16.902925
45.1043421	46.6138695	46.056345	45.4068316	46.6011709	41.834353	16.06273
0.07885374	0.36072642	0.14200312	0.24744867	0.31949872	0.12585545	0.05232472
313.023083	322.850145	296.407844	348.62425	313.245677	303.160618	306.088352
3.04901147	1.8036321	4.73343731	2.13424481	3.01241653	3.59946599	2.99462933
0.42055331	0.52104927	0.56801248	0.5876906	0.41078407	0.60410618	0.33449718
1.70849781	1.44290568	2.36671865	1.54655421	1.78006431	1.76197636	2.77185704
50.702958	52.1049272	46.8610293	58.8309222	49.2940887	46.7678868	17.0115694
1.52450574	1.8036321	1.98804367	1.23724337	1.41492292	2.16471381	2.24552127
0.5782608	0.72145284	0.80468434	0.43303518	1.00413884	0.88098818	2.28924892
0.13142291	0.08016143	0.28400624	0.12372434	0.31949872	0.15102655	0.42814303
6.86027581	6.13234913	6.20080287	5.38200866	5.38583562	7.4254718	21.7129997
5.91403087	5.450977	6.1534685	4.63966263	7.30282796	4.78250726	5.95926432
4.78379386	4.88984702	6.01146538	4.73245589	5.47712097	6.21725944	7.30124091
0.18399207	0.6012107	0.28400624	0.27837976	0.3651414	0.276882	0.10198223
9.48873397	10.0201783	8.33084966	10.0216713	9.2198203	9.79155433	7.89084198
5.57233131	5.17041201	7.57349969	5.53666408	5.11197957	6.54448362	24.7874576
13.1422908	12.5452633	9.75088085	18.4658573	5.52276364	8.68402634	9.91781051
0.05256916	0	0	0	0.09128535	0.02517109	0.05951604
68.4976198	67.8165668	71.7115752	66.7183487	67.4142306	67.8109187	54.3751652
150.847214	184.130797	187.870127	168.265098	188.960673	184.957175	176.414126
18.0049384	21.523343	14.6263213	20.940344	13.9210158	16.7387754	12.8689314
2.02391279	1.16234068	2.17738116	1.54655421	1.87134966	2.36608254	2.20170984
0.15770749	0.20040357	0.33134061	0.18558651	0.31949872	0.10068436	0.25119954
3.8638335	4.60928202	2.93473113	3.55707469	3.51448595	3.72532144	3.83339061
10.9869551	9.41896762	14.7683244	7.14508046	17.8919285	7.72752489	4.00362837
29.0181781	29.0585171	28.0692832	29.5391854	29.2113118	24.8186956	11.6768125
30.2798381	30.4212614	26.2705771	29.6319787	30.306736	29.9535981	20.0004752
1.76106697	1.60322853	1.51469994	1.20631228	1.18670954	2.06402945	2.93499631
167.485354	148.418881	156.487437	166.718544	159.201649	162.278023	203.660405
2.60217358	2.44492351	2.03537804	2.53634891	1.73442164	2.34091145	2.47623542
5.46719298	5.41089629	4.87544043	5.38200866	4.29041142	5.26075798	4.24496139
10.3298406	7.93598123	9.41954024	8.07301298	9.58496169	8.91056616	23.9098238
8.22707406	8.73759549	7.47883094	7.98021973	6.93768656	8.40714434	5.44003165
8.83161943	7.37485124	7.43149657	8.75349684	9.53931902	6.72068125	2.67184123
2506.26114	2522.11896	2864.77093	2707.83084	2575.88999	2961.35367	4126.34233
49.3624443	48.5377438	51.7838041	49.6753213	50.2982276	45.3331346	33.5468618
0.39426872	0.28056499	0.80468434	0.52582843	0.3651414	0.37756636	0.72201067
23.813831	21.5634237	21.6318085	21.4043103	20.8130597	22.6539818	20.6310969
16.1387331	11.9440526	13.2062901	13.3622284	12.2778795	13.9951265	19.8714389
600.076999	671.351947	736.002167	615.528576	641.188295	674.761432	1466.66405
27.2571112	27.0544815	31.335355	24.2809011	33.6386513	24.6928401	32.4666239

74.4905044	70.3015711	69.4868597	77.6060903	63.0781765	67.0054438	27.3150891
2.18162028	2.60524636	1.89337492	2.25796915	2.87548851	2.08920054	3.13049145
0.05256916	0	0	0.06186217	0.04564267	0	0.05775221
36.4567147	38.3973233	32.7553862	39.5299256	34.3232914	37.2532144	23.8805688
28.8079015	25.7318179	22.9571709	25.2706958	24.281903	25.5738283	40.5760296
79.8262745	81.1634444	110.525761	84.8130329	84.621519	112.640631	262.148473
150.374092	162.206647	156.487437	148.190825	170.018963	134.967389	111.316437
162.018161	175.353121	146.499885	160.532327	168.832254	137.76138	87.9300936
32.7768733	30.1807771	32.7080518	33.3746399	33.2278672	32.0931408	52.343951
23.3407085	19.5994688	18.8864149	17.4142004	22.6844093	24.4914714	12.3844481
5.38833924	4.72952416	5.30144978	5.22735323	5.29455027	5.66349544	7.80980574
11.8280617	10.0201783	8.99353088	11.382639	8.76339355	9.36364579	6.6601122
72.8345757	76.0331131	70.2442096	69.0072489	74.3519171	69.4973818	18.0890192
0.13142291	0.28056499	0.04733437	0.09279325	0.1825707	0.12585545	0.11199422
2.89130398	2.20443923	1.9407093	1.94865831	2.87548851	2.81916217	1.6347999
40.3205482	40.6017625	43.5002888	43.1797936	42.3564021	42.4132881	19.0401594
0.86739119	0.88177569	0.89935309	0.64955277	0.91285349	0.88098818	1.53112103
9.3047419	9.01816048	7.99950905	7.94928865	9.49367634	7.92889361	12.3319109
8.48991987	11.0622769	9.18286837	9.99074021	9.26546297	8.20577561	2.60127588
64.1869484	63.9688184	65.1794317	67.8627988	63.5802459	56.0560193	39.1225455
1.5770749	2.36476208	2.03537804	2.28890023	1.96263501	2.06402945	0.88801496
79.0114524	63.327527	69.4868597	73.4922561	78.3684725	67.2571547	59.1334024
29.2021702	28.7378714	28.2586207	28.7040462	28.7092424	26.2786188	6.50329234
12.7743067	13.9480882	12.3542714	13.2694351	13.1450903	14.146153	12.8720109
0.94624494	0.72145284	1.04135621	0.74234602	1.41492292	0.88098818	3.14545516
122.48615	126.775296	135.707648	122.3943	132.272471	142.996967	169.019181
3.04901147	3.40686063	3.45540923	3.49521252	2.78420316	4.10288781	7.9021416
32.35632	33.4273149	29.7733207	35.7563334	28.9374558	27.6630288	25.2833023
1.78735155	2.04411638	1.70403743	2.31983132	1.64313629	1.9130029	6.95211712
0.18399207	0.28056499	0.09466875	0.15465542	0.1825707	0.30205309	0.13799803
0.05256916	0.04008071	0.09466875	0.03093108	0.13692802	0.07551327	0.26141091
0.10513833	0.04008071	0.04733437	0.18558651	0.09128535	0	0.19369603
0.10513833	0.04008071	0.04733437	0.18558651	0.09128535	0	0.19817283
0.13142291	0.04008071	0.04733437	0.09279325	0	0.17619764	0.17389693
0	0.04008071	0.09466875	0.12372434	0	0.02517109	0
0.18399207	0.32064571	0.09466875	0.06186217	0.22821337	0.22653982	0.09421297
17.6106697	18.7577738	16.7563681	20.6928953	15.153368	15.1026545	5.8780008
1.23537534	0.84169498	0.37867498	1.23724337	0.1825707	0.72996163	1.12108046
2.41818151	1.84371281	2.27204991	1.82493397	1.78006431	2.4667669	4.28713889
0.76225287	0.48096856	0.66268122	0.52582843	1.00413884	0.65444836	1.7109224
11.1709472	11.0622769	11.549587	11.1042592	11.4106687	12.5352032	11.1235289
43.5272672	41.9645068	39.0508578	47.726663	42.9041142	38.1342026	23.1003319
0.18399207	0.12024214	0.04733437	0.12372434	0.22821337	0.12585545	0.49304445
0.21027665	0.44088785	0.61534685	0.37117301	0.41078407	0.553764	0.21697877
4.02154099	5.05016987	5.34878416	3.86638553	5.29455027	4.45528308	3.17079721

1.99762821	1.2024214	1.56203431	1.88679614	2.00827769	1.35923891	2.56687938
0.7096837	1.16234068	0.99402183	0.77327711	1.00413884	0.830646	0.49326456
0.13142291	0.20040357	0.37867498	0.27837976	0.22821337	0.25171091	0.5314635
1.73478239	1.68338996	3.171403	2.50541782	1.55185094	1.9130029	7.03858624
18.5569146	17.5954331	13.3482932	16.4553368	19.3524941	16.059156	7.30268632
5.07292426	5.17041201	6.05879975	4.5159383	10.9998846	2.56745127	2.68991484
0.07885374	0.08016143	0.04733437	0.09279325	0.1825707	0.02517109	0.05451293
115.231606	118.598831	129.648848	120.352849	110.272702	121.450513	53.3337596
55.854736	50.3413759	42.884942	47.1699034	48.0160938	49.1843115	21.4088285
9.98814103	9.13840262	9.23020275	9.49584286	9.0372496	11.0249378	10.1989531
1230.80182	1142.42057	1276.04003	1335.3877	1183.92534	1279.11932	2348.86387
28.1507869	23.1666523	24.613874	26.6316635	19.5807075	26.7820406	15.0772518
28.3610636	32.986427	33.2287299	29.755703	41.534834	27.3358046	13.1739782
22.4996019	18.2367245	22.3418241	21.5589657	16.8877897	22.0750467	52.9806192
0.05256916	0	0.04733437	0	0	0.12585545	2.04149471
0.05256916	0	0	0	0.09128535	0.02517109	1.53112103
0.26284582	0.24048428	0.33134061	0.21651759	0.1825707	0.30205309	8.8091895
0.10513833	0.04008071	0.04733437	0.12372434	0.13692802	0.02517109	2.76589606
12.4063225	12.304779	16.2830243	12.2177783	12.9625196	13.9447843	73.6721926
48.1796382	43.2470896	53.2511697	44.3242437	55.5014925	49.9394442	136.429264
44.2632355	41.0827311	41.8909202	43.8602774	44.9580346	42.3377748	55.9076553
3.89011808	3.80766776	4.78077168	3.27869493	3.92527003	5.99071962	2.13827192
30.2798381	37.1548212	35.4534454	27.0646987	34.5058621	37.3790699	41.3633501
1.13023701	0.88177569	0.89935309	1.02072578	1.04978152	0.67961945	0.5651348
108.71303	113.107773	126.288107	108.320657	102.696018	125.377203	474.107124
697.513943	673.115498	688.005112	682.772753	717.594132	582.333186	350.630177
0.44683789	0.20040357	0.28400624	0.24744867	0.27385605	0.32722418	0.54389075
201.813018	163.52931	223.607578	204.207018	181.155776	226.338449	422.730981
6.99169872	10.1003397	8.18884654	9.18653202	8.3069668	8.86022397	7.71221108
0.60454538	0.6012107	0.56801248	0.40210409	0.5477121	0.65444836	0.46336558
12.2748996	11.0221961	8.66219027	12.3415026	10.3152445	10.8739112	16.050993
55.854736	52.8263801	58.4579507	59.480475	53.3106441	59.3786033	32.221776
24.8914988	25.5314143	27.3592676	28.1163556	24.9209004	22.1505599	15.2361291
75.016196	75.3918216	71.7115752	79.8021973	72.8913515	62.5249896	47.9572553
26.4422891	28.5374678	24.4245365	24.8376606	25.5598978	26.5806719	11.0605105
3.5747031	4.60928202	4.82810605	4.48500721	4.01655538	5.05938926	0.93577609
0.81482203	0.84169498	0.85201872	0.89700144	0.68464012	0.70479054	0.34974028
6.20316127	6.49307555	6.95815284	6.74297636	5.93354771	6.0410618	7.92505048
1.97134362	1.60322853	1.98804367	1.39189879	1.87134966	1.93817399	1.58495933
90.8132296	96.6345997	87.7579277	95.5461192	91.1027788	80.6230039	58.2846204
30.7003914	35.0305434	33.1340611	30.0650139	31.7216589	33.8802883	53.5739468
18.0837922	18.1565631	15.6203431	17.7544423	17.3898591	18.6517783	14.0321197
236.166966	207.89866	258.919021	223.198704	210.686587	244.939885	247.557474
276.829214	247.017436	250.162162	276.771342	270.661061	252.843607	106.944924
123.353542	136.875636	135.991654	114.630598	140.305582	128.146023	188.620714

0.13142291	0.08016143	0.23667187	0.15465542	0.13692802	0.20136873	0.3800655
16.3227252	11.9841333	10.6028996	13.3931595	13.327661	14.1964952	16.1941195
0.31541498	0.32064571	0.37867498	0.24744867	0.31949872	0.32722418	0.25087289
47.6539465	39.3592604	48.8017386	35.2923671	55.3645644	39.8710079	60.1903677
30.4375455	21.7638273	26.7439208	28.5184597	21.7259132	25.6745127	25.3205041
11.118378	8.53719192	10.2242246	10.9186727	9.53931902	8.18060452	8.07897127
4.59980179	4.64936274	3.21873737	5.41293974	3.6057713	4.22874326	1.48701633
47.1545395	57.9166307	43.8789638	45.2521762	51.0285103	47.6237039	61.9918901
16.4015789	15.9922046	17.5610524	17.259545	16.0662215	18.3497252	22.3303395
18.2152151	18.316886	17.135043	19.1154101	18.2570699	18.1986987	11.5993776
66.7102682	63.0068812	66.6941316	70.3991477	60.4309013	59.5548009	45.4881208
78.0126383	84.1694978	79.805753	85.4935168	82.2937425	66.778904	46.4613981
19.6871517	20.0002759	22.578496	18.4967884	22.0910546	23.9377074	18.1484572
8.5950582	9.25864476	6.72148097	9.71236045	6.11611841	7.47581398	6.18300978
13.9833974	13.4671197	12.6382776	13.3312973	14.2405145	14.146153	20.4981236
[truncated: 456,697 more chars]
